# Physical rehabilitation approaches for the recovery of function and mobility following stroke

**DOI:** 10.1002/14651858.CD001920.pub4

**Published:** 2025-02-11

**Authors:** Alex Todhunter-Brown, Ceri E Sellers, Gillian D Baer, Pei Ling Choo, Julie Cowie, Joshua D Cheyne, Peter Langhorne, Julie Brown, Jacqui Morris, Pauline Campbell

**Affiliations:** Department of Nursing and Community HealthGlasgow Caledonian UniversityGlasgowUK; Department of PhysiotherapyQueen Margaret UniversityEdinburghUK; Health & Social SciencesSingapore Institute of TechnologySingaporeSingapore; Yunus CentreGlasgow Caledonian UniversityGlasgowUK; UWS Library ServicesUniversity of the West of ScotlandPaisleyUK; Academic Section of Geriatric Medicine, ICAMSUniversity of GlasgowGlasgowUK; GlasgowUK; School of Health SciencesUniversity of DundeeDundeeUK

## Abstract

**Background:**

Various approaches to physical rehabilitation to improve function and mobility are used after stroke. There is considerable controversy around the relative effectiveness of approaches, and little known about optimal delivery and dose. Some physiotherapists base their treatments on a single approach; others use components from several different approaches.

**Objectives:**

**Primary objective:** To determine whether physical rehabilitation is effective for recovery of function and mobility in people with stroke, and to assess if any one physical rehabilitation approach is more effective than any other approach.

**Secondary objective:** To explore factors that may impact the effectiveness of physical rehabilitation approaches, including time after stroke, geographical location of study, intervention dose/duration, intervention provider, and treatment components.

**Stakeholder involvement:** Key aims were to clarify the focus of the review, inform decisions about subgroup analyses, and co‐produce statements relating to key implications.

**Search methods:**

For this update, we searched the Cochrane Stroke Trials Register (last searched November 2022), CENTRAL (2022, Issue 10), MEDLINE (1966 to November 2022), Embase (1980 to November 2022), AMED (1985 to November 2022), CINAHL (1982 to November 2022), and the Chinese Biomedical Literature Database (to November 2022).

**Selection criteria:**

**Inclusion criteria:** Randomised controlled trials (RCTs) of physical rehabilitation approaches aimed at promoting the recovery of function or mobility in adult participants with a clinical diagnosis of stroke.

**Exclusion criteria**: RCTs of upper limb function or single treatment components.

**Primary outcomes:** measures of independence in activities of daily living (IADL) and motor function.

**Secondary outcomes:** balance, gait velocity, and length of stay.

**Data collection and analysis:**

Two independent authors selected studies according to pre‐defined eligibility criteria, extracted data, and assessed the risk of bias in the included studies. We used GRADE to assess the certainty of evidence.

**Main results:**

In this review update, we included 267 studies (21,838 participants). Studies were conducted in 36 countries, with half (133/267) in China. Generally, studies were heterogeneous, and often poorly reported. We judged only 14 studies in meta‐analyses as at low risk of bias for all domains and, on average, we considered 33% of studies in analyses of primary outcomes at high risk of bias.

Is physical rehabilitation more effective than no (or minimal) physical rehabilitation?

Compared to no physical rehabilitation, physical rehabilitation may improve IADL (standardised mean difference (SMD) 1.32, 95% confidence interval (CI) 1.08 to 1.56; 52 studies, 5403 participants; low‐certainty evidence) and motor function (SMD 1.01, 95% CI 0.80 to 1.22; 50 studies, 5669 participants; low‐certainty evidence). There was evidence of long‐term benefits for these outcomes.

Physical rehabilitation may improve balance (MD 4.54, 95% CI 1.36 to 7.72; 9 studies, 452 participants; low‐certainty evidence) and likely improves gait velocity (SMD 0.23, 95% CI 0.05 to 0.42; 18 studies, 1131 participants; moderate‐certainty evidence), but with no evidence of long‐term benefits.

Is physical rehabilitation more effective than attention control?

The evidence is very uncertain about the effects of physical rehabilitation, as compared to attention control, on IADL (SMD 0.91, 95% CI 0.06 to 1.75; 2 studies, 106 participants), motor function (SMD 0.13, 95% CI ‐0.13 to 0.38; 5 studies, 237 participants), and balance (MD 6.61, 95% CI ‐0.45 to 13.66; 4 studies, 240 participants).

Physical rehabilitation likely improves gait speed when compared to attention control (SMD 0.34, 95% CI 0.14 to 0.54; 7 studies, 405 participants; moderate‐certainty evidence).

Does additional physical rehabilitation improve outcomes?

Additional physical rehabilitation may improve IADL (SMD 1.26, 95% CI 0.82 to 1.71; 21 studies, 1972 participants; low‐certainty evidence) and motor function (SMD 0.69, 95% CI 0.46 to 0.92; 22 studies, 1965 participants; low‐certainty evidence). Very few studies assessed these outcomes at long‐term follow‐up.

Additional physical rehabilitation may improve balance (MD 5.74, 95% CI 3.78 to 7.71; 15 studies, 795 participants; low‐certainty evidence) and gait velocity (SMD 0.59, 95% CI 0.26 to 0.91; 19 studies, 1004 participants; low‐certainty evidence). Very few studies assessed these outcomes at long‐term follow‐up.

Is any one approach to physical rehabilitation more effective than any other approach?

Compared to other approaches, those that focus on *functional task training* may improve IADL (SMD 0.58, 95% CI 0.29 to 0.87; 22 studies, 1535 participants; low‐certainty evidence) and motor function (SMD 0.72, 95% CI 0.21 to 1.22; 20 studies, 1671 participants; very low‐certainty evidence) but the evidence in the latter is very uncertain. The benefit was sustained long‐term.

The evidence is very uncertain about the effect of *functional task training* on balance (MD 2.16, 95% CI ‐0.24 to 4.55) and gait velocity (SMD 0.28, 95% CI ‐0.01 to 0.56).

Compared to other approaches, *neurophysiological approaches* may be less effective than other approaches in improving IADL (SMD ‐0.34, 95% CI ‐0.63 to ‐0.06; 14 studies, 737 participants; low‐certainty evidence), and there may be no difference in improving motor function (SMD ‐0.60, 95% CI ‐1.32 to 0.12; 13 studies, 663 participants; low‐certainty evidence), balance (MD ‐0.60, 95% CI ‐5.90 to 6.03; 9 studies, 292 participants; low‐certainty evidence), and gait velocity (SMD ‐0.17, 95% CI ‐0.62 to 0.27; 16 studies, 630 participants; very low‐certainty evidence), but the evidence is very uncertain about the effect on gait velocity.

For all comparisons, the evidence is very uncertain about the effects of physical rehabilitation on adverse events and length of hospital stay.

**Authors' conclusions:**

Physical rehabilitation, using a mix of different treatment components, likely improves recovery of function and mobility after stroke. Additional physical rehabilitation, delivered as an adjunct to 'usual' rehabilitation, may provide added benefits. Physical rehabilitation approaches that focus on functional task training may be useful. Neurophysiological approaches to physical rehabilitation may be no different from, or less effective than, other physical rehabilitation approaches.

Certainty in this evidence is limited due to substantial heterogeneity, with mainly small studies and important differences between study populations and interventions. We feel it is unlikely that any studies published since November 2022 would alter our conclusions. Given the size of this review, future updates warrant consensus discussion amongst stakeholders to ensure the most relevant questions are explored for optimal decision‐making.

## Plain language summary

**How effective are different physical rehabilitation approaches in the recovery of function, balance, and walking after stroke?**

**Key messages**

For people who have had a stroke:

**‐** Physical rehabilitation may improve daily activities, moving legs, balance, and walking, when compared to no physical rehabilitation. There may be greater benefits when more than 2.5 hours/week of physical rehabilitation is delivered.

‐ Extra physical rehabilitation, given in addition to 'usual' physical rehabilitation, may also improve outcomes. The greater the amount of extra physical rehabilitation, the greater the benefit may be.

‐ Physical rehabilitation focussed on *functional task training* (the active practice of real‐life tasks with the aim of acquiring ‐ or reacquiring ‐ a movement skill) may improve daily activities and movement.

**Why is it important to review the evidence on this topic?**

Stroke can cause paralysis of some parts of the body and can create difficulties with physical functions. Over the years, various approaches to physical rehabilitation have been developed, based on ideas about how people recover after a stroke. Often, physiotherapists will choose one particular approach, based on their clinical experience and the rationale, but clear research evidence is lacking. This means that techniques used by individual physiotherapists may differ (e.g. one may provide strengthening exercises, while another may focus on passive movements). Historically, a number of named physical rehabilitation approaches (e.g. the ‘Bobath’ approach) have been used; together we call these neurophysiological approaches, as they were developed based on knowledge and theories relating to the function and recovery of the nervous system. It is important to help physiotherapists select the approach that will help their patients gain the best recovery.

*Note:* Physiotherapist/physiotherapy can be called physical or rehabilitation therapist/therapy, meaning the same. We use the term physical rehabilitation and describe the person providing physical rehabilitation as a therapist.

**What did we want to find out?**

We wanted to know:

‐ Are physical rehabilitation approaches effective in the recovery of function and mobility in people with stroke?

‐ Is one physical rehabilitation approach more effective than another approach?

**What did we do?**

We searched for relevant studies, called randomised controlled trials. We brought together studies in which people who had a stroke received physical rehabilitation with the goal of improving the ability to walk and carry out activities of daily living. We were interested in different *approaches* to physical rehabilitation (i.e. a programme of treatment based on a particular scientific rationale). These approaches might involve therapist‐delivered, group, or remote treatment. Therapists may select specific treatments/exercises according to individual patient needs, or deliver standard exercises based on the stage of patient recovery. We excluded studies that only looked at 'single' treatments (e.g. electrical stimulation, robotic device) or were focused *only* on arm function.

**What did we find?**

We found 267 studies, which included 21,838 people with stroke. Studies were from 36 different countries, but half (133 studies) were carried out in China.

One hundred and five studies looked at whether physical rehabilitation was better than no physical rehabilitation. Most of these studies were carried out in hospital in‐patient settings in China where physical rehabilitation was not part of routine care, but a few were carried out in outpatient settings after the patient had been discharged from routine physical rehabilitation. These studies showed that physical rehabilitation may improve a person's ability to carry out activities of daily living, move the legs, remain balanced, and walk, in comparison to no physical rehabilitation.

Fifty‐six studies looked at the effect of giving extra, or additional, physical rehabilitation. Everyone in these studies received their usual physical rehabilitation, but one group of stroke survivors received some additional treatment based on a particular physical rehabilitation approach. These studies showed that additional physical rehabilitation may improve the ability to carry out activities of daily living, move the legs, remain balanced, and walk; the greater the amount of additional rehabilitation, the greater the possible benefit.

Ninety‐two studies compared different physical rehabilitation approaches. There were many variations in the types and amount of physical rehabilitation, and the types of people (e.g. different lengths of time post‐stroke). These studies showed that physical rehabilitation that focused on functional task training may improve the ability to carry out activities of daily living and move the legs (but not balance or walking). Neurophysiological approaches to physical rehabilitation may be less effective than other approaches at improving daily activities (but no different for other outcomes).

For all comparisons, there was very limited information about potential adverse events relating to physical rehabilitation.

Few studies took long‐term follow‐up measurements after the physical rehabilitation had stopped.

**What are the limitations of the evidence?**

There were large variations between participants, interventions, outcomes, and comparisons in the studies included in this review. There were also geographical and cultural differences that may influence the results. Generally, the reporting of the details of these studies was very poor. These issues mean that we have limited confidence in the results of our statistical analyses.

**How up‐to‐date is this evidence?**

The evidence is up‐to‐date to November 2022. It is unlikely that any studies published since November 2022 would alter our conclusions.

## Summary of findings

**Summary of findings 1:** Physical rehabilitation versus no physical rehabilitation ‐ immediate outcomes

**Physical rehabilitation versus no physical rehabilitation for recovery after stroke**						
**Patient or population:** adults (> 18 years) with clinical diagnosis of stroke **Setting:** any **Intervention:** programmes of physical rehabilitation **Comparison:** no, or minimal, physical rehabilitation						
**Outcomes**	**Relative effect (95% CI)**	**Anticipated absolute effects (95% CI)**		**No. of participants** **(no. of studies)**	**Certainty of the evidence** **(GRADE)**	**Comments**
		**No physical rehabilitation**	**With physical rehabilitation**			
**Primary outcome: Independence in ADL scales** Immediately after intervention (Higher number indicates a better outcome) Analysis 1.1	‐	See comments	SMD 1.32 higher (1.08 higher to 1.56 higher)	5403 (52)	⊕⊕⊝⊝ **Low**^a,b^	Compared to no physical rehabilitation, physical rehabilitation may improve activities of daily living. A standard deviation of 1.32 represents a large difference between groups.
**Primary outcome: Motor function scales** Immediately after intervention (Higher number indicates a better outcome) Analysis 1.2	‐	See comments	SMD 1.01 higher (0.80 higher to 1.22 higher)	5669 (50)	⊕⊕⊝⊝ **Low**^a,b^	Compared to no physical rehabilitation, physical rehabilitation may improve motor function. A standard deviation of 1.01 represents a large difference between groups.
**Secondary outcome: Balance (Berg Balance Scale)** Immediately after intervention (Higher number indicates a better outcome) Analysis 1.3	‐	The mean score in the no treatment group ranged from 31.1 to 53	MD 4.54 higher (1.36 to 7.72 higher)	452 (9)	⊕⊕⊝⊝ **Low**^a,c^	The evidence suggests that, compared to no physical rehabilitation, physical rehabilitation may improve balance, as measured by the Berg Balance Scale.
**Secondary outcome: Gait velocity** Immediately after intervention (Higher number indicates a better outcome) Analysis 1.4		See comments	SMD 0.23 higher (0.05 to 0.42 higher)	1131 (18)	⊕⊕⊕⊝ **Moderate**^c,d^	The evidence suggests that, compared to no physical rehabilitation, physical rehabilitation is likely to improve gait velocity. A standard deviation of 0.23 represents a small difference between groups.
**Secondary outcome: Length of hospital stay** (Low number indicates a better outcome) Analysis 1.5		The mean length of stay for the control group of the included study was 6.3 days.	MD 0.80 higher (0.93 lower to 2.53 higher)	110 (1)	⊕⊝⊝⊝ **Very low**^e,f^	The evidence is very uncertain about the effect of physical rehabilitation on the length of inpatient hospital stay.
**Secondary outcome: Adverse events** (Any adverse events during the intervention period, including serious adverse events and falls, which were defined by the study investigators as possibly, probably, or definitely related to the study/intervention) Analysis 1.6; Table	RR 8.70 (2.90 to 26.06)	‐	‐	283 (5)	⊕⊝⊝⊝ **Very low**^f,g^	The evidence is very uncertain about the effect of physical rehabilitation on adverse events.
GRADE Working Group grades of evidence. **High certainty:** Further research is very unlikely to change our confidence in the estimate of effect. **Moderate certainty:** Further research is likely to have an important impact on our confidence in the estimate of effect and may change the estimate. **Low certainty:** Further research is very likely to have an important impact on our confidence in the estimate of effect and is likely to change the estimate. **Very low certainty:** We are very uncertain about the estimate. **Abbreviations:** ADL: activities of daily living; CI: confidence interval; MD: mean difference; RR: risk ratio; SMD: standardised mean difference						

^a^Downgraded twice for inconsistency, as there is considerable heterogeneity, with no overlap of confidence intervals for a number of studies. ^b^This finding does not change with sensitivity analyses to remove studies at high or uncertain risk of bias (Table). ^c^Removing studies at high risk of bias in the analysis does not change this finding. ^d^Downgraded one level for inconsistency as heterogeneity is moderate (I² = 53%) but it was reduced (to I² = 24%) when only direct gait speed data are included (SMD 0.26, 95% CI 0.08 to 0.43). ^e^Downgraded twice for imprecision, as the data relate to a single study with only 110 participants. ^f^Downgraded once due to concerns about missing data from other studies, as this outcome is not reported in the majority of studies. ^g^Downgraded twice, as data combine serious, moderate, and mild adverse events, with different studies gathering varied data relating to adverse events, and study authors reaching different decisions about whether adverse events are potentially related to the study procedures or not.

**1:** Adverse events ‐ trials actively monitoring adverse events

**Study**	**Number of participants in intervention group**	**Number of adverse events in intervention group**	**Number of participants in control group**	**Number of adverse events in control group**	**Additional Information**
**Comparison: Physical rehabilitation versus no physical rehabilitation (**Analysis 1.6)					
ACTIV 2021^a^	47	AE 28	48	SAE 1. AE 25	1 death (control group) but "no severe injuries in either group". No severe injuries. Several mild to moderate events that did not require medical intervention (ACTIV: n = 28; control: n = 25); however, only one of these events was attributed to the intervention (mild exacerbation of osteoarthritis) ‐ unclear which group this event occurred in.
Aravind 2022	16	0	17	1	No injurious falls occurred during exercise classes. One participant in the waitlist (control group) group experienced an injurious fall in month 4 while performing usual activities and sought the advice of their physician.
ReTrain 2018	21	Intervention period: AEs 22 SAEs 6 Assessment period: AEs 125 SAEs 1	20	Intervention period: Not reported Assessment period: AEs 150 SAEs 0	During intervention period (ReTrain group only reported): SAEs : unrelated 4; possibly related 1 (fainted); probably related 1 (TIA); related 0 AEs: unrelated 3; possibly related 12; probably related 0; related 7 During assessment period ‐ ReTrain group: SAEs : unrelated 1 AEs: unrelated 41; possibly related 73; probably related 5; related 6 Control group: SAEs: no SAEs AEs: unrelated 150
Wang 2022	52	Not clearly reported (assumed 2)	52	Not clearly reported (assumed 0)	Monitored adverse events included: stroke progression, cardiovascular complications, fall‐induced injury, venous thromboembolism, pressure ulcers, pneumonia, urinary tract infections, and complications caused by improper exercise. Dropouts were reported in Figure 1: Flowchart of Study Participants. Authors reported "None of the patients experienced severe adverse events during the study period". Study flowchart reported that at "assessment after 7 days": Intervention group: 2 Stroke progression (and 5 discharged early) Control group: 0 dropouts due to adverse events (and 6 discharged early)
**Comparison: Physical rehabilitation versus attention control (**Analysis 3.6)					
Dean 2006	76	See comments (no falls or other adverse events during exercise class)	75	See comments	No falls or other adverse events occurred during the exercise classes, home programme, or assessments. Of the 18 withdrawals, only 1 was related to the intervention: 1 participant withdrew as the experimental exercise exacerbated an incontinence problem. Of the 3 deaths during the trial, 2 may have been falls related: 1 participant died several months after a fall at home, and 1 had a stroke and fractured his shoulder and died in hospital
Pang 2005	32	Not reported	31	Not reported	1 intervention group participant completed baseline but not follow‐up at 19 weeks as found exercise too fatiguing. 5 falls in intervention group (4 individuals). 1 fall in control group.
Stuart 2019^a^	43	6	33	4	There were no serious adverse events. Minor study‐related adverse events were infrequent. A total of 6 of APA‐Stroke subjects reported minor study related adverse events: faintness (2), shortness of breath (1), chest pain (1), muscle pain (1) or a controlled fall without injury (1). A total of 4 Sittercise subjects reported study‐related minor adverse events: muscle pain (2) or experienced a controlled fall without injury (2).
**Comparison: Additional rehabilitation plus usual therapy versus usual therapy (**Analysis 5.5)					
Aries 2021	19	AEs MTS+TSGT group: 14 SAEs Whole group: 3 (unrelated to trial)	15	AEs TI+TSGT group: 13	Twenty‐seven adverse events were recorded, 14 from the MTS+TSGT group (Intervention 1) and 13 from the TI+TSGT group (Intervention 2). These consisted of: falls (n = 16), back pain (n = 2), neck pain (n= 1), ipsilesional heel pain (n = 1), viral infection (n = 1), atrial fibrillation (n = 1), pressure sore (n = 1), scratch on dorsum of foot (n = 1), tiredness with swollen painful ankles (n = 1), urinary tract infection (n = 1), hip and knee pain (n= 1). There were no adverse reactions to the interventions or outcome measures (confirmed by an independent assessor). There were 3 unrelated serious adverse events (1 further stroke and 2 hospital admissions).
Duncan 2003	50	See comments	50	See comments	There were no deaths or heart attacks. Seven subjects were hospitalised, 4 in the intervention group and 3 in the usual care group. Three subjects, all from the intervention group, had diagnoses of second strokes. Two of the strokes occurred within the first 2 weeks after randomisation, and 1 occurred after 7 weeks. No strokes occurred during a treatment session.
LAST 2018	186	67	194	88	Death: Intervention (IG) 9; Control (CG) 9. Myocardial infarction: IG 4; CG 4. Other cardiovascular events: IG 4; CG 10, Recurrent stroke: IG 7; CG 12. Transient ischaemic attack: IG 5; CG 5. Any vascular event: IG 17; CG 28. Unspecific cerebral symptoms: IG 7, CG 5. Fracture: IG 11, GC 11. Fall: IG 3, CG 4.
Zhuang 2012	Physiotherapy: 86 Acupuncture + Physiotherapy: 97	Physiotherapy: 0 Acupuncture + Physiotherapy: 1	Acupuncture: 91	Acupuncture: 1	The research team reported that there were no significant adverse effects during the trial. Adverse events reported were: Acupuncture group: 1 second stroke 2 poor health 1 small scalp haematoma following a treatment which disappeared after a few days; Physiotherapy: 1 died; Acupuncture + Physiotherapy: 1 displayed syncope during an acupuncture treatment (dizziness, weakness and sweating) ‐ immediate recovery.
**Comparison: Comparison of different approaches ‐ functional task training compared with other (**Analysis 7.6)					
DOSE 2020	DOSE1 25 DOSE2 25	SAEs DOSE 1or2 (not stated) 2	25	SAEs Usual care 2	Two usual care subjects experienced a serious adverse event: 1 hospitalisation for a transient ischaemic attack during the 4‐week intervention; 1 myocardial infarction reported at 12‐months post‐stroke. Two DOSE subjects reported a serious adverse event at 6 months post‐stroke (the specific DOSE group the participants were in was not reported): 1 recurrent stroke and 1 leg fracture from a fall on ice. The subject with a transient ischaemic attack returned to the rehabilitation hospital and completed the remaining evaluations, but the others did not complete their remaining evaluations.
Hendrey 2018	15	1 AE (see comments)	15	1 SAE (see comments)	One serious adverse event occurred in the control group (n = 15). A participant had an extension of stroke during the study period which was not attributed to the study. They were cleared by the neurologist to continue and missed 1 session. One participant in the ballistic strength training (BST) group (n = 15) reported low back pain during the study period, which was not attributed to the intervention, and missed 3 sessions after which they completed the trial.
Kwakkel 2008	126	SAEs 2	124	SAEs 0	Two serious adverse events were reported in the circuit training group: 1 participant fell and consulted a GP and 1 patient experienced arrhythmias during one session.
Mansfield 2018^a^	44	AEs 26 SAEs 3	44	AEs 22 SAEs 6	48 adverse events were possibly, probably or definitely related to study procedures or interventions: Fatigue with training (3 PBT, 1 control), joint pain during or soon after training (14 PBT, 11 control), delayed onset muscle soreness (5 PBT, 8 control), seizure during training (1 PBT group with a history of frequent seizures), abnormal elevated heart rate and low blood pressure during training(1 control). 8 participants experienced serious adverse events not related to study procedures: prolonged hospitalisation (1 PBT, 1 control), another stroke (2 PBT, 3 control), death (1 control), cancer diagnosis (1 control), falls (3 PBT, 1 control)
SPIRES 2022^a^	22	AEs 59 SAEs 17	23	AEs 59 SAEs 13	AE: Intervention group 59 adverse events from 20 participants. Control group 59 AE from 18 participants. SAE: Intervention group 17 SAE reported by 12 participants. Control group 13 SAE reported by 13 participants. (Not specified if due to treatment or otherwise.)
**Comparison: Comparison of different approaches ‐ neurophysiological approach compared with other (**Analysis 7.11)					
Epple 2020	20	12 (SAE+AE) Includes 1 death	20	13 (SAE+AE) Includes 2 deaths	Adverse events (AE) and serious adverse events (SAE) during the hospital stay and all deaths and SAE until day 90 were recorded and assessed by the investigators according to standard definitions. All AE and SAE were evaluated and forwarded to a medical expert for assessment of relatedness to the study treatment.
**Comparison: Other (see description)**					
Morreale 2016^a^ (Comparison of different neurophysiological approaches [not relevant for inclusion in analyses])	e‐PNF: 110 e‐CTE: 110	IEAs at 3 months: e‐PNF: 15 e‐CTE: 16	d‐PNF: 60 d‐CTE: 60	IEAs at 3 months d‐PNF: 11 d‐CTE: 13	Immobility‐related adverse events (IEAs) at 3 months: ALL IAEs: 15 ePNF group, 11 d‐PNF group. Pressure sores: 6 e‐PNF, 5 d‐PNF, Shoulder pain syndrome 1 e‐PNF, 4 d‐PNF, DVT 4 e‐PNF, 1 d‐PNF, infections 4 e‐PNF, 1 d‐PNF. 13 patients lost due to death at 3 months and a further 9 at 12 months follow‐up. 4 patients lost due to recurrence of stroke at 12 months.
Signal 2014 (Multiple comparisons: physical rehabilitation versus no physical rehabilitation, comparison of different approaches ‐ functional task training compared with other)	Strength and task‐specific training (STT) 5 Progressive resisted strength training (PRST) 5 Task‐specific training (TST) 5	STT: 1 PRST: 4 TST: 4	Usual care (UCC) 5	UCC: 0	AEs unrelated to intervention: STT group: minor 1. TST group: moderate 1. PRST group: minor 2, Serious 1. AEs related to intervention: TST group: minor 2, moderate 1. PRST group: moderate 1.

'Number in intervention group' and 'Number in control group' columns report the number of participants randomised to that group. *Number of adverse events columns show the number of adverse events occurring ‐ individual study participants may experience more than one event. ^a^These studies were not included in quantitative analyses for other outcomes (see Table for reasons). AE: adverse event APA‐Stroke: Adaptive Physical Activity exercise programme for stroke survivors CTE: cognitive therapeutic exercise e‐PNF: PNF (early rehabilitation) e‐CTE: cognitive therapeutic exercise (early rehabilitation) d‐PNF: PNF (standard) d‐CTE: cognitive therapeutic exercise (standard) IEAs: immobility‐related adverse events MTS+TSGT: task training + mobilisation and tactile stimulation PBT: perturbation‐based training PNF: proprioceptive neuromuscular facilitation PRST: progressive resisted strength training SAE: serious adverse event Sittercise: seated non‐progressive aerobic upper body exercise programme STT: strength and task‐specific training TI+TSGT: task training + insole TIA: transient ischaemic attack TST: task‐specific training UC: usual care

**2:** Sensitivity analyses: Physical rehabilitation versus no physical rehabilitation ‐ primary outcomes

	**Effect size (95% CI)**	**Number of participants (studies)**	**Comments**
**Activities of daily living (ADL)**			
**Main analysis result (****Analysis 1.1****)**	SMD 1.32 (1.08 to 1.56) I² = 93%	5403 (52)	Compared to no physical rehabilitation, physical rehabilitation improves ADL.
**Removal of studies with overall high risk of bias**	SMD 1.52 (1.12 to 1.91) I² = 94%	2189 (27)	Does not change conclusion from main analysis.
**Additional removal of studies with concerns relating to data entered^1^**	SMD 1.57 (1.17 to 1.97) I² = 94%	2169 (26)	Does not change conclusion from main analysis.
**Additional removal of all studies with ≥ 4 domains judged as some concerns**	SMD 0.60 (0.25 to 0.94) I² = 84%	876 (11)	Does not change conclusion from main analysis, although effect size reduces.
**Motor function**			
**Main analysis result (****Analysis 1.2****)**	SMD 1.01 (0.80 to 1.22) I² = 92%	5669 (50)	Compared to no physical rehabilitation, physical rehabilitation improves motor function.
**Removal of studies with overall high risk of bias**	SMD 1.04 (0.77 to 1.32) I² = 89%	2455 (27)	Does not change conclusion from main analysis.
**Additional removal of studies with concerns relating to data entered^2^**	SMD 1.05 (0.77 to 1.32) I² = 89%	2294 (26)	Does not change conclusion from main analysis.
**Additional removal of all studies with ≥ 4 domains judged as some concerns**	SMD 0.87 (0.41 to 1.33) I² = 91%	981 (12)	Does not change conclusion from main analysis.

^1^Additional studies removed: Aravind 2022 (estimated mean and SD). ^2^Additional studies to be removed: Green 2002 (estimated SD). (Also ‐ Fan 2006, Wu 2006, Zhu 2007 (these study results may overlap with Hu 2007) ‐ however, all already removed as judged as high ROB).

**Summary of findings 2:** Physical rehabilitation versus attention control ‐ immediate outcomes

**Physiotherapy intervention versus attention control for recovery after stroke**						
**Patient or population:** adults (> 18 years) with clinical diagnosis of stroke **Setting:** any **Intervention:** programmes of physical rehabilitation **Comparison:** attention control (dose matched, non‐physical, control intervention)						
**Outcomes**	**Relative effect**	**Anticipated absolute effects (95% CI)**		**No. of participants** **(studies)**	**Certainty of the evidence** **(GRADE)**	**Comments**
		**With attention control**	**With physical rehabilitation**			
**Primary outcome: Independence in ADL scales** Immediately after intervention (Higher number indicates a better outcome) Analysis 3.1	‐	See comment	SMD 0.91 higher (0.06 higher to 1.75 higher)	106 (2)	⊕⊝⊝⊝ **Very low**^a,b,c^	The evidence suggests that, compared to attention control, physical rehabilitation may improve activities of daily living, but the evidence is very uncertain. A standard deviation of 0.91 represents a large difference between groups.
**Primary outcome: Motor function scales** Immediately after intervention (Higher number indicates a better outcome) Analysis 3.2	‐	See comments	SMD 0.13 higher (0.13 lower to 0.38 higher)	237 (5)	⊕⊝⊝⊝ **Very low**^a,d,e^	The evidence suggests that, compared to attention control, physical rehabilitation may not improve motor function, but the evidence is very uncertain. A standard deviation of 0.13 represents a small difference between groups.
**Secondary outcome: Balance (Berg Balance Scale)** Immediately after intervention (Higher number indicates a better outcome) Analysis 3.3	‐	The mean score in the control group ranged from 26 to 52	MD 6.61 higher (0.45 lower to 13.66 higher)	240 (4)	⊕⊝⊝⊝ **Very low**^e,f^	The evidence suggests that, compared to attention control, physical rehabilitation may not improve balance, as measured by the Berg Balance Scale, but the evidence is very uncertain.
**Secondary outcome: Gait velocity** Immediately after intervention (Higher number indicates a better outcome) Analysis 3.4	‐	See comments	SMD 0.27 higher (0.01 lower to 0.55 higher)	474 (9)	⊕⊕⊕⊝ **Moderate**^e,g^	The evidence suggests that, compared to attention control, physical rehabilitation is likely to improve gait velocity^h^. A standard deviation of 0.27 represents a small difference between groups.
**Secondary outcome: Length of hospital stay** (Low number indicates a better outcome) Analysis 3.5	‐	The mean length of stay for the control group of the included study was 91.3 days	MD 33.0 lower (64.11 lower to 1.89 lower)	30 (1)	⊕⊝⊝⊝ **Very low**^c,i^	The evidence is very uncertain about the effect of physical rehabilitation on the length of hospital stay.
**Secondary outcome: Adverse events** (Any adverse events during the intervention period, including serious adverse events and falls, which were defined by study authors as possibly, probably, or definitely related to the study/intervention) Analysis 3.6; Table	RR 1.92 (0.75 to 4.93)	‐	‐	290 (3)	⊕⊝⊝⊝ **Very low**^d,i,j^	The evidence is very uncertain about the effect of physical rehabilitation on adverse events.
GRADE Working Group grades of evidence. **High certainty:** Further research is very unlikely to change our confidence in the estimate of effect. **Moderate certainty:** Further research is likely to have an important impact on our confidence in the estimate of effect and may change the estimate. **Low certainty:** Further research is very likely to have an important impact on our confidence in the estimate of effect and is likely to change the estimate. **Very low certainty:** We are very uncertain about the estimate. **Abbreviations:** ADL: activities of daily living; CI: confidence interval; MD: mean difference; RR: risk ratio; SMD: standardised mean difference						

^a^Downgraded once for indirectness, as the studies use different measures of outcome, with no/few studies using our preferred outcome measure. ^b^Downgraded once for inconsistency, as there is substantial heterogeneity. ^c^Downgraded twice for imprecision, as the data relate to only one/two small studies. ^d^Downgraded once for study limitations, as a large proportion of studies are judged to be at high risk of bias. ^e^Downgraded once for imprecision, as the data relate to a low number of small studies. ^f^Downgraded twice for inconsistency, as there is considerable heterogeneity, with a study where the confidence intervals do not overlap. ^g^No downgrade for heterogeneity as heterogeneity reduces (to I² = 0%) when only direct gait speed data are included. ^h^Although the main analysis result is SMD 0.27 (95% CI ‐0.01 to 0.55) (i.e. no benefit), this changes when only direct gait speed data are included (SMD 0.34, 95% CI 0.14 to 0.54; 7 studies, 405 participants), supporting the statement that evidence suggests that physical rehabilitation does improve gait speed. ^i^Downgraded once due to concerns about missing data from other studies, as this outcome is not reported in the majority of studies. ^j^Downgraded once as different studies gathered varied data relating to adverse events, and study authors reached different decisions about whether adverse events are potentially related to the study procedures or not.

**Summary of findings 3:** Additional physical rehabilitation plus usual therapy versus usual therapy only ‐ immediate outcomes

**Additional physical rehabilitation plus usual therapy versus usual therapy only for recovery after stroke**						
**Patient or population:** adults (> 18 years) with clinical diagnosis of stroke **Setting:** any **Intervention:** programme of physical rehabilitation, delivered in addition to usual therapy **Comparison:** usual therapy						
**Outcomes**	**Relative effect**	**Anticipated absolute effects (95% CI)**		**No. of participants** **(studies)**	**Certainty of the evidence** **(GRADE)**	**Comments**
		**Usual therapy only**	**Additional rehab plus usual therapy**			
**Primary outcome: Independence in ADL scales** Immediately after intervention (Higher number indicates a better outcome) Analysis 5.1	‐	See comments	SMD 1.26 higher (0.82 higher to 1.71 higher)	1972 (21)	⊕⊕⊝⊝ **Low**^a,b^	The evidence suggests that additional physical rehabilitation may improve activities of daily living. A standard deviation of 1.26 represents a large difference between groups.
**Primary outcome: Motor function scales** Immediately after intervention (Higher number indicates a better outcome) Analysis 5.2	‐	See comments	SMD 0.69 higher (0.46 to 0.92 higher)	1965 (22)	⊕⊕⊝⊝ **Low**^a,b^	The evidence suggests that additional physical rehabilitation may improve motor function. A standard deviation of 0.69 represents a moderate difference between groups.
**Secondary outcome: Balance (Berg Balance Scale)** Immediately after intervention (Higher number indicates a better outcome) Analysis 5.3	‐	The mean score in the usual therapy group ranged from 25.8 to 47.25	MD 5.74 higher (3.78 to 7.71 higher)	795 (15)	⊕⊕⊝⊝ **Low**^a,c^	The evidence suggests that additional physical rehabilitation may improve balance, as measured by the Berg Balance Scale.
**Secondary outcome: Gait velocity** Immediately after intervention (Higher number indicates a better outcome) Analysis 5.4	‐	See comments	SMD 0.59 higher (0.26 to 0.91 higher)	1004 (19)	⊕⊕⊝⊝ **Low**^a,d^	The evidence suggests that additional physical rehabilitation may improve gait velocity. A standard deviation of 0.59 represents a moderate difference between groups.
**Secondary outcome: Length of hospital stay** *Not reported*	‐	‐	‐	‐	‐	‐
**Secondary outcome: Adverse events** (Any adverse events during the intervention period, including serious adverse events and falls, defined by the study authors as possibly, probably, or definitely related to the study/intervention) Analysis 5.5; Table	RR 0.80 (0.64 to 0.98)	‐	‐	702 (4)	⊕⊝⊝⊝ **Very low**^e,f,g^	The evidence is very uncertain about the effect of additional physical rehabilitation on adverse events.
GRADE Working Group grades of evidence **High certainty:** Further research is very unlikely to change our confidence in the estimate of effect. **Moderate certainty:** Further research is likely to have an important impact on our confidence in the estimate of effect and may change the estimate. **Low certainty:** Further research is very likely to have an important impact on our confidence in the estimate of effect and is likely to change the estimate. **Very low certainty:** We are very uncertain about the estimate. **Abbreviations:** ADL: activities of daily living; CI: confidence interval; MD: mean difference; RR: risk ratio; SMD: standardised mean difference						

^a^Downgraded twice for inconsistency, as there is substantial heterogeneity and confidence intervals of some studies do not overlap. ^b^This finding does not change with sensitivity analyses to remove studies at high risk of bias, but does when studies at uncertain risk of bias are removed (Table). ^c^This finding does not change when studies at high risk of bias are removed (MD 4.65, 95% CI 2.45 to 6.86; 7 studies, 343 participants), or when studies at uncertain risk of bias are removed. ^d^This finding does not change when studies at high risk of bias are removed (SMD 0.49, 95% CI 0.17, 0.81; 11 studies, 417 participants), or when studies at uncertain risk of bias are removed. ^e^Downgraded once for study limitations as a large proportion of studies are judged to be at high risk of bias. ^f^Downgraded once due to concerns about missing data from other studies, as this outcome is not reported in the majority of studies. ^g^Downgraded once as data combine number lost to follow‐up with falls data and data relating to other adverse events during the intervention period.

**3:** Sensitivity analyses: Additional physical rehabilitation + usual therapy versus usual therapy only ‐ primary outcomes

	**Effect size (95% CI)**	**Number of participants (studies)**	**Comments**
**Activities of daily living (ADL)**			
**Main analysis result (****Analysis 5.1****)**	SMD 1.26 (0.82 to 1.71) I² = 95%	1972 (21)	Additional physical rehabilitation improves ADL.
**Removal of studies with overall high risk of bias**	SMD 1.31 (0.64 to 1.98) I² = 95%	946 (13)	Does not change conclusion from main analysis.
**Additional removal of studies with concerns relating to data entered^1^**	‐	‐	‐
**Additional removal of all studies with ≥ 4 domains judged as some concerns.**	SMD 0.71 (‐0.03 to 1.45) I² = 90%	394 (5)	Changes conclusion: There is no benefit with additional rehabilitation.
**Motor function**			
**Main analysis result (****Analysis 5.2****)**	SMD 0.69 (0.46 to 0.92) I² = 82%	1965 (22)	Compared to no physical rehabilitation, physical rehabilitation improves motor function.
**Removal of studies with overall high risk of bias**	SMD 0.68 (0.42 to 0.95) I² = 73%	956 (12)	Does not change conclusion from main analysis.
**Additional removal of studies with concerns relating to data entered^1^**	‐	‐	‐
**Additional removal of all studies with ≥ 4 domains judged as some concerns.**	SMD 0.18 (‐0.04 to 0.39) I² = 0%	338 (5)	Changes conclusion: There is no benefit with additional rehabilitation.

^1^ LAST 2018 (estimated SD & zeros imputed for missing data), but already removed due to high risk of bias judgement relating to missing outcome data. ADL = activities of daily living; CI = confidence interval; SMD = standardised mean difference

**Summary of findings 4:** Physical rehabilitation with a focus on functional task training versus another approach ‐ immediate outcomes

**Physical rehabilitation with a focus on functional task training versus another approach (with less/no functional task training) for recovery after stroke**						
**Patient or population:** adults (> 18 years) with clinical diagnosis of stroke **Setting:** any **Intervention:** physical rehabilitation with a focus on functional task training **Comparison:** equal dose of a different physical rehabilitation programme with less or no functional task training						
**Outcomes**	**Relative effect**	**Anticipated absolute effects (95% CI)**		**No. of participants** **(studies)**	**Certainty of the evidence** **(GRADE)**	**Comments**
		**Approach with less or no functional task training**	**Approach with a focus on functional task training**			
**Primary outcome: Independence in ADL scales** Immediately after intervention (Higher number indicates a better outcome) Analysis 7.1	‐	See comments	SMD 0.58 higher (0.29 higher to 0.87 higher)	1535 (22)	⊕⊕⊝⊝ **Low**^a,b^	Functional task training approaches may improve ADL more than other approaches. A standard deviation of 0.58 represents a moderate difference between groups.
**Primary outcome: Motor function scales** Immediately after intervention (Higher number indicates a better outcome) Analysis 7.2	‐	See comments	SMD 0.72 higher (0.21 higher to 1.22 higher)	1671 (20)	⊕⊝⊝⊝ **Very low**^a,b,c,d^	Functional task training approaches may improve motor function more than other approaches, but the evidence is very uncertain. A standard deviation of 0.72 represents a moderate difference between groups.
**Secondary outcome: Balance (Berg Balance Scale)** Immediately after intervention (Higher number indicates a better outcome) Analysis 7.3	‐	The mean score in the other group ranged from 17.16 to 54.1.	MD 2.16 higher (0.24 lower to 4.55 higher) Subgroup of 'Functional task training' versus 'Less functional task training': MD 2.56 higher (0.47 higher to 4.64 higher)	1194 (25) (subgroup result: 988 (19))	⊕⊝⊝⊝ **Very low**^a,b,c,e^	Functional task training approaches may not improve balance more than other approaches, but approaches with more functional task training may improve balance more than those with less functional task training. However, the evidence is very uncertain.
**Secondary outcome: Gait velocity** Immediately after intervention (Higher number indicates a better outcome) Analysis 7.4	‐	See comments	SMD 0.28 higher (0.01 lower to 0.56 higher)	1719 (27)	⊕⊝⊝⊝ **Very low**^a,b,c,e^	The evidence suggests that functional task training approaches may not improve gait velocity more than other approaches, but the evidence is very uncertain. A standard deviation of 0.28 represents a small difference between groups.
**Secondary outcome: Length of hospital stay** (Lower number indicates a better outcome) Analysis 7.5	‐	The mean length of stay for the control group of the included study was 16 days	MD 6.00 longer (0.69 longer to 11.31 longer)	75 (1)	⊕⊝⊝⊝ **Very low**^c,f^	There is uncertainty about the effect of functional task training, as compared to less functional task training, on the length of hospital stay.
**Secondary outcome: Adverse events** (Any adverse events during the intervention period, including serious adverse events and falls, which were defined by the study authors as possibly, probably or definitely related to the study/intervention) Analysis 7.6; Table	RR 1.33 (95% CI 0.91 to 1.94)	‐	‐	473 (6)	⊕⊝⊝⊝ **Very low**^g,h^	There is uncertainty about the effect of functional task training, as compared to less functional task training, on adverse events.
GRADE Working Group grades of evidence **High certainty:** Further research is very unlikely to change our confidence in the estimate of effect. **Moderate certainty:** Further research is likely to have an important impact on our confidence in the estimate of effect and may change the estimate. **Low certainty:** Further research is very likely to have an important impact on our confidence in the estimate of effect and is likely to change the estimate. **Very low certainty:** We are very uncertain about the estimate. **Abbreviations:** ADL: activities of daily living; CI: confidence interval; MD: mean difference; RR: risk ratio; SMD: standardised mean difference						

^a^Downgraded twice for inconsistency, as there is substantial heterogeneity and confidence intervals of some studies do not overlap. ^b^This finding does not change with sensitivity analyses to remove studies at high or uncertain risk of bias (Table). ^c^Downgraded once due to study limitations, as there are serious concerns about risk of bias for some studies. ^d^This finding does not change with sensitivity analyses to remove studies at high risk of bias, but when studies with high uncertainty are removed there is no longer a difference between functional task training and other approaches (Table). ^e^This finding does not change, but heterogeneity is reduced when studies at high risk of bias are removed. ^f^Downgraded twice for imprecision as there is only one small study. ^g^Downgraded once due to concerns about missing data from other studies, as this outcome is not reported in the majority of studies. ^h^Downgraded twice, as data combined serious, moderate, and mild adverse events, with different studies collecting different types of adverse event data.

**4:** Sensitivity analysis: One physical rehabilitation approach compared to another ‐ primary outcomes

	**Effect size (95% CI)**	**Number of participants (studies)**	**Comments**
**Functional task training versus less functional task training: Activities of daily living (ADL)**			
**Main analysis result (****Analysis 7.1****)**	SMD 0.58 (0.29 to 0.87) I² = 88%	1535 (22)	Functional task training approaches improve ADL more than approaches with less functional task training.
**Removal of studies with overall high risk of bias**	SMD 0.70 (0.38 to 1.03) I² = 85%	1173 (17)	Does not change conclusion from main analysis.
**Additional removal of studies with concerns relating to data entered^1^**	SMD 0.72 (0.38 to 1.07) I² = 86%	1123 (16)	Does not change conclusion from main analysis.
**Additional removal of all studies with ≥ 4 domains judged as some concerns.**	SMD 0.61 (0.34 to 0.88) I² = 6%	243 (6)	Does not change conclusion from main analysis.
**Functional task training versus less functional task training: Motor function**			
**Main analysis result (****Analysis 7.2****)**	SMD 0.72 (0.21 to 1.22) I² = 95%	1671 (20)	Functional task training approaches improve motor function more than approaches with less functional task training.
**Removal of studies with overall high risk of bias**	SMD 0.88 (0.31 to 1.45) I² = 96%	1137 (14)	Does not change conclusion from main analysis.
**Additional removal of studies with concerns relating to data entered^1^**	SMD 0.75 (0.31 to 1.19) I² = 96%	1087 (13)	Does not change conclusion from main analysis.
**Additional removal of all studies with ≥ 4 domains judged as some concerns.**	SMD 1.01 (‐0.39 to 2.42) I² = 6%	333 (5)	Changes result: There is no difference between functional task training approaches and other approaches at improving motor function. Heterogeneity is reduced.
**Neurophysiological approach versus other approach: Activities of daily living (ADL)**			
**Main analysis result (****Analysis 7.7****)**	SMD ‐0.34 (‐0.63 to ‐0.06) I² = 70%	737(14)	Neurophysiological approaches are less effective than other approaches at improving ADL.
**Removal of studies with overall high risk of bias**	SMD ‐0.40 (‐0.70 to ‐0.10) I² = 68%	611 (12)	Does not change conclusion from main analysis.
**Additional removal of studies with concerns relating to data entered^1, 2^**	SMD ‐0.42 (‐0.77 to ‐0.06) I² = 73%	522 (10)	Does not change conclusion from main analysis.
**Additional removal of all studies with ≥ 4 domains judged as some concerns.**	SMD ‐0.33 (‐0.66 to ‐0.01) I² = 0%	149 (4)	Does not change conclusion from main analysis. Heterogeneity is removed.
**Neurophysiological approach versus other approach: motor function**			
**Main analysis result (****Analysis 7.8****)**	SMD ‐0.60 (‐1.32 to 0.12) I² = 94%	663 (13)	There is no difference between neurophysiological approaches and other approaches at improving motor function.
**Removal of studies with overall high risk of bias**	SMD ‐0.80 (‐1.80 to 0.20) I² = 95%	431 (10)	Does not change conclusion from main analysis.
**Additional removal of studies with concerns relating to data entered^1^**	SMD ‐0.77 (‐1.90 to 0.37) I² = 87%	381 (9)	Does not change conclusion from main analysis.
**Additional removal of all studies with ≥ 4 domains judged as some concerns.**	SMD 0.07 (‐0.39 to 0.54) I² = 0%	72 (4)	Does not change conclusion from main analysis. Heterogeneity is removed.

^1^Arya 2019 (estimated mean and SD). ^2^Epple 2020 (estimated mean and SD). ADL = activities of daily living; CI = confidence interval; SD = standard deviation; SMD = standardised mean difference

**Summary of findings 5:** Physical rehabilitation with a focus on neurophysiological treatment versus another approach ‐ immediate outcomes

**Neurophysiological approach to physical rehabilitation versus another approach to physical rehabilitation for recovery after stroke**						
**Patient or population:** adults (> 18 years) with clinical diagnosis of stroke **Setting:** any **Intervention:** physical rehabilitation with a focus on neurophysiological treatment **Comparison:** equal dose of a different physical rehabilitation programme						
**Outcomes**	**Relative effect**	**Anticipated absolute effects (95% CI)**		**No. of participants** **(studies)**	**Certainty of the evidence** **(GRADE)**	**Comments**
		**Another approach**	**Approach with a focus on neurophysiological treatment**			
**Primary outcome: Independence in ADL scales** Immediately after intervention (Higher number indicates a better outcome) Analysis 7.7	‐	See comments	SMD 0.34 lower (0.63 lower to 0.06 lower)	737 (14)	⊕⊕⊝⊝ **Low**^a,b^	The evidence suggests that neurophysiological approaches may be less effective than other approaches at improving ADL. A standard deviation of 0.34 represents a small difference between groups.
**Primary outcome: Motor function scales** Immediately after intervention (Higher number indicates a better outcome) Analysis 7.8	‐	See comments	SMD 0.60 lower (1.32 lower to 0.12 higher)	663 (13)	⊕⊕⊝⊝ **Low**^a,b^	The evidence suggests that there may be no difference between neurophysiological approaches and other approaches in improving motor function. A standard deviation of 0.60 represents a moderate difference between groups.
**Secondary outcome: Balance (Berg Balance Scale)** Immediately after intervention (Higher number indicates a better outcome) Analysis 7.9	‐	The mean score in the other group ranged from 20.42 to 51.4	MD 0.06 lower (5.90 lower to 6.03 higher)	292 (9)	⊕⊕⊝⊝ **Low**^a,c^	The evidence suggests that there may be no difference between neurophysiological approaches and other approaches in improving balance.
**Secondary outcome: Gait velocity** Immediately after intervention (Higher number indicates a better outcome) Analysis 7.10	‐	See comments	SMD 0.17 lower (0.62 lower to 0.27 higher)	630 (16)	⊕⊝⊝⊝ **Very low**^a,d,e^	The evidence suggests that there may be no difference between neurophysiological approaches and other approaches in improving gait velocity, but the evidence is very uncertain.
**Secondary outcome: Length of hospital stay** *Not reported*	‐	‐	‐	‐	‐	‐
**Secondary outcome: Adverse events** (Any adverse events during the intervention period, including serious adverse events and falls, which were defined by the study authors as possibly, probably or definitely related to the study/intervention) Analysis 7.11; Table	Not estimable	‐	‐	40 (1)	⊕⊝⊝⊝ **Very low**^e,f^	The evidence is very uncertain about the effect of neurophysiological approaches, as compared to other approaches, on adverse events.
GRADE Working Group grades of evidence **High certainty:** Further research is very unlikely to change our confidence in the estimate of effect. **Moderate certainty:** Further research is likely to have an important impact on our confidence in the estimate of effect and may change the estimate. **Low certainty:** Further research is very likely to have an important impact on our confidence in the estimate of effect and is likely to change the estimate. **Very low certainty:** We are very uncertain about the estimate. **Abbreviations:** ADL: activities of daily living; CI: confidence interval; MD: mean difference; RR: risk ratio; SMD: standardised mean difference						

^a^Downgraded twice for inconsistency, as there is substantial heterogeneity and confidence intervals of some studies do not overlap. ^b^This finding does not change with sensitivity analyses to remove studies at high or uncertain risk of bias (Table). ^c^This finding does not change, but heterogeneity reduces when studies at high risk of bias are removed. ^d^Downgraded once due to study limitations, as there are serious concerns about risk of bias for some studies, and no studies are judged to have an overall low risk of bias. ^e^Downgraded twice for imprecision as there is only one small study, reporting 0 events. ^f^Downgraded once due to concerns about missing data from other studies, as this outcome is not reported in the majority of studies.

## Background

### Stakeholder involvement

This review update was conducted with the involvement of key stakeholders (stroke survivors, carers, and physiotherapists). Stakeholder involvement in the 2014 update of this Cochrane review informed the categorisation of physical rehabilitation treatment components within the review. This is described in the previous version of the review (Todhunter‐Brown 2014) and in an article describing the methods of stakeholder involvement (Pollock 2015). To ensure the continued relevance and accessibility of this review, for the current update, a new stakeholder group was convened.

Details of the stakeholder involvement are provided in Appendix 1 and referred to as appropriate within the review.

### Description of the condition

Worldwide, stroke is the leading cause of disability and the second leading cause of death (Feigin 2022). The most common and widely recognised impairment caused by stroke is motor impairment, which can be regarded as loss or limitation of function in muscle control or movement or limitation in mobility (Wade 1992a). Motor impairment after stroke typically affects the control of movement of the face, arm, and leg on one side of the body (Warlow 2008) and is the most common impairment after stroke (Clery 2020). Around two‐thirds of stroke survivors have initial limb impairment and/or mobility deficits (Clery 2020; Jorgensen 1995; Shaughnessy 2005), and six months after a stroke, more than 30% of survivors still cannot walk independently (Jorgensen 1995; Mayo 2002; Patel 2000). Recovery of mobility is commonly perceived as an unmet need by stroke survivors (Chen 2019; Lin 2021). Therefore, much of the focus of stroke rehabilitation, in particular the work of physiotherapists (also known as physical therapists or rehabilitation therapists), is focused on recovery of physical independence and functional ability during activities of daily living; commonly the ultimate goal of therapy is to improve the function of walking and recovery of balance and movement (Langhorne 2009; Winstein 2016).

### Physical rehabilitation 'approaches': historical context

Various approaches to physical rehabilitation can be used after stroke and, historically, the relative effectiveness of these approaches has been an important question (Carlisle 2010; Kollen 2009). Appendix 2 briefly summarises the historical context for physical rehabilitation approaches, and provides an overview of key 'named' approaches.

Previous versions of this review have concluded that "physical rehabilitation should not be limited to compartmentalised, named rehabilitation approaches, but should comprise clearly defined, well‐described, evidence‐based physical treatments regardless of historical or philosophical origin" (Todhunter‐Brown 2014). Thus, in line with recommendations arising from previous versions, this updated Cochrane review will primarily focus on the content of physical rehabilitation, regardless of any 'named' approach or historical origin.

### Physical rehabilitation: evidence‐based description

Since the last version of this review, the need for an evidence‐based description of 'rehabilitation' has been raised, with a lack of agreement and understanding of the meaning of rehabilitation highlighted (Negrini 2022; Wade 2020).

A recent Cochrane Rehabilitation project has developed a consensus‐based definition of rehabilitation for research purposes as a “*multimodal, person‐centered, collaborative process*” including interventions targeting a person’s “capacity (by addressing body structures, functions, and activities/participation) and/or contextual factors related to performance” (Negrini 2022).

Based on this definition of rehabilitation, the intervention of relevance to this review ‐ physical rehabilitation ‐ must comprise more than one different treatment component, delivered as a "programme" of rehabilitation. We use the phrase "physical" rehabilitation to indicate the focus on physical functioning and mobility.

The individual treatment components included within a programme of physical rehabilitation *may* vary between patients, as these may be selected according to an assessment of each patient's individual needs. However, some approaches to physical rehabilitation may be more structured, with all patients receiving the same or similar treatment components.

Thus, this Cochrane review is focussed on programmes of physical rehabilitation delivered to people who have had a stroke, with an aim of improving function and mobility. We are interested in whether different programmes of/approaches to physical rehabilitation are effective, and whether any particular approaches to physical rehabilitation are any more effective than other approaches.

We are interested in the *content* of the physical rehabilitation, rather than in the 'name' of the approach. We aim to explore this by considering the individual treatment components that make up physical rehabilitation and grouping similar 'approaches' together. Physical rehabilitation can be delivered in a number of different ways, such as one‐to‐one sessions, group classes, or self‐practice sessions. For this review, stakeholders developed a framework to describe the physical rehabilitation interventions within the studies included in this review: see Figure and Table. This framework was informed by the intervention taxonomy within the previous version of the review (Todhunter‐Brown 2014), papers around definitions and descriptions of rehabilitation (DeJong 2004; Hart 2014; Negrini 2022; Stucki 2007; Wade 2020), and the lived experience of the stakeholder group members (see Appendix 1).

**1:**
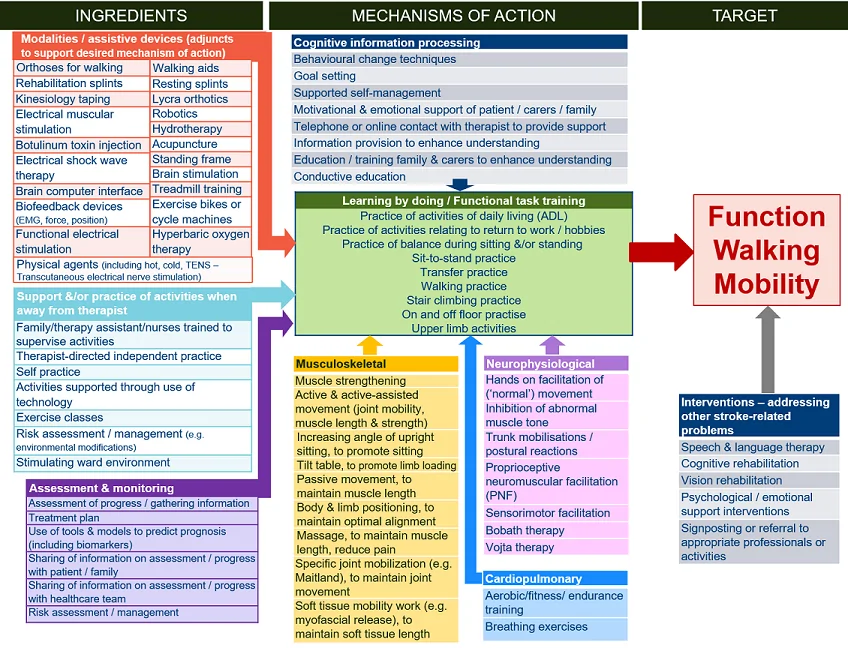
Framework for describing physical rehabilitation treatment components, co‐produced with stakeholder group. Definitions of terms: see Table

**5:** Physical rehabilitation intervention components

**Functional task training**	
Functional task training involves the active practice of real‐life tasks with the aim of acquiring (or reacquiring) a skill. The practice may involve both practice of the ‘whole’ task (e.g. walking) or ‘parts’ of the task (e.g. stepping). Support or assistance may be provided. This may also be called task‐specific training, task‐oriented training, or functional task practice.	
Component	Definition
**Activity of daily living (ADL) training**	Practice of tasks relevant to daily life, including both part, and whole, task practice.
**Sitting and/or standing balance training**	Various activities performed sitting and/or standing with the aim of improving the ability to balance safely and independently.
**Sit‐to‐stand practice**	Practice of tasks aimed at improving ability to stand up and sit down safely and independently.
**Transfer practice**	Practice of tasks aimed at improving ability to move from one position to another.
**Walking**	Practice of tasks aimed at improving ambulation.
**Stair climbing**	Practice of tasks aimed at ability to go up and down stairs.
**Upper limb function training**	Practice of tasks aimed at improving the ability to move and use the arm, such as reach, grasp, and hand‐to‐mouth activities. May include training to improve sensation.
**Activities relating to return to work or hobbies**	Task‐specific practice of activities which have been identified as important to the patient, often in relation to their job or hobbies, for example; typing, riding a bike, swimming.
**On‐and‐off the floor practice**	Practice of getting from the floor to a chair, or to standing, possibly as education as to what to do in the event of a fall.
Approaches (programmes which can involve any/all functional task training components):	
**Motor Relearning Programme (MRP)** ‐ An approach to physical rehabilitation based on movement science, aimed at recovery of functional movement. This may also be referred to as Motor Learning Approach. (Described in ‘A motor relearning programme for stroke’ by J Carr & R Shepherd, 2^nd^ Edition, 1987).	
**Musculoskeletal intervention (active)**	
Active musculoskeletal interventions involve the active participation of a patient in movements or exercises aimed at improving the function of the musculoskeletal system (i.e. bone, muscle and soft tissue function).	
Component	Definition
**Muscle strengthening**	Practice of activities to progressively increase the ability to generate and increase muscle force, including using body weight and external resistance
**Active and active‐assisted movement**	Moving a body part through its range of movement, under the patient’s active control with or without assistance, e.g. from a therapist, or by use of equipment.
**Musculoskeletal intervention (passive)**	
Passive musculoskeletal interventions involve placement of, or movement of, the patient or patient’s limbs with the aim of changing the musculoskeletal system (e.g. muscle or other soft tissue length). Placement or movement may be done by a person (e.g. therapist) or a device (e.g. using an orthotic).	
Component	Definition
**Increasing angle of upright sitting**	A form of positioning, to promote early sitting and the benefits associated with this; for example reducing respiratory complications and/or blood pressure.
**Tilt table**	To promote early lower limb loading and the benefits associated with this; for example reducing respiratory complications, maintaining muscle length.
**Passive movement**	Moving a limb through its range of movement, whilst the patient is passive.
**Body and limb positioning**	Placing a limb or body part in a supported position, to maintain optimal alignment.
**Massage**	Manipulation of soft tissue, using the hands or a tool designed for the purpose of massage.
**Specific joint mobilisation (e.g. Maitland)**	Passive movement of a joint, possibly using small rhythmic movements, to improve or reduce pain, or to maintain or improve movement at the joint
**Soft tissue work (e.g. myofascial release)**	A type of massage which stretches tight muscles and other soft tissues, to reduce pain, or to maintain or improve movement
**Cardiopulmonary interventions**	
Cardiopulmonary interventions are exercises, training or activities aimed at the cardiovascular and pulmonary system (systems involving heart, lungs and blood vessels). This may also be described as cardiopulmonary, cardiovascular or cardiorespiratory.	
Component	Definition
**Aerobic/fitness/endurance training**	Activities to improve cardiopulmonary fitness.
**Breathing exercises**	Exercises or training, aimed at improving respiratory function.
**Neurophysiological intervention**	
Interventions which are based on the function of the nervous system (which includes the brain, nerves and sensory organs), and may alter some of the functions this system. This includes approaches to treatment which are based on neurophysiological mechanisms and theories (e.g. Bobath Approach, Vojta therapy).	
Component	Definition
**Hands on facilitation of ('normal') movement**	Intervention which is described as facilitation of movement, often referenced to Bobath or Davies (see below).
**Inhibition of abnormal muscle tone/normalising tone**	Intervention which is described as inhibition of abnormal muscle tone or as normalising muscle tone, often referenced to Bobath or Davies (see below).
**Trunk mobilisations/postural reactions**	Intervention which is described as trunk mobilisations or postural reactions to perturbations. May be referenced to Bobath or Davies.
**Proprioceptive neuromuscular facilitation (PNF)**	PNF was developed by Kabat and Knott in the 1940s. The PNF approach uses the body’s proprioceptive system (sensory system which provides information about movement and position of body) to facilitate or inhibit muscle contractions. The approach includes a number of techniques based around patterns of muscle contraction and relaxation.
**Sensorimotor facilitation**	The use of excitatory techniques, such as brushing, striking, tapping, icing, to improve sensory awareness and promote muscle activity.
Approaches (programmes based on neurophysiological theories and mechanisms):	
**Bobath therapy or approach**: An approach to evaluation and treatment, described in published texts (e.g. Bobath 1978 ; Davies 1985) and taught in training programmes (e.g. https://bbta.org.uk/).	
**Vojta Therapy:** described as a neuromuscular treatment method, and was developed principally for use in children. Pressure is applied to specific points to cause an involuntary response and performance of a certain movement.	
**Modalities/assistive devices**	
A specific method of treatment which uses a tool, product or technological device, directly or indirectly aimed at assisting a person with a specific movement or task. These may provide physical assistance (e.g. an orthosis) or act to change body function (e.g. through the relief of pain, or reduction in muscle spasm).	
**Component**	**Definition**
**Walking aids**	Devices to assist walking, for example, sticks and frames.
**Orthoses for walking**	Externally applied orthoses to assist walking, for example, ankle foot orthosis (AFO), knee braces, shoe inserts.
**Resting splints**	Externally applied orthoses used, for example, to maintain or improve limb alignment or muscle length.
**Rehabilitation splints**	Externally applied orthoses to assist mobility or function.
**Lycra orthotics**	Externally applied ‘dynamic’ orthoses, which may be in a variety of forms including suits, vests, leggings or socks, designed to assist movement improve postural stability/balance, control and function.
**Functional electrical stimulation**	Application of an electrical current, via electrodes, to facilitate functional movement.
**Acupuncture**	A treatment derived from ancient Chinese medicine involving insertion of fine needs into certain sites in the body, delivered for either pain relief or movement therapy.
**Physical agents (including hot, cold, Transcutaneous Electrical Nerve Stimulation (TENS))**	Adjuncts, delivered for either pain relief or movement therapy.
**Electrical muscular stimulation**	Use of electrodes to deliver electrical charges which stimulate a muscle and cause it to contract.
**Botulinum toxin injection**	An injection into the muscle to reduce muscle spasm
**Electrical shock wave therapy**	Delivery of impulses of energy to reduce muscle spasticity
**Kinesiology taping**	Application of a specialised sports tape to assist muscle function, movement or relieve pain
**Robotics**	A range of automatically operated machines engineered to support, assist or replace movement of the joints and limbs
**Hydrotherapy**	Rehabilitation treatment in warm water (e.g. a pool).
**Treadmill training, with or without body weight support harness**	A device used for walking or running while staying in the same place. Body weight support uses a harness to suspend, or take some of the weight, of the person whilst they walk on a treadmill.
**Exercise bikes or cycle machines**	A device used for pedalling while staying in the same place. May be passive (legs are moved by the machine), assisted (the machine provides some assistance) or active (movement is by muscle power).
**Standing frame**	A device used to assist a person to stand, enabling other activities or exercises to be carried out in a safe way. Some frames may also move to allow walking activities, with a harness taking some weight, or preventing falling.
**Biofeedback devices (including electromyography (EMG), force, position)**	A machine which provides a person with information about their performance or movement. May provide a variety of types of feedback/information, such as EMG signals from muscle, force feedback and feedback about position/movement of body parts.
**Brain stimulation**	The use of electrodes to apply an electrical current to the brain. Includes transcranial Direct Current Stimulation (tDCS) and Transcranial Magnetic Stimulation (TMS).
**Brain computer interface training**	A computer‐based system which creates a direct pathway between the brain’s electrical activity and an external device which is designed to carry out a movement or response.
**Hyperbaric oxygen therapy**	The use of a specialised pressure chamber, or room, to increase the level of oxygen which is breathed, with the goal of increasing the supply of oxygen to parts of the brain, reducing brain damage caused by stroke and improving patient’s outcomes.
**Cognitive information processing**	
Cognition is “the mental action or process of acquiring knowledge and understanding through thought, experience and the senses” (World Health Organization). Cognitive information processing involves mental activity to focus on information and to inform behaviour. Processes may involve perceiving, analysing, using and remembering information.	
Component	Definition
**Behavioural change techniques**	A strategy that helps someone change their behaviour to engage better with rehabilitation
**Goal setting**	The act of selecting one, or several, target(s) or objective(s) to achieve, by – or in partnership with – a patient. Goals may be short or long term, and may adapt over time.
**Supported self‐management**	The steps to supporting people manage their life after stroke, including their limitations and coaching to achieve their rehabilitation potential. Steps may involve different people or services, for example patients, family members, health service providers. A wide range of activities may be addressed and progressed as part of supported self‐management.
**Motivational & emotional support of patient/carers/family**	Provision of encouragement, empathy, care and/or reassurance, and ambition aimed at helping someone continue with their rehabilitation
**Telephone or online contact with therapist to provide support**	Telephone or video‐call between patient and therapist with the aim of providing advice, motivation and/or emotional support. May involve specialist telephone/video/telehealth platforms.
**Information provision to enhance understanding**	Provision of information to someone who has had a stroke about stroke or stroke rehabilitation. Information may be in a variety of formats and adapted to individual needs.
**Education/training of family and carers to enhance understanding**	Provision of information, or practical teaching of skills and techniques, to family or carers of stroke survivors, related to stroke rehabilitation. Information may be in a variety of formats and adapted to individual needs.
Approaches (programmes based on cognitive information processing theories and techniques):	
**Conductive Education:** a method of learning designed specifically for individuals with neurological and mobility impairment by using normal life experiences. Stroke survivors are supported and guided to learn how to solve movement problems, develop skills and confidence and find ways to move in a more optimal way.	
**Support and/or practice of activities when away from therapist**	
The patient practices rehabilitation tasks or activities when they are away from the therapist, but with some form of support. Support could be in a variety of formats, such as information, a physical device, a change to an environment or from another person(s). This could involve practice to learn new skills and techniques or to maintain current function and abilities.	
Component	Definition
**Family/therapy assistant/nurses trained to supervise activities**	A stroke survivor is able to practice exercises or activities when away from the therapist because another person (such as a family member, carer, assistant or nurse) has been trained to assist or supervise, to ensure the exercises or activities are done correctly and safely.
**Therapist‐directed independent practice**	Individualised tasks to practise are provided/taught by therapist. A therapist teaches a stroke survivor specific tasks, activities or exercises that they can safely perform when away from the therapist.
**Self‐practice**	A stroke survivor has tasks, activities or exercises to do when away from the therapist, but these are ‘general’ exercises, rather than exercises specifically designed for that patient. May include: standardised exercise sheet, instructional videos, phone App, or verbal encouragement to practice tasks, without individualised instructions.
**Activities supported through use of technology**	A stroke survivor made able, with or without assistance, to use a technological device to carry out an activity or exercise. For example, walking on a treadmill, playing video games (e.g. using a Wii) to encourage balance or arm movement.
**Exercise classes**	A stroke survivor is able to attend a community or gym‐based exercise class, by being prescribed or signposted to a class. Classes may include yoga, pilates or general exercise. Generally this is a class for the general population rather than specifically for stroke survivors, although some adaptations may be made by the instructor.
**Risk assessment/management (e.g. environmental modifications)**	Aimed at provision of a safe environment for practice of daily living activities or exercise. Following assessment, changes are made to the environment within which the stroke survivor is in, aimed at supporting increased safe participation in activities or exercises.
**Stimulating Ward Environment**	Strategies aiming to ensure that the ward environment in which the patient is cared for provides the optimal level of stimulus to enable their recovery.
**Assessment and monitoring**	
The gathering of information about patient progress, and systematic use of this information to inform decisions (e.g. to inform treatment plans).	
Component	Definition
**Assessment of progress/gathering information**	A process of use of assessment tools and measurements to gather information about progress during rehabilitation. May be used to support the setting of targets/goals.
**Treatment plan**	An outline of intended goals and treatment plans, updated on a regular basis and/or when required.
**Use of tools and models to predict prognosis (including biomarkers which measure brain structure or function)**	A process of use of assessment tools, measurements and models to predict recovery and inform prediction of prognosis, including estimated timescales for recovery. Biomarkers may include measure of brain structure or injury (e.g. computed tomography (CT) scan) or measures of function (e.g. electroencephalography (EEG)).
**Sharing of information on assessment/progress with patient/ family**	A process or system of sharing information (including results of assessments and measurements) with the patient, their family members and carers.
**Sharing of information on assessment/progress with healthcare team**	A process or system of sharing information (including results of assessments and measurements) with other members of the healthcare team.
**Risk assessment/management**	Aimed at provision of an appropriate and safe environment for assessment and rehabilitation activities.
**Interventions – addressing other stroke‐related problems (including impairments, functional limitations, participation and inclusion)**	
Type of intervention	Definition
**Speech and language therapy**	Intervention aimed at improving communication, often in stroke survivors with aphasia (a difficulty speaking or understanding words), but also for problems such as dysarthria (weakness in muscles affecting speech) or speech apraxia (co‐ordination of words).
**Cognitive rehabilitation**	Intervention aimed at improving cognitive abilities, or supporting people develop compensatory strategies to deal with cognitive impairment. Includes interventions aimed at memory, neglect, executive function, and other cognitive impairments.
**Vision rehabilitation**	Intervention aimed at improving ability to see, or supporting people develop compensatory strategies to deal with visual impairment. Includes interventions aimed at visual field loss and eye movement disorders.
**Psychological/emotional support interventions**	Intervention aimed at providing a person, and/or their family members or carers, with support, advice or strategies to deal with the psychological and emotional consequences of stroke.
**Occupational therapy/vocational rehabilitation**	Intervention or support provided to enable inclusion and participation in all aspects of life.
**Signposting or referral to appropriate professionals or activities**	Action to put stroke survivor, their family or carers, in touch with other professionals, service providers or groups to provide appropriate support for impairments and problems relating to stroke. For example, this could include peer support groups or organisations or individuals who provide financial advice.

### How the intervention might work

Mechanisms of recovery from motor impairment and loss of function resulting from a stroke can be complex (Langhorne 2009). The International Classification of Functioning, Disability and Health (ICF) framework identifies that loss of functioning may involve impairments, activity limitations, and participation restrictions. Interventions that target motor recovery may therefore work through a number of different, inter‐related mechanisms (Levin 2009), and may target the health condition, body function/structure or activity, or a combination of these.

Interventions may aim to change a person's:

- Health condition. This could be through recovery/restoration of function in brain areas affected by the stroke, or activation of alternative areas of neural tissue so that other areas of the brain take on a new function ('compensation').
- Body functions and structures. For example, through recovery of muscle strength or soft tissue length, through use of alternative movement patterns ('adaptation') or by using assistive aids or devices to replace or support impaired body function to complete a task ('substitution').
- Ability to carry out an activity. For example, by re‐learning movement patterns or by learning to perform the task in a new way, or by using different techniques.

As described above, physical rehabilitation comprises a programme of treatment components, often selected according to a patient's individual needs (Negrini 2022). How each of these treatment components may work can vary, according to the target of the rehabilitation. For example, treatment components based on neurophysiological principles and comprising normal movement patterns may work through neuroplastic changes and recovery of brain function; treatment components involving the training or practice of a specific task or function may work through the (re) learning of movement patterns; treatment components involving muscle strengthening or stretching may work by changing body function and structures. The use of modalities and assistive devices (for example, orthotics, walking sticks, electrical stimulation, biofeedback, robotic devices) may work by supporting or facilitating an impaired function and enabling the execution of a movement. The effectiveness of physical rehabilitation may be enhanced by strategies (e.g. goal‐setting) that support or encourage motivation and/or behaviour change, and by the provision of opportunities for practice of movement or activities when away from the therapist (e.g. by providing exercise sheets, or by teaching family members to assist).

### Why it is important to do this review

Identifying the best ways of improving mobility and walking after stroke remains a top priority of stroke survivors (Hill 2022; Leitch 2023; Rudberg 2021).

Continued debate about the relative effectiveness of physical rehabilitation approaches and evidence of clear preferences for particular named approaches in some parts of the world, despite increasing calls for this to change, justify the importance of this review.

National guidelines are inconsistent in their recommendations. For example, a 2023 update of the UK National Clinical Guidelines for Stroke concludes that "Exercise and repetitive task practice should be the principal rehabilitation approaches, in preference to other therapy approaches including Bobath" (UK Stroke Guidelines 2023); while, in contrast, the 2022 Evidence‐Based Review of Stroke Rehabilitation, supported by the Canadian Partnership for Stroke Recovery, concludes that evidence remains mixed in relation to the relative effectiveness of motor relearning programmes and the Bobath approach (EBRSR 2022); the Australian and New Zealand Living Clinical Guidelines for Stroke Management do not make any specific recommendations relating to physical rehabilitation approaches (Stroke Foundation 2022).

Since the publication of the last version of this review (Todhunter‐Brown 2014), a number of other reviews have concluded that the Bobath (or neurodevelopment) approach is not more effective, and may be less effective, than other interventions (Díaz‐Arribas 2020; Pathak 2021; Scrivener 2020). New trials exploring the effectiveness of different approaches to rehabilitation continue to be planned and conducted around the world, including multiple trials exploring the effectiveness of the Bobath approach/neurodevelopmental approach (e.g. CTRI/2022/06/043037 2022; NCT04816929 2021) and task‐specific training/motor re‐learning approach (e.g. CTRI/2018/10/016163 2018; Ghrouz 2022; Traxler 2021), and trials directly comparing these approaches (e.g. NCT05425082 2022). New trials continue to explore the effectiveness of other 'named' approaches, such as Vojta therapy (e.g. Epple 2020), conductive education (e.g. Nagy 2017), and lower‐limb constraint‐induced therapy (e.g. Menezes‐Oliveira 2021; NCT04757467 2021; NCT05191524 2022). This growing body of new trials demonstrates the continued importance of this review and justifies the need for an update.

## Objectives

**Primary objective:** To determine whether physical rehabilitation is effective for recovery of function and mobility in people with stroke, and to assess if any one physical rehabilitation approach is more effective than any other approach.

For this update of the review, we sought to answer four key questions:

1. Is physical rehabilitation more effective than no (or minimal) physical rehabilitation?
2. Is physical rehabilitation more effective than attention control?
3. Does additional physical rehabilitation*, delivered as an adjunct to 'usual' or 'conventional' rehabilitation, improve outcomes?
4. Is any one approach to physical rehabilitation more effective than any other approach to physical rehabilitation?

**Secondary objective:** To explore factors that may impact the effectiveness of physical rehabilitation approaches. These include: time after stroke, geographical location of the study, dose and duration of the intervention, provider of the intervention and treatment components included within an intervention.

**Stakeholder involvement:** To ensure the relevance and accessibility of this review. Key aims of stakeholder involvement were to clarify the focus of the review, inform decisions about subgroup analyses, and co‐produce statements relating to key implications arising from the review. Physical rehabilitation was described using a co‐produced framework for categorising physical rehabilitation treatment components (see Figure and Table).

*We were only interested in 'additional' physical rehabilitation where the approach to physical rehabilitation provided as 'additional' therapy differed from that of the 'usual' or 'conventional' rehabilitation. Questions about dose/amount of therapy (where the approach to therapy was the same, but the amount differed) are addressed in another Cochrane review (Clark 2021).

## Methods

### Criteria for considering studies for this review

#### Types of studies

We included controlled trials if the participants were randomly assigned to one of two or more treatment groups. Random assignment gives each participant entering the trial the same, predetermined chance of receiving each of the possible treatments (e.g. by using sequentially numbered, opaque, sealed envelopes, or computer‐generated random numbers). We included trials with or without blinding of participants, physiotherapists, and assessors. We excluded trials with quasi‐random assignment, as these are at higher risk of introducing bias, with greater likelihood of baseline differences between participants.

#### Types of participants

We included trials enrolling adult participants (over 18 years of age) with a clinical diagnosis of stroke (World Health Organization (WHO) definition; Hatano 1976), which could be ischaemic or haemorrhagic in origin (confirmation of the clinical diagnosis by imaging was not compulsory). Where trials enrolled a mixed population (e.g. participants with stroke plus participants with other acquired brain injuries), we included such trials with only a subset of eligible participants only if the data for participants with stroke were available separately. If the only data available were for a mixed population, we excluded the trial.

#### Types of interventions

We included physical rehabilitation approaches that were aimed at promoting recovery of function and mobility. This included interventions that were specifically aimed at improvement of walking, or functions relating to walking (e.g. standing balance, stepping), as well as interventions that had a more generalised stated aim, such as improving functional ability, where this included walking ability.

We excluded rehabilitation approaches that were primarily aimed at promoting recovery of upper limb movement or upper limb function, or which were focussed on a specific function (e.g. sit‐to‐stand) or training of a specific body part (e.g. trunk training). In line with definitions of rehabilitation (Negrini 2022), we excluded studies that explored the effectiveness of a single component (only), but we included studies in which these components were part of a wider programme of rehabilitation (e.g. electrical stimulation, treadmill training, balance platform training, robotic devices). Further, to avoid duplication, we excluded studies that explored the effectiveness of specific programmes of physical rehabilitation that have been addressed within other Cochrane reviews (e.g. Tai Chi, yoga). We also excluded studies that were specifically focussed on the mode of delivery (e.g. remote/telerehabilitation, circuit classes); this included studies focussed on the amount, or dose, of physical rehabilitation as we were interested in the approach to physical rehabilitation, rather than the amount of (the same type of) therapy. Table provides details of interventions that are excluded from this review, with justifications for these decisions.

**6:** Exclusion criteria: studies of specific interventions not included in this review

**Intervention/topic excluded from this review**	**Reason for exclusion**	**Cochrane review reference: title**
**Studies focussed on a modality/assistive device**		
Acupuncture	Not a physical rehabilitation approach. Covered by a Cochrane review.	Yang 2016: Acupuncture for stroke rehabilitation
Electrostimulation	A device, not a physical rehabilitation approach. Covered by a Cochrane review.	Pomeroy 2006: Electrostimulation for promoting recovery of movement or functional ability after stroke
Virtual reality	A device, not a physical rehabilitation approach. Covered by a Cochrane review.	Laver 2017: Virtual reality for stroke rehabilitation
Magnetic stimulation; direct current stimulation	A device, not a physical rehabilitation approach. Covered by other Cochrane reviews.	Kamo 2022: Repetitive peripheral magnetic stimulation for impairment and disability in people after stroke Hao 2013: Repetitive transcranial magnetic stimulation for improving function after stroke Elsner 2020: Transcranial direct current stimulation (tDCS) for improving activities of daily living, and physical and cognitive functioning, in people after stroke
Force feedback and/or biofeedback devices	A device, not a physical rehabilitation approach. Covered by other Cochrane reviews.	Barclay‐Goddard 2004: Force platform feedback for standing balance training after stroke Woodford 2007: EMG biofeedback for the recovery of motor function after stroke
Orthotics and prosthetics	A device, not a physical rehabilitation approach. Covered by other Cochrane reviews.	Shrivastava 2014: Ankle foot orthosis for walking in stroke rehabilitation Mendes 2020: Motor neuroprosthesis for promoting recovery of function after stroke
Treadmill training	A device, not a physical rehabilitation approach. Covered by a Cochrane review.	Mehrholz 2017: Treadmill training and body weight support for walking after stroke.
Robotic devices	A device, not a physical rehabilitation approach. Covered by a Cochrane review.	Mehrholz 2020: Electromechanical‐assisted training for walking after stroke
**Studies focussed on a 'single' rehabilitation treatment or exercise approach**		
Yoga	Exercise approach, rather than approach to rehabilitation after stroke. Covered by a Cochrane review.	Lawrence 2017: Yoga for stroke rehabilitation
Tai Chi	Exercise approach, rather than approach to rehabilitation after stroke. Covered by a Cochrane review.	Zhang 2018: Tai Chi for improving recovery after stroke
Motor imagery	A 'single' treatment component which can be incorporated into a rehabilitation programme.	Silva 2020: Motor imagery for gait rehabilitation after stroke
Mirror therapy	A 'single' treatment component which can be incorporated into a rehabilitation programme.	Thieme 2018: Mirror therapy for improving motor function after stroke
Overground gait training	A 'single' treatment component which can be incorporated into a rehabilitation programme.	States 2009: Overground physical therapy gait training for chronic stroke patients with mobility deficits
Repetitive task training	A 'single' treatment component which can be incorporated into a rehabilitation programme.	French 2016: Repetitive task training for improving functional ability after stroke.
Water‐based exercises	Exercise approach, rather than approach to rehabilitation after stroke. Covered by a Cochrane review.	Mehrholz 2011: Water‐based exercises for improving activities of daily living after stroke
Strength training	A 'single' treatment component which can be incorporated into a rehabilitation programme.	Saunders 2020: Physical fitness training for stroke patients
**Studies focussed on mode of delivery of rehabilitation**		
Telerehabilitation	Focus is on mode of delivery, rather than approach to physical rehabilitation. Covered by a Cochrane review.	Laver 2020: Telerehabilitation services for stroke
Time spent in rehabilitation	Focus is on mode of delivery, rather than approach to physical rehabilitation. Covered by a Cochrane review.	Clark 2021: The effect of time spent in rehabilitation on activity limitation and impairment after stroke
Caregiver‐mediated exercise	Focus is on personnel, and on exercise rather than rehabilitation. Covered by a Cochrane review.	Vloothuis 2016: Caregiver‐mediated exercises for improving outcomes after stroke
Circuit class	Focus is on mode of delivery, rather than approach to physical rehabilitation. Covered by a Cochrane review.	English 2017: Circuit class therapy for improving mobility after stroke
**Target of rehabilitation**		
Upper limb	Not the focus of this review. Covered by multiple Cochrane reviews.	Pollock 2014: Interventions for improving upper limb function after stroke
Sit‐to‐stand	Focus is on single function, rather than wider programme of physical rehabilitation	Pollock 2014b: Interventions for improving sit‐to‐stand ability following stroke
Trunk	Focus is on single part of body, rather than wider programme of physical rehabilitation	Thijs 2023: Trunk training following stroke

During the screening of potential studies from Chinese databases for this review update, we identified that there are now hundreds of randomised controlled trials (RCTs) exploring the effectiveness of programmes of stroke rehabilitation that include a physical rehabilitation element as part of a broader post‐stroke rehabilitation programme. These are often described using phrases such as 'comprehensive nursing intervention', 'systematic rehabilitation nursing', 'stroke unit nursing', or 'rehabilitation nursing training', and often described as 'early' interventions (e.g. 'early rehabilitation nursing'). The interventions were multi‐faceted, involving speech and language and psychological therapy, as well as physical therapy and training to enhance function during activities of daily living. These trials often included measures of motor function and activities of daily living. The review authors made the decision that (i) it was not practical to include this large body of evidence within this review and (ii) the studies exploring the effectiveness of these multi‐faceted rehabilitation programmes do not directly address the research question addressed by this Cochrane review, which is specifically focussed on the effect of different approaches to *physical* rehabilitation. We consider that these trials would best be synthesised within a separate (new) Cochrane review.

#### Types of outcome measures

Eligible studies were included regardless of the outcomes reported.

##### Primary outcomes

We defined primary outcomes as measures of disability (activity limitations; WHO 2002; WHO 2022) and the relevant measures are as follows.

- Independence in activities of daily living (ADL) scales.
  - These include Barthel Activities of Daily Living Index (Mahoney 1965), Functional Independence Measure (FIM) (Keith 1987), Modified Rankin Scale (van Swieten 1988), Katz Index of Activities of Daily Living (Katz 1970), and Rehabilitation Activities Profile (van Bennekom 1995).
- Motor function scales.
  - These include Motor Assessment Scale (MAS) (Carr 1985), Fugl‐Meyer Assessment (lower limb section) (Fugl‐Meyer 1975), Rivermead Mobility Index (Forlander 1999), and Rivermead Motor Assessment (Lincoln 1979).

Where studies reported more than one relevant measure, we used the one listed first in the lists above.

##### Secondary outcomes

- Balance (Berg Balance Scale) (Berg 1989; Berg 1992).
- Gait velocity.
  - We included measures of gait speed (a measure of distance/time), a timed walk (a measure of time to walk a set distance), a Timed Up and Go Test, or any other measure directly relating to gait velocity. Where studies reported more than one of these, we used the first from this list.
  - Where gait velocity was measured in different conditions (e.g. at comfortable and fast speeds), we used comfortable speed.
  - We accepted measures where participants used assistive devices.
  - The six‐minute walking test (6MWT), or other measures of distance walked in a set time, generally considered measures of exercise tolerance or fitness, were classed as 'other measures'.
- Length of hospital stay.
- Adverse events, as reported by the trial investigators.
  - We collected any data relating to any adverse events, including serious adverse events and falls, that were defined by the study authors as possibly, probably, or definitely related to the study/intervention.

We were interested in outcomes that were assessed both immediately after the end of an intervention period ('immediate outcome') and at a follow‐up period ('persisting outcomes').

### Search methods for identification of studies

We searched for trials in all languages and arranged translation of relevant papers where necessary.

#### Electronic searches

For this update, the search strategies used for this review have been significantly revised and updated to account for newly identified relevant terms and to improve sensitivity and specificity. All discontinued versions of the search strategies used are still available in the previous version of this review (Todhunter‐Brown 2014)

We searched the Cochrane Stroke Specialised Register (an international register of stroke trials that was maintained until April 2021), using a comprehensive search strategy for retrieval of references on stroke health care (Cheyne 2020), and the following electronic databases on 24 November 2022:

- Cochrane Central Register of Controlled Trials (CENTRAL; 2022, Issue 10 of 12) in the Cochrane Library; Appendix 3);
- MEDLINE Ovid (from 1946 to 24 November 2022; Appendix 4);
- Embase Ovid (from 1974 to 24 November 2022; Appendix 5);
- CINAHL EBSCO (Cumulative Index to Nursing and Allied Health Literature; from 1937 to 24 November 2022; Appendix 6);
- AMED (Allied and Complementary Medicine; from 1985 to 24 November 2022; Appendix 7);
- Chinese Biomedical Literature Database (CBM; sinomed.imicams.ac.cn; last searched 22 February 2022; Appendix 8).

The search strategies utilised in this update have been comprehensively updated since the previous version of this review was published to account for newly identified relevant unqualified terms and controlled vocabulary terms. The search strategy includes the Cochrane Highly Sensitive Search Strategy for identifying randomised trials in MEDLINE Ovid: sensitivity‐maximising version (2008 revision), as described in Lefebvre 2024, and Cochrane Stroke's search strategies for the identification of 'stroke' studies in respective databases and other resources. These are supplemented with strategies to identify exercise interventions. The MEDLINE Ovid search was translated into Simplified Chinese and adapted for use in the CBM interface; the decision to search CBM was made as more than half of the relevant studies in the last version of this review had been conducted in China.

Additionally, we used Cochrane’s Screen4Me workflow to help assess the search results. Screen4Me comprises three components: known assessments – a service that matches records in the search results to records that have already been screened in Cochrane Crowd and been labelled as an RCT or as Not an RCT; the RCT classifier (a machine learning model that distinguishes RCTs from non‐RCTs); and, if appropriate, Cochrane Crowd (Cochrane’s citizen science platform where the Crowd help to identify and describe health evidence) (Marshall 2018; McDonald 2017; Noel‐Storr 2018; Noell‐Storr 2021; Thomas 2017).

#### Searching other resources

We screened the reference lists of all trials found using the above search methods.

In order to identify other published, unpublished, and ongoing studies, we searched for ongoing studies using the following registries (Appendix 9) on 24 November 2022:

- US National Institutes of Health Ongoing Trials Register ClinicalTrials.gov (clinicaltrials.gov/);
- World Health Organization International Clinical Trials Registry Platform (ICTRP; trialsearch.who.int).

We also searched REHABDATA (National Rehabilitation Information Center) on 24 November 2022 (https://www.naric.com/?q=en/REHABDATA; Appendix 10).

We contacted researchers of all ongoing studies to check publication status. If required for clarification, we contacted study authors to obtain additional information or clarification on potentially relevant studies.

### Data collection and analysis

#### Selection of studies

We used Covidence to manage the selection of studies. Title and abstract screening was conducted independently by two review authors (from ATB, CS, PC, GB, JM, PLC). The translate to English function was used for abstracts published in languages other than English. Disagreements were resolved through discussion (between ATB and either CS or GB).

Full‐text review of all English language papers, and all papers where full texts could be satisfactorily translated into English using Google Translate, was conducted independently by two review authors (ATB and CS). Disagreements were resolved through discussion (with GB for English‐language papers or PLC for Chinese‐language papers).

Full‐text review of Chinese language papers that could not be satisfactorily translated into English using Google Translate was conducted through discussion with one review author (PLC) translating relevant sections and verbally providing information to other review authors in English (AP, PC).

#### Data extraction and management

One review author extracted study characteristics from study reports into a pre‐piloted standardised form and a second author checked the extracted study characteristics (involving CS, JC, PC, ATB). Outcome data were extracted independently by two review authors (CS, ATB); one extracted data into a pre‐piloted standardised form and the other extracted data directly into RevMan 2024, and data were compared to identify any discrepancies. The review authors resolved disagreements by discussion, involving a third review author if necessary.

Data extracted included the following (when possible): study aim; trial setting (e.g. hospital, community); geographical location; details of participants (side of hemiplegia, type of stroke, time since stroke); health equity (using PROGRESS domains, O'Neill 2014); inclusion and exclusion criteria; all assessed outcomes; intervention details; funding and conflicts of interest. The review authors resolved disagreements by discussion and contacted study authors for clarification when necessary. For papers published in the Chinese language, one review author (PLC) verbally translated relevant sections of text, during an audio‐recorded meeting with a second author (PC), who documented relevant data extraction items. For papers published in languages other than English or Chinese, we sought a relevant translation of the paper.

Extracted data were managed in Excel. We categorised all studies according to whether they had data suitable for inclusion within quantitative analysis, or were included in qualitative synthesis only; and the nature of the study comparison. We categorised each study comparison with consideration of the dose of delivered interventions, as follows:

- Physical rehabilitation versus no physical rehabilitation or versus limited physical rehabilitation (dose differs).
  - Limited physical rehabilitation was defined as being a dose of < 50% of the treatment group.
- Physical rehabilitation versus attention control (dose of physical rehabilitation differs between groups; dose of intervention equivalent in both groups).
- Additional physical rehabilitation plus usual therapy versus usual therapy.
- Comparison of different physical rehabilitation approaches (dose equivalent).
  - This was further subcategorised according to whether the treatment groups were receiving any other physical rehabilitation (i.e. both groups may have received some 'standard' intervention).

These categories informed the analyses in which studies were included (for studies included in quantitative analyses).

#### Assessment of risk of bias in included studies

For this update, we used Cochrane's new risk of bias tool (RoB 2) (Higgins 2023a) and the Excel tool to implement RoB 2 (available at www.riskofbias.info/welcome/rob-2-0-tool). We assessed the risk of bias for all studies included in meta‐analyses for our primary and secondary outcomes (see Types of outcome measures):

- Independence in activities of daily living ‐ 'immediate' and 'persisting' time points
- Motor function ‐ 'immediate' and 'persisting' time points
- Balance ‐ 'immediate' time point only
- Gait velocity ‐ 'immediate' time point only
- Length of stay ‐ 'immediate' time point only
- Adverse events ‐ 'immediate' time point only

Assessment of risk of bias was conducted independently by two authors (ATB, JC). For the assessment of risk of bias in studies that were included in the previous review version, the two review authors (ATB, JC) independently assessed risk of bias, and resolved any disagreements through discussion. During this time, notes were taken to guide decisions relating to different sets of circumstances/trial details. Subsequently, the two review authors conducted assessments for all newly included trials (ATB, JC), and one author entered the data into RevMan (ATB). Where there were disagreements that could be resolved by referring to the notes collected from the previous review version or by checking study reports, this was performed by one author (ATB); where necessary, disagreements were discussed between two authors.

We used the RoB 2 tool to consider the risk of bias in the following domains:

1. Bias arising from the randomisation process.
2. Bias due to deviations from intended interventions.
3. Bias due to missing outcome data.
4. Bias in measurement of the outcome.
5. Bias in selection of the reported result.

We investigated the effect of assignment to intervention at baseline, regardless of whether the interventions were received as intended or not. For each of the five domains, using the Excel tool, we answered all signalling questions with response options 'Yes', 'Probably Yes', 'Probably No', 'No', or 'No Information', and recorded reasons within the free‐text boxes. We considered the proposed risk of bias judgement from the algorithm within the Excel tool for each domain and reached a final risk of bias judgement of 'Low', 'High', or 'Some concerns'.

Specifically, for domain 2 'Bias due to deviations from intended interventions', we paid particular attention to whether the same rehabilitation professionals delivered treatments to both intervention and 'usual care' groups (and considered that this had the potential to introduce bias). For domain 3 'Bias due to missing outcome data', we assumed that the data presented were for all recruited participants, unless the study authors discussed dropouts; where there were dropouts reported, we considered whether this was evenly distributed between groups, or could potentially have influenced the results. For domain 4 'Bias in measurement of the outcome', we generally considered that studies with a blinded outcome assessor would be at low risk of bias, and those without ‐ or where it was unclear whether the assessor was blinded ‐ to be at high risk of bias. For domain 5 'Bias in selection of the reported result', we considered whether there was a published protocol or a trial registration, registered prior to the start of trial; where we were unable to identify either of these, we generally considered this to be of some concerns, unless further concerns were identified relating to bias in selection.

To reach an overall judgement for an outcome, we first considered that studies judged to be at low risk of bias for all domains should have an overall judgement of low risk of bias; that studies judged to have some concerns in at least one domain should have an overall judgement of some concerns; and that studies judged to be at high risk of bias in at least one domain should have an overall judgement of high risk of bias. We then considered whether we agreed with this judgement, or whether there were reasons not to follow the proposed RoB 2 algorithm, and recorded our reasons using free text. Risk of bias assessment findings were viewed and critiqued by members of the Cochrane Methods Support Unit, and a number of assessment decisions were amended in response to feedback.

For cross‐over RCTs, we only considered data from the first period prior to the cross‐over; we used the main RoB 2 tool by following guidance in Higgins 2021 and Higgins 2023b and considered (within domain 5 'Risk of bias in selection of the reported result' of the main RoB 2 tool) the possibility that the phase one result was selected because it was preferred to a result based on both periods, i.e. data from phase one reported when carry‐over was observed by the study investigators. If we include any cluster‐randomised trials in future updates, we will follow the relevant guidance in Higgins 2023b, and consider the additional domain of bias arising from the identification or recruitment of individual participants within clusters.

#### Measures of treatment effect

We presented outcome measures of independence in ADL, motor function, balance, gait velocity, and length of stay as continuous data. For outcomes of independence in ADL, motor function, and gait velocity, we calculated standardised mean differences (SMDs) and 95% confidence intervals (CIs). For outcomes of balance (Berg Balance Scale) and of length of stay, we calculated mean differences (MDs) and 95% CIs. We used a random‐effects model for all outcomes analysed.

For measures of gait velocity, we considered a higher value to denote a better outcome, and we multiplied assessments where this was reversed (e.g. time to walk a set distance, where a lower time denotes a better outcome) by minus one.

We presented the outcome measure of adverse events as count data. We used data that pertained to the number of participants experiencing an adverse event (rather than the total number of adverse events). We calculated risk ratios using Mantel‐Haenszel fixed‐effect meta‐analysis methods, as this has been shown to have better statistical properties when there are few events (Deeks 2023).

#### Unit of analysis issues

We did not anticipate any specific unit of analysis issues. We anticipated that the unit of analysis for studies included in this review would be individual participants. If the unit of analysis was not individual participants (e.g. in the case of cluster trials), we planned to record this and adjust accordingly. For cross‐over trials, we only used data for the first phase of the study (i.e. before the cross‐over, so individual participants only had one intervention). For studies where outcomes were reported at several different time points, we used (i) the 'immediate' time point (i.e. outcome assessed immediately after the end of the period of treatment) and (ii) a 'persisting' time point (i.e. a follow‐up measure). Where studies reported multiple follow‐up measures, we used the shortest time point, and recorded the time point that we used. Where trials had more than one eligible active intervention group within the same comparison, we divided the control group data between the multiple pairwise comparisons to ensure there was no double counting of participants within any one analysis.

#### Dealing with missing data

For studies included in the 2014 and earlier versions, we contacted and ‐ where possible ‐ obtained missing data from study authors. Due to the number of studies included in the 2023 update, missing data were not obtained from study authors.

Where studies reported a mean but no standard deviation, but instead either a confidence interval or P value, we used the calculator within Review Manager (RevMan) to calculate a standard deviation (RevMan 2024).

Where studies reported a median, but no mean, and measures such as minimum, maximum, range and/or interquartile range, we imputed the medians and used methods recommended by Wan 2014 to estimate standard deviations, using the provided Excel spreadsheets for calculations. We noted whether our estimate was based on the minimum or maximum, or on interquartile ranges, within analysis footnotes. We conducted sensitivity analyses to explore the effect of including these values.

Where there were missing standard deviations for the results data, but standard deviations reported for baseline data, we used these values as estimates of the standard deviations.

Where there were missing data, and we were unable to use the above approaches, we did not include the study within the analysis.

#### Assessment of heterogeneity

We visually inspected forest plots to explore whether the confidence intervals of individual studies overlap; where there were visual outliers, we checked the data (for data extraction or entry errors) and considered reasons for heterogeneity (e.g. a very high treatment dose). We considered the I^2^ statistic as reported in forest plots, and used the following as a rough guide to interpretation:

- I^2^ of 0% represents no heterogeneity;
- 0% < I^2^ < 30% may represent some heterogeneity;
- 30% ≤ I^2^ < 50% may represent moderate heterogeneity;
- 50% ≤ I^2^ < 75% may represent substantial heterogeneity;
- I^2^ ≥ 75% may represent considerable heterogeneity.

We conducted subgroup and sensitivity analyses to further explore heterogeneity (Subgroup analysis and investigation of heterogeneity).

For the current update, we pre‐planned that we would group the main analyses for outcomes of ADL, motor function, and gait velocity according to the method of assessment (i.e. outcome measure), to explore whether the outcome tool contributed to heterogeneity. We were not able to do this for the analyses comparing different types of treatment approach, as here the groups had to reflect the types of treatment compared in order to make these analyses meaningful. For these analyses, we noted the outcome tool in the footnotes, and visually inspected plots to explore whether outliers may be related to the type of outcome measure.

#### Assessment of reporting biases

We compared the availability of our planned outcomes (i.e. as listed in study protocols or methods) with those reported in the included trials. We noted where study authors described an outcome as measured but did not report it or where data were unavailable for analysis. We incorporated this information into our judgement of risk of bias for each outcome measure (for the domain of bias in selection of the reported result).

We produced funnel plots for comparisons of (i) physical rehabilitation versus no physical rehabilitation and (ii) effect of additional physical rehabilitation for our primary outcomes of independence in ADL and motor function. These were produced in RevMan Web, plotting the SMD (horizontal axis) against the standard error of the effect estimate (vertical axis). We visually inspected funnel plots for asymmetry, considering whether any asymmetry could be explained by non‐reporting biases, poor methodological quality leading to spuriously inflated effects in smaller studies, true heterogeneity, or other reasons (Page 2023).

#### Data synthesis

The previous version of this review compared active (physical rehabilitation) interventions versus no treatment, versus usual care/control, and versus another active intervention (Todhunter‐Brown 2014). Based on discussions with our stakeholder group, for this updated version we changed to the comparisons below:

1. Physical rehabilitation versus no physical rehabilitation
2. Physical rehabilitation versus attention control
3. Additional physical rehabilitation (plus usual therapy compared to usual therapy only)
4. Different approaches to physical rehabilitation (one physical rehabilitation approach compared to another physical rehabilitation approach)

All eligible studies with data on independence in activities of daily living or motor function (Primary outcomes), or on balance, gait velocity, length of stay, or adverse events (Secondary outcomes), were included in quantitative analyses.

For studies included in quantitative analyses: for Comparisons 1 to 3, we created subgroups based on type of outcome measure. For Comparison 4, we created subgroups based on an assessment and categorisation of the content of the different physical rehabilitation approaches, using the framework for describing physical rehabilitation that was co‐produced with stakeholders (see Figure; Table; Appendix 1). For Comparison 4, we combined data from different outcome measures within the analyses using SMDs, noting the outcome tool used within the footnotes.

For studies included in qualitative syntheses only, their study details are summarised in Characteristics of included studies, and reasons for not being included in quantitative (meta‐) analyses are reported.

#### Subgroup analysis and investigation of heterogeneity

For our comparisons of 'Physical rehabilitation versus no physical rehabilitation' and 'Additional physical rehabilitation plus usual therapy versus usual therapy alone', for outcomes 'Independence in ADL' and 'Motor function' (see Primary outcomes), we carried out subgroup analyses to explore the following:

- Time post‐stroke of participants ‐ early (two weeks or less post‐stroke) or later (more than two weeks post‐stroke).
  - These groups were determined pragmatically, aiming to get two groups of approximately equal numbers of trials.
- Geographical location of the study ‐ Asia or any other continent.
  - The decision to group studies according to Asia or 'other' continent was due to the large proportion of studies conducted in Asia.
- Dose of intervention ‐ more than or less than 2.5 hours per week.
  - For the comparison 'Additional physical rehabilitation plus usual therapy versus usual therapy alone', we further subdivided the group 'More than 2.5 hours per week' into 'Between 2.5 and 5 hours per week' or 'More than 5 hours per week'. This was pursued as very few studies delivered physical rehabilitation for less than 2.5 hours a week.
- Duration of intervention ‐ short (≤ 2 months) or longer (> 2 months).
  - These groups were determined pragmatically, aiming to get two groups of approximately equal numbers of trials.
- Focus of treatment components.
  - The groupings explored were determined from an exploration of the data extracted relating to individual treatment components.
  - For the comparison 'Physical rehabilitation versus no physical rehabilitation', the physical rehabilitation approaches were grouped according to whether there was a focus on functional task training or comprised mixed treatment components.
  - For the comparison 'Additional physical rehabilitation plus usual therapy versus usual therapy alone', we explored groups where functional task training was added to a less functional approach, or a neurophysiological approach was added to mixed or other approaches.
- Named approaches.
  - For the comparison 'Physical rehabilitation versus no physical rehabilitation', we explored subgroups based on the intervention 'name' provided by the study authors, considering physical rehabilitation described as a 'three‐stage' or 'Bobath' approach.
- Profession of the person who delivered the intervention.
  - We planned to categorise studies according to the professional background of the intervention provider, but due to insufficient reported information we instead grouped studies according to whether the intervention was reported to be delivered by 'medical/therapeutic staff', 'medical/therapeutic staff with input from family/carers', or 'others/not stated'.

These subgroups were informed by our stakeholder group (see Appendix 1). The stakeholder group also highlighted the importance of mode of delivery (face‐to‐face or virtual sessions; and one‐to‐one or group rehabilitation) and whether rehabilitation was individualised or followed a standard/prescribed set of exercises or treatments. However, we had insufficient data to explore these within subgroup analyses (due to either lack of reporting, or because a large majority of the included trials all had the same mode of delivery).

#### Sensitivity analysis

We carried out sensitivity analyses to explore the effects of risk of bias in included studies, for our primary outcomes, for all four comparisons. We adopted a sequential approach, exploring the effect of removing: studies with an overall high risk of bias, studies with concerns relating to data entered, and studies where ≥ 4 domains were judged as 'some concerns' using the RoB 2 tool. We tabulated the results of all sensitivity analyses and noted whether there were any changes from the main analysis result.

#### Summary of findings and assessment of the certainty of the evidence

We presented our findings for each of our four main comparisons, for our primary and secondary outcomes at the 'immediate' time point and for adverse events, in summary of findings tables created in GRADEpro GDT. We summarised the results for outcomes at the 'persisting' time point (long‐term effects) within an additional table.

We rated our certainty in the cumulative evidence for each synthesis using GRADE methodology and considering design, inconsistency, indirectness, imprecision, and publication bias (Schünemann 2023). We considered our overall RoB 2 judgements during the GRADE assessment, as well as the results of sensitivity analyses to remove studies judged to be of some concerns or high risk of bias. We categorised our certainty in the body of evidence as 'high', 'moderate', 'low', or 'very low', and provided explanations for our judgements.

## Results

### Description of studies

#### Results of the search

Results of the search for this update are displayed in Figure. For this update, we screened 20,644 de‐duplicated records (including 107 studies carried over from the previous review version). We assessed 508 full‐text articles for eligibility criteria and included 267 studies (including 81 from previous review versions). See Characteristics of included studies. This update also identified eight studies awaiting classification (Characteristics of studies awaiting classification) and 36 ongoing studies (Characteristics of ongoing studies). Details of the screening done using Cochrane's Screen4Me workflow and Cochrane Crowd are provided in Appendix 11.

**2:**
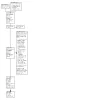
PRISMA flow diagram

For details of the searches for and studies included/excluded from previous versions of this review, please see Pollock 2003; Pollock 2007; Todhunter‐Brown 2014.

#### Included studies

We included 267 studies (21,838 participants) in this review update. Details are summarised in Characteristics of included studies.

#### Study size

The mean number of participants was 82 (SD 84.4); 254 of the 267 studies included fewer than 200 participants. Two studies had more than 1000 participants (Hu 2007, n = 1365; Zhang 2004, n = 1078); six had between 250 and 1000 participants (Bai 2008, n = 364; Behrman 2011, n = 408; Chen L 2019, n = 488; Kwakkel 2008, n = 250; LAST 2018, n = 380; Zhao 2003, n = 300). Fourteen studies included fewer than 20 participants (Ain 2022 n = 14; Allison 2007, n = 17; Bale 2008, n = 18; Carlson 2006, n = 11; Dean 2000, n = 12; Khallaf 2014, n = 16; Kim 2018, n = 13; Kunkel 2013, n = 14; Letombe 2010, n = 18; Medina‐Rincón 2019, n = 14; Meier 2021, n = 7; Mendoza 2015, n = 18; Stephenson 2004, n = 18; Teixeira‐Salmela 1999, n = 13).

#### Settings

Table lists the study settings and geographical locations of the included studies. Table summarises studies according to comparison and region, and Table illustrates the included studies by country income level.

**7:** Summary of study setting and trial registration reference

**Study**	**Study setting at recruitment**	**Date of recruitment**	**Study setting for intervention**	**Geographical location**	**Region**	**Income group**	**Trial registration details**
ACTIV 2021	Recruited through publicly funded District Health Board inpatient and community stroke rehabilitation services	Minor ethics amendment approved May 2013, 9 months after recruitment began. Recruitment started Aug/Sep 2012. End date not specified	ACTIV intervention took place in participants’ own homes or remotely via telephone contact and text messages	New Zealand	East Asia & Pacific	High income	ACTRN12612000464864
Ain 2022	Hospital	September 2019 ‐ March 2020	Unclear	Pakistan	South Asia	Lower middle income	Not reported
Aksu 2001	Not reported	Not reported	Not reported	Türkiye	Europe & Central Asia	Upper middle income	Not reported
Alabdulwahab 2015	Outpatient rehabilitation unit at Department of Neurology & Neurosurgery, All India Institute of Medical Sciences	Not reported	Outpatients	India	South Asia	Lower middle income	Not reported
Allison 2007	Stroke Rehabilitation Unit at Newton Abbot Hospital	Not reported	Ward	United Kingdom	Europe & Central Asia	High income	Not reported
Aloraini 2022	Local healthcare centres and from the community through advertisements and word of mouth	Not reported	Supervised sessions: Not reported Home exercises: Home	Saudi Arabia	Middle East & North Africa	High income	Not reported
Anandan 2020	Neurological centres in and around Erode	Not reported	Neurological centres in and around Erode	India	South Asia	Lower middle income	Not reported
Arabzadeh 2018	Outpatient clinics of neurology and physiotherapy at Asadabady and Razi hospitals affiliated with Tabriz University of Medical Sciences	October 2015 ‐ January 2016	Outpatient clinic	Iran, Islamic Rep.	Middle East & North Africa	Lower middle income	IRCT2015100224297N1
Aravind 2022	Three urban centres in (Toronto, Pembroke, and London) in Ontario, Canada	March 2017 ‐ April 2019	Recreation centres	Canada	North America	High income	NCT03122626
Aries 2021	An in‐patient stroke rehabilitation ward and community‐based Stroke Early Supported Discharge service	Not reported	Study interventions and outcome measurements were performed either in an inpatient clinical setting within an NHS organisation or at the participant’s own home, depending upon where the participant res	United Kingdom	Europe & Central Asia	High income	ISRCTN13676183
Arya 2019	Rehabilitation institute situated in a metropolitan city of India	Not reported	Rehabilitation institute	India	South Asia	Lower middle income	CTRI/2016/09/007258
Baer 2007	Not reported	Not reported	Home	United Kingdom	Europe & Central Asia	High income	Not reported
Bai 2008	Not clear. Recruited "with cerebral hemorrhage who were treated in neurology departments, emergency departments and inpatients in 21 hospitals nationwide "	January 2002 ‐ June 2003	Emergency Hospitals, rehabilitation centres, and communities	China	East Asia & Pacific	Upper middle income	Not reported
Bai 2013	Hospital	26 May 2008 ‐ 30 November 2009	Hospital	China	East Asia & Pacific	Upper middle income	Not reported
Bai 2014	4 hospitals in Shanghai	1 January 2002 ‐ 13 June 2003	Hospital ward, home	China	East Asia & Pacific	Upper middle income	Not reported
Bale 2008	Two rehabilitation units, a hospital ward, and a rehabilitation centre	Not reported	Training sessions, rehabilitation on the ward and in occupational therapy	Norway	Europe & Central Asia	High income	Not reported
Batchelor 2012	Recruited from 9 health services across Melbourne and Adelaide between October 2006 and November 2010	October 2006 ‐ November 2010	Ongoing physio/OT in community, home exercises	Australia	East Asia & Pacific	High income	ACTRN12607000398404
Behrman 2011	Community rehabilitation hospitals	Not reported	Clinics, home	United States	North America	High income	Not reported
Bek 2016	Advertisement to local support groups, social care organisations, open days, and local media	between February and September 2010.	National Institute for Conductive Education, Birmingham	United Kingdom	Europe & Central Asia	High income	ISRCTN84064492
Bhatia 2014	Physiotherapy OPD, K S Hegde charitable hospital	Not reported	Physiotherapy OPD, K S Hegde charitable Hospital	India	South Asia	Lower middle income	Not reported
Blennerhassett 2004	Inpatients at Austin Health, Royal Talbot Rehabilitation Centre	Total study (not just recruitment) between September 2001 ‐ February 2003	Physiotherapy clinic/gym (circuit training)	Australia	East Asia & Pacific	High income	Not reported
Bordoloi 2020	Department of Neurology, Gauhati Medical College and Hospital (GMCH)	12 May 2014 ‐ 10 December 2017	Clinics, home	India	South Asia	Lower middle income	Not reported
Brock 2005	2 rehabilitation centres	Not reported	Rehabilitation centres	Australia and Germany	East Asia & Pacific and Europe & Central Asia	High income	Not reported
Brouwer 2018	Inpatient rehabilitation units	28 month period	Home	Canada	North America	high income	NCT00400712
Bui 2019	Traditional Medicine Hospital of Ho Chi Minh City, General Hospital of Soc Trang province, and People Military Hospital of Sco Trang Province	July 2014 ‐ July 2015	Hospital	Vietnam	East Asia & Pacific	Lower middle income	Not reported
Candan 2017	2 hospitals	February 2014‐February 2015	Clinics, home	Türkiye	Europe & Central Asia	Upper middle income	Not reported
Cao 2014	Hospital	July 2012 ‐ July 2013	Hospital	China	East Asia & Pacific	Upper middle income	Not reported
Capisizu 2016	Geriatrics Department, Chronic Diseases Hospital "Sf Luca"	Not reported	Hospital	Romania	Europe & Central Asia	High income	Not reported
Carlson 2006	Not reported	Not reported	No info	United States	North America	High income	Not reported
Chae 2017	C Hospital located in Gyeonggido	Not reported	Hospital	Korea, Rep.	East Asia & Pacific	High income	Not reported
Chan DY 2006	Outpatient rehabilitation centre in Hong Kong	Not reported	OT department, hospital	Hong Kong SAR, China	East Asia & Pacific	High income	Not reported
Chan WN 2017	Patient self‐help groups and hospitals in Hong Kong by distributing leaflet	October 2014 ‐ December 2016	University, local community centres	Hong Kong SAR, China	East Asia & Pacific	High income	Not reported
Chang 2015	Hospital	October 2011 ‐ October 2014	Hospital	China	East Asia & Pacific	Upper middle income	Not reported
Chen L 2019	Stroke unit of the Department of Neurology at Ji’an Central People’s Hospital in China	September 2013 ‐ August 2016	Ward, hospital	China	East Asia & Pacific	Upper middle income	Not reported
Chen S 2021	Inpatients, Department of Neurology at the Second Affiliated Hospital of Wenzhou Medical University, China	January 2015 ‐ January 2018	Home, outpatient clinic	China	East Asia & Pacific	Upper middle income	Not reported
Chen G 2014	Hospital	January 2012 ‐ April 2013	Hospital	China	East Asia & Pacific	Upper middle income	Not reported
Chen J 2014	Community health centre	January 2010 ‐ December2013	Community rehabilitation clinic, home	China	East Asia & Pacific	Upper middle income	Not reported
Chen P 2014	Hospital	September 2012 ‐ October 2013	Hospital	China	East Asia & Pacific	Upper middle income	Not reported
Chen Y 2011	Hospital	September 2009 ‐ March 2010	Hospital	China	East Asia & Pacific	Upper middle income	Not reported
Cheng 2021	Hospital	January 1 2018 ‐ June 30 2020	Hospital	China	East Asia & Pacific	Upper middle income	Not reported
Choi YK 2013	C Hospital in Cheong‐ju	Not reported	Hospital	Korea, Rep.	East Asia & Pacific	High income	Not reported
Choi JU 2015	A hospital in Cheongju, Korea	July 2013 ‐ end of August 2013	Hospital	Korea, Rep.	East Asia & Pacific	High income	Not reported
Chu 2003	Hospital	March 1999 ‐ October 2001	Hospital	China	East Asia & Pacific	Upper middle income	Not reported
Cooke 2006	Hospital	Not reported	Hospital	United Kingdom	Europe & Central Asia	High income	NCT00322192
Dai 2015	Hospital	June 2013 ‐ August 2014	Hospital	China	East Asia & Pacific	Upper middle income	Not reported
Dalal 2018	Teaching hospital affiliated to Kasturba Medical College (Manipal University)	April 2015 ‐ January 2016	Hospital	India	South Asia	Lower middle income	CTRI/2016/06/007051
Danlami 2017	Stroke patients attending physio at Murtala Muhammad Specialists Hospital (MMSH) and Muhammad Abdullahi Wase Specialists Hospital in Kano, Nigeria	Not reported	All tasks were initially administered by a physiotherapist, but patients and relatives were taught how to carry out the tasks at home	Nigeria	Sub‐Saharan Africa	Lower middle income	PACTR201703002073205
Dean 2000	Rehabilitation centre	Not reported	Rehabilitation centre	Canada	North America	High income	Not reported
Dean 2006	Community‐dwelling people	Not reported	Weekly exercise class and home programme	Australia	East Asia & Pacific	High income	Not reported
Deng 2011	Department of Neurology	Not reported	Department of Neurology	China	East Asia & Pacific	Upper middle income	Not reported
Ding 2015	Hospital	May 2013 ‐ May 2014	Hospital	China	East Asia & Pacific	Upper middle income	Not reported
DOSE 2020	Inpatients in rehabilitation units	1st March 2014 ‐ 1st July 2018	Rehabilitation units	Canada	North America	High income	NCT01915368
Du 2014	Department of General Internal Medicine	November 2010 ‐ June 2012	Department of General Internal Medicine	China	East Asia & Pacific	Upper middle income	Not reported
Duan 2011	Hospital	2009 ‐2010	Hospital	China	East Asia & Pacific	Upper middle income	Not reported
Duncan 1998	Local participating hospitals and the registry of the Kansas City Stroke Study (an ongoing prospective cohort study of individuals with stroke who were admitted to 12 participating hospitals in the greater Kansas City area)	Not reported	Home	United States	North America	High income	Not reported
Duncan 2003	Kansas City Stroke Registry (an ongoing prospective cohort study of individuals with stroke who were admitted to 12 participating hospitals in the greater Kansas City area)	Not reported	Home	United States	North America	High income	Not reported
Epple 2020	Stroke unit	Not reported	Stroke unit	Germany	Europe & Central Asia	High income	NCT03035968
Fan WK 2006	Hospital	Not reported	Hospital	China	East Asia & Pacific	Upper middle income	Not reported
Fan Y 2015	Hospital	1st January 2013 ‐ June 2014	Hospital ‐ rehabilitation ward/centre	China	East Asia & Pacific	Upper middle income	Not reported
Fan L 2014	Hospital	January 2012 ‐ August 2013	Hospital	China	East Asia & Pacific	Upper middle income	Not reported
Fan WS 2006	Hospital	May 2001 ‐May 2003	Hospital	China	East Asia & Pacific	Upper middle income	Not reported
Fan X 2009	Not reported (only abstract available)	Not reported	Hospital rehabilitation department	China	East Asia & Pacific	Upper middle income	Not reported
Fang 2003	Stroke centre of the First Affiliated Hospital of Sun Yat‐Sen University in southern China	Not reported	Stroke centre	China	East Asia & Pacific	Upper middle income	Not reported
Fang YN 2004	Hospital	January 1996 ‐ December 2001	Not reported	China	East Asia & Pacific	Upper middle income	Not reported
Fang H 2010	Acupuncture and Rehabilitation Department	January 2008 ‐ May 2009	Acupuncture and rehabilitation department	China	East Asia & Pacific	Upper middle income	Not reported
FeSTivaLS 2014	Participants were recruited from the discharge database of one acute stroke service, the 6‐month post‐stroke clinic of the same stroke service, and therapist referral	March 2009 ‐ March 2012	Home	United Kingdom	Europe & Central Asia	High income	ISRCTN71632550
Frimpong 2014	Physiotherapy Department of Korle‐Bu Teaching Hospital	Not reported	Physiotherapy rehabilitation unit	Ghana	Sub‐Saharan Africa	Lower middle income	Not reported
Ge W 2003	Rehabilitation department Ruijin Hospital, China	Not reported	Not reported	China	East Asia & Pacific	Upper middle income	Not reported
Ge Y 2020	Hospital	October 2018 ‐ October 2019	Rehab unit, hospital	China	East Asia & Pacific	Upper middle income	Not reported
Gelber 1995	Acute neurorehabilitation inpatient service	Not reported	Hospital and then outpatient rehabilitation programme	United States	North America	High income	Not reported
Ghasemi 2018	Rehabilitation centres in Isfahan, Iran	Not reported	Outpatient clinic	Iran, Islamic Rep.	Middle East & North Africa	Lower middle income	IRCT201610194738N6
Gong Y 2009	Hospital	2004 ‐ 2006	Hospital	China	East Asia & Pacific	Upper middle income	Not reported
Green 2002	Patients identified via hospital and community therapy stroke registers	January 1995 ‐ October 1997	At home or outpatient rehabilitation centre	United Kingdom	Europe & Central Asia	High Income	Not reported
Guan 2017	West China Rehabilitation Medical Centre, West China Hospital, Sichuan University	September 214 ‐ September 2015	Not reported	China	East Asia & Pacific	Upper middle income	Not reported
Guo L 2012	Hospital	September 2009 ‐ August 2011	Home or at hospital, depending on group	China	East Asia & Pacific	Upper middle income	Not reported
Guo L 2013	Hospitalised patients in the Department of Rehabilitation Medicine, Heping Hospital, Changzhi Medical College	January 2009 ‐ June 2011	Not reported	China	East Asia & Pacific	Upper middle income	Not reported
Guo Z 2015	Hospital	June 2012 ‐ October 2014	Physical rehabilitation training guided by the community health service centre affiliated to the hospital	China	East Asia & Pacific	Upper middle income	Not reported
Haral 2014	Dr. DY Patil Hospital	Not reported	Not reported	India	South Asia	Lower middle income	Not reported
Harjpal 2021	Not reported "... people who had experienced their first stroke and were living in the community"	June 2021 ‐ June 2022	Neuro‐physiotherapy outpatient department	India	South Asia	Lower middle income	CTRI/2021/05/033621
Hendrey 2018	Subacute inpatient rehabilitation, Caulfield hospital	February 2014 ‐ August 2016	Inpatient rehab then outpatient rehab if discharged	Australia	East Asia & Pacific	High income	NCT01958736
Holmgren 2006	Rehabilitation unit	Not reported	Outpatient rehabilitation centre, patient homes	Sweden	Europe & Central Asia	High income	NCT00377689
Hong Cuicui 2016	Hospital	January 2014 ‐ May 2015	Hospital	China	East Asia & Pacific	Upper middle income	Not reported
Hong Hye Jin 2012	Hospital	April 2009 ‐ April 2011	Hospital	China	East Asia & Pacific	Upper middle income	Not reported
Hoseinabadi 2013	All male patients referred to Mohammed Ali Fayaz Bakhash Physiotherapy Centre in Neyshabur city, Iran	conducted in 2011	Not reported	Iran, Islamic Rep.	Middle East & North Africa	Lower middle income	Not reported
Hou 2006	Neurology ward	Not reported	Neurology ward, rehab zone/centre, own home, community ‐ depending on level of rehab	China	East Asia & Pacific	Upper middle income	Not reported
Hou Zhi 2014	Hospital	August 2010 ‐ October 2012	Neurology ward	China	East Asia & Pacific	Upper middle income	Not reported
Howe 2005	Patients admitted to stroke unit at The James Cook University Hospital, Middlesbrough, UK	10 September 2001 ‐ 14 February 2002	Hospital	United Kingdom	Europe & Central Asia	High Income	Not reported
Hu 2007	Outpatient, emergency and inpatient departments of neurology in 22 affiliated hospitals of medical colleges or provincial hospitals across the country	13 January 2002 ‐ 30 June 2003	Not reported	China	East Asia & Pacific	Upper middle income	Not reported
Huang 2003	Not reported	Not reported	Not reported	China	East Asia & Pacific	Upper middle income	Not reported
Huang 2014	Department of Neurology and Rehabilitation in hospital	May 2012 ‐ December 2013	Hospital	China	East Asia & Pacific	Upper middle income	Not reported
Huang Yangfang 2016	Hospital	February 2015 to February 2015	Hospital	China	East Asia & Pacific	Upper middle income	Not reported
Hui‐Chan 2009	Not reported	Not reported	Home	Hong Kong SAR, China	East Asia & Pacific	High income	Not reported
Imhof 2015	A specialised neurorehabilitation clinic in the German‐speaking part of Switzerland	2011‐2013	Neurorehabilitation clinic	Switzerland	Europe & Central Asia	High income	NCT02198599
Indurkar 2013	Physiotherapy OPD of Tertiary Care Hospital	Not reported	Physiotherapy OPD of tertiary care hospital	India	South Asia	Lower middle income	Not reported
Jandaghi 2021	Patients on the physiotherapy clinic of Loghman hospital, university of Shahid Beheshti medical sciences, Iran	Not reported	Stroke rehabilitation centre	Iran, Islamic Rep.	Middle East & North Africa	Lower middle income	IRCT20190812044516N1
Jeon 2018	Not reported	Not reported	Not reported	Korea, Rep.	East Asia & Pacific	High income	Not reported
Ji Pei 2014	Not reported	Not reported	Not reported	China	East Asia & Pacific	Upper middle income	Not reported
Jing 2006	Not reported	Not reported	Not reported	China	East Asia & Pacific	Upper middle income	Not reported
Jongbloed 1989	Patients admitted to Holy Family Hospital in Vancouver, Canada	August 1985 ‐ November 1986	Hospital	Canada	North America	High income	Not reported
Khallaf 2014	Physical Therapy Department, King Khalid Hospital, Hail, KSA	Not reported	Clinic, home	Saudi Arabia	Middle East & North Africa	High income	Not reported
Kim 2007	National Rehabilitation Hospital	February 2005 ‐ July 2005	Not reported	Korea, Rep.	East Asia & Pacific	High income	Not reported
Kim 2012	In patient hospital	Not reported	Not reported	Korea, Rep.	East Asia & Pacific	High income	Not reported
Kim 2012a	Inpatients of ‘S’ Rehabilitation Hospital, located in Seoul	Not reported	Not reported	Korea, Rep.	East Asia & Pacific	High income	Not reported
Kim 2014	Patients hospitalised in P municipal nursing centre for the severely handicapped located in Seoul, Republic of Korea	Not reported	Not reported	Korea, Rep.	East Asia & Pacific	High income	Not reported
Kim 2016	Department of Physical Medicine and Rehabilitation of Jeju National University Hospital	August 2012 ‐ October 2013	Hospital	Korea, Rep.	East Asia & Pacific	High income	Not reported
Kim 2017	Inpatients in the rehabilitation centres from South Korea	Not reported	Rehabilitation centres	Korea, Rep.	East Asia & Pacific	High income	Not reported
Kim 2018	Patients admitted to the rehabilitation department in a long‐term care hospital in Gwangju, Korea	Not reported	Not reported	Korea, Rep.	East Asia & Pacific	High income	Not reported
Kim 2021	Patients who were in Bundang Jesaeng hospital located in Gyeonggi‐do	Not reported	Not reported	Korea, Rep.	East Asia & Pacific	High income	KCT0006579
Knox 2018	Two of the teaching hospitals associated with the University of the Witwatersrand	January 2009 ‐ December 2011	Weekly visits to the Hospital, home programme	South Africa	Sub‐Saharan Africa	Upper middle income	PACTR201802003054396
Koç 2015	Recruited from local Yozgat Province hospitals	Not reported	Nurse‐supervised, home‐based intervention	Türkiye	Europe & Central Asia	Upper middle income	Not reported
Krawczyk 2014	Patients treated at the Institute of Psychiatry and Neurology	2007–2009	At the beginning, all patients were treated for 6 weeks at the inpatient neurorehabilitation department, and then received physiotherapy for 6 weeks at the outpatient neurorehabilitation department	Poland	Europe & Central Asia	High income	Not reported
Krukowska 2016	Clinic of Rehabilitation and Physical Medicine of the Medical University in Lodz	Not reported	Not reported	Poland	Europe & Central Asia	High income	Not reported
Kuberan 2017	In tertiary care hospitals Mangalore	March 2011 ‐ April 2012	Rehabilitation clinic	India	South Asia	Lower middle income	Not reported
Kumaran 2015	Patients with stroke were recruited from the following centres. Department of Physiotherapy, Kasturba Hospital, Manipal (Manipal University, Manipal) Department of Physiotherapy, Dr. T M A Pai Hospital	Not reported	Home under supervision of a caregiver. Also rehab education programme (but not reported where this was given)	India	South Asia	Lower middle income	CTRI/2010/091/000278
Kunkel 2013	Four local stroke rehabilitation units	December 2007 ‐ November 2008	The intervention was delivered in hospital but was continued in the community if a participant was discharged before trial completion	United Kingdom	Europe & Central Asia	High Income	Not reported
Kwakkel 2002	Rehabilitation centres and nursing homes	1st September 1994 ‐ 1st May 1997	Rehabilitation centres and nursing homes	Netherlands	Europe & Central Asia	High income	Not reported
Kwakkel 2008	Nine rehabilitation centres in the Netherlands	June 2008 ‐ December 2010	Outpatient rehabilitation in one of the 9 participating centres	Netherlands	Europe & Central Asia	High income	NTR1534
Langhammer 2000	Stroke patients attending the hospital	October 1996 ‐ August 1997	Hospital. After discharge ‐ homes, rehabilitation centres in the community or in private outpatient departments.	Norway	Europe & Central Asia	High income	Not reported
Langhammer 2007	Inpatients acute hospital	1st September 2003 ‐ 1st September 2004	Rehabilitation institutions, community, patients' homes and nursing homes	Norway	Europe & Central Asia	High income	Not reported
LAST 2018	Stroke unit screened, outpatient clinic recruited (at Trondheim University Hospital and Bærum Hospital)	18 October 2011 ‐ 15 January 2016	Stroke unit treatment, outpatients clinic, home	Norway	Europe & Central Asia	High income	NCT01467206
Lawal 2016	Patients attending or being referred after inpatient acute stroke care to attend the outpatient physiotherapy or outpatient hypertension clinic of Aminu Kano Teaching Hospital	Not reported	Outpatient clinic	Nigeria	Sub‐Saharan Africa	Lower middle income	PACTR201311000701191
Lee 2015	Hanwoori Community Center (Seoul, Korea)	Not reported	Not reported	Korea, Rep.	East Asia & Pacific	High income	Not reported
Lee 2018	Department of Neurology or Neurosurgery	January 2016 ‐ January 2017	Not reported	Korea, Rep.	East Asia & Pacific	High income	Not reported
Lennon 2006	Not reported	Not reported	Not reported	United Kingdom	Europe & Central Asia	High income	Not reported
Letombe 2010	Inpatients at stroke unit of 3 major teaching hospitals	Not reported	Not reported	France	Europe & Central Asia	High income	Not reported
Li 1999	Not reported	Not reported	Not reported	China	East Asia & Pacific	Upper middle income	Not reported
Li 2005	Not reported	Not reported	Not reported	China	East Asia & Pacific	Upper middle income	Not reported
Li 2013	Inpatients at hospital	October 2008 ‐ October 2011	Not reported	China	East Asia & Pacific	Upper middle income	Not reported
Li Jingqian 2013	Inpatients in rehabilitation department of hospital	October 2009 ‐ October 2012	Not reported	China	East Asia & Pacific	Upper middle income	Not reported
Li Weiwei 2015	Inpatients of rehabilitation department of hospital	Not reported	Inpatients of rehabilitation department, hospital	China	East Asia & Pacific	Upper middle income	Not reported
Li Xiaojun 2016	Admitted patients (hospital)	July 2012 ‐ July 2013	Intervention setting dependent on stage of rehabilitation: month 1: inpatients on ward; up to month 3: rehabilitation treatment in rehabilitation centre; and months 3 to 6: intervention provided at home	China	East Asia & Pacific	Upper middle income	Not reported
Li Yuanzheng 2014	Patients admitted to rehab centre of the hospital	Not reported	Not reported	China	East Asia & Pacific	Upper middle income	Not reported
Li Yuanzheng 2014a	Admitted patients (hospital)	July 2011 ‐ July 2013	Rehabilitation department	China	East Asia & Pacific	Upper middle income	Not reported
Lincoln 2003	Inpatients, stroke rehabilitation ward	Not reported	Rehabilitation ward	United Kingdom	Europe & Central Asia	High income	Not reported
Lindvall 2014	Primary healthcare in Orebro County Council	Not reported	Primary healthcare centre	Sweden	Europe & Central Asia	High income	NCT01613339
Liu 2014	Department of Rehabilitation Medicine, Hospital	February 2012 ‐ October 2013	Hospital	China	East Asia & Pacific	Upper middle income	Not reported
Liu Xuan 2016	Hospital	July 2012 ‐July 2013	Hospital	China	East Asia & Pacific	Upper middle income	Not reported
Liu Yanhua 2020	Hospital	February 2017 ‐ November 2018	Not reported	China	East Asia & Pacific	Upper middle income	Not reported
Lu 2004	Inpatients, Dept of Neurology, Union Hospital, China	March 2002 ‐ October 2003	Hospital, home	China	East Asia & Pacific	Upper middle income	Not reported
Lu 2014	Inpatients, Dept of Rehabilitation Medicine, The First People's hospital, China	October 2012 ‐ September 2013	Ward, outpatient rehabilitation department or home for rehab training depending on personal situation.	China	East Asia & Pacific	Upper middle income	Not reported
Lu Liangyan 2014	Inpatients, Dept of Neurology, hospital	April 2014 ‐ September 2014	Neurology dept (1st stage), rehab outpatient department (2nd stage), community rehab (3rd stage)	China	East Asia & Pacific	Upper middle income	Not reported
Ma Xue 2010	Inpatients in the rehabilitation department of Shanghai Xuhui District Central Hospital	May 2007 ‐ December 2008	Inpatients in the rehabilitation department	China	East Asia & Pacific	Upper middle income	Not reported
Mai Guanghuai 2016	Inpatients in Neurology department, Hospital	January 2013 ‐ January 2015	Not explicit, but appears to have been delivered while the stroke survivors were still inpatients in hospital	China	East Asia & Pacific	Upper middle income	Not reported
Mansfield 2018	Community‐dwelling adults were recruited from research volunteer databases and advertisements in the community.	24 April 2014 ‐ 29 June 2016	Research labs in academic hospitals	Canada	North America	High income	ISRCTN05434601
Marigold 2005	Participants living in the community were recruited from hospital databases, stroke groups, and advertisements.	Not reported	Local community centre	Canada	North America	High income	Not reported
Martins 2020	Community ‐ contacted via health centres and research groups	June 2016 ‐ November 2017	Health centres and laboratory settings	Brazil	Latin America & Caribbean	Upper middle income	NCT02937480
Matthew Hall 2013	Inpatients in rehabilitation centre of hospital	March 2010 ‐ October 2012	Not reported	China	East Asia & Pacific	Upper middle income	Not reported
McClellan 2004	Were recruited on discharge from physiotherapy services in 5 public and 1 private hospital in metropolitan and regional NSW	Not reported	Local physiotherapy outpatient department. Home	Australia	East Asia & Pacific	High income	Not reported
Medina‐Rincón 2019	Inpatients, rehabilitation unit of an intermediate care hospital in Barcelona, Spain	Not reported	Intermediate care hospital	Spain	Europe & Central Asia	High income	NCT03406026
Meier 2021	Medical university, Innsbruck, Austria (not clear if inpatients)	Not reported	Rehab clinic, home	Austria	Europe & Central Asia	High income	ISRCTN14527641
Mendoza 2015	Word‐of‐mouth advertisement to recruit participants from a hospital‐based stroke support group and their community‐based networked	Not reported	Not reported	Philippines	East Asia & Pacific	Lower middle income	Not reported
Meng 2022	Hospital outpatient and emergency departments	1 October 2019 ‐ 14 September 2021	Hospital	China	East Asia & Pacific	Upper middle income	ChiCTR1900026225
Meng Fanda 2021	Inpatient, hospital	May 2018 ‐ May 2020	Hospital	China	East Asia & Pacific	Upper middle income	Not reported
Meng Qingling 2015	Inpatient, Dept of Neurology, Qianxi county People's Hospital	2013‐2014	Hospital	China	East Asia & Pacific	Upper middle income	Not reported
Mikolajewska 2017	Patients (doesn't state where but assume hospital)	Not reported	Hospital	Poland	Europe & Central Asia	High income	not reported
Mohaideen 2014	Inpatients at District Government Hospital, Warangal, India	Not reported	Not reported	India	South Asia	Lower middle income	not reported
Moore 2016	Participants were recruited from community stroke services by National Institute for Health North East Stroke Local Research Network clinical trial officers, stroke health professionals, or advert	Not reported	Classes delivered in the community	United Kingdom	Europe & Central Asia	High income	ISRCTN41026907
Morreale 2016	Patients at Operative Unit of Neurology of the Department of Clinical Neurosciences, Neurological Centre of Latium and to the Section of Neurology of the Department of Medical and Surgical Sciences and	January 2008 ‐ January 2013	Hospital, then intensive rehabilitation unit after discharge	Italy	Europe & Central Asia	High income	Not reported
Mudge 2009	Private rehabilitation clinic	June 2007 ‐ February 2008	Private rehabilitation clinic	New Zealand	East Asia & Pacific	High income	ACTRN12607000081415
Mustafaoğlu 2018	Public rehabilitation hospital	November 2014 ‐ November 2015	Not stated	Türkiye	Europe & Central Asia	Upper middle income	NCT02735148
Nagy 2017	Rehabilitation clinic	Not reported	Rehabilitation clinic	Hungary	Europe & Central Asia	High income	Not reported
Ni 1997	Not reported	Not reported	Not reported	China	East Asia & Pacific	Upper middle income	Not reported
Nindorera 2022	Potential participants were identified from hospitals and rehabilitation centres using admission records. Those who were living in the community and who had completed or dropped out of conventional physical therapy were invited to enrol.	February 2020 ‐ March 2020	Community facility attached to the National Teaching hospital (CHU Roi Khaled)	Burundi	Sub‐Saharan Africa	Low income	PACTR202001714888482
Outermans 2010	Neurorehabilitation clinic in Bad Berleburg, Germany	Not reported	Neurorehabilitation clinic in Bad Berleburg, Germany	Netherlands	Europe & Central Asia	High income	Not reported
Pan 2004	Not reported	Not reported	Not reported	China	East Asia & Pacific	Upper middle income	Not reported
Pandian 2014	Department of occupational therapy, Pandit Deendayal Upadhaya Institute for handicapped	Not reported	Outpatient programme in occupational therapy unit	India	South Asia	Lower middle income	Not reported
Pang 2003	Department of internal neurology	January 2000 ‐ December 2000	Department of internal neurology	China	East Asia & Pacific	Upper middle income	Not reported
Pang 2005	Recruited from a local rehabilitation hospital database, community stroke clubs and local newspaper advertisements	Not reported	Community hall multi‐purpose room	Canada	North America	High income	Not reported
Pang 2006	Department of Neurology, First Affiliated Hospital of Kunming Medical College	January 2004 ‐ March 2005	Not reported	China	East Asia & Pacific	Upper middle income	Not reported
Pang 2018	Participants were recruited from the community stroke patient groups	October 2014 ‐ February 2016	Exercise room located in the university	Hong Kong SAR, China	East Asia & Pacific	High income	NCT02270398
Park 2021	Inpatients at the K— Rehabilitation Hospital in South Korea	February 2017 ‐ May 2017	Inpatients at Rehabilitation Hospital in South Korea	Korea, Rep.	East Asia & Pacific	High income	Not reported
Pirayesh 2021	All stroke patients referring to the educational and medical centres of Yasuj University of Medical Sciences	April 2020 ‐ July 2020	Exercises were performed individually in a suitable room (maintaining the principle of patient privacy) in Shahid Beheshti Hospital, Shahid Mofteh Clinic No. 1, and private clinics	Iran, Islamic Rep.	Middle East & North Africa	Lower middle income	IRCT20200107046046N1
Puckree 2014	Patients reporting for therapy at a community‐based assessment and therapy centre	Not reported	Community‐based assessment and therapy centre	South Africa	Sub‐Saharan Africa	Upper middle income	Not reported
Qian 2004	Not reported	Not reported	Not reported	China	East Asia & Pacific	Upper middle income	Not reported
Qin 2013	Recruited from stroke reporting card network system of Yinxing Community Health Service Center	June 2009 ‐ March 2011	MRP was only performed in the Hospital. Acupuncturists group seen in hospital outpatient or door‐to‐door	China	East Asia & Pacific	Upper middle income	Not reported
Qin JianJian 2014	Patients admitted to rehabilitation centre of our hospital	January 2013 ‐ December 2013	Not reported	China	East Asia & Pacific	Upper middle income	Not reported
Rahayu 2020	Recruiting the participants who were warded for a 7‐day observation from 3 hospitals in Surakarta region	April 2018 ‐ November 2018	Not reported	Indonesia	East Asia & Pacific	Lower middle income	Not reported
Renner 2016	Inpatients at Neurological Rehabilitation Center, Leipzig‐Bennewitz	November 2008 ‐ November 2011	Inpatient rehabilitation at hospital	Germany	Europe & Central Asia	High income	DRKS 00005353
ReTrain 2018	Participants were identified by: (1) clinicians in NHS primary care, hospital and community stroke services; (2) contacts in the local Clinical Research Network and Clinical Research Facility	June 2015 ‐ January 2016	ReTrain was delivered in a community setting (1 gym, 2 church halls and 1 community centre)	United Kingdom	Europe & Central Asia	High income	NCT02429180
Richards 1993	Inpatients at hospital	5th May 1988 ‐ 31st May 1990	Inpatients at hospital	Canada	North America	High income	Not reported
Salbach 2004	Subjects were recruited from 9 hospitals and 2 rehabilitation centres in Montreal or Quebec City	May 2000 ‐ Feb 2003	Hospital setting	Canada	North America	High income	Not reported
Sekhar 2013	Department of physiotherapy, Sri Venkateswara Institute of Medical Sciences (Svims) Tirupati	Not reported	Department of physiotherapy, Sri Venkateswara Institute of Medical Sciences (Svims) Tirupati	India	South Asia	Lower middle income	not reported
Seo 2015	Patients in n K hospital located in Daegu Metropolitan City	August 20 ‐ September 30, 2014 (all study, not just recruitment)	Hospital	Korea, Rep.	East Asia & Pacific	High income	Not reported
Severinsen 2014	Identified from a national database including all hospitalised stroke patients within the catchment area of Aarhus University Hospital during the period of 2004‐2007	Not reported	Not reported	Denmark	Europe & Central Asia	High income	Not reported
Shin 2011	Patients with stroke who were attending a rehabilitation centre	Not reported	Rehabilitation centre	Korea, Rep.	East Asia & Pacific	High income	Not reported
Shuai 2013	Hospitalised in Department of Rehabilitation Medicine, Tongji Hospital Affiliated to Tongji Medical College, Huazhong University of Science and Technology	March 2011 ‐ March 2013	Hospital	China	East Asia & Pacific	Upper middle income	Not reported
Signal 2014	Recruited via adverts in local newspapers, at local stroke foundation meetings, via AUT Akoranga Campus community and AUT university physiotherapy clinic notice boards	June 2010 ‐ June 2011	Health and rehabilitation research institute of AUT university	New Zealand	East Asia & Pacific	High income	ACTRN12610000460000
Song 2015	Patients at C and S hospitals (not clear if inpatients or not)	Not reported	Not reported	Korea, Rep.	East Asia & Pacific	High income	Not reported
SPIRES 2022	Inpatient for stroke rehab at stroke rehabilitation units	01 January 2017 ‐ 30 September 2017	Three healthcare sites, comprising 4 Stroke Rehabilitation Units (SRUs) based in 2 counties in the South West Peninsula of England	United Kingdom	Europe & Central Asia	high income	ISRCTN15412695
Stephenson 2004	Not reported	Not reported	Not reported	United States	North America	High income	Not reported
Stuart 2019	Recruited from the VAMHCS in Baltimore, Maryland, NRH, and the community at large	Not reported	Classes were offered in 5 community locations	United States	North America	High income	Not reported
Sun Juanjuan 2014	Inpatients Zhengzhou Hospital of Traditional Chinese Medicine, Henan	June 2010 ‐ June 2012	Inpatients	China	East Asia & Pacific	Upper middle income	Not reported
SunRISe 2021	Recruited from the database of the Academic Hospital Paramaribo (AZP) and the community	April 2016 ‐ April 2017	Home‐based	Suriname	Latin America & Caribbean	Upper middle income	NCT02717715
Tang 2009	Not reported	Not reported	Not reported	China	East Asia & Pacific	Upper middle income	Not reported
Tang Yao 2015	Inpatients, Neurology, Hospital	November 2012 to November 2013	Hospital and home	China	East Asia & Pacific	Upper middle income	Not reported
Teixeira‐Salmela 1999	Community dwelling. Recruited from local stroke club and through newspaper and cable television advertisements.	Not reported	Programme delivered in community venues with exercises to do at home	Canada	North America	High income	Not reported
Thaut 2007	Patients from 2 research centres in Germany and the United States	Not reported	Not reported	Germany and United States	Europe & Central Asia and North America	High income	Not reported
Torres‐Arreola 2009	Inpatient at one of 3 general hospitals of the IMSS (Mexico City)	April 2003 ‐ May 2004	Hospital	Mexico	Latin America & Caribbean	Upper middle income	Not reported
Tyson 2015	Recruited through the North‐West Stroke Local Research Network from inpatient stroke rehabilitation services in 12 hospitals across North‐West England.	Not reported	Hospital setting and at home if discharged during treatment period	United Kingdom	Europe & Central Asia	High income	ISRCTN29533052
Vahlberg 2017	Community, identified via the Swedish national stroke discharge register	October 2009 ‐ April 2011	Circuit class and at home	Sweden	Europe & Central Asia	High income	NCT1161329
Verma 2011	Inpatient neurology ward	March 2010 ‐ September 2010	Inpatient rehab and/or outpatient rehab in day care units	India	South Asia	Lower middle income	Not reported
Wade 1992	Homes (most patients (n = 60/94 ‐ does not state how rest were recruited) were recruited from the 328 survivors in the Oxfordshire community stroke project.)	Not reported	Home	United Kingdom	Europe & Central Asia	High income	Not reported
Wan Xueli 2014	Inpatients, Dept of Rehabilitation Physiotherapy, Xinqiao Hospital	January 2011 ‐ December 2013	Hospital	China	East Asia & Pacific	Upper middle income	Not reported
Wang 2004a	Inpatients in Department of Neurology, Ruikang Hospital of Guangxi College of Traditional Chinese Medicine	Not reported	Bedside and treatment room	China	East Asia & Pacific	Upper middle income	Not reported
Wang 2004b	Inpatients, Department of Neurology, Second Affiliated Hospital, Hehan University of Science and Technology, Luoyang	Not reported	Hospital	China	East Asia & Pacific	Upper middle income	Not reported
Wang 2005	Rehabilitation inpatient department of a medical centre	Not reported	Not reported	China	East Asia & Pacific	Upper middle income	Not reported
Wang 2013	Inpatients, hospital rehabilitation centre	February 2008 ‐ February 2010	Rehabilitation centre	China	East Asia & Pacific	Upper middle income	Not reported
Wang 2015	Patients at rehabilitation and neurology departments of 3 teaching hospitals in Southern Taiwan	Not reported	Home	Taiwan, China	East Asia & Pacific	High income	Not reported
Wang 2021	Inpatient, in 3 wards of neurology departments at a tertiary hospital in China	December 2018 ‐ June 2020	Hospital	China	East Asia & Pacific	Upper middle income	NCT03702452
Wang 2022	Newly admitted patients with a diagnosis of acute ischaemic stroke at the hospital electronic information service platform	January 2018 ‐ October 2018	Hospital	China	East Asia & Pacific	Upper middle income	NCT04402736
Wang Dongya 2015	Not reported	Not reported	Home	China	East Asia & Pacific	Upper middle income	Not reported
Wang Leilei 2020	Cangzhou Integrated Traditional Chinese and Western Medicine Hospital	July 2016 to June 2018	Department of Encephalopathy and Rehabilitation of the hospital	China	East Asia & Pacific	Upper middle income	Not reported
Wang Wenwei 2012	Inpatients in the Department of Neurology and Rehabilitation of the Second Affiliated Hospital of the Hospital	March 2010 ‐ September 2011	Unclear ‐ appears to be a mix of home‐based practice with family support and out‐patient appointments	China	East Asia & Pacific	Upper middle income	Not reported
Wei 2014	Patients treated in neurology department of Hankou Hospital, Wuhan	September 2009 ‐ September 2012	Hospital, rehabilitation centre of hospital, community health service centre	China	East Asia & Pacific	Upper middle income	Not reported
Werner 1996	Home: All stroke patients discharged from the 3 rehabilitation centres within the previous 5 years and local stroke support group members were mailed a questionnaire	Not reported	"outpatient therapy"	United States	North America	High income	Not reported
Wu 2006	Not reported	Not reported	Ward, rehab ward, community	China	East Asia & Pacific	Upper middle income	Not reported
Wu 2020	Adult stroke patients who were treated in the Department of Neurology in the Third Affiliated Hospital of Soochow University	December 2016 ‐ December 2017	Hospital then home remote rehabilitation	China	East Asia & Pacific	Upper middle income	ChiCTR1800018934
Wu Jiaming 2006	Neurology Department, Hospital	April 2004 ‐ January 2006	Hospital	China	East Asia & Pacific	Upper middle income	Not reported
Wu Jing 2015	Patients admitted to Department of Neurology, People's Hospital of Cangzhou City	June 2011 ‐ June 2013	Bedside (hospital), in the ward, home/community rehab	China	East Asia & Pacific	Upper middle income	Not reported
Wu Lotus 2016	Hospital	May 2014 ‐ March 2015	Unclear ‐ appears to be hospital	China	East Asia & Pacific	Upper middle income	Not reported
Xiao 2003	Not reported	Not reported	Not reported	China	East Asia & Pacific	Upper middle income	Not reported
Xiao Yuhua 2015	Not reported	Not reported	Not reported	China	East Asia & Pacific	Upper middle income	Not reported
Xiao Zhen‐dong 2014	Hospital	October 2011 ‐ October 2014	Not reported	China	East Asia & Pacific	Upper middle income	Not reported
Xie 2003	Hospital	September 2009 ‐ March 2010	Hospital	China	East Asia & Pacific	Upper middle income	Not reported
Xie 2005	Patients treated in Dept of Emergency and Dept of Neurology, First People's Affiliated Hospital, Shanghai	April 2002 ‐ April 2003	Hospital ward, home	China	East Asia & Pacific	Upper middle income	Not reported
Xu 1999	Hospital	2009‐2010 September	Not reported	China	East Asia & Pacific	Upper middle income	Not reported
Xu 2003a	Hospital	November 2000 ‐ May 2002	Neurology Department, hospital	China	East Asia & Pacific	Upper middle income	Not reported
Xu 2003b	Inpatients, Department of Neurology	Not reported	Inpatients, Department of Neurology	China	East Asia & Pacific	Upper middle income	Not reported
Xu 2004	Acupuncture and Rehab department	January 2008 ‐ May 2009	Not reported	China	East Asia & Pacific	Upper middle income	Not reported
Xu 2013	Inpatients, Department of Neurology	2004 ‐ 2006	Not reported	China	East Asia & Pacific	Upper middle income	Not reported
Xu 2015	Patients recruited from Pt. D.D.U. Institute for Handicapped and from ISIC Institute of Rehabilitation Sciences, New Delhi	March 2010 ‐ October 2011	Not reported	China	East Asia & Pacific	Upper middle income	Not reported
Xu 2022	Patients with acute stroke hemiplegia admitted to the hospital	January 2019 ‐ December 2020	Hospital	China	East Asia & Pacific	Upper middle income	Not reported
Xu Wenyu 2012	Hospital	September 2010 ‐ June 2012	Not reported	China	East Asia & Pacific	Upper middle income	Not reported
Xu Yumei 2013	Hospital	August 2009 ‐ June 2010	Hospital	China	East Asia & Pacific	Upper middle income	Not reported
Xue 2006	Hospital	March 2000 ‐ October 2002	Hospital	China	East Asia & Pacific	Upper middle income	Not reported
Yadav 2016	Recruited from Pt. D.D.U. Institute for Handicapped and from ISIC Institute of Rehabilitation Sciences, New Delhi	Not reported	Not reported	India	South Asia	Lower middle income	Not reported
Yan 2002	Hospital	October 1999 ‐ August 2001	Hospital ward, rehabilitation centre	China	East Asia & Pacific	Upper middle income	Not reported
Yan 2015	Inpatients at Dongchangfu People's hospital, Liaocheng City	November 2011 ‐ November 2013	Hospital, community/home	China	East Asia & Pacific	Upper middle income	Not reported
Yang 2006	Recruited from local participating hospitals and through referrals from a local volunteer database	Not reported	Medical centre, district hospital	Taiwan, China	East Asia & Pacific	High income	Not reported
Yang 2017	Rehabilitation Department, The First Hospital, China Medical University, Shenyang	August 2014 ‐ October 2016	Unclear ‐ appears to be hospital	China	East Asia & Pacific	Upper middle income	Not reported
Yang Aiguo 2015	Inpatients, in hospital	January 2011 ‐ November 2013	Not reported	China	East Asia & Pacific	Upper middle income	Not reported
Yang Jian 2007	Rehabilitation Medicine, Central Hospital of Xuhui District, Shanghai	May 2005 ‐ December 2006	Hospital	China	East Asia & Pacific	Upper middle income	Not reported
Yang Zhihong 2015	Inpatients hospital	January 2010 ‐ January 2013	Hospital and home	China	East Asia & Pacific	Upper middle income	Not reported
Yazici 2021	Patients admitted to the Emergency Service of Gazi University Hospital	May 2018 ‐ July 2018	Hospital	Türkiye	Europe & Central Asia	Upper middle income	NCT03602326
Ye Dayong 2010	Multi‐centre rehabilitation units	May 2007 ‐ October 2009	Multi‐centre rehabilitation units	China	East Asia & Pacific	Upper middle income	Not reported
Yelnik 2008	Multi‐centre rehabilitation units	Not reported	Multi‐centre rehabilitation units	France	Europe & Central Asia	High income	Not reported
Yin 2003a	Neurology department	October 2001	Rehabilitation centre, hospital	China	East Asia & Pacific	Upper middle income	Not reported
Yue Chunjiang 2014	Inpatients, internal medicine department of our hospital	January 2011 ‐ June 2012	Not reported	China	East Asia & Pacific	Upper middle income	Not reported
Yue Lin 2012	Hospital Rehabilitation Department	January 2007 ‐ December 2009	Not reported	China	East Asia & Pacific	Upper middle income	Not reported
Zang 2013	Inpatients in Qingdao Haici Medical Group and Jiaonan City Hospital of Traditional Medicine, Dept of Neurology	January 1st 2011 ‐ June 30 2012	Neurology ward, rehabilitation ward/encephalopathy rehabilitation room, township Hospital, community, home	China	East Asia & Pacific	Upper middle income	Not reported
Zhang 1998	Not reported	Not reported	Not reported	China	East Asia & Pacific	Upper middle income	Not reported
Zhang 2004	Hospitalised patients in various tertiary rehabilitation networks	December 2001 ‐ October 2003	Department of Neurology rehab centres, dept of rehabilitation, community rehabilitation organisations, home	China	East Asia & Pacific	Upper middle income	Not reported
Zhang Huiyu 2021	Hospitalised patients	January 2017 ‐ January 2019	Not reported	China	East Asia & Pacific	Upper middle income	Not reported
Zhang Jianhong 2013	Inpatients in the neurology department of our hospital	July 2008 ‐ December 2012	Not reported	China	East Asia & Pacific	Upper middle income	Not reported
Zhang Lifang 2015	Rehabilitation in the undergraduate course (not sure what this is)	July 2011 ‐ September 2013	Not reported	China	East Asia & Pacific	Upper middle income	Not reported
Zhao 2002	Not reported	Not reported	Hospital bedside, hospital outpatient, home	China	East Asia & Pacific	Upper middle income	Not reported
Zhao 2003	Hospital	January 2001 ‐ October 2002	Neurology department, hospital	China	East Asia & Pacific	Upper middle income	Not reported
Zhao Ailiang 2016	Inpatients hospital	October 2010 ‐ October 2014	Ward, hospital	China	East Asia & Pacific	Upper middle income	Not reported
Zhao Haihong 2013	Hospital	August 2010‐ August 2012	Hospital	China	East Asia & Pacific	Upper middle income	Not reported
Zheng 2014	Rehab department, hospital (not clear if in/out patient)	May 2012 ‐ October 013	Not reported	China	East Asia & Pacific	Upper middle income	Not reported
Zhong Qiue 2014	Inpatients hospital	January 2010 ‐ March 2013	Not reported	China	East Asia & Pacific	Upper middle income	Not reported
Zhu 2001	Not reported	Not reported	Hospital, home	China	East Asia & Pacific	Upper middle income	Not reported
Zhu 2004b	Not reported	Not reported	Hospital, community, home	China	East Asia & Pacific	Upper middle income	Not reported
Zhu 2006	Not reported	Not reported	Not reported	China	East Asia & Pacific	Upper middle income	Not reported
Zhu 2007	Not reported	Not reported	Hospital, rehabilitation centre, home for intervention groups	China	East Asia & Pacific	Upper middle income	Not reported
Zhu 2016	Stroke patients were recruited from Hua Shan Hospital and branch courts of Fu Dan University, Shanghai, China	May 2014 ‐ December 2014	Not reported	China	East Asia & Pacific	Upper middle income	Not reported
Zhuang 2012	Stroke units in inpatient settings at 7 hospitals located in 4 Chinese cities	June 2007 ‐ June 2009 (trial period)	Stroke units in inpatient settings at 7 hospitals located in 4 Chinese cities	China	East Asia & Pacific	Upper middle income	Not reported

**8:** Summary of geographical region of study conduct and study comparison

**Comparison**	**> 1 Region**	**East Asia & Pacific**	**Europe & Central Asia**	**Latin America & Caribbean**	**Middle East & North Africa**	**North America**	**South Asia**	**Sub‐Saharan Africa**	**Grand Total**
Physical rehabilitation versus no physical rehabilitation		81	9	2	2	6			**100**
Physical rehabilitation versus attention control		8	4	1		4		1	**18**
Additional physical rehabilitation		31	11		2	3	5	1	**53**
Comparison of different approaches	2	39	16		3	6	12	2	**80**
Multiple comparisons		6	5					2	**13**
Unable to categorise		3							**3**
**Grand Total**	**2**	**168**	**45**	**3**	**7**	**19**	**17**	**6**	**267**

Regions sourced from World Bank https://datahelpdesk.worldbank.org/knowledgebase/articles/906519-world-bank-country-and-lending-groups

**9:** Summary of country income level and study comparison

**Comparison**	**Low income**	**Lower middle income**	**Upper middle income**	**High income**	**Grand Total**
Physical rehabilitation versus no physical rehabilitation		2	77	21	**100**
Physical rehabilitation versus attention control	1		4	13	**18**
Additional physical rehabilitation		8	27	18	**53**
Comparison of different approaches		17	28	35	**80**
Multiple comparisons		1	4	8	**13**
Unable to categorise			3		**3**
**Grand total**	**1**	**28**	**143**	**95**	**267**

Regions sourced from World Bank https://datahelpdesk.worldbank.org/knowledgebase/articles/906519-world-bank-country-and-lending-groups

Studies were conducted in Australia (n = 5), Austria (n = 1), Brazil (n = 1), Burundi (n = 1), Canada (n = 11), China (n = 133), Denmark (n = 1), France (n = 2), Germany (n = 2), Ghana (n = 1), Hong Kong (n = 4), India (n = 16), Indonesia (n = 1), Iran (n = 5), Italy (n = 1), Korea (n = 18), Mexico (n = 1), Netherlands (n = 3), New Zealand (n = 3), Nigeria (n = 2), Norway (n = 4), Pakistan (n = 1), Philippines (n = 1), Poland (n = 3), Romania (n = 1), Saudi Arabia (n = 2), South Africa (n = 2), Spain (n = 1), Suriname (n = 1), Sweden (n = 3), Switzerland (n = 1), Taiwan (n = 2), Turkey (n = 5), United Kingdom (n = 16), United States (n = 8), and Vietnam (n = 1), with two studies carried out in multiple countries (Australia + Germany; Germany + United States). Overall, 63% (168/267) of studies were conducted in East Asia and Pacific regions, with 50% (133/267) from China.

Of the studies comparing physical rehabilitation with no physical rehabilitation, 81% (81/100) were conducted in East Asia and Pacific regions; this is compared to 58% (31/53) of studies exploring additional rehabilitation and 49% (39/80) comparing different approaches.

#### Participants

Details of study participants are summarised in Table.

**10:** Details of study participants ‐ PROGRESS framework

**Study**	**Group**	**No. of participants**	**Mean age (SD), years**	**Sex** **(M/F)**	**Place of** **residence**	**Race/** **Ethnicity**	**Occupation**	**Religion**	**Education**	**SES** **status**	**Social** **Capital**
ACTIV 2021	Augmented Community Telerehabilitation Intervention (ACTIV)	47	74.1 (11.7)	23/24	NR	European: 43, Non‐European: 3, New Zealand Maori: 1	NR	NR	NR	NR	Living situation: accompanied ‐ 34, alone ‐ 13, missing ‐ 0
	Usual care	48	72.9 (11.7)	26/22	NR	European: 43, Non‐European: 4, New Zealand Maori: 1	NR	NR	NR	NR	Living situation: accompanied ‐ 35, alone ‐ 11, missing ‐ 2
Ain 2022	Functional training	7	Whole group mean (SD): 48.08 (4.833)	Whole group: 8/6	NR	NR	NR	NR	NR	NR	NR
	Conventional therapy	7			NR	NR	NR	NR	NR	NR	NR
Aksu 2001	Group 1	9	NR	Whole group 9/11	NR	NR	NR	NR	NR	NR	NR
	Group 2	7	NR		NR	NR	NR	NR	NR	NR	NR
	Group 3	4	NR		NR	NR	NR	NR	NR	NR	NR
Alabdulwahab 2015	Functional limb overloading (FLO)	13	Whole group mean (SD): 45.2 (12.5)	Whole group: 20/6	NR	NR	NR	NR	NR	NR	NR
	Limb Overloading Resistance Training (LORT)	13			NR	NR	NR	NR	NR	NR	NR
Allison 2007	Intervention	7	72.4 (17.9)	Whole group: 10/7	NR	NR	NR	NR	NR	NR	NR
	Control	10	78 (7.9)		NR	NR	NR	NR	NR	NR	NR
Aloraini 2022	Constraint‐induced movement therapy	19	60.1 (10.8)	10/9	NR	NR	NR	NR	NR	NR	NR
	Conventional rehabilitation	19	59.3 (11.4)	9/10	NR	NR	NR	NR	NR	NR	NR
Anandan 2020	Task specific training (TST)	37	55.88 (6.831)	13/12	NR	NR	NR	NR	NR	NR	NR
	Proprioceptive neuromuscular facilitation group (PNG)	37	57 (7.37)	12/13	NR	NR	NR	NR	NR	NR	NR
Arabzadeh 2018	Task‐orientated exercise programme	10	58.9 (26.8)	8/2	NR	NR	NR	NR	NR	NR	NR
	Traditional physiotherapy	10	59.6 (7.16)	7/3	NR	NR	NR	NR	NR	NR	NR
Aravind 2022	Community‐based exercise programmes supported through healthcare‐community partnership (CBEP‐HCP)	16	Median (P25, P75): 71 (65, 80)	9/7	NR	NR	No. employed 1/16	NR	Level of education: Secondary school or lower: 9/16; College: 6/16; Graduate or post‐graduate: 1/16	Financial status: Some money left over: 10/16; Just enough to make ends meet: 6/16, Not enough to make ends meet: 0/16, refused to answer: 1/16	Number with caregiver: 8/16
	Wait list control	17	Median (P25, P75): 67 (58, 79)	9/8	NR	NR	No. employed 1/17	NR	Level of education: Secondary school or lower: 12/17; College: 2/17; Graduate or post‐graduate: 1/17 (some missing data)	Financial status: Some money left over: 3/17; Just enough to make ends meet: 8/17, Not enough to make ends meet: 3/17, refused to answer: 3/17	Number with caregiver: 11/17
Aries 2021	Mobilisation and tactile stimulation + task‐specific gait training (MTS + TSGT)	19	73.8 (14.1)	9/10	NR	NR	NR	NR	NR	NR	NR
	Unlimited textured insole wearing + task‐specific gait training (TI + TSGT)	15	72.4 (9.8)	9/6	NR	NR	NR	NR	NR	NR	NR
Arya 2019	Interlimb coupling	26	50.04 (9.34)	20/6	NR	NR	NR	NR	Educational qualification: 5th standard: 5, 10th standard: 12, 12th standard: 8, above: 1	SES: below poverty line: 4, lower: 13, middle: 8, higher: 1	Marital status: married: 23, unmarried: 2, widow: 1
	Conventional rehabilitation based on neurophysiological approaches (e.g. Brunnstrom, Bobath)	24	51.35 (8.90	20/4	NR	NR	NR	NR	Educational qualification: 5th standard: 10, 10th standard: 9, 12th standard: 3, above: 2	SES: below poverty line: 11, lower: 10, middle: 3, higher: 0	Marital status: married: 20, unmarried: 2, widow: 2
Baer 2007	Part practice	Not stated	Whole group mean 72.9 (9.0)	Whole group mean 31/33	NR	NR	NR	NR	NR	NR	NR
	Whole practice	Not stated			NR	NR	NR	NR	NR	NR	NR
	Control (no treatment)	Not stated			NR	NR	NR	NR	NR	NR	NR
Bai 2008	Early rehabilitation	183	61.5 (9.4)	119/64	NR	NR	NR	NR	NR	NR	NR
	Control (no treatment)	181	60.8 (10.1)	113/68	NR	NR	NR	NR	NR	NR	NR
Bai 2013	Physiotherapy	41	59.3 (9.66)	30/11	NR	NR	NR	NR	NR	NR	NR
	Acupuncture	39	63.69 (8.70)	29/10	NR	NR	NR	NR	NR	NR	NR
	Combined	40	61.65 (11.05)	25/15	NR	NR	NR	NR	NR	NR	NR
Bai 2014	Three‐stage rehabilitation intervention	83	66.04 (10.13)	51/32	NR	NR	NR	NR	NR	NR	NR
	Usual care (no physical rehabilitation included)	82	67.63 (9.52)	51/31	NR	NR	NR	NR	NR	NR	NR
Bale 2008	Functional strength training	8	60.8 (13)	3/5	NR	NR	NR	NR	NR	NR	NR
	Training as usual	10	64.9 (8.9)	4/6	NR	NR	NR	NR	NR	NR	NR
Batchelor 2012	Multifactorial individually tailored falls prevention programme + usual care	71	70.8 (11.4)	45/26	NR	NR	NR	NR	NR	NR	Living arrangements: alone:19, spouse/carer: 34, family: 18
	Usual care	85	72.2 (9.9)	54/31	NR	NR	NR	NR	NR	NR	Living arrangements: alone:18, spouse/carer: 53, family: 14
Behrman 2011	Locomotor training programme	139	Not stated	Not stated	NR	NR	NR	NR	NR	NR	NR
	Home exercise programme	126	Not stated	Not stated	NR	NR	NR	NR	NR	NR	NR
	Usual care	143	Not stated	Not stated	NR	NR	NR	NR	NR	NR	NR
Bek 2016	Conductive education	41	60.4 (12.6) Range: 34‐85	25/16	NR	NR	NR	NR	NR	NR	NR
	Control (2 meetings) Wait list	36	64.3 (13.2) Range: 36‐88	21/15	NR	NR	NR	NR	NR	NR	NR
Bhatia 2014	Task specific strength training (TSST)	15	Whole group range: 45‐65 years	Not stated	NR	NR	NR	NR	NR	NR	NR
	Resistance training (RT)	15		Not stated	NR	NR	NR	NR	NR	NR	NR
Blennerhassett 2004	Mobility	15	53.9 (19.8)	8/7	NR	NR	NR	NR	NR	NR	NR
	Upper limb	15	56.3 (10.5)	9/6	NR	NR	NR	NR	NR	NR	NR
Bordoloi 2020	Neuro‐facilitation (Rood) + Home Exercise Programme (HEP) + conventional physiotherapy (Group B)	118	NR (inclusion criteria was 20‐65 years)	Not stated	NR	NR	NR	NR	NR	NR	NR
	HEP with conventional physiotherapy (Group A)	118	As above	Not stated	NR	NR	NR	NR	NR	NR	NR
Brock 2005	Bobath	12	61.3 (13.0) Range: 35–75y	7/5	NR	NR	NR	NR	NR	NR	NR
	Task practice	14	56.6 (15.8) Range: 29–77y	12/2	NR	NR	NR	NR	NR	NR	NR
Brouwer 2018	Tune‐up Group	51	62.7 (1.9)	24/27	Living setting (pre‐stroke): Private home: 50	NR	NR	NR	NR	NR	Living status (prestroke): Alone:12, Spouse/partner: 29, family: 9 Unpaid non‐family:1
	Control	52	62.1 (1.8)	30/22	Living setting (pre‐stroke): Private home: 51	NR	NR	NR	NR	NR	Living status (prestroke): Alone:10, Spouse/partner: 37, family: 5 Unpaid non‐family:0
Bui 2019	Modified acupuncture + Motor relearning method	33	< 50 y: 6; 50 years+: 27	17/16	NR	NR	NR	NR	NR	NR	NR
	Modified acupuncture + Bobath method	33	< 50 y: 5; 50 years+: 28	14/19	NR	NR	NR	NR	NR	NR	NR
Candan 2017	Modified constraint‐induced movement therapy (mCIMT)	18	55.13 (14.7)	8/7*	NR	NR	NR	NR	NR	NR	NR
	Neurodevelopmental therapy (NDT)	15	57.67 (12.2)	6/9	NR	NR	NR	NR	NR	NR	NR
Cao 2014	Intensive walking training (SAT)	43	54.1 (2.7)	31/12	NR	NR	NR	NR	NR	NR	NR
	Routine rehabilitation therapy	43	54.4 (1.9)	26/14	NR	NR	NR	NR	NR	NR	NR
Capisizu 2016	Neuroprotective multimodal pharmacological therapy (NT)	Whole group 115	Whole group: 76.4 (8.62)	Whole group 44/71	NR	NR	NR	NR	NR	NR	NR
	Physical therapy (PT)	As above			NR	NR	NR	NR	NR	NR	NR
	Combined treatment (NT+KT)	As above			NR	NR	NR	NR	NR	NR	NR
Carlson 2006	Treatment	6	Not stated	Not stated	NR	NR	NR	NR	NR	NR	NR
	Control (no treatment)	5	Not stated	Not stated	NR	NR	NR	NR	NR	NR	NR
Chae 2017	Proprioceptive training group + General physical therapy	15	58.27 (13.11)	13/2	NR	NR	NR	NR	NR	NR	NR
	General physical therapy	15	55.05 (13.83)	12/3	NR	NR	NR	NR	NR	NR	NR
Chan DY 2006	Motor relearning	33	53.8 (15.4)	12/14*	NR	NR	NR	NR	NR	NR	NR
	Conventional therapy	33	54.4 (13.7)	12/14*	NR	NR	NR	NR	NR	NR	NR
Chan WN 2017	Tai Chi training	9	63.9 (6.1)	5/4	NR	NR	NR	NR	Education (SD): 8.4 (2.1) years	NR	NR
	Conventional training	8 (states 5 in table)	63.2 (9.7)	3/2*	NR	NR	NR	NR	Education (SD): 9.7 (3.2) years	NR	NR
	Control (no training)	9	63.2 (6.0)	4/5	NR	NR	NR	NR	Education (SD): 10.3 (4.7) years	NR	NR
Chang 2015	Three‐stage rehabilitation intervention	25	60.86 (8.17), range: 40‐78 years	16/9	NR	NR	NR	NR	NR	NR	NR
	No physical rehabilitation	25	61.62 (8.86), range: 41‐80	17/8	NR	NR	NR	NR	NR	NR	NR
Chen L 2019	Motor Relearning Program (Group A)	245	65.69 (7.97)	130/115	NR	NR	Working status: working 97, not working 135, unknown 13	NR	Education (SD): 7.78 (2.62) years	NR	Marital status: single 6, married 180, divorced 20, separated/widowed 39; Living status: living alone 51, living with others 183, unknown 11
	Bobath approach (Group B)	243	65.53 (7.2)	128/115	NR	NR	Working status: working 99, not working 127, unknown 17	NR	Education (SD): 7.39 (2.76) years	NR	Marital status: single 7, married 178, divorced 25, separated/widowed 33; Living status: living alone 39, living with others 192, unknown 12
Chen S 2021	Advanced practice nurse‐guided home‐based rehabilitation exercise programme (HREPro)	70	55.41 (6.78)	41/18*	Location of residence: urban 35, rural 24	NR	NR	NR	Education attainment: none 4, low 35, middle 12, high 8	NR	NR
	Conventional rehabilitation	70	56.41 (6.13)	44/18*	Location of residence: urban 36, rural 26	NR	NR	NR	Education attainment: none 5, low 36, middle 12, high 9	NR	NR
Chen G 2014	Task function training group	40	65.4 (6.9)	22/18	NR	NR	NR	NR	NR	NR	NR
	Strength training group	40	62.1 (8.7)	23/17	NR	NR	NR	NR	NR	NR	NR
Chen J 2014	Community‐level three‐level rehabilitation therapy + conventional medical therapy	40	60.1 (10.1)	26/14	NR	NR	NR	NR	NR	NR	NR
	Community‐level rehabilitation therapy + conventional medical therapy	40	59.6 (10.3)	24/16	NR	NR	NR	NR	NR	NR	NR
Chen P 2014	Functional exercise training	50	Whole group: range: 30‐75y	Whole group: 52/48	NR	NR	NR	NR	NR	NR	NR
	Antispasmodic training	50			NR	NR	NR	NR	NR	NR	NR
Cheng 2021	Three‐level rehabilitation treatment + conventional treatment	78	65.78 (8.91)	47/31	NR	NR	NR	NR	NR	NR	NR
	Conventional treatment	78	66.46 (9.79)	48/30	NR	NR	NR	NR	NR	NR	NR
Chen Y 2011	Rehabilitation	33	NR	NR	NR	NR	NR	NR	NR	NR	NR
	No physical rehabilitation	33	NR	NR	NR	NR	NR	NR	NR	NR	NR
Choi YK 2013	Proprioceptive neuromuscular facilitation (PNF) combination patterns and kinesio taping	15	53.4 (9.5)	8/7	NR	NR	NR	NR	NR	NR	NR
	Neurodevelopmental treatment	15	54.1 (8.6)	7/8	NR	NR	NR	NR	NR	NR	NR
Choi JU 2015	Task‐oriented training	10	61.5 (7.2)	4/6	NR	NR	NR	NR	NR	NR	NR
	General physical therapy	10	66.4 (9.3)	4/6	NR	NR	NR	NR	NR	NR	NR
Chu 2003	Rehabilitation	30	Whole group mean 62.4 y Range: 54‐68 y	Whole group 31/27	NR	NR	NR	NR	NR	NR	NR
	Control (no treatment)	28			NR	NR	NR	NR	NR	NR	NR
Cooke 2006	Additional conventional therapy (CPT + CPT)	35	67.46 (11.3)	22/13	NR	NR	NR	NR	NR	NR	NR
	Functional strength training (FST + CPT)	36	71.17 (10.6)	22/14	NR	NR	NR	NR	NR	NR	NR
	Conventional physiotherapy (CPT)	38	66.37 (13.7)	21/17	NR	NR	NR	NR	NR	NR	NR
Dai 2015	Rehabilitation training + routine acupuncture	31	Whole group: mean age: 60.8 years; range: 36‐81 years	Not stated	NR	NR	NR	NR	NR	NR	NR
	Routine acupuncture	31		Not stated	NR	NR	NR	NR	NR	NR	NR
Dalal 2018	Rehabilitation training + routine acupuncture	16	Age group: > 40 years: 1, 40‐60 years: 10, 60‐80 years: 5	13/3	NR	NR	NR	NR	NR	NR	NR
	Routine physiotherapy	16	Age group: > 40 years: 5, 40 ‐60 years: 9, 60‐80 years: 2	11/5	NR	NR	NR	NR	NR	NR	NR
Danlami 2017	Constraint‐induced movement therapy ‐ 4 tasks repeated 40 times (sCIMT)	7	48.2 (7.89)	1/4	NR	NR	NR	NR	NR	NR	NR
	Constraint‐induced movement therapy ‐ 4 tasks repeated for 2 hours (tCIMT)	8	55.67 (9)	2/4	NR	NR	NR	NR	NR	NR	NR
	Conventional therapy	7	54.14 (6.87)	6/1	NR	NR	NR	NR	NR	NR	NR
Dean 2000	Motor learning	6	66.2 (7.7)	3/3	NR	NR	NR	NR	NR	NR	NR
	Placebo	6	62.3 (6.6)	4/2	NR	NR	NR	NR	NR	NR	NR
Dean 2006	Experimental	76	66.7 (14.3) Range: 31‐91y	38/38	NR	NR	NR	NR	NR	NR	NR
	Control	75	67.5 (10.2) Range: 40‐85 y	40/35	NR	NR	NR	NR	NR	NR	NR
Deng 2011	Intervention	50	57.08 (9.15)	36/14	NR	NR	NR	NR	NR	NR	NR
	Control (no treatment)	50	56.98 (9.05)	35/15	NR	NR	NR	NR	NR	NR	NR
Ding 2015	Strength training group + routine treatment	50	64.5 (6.5)	26/24	NR	NR	NR	NR	NR	NR	NR
	Task function training group + routine treatment	50	65.0 (7.0)	28/27*	NR	NR	NR	NR	NR	NR	NR
DOSE 2020	Determining Optimal Post‐Stroke Exercise (DOSE1) 1 hour/day	25	56 (11) years; range 27‐73	16/9	NR	NR	NR	NR	NR	NR	NR
	Determining Optimal Post‐Stroke Exercise (DOSE2) 2 hours/day	25	58 (10) years; range 31‐76	14/11	NR	NR	NR	NR	NR	NR	NR
	Usual care physical therapy	25	58 (13) years; range 35‐76	14/10	NR	NR	NR	NR	NR	NR	NR
Duan 2011	Task‐orientated training	10	61.6 (14.5); range: 37‐79	7/3	NR	NR	NR	NR	NR	NR	NR
	Neurodevelopmental training (NDT)	10	63.5 (10.5); range: 46‐78	7/3	NR	NR	NR	NR	NR	NR	NR
Du 2014	Intensive walking training	40	56.7 (8.9)	23/17	NR	NR	NR	NR	NR	NR	NR
	Conventional rehabilitation	40	58.3 (9.3)	26/14	NR	NR	NR	NR	NR	NR	NR
Duncan 1998	Mixed	10	67.3 (9.6)	Not stated	NR	NR	NR	NR	NR	NR	NR
	Control	10	67.8 (7.2)	Not stated	NR	NR	NR	NR	NR	NR	NR
Duncan 2003	Mixed	50 (44 completed intervention)	68.5 (9.0)	23/21	NR	NR	NR	NR	NR	NR	NR
	Control	50 (48 completed intervention)	70.2 (11.4)	27/21	NR	NR	NR	NR	NR	NR	NR
Epple 2020	Vojta therapy	20	Median (IQR): 77 (72.5‐85) years	9/11	NR	NR	NR	NR	NR	NR	NR
	Standard physiotherapy	20	Median (IQR): 72.5 (64‐78) years	11/9	NR	NR	NR	NR	NR	NR	NR
Fan WK 2006	Treated	42	64.53 (10.77)	22/20	NR	NR	NR	NR	NR	NR	NR
	Control (no treatment)	40	65.82 (10.61)	27/13	NR	NR	NR	NR	NR	NR	NR
Fan Y 2015	Three‐stage rehabilitation	62	60.33 (8.22)	42/20	NR	NR	NR	NR	NR	NR	NR
	Conventional treatment	62	61.09 (8.33)	39/23	NR	NR	NR	NR	NR	NR	NR
Fan L 2014	Intense task training + early rehabilitation therapy	30	60.67 (5.69)	18/12	NR	NR	NR	NR	NR	NR	NR
	Early rehabilitation therapy	30	57.60 (6.84)	19/11	NR	NR	NR	NR	NR	NR	NR
Fan WS 2006	Proprioceptive neuromuscular facilitation (PNF)	23	Whole group: 54 (14) years; range: 43‐66	Not stated "no significant difference in gender or age"	NR	NR	NR	NR	NR	NR	NR
	Conventional rehabilitation	22			NR	NR	NR	NR	NR	NR	NR
Fan X 2009	Exercise training	Whole group 80	Not stated (abstract only available)	Not stated (abstract only available)	Not stated (abstract only available)	Not stated (abstract only available)	Not stated (abstract only available)	Not stated (abstract only available)	Not stated (abstract only available)	Not stated (abstract only available)	Not stated (abstract only available)
	Conventional rehabilitation										
Fang 2003	Additional early physiotherapy intervention	78	65.49 (10.94)	33/17	NR	NR	NR	NR	NR	NR	NR
	Routine therapy	78	61.8 (10.94)	44/34	NR	NR	NR	NR	NR	NR	NR
Fang YN 2004	Rehabilitation	25	Whole group mean 65.49 (10.94)	16/9	NR	NR	NR	NR	NR	NR	NR
	Control (no treatment)	33	Whole group mean 61.8 (10.9)	18/15	NR	NR	NR	NR	NR	NR	NR
Fang H 2010	Balance training	25	57.0 (10.3)	16/16*	NR	NR	NR	NR	NR	NR	NR
	Conventional rehabilitation	25	58.5 (9.47)	18/7	NR	NR	NR	NR	NR	NR	NR
FeSTivaLS 2014	Functional Strength Training Lower Limb (FST‐LL)	Not stated	Not stated	Not stated	NR	NR	NR	NR	NR	NR	NR
	Functional Strength Training Upper Limb (FST‐UL)	Not stated	Not stated	Not stated	NR	NR	NR	NR	NR	NR	NR
Frimpong 2014	Task‐oriented circuit training	10	57.5 (0.3)	7/3	NR	NR	NR	NR	NR	NR	NR
	Conventional therapy	10	55.8 (6.7)	6/4	NR	NR	NR	NR	NR	NR	NR
Ge W 2003	Rehabilitation	20	61 (5)	14/6	NR	NR	NR	NR	NR	NR	NR
	Control (no treatment)	28	60 (5)	20/8	NR	NR	NR	NR	NR	NR	NR
Ge Y 2020	Compulsory (Constraint) Exercise Therapy	48	58.65 (3.2)	27/21	NR	NR	NR	NR	NR	NR	NR
	Conventional exercise therapy	48	58.19 (2.11)	25/23	NR	NR	NR	NR	NR	NR	NR
Gelber 1995	Neurophysiological (NDT)	15	Mean = 73.7 SEM = 2.0	9/6	NR	NR	NR	NR	NR	NR	NR
	Orthopaedic (TFR)	12	Mean = 69.8 SEM = 2.9	4/8	NR	NR	NR	NR	NR	NR	NR
Ghasemi 2018	Functional stretching exercises	15	49.67 (13.01)	5/10	NR	NR	NR	NR	NR	NR	NR
	Routine physical therapy	15	54.87 (9.13)	9/6	NR	NR	NR	NR	NR	NR	NR
Green 2002	Mixed	85	71.5 (8.7)	49/36	NR	NR	NR	NR	NR	NR	NR
	Control (no treatment)	85	73.5 (8.3)	46/39	NR	NR	NR	NR	NR	NR	NR
Gong Y 2009	Bilateral training	40	Whole group: 63.5 (15.7) years	Whole group: 46/34	NR	NR	NR	NR	NR	NR	NR
	Unilateral training	40			NR	NR	NR	NR	NR	NR	NR
Guan 2017	Motor relearning training	32	62.66 (9.65)	14/18	NR	NR	NR	NR	NR	NR	NR
	Conventional rehabilitation training	32	61.97 (9.78)	18/14	NR	NR	NR	NR	NR	NR	NR
Guo L 2012	Rehabilitation	46	57.01 (6.45); range: 39‐72	26/20	NR	NR	NR	NR	14 illiterate, 18 primary school and 14 educated to junior high school or above	NR	NR
	Standard care	45	55.06 (8.57); range: 41‐73	27/18	NR	NR	NR	NR	13 illiterate, 22 primary school and 10 educated to junior high school or above	NR	NR
Guo L 2013	Task‐oriented training combined with muscle strength training	21	56.02 (9.23)	13/8	NR	NR	NR	NR	NR	NR	NR
	Facilitation techniques	21	55.25 (10.11)	14/7	NR	NR	NR	NR	NR	NR	NR
Guo Z 2015	Muscle strength exercises and lower limb balance training	40	64.9 (4.7)	22/18	NR	NR	NR	NR	NR	NR	NR
	Routine post‐stroke rehabilitation guidance	40	65.2 (5.8)	21/19	NR	NR	NR	NR	NR	NR	NR
Haral 2014	Sensorimotor Integration	Whole group: 30	NR	NR	NR	NR	NR	NR	NR	NR	NR
	Conventional training		NR	NR	NR	NR	NR	NR	NR	NR	NR
Harjpal 2021	Lower limb bilateral training	20	51.50 (8.40) Range 40‐64	11/9	NR	NR	NR	NR	NR	NR	NR
	Lower limb training to the affected side only	20	51.75 (7.06) Range 40‐63	12/8	NR	NR	NR	NR	NR	NR	NR
Hendrey 2018	Ballistic strength training	15	50.4 (17.0), Range: 18‐76	9/6	NR	NR	NR	NR	NR	NR	NR
	Standard therapy	15	49.3 (18.6), Range: 21‐75	7/8	NR	NR	NR	NR	NR	NR	NR
Holmgren 2006	Intervention	15	77.7 (7.6)	9/6	NR	NR	NR	NR	NR	NR	NR
	Control	19	79.2 (7.5)	12/7	NR	NR	NR	NR	NR	NR	NR
Hong Cuicui 2016	Additional therapy	68	55.32 (11.12)	28/40	NR	NR	NR	NR	Illiterate: 8 Elementary school: 26 Middle school: 20 University: 4	NR	NR
	Conventional rehabilitation	68	56.21 (12.11)	32/36	NR	NR	NR	NR	Illiterate: 6 Elementary school: 32 Middle school: 28 University: 2	NR	NR
Hong Hye Jin 2012	Three‐stage rehabilitation	40	45‐55 years: 11; 55 years+: 29	25/15	NR	NR	NR	NR	NR	NR	NR
	Conventional rehabilitation	40	45‐55 years: 13; 55 years+: 27	23/17	NR	NR	NR	NR	NR	NR	NR
Hoseinabadi 2013	Physiotherapy	12	54.25 (4.97)	NR	NR	NR	NR	NR	NR	NR	NR
	No treatment	12	52.41 (5.46)	NR	NR	NR	NR	NR	NR	NR	NR
Hou 2006	Rehabilitation	40	61.38 (9.99)	25/15	NR	NR	NR	NR	NR	NR	NR
	Control (no treatment)	40	62.55 (9.60)	24/16	NR	NR	NR	NR	NR	NR	NR
Hou Zhi 2014	Three‐level rehabilitation training	58	58.5 (10.3)	35/23	NR	NR	NR	NR	NR	NR	NR
	Conventional rehabilitation training	58	59.2 (11.5)	32/26	NR	NR	NR	NR	NR	NR	NR
Howe 2005	Mixed	17 (15 at 4‐week follow up)	71.5 (10.9)	9/8	NR	NR	NR	NR	NR	NR	NR
	Control (neurophysiological)	18 (18 at 4‐week follow up)	70.7 (17.5)	9/9	NR	NR	NR	NR	NR	NR	NR
Hu 2007	Test (haemorrhagic group)	178	Whole group 61 (10y)	NR	NR	NR	NR	NR	NR	NR	NR
	Control (no treatment)	174		NR	NR	NR	NR	NR	NR	NR	NR
Huang 2003	Rehabilitation	25	64.61 (12.37)	17/8	NR	NR	NR	NR	NR	NR	NR
	Control (no treatment)	25	65.35 (11.71)	17/8	NR	NR	NR	NR	NR	NR	NR
Huang 2014	Bobath	17	64.35 (12.26) Range 42‐82 yrs	12/5	NR	NR	NR	NR	NR	NR	NR
	Rood	17	64.31 (11.76) Range 41‐84 yrs	11/6	NR	NR	NR	NR	NR	NR	NR
	Brunnstorm	17	65.34 (10.26) Range 43‐82 yrs	13/4	NR	NR	NR	NR	NR	NR	NR
	PNF	17	63.31 (15.36) Range 42‐83 yrs	12/5	NR	NR	NR	NR	NR	NR	NR
	Basic treatment (control)	17	64.37 (13.76) Range 41‐84 yrs	10/7	NR	NR	NR	NR	NR	NR	NR
Huang Yangfang 2016	Three‐stage rehabilitation	28	67.9 (2. 4); range: 41‐78	18/10	NR	NR	NR	NR	NR	NR	NR
	No physical rehabilitation	27	66.2 (2.3); range: 40‐78	16/11	NR	NR	NR	NR	NR	NR	NR
Hui‐Chan 2009	PLBO+TRT	25	Whole group mean = 56.6 y SD = 7.9 y	NR	NR	NR	NR	NR	NR	NR	NR
	Control (no treatment)	29		NR	NR	NR	NR	NR	NR	NR	NR
Imhof 2015	Mobility Enhancing Nursing Intervention	70	61.8 (14.5)	38/32	NR	NR	NR	NR	NR	NR	NR
	Standard care	70	62.9 (12.7)	34/36	NR	NR	NR	NR	NR	NR	NR
Indurkar 2013	Task oriented activities + physiotherapy	15	Median (IQR): 55.0 (4.0) years	NR	NR	NR	NR	NR	NR	NR	NR
	Physiotherapy	15	Median (IQR): 54.0 (4.0) years	NR	NR	NR	NR	NR	NR	NR	NR
Jandaghi 2021	Visual deprivation‐stable based training	15	67.2 (9.6)	7/8	NR	NR	NR	NR	NR	NR	NR
	Unstable base training	15	68.8 (8.9)	9/6	NR	NR	NR	NR	NR	NR	NR
	General physiotherapy exercise (Control)	15	67.5 (9.9)	8/7	NR	NR	NR	NR	NR	NR	NR
Jeon 2018	Bilateral lower limb strengthening exercise	10	42.3 (4.2)	6/4	NR	NR	NR	NR	NR	NR	NR
	Unilateral therapy (paretic lower limb)	10	43.6 (5.6)	7/3	NR	NR	NR	NR	NR	NR	NR
Ji Pei 2014	Bobath technique‐based exercise therapy	48	Whole group: Mean: 65 years; range: 39‐75 years	56/34	NR	NR	NR	NR	NR	NR	NR
	Routine treatment (no physical rehabiltation)	42			NR	NR	NR	NR	NR	NR	NR
Jing 2006	Exercise and occupational therapy	120	57.3 (12.5)	69/51	NR	NR	NR	NR	NR	NR	NR
	Exercise therapy	40	54.5 (9.6)	23/17	NR	NR	NR	NR	NR	NR	NR
Jongbloed 1989	Sensorimotor Integration	43	Whole group: Mean (SD): 71.32 (9.07) years	Whole group: 41M/49F	NR	NR	NR	NR	NR	NR	NR
	Functional training	47			NR	NR	NR	NR	NR	NR	NR
Khallaf 2014	Task specific exercises, gait training, and visual biofeedback	8	40.38 (2.67)	6/2	NR	NR	NR	NR	NR	NR	NR
	Traditional physical therapy program	8	41.25 (3.11)	6/2	NR	NR	NR	NR	NR	NR	NR
Kim 2007	Task‐related circuit training	12	66.9 (4.5)	8/4	NR	NR	NR	NR	NR	NR	NR
	Conventional physical therapy	12	65.4 (6.4)	4/5	NR	NR	NR	NR	NR	NR	NR
Kim 2012	Experimental	10	52.5 (11.72)	NR	NR	NR	NR	NR	NR	NR	NR
	Control	10	53.4 (12.11)	NR	NR	NR	NR	NR	NR	NR	NR
Kim 2012a	Rhythmic auditory stimulation gait training group	10	58.3 (11.8)	6/4	NR	NR	NR	NR	NR	NR	NR
	Conventional physical therapy	10	51.8 (13.7)	7/3	NR	NR	NR	NR	NR	NR	NR
Kim 2014	Gross motor group exercise group plus conventional	14	58.2 (10.3)	14/0	NR	NR	NR	NR	NR	NR	NR
	Conventional therapy (morning bed exercise)	14	55.9 (10.1)	14/0	NR	NR	NR	NR	NR	NR	NR
Kim 2016	Inpatient circuit training program	10	65.2 (10.1)	6/4	NR	NR	NR	NR	NR	NR	NR
	individual neurodevelopmental physiotherapy	10	66.0 (8.8)	7/3	NR	NR	NR	NR	NR	NR	NR
Kim 2017	Task‐oriented circuit training	15	57.3 (12.3)	10/5	NR	NR	NR	NR	NR	NR	NR
	Conventional physical therapy	15	54.0 (11.8)	9/6	NR	NR	NR	NR	NR	NR	NR
Kim 2018	Coordinative locomotor training	7	59.57 (11.75)	6/1	NR	NR	NR	NR	NR	NR	NR
	Conventional neurodevelopment treatment	6	64.5 (13.03)	5/1	NR	NR	NR	NR	NR	NR	NR
Kim 2021	Task‐specific training after cognitive sensorimotor exercise	13	50.23 (14.89)	7/6	NR	NR	NR	NR	NR	NR	NR
	Task‐specific training	12	52.75 (17.0)	7/5	NR	NR	NR	NR	NR	NR	NR
	Conventional physical therapy	12	55.08 (10.55)	8/4	NR	NR	NR	NR	NR	NR	NR
Knox 2018	Task‐oriented training	51	51 (15)	25/26	NR	NR	Employment status: employed: 33, unemployed 18	NR	NR	Income level: US$/month: <US$75: 16, US$75‐224: 11, >US$224: 24	Carer: Relative: 45, Other: 6
	Strength training of lower extremities	45	51 (12)	25/20	NR	NR	Employment status: employed: 33, unemployed 12	NR	NR	Income level: US$/month: <US$75: 14, US$75‐224: 18, >US$224: 13	Carer: Relative: 41, Other: 4
	No physical therapy	48	48 (14)	22/26	NR	NR	Employment status: employed: 33, unemployed 15	NR	NR	Income level: US$/month: <US$75: 15, US$75‐224: 14, >US$224: 19	Carer: Relative: 42, Other: 6
Koç 2015	Home‐based exercise	Whole group: 134	Whole group: 67 years	NR	NR	NR	NR	NR	NR	NR	NR
	Usual care (no treatment)			NR	NR	NR	NR	NR	NR	NR	NR
Krawczyk 2014	Closed chain exercises	Whole group 51	Whole group: Mean (SD): 59 (10) years	Whole group: 38/13	NR	NR	NR	NR	NR	NR	NR
	open chain exercises				NR	NR	NR	NR	NR	NR	NR
Krukowska 2016	Bobath – Neurodevelopmental Treatment (NDT‐Bobath)	38	Whole group: mean age: 53.7 years; range: 20‐69 years	17/21	NR	NR	NR	NR	NR	NR	NR
	Proprioceptive Neuromuscular Facilitation (PNF)	34		15/19	NR	NR	NR	NR	NR	NR	NR
Kuberan 2017	Task oriented training	13	58.82 (9.12)	9/4	NR	NR	NR	NR	NR	NR	NR
	Conventional physical therapy	13	60.07 (7.56)	9/4	NR	NR	NR	NR	NR	NR	NR
Kumaran 2015	Task and context based exercise program	34	Not stated	Not stated	NR	NR	NR	NR	NR	NR	NR
	Conventional training	32	Not stated	Not stated	NR	NR	NR	NR	NR	NR	NR
Kunkel 2013	Balance training	7	71.1 (18.8)	4/3	NR	NR	NR	NR	NR	NR	Living status: lives alone: 4, living with partner:3
	Usual care	7	70 (10.6)	4/3	NR	NR	NR	NR	NR	NR	Living status: lives alone: 2 , living with partner: 2, family friends: 3
Kwakkel 2002	Lower extremities	17	60.8 (10.6) Range 38‐76 yrs	13/4	NR	NR	NR	NR	NR	NR	NR
	Upper extremities	18	64.3 (10.6) Range 46‐80 yrs	9/9	NR	NR	NR	NR	NR	NR	NR
	Control	18	62.1 (10.6) Range 30‐76 yrs	14/4	NR	NR	NR	NR	NR	NR	NR
Kwakkel 2008	Circuit training	126	56 (10)	82/44	NR	NR	NR	NR	NR	NR	NR
	Usual physiotherapy	124	58 (10)	80/44	NR	NR	NR	NR	NR	NR	NR
Langhammer 2000	Neurophysiological (Bobath)	28	Whole group Mean = 78(9) Range 49‐ 95 yrs	16/12	NR	NR	NR	NR	NR	NR	NR
	Motor learning	33		20/13	NR	NR	NR	NR	NR	NR	NR
Langhammer 2007	Intensive exercise	35	76 (12.7)	NR	NR	NR	NR	NR	NR	NR	NR
	Regular exercise	40	72 (13.6)	NR	NR	NR	NR	NR	NR	NR	NR
LAST 2018	Individualised coaching	186	71.7 (11.9)	104/82	NR	NR	NR	NR	NR	NR	Living with someone: 130 living alone: 56
	Usual care	194	72.3 (11.3)	127/67	NR	NR	NR	NR	NR	NR	Living with someone: 143 living alone: 51
Lawal 2016	Circuit training	21	53.5 (12.4)	13/8	NR	Hausa: 14 Yoruba: 2 Igbo: 1 Others: 4	Employment: unemployed: 5, elementary occupation: 6, crafts/related trades: 7, technician/related workers: 2, managers/professionals: 1	NR	Level of education: Nil: 5, Primary: 6, Secondary: 7, Tertiary: 3	NR	Marital status: Married: 21
	Standard physiotherapy	23	50.5 (9.5)	14/9	NR	Hausa: 17 Yoruba: 2 Igbo: 1 Others: 3	Employment: unemployed: 5, elementary occupation: 12, crafts/related trades: 3, technician/related workers: 1, managers/professionals: 12	NR	Level of education: Nil: 7, Primary: 10, Secondary: 3, Tertiary: 3	NR	Marital status: Divorced: 1, Married: 20, Widow: 2
Lee 2015	Combined aerobic and resistance exercise	15 (data reported for 13)	64 (7.4)	NR	NR	NR	NR	NR	NR	NR	NR
	Unsystematic physical activities or played Korean chess	15 (data reported for 12)	63 (5.45)	NR	NR	NR	NR	NR	NR	NR	NR
Lee 2018	Conventional rehabilitation followed by caregiver‐Mediated Exercise	40 (data reported for 35)	60.1 (6.4)	19/16	NR	NR	NR	NR	NR	NR	NR
	Conventional rehabilitation followed by no treatment	40 (data reported for 37)	59.3 (6.4)	20/17	NR	NR	NR	NR	NR	NR	NR
Lennon 2006	Bobath	30	NR	NR	NR	NR	NR	NR	NR	NR	
	Gait specific group	31	NR	NR	NR	NR	NR	NR	NR	NR	
Letombe 2010	Early post‐stroke physical exercises	9	59.1 (9.4)	5/4	NR	NR	NR	NR	NR	NR	NR
	Conventional rehabilitation	9	60.6 (8.2)	6/3	NR	NR	NR	NR	NR	NR	NR
Li 1999	Early rehabilitation	30	58.1 (11.9)	NR	NR	NR	NR	NR	NR	NR	NR
	Control (no treatment)	31	59.20 (10.2)	NR	NR	NR	NR	NR	NR	NR	NR
Li 2005	Motor relearning	31	51.4 (8.9)	NR	NR	NR	NR	NR	NR	NR	NR
	Neurodevelopmental therapy	30	54.6 (9.9)	NR	NR	NR	NR	NR	NR	NR	NR
Li 2013	Early intensive walking basic skills training	88	56.2 (14.6)	51/37	NR	NR	NR	NR	NR	NR	NR
	Conventional rehabilitation training	88	56.3 (12.9)	52/36	NR	NR	NR	NR	NR	NR	NR
Li Jingqian 2013	Motor re‐learning program (MRP)	60	Mean: 63.24 years; range: 13‐83 years	45/15	NR	NR	NR	NR	NR	NR	NR
	Conventional rehabilitation	60	Mean: 62.32 years; range: 11‐85 years	43/17	NR	NR	NR	NR	NR	NR	NR
Li Xiaojun 2016	Task‐oriented training	37	55.7 (3.4)	26/11	NR	NR	NR	NR	NR	NR	NR
	Conventional rehabilitation	37	54.1 (4.2)	24/13	NR	NR	NR	NR	NR	NR	NR
Li Weiwei 2015	Task‐oriented training	30	55.37 (15.89)	20/10	NR	NR	NR	NR	NR	NR	NR
	Routine rehabilitation	30	58.17 (13.01)	21/9	NR	NR	NR	NR	NR	NR	NR
Li Yuanzheng 2014	Bobath therapy	60	Not stated	34/26	NR	NR	NR	NR	NR	NR	NR
	Conventional treatment	62	Not stated	22/40	NR	NR	NR	NR	NR	NR	NR
Li Yuanzheng 2014a	Motor relearning	28	Not stated	NR	NR	NR	NR	NR	NR	NR	NR
	routine rehabilitation training	30	Not stated	NR	NR	NR	NR	NR	NR	NR	NR
Lincoln 2003	Neurophysiological (Bobath)	60	73.3 (10.4)	27/33	NR	NR	NR	NR	NR	NR	NR
	Motor learning	60	75.0 (9.1)	33/27	NR	NR	NR	NR	NR	NR	NR
Lindvall 2014	Body awareness therapy	24	62.1 (11.4) years; range 42‐82	12/12	NR	NR	NR	NR	NR	NR	NR
	No intervention	22	65.6 (9.2) years; range 42‐81	15/7	NR	NR	NR	NR	NR	NR	NR
Liu 2014	Task oriented training	40	57.39 (8.71)	28/12	NR	NR	NR	NR	NR	NR	NR
	Conventional rehabilitation	40	58.72 (9.11)	27/13	NR	NR	NR	NR	NR	NR	NR
Liu Yanhua 2020	Compulsory exercise therapy	42 (Paper states 60)	Not stated	34/26	NR	NR	NR	NR	NR	NR	NR
	Routine exercise therapy	42 (Paper states 62)	Not stated	22/40	NR	NR	NR	NR	NR	NR	NR
Liu Xuan 2016	Three‐stage rehabilitation	30	62.87 (2.87)	16/14	NR	NR	NR	NR	NR	NR	NR
	Conventional rehabilitation	30	62.17 (2.67)	15/15	NR	NR	NR	NR	NR	NR	NR
Lu 2004	Three‐stage Rehabilitation	21	Whole group mean (SD): 58.5 (12.7)	Whole group: 27/15	NR	NR	NR	NR	NR	NR	NR
	Standard care	21			NR	NR	NR	NR	NR	NR	NR
Lu 2014	Three‐stage Rehabilitation	90	66.7 (5.1); range: 45‐72	48/42	NR	NR	NR	NR	NR	NR	NR
	Standard care	90	67.3 (3.2); range: 44‐71	51/39	NR	NR	NR	NR	NR	NR	NR
Lu Liangyan 2014	Three‐level rehabilitation treatment	44	Whole group mean: 56.16 (6.81); range: 48‐73	Whole group: 47/41	NR	NR	NR	NR	NR	NR	NR
	Routine rehabilitation guidance	44			NR	NR	NR	NR	NR	NR	NR
Mai Guanghuai 2016	Lower limb training	45	57. 8 (9.8); range: 38‐74	28/17	NR	NR	NR	NR	NR	NR	NR
	Conventional rehabilitation	45	59. 1 (9.6); range: 42‐75	25/20	NR	NR	NR	NR	NR	NR	NR
Mansfield 2018	Perturbation‐based balance training	44 (based on 41)	66 (17)	26/15	NR	NR	NR	NR	NR	NR	NR
	Keep Moving with Stroke programme (balance and mobility exercise programme for community‐dwelling individuals)	44 (based on 42)	67 (13)	30/12	NR	NR	NR	NR	NR	NR	NR
Marigold 2005	Agility exercise	30 (based on 22)	68.1 (9)	17/8	NR	NR	NR	NR	NR	NR	NR
	Stretching/weight‐shifting exercise	31 (based on 26)	67.5 (7.2)	18/8	NR	NR	NR	NR	NR	NR	NR
Martins 2020	Task‐specific circuit training	18	56 (17)	8/10	NR	NR	NR	NR	NR	NR	NR
	Global stretching, memory exercises, and education sessions	18	55 (13)	8/10	NR	NR	NR	NR	NR	NR	NR
Matthew Hall 2013	Task‐oriented training	20	30 (9.72)	16/4	NR	NR	NR	NR	NR	NR	NR
	Standard rehabitation	20	32 (8.94)	14/6	NR	NR	NR	NR	NR	NR	NR
Ma Xue 2010	Rehabilitation	31	63.06 (10.19)	17/14	NR	NR	NR	NR	NR	NR	NR
	Attention control	29	65.86 (11.46)	14/15	NR	NR	NR	NR	NR	NR	NR
McClellan 2004	Motor learning	15	69 (13)	10/3 (at end of intervention)	NR	NR	NR	NR	NR	NR	NR
	Placebo (upper limb control)	11	72 (9)	2/8 (at end of intervention)	NR	NR	NR	NR	NR	NR	NR
Medina‐Rincón 2019	Balance impairment exercise programme	7	Median (IQR): 70 (17)	NR	NR	NR	NR	NR	NR	NR	NR
	Usual rehabilitation	7	Median (IQR): 71 (23)	NR	NR	NR	NR	NR	NR	NR	NR
Meier 2021	Coordinative training treatment group	3	Not stated	NR	NR	NR	NR	NR	NR	NR	NR
	Conventional physical therapy	4	Not stated	NR	NR	NR	NR	NR	NR	NR	NR
Mendoza 2015	Task‐oriented circuit class	9	47.2 (8.8)	9/0	NR	NR	NR	NR	NR	NR	NR
	Impairment‐focused circuit class	9	49 (11.2)	7/2	NR	NR	NR	NR	NR	NR	NR
Meng 2022	Robot‐assisted gait training (not relevant to this review)	64 (based on 62)	59.36 (1.65)	33/29	NR	NR	NR	NR	NR	NR	NR
	Enhanced lower limb therapy	64	55.26 (1.32)	35/29	NR	NR	NR	NR	NR	NR	NR
	Conventional rehabilitation	64 (based on 61)	60.12 (1.73)	35/26	NR	NR	NR	NR	NR	NR	NR
Meng Fanda 2021	Routine rehabilitation training	20	53.21 (2.01); range: 35‐74	12/8	NR	NR	NR	NR	NR	NR	NR
	Routine rehabilitation nursing	20	53.52 (2.11); range: 36‐75	13/7	NR	NR	NR	NR	NR	NR	NR
Meng Qingling 2015	Task functional training	45	63.4 (4.9)	25/20	NR	NR	NR	NR	NR	NR	NR
	Strength training	45	62.1 (6.6)	25/20	NR	NR	NR	NR	NR	NR	NR
Mikolajewska 2017	Bobath neuro‑developmental treatment (NDT‐Bobath)	15	54.85 (12.77)	8/7	NR	NR	NR	NR	NR	NR	NR
	Traditional treatment	15	52.5 (13.2)	9/6	NR	NR	NR	NR	NR	NR	NR
Mohaideen 2014	Symmetrical weight bearing + conventional stroke training	15	Not stated	NR	NR	NR	NR	NR	NR	NR	NR
	Conventional stroke training	15	Not stated	NR	NR	NR	NR	NR	NR	NR	NR
Moore 2016	Community group exercise programme	20	Whole group mean (SD): 69 (9)	Whole group: 34/6	NR	NR	NR	NR	NR	NR	NR
	Matched duration home stretching programme	20			NR	NR	NR	NR	NR	NR	NR
Morreale 2016	Proprioceptive Neuromuscular Facilitation (early)	110	64 (14)	80/30	NR	NR	NR	NR	NR	NR	NR
	Cognitive therapeutic exercise (early)	110	63 (12)	81/29	NR	NR	NR	NR	NR	NR	NR
	Proprioceptive Neuromuscular Facilitation (standard)	60	63 (15)	42/18	NR	NR	NR	NR	NR	NR	NR
	Cognitive therapeutic exercise (standard)	60	64 (13)	43/17	NR	NR	NR	NR	NR	NR	NR
Mudge 2009	Exercise	31	Median = 76.0 y Range = 39.0–89.0 y	19/12	NR	NR	NR	NR	NR	NR	NR
	Control	27	Median = 71.0 y Range = 44.0–86.0 y	13/14	NR	NR	NR	NR	NR	NR	NR
Mustafaoğlu 2018	Body weight support treadmill training (BWSTT)	15	53.7 (11.6)	11/4	NR	NR	NR	NR	NR	NR	NR
	BWSTT + conventional therapy	15	52.8 (13.8)	10/5	NR	NR	NR	NR	NR	NR	NR
Nagy 2017	Conductive education	11 (based on 9)	Median (IQR): 56 (54‐65)	5/4	NR	NR	NR	NR	NR	NR	NR
	Conventional physiotherapy	9 (based on 8)	Median (IQR): 52 (48‐63) years	5/3	NR	NR	NR	NR	NR	NR	NR
Ni 1997	Comprehensive rehabilitation training	34	55.56 (17.64)	26/8	NR	NR	NR	NR	NR	NR	NR
	Control (no treatment)	34	53.25 (13.46)	23/11	NR	NR	NR	NR	NR	NR	NR
Nindorera 2022	Physical rehabilitation (IG)	23	50.9 (10.7)	18/5	NR	NR	NR	NR	NR	NR	NR
	Attention control (DG)	23	50.1 (11.2)	18/5	NR	NR	NR	NR	NR	NR	NR
Outermans 2010	High‐intensity task‐oriented training	23 (based on 22)	56.8 (8.6)	19/3	NR	NR	NR	NR	NR	NR	NR
	Low‐intensity physiotherapy	21	56.3 (8.6)	17/4	NR	NR	NR	NR	NR	NR	NR
Pan 2004	Rehabilitation	48	64.2 (11.5)	36/12	NR	NR	NR	NR	NR	NR	NR
	Control	48	62.5 (13.7)	32/16	NR	NR	NR	NR	NR	NR	NR
Pandian 2014	Motor training of both sides	20	44.5 (13.59)	11/8*	NR	NR	NR	NR	NR	NR	NR
	Conventional (standard motor rehabilitation based on Brunnstrom’s movement therapy)	19	40.16 (14.96)	10/10*	NR	NR	NR	NR	NR	NR	NR
Pang 2003	Rehabilitation	50	Mean = 61.4 Range: 37‐76 y	32/18	NR	NR	NR	NR	NR	NR	NR
	Control (no treatment)	36	Mean = 60 Range: 39‐75 y	25/11	NR	NR	NR	NR	NR	NR	NR
Pang 2005	Community‐based Fitness and Mobility Exercise (FAME) Programme	32	65.8 (9.1)	19/13	Race: Caucasian: 20; Asian: 11; Black: 1	NR	NR	Years of education (SD):13.9 (3.8)	NR	NR	NR
	Seated upper extremity programme	31	64.7 (8.4)	18/13	Race: Caucasian: 18; Asian: 13; Black: 0	NR	NR	Years of education (SD):13.9 (3.4)	NR	NR	NR
Pang 2006	Treatment	41	Not stated	NR	NR	NR	NR	NR	NR	NR	NR
	Control (no treatment)	39	Not stated	NR	NR	NR	NR	NR	NR	NR	NR
Pang 2018	Single‐Task Exercise	28	61.2 (6.2)	20/8	NR	NR	NR	NR	NR	NR	NR
	Upper‐limb exercise group	28	62.4 (6.3)	18/10	NR	NR	NR	NR	NR	NR	NR
Park 2021	Affected side cross‐training	20 (based on 15)	58.07 (7.14)	12/3	NR	NR	NR	NR	NR	NR	NR
	Unaffected side cross‐training	20 (based on18)	58.11 (7.15)	11/7	NR	NR	NR	NR	NR	NR	NR
	General neurological physiotherapy	20 (based on 19)	60.79 (6.75)	10/9	NR	NR	NR	NR	NR	NR	NR
Pirayesh 2021	Otago exercises group	23	Whole group mean 63.81 (6.13) (based on 69 participants from 3 groups)	Whole group: 36/33	NR	NR	Whole group: Housewife: 32; Manual worker: 4; Employee: 5; Others: 28	NR	Whole group educational status: Illiterate: 39; Below diploma: 21; Diploma: 8; Above diploma: 1	NR	Marital status: Married: 55; Widow/widower: 14
	No treatment	23			NR	NR		NR		NR	
Puckree 2014	Balance and stability exercise programme	25	Age groups: 0‐34 years: 0; 35‐49 years: 2; 50‐74 years: 22; > 75 years: 1	11/14	NR	NR	NR	NR	NR	NR	NR
	Regular physiotherapy programme	25	Age groups: 0‐34 years: 1; 35‐49 years: 6; 50‐74 years: 16; > 75 years: 2	15/10	NR	NR	NR	NR	NR	NR	NR
Qian 2004	Treatment	23	62.8 (14.3)	11/12	NR	NR	NR	NR	NR	NR	NR
	Control (no treatment)	19	62.8 (17.2)	9/10	NR	NR	NR	NR	NR	NR	NR
Qin 2013	Motor re‐learning programme (MRP)	20	59.95 (6.35); Range: 51‐75	12/8	NR	NR	NR	NR	NR	NR	NR
	Motor re‐learning programme (MRP) + acupuncture	20	60.95 (7.12)	11/9	NR	NR	NR	NR	NR	NR	NR
	Acupuncture	20	60 (6.47)	12/8	NR	NR	NR	NR	NR	NR	NR
Qin JianJian 2014	Bobath treatment	30	55.38 (6.66)	17/13	NR	NR	NR	NR	NR	NR	NR
	Conventional rehabilitation training	31	54.46 (6.48)	11/20	NR	NR	NR	NR	NR	NR	NR
Rahayu 2020	Neurorestoration	34 (based on 32)	58.84 (8.681)	20/12	NR	NR	NR	NR	NR	NR	NR
	Conventional	33 (based on 32)	59.93 (10.647)	16/16	NR	NR	NR	NR	NR	NR	NR
Renner 2016	Group task training	34	56 (10)	22/12	NR	NR	NR	NR	NR	NR	NR
	Individual task training	39	55 (10)	29/10	NR	NR	NR	NR	NR	NR	NR
ReTrain 2018	Community‐based rehabilitation training (ReTrain)	23	70 (12)	16/7	NR	NR	NR	NR	NR	NR	NR
	No training (exercise after stroke advice booklet)	22	71 (10)	14/8	NR	NR	NR	NR	NR	NR	NR
Richards 1993	Mixed (early)	10	69.6 (7.4)	5/5	NR	NR	NR	NR	NR	NR	NR
	Neurophysiological (early)	8	67.3 (11.2)	2/6	NR	NR	NR	NR	NR	NR	NR
	Neurophysiological (conventional)	9	70.3 (7.3)	6/3	NR	NR	NR	NR	NR	NR	NR
Salbach 2004	Motor learning	44	71 (12)	26/18	NR	NR	NR	NR	NR	NR	NR
	Placebo (upper limb control)	47	73 (8)	30/17	NR	NR	NR	NR	NR	NR	NR
Sekhar 2013	Isokinetic Strength Training and Balance Exercises	20	Whole group: range: 30‐50 years	NR	NR	NR	NR	NR	NR	NR	NR
	Conventional physiotherapy	20		NR	NR	NR	NR	NR	NR	NR	NR
Seo 2015	Ramp gait exercise + proprioceptive neuromuscular facilitation (PNF) gait pattern traning	15	62.1 (6.2)	6/4	NR	NR	NR	NR	NR	NR	NR
	PNF gait pattern training	15	60.5 (2.1)	5/5	NR	NR	NR	NR	NR	NR	NR
Severinsen 2014	Resistance training	14	Median: 68; range: 57‐78	11/3	NR	NR	NR	NR	NR	NR	NR
	Low‐intensity sham training	17 (based on 16)	Median: 66; range: 52‐80	11/5	NR	NR	NR	NR	NR	NR	NR
Shin 2011	Combined Exercise	11	58.1 (4.6)	5/6	NR	NR	NR	NR	NR	NR	NR
	Conventional exercise	10	57.3 (4.4)	3/7	NR	NR	NR	NR	NR	NR	NR
Shuai 2013	Motor relearning programme	33	57.8 (11.2)	27/6	NR	NR	NR	NR	NR	NR	NR
	Routine rehabilitation therapy	29	61.7 (9.2)	21/8	NR	NR	NR	NR	NR	NR	NR
Signal 2014	Strength and task‐specific training (STT)	5	Not stated	NR	NR	NR	NR	NR	NR	NR	NR
	Strength training (PRST)	5	Not stated	NR	NR	NR	NR	NR	NR	NR	NR
	Task‐specific training (TST)	5	Not stated	NR	NR	NR	NR	NR	NR	NR	NR
	Usual care (UCC)	5	Not stated	NR	NR	NR	NR	NR	NR	NR	NR
Song 2015	Conventional physiotherapy	10	62.78 (9.97)	NR	NR	NR	NR	NR	NR	NR	NR
	Individual‐based task‐oriented circuit training + conventional physiotherapy	10	64.1 (8.61)	NR	NR	NR	NR	NR	NR	NR	NR
	Class‐based task‐oriented circuit training + conventional physiotherapy	10	59.28 (5.23)	NR	NR	NR	NR	NR	NR	NR	NR
SPIRES 2022	Functional standing frame programme plus usual physiotherapy	22	81.7 (11.7)	10/12	NR	NR	NR	NR	NR	NR	NR
	Usual physiotherapy	23	78.9 (10.5)	9/14	NR	NR	NR	NR	NR	NR	NR
Stephenson 2004	Body Weight Support Treadmill Training	6	Whole group Mean = 59.8 Range: 42‐80 y	NR	NR	NR	NR	NR	NR	NR	NR
	Proprioceptive Neuromuscular Facilitation‐PNF training	6		NR	NR	NR	NR	NR	NR	NR	NR
	Control (no treatment)	6		NR	NR	NR	NR	NR	NR	NR	NR
Stuart 2019	Adaptive Physical Activity exercise programme for stroke survivors (APA‐Stroke)	43	Mean (SE): 63 (1.41) (n = 42)	22/21	NR	NR	NR	NR	NR	NR	NR
	Sittercise (seated, nonprogressive aerobic upper body general exercise programme)	33	Mean (SE): 65 (2.07) (n = 32)	17/16	NR	NR	NR	NR	NR	NR	NR
Sun Juanjuan 2014	Motor function rehabilitation training and acupoint pressing	50	72.6 (9.8)	30/20	NR	NR	NR	NR	NR	NR	NR
	Routine nursing	50	73.8 (10.3)	28/22	NR	NR	NR	NR	NR	NR	NR
SunRISe 2021	Home‐based physiotherapy intervention	20	61.6 (9.1)	9/11	NR	Ethnic background: Asian: 10; African: 7; Other: 3	NR	NR	NR	NR	NR
	Usual care	10	62.2 (9.1)	4/6	NR	Ethnic background: Asian: 6; African: 2; Other: 2	NR	NR	NR	NR	NR
Tang 2009	Observation	35	Whole group mean = 61.98 Range: 44‐75 y	11/9*	NR	NR	NR	NR	NR	NR	NR
	Control	35		12/8*	NR	NR	NR	NR	NR	NR	NR
Tang Yao 2015	Three‐stage rehabilitation	50	61.21 (10.21); range: 40‐78	36/14	NR	NR	NR	NR	NR	NR	NR
	No physical rehabilitation	50	62.31 (12.33); range: 13‐79	40/10	NR	NR	NR	NR	NR	NR	NR
Teixeira‐Salmela 1999	Aerobic exercise and muscle strength training	6	65.87 (10.16)	1/5	NR	NR	NR	NR	NR	NR	NR
	No treatment	7	69.42 (8.85)	6/1	NR	NR	NR	NR	NR	NR	NR
Thaut 2007	Rhythmic auditory stimulation	43	69.2 (11)	22/21	NR	NR	NR	NR	NR	NR	NR
	Neurodevelopmental therapy (NDT)/Bobath− based training	35	69.7 (11)	19/16	NR	NR	NR	NR	NR	NR	NR
Torres‐Arreola 2009	Strategy 1	59	69.4 (12)	16/43	NR	NR	NR	NR	NR	NR	NR
	Strategy 2	51	69.8 (8.8)	21/30	NR	NR	NR	NR	NR	NR	NR
Tyson 2015	Patient‐led upper‐limb mirror therapy	63	64 (15)	37/25	NR	NR	NR	NR	NR	NR	NR
	Patient‐led lower leg exercises	31	64 (13)	23/8	NR	NR	NR	NR	NR	NR	NR
Vahlberg 2017	Progressive resistance and balance exercise programme	20	72.7 (5.5)	17/3	NR	NR	NR	NR	NR	NR	NR
	Usual care	23	73.7 (5.4)	16/7	NR	NR	NR	NR	NR	NR	NR
Verma 2011	Experimental	15	53.27 (8.53)	10/5	NR	NR	NR	NR	NR	NR	NR
	Control	15	55.07 (6.80)	12/3	NR	NR	NR	NR	NR	NR	NR
Wade 1992	Mixed	49	72.3 (9.7)	27/22	NR	NR	NR	NR	NR	NR	NR
	Control (no treatment)	45	72.0 (10.6)	20/25	NR	NR	NR	NR	NR	NR	NR
Wan Xueli 2014	Bobath therapy	24	62.3 (10.9); Range: 52‐75	13/11	NR	NR	NR	NR	NR	NR	NR
	Usual care (no physical therapy)	24	61.5 (11.2); range: 51‐76	14/10	NR	NR	NR	NR	NR	NR	NR
Wang 2004a	Rehabilitation	70	63.1 (9.8)	36/30	NR	NR	NR	NR	NR	NR	NR
	Control (no treatment)	35	65.2 (11.3)	18/14	NR	NR	NR	NR	NR	NR	NR
Wang 2004b	Treatment	25	62.1 (10.2)	16/9	NR	NR	NR	NR	NR	NR	NR
	Control (no treatment)	25	59.5 (11.4)	15/10	NR	NR	NR	NR	NR	NR	NR
Wang 2005	Neurophysiological	21	Patients with spasticity 53.9 (11.8) Patients with relative recovery 62.4 (11.6)	14/7	NR	NR	NR	NR	NR	NR	NR
	Orthopaedic	23	Patients with spasticity 59.3 (12.2) Patients with relative recovery 63.8 (13.1)	14/9	NR	NR	NR	NR	NR	NR	NR
Wang 2013	Rehabilitation exercises (Bobath)	65	Range: 43‐72	38/27	NR	NR	NR	NR	NR	NR	NR
	Usual care (no physical therapy)	65	Range: 41‐74	38/27	NR	NR	NR	NR	NR	NR	NR
Wang 2015	Caregiver‐mediated Intervention	25	62 (9.5)	13/12	Type of housing: Apartment with elevator: 8; Apartment without elevator: 4; Single homes without elevator: 13; Home with mobility barriers: Yes: 16; No: 9	NR	NR	NR	Caregiver's level of education: Elementary school: 4; Junior High School: 5; Senior High School:11; College or higher: 3; Unknown (foreign caregivers): 2	NR	Relationship with the caregiver: Spouse: 18; Adult children: 4; Close relatives or friends: 2; Paid caregiver: 1
	No treatment	26	65.4 (10.6)	17/9	Type of housing: Apartment with elevator: 6; Apartment without elevator: 4; Single homes without elevator: 16. Home with mobility barriers: Yes: 22; No: 4	NR	NR	NR	Caregiver's level of education: Elementary school: 10; Junior High School: 5; Senior High School:4; College or higher: 2; Unknown (foreign caregivers): 5	NR	Relationship with the caregiver: Spouse: 18; Adult children: 3; Close relatives or friends: 2; Paid caregiver: 3
Wang 2021	Rehabilitation Nursing	121	64.35 (10.59)	79/29	NR	NR	NR	NR	Educational background: Illiteracy: 10, Primary school: 33, Junior high school: 33, High school/technical secondary school: 20, College degree and above: 12	Medical payment: local medical insurance: 53, Offsite medical insurance: 36, No Medical insurance: 19	NR
	Usual therapist‐led treatment	103	61.36 (15.27)	68/33	NR	NR	NR	NR	Educational background: Illiteracy: 11, Primary school: 36, Junior high school: 33, High school/technical secondary school: 20, College degree and above: 1	Medical payment: local medical insurance: 55, Offsite medical insurance: 27, No Medical insurance: 19	NR
Wang 2022	Nurse‐led rehabilitation	52	64.35 (10.59)	23/20*	NR	NR	NR	NR	Educational background: Illiterate: 10, Primary school: 12, Junior high school: 8, High school and above: 13	Medical payment: medical insurance for urban workers: 8, Medical insurance for urban residents: 13, Self‐paying: 22	NR
	Usual care	52	61.36 (15.27)	26/19*	NR	NR	NR	NR	Educational background: Illiterate: 7, Primary school: 14, Junior high school: 17, High school and above: 7	Medical payment: medical insurance for urban workers: 5, Medical insurance for urban residents: 20, Self‐paying: 20	NR
Wang Dongya 2015	Three‐stage rehabilitation	63	63.2 (8.9) Range: 45‐88	52/11	NR	NR	NR	NR	NR	NR	NR
	Usual care	63	62.4 (8.5) Range: 46‐87	48/15	NR	NR	NR	NR	NR	NR	NR
Wang Leilei 2020	Task‐oriented training + acupuncture	100	44.9 (2.1); range: 39‐74	56/44	NR	NR	NR	NR	NR	NR	NR
	Conventional rehabilitation + acupuncture	100	43.2 (2.9); range: 37‐75	58/42	NR	NR	NR	NR	NR	NR	NR
Wang Wenwei 2012	Bobath training	40	58.23 (12.85)	20/20	NR	NR	NR	NR	NR	NR	NR
	Conventional rehabilitation	40	57.18 (13.16)	23/17	NR	NR	NR	NR	NR	NR	NR
Wei 2014	Three‐stage rehabilitation (all stages)	40	65.32 (12.43)	28/12	NR	NR	NR	NR	NR	NR	NR
	Three‐stage rehabilitation (1st and 2nd stages only)	40	65.25 (12.67)	28/12	NR	NR	NR	NR	NR	NR	NR
	Three‐stage rehabilitation (1st and 3rd stages only)	40	65.58 (12.39)	28/12	NR	NR	NR	NR	NR	NR	NR
Werner 1996	Intensive outpatient rehabilitation programme	33 (data reported for 28)	59 (9)	14/14*	NR	NR	NR	NR	NR	NR	NR
	No treatment	16 (data reported 12)	66 (13)	5/7*	NR	NR	NR	NR	NR	NR	NR
Wu 2006	Rehabilitation	50	61 (8.69)	29/19							
	Control (no treatment)	50	63.13 (7.79)	35/13							
Wu 2020	Collaborative Care Model Based Telerehabilitation Exercise Training Programme	32	56.73 (11.85)	19/11	Whole group: Urban residence accounts for 46.7%	NR	NR	NR	Whole group mean education (SD) years: 8.78 (3.95)	NR	Caregiver: Spouse: 23, Child: 6, Others: 1
	Usual care	32	59.1 (8.6)	17/14		NR	NR	NR		NR	Caregiver: Spouse: 19, Child: 9, Others: 3
Wu Jiaming 2006	Rehabilitation	40	Whole group: 64.3 (10.5) years	Whole group: 46/34	NR	NR	NR	NR	NR	NR	NR
	Conventional rehabilitation	40			NR	NR	NR	NR	NR	NR	NR
Wu Jing 2015	Three‐stage rehabilitation	46	35.2 (3.6) Range: 18‐44	39/7	NR	NR	NR	NR	NR	NR	NR
	Conventional treatment patients self exericse)	46	35.0 (3.4) Range 18‐43	40/6	NR	NR	NR	NR	NR	NR	NR
Wu Lotus 2016	Walking training	40	54.2 (3.7); range: 39‐70	26/14	NR	NR	NR	NR	NR	NR	NR
	Conventional rehabilitation	40	53.9 (3.9); range: 37‐72	24/16	NR	NR	NR	NR	NR	NR	NR
Xiao 2003	Intensive rehabilitation	67	62.9 (1.4)	45/22	NR	NR	NR	NR	NR	NR	NR
	Conventional (no treatment)	67	65.5 (1.1)	47/20	NR	NR	NR	NR	NR	NR	NR
Xiao Yuhua 2015	Three‐stage rehabilitation	42	Not stated	NR	NR	NR	NR	NR	NR	NR	
	Routine treatment	41	Not stated	NR	NR	NR	NR	NR	NR	NR	
Xiao Zhen‐dong 2014	Balance training	30	53.42 (11.25); Range: 32‐67	23/7	NR	NR	NR	NR	NR	NR	NR
	Routine rehabilitation therapy	30	54.65 (10.76); range: 36‐70	24/6	NR	NR	NR	NR	NR	NR	NR
Xie 2003	Rehabilitation	32	Whole group 60 (8); Range: 51‐72	Whole group: 35/29	NR	NR	NR	NR	NR	NR	NR
	Control (no treatment)	32			NR	NR	NR	NR	NR	NR	NR
Xie 2005	Rehabilitation	35	67.2 (9.9)	21/14	NR	NR	NR	NR	NR	NR	NR
	Control (no treatment)	35	64.7 (9.2)	18/17	NR	NR	NR	NR	NR	NR	NR
Xu 1999	Rehabilitation	32	Mean 55 Range: 37‐69 y	24/8	NR	NR	NR	NR	NR	NR	NR
	Control (no treatment)	30	Mean 57 Range: 38‐72 y	20/10	NR	NR	NR	NR	NR	NR	NR
Xu 2003a	Rehabilitation	94	58.3 (not stated)	48/46	NR	NR	NR	NR	NR	NR	NR
	Control (no treatment)	92	55.4 (not stated)	45/47	NR	NR	NR	NR	NR	NR	NR
Xu 2003b	Rehabilitation	92	57.6 (not stated)	48/44	NR	NR	NR	NR	NR	NR	NR
	Control (no treatment)	88	56.9 (not stated)	45/43	NR	NR	NR	NR	NR	NR	NR
Xu 2004	Rehabilitation	30	59.8 (10.0)	21/9	NR	NR	NR	NR	NR	NR	NR
	Control (no treatment)	27	63.3 (8.7)	18/19	NR	NR	NR	NR	NR	NR	NR
Xu 2013	Medical exercise	58	Whole group mean: 61.5yrs Range: 43‐80	Whole group: 61/56	NR	NR	NR	NR	"no significant differences between the two groups in terms of age, sex composition ratio, educational level.." No data reported	NR	NR
	Conventional medicine therapy	59			NR	NR	NR	NR		NR	NR
Xu 2015	EMG‐triggered stimulation + comprehensive rehabilitation	20	58.0 (10.6)	9/11	NR	NR	NR	NR	NR	NR	NR
	Conventional therapy	20	58.8 (11)	13/7	NR	NR	NR	NR	NR	NR	NR
Xu 2022	Physical rehabilitation	80	61.89 (2.56)	58/22	NR	NR	NR	NR	NR	NR	NR
	No physical rehabilitation	80	62.04 (3.12)	60/20	NR	NR	NR	NR	NR	NR	NR
Xu Wenyu 2012	"inertial guided gait rehabilitation training"	50	57.8 years SD: not reported	32/18	NR	NR	NR	NR	NR	NR	NR
	"conventional rehab gait training"	50	55.4 years SD: not reported	28/22	NR	NR	NR	NR	NR	NR	NR
Xu Yumei 2013	Rehabilitation training	20	Not stated	NR	NR	NR	NR	NR	NR	NR	NR
	Conventional facilitation techniques (Bobath) and TCM	20	NR	NR	NR	NR	NR	NR	NR	NR	NR
Xue 2006	Training	78	58 (11)	44/34	NR	NR	NR	NR	NR	NR	NR
	Control (no treatment)	72	59 (10)	40/32	NR	NR	NR	NR	NR	NR	NR
Yadav 2016	Specific Balance Strategy Training Programme	12	53.83 (10.73)	10/2	NR	NR	NR	NR	NR	NR	NR
	General balance training	12	56.75 (9.58)	9/3	NR	NR	NR	NR	NR	NR	NR
Yan 2002	Rehabilitation	40	62.5 (not stated)	25/15	NR	NR	NR	NR	NR	NR	NR
	Control (no treatment)	38	60.3 (not stated)	24/14	NR	NR	NR	NR	NR	NR	NR
Yan 2015	Three‐stage rehabilitation	30	56.8 (3.1)	20/10	NR	NR	NR	NR	NR	NR	NR
	Convnetional rehabilitation training	30	57.5 (2.9	17/13	NR	NR	NR	NR	NR	NR	NR
Yang 2006	Task‐oriented progressive resistance strength training	24	56.8 (10.2) Range: 45‐74 years	16/8	NR	NR	NR	NR	NR	NR	NR
	No rehabilitation training	24	60 (10.4) Range: 50‐74 years	16/8	NR	NR	NR	NR	NR	NR	NR
Yang 2017	Bobath + electroacupuncture	29	55.85 (6.09); range: 36‐73	17/12	NR	NR	NR	NR	NR	NR	NR
	Electroacupuncture	29	55.37 (8.16); range: 35‐76	15/14	NR	NR	NR	NR	NR	NR	NR
Yang Aiguo 2015	Bobath therapy combined with acupuncture	48	54.29 (7.30)	28/20	NR	NR	NR	NR	NR	NR	NR
	Acupuncture	48	53.98 (9.92)	22/26	NR	NR	NR	NR	NR	NR	NR
	No physical therapy	48	55.17 (10.68)	25/23	NR	NR	NR	NR	NR	NR	NR
Yang Jian 2007	Rehabilitation	31	63.06 (10.19)	17/14	NR	NR	NR	NR	NR	NR	NR
	Attention control	29	65.86 (11.46)	14/15	NR	NR	NR	NR	NR	NR	NR
Yang Zhihong 2015	Three‐stage rehabilitation	37	Whole group: : 53.33 (6.15) range: 32‐78	Whole group: 41/33	NR	NR	NR	NR	NR	NR	NR
	Traditional nursing	37			NR	NR	NR	NR	NR	NR	NR
Yazici 2021	Neurodevelopmental‐Bobath approach	21	Median (IQR 25/75): 66 (59.5‐78.5)	NR	NR	NR	NR	NR	NR	NR	NR
	Standard rehabilitation	18	Median (IQR 25/75): 65.5 (58.5‐79.25)	NR	NR	NR	NR	NR	NR	NR	NR
Ye Dayong 2010	Rehabilitation	30	51.60 (9.08)	17/13	NR	NR	NR	NR	NR	NR	NR
	No physical rehabilitation	30	56.95 (11.54)	18/12	NR	NR	NR	NR	NR	NR	NR
Yelnik 2008	NDT‐based treatment	35	54.9 (11.8) range: 26.5‐77.3	22/13	NR	NR	NR	NR	NR	NR	NR
	Multisensorial	33	55.5 (11.6) range: 32.5‐78.3	22/11	NR	NR	NR	NR	NR	NR	NR
Yin 2003a	Rehabilitation	30	68 years SD = not reported	26/4	NR	NR	NR	NR	NR		
	Rehabilitation with therapy with intermediate frequency	30	65 years SD = not reported	24/6	NR	NR	NR	NR	NR	NR	NR
	Control (no treatment)	30	66 years SD = not reported	21/9	NR	NR	NR	NR	NR	NR	NR
Yue Chunjiang 2014	Exercise re‐learning rehabilitation + acupuncture	45	63.28 (6.15)	24/21	NR	NR	NR	NR	NR	NR	NR
	Routine rehabilitation training	45	61.76 (7.03)	22/23	NR	NR	NR	NR	NR	NR	NR
Yue Lin 2012	Balance training	46	51.1 (10.4)	31/15	NR	NR	NR	NR	NR	NR	NR
	Conventional rehabilitation	46	53.4 (9.83)	33/13	NR	NR	NR	NR	NR	NR	NR
Zang 2013	Three‐stage rehabilitation TCM programme	50	63.28 (7.52); range: 47‐75	33/17	NR	NR	NR	NR	NR	NR	NR
	2‐week western medicine rehabilitation (1st stage)	50	65.2 (8.25); range: 44‐75	32/18	NR	NR	NR	NR	NR	NR	NR
Zhang 1998	Early rehabilitation	29	66 years SD = not reported	NR	NR	NR	NR	NR	NR	NR	NR
	Control (no treatment)	27	63 years SD = not reported	NR	NR	NR	NR	NR	NR	NR	NR
Zhang 2004	Rehabilitation	439	61 (11)	266/173	NR	NR	NR	NR	NR	NR	NR
	Control (no treatment)	463	60 (11)	281/182	NR	NR	NR	NR	NR	NR	NR
Zhang Huiyu 2021	Goal‐oriented functional exercise	52	58.25 (3.68); range: 46‐79	29/23	NR	NR	NR	NR	Education level: 8 cases of primary school and below, 11 cases of junior high school, 19 cases of high school or technical secondary school, 14 cases of college and above	NR	NR
	Routine therapy	52	58.16 (3.75); range: 45‐79	30/22	NR	NR	NR	NR	Education level: 10 cases of primary school and below, 12 cases of junior high school, 18 cases of high school or technical secondary school, 12 cases of junior college and above	NR	NR
Zhang Jianhong 2013	Neurorehabilitation	84	63.5 (8.9); range: 34‐79	64/20	NR	NR	NR	NR	NR	NR	NR
	Self‐training without regulated rehabilitation	82	63.1 (8.2); range: 38‐78	62/20	NR	NR	NR	NR	NR	NR	NR
Zhang Lifang 2015	Balance function training	26	Whole group: 50.1 (14.2); range: 45‐65	NR	NR	NR	NR	NR	NR	NR	NR
	Routine training	26	As above	NR	NR	NR	NR	NR	NR	NR	NR
Zhao 2002	Rehabilitation nursing	100	55.2 (8.4)	58/42	NR	NR	NR	NR	NR	NR	NR
	Control (no treatment)	80	56.6 (9.2)	42/38	NR	NR	NR	NR	NR	NR	NR
Zhao 2003	Rehabilitation	150	57 years SD = not reported; range: 36‐81	91/59	NR	NR	NR	NR	NR	NR	NR
	Control (no treatment)	150	59 years SD = not reported; range: 41‐76	82/68	NR	NR	NR	NR	NR	NR	NR
Zhao Ailiang 2016	Rehabilitation + acupuncture	65	58.97 (8.27); range: 43‐87	40/25	NR	NR	NR	NR	51 cases with education level > high school 14 cases with education level < high school	NR	NR
	Acupuncture	61	59.13 (8.04); range: 42‐88	38/23	NR	NR	NR	NR	49 cases with education level > high school 12 cases with education level < high school	NR	NR
Zhao Haihong 2013	Bobath technique plus conventional treatment	30	54.7 (4.9); range: 41‐76	16/14	NR	NR	NR	NR	NR	NR	NR
	Conventional neurological treatment	30	53.2 (4.6); range: 42‐75	17/13	NR	NR	NR	NR	NR	NR	NR
Zheng 2014	Rehabilitation therapy (Bobath and motor relearning) + routine medical treatment	30	Whole group: 50.2 (10.6); range: 35‐70	Whole group: 38/22	NR	NR	NR	NR	NR	NR	NR
	Routine medical treatment (self‐rehabilitation training)	30	As above	As above	NR	NR	NR	NR	NR	NR	NR
Zhong Qiue 2014	Bilateral limb function training	32	67.3 (12.4) years; range: 41‐82	18/14	NR	NR	NR	NR	NR	NR	NR
	Unilateral (affected) limb function training	32	67.3 (12.4) years; range: 41‐82	19/13	NR	NR	NR	NR	NR	NR	NR
Zhu 2001	Rehabilitation	72	64.51 (8.87)	57/15	NR	NR	NR	NR	NR	NR	NR
	Control (no treatment)	53	66.04 (8.80)	35/17*	NR	NR	NR	NR	NR	NR	NR
Zhu 2004b	Treated	26	66 (11)	14/12	NR	NR	NR	NR	NR	NR	NR
	Controlled (no treatment)	26	65 (11)	18/8	NR	NR	NR	NR	NR	NR	NR
Zhu 2006	Test	35	61.3 (6.8)	19/16	NR	NR	NR	NR	NR	NR	NR
	Controlled (no treatment)	35	62.1 (5.9)	20/15	NR	NR	NR	NR	NR	NR	NR
Zhu 2007	Cerebral haemorrhage rehabilitation	12	61 (10)	10/2	NR	NR	NR	NR	NR	NR	NR
	Cerebral haemorrhage control	10	63 (13)	8/2	NR	NR	NR	NR	NR	NR	NR
Zhu 2016	Modified constraint‐induced movement therapy	11	59.18 (7.34); range: 40‐70	8/3	NR	NR	NR	NR	NR	NR	NR
	Conventional therapy	11	58 (6.97); range: 40‐70	8/3	NR	NR	NR	NR	NR	NR	NR
Zhuang 2012	Physiotherapy	86	64.29 (8.42); range: 42‐75	54/32	NR	NR	NR	NR	NR	NR	NR
	Acupuncture	91	63.87 (9.23); range: 42‐75	61/30	NR	NR	NR	NR	NR	NR	NR
	Combination therapy	97	64.03 (9.19); range: 40‐75	63/34	NR	NR	NR	NR	NR	NR	NR

Abbreviations: IQR: interquartile range; NR: not reported; SD: standard deviation Additional notes: ^1^ unless otherwise specified; *mismatch in the gender data reported in the paper compared to group data reported elsewhere

The time since stroke was:

- 7 days or less for 28/267 studies;
- 14 days or less for 23/267 studies;
- 14 days to 6 weeks for 34/267 studies;
- 6 weeks to 6 months for 36/267 studies;
- more than 6 months for 15/267 studies;
- wide‐ranging for 52/267 studies;
- unclear or not reported for 79/267 studies.

#### Interventions

Intervention details are provided in Characteristics of included studies. Intervention dose (duration, frequency, and session length) and intervention provider are provided in Table.

**11:** Length, dose and provider ‐ all trials

**Study**	**Intervention duration**	**Session frequency**	**Session length**	**Provider**
ACTIV 2021	6 months	Face‐to‐face (remote): 4 session (baseline and 2, 12, 24 weeks) Phone calls: 5 (1, 4, 8, 16, 20 weeks) Text messages: Weeks 1‐10: 2/week Weeks 11‐26: 1/week	Face‐to‐face: 45 minutes Phone calls: 20 minutes	Physical therapists who had completed ACTIV training
Ain 2022	Not stated	Not stated	Not stated	Not stated
Aksu 2001	Not stated	Not stated	Not stated	Not stated
Alabdulwahab 2015	4 weeks	FLO group: 3/week LORT group: Unclear	FLO group: 60 minutes LORT group: Unclear ‐ included 5–10 minutes warm‐up and 3 sets of 10–15 reps max	Therapist
Allison 2007	14 ‐ 28 days (length of hospital stay)	1/day on working days during hospital stay. After discharge, frequency dependent on community assessment ‐ generally 1‐2/week.	Intervention group: 45 minutes standing practice Both groups: 45 minutes conventional PT	Physiotherapists (conventional PT) and physiotherapy assistants (standing practice)
Aloraini 2022	2 weeks	5 days/week	180 minutes	Therapist
Anandan 2020	10 weeks	Not stated	60 minutes	Not stated
Arabzadeh 2018	4 weeks	Not stated	50 minutes	Physiotherapist
Aravind 2022	12 weeks	2/week	60 minutes	Fitness instructors with TIME^TM^ training
Aries 2021	6 weeks	Total 20 sessions	30–60 minutes (MTS)	Research therapists
Arya 2019	8 weeks	3/week	60 minutes	Therapist
Baer 2007	4 weeks	Total of 3 sessions over 4 weeks	Not stated	Research physiotherapist
Bai 2008	6 months	Level 1: 1/day, 5/week Level 2 and 3: 2/day 5/week	Level 1: 45 minutes Level 2 and 3: 30 minutes	Therapist, family members, nurses
Bai 2013	4 weeks	6/week	All groups 30‐75 mins: Physiotherapy 45 minutes Acupuncture 30 minutes	Acupuncturists, physicians, and therapists
Bai 2014	6 months	Levels 1 and 2: 2/day, 5 days/week Level 3: 1/day (relatives/carers) Visits from medical staff every 2 weeks	Levels 1 and 2: 45 minutes Level 3: "most of waking hours"	Experienced therapists, rehabilitation physicians, relatives/caregivers
Bale 2008	4 weeks	5 days/week	50 minutes	Physiotherapists
Batchelor 2012	12 months	3–5/week (HEP + walk)	30–40‐minutes (HEP + walk)	Home ex (self) prescribed by physiotherapist
Behrman 2011	12 ‐ 16 weeks	3 days/week	90 minutes	Physical therapists
Bek 2016	10 weeks	1/week	90 minutes	Conductors (had 3 years practical and theoretical training for degree in Conductive Education)
Bhatia 2014	4 weeks	3 days/week	45 minutes	Not stated
Blennerhassett 2004	4 weeks	5 days/week	60 minutes	Physiotherapists and physiotherapy department staff members
Bordoloi 2020	3 months	Not stated	Not stated, but in addition to HEP programme (6 days a week)	consultant physiotherapist, patients, caretakers
Brock 2005	2 weeks	Total of 6 sessions over 2 weeks	60 minutes	Physiotherapists with ≥ 5 years postgraduate experience + ≥ 2 years rehabilitation/neurology experience. Bobath group therapists also had to have completed a Basic Bobath Course + ≥ 2 Advanced Bobath Courses (min of 180 hours of formal training)
Brouwer 2018	6 months	Tune‐up 1 (6 months post‐discharge): 3/week for 2 weeks Tune‐up 2 (12 months post‐discharge): 3/week for 2 weeks	60 minutes	Physiotherapists
Bui 2019	6 weeks	Acupuncture: 5 days/week Exercises: not explicitly stated	Not stated	Not stated
Candan 2017	6 weeks	5/week	120 minutes	Physiotherapist
Cao 2014	4 weeks	2/day	45 minutes	Rehabilitation therapist, family
Capisizu 2016	1 month	Not stated	Not stated	Not stated
Carlson 2006	2 weeks	1/day	180 minutes	Not stated
Chae 2017	4 weeks	5/week	Proprioceptive training group: 30 minutes Control group: 60 minutes	Physical therapists with more than 3 years experience
Chan DY 2006	6 weeks	3 days/week	120 minutes	Occupational therapists
Chan WN 2017	12 weeks	Tai Chi and Conventional Exercise Groups: 2/week Control: No training	60 minutes	Physiotherapist who is also an experienced Tai Chi practitioner
Chang 2015	6 months	Three‐stage rehabilitation: Stage 1 ‐ 2 sessions/day; 5 days per week; Stage 2 ‐ 3 sessions/day; 6 days/week Stage 3: 1 session/week Control: No training	Three‐stage rehabilitation: 40 minutes in stage 1 and 2; stage 3: not stated	Rehabilitation instructor; nurses and family members (stage 3)
Chen L 2019	4 weeks	5/week	40 minutes	Physiotherapist
Chen S 2021	12 months	Supervised sessions: Month 1‐3: 3/week Month 4‐6: 1/week Month 7‐12: 1/month Month 10‐12: 1/2 month	30 minutes	Advanced practice registered nurse, family members, patient
Chen G 2014	6 months	Task function training: Not stated Strength training: 1/day, 5/week	Task function training: Not stated Strength training: 40 minutes	Rehabilitation therapist
Chen J 2014	6 months As both groups receive Level 1 care, intervention is 4.75 months	Level 1 (hospital) (1‐1.5 months) 1‐2/day Level 2 (community rehabilitation centre) ≥ 2/day Level 3 (home) 1/day	Level 1 Not stated Level 2 and 3 40 minutes	Rehabilitation physicians, family members
Chen P 2014	4 weeks	1/day, 5.5 days/week	40 minutes	Not stated
Chen Y 2011	5 months	Brunnstrom stages: Stage I‐II: 4 sessions/day; Stage III‐IV: 4 sessions/day; Stage V‐VI: 4‐6 sessions/day Control: No training	Brunnstrom stages: Stage I‐II: 30 minutes Stage III‐IV: 15 minutes Stage V‐VI: 20 minutes	Therapist
Cheng 2021	6 months	Level 1 Not stated Level 2 1 ‐2/week Level 3 1/ 2 weeks	Not stated	Rehabilitation therapists, patients’ families
Choi YK 2013	4 weeks	PNF group: 3/week Control (neurodevelopmental treatment): Not stated	PNF group: 30 minutes Control (neurodevelopmental treatment): Not stated	Therapist
Choi JU 2015	4 weeks	5/week	30 minutes	Therapist
Chu 2003	20 days ‐ 14 months (mean 41.3 days)	Daily	40‐60 minutes	Nurses
Cooke 2006	6 weeks	4/week	60 minutes	Research physiotherapists
Dai 2015	6 months	Not stated	Not stated	Unclear
Dalal 2018	6 Days	1/day	Routine physiotherapy (both groups) 45–60 minutes including rest periods Prowling and proprioceptive training: 15–20 minutes	Not stated
Danlami 2017	4 weeks	sCIMT and tCIMT: 2/day, 5/week	sCIMT Not stated ‐ target was to complete a set number of repetitions tCIMT: 60 minutes	Physiotherapist, patients and relatives
Dean 2000	4 weeks	3 days/week	60 minutes	Physiotherapists
Dean 2006	12 months	1/week for 40 weeks of the year + home exercise program (3/week)	45‐60 minutes	Physiotherapists
Deng 2011	6 weeks	2/week	60 minutes	Therapists and rehabilitation nurses.
Ding 2015	3 months	Not stated	Not stated	Clinical nurses
DOSE 2020	4 weeks	Control and Dose 1/day, 5 days/week DOSE2: 2/day, 5 days/week	Control: 60 minutes	Physical therapists trained to deliver the DOSE intervention
Du 2014	1 month	3/day	30 minutes	Rehabilitation specialists, family members
Duan 2011	4 weeks	Not stated	Not stated	Therapist
Duncan 1998	8 weeks supervised 4 weeks patient only	3/week	90 minutes	Physical Therapist and Occupational Therapist
Duncan 2003	12 ‐ 14 weeks	Total 36 sessions	90 minutes	Physical or Occupational Therapist
Epple 2020	7 days	1/day	40 minutes	Physiotherapists who had received special training and certification from the International Vojta Society
Fan WK 2006	6 months	Level 1: 1/day, 5/week Level 2: 2/day 5/week Level 3: 2/day, 5–7/week	Level 1: 45 minutes Level 2 and 3: 30–45 minutes	Therapist, patient’s family, nursing workers outside therapy times
Fan Y 2015	Not stated	Not stated	Not stated	Not stated
Fan L 2014	8 weeks	Variable depending on status 1‐3/day	10‐30 min	Nurse, family members
Fan WS 2006	40 days	Both groups: 1 session/day	30‐45 minutes	Not stated
Fan X 2009	4 weeks	Exercise training: one session/day; 6/week Conventional rehabilitation: not stated	30 minutes	Not stated (abstract only)
Fang 2003	4 weeks	5/week	45 minutes	Rehabilitation therapists
Fang YN 2004	3 days	Daily	45 minutes	Not stated
Fang H 2010	8 weeks	2/day; 6 days/week	30 minutes	Rehabilitation therapists
FeSTivaLS 2014	6 weeks	4 days/week	60 minutes	Research therapist
Frimpong 2014	8 weeks	3/week	Circuit training; 35 minutes Control: Not stated	Not stated
Ge W 2003	Not stated	1/day	30‐45 minutes	Not stated
Ge Y 2020	2 months	Both groups: 2/day, 5 days/week	120 minutes	Nursing staff
Gelber 1995	25 days on average	Not stated	Not stated	Physical and occupational therapists trained in the therapy techniques being used. Patient practice of techniques reinforced by nursing staff.
Ghasemi 2018	8 weeks	3/week	Not stated	Trained physiotherapist
Gong Y 2009	5 months of treatment	Not stated	Not stated	Rehabilitation therapist
Green 2002	Maximum 13 weeks	Minimum 3 contacts	Median number of treatments per patient was 3 (IQR 2–7, range 0–22) and the mean duration of every treatment was 44 min (SD 21, range 10–90)	Physiotherapists
Guan 2017	2 weeks	3/day, 5/week (1/d with therapist + 2/day with family)	30‐45 minutes (45 mins therapist, 30 mins family)	Rehabilitation therapist, family members
Guo L 2012	6 months	Daily practice, follow‐up hospital visits every 2‐4 weeks	30‐60 minutes	Rehabilitation therapist, family members
Guo L 2013	6 weeks	2/day, 5 days/week	40‐60 minutes	Therapist
Guo Z 2015	6 months	2/day	30 minutes	Specialised personnel
Haral 2014	4 weeks	3/week	60 minutes	Not stated
Harjpal 2021	6 weeks	5 days/week	20 minutes (non‐involved side) in addition to 20 minutes (involved side)	Physiotherapist
Hendrey 2018	6 weeks	3/week	45 minutes (including rest breaks)	Physiotherapists with 5‐10 years stroke rehabilitation experience and trained to deliver the standardised intervention protocol
Holmgren 2006	5 weeks	6/week (+ 3/week unsupervised HEP until 3 months follow‐up)	45 minutes	Physiotherapist, occupational therapist (education sessions)
Hong Cuicui 2016	6 weeks	Both groups: 2 sessions/day, 5 days/week	45 minutes	Professionally trained therapists; family members supported additional training outside of the treatment delivered in the 'Additional therapy' group
Hong Hye Jin 2012	Not stated	Depended on stage of rehabilitation; stage 1: 2‐4 sessions/day; stage 2: 1‐2 sessions/day 4‐5 days/week	30‐45 minutes depending on stage of rehabilitation	Neurologist, nurse and therapists
Hoseinabadi 2013	4 weeks	3/week	60 minutes	Not stated
Hou 2006	6 months	1‐2 times/day, 5/week; increasing to 2/day, 5‐6/week	30‐40 minutes	Therapists. Outwith therapy time, patient’s family members and nurses assisted patients with rehabilitative training
Hou Zhi 2014	6 months	Level 1 1/day Level 2 2/day Level 3 (home) 1/day	Level 1 60 mins Level 2 45 minutes Level 3 Not stated	Nursing staff, family members
Howe 2005	4 weeks	12 sessions over 4 weeks	Total of 6 hours (30 minutes average/session)	Physiotherapy assistants
Hu 2007	6 months	Not stated	Not stated	Not stated
Huang 2003	30 days	Daily	45 minutes	Therapist
Huang 2014	Different for each group: 8.23 ‐ 12.94 days	1/day	40 minutes	Therapist
Huang Yangfang 2016	Three levels ‐ rehab up to 6 months	Stage I: 1/day; 5 days/week Stage II: 2/day; 5 days/week Stage III: 2/day; 5‐7 days/week	Stage I: 40‐45 minutes; Stage II: 35‐45 minutes Stage III: 30‐45 minutes	Not clearly stated; family members are involved
Hui‐Chan 2009	4 weeks	5 days/week	Task‐related training: 60 minutes Placebo‐TENS: 60 minutes	Physiotherapists
Imhof 2015	30 days	Not stated	Not stated	Registered nurses with training in kinaesthetic principles.
Indurkar 2013	3 weeks	6 days/week	Task oriented group: 30 minutes task oriented activities + Both groups: 60 mins physiotherapy	Therapist
Jandaghi 2021	1 month	3/week	30 minutes	Train[ed] researcher
Jeon 2018	4 weeks	5/week	60 minutes	Not stated
Ji Pei 2014	1 month	1/day, 5 days/week	60 minutes	Not stated
Jing 2006	7 weeks (on average)	1/day	Intervention group: 40‐50 minutes Control: 45‐60 minutes	Exercise therapist (intervention group), occupational therapist (control)
Jongbloed 1989	8 weeks	1/day, 5 days/week	40 minutes	Occupational therapists who had attended a workshop on the treatment technique (sensorimotor integrative or functional training) and had practised using these techniques treated subjects assigned to the relevant treatment group
Khallaf 2014	8 weeks	5/week	Study group: 90 minutes Control: 50 minutes	Neurophysiotherapists
Kim 2007	6 weeks	1/day, 5 days/week	30‐50 minutes Circuit: 50 minutes three time/week Conventional: 30 minutes twice/week	Physical therapists
Kim 2012	4 weeks	Task‐oriented training (intervention group): 3/week General PT (both groups): 5/week	All sessions: 60 minutes	Task‐oriented training supervised by physical or occupational therapists. Conventional therapy provider not stated.
Kim 2012a	5 weeks	RAS (RAS Group only): 3/week Conventional therapy (both groups) 2/day, 5/week	30 minutes	Therapist
Kim 2014	6 weeks	1/day, 5 days/week	50 minutes + 30 mins morning bed exercise	Motor ex: not stated Morning ex: video‐based led by trainer
Kim 2016	4 weeks	5 days/week	Study group: 90 minutes Control: 60 minutes	Physiotherapist
Kim 2017	4 weeks	5/week	Intervention group: 50 minutes task‐oriented circuit training Both groups: daily neuro‐developmental treatment 60 minutes	Physical therapists (with 3 years of experience in stroke rehabilitation).
Kim 2018	4 weeks	3/week	30 minutes	Physical therapist with 2 or more years clinical experience
Kim 2021	8 weeks	5/week	30 minutes	Physical therapists with more than 5 years of clinical experience
Knox 2018	12 weeks	Strength and Task groups: 6 sessions in total across 12 weeks Control: 1 educational session including 20 minutes exercise	60 minutes	First author and a physiotherapist, experienced in neurological rehabilitation, after training with the first author
Koç 2015	12 weeks	2/week (supervised)	60 minutes	Nurse researcher, patient (HEP)
Krawczyk 2014	12 weeks	1/day	120 minutes	Physiotherapist
Krukowska 2016	6 weeks	6 days/week	35 minutes	Professional physiotherapists
Kuberan 2017	3 weeks	5 days/week	45‐60 minutes	Tester
Kumaran 2015	12 weeks	3/week	60 minutes	Therapists
Kunkel 2013	2 weeks	4/week	60 minutes	Therapist
Kwakkel 2002	20 weeks	5 days/week	30 minutes according to group assignment + 15 minutes upper limb exercises + 15 minutes lower limb exercise	Physical and occupational therapists
Kwakkel 2008	12 weeks	2 days/week	90 minutes	Physiotherapist and sports therapists
Langhammer 2000	Not stated	5 days/week	40 minutes (minimum) while in hospital	Physiotherapists
Langhammer 2007	12 months	Intermittent and variable: 1‐3/day in 4 separate period over year	20 hours every 3rd month	Physiotherapist
LAST 2018	18 months	in addition to standard care: Monthly coaching meeting Exercise (including 2 to 3 periods vigorous activity): 1/week Physical activity: 7 days/week	Exercise: 45 ‐ 60 minutes Physical activity: 30 minutes	Physiotherapist (coaching); weekly exercise provided by outpatient, private, and community‐based treatment staff
Lawal 2016	8 weeks	3/week	60 minutes 2 other groups not included in the analysis carried out training for 90 mins and 180 mins	Trained physiotherapists
Lee 2015	16 weeks	Exercise intervention group: 3/week	Exercise intervention group: 60 minutes	Trained exercise rehabilitation specialist and physical therapist
Lee 2018	4 weeks	1/day, 5 days/week	120 minutes CME: 60 minutes Conventional PT: 60 minutes	Physical therapist, physician (supervising), caregiver
Lennon 2006	8 – 35 weeks (average 17.4 weeks)	From 2‐3/week to daily (dependent on patient)	Not stated	Senior physiotherapists who had attended a 3‐week Bobath course and advanced courses
Letombe 2010	4 weeks	1/day, 4 days/week + Conventional	40‐60 minutes + conventional	Therapists (physiotherapy, occupational therapy and neuropsychological/speech therapy)
Li 1999	1 month	2/day	30 minutes	Not stated
Li 2005	Not stated	Neurodevelopmental therapy: 1/day	Neurodevelopmental therapy: 45 minutes	Not stated
Li 2013	4 weeks	2/day, 5 days/week	30 minutes	Nurse, therapist
Li Jingqian 2013	1 month	1/day	45 minutes	Not stated
Li Weiwei 2015	Not stated	2/day, 5 days/week	30 minutes	Nurse
Li Xiaojun 2016	8 weeks	1‐2 sessions/day; 5 days/week	30‐45 minutes	Therapist
Li Yuanzheng 2014	2 months	"qd"	45 minutes	Not stated
Li Yuanzheng 2014a	4 weeks	2/day, 5 days/week	30 minutes	Therapist
Lincoln 2003	"as long as was needed"	5 days/week	Variable ‐ median 23 minutes (IQR 13–32 minutes) per weekday	Physiotherapists and physiotherapy assistants
Lindvall 2014	8 weeks	1/week	60 minutes	Physiotherapists with further training and 15–20 years of clinical experience in body awareness therapy
Liu 2014	4 weeks	1/day	Not stated	Therapist
Liu Xuan 2016	6 months	Tailored training depending on participant ability	Tailored training depending on participant ability	Not stated
Liu Yanhua 2020	6 weeks	Exp group: Not stated directly. At least 1/day Control: 1/day	Exp group: Not stated directly. At least 50 minutes. Control: 120 minutes	Medical staff
Lu 2004	Not stated	Level 1: 1/day Level 2 and 3: Not stated	Level 1: 45 minutes Level 2 and 3: Not stated	Level 1 and 2: Not stated Level 3: rehabilitation therapist
Lu 2014	6 months	Level 1: 1/day Level 2: 2/day Level 3: Not clearly stated	Level 1: 60 minutes Level 2 and 3: 30‐60 minutes	Nursing staff, family members, therapist
Lu Liangyan 2014	6 months	Level 1: Not stated Level 2: 2/day, 5 ‐7 days/week Level 3: 2/day, 6/week	Level 2 and 3: 40 minutes	Therapist, nurse, family members
Ma Xue 2010	8 weeks	Not stated	Not stated	Members of the rehabilitation team, doctor, stroke survivors family
Mai Guanghuai 2016	6 weeks	Conventional rehabilitation: 1‐2 sessions/day; 6 days/week Lower limb training: delivered in addition to conventional rehabilitation ‐ 6 days/week	20‐40 minutes	Not stated
Mansfield 2018	6 weeks excluding the 2 booster sessions	Weeks 1‐6 2/week 3 and 9 months post‐intervention One 1‐hour session	Weeks 1‐6 60 minutes	Physiotherapists trained in delivering the relevant intervention
Marigold 2005	10 weeks	3/week	60 minutes	Physical therapist, kinesiologist, and recreation therapist
Martins 2020	12 weeks	1/week	60 minutes	Physiotherapist with > 7 years clinical and research experience in neurological rehabilitation. Research assistants trained to assist the participants when necessary.
Matthew Hall 2013	3 months	1/day, 6/week	45 minutes	Therapist
McClellan 2004	6 weeks	2/day	Total of 30 hours (2 hours supervised)	Physiotherapists
Medina‐Rincón 2019	4 weeks	5/week	60 minutes	Physiotherapists experts in neurorehabilitation and received 2 days training in the application of the intervention programme
Meier 2021	2 weeks	Total of 10 sessions in 2 weeks	45 minutes	Physical therapist
Mendoza 2015	4 weeks	3/week	60 minutes	Trained physical therapists with clinical experience ranging from 1‐10 years, assisted by physical therapy students who were trained in the conduct of the treatment protocol.
Meng 2022	4 weeks	3 days/week	45 minutes	Nationally accredited therapist with > 5 years of experience
Meng Fanda 2021	Not stated	1‐2/day	30 minutes	Not stated
Meng Qingling 2015	3 months	Not stated	Not stated	Not stated
Mikolajewska 2017	2 weeks	10 sessions in total	Not stated	A physical therapist with > 15 years of experience in neurorehabilitation, and NDT‐Bobath skills confirmed by basic and advanced courses in NDT‐Bobath for adults, and NDT‐Bobath for children and baby courses.
Mohaideen 2014	3 months	5 days/week	Not stated	Physiotherapist
Moore 2016	19 weeks	3/week	45‐60 minutes	Fitness instructor, physiotherapist
Morreale 2016	3 months	Day 1‐4: 1/day Day 5 onwards: 5 days/week	Day 1‐4 (Early groups only): 60 minutes Day 5‐60: 135 minutes After discharge: 90 minutes	Physical therapists specifically trained for treatment protocols.
Mudge 2009	4 weeks	8 sessions over 4 weeks	90 minutes	Physiotherapist and physiotherapy students
Mustafaoğlu 2018	6 weeks	5/week	45 minutes	Physiotherapist
Nagy 2017	~ 4 weeks (18 consecutive week days)	CPT: 3/day CE: 1 individual and 1 group session in addition to above	CPT: 20 minutes CE: 45 minutes (individual) 30 minutes (group) in addition to above	Not stated
Ni 1997	Average of 2 months	2/day	30‐45 minutes	Not stated
Nindorera 2022	12 weeks	3 days/week	120 minutes	Not stated
Outermans 2010	4 weeks	3/week	45 minutes	Therapist
Pan 2004	3‐4 weeks	3‐4/day	30 minutes	Therapist and "family of participants were instructed to assist in practice"
Pandian 2014	8 weeks	3/week	60 minutes	Therapists
Pang 2003	Not stated	1) Super‐early stage care: Not stated 2) Flaccid paralysis stage care: 2‐3/day 3) Early rehabilitation care: 1/day	1) Not stated 2) 30 minutes 3) 30‐60 minutes	Not stated
Pang 2005	19 weeks	3/week	60 minutes	Physical therapist, occupational therapist and an exercise instructor
Pang 2006	10 sessions	5/week	30 minutes	Not stated
Pang 2018	8 weeks	3/week	60 minutes	Instructors with physical therapy background
Park 2021	4 weeks	2/day, 5 days/week Control group: All general neurological physiotherapy Intervention Groups: 3 sessions/week replaced with cross‐training instead of general neurological physiotherapy (max 1/day)	30 minutes	Therapists with at least 120 hours of professional treatment training using identical methods, provided by the researchers
Pirayesh 2021	8 weeks	3/week	30 minutes increasing to 45 minutes by the last week	Researcher
Puckree 2014	6 months	1/2 weeks	30 minutes (with break every 10 minutes)	Therapists (and assistants for the control intervention)
Qian 2004	Not stated	1/day	Neuromuscular facilitation techniques: 20 minutes Limb exercises: 20 minutes Electrical stimulation :20 minutes + ADL practice + acupuncture	Therapist
Qin 2013	3 months	3/week	Not stated	Therapist
Qin JianJian 2014	4 weeks	1/day	45 minutes	Not stated
Rahayu 2020	7 days	1/day	60 minutes	Experimental intervention provided by a research assistant trained by the principal investigator for 1 week on the protocol until they could conduct the practical session with healthy subjects to a satisfactory level by the observation of the investigator. Control carried out by the institutions’ physiotherapists.
Renner 2016	6 weeks	5/week	90 minutes	Physiotherapist or sports therapists trained in a 1‐day course before the study started
ReTrain 2018	6 months	Home visit (1:1) (2 sessions) Groups sessions 2/week (20 sessions) for 10 weeks Drop‐in visits 1/month 2 hour for 3 months	Home visit 30‐60 minutes Group sessions and drop‐in visits 120 minutes	Personal trainers on the UK Register of Exercise Professionals (≥ level 3) ARNI‐trained and accredited and have had additional training in the delivery of ReTrain
Richards 1993	6 weeks	1/day		Physiotherapists
Salbach 2004	6 weeks	3/week	60 minutes	Physical or occupational therapist
Sekhar 2013	6 weeks	Not stated	Not stated	Not stated
Seo 2015	4 weeks	3/week	30 minutes	A physical therapist with clinical experience > 1 year conducted the muscle strengthening exercise, range of motion exercise, and stretching exercise, for all subjects
Severinsen 2014	12 weeks	3/week	Not stated	Physiotherapist
Shin 2011	4 weeks	5/week	60 minutes	Physical therapists. Caregiver/family member assisted therapist with aerobic exercise
Shuai 2013	8 weeks	1/day, 5 days/week	45‐60 minutes	Not stated
Signal 2014	4 weeks	Intervention groups: 3/week	Intervention groups: 60 minutes	NZ registered physiotherapists ≥ 5 years post‐graduate with experience in stroke and older adults' rehabilitation. Therapy assistants recruited from 3rd year undergraduate physiotherapy degree at Auckland University of Technology
Song 2015	4 weeks	All groups: 1/day, 5 days/week conventional therapy ITCT and CTCT: 3/week	All groups: 30 minutes conventional therapy ITCT and CTCT: 30 minutes circuit training	ITCT and CTCT: physiotherapists with > 10 years of physiotherapy experience
SPIRES 2022	3 weeks	1/day, 5‐7 days/week conventional therapy	45 minutes	Physiotherapists
Stephenson 2004	4 weeks	Groups 1 and 2: 3/week	Groups 1 and 2: 20 minutes	Not stated
Stuart 2019	6 months	3/week	60 minutes	Lay exercise instructors trained in the exercise protocols and monitored regularly during classes. Fitness certification from an accredited certifying agency such as the Athletics and Fitness Association of America (AFAA) or American Council on Exercise (ACE)
Sun Juanjuan 2014	Not stated	Not stated	Not stated	Not stated
SunRISe 2021	8 weeks (4 weeks home‐based supervision; 4 weeks tele‐supervised)	3 days/week	70 minutes	Physiotherapist
Tang 2009	8 weeks	1/day	45 minutes	Therapist
Tang Yao 2015	Not clearly stated but training was progressive: up to 3 months	One session/day	Stage I‐II: 45 minutes Stage III: 30‐45 minutes	Nurses, therapist
Teixeira‐Salmela 1999	10 weeks	3 days/week	60‐90 minutes	Exercise physiologist and a physiotherapist
Thaut 2007	3 weeks	5/week	30 minutes	Gait therapists
Torres‐Arreola 2009	Not stated, but started at the hospital and continued up to 4 months post‐discharge	Intensive phase (in hospital + 2 weeks post‐discharge): 1/day Intermediate phase (post‐discharge week 3 and 4): in‐home visits 2/week Support phase: in‐home visits 1/week	90 minutes	Two general nurses who had received 160 hours training for the intervention strategy used
Tyson 2015	4 weeks	1/day	30 minutes (unsupervised)	The trial therapist and local clinician (taught by trial therapist)
Vahlberg 2017	3 months	2/week	55 minute exercise 20‐minute motivational session	Physiotherapist and assistant
Verma 2011	2 weeks	7 days/week	Experimental group: 15‐25 minutes motor imagery (MI) 25 minutes Task‐Oriented Circuit Class Training (TOCCT) Control group: matched duration	Physiotherapist and occupational therapists
Wade 1992	Mean visits = 4 (range 1‐11); 73% patients were seen 1 to 6 times	Not stated	Ranged from 1 hour 10 minutes to 3 hours 10 minutes (mean = 2 hours 4 minutes)	Physiotherapist and occupational therapist
Wan Xueli 2014	60 days	2/day	30‐60 minutes	Staff + family members
Wang 2004a	30 days	1‐2/day	45 minutes	Therapist, family members also taught
Wang 2004b	4 weeks	5/week	30‐45 minutes	Doctor, nurse
Wang 2005	4 weeks	5/week	40 minutes	2 physical therapists qualified ≥ 10 years with ≥ 5 years Bobath practice and who had attended the Bobath course on adult hemiplegia
Wang 2013	1 month	2/day	30 minutes	Rehabilitation doctor
Wang 2015	12 weeks	1/week supervised 2/week caregiver	90 minutes (supervised) 60‐ to 90‐minute (with caregiver)	Physical therapist
Wang 2021	7 days	Experimental group: 2/day for 7 days Control group: Not stated	Experimental group: 30 minutes Control group: Not stated	Experimental group: nurse Control group: therapist
Wang 2022	7 days	2/day, 7 days/week	30 minutes	Trained and qualified nurses
Wang Dongya 2015	Not stated	Levels 1 and 2 Not stated Level 3 1/week ‐ 1/2 weeks (> 3 months)	Levels 1 and 2 Not stated Level 3 2 hours	Nurses
Wang Leilei 2020	8 weeks	Two sessions/day; 6/7 days/week	40 minutes/session	Not stated
Wang Wenwei 2012	4 weeks	Two sessions/day; 5/week	30 minutes/session	Experienced physical therapists
Wei 2014	6 months	Not stated	Not stated	Rehabilitation physicians and therapists
Werner 1996	12 weeks	4/week	60 minutes physical therapy 60 minutes occupational therapy	One registered physical and occupational therapist
Wu 2006	6 months	Daily	Not stated	Not stated
Wu 2020	12 weeks	2/week remote rehabilitation	Variable according to status	Rehabilitation therapists, family caregivers
Wu Jiaming 2006	Not stated	5 times/week	40‐60 minutes	Therapist
Wu Jing 2015	6 months	Level 1 1/day Level 2 2 /day (2‐3 months after onset) Level 3 2 /day (Home/community‐based 4‐6 months after onset) supervised 1/15 days	40‐45 minutes	Nurses, doctors, families
Wu Lotus 2016	4 weeks	Two sessions/day	Sessions progressively longer: ranging from 10‐15 mins increasing to 45 minutes per session	Not stated
Xiao 2003	Up to 2 weeks	3/week	30 minutes	Not stated
Xiao Yuhua 2015	6 months	Not stated	Not stated	Not stated
Xiao Zhen‐dong 2014	1 month	3/week	30 minutes	Not stated
Xie 2003	30 days	Massage 5‐6/day; ADL 2/day	Massage 15‐20 minutes; ADL 30 minutes	Rehabilitation therapist
Xie 2005	6 months	Level 1: 1/day Level 2: 2‐3/week Level 3: 2/week	Level 1 and 2: 45 minutes Level 3: Not stated	Therapists, family members
Xu 1999	1 month	2/day	60 minutes	Not stated but 'with participation from family'
Xu 2003a	21 days	1/day	Not stated	Not stated
Xu 2003b	4 weeks	Daily	60 minutes	Not stated
Xu 2004	1 month	5/week	40‐50 minutes	Not stated but 'on commencement of intervention, participants were taught appropriate positioning and family members were taught basic exercises so they could supervise participants'
Xu 2013	1 month	1/day	Unclear	Medical staff, family members, patients
Xu 2015	Not stated	5/week	40 minutes	Not stated
Xu 2022	4 weeks	1/day, 4‐6/week	30‐45 minutes	Therapist
Xu Wenyu 2012	2 months	2/day (once in the morning and once in the afternoon)	45 minutes	Therapist
Xu Yumei 2013	12 weeks	2/day, 6 days/week	45 minutes	Uniformly trained therapists
Xue 2006	1 month	3/day	30 minutes	Not stated
Yadav 2016	2 weeks	5 days/week	60 minutes	Not stated
Yan 2002	38 days	Dependent on phase of recovery: Early phase: 2/day; Rehabilitative treatment (on bed): 2/day, increasing to 3‐4/day if participants had no discomfort; Rehabilitative treatment (after leaving bed): 2/day	Dependent on phase of recovery: Early phase: 15min/session; Rehabilitative treatment (on bed): 30 min/session; Rehabilitative treatment (after leaving bed): 60 minutes	Not stated
Yan 2015	6 months	Experimental group: Level 1: 1/day, 5 days/week Level 2: 2/day, 5 days/week Level 3: Not stated Control group: 3/week	Experimental group: Levels 1 and 2: 40 minutes Control group: 20‐40 minutes	Experimental group: Levels 2 and 3: Therapist and family members Level 1 and Control group: Not stated
Yang 2006	4 weeks	3/week	30 minutes	Qualified and experienced physical therapist
Yang 2017	Not stated	Bobath: one session/day Electroacupuncture: one session/day for total of 12 sessions	Bobath: 45 minutes Electroacupuncture: 20 minutes	Not stated
Yang Aiguo 2015	33 days	2 x 15‐day course of daily treatment, with interval of 3 days	45 minutes	Therapist
Yang Jian 2007	8 weeks	Not stated	Not stated	Therapist
Yang Zhihong 2015	6 months	Level 1: Multiple times a day Level 2: 3‐4/day Level 3: 1/week	Level 2: 30 minutes Level 1 and 3: Not stated	Nursing staff, medical staff and family members
Yazici 2021	Not stated	5 days/week	60 minutes	Experienced Neurodevelopmental‐Bobath approach physiotherapist. (Standard rehabilitation sessions were performed by clinical physiotherapists).
Ye Dayong 2010	Up to 6 months following stroke	Stage I‐II: 1 session/day; 5 days/week Stage III: 2 sessions/day; 5 days/week Outpatient clinic: once/2 weeks	Stage I‐II: 45 minutes; Stage III: 30‐45 minutes	Nurses, rehabilitation therapists (trained full‐time rehabilitation instructors from the Disabled Persons’ Federation)
Yelnik 2008	4 weeks	5 days/week	60‐70 minutes	Physical therapists
Yin 2003a	Not stated	1/day	40 minutes	Not stated
Yue Chunjiang 2014	6 months	1/day	45 minutes	Not stated
Yue Lin 2012	8 weeks	2/day, 11 times/week	30 minutes	Not stated
Zang 2013	12 weeks	Level 1 (onset‐day 14) 1/day, 5/week Level 2 (day 15‐28) and Level 3 (day 29‐90) 2/day, 5/week	Level 1 45 minutes Levels 2 and 3 30‐45 minutes	Physicians, nurses, family members
Zhang 1998	Not stated	Daily	60 minutes	Not stated but does mention that "therapy was conducted… with the help of the patients family"
Zhang 2004	6 months	Not stated	Not stated	Physiotherapists, family members
Zhang Huiyu 2021	6 months	Unclear ≥ 2/day	Unclear	Not stated
Zhang Jianhong 2013	5 weeks	2 ‐3 /day, 7 days/week	~ 30 minutes (total 20 hours)	Not stated
Zhang Lifang 2015	12 weeks	5/week	30‐40 minutes	Therapist
Zhao 2002	Mean 31.6 days (SD 11.2 days)	5/week	30‐45 minutes	Nurse, family members
Zhao 2003	PT and OT: ‘10 days as a treatment course, persisting 2 courses'	Daily	40 minutes physiotherapy 30‐40 minutes occupational therapy	Not stated
Zhao Ailiang 2016	Not stated	2‐ 4 sessions/day	20‐30 minutes	Therapist, doctor, family members
Zhao Haihong 2013	Not stated	1/day, 7 days/week	Not stated	Nursing staff
Zheng 2014	30 days	1/day, 5‐6/week	45 minutes	Professional rehabilitation physicians
Zhong Qiue 2014	1 month	1/day	45‐60 minutes in addition to on exercise therapy performed by both groups	Nurse
Zhu 2001	3 months	5/week	45 minutes (plus 20 minutes electrotherapy)	Staff, family members
Zhu 2004b	6 months	Level 1: 1/day, 5/week Level 2: 2/day, 5/week Level 3: 2/day, 5‐7/week	Level 1: 45 minutes Level 2 and 3: 30‐45 minutes	Therapists, family members
Zhu 2006	Not stated	5/week	60 minutes	Rehabilitation nurses
Zhu 2007	3 months	5/week	45 minutes	Therapists
Zhu 2016	4 weeks	4/day, 5 days/week	120 minutes in addition to rehabilitation treatment received by both groups	Therapist
Zhuang 2012	4 weeks	1/day, 6 days/week	60 minutes physiotherapy	Physiotherapist

ADL = activities of daily living; FLO = functional limb overloading group; HEP = home exercise programme; IQR = interquartile range; LORT = limb overloading resistance training group; MTS = mobilisation and tactile stimulation group; OT: occupational therapy; PT = physical therapy; sCIMT = repetitions during constraint induced movement therapy group; SD = standard deviation; tCIMT = time spent in constraint induced movement therapy group

Further details of the interventions within studies included in quantitative analyses are provided in Table, Table, Table, Table, and Table.

**12:** Summary of intervention treatment components ‐ Comparison of physical rehabilitation versus no physical rehabilitation

**Study**	**Description**	**Routine physical therapy**	**Functional task training**	**Musculoskeletal intervention**	**Cardio‐pulmonary interventions**	**Neuro‐physiological intervention**	**Modalities/assistive devices**	**Cognitive information processing**	**Support and/or practice of activities**	**Assessment and monitoring**	**Interventions ‐stroke‐related problems**	**Other reported treatment components**
Aravind 2022	Community‐based exercise programmes supported through healthcare‐community partnerships		Y	Y	Y				Y			
Bai 2013	Acupuncture + physiotherapy		Y	Y								
Batchelor 2012	Falls prevention programme		Y	PY				Y	Y	Y	PY	
Bek 2016	Conductive education							Y				
Brouwer 2018	Tune‐up intervention		PY	PY	PY			Y		Y		Motor coordination goals
Chang 2015	Three‐stage rehabilitation		Y	Y		Y	Y		Y			
Chen S 2021	Advanced practice nurse‐guided home‐based rehabilitation exercise programme (HREPro)		Y	Y				Y	Y			
Chen J 2014	Community‐based three‐level rehabilitation therapy		Y	Y	Y				Y			
Cheng 2021	Three stage rehabilitation								Y			
Chu 2003	"Unobstruction techniques"		Y	Y		Y						Bobath shaking hands exercise
Dai 2015	Acupuncture + rehabilitation training		Y	Y		Y						
Deng 2011	Rehabilitation		Y	Y								Rehabilitation training according to Brunnstrom stages; turning over; separation exercise training of upper limbs and hands
Fan WK 2006	Three‐stage rehabilitation		Y	Y					Y		Y	Rolling practice; relaxation practice
Fang 2003	Additional early physiotherapy			Y		Y						
Fang YN 2004	Bobath			Y		Y						
Green 2002	Community physiotherapy											
Guo L 2012	Rehabilitation		Y	Y					Y		Y	Guided family rehabilitation with a training plan that is updated regularly
Guo Z 2015	Lower extremity rehabilitation training		Y	Y								
Holmgren 2006	High intensity functional exercise		Y	Y				Y	Y	Y		
Hoseinabadi 2013	Physical therapy		Y	Y								
Hou 2006	Three‐stage rehabilitation		Y	Y	Y	Y	Y		Y			Rood technique and Brunnstrom technique neural network
Hou Zhi 2014	Three stage rehabilitation		Y	Y				Y	Y			
Hu 2007	Three‐stage rehabilitation											
Huang 2003	Exercise therapy (combined Bobath, Rood, MRP and PNF)		Y	Y	Y	Y	Y		Y			Rood; rolling practice
Huang 2014	Bobath technique		Y	Y		Y						Key point control; Bobath handshake; roll over training
	Brunnstrom technique											Brunnstrom
	PNF					Y						
	Rood approach					Y	Y					Rood technology; visual and auditory stimulation
Hui‐Chan 2009	Task‐related training and Placebo TENS		Y	Y			N					Rhythmic auditory cues
Ji Pei 2014	Bobath		Y	Y	Y	Y						Turning over in bed
Knox 2018	Task‐oriented circuit gait training		Y	PY					Y			
	Strength training of lower extremities		PY	Y				Y				
Koç 2015	Nurse‐supervised, home‐based intervention to improve ADL		Y	Y								
Lee 2015	Combined aerobic and resistance exercise group		Y	Y	Y		Y					
Li 1999	Bobath		Y	Y		Y						Rolling in bed
Li Yuanzheng 2014	Bobath		Y	Y		Y						Dissociative exercise overcoming abnormal patterns; rollover training
Lu 2004	Three‐stage rehabilitation		Y	Y		Y						
Lu Liangyan 2014	Three‐stage rehabilitation		Y	Y	Y		Y	Y	Y			
Meng Fanda 2021	Routine rehabilitation training		Y	Y			Y					Swimming; Tai Chi
Ni 1997	Bobath and Brunnstrom focused exercise therapy		Y	Y		Y	Y					Bobath and Brunnstrom focused exercise therapy
Pan 2004	Early rehabilitation		Y	Y								Bobath method of holding hands
Pang 2006	"Cocktail treatment"		PY	PY		Y	Y					
Qin 2013	Motor relearning + Needle retention acupuncture		Y	Y			Y		Y			
ReTrain 2018	ReTrain		Y	Y	PY			Y	Y			
Signal 2014	Strength for Task Training (STT) ‐ Progressive Strength Training (PRST) + Task specific training (TST)		Y	Y								
SunRISe 2021	Home‐based physiotherapy programme		Y	Y	Y	Y		Y		Y		
Teixeira‐Salmela 1999	Lower limb muscle strengthening + aerobic exercise		Y	Y	Y		PY		Y	Y		
Torres‐Arreola 2009	Physiotherapy plus caregiver education in rehabilitation		Y	Y		PY	Y	Y	Y			Physiotherapy strategy was designed and applied based on patient condition according to the Brunnstrom scale.
Wade 1992	Problem solving physiotherapy intervention		Y		PY		PY	Y			PY	
Wan Xueli 2014	Bobath		Y	Y		Y			Y			Shaking hands; turning over
Wang 2004a	Early‐stage physical rehabilitation		Y	Y		Y	Y	Y	Y			
Wang 2013	Early Bobath rehabilitation		Y	Y		Y						Turn over
Wang 2015	Caregiver ‐mediated training		Y	Y								
Wang 2022	Nurse‐led motor function intervention programme based on Orem's theory		Y	Y								
Wang Dongya 2015	Three‐stage rehabilitation		Y	Y	Y		Y	Y		Y	Y	Teach the patient to turn over [in bed]
Wu 2006	Rehabilitation		Y	Y		Y					Y	Bobath method of holding hands; traction; rolling practice on bed
Wu 2020	Home remote rehabilitation		Y	Y		PY	Y	Y				
Wu Jing 2015	Three‐stage rehabilitation		Y	Y	Y				Y			"Cognitive function training"
Xie 2003	Early rehabilitation nursing		Y	Y			PY					Intermittent or constant traction
Xu 1999	Rehabilitation					Y			Y			Various exercise treatment techniques with Bobath and Brunnstrom as focus
Xu 2003a	Early rehabilitation		Y	Y								
Xu 2003b	Early rehabilitation		Y	PY		PY						
Xu 2004	Early rehabilitation		Y	Y		Y			Y			
Xu 2015	Comprehensive rehabilitation	Y				Y	Y			Y	Y	
Xu 2022	Rehabilitation training		Y	Y			Y	Y				Trunk muscle training: upper trunk flexion and rotation training; lower trunk flexion and rotation training
Xue 2006	Early motor relearning and Bobath		Y	Y		Y						Turning over in bed; weight‐loading training for upper limb
Yan 2002	Early rehabilitation		Y	Y				Y				Brunnstrom technique’s rapid traction stimulation through over‐pressuring of joints; Bobath method of holding hands; rolling over in bed
Yan 2015	Three‐stage rehabilitation		Y	Y		Y			Y			
	Bobath		Y	Y		Y						Knee, and elbow induced separation exercises; bed rolling exercises
Yang 2006	Task‐oriented progressive resistance strength training		Y	Y								
Yang Aiguo 2015	Bobath and acupuncture		Y	Y		Y	Y					
Ye Dayong 2010	Three‐stage rehabilitation (level 3)		Y	Y		Y	Y		Y			Bedside rehabilitation and positioning in stage I
Yin 2003a	Rehabilitation		PY	Y		Y		Y				Turning over in bed
	Rehabilitation "with therapy with intermediate frequency"		PY	Y		Y		Y				Turning over in bed
Zang 2013	Three‐stage rehabilitation and traditional Chinese medicine			Y			Y	Y				"bedside rehabilitation therapy"
Zhang 1998	Early rehabilitation		Y	Y		Y		Y	Y		Y	Rolling the patient on the bed
Zhang 2004	Three‐stage rehabilitation		Y	Y		Y		Y	Y		PY	Use of Brunnstrom etc. and rolling in bed
Zhang Jianhong 2013	Rehabilitation treatment			Y		Y						Brunnstrom technology; Rood technique
Zhao 2002	Early stage rehabilitation treatment		Y	Y					Y			
Zhao 2003	Early rehabilitation		Y	Y		PY						Exercise for speed; mental movement training
Zhao Ailiang 2016	Rehabilitation + acupuncture		Y	Y		Y	Y		Y			
Zheng 2014	Targeted rehabilitation training programme		Y	Y		Y	Y					
Zhu 2001	Rehabilitation		Y	Y		Y	Y		Y		Y	Rolling practice
Zhu 2006	Early rehabilitative nursing intervention		Y	Y		Y	Y		PY		Y	Utilising Rood technique to brush, tap, pat; arbitrary exercises to stimulate affected limbs; holding hands Bobath‐style in sit to stand
Zhu 2007	Three‐stage rehabilitation		Y	Y		Y		Y	Y			Rolling

ADL = activities of daily living; MRP = motor relearning programme; PNF = proprioceptive neuromuscular facilitation; TENS = transcutaneous electrical nerve stimulation. Y = yes; PY = probably yes; N = no

**13:** Summary of intervention treatment components ‐ Comparison of physical rehabilitation versus attention control

**Study**	**Description**	Routine **physical therapy**	**Functional task training**	**Musculoskeletal intervention**	**Cardio‐pulmonary interventions**	**Neuro‐physiological intervention**	**Modalities/assistive devices**	**Cognitive information processing**	**Support and/or practice of activities**	**Assessment and monitoring**	**Interventions ‐stroke‐related problems**	**Other reported treatment components**
Blennerhassett 2004	Movement science		Y	Y	Y		Y					
	Upper limb training (attention control)		Y				Y					
Dean 2000	Lower limb task‐related circuit training		Y	Y			Y	Y				
	Upper limb task‐related circuit training (attention control)							Y				
Dean 2006	Lower limb exercise program		Y	Y				Y	Y			
	Upper limb/cognition exercise programme (attention control)		Y						Y			
FeSTivaLS 2014	Functional strength training ‐ lower limb (FST‐LL)		Y	Y								
	Functional strength training ‐ upper limb (FST‐UL) (attention control)		Y	Y								
Ma Xue 2010	Rehabilitation		Y	Y		Y	Y		Y		Y	Rood
	Attention control		Y			Y						
Martins 2020	Task‐specific training upper limb and lower limb		Y	Y						Y		Over‐ground walking with auditory stimulus (metronome beat)
	Global stretching, memory exercises and health education (attention control)		PY	Y				Y		Y		Memory games using images, reminding the sequence of the objects, bingo using charts with pictures, speak names of fruits or animals starting with a specific letter
McClellan 2004	Home‐based mobility programme		Y					Y	Y			
	Home‐based upper limb exercises (attention control)		Y									
Moore 2016	Community‐based exercise		Y	Y	Y		Y			Y		
	Home stretching programme (attention control)			Y						Y		
Mudge 2009	Group circuit exercise		Y	Y								
	Social and educational programme sessions (attention control)							Y				Bowls club; quiz club; cafe outing; board games group
Nindorera 2022	Circuit walking, balance, cycling and strength training (CBCS)		Y	Y	Y		Y			Y		
	Delayed CBCS group ‐ participated in sociocultural activities for 12 weeks (attention control)											3 activities that did not involve energy expenditure: board games in groups; cultural discourse; drama
Pang 2005	Fitness and Mobility Exercise (FAME)		Y	Y				Y		Y		
	Seated upper extremity programme (attention control)		Y	Y			Y					
Salbach 2004	Mobility training		Y	Y	Y		Y		Y			
	Upper extremity training (attention control)		Y						Y			
Yang Jian 2007	Rehabilitation		Y	Y		Y	Y	Y	Y	Y	Y	Home‐based practice, orthotics, counselling
	Attention control					Y	Y		Y			

Y = yes; PY = probably yes; N = no

**14:** Summary of intervention treatment components ‐ Comparison of additional physical rehabilitation + usual care versus usual care

**Study**	**Description**	Routine **physical therapy**	**Functional task training**	**Musculoskeletal intervention**	**Cardio‐pulmonary interventions**	**Neuro‐physiological intervention**	**Modalities/assistive devices**	**Cognitive information processing**	**Support and/or practice of activities**	**Assessment and monitoring**	**Interventions ‐stroke‐related problems**	**Other reported treatment components**
Aries 2021	Task‐specific gait training (TSGT) (received by both groups)		Y	Y	PY		PY				PY	
	Mobilisation and tactile stimulation [+ task‐specific gait training]		Y	Y		Y						
	Textured insole [+ task‐specific gait training]						Y					
Bordoloi 2020	Rood approach [+ conventional home exercise programme]		Y	Y		Y	Y					Rood approach ‐ repetitions, traction
	Conventional home exercise programme (HEP)		Y	Y					Y	Y		Co‐ordination exercises
Cao 2014	Intensive walking training [+ routine rehabilitation therapy]		Y					Y	Y	Y		
	Routine rehabilitation therapy			Y					Y			
Cooke 2006	Functional strength training [+ routine conventional physiotherapy]		Y	Y	PY		Y	Y				Repetition; bed mobility
	Routine conventional physiotherapy (CPT)	Y	Y	Y		Y						
Dalal 2018	Prowling + proprioceptive training [+ routine physiotherapy]		Y	Y					Y			
	Routine physiotherapy	Y										
Du 2014	Early intensive walking training [+ conventional rehabilitation therapy]		Y									
	Conventional rehabilitation therapy			Y								
Duncan 1998	Home‐based exercise programme (therapist‐supervised)		Y	Y	Y	PY	PY				PY	
	Usual care	Y										
Duncan 2003	Home‐based exercise programme (therapist‐supervised)		Y	Y	Y	Y	Y				PY	
	Usual care	Y						Y				
Fang H 2010	Balance training		Y	Y		PY	Y		Y			
	Standard care		Y	Y		PY	Y		Y			
Fan X 2009	Exercise training		Y	Y			Y					
	Conventional rehabilitation		Y	Y			Y					
Frimpong 2014	Task‐oriented circuit training [+ conventional therapy]		Y	Y			Y			Y		
	Conventional therapy		Y	Y								
Guan 2017	Motor relearning programme [+ conventional rehabilitation training]		Y	Y				PY				
	Conventional rehabilitation training			Y			Y					
Harjpal 2021	Lower limb bilateral training (BTG) [+ lower limb unilateral training]			Y								
	Lower limb unilateral training (UTG)		Y	Y		Y						Rolling to both sides; Proprioceptive Neuromuscular Facilitation (PNF); flexion used along with pelvic PNF
Hong Cuicui 2016	Additional therapy		Y	Y		Y			Y			PNF and Bobath principles informed the additional therapy
	Conventional rehabilitation		Y	Y		Y			Y			
Jandaghi 2021	Visual deprivation‐stable based training [+ control]		Y									Patients instructed to close eyes whilst performing balance exercises on firm floor
	Control ‐ general physical therapy			Y								
Kim 2007	Task‐related circuit training [+ conventional physical therapy]		Y	Y								
	Conventional physical therapy	Y										
Kim 2012	Task oriented training [+ conventional physical therapy]		Y				Y					
	Conventional physical therapy	Y	Y	Y								
Kim 2012a	Rhythmic auditory stimulation gait training [+ conventional physical therapy]		Y				Y					
	Conventional physical therapy		Y	PY		PY						
Kim 2014	Gross motor group exercise [+ morning exercise]		Y	Y								Elements such as coordination
	Morning exercise			Y	Y							
Kim 2021	Task‐specific training (TST) [+ conventional physical therapy]		Y	Y	Y		Y					
	Conventional physical therapy		PY	Y	PY		Y					Superdynamics exercises
Kunkel 2013	Exercises alone [+ usual care]		Y	PY								
	Usual care	Y										
LAST 2018	Individualised coaching [+ usual care]			Y	Y			Y		Y		
	Usual care	Y										
Lee 2018	Caregiver‐mediated exercise [+ conventional post‐stroke rehabilitation]		Y	Y					PY			
	Conventional post‐stroke rehabilitation	Y	Y								Y	
Letombe 2010	Adapted physical activities (APA) [+ conventional rehabilitation]		Y	Y	Y		Y	Y		Y		Games and group activities (such as balneotherapy and table tennis) were also scheduled to improve the patients’ psychological and physical status. These activities stimulated and optimised motor control, executive functions and balance
	Conventional rehabilitation		Y	Y			Y				Y	Learning to use a wheelchair
Li Jingqian 2013	Motor relearning programme [+ conventional treatments]		Y					Y				Orofacial function
	Conventional treatments	Y	PY	PY			Y					
Li Xiaojun 2016	Task‐oriented training		Y	Y			Y					
	Conventional rehabilitation											No description ‐ just 'conventional rehabilitation'
Lindvall 2014	Body awareness therapy [+ control]		Y		PY		Y					Body awareness therapy
	Control ‐ usual care	Y										
Mai Guanghuai 2016	Lower limb training		Y	Y		Y	Y					
	Conventional rehabilitation		Y	Y		Y	Y					
Mustafaoğlu 2018	Conventional therapy (CTG) [+ body weight‐supported treadmill training]		Y	PY				Y		Y		
	Body weight‐supported treadmill training (BWSTTG)		Y				Y	Y				
Park 2021	Cross‐training to lower extremity on affected side [+ general physical therapy]			Y		PY						
	Cross‐training to lower extremity on unaffected side [+ general physical therapy]			Y		PY						
	General physical therapy (GPT)		Y	Y								
Qin JianJian 2014	Bobath technique [+ conventional rehabilitation training]		Y	Y		Y						Induce dissociative exercise, overcome abnormal patterns
	Conventional rehabilitation training	Y										
Song 2015	Individual‐based task‐oriented circuit training [+ conventional physiotherapy group]		Y									
	Conventional physiotherapy group	Y	PY									
Tang 2009	Sensory function training [+ conventional]		Y	Y		Y	Y					Superficial sensation training; deep sensation training; touch sensation training
	Conventional (Bobath)	Y	Y	Y		Y					Y	
Wang 2004b	Neural facilitation [+ basic rehabilitative training]			Y		Y						Prior to neural facilitation, patients had to undergo relaxation: compressing the joint to reduce tension using co‐contraction principles; using resistive forces exerted during flexion and extension of the non‐affected upper limb to elicit flexion and extension of the affected upper limb
	Basic rehabilitative training	Y		Y								
Wu Jiaming 2006	Rehabilitation		Y	Y		Y	Y		Y			
	Conventional rehabilitation										Y	Limited information about the conventional therapy
Wu Lotus 2016	Walking training		Y	Y			Y		Y			
	Conventional rehabilitation		Y	Y			Y					
Xiao Zhen‐dong 2014	Balance training group [+ routine rehabilitation therapy]		Y				Y					
	Routine rehabilitation therapy	Y	Y	Y		Y						
Xu 2013	Medical gymnastics [+ routine rehabilitation therapy]		Y	Y					Y	Y		
	Routine rehabilitation training	Y	PY	Y								Turning over exercises
Xu Yumei 2013	Balance training [+ conventional rehabilitation]		Y	Y	PY		Y					Turn over on the bed
	Conventional rehabilitation ‐ including traditional Chinese medicine therapy		Y	Y		Y	Y					"traditional Chinese medicine therapy proposed by Bobath"
Yang 2017	Bobath + electroacupuncture		Y	Y		Y			Y			
	Electroacupuncture		Y	Y		Y	Y		Y			
Yue Chunjiang 2014	Acupuncture and exercise re‐learning [+ conventional rehabilitation training]		Y	Y			Y					Orofacial function training
	Conventional rehabilitation training			Y			PY	Y				
Yue Lin 2012	Balance training [+ conventional rehabilitation]		Y									
	Conventional rehabilitation	Y	PY	PY			Y					
Zhu 2016	m‐CIMT gait training [+ standardised comprehensive rehabilitation]		Y	Y		PY		Y	Y			High number of repetitions: sit to stand transfers using a suitable chair 200–300 times per day
	Standardised comprehensive rehabilitation		Y	Y				Y				
Zhuang 2012	Physiotherapy [+ acupuncture]			PY		Y						
	Acupuncture						Y					
	Conventional care (received by both groups)										Y	

Square brackets are used to indicate therapies which are received by that trial group, but described separately for that trial (as indicated by the name in the square brackets). m‐CIMT = modified constraint induced movement therapy; PNF = proprioceptive neuromuscular facilitation Y = yes; PY = probably yes; N = no; * = study awaiting translation

**15:** Summary of intervention treatment components ‐ Comparison of different approaches

**Study**	**Description**	Routine **physical therapy**	**Functional task training**	**Musculoskeletal intervention**	**Cardio‐pulmonary interventions**	**Neuro‐physiological intervention**	**Modalities/assistive devices**	**Cognitive information processing**	**Support and/or practice of activities**	**Assessment and monitoring**	**Interventions ‐stroke‐related problems**	**Other reported treatment components**
Alabdulwahab 2015	Functional limb overloading (FLO)		Y		PY		Y					
	Limb Overloading Resistance Training LORT			Y			Y					
Aloraini 2022	Constraint‐induced movement therapy for the lower extremity (CIMT‐LE)		Y					Y	Y			Exercises were targeted towards the more affected lower extremity and administered using a shaping technique. Shaping tasks were selected by the therapist based on the targeted movement (e.g. knee extension).
	Conventional post‐stroke rehabilitation programme		Y	Y	Y			Y	Y			
Anandan 2020	Task‐specific training		Y									
	Proprioceptive neuromuscular facilitation					Y						
Arabzadeh 2018	Task‐oriented exercise		Y	Y			Y					
	Traditional		Y	PY								
Arya 2019	Interlimb coupling		PY	PY			Y					
	Conventional					PY	Y					
Bale 2008	Strength training		Y	Y							PY	
	Bobath (usual training)		Y			Y					Y	Focussing on movements on the affected side and symmetrical use of the body. Use of excessive muscle power was avoided to prevent associated reactions during training
Bhatia 2014	Task‐specific strength training		Y	Y				Y		PY		
	Resistance training			Y						Y		
	Conventional therapy (received by both groups)		Y	Y								
Brock 2005	Bobath		Y			Y						
	Task practice		Y		Y							Repeated task‐specific practice
Bui 2019	Motor relearning and acupuncture		Y				Y					
	Bobath and acupuncture		Y	Y		Y	Y					
Candan 2017	mCIMT		Y				Y		Y			
	Rehabilitation programme		Y	Y					N			
Chae 2017	Proprioceptive training [+ General physical therapy]		Y									
	General physical therapy		Y	Y	PY	Y	Y					
Chen G 2014	Task function training group		Y	Y				Y				
	Strength training group			Y								
	Routine rehabilitation (received by both groups)		Y	Y								
Chen P 2014	Functional exercise training		Y	Y		Y	Y					
	Motor control training and antispasmodic training					Y						
	Routine rehabilitation therapy (received by both groups)		Y									
Choi YK 2013	PNF and kinesio tape					Y	Y					
	Neurodevelopmental treatment					PY						
Choi JU 2015	Task‐oriented training programme		Y									
	Traditional rehabilitation therapy		PY	PY								
Ding 2015	Task‐specific training		Y									
	Strength training			Y								
	Physical rehabilitation (received by both groups)		Y	Y								
DOSE 2020	High‐intensity exercise programme (1 hour/day) based on aerobic and walking exercise		Y	Y	Y		PY		Y	Y		
	Usual care	Y	PY	PY						Y		
Duan 2011	Task‐oriented training		Y				Y					
	Neurodevelopmental training (NDT)		Y	Y		Y	Y					
Epple 2020	Vojta therapy		PY			Y						
	Conventional physiotherapy		Y	Y								Repetitive sensorimotor exercises
Fan WS 2006	PNF					Y				Y		
	Conventional rehabilitation		Y	Y						Y		
Ge Y 2020	Mandatory exercise therapy		Y	Y	Y		Y					
	Conventional exercise therapy		Y	Y		Y						
Gelber 1995	Neurodevelopment techniques		Y			Y			Y			Encouragement of use of affected side. Resistive exercises and use of abnormal reflexes and mass movements were avoided.
	Functional training		Y	Y			Y		Y			Patients allowed to use unaffected side to perform functional tasks
Guo L 2013	Task‐oriented training and muscle strength training		Y	Y								
	Facilitation techniques			Y		Y						Rood excitatory and inhibitory stimulation application; key point control
Haral 2014	Sensorimotor Integration		Y			Y						
	Conventional training		Y	Y								
Hendrey 2018	Ballistic strength training		Y	Y								
	Standard therapy		Y	Y	Y		Y					
Huang 2014	Bobath technique		Y	Y		Y						Key point control; Bobath handshake plus "other training‐based"; rollover training
	Rood approach					Y	Y					Rood technology; visual and auditory stimulation; pulling the intrinsic muscles of the hand or foot to cause synergistic contraction of the adjacent fixed muscles
	Brunnstrom technique											Brunnstrom: "Resistance exercise is applied to the patient's head and the unaffected limb to induce the joint response or joint movement of the affected limb."
	PNF					Y						
Kim 2016	Mobility and gait training circuit training		Y	Y	Y		Y				Y	
	Individual physiotherapy of neurodevelopmental treatment					PY					Y	
Kim 2018	Co‐ordinative locomotor training		Y			Y						
	Conventional neurodevelopment treatment		Y			Y						
Kim 2021	Cognitive sensorimotor exercise (CSE) [+ task‐specific training]					Y						
	Task‐specific training (TST) [+ conventional physical therapy]		PY	Y		PY	Y					
	Conventional physical therapy		PY	Y	PY		Y					
Knox 2018	Task‐oriented circuit gait training		Y	PY					Y			
	Strength training of lower extremities		PY	Y				Y				
Kuberan 2017	Task‐oriented training (TOT) with sensory manipulation		Y									Sensory manipulation (e.g. blindfolding) was used during exercises after first session getting progressively more challenging
	Conventional physical therapy		Y	Y		Y						
Kumaran 2015	Exercise programme		Y	Y			Y	Y				
	Conventional physiotherapy		Y					Y	Y			
Kwakkel 2008	Task‐oriented circuit class training		Y		PY					Y		Whole group performs a game for 15 minutes to improve walking competency.
	Usual physiotherapy	Y	Y	PY	PY							
Langhammer 2000	Motor learning		Y									
	Bobath					Y						
Langhammer 2007	Intensive exercise group		Y	Y	Y		PY		PY			Possible exercise activities for balance: dancing, tai chi. Balance exercises in sitting (where required): senior dance, balls, balloons
	Usual physiotherapy	Y										
Lawal 2016	Circuit Class Therapy (60 minutes)		Y	Y	Y		Y					
	Standard physiotherapy		Y	Y								
Li 2005	Motor learning		Y	Y	Y				Y			
	Neurodevelopmental therapy		Y	Y		Y						Rolling practice
	Acupuncture, moxibustion therapy and absent‐nerve therapy (received by both groups)						Y					Moxibustion therapy
Li 2013	Early intensive walking basic skills training [+ conventional drug treatment and rehabilitation training]		Y	Y		PY						
	Conventional drug treatment and rehabilitation training		Y									
Li Weiwei 2015	Task‐oriented training [+ routine care and rehabilitation]		Y	PY				Y	Y			
	Routine care and rehabilitation	Y										
Lincoln 2003	Bobath					Y						
	Movement science		Y									
Liu Xuan 2016	Three‐stage rehabilitation		Y	Y			Y		Y			
	Conventional rehabilitation											Usual neurology treatment
Liu Yanhua 2020	Mandatory exercise therapy		Y	Y	PY		Y					Joint training
	Routine exercise therapy		Y	Y		PY						
Marigold 2005	Agility exercise programme. Multisensory approach.		Y	Y								Standing perturbations
	Slow, low‐impact movements consisting of stretching and weight shifting		Y	Y								
Mendoza 2015	Task‐oriented circuit class		Y	Y								
	Impairment‐focused circuit class		Y	Y								
Meng 2022	Robot‐assisted gait training (RAGT) using the Walkbot robotic gym											
	Enhanced lower limb therapy (ELLT) program		Y	Y				Y				
	Conventional rehabilitation therapy (CRT)		Y	Y								
Meng Qingling 2015	Task functional training		Y									
	Strength training			Y								
	Common training (received by both groups)		Y	Y								
Outermans 2010	High‐intensity task‐oriented training programme		Y	Y	Y							
	Low‐intensity physiotherapy		Y									
Pandian 2014	Motor therapy on non‐paretic side and bimanual activities		Y	Y								Bimanual activities such as arm cycling, rowing
	Standard motor rehabilitation (Brunnstrom) ‐ affected side only		Y	PY								
Puckree 2014	Balance and stability‐focused rehabilitation		Y						Y			
	Regular physiotherapy		Y	Y					Y			
Rahayu 2020	Neurorestoration protocol		PY			PY						Constraint‐Induced Movement Therapy (CIMT); Rood
	Conventional physiotherapy	Y		Y	Y							
Richards 1993	Motor learning		Y	PY			Y				Y	
	Bobath					Y	Y				Y	
Sekhar 2013	Isokinetic strengthening and balance		Y	Y			Y					
	Conventional physiotherapy		Y	Y								
Shin 2011	Combined exercise training		Y	Y	Y		Y		Y			
	Conventional		Y	Y				PY				
Shuai 2013	Motor relearning therapy		Y									
	Bobath, Brunnstrom and PNF					Y						Brunnstrom technique
Signal 2014	Progressive Strength Training (PRST)			Y								
	Task‐specific training (TST)		Y									
Thaut 2007	Rhythmic Auditory Stimulation		Y				Y					Rhythmic Auditory Stimulation
	Neurodevelopmental therapy/Bobath‐based training		Y			Y						
Verma 2011	Task‐specific training		Y	Y					Y			Motor imagery (MI). Participants were familiarised with the MI during a pre‐intervention session and educated about the basic imagery principles.
	Bobath	Y				Y						
Wang 2005	Bobath					Y		Y				Key point control
	Orthopaedic treatment techniques		Y	Y								Rolling
Wang Leilei 2020	Task‐oriented training + acupuncture		Y	Y					Y			
	Conventional rehabilitation + Acupuncture		Y	Y		Y	Y					
Wang Wenwei 2012	Bobath training		Y	Y		Y	Y		Y			
	Conventional rehabilitation		Y	Y								
Xu Wenyu 2012	Walking training		Y			Y						
	Conventional rehabilitation		Y			Y						
Yadav 2016	Specific balance strategy training programme		Y	Y								Multitask practice carried out during balance exercises, e.g. balancing a cup with or without water on a saucer or while adding a cognitive task to the manual task.
	General balance exercise programme		Y	Y								
Zhang Huiyu 2021	Goal‐oriented functional exercise		Y	Y						Y		Medium‐term goals: cognitive function training. Long‐term goals: intelligence training. Interventions guide patients through activities such as puzzles, sensory stimulation, and numerical calculations, and improve their memory skills by asking them to retell stories.
	Routine intervention		Y	Y	Y							

Square brackets are used to indicate therapies which are received by that trial group, but described separately for that trial (as indicated by the name in the square brackets). m‐CIMT or mCIMT = modified constraint induced movement therapy; PNF = proprioceptive neuromuscular facilitation Y = yes; PY = probably yes; N = no

**16:** Summary of intervention delivery modes

**Study**	**Description**	**One to one vs group**	**Treatment delivered** **face to face or remotely**	**Individualised treatment?**
**Physical rehabilitation versus no physical rehabilitation**				
Aravind 2022	Community‐based exercise programmes supported through healthcare‐community partnerships	Group session	Face to face	Unclear
Bai 2013	Acupuncture + physiotherapy	One to one with therapist	Face to face	Unclear
Batchelor 2012	Falls prevention programme	One to one with therapist	Face to face	Y
Bek 2016	Conductive education	Group session	Face to face	Unclear
Brouwer 2018	Tune‐up intervention	One to one with therapist	Face to face	Y
Chang 2015	Three‐stage rehabilitation	One to one with therapist	Face to face	Y
Chen S 2021	Advanced practice nurse‐guided home‐based rehabilitation exercise programme (HREPro)	One to one with therapist	Face to face	N
Chen J 2014	Community‐based three‐level rehabilitation therapy	One to one with therapist	Face to face	Unclear
Cheng 2021	Three‐stage rehabilitation	Unclear	Face to face	Unclear
Chu 2003	"Unobstruction techniques"	Unclear	Face to face	N
Dai 2015	Acupuncture + rehabilitation training	One to one with therapist	Face to face	Unclear
Deng 2011	Rehabilitation	Unclear	Face to face	Y
Fan WK 2006	Three‐stage rehabilitation	One to one with therapist	Face to face	N
Fang 2003	Additional early physiotherapy	Unclear	Face to face	Unclear
Fang YN 2004	Bobath	Unclear	Face to face	Unclear
Green 2002	Community physiotherapy	Unclear	Face to face	PY
Guo L 2012	Rehabilitation	One to one with therapist; family‐assisted training	Face to face	Y
Guo Z 2015	Lower extremity rehabilitation training	Unclear	Face to face	N
Holmgren 2006	High‐intensity functional exercise	Unclear	Unclear	Y
Hoseinabadi 2013	Physical therapy	Unclear	Face to face	Unclear
Hou 2006	Three‐stage rehabilitation	Unclear	Face to face	N
Hou Zhi 2014	Three‐stage rehabilitation	Unclear	Face to face	Unclear
Hu 2007	Three‐stage rehabilitation	Unclear	Unclear	Unclear
Huang 2003	Exercise therapy (combined Bobath, Rood, MRP and PNF)	Unclear	Face to face	Unclear
Huang 2014	Bobath technique	Unclear	Face to face	N
	Brunnstrom technique	Unclear	Face to face	N
	PNF	Unclear	Face to face	N
	Rood approach	Unclear	Face to face	N
Hui‐Chan 2009	Task‐related training and placebo TENS	Unclear	Face to face	N
Ji Pei 2014	Bobath	Unclear	Face to face	N
Knox 2018	Task‐oriented circuit gait training	Unclear	Face to face	Unclear
	Strength training of lower extremities	Unclear	Face to face	Unclear
Koç 2015	Nurse‐supervised, home‐based intervention to improve ADL	One to one with therapist	Face to face	N
Lee 2015	Combined aerobic and resistance exercise group	Unclear	Face to face	Y
Li 1999	Bobath	One to one with therapist	Face to face	N
Li Yuanzheng 2014	Bobath	Unclear	Face to face	Unclear
Lu 2004	Three‐stage rehabilitation	Unclear	Face to face	N
Lu Liangyan 2014	Three‐stage rehabilitation	One to one with therapist	Face to face	PY
Meng Fanda 2021	Routine rehabilitation training	Unclear	Face to face	Unclear
Ni 1997	Bobath and Brunnstrom focused exercise therapy	Unclear	Face to face	Unclear
Pan 2004	Early rehabilitation	Unclear	Face to face	N
Pang 2006	"Cocktail treatment"	Unclear	Face to face	Unclear
Qin 2013	Motor relearning + needle retention acupuncture	Unclear	Face to face	N
ReTrain 2018	ReTrain	Mixed	Face to face	Y
Signal 2014	Strength for Task Training (STT) ‐ Progressive Strength Training (PRST) + Task specific training (TST)	Group session	Face to face	Y
SunRISe 2021	Home‐based physiotherapy programme	One to one with therapist	Mixed	N
Teixeira‐Salmela 1999	Lower limb muscle strengthening + aerobic exercise	Group session	Face to face	Y
Torres‐Arreola 2009	Physiotherapy plus caregiver education in rehabilitation	One to one with therapist	Face to face	PY
Wade 1992	Problem solving physiotherapy intervention	One to one with therapist	Face to face	Y
Wan Xueli 2014	Bobath	Unclear	Face to face	Unclear
Wang 2004a	Early‐stage physical rehabilitation	One to one with therapist	Face to face	Unclear
Wang 2013	Early Bobath rehabilitation	Unclear	Face to face	N
Wang 2015	Caregiver‐mediated training	One to one with therapist	Face to face	Y
Wang 2022	Nurse‐led motor function intervention programme based on Orem's theory	Unclear	Face to face	Unclear
Wang Dongya 2015	Three‐stage rehabilitation	One to one with therapist	Face to face	PY
Wu 2006	Rehabilitation	Unclear	Face to face	Unclear
Wu 2020	Home remote rehabilitation	One to one with therapist	Remote (online or telephone)	Y
Wu Jing 2015	Three‐stage rehabilitation	Unclear	Face to face	N
Xie 2003	Early rehabilitation nursing	Unclear	Face to face	Unclear
Xu 1999	Rehabilitation	Unclear	Face to face	Unclear
Xu 2003a	Early rehabilitation	Unclear	Face to face	Unclear
Xu 2003b	Early rehabilitation	Unclear	Face to face	Unclear
Xu 2004	Early rehabilitation	Unclear	Face to face	Unclear
Xu 2015	Comprehensive rehabilitation	Unclear	Face to face	Unclear
Xu 2022	Rehabilitation training	One to one with therapist	Face to face	Y
Xue 2006	Early motor relearning and Bobath	Unclear	Face to face	Unclear
Yan 2002	Early rehabilitation	Unclear	Face to face	N
Yan 2015	Three‐stage rehabilitation	Unclear	Face to face	N
	Bobath	Unclear	Face to face	Unclear
Yang 2006	Task‐oriented progressive resistance strength training	One to one with therapist	Face to face	Y
Yang Aiguo 2015	Bobath and acupuncture	One to one with therapist	Face to face	N
Ye Dayong 2010	Three‐stage rehabilitation (level 3)	One to one with therapist	Face to face	Y
Yin 2003a	Rehabilitation	Unclear	Face to face	Unclear
	Rehabilitation "with therapy with intermediate frequency"	Unclear	Face to face	Unclear
Zang 2013	Three‐stage rehabilitation and Traditional Chinese medicine	Unclear	Face to face	Unclear
Zhang 1998	Early rehabilitation	One to one with therapist	Face to face	N
Zhang 2004	Three‐stage rehabilitation	Unclear	Face to face	N
Zhang Jianhong 2013	Rehabilitation treatment	Unclear	Face to face	Unclear
Zhao 2002	Early stage rehabilitation treatment	One to one with therapist	Face to face	Unclear
Zhao 2003	Early rehabilitation	Unclear	Face to face	Unclear
Zhao Ailiang 2016	Rehabilitation + acupuncture	One to one with therapist	Face to face	Y
Zheng 2014	Targeted rehabilitation training programme	One to one with therapist	Face to face	Unclear
Zhu 2001	Rehabilitation	Unclear	Face to face	Y
Zhu 2006	Early rehabilitative nursing intervention	Unclear	Face to face	N
Zhu 2007	Three‐stage rehabilitation	Unclear	Face to face	N
**Physical rehabilitation versus attention control**				
Blennerhassett 2004	Movement science	Group session	Face to face	PY
	Upper limb training (attention control)	Group session	Face to face	PY
Dean 2000	Lower limb task‐related circuit training	Group session	Face to face	N
	Upper limb task‐related circuit training (attention control)	Group session	Face to face	N
Dean 2006	Lower limb exercise programme	Group session	Face to face	Y
	Upper limb/cognition exercise programme (attention control)	Group session	Face to face	Unclear
FeSTivaLS 2014	Functional strength training ‐ lower limb (FST‐LL)	Unclear	Face to face	Y
	Functional strength training ‐ upper limb (FST‐UL) (attention control)	Unclear	Face to face	Y
Ma Xue 2010	Rehabilitation	One to one with therapist	Face to face	Y
	Attention control	One to one with therapist	Face to face	Unclear
Martins 2020	Task‐specific training upper limb and lower limb	Group session	Face to face	PY
	Global stretching, memory exercises and health education (attention control)	Group session	Face to face	Unclear
McClellan 2004	Home‐based mobility programme	One to one with therapist	Mixed	PY
	Home‐based upper limb exercises (attention control)	One to one with therapist	Mixed	PY
Moore 2016	Community‐based exercise	Group session	Face to face	PY
	Home stretching programme (attention control)	Self‐guided	N/A	N
Mudge 2009	Group circuit exercise	Group session	Face to face	N
	Social & Educational Programme sessions (attention control)	Group session	Face to face	N
Nindorera 2022	Circuit walking, balance, cycling and strength training (CBCS)	Group session	Face to face	Y
	Delayed CBCS group ‐ participated in sociocultural activities for 12 weeks (attention control)	Group session	Face to face	Unclear
Pang 2005	Fitness and Mobility Exercise (FAME)	Group session	Face to face	N
	Seated upper extremity programme (attention control)	Group session	Face to face	N
Salbach 2004	Mobility training	Unclear	Face to face	N
	Upper extremity training (attention control)	Unclear	Face to face	PY
Yang Jian 2007	Rehabilitation	One to one with therapist	Face to face	Y
	Attention control	One to one with therapist	Face to face	Unclear
**Additional therapy + usual therapy versus usual therapy**				
Aries 2021	Task‐specific gait training (TSGT) (received by both groups)	One to one with therapist	Face to face	From menu
	Mobilisation & tactile stimulation [+ Task‐specific gait training]	One to one with therapist	Face to face	From menu
	Textured Insole [+ Task‐specific gait training]	One to one with therapist	Face to face	N
Bordoloi 2020	Rood approach [+ Conventional home exercise program]	One to one with therapist	Face to face	Y
	Conventional home exercise programme (HEP)	One to one with therapist	Face to face	Y
Cao 2014	Intensive walking training [ + Routine rehabilitation therapy]	Unclear	Face to face	N
	Routine rehabilitation therapy	Unclear	Face to face	Unclear
Cooke 2006	Functional strength training [+ Routine conventional physiotherapy]	Unclear	Face to face	Unclear
	Routine conventional physiotherapy (CPT)	Unclear	Face to face	Unclear
Dalal 2018	Prowling + proprioceptive training [+ Routine physiotherapy]	One to one with therapist	Face to face	Unclear
	Routine physiotherapy	One to one with therapist	Face to face	PY
Du 2014	Early intensive walking training [+ Conventional rehabilitation therapy]	One to one with therapist	Face to face	N
	Conventional rehabilitation therapy	Unclear	Face to face	N
Duncan 1998	Home‐based exercise programme (therapist‐supervised)	One to one with therapist	Face to face	Unclear
	Usual care	Unclear	Unclear	Unclear
Duncan 2003	Home‐based exercise programme (therapist‐supervised)	One to one with therapist	Face to face	N
	Usual care	Unclear	Unclear	Unclear
Fang H 2010	Balance training	One to one with therapist	Face to face	Unclear
	Standard care	One to one with therapist	Face to face	Unclear
Frimpong 2014	Task‐oriented circuit training [+ Conventional therapy]	Unclear	Face to face	N
	Conventional therapy	Unclear	Face to face	Unclear
Guan 2017	Motor relearning programme [+ Conventional rehabilitation training]	One to one with therapist	Face to face	PY
	Conventional rehabilitation training	One to one with therapist	Face to face	Unclear
Harjpal 2021	Lower limb bilateral training (BTG) [+ Lower limb unilateral training]	Unclear	Face to face	N
	Lower limb unilateral training (UTG)	Unclear	Face to face	N
Hong Cuicui 2016	Additional therapy	One to one with therapist; family members also supported delivery of therapy	Face to face	Y
	Conventional rehabilitation	One to one with therapist	Face to face	Unclear
Jandaghi 2021	Visual deprivation‐stable based training [+ Control]	One to one with therapist	Face to face	N
	Control ‐ general physical therapy	One to one with therapist	Face to face	Y
Kim 2007	Task‐related circuit training [+ Conventional physical therapy]	One to one with therapist	Face to face	N
	Conventional physical therapy	Unclear	Face to face	Unclear
Kim 2012	Task oriented training [+ Conventional physical therapy]	Unclear	Face to face	N
	Conventional physical therapy	Unclear	Face to face	Unclear
Kim 2012a	Rhythmic auditory stimulation gait training [+ Conventional physical therapy]	Unclear	Face to face	Y
	Conventional physical therapy	One to one with therapist	Face to face	Unclear
Kim 2014	Gross motor group exercise [+ Morning exercise]	Unclear	Face to face	PY
	Morning exercise	Unclear	Face to face	N
Kim 2021	Task‐specific training (TST) [+ Conventional physical therapy]	One to one with therapist	Face to face	N
	Conventional physical therapy	Unclear	Face to face	Unclear
Kunkel 2013	Exercises alone [+ Usual care]	Unclear	Face to face	PY
	Usual care	Unclear	Face to face	PY
LAST 2018	Individualised coaching [+ Usual care]	Mixed	Mixed	PY
	Usual care	Unclear	Face to face	Unclear
Lee 2018	Caregiver‐Mediated Exercise [+ Conventional post‐stroke rehabilitation]	One to one with therapist	Unclear	N
	Conventional post‐stroke rehabilitation	Mixed	Face to face	Unclear
Letombe 2010	Adapted physical activities (APA) [+ Conventional rehabilitation]	One to one with therapist	Face to face	Y
	Conventional rehabilitation	Unclear	Face to face	PY
Li Jingqian 2013	Motor relearning programme [+ Conventional treatments]	Unclear	Face to face	Y
	Conventional treatments	Unclear	Face to face	Unclear
Li Xiaojun 2016	Task‐oriented training	One to one with therapist	Face to face	Y
	Conventional rehabilitation	Unclear	Unclear	Unclear
Lindvall 2014	Body awareness therapy [+ Control]	Group session	Face to face	PY
	Control ‐ usual care	Unclear	Unclear	Unclear
Mai Guanghuai 2016	Lower limb training	One to one with therapist	Face to face	Y
	Conventional rehabilitation	Unclear	Face to face	Unclear
Mustafaoğlu 2018	Conventional therapy (CTG) [+ Body weight‐supported treadmill training]	Unclear	Face to face	Unclear
	Body weight‐supported treadmill training (BWSTTG)	One to one with therapist	Face to face	N
Park 2021	Cross‐training to lower extremity on affected side [+ General physical therapy]	One to one with therapist	Face to face	N
	Cross‐training to lower extremity on unaffected side [+ General physical therapy]	One to one with therapist	Face to face	N
	General physical therapy (GPT)	Unclear	Face to face	N
Qin JianJian 2014	Bobath technique [+ Conventional rehabilitation training]	Unclear	Face to face	Unclear
	Conventional rehabilitation training	Unclear	Face to face	Unclear
Song 2015	Individual‐based task‐oriented circuit training [+ Conventional physiotherapy group]	One to one with therapist	Face to face	Unclear
	Conventional physiotherapy group	Unclear	Unclear	Unclear
Tang 2009	Sensory function training [+ Conventional]	One to one with therapist	Face to face	Unclear
	Conventional (Bobath)	Unclear	Face to face	Unclear
Wang 2004b	Neural facilitation [+ Basic rehabilitative training]	Unclear	Face to face	Unclear
	Basic rehabilitative training	Unclear	Face to face	Unclear
Wu Jiaming 2006	Rehabilitation	One to one with therapist	Face to face	Y
	Conventional rehabilitation	Unclear	Unclear	Unclear
Wu Lotus 2016	Walking training	One to one with therapist	Face to face	Y
	Conventional rehabilitation	One to one with therapist	Face to face	Unclear
Xiao Zhen‐dong 2014	Balance training group [+ Routine rehabilitation therapy]	Unclear	Face to face	Unclear
	Routine rehabilitation therapy	Unclear	Face to face	Unclear
Xu 2013	Medical gymnastics [+ Routine rehabilitation therapy]	Unclear	Face to face	PY
	Routine rehabilitation training	Unclear	Face to face	Unclear
Xu Yumei 2013	Balance training [+ Conventional rehabilitation]	Unclear	Face to face	N
	Conventional rehabilitation ‐ including Traditional Chinese medicine therapy	Unclear	Face to face	Unclear
Yang 2017	Bobath + electroacupuncture	One to one with therapist	Face to face	Y
	Electroacupuncture	One to one with therapist	Face to face	Y
Yue Chunjiang 2014	Acupuncture and exercise re‐learning [+ Conventional rehabilitation training]	One to one with therapist	Face to face	Unclear
	Conventional rehabilitation training	Unclear	Face to face	Unclear
Yue Lin 2012	Balance training [+ Conventional rehabilitation]	Unclear	Face to face	Unclear
	Conventional rehabilitation	Unclear	Face to face	Unclear
Zhu 2016	m‐CIMT gait training [+ Standardised comprehensive rehabilitation]	One to one with therapist	Face to face	Y
	Standardised comprehensive rehabilitation	Unclear	Face to face	Unclear
Zhuang 2012	Physiotherapy [+ Acupuncture]	One to one with therapist	Face to face	Unclear
	Acupuncture	One to one with therapist	Face to face	N
**Comparison of different approaches**				
Alabdulwahab 2015	Functional limb overloading (FLO)	Unclear	Face to face	PY
	Limb Overloading Resistance Training LORT	Unclear	Face to face	PY
Aloraini 2022	Constraint‐induced movement therapy for the lower extremity (CIMT‐LE)	Unclear	Face to face	Unclear
	Conventional post‐stroke rehabilitation programme	Unclear	Face to face	Unclear
Anandan 2020	Task specific training	Unclear	Face to face	Unclear
	Proprioceptive neuromuscular facilitation	Unclear	Face to face	Unclear
Arabzadeh 2018	Task oriented exercise	Unclear	Face to face	Unclear
	Traditional	Unclear	Face to face	Unclear
Arya 2019	Interlimb coupling	One to one with therapist	Face to face	Y
	Conventional	One to one with therapist	Face to face	PY
Bale 2008	Strength training	Unclear	Face to face	Y
	Bobath (usual training)	Unclear	Face to face	Unclear
Bhatia 2014	Task specific strength training	Group session	Face to face	Y
	Resistance training	Unclear	Face to face	Y
Brock 2005	Bobath	One to one with therapist	Face to face	Y
	Task practice	One to one with therapist	Face to face	Unclear
Bui 2019	Motor relearning and acupuncture	Unclear	Face to face	Unclear
	Bobath and acupuncture	Unclear	Face to face	Y
Candan 2017	mCIMT	One to one with therapist	Face to face	Y
	Rehabilitation programme	Unclear	Face to face	Y
Chae 2017	Proprioceptive training [+ General physical therapy]	Unclear	Face to face	Unclear
	General physical therapy	Unclear	Face to face	Unclear
Chen G 2014	Task function training group	One to one with therapist	Face to face	N
	Strength training group	One to one with therapist	Face to face	N
Chen P 2014	Functional exercise training	Unclear	Face to face	Unclear
	Motor control training and antispasmodic training	Unclear	Face to face	Unclear
Choi YK 2013	PNF and kinesio tape	Unclear	Face to face	PY
	Neurodevelopmental treatment	Unclear	Face to face	Unclear
Choi JU 2015	Task‐oriented training programme	Unclear	Face to face	PY
	Traditional rehabilitation therapy	Unclear	Face to face	Unclear
Ding 2015	Task‐specific training	One to one with therapist	Face to face	Unclear
	Strength training	One to one with therapist	Face to face	Unclear
DOSE 2020	High intensity exercise programme (1 hour/day) based on aerobic and walking exercise	One to one with therapist	Face to face	PY
	Usual care	Unclear	Face to face	Unclear
Duan 2011	Task‐oriented training	One to one with therapist	Face to face	Unclear
	Neurodevelopmental training (NDT)	One to one with therapist	Face to face	Unclear
Epple 2020	Vojta therapy	One to one with therapist	Face to face	PY
	Conventional physiotherapy	One to one with therapist	Face to face	PY
Fan WS 2006	PNF	One to one with therapist	Face to face	Unclear
	Conventional rehabilitation	One to one with therapist	Face to face	Unclear
Ge Y 2020	Mandatory exercise therapy	Unclear	Face to face	N
	Conventional exercise therapy	Unclear	Face to face	Y
Gelber 1995	Neurodevelopment techniques	Unclear	Face to face	Unclear
	Functional training	Unclear	Face to face	Unclear
Guo L 2013	Task‐oriented training and muscle strength training	Unclear	Face to face	N
	Facilitation techniques	Unclear	Face to face	Unclear
Haral 2014	Sensorimotor Integration	One to one with therapist	Face to face	PY
	Conventional training	Unclear	Face to face	PY
Hendrey 2018	Ballistic strength training	One to one with therapist	Face to face	PY
	Standard therapy	One to one with therapist	Face to face	Y
Huang 2014	Bobath technique	Unclear	Face to face	N
	Rood approach	Unclear	Face to face	N
	Brunnstrom technique	Unclear	Face to face	N
	PNF	Unclear	Face to face	N
Kim 2016	Mobility and gait training circuit training	Group session	Face to face	Y
	Individual physiotherapy of neurodevelopmental treatment	Unclear	Face to face	PY
Kim 2018	Coordinative locomotor training	One to one with therapist	Face to face	PY
	Conventional neurodevelopment treatment	One to one with therapist	Face to face	PY
Kim 2021	Cognitive sensorimotor exercise (CSE) [+ Task‐specific training]	One to one with therapist	Face to face	N
	Task‐specific training (TST) [+ Conventional physical therapy]	One to one with therapist	Face to face	N
	Conventional physical therapy	Unclear	Face to face	Unclear
Knox 2018	Task‐oriented circuit gait training	Unclear	Face to face	Unclear
	Strength training of lower extremities	Unclear	Face to face	Unclear
Kuberan 2017	Task oriented training (TOT) with sensory manipulation	One to one with therapist	Face to face	From menu
	Conventional physical therapy	One to one with therapist	Face to face	Unclear
Kumaran 2015	Exercise programme	One to one with therapist	Face to face	PY
	Conventional physiotherapy	One to one with therapist	Face to face	Unclear
Kwakkel 2008	Task‐oriented circuit class training	Group session	Face to face	PY
	Usual physiotherapy	One to one with therapist	Face to face	Unclear
Langhammer 2000	Motor learning	One to one with therapist	Face to face	PY
	Bobath	One to one with therapist	Face to face	PY
Langhammer 2007	Intensive exercise group	One to one with therapist	Face to face	Y
	Usual physiotherapy	Unclear	Face to face	Unclear
Lawal 2016	Circuit Class Therapy (60 minutes)	Group session	Face to face	PY
	Standard physiotherapy	One to one with therapist	Face to face	PY
Li 2005	Motor learning	Unclear	Face to face	PY
	Neurodevelopmental therapy	Unclear	Face to face	PY
Li 2013	Early intensive walking basic skills training [+ Conventional drug treatment and rehabilitation training]	Unclear	Face to face	N
	Conventional drug treatment and rehabilitation training	Unclear	Face to face	Unclear
Li Weiwei 2015	Task‐oriented training [+ Routine care and rehabilitation]	One to one with therapist	Face to face	Y
	Routine care and rehabilitation	Unclear	Face to face	Unclear
Lincoln 2003	Bobath	One to one with therapist	Face to face	PY
	Movement science	One to one with therapist	Face to face	PY
Liu Xuan 2016	Three‐stage rehabilitation	One to one with therapist	Face to face	Y
	Conventional rehabilitation	Unclear	Unclear	Unclear
Liu Yanhua 2020	Mandatory exercise therapy	Unclear	Face to face	Unclear
	Routine exercise therapy	Unclear	Face to face	PY
Marigold 2005	Agility exercise programme. Multisensory approach.	Group session	Face to face	Unclear
	Slow, low‐impact movements consisting of stretching and weight shifting	Group session	Face to face	Unclear
Mendoza 2015	Task‐oriented circuit class	Group session	Face to face	Y
	Impairment‐focused circuit class	Group session	Face to face	Y
Meng 2022	Robot‐assisted gait training (RAGT) using the Walkbot robotic gym	One to one with therapist	Face to face	Unclear
	Enhanced lower limb therapy (ELLT) programme	One to one with therapist	Face to face	Unclear
	Conventional rehabilitation therapy (CRT)	One to one with therapist	Face to face	Unclear
Meng Qingling 2015	Task functional training	Unclear	Face to face	N
	Strength training	Unclear	Face to face	N
Outermans 2010	High‐intensity task‐oriented training programme	Group session	Face to face	N
	Low‐intensity physiotherapy	Group session	Face to face	N
Pandian 2014	Motor therapy on non‐paretic side and bimanual activities	Unclear	Face to face	PY
	Standard motor rehabilitation (Brunnstrom) ‐ affected side only	Unclear	Face to face	Unclear
Puckree 2014	Balance and stability‐focused rehabilitation	One to one with therapist	Face to face	N
	Regular physiotherapy	One to one with therapist	Face to face	N
Rahayu 2020	Neurorestoration protocol	One to one with therapist	Face to face	N
	Conventional physiotherapy	One to one with therapist	Face to face	Unclear
Richards 1993	Motor learning	Unclear	Face to face	Unclear
	Bobath	Unclear	Face to face	Unclear
Sekhar 2013	Isokinetic strengthening and balance	Unclear	Face to face	PY
	Conventional physiotherapy	Unclear	Face to face	PY
Shin 2011	Combined exercise training	Unclear	Face to face	Unclear
	Conventional	One to one with therapist	Face to face	Y
Shuai 2013	Motor relearning therapy	One to one with therapist	Face to face	PY
	Bobath, Brunnstrom and PNF	One to one with therapist	Face to face	Unclear
Signal 2014	Progressive Strength Training (PRST)	Group session	Face to face	Y
	Task specific training (TST)	Group session	Face to face	Y
Thaut 2007	Rhythmic Auditory Stimulation	Unclear	Face to face	N
	Neurodevelopmental therapy/Bobath‐based training	Unclear	Face to face	Unclear
Verma 2011	Task‐specific training	Group session	Face to face	N
	Bobath	Unclear	Face to face	Unclear
Wang 2005	Bobath	One to one with therapist	Face to face	Y
	Orthopaedic treatment techniques	One to one with therapist	Face to face	PY
Wang Leilei 2020	Task‐oriented training + acupuncture	One to one with therapist	Face to face	Y
	Conventional rehabilitation + acupuncture	One to one with therapist	Face to face	Y
Wang Wenwei 2012	Bobath training	One to one with therapist	Face to face	Y
	Conventional rehabilitation	Unclear	Face to face	Unclear
Xu Wenyu 2012	Walking training	One to one with therapist	Face to face	Y
	Conventional rehabilitation	Unclear	Unclear	Unclear
Yadav 2016	Specific balance strategy training programme	Unclear	Remote (online or telephone)	Y
	General balance exercise programme	Unclear	Face to face	Y
Zhang Huiyu 2021	Goal‐oriented functional exercise	Unclear	Face to face	Unclear
	Routine intervention	Unclear	Face to face	Unclear

Square brackets are used to indicate therapies which are received by that trial group, but described separately for that trial (as indicated by the name in the square brackets). ADL = activities of daily living; m‐CIMT or mCIMT = modified constraint induced movement therapy; MRP = motor relearning programme; N/A = not applicable; PNF = proprioceptive neuromuscular facilitation; TENS = transcutaneous electrical nerve stimulation. Y = yes; PY = probably yes; N = no; PN = probably no

#### Comparisons

Among the 267 included studies, 246 were parallel‐group studies, and 21 had more than one treatment group. For 8 of these 21 studies, only one comparison (i.e. two of the treatment groups) was relevant to this review, and we have dealt with these as parallel‐group studies. For the remaining 13 studies: seven had two relevant comparisons (Cooke 2006; Kim 2021; Kwakkel 2002; Morreale 2016; Signal 2014; Song 2015; Yin 2003a), five had three relevant comparisons (Aksu 2001; Baer 2007; Danlami 2017; Knox 2018; Park 2021), and one had six relevant comparisons (Huang 2014). This brings us to a total of 289 relevant comparisons from the 267 included studies.

The studies included in this review update addressed the following pre‐defined comparisons:

1. Physical rehabilitation versus no (or minimal) physical rehabilitation ‐ 105 studies (Table).
2. Physical rehabilitation versus attention control ‐ 19 studies (Table).
3. Additional physical rehabilitation + usual rehabilitation versus usual rehabilitation ‐ 56 studies (Table).
4. Comparison of different approaches to physical rehabilitation ‐ 92 studies (Table).

Three studies did not clearly fit into any of these pre‐stated comparisons (Liu 2014; Matthew Hall 2013; Wei 2014). These studies are described in Characteristics of included studies and summarised in Table; data were not included in quantitative analysis.

**17:** Summary of results of studies that did not fit pre‐stated comparisons

**Study**	**Group 1**	**Mean**	**SD**	**Total**	**Group 2**	**Mean**	**SD**	**Total**	**MD 95% CI**	**A**	**B**	**C**	**D**	**E**	**F**
	**Independence in ADL**														
**Huang 2014**	Rood	44.41	1.66	17	PNF	44.11	1.96	17	0.30 (‐0.92 to 1.52)	?	?	+	?	?	?
**Liu Xuan 2016**	Three‐stage	89.67	5.78	30	Conventional	60.98	4.98	30	28.69 (25.96 to 31.42)	?	?	+	‐	?	‐
**Xu Wenyu 2012**	Inertial guided gait rehabilitation training	79.5	2.9	50	Conventional rehab gait training	77.72	3.82	50	1.78 (0.45 to 3.11)	?	?	+	‐	?	‐
	**Motor function**														
**Huang 2014**	Rood	14.82	0.39	17	PNF	14.76	0.56	17	0.06 (‐0.26 to 0.38)	?	?	+	?	?	?

CI = confidence interval MD = mean difference PNF = proprioceptive neuromuscular proprioception SD = standard deviation Risk of bias: A = Bias arising from the randomisation process; B = Bias due to deviations from intended interventions; C = Bias due to missing outcome data; D = Bias in measurement of the outcome; E = Bias in selection of the reported result; F = Overall bias. + low risk of bias; ‐ high risk of bias; ? uncertain risk of bias.

#### Studies included in quantitative analysis

Of the included studies, 194 of 267 were suitable for inclusion in the quantitative synthesis (meta‐analysis). Six of the 194 studies were multi‐arm studies and included in more than one of the comparisons (Cooke 2006; Huang 2014; Kim 2021; Knox 2018; Park 2021; Signal 2014).

##### Interventions and comparisons

For studies included in quantitative synthesis:

- 79 studies compared physical rehabilitation with no physical rehabilitation. These are summarised in Table. Across these studies there were 85 interventions (four studies ‐ Huang 2014; Knox 2018; Yan 2015; Yin 2003a ‐ had more than one active intervention); the mode of delivery of each trial is shown in Table and summarised below:
  - 23/85 were a one‐to‐one treatment session with a therapist, 4/85 were a group session, and 1/85 was mixed. (It was unclear for 57 interventions.)
  - 77/85 interventions were delivered face‐to‐face, while 2/85 reported some element of virtual/remote‐delivered intervention and 1/85 was mixed. (It was unclear for six interventions.)
  - 18/85 interventions were either definitely or probably individualised according to each patient, while 26/85 delivered a standardised (i.e. not individualised) intervention. (This was unclear for 41 interventions.)
- 13 studies compared physical rehabilitation with attention control; across these studies, there were 26 interventions (i.e. 13 physical rehabilitation and 13 attention control interventions). These are summarised in Table; the mode of delivery of each trial is shown in Table and summarised below:
  - 2/26 were a one‐to‐one treatment session with a therapist, 15/26 were a group session, and 1/26 was a self‐guided treatment. (It was unclear for eight interventions.)
  - 19/26 interventions were delivered face‐to‐face, while 2/26 reported a mix of face‐to‐face with some element of virtual/remote‐delivered intervention. (This was not applicable to 1/26, where the treatment was self‐guided, and was unclear for four interventions)
  - 11/26 interventions were either definitely or probably individualised according to each patient, while 8/26 delivered a standardised (i.e. not individualised) intervention. (This was unclear for seven interventions.)
- 43 studies explored additional physical rehabilitation; across these studies, there were 88 interventions. These are summarised in Table; the mode of delivery of each trial is shown in Table and summarised below:
  - 28/88 were a one‐to‐one treatment session with a therapist, 1/88 was a group session, and 2/88 were mixed. (It was unclear for 57 interventions.)
  - 68/88 interventions were delivered face‐to‐face, while 1/88 reported some element of virtual/remote‐delivered intervention. (It was unclear for 19 interventions.)
  - 16/88 interventions were either definitely or probably individualised according to each patient, while 20/88 delivered a standardised (i.e. not individualised) intervention, and 2/88 used a menu‐based approach. (This was unclear for 50 interventions.)
- 63 studies compared different approaches to physical rehabilitation. These are summarised in Table; across these studies, there were 130 interventions; the mode of delivery of each trial is shown in Table and summarised below:
  - 43/130 were a one‐to‐one treatment session with a therapist, and 13/130 were a group session. (It was unclear for 74 interventions.)
  - 117/130 interventions were delivered face‐to‐face, while 1/130 reported some element of virtual/remote‐delivered intervention. (It was unclear for 12 interventions.)
  - 51/130 interventions were either definitely or probably individualised according to each patient, while 20/130 delivered a standardised (i.e. not individualised) intervention and 1/118 used a menu‐based approach. (This was unclear for 58 interventions.)

##### Outcome measures

For outcomes of independence in ADL, motor function, balance, gait velocity, and length of stay, Table, Table, Table, and Table report the outcome measures reported by the included studies and Table provides a summary of the outcome measures included within different comparisons in this review. Table and Table summarise data available on adverse events: only 19/267 studies reported adverse events in detail (Table). A further 69/267 studies provided limited details of adverse events and study dropouts (Table).

**18:** Outcome measures in studies included in comparison of physical rehabilitation versus no physical rehabilitation

**Study**	**Independence in ADL**	**Motor Function**	**Balance**	**Gait**
Aravind 2022	Barthel Index		Berg Balance Scale (BBS)	Gait velocity (m/s)
Bai 2013	Barthel Index	Fugl‐Meyer Assessment (FMA)		
Batchelor 2012				Gait velocity (m/min)
Bek 2016	Barthel Index			Timed walk over set distance ‐ 10m (s)
Brouwer 2018			Berg Balance Scale (BBS)	Timed Up and Go (s)
Chang 2015	Barthel Index			
Chen S 2021	Barthel Index	Fugl‐Meyer Assessment (FMA)		Gait velocity (m/s)
Chen J 2014	Barthel Index	Fugl‐Meyer Assessment (FMA)		
Cheng 2021		Fugl‐Meyer Assessment (FMA)		
Chu 2003	Barthel Index	Fugl‐Meyer Assessment (FMA)		
Dai 2015	Barthel Index	Fugl‐Meyer Assessment (FMA)		
Deng 2011		Fugl‐Meyer Assessment (FMA)		
Fan WK 2006		Fugl‐Meyer Assessment (FMA)		
Fang 2003	Barthel Index	Fugl‐Meyer Assessment (FMA)		
Fang YN 2004	Barthel Index	Fugl‐Meyer Assessment (FMA)		
Green 2002	Barthel Index	Rivermead Mobility Index		Gait velocity (m/min)
Guo L 2012	Barthel Index	Fugl‐Meyer Assessment (FMA)		
Guo Z 2015	Functional Independence Measure (FIM)	Fugl‐Meyer Assessment (FMA)		
Holmgren 2006	Barthel Index		Berg Balance Scale (BBS)	
Hoseinabadi 2013	Barthel Index		Berg Balance Scale (BBS)	
Hou 2006	Barthel Index			
Hou Zhi 2014	Barthel Index	Fugl‐Meyer Assessment (FMA)		
Hu 2007		Fugl‐Meyer Assessment (FMA)		
Huang 2003	Barthel Index	Fugl‐Meyer Assessment (FMA)		
Huang 2014	Barthel Index	Fugl‐Meyer Assessment (FMA)		
Hui‐Chan 2009				Gait velocity (cm/s)
Ji Pei 2014	Barthel Index			
Knox 2018			Berg Balance Scale (BBS)	Gait velocity (m/s)
Koç 2015	Barthel Index			
Lee 2015				Gait velocity (m/s)
Li 1999	Barthel Index	Fugl‐Meyer Assessment (FMA)		
Li Yuanzheng 2014	Barthel Index	Fugl‐Meyer Assessment (FMA)		
Lu 2004	Barthel Index	Fugl‐Meyer Assessment (FMA)		
Lu Liangyan 2014	Barthel Index			
Meng Fanda 2021		Fugl‐Meyer Assessment (FMA)	Berg Balance Scale (BBS)	
Ni 1997		Fugl‐Meyer Assessment (FMA)		
Pan 2004	Barthel Index	Fugl‐Meyer Assessment (FMA)		
Pang 2006	Barthel Index			
Qin 2013		Fugl‐Meyer Assessment (FMA)		Timed Up and Go (s)
ReTrain 2018		Rivermead Mobility Index		Timed Up and Go (s)
Signal 2014				Gait velocity (m/s)
SunRISe 2021			Berg Balance Scale (BBS)	Distance walked in set time ‐ 6MWT (m)
Teixeira‐Salmela 1999				Gait velocity (m/s)
Torres‐Arreola 2009				
Wade 1992	Barthel Index	Rivermead Mobility Index		Timed walk over set distance ‐ 10m (s)
Wan Xueli 2014	Barthel Index			
Wang 2004a		Fugl‐Meyer Assessment (FMA)		
Wang 2013	Barthel Index			
Wang 2015	Barthel Index		Berg Balance Scale (BBS)	Gait velocity (cm/s)
Wang 2022	Barthel Index	Motor Assessment Scale		
Wang Dongya 2015		Fugl‐Meyer Assessment (FMA)		
Wu 2006	Barthel Index	Fugl‐Meyer Assessment (FMA)		
Wu 2020	Barthel Index	Fugl‐Meyer Assessment (FMA)	Berg Balance Scale (BBS)	Timed Up and Go (s)
Wu Jing 2015		Fugl‐Meyer Assessment (FMA)		
Xie 2003	Barthel Index			
Xu 1999	Barthel Index			
Xu 2003a	Barthel Index	Fugl‐Meyer Assessment (FMA)		
Xu 2003b	Barthel Index	Fugl‐Meyer Assessment (FMA)		
Xu 2004	Barthel Index			
Xu 2015		Fugl‐Meyer Assessment (FMA)		
Xu 2022	Barthel Index	Fugl‐Meyer Assessment (FMA)		
Xue 2006	Barthel Index	Fugl‐Meyer Assessment (FMA)		
Yan 2002	Barthel Index			
Yan 2015	Functional Independence Measure (FIM)	Fugl‐Meyer Assessment (FMA)		
Yang 2006				Gait velocity (cm/s)
Yang Aiguo 2015	Barthel Index	Fugl‐Meyer Assessment (FMA)		
Ye Dayong 2010		Fugl‐Meyer Assessment (FMA)		
Yin 2003a		Fugl‐Meyer Assessment (FMA)		
Zang 2013		Fugl‐Meyer Assessment (FMA)		
Zhang 1998	Barthel Index	Fugl‐Meyer Assessment (FMA)		
Zhang 2004	Barthel Index	Fugl‐Meyer Assessment (FMA)		
Zhang Jianhong 2013	Barthel Index	Fugl‐Meyer Assessment (FMA)		
Zhao 2002		Fugl‐Meyer Assessment (FMA)		
Zhao 2003	Barthel Index			
Zhao Ailiang 2016	Barthel Index			
Zheng 2014	Barthel Index	Fugl‐Meyer Assessment (FMA)		
Zhu 2001		Fugl‐Meyer Assessment (FMA)		
Zhu 2006	Barthel Index	Fugl‐Meyer Assessment (FMA)		
Zhu 2007	Barthel Index	Fugl‐Meyer Assessment (FMA)		
**Total studies = 79**	**Studies included in analysis = 52 (see**Analysis 1.1**)**	**Studies included in analysis = 50 (see**Analysis 1.2**)**	**Studies included in analysis = 9 (see**Analysis 1.3**)**	**Studies included in analysis = 18 (see**Analysis 1.4**)**

6MWT: 6‐minute walk test m: metres min: minutes s: seconds

**19:** Outcome measures in studies included in comparison of physical rehabilitation versus attention control

**Study**	**Independence in ADL**	**Motor Function**	**Balance**	**Gait**
Blennerhassett 2004				Timed Up and Go (s)
Dean 2000				Gait velocity (cm/s)
Dean 2006				Gait velocity (m/s)
FeSTivaLS 2014		Rivermead Mobility Index		Timed Up and Go (s)
Ma Xue 2010		Fugl‐Meyer Assessment (FMA)		
Martins 2020				Gait velocity (m/s)
McClellan 2004		Motor Assessment Scale		
Moore 2016			Berg Balance Scale (BBS)	Gait velocity (m/s)
Mudge 2009		Rivermead Mobility Index		Gait velocity (m/s)
Nindorera 2022	ACTIVLIM‐Stroke scale		Berg Balance Scale (BBS)	Gait velocity (m/s)
Pang 2005			Berg Balance Scale (BBS)	
Salbach 2004			Berg Balance Scale (BBS)	Gait velocity (m/s)
Yang Jian 2007	Functional Independence Measure (FIM)	Fugl‐Meyer Assessment (FMA)		
**Total studies = 13**	**Studies included in analysis = 2 (see**Analysis 3.1**)**	**Studies included in analysis = 5 (see**Analysis 3.2**)**	**Studies included in analysis = 4 (see**Analysis 3.3**)**	**Studies included in analysis = 9 (see**Analysis 3.4**)**

6MWT: 6‐minute walk test ACTIVLIM‐Stroke scale: self‐reported Rasch‐built questionnaire that measures the ability of stroke patients to perform activities of daily living m: metres min: minutes s: seconds

**20:** Outcome measures in studies included in comparison of additional physical rehabilitation + usual therapy versus no usual therapy

**Study**	**Independence in ADL**	**Motor Function**	**Balance**	**Gait**
Aries 2021		Rivermead Mobility Index		Timed walk over set distance ‐ 5m (s)
Bordoloi 2020	Barthel Index			
Cao 2014	Barthel Index			
Cooke 2006		Rivermead Mobility Index		Gait velocity (m/s)
Dalal 2018				Timed walk over set distance ‐ 5m (s)
Du 2014	Barthel Index	Fugl‐Meyer Assessment (FMA)		
Duncan 1998	Barthel Index	Fugl‐Meyer Assessment (FMA)	Berg Balance Scale (BBS)	Gait velocity (no units)
Duncan 2003		Fugl‐Meyer Assessment (FMA)	Berg Balance Scale (BBS)	Gait velocity (no units)
Fang H 2010	Barthel Index		Berg Balance Scale (BBS)	
Frimpong 2014				Timed walk over set distance ‐ 10m (s)
Guan 2017	Barthel Index	Rivermead Mobility Index		
Harjpal 2021			Berg Balance Scale (BBS)	Gait velocity (m/s)
Hong Cuicui 2016		Fugl‐Meyer Assessment (FMA)		
Jandaghi 2021				Timed Up and Go (s)
Kim 2007	Barthel Index		Berg Balance Scale (BBS)	Distance walked in set time ‐ 6MWT (m)
Kim 2012			Berg Balance Scale (BBS)	Timed walk over set distance ‐ 10m (s)
Kim 2012a				Gait velocity (cm/s)
Kim 2014	Barthel Index			
Kim 2021				Gait velocity (m/s)
Kunkel 2013		Rivermead Mobility Index	Berg Balance Scale (BBS)	Timed walk over set distance ‐ 10m (s)
LAST 2018	Barthel Index	Motor Assessment Scale		Gait velocity (m/s)
Lee 2018	Barthel Index		Berg Balance Scale (BBS)	
Letombe 2010	Barthel Index			
Li Jingqian 2013	Barthel Index	Motor Assessment Scale		
Li Xiaojun 2016	Barthel Index	Fugl‐Meyer Assessment (FMA)		
Lindvall 2014			Berg Balance Scale (BBS)	Timed Up and Go (s)
Mai Guanghuai 2016	Barthel Index	Fugl‐Meyer Assessment (FMA)	Berg Balance Scale (BBS)	
Mustafaoğlu 2018		Rivermead Mobility Index	Berg Balance Scale (BBS)	Timed walk over set distance ‐ 10m (s)
Park 2021				Gait velocity (m/s)
Qin JianJian 2014	Barthel Index	Fugl‐Meyer Assessment (FMA)		
Song 2015				Gait velocity (cm/s)
Tang 2009		Fugl‐Meyer Assessment (FMA)		
Wang 2004b		Fugl‐Meyer Assessment (FMA)		
Wu Jiaming 2006	Barthel Index	Fugl‐Meyer Assessment (FMA)		
Wu Lotus 2016*	Barthel Index	Fugl‐Meyer Assessment (FMA)		
Xiao Zhen‐dong 2014			Berg Balance Scale (BBS)	
Xu 2013	Barthel Index		Berg Balance Scale (BBS)	
Xu Yumei 2013			Berg Balance Scale (BBS)	
Yang 2017	Barthel Index	Fugl‐Meyer Assessment (FMA)		
Yue Chunjiang 2014	Barthel Index	Fugl‐Meyer Assessment (FMA)	Berg Balance Scale (BBS)	
Yue Lin 2012		Fugl‐Meyer Assessment (FMA)		
Zhu 2016				Gait velocity (m/s)
Zhuang 2012	Barthel Index	Fugl‐Meyer Assessment (FMA)		
**Total studies = 43**	**Studies included in analysis = 21 (see**Analysis 5.1**)**	**Studies included in analysis = 22 (see**Analysis 5.2**)**	**Studies included in analysis = 15 (see**Analysis 5.3**)**	**Studies included in analysis = 19 (see**Analysis 5.4**)**

6MWT: 6‐minute walk test m: metres min: minutes s: seconds

**21:** Outcome measures in studies included in comparison of different physical rehabilitation approaches

**Study**	**Independence in ADL**	**Motor Function**	**Balance**	**Gait**
Alabdulwahab 2015				Gait velocity (m/s)
Aloraini 2022		Fugl‐Meyer Assessment (FMA)	Berg Balance Scale (BBS)	Gait velocity (m/s)
Anandan 2020			Berg Balance Scale (BBS)	
Arabzadeh 2018			Berg Balance Scale (BBS) ‐ Persian version	
Arya 2019	Modified Rankin scale	Fugl‐Meyer Assessment (FMA)		
Bale 2008		Motor Assessment Scale		Gait velocity (m/s)
Bhatia 2014				Timed Up and Go (s)
Brock 2005			Berg Balance Scale (BBS)	Gait velocity (m/min)
Bui 2019	Barthel Index			
Candan 2017			Berg Balance Scale (BBS)	Gait velocity (m/s)
Chae 2017			Berg Balance Scale (BBS)	Timed Up and Go (s)
Chen G 2014	Barthel Index	Fugl‐Meyer Assessment (FMA)		
Chen P 2014	Barthel Index	Fugl‐Meyer Assessment (FMA)		
Choi YK 2013			Berg Balance Scale (BBS)	Timed walk over set distance ‐ 10m (s)
Choi JU 2015	Barthel Index		Berg Balance Scale (BBS)	
Ding 2015	Barthel Index	Fugl‐Meyer Assessment (FMA)		
DOSE 2020			Berg Balance Scale (BBS)	Distance walked in set time ‐ 6MWT (m)
Duan 2011*			Berg Balance Scale (BBS)	Gait velocity (m/s)
Epple 2020	Barthel Index			
Fan WS 2006	Barthel Index	Fugl‐Meyer Assessment (FMA)		
Ge Y 2020	Barthel Index	Fugl‐Meyer Assessment (FMA)		Gait velocity (no units)
Gelber 1995	Functional Independence Measure			Gait velocity (m/s)
Guo L 2013	Barthel Index	Fugl‐Meyer Assessment (FMA)		
Haral 2014			Berg Balance Scale (BBS)	Gait velocity (no units)
Hendrey 2018				Gait velocity (m/s)
Huang 2014	Barthel Index	Fugl‐Meyer Assessment (FMA)		
Kim 2016	Barthel Index	Fugl‐Meyer Assessment (FMA)	Berg Balance Scale (BBS)	Distance walked in set time ‐ 6MWT (m)
Kim 2018				Gait velocity (cm/s)
Kim 2021				Gait velocity (m/s)
Knox 2018			Berg Balance Scale (BBS)	Gait velocity (m/s)
Kuberan 2017				Timed Up and Go (s)
Kumaran 2015			Berg Balance Scale (BBS)	Gait velocity (no units)
Kwakkel 2008		Rivermead Mobility Index		Gait velocity (m/s)
Langhammer 2000	Barthel index	Motor Assessment Scale		
Langhammer 2007	Barthel index	Motor Assessment Scale	Berg Balance Scale (BBS)	Gait velocity (m/s)
Lawal 2016	Barthel Index			Gait velocity (m/s)
Li 2005	Barthel index			
Li 2013	Barthel Index	Fugl‐Meyer Assessment (FMA)	Berg Balance Scale (BBS)	Gait velocity (cm/s)
Li Weiwei 2015	Barthel Index			
Lincoln 2003	Barthel index	Rivermead Mobility Index		Gait velocity (m/s)
Liu Xuan 2016	Barthel Index			
Liu Yanhua 2020			Berg Balance Scale (BBS)	Timed Up and Go (s)
Marigold 2005			Berg Balance Scale (BBS)	Timed Up and Go (s)
Mendoza 2015				Gait velocity (m/s)
Meng 2022	Barthel Index			Gait velocity (m/s)
Meng Qingling 2015	Barthel Index	Fugl‐Meyer Assessment (FMA)		
Outermans 2010			Berg Balance Scale (BBS)	Gait velocity (m/s)
Pandian 2014	Barthel Index	Fugl‐Meyer Assessment (FMA)	Berg Balance Scale (BBS)	
Puckree 2014			Berg Balance Scale (BBS)	
Rahayu 2020	Barthel Index		Berg Balance Scale (BBS)	
Richards 1993	Barthel Index	Fugl‐Meyer Assessment (FMA)	Berg Balance Scale (BBS)	Gait velocity (cm/s)
Sekhar 2013			Berg Balance Scale (BBS)	
Shin 2011			Berg Balance Scale (BBS)	
Shuai 2013	Barthel Index	Fugl‐Meyer Assessment (FMA)		
Signal 2014				Gait velocity (m/s)
Thaut 2007				Gait velocity (m/min)
Verma 2011				Gait velocity (m/s)
Wang 2005		Motor Assessment Scale	Berg Balance Scale (BBS)	
Wang Leilei 2020		Fugl‐Meyer Assessment (FMA)		Timed walk over set distance ‐ 10m (s)
Wang Wenwei 2012		Fugl‐Meyer Assessment (FMA)	Berg Balance Scale (BBS)	Gait velocity (cm/s)
Xu Wenyu 2012	Barthel Index			
Yadav 2016			Berg Balance Scale (BBS)	Timed Up and Go (s)
Zhang Huiyu 2021	Barthel Index	Fugl‐Meyer Assessment (FMA)		
**Total studies = 63**	**Studies included in analysis = 29 (see**Analysis 7.1; Analysis 7.7**)**	**Studies included in analysis = 24 (see**Analysis 7.2; Analysis 7.8**)**	**Studies included in analysis = 28 (see**Analysis 7.3; Analysis 7.9**)**	**Studies included in analysis = 37 (see**Analysis 7.4; Analysis 7.10**)**

6MWT: 6‐minute walk test m: metres min: minutes s: seconds

**22:** Summary of numbers of studies reporting outcomes relevant to comparisons in this review

	**IADL immediate**	**IADL persisting**	**Motor function immediate**	**Motor function persisting**	**Balance (Berg) immediate**	**Balance persisting**	**Gait velocity immediate**	**Gait velocity persisting**	**Length of stay**
**Physical rehabilitation versus no physical rehabilitation** **(Total studies = 79)**	52 (of which 50 were Barthel Index)	12 (of which 12 were Barthel Index)	50 (of which 46 were Fugl‐Meyer index)	11 (of which 8 were Fugl‐Meyer index)	9	4	18 (of which 11 were a direct measure of gait speed)	7 (of which 4 were a direct measure of gait speed)	1
**Physical rehabilitation versus attention control** **(Total studies = 13)**	2 (of which 0 were Barthel Index)	‐	5 (of which 2 were Fugl‐Meyer index)	3 (of which 0 were Fugl‐Meyer index)	4	0	9 (of which 7 were a direct measure of gait speed)	5 (of which 3 were a direct measure of gait speed)	1
**Additional physical rehabilitation** **(Total studies = 43)**	21 (of which 21 were Barthel Index)	‐	22 (of which 15 were Fugl‐Meyer index)	3 (of which 0 were Fugl‐Meyer index)	15	2	19 (of which 10 were a direct measure of gait speed)	4 (of which 1 were a direct measure of gait speed)	‐
**Different approaches to physical rehabilitation** **(Total studies = 63)**	29 (of which 27 were Barthel Index)	10 (of which 10 were Barthel index)	24 (of which 18 were Fugl‐Meyer index)	9 (of which 6 were Fugl‐Meyer index)	28	7	37 (of which 27 were a direct measure of gait speed)	12 (of which 10 were a direct measure of gait speed)	1
**Total** (Total studies = 198*)	104	22	101	26	56	13	83	28	3

*194 individual studies were included in the quantitative analysis for this review. 4 of these studies (which had more than 2 trial arms) contributed to more than one comparison category.

**23:** Adverse events ‐ trials providing limited information

**Study name**	**Information reported related to adverse events**
Ain 2022	AE not discussed explicitly, but dropouts shown: CONSORT FLOW CHART of patient participation in the study. 2 participants (1 in each group) dropped out before data was analysed but no reason given other than "Discontinued intervention".
Allison 2007	Not explicitly discussed, just dropout information: 3/7 from additional practice group withdrew citing fatigue (not stated if due to treatment or not, but does state "and this suggests that a more intensive therapy programme may not suit all stroke patients").
Aloraini 2022	No dropouts were reported (confirmed by Fig. 1. Flowchart of participant recruitment). AE were reported: "Adverse events were uncommon with 5 adverse event recorded for the CIMT (constraint‐induced movement therapy) group, and 3 for the control group". No details provided on the AEs or the stage at which they occurred.
Arya 2019	Any reporting of adverse events by the participants was considered. No adverse event was reported by any of the subjects.
Bai 2008^a^	Not explicitly discussed. Dropout information was provided: In the control group, 8 cases lost to follow‐up and 5 cases died. In the rehab group, 4 cases lost to follow‐up and 2 cases died.
Bai 2014	Adverse effects were documented in both groups during the study. No adverse effects were reported in the training group.
Batchelor 2012	AEs not discussed explicitly. Dropouts were reported. Fig 1, participants who just completed baseline and less than 1 monthly falls data available: control (3 deceased), intervention (1 unwell). Participants who did not reach 12‐month follow‐up‐ control: 2 deceased, 1 unwell; intervention: 3 deceased.
Bek 2016	No adverse events relating to the Conductive Education programme were reported during the study.
Behrman 2011^a^	AEs are reported: 149 SAE + 139 multiple falls from total of 408 participants. The frequency of serious adverse events did not differ significantly among the three groups (Table 3). Minor adverse events (mostly falls) were reported by 55.9% of participants, with no significant differences among groups, except that none of the participants in the home‐exercise group reported incidents of dizziness or faintness during exercise (0%), as compared with 7.9% of those in the early locomotor‐training group (P = 0.001) and 5.6% of those in the late locomotor‐training group (P = 0.008). However, participants in the "usual care" group (relevant to this review) received "late locomotor training". AE data relate to after the delivery of this late intervention, with no specific data for the intervention period during which "usual care" was provided.
Brouwer 2018	AEs not discussed explicitly. Dropouts were reported. Fig 1 Baseline: intervention ‐ 1 medical issue other than stroke. 3 months: control ‐ 3 medical issue other than stroke. 9 month: control ‐ 1 medical issue other than stroke. 12 months: control ‐ 1 medical other than stroke. 12.5 months: intervention ‐ 1 medical other than stroke. 15 months: intervention 1 stroke, 1 medical other than stroke.
Candan 2017	AEs not discussed explicitly. Dropouts were reported. Figure 2 2/18 intervention group did not receive baseline treatment: 1 high blood pressure, 1 knee pain.
Chan DY 2006^a^	AEs not discussed explicitly. Dropouts were reported. Motor relearning group: 1/33 re‐admitted to hospital due to medical problem. Conventional therapy group: 1/33 re‐admitted to hospital due to medical problem.
Chan WN 2017^a^	No adverse effect related to the intervention or the assessment was reported.
Cooke 2006	AEs not discussed explicitly. Dropouts were reported. End of treatment period: Conventional physiotherapy (CPT) group (n = 38): 3 unwell dropouts. Additional conventional therapy (CPT+CPT) group (n = 35): 2 unwell, 1 sectioned dropout. At follow‐up: CPT group (n = 31): 5 unwell, 2 housebound, 2 died. CPT+CPT group (n = 32): 5 unwell, 1 sectioned. Functional strength training (FST +CPT) group (n = 36): 5 lost to follow‐up as unwell.
Dalal 2018	AEs not explicitly discussed. Figure 1, Experimental group 1/16 dropped out due to poorly controlled hypertension.
Fan WK 2006	Control group: 2 deaths reported.
Fan L 2014^a^	No AE. Dropouts: "All patients in the observation group completed the intensive training according to the regulations, and 2 patients had mild dizziness symptoms during the training process, and the symptoms were relieved after rest"
Fang 2003	28/78 participants in the Additional early physiotherapy intervention (AEP) group were not able to tolerate a 45‐minute physiotherapy session daily with or without deteriorating illness and were lost to follow‐up at 6 weeks, and a further 102 were lost at 6 months (AEP group: 64; Control: 38).
FeSTivaLS 2014	For adverse reactions it was specifically postulated that paretic limb pain could occur if FST (Functional Strength Training) was provided in a dose (amount in hours) that was too much for a participant. Pain was considered an adverse reaction if the therapist providing FST received a verbal or behavioural report of pain on four consecutive treatment days. Paretic limb pain was included in the monitoring as it was highlighted to the research team that this was a specific clinical concern. Although reported that none of the participants experienced an adverse reaction, 1/27 Functional Strength Training Upper Limb (FST‐UL) were unwell and didn't complete outcome measures. In the Functional Strength Training Lower Limb (FST‐LL) group 3/23 were unwell for outcome measures assessment.
Green 2002	AEs not discussed explicitly. Dropouts were reported. During treatment: 4/85 withdrew (community physiotherapy group), 2/85 died and 3/85 withdrew (no therapy). Between 3 month and 6 month assessments: 3/81 withdrew, 4/81 died (community physiotherapy). 1/80 died and 2/80 withdrew (no therapy). Between 6 month and 9 month assessment: 2/74 withdrew (community physiotherapy), 2/77 died and 1/77 withdrew (control group)
Guan 2017	No serious adverse events occurred. A few patients occasionally experienced mild fatigue after training and recovered after rest.
Holmgren 2006	All assessments were done by blinded staff, who were instructed that if they had any reason to believe that they had revealed a subject’s group they should make an adverse event report. All but one subject completed the entire programme, although 2 subjects dropped out during follow‐up; the reason for dropout was worsening overall medical condition in all three cases. Intervention group (n = 15): lost to 3‐ and 6‐month follow‐ups 1 deceased, 1 discontinued intervention.
Howe 2005^a^	AEs not discussed explicitly. Dropouts were reported: Treatment group (n = 17), 1 developed a brain tumour prior to re‐test at week 4.
Kim 2016	Reported that there were no serious adverse events during the study period.
Koç 2015	No major adverse events occurred during the exercise sessions. Some hospitalisations occurred in both the experimental and usual care groups. Although there were no deaths or cardiac events, there were 12 recurrent strokes.
Kumaran 2015	Figure 4.13 Consort flow diagram: 12 week follow‐up: control group 5/27 lost to follow‐up, experimental group 3/29 lost to follow‐up.
Kunkel 2013	No explicit discussion of AEs. One participant with a urinary tract infection (Exercises Alone) could not attend the week 2 assessment, and three participants did not complete the week 4 assessment: one had suffered a second stroke (Usual Care), one was medically unwell, and one withdrew with no stated reason (both Exercise Alone).
Langhammer 2000	AEs not discussed explicitly. Deaths reported in Table 1: Motor learning (MRP) group (n = 33) 4 deaths at 3‐month data collection, 6 by 1 year, and 12 by 4 year follow‐up. Bobath group (n = 38): 4 deaths by 3 months, 7 by 1 year and 12 by 4 year follow‐up.
Langhammer 2007	AEs not explicitly discussed, nor dropouts, but Table 1a records death/withdrawal information. Intensive exercise group (n = 35): Acute ward/Discharge: 1 dead, 2 withdrawals. Regular exercise group (n = 40): Acute ward/Discharge: 3 dead, 2 withdrawals; at 3 months: 1 dead, 1 withdrawal; at 6 months: 2 dead. Reasons for withdrawals were: new diagnosis 1; anxiety 1; cognitive status/dementia 1; advanced age (98 years) 1; did not want to participate 1.
Lawal 2016	AEs not explicitly discussed. Figure 4.2 study flow diagram. Group C (60 mins circuit training): 1/21 died from diabetic complications. Control group: 1/23 suffered a recurrent stroke.
Lee 2018	Treatment‐emergent adverse events (TEAEs) served as safety outcome measures. The safety set comprised all patients who were enrolled in the current study and received safety analysis after the treatment.
Lincoln 2003	AEs not explicitly discussed. Figure 1 shows dropouts: After 1 month: Bobath 52/60 assessed. Reasons not assessed: 4 ill, 1 refused, 1 died, 1 unable to contact, 1 lost to follow‐up. Movement science based: 47/60 assessed. Reasons not assessed: 5 ill, 3 refused, 4 died, 1 administration error. After 3 months: Bobath 43/52 assessed. Reasons not assessed: 7 ill, 5 refused, 4 died, 1 lost to follow‐up. Movement science based: 42/47 assessed. Reasons not assessed: 5 ill, 2 refused, 8 died, 3 administration error. After 6 months: Bobath 45 assessed (3 returned for assessment) 4 ill, 7 died, 1 lost to follow‐up. Movement science: 42 assessed. 3 ill, 3 refused, 9 died, 1 unable to contact, 3 admin error, 1 moved away.
Lindvall 2014	No adverse events occurred during the intervention. Dropouts reported: Experimental group: 1 dropout pre 9‐week follow‐up (due to illness). Control group; 1 dropout pre 14‐week follow‐up (due to illness).
Martins 2020	There were no adverse events related to the interventions.
McClellan 2004	"no adverse events" but 3 dropouts were reported; 2 from the experimental group (both died) and 1 from the control group (who had another stroke). In addition, there were missing data for 2 subjects at Week 6, 1 subject from the experimental group (who failed to attend) and 1 from the control group (who was unable to attend due to a sprained ankle).
Meier 2021	There were no adverse events associated with this trial.
Mendoza 2015	The participants did not report any adverse effects in relation to either of the treatment programmes.
Meng 2022	"No adverse effects were observed during or after training." 5 drop outs were reported in the text and in Figure 1 (flow diagram) during the intervention stage; 2 in the Robot‐assisted gait training (not relevant to this review) (RAGT) group and 3 in the Conventional rehabilitation (CRT group). All were reported as being due to personal reasons.
Moore 2016	No dropouts or adverse events were reported.
Mustafaoğlu 2018	No adverse event was observed during the study.
Nindorera 2022	No dropouts were reported (confirmed by Figure 1. Consort diagram of eligibility and participant inclusion). Minor adverse effects were reported in 11% of participants during CBCS rehabilitation training, all of which resolved within short periods of time (minutes, hours, or days). Two participants experienced low back pain, 2 reported knee pain after walking, and 1 reported hypotension and dizziness during the second session. No report of the number or/% of events occurring each group (it was a cross‐over trial and both groups received the intervention).
Outermans 2010	"In neither group any adverse events occurred during the trial." However, health‐related dropouts were reported: During the programme, 1 participant dropped out of the control group (n = 21) due to acute gonarthritis.
Pandian 2014	The study participants reported no adverse event during the course of trial; however, a few experimental subjects experienced mild post‐exercise muscle soreness. Appropriate rest was recommended for those subjects.
Pang 2006	2/39 participants withdrew from control group.
Pang 2018	No adverse events were reported during the training period. Lost to follow‐up during intervention: Dual task group (n = 28): 1 illness, 1 fatigue; Single‐task group (n = 28): 1 illness, 1 fatigue. Control group (28): 1 illness
Park 2021	AE not discussed explicitly, but dropouts shown: Figure 1 flowchart of patient participation in the study. Not clear when patients dropped out but Figure 1 implies it was during treatment to 4 weeks post treatment period. Indirect cross‐training group 1/20 had health problem. Remaining dropouts were due to personal reasons/not specified.
Rahayu 2020	Control group: 1/33 intervention stopped (moved to HCU).
Renner 2016	Individual task training group (n = 39) ‐ 3 dropouts: too many therapies. Group task training (n = 34) ‐ 5 dropouts: too many therapies; hip pain.
Salbach 2004	AE discussed and separate dropouts shown in Figure 1. Dropouts: Motor learning group: 1/44 groin pain. Throughout the study, a total of 1638 mobility sessions were conducted, and 4 subjects experienced a fall. These individuals did not suffer an injury and were able to continue their participation. 2 additional falls occurred during evaluations, but the subjects were able to continue with testing. Placebo (upper limb control) group: 3/47 discontinued intervention due to illness/event (1 myocardial infarction, 1 prostate cancer metastases, 1 fell and fractured rib at home). 1 additional participant had foot pain which prevented mobility testing at follow‐up assessment.
Severinsen 2014^a^	During follow‐up 1/13 Aerobic Training (AT) group experienced a minor cerebral stroke without hospitalisation. One of the 5 dropouts was study‐related, caused by pain from a former knee replacement (AT group n=13). Other dropouts were due to reduced attendance (lack of motivation, kidney disease, alcoholism, epilepsy).
SunRISe 2021	No adverse events related to physical activity were noted during the study.
Teixeira‐Salmela 1999	The results of the assessment did not reveal any adverse changes associated with the training programme.
Thaut 2007	AEs not discussed explicitly. Dropouts were reported. The dropout rate in one centre was 23% of initially included patients. There was a 10% dropout rate in the transfer, early discharge, medical complication, or unspecified personal reasons at the other centre.
Torres‐Arreola 2009	Sixty‐seven patients (61%) completed the study, 5 died due to stroke complications, and 38 were lost during the follow‐up because of other reasons. At 1 month follow‐up: Strategy 1: 11/59 dropped out, Strategy 2: 10/51 dropped out (no reasons given). At 3‐month follow‐up: Strategy 1: 9/48 dropped out, 4/48 died, Strategy 2: 1/41 died, 3/41 dropped out. At 6‐month follow‐up Strategy 1: 3/35 dropped out, Strategy2: 2/37 dropped out.
Tyson 2015	No serious adverse events were reported. There were 8 reports of short‐lived upper‐ and lower‐limb aches or limb tightness (group not reported).
Vahlberg 2017	"No adverse events occurred during the exercise sessions". Dropouts: Intervention group (n = 34): After allocation to group: Medical condition 2. After 6 months, 1 due to acute disease. No AE or SAE reported.
Verma 2011	"no adverse effects were reported." Dropouts: 1/15 in the experimental group was lost for a follow‐up assessment due to a second stroke.
Wade 1992	By first follow‐up 2/45 had died in late treatment group.
Wang 2004a	Dropouts due to financial reasons or inability to adhere to study design.
Wang 2015	Home‐based rehabilitation programmes were observed to reduce initial hospitalisation significantly and had no adverse effect on mortality or the number of falls. The therapist also enquired about any experience of adverse events and planned new weekly activities accordingly. No report of any SAE/AE.
Wang 2021	We asked patients to report any unintended harms and severe adverse events directly during hospitalisation or follow‐up. Severe adverse events such as recurrence of stroke or death were recorded in detail by the nurse and reported to the data monitoring committee. 1 SAE (death) by 12 weeks follow‐up (group not reported). 4/108 (experimental group) dropped out as their "disease worsened". This exacerbation was not related to the intervention.
Werner 1996^a^	AEs not discussed explicitly. Dropouts were reported: Treatment group: 4/33 dropped out by 3‐month follow‐up for medical reasons (pneumonia, fractured hip, ulcers, recurrent stroke, or SIP score); 4/29 dropped out between 3‐month and 9‐month follow‐up due to medical reasons (2 further strokes, 2 died). Control group: 4/16 dropped out (not stated when) for medical reasons.
Wu 2006	Dropouts reported: Group 1 (Rehabilitation group): 2/50; Group 2 (Control): 2/50. Dropouts attributed to 3 deaths and 1 failure to attend assessment.
Wu 2020	AEs not discussed explicitly. Dropouts were reported. Intervention group: 2/32 (serious illness, death). Control group: 1/32 (disease progression)
Xie 2005	Dropouts due to death at Timepoint 1 (immediately after treatment had ended): Group 1 (Rehabilitation): 3/35 ; Group 2 (Control) 1/35.
Yazici 2021^a^	Dropouts: Neurodevelopmental‐Bobath approach (NDT‐B) group: 2/23 discontinued intervention (no reason reported); Standard rehabilitation group: 0 dropped out.
Yelnik 2008^a^	In the NDT‐based group (n = 35), 1 patient had to stop after 5 sessions of physical therapy for carotid surgery, which had not been planned. He could not be assessed. In the multisensorial group (n = 33), 1 patient was lost to follow‐up between D30 and D90 and another because of an adverse event unrelated to the treatment.
Zang 2013	AE not discussed explicitly, but dropouts reported: 1 patient in control group (n = 50) had recurrence of cerebral infarction.
Zhang 2004	19 deaths and 157 dropouts.

AE: Adverse event CBCS: circuit walking, balance, cycling and strength HCU: high care unit NDT: neurodevelopmental‐theory‐based treatment SAE: serious adverse event ^a^ These studies were not included in quantitative analyses (see Table for reasons).

#### Studies included in qualitative synthesis only

Of the included studies, 73 of 267 were included in qualitative synthesis only. These are summarised in Table. The main reasons for not being included in quantitative synthesis were that no relevant outcomes were measured and/or reported (n = 25), data were reported in a way that was not suitable for inclusion in meta‐analyses (n = 30), and the study comparisons did not fit within our review comparisons (n = 11).

**24:** Studies included in qualitative synthesis only

**Study**	**Intervention 1**	**Intervention 2**	**Reason not included in quantitative analysis**
**Physical rehabilitation versus no physical rehabilitation**			
ACTIV 2021	Home‐based rehabilitation	Usual care (no rehabilitation)	No relevant outcomes measured/reported
Bai 2008	Three‐stage rehabilitation	No physical rehabilitation	Data reported in a way that was not suitable for analysis
Bai 2014	Three stage rehabilitation	Usual care (no rehabilitation)	Data reported in a way that was not suitable for analysis
Capisizu 2016	Cerebrolysin	Cerebrolysin + physical rehabilitation	Data reported in a way that was not suitable for analysis
Carlson 2006	Rehabilitation	No intervention	No relevant outcomes measured/reported
Chan WN 2017	Conventional exercise	No training	No relevant outcomes measured/reported
Chen Y 2011	Rehabilitation	No physical rehabilitation	Data reported in a way that was not suitable for analysis
Fan Y 2015	Three‐stage rehabilitation	Usual care (no rehabilitation)	Data reported in a way that was not suitable for analysis
Ge W 2003	Rehabilitation	No physical rehabilitation	Data reported in a way that was not suitable for analysis
Huang Yangfang 2016	Three‐stage rehabilitation	No physical rehabilitation	No relevant outcomes measured/reported
Lu 2014	Three‐stage rehabilitation	Usual care (no rehabilitation)	No relevant outcomes measured/reported
Pang 2003	Rehabilitation	No physical rehabilitation	Data reported in a way that was not suitable for analysis
Pirayesh 2021	Otago exercises	No treatment	No relevant outcomes measured/reported
Stephenson 2004	PNF	No physical rehabilitation	Data reported in a way that was not suitable for analysis
Sun Juanjuan 2014	Rehabilitation	No physical rehabilitation	Data reported in a way that was not suitable for analysis
Tang Yao 2015	Three‐stage rehabilitation	No physical rehabilitation	No relevant outcomes measured/reported
Vahlberg 2017	Progressive resistance and balance	No treatment	Data reported in a way that was not suitable for analysis
Werner 1996	Outpatient rehabilitation	No treatment	Data reported in a way that was not suitable for analysis
Xiao Yuhua 2015	Three‐stage rehabilitation	Usual care (no rehabilitation)	Data reported in a way that was not suitable for analysis
Xie 2005	Rehabilitation	No physical rehabilitation	No relevant outcomes measured/reported
Yang Zhihong 2015	Three‐stage rehabilitation	Usual care (no rehabilitation)	Data reported in a way that was not suitable for analysis
Zhang Lifang 2015	Balance training	Routine rehabilitation	Serious concerns about data, so not included in analyses
Zhao Haihong 2013	Rehabilitation	Usual care (no rehabilitation)	No relevant outcomes measured/reported
Zhu 2004b	Rehabilitation	No physical rehabilitation	No relevant outcomes measured/reported
**Physical rehabilitation versus attention control**			
Pang 2018	Single task training (motor relearning)	UL exercise	Data reported in a way that was not suitable for analysis
Qian 2004	Rehabilitation	No physical rehabilitation	No relevant outcomes measured/reported
Severinsen 2014	Resistance training	Sham training	Data reported in a way that was not suitable for analysis
Stuart 2019	Adaptive physical activity	Sittercise	Data reported in a way that was not suitable for analysis
Tyson 2015	Upper limb mirror therapy	Lower limb exercise	Data reported in a way that was not suitable for analysis
**Additional rehabilitation**			
Allison 2007	Standing practice	Conventional rehabilitation	Data reported in a way that was not suitable for analysis
Behrman 2011	Locomotor training programme	Home‐based program	Data reported in a way that was not suitable for analysis
Fan L 2014	Task specific training	Conventional rehabilitation	No relevant outcomes measured/reported
Fan X 2009	Exercise training	Conventional rehabilitation	Unable to obtain full paper, so abstract only available, which did not report relevant outcome data.
Ghasemi 2018	Functional stretch	Control	Data reported in a way that was not suitable for analysis
Hong Hye Jin 2012	Three‐stage rehabilitation	Traditional rehabilitation nursing care	No relevant outcomes measured/reported
Howe 2005	Additional therapy	Usual care	No relevant outcomes measured/reported
Imhof 2015	Mobility‐enhancing nursing intervention	Conventional rehabilitation	No separate stroke survivor data
Indurkar 2013	Task oriented activities	Conventional rehabilitation	Data reported in a way that was not suitable for analysis
Li Yuanzheng 2014a	Motor re‐learning	Conventional rehabilitation	Serious concerns about data, so not included in analyses
Medina‐Rincón 2019	Balance rehabilitation programme	Conventional rehabilitation	No relevant outcomes measured/reported
Mohaideen 2014	Symmetrical weight training	Conventional rehabilitation	No relevant outcomes measured/reported
Nagy 2017	Conductive education	Conventional rehabilitation	Data reported in a way that was not suitable for analysis
Xiao 2003	Intensive rehabilitation	Conventional rehabilitation	No relevant outcomes measured/reported
**Comparison of different approaches**			
Ain 2022	Functional training	Conventional therapy	Data reported in a way that was not suitable for analysis
Chan DY 2006	Motor relearning	Skills training	Comparison of similar approaches
Chen L 2019	Motor relearning	Bobath	No relevant outcomes measured/reported
Gong Y 2009	Bilateral training	Unilateral training	Data reported in a way that was not suitable for analysis
Jeon 2018	Unilateral training	Bilateral training	Comparison of similar approaches
Jing 2006	Exercise + OT	Exercise	Comparison of similar approaches
Jongbloed 1989	Sensorimotor training	Functional training	Data reported in a way that was not suitable for analysis
Khallaf 2014	Task specific exercise	Traditional physiotherapy	No relevant outcomes measured/reported
Kim 2017	Circuit training	Conventional	Comparison of similar approaches
Krawczyk 2014	Closed chain exercise	Open chain exercise	Comparison of similar approaches
Krukowska 2016	NDT‐Bobath	PNF	No relevant outcomes measured/reported
Lennon 2006	Bobath	Bobath with walking training	Comparison of similar approaches
Mansfield 2018	Perturbation‐based balance training	Exercise	Data reported in a way that was not suitable for analysis
Meier 2021	Co‐ordinative training (Doris‐Broetz Concept)	Conventional physiotherapy	Insufficient information to categorise interventions
Mikolajewska 2017	Bobath + traditional	Traditional	Data reported in a way that was not suitable for analysis
Renner 2016	Group task training	Individual task training	Comparison of similar approaches
Seo 2015	Ramp gait exercise with PNF	PNF gait pattern training	Comparison of similar approaches
SPIRES 2022	Standing frame programme	Conventional	Data reported in a way that was not suitable for analysis
Wang 2021	Rehabilitation nursing	Therapist intervention	Comparison of similar approaches
Yazici 2021	Bobath	Standard	Data reported in a way that was not suitable for analysis
Yelnik 2008	Neurodevelopmental technique (NDT)	Multisensory	Data reported in a way that was not suitable for analysis
Zhong Qiue 2014	Bilateral limb training	Conventional rehabilitation	Comparison of similar approaches
**Multi‐group trials**			
Aksu 2001	Bobath (3 groups)		No relevant outcomes measured/reported
Baer 2007	Walking practice (part)/walking practice (whole)	Information	No relevant outcomes measured/reported
Danlami 2017	CIMT, delivered with 2 different timings	Conventional rehabilitation	No relevant outcomes measured/reported
Kwakkel 2002	Rehabilitation	Attention control/No physical rehabilitation	No relevant outcomes measured/reported
Morreale 2016	PNF (early)/PNF (standard)	Cognitive therapeutic exercise (early rehab)	Comparisons do not fit within analysis comparisons (comparisons of different approaches)
**Unable to categorise**			
Liu 2014	Task oriented training	Conventional rehabilitation	Unable to categorise intervention for incorporation in review comparisons
Matthew Hall 2013	Task‐oriented training	Standard care	Unable to categorise intervention for incorporation in review comparisons
Wei 2014	Three‐stage rehabilitation (all stages)	Three‐stage rehabilitation (1st and 2nd stages only)	Unable to categorise intervention for incorporation in review comparisons

CIMT= constraint induced movement therapy; PNF = proprioceptive neuromuscular facilitation; NDT = neurodevelopmental technique; UL= upper limb

#### Excluded studies

A total of 49 studies were excluded at the full‐text screening stage (Figure); reasons for exclusion are listed in Characteristics of excluded studies. The majority (30/49) were excluded because they focussed on a trunk training intervention, which is covered by a Cochrane review of trunk training and is not included in this review. A further 4/49 were excluded as they explored interventions covered in other Cochrane reviews, and were therefore not eligible for inclusion; 7/49 were excluded as the intervention did not meet the inclusion criteria. We excluded 2/49 that did not include a relevant randomised comparison, and 6/49 had been listed in previous versions of this review as awaiting assessment, but there was still insufficient information to support a decision and no new information was expected.

### Risk of bias in included studies

We performed risk of bias assessments using the RoB 2 tool for studies included in meta‐analyses for primary outcomes (independence in ADL and motor function) at both immediate and persisting time points, and for secondary outcomes (balance, gait velocity, and length of stay) at the immediate time point only. The risk of bias assessments are visually presented within the forest plots with traffic lights. RoB 2 assessment data are available on request from the authors.

For the comparison of 'Physical rehabilitation versus no physical rehabilitation':

- 52 studies had data on **independence in ADL** at an immediate time point; of these, four were judged to be at low risk; 23 were judged to have some concerns; and 25 were judged to be at high risk of bias.
- 50 studies had data on **motor function** at an immediate time point; of these, only three were judged to be at low risk; 24 were judged to have some concerns; and 23 were judged to be at high risk of bias.

For the comparison 'Physical rehabilitation versus attention control':

- Two studies had data on **independence in ADL** at an immediate time point; one study was judged to be low risk of bias for all domains; and one study was judged to have an overall high risk of bias.
- Five studies had data on **motor function** at an immediate time point; of these, one was judged to have low risk for all domains; two were judged to have some concerns relating to overall risk of bias; and two were judged to have an overall high risk of bias.

Across the two primary outcomes for comparisons 'Physical rehabilitation versus no physical rehabilitation' and 'Physical rehabilitation versus attention control' the majority of high risk of bias judgements were related to concerns about potential deviations from intended interventions, often with concerns about potential for contamination between groups (with no treatment/control group participants receiving the intervention). There were also high risk of bias judgements for a number of studies due to inadequate description of the randomisation process and/or concerns about imbalance between groups, and uncertainty about whether the outcome assessor was blinded, with particular concerns when it appeared that study authors were assessing outcomes. Very few studies had a protocol available, resulting in some concerns about selection of the reported result for a majority of the studies. Few studies reported sufficient information relating to methods of randomisation and allocation concealment or measurement of the outcome, resulting in frequent judgements of some concerns. We judged most studies to be at low risk of bias for missing outcome data, but we judged a small number to have some concerns or high risk of bias due to imbalances between groups and/or unexplained missing outcome data for a relatively high number of participants.

For the comparison 'Additional physical rehabilitation plus usual therapy versus usual therapy alone':

- 21 studies had data on **independence in ADL** at an immediate time point; of these, none were judged to have low risk for all domains; 13 were judged to have some concerns relating to overall risk of bias; and eight were judged to have an overall high risk of bias.
- 22 studies had data on **motor function** at an immediate time point; of these, only one was judged to have low risk for all domains; 11 were judged to have some concerns relating to overall risk of bias; and 10 were judged to have an overall high risk of bias.

Inadequate reporting of randomisation, allocation concealment, and blinding of outcome assessor, and the absence of a study protocol were common across studies within these comparisons, resulting in the majority of studies being judged to have some concerns. Judgement of high risk of bias was most commonly due to concerns about lack of blinding of outcome assessors. As above, we judged most studies to be at low risk of bias for missing outcome data.

For the comparison 'Physical rehabilitation with a focus on functional task training versus other approaches with less/no focus on functional task training':

- 22 studies had data relating to **independence in ADL** at an immediate time point; of these, only one was judged to have low risk for all domains; 16 were judged to have some concerns relating to overall risk of bias; and five were judged to have an overall high risk of bias.
- 20 studies reported data on **motor function** at an immediate time point; of these only one was judged to have low risk for all domains; 13 were judged to have some concerns relating to overall risk of bias; and six were judged to have an overall high risk of bias.

For the comparison of 'Physical rehabilitation with a focus on neurophysiological treatment components versus other approaches':

- 14 studies had data on **independence in ADL** at an immediate time point; of these, none were judged to have low risk for all domains; 12 were judged to have some concerns relating to overall risk of bias; and two were judged to have an overall high risk of bias.
- 13 studies had data on **motor function** at an immediate time point; of these, none were judged to have low risk for all domains; 10 were judged to have some concerns relating to overall risk of bias; and three were judged to have an overall high risk of bias.

Across the two primary outcomes for the aforementioned comparisons, inadequate reporting of randomisation, allocation concealment, and blinding of outcome assessor and the absence of a study protocol were common, resulting in the majority of studies being judged to have some concerns. However, we judged a number of these studies to clearly report having a blinded outcome assessor, resulting in a judgement of low risk of bias for measurement of the outcome. As above, we judged most studies to be at low risk of bias for missing outcome data. Reasons for high risk of bias judgements varied across these studies.

Detailed risk of bias assessment data with consensus responses to the signalling questions in the RoB 2 Excel tool are available on reasonable request.

#### Non‐reporting bias

Table summarises the number of studies with different comparisons that report outcomes relevant to this review. Only 42% (114/272) of studies report our primary outcome of independence in ADL and 41% (111/272) our primary outcome of motor function. Balance, gait velocity, and length of stay were reported by 23% (62/272), 33% (89/272), and 0.7% (2/272) respectively. Funnel plots for primary outcomes of independence in ADL and motor function for comparisons of physical rehabilitation versus no physical rehabilitation and additional physical rehabilitation are provided in Figure; Figure; Figure; and Figure. Visual inspection of these funnel plots demonstrates asymmetry (particularly for the comparison of physical rehabilitation versus no physical rehabilitation) (Figure; Figure), suggesting that some small studies report larger effect sizes.

**3:**
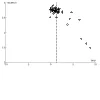
Comparison: Physical rehabilitation versus no physical rehabilitation

**4:**
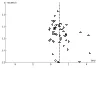
Comparison: Physical rehabilitation versus no physical rehabilitation

**5:**
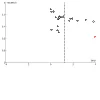
Comparison: Additional therapy + usual therapy versus usual therapy

**6:**
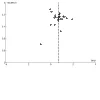
Comparison: Additional therapy + usual therapy versus usual therapy

### Effects of interventions

See: **Summary of findings 1** Physical rehabilitation versus no physical rehabilitation ‐ immediate outcomes; **Summary of findings 2** Physical rehabilitation versus attention control ‐ immediate outcomes; **Summary of findings 3** Additional physical rehabilitation plus usual therapy versus usual therapy only ‐ immediate outcomes; **Summary of findings 4** Physical rehabilitation with a focus on functional task training versus another approach ‐ immediate outcomes; **Summary of findings 5** Physical rehabilitation with a focus on neurophysiological treatment versus another approach ‐ immediate outcomes

#### Comparison 1. Physical rehabilitation versus no physical rehabilitation

Seventy‐nine studies provided data on 83 comparisons of physical rehabilitation versus no physical rehabilitation. See Table.

##### Primary outcomes

###### Independence in ADL scales

Physical rehabilitation was more effective than no physical rehabilitation for immediate outcome (SMD 1.32, 95% CI 1.08 to 1.56; I² = 93%; 52 studies, 5403 participants; low‐certainty; Analysis 1.1).

Sensitivity analysis, removing studies with high risk of bias (n = 25) or considerable uncertainty (n = 16), did not change this conclusion (SMD 0.60, 95% CI 0.25 to 0.94; I² = 84%; 11 studies, 876 participants) (Table).

Evidence suggests that this benefit may be sustained long‐term (i.e. at a follow‐up time point a period of time after the intervention has stopped) (Analysis 2.1; Table).

**25:** Summary of long‐term effects (persisting time points)

**Outcome**	**Immediate effect** **Effect size (95% CI)** **No. of participants (studies)**	**Persisting effect** **Effect size (95% CI)** **No. of participants (studies)**	**Comment**
**Physical rehabilitation versus no physical rehabilitation**			
**Primary outcome: Independence in ADL scales**	SMD 1.32 (1.08 to 1.56) 5403 (52) Analysis 1.1	SMD 0.52 (0.17 to 0.88) 757 (12) Analysis 2.1	Evidence suggests that the beneficial effect of physical rehabilitation, when compared to no physical rehabilitation, may be sustained long‐term (i.e. effect on ADL may persist).
**Primary outcome: Motor function scales**	SMD 1.01 (0.80 to 1.22) 5669 (50) Analysis 1.2	SMD 0.50 (0.22 to 0.78) 1967 (11) Analysis 2.2	Evidence suggests that the beneficial effect of physical rehabilitation, when compared to no physical rehabilitation, may be sustained long‐term (i.e. effect on motor function may persist).
**Secondary outcome: Balance (Berg Balance Scale)**	MD 4.54 (1.36 to 7.72) 452 (9) Analysis 1.3	MD 0.58 (‐1.71 to 2.87) 239 (4) Analysis 2.3	Very limited evidence suggests that the beneficial effect of physical rehabilitation, when compared to no physical rehabilitation, may not be sustained long‐term (i.e. effect on balance may not persist).
**Secondary outcome: Gait velocity**	SMD 0.23 (0.05 to 0.42) 1131 (18) Analysis 1.4	SMD 0.01 (‐0.18 to 0.20) 509 (7) Analysis 2.4	Very limited evidence suggests that the beneficial effect of physical rehabilitation, when compared to no physical rehabilitation, may not be sustained long‐term (i.e. effect on gait velocity may not persist).
**Secondary outcome: Adverse events**	RR 8.70 (2.90 to 26.06) 283 (5) Analysis 1.6	Not estimable 41 (1) Analysis 2.5	There is insufficient evidence to support a conclusion about the long‐term effects of physical rehabilitation, when compared to no physical rehabilitation, on adverse events.
**Physical rehabilitation versus attention control**			
**Primary outcome: Independence in ADL scales**	SMD 0.91 (0.06 to 1.75) 106 (2) Analysis 3.1	Not reported	There is no evidence to support a conclusion about the long‐term effects of physical rehabilitation, when compared to attention control, on independence in ADL.
**Primary outcome: Motor function scales**	SMD 0.13 (‐0.13 to 0.38) 237 (5) Analysis 3.2	SMD ‐0.10 (‐0.46 to 0.26) 119 (3) Analysis 4.2	Only 3 studies provide follow‐up data, providing insufficient evidence to support a conclusion about the long‐term effects of physical rehabilitation, when compared to attention control, on motor function.
**Secondary outcome: Balance (Berg Balance Scale)**	MD 6.61 (‐0.45 to 13.66) 240 (4) Analysis 3.3	Not reported	There is no evidence to support a conclusion about the long‐term effects of physical rehabilitation, when compared to attention control, on balance.
**Secondary outcome: Gait velocity**	SMD 0.27 (‐0.01 to 0.55) 474 (9) Analysis 3.4	SMD 0.26 (‐0.05 to 0.58) 156 (5) Analysis 4.4	Very limited evidence suggests that the effect on direct measures of gait speed observed at immediate time point might not be sustained at the longer‐term follow‐up.
**Secondary outcome: Adverse events**	RR 1.92 (0.75 to 4.93) 290 (3) Analysis 3.6	Not reported	There is no evidence to support a conclusion about the long‐term effects of physical rehabilitation, when compared to attention control, on adverse events.
**Additional physical rehabilitation versus usual rehabilitation only**			
**Primary outcome: Independence in ADL scales**	SMD 1.26 (0.82 to 1.71) 1972 (21) Analysis 5.1	Not reported	There is insufficient evidence to support a conclusion about the long‐term effects of additional physical rehabilitation on independence in ADL.
**Primary outcome: Motor function scales**	SMD 0.69 (0.46 to 0.92) 1965 (22) Analysis 5.2	SMD 0.12 (‐0.60 to 0.83) 103 (3) Analysis 6.2	Only 3 studies provide follow‐up data, providing insufficient evidence to support a conclusion about the long‐term effects of additional physical rehabilitation on motor function.
**Secondary outcome: Balance (Berg Balance Scale)**	MD 5.74 (3.78 to 7.71) 795 (15) Analysis 5.3	MD ‐2.30 (‐15.41 to 10.81) 56 (2) Analysis 6.3	Only 2 studies provide follow‐up data, providing insufficient evidence to support a conclusion about the long‐term effects of additional physical rehabilitation on balance.
**Secondary outcome: Gait velocity**	SMD 0.59 (0.26 to 0.91) 1004 (19) Analysis 5.4	SMD 0.20 (‐0.12 to 0.52) 159 (4) Analysis 6.4	Only 4 studies provide follow‐up data, providing insufficient evidence to support a conclusion about the long‐term effects of additional physical rehabilitation on gait velocity.
**Secondary outcome: Adverse events**	RR 0.80 (0.64 to 0.98) 702 (4) Analysis 5.5	Not reported	There is insufficient evidence to support a conclusion about the long‐term effects of additional physical rehabilitation on adverse events.
**One physical rehabilitation approach versus another physical rehabilitation approach** **(i) Functional task training versus other approaches**			
**Primary outcome: Independence in ADL scales**	SMD 0.58 (0.29 to 0.87) 1535 (22) Analysis 7.1	SMD 1.20 (0.22 to 2.18) 631 (8) Analysis 8.1	Evidence suggests that the beneficial effect of functional task training, when compared to other approaches, may be sustained long‐term (i.e. effect on ADL may persist).
**Primary outcome: Motor function scales**	SMD 0.72 (0.21 to 1.22) 1671 (20) Analysis 7.2	SMD 1.20 (0.22 to 2.18) 631 (8) Analysis 8.2	Evidence suggests that the beneficial effect of functional task training, when compared to other approaches, may be sustained long‐term (i.e. effect on motor function may persist).
**Secondary outcome: Balance (Berg Balance Scale)**	MD 2.44 (0.06 to 4.81) 1194 (15) Analysis 7.3	MD 0.44 (‐6.27 to 7.14) 541 (7) Analysis 8.3	Evidence suggests that the beneficial effect of functional task training, when compared to other approaches, may not be sustained long‐term (i.e. effect on balance may not persist).
**Secondary outcome: Gait velocity**	SMD 0.28 (‐0.01 to 0.56) 1194 (15) Analysis 7.4	SMD 0.33 (‐0.01 to 0.66) 893 (11) Analysis 8.4	Evidence suggests that the lack of effect of functional task training, as compared to other approaches, is seen at follow‐up as well as immediate time points for measures of gait velocity.
**Secondary outcome: Adverse events**	RR 1.33 (0.91 to 1.94) 473 (6) Analysis 7.6	RR 0.33 (0.01 to 7.68) 36 (1) Analysis 8.5	Only 1 study provides follow‐up data, providing insufficient evidence to support a conclusion about the long‐term effects of functional task training, as compared to other approaches, on long‐term adverse events.
**One physical rehabilitation approach versus another physical rehabilitation approach** **(ii) Neurophysiological approaches versus other approaches**			
**Primary outcome: Independence in ADL scales**	SMD ‐0.34 (‐0.63 to ‐0.06) 737 (14) Analysis 7.7	SMD ‐0.53 (‐1.01 to ‐0.06) 142 (4) Analysis 8.6	Evidence suggests that the less effectiveness of neurophysiological approaches, when compared to other approaches, may be sustained long‐term (i.e. negative effect on ADL may persist).
**Primary outcome: Motor function scales**	SMD ‐0.60 (‐1.32 to 0.12) 663 (13) Analysis 7.8	SMD 0.37 (‐0.64 to 1.38) 128 (2) Analysis 8.7	Evidence suggests that the lack of difference between neurophysiological approaches and other approaches is seen at follow‐up as well as immediate time points, for measures of motor function.
**Secondary outcome: Balance (Berg Balance Scale)**	MD 0.06 (‐5.90 to 6.03) 292 (9) Analysis 7.9	MD 10.63 (8.53 to 12.73) 80 (1) Analysis 8.8	There is insufficient evidence to support a conclusion about the long‐term effects of neurophysiological approaches, as compared to other approaches, on balance.
**Secondary outcome: Gait velocity**	SMD ‐0.17 (‐0.62 to 0.27) 630 (16) Analysis 7.10	SMD ‐0.27 (‐1.41 to 0.87) 124 (3) Analysis 8.9	Evidence suggests that the lack of difference between neurophysiological approaches and other approaches is seen at follow‐up as well as immediate time points, for measures of gait velocity.
**Secondary outcome: Adverse events**	Not estimable 40 (1) Analysis 7.11	Not reported	There is insufficient evidence to support a conclusion about the long‐term effects of neurophysiological approaches, as compared to other approaches, on adverse events.

ADL = activities of daily living; CI = confidence interval; MD = mean difference; RR = risk ratio; SMD = standardised mean difference

###### Motor function scales

Physical rehabilitation was more effective than no physical rehabilitation for immediate outcome (SMD 1.01, 95% CI 0.80 to 1.22; I² = 92%; 50 studies, 5669 participants; low‐certainty; Analysis 1.2).

Sensitivity analysis, removing studies with high risk of bias (n = 23) or considerable uncertainty (n = 15), did not change this conclusion (SMD 0.87, 95% CI 0.41 to 1.33; I² = 91%; 12 studies, 981 participants) (Table).

Evidence suggests that this benefit may be sustained long‐term (Analysis 2.2; Table).

##### Secondary outcomes

- Balance (Berg Balance Scale):
  - Physical rehabilitation was more effective than no physical rehabilitation for immediate outcome (MD 4.54, 95% CI 1.36 to 7.72; I² = 91%; 9 studies, 452 participants; low‐certainty; Analysis 1.3).
  - This benefit was not sustained long‐term (Analysis 2.3; Table).
- Gait velocity:
  - Physical rehabilitation was more effective than no physical rehabilitation for immediate outcome (SMD 0.23, 95% CI 0.05 to 0.42; I² = 53%; 18 studies, 1131 participants; moderate‐certainty; Analysis 1.4).
  - This benefit was not sustained long‐term (Analysis 2.4; Table).
- Length of stay: Only one study (110 participants) assessed length of stay, providing very low‐certainty evidence (Analysis 1.5)

###### Adverse events

Five studies actively monitored and reported adverse events (ACTIV 2021; Aravind 2022; ReTrain 2018; Signal 2014; Wang 2022; Table). The reported data suggest that there may be an increase in the risk of adverse events for those undergoing physical rehabilitation (RR 8.70, 95% CI 2.90 to 26.06; I² = 45%; 5 studies, 283 participants; very low‐certainty; Analysis 1.6). The majority of adverse events come from one study (ReTrain 2018); this study reported "related, probably related and possible related adverse events and serious adverse events", while other studies reported serious adverse events only. Further, ACTIV 2021 and ReTrain 2018 both reported the number of adverse events, rather than the number of participants experiencing an adverse event. This evidence, from only five studies, is insufficient to support generalised conclusions.

One study also reported longer‐term follow‐up data on adverse events, reporting the total number of events that were related, probably related, or possibly related to the intervention (ReTrain 2018). This provides very low‐certainty evidence (Analysis 2.5).

##### Subgroup analyses

The results of subgroup analyses are provided in Table, and in Analysis 9 (independence in ADL) and Analysis 10 (motor function). These indicated that there were differences between subgroups, based on the following:

**26:** Subgroup analyses: Physical rehabilitation versus no physical rehabilitation ‐ primary outcomes

**Outcome**	**Subgroup comparison** **Analysis**	**Subgroup 1**	**Studies (participants)**	**Subgroup 2**	**Studies (participants)**	**Test for subgroup differences**	**Comment**
Independence in ADL scales	Time post stroke Analysis 9.1	Early (2 weeks or less post‐stroke)	16 (1463)	Later (more than 2 weeks post stroke)	13 (1057)	Chi² = 0.97, df = 1 (P = 0.32), I² = 0%	No difference between subgroups
	Geographical location ‐ continent of study conduct Analysis 9.2	Asia	46 (4933)	All other continents	6 (438)	Chi² = 4.92, df = 1 (P = 0.03), I² = 79.7%	Greater treatment effect in studies conducted in Asia
	Amount of treatment (less or more than 2.5 hours/week) Analysis 9.3	Less than 2.5 hours/week	7 (603)	More than 2.5 hours/week	41 (3589)	Chi² = 5.86, df = 1 (P = 0.02), I² = 82.9%	Smaller treatment effect when treatment was less than 2.5 hours/ week
	Duration of intervention Analysis 9.4	Short (≤ 2 months)	29 (2851)	Longer (> 2 months)	21 (2356)	Chi² = 1.05, df = 1 (P = 0.31), I² = 4.4%	No difference between groups with less or more than 2 months of treatment.
	Focus of treatment components Analysis 9.5	Functional task training	19 (1592)	Mixed treatment components (combining functional task training, musculoskeletal and neurophysiological components)	28 (3342)	Chi² = 0.17, df = 1 (P = 0.68), I² = 0%	No difference between subgroups
	Named approaches Analysis 9.6	Named as "three‐stage approach"	11 (1592)	Named as "Bobath" approach	12 (1120)	Including 'other' trials (30 trials, 2691 participants): Chi² = 2.21, df = 2 (P = 0.33), I² = 9.5% Comparing only the "three‐stage" and "Bobath" groups: Chi² = 0.04, df = 1 (P = 0.84), I² = 0%	No difference between subgroups
	Provider of intervention Analysis 9.7	Medical/therapeutic staff Medical/therapeutic staff + family/carers	17 (1381) 16 (2077)	Others/not stated	19 (1945)	Chi² = 1.93, df = 2 (P = 0.38), I² = 0% Comparing only the Medical/therapeutic staff providers groups: Chi² = 0.05, df = 1 (P = 0.83), I² = 0%	No difference between subgroups
Motor function scales	Time post stroke Analysis 10.1	Early (2 weeks or less post‐stroke)	21 (2366)	Later (more than 2 weeks post stroke)	10 (748)	Chi² = 2.62, df = 1 (P = 0.11), I² = 61.8%	No difference between subgroups
	Geographical location ‐ continent of study conduct Analysis 10.2	Asia	47 (5347)	All other continents	3 (290)	Chi² = 22.93, df = 1 (P < 0.00001), I² = 95.6%	Greater treatment effect in studies conducted in Asia
	Amount of treatment (less or more than 2.5 hours/week) Analysis 10.3	Less than 2.5 hours/week	7 (707)	More than 2.5 hours/week	37 (3273)	Chi² = 0.99, df = 1 (P = 0.32), I² = 0%	No difference between subgroups
	Duration of intervention Analysis 10.4	Short (≤2 months)	22 (2332)	Longer (> 2 months)	25 (3)	Chi² = 0.00, df = 1 (P = 0.98), I² = 0%	No difference between groups with less or more than 2 months of treatment.
	Focus of treatment components Analysis 10.5	Functional task training	19 (1891)	Mixed treatment components (combining functional task training, musculoskeletal and neurophysiological components)	24 (2803)	Chi² = 3.51, df = 1 (P = 0.06), I² = 71.5%	No difference between subgroups
	Named approaches Analysis 10.6	Named as "three‐stage approach"	14 (2274)	Named as "Bobath" approach	9 (879)	Including 'other' trials (28 trials, 2516 participants): Chi² = 2.22, df = 2 (P = 0.33), I² = 9.9% Comparing only the "three‐stage" and "Bobath" groups: Chi² = 1.48, df = 1 (P = 0.22), I² = 32.3%	No difference between subgroups
	Provider of intervention Analysis 10.7	Medical/therapeutic staff Medical/therapeutic staff + family/carers	15 (1240) 16 (2391)	Others/not stated	19 (2038)	Chi² = 5.45, df = 2 (P = 0.07), I² = 63.3% Comparing only the Medical/therapeutic staff providers groups: Chi² = 1.77, df = 1 (P = 0.18), I² = 43.6%	No difference between subgroups

- Geographical location, with a greater treatment effect in studies conducted in Asia, for the outcomes of independence in ADL (P = 0.03; Analysis 9.2) and motor function (P < 0.00001; Analysis 10.2).
- Amount of treatment, with a smaller treatment effect when treatment was less than 2.5 hours/week, for independence in ADL (P = 0.02; Analysis 9.3), but not motor function (P = 0.32; Analysis 10.3).

No other differences between subgroups were observed (see Table).

#### Comparison 2. Physical rehabilitation versus attention control

Thirteen studies provided data on 13 comparisons of physical rehabilitation versus attention control. See Table.

##### Primary outcomes

###### Independence in ADL

Physical rehabilitation was more effective than attention control for the immediate outcome (SMD 0.91, 95% CI 0.06 to 1.75; I² = 76%; 2 studies, 106 participants; very low‐certainty; Analysis 3.1).

No studies assessed long‐term/follow‐up outcomes (Table).

###### Motor function

Physical rehabilitation was no more effective than attention control for immediate outcome (SMD 0.13, 95% CI ‐0.13 to 0.38; I² = 0%; 5 studies, 237 participants; very low‐certainty; Analysis 3.2).

This finding is similar at long‐term/follow‐up time points (Analysis 4.2; Table).

##### Secondary outcomes

- Balance (Berg Balance Scale):
  - Physical rehabilitation was no more effective than attention control for immediate outcome (MD 6.61, 95% CI ‐0.45 to 13.66; I² = 94%; 4 studies (240 participants); very low‐certainty; Analysis 3.3).
  - No studies assessed long‐term/follow‐up outcomes.
- Gait velocity:
  - Physical rehabilitation may be more effective than attention control for immediate outcome (SMD 0.27, 95% CI ‐0.01 to 0.55; I² = 51%; 9 studies, 474 participants; and SMD 0.34, 95% 0.14 to 0.54; I² = 0%; 7 studies, 405 participants, when only direct gait speed data are included; moderate‐certainty; Analysis 3.4).
  - This benefit was not sustained long‐term (Analysis 4.4; Table).
- Length of stay: Only one study (30 participants) assessed length of stay, providing very low‐certainty evidence (Analysis 3.5).

###### Adverse events

Three studies actively monitored and reported adverse events (Dean 2006; Pang 2005; Stuart 2019; Table). The reported data suggest that there was no increase in the risk of adverse events for those undergoing physical rehabilitation (RR 1.92, 95% CI 0.75 to 4.93; I² = 0%; 3 studies, 290 participants; very low‐certainty; Analysis 3.6), but this evidence is insufficient to support generalised conclusions.

#### Comparison 3. Additional physical rehabilitation + usual therapy versus usual therapy alone

Forty‐three studies provided data on 44 comparisons exploring additional physical rehabilitation. See Table.

##### Primary outcomes

###### Independence in ADL

Additional physical rehabilitation was beneficial for immediate outcome (SMD 1.26, 95% CI 0.82 to 1.71; I² = 95%; 21 studies, 1972 participants; low‐certainty; Analysis 5.1).

Sensitivity analysis to remove studies at high risk of bias (n = 8) did not change this conclusion, but the benefit was no longer observed when studies with considerable uncertainty (n = 8) were also removed (SMD 0.71, 95% CI ‐0.03 to 1.45; I² = 90%; 5 studies, 394 participants) (Table).

No studies assessed long‐term/follow‐up outcomes (Table).

###### Motor function

Additional physical rehabilitation was beneficial for immediate outcome (SMD 0.69, 95% CI 0.46 to 0.92; I² = 82%; 22 studies, 1965 participants; low‐certainty; Analysis 5.2).

Sensitivity analysis to remove studies at high risk of bias (n = 10) did not change this conclusion, but the benefit was no longer observed when studies with considerable uncertainty (n = 7) were also removed (SMD 0.18, 95% CI ‐0.04 to 0.39; I² = 0%; 5 studies, 338 participants) (Table).

The benefit was not seen at long‐term/follow‐up time points (Analysis 6.2; Table).

##### Secondary outcomes

- Balance (Berg Balance Scale):
  - Additional physical rehabilitation was beneficial for immediate outcome (MD 5.74, 95% CI 3.78 to 7.71; I² = 86%; 15 studies, 795 participants; low‐certainty; Analysis 5.3).
  - No studies assessed a long‐term/follow‐up outcome.
- Gait velocity:
  - Additional physical rehabilitation was beneficial for immediate outcome (SMD 0.59, 95% CI 0.26 to 0.91; I² = 79%; 19 studies, 1004 participants; low‐certainty; Analysis 5.4).
  - This benefit was not sustained long‐term (Analysis 6.4; Table).
- Length of stay: No studies assessed length of stay.

###### Adverse events

Four studies actively monitored and reported adverse events (Aries 2021; Duncan 2003; LAST 2018; Zhuang 2012; Table). Our meta‐analysis suggests that there was no increase in the risk of adverse events for those undergoing additional physical rehabilitation, and that the risk may be lower than for the 'usual care only' group (RR 0.80, 95% CI 0.64 to 0.98; I² = 0%; 4 studies, 702 participants; very low‐certainty; Analysis 5.5). The majority of adverse events come from a single study (LAST 2018), with a statistical weighting of 79.9%; this study reported a wide range of different adverse events, while other studies reported limited types of adverse events only. Further, LAST 2018 and Aries 2021 both reported the total number of adverse events, rather than the number of participants experiencing at least one adverse event. This evidence, from only four studies, is insufficient to support generalised conclusions.

No studies reported follow‐up data relating to adverse events.

##### Subgroup analyses

The results of subgroup analyses are provided in Table and in Analysis 11 (independence in ADL) and Analysis 12 (motor function). These indicated that there were differences between subgroups, based on the following:

**27:** Subgroup analyses: Additional physical rehabilitation + usual therapy versus usual therapy only ‐ primary outcomes

**Outcome**	**Subgroup comparison** **analysis**	**Subgroup 1**	**Studies (participants)**	**Subgroup 2**	**Studies (participants)**	**Test for subgroup differences**	**Comment**
Independence in ADL scales	Time post‐stroke Analysis 11.1	Early (2 weeks or less post‐stroke)	2 (136)	Later (more than 2 weeks post stroke)	13 (1210)	Chi² = 0.86, df = 1 (P = 0.35), I² = 0%	No difference between subgroups. Excluding studies with participants > 6 weeks post‐stroke did not change the result.
	Geographical location ‐ continent of study conduct Analysis 11.2	Asia	18 (1554)	All other continents	3 (418)	Chi² = 25.45, df = 1 (P < 0.00001), I² = 96.1%	Greater treatment effect in studies conducted in Asia. Excluding studies (n = 3) conducted in Asian countries other than China did not change the result.
	Amount of additional treatment (less or more than 2.5 hours/week) Analysis 11.3	Less than 2.5 hours/week	2 (401)	Between 2.5 and 5 hours/week More than 5 hours/week	7 (508) 12 (1065)	Chi² = 14.45, df = 2 (P = 0.0007), I² = 86.2%	The greater the amount of additional treatment, the greater the treatment effect.
	Duration of intervention Analysis 11.4	Short (≤ 2 months)	15 (1146)	Longer (> 2 months)	4 (688)	Chi² = 13.36, df = 1 (P = 0.0003), I² = 92.5%	Smaller treatment effect in studies of longer duration.
	Focus of treatment components Analysis 11.5	Functional task training added to less functional approach	14 (917)	Neurophysiological approach added to a mixed or other approach	5 (585)	Chi² = 5.13, df = 1 (P = 0.02), I² = 80.5%	Greater treatment effect when additional rehabilitation was focussed on functional task training, rather than neurophysiological approaches.
	Provider of intervention Analysis 11.6	Medical/therapeutic staff Medical/therapeutic staff + family/carers	8 (831) 6 (614)	Others/not stated	7 (527)	Chi² = 2.77, df = 2 (P = 0.25), I² = 27.8% Comparing only the Medical/therapeutic staff providers groups: Chi² = 1.69, df = 1 (P = 0.19), I² = 40.8%	No difference between subgroups
Motor function scales	Time post stroke Analysis 12.1	Early (2 weeks or less post‐stroke)	2 (200)	Later (more than 2 weeks post‐stroke)	17 (1495)	Chi² = 1.63, df = 1 (P = 0.20), I² = 38.7%	No difference between subgroups
	Geographical location ‐ continent of study conduct Analysis 12.2	Asia	15 (1333)	All other continents	7 (632)	Chi² = 14.90, df = 1 (P = 0.0001), I² = 93.3%	Greater treatment effect in studies conducted in Asia
	Amount of additional treatment (less or more than 2.5 hours/week) Analysis 12.3	Less than 2.5 hours/week	2 (412)	Between 2.5 and 5 hours/week More than 5 hours/week	8 (422) 11 (1039)	Chi² = 17.73, df = 2 (P = 0.0001), I² = 88.7%	The greater the amount of additional treatment, the greater the treatment effect.
	Duration of intervention Analysis 12.4	Short (≤ 2 months)	16 (1245)	Longer (> 2 months)	4 (582)	Chi² = 0.17, df = 1 (P = 0.68), I² = 0%	No difference between groups with less or more than 2 months of treatment.
	Focus of treatment components Analysis 12.5	Functional task training added to less functional approach	13 (910)	Neurophysiological approach added to a mixed or other approach	6 (573)	Chi² = 0.49, df = 1 (P = 0.49), I² = 0%	No difference between subgroups
	Provider of intervention Analysis 12.6	Medical/therapeutic staff Medical/therapeutic staff + family/carers	12 (1094) 3 (280)	Others/not stated	7 (591)	Chi² = 19.30, df = 2 (P < 0.0001), I² = 89.6% Comparing only the Medical/therapeutic staff providers groups: Chi² = 9.47, df = 1 (P = 0.002), I² = 89.4%	Studies with input from families/carers had greater effect size than studies reporting input from medical/therapeutic staff only.

- Geographical location, with a greater treatment effect in studies conducted in Asia, for independence in ADL (P < 0.00001; Analysis 11.2) and motor function (P = 0.0001; Analysis 11.2; Analysis 12.2).
- Amount of treatment, with a greater treatment effect the greater the amount of additional therapy, for independence in ADL (P = 0.0007; Analysis 11.3) and motor function (P = 0.0001; Analysis 12.3).
- Duration of intervention, with a greater treatment effect in studies with a *shorter* duration, for independence in ADL (P = 0.0003; Analysis 11.4) but not for motor function (P = 0.68; Analysis 12.4). Studies with shorter duration tended to have a higher treatment dose (amount per week).
- Focus of treatment, with a greater treatment effect when the additional therapy focussed on functional task training rather than neurophysiological approaches, for independence in ADL (P = 0.02; Analysis 11.5), but not for motor function (P = 0.49; Analysis 12.5).

A difference between subgroups was also found when comparing studies in which there was (or was not) input from families/carers reported, with a greater effect size in studies reporting input from families/carers for motor function (P < 0.0001; Analysis 12.6), but not for independence in ADL (P = 0.25; Analysis 11.6).

No other differences between subgroups were observed (see Table).

#### Comparison 4. Comparisons of different physical rehabilitation approaches

Sixty‐three studies provided data on 65 comparisons of different approaches to physical rehabilitation. These studies were divided into those that compared the following:

1. An intervention with a focus on functional task training to another approach (an intervention with much less, or no, functional task training).
2. An intervention with a focus on neurophysiological treatment components with an intervention with a different approach to physical rehabilitation.
3. Other comparisons (three trials had intervention comparisons that did not fit within the above two groups).

##### 4.1. Functional task training versus other approaches

See Table.

###### Primary outcomes

####### Independence in ADL

Functional task training was more effective than other approaches (SMD 0.58, 95% CI 0.29 to 0.87; I² = 86%; 22 studies, 1535 participants; low‐certainty; Analysis 7.1).

Sensitivity analysis, removing studies with high risk of bias (n = 5) or considerable uncertainty (11), did not change this conclusion (SMD 0.61, 95% CI 0.34 to 0.88; I² = 6%; 6 studies, 243 participants) (Table).

Functional task training remained more effective than other approaches at a long‐term/follow‐up time point (Analysis 8.1; Table).

####### Motor function

Functional task training was more effective than other approaches (SMD 0.72, 95% CI 0.21 to 1.22; I² = 95%; 20 studies, 1671 participants; very low‐certainty; Analysis 7.2).

Sensitivity analysis to remove studies at high risk of bias (n = 6) did not change this conclusion, but the benefit was no longer observed when studies with considerable uncertainty (n = 9) were also removed (SMD 1.01, 95% C ‐0.39 to 2.42; I² = 6%; 5 studies, 333 participants) (Table).

Functional task training remained more effective than other approaches at a long‐term/follow‐up time point (Analysis 8.2; Table).

###### Secondary outcomes

- Balance (Berg Balance Scale):
  - Functional task training was not more effective than other approaches for immediate outcome (MD 2.16, 95% CI ‐0.24 to 4.53; I² = 94%; 25 studies, 1194 participants; very low‐certainty; Analysis 7.3), but it was more effective for the subgroup of studies where the other approach comprised 'less' functional task training (MD 2.56, 95% CI 0.47 to 4.64; I² = 89%; 19 studies, 988 participants; Analysis 7.3.1).
  - This benefit was not sustained long‐term (Analysis 8.3; Table).
- Gait velocity:
  - Functional task training was not more effective than other approaches for immediate outcome (SMD 0.28, 95% CI ‐0.01 to 0.56; I² = 87%; 27 studies, 1719 participants; very low‐certainty; Analysis 7.4).
  - This finding was sustained long‐term (Analysis 8.4; Table).
- Length of stay:
  - Only one study (75 participants) assessed length of stay, providing very low‐certainty evidence (Analysis 7.5).

####### Adverse events

Six studies actively monitored and reported adverse events (DOSE 2020; Hendrey 2018; Kwakkel 2008; Mansfield 2018; Signal 2014; SPIRES 2022; Table). Meta‐analysis suggests that there was no increase in the risk of adverse events for those receiving functional task training, compared to those receiving less functional task training (RR 1.33, 95% CI 0.91 to 1.94; I² = 0%; 6 studies, 473 participants; very low‐certainty; Analysis 7.6). The majority of adverse events come from one study (Mansfield 2018), with a statistical weighting of 89.8%. This study recorded mild/moderate adverse events such as fatigue and muscle soreness, while other studies gathered data on more limited types of adverse events only. Further, Mansfield 2018 reported the total number of adverse events, rather than the number of participants experiencing an adverse event. The very low‐certainty evidence is insufficient to support generalised conclusions.

One study reported follow‐up data relating to adverse events, collecting data at 12 months following the intervention (DOSE 2020) (Analysis 8.5; Table).

##### 4.2. Neurophysiological approach versus other approaches

See Table.

###### Primary outcomes

####### Independence in ADL

A neuropsychological approach was less effective than other approaches (SMD ‐0.34, 95% CI ‐0.63 to ‐0.06; I² = 70%; 14 studies, 737 participants; low‐certainty; Analysis 7.7).

Sensitivity analysis, removing studies with high risk of bias (n = 2) or considerable uncertainty (n = 8), did not change this conclusion and removed heterogeneity (SMD ‐0.33, 95% CI ‐0.66 to ‐0.01; I² = 0%; 4 studies, 149 participants) (Table).

A neuropsychological approach remained less effective than other approaches at a long‐term/follow‐up time point (Analysis 8.6; Table).

####### Motor function

A neuropsychological approach was no different from other approaches (SMD ‐0.60, 95% CI ‐1.32 to 0.12; I² = 94%; 13 studies, 663 participants; low‐certainty; Analysis 7.8).

Sensitivity analysis, removing studies with high risk of bias (n = 3) or considerable uncertainty (n = 6), did not change this conclusion and removed heterogeneity (SMD 0.07, 95% CI ‐0.39 to 0.54; I² = 0%; 4 studies, 72 participants) (Table).

A neuropsychological approach remained no different from other approaches at a long‐term/follow‐up time point (Analysis 8.7; Table).

###### Secondary outcomes

- Balance (Berg Balance Scale):
  - A neuropsychological approach was no different from other approaches for immediate outcome (MD 0.06, 95% CI ‐5.90 to 6.03; I² = 94%; 9 studies, 292 participants; low‐certainty; Analysis 7.9).
  - There remained no difference long‐term (Analysis 8.8; Table).
- Gait velocity:
  - A neuropsychological approach was no different from other approaches for immediate outcome (SMD ‐0.17, 95% CI ‐0.62 to 0.27; I² = 85%; 16 studies, 630 participants; very low‐certainty; Analysis 7.10).
  - There remained no difference long‐term (Analysis 8.9, Table).
- Length of stay:
  - No studies assessed length of stay between a neurophysiological approach versus other approaches.

####### Adverse events

Two studies actively monitored and reported adverse events (Epple 2020; Morreale 2016; see Table), but only data from Epple 2020 were suitable for analysis. However, this study reported no adverse events that were considered related to the intervention in both arms, and the effect size is therefore not estimable (1 study, 40 participants; very low‐certainty; Analysis 7.11). This evidence is insufficient to support generalised conclusions.

No studies reported follow‐up data relating to adverse events.

##### Comparisons of other approaches

Three studies compared physical rehabilitation approaches that did not fit within the groups above. Results of these individual trials are reported in Table.

## Discussion

### Key findings

This review included 267 studies (21,838 participants) that explored the effects of different physical rehabilitation approaches; data from 194 (15,815 participants) were included in meta‐analyses. Half of these studies (133/267) were carried out in China.

#### Is physical rehabilitation more effective than no rehabilitation?

A total of 105 studies compared physical rehabilitation with no physical rehabilitation. Compared to no physical rehabilitation, the evidence suggests the following:

- Physical rehabilitation may improve **independence in ADL**, **motor function**, and **balance** immediately after intervention (low‐certainty evidence).
- Physical rehabilitation likely improves **gait speed** immediately after intervention (moderate‐certainty evidence).
- The evidence is very uncertain about the effects of physical rehabilitation on **length of stay** and **adverse events** (very low‐certainty evidence).

Improvements in independence of daily living and motor function may be sustained in the long term. Our certainty in the effect size is low (moderate for gait speed), due to variations between participants, interventions, and outcome measures, and poor reporting. Certainty in long‐term outcomes is limited by lack of data with very low numbers of studies reporting follow‐up data for independence in ADL (n = 12), motor function (n = 11), balance (n = 4), or gait velocity (n = 7).

##### Can we explain the heterogeneity in these findings?

There was substantial heterogeneity in all meta‐analyses. Removing studies at high risk of bias or considerable uncertainty did not change conclusions. Exploration of subgroups demonstrated that, for the primary outcomes of independence in ADL and motor function:

- Physical rehabilitation had a greater treatment effect in studies conducted in Asia than in studies conducted elsewhere. However, relatively few studies were conducted in countries other than China (only 6/52 and 3/50 studies for outcomes of independence in ADL and motor function respectively). Further, the studies conducted in continents other than Asia all recruited participants who were living at home and/or were several months or years post‐stroke, while the majority of those conducted in Asia recruited hospital inpatients. Differences in risk of bias were also noted, with more concerns about risk of bias in studies from Asia. Thus, this subgroup difference may be a reflection of the different research designs, conduct, and reporting, and not an indication that there are differences in outcomes for patients living in different countries.
- Studies delivering less than 2.5 hours/week had a smaller treatment effect than those delivering more. There was a statistically significant subgroup difference for independence in ADL but not for motor function, but considerable heterogeneity between studies limiting certainty in findings.

No subgroup effects were found for time post‐stroke, duration of intervention, focus of treatment components, name of approach, or provider of intervention.

#### Is physical rehabilitation more effective than attention control?

A total of 19 studies compared physical rehabilitation with attention control. Limited evidence suggests that, compared to attention control:

- Physical rehabilitation may improve **independence in ADL**, but the evidence is very uncertain (very low‐certainty evidence).
- Physical rehabilitation likely improves **gait speed** (moderate‐certainty evidence).
- Physical rehabilitation may *not* improve **motor function** or **balance**, but the evidence is very uncertain (very low‐certainty evidence).
- The evidence is very uncertain about the effects of physical rehabilitation on **length of stay** and **adverse events** (very low‐certainty evidence).

Our certainty in these findings is limited by the volume of data, variations between participants, interventions, and outcome measures, and poor reporting. There was insufficient evidence to support conclusions about the long‐term benefits of physical rehabilitation compared to attention control.

#### Is additional physical rehabilitation beneficial?

We identified 56 studies that explored the effect of additional physical rehabilitation (added to usual rehabilitation). The evidence suggests that:

- Additional physical rehabilitation plus usual therapy may improve **independence in ADL**, **motor function**, **balance**, and **gait speed** (low‐certainty evidence). Our certainty in these findings is low, due to variations between participants, interventions, outcomes, and poor reporting.
- None of the 56 studies reported data on **length of stay**.
- The evidence is very uncertain about the effect of additional physical rehabilitation on **adverse events** (very low‐certainty evidence).

There was very limited data for follow‐up measures, providing insufficient evidence to support conclusions about long‐term benefits.

##### Can we explain the heterogeneity in these findings?

There was substantial heterogeneity in all meta‐analyses. Removing studies at high risk of bias did not change conclusions, but the benefits of additional physical rehabilitation were no longer observed when studies with considerable uncertainty were removed. Exploration of subgroups demonstrated that, for primary outcomes of independence in ADL and motor function:

- Additional physical rehabilitation had a greater treatment effect in studies conducted in Asia than in studies conducted elsewhere. As above, there are a number of factors ‐ particularly time post‐stroke of participants and risk of bias ‐ which may explain this finding.
- The greater the amount of additional physical rehabilitation, the greater the treatment effect. The majority of studies (12/21 and 11/21 respectively for independence in ADL and motor function) delivered more than five hours/week of additional rehabilitation.
- There was a smaller treatment effect in studies of *longer* duration, for independence in ADL, but not for measures of motor function. In general, studies with longer duration tended to have a lower treatment dose (amount per week).
- Studies involving families/carers (in addition to medical/therapeutic staff) had a greater effect size than studies not reporting any family/carer involvement, for measures of motor function but not for independence in ADL.
- Studies delivering additional therapy that focussed on functional task training had a greater effect size than those where the additional therapy focussed on neurophysiological approaches, for measures of motor function, but not for independence in ADL.

No subgroup effects were found for subgroups based on time post‐stroke.

#### Is one approach to physical rehabilitation better than any other approach to physical rehabilitation?

A total of 92 studies explored the effect of different approaches to physical rehabilitation. The evidence relating to ***functional task training*** suggests that, compared to other approaches:

- Functional task training approaches may improve **independence in ADL**. This is low‐certainty evidence.
- Functional task training approaches may improve **motor function**, but this evidence is of very low certainty.
- Functional task training approaches may *not* improve **balance** when compared to neurophysiological approaches, but may improve balance when compared to approaches with less functional task training. However, this evidence is of very low certainty.
- Functional task training approaches may *not* improve **gait speed**, but the evidence is very uncertain.
- There is considerable uncertainty about the effects of functional task training on **length of stay** and **adverse events** (very low‐certainty evidence).

The evidence relating to **neurophysiological approaches** suggests that, compared to other approaches:

- Neurophysiological approaches may be less effective for **independence in ADL**. This is based on low‐certainty evidence.
- Neurophysiological approaches may be no different from other approaches for **motor function** or **balance**. This is low‐certainty evidence.
- Neurophysiological approaches may be no different from other approaches for **gait speed**, but this evidence is of very low certainty.
- No conclusions can be made about **length of stay**, as none of the studies reported this outcome.
- There is considerable uncertainty about the effect of neurophysiological approaches on **adverse events** (very low‐certainty evidence).

Our certainty in these findings is low to very low, due to variations between participants, interventions, outcomes, and comparisons, and poor reporting. Removing studies at high risk of bias did not change conclusions relating to functional task training for primary outcomes, but removing studies with considerable uncertainty changed the findings for the motor function (but not the independence in ADL) outcome. Removing studies at high risk of bias did not change conclusions relating to neurophysiological approaches for the primary outcomes.

### Identification of relevant trials

The identification of all relevant trials was confounded by several factors.

1. Inconsistent and poorly defined terminology: Electronic searching was difficult because the names given to different physiotherapy rehabilitation approaches are poorly documented, often have several derivations, and have varied over time. Furthermore, the interventions were not always described as 'physiotherapy' or 'physical therapy,' but sometimes were described as 'rehabilitation,' 'training', or 'exercise.' In an attempt to identify all relevant trials, we used broad and multiple search terms to identify interventions that may meet our definition of physical rehabilitation to improve function and mobility. However, the challenges of inconsistent terminology and poor descriptions of rehabilitation interventions introduced considerably uncertainty and subjectivity into the process of study identification and selection.
2. Change in focus of the review: As described above, for this update of the review, additional criteria were introduced to limit the scope of this review. No change to the search strategy or to the selection criteria was implemented, and we do not believe that any changes would be justified. These criteria have narrowed the scope of the review, so we are confident that decisions made relating to the exclusion of studies from the search results for previous versions of this review would not be different in light of the changed focus. However, selection of relevant trials for this review has always been challenging, and we do not believe that the change in focus of the review has affected study selection in one direction or another.
3. Lack of detail within the abstracts: Lack of information on study methods, participants, and interventions potentially increases the chance that a relevant trial may be excluded.
4. Material published in Chinese language: A substantial number of the included studies were carried out in China and were published in Chinese language. For this version of the review, we conducted searches of Chinese databases, increasing our chances of identifying relevant RCTs published in Chinese language. However, these searches identified thousands of RCTs of rehabilitation (including but not limited to physical rehabilitation) for people after stroke. Due to the numbers involved, we made many decisions to exclude based on abstracts and did not always obtain full papers. Further, due to limitations in the time and resources for this review update, we did not complete screening of the search results of Chinese‐language databases. These actions may have led to the exclusion of some relevant trials. However, given the large number of (small) heterogeneous trials included in this review, we are confident that inclusion of further trials is unlikely to change the conclusions of this review.

We note the search for this large‐scale review update was performed in November 2022 and recognise this as a potential limitation. However, it is unlikely that evidence that has emerged since November 2022 would substantially alter our current findings/conclusions. Furthermore, given the sheer size of this review, future updates in its current form may lead to versions being too large to be useful for decision‐making. Therefore, we advise against an update of this review in its current form and will commit to revisiting the review scope with relevant stakeholders to ensure the most pertinent questions for decision‐making are explored.

### Completeness of published studies

Reporting was considered poor or incomplete for the majority of included studies. For example, time since stroke was unclear or not reported in almost one‐third of all included studies, and for over half of the reported interventions, it was not clear if the mode of delivery involved a one‐to‐one treatment session.

Reporting of methodological details required to judge risk of bias was generally incomplete. Often studies reported the number of included participants, inferring that there were no dropouts. While we have taken this information at face value, we suspect that in some cases, study authors have excluded dropouts from the results, potentially introducing biases.

Many of the relevant trials that we included were published only as abstracts or as brief reports. This was frequently the case for studies published in Chinese, for which published versions were often less than two pages long. Due to the number of studies included in this review, we did not have the time or resources to contact all study authors for further information on trial design or study results. Thus, in general, the completeness of study information is low, resulting in a high number of studies for which risk of bias judgements are 'unclear' and a high number of studies that do not contribute data to the analyses.

### Completeness of outcome data

Very few studies had a published protocol that we were able to access (only five studies with primary outcome data contributing to analysis of physical rehabilitation versus no physical rehabilitation; and only two studies with primary outcome data contributing to analysis of additional physical rehabilitation). The lack of protocol made it impossible to judge whether reporting of outcomes and outcome data was as pre‐stated, reducing our certainty in the evidence.

There was insufficient evidence to reach any conclusions relating to length of stay, with only three studies reporting data on this as an outcome.

Relatively few studies followed up with participants after the intervention had ended: data were available immediately at the end of intervention for 114 studies for the independence in ADL outcome and for 111 studies for the motor function outcome, but only for 24 and 28 studies, respectively, for a longer‐term follow‐up outcome. Follow‐up data from studies comparing physical rehabilitation with no physical rehabilitation demonstrate that the beneficial effect of intervention is maintained for primary outcomes. Limited evidence from studies comparing different approaches to physical rehabilitation also suggests that effects may be sustained long‐term. However, in the comparisons of physical rehabilitation versus attention control and effects of additional physical rehabilitation, there were insufficient primary outcome data to support conclusions about long‐term effects. Lack of follow‐up data therefore limits the ability of review authors to draw generalisable conclusions relating to whether observed benefits are maintained for some comparisons.

### Reporting of adverse events

Only 19 studies actively monitored adverse events. There was significant inconsistency in the way adverse events and dropouts were reported in the studies, making synthesis problematic. Some study reports distinguished between adverse events and dropouts; others reported no adverse events but detailed participants dropping out due to adverse events. Some did not report on adverse events at all, or simply stated that none occurred. A post‐hoc decision was made to focus on adverse events that were possibly, probably, or definitely related to the study/study interventions, as attributed by the study authors. This decision was made in order to exclude data about adverse events that were clearly unrelated to the study (e.g. reactions to drugs). However, inconsistencies were noted in judgements across our author team as to whether adverse events, where reported, could possibly be related to the study or not. For example, some studies considered some (or any) cardiovascular events (e.g. stroke progression, transient ischaemic attack (TIA)) to be possibly related to the study, while others did not. Further, studies reporting the greatest number of adverse events tended to have procedures in place to gather information about mild/moderate events (e.g. muscle strains and fatigue) while other studies did not gather data relating to these events. Studies gathering information about these mild/moderate events often reported the total number of reported events, rather than the number of participants experiencing an adverse event. These differences mean that we have very low confidence in the findings of the analyses of adverse events.

### Descriptions of interventions

Clear, concise documentation of complex physical interventions is exceptionally difficult to achieve. Information provided by study authors regarding interventions administered in the included trials is in the Characteristics of included studies. Although many of the included studies attempt to describe all administered interventions, the available documentation is often insufficient to allow confident and accurate repetition of the applied rehabilitation approach. Problems with documentation of interventions generally are not the fault of researchers or therapists, but rather are due to the fundamental problem of recording methods of physical handling skills and techniques, and the nature of the relationship between stroke survivor and physiotherapist. Documentation of this process would generally be complex and 'wordy'; therefore, often it is not possible to present within research papers with limitations on length. These problems are confounded by the fact that treatments applied are often ultimately the decision of a single physiotherapist, based on an individual assessment of a unique stroke survivor's movement disorders, and adapted or modified throughout a treatment session. However, it is important to note that the interventions we have included, which have this variability, align with the definition of rehabilitation (Negrini 2022) and with the interventions stated as relevant to our review.

Furthermore, the common basis of physical rehabilitation 'approaches' is that they are holistic. All body parts and movements can be assessed and treated based on the selected approach; however, a physiotherapist may select to concentrate on the treatment of one particular body part or movement during a treatment session. Subsequently, treatments given to specific stroke survivors by individual therapists may vary enormously. This review attempted to limit this variation slightly by excluding trials that had provided interventions only to the upper limb or trunk. Nevertheless, although we attempted to explore the use of different treatment components through subgroup analyses, it remains conceivable that substantial differences exist between the physical rehabilitation interventions given to participants within the same subgroups.

Furthermore, we found that 'basic' information about the interventions (such as whether it was delivered face‐to‐face, the number and frequency of sessions, and who provided the intervention) was often inadequate: templates to promote adequate and consistent reporting of interventions are essential.

### Categorisation of treatment components within interventions

The comparisons carried out within the review relied on categorisation of treatment components that were described within the published papers. One review author categorised the described treatments using agreed definitions of individual treatment components, and a second review author checked these. This categorisation process relied on adequate descriptions within published papers. Papers that published only very brief descriptions of interventions therefore may have resulted in categorisations that were not truly reflective of the intervention delivered.

Furthermore, this process of categorisation was highly dependent on the language and terminology provided within a written description. For example, an author may state "activities aimed at improved gait." This description would result in categorisation only within the functional task training component of "walking". However, in practice, this intervention could have included components such as active or active‐assisted movement, sensorimotor facilitation, and muscle strengthening. Therefore, our method of categorisation is likely to have underestimated rather than overestimated the numbers of treatment components and intervention categories. Hence, if any inaccuracy exists, the interventions are likely to be more "mixed" and eclectic than has been captured by our method of categorisation.

A number of difficulties were encountered in distinguishing between interventions that included only functional task training components and those that also included musculoskeletal (active) components. In particular, the review authors encountered difficulties in determining whether an intervention focused on a functional task might also include active or active‐assisted movement. This reality was due to the fact that all functional task training necessitates active movement, and overlap between practice of an active movement and practice of a functional task can be inevitable. The framework for categorisation of treatment components was co‐produced in partnership with our stakeholder group for this review update (see Appendix 1; Figure); this was developed as there was no suitable existing framework. Further work is required to explore and refine the clinical relevance and validity of this framework.

### Treatment components within named approaches

We were aware when developing the definitions for categorisation of described interventions that a number of studies have stated a named approach (e.g. 'Bobath,' 'Motor‐relearning programme') without providing any description of the treatment components included within the approach. We therefore wrote definitions such that these studies could be captured by our system of categorisation of individual treatment components. However, including studies that have provided only the name of an approach without providing any description potentially introduces a number of biases. These biases occur as a result of the fact that the content of named approaches potentially changes over time and in keeping with geographical or personal preferences and biases. In particular, several studies reported that the intervention was 'Bobath', and much debate has surrounded the content of physiotherapy interventions based on the Bobath concept. This debate arises largely from the fact that the content of the Bobath approach has changed over time, published descriptions are limited, and the content of current therapy is variable (Carr 1994a; DeJong 2004; Langhammer 2012; Mayston 2008; Nilsson 1992; Pomeroy 2001b; Sackley 1996; Turner 1995; Tyson 2009b). A summary of the philosophy or theory of some of the key named approaches was drawn up for the first version of this review and is provided in Appendix 2. Some of the studies included in this review described the intervention as "Bobath" without providing any further details (e.g. Fang 2003; Fang YN 2004; Xu 1999), and some described techniques that may be considered outdated (e.g. "Bobath shaking hands technique", Wan Xueli 2014); thus we cannot be confident about the relevance or applicability of the content of the interventions delivered in these studies.

Thirty studies, all conducted in China, described the intervention as a 'three‐staged' approach. Generally, there was a description ‐ often relatively detailed ‐ of the intervention/treatment components involved in this named approach. However, we did note a number of differences between trials in the treatment components that were described as comprising part of this. These differences led to differences in how we categorised the treatment components of these studies, with some categorised as comprising a mix of functional task training, musculoskeletal and neurophysiological components (e.g. Bai 2008; Hou 2006; Lu 2004; Wu 2006; Yan 2015; Zhang 1998; Zhang 2004; Zhu 2007), and some categorised as being primarily focussed on functional task training (e.g. Bai 2014; Chen J 2014; Fan WK 2006; Hou Zhi 2014; Lu 2014; Lu Liangyan 2014; Wang Dongya 2015; Wu Jing 2015; Xie 2005; Yang Zhihong 2015; Zang 2013; Zhu 2004b). We are unclear as to whether these differences were due to variations in the delivered intervention, or differences in the reporting of the intervention.

### Translation into English

The review team worked in English language, and the majority of screening and trial selection was conducted in English. We used the translation function within Covidence to translate foreign‐language abstracts into English. There is a risk of translation errors leading to inadvertent exclusion of studies. Where we were uncertain about the 'sense' of the English translation, we sought input from a person who was fluent in the relevant language.

All of the included full papers were published in either English or Chinese language. For the Chinese‐language papers, we sought translation of the intervention description into English. We also used Google Translate to translate sections of text from some of Chinese‐language studies, and this translated text was used during full‐text screening and data extraction from included studies. Our Chinese‐speaking review member checked a random 10% of the data extracted from papers where we used Google Translate, and this did not identify any serious errors or concerns. Again, where there were any uncertainties in the Google Translate text, we sought input from our Chinese‐speaking review author (PLC) or volunteers (see Acknowledgements). While we acknowledge that there are limitations associated with the use of Google Translate (that concerns have been raised about the accuracy of technical translations) and that this may have introduced some errors within our data extraction, we do not think that our use of Google Translate (as opposed to in‐person translation) should have affected the conclusions. Numerical data within Chinese‐language papers were generally presented as Arabic numerals within tables, aiding accurate extraction of results data.

In addition, several included studies were carried out in China and the papers were published in English, but by authors for whom English clearly was not the first language. These translations provided a number of challenges in relation to the interpretation of meanings and subsequent classification of treatment components. For example, in several papers, it was unclear whether 'standing up training' referred to activities carried out in standing (i.e. training to promote standing balance) or to sit‐to‐stand training. In these cases, decisions were made based on discussion between two review authors (one of whom was a Chinese‐speaking physiotherapist (PLC)).

### Geographical location of studies

Different cultures and healthcare systems: This review brings together trial evidence from 36 countries around the world. Consequently, we have combined data from very heterogeneous settings, where culture, beliefs, and healthcare practice vary substantially. As 50% of the studies were carried out in China, our subgroup analyses focussed on comparing the results of studies carried out in China with studies carried out in the rest of the world. These subgroup analyses indicated that studies carried out in China may have a greater effect size than those carried out elsewhere in the world. This finding may be due to reporting biases and may reflect biases associated with publication, location, citation, and language. This finding may also reflect a true difference in the effects of interventions carried out in different geographical locations, which may be a result of differences in culture, traditions, training, and implementation of interventions. However, all studies that compared physical rehabilitation against no physical rehabilitation in the acute phase of stroke were carried out in China; this is a reflection of the fact that 'usual care' can comprise no routine physical rehabilitation within these geographical settings. Hence, studies conducted in China generally had participants who had a shorter time since stroke, and this provides a likely explanation for the greater treatment size observed in studies conducted in Asia.

### Risk of bias in included studies

Judgement of certainty of evidence was very difficult because of poor, incomplete, or brief reporting of information. Only 13 studies included in the analyses were judged to be at low risk of bias for all domains. We carried out sensitivity analyses to explore the effect of including studies with high or unclear risk of bias. These sensitivity analyses generally found that removal of studies with high or unclear risk of bias did not alter the direction (or significance) of the results. Thus, although the quality of most of the evidence included in this review remains uncertain, the fact that inclusion of these studies does not affect the direction of results gives us greater confidence in our findings. The main message arising from this review in relation to certainty of the evidence is that it is essential that reporting of methodological features of RCTs of physical rehabilitation interventions is improved, and that studies are reported using the CONSORT guidelines for reporting (Schulz 2010).

Studies that used quasi‐random assignment are excluded from this review. However, we found information about the method of randomisation particularly difficult to judge in a number of studies, particularly studies published in Chinese, in which use of the term 'random' in English abstracts did not always reflect the descriptions provided in Chinese versions of the study. There is an urgent need for trialists to address the issue of adequate reporting of methods of randomisation. It is possible that we have inadvertently included in this review trials that used quasi‐random assignment, rather than true random assignment.

The nature of rehabilitation interventions and the ethical requirement to obtain informed consent often make it difficult, if not impossible, to blind participants. It is generally impossible to blind the treating therapist because treating therapists have to be familiar with the intervention they are administering. Therapists who strongly favoured one approach over another could introduce performance bias. In several studies, the same therapist administered treatment to participants in both study groups; this potentially introduced considerable contamination between groups. The 'beliefs' of stroke survivors and therapists may further contribute to biases within many of these studies, and the large number of different geographical locations in which studies were carried out means that the studies were carried out with participants living in a wide variety of cultures, which could potentially impact the response to physical rehabilitation. Frequently there was insufficient evidence to judge if there was likely to have been bias due to deviations from the intended interventions. Many of the included trials did not state whether they used a blinded assessor. Lack of blinding of assessors potentially introduces considerable bias into the study results. This is particularly important in studies in which therapists often have strong beliefs in support of a particular approach.

We judged the majority of studies included in the analyses to have a low risk of bias due to missing outcome data. This is generally because studies did not report any dropouts. However, while for these studies we found no evidence to suggest that there were unreported dropouts, often we lacked confidence in these judgements. It is challenging, if not impossible, to identify information that trialists have failed to report.

We identified published protocols for very few of the included trials, meaning that there was considerable uncertainty about potential biases in the selection of the reported result. We did not have protocols for any of the Chinese‐language trials; there is a chance that we have failed to find these due to the fact that (other than our searching of Chinese electronic databases) we conducted all our searching in the English language.

The most frequent risk of bias judgement for all domains (except for bias due to missing outcome data; see above) was one of 'uncertain'. This is due to the poor nature of reporting across the trials. Where trials reported more detail, this sometimes led to a judgement of high risk of bias; thus, it is possible that the poor reporting in some trials resulted in an overly 'generous' risk of bias judgement (i.e. 'uncertain' rather than 'high' risk of bias).

### Heterogeneity of included trials

In addition to the limitations of the study methods, the trials included in the review had considerable heterogeneity. The key areas of heterogeneity were related to interventions and to participants.

1. Interventions: Although attempts have been made to categorise the interventions using a systematic, rigorous, and valid method, considerable variation may still exist between studies that have used similar types of treatment components. Furthermore, substantial variations in dose and intensity and in the length of the treatment period were noted. Also different is the fact that some interventions were carried out only when a therapist was present, whilst in other studies, independent practice of activities outside therapy sessions was encouraged.
2. Participants: The participant populations in the different included studies were heterogeneous. They varied from limited populations (e.g. pure motor stroke only) to those inclusive of all stroke survivors. Considerable variation in the time since stroke was also noted. The validity of combining results from such heterogeneous samples is debatable. We previously recommended that updates consider subgroup analysis to explore the initial impairment of included participants; however, there remains a lack of data relating to initial impairment in the majority of included studies.

Although we have carried out subgroup analyses to explore issues relating to the heterogeneity of both the interventions (i.e. dose, duration, components) and the participants (time since stroke) and other issues (geographical location, provider of intervention), it is likely that a complex interrelationship exists between some of the subgroups that we have been unable to explore. For example, studies carried out in China tended to be those with the least time since stroke, meaning that effects found that have been attributed to geographical location could be due equally to time since stroke (and vice versa). We believe that this may be true for a number of other variables. An example of this was demonstrated by the finding within a subgroup analysis that studies with a shorter duration had a greater benefit (see Table; Analysis 11.4): exploration reveals that studies with a shorter treatment duration generally had a greater daily dose, and it seems reasonable to assume that there may also be a difference in participants, as participants who are able to comply with a high daily dose may differ from those who cannot.

### Publication bias

As has been discussed above, the identification of all relevant trials was confounded by a number of factors, and, despite a rigorous search strategy, we are not fully confident that we will have successfully identified all studies. Consequently, this review may be biased towards particular types of studies and publications.

In the last version of this review (Todhunter‐Brown 2014), we identified a large proportion of relevant studies that were conducted in China. This led to the decision to search the Chinese Biomedical Literature Database for this update, and provides some explanation for the proportion (133/267) of Chinese‐language studies included in this update. However, we did not search any other non‐English language databases. Therefore, this review may be biased towards studies listed in English‐ and Chinese‐language databases, and we may be missing other non‐English studies or studies published in journals that are not included in the electronic databases that we searched.

Further, despite our searching of the Chinese Biomedical Literature Database, we are not confident that we will have successfully identified all relevant studies published in China, or in the Chinese language.

### Non‐reporting bias

Results of funnel plots revealed that some small studies report larger effect sizes. This could be an indication that small studies, with poor methodological quality, have spuriously inflated effects (Page 2016). Our sensitivity analyses, removing studies at high or unclear risk of bias from analyses, generally did not change the conclusion (although the effect size may change/reduce): these sensitivity analyses increased our confidence in the findings of our analyses.

A small number of studies presented data that we were not able to incorporate into meta‐analyses. Very few studies had published study protocols that we were able to access, limiting our ability to make judgements relating to potential reporting biases (e.g. only 3/52 studies in Analysis 1.1 and 3/50 in Analysis 1.2 were judged as low risk of bias for bias in selection of the reported result, with the remaining uncertain due to lack of protocol).

### Subgroup analyses

We carried out subgroup analyses to explore seven different effect modifiers (see Subgroup analysis and investigation of heterogeneity). False‐negative and false‐positive significance tests increase in likelihood rapidly as more subgroup analyses are performed (Deeks 2023), therefore there is a risk of errors as a result of the number of effect modifiers performed. We have included these effect modifiers as these were considered of high importance by our stakeholder group and the list of these important subgroups was decided prior to completion of the analyses, meaning that this decision was not influenced by the study findings. However, this is a high number of subgroup analyses within a large and complex review, and exploration of these potentially introduces errors and misleading findings. Future updates of this review should consider, identify, and pre‐state a smaller number of priority effect modifiers to explore.

### Treatment components and categorisation of interventions

We introduced a method of categorisation of interventions that was agreed by a stakeholder group of physiotherapists, stroke survivors, and carers. This method of categorisation has not been tested or explored further, and such testing is necessary to confirm the relevance and validity of the identified categories. In the absence of any other suitable method of categorisation of treatment components, we believe that we have adopted a robust, justifiable method—based on consensus between physiotherapists, stroke survivors, and carers. We argue that the involvement of this expert stakeholder group has considerably enhanced our review and is substantially advantageous compared with the alternative of having researchers make decisions over the categorisation of interventions and the structure of comparisons. Feedback from the stakeholder group members confirms that the group perceived that their input benefited the format of the review and made the review more clinically relevant. Due to the limited nature of our resources, members of the stakeholder group were based in the UK; however, two international webinars were held at which the proposed framework for categorising the physical rehabilitation interventions was presented and feedback received. This did not identify any concerns from international stakeholders with the framework that had been developed. Nevertheless, given known differences in physiotherapy practice in different parts of the world, we recommend that the proposed categories are explored and amended to reflect international practices in relation to stroke rehabilitation.

We recognise that the terminology used, particularly in the titles of the categories, may not be universally accepted or understood. We acknowledge that the appropriateness of terms such as 'functional,' 'neurophysiological', and 'musculoskeletal' can be debated when used in the way we have used them within this review. However, these terms were selected by the stakeholder group to have clinical meaning, and our stakeholder group reached a consensus on definitions of all terms used to categorise the interventions (Table; Appendix 1).

### Appraisal of quality in Chinese‐language papers

As described earlier, while no language restrictions were applied, and studies were conducted in 36 different countries, all included studies were published in either English or Chinese language. Chinese papers included in the review for which we were unable to use Google Translate (due to formatting issues in obtained papers) were discussed between one review author with the language skills to translate relevant sections of the papers and a second author who did not speak Chinese. The review author with Chinese language skills also possessed the necessary quality appraisal skills and had detailed expertise of physiotherapy and stroke rehabilitation. These two authors discussed the papers and completed data extraction together. Audio recordings of discussions/live translations were made to enable the review team to refer back to these if further information was required. Thus, although two review authors did consider the quality of these non‐English language papers, the assessment of the second review author was based entirely on the translation provided by the first review author. This method means that if the first review author made any errors in translation, or missed information provided in the non‐English text, the second review author will not have identified this. Thus, although having two review authors for these papers offers advantages, it does not provide the same level of 'independence' as the process of having two independent review authors for the English language papers. However, given the volume of Chinese‐language papers that we have included, and the available resources for this review, we believe that we have taken all steps available to us to minimise potential biases in this process.

### Conclusions arising from this review

Following completion of the analyses and results of this review, this information was presented to our stakeholder group, which comprised physiotherapists, stroke survivors, and carers. For each of the main comparisons and associated subgroup analyses, group members discussed the clinical implications and key messages arising from the results. The points discussed have been incorporated within the Discussion and Authors' conclusions sections of this review. In particular, the stakeholder group members highlighted the need to specifically draw out information pertaining to the dose of interventions delivered within the studies, as this was believed to have important implications for clinical practice. We believe that this process of consultation considerably removes potential biases from the process of reaching conclusions from this review, as the conclusions reflect the views of expert clinicians, stroke survivors, and carers, rather than only the potentially biased viewpoints of researchers and academics.

### Previous version of this review

The previous version of this review concluded that "no one approach to physical rehabilitation is more (or less) effective in promoting recovery of function and mobility after stroke" and that "physical rehabilitation, using a mix of components from different approaches, is effective for recovery of function and mobility after stroke" (Todhunter‐Brown 2014).

These findings supported the conclusion that "physical rehabilitation should not be limited to compartmentalised, named rehabilitation approaches, but should comprise clearly defined, well‐described, evidence‐based physical treatments regardless of historical or philosophical origin". This updated review agrees with, and adds considerable evidence to, these previous conclusions. While the 2014 version concluded that evidence indicates that there is no difference between physical rehabilitation approaches, this updated version provides evidence that physical rehabilitation focussing on functional task training is most effective, with some evidence that treatments described as "Bobath" approach may be less effective than other approaches to rehabilitation.

### Other reviews

This updated review is in agreement with the conclusions of other published reviews. A number of other published reviews agree with the conclusion that physiotherapists should not use compartmentalised, named approaches, but should select clearly defined and described techniques and task‐specific treatments, regardless of their historical or philosophical origin (Kollen 2009; Langhammer 2012; Mayston 2008; Pomeroy 2005). A number of reviews have specifically addressed questions relating to the efficacy of 'Bobath' or 'neurodevelopmental' approaches, and agree with the conclusion that the Bobath (or neurodevelopment) approach is not more effective, and may be less effective, than other interventions (Díaz‐Arribas 2020; Eng 2007; Kollen 2009; Pathak 2021; Scrivener 2020).

A scoping review has highlighted serious concerns about the description and consideration of Bobath therapy within randomised controlled trials and has provided an "updated Bobath clinical framework" to inform practice and research (Vaughan‐Graham 2015a; Vaughan‐Graham 2015b). The framework co‐produced with our stakeholder group and used in this review did not consider this published framework, as the focus was on the description of individual treatment components, regardless of the philosophical origin.

Zhang 2013 aimed specifically to review RCTs that compared rehabilitation versus standard care after stroke in China. This review pooled evidence from 31 trials (5220 participants) that reported independence in ADL (Barthel Index) and 27 trials (4501 participants) that reported motor function (Fugl‐Meyer Assessment). Meta‐analyses supported the conclusion that "there is some evidence that rehabilitation post‐stroke is more effective than no rehabilitation, improving activities of daily living and reducing disability." Zhang 2013 highlighted limitations relating to low reporting quality and study heterogeneity. Our updated review adds to this review, providing greater certainty that physical rehabilitation is more effective than no rehabilitation.

McGlinchey 2020 conducted a systematic review focussed on rehabilitation for physical function in people with severe stroke, including 28 trials (2677 participants) of interventions including early mobilisation and upper limb rehabilitation, as well as lower limb therapy. The review authors concluded that additional lower limb rehabilitation therapy improved ADL independence, but that this effect may not be sustained long‐term (at six months). Our findings are in agreement with this conclusion, with our review providing further information relating to the content and dose of the interventions.

## Authors' conclusions

**Implications for practice**

**Physical rehabilitation compared to no physical rehabilitation:** Physical rehabilitation, using a mix of different treatment components, appears more effective than no physical rehabilitation for recovery of function and mobility after stroke. There is low‐certainty evidence that, compared to no physical rehabilitation, physical rehabilitation may improve independence in ADL, motor function, and balance. There is moderate‐certainty evidence that physical rehabilitation likely improves gait speed. There is insufficient evidence to reach conclusions about the effects on length of stay or adverse events. There is some evidence of greater improvements in measures of activities of daily living when more than 2.5 hours/week of physical rehabilitation was delivered.

**Physical rehabilitation compared to attention control:** There is very low‐certainty evidence that, compared to attention control, physical rehabilitation may improve independence in ADL, and moderate‐certainty evidence that physical rehabilitation likely improves gait speed. However, there is very low‐certainty evidence that physical rehabilitation may *not* improve motor function or balance. There is insufficient evidence to reach conclusions about the effects on length of stay or adverse events.

**Additional physical rehabilitation:** There is low‐certainty evidence that additional rehabilitation (i.e. provided in addition to usual/conventional rehabilitation) may improve independence in ADL, motor function, balance, and gait speed. The greater the amount of additional physical rehabilitation, the greater the treatment effect may be. There is insufficient evidence to reach conclusions about the effects on length of stay or adverse events.

**Different approaches to physical rehabilitation:***Functional task training:* Physical rehabilitation focussing on functional task training (the active practice of real‐life tasks with the aim of acquiring, or re‐acquiring, a skill) comprises a mixture of treatment components, selected according to the individual needs of the patient, and does not align with any "named" rehabilitation approach. Physical rehabilitation that focusses on functional task training appears to be more effective than other approaches: there is low‐certainty evidence that functional task training may improve independence in ADL and very low‐certainty evidence that it may improve motor function. However, there was very low‐certainty evidence that functional task training may not improve gait speed or balance more than other approaches. *Neurophysiological approaches*: Neurophysiological approaches to physical rehabilitation are not superior to, and may be less effective than, other physical rehabilitation approaches: there is low‐certainty evidence that neurophysiological approaches may be less effective than other approaches for independence in ADL, low‐certainty evidence that neurophysiological approaches may be no different from other approaches for motor function and balance, and very low‐certainty evidence that neurophysiological approaches may be no different from other approaches for gait speed. Evidence is insufficient to support any conclusions about the effects of different approaches to physical rehabilitation on length of stay or adverse events.

Evidence relating to the dose of physical rehabilitation is limited by substantial heterogeneity and does not support robust conclusions. While the optimum amount of physical rehabilitation remains unknown, evidence does suggest that a greater dose may bring greater benefits, especially where the dose is more than 2.5 hours/week, and that intensive periods of physical rehabilitation may be beneficial even when delivered for a short period of time. Some limited evidence also suggests that the involvement of families/carers as part of the process of physical rehabilitation may bring positive impacts.

Members of the stakeholder group for this review discussed and agreed on the key implications for practice that they perceived as arising from this evidence (see Appendix 1).

**Implications for research**

Physical rehabilitation using a mixture of treatment components and focussed on functional task training appears beneficial, and additional ‐ or a higher dose of ‐ physical rehabilitation appears to bring greater benefit. However, the current evidence base remains limited; key factors limiting conclusions that can be supported by the current research studies include:

- Randomised controlled trials (RCTs) are generally small (often single‐centre studies, with a mean of only 82 participants (standard deviation 84.4)).
- Poor reporting of RCTs, including lack of published/publicly available protocols and failure to adhere to CONSORT reporting guidance.
- Lack of consistency between RCTs. Except for a body of trials investigating "Three‐stage rehabilitation", trials all address different questions (i.e. different treatment components, different doses and duration, different populations).

Further, many of the research studies explore interventions that may not be feasible to deliver in real‐world clinical settings. For example, delivering doses that are considerably higher than might be feasible in many settings.

These factors limit certainty in the evidence within this review, which is largely low to very low, due to substantial heterogeneity, with mainly small studies and important differences between populations and interventions in different studies.

This review synthesises the evidence in relation to function and mobility after stroke. Further small trials are unlikely to change the conclusions of this review. However, future research should consider the full range of outcomes that may be associated with improved function and mobility. These outcomes include the clinical and cost benefits potentially associated with a reduction in falls or emergency hospital admissions and the impact of community and social care teams and services. All benefits in relation to stroke survivor‐perceived quality of life, psychological mood, social participation, return to work, and carer strain and well‐being should be considered. This review found that many RCTs did not assess long‐term follow‐up, and it is essential that future RCTs plan follow‐up assessments as a key feature of their design. Adequate resources should be sought to ensure that follow‐up assessments are possible. Furthermore, future research should consider the effects of long‐term, follow‐up physiotherapy assessment, self‐management, and treatment in maintaining benefits and preventing deterioration, and the effects of shorter‐duration periods of intensive rehabilitation. Self‐referral systems that will enable stroke survivors to gain follow‐up physiotherapy when they believe it is necessary should also be explored. The potential impact of family involvement on rehabilitation outcomes was an important finding that should be more fully explored.

Continued conduct of multiple, heterogeneous, small, poorly reported RCTs is of very limited value and is potentially wasteful. There is an urgent need for researchers in this field to come together to find new, collaborative, innovative approaches to address the limitations in the current evidence. For future updates of this Cochrane review, authors and editors should consider pre‐stating minimum standards for reporting and minimum study size as additional selection criteria. However, given the size of this review, future updates of the review in its current form are likely to result in a review that risks being too large to be clinically useful for decision makers. We therefore advise against an update of this review in its current form, and urge key stakeholders to come together and reach consensus on the most important questions that need answering to inform optimal physical rehabilitation for stroke. Researchers and stakeholders should work in partnership to determine the optimal way to address these questions, which could potentially involve splitting the review into separate reviews, each exploring evidence for specific approaches, or conducting a network meta‐analysis.

A stakeholder group was central to this review update, and this update has demonstrated that user involvement in Cochrane reviews is feasible and valued and can significantly impact the review structure and methods. We recommend similar models of user involvement within other Cochrane reviews and evidence syntheses, and continued exploration to develop the best ways of involving people and optimal ways for reporting the method and impact of involving people.

## What's new

**Table:** 

**Date**	**Event**	**Description**
11 February 2025	New search has been performed	Review updated ‐ searches run in November 2022, with a new total number of 267 included studies (previously 96).
11 February 2025	New citation required and conclusions have changed	This update and the previous version both concluded that there is very low‐ to moderate‐certainty evidence that suggests physical rehabilitation, using a mix of different treatment components, may be effective for recovery of function and mobility after stroke. However, in a change to the previous version (which concluded that there was no evidence that any approach to physical rehabilitation was any more or less effective than another approach), this update concludes that the evidence suggests: functional task training may be more effective than other approaches (low‐ to very low‐certainty evidence); neurophysiological approaches may be less effective than other approaches (low‐ to very low‐certainty evidence). Further, in contrast to the previous version, which found insufficient evidence to support a conclusion relating to dose of physical rehabilitation, this update concludes that there is low‐certainty evidence that suggests: additional physical rehabilitation may provide added benefits; a dose of > 2.5 hours/day may bring greater benefits.

## History

Protocol first published: Issue 1, 2000 Review first published: Issue 2, 2003

**Table:** 

**Date**	**Event**	**Description**
24 August 2023	Amended	Correction to authors named in previously published amendment.
17 April 2023	Amended	Small typographical error in Plain Language Summary and results section fixed.
22 April 2014	New search has been performed	Title changed from "Physiotherapy treatment approaches for the recovery of postural control and lower limb function" to "Physical rehabilitation approaches for the recovery of function and mobility following stroke". We have updated the searches to December 2012. We included 79 new studies in this version: the review now has 96 included studies involving 10,401 participants
15 November 2013	New citation required and conclusions have changed	A substantial amount of new information has been included in this review. The conclusions of the review have changed since the previous version; the comparisons and the method of categorising interventions have also changed.
30 September 2008	Amended	Converted to new review format.
19 January 2006	New search has been performed	**2001 Version** 4114 trials from electronic searching 167 abstracts screened 71 full papers assessed 11 trials included (362 patients): Dean 1997; Dean 2000; Duncan 1998; Gelber 1995; Inaba 1973; Langhammer 2000; Pollock 1998; Richards 1993; Stern 1970; Wagenaar 1990; Wellmon 1997 Data for: four trials of neurophysiological versus other; four trials of motor learning versus other; four trials of mixed versus other; two comparisons of subgroups of the same approach. **2005 Update** 8408 (4294 new) trials from electronic searching 266 (99 new) abstracts screened 185 (114 new) full papers assessed 20 (11 new) trials included (1087 patients; 809 new). New trials: Duncan 2003, Green 2002, Hesse 1998, Howe 2005, Lincoln 2003, McClellan 2004, Mudie 2002, Ozdemir 2002, Salbach 2004, Wade 1992, Wang 2005a Trials comparing subgroups of the same approach were excluded (excluded Inaba 1973 and Wagenaar 1990, which were included in original version). Data for: eight (four new) trials of neurophysiological (all Bobath) versus other; eight (four new) trials of motor learning versus other; nine (five new) trials of mixed versus other.

## Risk of bias

**Table:** Risk of bias for analysis 1.1 Independence in ADL scales

**Study**	**Bias**											
	**Randomisation process**		**Deviations from intended interventions**		**Missing outcome data**		**Measurement of the outcome**		**Selection of the reported results**		**Overall**	
	**Authors' judgement**	**Support for judgement**	**Authors' judgement**	**Support for judgement**	**Authors' judgement**	**Support for judgement**	**Authors' judgement**	**Support for judgement**	**Authors' judgement**	**Support for judgement**	**Authors' judgement**	**Support for judgement**
**Subgroup 1.1.1 Barthel Index**												
Aravind 2022	Low risk of bias	"A Toronto‐based research assistant, unfamiliar with participants, prepared a list of randomization assignments for each site by flipping a coin (block size of 2)" and informed participants of group allocation by phone. Participants were stratified by site and gait speed There were differences between groups at baseline, but these were explored and accounted for in the analysis.	Low risk of bias	Participants were not blinded to their group allocation which could have resulted in a bias, especially on self‐report measures. Control group received no therapy; intervention was delivered after hospital discharge, limiting opportunties for contamination between groups. Participants were analysed according to the group to which they were randomised.	Low risk of bias	2 and 4 drop outs in intervention/follow up group .	Low risk of bias	"Trained evaluators, blinded to study hypotheses and group assignment"	Low risk of bias	Published protocol and trial registration available. Results reported as per pre‐published protocol.	Low risk of bias	Judged low risk of bias for all domains. Adequate randomisation and allocation concealment, blinded outcome assessor. Intervention delivered at home limiting opportunties for contamination between groups. Study protocol available.
Bai 2013	Low risk of bias	"Each patient was allocated by random number to the physiotherapy, acupuncture, or combined treatment group. In the randomization process, a research assistant opened envelopes containing random numbers and informed an acupuncturist or therapist if the patient was in the respective intervention group. The research assistant also informed physicians of the recruitment but not the group assignment. Additionally, she reminded the patients not to tell the physicians or the therapists whether they were treated with acupuncture." "Demographic characteristics (Table 1) were similar among the three groups, and gender, age, injured area, affected side, time from stroke onset, and comorbid conditions did not significantly differ among groups"	Low risk of bias	Patients told not to tell people delivering interventions whether they were in intervention or control arm.	Low risk of bias	No dropouts or missing data.	Low risk of bias	Assessors blinded to treatment group.	Some concerns	No protocol identified.	Some concerns	Low risk of bias for all domains, but no study protocol identified leaving concerns about selection of reported results.
Bek 2016	Low risk of bias	"Randomisation was performed by an independent administrator (who was not involved in outcome assessments), using an online randomisation tool, and the administrator informed patients and staff of group allocation"	Some concerns	Participants, carers and people delivering the intervention likely to be aware of assigned intervention group. Insufficient information to judge if there were deviations because of the trial context.	Some concerns	Data available for control: 32/36, intervention 30/41. "Reasons for dropping out included inability to commit (n=2), illness (n=1), transport problems (n=1), and participants not wishing to continue because they did not feel that the CE method suited them (n=2)". Higher number of dropouts in intervention group; although no indication that there were differences between those who did and did not drop‐out.	Some concerns	Method is Barthel Index. Unclear whether outcome assessors were blinded. No evidence to suggest that the assessment of the outcome might have been influenced by knowledge of the intervention received.	Low risk of bias	Protocol published in trials registry: ISRCTN84064492. No evidence of differences between protocol and final study.	Some concerns	Some concerns due to lack of information about intervention delivery, drop outs and blinded outcome assessment.
Chang 2015	Some concerns	States use of random number table, but no information about allocation concealment.	Some concerns	Insufficient information to judge if there were deviations from intended interventions.	Low risk of bias	No dropouts or missing data reported.	Some concerns	No mention of blinding. No evidence to suggest that assessment was influenced by knowledge of intervention.	Some concerns	No mention of protocol	Some concerns	Some concerns due to lack of information and lack of protocol.
Chen J 2014	Some concerns	States "randomly divided" but no further information. No significant difference between groups before treatment.	Some concerns	Insufficient information to judge whether there could be deviations due to study context. As interventions delivered on the ward, potentially could be contamination between study groups.	Low risk of bias	No evidence of drop outs or missing data.	Some concerns	No information relating to blinding of outcome assessor.	Some concerns	No evidence of a protocol.	Some concerns	Some concerns due to lack of information and lack of study protocol.
Chen S 2021	Some concerns	"Every patient who met the eligibility criteria was allotted a serial number by the computer with those having an odd number designated as the IG and those having an even number as the CG." Lack of information about allocation concealment. "There were no significant differences observed between the FMA scores in the IG and CG at 0 (the baseline	Low risk of bias	Control group had no therapy input. Intervention was delivered at home, following discharge from routine services, so contamination between groups unlikely.	Low risk of bias	"Of 164 patients enrolled in the study, 24 declined to participate, 11 were withdrawn after randomization (5 patients from the IG and 6 patients from the CG), five were lost to follow‐up (3 patients in the IG and 2 patients in the CG), and three refused to continue (3 patients in the IG and 0 patient in the CG)." The three "refusals" could be due to the demands of the intervention, however this is considered low.	Some concerns	"The outcome assessment for both groups were performed by one trained and certified nurse practitioner " No evidence of blinding. No evidence to suggest that assessment was influenced by knowledge of intervention.	Some concerns	No protocol available.	Some concerns	Some concerns due to lack of information about allocation concealment, no blinding and lack of a protocol.
Chu 2003	High risk of bias	States "cases were divided….randomly". Method of randomisation not mentioned . "There were significant differences in age, gender, lesion characteristics and side of paralysis between two groups".	Some concerns	Only one group received rehabilitation. No information to indicate if there may have been deviations from intended interventions (e.g. any rehabilitation carried out by no treatment group).	Low risk of bias	Reports 58 participants randomised. No drop outs reported, and it is assumed that there are no drop outs.	Some concerns	No evidence of blinding of outcome assessor; but no evidence that assessment was influenced by knowledge of the intervention received.	Some concerns	No protocol available.	High risk of bias	Method of randomisation not stated. Some concerns relating to possible deviations from the intended intervention, lack of blinding of outcome assessor and lack of protocol.
Dai 2015	Some concerns	States "randomly divided", but no details provided. "There was no significant difference in age, gender, onset time, and motor function between the two groups of patients, and they were comparable (P>0.05)"	Some concerns	Physio only given to intervention group. Insufficient details to judge if there could be deviations from the intended interventions.	Low risk of bias	"A total of 62 stroke patients admitted to our hospital from June 2013 to August 2014 were selected and randomly divided into a treatment group and a control group, with 31 cases in each group." No drop outs or missing data reported.	Some concerns	Not stated if outcome assessor was blinded.	Some concerns	No protocol available.	Some concerns	Some concerns due to lack of information.
Fang 2003	Low risk of bias	"Randomization was achieved through computer‐generated random numbers in sealed envelopes."	Low risk of bias	The control group received no physiotherapy, but did receive usual multidisciplinary care. "Therapists were blinded to patients’ groupings".	High risk of bias	78 participants randomised to each group. Treatment group had 28 drop outs; no treatment group had none. Authors report that the high drop out rate in the treatment group was due to participants not being able to tolerate the treatment, and acknowledge that the 28 missing participants probably had lower IADLI (Barthel Index) scores. It is therefore considered that the missing data is likely to contribute to bias in the outcome results.	Low risk of bias	Assessment performed by trained neurologists who were blinded to the grouping of the subjects.	Some concerns	No protocol available.	High risk of bias	High risk of bias due to missing data from participants who were unable to complete the intervention, and some concerns due to lack of protocol.
Fang YN 2004	High risk of bias	States "randomized" in English text, but no information provided in Chinese text. 50 in one group, 78 in other group. Reason for difference in group size not clear. Differences in gender balance between groups.	Some concerns	Lack of information.	High risk of bias	Group 1, n=50. Group 2, n=78. Outcome data available for n=45 and n=55. No information available relating to missing data.	Low risk of bias	"It was a blind experiment for the experimentor in charge of the evaluation" (translated from Chinese)	Some concerns	No protocol available.	High risk of bias	Insufficient information about randomisation and missing outcome data, and lack of information for other domains.
Green 2002	Low risk of bias	"Randomisation was achieved by numbered, sealed, opaque envelopes prepared from random number tables and used four length random permuted blocks." "Patients were assigned either community physiotherapy treatment (treatment group) or no treatment (controls) by an assistant, who was otherwise unconnected with the study." No baseline differences.	Low risk of bias	"Physiotherapy treatment was done by an established community physiotherapy service (13 staff) as part of their usual work". No contact between people in the treatment and control group.	Low risk of bias	Clear description of dropouts provided. 161/170 completed intervention.	Low risk of bias	No contact between physio and researcher who did the assessment. "Unmasking of treatment groups was assessed by the researcher guessing the trial group for every patient at the 3‐month assessment; we measured the agreement between guess and actual group allocation with the K statistic."	Some concerns	Reference to a protocol in contributors section, but this was not available.	Low risk of bias	Low risk of bias for all domains, with exception of bias in selection of the reported result. However there is evidence that a protocol was used to support this study, and it is considered likely that this outcome was pre‐specified.
Guo L 2012	Some concerns	States randomisation using a mathematical table. No information relating to allocation concealment.	Some concerns	Insufficient information to judge if there were deviations from intended intervention.	Low risk of bias	9 droputs from treatment group and 8 dropouts from control did not complete follow‐up visits. Were unable to get data from them. Drop outs are balanced between groups.	Some concerns	Blinding not reported	Some concerns	No reference to a protocol or ethics approval. No protocol identified.	Some concerns	Some concerns due to lack of information and lack of protocol.
Holmgren 2006	Low risk of bias	"Randomization of subjects into the intervention (IG) or control group (CG) was conducted with a minimization software program, MiniM (29) to avoid baseline risk factor imbalances between the two groups. Two variables were taken into account: cognition, using the Mini Mental State Examination, and fall risk, using the Fall Risk Index" "There were no significant differences in the baseline characteristics of the two groups except from the TOAST pathogenesis classification of ischemic stroke"	Low risk of bias	The investigation group (IG) and control group (CG) did not meet. The CG attended education sessions, but these were "about communication difficulties, fatigue,depressive symptoms, mood swings, personality changes and dysphagia, all more or less hidden dysfunctions after stroke and how to cope with these difficulties" and did not have a focus on physical rehabilitation. "For each participant in the IG and the CG and each session, there was a journal filled out by the care providers in each group. The IG contained information about all the chosen exercises, number of exercises, level of exercises and the participants feeling of exertion during the different exercises. The CG contained information on level of motivation and activity during the discussions"	Low risk of bias	All but one subject completed the entire program.	Low risk of bias	All assessments were done by blinded staff, who were instructed that if they had any reason to believe that they had revealed a subject’s group they should make an adverse event report.	Some concerns	The study protocol was approved by the Regional Ethical Review board for Human Research at Ume å University (Dnr 04‐022), but we were unable to access this for this review.	Some concerns	Low risk of bias for all domains, with exception of bias in selection of the reported result.
Hoseinabadi 2013	High risk of bias	States "randomly divided into two equal groups" but no detail on how randomisation performed. Also reports the study design as "quasi‐experimental" but it is not clear if this relates to randomisation or not. No information relating to allocation concealment. "The two groups were not significantly different from each other in terms of balance, muscle tonicity and quality of life in pre‐test evaluation." Assessors judgement changed to high risk of bias due to risk that assignment to groups was done using a quasi‐random method.	Some concerns	Insufficient information to judge if there could be deviations from the intended interventions.	Low risk of bias	No evidence of drop outs or missing data.	Some concerns	No information relating to blinding of outcome assessor, or information about who conducted the outcome assessments.	Some concerns	No protocol obtained.	High risk of bias	Judged as high risk of bias for randomisation as study is described as "quasi‐experimental" and, although it is reported that participants were "randomly divided", it is unclear if there was quasi‐randomisation. Some concerns due to lack of information and lack of protocol.
Hou 2006	Some concerns	States random, but no information provided. No significant difference (P value > 0.05) for gender, age, days after stroke, type of stroke and Brunnstrom score for upper limb, hand and lower limb at baseline.	High risk of bias	No information provided.	Low risk of bias	80 participants randomised and with data available.	Low risk of bias	Outcome assessors did not deliver intervention.	Some concerns	No protocol available.	High risk of bias	Lack of information about randomisation and delivery of interventions; no protocol available.
Hou Zhi 2014	Some concerns	States "randomly divided" but no further information about randomisation process. There was no significant difference in general data such as age, gender and course of disease between the two groups (P>0.05), and they were comparable.	Some concerns	Insufficient information to judge if there were deviations because of trial context.	Low risk of bias	No evidence of drop outs or missing data.	Some concerns	Blinding not discussed	Some concerns	No protocol identified.	Some concerns	Some concerns due to lack of information and lack of protocol.
Huang 2003	Low risk of bias	Stratified randomisation. No obvious difference between groups for time since stroke, gender, age, side and type of stroke, etc	Some concerns	Insufficient details.	Low risk of bias	No dropouts or missing data reported.	Low risk of bias	Outcome assessors did not deliver intervention and were blinded to group allocation.	Some concerns	No protocol available.	Some concerns	Insufficient details about some aspects of the study, and no protocol available.
Huang 2014	Some concerns	States randomly divided, but no description of randomisation process. The MBI scores were significantly higher than those before treatment (P<0.05), and the MBI scores of the four technical groups were significantly higher than those of the control group (P<0.05), but there was no significant difference in the FMA scores between the five groups after treatment (P<0.05). P> 0.05), there was no significant difference in MBI score among the four technical groups (P>0.05).	Some concerns	Insufficient information to judge if there were deviations from intended interventions.	Low risk of bias	No evidence of dropouts or missing data.	Some concerns	Blinding not discussed.	Some concerns	No reference to a protocol	Some concerns	Some concerns due to lack of information and lack of protocol.
Huang 2014	Some concerns	States randomly divided, but no description of randomisation process. The MBI scores were significantly higher than those before treatment (P<0.05), and the MBI scores of the four technical groups were significantly higher than those of the control group (P<0.05), but there was no significant difference in the FMA scores between the five groups after treatment (P<0.05). P> 0.05), there was no significant difference in MBI score among the four technical groups (P>0.05).	Some concerns	Insufficient information to judge if there were deviations from intended interventions.	Low risk of bias	No evidence of dropouts or missing data.	Some concerns	Blinding not discussed.	Some concerns	No reference to a protocol	Some concerns	Some concerns due to lack of information and lack of protocol.
Huang 2014	Some concerns	States randomly divided, but no description of randomisation process. The MBI scores were significantly higher than those before treatment (P<0.05), and the MBI scores of the four technical groups were significantly higher than those of the control group (P<0.05), but there was no significant difference in the FMA scores between the five groups after treatment (P<0.05). P> 0.05), there was no significant difference in MBI score among the four technical groups (P>0.05).	Some concerns	Insufficient information to judge if there were deviations from intended interventions.	Low risk of bias	No evidence of dropouts or missing data.	Some concerns	Blinding not discussed.	Some concerns	No reference to a protocol	Some concerns	Some concerns due to lack of information and lack of protocol.
Ji Pei 2014	Some concerns	States "divided according to the random number table method." No further information. There was no significant difference in gender, age, infarction site, and degree of limb paralysis between the two groups (P>0.05).	Some concerns	Insufficient information to judge if there were deviations from intended interventions.	Low risk of bias	No evidence of any dropouts or missing data.	Some concerns	Blinding not discussed.	Some concerns	No protocol identified.	Some concerns	Some concerns due to lack of information and lack of protocol.
Koç 2015	Low risk of bias	"Before beginning the study, a random list was generated for the group assignments. Randomisation was done in blocks of five. Only a medical secretary who had no input into subject selection or recruitment was aware of the group assignments. After the baseline assessments, the medical secretary assigned each subject ot either the experiement or control group".	Low risk of bias	States single‐blind study, implying that participants were unaware of allocation. The intervention was home‐based limiting opportunities for contamination between groups.	High risk of bias	Abstract states that 134 patients were enrolled, from a "screened sample of 765 patients" and "72 patients completed the study". The full paper only reports that 72 patients were studied. No information about drop‐outs or missing data are reported.	Some concerns	Not stated if outcome assessor was blinded.	Some concerns	No protocol available.	High risk of bias	High risk of bias due to absence of information about a potential 47% drop‐out (62/134). Some concerns due to lack of information about blinding, and no protocol.
Li 1999	High risk of bias	Lack of information about randomisation. Early rehabilitation group had a higher co‐morbidity score than the control group at baseline. No difference for age and past history rating between groups. No mention of other variables tested for baseline differences.	Some concerns	Lack of information.	Some concerns	Lack of information.	Low risk of bias	"assessor blinded to the treatment allocation assessed the subjects at four time intervals"	Some concerns	No protocol available.	High risk of bias	Lack of information for most domains. Concern about bias arising from randomisation process.
Li Yuanzheng 2014	Some concerns	States "randomly divided". No further information provided. "There was no significant difference in general data between the two groups (0.05)."	Some concerns	Insufficient information to judge if there were deviations because of the trial context.	Low risk of bias	122 participants randomised. Data available for all participants.	Some concerns	No information relating to blinding of outcome assessor.	Some concerns	No protocol available.	Some concerns	Some concerns due to lack of information and no study protocol.
Lu 2004	Some concerns	No discussion around randomisation process. No significant difference in general data between the groups.	Some concerns	Insufficient information to judge if there were deviations due to trial context.	Low risk of bias	No evidence of drop outs or missing data.	Some concerns	Not clear if blinded assessors.	Some concerns	No mention of protocol or ethics. No protocol obtained.	Some concerns	Some concerns due to lack of information and lack of protocol.
Lu Liangyan 2014	Some concerns	States "randomly divided" but no information on randomisation procedure. No evidence of any baseline differences.	Some concerns	Insufficient information to judge if there could be deviations due to trial context.	Low risk of bias	No evidence of any dropouts or missing data.	Some concerns	No information relating to blinding of outcome assessor.	Some concerns	No protocol identified.	Some concerns	Some concerns due to lack of information and lack of protocol.
Pan 2004	Some concerns	Method of randomisation: "by participants drawing lots". No further information.	High risk of bias	Lack of information	Low risk of bias	96 participants randomised and with data available.	Some concerns	No information about blinding.	Some concerns	No protocol identified	High risk of bias	Lack of information to support judgements.
Pang 2006	High risk of bias	"according to the enrolled sequence with 3 cases in 1 group". No further details provided.	High risk of bias	Implementation of study design was done by first study author, potentially introducing bias. Lack of further information.	Low risk of bias	80 participants randomised. Data available for 78.	High risk of bias	Authors assessed outcome. Unclear if blinded.	Some concerns	No protocol available.	High risk of bias	Concerns about randomisation, and lack of blinding. Lack of information about potential deviations from the intended interventions and no protocol available.
Wade 1992	Some concerns	Method of randomisation: permuted blocks of 10, using random number tables. No information about allocation concealment. No information provided about potential baseline differences.	Low risk of bias	Treatment group received community physiotherapy, control group got no physical rehabilitation. Participants were in the community so there was no contact between groups. Participants were analysed according to the group to which they were randomised.	Low risk of bias	89/94 completed intervention and had three‐month follow‐up. All participants included in the analysis unless they died or had not reached last follow‐up point	Some concerns	"Once accepted into the trial, each patient was assessed by an independent (non‐treating) physiotherapist...." "Of course, patients and relatives often mentioned whether or not the treating therapist had visited....." Although unblinding may have occured in some patients, the treating therapist was independent, so not considered likely that outcome was influenced.	Some concerns	No protocol available.	Some concerns	Some concerns about allocation concealment, unblinding of outcome assessor, and lack of study protocol.
Wan Xueli 2014	Some concerns	States randomisation using random number table method, but no further information. There was no significant difference in age, gender and disease condition between the two groups (P>0.05).	Some concerns	Insufficient information to judge if deviations due to study context.	Low risk of bias	No evidence of drop outs or missing data.	Some concerns	Not clear if assessors were blinded or not.	Some concerns	No protocol available/obtained.	Some concerns	Some concerns due to lack of information and lack of study protocol.
Wang 2013	Some concerns	States "randomly divided" but no further information. There was no significant difference in general conditions such as age, disease type and past history between the two groups, P>0.05	Some concerns	Insufficient information to judge if deviations due to study context.	Low risk of bias	No evidence of any dropouts or missing data.	Some concerns	Blinding not discussed.	Some concerns	No protocol available.	Some concerns	Some concerns due to lack of information and lack of protocol.
Wang 2015	Low risk of bias	"Each approved patient was asked to draw a folded piece of paper marked with a computer‐generated random number from a bag. Patients were assigned to groups per the number drawn. The trial was initiated immediately following group assignment."	Low risk of bias	"Patients in the control group maintained their everyday routines but also received weekly visits or telephone calls by the therapist to talk about their rehabilitation progress, daily activities, and general health conditions. However, patients in the control group were not given specific instructions or guidance related to rehabilitation skills." Intervention was delivered in patient's homes, limiting opportunities for contamination between groups. Participants were analysed according to the group to which they were randomised.	Low risk of bias	No drop outs or missing data.	Low risk of bias	"All outcome measurements were evaluated by an independent physical therapist who was blinded from knowing the treatment assignment."	Some concerns	No protocol obtained.	Some concerns	Low risk of bias for most domains; no study protocol obtained.
Wang 2022	Low risk of bias	"This is a single assessor blind parallel group, and one‐site RCT of rehabilitation nursing in acute ischaemic stroke. When patients are admitted to the hospital, the wCST‐LL is determined first and then patients are divided into a ‘large’ group (wCST‐LL is greater than or equal to 2mL) or ‘small’ group (wCST‐LL is less than 2mL). Then, patients are randomly assigned to an experimental or control group according to a computer‐generated random number". "After stratifying into large and small groups by wCST‐LL, each group of patients will be randomly divided into an experimental group and a control group by a computer ‐ generated random number method. After eligible patients sign an informed consent, a trained and qualified assessor, blinded for the implementation of the intervention, will conduct the baseline assessments. Patients will be assigned a number according to the order of baseline assessment, and each ordinal number will correspond to a random number. Due to the characteristics of the face‐to‐face treatment, patients may know their grouping and those who deliver the intervention cannot achieve blind ‐ ness, thus, it is a single assessor‐ blinded study" "No statistically significant between‐group differences were observed at baseline"	Low risk of bias	"Due to the characteristics of the face‐to‐face treatment, patients may know their grouping and those who deliver the intervention cannot achieve blind ‐ ness" "There was no lack of information in the rehabilitation record form, and no training was reported to be inconsistent with the interven‐ tion program during the weekly random checks conducted by head nurses. Thus, fidelity of nurses in the delivery of intervention was well ensured." "Our descriptive statistics will include means and SDs for continuous variables and numbers and proportions for categorical variables. We will conduct a non‐inferiority test between the intervention and control groups for the primary and secondary outcomes without regard to the wCST‐LL."	Low risk of bias	104 randomised; 101 baseline assessment; 88 with data in data analysis. Reasons for drop outs / missing data are balanced between groups.	Low risk of bias	Barthel Index Blinded outcome assessor.	Low risk of bias	Trial registration NCT03702452. Protocol published, including analysis plan. Analysis followed pre‐specified plan.	Low risk of bias	No concerns. Single blind randomised study following pre‐specified protocol.
Wu 2006	Some concerns	No details provided relating to randomisation process. No significant baseline differences.	High risk of bias	Lack of information	Low risk of bias	Dropouts n=4 attributed to n=3 deaths and n=1 failure to attend assessment. Data available for remaining participants.	Some concerns	Lack of information.	Some concerns	No protocol available.	High risk of bias	Lack of information to support judgements for most domains.
Wu 2020	Low risk of bias	"The randomization list was generated using a computer randomizer (https://www.randomizer.org). The randomization was conducted after baseline assessment." "There was no statistical difference in baseline data between the two groups, so the results after the intervention are comparable."	Low risk of bias	The intervention occurred after hospital discharge, so contamination between the treatment groups was unlikely. Participants were analysed according to the group to which they were randomised.	Low risk of bias	32 patients in each group. 2 cases were dropped from the intervention group and 1 case from the control group in early stages. Very few drop outs and balance between groups, so judged unlikely to bias result.	Low risk of bias	Barthel Index. "Assessed by two rehabilitation therapists who were blinded to the grouping."	Some concerns	Study was prospectively registered on a trial register (ChiCTR1800018934), however this only lists primary outcomes and does not state ADL as an outcome. Unclear if there is selection relating to the reporting of Barthel Index as a secondary outcome.	Some concerns	Low risk of bias for all domains except selection of reported results. A study protocol was published, but this did not list Barthel Index as an outcome measure.
Xie 2003	Some concerns	1.1 No information provided 1.3 ‘No significant difference in age or score of neural function assessment could be found between the two groups’	High risk of bias	2.1 No information provided 2.3 Possibly same therapist across 2 groups, providing potential for contamination. (Lack of information)	Low risk of bias	64 participants randomised with data available for all. No drop outs or missing data reported.	Some concerns	Lack of information.	Some concerns	No protocol available.	High risk of bias	Lack of information to support judgements.
Xu 1999	Some concerns	Abstract only. Very little information	High risk of bias	Lack of information.	Low risk of bias	62 participants randomised and with data available. No dropouts or missing data reported.	High risk of bias	Lack of information.	Some concerns	No protocol available.	High risk of bias	Lack of information available.
Xu 2003a	Some concerns	Randomisation method not stated ‘There was no obvious difference between two groups (P>0.05)’	High risk of bias	Lack of information.	Low risk of bias	"186 patients were randomly divided into rehabilitation group (n = 94) and control group (n = 92)". Data available for all participants.	Some concerns	Lack of information.	Some concerns	Protocol not available.	High risk of bias	Lack of information for most domains.
Xu 2003b	Some concerns	1.1. No information on randomisation process 1.3 Baseline demographics similar across both groups	High risk of bias	Lack of information (abstract only).	Low risk of bias	180 participants randomised and have data available.	Some concerns	Lack of information.	Some concerns	No protocol available.	High risk of bias	Lack of information to support judgements (abstract only).
Xu 2004	Some concerns	1.1 method of randomisation not stated 1.3 No significant baseline differences.	High risk of bias	Lack of information.	Low risk of bias	57 participants randomised and with data available.	Some concerns	Lack of information.	Some concerns	No protocol available.	High risk of bias	Lack of information to support judgements for all domains.
Xu 2022	Some concerns	States "randomly divided" but no further information. "There was no statistically significant difference in the treatment of stroke patients between the two groups. X2=2.7778, P=2.4198."	High risk of bias	Nurses were delivering rehabilitation to one group and not to another, within an acute stroke setting. Families were involved in delivering the rehabilitation to the treatment group. There is therefore potential for deviations due to contamination between groups Reporting within this published study appears to have some anomolies. There is a section reporting "establishment of a stroke model", including statistical formulae, but it is not clear how this relates to the reported study.	High risk of bias	160 participants randomised; presents demographics for 160; but results data available for 164. No explanation provided for this difference.	High risk of bias	Does not state blinded outcome assessor. If outcomes were assessed by nurses involved in the study, there is potential for influence by knowledge of treatment group.	Some concerns	No protocol identified.	High risk of bias	This study is considered high risk of bias due to some anomolies in reporting, potential for deviations from the intended interventions and bias in the measurement of the outcome.
Xue 2006	Some concerns	No information provided ‘the baseline data were comparable between the two groups, and there were no significant differences (P>0.05)	High risk of bias	Lack of information.	Low risk of bias	"All the 150 patients with post‐stroke hemiplegia were involved in the analysis, no one missed".	High risk of bias	Outcome assessor not blinded. "Patients were evaluated by the professional group (the fourth and fifth authors) before treatment and 1 month after treatment respectively".	Some concerns	No protocol available.	High risk of bias	_Lack of information to support judgements, and concerns about potential deviaions from intended interventions and lack of blinding of outcome assessor._
Yan 2002	Some concerns	1.1 Randomisation ‐ No information provided. 1.3 No significant baseline differences.	High risk of bias	Lack of information.	Low risk of bias	78 participants randomised with data available.	Some concerns	No information as to whether outcome assessors blinded	Some concerns	No protocol available.	High risk of bias	Lack of information to support judgements.
Yang Aiguo 2015	Some concerns	States randomly divided but no discussion of randomisation process. No statistically significant difference between groups	Some concerns	Insufficient information to judge if there were deviations due to trial context.	Low risk of bias	No evidence of any dropouts or missing data.	Some concerns	No discussion of blinding.	Some concerns	No reference to protocol.	Some concerns	Some concerns due to lack of information and lack of protocol.
Zhang 1998	Some concerns	Randomisation method not stated ‐ ‘participants were randomly allocated to 2 groups’ Limited information available regarding baseline demographics although the BI and FMA scores at baseline are similar.	High risk of bias	Intervention delivered in ward, with control (no treatment) group in same ward. Patient family were encouraged to be involved in delivery of physical rehabilitation to treatment group. There is a possibility of contamination between groups (e.g. with patients in no treatment group being delivered physical rehabilitation exercises by family members, having observed or spoken with other families).	Low risk of bias	56 participants randomised with data available for all; no drop outs or missing data reported.	Some concerns	Lack of information.	Some concerns	No protocol available.	High risk of bias	Lack of information for most domains, with concerns of potential risk of contamination between groups.
Zhang 2004	Some concerns	No information as to how randomised. No statistical information provided.	Low risk of bias	The control group were discharged home, limiting potential for contamination between treatment groups.	High risk of bias	19 deaths and 157 dropout out of 800+ participants. (SARS epidemic prevented follow‐up in "more than 50 percent of the 157 cases" and no information reported in the remaining cases)	Some concerns	No mention of blinding of assessors	Some concerns	No protocol available.	High risk of bias	Lack of information available for some domains and concern over missing outcome data.
Zhang Jianhong 2013	Some concerns	States randomised but no discussion of randomisation process. Two groups seemed comparable at baseline.	Some concerns	Insufficient information to judge if deviations from intended intervention.	Low risk of bias	No evidence of drop outs or missing data.	Some concerns	Blinding of assessors not discussed	Some concerns	No protocol	Some concerns	Some concerns due to lack of information and lack of protocol.
Zhao 2003	Some concerns	1.1 Abstract only ("All the patients were divided into two groups randomly") 1.3 ‘There is no difference in age, gender and side of hemiplegia between groups’	High risk of bias	Lack of information.	Some concerns	Lack of information	Some concerns	Lack of information	Some concerns	No protocol available.	High risk of bias	Lack of information for all domains.
Zhao Ailiang 2016	Some concerns	States randomised. Lack of information about allocation concealment. No differences between groups at baseline.	Some concerns	Insufficient information to judge if there were deviations from intended interventions because of trial context. Participants were analysed according to assigned intervention.	Low risk of bias	No drop outs or missing data reported.	High risk of bias	Not clear if outcome assessor was blinded. Insufficient information to judge if outcome could have been influenced by knowledge of intervention received.	Some concerns	No protocol obtained.	High risk of bias	This is a Chinese language trial, with sections translated by one member of our team. There is generally a lack of information about the study methods. It is not clear whether the outcome assessor was blinded.
Zheng 2014	Some concerns	Randomisation process not descripbed. No differences at baseline.	Some concerns	Insufficient information to judge if there were deviations from intended interventions.	Low risk of bias	No evidence of dropouts or missing data.	Some concerns	Assessor blinding not discussed.	Some concerns	No protocol identified.	Some concerns	Some concerns due to lack of information and lack of protocol.
Zhu 2006	High risk of bias	English abstract states "divided...randomly and equally", but Chinese language indicates that method of randomisation was "according to the time of hospital admission". Unclear if this method meets the criteria for random allocation. No statistical significant differences in baseline values.	Some concerns	Lack of information.	Low risk of bias	70 participants randomised and data available for 70.	Some concerns	Lack of information. Unclear if blinded outcome assessor.	Some concerns	No protocol available.	High risk of bias	Serious concerns about randomisation process and lack of information for other domains.
Zhu 2007	Some concerns	“Stratified block randomisation” (divided by type of stroke before allocation to intervention or control groups) No information about allocation concealment. No statistical significant differences in baseline values.	High risk of bias	For some patients who returned to community setting, therapists would conduct weekly home‐visits to guide the patient on rehabilitative treatment. (3) Once training had commenced, therapists concurrently taught the patients’ family members or caregiver on the correct assistive training methods and care methods, such that they could provide some training outwith therapy time, while also reducing the secondary damage due to inappropriate care. (4) Patients learned to monitor their own body for discomfort, and report on time to therapist and caregiver. Potential for contamination between groups for patients on the ward. No attempt to record therapy provided by family members to either group. Control group ‐ therapy delivered by family members: ""Control group was not given standard rehabilitative treatment, but were allowed to perform activities independently under doctor’s advice or with assistance from nurses". Both groups were potentially receiving therapy delivered by family members after hospital discharge; but within the trial context, this should only have occured in the treatment group.	Low risk of bias	78 participants randomised and data available for 78. No standard deviations were available, so standard deviations have been estimated from ranges.	Low risk of bias	Blinded assessors.	Some concerns	No protocol available.	High risk of bias	Judged to be high risk of contamination between groups; some concerns as lack of information about randomisation and no protocol available.
**Subgroup 1.1.2 Other ADL measure**												
Guo Z 2015	Some concerns	States "randomly divided" but no details around randomisation process. "There was no significant difference in clinical data between the two groups ( P > 0. 05 ) , and they were comparable".	Low risk of bias	Interventions were delivered after hospital discharge, limiting opportunity for contamination between groups.	Low risk of bias	No evidence of drop outs or missing data.	Some concerns	Blinding not discussed.	Some concerns	No protocol identified.	Some concerns	Some concerns due to lack of information and lack of protocol.
Yan 2015	Some concerns	Divided "according to the random number table method". Lack of information relating to allocation concealment. No statistically significant differences at baseline.	Some concerns	Insufficient information to judge if there may have been deviations from the intended interventions. There may have been potential for contamination between groups during the hospital phase, but no information given on who provided the intervention to the different groups.	Low risk of bias	No drop outs or missing data reported.	High risk of bias	No information relating to who completed the outcome assessments and whether they were blinded.	Some concerns	No protocol identified.	High risk of bias	Concerns due to lack of information relating to allocation concealment and outcome assessment, with lack of details also for other domains.

**Table:** Risk of bias for analysis 1.2 Motor function scales

**Study**	**Bias**											
	**Randomisation process**		**Deviations from intended interventions**		**Missing outcome data**		**Measurement of the outcome**		**Selection of the reported results**		**Overall**	
	**Authors' judgement**	**Support for judgement**	**Authors' judgement**	**Support for judgement**	**Authors' judgement**	**Support for judgement**	**Authors' judgement**	**Support for judgement**	**Authors' judgement**	**Support for judgement**	**Authors' judgement**	**Support for judgement**
**Subgroup 1.2.1 Fugl Meyer**												
Bai 2013	Low risk of bias	"Each patient was allocated by random number to the physiotherapy, acupuncture, or combined treatment group. In the randomization process, a research assistant opened envelopes containing random numbers and informed an acupuncturist or therapist if the patient was in the respective intervention group. The research assistant also informed physicians of the recruitment but not the group assignment. Additionally, she reminded the patients not to tell the physicians or the therapists whether they were treated with acupuncture." "Demographic characteristics (Table 1) were similar among the three groups, and gender, age, injured area, affected side, time from stroke onset, and comorbid conditions did not significantly differ among groups"	Low risk of bias	Patients told not to tell people delivering interventions whether they were in intervention or control arm.	Low risk of bias	No dropouts or missing data.	Low risk of bias	Assessors blinded to treatment group.	Some concerns	No protocol identified.	Some concerns	Low risk of bias for all domains, but no study protocol identified leaving concerns about selection of reported results.
Chen J 2014	Some concerns	States "randomly divided" but no further information. No significant difference between groups before treatment.	Some concerns	Insufficient information to judge whether there could be deviations due to study context. As interventions delivered on the ward, potentially could be contamination between study groups.	Low risk of bias	No evidence of drop outs or missing data.	Some concerns	No information relating to blinding of outcome assessor.	Some concerns	No evidence of a protocol.	Some concerns	Some concerns due to lack of information and lack of study protocol.
Chen S 2021	Some concerns	"Every patient who met the eligibility criteria was allotted a serial number by the computer with those having an odd number designated as the IG and those having an even number as the CG." Lack of information about allocation concealment. "There were no significant differences observed between the FMA scores in the IG and CG at 0 (the baseline	Low risk of bias	Control group had no therapy input. Intervention was delivered at home, following discharge from routine services, so contamination between groups unlikely.	Low risk of bias	"Of 164 patients enrolled in the study, 24 declined to participate, 11 were withdrawn after randomization (5 patients from the IG and 6 patients from the CG), five were lost to follow‐up (3 patients in the IG and 2 patients in the CG), and three refused to continue (3 patients in the IG and 0 patient in the CG)." The three "refusals" could be due to the demands of the intervention, however this is considered low.	Some concerns	"The outcome assessment for both groups were performed by one trained and certified nurse practitioner " No evidence of blinding.	Some concerns	No protocol available.	Some concerns	Some concerns due to lack of information about allocation concealment, no blinding and lack of a protocol.
Cheng 2021	Some concerns	States "randomly divided" but randomisation and allocation concealment process not discussed.	Some concerns	Patients either received physical rehabilitation or no physical rehabilitation. It is unclear if patients were on the same ward. If so, there would be potential for contamination between groups, with family members of control group members observing (and then potentially implementing) interentions.	Low risk of bias	156 patients randomised and data available for 156 for Fugl Meyer score.	Low risk of bias	Double‐blinded evaluation.	Some concerns	No protocol identified.	Some concerns	Some concerns due to lack of information about randomisation, implementation of treatment and lack of protocol.
Chu 2003	High risk of bias	States "cases were divided….randomly". Method of randomisation not mentioned . "There were significant differences in age, gender, lesion characteristics and side of paralysis between two groups".	Some concerns	Only one group received rehabilitation. No information to indicate if there may have been deviations from intended interventions (e.g. any rehabilitation carried out by no treatment group).	Low risk of bias	Reports 58 participants randomised. No drop outs reported, and it is assumed that there are no drop outs.	Some concerns	No evidence of blinding of outcome assessor; but no evidence that assessment was influenced by knowledge of the intervention received.	Some concerns	No protocol available.	High risk of bias	Method of randomisation not stated. Some concerns relating to possible deviations from the intended intervention, lack of blinding of outcome assessor and lack of protocol.
Dai 2015	Some concerns	States "randomly divided", but no details provided. "There was no significant difference in age, gender, onset time, and motor function between the two groups of patients, and they were comparable (P>0.05)"	Some concerns	Physio only given to intervention group. Insufficient details to judge if there could be deviations from the intended interventions.	Low risk of bias	"A total of 62 stroke patients admitted to our hospital from June 2013 to August 2014 were selected and randomly divided into a treatment group and a control group, with 31 cases in each group." No drop outs or missing data reported.	Some concerns	Not stated if outcome assessor was blinded.	Some concerns	No protocol available.	Some concerns	Some concerns due to lack of information.
Deng 2011	Some concerns	"Based on the unbalance index minimum principle, age, nature of lesion, side of lesion, commencement of treatment time and cognitive deficits, 100 patients were randomly divided" (translated from Chinese) No information about allocation concealment.	Some concerns	Intervention was initially delivered on the ward (continued at home after discharge). Both groups received "conventional therapy". Could potentially be contamination between groups, with treatment group interventions being delivered to control group.	Low risk of bias	100 participants randomised and data available for 100.	Some concerns	No information relating to blinding.	Some concerns	No protocol identified.	Some concerns	Some concerns due to lack of information for most domains, and lack of protocol.
Fan WK 2006	Some concerns	Study design: “RCT with 2 treatment groups” Method of randomisation: stratified by the type of stroke, ischaemic or haemorrhagic, into the two groups. No information provided about allocation concealment. Study commented no obvious difference in baseline characteristics outlined in Table 1.	Some concerns	Insufficient information to judge if there could be deviations from intended intervention.	Low risk of bias	82 randomised; data available for 79	Low risk of bias	simplified Fugl‐Meyer motor function score All outcome assessment was undertaken by the same assessor, assessor did not deliver any therapy.	Some concerns	No protocol identified	Some concerns	Some concerns due to lack of information relating to allocation concealment, delivery of intervention, and no pre‐specified protocol.
Fang 2003	Low risk of bias	"Randomization was achieved through computer‐generated random numbers in sealed envelopes."	Low risk of bias	The control group received no physiotherapy, but did receive usual multidisciplinary care. "Therapists were blinded to patients’ groupings".	High risk of bias	78 participants randomised to each group. Treatment group had 28 drop outs; no treatment group had none. Authors report that the high drop out rate in the treatment group was due to participants not being able to tolerate the treatment. It is therefore considered that the missing data is likely to contribute to bias in the outcome results.	Low risk of bias	Assessment performed by trained neurologists who were blinded to the grouping of the subjects.	Some concerns	No protocol available.	High risk of bias	High risk of bias due to missing data from participants who were unable to complete the intervention, and some concerns due to lack of protocol.
Fang YN 2004	High risk of bias	States "randomized" in English text, but no information provided in Chinese text. 50 in one group, 78 in other group. Reason for difference in group size not clear. Differences in gender balance between groups.	Some concerns	Lack of information.	High risk of bias	Group 1, n=50. Group 2, n=78. Outcome data available for n=45 and n=55. No information available relating to missing data.	Low risk of bias	"It was a blind experiment for the experimentor in charge of the evaluation" (translated from Chinese)	Some concerns	No protocol available.	High risk of bias	Insufficient information about randomisation and missing outcome data, and lack of information for other domains.
Guo L 2012	Some concerns	States randomisation using a mathematical table. No information relating to allocation concealment.	Some concerns	Insufficient information to judge if there were deviations from intended intervention.	Low risk of bias	9 droputs from treatment group and 8 dropouts from control did not complete follow‐up visits. Were unable to get data from them. Drop outs are balanced between groups.	Some concerns	Blinding not reported	Some concerns	No reference to a protocol or ethics approval. No protocol identified.	Some concerns	Some concerns due to lack of information and lack of protocol.
Guo Z 2015	Some concerns	States "randomly divided" but no details around randomisation process. "There was no significant difference in clinical data between the two groups ( P > 0. 05 ) , and they were comparable".	Low risk of bias	Interventions were delivered after hospital discharge, limiting opportunity for contamination between groups.	Low risk of bias	No evidence of drop outs or missing data.	Some concerns	Blinding not discussed.	Some concerns	No protocol identified.	Some concerns	Some concerns due to lack of information and lack of protocol.
Hou Zhi 2014	Some concerns	States "randomly divided" but no further information about randomisation process. There was no significant difference in general data such as age, gender and course of disease between the two groups (P>0.05), and they were comparable.	Some concerns	Insufficient information to judge if there were deviations because of trial context.	Low risk of bias	No evidence of drop outs or missing data.	Some concerns	Blinding not discussed	Some concerns	No protocol identified.	Some concerns	Some concerns due to lack of information and lack of protocol.
Hu 2007	Some concerns	"Random grouping method: This study involves the rehabilitation medicine departments of 22 affiliated hospitals of medical colleges or provincial hospitals. Each department is randomly grouped according to the random number table. The selected patients are stratified according to cerebral infarction and cerebral hemorrhage, and then each division The groups were randomized to either the rehabilitation group or the control group." No information about allocation concealment.	Some concerns	No information about who delivered the interventions, or whether there could have been deviations or contamination between groups.	High risk of bias	Drop outs not accounted for. 1 month: Group 1: 485/688 group 2 480/677.	Low risk of bias	Outcome assessors blinded.	Some concerns	No protocol identified.	High risk of bias	High risk of bias due to concerns about missing outcomes (drop outs not accounted for). Some concerns due to lack of information for other domains.
Huang 2003	Low risk of bias	Stratified randomisation. No obvious difference between groups for time since stroke, gender, age, side and type of stroke, etc	Some concerns	Insufficient details.	Low risk of bias	No dropouts or missing data reported.	Low risk of bias	Outcome assessors did not deliver intervention and were blinded to group allocation.	Some concerns	No protocol available.	Some concerns	Insufficient details about some aspects of the study, and no protocol available.
Huang 2014	Some concerns	States randomly divided, but no description of randomisation process. The MBI scores were significantly higher than those before treatment (P<0.05), and the MBI scores of the four technical groups were significantly higher than those of the control group (P<0.05), but there was no significant difference in the FMA scores between the five groups after treatment (P<0.05). P> 0.05), there was no significant difference in MBI score among the four technical groups (P>0.05).	Some concerns	Insufficient information to judge if there were deviations from intended interventions.	Low risk of bias	No evidence of dropouts or missing data.	Some concerns	Blinding not discussed.	Some concerns	No reference to a protocol	Some concerns	Some concerns due to lack of information and lack of protocol.
Huang 2014	Some concerns	States randomly divided, but no description of randomisation process. The MBI scores were significantly higher than those before treatment (P<0.05), and the MBI scores of the four technical groups were significantly higher than those of the control group (P<0.05), but there was no significant difference in the FMA scores between the five groups after treatment (P<0.05). P> 0.05), there was no significant difference in MBI score among the four technical groups (P>0.05).	Some concerns	Insufficient information to judge if there were deviations from intended interventions.	Low risk of bias	No evidence of dropouts or missing data.	Some concerns	Blinding not discussed.	Some concerns	No reference to a protocol	Some concerns	Some concerns due to lack of information and lack of protocol.
Huang 2014	Some concerns	States randomly divided, but no description of randomisation process. The MBI scores were significantly higher than those before treatment (P<0.05), and the MBI scores of the four technical groups were significantly higher than those of the control group (P<0.05), but there was no significant difference in the FMA scores between the five groups after treatment (P<0.05). P> 0.05), there was no significant difference in MBI score among the four technical groups (P>0.05).	Some concerns	Insufficient information to judge if there were deviations from intended interventions.	Low risk of bias	No evidence of dropouts or missing data.	Some concerns	Blinding not discussed.	Some concerns	No reference to a protocol	Some concerns	Some concerns due to lack of information and lack of protocol.
Li 1999	High risk of bias	Lack of information about randomisation. Early rehabilitation group had a higher co‐morbidity score than the control group at baseline. No difference for age and past history rating between groups. No mention of other variables tested for baseline differences.	Some concerns	Lack of information.	Some concerns	Lack of information.	Low risk of bias	"assessor blinded to the treatment allocation assessed the subjects at four time intervals"	Some concerns	No protocol available.	High risk of bias	Lack of information for most domains. Concern about bias arising from randomisation process.
Li Yuanzheng 2014	Some concerns	States "randomly divided". No further information provided. "There was no significant difference in general data between the two groups (0.05)."	Some concerns	Insufficient information to judge if there were deviations because of the trial context.	Low risk of bias	122 participants randomised. Data available for all participants.	Some concerns	No information relating to blinding of outcome assessor.	Some concerns	No protocol available.	Some concerns	Some concerns due to lack of information and no study protocol.
Lu 2004	Some concerns	No discussion around randomisation process. No significant difference in general data between the groups.	Some concerns	Insufficient information to judge if there were deviations due to trial context.	Low risk of bias	No evidence of drop outs or missing data.	Some concerns	Not clear if blinded assessors.	Some concerns	No mention of protocol or ethics. No protocol obtained.	Some concerns	Some concerns due to lack of information and lack of protocol.
Meng Fanda 2021	Some concerns	"Random number table method used for dividing into 2 groups" (translated from Chinese). No information relating to allocation concealment. "The general data such as age and gender of all patients (P>0.05) have comparative significance" (translated from Chinese).	Some concerns	Lack of information to judge whether there could be deviations from intended interventions.	Low risk of bias	40 patients randomised and data available for 40.	Some concerns	No discussion of assessor blinding.	Some concerns	No protocol identified.	Some concerns	Some concerns due to lack of information.
Ni 1997	Some concerns	States "randomly divided" but method of randomisation not stated. No information relating allocation concealment.	Some concerns	Insufficient information to judge if there may have been deviations from intended interventions.	Low risk of bias	No dropouts or missing data.	Some concerns	No information relating to blinding.	Some concerns	No protocol identified.	Some concerns	Some concerns due to lack of information for all domains.
Pan 2004	Some concerns	Method of randomisation: "by participants drawing lots". No further information.	High risk of bias	Lack of information.	Low risk of bias	96 participants randomised and with data available.	Some concerns	No information about blinding.	Some concerns	No protocol identified	High risk of bias	Lack of information to support judgements.
Qin 2013	Some concerns	States randomly divided using a randomisation table. No information relating to allocation concealment. No significant difference between groups at baseline.	Some concerns	The treatments were carried out in hospital out‐patient settings, but these appear to have been at the same time for both groups so there may have been potential for contamination between groups. Insufficient information to judge. Participants were analysed according to the group to which they were randomised.	Low risk of bias	No evidence of any drop outs or missing data.	High risk of bias	No information relating to blinding of assessors. Insufficient evidence to judge if assessment of the outcome could have been influenced by knowledge of the intervention received.	Some concerns	No protocol identified.	High risk of bias	Some concerns due to lack of information for most domains, particularly around lack of blinding and measurement of outcome..
Wang 2004a	Some concerns	Randomised according to the assessment time, in a 2: 1 (rehabilitation: control) ratio. No information about allocation concealment. Baseline clinical data not provided.	Some concerns	Insufficient information to judge if there could be deviations or contamination between groups.	Low risk of bias	105 recruited; data available for 98. Dropouts due to financial reasons or inability to adhere to study design.	Low risk of bias	Blinded outcome assessment.	Some concerns	No protocol identified.	Some concerns	Some concerns relating to randomisation process and potential deviation from intended interventions, due to lack if information; and lack of protocol.
Wang Dongya 2015	Some concerns	States randomly divided, but no further details. Lack of baseline information.	Some concerns	Doesn't state if same nurses delivered intervention as those involved in usual care, which could provide opportunity for contamination.	Low risk of bias	126 randomised and data available for 126.	Some concerns	No information relating to blinding of outcome assessor.	Some concerns	No protocol identified.	Some concerns	Some concerns due to lack of information across all domains.
Wu 2006	Some concerns	No details provided relating to randomisation process. No significant baseline differences.	High risk of bias	Lack of information	Low risk of bias	Dropouts n=4 attributed to n=3 deaths and n=1 failure to attend assessment. Data available for remaining participants.	Some concerns	Lack of information.	Some concerns	No protocol available.	High risk of bias	Lack of information to support judgements for most domains.
Wu 2020	Low risk of bias	"The randomization list was generated using a computer randomizer (https://www.randomizer.org). The randomization was conducted after baseline assessment." "There was no statistical difference in baseline data between the two groups, so the results after the intervention are comparable."	Low risk of bias	The intervenion occured after hospital discharge, so contamination between the treatment groups was unlikely. Participants were analysed according to the group to which they were randomised.	Low risk of bias	32 patients in each group. 2 cases were dropped from the intervention group and 1 case from the control group in early stages. Very few drop outs and balance between groups, so judged unlikely to bias result.	Low risk of bias	Fugl Meyer. "Assessed by two rehabilitation therapists who were blinded to the grouping."	Low risk of bias	Study was prospectively registered on a trial register (ChiCTR1800018934), Fugl Meyer is listed as primary outcome.	Low risk of bias	Low risk of bias for all domains.
Wu Jing 2015	Some concerns	States randomly divided but no descripton of randomisation process. There were no significant differences in gender, age, type of disease, and location of disease between the two groups (all P>0.05), which were comparable.	Some concerns	Initial treatment is given in hospital ward; unclear if there is potential for contamination between groups.	Low risk of bias	92 patients randomised; data available for all 92.	Some concerns	No informaiton on blinding given.	Some concerns	No protocol identified.	Some concerns	Some concerns due to lack of information.
Xu 2003a	Some concerns	Randomisation method not stated ‘There was no obvious difference between two groups (P>0.05)’	High risk of bias	Lack of information.	Low risk of bias	"186 patients were randomly divided into rehabilitation group (n = 94) and control group (n = 92)". Data available for all participants.	Some concerns	Lack of information.	Some concerns	Protocol not available.	High risk of bias	Lack of information for most domains.
Xu 2003b	Some concerns	1.1. No information on randomisation process 1.3 Baseline demographics similar across both groups	High risk of bias	Lack of information (abstract only).	Low risk of bias	180 participants randomised and have data available.	Some concerns	Lack of information.	Some concerns	No protocol available.	High risk of bias	Lack of information to support judgements (abstract only).
Xu 2015	Some concerns	No details of how randomisation was performed. No differences at baseline.	Some concerns	Insufficient information to judge if there could be deviations or contamination between groups. Not clear if same therapists for control and intervention.	Low risk of bias	No missing data or dropouts.	Low risk of bias	"blinded fashion".	Some concerns	No protocol identified.	Some concerns	Some concerns due to lack of information about randomisation process and delivery of interventions; and lack of protocol.
Xu 2022	Some concerns	States "randomly divided" but no further information. "There was no statistically significant difference in the treatment of stroke patients between the two groups. X2=2.7778, P=2.4198."	High risk of bias	Nurses were delivering rehabilitation to one group and not to another, within an acute stroke setting. Families were involved in delivering the rehabilitation to the treatment group. There is therefore potential for deviations due to contamination between groups Reporting within this published study appears to have some anomolies. There is a section reporting "establishment of a stroke model", including statistical formulae, but it is not clear how this relates to the reported study.	High risk of bias	160 participants randomised; presents demographics for 160; but results data available for 164. No explanation provided for this difference.	High risk of bias	Does not state blinded outcome assessor. If outcomes were assessed by nurses involved in the study, there is potential for influence by knowledge of treatment group.	Some concerns	No protocol identified.	High risk of bias	This study is considered high risk of bias due to some anomolies in reporting, potential for deviations from the intended interventions and bias in the measurement of the outcome.
Xue 2006	Some concerns	No information provided ‘the baseline data were comparable between the two groups, and there were no significant differences (P>0.05)	High risk of bias	Lack of information.	Low risk of bias	"All the 150 patients with post‐stroke hemiplegia were involved in the analysis, no one missed".	High risk of bias	Outcome assessor not blinded. "Patients were evaluated by the professional group (the fourth and fifth authors) before treatment and 1 month after treatment respectively".	Some concerns	No protocol available.	High risk of bias	_Lack of information to support judgements, and concerns about potential deviaions from intended interventions and lack of blinding of outcome assessor._
Yan 2015	Some concerns	Divided "according to the random number table method". Lack of information relating to allocation concealment. No statistically significant differences at baseline.	Some concerns	Insufficient information to judge if there may have been deviations from the intended interventions. There may have been potential for contamination between groups during the hospital phase, but no information given on who provided the intervention to the different groups.	Low risk of bias	No drop outs or missing data reported.	High risk of bias	No information relating to who completed the outcome assessments and whether they were blinded.	Some concerns	No protocol identified.	High risk of bias	Concerns due to lack of information relating to allocation concealment and outcome assessment, with lack of details also for other domains.
Yang Aiguo 2015	Some concerns	States randomly divided but no discussion of randomisation process. No statistically significant difference between groups	Some concerns	Insufficient information to judge if there were deviations due to trial context.	Low risk of bias	No evidence of any dropouts or missing data.	Some concerns	No discussion of blinding.	Some concerns	No reference to protocol.	Some concerns	Some concerns due to lack of information and lack of protocol.
Ye Dayong 2010	Some concerns	States randomised. Lack of information about allocation concealment. No differences between groups at baseline.	Some concerns	Insufficient information to judge if there were deviations from intended interventions because of trial context. Participants were analysed according to assigned intervention.	Low risk of bias	No drop outs or missing data reported.	High risk of bias	Not clear if outcome assessor was blinded. Insufficient information to judge if outcome could have been influenced by knowledge of intervention received.	Some concerns	No protocol obtained.	High risk of bias	This is a Chinese language trial, with sections translated by one member of our team. There is generally a lack of information about the study methods. It is not clear whether the outcome assessor was blinded.
Yin 2003a	Some concerns	"among them, randomly selected 30 persons as the rehabilitation group, and another 30 randomly selected persons were grouped into rehabilitation with intermediate frequency". No information relating to allocation concealment. Limited information available regarding the baseline characteristics – Gender and age are similar in the rehabilitation groups but there is a potential gender bias in the control group. However, baseline Fugl‐Meyer scores are similar across all groups.	Some concerns	Insufficient information (single page report).	Low risk of bias	90 participants randomised. No drop‐outs mentioned, and categorical data available for 89 after treatment.	High risk of bias	No information relating to blinding.	Some concerns	No protocol.	High risk of bias	Some concerns due to lack of information (single page report only), and high risk of bias due to potential lack of blinded outcome assessor; no protocol identified.
Yin 2003a	Some concerns	"among them, randomly selected 30 persons as the rehabilitation group, and another 30 randomly selected persons were grouped into rehabilitation with intermediate frequency". No information relating to allocation concealment. Limited information available regarding the baseline characteristics – Gender and age are similar in the rehabilitation groups but there is a potential gender bias in the control group. However, baseline Fugl‐Meyer scores are similar across all groups.	Some concerns	Insufficient information (single page report).	Low risk of bias	90 participants randomised. No drop‐outs mentioned, and categorical data available for 89 after treatment.	High risk of bias	No information relating to blinding.	Some concerns	No protocol.	High risk of bias	Some concerns due to lack of information (single page report only), and high risk of bias due to potential lack of blinded outcome assessor; no protocol identified.
Zang 2013	Some concerns	States randomly divided but randomisation process not discussed. No significant difference in general between the 2 groups.	Some concerns	Insufficient information to judge whether there could be deviations from the intended interventions.	Low risk of bias	100 participants randomised; data available for 95.	Some concerns	No information on assessor blinding provided.	Some concerns	No protocol identified.	High risk of bias	High risk of bias due to potential lack of blinded outcome assessor. Some concerns due to lack of information for most domains.
Zhang 1998	Some concerns	Randomisation method not stated ‐ ‘participants were randomly allocated to 2 groups’ Limited information available regarding baseline demographics although the BI and FMA scores at baseline are similar.	High risk of bias	Intervention delivered in ward, with control (no treatment) group in same ward. Patient family were encouraged to be involved in delivery of physical rehabilitation to treatment group. There is a possibility of contamination between groups (e.g. with patients in no treatment group being delivered physical rehabilitation exercises by family members, having observed or spoken with other families).	Low risk of bias	56 participants randomised with data available for all; no drop outs or missing data reported.	Some concerns	Lack of information.	Some concerns	No protocol available.	High risk of bias	Lack of information for most domains, with concerns of potential risk of contamination between groups.
Zhang 2004	Some concerns	No information as to how randomised. No statistical information provided.	Low risk of bias	The control group were discharged home, limiting potential for contamination between treatment groups.	High risk of bias	19 deaths and 157 dropout out of 800+ participants. (SARS epidemic prevented follow‐up in "more than 50 percent of the 157 cases" and no information reported in the remaining cases)	Some concerns	No mention of blinding of assessors	Some concerns	No protocol available.	High risk of bias	Lack of information available for some domains and concern over missing outcome data.
Zhang Jianhong 2013	Some concerns	States randomised but no discussion of randomisation process. Two groups seemed comparable at baseline.	Some concerns	Insufficient information to judge if deviations from intended intervention.	Some concerns	No evidence of drop outs or missing data.	Some concerns	Blinding of assessors not discussed	Some concerns	No protocol	Some concerns	Some concerns due to lack of information and lack of protocol.
Zhao 2002	High risk of bias	States randomly divided, but no information about method of randomisation or allocation concealment. No statistical significant differences in baseline values.	Some concerns	Insufficient information to judge if there may have been deviations from intended interventions.	High risk of bias	States that 180 patients were randomised. Results data show 100 were allocated to treatment and 80 to control; no explanation for difference. Not clear if imbalance between groups is due to problem with randomisation process or due to unreported drop outs (or chance).	Low risk of bias	Blinded assessors.	Some concerns	No protocol identified.	High risk of bias	Judged as high risk of bias as imbalance in numbers between groups, with no explanation ‐ uncertain if this could be due to drop outs or problems with the randomisation process.
Zheng 2014	Some concerns	Randomisation process not descripbed. No differences at baseline.	Some concerns	Insufficient information to judge if there were deviations from intended interventions.	Low risk of bias	No evidence of dropouts or missing data.	Some concerns	Assessor blinding not discussed.	Some concerns	No protocol identified.	Some concerns	Some concerns due to lack of information and lack of protocol.
Zhu 2001	High risk of bias	States "randomly divided" but no information on randomisation process or allocation concealment. No statistical significant differences in baseline values. "125 patients were randomly divided into two groups, rehabilitation group (72 cases) and control group (53 cases)". Unclear why the groups are imbalanced.	Some concerns	Insufficient information to judge if there could be deviations from intended interventions.	Low risk of bias	"125 patients were randomly divided into two groups, rehabilitation group (72 cases) and control group (53 cases)". Unclear if the difference may have been due to drop outs.	Some concerns	No information relating to blinding of outcome assessor.	Some concerns	No protocol available.	High risk of bias	High risk of bias due to concerns in differences in numbers between treatment groups; uncertain if this indicates bias in the randomisation process or may be due to study dropouts. Some concerns for other domains due to lack of information.
Zhu 2006	High risk of bias	English abstract states "divided...randomly and equally", but Chinese language indicates that method of randomisation was "according to the time of hospital admission". Unclear if this method meets the criteria for random allocation. No statistical significant differences in baseline values.	Some concerns	Lack of information.	Low risk of bias	70 participants randomised and data available for 70.	Some concerns	Lack of information. Unclear if blinded outcome assessor.	Some concerns	No protocol available.	High risk of bias	Serious concerns about randomisation process and lack of information for other domains.
Zhu 2007	Some concerns	“Stratified block randomisation” (divided by type of stroke before allocation to intervention or control groups) No information about allocation concealment. No statistical significant differences in baseline values.	High risk of bias	For some patients who returned to community setting, therapists would conduct weekly home‐visits to guide the patient on rehabilitative treatment. (3) Once training had commenced, therapists concurrently taught the patients’ family members or caregiver on the correct assistive training methods and care methods, such that they could provide some training outwith therapy time, while also reducing the secondary damage due to inappropriate care. (4) Patients learned to monitor their own body for discomfort, and report on time to therapist and caregiver. Potential for contamination between groups for patients on the ward. No attempt to record therapy provided by family members to either group. Control group ‐ therapy delivered by family members: ""Control group was not given standard rehabilitative treatment, but were allowed to perform activities independently under doctor’s advice or with assistance from nurses". Both groups were potentially receiving therapy delivered by family members after hospital discharge; but within the trial context, this should only have occured in the treatment group.	Low risk of bias	78 participants randomised and data available for 78. No standard deviations were available, so standard deviations have been estimated from ranges.	Low risk of bias	Blinded assessors.	Some concerns	No protocol available.	High risk of bias	Judged to be high risk of contamination between groups; some concerns as lack of information about randomisation and no protocol available.
**Subgroup 1.2.2 Other motor function measure**												
Green 2002	Low risk of bias	"Randomisation was achieved by numbered, sealed, opaque envelopes prepared from random number tables and used four length random permuted blocks." "Patients were assigned either community physiotherapy treatment (treatment group) or no treatment (controls) by an assistant, who was otherwise unconnected with the study." No baseline differences.	Low risk of bias	Clear description of dropouts provided. 161/170 completed intervention.	Low risk of bias	No contact between physio and researcher who did the assessment. "Unmasking of treatment groups was assessed by the researcher guessing the trial group for every patient at the 3‐month assessment; we measured the agreement between guess and actual group allocation with the K statistic."	Low risk of bias	No contact between physio and researcher who did the assessment. "Unmasking of treatment groups was assessed by the researcher guessing the trial group for every patient at the 3‐month assessment; we measured the agreement between guess and actual group allocation with the K statistic."	Some concerns	Reference to a protocol in contributors section, but this was not available.	Some concerns	Low risk of bias for all domains, with exception of bias in selection of the reported result.
ReTrain 2018	Low risk of bias	"Participants were individually randomised (1:1) via a computer‐generated randomisation sequence minimised for time since stroke and level of functional disability." "The Trial Manager requested randomisation only after a cohort of participants had been consented" Balance of characteristics across trial arms.	Low risk of bias	The intervention was delivered in a community setting, so no potential for participants to observe treatments for other groups.	Low risk of bias	2 dropouts in each of contol (22) and intervention (23) groups	Low risk of bias	"Only outcome assessors independent of the research team were blinded to group allocation." "participants, who have been informed of their allocation, will be reminded to hide their allocation from the assessor. Any incidents of unblinding will be recorded, and the assessor will be asked to record their guess of participant allocation after undertaking the assessments. Following recom‐mended strategies to maintain and assess blinding, the outcomes assessor will not be based at the research centre."	Low risk of bias	Comprehensive protocol; no evidence of difference between protocol and study.	Low risk of bias	Low risk of bias for all domains ‐ no concerns.
Wade 1992	Some concerns	Method of randomisation: permuted blocks of 10, using random number tables. No information about allocation concealment. No information provided about potential baseline differences.	Low risk of bias	Treatment group received community physiotherapy, control group got no physical rehabilitation. Participants were in the community so there was no contact between groups.	Low risk of bias	89/94 completed intervention and had three‐month follow‐up. All participants included in the analysis unless they died or had not reached last follow‐up point	Some concerns	"Once accepted into the trial, each patient was assessed by an independent (non‐treating) physiotherapist...." "Of course, patients and relatives often mentioned whether or not the treating therapist had visited....."	Some concerns	No protocol identified.	Some concerns	Some concerns about allocation concealment, unblinding of outcome assessor, and lack of study protocol, and lack of details about allocation concealment.
Wang 2022	Low risk of bias	"This is a single assessor blind parallel group, and one‐site RCT of rehabilitation nursing in acute ischaemic stroke. When patients are admitted to the hospital, the wCST‐LL is determined first and then patients are divided into a ‘large’ group (wCST‐LL is greater than or equal to 2mL) or ‘small’ group (wCST‐LL is less than 2mL). Then, patients are randomly assigned to an experimental or control group according to a computer‐generated random number". "After stratifying into large and small groups by wCST‐LL, each group of patients will be randomly divided into an experimental group and a control group by a computer ‐ generated random number method. After eligible patients sign an informed consent, a trained and qualified assessor, blinded for the implementation of the intervention, will conduct the baseline assessments. Patients will be assigned a number according to the order of baseline assessment, and each ordinal number will correspond to a random number. Due to the characteristics of the face‐to‐face treatment, patients may know their grouping and those who deliver the intervention cannot achieve blind ‐ ness, thus, it is a single assessor‐ blinded study" "No statistically significant between‐group differences were observed at baseline"	Low risk of bias	"Due to the characteristics of the face‐to‐face treatment, patients may know their grouping and those who deliver the intervention cannot achieve blind ‐ ness" "There was no lack of information in the rehabilitation record form, and no training was reported to be inconsistent with the intervention program during the weekly random checks conducted by head nurses. Thus, fidelity of nurses in the delivery of intervention was well ensured." "Our descriptive statistics will include means and SDs for continuous variables and numbers and proportions for categorical variables. We will conduct a non‐inferiority test between the intervention and control groups for the primary and secondary outcomes without regard to the wCST‐LL."	Low risk of bias	104 randomised; 101 baseline assessment; 88 with data in data analysis. Reasons for drop outs / missing data are balanced between groups.	Low risk of bias	Motor Assessment Scale Blinded outcome assessor.	Low risk of bias	Trial registration NCT03702452. Protocol published, including analysis plan. Analysis followed pre‐specified plan.	Low risk of bias	No concerns. Single blind randomised study following pre‐specified protocol.

**Table:** Risk of bias for analysis 1.3 Balance (Berg Balance Scale)

**Study**	**Bias**											
	**Randomisation process**		**Deviations from intended interventions**		**Missing outcome data**		**Measurement of the outcome**		**Selection of the reported results**		**Overall**	
	**Authors' judgement**	**Support for judgement**	**Authors' judgement**	**Support for judgement**	**Authors' judgement**	**Support for judgement**	**Authors' judgement**	**Support for judgement**	**Authors' judgement**	**Support for judgement**	**Authors' judgement**	**Support for judgement**
Aravind 2022	Low risk of bias	"A Toronto‐based research assistant, unfamiliar with participants, prepared a list of randomization assignments for each site by flipping a coin (block size of 2)" and informed participants of group allocation by phone. Participants were stratified by site and gait speed There were differences between groups at baseline, but these were explored and accounted for in the analysis.	Low risk of bias	Participants were not blinded to their group allocation which could have resulted in a bias, especially on self‐report measures. Control group received no therapy; intervention was delivered after hospital discharge, limiting opportunties for contamination between groups. Participants were analysed according to the group to which they were randomised.	Low risk of bias	2 and 4 drop outs in intervention/followup group .	Low risk of bias	"Trained evaluators, blinded to study hypotheses and group assignment"	Low risk of bias	Published protocol and trial registration available. Results reported as per pre‐published protocol.	Low risk of bias	Judged low risk of bias for all domains. Adequate randomisation and allocation concealment, blinded outcome assessor. Intervention delivered at home limiting opportunties for contamination between groups. Study protocol available.
Brouwer 2018	Low risk of bias	After entering the FIM score, a web‐based randomization system provided the group assignment (tune‐up or control) using a 1:1 allocation in permuted blocks of 2 and 4. Eligible and consenting participants were stratified before randomization. Outcomes obtained at baseline confirm that the 2 groups were comparable on all measures at the outset.	Low risk of bias	"A modified Zelen approach18 was used to acquire second level consent, meaning that participants randomized to receive the tune up intervention were provided details about the trial by the site project coordinator whereas those randomized to the control group (ie, no tune‐up) were blind to being part of an RCT. ... thus eliminating any possibility of biases caused by conscious or unconscious participant opinions about the benefit of additional therapy, a limitation of many RCT designs"	Low risk of bias	103 randomised. 70 at immediate (12 month) time point. Dropouts balanced between groups.	Low risk of bias	"All outcome evaluations were administered by trained research assistants who were blind to group allocation."	Some concerns	The trial was registered in the international registry (ClinicalTrials.gov identifier no. NCT00400712) Berg Balance scale was not listed as a pre‐planned outcome in the protocol.	Some concerns	Some concerns as Berg balance assessment was not pre‐stated in the protocol. Judged low risk of bias for other domains.
Holmgren 2006	Low risk of bias	"Randomization of subjects into the intervention (IG) or control group (CG) was conducted with a minimization software program, MiniM (29) to avoid baseline risk factor imbalances between the two groups. Two variables were taken into account: cognition, using the Mini Mental State Examination, and fall risk, using the Fall Risk Index" "There were no significant differences in the baseline characteristics of the two groups except from the TOAST pathogenesis classification of ischemic stroke"	Low risk of bias	The investigation group (IG) and control group (CG) did not meet. The CG attended education sessions, but these were "about communication difficulties, fatigue,depressive symptoms, mood swings, personality changes and dysphagia, all more or less hidden dysfunctions after stroke and how to cope with these difficulties" and did not have a focus on physical rehabilitation. "For each participant in the IG and the CG and each session, there was a journal filled out by the care providers in each group. The IG contained information about all the chosen exercises, number of exercises, level of exercises and the participants feeling of exertion during the different exercises. The CG contained information on level of motivation and activity during the discussions"	Low risk of bias	All but one subject completed the entire program.	Low risk of bias	All assessments were done by blinded staff, who were instructed that if they had any reason to believe that they had revealed a subject’s group they should make an adverse event report.	Some concerns	The study protocol was approved by the Regional Ethical Review board for Human Research at Ume å University (Dnr 04‐022), but we were unable to access this for this review. Not possible to judge if this outcome was pre‐planned.	Some concerns	Low risk of bias for all domains, with exception of bias in selection of the reported result, where there were some concerns due to lack of information about pre‐planned outcomes.
Hoseinabadi 2013	High risk of bias	States "randomly divided into two equal groups" but no detail on how randomisation performed. Also reports the study design as "quasi‐experimental" but it is not clear if this relates to randomisation or not. No information relating to allocation concealment. "The two groups were not significantly different from each other in terms of balance, muscle tonicity and quality of life in pre‐test evaluation." Assessors judgement changed to high risk of bias due to risk that assignment to groups was done using a quasi‐random method.	Some concerns	No information about therapists assigned to each group. Insufficient information to judge if there could be deviations from the intended interventions.	Low risk of bias	No evidence of drop outs or missing data.	Some concerns	No information relating to blinding of outcome assessor, or information about who conducted the outcome assessments.	Some concerns	No protocol obtained.	High risk of bias	Judged as high risk of bias for randomisation as study is described as "quasi‐experimental" and, although it is reported that participants were "randomly divided", it is unclear if there was quasi‐randomisation. Some concerns due to lack of information and lack of protocol.
Knox 2018	Low risk of bias	"Randomized into the three intervention arms using computer‐generated random numbers and concealed allocation. A physiotherapist, blinded to group interventions, was responsible for the randomization scheme, preparation of the stratification envelopes, and group allocation." "At baseline, there were no significant differences in all the outcome measures among the groups"	Some concerns	Task intervention delivered by 1st author. Unclear if this could have introduced deviations.	Low risk of bias	144 randomised. 128 with outcome data at immediate tiimepoint. Drop outs accounted for, and not related to intervention.	Low risk of bias	"A physiotherapist, experienced in stroke rehabilitation, trained in the application of the outcome measures, and blinded to group allocation, performed the assessment."	Some concerns	Registered in clinical trials registry, but states "Retrospective registration ‐ This trial was registered after enrolment of the first participant" Trial was completed 2009‐2011, but not registered until 2018.	Some concerns	Some concerns due to lack of information about delivery of interventions, and retrospective registration of trial protocol.
Knox 2018	Low risk of bias	"Randomized into the three intervention arms using computer‐generated random numbers and concealed allocation. A physiotherapist, blinded to group interventions, was responsible for the randomization scheme, preparation of the stratification envelopes, and group allocation." "At baseline, there were no significant differences in all the outcome measures among the groups"	Some concerns	Task intervention delivered by 1st author. Unclear if this could have introduced deviations.	Low risk of bias	144 randomised. 128 with outcome data at immediate tiimepoint. Drop outs accounted for, and not related to intervention.	Low risk of bias	"A physiotherapist, experienced in stroke rehabilitation, trained in the application of the outcome measures, and blinded to group allocation, performed the assessment."	Some concerns	Registered in clinical trials registry, but states "Retrospective registration ‐ This trial was registered after enrolment of the first participant" Trial was completed 2009‐2011, but not registered until 2018.	Some concerns	Some concerns due to lack of information about delivery of interventions, and retrospective registration of trial protocol.
Meng Fanda 2021	Some concerns	"Random number table method used for dividing into 2 groups" (translated from Chinese). No information relating to allocation concealment. "The general data such as age and gender of all patients (P>0.05) have comparative significance" (translated from Chinese).	Some concerns	Lack of information to judge whether there could be deviations from intended interventions.	Low risk of bias	40 patients randomised and data available for 40.	Some concerns	No discussion of assessor blinding.	Some concerns	No protocol identified.	Some concerns	Some concerns due to lack of information.
SunRISe 2021	Low risk of bias	"Participants were randomized on a 2:1 ratio to either the intervention group (IG) or usual care (UC), using a computerized random number generator (www.randomization.com). The group allocation list was kept with the supervisor (R.B.) and every new inclusion was consequently added and allocated to the group as indicated in this list. Although initially a 1:1 randomization was planned, after discussing the resources for the testing following delays due to equipment issues, the number of patient tests was reduced by shifting to a 2:1 randomization" "No significant differences were present between IG and CG, apart from the presence of diabetes, which was higher in the control group (p = 0.045)"	Low risk of bias	"As all aspects of the trial (recruitment, assessment, treatment allocation and intervention) were executed by one single person (A.J.), no blinding for group allocation was performed." Although the treatment was delivered by the first author, no treatment was delivered to the control group, meaning that deviations due to the trial context were unlikely. Participants were analysed according to the group to which they were randomised.	Low risk of bias	30 randomised. 6/20 from intervention group lost to follow‐up. Reasons for drop outs provided. 2/6 transport issues, 3/6 health issues not related to trial, 1/6 related to "presence of physiotherapist" Very small number of drop outs relating to trial.	High risk of bias	Berg balance scale. Outcome assessor was not blinded. "The study measurements were not performed by a blinded researcher as the full study was executed by one investigator, assisted by master students. Although all possible efforts were made to conduct the measurements in a standardized manner, potential bias from knowing the group allocation cannot be ruled out"	Some concerns	The study was prospectively registered on ClinicalTrials.gov with identifier: NCT02717715, however secondary outcome measures are not stated within the trials register. Insufficient information in protocol means it is not possible to judge if there was selection of the reported result.	High risk of bias	Concerns as no blinded outcome assessor with potential for bias, and no information relating to this outcome within study protocol.
Wang 2015	Low risk of bias	"Each approved patient was asked to draw a folded piece of paper marked with a computer‐generated random number from a bag. Patients were assigned to groups per the number drawn. The trial was initiated immediately following group assignment."	Low risk of bias	"Patients in the control group maintained their everyday routines but also received weekly visits or telephone calls by the therapist to talk about their rehabilitation progress, daily activities, and general health conditions. However, patients in the control group were not given specific instructions or guidance related to rehabilitation skills." Intervention was delivered in patient's homes, limiting opportunities for contamination between groups. Participants were analysed according to the group to which they were randomised.	Low risk of bias	No drop outs or missing data.	Low risk of bias	Berg Balance scale. "All outcome measurements were evaluated by an independent physical therapist who was blinded from knowing the treatment assignment."	Some concerns	No protocol obtained.	Some concerns	Low risk of bias for most domains; no study protocol obtained.
Wu 2020	Low risk of bias	"The randomization list was generated using a computer randomizer (https://www.randomizer.org). The randomization was conducted after baseline assessment." "There was no statistical difference in baseline data between the two groups, so the results after the intervention are comparable."	Low risk of bias	The intervention occured after hospital discharge, so contamination between the treatment groups was unlikely. Participants were analysed according to the group to which they were randomised.	Low risk of bias	32 patients in each group. 2 cases were dropped from the intervention group and 1 case from the control group in early stages. Very few drop outs and balance between groups, so judged unlikely to bias result.	Low risk of bias	Berg balance scale. "Assessed by two rehabilitation therapists who were blinded to the grouping."	Some concerns	Study was prospectively registered on a trial register (ChiCTR1800018934), however this only lists primary outcomes and does not state Berg balance scale as an outcome. Unclear if there is selection relating to the reporting of balance as a secondary outcome.	Some concerns	Low risk of bias for all domains except selection of reported results. A study protocol was published, but this did not list Berg balance scale as an outcome measure.

**Table:** Risk of bias for analysis 1.4 Gait velocity

**Study**	**Bias**											
	**Randomisation process**		**Deviations from intended interventions**		**Missing outcome data**		**Measurement of the outcome**		**Selection of the reported results**		**Overall**	
	**Authors' judgement**	**Support for judgement**	**Authors' judgement**	**Support for judgement**	**Authors' judgement**	**Support for judgement**	**Authors' judgement**	**Support for judgement**	**Authors' judgement**	**Support for judgement**	**Authors' judgement**	**Support for judgement**
**Subgroup 1.4.1 Gait speed (distance/time)**												
Aravind 2022	Low risk of bias	"A Toronto‐based research assistant, unfamiliar with participants, prepared a list of randomization assignments for each site by flipping a coin (block size of 2)" and informed participants of group allocation by phone. Participants were stratified by site and gait speed There were differences between groups at baseline, but these were explored and accounted for in the analysis.	Low risk of bias	Participants were not blinded to their group allocation which could have resulted in a bias, especially on self‐report measures. Control group received no therapy; intervention was delivered after hospital discharge, limiting opportunties for contamination between groups. Participants were analysed according to the group to which they were randomised.	Low risk of bias	2 and 4 drop outs in intervention/followup group .	Low risk of bias	"Trained evaluators, blinded to study hypotheses and group assignment"	Low risk of bias	Published protocol and trial registration available. Results reported as per pre‐published protocol.	Low risk of bias	Judged low risk of bias for all domains. Adequate randomisation and allocation concealment, blinded outcome assessor. Intervention delivered at home limiting opportunties for contamination between groups. Study protocol available.
Batchelor 2012	Low risk of bias	"(1:1 allocation ratio, simple randomization) using a computer‐generated random allocation sequence concealed from all researchers in opaque envelopes." Staff independent of the study undertook sequence and concealment. Baseline measures were similar.	Some concerns	"the provision of information about the project to rehabilitation staff discharging participants may have led to an increase in the number of falls prevention strategies provided to participants in both groups". These deviations are likely to have been balanced between groups. Participants were analysed according to the group to which they were randomised.	Low risk of bias	Overall, there was an 85% retention rate. Drop outs were balanced between groups.	Low risk of bias	The physiotherapists conducting baseline and the follow‐up assessment were blind to group allocation.	Low risk of bias	The trial was registered with the Australian New Zealand Clinical Trials registry (ACTRN12607000398404), and the protocol has been published. No evidence of deviations between protocol and analyses.	Some concerns	Judged as low risk of bias for all domains except for deviations from intended interventons, where there were some concerns that both groups received additional treatment strategies.
Chen S 2021	Some concerns	"Every patient who met the eligibility criteria was allotted a serial number by the computer with those having an odd number designated as the IG and those having an even number as the CG." Lack of information about allocation concealment. "There were no significant differences observed between the FMA scores in the IG and CG at 0 (the baseline	Low risk of bias	Control group had no therapy input. Intervention was delivered at home, following discharge from routine services, so contamination between groups unlikely.	Low risk of bias	"Of 164 patients enrolled in the study, 24 declined to participate, 11 were withdrawn after randomization (5 patients from the IG and 6 patients from the CG), five were lost to follow‐up (3 patients in the IG and 2 patients in the CG), and three refused to continue (3 patients in the IG and 0 patient in the CG)." The three "refusals" could be due to the demands of the intervention, however this is considered low.	Some concerns	"The outcome assessment for both groups were performed by one trained and certified nurse practitioner " No evidence of blinding. No evidence to suggest that assessment was influenced by knowledge of intervention.	Some concerns	No protocol available.	Some concerns	Some concerns due to lack of information about allocation concealment, no blinding and lack of a protocol.
Green 2002	Low risk of bias	"Randomisation was achieved by numbered, sealed, opaque envelopes prepared from random number tables and used four length random permuted blocks." "Patients were assigned either community physiotherapy treatment (treatment group) or no treatment (controls) by an assistant, who was otherwise unconnected with the study." No baseline differences.	Low risk of bias	"Physiotherapy treatment was done by an established community physiotherapy service (13 staff) as part of their usual work". No contact between people in the treatment and control group, therefore contamination considered unlikely. Participants were analysed according to the group to which they were randomised.	Low risk of bias	161/170 completed intervention. Clear description of dropouts provided, and these were not considered to be related to delivered intervention.	Low risk of bias	Gait velocity. No contact between physio and researcher who did the assessment. "Unmasking of treatment groups was assessed by the researcher guessing the trial group for every patient at the 3‐month assessment; we measured the agreement between guess and actual group allocation with the K statistic."	Some concerns	Reference to a protocol in contributors section, but this was not available.	Some concerns	Low risk of bias for all domains, with exception of bias in selection of the reported result where the protocol was not available.
Hui‐Chan 2009	Low risk of bias	Subjects were allocated randomly, using a computer program, to one of four groups. No information about allocation concealment. No baseline differences reported.	Some concerns	Subjects in the control group participated in four assessment sessions only, so the frequency of therapist‐patient contacts was less than that for the three treatment groups. Due to resource limitation, treatment effectiveness was examined for only 4 weeks after treatment. These differences in therapist contacts are considered unlikely to impact on the outcome. Participants were analysed according to the group to which they were randomised.	Low risk of bias	Eight drop outs reported. Reasons for drop outs reported and balanced between groups. Not considered to be related to the intervention.	Low risk of bias	"An assessor blinded to the treatment allocation assessed the subjects at four time intervals"	Some concerns	No protocol identified.	Some concerns	Some concerns relating to possible deviations from intended interventions and lack of protocol.
Knox 2018	Low risk of bias	"Randomized into the three intervention arms using computer‐generated random numbers and concealed allocation. A physiotherapist, blinded to group interventions, was responsible for the randomization scheme, preparation of the stratification envelopes, and group allocation." "At baseline, there were no significant differences in all the outcome measures among the groups"	Some concerns	Task intervention delivered by 1st author. Unclear if this could have introduced deviations. Participants were analysed according to the group to which they were randomised.	Low risk of bias	144 randomised. 128 with outcome data at immediate tiimepoint. Drop outs accounted for, and not related to intervention.	Low risk of bias	"A physiotherapist, experienced in stroke rehabilitation, trained in the application of the outcome measures, and blinded to group allocation, performed the assessment."	Some concerns	Registered in clinical trials registry, but states "Retrospective registration ‐ This trial was registered after enrolment of the first participant" Trial was completed 2009‐2011, but not registered until 2018.	Some concerns	Some concerns due to lack of information about delivery of interventions, and retrospective registration of trial protocol.
Knox 2018	Low risk of bias	"Randomized into the three intervention arms using computer‐generated random numbers and concealed allocation. A physiotherapist, blinded to group interventions, was responsible for the randomization scheme, preparation of the stratification envelopes, and group allocation." "At baseline, there were no significant differences in all the outcome measures among the groups"	Some concerns	Task intervention delivered by 1st author. Unclear if this could have introduced deviations. Participants were analysed according to the group to which they were randomised.	Low risk of bias	144 randomised. 128 with outcome data at immediate tiimepoint. Drop outs accounted for, and not related to intervention.	Low risk of bias	"A physiotherapist, experienced in stroke rehabilitation, trained in the application of the outcome measures, and blinded to group allocation, performed the assessment."	Some concerns	Registered in clinical trials registry, but states "Retrospective registration ‐ This trial was registered after enrolment of the first participant" Trial was completed 2009‐2011, but not registered until 2018.	Some concerns	Some concerns due to lack of information about delivery of interventions, and retrospective registration of trial protocol.
Lee 2015	Low risk of bias	Random drawings were carried out before the experiment: each subject had to draw a sealed envelope and was randomly assigned to either the exercise intervention or control. "There were no baseline differences in clinical and anthropometric variables, arterial stiffness indexes, gait velocity parameters, and physical fitness component between the intervention and control groups".	Low risk of bias	The control group is reported to complete "unsystematic physical activities" or leisure activities at "the identifical time", suggesting that there was no potential for contamination between groups. Participants were analysed according to the group to which they were randomised.	Low risk of bias	30 randomised. 4 drop outs (1 intervention, 3 control). Low number of drop outs; not considered to be relating to intervention.	Low risk of bias	"examiners for all outcome variables were blinded to patient group assignment"	Some concerns	No protocol identified.	Some concerns	Some concerns due to lack of protocol; considered low risk of bias for all other domains.
Signal 2014	Low risk of bias	"Following baseline assessment each participant was assigned using pseudo‐randomisation (minimisation)...". Minimisation is considered low risk of bias. "All participants were assigned within the same session by a blinded researcher not involved in other aspects of the trial to ensure that no selection bias was introduced." Intervention groups balanced.	Low risk of bias	"All participants were blinded to the study hypothesis and were only informed whether theyhad been allocated to a rehabilitation or control group." Unblinded physio observed each group to ensure fidelity to the intervention was maintained. Participants were analysed according to the group to which they were randomised.	Low risk of bias	20 randomised. Data from 19 analysed.	Low risk of bias	All assessments conducted by blinded personnel.	Low risk of bias	Trial registered: ACTRN12610000460000 Data reported and analysed according to pre‐stated plan.	Low risk of bias	Considered low risk of bias for all domains.
Teixeira‐Salmela 1999	Some concerns	Crossover design ‐ "With subjects randomly assigned to one of the two groups (treatment and control) with equal probability and balanced into similar blocks." No information relating to allocation concealment. Baseline characteristics were similar.	Some concerns	Study results are presented according to assigned intervention (although are also presented with intervention period data from both groups combined).	Low risk of bias	Control group 7, treatment group 6. No drop outs or missing data reported.	Some concerns	Gait velocity at "comfortable speed". No information relating to blinding. Different levels of encouragement can be used for comfortable speed.	Some concerns	No protocol identified.	Some concerns	Some concerns for most domains due to lack of information.
Wang 2015	Low risk of bias	"Each approved patient was asked to draw a folded piece of paper marked with a computer‐generated random number from a bag. Patients were assigned to groups per the number drawn. The trial was initiated immediately following group assignment."	Low risk of bias	"Patients in the control group maintained their everyday routines but also received weekly visits or telephone calls by the therapist to talk about their rehabilitation progress, daily activities, and general health conditions. However, patients in the control group were not given specific instructions or guidance related to rehabilitation skills." Intervention was delivered in patient's homes, limiting opportunities for contamination between groups. Participants were analysed according to the group to which they were randomised.	Low risk of bias	No drop outs or missing data.	Low risk of bias	Gait velocity. "All outcome measurements were evaluated by an independent physical therapist who was blinded from knowing the treatment assignment."	Some concerns	No protocol obtained.	Some concerns	Low risk of bias for most domains; no study protocol obtained.
Yang 2006	Low risk of bias	"Subjects were randomized to the control group or experimental group by an independent person who picked one of the sealed envelopes 30 min before the start of the intervention". "At baseline, there were no differences between groups for bilateral hip flexors, hip extensors, knee flexors, knee extensors, ankle dorsiflexors and ankle plantarflexors."	Low risk of bias	Participants were living in the community so unlikely to be any contamination between groups. Participants were analysed according to the group to which they were randomised.	Low risk of bias	48 randomised. No drop outs or missing data.	Low risk of bias	single blind study	Some concerns	Protocol is mentioned but we were unable to obtain this.	Some concerns	Some concerns due to lack of protocol, but considered low risk of bias for other domains.
**Subgroup 1.4.2 Timed walk (time to walk set distance)**												
Bek 2016	Low risk of bias	"Randomisation was performed by an independent administrator (who was not involved in outcome assessments), using an online randomisation tool, and the administrator informed patients and staff of group allocation"	Some concerns	Participants, carers and people delivering the intervention likely to be aware of assigned intervention group. Insufficient information to judge if there were deviations because of the trial context.	Some concerns	Data available for control: 32/36, intervention 30/41. "Reasons for dropping out included inability to commit (n=2), illness (n=1), transport problems (n=1), and participants not wishing to continue because they did not feel that the CE method suited them (n=2)". Higher number of dropouts in intervention group; although no indication that there were differences between those who did and did not drop‐out.	Some concerns	Method is time to walk 10 metres. Unclear whether outcome assessors were blinded. No evidence to suggest that the assessment of the outcome might have been influenced by knowledge of the intervention received.	Low risk of bias	Protocol published in trials registry: ISRCTN84064492. No evidence of differences between protocol and final study.	Some concerns	Some concerns due to lack of information about intervention delivery, drop outs and blinded outcome assessment.
Wade 1992	Some concerns	Method of randomisation: permuted blocks of 10, using random number tables. No information about allocation concealment. No information provided about potential baseline differences.	Low risk of bias	Treatment group received community physiotherapy, control group got no physical rehabilitation. Participants were in the community so there was no contact between groups. Participants were analysed according to the group to which they were randomised.	Low risk of bias	89/94 completed intervention and had three‐month follow‐up. All participants included in the analysis unless they died or had not reached last follow‐up point	Some concerns	"Once accepted into the trial, each patient was assessed by an independent (non‐treating) physiotherapist...." "Of course, patients and relatives often mentioned whether or not the treating therapist had visited....." Although unblinding may have occured in some patients, the treating therapist was independent, so not considered likely that outcome was influenced.	Some concerns	No protocol available.	Some concerns	Some concerns about allocation concealment, unblinding of outcome assessor, and lack of study protocol.
**Subgroup 1.4.3 Timed up and go test**												
Brouwer 2018	Low risk of bias	After entering the FIM score, a web‐based randomization system provided the group assignment (tune‐up or control) using a 1:1 allocation in permuted blocks of 2 and 4. Eligible and consenting participants were stratified before randomization. Outcomes obtained at baseline confirm that the 2 groups were comparable on all measures at the outset.	Low risk of bias	"A modified Zelen approach18 was used to acquire second level consent, meaning that participants randomized to receive the tune up intervention were provided details about the trial by the site project coordinator whereas those randomized to the control group (ie, no tune‐up) were blind to being part of an RCT. ... thus eliminating any possibility of biases caused by conscious or unconscious participant opinions about the benefit of additional therapy, a limitation of many RCT designs" Participants were analysed according to the group to which they were randomised.	Low risk of bias	103 randomised. 70 at immediate (12 month) time point. Dropouts balanced between groups.	Low risk of bias	"All outcome evaluations were administered by trained research assistants who were blind to group allocation."	Some concerns	The trial was registered in the international registry (ClinicalTrials.gov identifier no. NCT00400712) Gait velocity was not listed as a pre‐planned outcome in the protocol.	Some concerns	Some concerns as gait velocity was not pre‐stated in the protocol. Judged low risk of bias for other domains.
Qin 2013	Some concerns	States randomly divided using a randomisation table. No information relating to allocation concealment. No significant difference between groups at baseline.	Some concerns	The treatments were carried out in hospital out‐patient settings, but these appear to have been at the same time for both groups so there may have been potential for contamination between groups. Insufficient information to judge. Participants were analysed according to the group to which they were randomised.	Low risk of bias	No evidence of any drop outs or missing data.	High risk of bias	No information relating to blinding of assessors. Insufficient evidence to judge if assessment of the outcome could have been influenced by knowledge of the intervention received.	Some concerns	No protocol identified.	High risk of bias	Some concerns due to lack of information for most domains, particularly around lack of blinding and measurement of outcome..
ReTrain 2018	Low risk of bias	"Participants were individually randomised (1:1) via a computer‐generated randomisation sequence minimised for time since stroke and level of functional disability." "The Trial Manager requested randomisation only after a cohort of participants had been consented" Balance of characteristics across trial arms.	Low risk of bias	The intervention was delivered in a community setting, so no potential for pariticpants to observe treatments for other groups. Participants were analysed according to the group to which they were randomised.	Low risk of bias	2 dropouts in each of contol (22) and intervention (23) groups Low number of drop outs, balanced between groups.	Low risk of bias	"Only outcome assessors independent of the research team were blinded to group allocation." "participants, who have been informed of their allocation, will be reminded to hide their allocation from the assessor. Any incidents of unblinding will be recorded, and the assessor will be asked to record their guess of participant allocation after undertaking the assessments. Following recom‐mended strategies to maintain and assess blinding, the outcomes assessor will not be based at the research centre."	Low risk of bias	Comprehensive protocol; no evidence of difference between protocol and study.	Low risk of bias	Low risk of bias for all domains ‐ no concerns.
Wu 2020	Low risk of bias	"The randomization list was generated using a computer randomizer (https://www.randomizer.org). The randomization was conducted after baseline assessment." "There was no statistical difference in baseline data between the two groups, so the results after the intervention are comparable."	Low risk of bias	The intervention occured after hospital discharge, so contamination between the treatment groups was unlikely. Participants were analysed according to the group to which they were randomised.	Low risk of bias	32 patients in each group. 2 cases were dropped from the intervention group and 1 case from the control group in early stages. Very few drop outs and balance between groups, so judged unlikely to bias result.	Some concerns	"Assessed by two rehabilitation therapists who were blinded to the grouping."	Some concerns	Study was prospectively registered on a trial register (ChiCTR1800018934), however this only lists primary outcomes and does not state gait velocity as an outcome. Unclear if there is selection relating to the reporting of gait as a secondary outcome.	Some concerns	Low risk of bias for all domains except selection of reported results. A study protocol was published, but this did not list gait velocity as an outcome measure.
**Subgroup 1.4.4 Other measure relating to gait speed**												
SunRISe 2021	Low risk of bias	"Participants were randomized on a 2:1 ratio to either the intervention group (IG) or usual care (UC), using a computerized random number generator (www.randomization.com). The group allocation list was kept with the supervisor (R.B.) and every new inclusion was consequently added and allocated to the group as indicated in this list. Although initially a 1:1 randomization was planned, after discussing the resources for the testing following delays due to equipment issues, the number of patient tests was reduced by shifting to a 2:1 randomization" "No significant differences were present between IG and CG, apart from the presence of diabetes, which was higher in the control group (p = 0.045)"	Low risk of bias	"As all aspects of the trial (recruitment, assessment, treatment allocation and intervention) were executed by one single person (A.J.), no blinding for group allocation was performed." Although the treatment was delivered by the first author, no treatment was delivered to the control group, meaning that deviations due to the trial context were unlikely. Participants were analysed according to the group to which they were randomised.	Low risk of bias	30 randomised. 6/20 from intervention group lost to follow‐up. Reasons for drop outs provided. 2/6 transport issues, 3/6 health issues not related to trial, 1/6 related to "presence of physiotherapist" Very small number of drop outs relating to trial.	High risk of bias	6 minute walk test (distance). Outcome assessor was not blinded. "The study measurements were not performed by a blinded researcher as the full study was executed by one investigator, assisted by master students. Although all possible efforts were made to conduct the measurements in a standardized manner, potential bias from knowing the group allocation cannot be ruled out"	Some concerns	The study was prospectively registered on ClinicalTrials.gov with identifier: NCT02717715, however secondary outcome measures are not stated within the trials register. Insufficient information in protocol means it is not possible to judge if there was selection of the reported result.	High risk of bias	Concerns as no blinded outcome assessor with potential for bias, and no information relating to this outcome within study protocol.

**Table:** Risk of bias for analysis 1.5 Length of stay

**Study**	**Bias**											
	**Randomisation process**		**Deviations from intended interventions**		**Missing outcome data**		**Measurement of the outcome**		**Selection of the reported results**		**Overall**	
	**Authors' judgement**	**Support for judgement**	**Authors' judgement**	**Support for judgement**	**Authors' judgement**	**Support for judgement**	**Authors' judgement**	**Support for judgement**	**Authors' judgement**	**Support for judgement**	**Authors' judgement**	**Support for judgement**
Torres‐Arreola 2009	Some concerns	"Each patient was randomly allocated to one strategy after they had given written informed consent and completed questionnaires and they were randomised by the coordinator of the study using consecutive opaque envelopes, which were chosen by the patients or their relatives." There were statistically significant differences between groups for a number of baseline demographics, including stroke symptoms and Brunnstrom score.	Some concerns	There is insufficient information to judge whether there could have been deviations from the intended intervention. The "no treatment" group received an education intervention and it could have been possible that the nurse delivering this intervention delivered some rehabilitation. Participants were analysed according to assigned intervention	Low risk of bias	110 randomised and data available for 110. There were a number of drop outs but length of stay data were available for all.	Low risk of bias	We (the review team) have used the reported length of stay data, but the intervention continued following hospital discharge. Therefore this assessment is only appropriate for judging the effect of the hospital based part of this intervention. "All outcome variables were gathered by a team of nurses who were different from the intervention team and were blinded to the randomised group allocation."	Some concerns	No protocol identified	Some concerns	Some concerns due to baseline differences, potential deviations from interventions and lack of protocol. The study results only apply to the period of hospital based rehabilitation.

**Table:** Risk of bias for analysis 1.6 Adverse events

**Study**	**Bias**											
	**Randomisation process**		**Deviations from intended interventions**		**Missing outcome data**		**Measurement of the outcome**		**Selection of the reported results**		**Overall**	
	**Authors' judgement**	**Support for judgement**	**Authors' judgement**	**Support for judgement**	**Authors' judgement**	**Support for judgement**	**Authors' judgement**	**Support for judgement**	**Authors' judgement**	**Support for judgement**	**Authors' judgement**	**Support for judgement**
ACTIV 2021	Low risk of bias	"Randomization occurred after the baseline assessment.Participants were allocated to an ACTIV group or a usualcare control group using a 1:1 ratio. Stratified block ran‐domization was used according to participants’ geographiccenter and baseline mobility. Random block sizes were usedthat ensured a probability smaller than 0.1% that balancewould be broken across strata by 4 or more participants.The randomization software was coded and tested by thetrial statistician, then handed to an independent party forrandom number generator seeding, execution of allocation,and day‐to‐day management of randomization. The recruit‐ers, assessors, and personnel involved in data managementand analysis were blinded to participants’ treatment alloca‐tion."	Low risk of bias	The intervention was provided by a trained therapost following a specified treatment program. An "intervention physical therapist" provided the treatment, while a different research assistant was in contact with control group participants, preventing possibility for contamination between groups.	Some concerns	Small number of participants lost to follow up (1 from treatment group, 3 from control group. But also 6 participants discontinued intervention, and one discontinued control.	Low risk of bias	"A research assistant blinded to group allo‐cation telephoned each participant on a monthly basis torecord adverse events. Physical activity outside the study(PAOS) was also recorded to ascertain potential relatednessof any adverse events. Adverse events were coded by 2 independent assessors, also blinded to group allocation,according to the Common Terminology Criteria for AdverseEvents version 4.0."	Low risk of bias	Trials registry and published protocol. No noted differences between protocol and review.	Some concerns	Some concerns due to drop‐outs from treatment group.
Aravind 2022	Low risk of bias	"A Toronto‐based research assistant, unfamiliar with participants, prepared a list of randomization assignments for each site by flipping a coin (block size of 2)" and informed participants of group allocation by phone. Participants were stratified by site and gait speed There were differences between groups at baseline, but these were explored and accounted for in the analysis.	Low risk of bias	Participants were not blinded to their group allocation which could have resulted in a bias, especially on self‐report measures. Control group received no therapy; intervention was delivered after hospital discharge, limiting opportunties for contamination between groups. Participants were analysed according to the group to which they were randomised.	Low risk of bias	2 and 4 drop outs in intervention/followup group. Adverse events data fully reported.	Low risk of bias	Assessment of adverse events pre‐planned: "Fitness instructors documented attendance and adverse events that occurred during exercise classes using a standardized form."	Low risk of bias	Published protocol and trial registration available. Results reported as per pre‐published protocol.	Low risk of bias	Judged low risk of bias for all domains. Adequate randomisation and allocation concealment. Assessment of adverse events clearly planned and reported. Study protocol available.
ReTrain 2018	Low risk of bias	"Participants were individually randomised (1:1) via a computer‐generated randomisation sequence minimised for time since stroke and level of functional disability." "The Trial Manager requested randomisation only after a cohort of participants had been consented" Balance of characteristics across trial arms.	Low risk of bias	The intervention was delivered in a community setting, so no potential for pariticpants to observe treatments for other groups. Participants were analysed according to the group to which they were randomised.	Some concerns	2 dropouts in each of contol (22) and intervention (23) groups. Low number of drop outs, balanced between groups. Some concerns as adverse events not recorded for control group during intervention period.	Low risk of bias	Assessment of adverse events pre‐planned.	Low risk of bias	Comprehensive protocol; no evidence of difference between protocol and study.	Some concerns	Some concerns as adverse events not recorded for control group during intervention period.
Signal 2014	Low risk of bias	"Following baseline assessment each participant was assigned using pseudo‐randomisation (minimisation)...". Minimisation is considered low risk of bias. "All participants were assigned within the same session by a blinded researcher not involved in other aspects of the trial to ensure that no selection bias was introduced." Intervention groups balanced.	Low risk of bias	"All participants were blinded to the study hypothesis and were only informed whether theyhad been allocated to a rehabilitation or control group." Unblinded physio observed each group to ensure fidelity to the intervention was maintained. Participants were analysed according to the group to which they were randomised.	Low risk of bias	20 randomised. Data from 19 analysed. Adverse events reported for all groups.	Low risk of bias	Recording of adverse events pre‐planned and clearly reported.	Low risk of bias	Trial registered: ACTRN12610000460000 Data reported and analysed according to pre‐stated plan.	Low risk of bias	Considered low risk of bias for all domains.
Wang 2022	Low risk of bias	"This is a single assessor blind parallel group, and one‐site RCT of rehabilitation nursing in acute ischaemic stroke. When patients are admitted to the hospital, the wCST‐LL is determined first and then patients are divided into a ‘large’ group (wCST‐LL is greater than or equal to 2mL) or ‘small’ group (wCST‐LL is less than 2mL). Then, patients are randomly assigned to an experimental or control group according to a computer‐generated random number". "After stratifying into large and small groups by wCST‐LL, each group of patients will be randomly divided into an experimental group and a control group by a computer ‐ generated random number method. After eligible patients sign an informed consent, a trained and qualified assessor, blinded for the implementation of the intervention, will conduct the baseline assessments. Patients will be assigned a number according to the order of baseline assessment, and each ordinal number will correspond to a random number. Due to the characteristics of the face‐to‐face treatment, patients may know their grouping and those who deliver the intervention cannot achieve blind ‐ ness, thus, it is a single assessor‐ blinded study" "No statistically significant between‐group differences were observed at baseline"	Low risk of bias	"Due to the characteristics of the face‐to‐face treatment, patients may know their grouping and those who deliver the intervention cannot achieve blind ‐ ness" "There was no lack of information in the rehabilitation record form, and no training was reported to be inconsistent with the intervention program during the weekly random checks conducted by head nurses. Thus, fidelity of nurses in the delivery of intervention was well ensured."	Low risk of bias	104 randomised; 101 baseline assessment; 88 with data in data analysis. Reasons for drop outs / missing data are balanced between groups.	High risk of bias	States that monitored adverse events included: stroke progression, cardiovascular complications, fall‐induced injury, venous thromboembolism, pressure ulcers, pneumonia, urinary tract infections, and complications caused by improper exercise. Drop outs were reported in Figure 1: Flowchart of Study Participants. Authors reported "None of the patients experienced severe adverse events during the study period". However, study flowchart reported: Intervention group: 1 thrombolysis in limb; 2 Stroke progression; Control group: 1 "cardiac vegetation". We have assumed that the drop‐outs reported after 7 days represent all participants with adverse events, but are uncertain about this.	Low risk of bias	Trial registration NCT03702452. Protocol published, including analysis plan. Analysis followed pre‐specified plan.	High risk of bias	Judged high risk of bias due to lack of clarity over reporting of adverse events.

**Table:** Risk of bias for analysis 2.1 Independence in ADL scales

**Study**	**Bias**											
	**Randomisation process**		**Deviations from intended interventions**		**Missing outcome data**		**Measurement of the outcome**		**Selection of the reported results**		**Overall**	
	**Authors' judgement**	**Support for judgement**	**Authors' judgement**	**Support for judgement**	**Authors' judgement**	**Support for judgement**	**Authors' judgement**	**Support for judgement**	**Authors' judgement**	**Support for judgement**	**Authors' judgement**	**Support for judgement**
**Subgroup 2.1.1 Barthel Index**												
Aravind 2022	Low risk of bias	"A Toronto‐based research assistant, unfamiliar with participants, prepared a list of randomization assignments for each site by flipping a coin (block size of 2)" and informed participants of group allocation by phone. Participants were stratified by site and gait speed There were differences between groups at baseline, but these were explored and accounted for in the analysis.	Low risk of bias	Participants were not blinded to their group allocation which could have resulted in a bias, especially on self‐report measures. Control group received no therapy; intervention was delivered after hospital discharge, limiting opportunties for contamination between groups. Participants were analysed according to the group to which they were randomised.	Low risk of bias	2 and 4 drop outs in intervention/followup group .	Low risk of bias	"Trained evaluators, blinded to study hypotheses and group assignment"	Low risk of bias	Published protocol and trial registration available. Results reported as per pre‐published protocol.	Low risk of bias	Judged low risk of bias for all domains. Adequate randomisation and allocation concealment, blinded outcome assessor. Intervention delivered at home limiting opportunties for contamination between groups. Study protocol available.
Bai 2013	Low risk of bias	"Each patient was allocated by random number to the physiotherapy, acupuncture, or combined treatment group. In the randomization process, a research assistant opened envelopes containing random numbers and informed an acupuncturist or therapist if the patient was in the respective intervention group. The research assistant also informed physicians of the recruitment but not the group assignment. Additionally, she reminded the patients not to tell the physicians or the therapists whether they were treated with acupuncture." "Demographic characteristics (Table 1) were similar among the three groups, and gender, age, injured area, affected side, time from stroke onset, and comorbid conditions did not significantly differ among groups"	Low risk of bias	Patients told not to tell people delivering interventions whether they were in intervention or control arm.	Low risk of bias	No dropouts or missing data.	Low risk of bias	Assessors blinded to treatment group.	Some concerns	No protocol identified.	Some concerns	Low risk of bias for all domains, but no study protocol identified leaving concerns about selection of reported results.
Fang 2003	Low risk of bias	"Randomization was achieved through computer‐generated random numbers in sealed envelopes."	Low risk of bias	The control group received no physiotherapy, but did receive usual multidisciplinary care. "Therapists were blinded to patients’ groupings".	High risk of bias	In the treatment group, 38 dropped out from 50 (measured at 3 months) by 6 month follow up. In the non‐treatment group, 64 dropped out from 78 (measured at 3 months) by 6 month follow up. Authors report that the high drop out rate in the treatment group was due to participants not being able to tolerate the treatment, and acknowledge that the 28 missing participants probably had lower IADLI (Barthel Index) scores. It is therefore considered that the missing data is likely to contribute to bias in the outcome results. No rationale given for high drop out rate in non‐treatment group.	Low risk of bias	Assessment performed by trained neurologists who were blinded to the grouping of the subjects.	Some concerns	No protocol available.	High risk of bias	High risk of bias due to missing data from participants who were unable to complete the intervention, and some concerns due to lack of protocol.
Fang YN 2004	High risk of bias	States "randomized" in English text, but no information provided in Chinese text. 50 in one group, 78 in other group. Reason for difference in group size not clear. Differences in gender balance between groups.	Some concerns	Lack of information.	High risk of bias	Group 1, n=50. Group 2, n=78. Outcome data at end of intervention: n=45 and n=55. Outcome data at follow‐up: Group 1: 14, Group 2: 12. No information available relating to missing data.	Low risk of bias	"It was a blind experiment for the experimentor in charge of the evaluation" (translated from Chinese)	Some concerns	No protocol available.	High risk of bias	Insufficient information about randomisation and missing outcome data, and lack of information for other domains.
Fang YN 2004	High risk of bias	States "randomized" in English text, but no information provided in Chinese text. 50 in one group, 78 in other group. Reason for difference in group size not clear. Differences in gender balance between groups.	Some concerns	Lack of information.	High risk of bias	Group 1, n=50. Group 2, n=78. Outcome data at end of intervention: n=45 and n=55. Outcome data at follow‐up: Group 1: 14, Group 2: 12. No information available relating to missing data.	Low risk of bias	"It was a blind experiment for the experimentor in charge of the evaluation" (translated from Chinese)	Some concerns	No protocol available.	High risk of bias	Insufficient information about randomisation and missing outcome data, and lack of information for other domains.
Green 2002	Low risk of bias	"Randomisation was achieved by numbered, sealed, opaque envelopes prepared from random number tables and used four length random permuted blocks." "Patients were assigned either community physiotherapy treatment (treatment group) or no treatment (controls) by an assistant, who was otherwise unconnected with the study." No baseline differences.	Low risk of bias	"Physiotherapy treatment was done by an established community physiotherapy service (13 staff) as part of their usual work". No contact between people in the treatment and control group.	Low risk of bias	Clear description of dropouts provided. 146(completed 9 month assessment)/161(those assessed at end of intervention)	Low risk of bias	No contact between physio and researcher who did the assessment. "Unmasking of treatment groups was assessed by the researcher guessing the trial group for every patient at the 3‐month assessment; we measured the agreement between guess and actual group allocation with the K statistic."	Some concerns	Reference to a protocol in contributors section, but this was not available.	Some concerns	Low risk of bias for all domains, with exception of bias in selection of the reported result.
Holmgren 2006	Low risk of bias	"Randomization of subjects into the intervention (IG) or control group (CG) was conducted with a minimization software program, MiniM (29) to avoid baseline risk factor imbalances between the two groups. Two variables were taken into account: cognition, using the Mini Mental State Examination, and fall risk, using the Fall Risk Index" "There were no significant differences in the baseline characteristics of the two groups except from the TOAST pathogenesis classification of ischemic stroke"	Low risk of bias	The investigation group (IG) and control group (CG) did not meet. The CG attended education sessions, but these were "about communication difficulties, fatigue,depressive symptoms, mood swings, personality changes and dysphagia, all more or less hidden dysfunctions after stroke and how to cope with these difficulties" and did not have a focus on physical rehabilitation. "For each participant in the IG and the CG and each session, there was a journal filled out by the care providers in each group. The IG contained information about all the chosen exercises, number of exercises, level of exercises and the participants feeling of exertion during the different exercises. The CG contained information on level of motivation and activity during the discussions"	Low risk of bias	Drop outs discussed ‐ two subjects dropped out during follow‐up	Low risk of bias	All assessments were done by blinded staff, who were instructed that if they had any reason to believe that they had revealed a subject’s group they should make an adverse event report.	Some concerns	The study protocol was approved by the Regional Ethical Review board for Human Research at Ume å University (Dnr 04‐022), but we were unable to access this for this review.	Some concerns	Low risk of bias for all domains, with exception of bias in selection of the reported result.
Lu 2004	Some concerns	No discussion around randomisation process. No significant difference in general data between the groups.	Some concerns	Insufficient information to judge if there were deviations due to trial context.	Low risk of bias	No evidence of drop outs or missing data.	Some concerns	Not clear if blinded assessors.	Some concerns	No mention of protocol or ethics. No protocol obtained.	Some concerns	Some concerns due to lack of information and lack of protocol.
Wade 1992	Some concerns	Method of randomisation: permuted blocks of 10, using random number tables. No information about allocation concealment. No information provided about potential baseline differences.	Low risk of bias	Treatment group received community physiotherapy, control group got no physical rehabilitation. Participants were in the community so there was no contact between groups. Participants were analysed according to the group to which they were randomised.	Low risk of bias	86 out of 89 (3month followup) completed intervention and had six‐month follow‐up. All participants included in the analysis unless they died or had not reached last follow‐up point	Some concerns	"Once accepted into the trial, each patient was assessed by an independent (non‐treating) physiotherapist...." "Of course, patients and relatives often mentioned whether or not the treating therapist had visited....." Although unblinding may have occured in some patients, the treating therapist was independent, so not considered likely that outcome was influenced.	Some concerns	No protocol available.	Some concerns	Some concerns about allocation concealment, unblinding of outcome assessor, and lack of study protocol.
Xie 2003	Some concerns	1.1 No information provided 1.3 ‘No significant difference in age or score of neural function assessment could be found between the two groups’	High risk of bias	2.1 No information provided 2.3 Possibly same therapist across 2 groups, providing potential for contamination. (Lack of information)	Low risk of bias	64 participants randomised with data available for all. No drop outs or missing data reported.	Some concerns	Lack of information.	Some concerns	No protocol available.	High risk of bias	Lack of information to support judgements.
Yang Aiguo 2015	Some concerns	States randomly divided but no discussion of randomisation process. No statistically significant difference between groups	Some concerns	Insufficient information to judge if there were deviations due to trial context.	Low risk of bias	No evidence of any dropouts or missing data.	Some concerns	No discussion of blinding.	Some concerns	No reference to protocol.	Some concerns	Some concerns due to lack of information and lack of protocol.
Zheng 2014	Some concerns	Randomisation process not descripbed. No differences at baseline.	Some concerns	Insufficient information to judge if there were deviations from intended interventions.	Low risk of bias	No evidence of dropouts or missing data.	Some concerns	Assessor blinding not discussed.	Some concerns	No protocol identified.	Some concerns	Some concerns due to lack of information and lack of protocol.
Zhu 2007	Some concerns	“Stratified block randomisation” (divided by type of stroke before allocation to intervention or control groups) No information about allocation concealment. No statistical significant differences in baseline values.	High risk of bias	For some patients who returned to community setting, therapists would conduct weekly home‐visits to guide the patient on rehabilitative treatment. (3) Once training had commenced, therapists concurrently taught the patients’ family members or caregiver on the correct assistive training methods and care methods, such that they could provide some training outwith therapy time, while also reducing the secondary damage due to inappropriate care. (4) Patients learned to monitor their own body for discomfort, and report on time to therapist and caregiver. Potential for contamination between groups for patients on the ward. No attempt to record therapy provided by family members to either group. Control group ‐ therapy delivered by family members: ""Control group was not given standard rehabilitative treatment, but were allowed to perform activities independently under doctor’s advice or with assistance from nurses". Both groups were potentially receiving therapy delivered by family members after hospital discharge; but within the trial context, this should only have occured in the treatment group.	Low risk of bias	78 participants randomised and data available for 78. No standard deviations were available, so standard deviations have been estimated from ranges.	Low risk of bias	Blinded assessors.	Some concerns	No protocol available.	High risk of bias	Judged to be high risk of contamination between groups; some concerns as lack of information about randomisation and no protocol available.
Zhu 2007	Some concerns	“Stratified block randomisation” (divided by type of stroke before allocation to intervention or control groups) No information about allocation concealment. No statistical significant differences in baseline values.	High risk of bias	For some patients who returned to community setting, therapists would conduct weekly home‐visits to guide the patient on rehabilitative treatment. (3) Once training had commenced, therapists concurrently taught the patients’ family members or caregiver on the correct assistive training methods and care methods, such that they could provide some training outwith therapy time, while also reducing the secondary damage due to inappropriate care. (4) Patients learned to monitor their own body for discomfort, and report on time to therapist and caregiver. Potential for contamination between groups for patients on the ward. No attempt to record therapy provided by family members to either group. Control group ‐ therapy delivered by family members: ""Control group was not given standard rehabilitative treatment, but were allowed to perform activities independently under doctor’s advice or with assistance from nurses". Both groups were potentially receiving therapy delivered by family members after hospital discharge; but within the trial context, this should only have occured in the treatment group.	Low risk of bias	78 participants randomised and data available for 78. No standard deviations were available, so standard deviations have been estimated from ranges.	Low risk of bias	Blinded assessors.	Some concerns	No protocol available.	High risk of bias	Judged to be high risk of contamination between groups; some concerns as lack of information about randomisation and no protocol available.

**Table:** Risk of bias for analysis 2.2 Motor function scales

**Study**	**Bias**											
	**Randomisation process**		**Deviations from intended interventions**		**Missing outcome data**		**Measurement of the outcome**		**Selection of the reported results**		**Overall**	
	**Authors' judgement**	**Support for judgement**	**Authors' judgement**	**Support for judgement**	**Authors' judgement**	**Support for judgement**	**Authors' judgement**	**Support for judgement**	**Authors' judgement**	**Support for judgement**	**Authors' judgement**	**Support for judgement**
**Subgroup 2.2.1 Fugl Meyer**												
Bai 2013	Low risk of bias	"Each patient was allocated by random number to the physiotherapy, acupuncture, or combined treatment group. In the randomization process, a research assistant opened envelopes containing random numbers and informed an acupuncturist or therapist if the patient was in the respective intervention group. The research assistant also informed physicians of the recruitment but not the group assignment. Additionally, she reminded the patients not to tell the physicians or the therapists whether they were treated with acupuncture." "Demographic characteristics (Table 1) were similar among the three groups, and gender, age, injured area, affected side, time from stroke onset, and comorbid conditions did not significantly differ among groups"	Low risk of bias	Patients told not to tell people delivering interventions whether they were in intervention or control arm.	Low risk of bias	No dropouts or missing data.	Low risk of bias	Assessors blinded to treatment group.	Some concerns	No protocol identified.	Some concerns	Low risk of bias for all domains, but no study protocol identified leaving concerns about selection of reported results.
Fang 2003	Low risk of bias	"Randomization was achieved through computer‐generated random numbers in sealed envelopes."	Low risk of bias	The control group received no physiotherapy, but did receive usual multidisciplinary care. "Therapists were blinded to patients’ groupings".	High risk of bias	In the treatment group, 38 dropped out from 50 (measured at 3 months) by 6 month follow up. In the non‐treatment group, 64 dropped out from 78 (measured at 3 months) by 6 month follow up. Authors report that the high drop out rate in the treatment group was due to participants not being able to tolerate the treatment, and acknowledge that the 28 missing participants probably had lower IADLI (Barthel Index) scores. It is therefore considered that the missing data is likely to contribute to bias in the outcome results. No rationale given for high drop out rate in non‐treatment group.	Low risk of bias	Assessment performed by trained neurologists who were blinded to the grouping of the subjects.	Some concerns	No protocol available.	High risk of bias	High risk of bias due to missing data from participants who were unable to complete the intervention, and some concerns due to lack of protocol.
Fang YN 2004	High risk of bias	States "randomized" in English text, but no information provided in Chinese text. 50 in one group, 78 in other group. Reason for difference in group size not clear. Differences in gender balance between groups.	Some concerns	Lack of information.	High risk of bias	Group 1, n=50. Group 2, n=78. Outcome data at end of intervention: n=45 and n=55. Outcome data at follow‐up: Group 1: 14, Group 2: 12. No information available relating to missing data.	Low risk of bias	"It was a blind experiment for the experimentor in charge of the evaluation" (translated from Chinese)	Some concerns	No protocol available.	High risk of bias	Insufficient information about randomisation and missing outcome data, and lack of information for other domains.
Fang YN 2004	High risk of bias	States "randomized" in English text, but no information provided in Chinese text. 50 in one group, 78 in other group. Reason for difference in group size not clear. Differences in gender balance between groups.	Some concerns	Lack of information.	High risk of bias	Group 1, n=50. Group 2, n=78. Outcome data at end of intervention: n=45 and n=55. Outcome data at follow‐up: Group 1: 14, Group 2: 12. No information available relating to missing data.	Low risk of bias	"It was a blind experiment for the experimentor in charge of the evaluation" (translated from Chinese)	Some concerns	No protocol available.	High risk of bias	Insufficient information about randomisation and missing outcome data, and lack of information for other domains.
Hu 2007	Some concerns	"Random grouping method: This study involves the rehabilitation medicine departments of 22 affiliated hospitals of medical colleges or provincial hospitals. Each department is randomly grouped according to the random number table. The selected patients are stratified according to cerebral infarction and cerebral hemorrhage, and then each division The groups were randomized to either the rehabilitation group or the control group." No information about allocation concealment.	Some concerns	No information about who delivered the interventions, or whether there could have been deviations or contamination between groups.	High risk of bias	Drop outs not accounted for. 6 months: group 1 471/ recruited 866. group 2 469 / recruited 677	Low risk of bias	Outcome assessors blinded.	Some concerns	No protocol identified.	High risk of bias	High risk of bias due to concerns about missing outcomes (drop outs not accounted for). Some concerns due to lack of information for other domains.
Hu 2007	Some concerns	"Random grouping method: This study involves the rehabilitation medicine departments of 22 affiliated hospitals of medical colleges or provincial hospitals. Each department is randomly grouped according to the random number table. The selected patients are stratified according to cerebral infarction and cerebral hemorrhage, and then each division The groups were randomized to either the rehabilitation group or the control group." No information about allocation concealment.	Some concerns	No information about who delivered the interventions, or whether there could have been deviations or contamination between groups.	High risk of bias	Drop outs not accounted for. 6 months: group 1 471/ recruited 866. group 2 469 / recruited 677	Low risk of bias	Outcome assessors blinded.	Some concerns	No protocol identified.	High risk of bias	High risk of bias due to concerns about missing outcomes (drop outs not accounted for). Some concerns due to lack of information for other domains.
Lu 2004	Some concerns	No discussion around randomisation process. No significant difference in general data between the groups.	Some concerns	Insufficient information to judge if there were deviations due to trial context.	Low risk of bias	No evidence of drop outs or missing data.	Some concerns	Not clear if blinded assessors.	Some concerns	No mention of protocol or ethics. No protocol obtained.	Some concerns	Some concerns due to lack of information and lack of protocol.
Yang Aiguo 2015	Some concerns	States randomly divided but no discussion of randomisation process. No statistically significant difference between groups	Some concerns	Insufficient information to judge if there were deviations due to trial context.	Low risk of bias	No evidence of any dropouts or missing data.	Some concerns	No discussion of blinding.	Some concerns	No reference to protocol.	Some concerns	Some concerns due to lack of information and lack of protocol.
Zheng 2014	Some concerns	Randomisation process not descripbed. No differences at baseline.	Some concerns	Insufficient information to judge if there were deviations from intended interventions.	Low risk of bias	No evidence of dropouts or missing data.	Some concerns	Assessor blinding not discussed.	Some concerns	No protocol identified.	Some concerns	Some concerns due to lack of information and lack of protocol.
Zhu 2007	Some concerns	“Stratified block randomisation” (divided by type of stroke before allocation to intervention or control groups) No information about allocation concealment. No statistical significant differences in baseline values.	High risk of bias	For some patients who returned to community setting, therapists would conduct weekly home‐visits to guide the patient on rehabilitative treatment. (3) Once training had commenced, therapists concurrently taught the patients’ family members or caregiver on the correct assistive training methods and care methods, such that they could provide some training outwith therapy time, while also reducing the secondary damage due to inappropriate care. (4) Patients learned to monitor their own body for discomfort, and report on time to therapist and caregiver. Potential for contamination between groups for patients on the ward. No attempt to record therapy provided by family members to either group. Control group ‐ therapy delivered by family members: ""Control group was not given standard rehabilitative treatment, but were allowed to perform activities independently under doctor’s advice or with assistance from nurses". Both groups were potentially receiving therapy delivered by family members after hospital discharge; but within the trial context, this should only have occured in the treatment group.	Low risk of bias	78 participants randomised and data available for 78. No standard deviations were available, so standard deviations have been estimated from ranges.	Low risk of bias	Blinded assessors.	Some concerns	No protocol available.	High risk of bias	Judged to be high risk of contamination between groups; some concerns as lack of information about randomisation and no protocol available.
Zhu 2007	Some concerns	“Stratified block randomisation” (divided by type of stroke before allocation to intervention or control groups) No information about allocation concealment. No statistical significant differences in baseline values.	High risk of bias	For some patients who returned to community setting, therapists would conduct weekly home‐visits to guide the patient on rehabilitative treatment. (3) Once training had commenced, therapists concurrently taught the patients’ family members or caregiver on the correct assistive training methods and care methods, such that they could provide some training outwith therapy time, while also reducing the secondary damage due to inappropriate care. (4) Patients learned to monitor their own body for discomfort, and report on time to therapist and caregiver. Potential for contamination between groups for patients on the ward. No attempt to record therapy provided by family members to either group. Control group ‐ therapy delivered by family members: ""Control group was not given standard rehabilitative treatment, but were allowed to perform activities independently under doctor’s advice or with assistance from nurses". Both groups were potentially receiving therapy delivered by family members after hospital discharge; but within the trial context, this should only have occured in the treatment group.	Low risk of bias	78 participants randomised and data available for 78. No standard deviations were available, so standard deviations have been estimated from ranges.	Low risk of bias	Blinded assessors.	Some concerns	No protocol available.	High risk of bias	Judged to be high risk of contamination between groups; some concerns as lack of information about randomisation and no protocol available.
**Subgroup 2.2.2 Other motor function measure**												
Green 2002	Low risk of bias	"Randomisation was achieved by numbered, sealed, opaque envelopes prepared from random number tables and used four length random permuted blocks." "Patients were assigned either community physiotherapy treatment (treatment group) or no treatment (controls) by an assistant, who was otherwise unconnected with the study." No baseline differences.	Low risk of bias	"Physiotherapy treatment was done by an established community physiotherapy service (13 staff) as part of their usual work". No contact between people in the treatment and control group.	Low risk of bias	Clear description of dropouts provided. 146(completed 9 month assessment)/161(those assessed at end of intervention)	Low risk of bias	No contact between physio and researcher who did the assessment. "Unmasking of treatment groups was assessed by the researcher guessing the trial group for every patient at the 3‐month assessment; we measured the agreement between guess and actual group allocation with the K statistic."	Some concerns	Reference to a protocol in contributors section, but this was not available.	Some concerns	Low risk of bias for all domains, with exception of bias in selection of the reported result.
ReTrain 2018	Low risk of bias	"Participants were individually randomised (1:1) via a computer‐generated randomisation sequence minimised for time since stroke and level of functional disability." "The Trial Manager requested randomisation only after a cohort of participants had been consented" Balance of characteristics across trial arms.	Low risk of bias	The intervention was delivered in a community setting, so no potential for pariticpants to observe treatments for other groups.	Low risk of bias	2 dropouts in each of contol (22) and intervention (23) groups	Low risk of bias	"Only outcome assessors independent of the research team were blinded to group allocation." "participants, who have been informed of their allocation, will be reminded to hide their allocation from the assessor. Any incidents of unblinding will be recorded, and the assessor will be asked to record their guess of participant allocation after undertaking the assessments. Following recom‐mended strategies to maintain and assess blinding, the outcomes assessor will not be based at the research centre."	Low risk of bias	Comprehensive protocol; no evidence of difference between protocol and study.	Low risk of bias	Low risk of bias for all domains ‐ no concerns.
Wade 1992	Some concerns	Method of randomisation: permuted blocks of 10, using random number tables. No information about allocation concealment. No information provided about potential baseline differences.	Low risk of bias	Treatment group received community physiotherapy, control group got no physical rehabilitation. Participants were in the community so there was no contact between groups. Participants were analysed according to the group to which they were randomised.	Low risk of bias	86 out of 89 (3month followup) completed intervention and had six‐month follow‐up. All participants included in the analysis unless they died or had not reached last follow‐up point	Some concerns	"Once accepted into the trial, each patient was assessed by an independent (non‐treating) physiotherapist...." "Of course, patients and relatives often mentioned whether or not the treating therapist had visited....." Although unblinding may have occured in some patients, the treating therapist was independent, so not considered likely that outcome was influenced.	Some concerns	No protocol available.	Some concerns	Some concerns about allocation concealment, unblinding of outcome assessor, and lack of study protocol.

**Table:** Risk of bias for analysis 3.1 Independence in ADL scales

**Study**	**Bias**											
	**Randomisation process**		**Deviations from intended interventions**		**Missing outcome data**		**Measurement of the outcome**		**Selection of the reported results**		**Overall**	
	**Authors' judgement**	**Support for judgement**	**Authors' judgement**	**Support for judgement**	**Authors' judgement**	**Support for judgement**	**Authors' judgement**	**Support for judgement**	**Authors' judgement**	**Support for judgement**	**Authors' judgement**	**Support for judgement**
**Subgroup 3.1.1 Other ADL measure**												
Nindorera 2022	Low risk of bias	"A public health researcher assistant not involved in the study used a computer‐generated random allocation sequence to randomize the participants into 1 of 2 groups: immediate CBCS (IG) and delayed CBCS group (DG) using blocks according to age, disability, sex and study variables. The allocation was carried out with opaque, sealed envelopes" "The groups did not differ in demographic char‐ acteristics, baseline primary outcome values or secondary outcome measures"	Low risk of bias	"Participants were informed of the study objectives" Control group received an attention control intervention. Participants were aware that this was a cross‐over trial and that they would receive the physical rehabilitation intervention after 4 weeks.	Low risk of bias	46 participants randomised, and data available for all 46.	Low risk of bias	"A blind assessor collected participant data..."	Low risk of bias	Study protocol available on Trials Register: PACTR202001714888482. Reported data are in line with pre‐specified analysis plan.	Low risk of bias	Low risk of bias for all domains ‐ no concerns.
Yang Jian 2007	Some concerns	States randomised. Lack of information about allocation concealment. No differences between groups at baseline.	Some concerns	Insufficient information to judge if there were deviations from intended interventions because of trial context. Participants were analysed according to assigned intervention.	Low risk of bias	No drop outs or missing data reported.	High risk of bias	Not clear if outcome assessor was blinded. Insufficient information to judge if outcome could have been influenced by knowledge of intervention received.	Some concerns	No protocol obtained.	High risk of bias	This is a Chinese language trial, with sections translated by one member of our team. There is generally a lack of information about the study methods. It is not clear whether the outcome assessor was blinded.

**Table:** Risk of bias for analysis 3.2 Motor function scales

**Study**	**Bias**											
	**Randomisation process**		**Deviations from intended interventions**		**Missing outcome data**		**Measurement of the outcome**		**Selection of the reported results**		**Overall**	
	**Authors' judgement**	**Support for judgement**	**Authors' judgement**	**Support for judgement**	**Authors' judgement**	**Support for judgement**	**Authors' judgement**	**Support for judgement**	**Authors' judgement**	**Support for judgement**	**Authors' judgement**	**Support for judgement**
**Subgroup 3.2.1 Fugl Meyer**												
Ma Xue 2010	Some concerns	States randomised. Lack of information about allocation concealment. No differences between groups at baseline.	Some concerns	Insufficient information to judge if there were deviations from intended interventions because of trial context. Participants were analysed according to assigned intervention.	Low risk of bias	No drop outs or missing data reported.	High risk of bias	Not clear if outcome assessor was blinded. Insufficient information to judge if outcome could have been influenced by knowledge of intervention received.	Some concerns	No protocol obtained.	High risk of bias	This is a Chinese language trial, with sections translated by one member of our team. There is generally a lack of information about the study methods. It is not clear whether the outcome assessor was blinded.
Yang Jian 2007	Some concerns	States randomised. Lack of information about allocation concealment. No differences between groups at baseline.	Some concerns	Insufficient information to judge if there were deviations from intended interventions because of trial context. Participants were analysed according to assigned intervention.	Low risk of bias	No drop outs or missing data reported.	High risk of bias	Not clear if outcome assessor was blinded. Insufficient information to judge if outcome could have been influenced by knowledge of intervention received.	Some concerns	No protocol obtained.	High risk of bias	This is a Chinese language trial, with sections translated by one member of our team. There is generally a lack of information about the study methods. It is not clear whether the outcome assessor was blinded.
**Subgroup 3.2.2 Other motor function measure**												
FeSTivaLS 2014	Low risk of bias	"An independent randomization service concealed group allocation until contacted by a researcher," "Group allocation to either FST‐UL or FST‐LL will be deter‐mined by telephone call to an independent automated system within the Norwich Clinical Trials Unit. Minimiza‐ tion of baseline imbalance between treatment groups will be based on the Pocock and Simon’s range method and used to determine the allocation for each participant by Functional Ambulation category (FAC) (22) and Action Research Arm Test (ARAT) (23). " "All characteristics were balanced across the two groups except for stroke classification. The FST‐UL group had a higher percentage of people clinically classified as having a partial anterior circulation stroke and the FST‐LL had a higher percentage of people as having a lacunar stoke or posterior circulatory stroke"	Low risk of bias	Assignment was to either upper limb or lower limb training. "All participants received the intervention as allocated except one participant who withdrew from the FST‐LL group as he wanted FST‐UL. The content of FST‐UL and FST‐LL was consistent with the protocol (Table 3) and the amount of therapy was essentially the same in the two groups"	Low risk of bias	52 participants randomised; data available for 44. Reasons for dropouts / missing data reported, and balanced between groups.	Low risk of bias	"The assessor, who conducted the efficacy and health economics measurement battery at baseline, outcome, and follow‐up time‐points, remained blinded to participants’ group allocation throughout the trial."	Low risk of bias	Published protocol and trial registration. No evidence of differences between protocol and conducted study.	Low risk of bias	No concerns ‐ judged as low risk of bias for all domains.
McClellan 2004	Low risk of bias	"To ensure allocation was concealed, randomisation was by numbered, sealed, opaque envelopes." No baseline differences.	Some concerns	"To increase the likelihood that subjects were blind to group allocation, neither the exact purpose of the research nor the types of exercises that subjects would be receiving were specified and both mobility and upper limb function was measured." However, it is not clear if same therapists delivered intervention to both groups, which could result in deviations from intended interventions.	Low risk of bias	26 randomised; data available for 12/15 and 9/11 ‐ "81% of the sample was available for analysis at Week 6". Drop out reasons discussed, and were balanced between groups.	Low risk of bias	"Outcome measures were collected at Weeks 0, 6, and 14 by a measurer blinded to group allocation."	Some concerns	No protocol identified.	Some concerns	Some concerns due to lack of information relating to potential deviations from intended interventions, and lack of protocol.
Mudge 2009	Low risk of bias	"Use of computer‐generated random numbers by an individual not associated with the study. Randomization was revealed to each participant by the principal investigator after the second baseline assessment." "There were differences between control and exercise group clinical tests at baseline. The exercise group had greater distance on the 6MWT ( P .028), mean steps a day (P .021), peak activity index (P .008), and highest step rate in 1 minute (P.019) (see table 2). Imbalances seen were likely to be a result of chance because they were collected while randomization was concealed from the assessor and the participant. These differences were used as covariates in subsequent analysis."	Low risk of bias	"exercise group led by 1 of the investigators (S.M.) assisted by 2 physiotherapy students. control group was run by an occupational therapist and consisted of 4 social and 4 educational sessions". Participants lived in the community and interventions were carried out in a clinic. Limited opportunity for contamination between groups.	Low risk of bias	60 participants randomised; 2 dropped out before any treatment; 30/31 and 25/27 at post intervention assessment.	Low risk of bias	"Outcome assessment was performed by an independent physiotherapist blind to treatment assignment. Participants were not blind because they were aware of their own group allocation, which was revealed after the second testing session. Participants were instructed not to discuss group allocation with the assessor. The testing sessions were carried out in the same rehabilitation clinic as the intervention groups but were scheduled at different times to maintain blinding of the assessor. Unmasking of the independent assessor occurred in the case of 3 participants who inadvertently stated or implied their group allocation" (small number only).	Some concerns	No protocol identified or referenced.	Some concerns	Low risk of bias for all domains except for selection of reported results were there are some concerns due to the lack of a pre‐specified protocol.

**Table:** Risk of bias for analysis 3.3 Balance (Berg Balance Scale)

**Study**	**Bias**											
	**Randomisation process**		**Deviations from intended interventions**		**Missing outcome data**		**Measurement of the outcome**		**Selection of the reported results**		**Overall**	
	**Authors' judgement**	**Support for judgement**	**Authors' judgement**	**Support for judgement**	**Authors' judgement**	**Support for judgement**	**Authors' judgement**	**Support for judgement**	**Authors' judgement**	**Support for judgement**	**Authors' judgement**	**Support for judgement**
Moore 2016	Low risk of bias	"An allocation sequence to randomize to either the exercise or the control group was created using a computer “true” randomnumber generator (www.random.org) and delivered after screening by an administrator not associated with the trial." "matching baseline characteristics"	Low risk of bias	Control group just did exercises at home, meaning that there was no potential for contamination between groups. Participants were analysed according to the group to which they were randomised.	Low risk of bias	No dropouts or missing data.	Low risk of bias	"Outcome assessment was conducted within 2 weeks preintervention and 1 week postintervention by trained assessors blinded to the study hypotheses and group assignment"	Low risk of bias	Pre‐defined protocol. Clinical Trial Registration No.: ISRCTN41026907. Study results and analyses reflect pre‐planned protocol.	Low risk of bias	Judged to be low risk of bias for all domains.
Nindorera 2022	Low risk of bias	"A public health researcher assistant not involved in the study used a computer‐generated random allocation sequence to randomize the participants into 1 of 2 groups: immediate CBCS (IG) and delayed CBCS group (DG) using blocks according to age, disability, sex and study variables. The allocation was carried out with opaque, sealed envelopes" "The groups did not differ in demographic char‐ acteristics, baseline primary outcome values or secondary outcome measures"	Low risk of bias	"Participants were informed of the study objectives" Control group received an attention control intervention. Participants were aware that this was a cross‐over trial and that they would receive the physical rehabilitation intervention after 4 weeks. Participants were analysed according to the group to which they were randomised.	Low risk of bias	46 participants randomised, and data available for all 46.	Low risk of bias	"A blind assessor collected participant data..."	Low risk of bias	Study protocol available on Trials Register: PACTR202001714888482. Reported data are in line with pre‐specified analysis plan.	Low risk of bias	Judged as low risk of bias for all domains.
Pang 2005	Some concerns	No information relating to allocation concealment. There was no significant difference in any of the variables between the intervention and control groups at baseline.	Low risk of bias	Participants were instructed not to tell the assessors about the group assignment or the treatment they received and not to discuss the protocol with the stroke community. Participants were analysed according to the group to which they were randomised.	Low risk of bias	63 enrolled. 3 dropouts. Small number of drop outs, all accounted for and not related to trial intervention.	Low risk of bias	The research personnel who performed the outcome assessments were blinded to the group assignment	Some concerns	No protocol identified.	Some concerns	Some concerns relating to allocation concealment and lack of protocol; judged as low risk of bias for other domains.
Salbach 2004	Low risk of bias	"The sequence of random assignments was computer generated in randomly ordered block sizes of two and four for each stratum, and maintained in sealed opaque envelopes. Envelopes were prepared prior to recruitment by persons not involved in the study." "Envelopes were provided to the evaluator when a new subject was scheduled for assessment. Once consent and baseline assessment were complete, evaluators stratified subjects and unveiled their treatment assignment." No baseline differences.	Some concerns	Two participants discontinued the treatment because of a desire to have "both" interventions. This suggests that participants were aware that they were only receiving either upper limb or lower limb exercises and may have prompted contamination between groups. Participants were analysed according to the group to which they were randomised.	Low risk of bias	91 randomised. 9 patients with missing data. 7 patients discontinued treatment. Missing data accounted for and balanced between groups. Small numbers of drop outs.	Low risk of bias	"Only raters who were unaware of the group assignment performed postintervention evaluations."	Some concerns	No protocol identified.	Some concerns	Some concerns regarding potential for contamination between groups, and lack of a protocol. Judged as low risk of bias for other domains.

**Table:** Risk of bias for analysis 3.4 Gait velocity

**Study**	**Bias**											
	**Randomisation process**		**Deviations from intended interventions**		**Missing outcome data**		**Measurement of the outcome**		**Selection of the reported results**		**Overall**	
	**Authors' judgement**	**Support for judgement**	**Authors' judgement**	**Support for judgement**	**Authors' judgement**	**Support for judgement**	**Authors' judgement**	**Support for judgement**	**Authors' judgement**	**Support for judgement**	**Authors' judgement**	**Support for judgement**
**Subgroup 3.4.1 Gait speed (distance/time)**												
Dean 2000	Some concerns	"The randomization process involved drawing two cards, one with subjects’ names, and the other with the group allocation from two separate boxes. The cards were drawn by a person independent of the study" Evidence of some baseline differences: "the subjects in both groups had a range of abilities at pretraining". This included large difference between groups at Baseline for gait velocity: Exp: 70.7cm/s Control: 86.1 cm/s	Low risk of bias	Possibly participants were not aware of assigned intervention, as both groups received an hour of similar training (experimental group receiving lower limb and control upper limb exercises). Design of this study makes deviations unlikely. Between group effects were calculated.	Low risk of bias	2 subjects withdrew, but assumed rest of data relates to the remaining subjects. 9 (5 intervention group, 4 control group) completed pre and post training, 8 completed follow up.	Low risk of bias	Laboratory measures of gait velocity. Objective measures of gait speed, using standardised instructions. "The clinical assessments....were conducted by an independent rater who was blinded to subject allocation."	Some concerns	No protocol identified.	Some concerns	Some concerns relating to baseline differences (particularly for gait velocity outcome) and lack of protocol.
Dean 2006	Low risk of bias	"Randomisation will be stratified by stroke club site using a computer gen‐erated random number schedule with variable block sizes 2–6. Generation of the randomisation sequence will be performed centrally by a researcher not involved in recruitment or assessment. Group allocation will be con‐cealed using consecutively‐numbered opaque envelopes. The opaque envelope will be opened after completion of the assessment in the presence of the participant." "At baseline, the groups were similar in terms of demographic characteristics and other comorbidities"	Low risk of bias	"The participants and therapists delivering the intervention could not be blinded to intervention group allocation" Both treatment groups received similar programs, but one focussed on lower limb and one on upper limb. "The experimental and control classes were held in different areas of the stroke club and at different times to minimize the risk of “contamination” between the groups." Intention to treat analysis.	Low risk of bias	151 randomised. 65/76 in experimental and 68/75 in control group at end of intervention. Drop outs were accounted for and balanced between groups. 2 participants (both from control group) refused to continue classes and reassessment.	Low risk of bias	"Walking speed (m/s) was measured using the 10‐m Walk Test at the participant’s comfortable and fastest speed over the middle 10 m of a level, 14‐m walking track" "outcome measures were collected by an assessor who was blinded to group allocation. Blinding was ensured using several strategies. Participants were asked not to reveal details of their program to the assessors, and assessments were collected outside the times for exercise classes."	Low risk of bias	Trial registered and study protocol published. ACTRN12606000479505. Results data as pre‐specified in protocol.	Low risk of bias	Judged as low risk of bias for all domains.
Martins 2020	Low risk of bias	Eligible participants were randomly allocated to either the experimental or control groups, based on the content of the sealed envelopes. No baseline differences reported.	Some concerns	"The experimental and control interventions were delivered by the same physiotherapist, who had more than seven years of clinical and research experience in neurological rehabilitation." No further information which enables judgement of whether there could have been deviations from the intended intervention. Participants were analysed according to the group to which they were randomised.	Low risk of bias	36 randomised. 15/18 and 13/18 with measured outcomes at 12 weeks. (NB higher than expected drop out rate at 24 weeks) Drop outs / participants with missing data reasonably balance between groups, and reasons not related to treatment.	Low risk of bias	10 meter walk test. "A trained researcher, blinded to the participant allocation, collected sociodemographic, anthropometric, clinical data and the outcomes"	Low risk of bias	Registered at ClinicalTrials.gov (NCT02937480) before the recruitment started. The protocol was previously published (Martins et al., 2017). Results as pre‐stated in protocol.	Some concerns	Some concerns due to insufficient information to judge if there could have been deviations from intended interventions. Judged low risk of bias for other domains.
Moore 2016	Low risk of bias	"An allocation sequence to randomize to either the exercise or the control group was created using a computer “true” randomnumber generator (www.random.org) and delivered after screening by an administrator not associated with the trial." "matching baseline characteristics"	Low risk of bias	Control group just did exercises at home, meaning that there was no potential for contamination between groups. Participants were analysed according to the group to which they were randomised.	Low risk of bias	No dropouts or missing data.	Low risk of bias	"Outcome assessment was conducted within 2 weeks preintervention and 1 week postintervention by trained assessors blinded to the study hypotheses and group assignment"	Low risk of bias	Pre‐defined protocol. Clinical Trial Registration No.: ISRCTN41026907. Study results and analyses reflect pre‐planned protocol.	Low risk of bias	Judged to be low risk of bias for all domains.
Mudge 2009	Low risk of bias	"Use of computer‐generated random numbers by an individual not associated with the study. Randomization was revealed to each participant by the principal investigator after the second baseline assessment." "There were differences between control and exercise group clinical tests at baseline. The exercise group had greater distance on the 6MWT ( P .028), mean steps a day (P .021), peak activity index (P .008), and highest step rate in 1 minute (P.019) (see table 2). Imbalances seen were likely to be a result of chance because they were collected while randomization was concealed from the assessor and the participant. These differences were used as covariates in subsequent analysis."	Low risk of bias	"Exercise group led by 1 of the investigators (S.M.) assisted by 2 physiotherapy students. control group was run by an occupational therapist and consisted of 4 social and 4 educational sessions". Participants lived in the community and interventions were carried out in a clinic. Limited opportunity for contamination between groups. Participants were analysed according to the group to which they were randomised.	Low risk of bias	60 participants randomised; 2 dropped out before any treatment; 30/31 and 25/27 at post intervention assessment.	Low risk of bias	"Outcome assessment was performed by an independent physiotherapist blind to treatment assignment. Participants were not blind because they were aware of their own group allocation, which was revealed after the second testing session. Participants were instructed not to discuss group allocation with the assessor. The testing sessions were carried out in the same rehabilitation clinic as the intervention groups but were scheduled at different times to maintain blinding of the assessor. Unmasking of the independent assessor occurred in the case of 3 participants who inadvertently stated or implied their group allocation". This is judged to be a small number only, meaning that the assessor was blinded for the majority of assessments.	Some concerns	No protocol identified.	Some concerns	Low risk of bias for all domains except for selection of reported results were there are some concerns due to the lack of a pre‐specified protocol.
Nindorera 2022	Low risk of bias	"A public health researcher assistant not involved in the study used a computer‐generated random allocation sequence to randomize the participants into 1 of 2 groups: immediate CBCS (IG) and delayed CBCS group (DG) using blocks according to age, disability, sex and study variables. The allocation was carried out with opaque, sealed envelopes" "The groups did not differ in demographic char‐ acteristics, baseline primary outcome values or secondary outcome measures"	Low risk of bias	"Participants were informed of the study objectives" Control group received an attention control intervention. Participants were aware that this was a cross‐over trial and that they would receive the physical rehabilitation intervention after 4 weeks. Participants were analysed according to the group to which they were randomised.	Low risk of bias	46 participants randomised, and data available for all 46.	Low risk of bias	"A blind assessor collected participant data..."	Low risk of bias	Study protocol available on Trials Register: PACTR202001714888482. Reported data are in line with pre‐specified analysis plan.	Low risk of bias	Judged as low risk of bias for all domains.
Salbach 2004	Low risk of bias	"The sequence of random assignments was computer generated in randomly ordered block sizes of two and four for each stratum, and maintained in sealed opaque envelopes. Envelopes were prepared prior to recruitment by persons not involved in the study." "Envelopes were provided to the evaluator when a new subject was scheduled for assessment. Once consent and baseline assessment were complete, evaluators stratified subjects and unveiled their treatment assignment." No baseline differences.	Some concerns	Two participants discontinued the treatment because of a desire to have "both" interventions. This suggests that participants were aware that they were only receiving either upper limb or lower limb exercises and may have prompted contamination between groups. Participants were analysed according to the group to which they were randomised.	Low risk of bias	91 randomised. 9 patients with missing data. 7 patients discontinued treatment. Missing data accounted for and balanced between groups. Small numbers of drop outs.	Low risk of bias	"Only raters who were unaware of the group assignment performed postintervention evaluations."	Some concerns	No protocol identified.	Some concerns	Some concerns regarding potential for contamination between groups, and lack of a protocol. Judged as low risk of bias for other domains.
**Subgroup 3.4.2 Timed up and go test**												
Blennerhassett 2004	Some concerns	Randomisation was performed by a person independent from the study drawing a pre‐sealed opaque envelope that specified group allocation. Stroke onset to start varied between 2 groups (mobility mean 36 days, upper limb mean 50.1 days)	Low risk of bias	"The duration of interdisciplinary therapy was recorded. The amount of physiotherapy time related to mobility and upper limb tasks was also documented. Subjects were not blinded to the research procedure although they were not told of the study hypotheses. Treating physiotherapists were not told of group allocations although they may have found out through interaction with subjects during physiotherapy treatments." "Mobility circuit classes were conducted separately from the Upper Limb sessions" reducing possibility of contamination between groups. "Independent sample t‐tests and chi‐square tests were used to examine between‐group differences for baseline, treatment time and length of stay data"	Low risk of bias	30 randomised. "All subjects completed four weeks of additional training and follow‐up was 100% at four weeks and 97% at six months. All subjects completed the mobility and MAS measurements on the initial and four week test."	Low risk of bias	Gait velocity. "An independent assessor who was blinded to group allocation and previous test results, and was not involved in the treatment of the subject, performed all tests. The order of tests was consistent for all subjects. Testing procedures were standardised in accordance to previous reports."	Some concerns	No protocol available.	Some concerns	Some concerns about baseline differences and lack of protocol.
FeSTivaLS 2014	Low risk of bias	"An independent randomization service concealed group allocation until contacted by a researcher," "Group allocation to either FST‐UL or FST‐LL will be deter‐mined by telephone call to an independent automated system within the Norwich Clinical Trials Unit. Minimiza‐ tion of baseline imbalance between treatment groups will be based on the Pocock and Simon’s range method and used to determine the allocation for each participant by Functional Ambulation category (FAC) (22) and Action Research Arm Test (ARAT) (23). " "All characteristics were balanced across the two groups except for stroke classification. The FST‐UL group had a higher percentage of people clinically classified as having a partial anterior circulation stroke and the FST‐LL had a higher percentage of people as having a lacunar stoke or posterior circulatory stroke"	Low risk of bias	Assignment was to either upper limb or lower limb training. "All participants received the intervention as allocated except one participant who withdrew from the FST‐LL group as he wanted FST‐UL. The content of FST‐UL and FST‐LL was consistent with the protocol (Table 3) and the amount of therapy was essentially the same in the two groups", reducing likelihood of contamination. Participants were analysed according to the group to which they were randomised.	Low risk of bias	52 participants randomised; data available for 44. Reasons for dropouts / missing data reported, and balanced between groups. Reasons for dropouts / missing data reported, and balanced between groups.	Low risk of bias	Gait velocity. "The assessor, who conducted the efficacy and health economics measurement battery at baseline, outcome, and follow‐up time‐points, remained blinded to participants’ group allocation throughout the trial."	Low risk of bias	Published protocol and trial registration. No evidence of differences between protocol and conducted study.	Low risk of bias	No concerns ‐ judged as low risk of bias for all domains.

**Table:** Risk of bias for analysis 3.5 Length of stay

**Study**	**Bias**											
	**Randomisation process**		**Deviations from intended interventions**		**Missing outcome data**		**Measurement of the outcome**		**Selection of the reported results**		**Overall**	
	**Authors' judgement**	**Support for judgement**	**Authors' judgement**	**Support for judgement**	**Authors' judgement**	**Support for judgement**	**Authors' judgement**	**Support for judgement**	**Authors' judgement**	**Support for judgement**	**Authors' judgement**	**Support for judgement**
Blennerhassett 2004	Some concerns	Randomisation was performed by a person independent from the study drawing a pre‐sealed opaque envelope that specified group allocation. Stroke onset to start varied between 2 groups (mobility mean 36 days, upper limb mean 50.1 days)	Low risk of bias	"The duration of interdisciplinary therapy was recorded. The amount of physiotherapy time related to mobility and upper limb tasks was also documented. Subjects were not blinded to the research procedure although they were not told of the study hypotheses. Treating physiotherapists were not told of group allocations although they may have found out through interaction with subjects during physiotherapy treatments." "Mobility circuit classes were conducted separately from the Upper Limb sessions" reducing possibility of contamination between groups. "Independent sample t‐tests and chi‐square tests were used to examine between‐group differences for baseline, treatment time and length of stay data"	Low risk of bias	30 randomised. "All subjects completed four weeks of additional training and follow‐up was 100% at four weeks and 97% at six months. All subjects completed the mobility and MAS measurements on the initial and four week test."	Low risk of bias	Length of stay is an objective measure, unlikely to be influenced by a study assessor.	Some concerns	No protocol available.	Some concerns	Some concerns about baseline differences and lack of protocol.

**Table:** Risk of bias for analysis 3.6 Adverse events

**Study**	**Bias**											
	**Randomisation process**		**Deviations from intended interventions**		**Missing outcome data**		**Measurement of the outcome**		**Selection of the reported results**		**Overall**	
	**Authors' judgement**	**Support for judgement**	**Authors' judgement**	**Support for judgement**	**Authors' judgement**	**Support for judgement**	**Authors' judgement**	**Support for judgement**	**Authors' judgement**	**Support for judgement**	**Authors' judgement**	**Support for judgement**
Dean 2006	Low risk of bias	"Randomisation will be stratified by stroke club site using a computer gen‐erated random number schedule with variable block sizes 2–6. Generation of the randomisation sequence will be performed centrally by a researcher not involved in recruitment or assessment. Group allocation will be con‐cealed using consecutively‐numbered opaque envelopes. The opaque envelope will be opened after completion of the assessment in the presence of the participant." "At baseline, the groups were similar in terms of demographic characteristics and other comorbidities"	Low risk of bias	"The participants and therapists delivering the intervention could not be blinded to intervention group allocation" Both treatment groups received similar programs, but one focussed on lower limb and one on upper limb. "The experimental and control classes were held in different areas of the stroke club and at different times to minimize the risk of “contamination” between the groups." Intention to treat analysis.	Low risk of bias	151 randomised. 65/76 in experimental and 68/75 in control group at end of intervention. Drop outs were accounted for and balanced between groups. 2 participants (both from control group) refused to continue classes and reassessment.	High risk of bias	Falls was defined as a primary outcome, but not wider adverse events. Other minor adverse events are not reported. "Falls will be assessed by comparing the number of falls in intervention and control groups. The proportion of fallers in each group will also be compared. Falls will be monitored for one year with monthly fall calendars. All participants will receive monthly calendars on entry to the study, with instructionsto record the following events: number of falls, visits to or by nursing and allied health personnel, general practi‐tioner or specialists appointments and hospitalisations.Participants will be asked to return the completed calen‐dar monthly at their weekly exercise class. If calendars are not returned, further contact will be made by telephone. Details of any falls (including how and where the fallo ccurred, injuries suffered, medical intervention required and limitations to activity as a result of a fall) will be ver‐ified". Adverse Events results: "No falls or other adverse events occurred during theexercise classes, home program, or assessments. Of the 18 withdrawals, only 1 was related to the intervention:1 participant withdrew as the experimental exercise exacer‐bated an incontinence problem. Of the 3 deaths during the trial, 2 may have been falls related: 1 participant died sev‐eral months after a fall at home, and 1 had a stroke andfractured his shoulder and died in hospital."	Some concerns	Trial registered and study protocol published. ACTRN12606000479505. Falls were pre‐stated as a primary outcome, but protocol does not state plans to record other adverse events.	High risk of bias	Judged as high risk of bias as falls were recorded, but not other adverse events.
Pang 2005	Some concerns	No information relating to allocation concealment. There was no significant difference in any of the variables between the intervention and control groups at baseline.	Low risk of bias	Participants were instructed not to tell the assessors about the group assignment or the treatment they received and not to discuss the protocol with the stroke community. Participants were analysed according to the group to which they were randomised.	Low risk of bias	63 enrolled. 3 dropouts. Small number of drop outs, all accounted for and not related to trial intervention.	Some concerns	Adverse events were not stated as an outcome, but it is stated that "Adverse events (e.g.falls) were monitored and recorded. A fall was defined as unintentionally coming to rest onthe floor or another lower level".	Some concerns	No protocol identified.	Some concerns	Some concerns as no protocol and adverse events not defined as an outcome.
Stuart 2019	Low risk of bias	"Qualified individuals, following informed consent, were block randomized." "There were no statistically significant differences between the groups in terms of demographics or functional measures at baseline, with the exception of the SPPB subscale for walking (P = .04)"	Low risk of bias	The same instructors and community locations were used for both exercise interventions. Treatment fidelity was monitored at least monthly, and when deemed necessary, weekly. When a protocol deviation was noted, this was reviewed with the instructor, and the frequency of monitoring increased.	High risk of bias	Concerns due to large percentage of drop‐outs: "Of APA participants, 58% completed the 6‐month intervention and functional tests, compared to 70% of Sittercise participants. Seventeen subjects (9 APA and 8 Sittercise) continued after the completion of the 6‐month study."	Low risk of bias	"Safety was measured using Study Related Adverse Events, which were further classified as Serious Adverse Events(those which required emergency care or subsequent hos‐pitalization) and Other Adverse Events (those not classi‐fied as serious)."	Some concerns	Some concerns as no protocol identified.	High risk of bias	High risk of bias due to large numbers of drop‐outs. Some concerns due to lack of identified protocol.

**Table:** Risk of bias for analysis 4.2 Motor function scales

**Study**	**Bias**											
	**Randomisation process**		**Deviations from intended interventions**		**Missing outcome data**		**Measurement of the outcome**		**Selection of the reported results**		**Overall**	
	**Authors' judgement**	**Support for judgement**	**Authors' judgement**	**Support for judgement**	**Authors' judgement**	**Support for judgement**	**Authors' judgement**	**Support for judgement**	**Authors' judgement**	**Support for judgement**	**Authors' judgement**	**Support for judgement**
**Subgroup 4.2.1 Other motor function measure**												
FeSTivaLS 2014	Low risk of bias	"An independent randomization service concealed group allocation until contacted by a researcher," "Group allocation to either FST‐UL or FST‐LL will be deter‐mined by telephone call to an independent automated system within the Norwich Clinical Trials Unit. Minimiza‐ tion of baseline imbalance between treatment groups will be based on the Pocock and Simon’s range method and used to determine the allocation for each participant by Functional Ambulation category (FAC) (22) and Action Research Arm Test (ARAT) (23). " "All characteristics were balanced across the two groups except for stroke classification. The FST‐UL group had a higher percentage of people clinically classified as having a partial anterior circulation stroke and the FST‐LL had a higher percentage of people as having a lacunar stoke or posterior circulatory stroke"	Low risk of bias	Assignment was to either upper limb or lower limb training. "All participants received the intervention as allocated except one participant who withdrew from the FST‐LL group as he wanted FST‐UL. The content of FST‐UL and FST‐LL was consistent with the protocol (Table 3) and the amount of therapy was essentially the same in the two groups"	Low risk of bias	Data available at follow up: 44. Reasons for dropouts / missing data reported, and balanced between groups.	Low risk of bias	"The assessor, who conducted the efficacy and health economics measurement battery at baseline, outcome, and follow‐up time‐points, remained blinded to participants’ group allocation throughout the trial."	Low risk of bias	Published protocol and trial registration. No evidence of differences between protocol and conducted study.	Low risk of bias	No concerns ‐ judged as low risk of bias for all domains.
McClellan 2004	Low risk of bias	"To ensure allocation was concealed, randomisation was by numbered, sealed, opaque envelopes." No baseline differences.	Some concerns	"To increase the likelihood that subjects were blind to group allocation, neither the exact purpose of the research nor the types of exercises that subjects would be receiving were specified and both mobility and upper limb function was measured." However, it is not clear if same therapists delivered intervention to both groups, which could result in deviations from intended interventions.	Low risk of bias	26 randomised; data available at week 14 : experiment group 13/15, control group 10/11 Drop out reasons discussed, and were balanced between groups.	Low risk of bias	"Outcome measures were collected at Weeks 0, 6, and 14 by a measurer blinded to group allocation."	Some concerns	No protocol identified.	Some concerns	Some concerns due to lack of information relating to potential deviations from intended interventions, and lack of protocol.
Mudge 2009	Low risk of bias	"Use of computer‐generated random numbers by an individual not associated with the study. Randomization was revealed to each participant by the principal investigator after the second baseline assessment." "There were differences between control and exercise group clinical tests at baseline. The exercise group had greater distance on the 6MWT ( P .028), mean steps a day (P .021), peak activity index (P .008), and highest step rate in 1 minute (P.019) (see table 2). Imbalances seen were likely to be a result of chance because they were collected while randomization was concealed from the assessor and the participant. These differences were used as covariates in subsequent analysis."	Low risk of bias	"exercise group led by 1 of the investigators (S.M.) assisted by 2 physiotherapy students. control group was run by an occupational therapist and consisted of 4 social and 4 educational sessions". Participants lived in the community and interventions were carried out in a clinic. Limited opportunity for contamination between groups.	Low risk of bias	60 participants randomised; At 3 month follow up 20/31 (expt) and 23/27 (control) left in groups. Drop outs accounted for	Low risk of bias	"Outcome assessment was performed by an independent physiotherapist blind to treatment assignment. Participants were not blind because they were aware of their own group allocation, which was revealed after the second testing session. Participants were instructed not to discuss group allocation with the assessor. The testing sessions were carried out in the same rehabilitation clinic as the intervention groups but were scheduled at different times to maintain blinding of the assessor. Unmasking of the independent assessor occurred in the case of 3 participants who inadvertently stated or implied their group allocation" (small number only).	Some concerns	No protocol identified or referenced.	Some concerns	Low risk of bias for all domains except for selection of reported results were there are some concerns due to the lack of a pre‐specified protocol.

**Table:** Risk of bias for analysis 5.1 Independence in ADL scales

**Study**	**Bias**											
	**Randomisation process**		**Deviations from intended interventions**		**Missing outcome data**		**Measurement of the outcome**		**Selection of the reported results**		**Overall**	
	**Authors' judgement**	**Support for judgement**	**Authors' judgement**	**Support for judgement**	**Authors' judgement**	**Support for judgement**	**Authors' judgement**	**Support for judgement**	**Authors' judgement**	**Support for judgement**	**Authors' judgement**	**Support for judgement**
**Subgroup 5.1.1 Barthel Index**												
Bordoloi 2020	High risk of bias	States "the patients were randomly divided into two groups (A and B) using block randomization (blocks of four) to achieve the predetermined sample size. The consultant physiotherapist (first author), with the help of second author, generated the random allocation sequence and enrolled participants based on the inclusion and exclusion criteria"	Some concerns	States: "Group B was taught exercises based on Rood’s approach which included ..... The Group A (control) patients and caregivers were blinded from the Rood’s approach techniques" Lack of information to judge if there may have been deviations because of the trial context.	Some concerns	"Out of the 236 patients, completely study was conducted for 198 patients while 38 patients were lost/missing/did not turn up during follow up". (16% drop out) . Drop‐outs/missing data are balanced between groups, with 10 control and 13 treatment group participants having missing data due to "missed phoned connectivity" and 8 control and 7 treatment group participants dying.	Some concerns	No information provided about blindiing of outcome assessors. No evidence to suggest that assessment was influenced by knowledge of intervention.	Some concerns	No protocol obtained.	High risk of bias	Lack of allocation concealment creates high risk of bias. Lack of information to judge if there may have been deviations because of the trial context; lack of information about blinding of outcome assessor and no protocol available.
Cao 2014	Some concerns	"randomly divided.....by numerical method". No further information. There was no significant difference in FMA and MBL scores between the two groups before treatment (all P>0.05)	Low risk of bias	Control group therapy implemented at home, reducing opportunities for contamination.	Low risk of bias	86 participants randomised. No drop outs or missing data.	Some concerns	Not stated if outcome assessor was blinded.	Some concerns	No reference to protocol	Some concerns	Some concerns due to lack of information and lack of study protocol.
Du 2014	Some concerns	States randomisation was done using "random number table method" but no further details provided. States there was no significant difference in the baseline data.	Some concerns	Not clear if same therapists involved in control and intervention, potentially providing opportunities for contamination between groups.	Low risk of bias	No evidence of dropouts or missing data.	Some concerns	No information relating to blinding of outcome assessor.	Some concerns	No protocol available.	Some concerns	Some concerns due to lack of information and lack of protocol.
Duncan 1998	Low risk of bias	"After baseline assessments, the subjects were randomly assigned to the experimental or control group. Randomization was done in blocks of 10. Before initiation of this study, a random list was generated by group assignments. Only a laboratory technician who had no input into subject selection or recruitment was aware of group assignment. After baseline assessment, the technician assigned thesubject to the experimental or the control group."	Low risk of bias	"The clinicians providing therapeutic interventions to the usual care group were asked to complete an intervention log to capture type of exercises and frequency and duration of therapy visits during treatment or in a home exercise program. The study coordinator met with the treating therapists at least twice to discuss the therapy logs and intervention programs". These strategies are considered to make it unlikely that there were deviations from intended interventions. Participants were analysed according to the group to which they were randomised.	Low risk of bias	"Twenty individuals with stroke were studied. Of 22 subjects recruited, 2 refused to participate." Data available for 20 participants.	Some concerns	Research assistants visited homes of the subjects to assess. Not stated if they knew whether patient was in intervention group, but possible.	Some concerns	Protocol not available	Some concerns	Potential bias in measurement of outcome due to research assistant awareness of group allocation. No protocol available.
Fang H 2010	Some concerns	No details as to randomisation process provided.	Some concerns	No information regarding whether same therapist delivered across both groups, which could have impacted on delivery of interventions.	Low risk of bias	No dropouts	High risk of bias	Blinding not reported.	Some concerns	No reference to a protocol .	High risk of bias	High risk of bias due to potential lack of blinding. Some concerns due to lack of information and lack of protocol.
Guan 2017	Some concerns	Divided into 2 groups using the random number table method. No information relating to allocation concealment.	Some concerns	No information given as to whether different therapists for control/intervention groups, meaning that there is a potential for contamination between groups.	Low risk of bias	No evidence of drop outs or missing data.	Some concerns	No information relating to blinding of outcome assessor.	Some concerns	No protocol available.	Some concerns	Some concerns due to lack of information available.
Kim 2007	Some concerns	States "randomised", but no description of randomisation or allocation process.	Some concerns	No information about whether same therapists involved in control/intervention, creating some concerns about potential for contamination between groups. Participants were analysed according to the group to which they were randomised.	Some concerns	3 drop‐outs from control group "due to emergency discharge", none from treatment group. Not clear whether the "emergency discharge" was related to the trial context. Possible that the discharged patients were those with better outcomes.	Some concerns	Not clear if outcome assessor was blinded. No evidence that knowledge of intervention influenced outcome	Some concerns	No protocol available.	Some concerns	Some concerns, primarily due to lack of information.
Kim 2014	Low risk of bias	"1 Randomization was performed with a computer using a basic random number generator." Subjects didn't know which intervention they'd been allocated to.	Low risk of bias	"single blinded study" ‐ participants did not know which of two treatment groups they were assigned to. Participants were analysed according to the group to which they were randomised.	Low risk of bias	No evidence of drop‐outs or missing data.	Some concerns	Method was Barthel Index. No information relating to blinding, or who performed outcome assessment (states "single‐blind study" but unclear what this relates to).	Some concerns	No protocol available.	Some concerns	Some concerns due to lack of information relating to blinding and lack of protocol.
LAST 2018	Low risk of bias	"Participants randomly assigned (1:1) in blocks of 2 and 4, to an intervention group receiving individualized coaching on physical activitiy or to a control group receiving standard care. Randomization was performed by a web‐based randomization system." "The randomisation procedure will be computerised and organised by Unit for Applied Clinical Research at Norwegian University of Science and Technology (NTNU)." Demographic and baseline characteristics were similar in both groups.	Low risk of bias	Intervention was delivered as outpatient, limiting opportunities for contamination between groups.	High risk of bias	Intervention group 42/184 discontinued allocated intervention; control group 9/194 discontinued allocated intervention. Intervention group 153/186 assessed, control group 162/194 assessed. Substantially more discontinued from intervention group than control group. Increased drop outs in intervention group are likely to be related to acceptability of the intervention.	Low risk of bias	"A group of well‐trained research assistants, blinded to the treatment allocation, screened patients for eligibility and did all assessments face‐to‐face at inclusion and at 18 month follow up."	Low risk of bias	Published study protocol and trials registry. Data presented are in accordance with pre‐specified protocol.	High risk of bias	Concerns due to greater number of drop outs in intervention than control group, which could be related to the intervention. Considered low risk of bias for other domains.
Lee 2018	Low risk of bias	"patients were randomly assigned to either the trial group (n=35) or the control group (n=37) using a permuted block design. This was done by a study personnel who was blinded to details of the current study"	Low risk of bias	All participants received conventional rehabilitation. The additional rehab "...was done by a physical therapist under the supervision of a physician who was not involved in the current study." Participants were analysed in the group to which they were assigned.	Some concerns	"The 80 recruited patients were initially assigned to the trial group (n=40) and the control group (n=40). Of these patients in the trial group, 2 and 3 discontinued the study because of discharge and non‐compliance, respectively. Of patients in the control group, 3 discontinued the study because of discharge. Therefore, 35 and 37 patients were assigned to the trial group and the control group, respec‐ tively." Number of missing participants were small, but 3 of the missing participants from the treatment group were due to "non compliance" suggesting that this could be related to the treatment. While the 3 participants missing from the treatment group due to non‐compliance could have affected the result, the chance of this is considered low due to the small number of missing participants.	Low risk of bias	states "observer‐blind"	Some concerns	No protocol obtained.	Some concerns	Some concerns due to missing data from participants with low compliance with the intervention, and lack of protocol. Considered low risk of bias for other domains.
Letombe 2010	Some concerns	States "randomised", but no information on randomisation or allocation concealment provided.	Some concerns	Both groups possibly treated by same therapists, providing potential for contamination between groups.	Low risk of bias	No evidence of drop outs or missing data.	Some concerns	Not stated who conducted assessments, or whether they were blinded.	Some concerns	Refers to a study protocol, but we have not obtained this.	Some concerns	Some concerns due to lack of information relating to study conduct.
Li Jingqian 2013	Some concerns	Randomisation process not clearly defined. There was no significant difference in age, gender, lesion nature and lesion location between the two groups (P > 0.05), and they were comparable.	Some concerns	Possibly same therapist involved in both groups, but insufficient information to judge if this could have contributed to deviations from intended interventions.	Low risk of bias	No evidence of any dropouts or missing data.	Some concerns	Blinding of assessors not discussed.	Some concerns	No reference to a protocol.	Some concerns	Some concerns due to lack of information and lack of protocol.
Li Xiaojun 2016	Some concerns	States randomised via a mathematical table, but lack of information about allocation concealment.	Some concerns	Insufficient information. Not clear if same therapist delivered across both groups, which could have led to contamination.	Some concerns	States 9 dropouts from treatment group and 8 from control group. Balanced number of dropouts suggests that this was not due to the study intervention.	Some concerns	Blinding not reported.	Some concerns	No reference to a protocol .	Some concerns	Some concerns due to lack of information.
Mai Guanghuai 2016	Some concerns	States randomised. Lack of information about allocation concealment. No differences between groups at baseline.	Some concerns	Insufficient information to judge if there were deviations from intended interventions because of trial context. Participants were analysed according to assigned intervention.	Low risk of bias	No drop outs or missing data reported.	High risk of bias	Not clear if outcome assessor was blinded. Insufficient information to judge if outcome could have been influenced by knowledge of intervention received.	Some concerns	No protocol obtained.	High risk of bias	This is a Chinese language trial, with sections translated by one member of our team. There is generally a lack of information about the study methods. It is not clear whether the outcome assessor was blinded.
Qin JianJian 2014	Some concerns	States "randomly divided" but no discussion on randomisation process. No significant difference in general data between the two groups (P>0.05)	Some concerns	Not clear if same therapists across both groups, which could cause potential for contamination between groups.	Low risk of bias	No evidence of dropouts or missing data.	Some concerns	No information about whether assessors were blinded.	Some concerns	No protocol available.	Some concerns	Some concerns due to lack of information and lack of protocol.
Wu Jiaming 2006	Some concerns	States randomised. Lack of information about allocation concealment. No differences between groups at baseline.	Some concerns	Insufficient information to judge if there were deviations from intended interventions because of trial context. Participants were analysed according to assigned intervention.	Low risk of bias	No drop outs or missing data reported.	High risk of bias	Not clear if outcome assessor was blinded. Insufficient information to judge if outcome could have been influenced by knowledge of intervention received.	Some concerns	No protocol obtained.	High risk of bias	This is a Chinese language trial, with sections translated by one member of our team. There is generally a lack of information about the study methods. It is not clear whether the outcome assessor was blinded.
Wu Lotus 2016	Some concerns	States randomised. Lack of information about allocation concealment. No differences between groups at baseline.	Some concerns	Insufficient information to judge if there were deviations from intended interventions because of trial context. Participants were analysed according to assigned intervention.	Low risk of bias	No drop outs or missing data reported.	High risk of bias	Not clear if outcome assessor was blinded. Insufficient information to judge if outcome could have been influenced by knowledge of intervention received.	Some concerns	No protocol obtained.	High risk of bias	This is a Chinese language trial, with sections translated by one member of our team. There is generally a lack of information about the study methods. It is not clear whether the outcome assessor was blinded.
Xu 2013	Some concerns	States "randomly divided" but randomisation details not provided. There was no significant difference in age, gender composition ratio, educational level, and nature of lesions between the two groups, and they were comparable.	Some concerns	Insufficient information to judge if there were deviations due to study context.	Low risk of bias	No evidence of any dropouts or missing data.	Some concerns	Blinding of assessors not discussed	Some concerns	No protocol available.	Some concerns	Some concerns due to lack of information and lack of protocol.
Yang 2017	Some concerns	"Randomly divided". No information about allocation concealment. "Before treatment, no significant differences in FMA and MBI scores were found amoung the three groups (p>0.05)".	Some concerns	Insufficient information to judge if likely deviations from intended interventions.	Low risk of bias	No evidence of drop‐outs.	High risk of bias	Not clear if outcome assessor was blinded. Insufficient information to judge if outcome could have been influenced by knowledge of intervention received.	Some concerns	No protocol available	High risk of bias	This is a Chinese language trial, with sections translated by one member of our team. There is generally a lack of information about the study methods. It is not clear whether the outcome assessor was blinded.
Yue Chunjiang 2014	Some concerns	States "randomly divided" but randomisation process not discussed. No statistical significant difference between the groups at baseline.	Some concerns	Possibly same therapists working across both groups, meaning that there may have been potential for contamination between groups. But insufficient information to judge. Participants were analysed according to group to which they were assigned.	Low risk of bias	90 participants randomised. No evidence of dropouts or missing data.	High risk of bias	No information relating to blinding of outcome assessors. Insufficient information to judge if outcome assessment could have been influenced by lack of blinding.	Some concerns	No protocol.	High risk of bias	High risk of bias due to potential lack of blinded outcome assessor. Some concerns due to lack of information and lack of protocol.
Zhuang 2012	Low risk of bias	"The team’s data management center generated the randomization numbers with SAS9.1.3 (Statistical Analysis System provided by SAS Institute Inc,Cary, North Carolina). Each of the seven sites had a designated research assistant who was responsible for obtaining a random number for each participant from a web‐based, password‐protected Internet site and who actually assigned the participant to one of the three treatment groups" "At baseline, no significant differences existed between the three groups in terms of gender, age, or length or severity of disease (P >.05)"	Low risk of bias	‘research assistant instructed participants not to discuss other treatments that they were receiving with their therapists’	Low risk of bias	310 participants were screened, of those: "274 completed the study, 15 did not meet the inclusion/exclusion criteria, and 21 dropped out. Adverse events were rare (less than 1%), mild, and temporary" (i.e. 21 drop outs from 295 possible completers). ‘Of those who dropped out, nine left the hospital, eight discontinued treatment, two dropped out due to poor health, one (from the acupuncture group) suffered a second stoke and one (from the physiotherapy group) died due to a respiratory tract infection’. Non‐completers appear balanced across groups.	Low risk of bias	"Physicians who performed the outcome assessments were blinded to treatment assignments."	Some concerns	No protocol available.	Some concerns	No protocol available so potential bias in selection of the reported result, but judged as low risk of bias for other domains.

**Table:** Risk of bias for analysis 5.2 Motor function scales

**Study**	**Bias**											
	**Randomisation process**		**Deviations from intended interventions**		**Missing outcome data**		**Measurement of the outcome**		**Selection of the reported results**		**Overall**	
	**Authors' judgement**	**Support for judgement**	**Authors' judgement**	**Support for judgement**	**Authors' judgement**	**Support for judgement**	**Authors' judgement**	**Support for judgement**	**Authors' judgement**	**Support for judgement**	**Authors' judgement**	**Support for judgement**
**Subgroup 5.2.1 Fugl Meyer**												
Du 2014	Some concerns	States randomisation was done using "random number table method" but no further details provided. States there was no significant difference in the baseline data.	Some concerns	Not clear if same therapists involved in control and intervention, potentially providing opportunities for contamination between groups.	Low risk of bias	No evidence of dropouts or missing data.	Some concerns	No information relating to blinding of outcome assessor.	Some concerns	No protocol available.	Some concerns	Some concerns due to lack of information and lack of protocol.
Duncan 1998	Low risk of bias	"After baseline assessments, the subjects were randomly assigned to the experimental or control group. Randomization was done in blocks of 10. Before initiation of this study, a random list was generated by group assignments. Only a laboratory technician who had no input into subject selection or recruitment was aware of group assignment. After baseline assessment, the technician assigned thesubject to the experimental or the control group."	Low risk of bias	"The clinicians providing therapeutic interventions to the usual care group were asked to complete an intervention log to capture type of exercises and frequency and duration of therapy visits during treatment or in a home exercise program. The study coordinator met with the treating therapists at least twice to discuss the therapy logs and intervention programs". These strategies are considered to make it unlikely that there were deviations from intended interventions. Participants were analysed according to the group to which they were randomised.	Low risk of bias	"Twenty individuals with stroke were studied. Of 22 subjects recruited, 2 refused to participate." Data available for 20 participants.	Some concerns	Research assistants visited homes of the subjects to assess. Not stated if they knew whether patient was in intervention group, but possible.	Some concerns	Protocol not available	Some concerns	Potential bias in measurement of outcome due to research assistant awareness of group allocation. No protocol available.
Duncan 2003	Low risk of bias	"...randomly assigned...through the use of a random number generator with a block size of 6 and sealed envelopes". No further information relating to allocation concealment. "The 2 treatment arms were comparable in all baseline measures".	High risk of bias	Participants were not blinded to their assignment but were unaware of the study hypotheses or primary outcome measures. The amount of rehabilitation provided to the usual care group is reported: "In the usual care group, 46% of the subjects did not recieve any postacute rehabilitation services from physical or occu‐ pational therapy. Two thirds were provided recommendations for an unsupervised exercise program. Among the usual care group members who did receive therapy, participants re‐ ceived an average of 8.7+/‐5.3 physical therapy visits and 10.4+/‐7 occupational therapy visits. Physical and occupa‐tional therapy services were received separately as prescribed by their physicians. The total duration of the combined physical and occupational therapy visits in the usual care group was similar to the intervention group (90 minutes)." It is unclear if this deviated from usual care prior to the trial. 46% of the participants in the usual care group got comparable amounts of rehabilitation to the treatment group. It is possible that more "usual" care was given to the usual care group than the treatment group.	Some concerns	100 participants were randomised. "Ninety‐two subjects completed the 3‐month post treatment assessment; 8 dropped out. Six subjects dropped from theinterven assessment; 8 dropped out. Six subjects dropped from the intervention arm: 1 had significant renal insufficiency de‐tected after randomization but before therapy; 1 had sub cla‐vian steal syndrome diagnosed the first 2 weeks after ran‐domization; 1 chose to withdraw after 18 visits; and 3 experienced a second stroke. Two subjects dropped from the usual care group: 1 withdrew from the study immediately after randomization, and 1 did not return for the 3‐month assessment."	Low risk of bias	Outcome assessment was performed by research staff blinded to treatment assignment.	Some concerns	No study protocol.	High risk of bias	High risk of bias due to concerns about potential deviations from intended deviations. Some concerns as no study protocol., but judged as low risk of bias for other domains.
Hong Cuicui 2016	Some concerns	States randomisation via "lot picking" (translated from Chinese). No information about allocation concealment.	Some concerns	Not clear if same therapist delivered across both groups.	Low risk of bias	No dropouts or missing data.	Some concerns	Blinding not reported.	Some concerns	No reference to a protocol .	Some concerns	Some concerns due to lack of information for all domains, and no study protocol.
Li Xiaojun 2016	Some concerns	States randomised via a mathematical table, but lack of information about allocation concealment.	Some concerns	Not clear if same therapist delivered across both groups.	Some concerns	States 9 dropouts from treatment group and 8 from control group. Balanced number of dropouts suggests that this was not due to the study intervention.	Some concerns	Blinding not reported	Some concerns	No protocol.	Some concerns	Some concerns due to lack of information across all domains, and lack of protocol.
Mai Guanghuai 2016	Some concerns	States randomised. Lack of information about allocation concealment. No differences between groups at baseline.	Some concerns	Insufficient information to judge if there were deviations from intended interventions because of trial context. Participants were analysed according to assigned intervention.	Low risk of bias	No drop outs or missing data reported.	High risk of bias	Not clear if outcome assessor was blinded. Insufficient information to judge if outcome could have been influenced by knowledge of intervention received.	Some concerns	No protocol obtained.	High risk of bias	This is a Chinese language trial, with sections translated by one member of our team. There is generally a lack of information about the study methods. It is not clear whether the outcome assessor was blinded.
Qin JianJian 2014	Some concerns	States "randomly divided" but no discussion on randomisation process. No significant difference in general data between the two groups (P>0.05)	Some concerns	Not clear if same therapists across both groups, which could cause potential for contamination between groups.	Low risk of bias	No evidence of dropouts or missing data.	Some concerns	No information about whether assessors were blinded.	Some concerns	No protocol available.	Some concerns	Some concerns due to lack of information and lack of protocol.
Tang 2009	Some concerns	States random but method of randomisation and concealment not reported.	High risk of bias	Not clear if both control and intervention delivered by same therapist, providing potential for contamination between groups.	Low risk of bias	No dropouts or missing data.	Some concerns	No information relating to blinding of outcome assessor.	Some concerns	No protocol available.	High risk of bias	Judged at high risk of bias relating to deviations from the intended interventions, with some concerns due to lack of information for all other domains.
Wang 2004b	Some concerns	No information provided about method of randomisation. Allocation was conducted by study authors. No significant baseline differences.	Some concerns	Insufficient information to judge if there could be deviations due to delivery of intervention.	Low risk of bias	No dropouts or missing data.	High risk of bias	Outcome assessment conducted by authors, with no evidence of any blinding.	Some concerns	No protocol identified.	High risk of bias	Judged as high risk of bias due to involvement of study authors in allocation and outcome measurement, with some concerns due to lack of information for other domains.
Wu Jiaming 2006	Some concerns	States randomised. Lack of information about allocation concealment. No differences between groups at baseline.	Some concerns	Insufficient information to judge if there were deviations from intended interventions because of trial context. Participants were analysed according to assigned intervention.	Low risk of bias	No drop outs or missing data reported.	High risk of bias	Not clear if outcome assessor was blinded. Insufficient information to judge if outcome could have been influenced by knowledge of intervention received.	Some concerns	No protocol obtained.	High risk of bias	This is a Chinese language trial, with sections translated by one member of our team. There is generally a lack of information about the study methods. It is not clear whether the outcome assessor was blinded.
Wu Lotus 2016	Some concerns	States randomised. Lack of information about allocation concealment. No differences between groups at baseline.	Some concerns	Insufficient information to judge if there were deviations from intended interventions because of trial context. Participants were analysed according to assigned intervention.	Low risk of bias	No drop outs or missing data reported.	High risk of bias	Not clear if outcome assessor was blinded. Insufficient information to judge if outcome could have been influenced by knowledge of intervention received.	Some concerns	No protocol obtained.	High risk of bias	This is a Chinese language trial, with sections translated by one member of our team. There is generally a lack of information about the study methods. It is not clear whether the outcome assessor was blinded.
Yang 2017	Some concerns	"Randomly divided". No information about allocation concealment. "Before treatment, no significant differences in FMA and MBI scores were found amoung the three groups (p>0.05)".	Some concerns	Insufficient information to judge if there were deviations from intended interventions because of trial context. Participants were analysed according to assigned intervention.	Low risk of bias	No drop outs or missing data reported.	High risk of bias	Not clear if outcome assessor was blinded. Insufficient information to judge if outcome could have been influenced by knowledge of intervention received.	Some concerns	No protocol obtained.	High risk of bias	This is a Chinese language trial, with sections translated by one member of our team. There is generally a lack of information about the study methods. It is not clear whether the outcome assessor was blinded.
Yue Chunjiang 2014	Some concerns	States "randomly divided" but randomisation process not discussed. No statistical significant difference between the groups at baseline.	Some concerns	Possibly same therapists working across both groups, meaning that there may have been potential for contamination between groups. But insufficient information to judge. Participants were analysed according to group to which they were assigned.	Low risk of bias	90 participants randomised. No evidence of dropouts or missing data.	High risk of bias	No information relating to blinding of outcome assessors. Insufficient information to judge if outcome assessment could have been influenced by lack of blinding.	Some concerns	No protocol.	High risk of bias	High risk of bias due to potential lack of blinded outcome assessor. Some concerns due to lack of information and lack of protocol.
Yue Lin 2012	Some concerns	"According to the random number table, the patients were randomly divided..." No information about allocation concealment. "There were no significant differences in age, gender, and lesion degree between the two groups (P>0.05)."	Some concerns	Insufficient information to judge if there were deviations from intended intervention.	Low risk of bias	92 participants randomised and data available for 92.	Some concerns	No information relating to who conducted the assessment and whether they were blinded.	Some concerns	No protocol identfied.	Some concerns	Some concerns due to lack of information for all domains, and lack of protocol.
Zhuang 2012	Low risk of bias	"The team’s data management center generated the randomization numbers with SAS9.1.3 (Statistical Analysis System provided by SAS Institute Inc,Cary, North Carolina). Each of the seven sites had a designated research assistant who was responsible for obtaining a random number for each participant from a web‐based, password‐protected Internet site and who actually assigned the participant to one of the three treatment groups" "At baseline, no significant differences existed between the three groups in terms of gender, age, or length or severity of disease (P >.05)"	Low risk of bias	‘research assistant instructed participants not to discuss other treatments that they were receiving with their therapists’	Low risk of bias	310 participants were screened, of those: "274 completed the study, 15 did not meet the inclusion/exclusion criteria, and 21 dropped out. Adverse events were rare (less than 1%), mild, and temporary" (i.e. 21 drop outs from 295 possible completers). ‘Of those who dropped out, nine left the hospital, eight discontinued treatment, two dropped out due to poor health, one (from the acupuncture group) suffered a second stoke and one (from the physiotherapy group) died due to a respiratory tract infection’. Non‐completers appear balanced across groups.	Low risk of bias	"Physicians who performed the outcome assessments were blinded to treatment assignments."	Some concerns	No protocol available.	Some concerns	No protocol available so potential bias in selection of the reported result, but judged as low risk of bias for other domains.
**Subgroup 5.2.2 Other motor function measure**												
Aries 2021	Low risk of bias	"Randomization of participants to one of two groups (MTS+TSGT or TIs+TSGT) was undertaken by the Norwich Clinical Trials Unit, using a computer randomization system in a 1:1 ratio with permuted blocks of two and four. Stratification by left‐ or right‐sided brain lesion (identified from the medical notes) was used because this factor may influence rehabilitation potential (36). Group allocation order was concealed from the research team until after measurement at baseline." "No differences were discernible between the MTS+TSGT and TI+TSGT groups at baseline"	Low risk of bias	"Research therapists (n = 4) received training in applying and delivering all interventions. To assess fidelity to protocol, the research therapists were observed by a senior member of the project team (SMH) at various points during the study to ensure they were working to protocol."	Low risk of bias	2 discontinued TI+TSGT intervention due to adverse events. 32/34 included in analysis.	Low risk of bias	"In addition, participants were asked not to disclose group allocation to the blinded assessor and the case report forms were not accessible to the blinded assessor. The blinded assessor guessed accurately the group allocation of just three of the 32 participants completing the study: 9.38% (95% CI 3.2%, 24.2%)"	Low risk of bias	The study was registered on a clinical trials database (ISRCTN 13676183; Central Portfolio Management System ID 30449). Data were analysed in line with this study protocol.	Low risk of bias	Study judged to be low risk of bias for all domains.
Cooke 2006	Low risk of bias	"An independent statistician produced a pretrial computer‐generated randomized group allocation order in blocks of 9 per trial center. Allocation was stratified by baseline scores for unilateral visual spatial neglect (Star Cancella‐tion Test, 50‐54 no spatial neglect and 0‐49 unilateral spatial neglect present). Allocation was concealed in sequentially numbered sealed opaque envelopes held by an independent administrator. Envelopes were opened in response to a tele‐phone request from the research physiotherapist (blinded to measures) after the assessor (blinded to group allocation) had completed baseline measures." No baseline differences.	Low risk of bias	"To ensure adherence to experimental treatment schedules, the research physiotherapists were supervisedthroughout the trial. The clinical team was given no infor‐mation about participants’ group allocation and was notpresent when the additional intervention was provided." Participants analysed according to assigned group.	Some concerns	"109 (10%) of those screened were randomized as follows: 38 to CPT, 35 to CPT + CPT, and 36 to FST + CPT. At outcome, 10 (9%) participants had withdrawn. At follow‐up, a further 18 participants had withdrawn (26%)." Dropouts were balanced between groups, suggesting that the result was not biased.	Low risk of bias	Blinded outcome assessor.	Some concerns	No published protocol, but a conference poster described the planned study. However this did not pre‐state Rivermead Mobility Index as an outcome (but not all outcomes were listed).	Some concerns	There were some concerns as there was no study protocol, but some details were provided in a conference poster. Judged as low risk of bias for other domains.
Guan 2017	Some concerns	Divided into 2 groups using the random number table method. No information relating to allocation concealment.	Some concerns	No information given as to whether different therapists for control/intervention groups, meaning that there is a potential for contamination between groups.	Low risk of bias	No evidence of drop outs or missing data.	Some concerns	No information relating to blinding of outcome assessor.	Some concerns	No protocol available.	Some concerns	Some concerns due to lack of information available.
Kunkel 2013	Some concerns	"Those willing to participate gave informed consent the following day; demographic data were col‐lected and FES tolerance tested, prior to randomization. The trial therapist then contacted the Medical Statistics Group by telephone to obtain a concealed, computer generated random allocation to one of the three groups:" Differences in baseline data for Rivermead Mobility index (treatment group 5.3(2.6); control group 7.7(3.2)), Berg balance (treatment group 27.5(10.6); control group 40.7(8.4)).	High risk of bias	"The usual care control group was included to enable comparison to standard care. While of theoretical importance, this design proved problematic. While the assessor was successfully blinded, NHS therapists were aware of the group allocation and it would be impossible to blind them in a future trial. In the event, non‐trial therapists altered the focus of their attention delivering substantially greater amounts of routine therapy to the control arm potentially masking treatment effects compared with control. " Participants were analysed according to the group to which they were assigned.	Low risk of bias	21 participants recruited and 7 randomised to each of 3 arms. Exercise group had data from 4/7 and usual care group data from 5/7. Reason for drop outs did not appear to be related to the trial intervenion. Drop outs from exercise group were due to urinary tract infection, medically well, and withdrawal (no stated reason). Drop out from usual care group was due to second stroke.	Low risk of bias	"assessor was successfully blinded"	Some concerns	No protocol identified.	High risk of bias	High risk of bias due to evidence of deviations from intended interventions. Differences at baseline for this outcome. Low risk of bias for other domains, allthough no study protocol identified.
LAST 2018	Low risk of bias	"Participants randomly assigned (1:1) in blocks of 2 and 4, to an intervention group receiving individualized coaching on physical activitiy or to a control group receiving standard care. Randomization was performed by a web‐based randomization system." "The randomisation procedure will be computerised and organised by Unit for Applied Clinical Research at Norwegian University of Science and Technology (NTNU)." Demographic and baseline characteristics were similar in both groups.	Low risk of bias	Intervention was delivered as outpatient, limiting opportunities for contamination between groups. Participants were analysed according to assigned intervention.	High risk of bias	Intervention group 42/184 discontinued allocated intervention; control group 9/194 discontinued allocated intervention. Intervention group 153/186 assessed, control group 162/194 assessed. Substantially more discontinued from intervention group than control group. Increased drop outs in intervention group are likely to be related to acceptability of the intervention.	Low risk of bias	"A group of well‐trained research assistants, blinded to the treatment allocation, screened patients for eligibility and did all assessments face‐to‐face at inclusion and at 18 month follow up."	Low risk of bias	Published study protocol and trials registry. Data presented are in accordance with pre‐specified protocol.	High risk of bias	Concerns due to greater number of drop outs in intervention than control group, which could be related to the intervention. Considered low risk of bias for other domains.
Li Jingqian 2013	Some concerns	Randomisation process not clearly defined. There was no significant difference in age, gender, lesion nature and lesion location between the two groups (P > 0.05), and they were comparable.	Some concerns	Possibly same therapist involved in both groups, but insufficient information to judge if this could have contributed to deviations from intended interventions.	Low risk of bias	No evidence of any dropouts or missing data.	Some concerns	Blinding of assessors not discussed.	Some concerns	No reference to a protocol.	Some concerns	Some concerns due to lack of information and lack of protocol.
Mustafaoğlu 2018	Some concerns	"Randomization was performed by random number generator of the Microsoft Office Excel Software, which gives a random number between 0 and 1 to each treatment columns (Group distribution: 0‐<0.33=CombTG, 0.33‐<0.66=CT, 0.66‐1=BWSTT). Sorting of random number row was performed from the largest to the smallest numbers by the sort and filter menu. To ensure a distribution balance among three groups, stratification of treatment assignments was accomplished by the ambulation levels according to the Functional Ambulation Scale (FAS)." Baseline assessments were conducted after randomisation, and it is unclear how allocation was concealed. There were no statistically significant differences in demographic and clinical features at baseline.	Some concerns	Insufficient information to judge if there may have been deviations from intended interventions, for example by the treating therapists being aware of allocated intervention. Participants analysed according to assigned group.	Low risk of bias	45 participants randomised, with no drop outs or missing data.	Low risk of bias	"primary and secondary outcome assessments at baseline and after training were performed by a blinded assessor."	Some concerns	There is a clinical trials registration (ClinicalTrials.gov ID:NCT02735148), but this appears to have been registered after completion of the trial.	Some concerns	Some concerns relating to the randomisation process and potential deviations from the intended interventions. Some concerns that the clinical trials registration may have been retrospective.

**Table:** Risk of bias for analysis 5.3 Balance (Berg Balance Scale)

**Study**	**Bias**											
	**Randomisation process**		**Deviations from intended interventions**		**Missing outcome data**		**Measurement of the outcome**		**Selection of the reported results**		**Overall**	
	**Authors' judgement**	**Support for judgement**	**Authors' judgement**	**Support for judgement**	**Authors' judgement**	**Support for judgement**	**Authors' judgement**	**Support for judgement**	**Authors' judgement**	**Support for judgement**	**Authors' judgement**	**Support for judgement**
Duncan 1998	Low risk of bias	"After baseline assessments, the subjects were randomly assigned to the experimental or control group. Randomization was done in blocks of 10. Before initiation of this study, a random list was generated by group assignments. Only a laboratory technician who had no input into subject selection or recruitment was aware of group assignment. After baseline assessment, the technician assigned thesubject to the experimental or the control group."	Low risk of bias	"The clinicians providing therapeutic interventions to the usual care group were asked to complete an intervention log to capture type of exercises and frequency and duration of therapy visits during treatment or in a home exercise program. The study coordinator met with the treating therapists at least twice to discuss the therapy logs and intervention programs". These strategies are considered to make it unlikely that there were deviations from intended interventions. Participants were analysed according to the group to which they were randomised.	Low risk of bias	"Twenty individuals with stroke were studied. Of 22 subjects recruited, 2 refused to participate." Data available for 20 participants.	Some concerns	Research assistants visited homes of the subjects to assess. Not stated if they knew whether patient was in intervention group, but possible.	Some concerns	protocol not available	Some concerns	Potential bias in measurement of outcome due to research assistant awareness of group allocation. No protocol available.
Duncan 2003	Low risk of bias	"...randomly assigned...through the use of a random number generator with a block size of 6 and sealed envelopes". No further information relating to allocation concealment. "The 2 treatment arms were comparable in all baseline measures".	High risk of bias	Participants were not blinded to their assignment but were unaware of the study hypotheses or primary outcome measures. The amount of rehabilitation provided to the usual care group is reported: "In the usual care group, 46% of the subjects did not recieve any postacute rehabilitation services from physical or occu‐pational therapy. Two thirds were provided recommendations for an unsupervised exercise program. Among the usual care group members who did receive therapy, participants re‐ ceived an average of 8.7+/‐5.3 physical therapy visits and 10.4+/‐7 occupational therapy visits. Physical and occupa‐tional therapy services were received separately as prescribed by their physicians. The total duration of the combined physical and occupational therapy visits in the usual care group was similar to the intervention group (90 minutes)." It is unclear if this deviated from usual care prior to the trial. 46% of the participants in the usual care group got comparable amounts of rehabilitation to the treatment group. It is possible that more "usual" care was given to the usual care group than the treatment group.	Low risk of bias	100 participants were randomised. "Ninety‐two subjects completed the 3‐month post treatment assessment; 8 dropped out. Six subjects dropped from theinterven assessment; 8 dropped out. Six subjects dropped from the intervention arm: 1 had significant renal insufficiency de‐tected after randomization but before therapy; 1 had sub cla‐vian steal syndrome diagnosed the first 2 weeks after ran‐domization; 1 chose to withdraw after 18 visits; and 3 experienced a second stroke. Two subjects dropped from the usual care group: 1 withdrew from the study immediately after randomization, and 1 did not return for the 3‐month assessment." Drop outs are accounted for and balanced between groups (small number).	Low risk of bias	Outcome assessment was performed by research staff blinded to treatment assignment.	Some concerns	no study protocol	High risk of bias	High risk of bias due to concerns about potential deviations from intended deviations. Some concerns as no study protocol., but judged as low risk of bias for other domains.
Fang H 2010	Some concerns	No details as to randomisation process provided.	Some concerns	No information regarding whether same therapist delivered across both groups, which could have impacted on delivery of interventions.	Low risk of bias	No dropouts reported.	High risk of bias	Blinding not reported	Some concerns	No reference to a protocol	High risk of bias	High risk of bias due to potential lack of blinding. Some concerns due to lack of information and lack of protocol.
Harjpal 2021	Some concerns	"Computer generated randomization" using "Sequentially numbered, sealed, opaque envelopes". "Randomisation and allocation was done by the primary researcher" ‐ unclear if the allocation sequence remained concealed or whether the primary researcher could have introduced bias. No signficant baselinie differences.	Some concerns	The treatment group received 20 minutes additional physiotherapy/day, 5 days/week. Insufficient information to judge whether there may have been deviations from the intended intervention, such as contamination between groups. Participants were analysed according to the assigned intervention group.	Low risk of bias	40 participants randomised. No drop outs or missing data.	Low risk of bias	Blinded outcome assessor.	Low risk of bias	Prospective trial registration: CTRI/2021/05/033621 Outcomes and analyses as described in protocol.	Some concerns	Some concerns due to lack of information regarding allocation concealment and potential for contamination between groups. Low risk of bias for other domains.
Kim 2007	Some concerns	States "randomised", but no description of randomisation or allocation process.	Some concerns	No information about whether same therapists involved in control/intervention, creating some concerns about potential for contamination between groups. Participants were analysed according to the group to which they were randomised.	Some concerns	3 drop‐outs from control group "due to emergency discharge", none from treatment group. Not clear whether the "emergency discharge" was related to the trial context. Possible that the discharged patients were those with better outcomes.	Some concerns	Not clear if outcome assessor was blinded. No evidence that knowledge of intervention influenced outcome.	Some concerns	No protocol available.	Some concerns	Some concerns, primarily due to lack of information.
Kim 2012	Some concerns	States "randomly assigned", but no further details provided. Limited baseline data provided.	Some concerns	Insufficient information to judge whether there could have been deviations. Studied interventions create potential for contamination between groups. Participants were analysed according to the assigned intervention.	Low risk of bias	20 participants randomised. No drop outs or missing data.	High risk of bias	Does not state who conducted outcome assessments and whether they were blinded. Insufficient information to judge whether outcome assessment could have been influenced by knowledge of intervention.	Some concerns	No protocol identified.	High risk of bias	High risk of bias due to potential bias in measurement of the outcome, with no blinded assessor; some concerns for other domains due to lack of information.
Kunkel 2013	Some concerns	"Those willing to participate gave informed consent the following day; demographic data were col‐lected and FES tolerance tested, prior to randomization. The trial therapist then contacted the Medical Statistics Group by telephone to obtain a concealed, computer generated random allocation to one of the three groups:" Differences in baseline data for Rivermead Mobility index (treatment group 5.3(2.6); control group 7.7(3.2)), Berg balance (treatment group 27.5(10.6); control group 40.7(8.4)).	High risk of bias	"The usual care control group was included to enable comparison to standard care. While of theoretical importance, this design proved problematic. While the assessor was successfully blinded, NHS therapists were aware of the group allocation and it would be impossible to blind them in a future trial. In the event, non‐trial therapists altered the focus of their attention delivering substantially greater amounts of routine therapy to the control arm potentially masking treatment effects compared with control. " Participants were analysed according to the group to which they were assigned.	Low risk of bias	21 participants recruited and 7 randomised to each of 3 arms. Exercise group had data from 4/7 and usual care group data from 5/7. Reason for drop outs did not appear to be related to the trial intervenion. Drop outs from exercise group were due to urinary tract infection, medically well, and withdrawal (no stated reason). Drop out from usual care group was due to second stroke.	Low risk of bias	"assessor was successfully blinded"	Some concerns	No protocol identified.	High risk of bias	High risk of bias due to evidence of deviations from intended interventions. Differences at baseline for this outcome. Low risk of bias for other domains, allthough no study protocol identified.
Lee 2018	Low risk of bias	"patients were randomly assigned to either the trial group (n=35) or the control group (n=37) using a permuted block design. This was done by a study personnel who was blinded to details of the current study"	Low risk of bias	All participants received conventional rehabilitation. The additional rehab "...was done by a physical therapist under the supervision of a physician who was not involved in the current study." Participants were analysed in the group to which they were assigned.	Some concerns	"The 80 recruited patients were initially assigned to the trial group (n=40) and the control group (n=40). Of these patients in the trial group, 2 and 3 discontinued the study because of discharge and non‐compliance, respectively. Of patients in the control group, 3 discontinued the study because of discharge. Therefore, 35 and 37 patients were assigned to the trial group and the control group, respec‐ tively." Number of missing participants were small, but 3 of the missing participants from the treatment group were due to "non compliance" suggesting that this could be related to the treatment. While the 3 participants missing from the treatment group due to non‐compliance could have affected the result, the chance of this is considered low due to the small number of missing participants.	Low risk of bias	states "observer‐blind"	Some concerns	No protocol obtained.	Some concerns	Some concerns due to missing data from participants with low compliance with the intervention, and lack of protocol. Considered low risk of bias for other domains.
Lindvall 2014	Low risk of bias	"Randomization to experimental or control intervention was conducted in blocks of four to six patients at each primary healthcare centre, in the form of sealed envelopes that were opened after the baseline tests" No baseline differences.	Some concerns	The intervention was delivered in addition to usual care and occured in a group setting, delivered in a location away from control group participants. However the authors state "The physical activity levels were not measured in either group and some of the participants allocated to the control group expressed disappointment at not receiving the training in the intervention and may have started exercising on their own". There is insufficient information to judge if this occured and if it might have affected the outcome. Participants were analysed in the groups to which they were assigned. “Intention‐to‐treat analysis, with data from the last test carried forward, was performed in the analysis of changes over time.”	Low risk of bias	24 allocated to additional therapy ‐ 3 lost to follow‐up (1 declined further participation; 2 for reasons not related to the study). 21 allocated to control ‐ none lost to immediate follow up (1 lost to persisting follow up due to illness). Small number of drop outs, with only 1 potentially related to the intervention.	Some concerns	"One limitation of the study is that the data collectors were not blinded". "The data collectors were not blinded to the group allocation but did not take part in the intervention." ‐ this indicates that it is unlikely the assessors would have influenced the assessments.	Low risk of bias	The study is registered in the Clinical Trials Database (NCT01613339). Data analysed according to protocol. Outcomes and analyses as expected according to protocol.	Some concerns	Some concerns due to potential deviations from intended interventions and lack of blinded outcome assessor; considered low risk of bias for other domains.
Mai Guanghuai 2016	Some concerns	States randomised. Lack of information about allocation concealment. No differences between groups at baseline.	Some concerns	Insufficient information to judge if there were deviations from intended interventions because of trial context. Participants were analysed according to assigned intervention.	Low risk of bias	No drop outs or missing data reported.	High risk of bias	Not clear if outcome assessor was blinded. Insufficient information to judge if outcome could have been influenced by knowledge of intervention received.	Some concerns	No protocol obtained.	High risk of bias	This is a Chinese language trial, with sections translated by one member of our team. There is generally a lack of information about the study methods. It is not clear whether the outcome assessor was blinded.
Mustafaoğlu 2018	Some concerns	"Randomization was performed by random number generator of the Microsoft Office Excel Software, which gives a random number between 0 and 1 to each treatment columns (Group distribution: 0‐<0.33=CombTG, 0.33‐<0.66=CT, 0.66‐1=BWSTT). Sorting of random number row was performed from the largest to the smallest numbers by the sort and filter menu. To ensure a distribution balance among three groups, stratification of treatment assignments was accomplished by the ambulation levels according to the Functional Ambulation Scale (FAS)." Baseline assessments were conducted after randomisation, and it is unclear how allocation was concealed. There were no statistically significant differences in demographic and clinical features at baseline.	Some concerns	Insufficient information to judge if there may have been deviations from intended interventions, for example by the treating therapists being aware of allocated intervention. Participants were analysed according to the group to which they were assigned.	Low risk of bias	45 participants randomised (to 3 groups), with no drop outs or missing data.	Low risk of bias	"primary and secondary outcome assessments at baseline and after training were performed by a blinded assessor."	Some concerns	There is a clinical trials registration (ClinicalTrials.gov ID:NCT02735148), but this appears to have been registered after completion of the trial.	Some concerns	Some concerns relating to the randomisation process and potential deviations from the intended interventions. Some concerns that the clinical trials registration may have been retrospective.
Xiao Zhen‐dong 2014	Some concerns	States "randomly divided" but description of randomisation process and allocation concealment not provided. No statistically significant difference at baseline	Some concerns	Not clear if same therapist involved in both groups, so possibly opportunities for contamination / deviations. Participants analysed in groups to which they were assigned.	Low risk of bias	60 participants randomised. No drop outs or missing data.	High risk of bias	No information relating to blinding of outcome assessor. Insufficient information to judge whether knowledge of intervention may have influenced assessments.	Some concerns	No protocol identified	High risk of bias	High risk of bias as outcome assessor may not have been blinded; insufficient information relating to other domains.
Xu 2013	Some concerns	States "randomly divided" but randomisation details not provided. There was no significant difference in age, gender composition ratio, educational level, and nature of lesions between the two groups, and they were comparable.	Some concerns	Insufficient information to judge if there were deviations due to study context.	Low risk of bias	No evidence of any dropouts or missing data.	Some concerns	Blinding of assessors not discussed.	Some concerns	No protocol available.	Some concerns	Some concerns due to lack of information and lack of protocol.
Xu Yumei 2013	Some concerns	States "randomly divided" but methods of randomisation and allocation concealment not stated. There was no significant difference in the Berg balance score between the two groups before training (P>0.05)	Some concerns	Not stated if same therapists used across control and intervention groups, meaning there could be a risk of contamination or deviation from intended intervention. Participants analysed according to assigned intervention.	Low risk of bias	40 participants randomised. No missing data or drop outs.	High risk of bias	Berg balance scale. Not stated if outcome assessor was blinded, or who performed the outcome assessments. Insufficient information to judge if outcome assessment was likely to be influenced.	Some concerns	No protocol identified.	High risk of bias	High risk of bias as not clear if outcome assessor was blinded; lack of information for other domains.
Yue Chunjiang 2014	Some concerns	States "randomly divided" but randomisation process not discussed. No statistical significant difference between the groups at baseline.	Some concerns	Possibly same therapists working across both groups, meaning that there may have been potential for contamination between groups. But insufficient information to judge. Participants were analysed according to group to which they were assigned.	Low risk of bias	90 participants randomised. No evidence of dropouts or missing data.	High risk of bias	No information relating to blinding of outcome assessors. Insufficient information to judge if outcome assessment could have been influenced by lack of blinding.	Some concerns	No protocol.	High risk of bias	High risk of bias due to potential lack of blinded outcome assessor. Some concerns due to lack of information and lack of protocol.

**Table:** Risk of bias for analysis 5.4 Gait velocity

**Study**	**Bias**											
	**Randomisation process**		**Deviations from intended interventions**		**Missing outcome data**		**Measurement of the outcome**		**Selection of the reported results**		**Overall**	
	**Authors' judgement**	**Support for judgement**	**Authors' judgement**	**Support for judgement**	**Authors' judgement**	**Support for judgement**	**Authors' judgement**	**Support for judgement**	**Authors' judgement**	**Support for judgement**	**Authors' judgement**	**Support for judgement**
**Subgroup 5.4.1 Gait speed (distance/time)**												
Cooke 2006	Low risk of bias	"An independent statistician produced a pretrial computer‐generated randomized group allocation order in blocks of 9 per trial center. Allocation was stratified by baseline scores for unilateral visual spatial neglect (Star Cancella‐tion Test, 50‐54 no spatial neglect and 0‐49 unilateral spatial neglect present). Allocation was concealed in sequentially numbered sealed opaque envelopes held by an independent administrator. Envelopes were opened in response to a tele‐phone request from the research physiotherapist (blinded to measures) after the assessor (blinded to group allocation) had completed baseline measures." No baseline differences.	Low risk of bias	"To ensure adherence to experimental treatment schedules, the research physiotherapists were supervised throughout the trial. The clinical team was given no infor‐mation about participants’ group allocation and was notpresent when the additional intervention was provided." Participants analysed according to assigned group.	Low risk of bias	109 (10%) of those screened were randomized as follows: 38 to CPT, 35 to CPT + CPT, and 36 to FST + CPT. At outcome, 10 (9%) participants had withdrawn. At follow‐up, a further 18 participants had withdrawn (26%). Dropouts were balanced between groups, suggesting that the result was not biased.	Low risk of bias	Blinded outcome assessor.	Some concerns	No published protocol, but a conference poster described the planned study. However this did not pre‐state Rivermead Mobility Index as an outcome (but not all outcomes were listed). Gait velocity was listed	Some concerns	There were some concerns as there was no study protocol, but some details were provided in a conference poster. Judged as low risk of bias for other domains.
Duncan 1998	Low risk of bias	"After baseline assessments, the subjects were randomly assigned to the experimental or control group. Randomization was done in blocks of 10. Before initiation of this study, a random list was generated by group assignments. Only a laboratory technician who had no input into subject selection or recruitment was aware of group assignment. After baseline assessment, the technician assigned thesubject to the experimental or the control group."	Low risk of bias	"The clinicians providing therapeutic interventions to the usual care group were asked to complete an intervention log to capture type of exercises and frequency and duration of therapy visits during treatment or in a home exercise program. The study coordinator met with the treating therapists at least twice to discuss the therapy logs and intervention programs". These strategies are considered to make it unlikely that there were deviations from intended interventions. Participants were analysed according to the group to which they were randomised.	Low risk of bias	"Twenty individuals with stroke were studied. Of 22 subjects recruited, 2 refused to participate." Data available for 20 participants.	Some concerns	Research assistants visited homes of the subjects to assess. Not stated if they knew whether patient was in intervention group, but possible.	Some concerns	protocol not available	Some concerns	Potential bias in measurement of outcome due to research assistant awareness of group allocation. No protocol available.
Duncan 2003	Low risk of bias	"...randomly assigned...through the use of a random number generator with a block size of 6 and sealed envelopes". No further information relating to allocation concealment. "The 2 treatment arms were comparable in all baseline measures".	High risk of bias	Participants were not blinded to their assignment but were unaware of the study hypotheses or primary outcome measures. The amount of rehabilitation provided to the usual care group is reported: "In the usual care group, 46% of the subjects did not recieve any postacute rehabilitation services from physical or occu‐pational therapy. Two thirds were provided recommendations for an unsupervised exercise program. Among the usual care group members who did receive therapy, participants re‐ ceived an average of 8.7+/‐5.3 physical therapy visits and 10.4+/‐7 occupational therapy visits. Physical and occupa‐tional therapy services were received separately as prescribed by their physicians. The total duration of the combined physical and occupational therapy visits in the usual care group was similar to the intervention group (90 minutes)." It is unclear if this deviated from usual care prior to the trial. 46% of the participants in the usual care group got comparable amounts of rehabilitation to the treatment group. It is possible that more "usual" care was given to the usual care group than the treatment group.	Low risk of bias	100 participants were randomised. "Ninety‐two subjects completed the 3‐month post treatment assessment; 8 dropped out. Six subjects dropped from theinterven assessment; 8 dropped out. Six subjects dropped from the intervention arm: 1 had significant renal insufficiency de‐tected after randomization but before therapy; 1 had sub cla‐vian steal syndrome diagnosed the first 2 weeks after ran‐domization; 1 chose to withdraw after 18 visits; and 3 experienced a second stroke. Two subjects dropped from the usual care group: 1 withdrew from the study immediately after randomization, and 1 did not return for the 3‐month assessment." Drop outs are accounted for and balanced between groups (small number).	Low risk of bias	Outcome assessment was performed by research staff blinded to treatment assignment.	Some concerns	no study protocol	High risk of bias	High risk of bias due to concerns about potential deviations from intended deviations. Some concerns as no study protocol., but judged as low risk of bias for other domains.
Harjpal 2021	Some concerns	"Computer generated randomization" using "Sequentially numbered, sealed, opaque envelopes". "Randomisation and allocation was done by the primary researcher" ‐ unclear if the allocation sequence remained concealed or whether the primary researcher could have introduced bias. No signficant baselinie differences.	Some concerns	The treatment group received 20 minutes additional physiotherapy/day, 5 days/week. Insufficient information to judge whether there may have been deviations from the intended intervention, such as contamination between groups. Participants were analysed according to the assigned intervention group.	Low risk of bias	40 participants randomised. No drop outs or missing data.	Low risk of bias	Blinded outcome assessor.	Low risk of bias	Prospective trial registration: CTRI/2021/05/033621 Outcomes and analyses as described in protocol.	Some concerns	Some concerns due to lack of information regarding allocation concealment and potential for contamination between groups. Low risk of bias for other domains.
Kim 2012a	Some concerns	time after onset of stroke was 5.7 months for the RAS group and 4.8 months for the control group, with no significant difference between the groups." There were no other baseline differenes, other than age. Given the small study size this is judged not to suggest a problem with the randomisation process.	Some concerns	Insufficient information to judge if there could have been deviations from the intended intervention. Participants were analysed according to their assigned group.	Low risk of bias	"Except for one patient in each group who left the hospital halfway through the study, 18 subjects in all took part in the experiment to the end, and assessments were performed both before and after conduct of the experiment." Only 2 dropouts, and balanced between groups.	High risk of bias	Gait velocity. No information relating to who conducted assessments and whether they were blinded. Insufficient information to judge whether assessment could have been influenced by knowledge of the intervention.	Some concerns	No protocol identified.	High risk of bias	High risk of bias due to lack of information relating to blinding of outcome assessor; some concerns for other domains due to insufficient information and lack of protocol.
Kim 2021	Low risk of bias	Block randomization was determined using a randomization procedure in which each participant drew a ball from a box. Participants provided informed consent prior to participating were included in this study. No baseline differences.	Some concerns	Insufficient information to judge if there may have been deviations from the intended interventions. Participants were analysed according to their assigned intervention.	Low risk of bias	"During the 8‐week experimental period, two, three, and three participants dropped out from Experimental I (n = 13), Experimental II (n = 12), and the control group (n = 12), respectively, for a final total of 37 study participants." Drop outs were balanced between groups and reasons were not related to the study intervention.	Low risk of bias	"Evaluators blinded to group allocation performed the evaluations"	Low risk of bias	The trial was registered under trial registration no. KCT0006579. Reported study was as expected from protocol. Outcomes and analyses were pre‐stated in protocol.	Some concerns	Some concerns due to insufficient information to judge if there could be deviations from intended interventions; judged as low risk of bias for other domains.
LAST 2018	Low risk of bias	"Participants randomly assigned (1:1) in blocks of 2 and 4, to an intervention group receiving individualized coaching on physical activitiy or to a control group receiving standard care. Randomization was performed by a web‐based randomization system." "The randomisation procedure will be computerised and organised by Unit for Applied Clinical Research at Norwegian University of Science and Technology (NTNU)." Demographic and baseline characteristics were similar in both groups.	Low risk of bias	Intervention was delivered as outpatient, limiting opportunities for contamination between groups. Participants were analysed according to assigned intervention.	High risk of bias	Intervention group 42/184 discontinued allocated intervention; control group 9/194 discontinued allocated intervention. Intervention group 153/186 assessed, control group 162/194 assessed. Substantially more discontinued from intervention group than control group. Increased drop outs in intervention group are likely to be related to acceptability of the intervention.	Low risk of bias	"A group of well‐trained research assistants, blinded to the treatment allocation, screened patients for eligibility and did all assessments face‐to‐face at inclusion and at 18 month follow up."	Low risk of bias	Published study protocol and trials registry. Data presented are in accordance with pre‐specified protocol.	High risk of bias	Concerns due to greater number of drop outs in intervention than control group, which could be related to the intervention. Considered low risk of bias for other domains.
Park 2021	Low risk of bias	The subjects were divided into three groups: CG, DCG, and ICG, with 20 beads of different colors, each of which was the same weight and size, and a total of 60 beads of different colors, placed in a blind pocket and made the subjects pull them out. A one‐way ANOVA confirmed homogeneity of the participants’ general characteristics between the groups.	Some concerns	States "double blind" but no further details provided. Not clear if therapists blinded, potentially allowing contamination between groups or deviations from intended interventions. Participants analysed according to assigned intervention.	Low risk of bias	60 participants randomised to 3 groups. 8 drop‐outs (2/20, 5/20 and 1/20). Drop outs were reasonably balanced between groups and reasons for dropping out were provided.	Low risk of bias	States "double blind"	Some concerns	No protocol identified.	Some concerns	Some concerns due to lack of information about intervention delivery and lack of protocol.
Park 2021	Low risk of bias	The subjects were divided into three groups: CG, DCG, and ICG, with 20 beads of different colors, each of which was the same weight and size, and a total of 60 beads of different colors, placed in a blind pocket and made the subjects pull them out. A one‐way ANOVA confirmed homogeneity of the participants’ general characteristics between the groups.	Some concerns	States "double blind" but no further details provided. Not clear if therapists blinded, potentially allowing contamination between groups or deviations from intended interventions. Participants analysed according to assigned intervention.	Low risk of bias	60 participants randomised to 3 groups. 8 drop‐outs (2/20, 5/20 and 1/20). Drop outs were reasonably balanced between groups and reasons for dropping out were provided.	Low risk of bias	States "double blind"	Some concerns	No protocol identified.	Some concerns	Some concerns due to lack of information about intervention delivery and lack of protocol.
Song 2015	Some concerns	States "randomly assigned" ‐ no further details provided. No baseline differences.	Some concerns	Not clear if same physios involved in all groups, potentially providing opportunities for contamination. Participants analysed according to assigned intervention.	Low risk of bias	30 participant randomised (to 3 groups). No drop outs or missing data reported.	High risk of bias	Not stated who conducted outcome assessment, and whether they were blinded.	Some concerns	No protocol identified.	High risk of bias	High risk of bias due to concerns about lack of blinding of outcome assessor; some concerns due to lack of information about methods and no protocol.
Zhu 2016	Some concerns	States random, but no details as to how randomisation was performed. "There was no significant difference in any of the variables between the m‐CIMT group and control group at baseline"	Some concerns	The treatment group received approximately 2 hours/day additional therapy. Insufficient information to judge whether there could have been any contamination between groups, or deviations from intended interventions. Participants were analysed according to assigned intervention.	Low risk of bias	22 participants randomised. No drop outs or missing data reported.	Low risk of bias	"To maintain the confidentiality, gait analysis was performed blindly by the therapists and lab workers"	Some concerns	No protocol identified.	Some concerns	Some concerns due to lack of information relating to randomisation and intervention delivery, and lack of protocol.
**Subgroup 5.4.2 Timed walk (time to walk set distance)**												
Aries 2021	Low risk of bias	"Randomization of participants to one of two groups (MTS+TSGT or TIs+TSGT) was undertaken by the Norwich Clinical Trials Unit, using a computer randomization system in a 1:1 ratio with permuted blocks of two and four. Stratification by left‐ or right‐sided brain lesion (identified from the medical notes) was used because this factor may influence rehabilitation potential (36). Group allocation order was concealed from the research team until after measurement at baseline." "No differences were discernible between the MTS+TSGT and TI+TSGT groups at baseline"	Low risk of bias	"Research therapists (n = 4) received training in applying and delivering all interventions. To assess fidelity to protocol, the research therapists were observed by a senior member of the project team (SMH) at various points during the study to ensure they were working to protocol." Participants were analysed according to assigned intervention.	Low risk of bias	2 discontinued TI+TSGT intervention due to adverse events. 32/34 included in analysis. Low number of drop outs.	Low risk of bias	"In addition, participants were asked not to disclose group allocation to the blinded assessor and the case report forms were not accessible to the blinded assessor. The blinded assessor guessed accurately the group allocation of just three of the 32 participants completing the study: 9.38% (95% CI 3.2%, 24.2%)"	Low risk of bias	The study was registered on a clinical trials database (ISRCTN 13676183; Central Portfolio Management System ID 30449). Data were analysed in line with this study protocol.	Low risk of bias	Study judged to be low risk of bias for all domains.
Dalal 2018	Low risk of bias	Subjects were assigned to either group by block randomization, with concealed allocation. No statistically significant difference across the groups.	Some concerns	Possibly same physios delivering intervention and usual care; insufficient information to judge if this could have resulted in deviations from the intended intervention. Participants analysed according to assigned intervention.	Low risk of bias	32 participants randomised; 3 drop outs from intervention (reasons provided and balanced between groups) but outcome measurement completed for all 32.	Low risk of bias	Blinded assessors	Some concerns	Trial registration: CTRI/2016/06/00705, however this was retrospective. Retrospective protocol registration makes it impossible to judge.	Some concerns	Some concerns due to potential deviations from intended interventions and retrospective protocol registration.
Frimpong 2014	Some concerns	States randomised but no further details as to process given. "All subjects were drawn from the same population of stroke survivors and no statistically significant differences (in 10MWT, 6MWT and FAC) were found among the two groups at baseline."	Some concerns	No information on whether same therapists delivered across both groups, making it impossible to judge if there could have been deviations or contamination between groups. Participants analysed according to assigned intervention.	Low risk of bias	20 participants randomised. No drop outs or missing data reported.	High risk of bias	Does not state who carried out outcome assessments and whether there was blinding.	Some concerns	No protocol identified.	High risk of bias	High risk of bias due to possible lack of blinded outcome; some concerns due to lack of information on methods and lack of protocol.
Kim 2012	Some concerns	States "randomly assigned", but no further details provided. Limited baseline data provided.	Some concerns	Insufficient information to judge whether there could have been deviations. Studied interventions create potential for contamination between groups. Participants were analysed according to the assigned intervention.	Low risk of bias	20 participants randomised. No drop outs or missing data.	High risk of bias	Does not state who conducted outcome assessments and whether they were blinded. Insufficient information to judge whether outcome assessment could have been influenced by knowledge of intervention.	Some concerns	No protocol identified.	High risk of bias	High risk of bias due to potential bias in measurement of the outcome, with no blinded assessor; some concerns for other domains due to lack of information.
Kunkel 2013	Some concerns	"Those willing to participate gave informed consent the following day; demographic data were col‐lected and FES tolerance tested, prior to randomization. The trial therapist then contacted the Medical Statistics Group by telephone to obtain a concealed, computer generated random allocation to one of the three groups:" Differences in baseline data for Rivermead Mobility index (treatment group 5.3(2.6); control group 7.7(3.2)), Berg balance (treatment group 27.5(10.6); control group 40.7(8.4)).	High risk of bias	"The usual care control group was included to enable comparison to standard care. While of theoretical importance, this design proved problematic. While the assessor was successfully blinded, NHS therapists were aware of the group allocation and it would be impossible to blind them in a future trial. In the event, non‐trial therapists altered the focus of their attention delivering substantially greater amounts of routine therapy to the control arm potentially masking treatment effects compared with control. " Participants were analysed according to the group to which they were assigned.	High risk of bias	21 participants recruited and 7 randomised to each of 3 arms. Exercise group had data from 4/7 and usual care group data from 5/7. Reason for drop outs did not appear to be related to the trial intervenion. Drop outs from exercise group were due to urinary tract infection, medically well, and withdrawal (no stated reason). Drop out from usual care group was due to second stroke. However data were only available for this outcome (gait velocity) for 1/7 and 4/7 respectively. Reason for missing data not stated, but is likely to be related to ability to walk.	Low risk of bias	"assessor was successfully blinded"	Some concerns	No protocol identified.	High risk of bias	High risk of bias due to reported deviations from intended interventions; some concerns relating to randomisation process and lack of protocol.
Mustafaoğlu 2018	Some concerns	"Randomization was performed by random number generator of the Microsoft Office Excel Software, which gives a random number between 0 and 1 to each treatment columns (Group distribution: 0‐<0.33=CombTG, 0.33‐<0.66=CT, 0.66‐1=BWSTT). Sorting of random number row was performed from the largest to the smallest numbers by the sort and filter menu. To ensure a distribution balance among three groups, stratification of treatment assignments was accomplished by the ambulation levels according to the Functional Ambulation Scale (FAS)." Baseline assessments were conducted after randomisation, and it is unclear how allocation was concealed. There were no statistically significant differences in demographic and clinical features at baseline.	Some concerns	Insufficient information to judge if there may have been deviations from intended interventions, for example by the treating therapists being aware of allocated intervention. Participants were analysed according to the group to which they were assigned.	Low risk of bias	45 participants randomised (to 3 groups), with no drop outs or missing data.	Low risk of bias	"primary and secondary outcome assessments at baseline and after training were performed by a blinded assessor."	Some concerns	There is a clinical trials registration (ClinicalTrials.gov ID:NCT02735148), but this appears to have been registered after completion of the trial.	Some concerns	Some concerns relating to the randomisation process and potential deviations from the intended interventions. Some concerns that the clinical trials registration may have been retrospective.
**Subgroup 5.4.3 Timed up and go test**												
Jandaghi 2021	Low risk of bias	"Following consent, patients were randomized using a computer generated block randomizer to 1 of 3 groups: The visual deprivation‐ stable based training (VD‐SBT); unstable based training (UBT); and control (C) groups. Sealed envelopes opaque were used for allocation concealment" Protocol states: "The random sequence will be performed using the simple randomization. The random number table is used to perform the simple randomization. Allocation concealment is performed using Sequentially numbered, sealed, opaque envelopes To execute the random allocation process, the person involved in randomization is separated from the investigator to reduce possible bias" No baseline differences between groups.	Some concerns	Insufficient information to judge if there were deviations from intended interventions. Participants were analysed according to assigned intervention.	Low risk of bias	45 participants randomised. No drop outs or missing data reported.	High risk of bias	Not stated who performed the outcome assessments and whether they were blinded. Protocol states investigator "not blinded".	Low risk of bias	The study protocol was registered: IRCT20190812044516N1 Reported results are in line with study protocol.	High risk of bias	High risk of bias due to lack of blinded outcome assessor; some concerns about potential for deviations from intended interventions.
Lindvall 2014	Low risk of bias	"Randomization to experimental or control intervention was conducted in blocks of four to six patients at each primary healthcare centre, in the form of sealed envelopes that were opened after the baseline tests" No baseline differences.	Some concerns	The intervention was delivered in addition to usual care and occured in a group setting, delivered in a location away from control group participants. However the authors state "The physical activity levels were not measured in either group and some of the participants allocated to the control group expressed disappointment at not receiving the training in the intervention and may have started exercising on their own". There is insufficient information to judge if this occured and if it might have affected the outcome. Participants were analysed in the groups to which they were assigned. “Intention‐to‐treat analysis, with data from the last test carried forward, was performed in the analysis of changes over time.”	Some concerns	24 allocated to additional therapy ‐ 3 lost to follow‐up (1 declined further participation; 2 for reasons not related to the study). 21 allocated to control ‐ none lost to immediate follow up (1 lost to persisting follow up due to illness). Small number of drop outs, with only 1 potentially related to the intervention. However for this outcome baseline results carried forward for 6 missing participants (3 in each group). Carrying forward of baseline data could impact on the results, particularly if this was due to inability to walk. However numbers were balanced between groups suggesting that the result was not biased.	Some concerns	"One limitation of the study is that the data collectors were not blinded". "The data collectors were not blinded to the group allocation but did not take part in the intervention." ‐ this indicates that it is unlikely the assessors would have influenced the assessments.	Low risk of bias	The study is registered in the Clinical Trials Database (NCT01613339). Data analysed according to protocol. Outcomes and analyses as expected according to protocol.	Some concerns	Some concerns due to potential deviations from intended interventions, missing outcome data and lack of blinded outcome assessor; considered low risk of bias for other domains.
**Subgroup 5.4.4 Other measure relating to gait speed**												
Kim 2007	Some concerns	States "randomised", but no description of randomisation or allocation process.	Some concerns	No information about whether same therapists involved in control/intervention, creating some concerns about potential for contamination between groups. Participants were analysed according to the group to which they were randomised.	Some concerns	3 drop‐outs from control group "due to emergency discharge", none from treatment group. Not clear whether the "emergency discharge" was related to the trial context. Possible that the discharged patients were those with better outcomes.	Some concerns	Not clear if outcome assessor was blinded. No evidence that knowledge of intervention influenced outcome.	Some concerns	No protocol available.	Some concerns	Some concerns, primarily due to lack of information.

**Table:** Risk of bias for analysis 5.5 Adverse events

**Study**	**Bias**											
	**Randomisation process**		**Deviations from intended interventions**		**Missing outcome data**		**Measurement of the outcome**		**Selection of the reported results**		**Overall**	
	**Authors' judgement**	**Support for judgement**	**Authors' judgement**	**Support for judgement**	**Authors' judgement**	**Support for judgement**	**Authors' judgement**	**Support for judgement**	**Authors' judgement**	**Support for judgement**	**Authors' judgement**	**Support for judgement**
Aries 2021	Low risk of bias	"Randomization of participants to one of two groups (MTS+TSGT or TIs+TSGT) was undertaken by the Norwich Clinical Trials Unit, using a computer randomization system in a 1:1 ratio with permuted blocks of two and four. Stratification by left‐ or right‐sided brain lesion (identified from the medical notes) was used because this factor may influence rehabilitation potential (36). Group allocation order was concealed from the research team until after measurement at baseline." "No differences were discernible between the MTS+TSGT and TI+TSGT groups at baseline"	Low risk of bias	"Research therapists (n = 4) received training in applying and delivering all interventions. To assess fidelity to protocol, the research therapists were observed by a senior member of the project team (SMH) at various points during the study to ensure they were working to protocol." Participants were analysed according to assigned intervention.	Low risk of bias	2 discontinued TI+TSGT intervention due to adverse events. 32/34 included in analysis. Low number of drop outs.	Low risk of bias	Adverse events recorded and reported clearly.	Low risk of bias	The study was registered on a clinical trials database (ISRCTN 13676183; Central Portfolio Management System ID 30449). Data were analysed in line with this study protocol.	Low risk of bias	Study judged to be low risk of bias for all domains.
Duncan 2003	Low risk of bias	"...randomly assigned...through the use of a random number generator with a block size of 6 and sealed envelopes". No further information relating to allocation concealment. "The 2 treatment arms were comparable in all baseline measures".	High risk of bias	Participants were not blinded to their assignment but were unaware of the study hypotheses or primary outcome measures. The amount of rehabilitation provided to the usual care group is reported: "In the usual care group, 46% of the subjects did not recieve any postacute rehabilitation services from physical or occu‐pational therapy. Two thirds were provided recommendations for an unsupervised exercise program. Among the usual care group members who did receive therapy, participants re‐ ceived an average of 8.7+/‐5.3 physical therapy visits and 10.4+/‐7 occupational therapy visits. Physical and occupa‐tional therapy services were received separately as prescribed by their physicians. The total duration of the combined physical and occupational therapy visits in the usual care group was similar to the intervention group (90 minutes)." It is unclear if this deviated from usual care prior to the trial. 46% of the participants in the usual care group got comparable amounts of rehabilitation to the treatment group. It is possible that more "usual" care was given to the usual care group than the treatment group.	Low risk of bias	100 participants were randomised. "Ninety‐two subjects completed the 3‐month post treatment assessment; 8 dropped out. Six subjects dropped from theinterven assessment; 8 dropped out. Six subjects dropped from the intervention arm: 1 had significant renal insufficiency de‐tected after randomization but before therapy; 1 had sub cla‐vian steal syndrome diagnosed the first 2 weeks after ran‐domization; 1 chose to withdraw after 18 visits; and 3 experienced a second stroke. Two subjects dropped from the usual care group: 1 withdrew from the study immediately after randomization, and 1 did not return for the 3‐month assessment." Drop outs are accounted for and balanced between groups (small number).	Low risk of bias	"This study had a Safety Monitoring Board that reviewed, approved and monitored the adverse event monitoring and reporting process throughout the study. All subjects were contacted every 2 weeks to discuss adverse events, including healthcare use, medical events and symptoms".	Some concerns	no study protocol	High risk of bias	High risk of bias due to concerns about potential deviations from intended deviations. Some concerns as no study protocol., but judged as low risk of bias for other domains.
LAST 2018	Low risk of bias	"Participants randomly assigned (1:1) in blocks of 2 and 4, to an intervention group receiving individualized coaching on physical activitiy or to a control group receiving standard care. Randomization was performed by a web‐based randomization system." "The randomisation procedure will be computerised and organised by Unit for Applied Clinical Research at Norwegian University of Science and Technology (NTNU)." Demographic and baseline characteristics were similar in both groups.	Low risk of bias	Intervention was delivered as outpatient, limiting opportunities for contamination between groups.	High risk of bias	Intervention group 42/184 discontinued allocated intervention; control group 9/194 discontinued allocated intervention. Intervention group 153/186 assessed, control group 162/194 assessed. Substantially more discontinued from intervention group than control group. Increased drop outs in intervention group are likely to be related to acceptability of the intervention.	Low risk of bias	"Adverse Events. Information about new cardiovascular and cerebrovascular events,serious falls, fractures, or any event of syncope or dizziness withunknown reason, resulting in hospitalization, was collected from theNorwegian Patient Registry. Information about deaths was collectedfrom the hospital records or next‐of‐kin"	Low risk of bias	Published study protocol and trials registry. Data presented are in accordance with pre‐specified protocol.	High risk of bias	Concerns due to greater number of drop outs in intervention than control group, which could be related to the intervention. Considered low risk of bias for other domains.
Zhuang 2012	Low risk of bias	"The team’s data management center generated the randomization numbers with SAS9.1.3 (Statistical Analysis System provided by SAS Institute Inc,Cary, North Carolina). Each of the seven sites had a designated research assistant who was responsible for obtaining a random number for each participant from a web‐based, password‐protected Internet site and who actually assigned the participant to one of the three treatment groups" "At baseline, no significant differences existed between the three groups in terms of gender, age, or length or severity of disease (P >.05)"	Low risk of bias	‘research assistant instructed participants not to discuss other treatments that they were receiving with their therapists’	Low risk of bias	310 participants were screened, of those: "274 completed the study, 15 did not meet the inclusion/exclusion criteria, and 21 dropped out. Adverse events were rare (less than 1%), mild, and temporary" (i.e. 21 drop outs from 295 possible completers). ‘Of those who dropped out, nine left the hospital, eight discontinued treatment, two dropped out due to poor health, one (from the acupuncture group) suffered a second stoke and one (from the physiotherapy group) died due to a respiratory tract infection’. Non‐completers appear balanced across groups.	Some concerns	"The research team recorded adverse reactions occurring duringthe trial and reported them to the principal investigator. " Some concerns as definition of adverse reaction is not provided and is not clear.	Some concerns	No protocol available.	Some concerns	No protocol available so potential bias in selection of the reported result, and adverse events not clearly defined, but judged as low risk of bias for other domains.

**Table:** Risk of bias for analysis 6.2 Motor function scales

**Study**	**Bias**											
	**Randomisation process**		**Deviations from intended interventions**		**Missing outcome data**		**Measurement of the outcome**		**Selection of the reported results**		**Overall**	
	**Authors' judgement**	**Support for judgement**	**Authors' judgement**	**Support for judgement**	**Authors' judgement**	**Support for judgement**	**Authors' judgement**	**Support for judgement**	**Authors' judgement**	**Support for judgement**	**Authors' judgement**	**Support for judgement**
**Subgroup 6.2.1 Other motor function measure**												
Aries 2021	Low risk of bias	"Randomization of participants to one of two groups (MTS+TSGT or TIs+TSGT) was undertaken by the Norwich Clinical Trials Unit, using a computer randomization system in a 1:1 ratio with permuted blocks of two and four. Stratification by left‐ or right‐sided brain lesion (identified from the medical notes) was used because this factor may influence rehabilitation potential (36). Group allocation order was concealed from the research team until after measurement at baseline." "No differences were discernible between the MTS+TSGT and TI+TSGT groups at baseline"	Low risk of bias	"Research therapists (n = 4) received training in applying and delivering all interventions. To assess fidelity to protocol, the research therapists were observed by a senior member of the project team (SMH) at various points during the study to ensure they were working to protocol."	Low risk of bias	2 discontinued TI+TSGT intervention due to adverse events. 32/34 included in analysis.	Low risk of bias	"In addition, participants were asked not to disclose group allocation to the blinded assessor and the case report forms were not accessible to the blinded assessor. The blinded assessor guessed accurately the group allocation of just three of the 32 participants completing the study: 9.38% (95% CI 3.2%, 24.2%)"	Low risk of bias	The study was registered on a clinical trials database (ISRCTN 13676183; Central Portfolio Management System ID 30449). Data were analysed in line with this study protocol.	Low risk of bias	Study judged to be low risk of bias for all domains.
Cooke 2006	Low risk of bias	"An independent statistician produced a pretrial computer‐generated randomized group allocation order in blocks of 9 per trial center. Allocation was stratified by baseline scores for unilateral visual spatial neglect (Star Cancella‐tion Test, 50‐54 no spatial neglect and 0‐49 unilateral spatial neglect present). Allocation was concealed in sequentially numbered sealed opaque envelopes held by an independent administrator. Envelopes were opened in response to a tele‐phone request from the research physiotherapist (blinded to measures) after the assessor (blinded to group allocation) had completed baseline measures." No baseline differences.	Low risk of bias	"To ensure adherence to experimental treatment schedules, the research physiotherapists were supervisedthroughout the trial. The clinical team was given no infor‐mation about participants’ group allocation and was notpresent when the additional intervention was provided." Participants analysed according to assigned group.	Some concerns	"109 (10%) of those screened were randomized as follows: 38 to CPT, 35 to CPT + CPT, and 36 to FST + CPT. At outcome, 10 (9%) participants had withdrawn. At follow‐up, a further 18 participants had withdrawn (26%)." Dropouts were balanced between groups, suggesting that the result was not biased.	Low risk of bias	Blinded outcome assessor	Some concerns	No published protocol, but a conference poster described the planned study. However this did not pre‐state Rivermead Mobility Index as an outcome (but not all outcomes were listed).	Some concerns	There were some concerns as there was no study protocol, but some details were provided in a conference poster. Judged as low risk of bias for other domains.
Kunkel 2013	Some concerns	"Those willing to participate gave informed consent the following day; demographic data were col‐lected and FES tolerance tested, prior to randomization. The trial therapist then contacted the Medical Statistics Group by telephone to obtain a concealed, computer generated random allocation to one of the three groups:" Differences in baseline data for Rivermead Mobility index (treatment group 5.3(2.6); control group 7.7(3.2)), Berg balance (treatment group 27.5(10.6); control group 40.7(8.4)).	High risk of bias	"The usual care control group was included to enable comparison to standard care. While of theoretical importance, this design proved problematic. While the assessor was successfully blinded, NHS therapists were aware of the group allocation and it would be impossible to blind them in a future trial. In the event, non‐trial therapists altered the focus of their attention delivering substantially greater amounts of routine therapy to the control arm potentially masking treatment effects compared with control. " Participants were analysed according to the group to which they were assigned.	Low risk of bias	21 participants recruited and 7 randomised to each of 3 arms. Exercise group had data from 4/7 and usual care group data from 5/7. Reason for drop outs did not appear to be related to the trial intervenion. Drop outs from exercise group were due to urinary tract infection, medically well, and withdrawal (no stated reason). Drop out from usual care group was due to second stroke.	Low risk of bias	"assessor was successfully blinded"	Some concerns	No protocol identified.	High risk of bias	High risk of bias due to evidence of deviations from intended interventions. Differences at baseline for this outcome. Low risk of bias for other domains, allthough no study protocol identified.

**Table:** Risk of bias for analysis 7.1 Functional task training compared to other: Independence in ADL scales

**Study**	**Bias**											
	**Randomisation process**		**Deviations from intended interventions**		**Missing outcome data**		**Measurement of the outcome**		**Selection of the reported results**		**Overall**	
	**Authors' judgement**	**Support for judgement**	**Authors' judgement**	**Support for judgement**	**Authors' judgement**	**Support for judgement**	**Authors' judgement**	**Support for judgement**	**Authors' judgement**	**Support for judgement**	**Authors' judgement**	**Support for judgement**
**Subgroup 7.1.1 Functional task training compared to 'less' functional task training**												
Chen G 2014	Some concerns	States "randomly divided" but no further information. There were no significant differences in age, gender, paralyzed side and NIHSS score on admission between the two groups (P>0.05), which were comparable.	Some concerns	Insufficient information to judge if deviations arose because of trial context.	Low risk of bias	No evidence of drop outs or missing data.	Some concerns	No discussion of blinding.	Some concerns	No protocol available.	Some concerns	Some concerns due to lack of information and lack of protocol.
Chen P 2014	Some concerns	States randomisation software used, but no further information.	Some concerns	Insufficient information to judge if there were deviations from intended interventions.	Low risk of bias	100 participants recruited and randomised into two groups of 50. Reports results data for 100 participants.	Some concerns	No information relating to blinding of outcome assessor.	Some concerns	No protocol identified.	Some concerns	Some concerns due to lack of information and lack of protocol.
Choi JU 2015	Some concerns	States "randomly and equally assigned" but no decription as to how randomisation was performed.	High risk of bias	Possible that the therapist encouraged and praised the treatment group more than the control group: "it is likely that by encouraging and praising the subjects as they progressed to high‐level tasks, the physical therapist helped to improve self‐efficacy." More encouragement in the treatment group, possibly due to therapist providing the intervention having a desire to demonstrate effectiveness.	Low risk of bias	No mention of drop‐outs.	Some concerns	No information relating to blinding of outcome assessor. No evidence to suggest that assessment was influenced by knowledge of intervention.	Some concerns	No protocol available.	High risk of bias	No decription as to how randomisation was performed. Possible deviations as it is possible that the therapist encouraged and praised the treatment group more than the control group: "it is likely that by encouraging and praising the subjects as they progressed to high‐level tasks, the physical therapist helped to improve self‐efficacy." No information relating to blinding of outcome assessor and no protocol available.
Ding 2015	Some concerns	States "randomly divided" but no details around randomisation process. There was no significant difference in general data such as gender and age between the two groups (P>0.05), and they were comparable.	Some concerns	Insufficient information to judge if deviations may have occured because of the trial context.	Low risk of bias	No evidence of any drop outs or missing data.	Some concerns	No information on blinding provided.	Some concerns	No protocol available.	Some concerns	Some concerns due to lack of information and lack of protocol.
Langhammer 2007	High risk of bias	"Randomization was performed with a die: patients with uneven numbers went to group 1, an intensive exercise group, and those with even numbers to group 2, a regular exercise group. Stratification was according to gender and hemisphere lesion: the first male patient with a right hemisphere lesion and with an uneven number was allocated to the intensive exercise group, and the next male patient with a right hemisphere lesion was allocated to the regular exercise group. The procedure with the die was then used when the third male patient with a right hemisphere lesion entered the stroke unit and so on. A corresponding procedure was followed for female patients". "At discharge from the acute hospital, patients were randomized to one of two different groups by a person not involved with the patients or the treatment in the ward" "No significant differences between the groups regarding age, hemisphere lesion, marital status at baseline, or admission to the stroke unit". However, the regular exercise group had slightly higher baseline values for activities of daily living, motor function, balance and gait velocity, suggesting that the groups were not balanced.	High risk of bias	One fact that might be considered a weakness was that some therapists administered a submaximal programme to patients whom they had volunteered to exercise maximally. The reason for this was explained by the therapists involved as being a practical adaptation to pathological conditions such as heart failure, pain and a poor cognitive status, which inhibited a maximal effort. In order to carry out the exercises, adjustments were made so that routines could be maintained through the study period. This is probably also one of the reasons why so many patients complied with the exercise programmes.	Low risk of bias	8/75 dropped out; outcome data available for remaining participants.	Low risk of bias	"The study was an intention‐to‐treat trial with the aim of being double‐blind, that is, neither the investigator nor the participants knew to which group participants were allocated"	Some concerns	A protocol is referred to by the study authors, but this was not available.	High risk of bias	There was potential for bias due to deviations from the intended interventions, and some concerns over the randomisation process (which used a die) and no protocol available.
Lawal 2016	Low risk of bias	"Randomisation will be conducted using a computer‐generated random allocation sequence schedule held by a third party, who will randomly allocate recruited participants into the study group"	Low risk of bias	"All the activities for the control group were conducted by regular therapists (who are similar in qualification/experience to therapists implementing the CCT programme) in the Physiotherapy Department of AKTH." ‐ implies different therapists for the different groups, thus minimising potential for deviations.	Low risk of bias	"All participants completed the eight weeks therapy, with percentage range of drop‐out starting from a minimum of 7% in group D (control) to a maximum of 9% in groups A and B". Reasons for drop outs provided.	Low risk of bias	"To eliminate bias, the assessment of outcome will be performed by (experienced/trained) blinded assessors, who will be blinded to the nature/type of intervention as well as the intervention groups of the participants. Participants will also be instructed not to disclose their individual intervention groups to the assessors."	Low risk of bias	Published study protocol and trial registration. Final result presented as pre‐specified in protocol.	Low risk of bias	No concerns.
Li 2013	Some concerns	States "divided.....according to the random number table". No further information provided. Groups were clinically comparable at baseline.	Some concerns	Insufficient information to judge if there were potentially deviations due to trial context.	Low risk of bias	No evidence of any drop outs or missing data.	Some concerns	No discussion on blinding.	Some concerns	No protocol obtained.	Some concerns	Some concerns due to lack of information and lack of protocol.
Li Weiwei 2015	Some concerns	States "randomly divided" but no further information about randomisation process. No signifcant difference in gender and age between the groups.	Some concerns	Insufficient information to judge if there were deviations due to trial context.	Low risk of bias	No evidence of drop outs or missing data.	Some concerns	Blinding not discussed	Some concerns	No reference to ethics or protocol.	Some concerns	Some concerns due to lack of information and lack of protocol.
Meng 2022	Low risk of bias	"Patients who met the criteria were randomly allocated to the RAGT, ELLT, and CRT groups using the NCSS‐PASS program‐generated randomization table, at an allocation ratio of 1:1:1. A principal investigator generated random assignment sequences for participants in the NCSS‐PASS, and the random assignments were concealed in consecutively numbered sealed opaque enve‐ lopes, which were sequentially opened after each patient provided written informed consent" "There were no significant differences in age, sex, side and type of stroke, duration, and clinical measures (6MWT, FAC, TUG, DTW, Tinetti, BI, SS‐QOL, and gait parameters) at baseline among the three groups"	Some concerns	"Owing to the random and single‐blinded study design, only the evaluator and statistician were blinded to the grouping procedures" Insufficient information to judge if there may have been deviations from intended intervention.	Low risk of bias	Assume that data in unlabelled row in Table 2 is Barthel Index.	Low risk of bias	Evaluator was blinded.	High risk of bias	Protocol was registered ("registered in the Chinese Clinical Trial Registry (no. ChiCTR1900026225)"). However this states that this was a "Non‐randomized controlled study, no random methods involved", and only states two treatment groups (3 are reported). Outcomes in protocol are stated as NIHSS, Fugl Meyer and Barthel Index; outcomes in results paper are different, and do not include Fugl Meyer.	High risk of bias	Study described in results paper judged as low risk of bias for most domain, but published protocol differs from reported study in terms of study design and outcomes.
Meng Qingling 2015	Some concerns	States "According to the random number table method, they were divided" but no further information. No significant difference in general data between the two groups (P>0.05).	Some concerns	Insufficient information to judge if there were deviations from intended interventions.	Low risk of bias	No evidence of dropouts or missing data.	Some concerns	Blinding not discussed.	Some concerns	No protocol available.	Some concerns	Some concerns due to lack of information and lack of protocol.
Pandian 2014	Low risk of bias	"randomly assigned either the exper‐ imental group (n=17) or the control group (n=18), using computer‐generated random numbers. The blocks were numbered and a random‐number generator program was used to select the number. The established sequence of the blocks was allocated to either one or the other group (allocation ratio 1:1). The intervention assignments were enclosed in sealed, opaque and sequentially numbered envelopes. A research assistant at the study site (not the part of study) conducted the random‐number program and concealed the allocation till the final enrollment of participants"	Some concerns	"The experimental and control interventions were given by the two independent therapists": study flow diagram implies that each delivered a different intervention. However there may have been potential for contamination between groups.	Low risk of bias	"All the subjects in the experimental (n=17) and control (n=18) groups completed the treatment protocol of 2 months". 1 loss to follow up from treatment group; 2 from control group. Data from all participants anlaysed with last observation carried over.	Low risk of bias	"Pre‐ and post assessments, using the outcome measures were carried out by one of the investigators was not involved in the interventions and had no awareness of which intervention the subjects received"	Some concerns	No protocol obtained.	Some concerns	Insufficient information to judge if there could have been deviations from the intended interventions, and no protocol obtained.
Rahayu 2020	Some concerns	"randomly allocated....using coin‐tossing technique". No information relating to allocation concealment. No significant difference was found between the two groups in terms of participants’ characteristics However, the baseline functional performance of participants between the two groups showed a statistically significant difference (p = 0.045).	Low risk of bias	The intervention group received physiotherapy from the researchers while the control group received standard physiotherapy intervention from the institutions’ physiotherapists.	Low risk of bias	Data available for nearly all ‐ 69 participants randomised; 64 completed intervention: "Two participants refused to particpate during the randomisation phase, two were lost during the follow up in the intervention group, and one needed to withdraw from the study due to worsening of the condition in the control".	Low risk of bias	"The research assistants were only responsible for data collection purpose and were blinded to the groups."	Some concerns	No protocol obtained.	Some concerns	Some concerns relating to method of randomisation and allocation concealment, and lack of protocol.
Zhang Huiyu 2021	Some concerns	States participants were divided using the "random number table method". No further information provided. There was no significant difference in general data between the two groups (P>0.05), which was comparable.	Some concerns	Insufficient information to judge if deviations due to trial context.	Low risk of bias	No evidence of missing data or drop outs.	Some concerns	Blinding of assessors not discussed.	Some concerns	No protocol identified.	Some concerns	Some concerns due to lack of information and lack of protocol.
**Subgroup 7.1.2 Functional task training compared to Neurophysiological**												
Arya 2019	Low risk of bias	"They were allocated in a 1:1 by a simple, nonstratified randomization to undergo experimental or control intervention. The randomization process was conducted using computer‐generated random numbers by a staff not concerned with the trial." "The groups did not significantly differ in any of the characteristics"	Low risk of bias	"The outcome evaluation was conducted by a trained occupational therapist, blinded to the allocated group of the subjects. The participants were also blinded for the intervention of interest"	Low risk of bias	Data available for 47/50 participants (3 lost to follow‐up)	Low risk of bias	Assessors were blinded.	Some concerns	No protocol obtained (although a protocol is mentioned in relation to ethics approvals).	Some concerns	Low risk of bias for all domains, with exception of bias in selection of the reported result, where we have not obtained the protocol.
Bui 2019	Some concerns	States "randomized" but no further details about randomisation or allocation concealment. "There were no statistically significant difference between the two groups in population traits, stroke histories and accompanying diseases"	Some concerns	Insufficient information to judge if there were deviations from intended interventions.	Low risk of bias	No evidence of any dropouts or missing data.	Some concerns	Not stated who performed outcome assessment and whether they were blinded.	Some concerns	No protocol available.	Some concerns	Some concerns due to lack of information and lack of protocol.
Guo L 2013	Some concerns	States "randomly divided" but randomisation process not discussed. "There was no significant difference in general clinical data such as gender, age, lesion nature, hemiplegia side, and course of disease between the two groups (P>0.05)"	Some concerns	Insufficient information to judge if there were deviations from intended intervention.	Low risk of bias	No evidence of any dropouts or missing data.	Some concerns	No information relating to blinding of outcome assessor.	Some concerns	No protocol available.	Some concerns	Some concerns due to lack of information and lack of protocol.
Kim 2016	Low risk of bias	"The randomization was performed using a sealed envelope technique." "After subjects passed the screening criteria, they provided their informed consent to participation in this study." "No statistically significant differences between the two groups were found at baseline"	Low risk of bias	Interventions were delivered in different settings (circuit group or individual), and it is considered unlikely that there were deviations from the intended interventions as a result of the study.	Low risk of bias	"Both types of treatment were well‐tolerated with an attendance rate of 100%, and all participants completed the study."	Low risk of bias	"The examiner was blinded as to whether the participants were in the experimental group or control group"	Some concerns	No protocol available.	Some concerns	Low risk of bias for all domains, but some concerns as no protocol available.
Langhammer 2000	Some concerns	Double‐blind randomisation (stratified according to sex and side of lesion) and sealed coding. "The study was double blind, and the code was sealed until the last test was performed at three months follow‐up" The Bobath group was slightly more dependent at entry, a finding that could explain a poorer outcome in this group.	High risk of bias	Unclear whether the participant was blinded. Therapist was not blinded ("Information concerning the physiotherapy used was known only by the therapists who treated the patients and the secretary of the ward, who was in charge of the randomization") The same therapists provided treatment to participants in both treatment groups, creating the possibility of contamination between groups. Treatment following hospital discharge may not have been administered according to the randomisation process, potentially introducing performance bias to the postdischarge results.	Low risk of bias	29/33 in motor learning group and 24/28 in Bobath group completed intervention and had data available.	Low risk of bias	Assessor was blinded ("The tests were conducted by the project leader who had no information about which group the patient belonged to")	Some concerns	No protocol available.	High risk of bias	There was potential for bias due to deviations from the intended interventions, and some concerns over the randomisation process (which used a die) and no protocol available.
Li 2005	Some concerns	Divided by "draw method" (not clear what this is).	Some concerns	Design and implementation of study conducted by first study author. This means there is a risk of contamination relating to beliefs etc of the study author	Low risk of bias	No drop outs or missing data described.	Low risk of bias	Outcome assessor blinded	Some concerns	No protocol available.	Some concerns	Lack of information provided, and concerns around lack of blinding study author/treating therapist.
Lincoln 2003	Low risk of bias	Computer‐generated random sequence of numbers in opaque sealed envelopes opened sequentially by researcher. ("Allocation to treatment groups was by a computer generated random sequence provided by a therapist not involved with the trial, with notification delivered in opaque, sealed envelopes. Blocked randomisation was used to ensure approximately equal numbers of patients in each group at any time. Patients were screened consecutively on admission to the ward and those that met the inclusion criteria were referred for initial assessment. After the initial assessment was completed, a research therapist opened the next envelope and informed the therapists providing the treatments of the group allocation") "The groups were not significantly different in age, gender, side of stroke, type of lesion, or cognitive impairments"	High risk of bias	Different groups of physiotherapists delivering each intervention. Some possibility of contamination between groups, as physiotherapists providing the motor learning intervention were previously using Bobath therapy and therefore may have reverted to using some Bobath techniques. Also some possibility of contamination due to participants being inpatients on the same unit: the study authors state: "some aspects of the treatments could not be implemented because both treatments were occurring on the same rehabilitation wards and there was a risk of treatment contamination" Both groups had received treatment based on the Bobath approach before randomisationThe Bobath treatment was provided by physiotherapists who had previously used it, while the motor learning treatment was provided by physiotherapists previously inexperienced in motor learning who were given training before the interventions.	Low risk of bias	Dropouts were accounted for. 52/60 in Bobath group and 47/60 in motor learning group remained at one month.	Low risk of bias	"Patients were asked not to mention their treatment or therapist to the assessor"	Some concerns	No protocol available.	High risk of bias	Concerns about potential bias due to deviations from intended interventions; no protocol available.
Richards 1993	Low risk of bias	Patients stratified then block randomization scheme used. Sealed envelopes, opened remotely by telephone request	Some concerns	Unclear whether participant was blinded Therapist not blinded The same two therapists provided treatment to both treatment groups, creating the possibility of contamination between groups.	Low risk of bias	23/27 completed intervention. Participants with missing data were dropped from analysis.	Low risk of bias	At no point were independent assessors aware of the group assignment	Some concerns	No protocol available.	Some concerns	Some concerns about potential deviations from intended interventions, and lack of protocol available.
Shuai 2013	Some concerns	States "randomly divided" but no description of how randomised. There was no difference in general data between the two groups of patients.	Some concerns	Insufficient information to support a judgement.	Low risk of bias	No evidence of any dropouts or missing data.	Some concerns	No information on blinding provided.	Some concerns	No reference to a protocol .	Some concerns	Some concerns due to lack of information and lack of protocol.

**Table:** Risk of bias for analysis 7.2 Functional task training compared to other: Motor function scales

**Study**	**Bias**											
	**Randomisation process**		**Deviations from intended interventions**		**Missing outcome data**		**Measurement of the outcome**		**Selection of the reported results**		**Overall**	
	**Authors' judgement**	**Support for judgement**	**Authors' judgement**	**Support for judgement**	**Authors' judgement**	**Support for judgement**	**Authors' judgement**	**Support for judgement**	**Authors' judgement**	**Support for judgement**	**Authors' judgement**	**Support for judgement**
**Subgroup 7.2.1 Functional task training compared to 'less' functional task training**												
Aloraini 2022	Low risk of bias	"Participants were randomized into two groups using a computerized (block) randomization scheme. Pre‐stratification was done according to the participant’s pre‐morbid footedness and also the participant’s score on the main outcome measure (FMA‐LE)..." "Randomization was conducted following the consent and pre‐treatment assessments. Randomization was administered by an independent researcher who was not involved in the treatment or the assessment of participants." "Results of independent t‐tests for baseline measures showed that both groups were not significantly different at the outset of the study across all four outcome measure"	Some concerns	Insufficient information relating to delivery of interventions to judge if there could have been deviations from intended iinterventions.	Low risk of bias	"The number of participants who were randomized and allocated in their respective groups were 38 individuals. All the included individuals received the intervention program, attended the follow‐up session and were included in the final analysis of the study"	High risk of bias	Does not state that there was a blinded outcome assessor.	Some concerns	No reference to a protocol.	High risk of bias	Lack of blinded outcome assessor could introduce bias; some concerns due to lack of information about delivered interventions and lack of protocol.
Chen G 2014	Some concerns	States "randomly divided" but no further information. There were no significant differences in age, gender, paralyzed side and NIHSS score on admission between the two groups (P>0.05), which were comparable.	Some concerns	Insufficient information to judge if deviations arose because of trial context.	Low risk of bias	No evidence of drop outs or missing data.	Some concerns	No discussion of blinding	Some concerns	No protocol available.	Some concerns	Some concerns due to lack of information and lack of protocol.
Chen P 2014	Some concerns	States randomisation software used, but no further information.	Some concerns	Insufficient information to judge if there were deviations from intended interventions.	Low risk of bias	100 participants recruited and randomised into two groups of 50. Reports results data for 100 participants.	Some concerns	No information relating to blinding of outcome assessor.	Some concerns	No protocol identified.	Some concerns	Some concerns due to lack of information and lack of protocol.
Ding 2015	Some concerns	States "randomly divided" but no details around randomisation process. There was no significant difference in general data such as gender and age between the two groups (P>0.05), and they were comparable.	Some concerns	Insufficient information to judge if deviations may have occured because of the trial context.	Low risk of bias	No evidence of any drop outs or missing data.	Some concerns	No information on blinding provided.	Some concerns	No protocol available.	Some concerns	Some concerns due to lack of information and lack of protocol.
Kwakkel 2008	Low risk of bias	Participants were stratified by rehabilitation centre, and randomisation took place using an “online” minimisation procedure. Comparison between the observers’ guesses about allocation (circuit training or control) and actual allocation showed that 76 of 126 predictions were correct in the control group and 79 of 127 in the experimental group, resulting in a Cohen’s κ of 0.24. This suggests that the blinding procedure was successful. "We found significant baseline differences in favour of the circuit training group for a few secondary outcomes. All analyses, however, were adjusted for these covariates at baseline".	Low risk of bias	Interventions were delivered to participants living in the community; either a circuit training programme or usual outpatient physiotherapy. The outpatient physiotherapy was delivered by a "physiotherapist who had not been on the circuit training course at one of the participating rehabilitation centres."	Low risk of bias	"Of the 250 included patients, one patient in the circuit training group and seven in the usual care group were excluded from the analysis. Reasons were withdrawal from participation (n=3), death from cancer (n=2), and recurrent stroke (n=2), while one patient missed the 12 week assessment."	Low risk of bias	"Three trained research assistants (LW,HK,LK), who were blinded to treatment allocation, measured all outcomes"	Low risk of bias	Study protocol published, including a pre‐specified analysis plan.	Low risk of bias	No concerns ‐ study judged to be low risk of bias for all domains.
Langhammer 2007	High risk of bias	"Randomization was performed with a die: patients with uneven numbers went to group 1, an intensive exercise group, and those with even numbers to group 2, a regular exercise group. Stratification was according to gender and hemisphere lesion: the first male patient with a right hemisphere lesion and with an uneven number was allocated to the intensive exercise group, and the next male patient with a right hemisphere lesion was allocated to the regular exercise group. The procedure with the die was then used when the third male patient with a right hemisphere lesion entered the stroke unit and so on. A corresponding procedure was followed for female patients". "At discharge from the acute hospital, patients were randomized to one of two different groups by a person not involved with the patients or the treatment in the ward" "No significant differences between the groups regarding age, hemisphere lesion, marital status at baseline, or admission to the stroke unit". However, the regular exercise group had slightly higher baseline values for activities of daily living, motor function, balance and gait velocity, suggesting that the groups were not balanced.	High risk of bias	One fact that might be considered a weakness was that some therapists administered a submaximal programme to patients whom they had volunteered to exercise maximally. The reason for this was explained by the therapists involved as being a practical adaptation to pathological conditions such as heart failure, pain and a poor cognitive status, which inhibited a maximal effort. In order to carry out the exercises, adjustments were made so that routines could be maintained through the study period. This is probably also one of the reasons why so many patients complied with the exercise programmes.	Low risk of bias	8/75 dropped out; outcome data available for remaining participants.	Low risk of bias	"The study was an intention‐to‐treat trial with the aim of being double‐blind, that is, neither the investigator nor the participants knew to which group participants were allocated"	Some concerns	A protocol is referred to by the study authors, but this was not available.	High risk of bias	There was potential for bias due to deviations from the intended interventions, and some concerns over the randomisation process (which used a die) and no protocol available.
Li 2013	Some concerns	States "divided.....according to the random number table". No further information provided. Groups were clinically comparable at baseline.	Some concerns	Insufficient information to judge if there were potentially deviations due to trial context.	Low risk of bias	No evidence of any drop outs or missing data.	Some concerns	No discussion on blinding.	Some concerns	No protocol obtained.	Some concerns	Some concerns due to lack of information and lack of protocol.
Meng Qingling 2015	Some concerns	States "According to the random number table method, they were divided" but no further information. No significant difference in general data between the two groups (P>0.05).	Some concerns	Insufficient information to judge if there were deviations from intended interventions.	Low risk of bias	No evidence of dropouts or missing data.	Some concerns	Blinding not discussed.	Some concerns	No protocol available.	Some concerns	Some concerns due to lack of information and lack of protocol.
Pandian 2014	Low risk of bias	"randomly assigned either the exper‐ imental group (n=17) or the control group (n=18), using computer‐generated random numbers. The blocks were numbered and a random‐number generator program was used to select the number. The established sequence of the blocks was allocated to either one or the other group (allocation ratio 1:1). The intervention assignments were enclosed in sealed, opaque and sequentially numbered envelopes. A research assistant at the study site (not the part of study) conducted the random‐number program and concealed the allocation till the final enrollment of participants"	Some concerns	"The experimental and control interventions were given by the two independent therapists": study flow diagram implies that each delivered a different intervention. However there may have been potential for contamination between groups.	Low risk of bias	"All the subjects in the experimental (n=17) and control (n=18) groups completed the treatment protocol of 2 months". 1 loss to follow up from treatment group; 2 from control group. Data from all participants anlaysed with last observation carried over.	Low risk of bias	"Pre‐ and post assessments, using the outcome measures were carried out by one of the investigators was not involved in the interventions and had no awareness of which intervention the subjects received"	Some concerns	No protocol obtained.	Some concerns	Insufficient information to judge if there could have been deviations from the intended interventions, and no protocol obtained.
Wang Leilei 2020	Some concerns	States randomised. Lack of information about allocation concealment. No differences between groups at baseline.	Some concerns	Insufficient information to judge if there were deviations from intended interventions because of trial context. Participants were analysed according to assigned intervention.	Low risk of bias	No drop outs or missing data reported.	High risk of bias	Not clear if outcome assessor was blinded. Insufficient information to judge if outcome could have been influenced by knowledge of intervention received.	Some concerns	No protocol obtained.	High risk of bias	This is a Chinese language trial, with sections translated by one member of our team. There is generally a lack of information about the study methods. It is not clear whether the outcome assessor was blinded.
Zhang Huiyu 2021	Some concerns	States participants were divided using the "random number table method". No further information provided. There was no significant difference in general data between the two groups (P>0.05), which was comparable.	Some concerns	Insufficient information to judge if deviations due to trial context.	Low risk of bias	No evidence of missing data or drop outs.	Some concerns	Blinding of assessors not discussed.	Some concerns	No protocol identified.	Some concerns	Some concerns due to lack of information and lack of protocol.
**Subgroup 7.2.2 Functional task training compared to neurophysiological**												
Arya 2019	Low risk of bias	"They were allocated in a 1:1 by a simple, nonstratified randomization to undergo experimental or control intervention. The randomization process was conducted using computer‐generated random numbers by a staff not concerned with the trial." "The groups did not significantly differ in any of the characteristics"	Low risk of bias	"The outcome evaluation was conducted by a trained occupational therapist, blinded to the allocated group of the subjects. The participants were also blinded for the intervention of interest"	Low risk of bias	Data available for 47/50 participants (3 lost to follow‐up)	Low risk of bias	Assessors were blinded.	Some concerns	No protocol obtained (although a protocol is mentioned in relation to ethics approvals).	Some concerns	Low risk of bias for all domains, with exception of bias in selection of the reported result, where we have not obtained the protocol.
Guo L 2013	Some concerns	States "randomly divided" but randomisation process not discussed. "There was no significant difference in general clinical data such as gender, age, lesion nature, hemiplegia side, and course of disease between the two groups (P>0.05)"	Some concerns	Insufficient information to judge if there were deviations from intended intervention.	Low risk of bias	No evidence of any dropouts or missing data.	Some concerns	No information relating to blinding of outcome assessor.	Some concerns	No protocol available.	Some concerns	Some concerns due to lack of information and lack of protocol.
Kim 2016	Low risk of bias	"The randomization was performed using a sealed envelope technique." "After subjects passed the screening criteria, they provided their informed consent to participation in this study." "No statistically significant differences between the two groups were found at baseline"	Low risk of bias	Interventions were delivered in different settings (circuit group or individual), and it is considered unlikely that there were deviations from the intended interventions as a result of the study.	Low risk of bias	"Both types of treatment were well‐tolerated with an attendance rate of 100%, and all participants completed the study."	Low risk of bias	"The examiner was blinded as to whether the participants were in the experimental group or control group"	Some concerns	No protocol available.	Some concerns	Low risk of bias for all domains, but some concerns as no protocol available.
Langhammer 2000	Some concerns	Double‐blind randomisation (stratified according to sex and side of lesion) and sealed coding. "The study was double blind, and the code was sealed until the last test was performed at three months follow‐up" The Bobath group was slightly more dependent at entry, a finding that could explain a poorer outcome in this group.	High risk of bias	Unclear whether the participant was blinded. Therapist was not blinded ("Information concerning the physiotherapy used was known only by the therapists who treated the patients and the secretary of the ward, who was in charge of the randomization") The same therapists provided treatment to participants in both treatment groups, creating the possibility of contamination between groups. Treatment following hospital discharge may not have been administered according to the randomisation process, potentially introducing performance bias to the postdischarge results.	Low risk of bias	29/33 in motor learning group and 24/28 in Bobath group completed intervention and had data available.	Low risk of bias	Assessor was blinded ("The tests were conducted by the project leader who had no information about which group the patient belonged to")	Some concerns	No protocol available.	High risk of bias	There was potential for bias due to deviations from the intended interventions, and some concerns over the randomisation process and no protocol available.
Lincoln 2003	Low risk of bias	Computer‐generated random sequence of numbers in opaque sealed envelopes opened sequentially by researcher. ("Allocation to treatment groups was by a computer generated random sequence provided by a therapist not involved with the trial, with notification delivered in opaque, sealed envelopes. Blocked randomisation was used to ensure approximately equal numbers of patients in each group at any time. Patients were screened consecutively on admission to the ward and those that met the inclusion criteria were referred for initial assessment. After the initial assessment was completed, a research therapist opened the next envelope and informed the therapists providing the treatments of the group allocation") "The groups were not significantly different in age, gender, side of stroke, type of lesion, or cognitive impairments"	High risk of bias	Different groups of physiotherapists delivering each intervention. Some possibility of contamination between groups, as physiotherapists providing the motor learning intervention were previously using Bobath therapy and therefore may have reverted to using some Bobath techniques. Also some possibility of contamination due to participants being inpatients on the same unit: the study authors state: "some aspects of the treatments could not be implemented because both treatments were occurring on the same rehabilitation wards and there was a risk of treatment contamination" Both groups had received treatment based on the Bobath approach before randomisationThe Bobath treatment was provided by physiotherapists who had previously used it, while the motor learning treatment was provided by physiotherapists previously inexperienced in motor learning who were given training before the interventions.	Low risk of bias	Dropouts were accounted for. 52/60 in Bobath group and 47/60 in motor learning group remained at one month.	Low risk of bias	"Patients were asked not to mention their treatment or therapist to the assessor"	Some concerns	No protocol available.	High risk of bias	Concerns about potential bias due to deviations from intended interventions; no protocol available.
Richards 1993	Low risk of bias	Patients stratified then block randomization scheme used. Sealed envelopes, opened remotely by telephone request	Some concerns	Unclear whether participant was blinded Therapist not blinded The same two therapists provided treatment to both treatment groups, creating the possibility of contamination between groups.	Low risk of bias	23/27 completed intervention. Participants with missing data were dropped from analysis.	Low risk of bias	At no point were independent assessors aware of the group assignment	Some concerns	No protocol available.	Some concerns	Some concerns about potential deviations from intended interventions, and lack of protocol available.
Shuai 2013	Some concerns	States "randomly divided" but no description of how randomised. There was no difference in general data between the two groups of patients.	Some concerns	Insufficient information to support a judgement.	Low risk of bias	No evidence of any dropouts or missing data.	Some concerns	No information on blinding provided.	Some concerns	No reference to a protocol .	Some concerns	Some concerns due to lack of information and lack of protocol.
Wang 2005	Some concerns	"The random assignment was achieved by an independent person who chose one of the sealed envelopes 30 min before the start of the intervention." No further information about allocation concealment. "There were no differences between these two groups in age, side of hemiparesis, duration of hemiparesis and other general characteristics."	Some concerns	"Bobath group: Both therapists had been qualified for more than 10 years with at least five years of Bobath practice.Orthapaedic group: The two treating physical therapists had been qualified for more than 10 years with at least five years of orthopaedic practice on patients with stroke." This information implies that the groups were treated by different physiotherapists, but there is insufficient information to judge whether there could have been contamination between groups, with physiotherapists using 'techniques' meant for the other group.	Low risk of bias	In both groups, all randomised participants completed the treatment, and there were no missing data.	Low risk of bias	"Test results for each patient were assessed and evaluated by a separate physical therapist who was not involved in the treatment programme and did not know about the patient's group"	Some concerns	No protocol identified.	Some concerns	Some concerns due to lack of information about allocation concealment and potential deviations from intended deviations, and lack of protocol.
Wang Wenwei 2012	Some concerns	States randomised. Lack of information about allocation concealment. No differences between groups at baseline.	Some concerns	Insufficient information to judge if there were deviations from intended interventions because of trial context. Participants were analysed according to assigned intervention.	Low risk of bias	No drop outs or missing data reported.	High risk of bias	Not clear if outcome assessor was blinded. Insufficient information to judge if outcome could have been influenced by knowledge of intervention received.	Some concerns	No protocol obtained.	High risk of bias	This is a Chinese language trial, with sections translated by one member of our team. There is generally a lack of information about the study methods. It is not clear whether the outcome assessor was blinded.

**Table:** Risk of bias for analysis 7.3 Functional task training compared to other: Balance (Berg Balance Scale)

**Study**	**Bias**											
	**Randomisation process**		**Deviations from intended interventions**		**Missing outcome data**		**Measurement of the outcome**		**Selection of the reported results**		**Overall**	
	**Authors' judgement**	**Support for judgement**	**Authors' judgement**	**Support for judgement**	**Authors' judgement**	**Support for judgement**	**Authors' judgement**	**Support for judgement**	**Authors' judgement**	**Support for judgement**	**Authors' judgement**	**Support for judgement**
**Subgroup 7.3.1 Functional task training compared to 'less' functional task training**												
Aloraini 2022	Low risk of bias	"Participants were randomized into two groups using a computerized (block) randomization scheme. Pre‐stratification was done according to the participant’s pre‐morbid footedness and also the participant’s score on the main outcome measure (FMA‐LE)..." "Randomization was conducted following the consent and pre‐treatment assessments. Randomization was administered by an independent researcher who was not involved in the treatment or the assessment of participants." "Results of independent t‐tests for baseline measures showed that both groups were not significantly different at the outset of the study across all four outcome measure"	Some concerns	Insufficient information relating to delivery of interventions to judge if there could have been deviations from intended iinterventions.	Low risk of bias	"The number of participants who were randomized and allocated in their respective groups were 38 individuals. All the included individuals received the intervention program, attended the follow‐up session and were included in the final analysis of the study"	High risk of bias	Does not state that there was a blinded outcome assessor.	Some concerns	No reference to a protocol.	High risk of bias	Lack of blinded outcome assessor could introduce bias; some concerns due to lack of information about delivered interventions and lack of protocol.
Arabzadeh 2018	Low risk of bias	A randomization procedure was performed by a person who was not involved in the assessment or interventions of this study. The independent person prepared the sealed envelopes, and then folded and placed numbered cards in sealed envelopes. Before starting the exercise program sessions, each patient picked up one of the sealed envelopes in the order in which they entered into the study. Written consent obtained and allocation performed just before starting alloted exercises. The results showed no significant differences in the baseline before treatment between the two groups with respect to the clinical and laboratory measures.	Some concerns	The participants in both groups were assessed and treated by the same physiotherapist. Insufficient information to judge if this could have caused deviations or contamination between groups. Participants analysed according to assigned intervention.	Low risk of bias	20 participants randomised. No drop outs or missing data reported.	High risk of bias	Study was nonblinded. Unclear who conducted the assessments, but likely to have been same person as delivered the intervention, creating potential for influence. .	Low risk of bias	Registered in the Iranian registry of clinical trials (IRCT) under the IRCT number of 2015100224297N1. Registered trial and reported study have same outcomes and methods.	High risk of bias	High risk of bias as outcome assessment was not blinded, and some concerns about deviations from intended interventions. Low risk of bias for other domains.
Candan 2017	Low risk of bias	"After the inclusion criteria were fulfilled and the participants signed the written informed consent form, the randomization was conducted using the block randomization procedure by a physiotherapist who was not involved in this study. The randomization was stratified based on age, gender (male or female), the hemiplegic side (left or right) and type of stroke (ischemic or hemorrhagic) because of balanced distribution of the participants according to important parameters which have known prognostic effects." There were no significant differences between the groups at baseline, including demographic data and motor recovery of the lower extremity of Brunnstrom stages.	Some concerns	Intervention was delivered by the first author who "had eight years working experience in the neurologic rehabilitation field". Insufficient information to determine if this could have resulted in deviations from the intended intervention. Participants analysed according to assigned intervention.	Some concerns	33 participants randomised. 30 completed intervention. 3 drop outs in intervention group 3 drop outs were from intervention group ‐ "Two participants were excluded because of severe knee pain and having unstable blood pressure and one subject in the study group withdrew voluntarily from the study." Reasons for drop out appear to be related to the intervention, and could reflect participants who were not going to respond well to treatment. However numbers of drop outs are small.	Low risk of bias	"All participants were evaluated by a physiotherapist who was blinded to their grouping"	Some concerns	A protocol is described ("The Hacettepe University Non‐interventional Clinical Research Ethics Boards approved the study protocol of this study (approval no: GO 14/22‐15") but we not obtained this.	Some concerns	Some concerns due to potential deviations from intended interventions, greater number of drop outs from the treatment group and lack of protocol.
Chae 2017	Some concerns	Abstract states "randomly assigned" but no details of assignment in methods section. No observed differences at baseline.	Some concerns	Interventions were provided by different physical therapists, but there is insufficient information to judge whether there could have been contamination between groups or deviations from intended interventions. Participants were analysed according to the assigned group.	Low risk of bias	30 participants randomised. No missing data or drop outs reported.	High risk of bias	Does not state who performed outcome assessments and whether they were blinded to group allocation. Insufficient information to judge if lack of blinding could have influenced the result.	Some concerns	No protocol identified.	High risk of bias	Concerns due to potential lack of blinding of outcome assessor, uncertainty about randomisation and lack of information to judge if there could be deviations from intended interventions. No protocol identified.
Choi JU 2015	Some concerns	States "randomly and equally assigned" but no decription as to how randomisation was performed.	High risk of bias	Possible that the therapist through encouraging and praising improved self‐efficacy: "it is likely that by encouraging and praising the subjects as they progressed to high‐level tasks, the physical therapist helped to improve self‐efficacy." More encouragement in the treatment group, possibly due to therapist providing the intervention having a desire to demonstrate effectiveness. Partiipants were analysed according to assigned group.	Low risk of bias	No mention of dropouts or missing data.	Low risk of bias	No information relating to blinding of outcome assessor. No evidence to suggest that assessment was influenced by knowledge of intervention.	Some concerns	No protocol available.	High risk of bias	No decription as to how randomisation was performed. Possible deviations as it is possible that the therapist encouraged and praised the treatment group more than the control group: "it is likely that by encouraging and praising the subjects as they progressed to high‐level tasks, the physical therapist helped to improve self‐efficacy." No information relating to blinding of outcome assessor and no protocol available.
DOSE 2020	Low risk of bias	Randomised "using a fully concealed internet‐based dynamic allocation randomization that was generated in real time" "baseline characteristics are similar to other stroke rehabilitation trials conducted." No differences between groups.	Low risk of bias	Different therapists provided treatment for each group, limiting opportunities for contamination. Participants were analysed according to assigned group.	Low risk of bias	"Seventy‐five subjects were randomized to Usual Care (n=25), DOSE1 (n=25), or DOSE2 (n=25). One subject (randomized to DOSE2) completed the baseline, but not the post‐evaluation, because of ongoing investigational work for a suspected cardiac arrhythmia, which resulted in discontinuation of the protocol and further evaluations. After the 12‐month post‐evaluation, one subject was found to not meet the inclusion criteria of having a primary diagnosis of stroke and their data were removed from the study (usual care group)".	Low risk of bias	Blinded‐assessor.	Low risk of bias	Protocol published and study registered. Reported results are as expected according to study protocol.	Low risk of bias	No concerns. Judged as low risk of bias for all domains.
Knox 2018	Low risk of bias	"Randomized into the three intervention arms using computer‐generated random numbers and concealed allocation. A physiotherapist, blinded to group interventions, was responsible for the randomization scheme, preparation of the stratification envelopes, and group allocation." "At baseline, there were no significant differences in all the outcome measures among the groups"	Some concerns	Task intervention delivered by 1st author. Unclear if this could have introduced deviations.	Low risk of bias	144 randomised. 128 with outcome data at immediate tiimepoint. Drop outs accounted for, and not related to intervention.	Low risk of bias	"A physiotherapist, experienced in stroke rehabilitation, trained in the application of the outcome measures, and blinded to group allocation, performed the assessment."	Some concerns	Registered in clinical trials registry, but states "Retrospective registration ‐ This trial was registered after enrolment of the first participant" Trial was completed 2009‐2011, but not registered until 2018.	Some concerns	Some concerns due to lack of information about delivery of interventions, and retrospective registration of trial protocol.
Kumaran 2015	Low risk of bias	"Random sequence generation using lottery method was provided by one of the investigator blinded to the intervention." "Block randomization using 12 blocks, each consisting of six sealed envelopes" "Randomization sequence was done using sequentially numbered, opaque, sealed envelopes (SNOSE) method". No baseline differences.	Low risk of bias	States "Participants were blinded to the intervention, they received in this study". Assumed that this means participants were unaware of the two different interventions being investigated. As participants were unaware of the different interventions, contamination is considered unlikely. Participants were analysed according to assigned intervention.	Low risk of bias	62 participants randomised. No drop outs or missing data.	High risk of bias	Does not state if outcome assessor was blinded. Not clear who performed the outcome assessments. As this was a PhD study, possible that the researcher conducted outcome assessments, with potential for introduction of bias due to knowledge of intervention delivered.	Low risk of bias	Study registered in trials register. Pre‐published protocol and study results are similar in reported methods.	High risk of bias	Concerns due to lack of blinded outcome assessor and potential for influence of results. Judged as low risk of bias for other domains.
Langhammer 2007	High risk of bias	"Randomization was performed with a die: patients with uneven numbers went to group 1, an intensive exercise group, and those with even numbers to group 2, a regular exercise group. Stratification was according to gender and hemisphere lesion: the first male patient with a right hemisphere lesion and with an uneven number was allocated to the intensive exercise group, and the next male patient with a right hemisphere lesion was allocated to the regular exercise group. The procedure with the die was then used when the third male patient with a right hemisphere lesion entered the stroke unit and so on. A corresponding procedure was followed for female patients". "At discharge from the acute hospital, patients were randomized to one of two different groups by a person not involved with the patients or the treatment in the ward" "No significant differences between the groups regarding age, hemisphere lesion, marital status at baseline, or admission to the stroke unit". However, the regular exercise group had slightly higher baseline values for activities of daily living, motor function, balance and gait velocity, suggesting that the groups were not balanced.	High risk of bias	One fact that might be considered a weakness was that some therapists administered a submaximal programme to patients whom they had volunteered to exercise maximally. The reason for this was explained by the therapists involved as being a practical adaptation to pathological conditions such as heart failure, pain and a poor cognitive status, which inhibited a maximal effort. In order to carry out the exercises, adjustments were made so that routines could be maintained through the study period. This is probably also one of the reasons why so many patients complied with the exercise programmes.	Low risk of bias	8/75 dropped out; outcome data available for remaining participants.	Low risk of bias	"The study was an intention‐to‐treat trial with the aim of being double‐blind, that is, neither the investigator nor the participants knew to which group participants were allocated"	Some concerns	A protocol is referred to by the study authors, but this was not available.	High risk of bias	There was potential for bias due to deviations from the intended interventions, and some concerns over the randomisation process (which used a die) and no protocol available.
Li 2013	Some concerns	States "divided.....according to the random number table". No further information provided. Groups were clinically comparable at baseline.	Some concerns	Insufficient information to judge if there were potentially deviations due to trial context.	Low risk of bias	No evidence of any drop outs or missing data.	Some concerns	No discussion on blinding.	Some concerns	No protocol obtained.	Some concerns	Some concerns due to lack of information and lack of protocol.
Liu Yanhua 2020	Some concerns	States "randomly divided" but no further information. There was no statistically significant difference in the clinical data of the two groups of patients, and they were comparable.	Some concerns	Patients in both groups treated by "medical staff". Interventions are described, but insufficient information to know whether the same staff were delivering both interventions, whether patients in different groups could observe the treatment of the other group, and whether there could have been contamination or deviations. Participant data analysed according to assigned group.	Low risk of bias	42 people randomised to each group. No drop outs or missing data.	High risk of bias	Does not state who conducted the outcome assessments and whether they were blinded. Insufficient information to judge if assessment could be influenced by knowledge of intervention.	Some concerns	No protocol obtained.	High risk of bias	Judged as high risk of bias as no infromation relating to blinding of outcome assessor. Some concerns due to lack of information relating to allocation concealment, potential deviations from intended interventions and lack of a protocol.
Marigold 2005	Low risk of bias	A person independent of the study (i.e., concealed allocation) randomly assigned participants (using their codes) from each subgroup. "There were no differences between exercise groups for baseline descriptive variables (P>.20)"	Low risk of bias	The two exercise programme were held at a local community centre at different times, so unlikely to be opportunities for contamination between participants). Exercise programme was pre‐planned and prescribed so few opportunities for deviations from intended intervention. Participants were analysed according to assigned intervention.	Low risk of bias	"Thus, 61 persons underwent random assign‐ ment: 31 into the stretching/weight‐shifting and 30 into the agility program. Two individuals discontinued the study before baseline assessment because of time commitments. Eleven individuals discontinued the intervention..." Reasons for the 11 who discontinued intervention were: "because of time commitments (n=2), hip fracture (n=1, during exer‐cise in the agility program on a nonchallenging task that was included in both programs), illness (e.g., severe flu, hospital‐ization) (n=5), and personal reasons (n=3). Six partici‐pants were lost at retention testing because of illness (n=2), vacation (n=3), or personal reasons (n=1)". Withdrawals were balanced between groups, suggesting that the result was not biased.	Low risk of bias	All assessors were blinded to the group assignment, study design, and purpose.	Some concerns	No protocol identified.	Some concerns	Some concerns due to lack of protocol. Considered low risk of bias for all other domains.
Outermans 2010	Some concerns	"Allocation was performed by drawing randomly generated lots enclosed in opaque envelopes." No further information provided. No statistically significant differences between both groups.	Low risk of bias	Therapists were instructed not to depart from their usual care during the trial. This was monitored using the available documentation. Participants were analysed in the group to which they were assigned.	Low risk of bias	At post‐trial assessment 17/23 intervention group and 15/21 control group. Reasons for drop out are provided and appear balanced between groups.	High risk of bias	"All clinical assessments were conducted by one assessor (JO), who was not blinded for allocation. To minimize bias the assessor was not present at the group training at any time." Insufficient information to judge if lack of blinding could have influenced assessment.	Some concerns	No protocol identified.	High risk of bias	High risk of bias as outcome assessor not blinded. Some concerns due to lack of information relating to allocation concealment and no protocol.
Pandian 2014	Low risk of bias	"randomly assigned either the exper‐ imental group (n=17) or the control group (n=18), using computer‐generated random numbers. The blocks were numbered and a random‐number generator program was used to select the number. The established sequence of the blocks was allocated to either one or the other group (allocation ratio 1:1). The intervention assignments were enclosed in sealed, opaque and sequentially numbered envelopes. A research assistant at the study site (not the part of study) conducted the random‐number program and concealed the allocation till the final enrollment of participants"	Some concerns	"The experimental and control interventions were given by the two independent therapists": study flow diagram implies that each delivered a different intervention. However there may have been potential for contamination between groups.	Low risk of bias	"All the subjects in the experimental (n=17) and control (n=18) groups completed the treatment protocol of 2 months". 1 loss to follow up from treatment group; 2 from control group. Data from all participants anlaysed with last observation carried over.	Low risk of bias	"Pre‐ and post assessments, using the outcome measures were carried out by one of the investigators was not involved in the interventions and had no awareness of which intervention the subjects received"	Some concerns	No protocol obtained.	Some concerns	Insufficient information to judge if there could have been deviations from the intended interventions, and no protocol obtained.
Puckree 2014	Some concerns	States "randomly allocated" but no further details on randomisation process "the demographic profile of the participants in the groups was similar at baseline."	Some concerns	States "To reduce variability in this study, the participants in both groups received individual treatments by a therapist". Unclear what variability they are trying to avoid and how the allocation to therapists avoided this. Participants were analysed according to assigned group.	Low risk of bias	50 participants randomised. No drop outs or missing data.	High risk of bias	Does not state who conducted assessments and whether they were blinded. Insufficient information to judge if the outcome could have been influenced by an unblinded assessor.	Some concerns	No protocol obtained.	High risk of bias	High risk of bias due to lack of blinded outcome assessor. Some concerns due to insufficient information relating to randomisation, potential deviations from intended interventions and lack of protocol.
Rahayu 2020	Some concerns	"randomly allocated....using coin‐tossing technique". No information relating to allocation concealment. No significant difference was found between the two groups in terms of participants’ characteristics However, the baseline functional performance of participants between the two groups showed a statistically significant difference (p = 0.045).	Low risk of bias	The intervention group received physiotherapy from the researchers while the control group received standard physiotherapy intervention from the institutions’ physiotherapists.	Low risk of bias	Data available for nearly all ‐ 69 participants randomised; 64 completed intervention: "Two participants refused to particpate during the randomisation phase, two were lost during the follow up in the intervention group, and one needed to withdraw from the study due to worsening of the condition in the control".	Low risk of bias	"The research assistants were only responsible for data collection purpose and were blinded to the groups."	Some concerns	No protocol obtained.	Some concerns	Some concerns relating to method of randomisation and allocation concealment, and lack of protocol.
Sekhar 2013	Some concerns	States "Simple random sampling, the subjects were selected by lottery method." No further information provided. Baseline demographic data not presented.	Some concerns	Insufficient details about delivery of interventions to support a judgement about whether there could have been deviations. Analysis conducted with participants in assigned groups.	Low risk of bias	40 participants randomised and data available for 40.	High risk of bias	Does not state who conducted the outcome assessment and whether they were blinded. Insufficient information to judge if lack of blinding could have influenced the result.	Some concerns	No protocol identified.	High risk of bias	High risk of bias due to lack of blinded outcome assessor. Some concerns due to lack of information about randomisation process and interention delivery, with lack of protocol.
Shin 2011	Some concerns	"The twenty‐one subjects were randomly allocated to one of into two groups". No further information provided. "‘the pre‐intervention dynamic balance of the two groups was not significantly different’"	Some concerns	The intervention group followed a structured exercise programme, while the conventional training group received physical rehabiliation from a physical therapist who did not receive any "special instructions". Unclear if there could have been any contamination between groups. Participants analysed according to assigned group.	Low risk of bias	21 participants randomised. No drop outs or missing data.	High risk of bias	Does not state who performed outcome assessments and whether they were blind to intervention group. Insufficient information to judge if lack of blinding could have influenced results.	Some concerns	No protocol identified.	High risk of bias	High risk of bias due concerns there was no blinded outcome assessor. Some concerns due to lack of information relating to randomisation process and intervention delivery, and lack of protocol.
Yadav 2016	Some concerns	No details about randomisation process. Staes "Subjects were then randomly divided into 2 groups after getting the consent from the participants." "no statistically significant difference on pre intervention Berg balance scale (BBS) and Timed Up and Go Test (TUGT) scores was observed"	Some concerns	Groups conducted exercises at "stations". Insufficient information to judge whether there could have been deviations from the intended interventions. Participants analysed according to assigned group.	Low risk of bias	24 randomised participants. No drop outs or missing data reported.	High risk of bias	Does not state who conducted the outcome assessment and whether they were blinded. Insufficient information to judge whether lack of blinding could have influenced the results.	Some concerns	No protocol identified.	High risk of bias	High risk of bias as not clear if there was a blinded outcome assessor. Some concerns due to lack of information relating to randomisation and intervention delivered. No protocol identified.
**Subgroup 7.3.2 Functional Task Training compared to Neurophysiological**												
Anandan 2020	Low risk of bias	Computer assisted lottery method is used to divide the participants into the groups equally Written consent sought prior to the start of the study. "Data collected were homogenous in distribution, while comparing the data for the pre interventions there was no statistical significance."	Some concerns	Insufficient information on who delivered treatments to enable judgement on whether there could have been deviations from the intended intervention. Participants analysed according to assigned group.	High risk of bias	37 participants randomised to each group. "After 4 weeks there was decline in the participants noted in both the groups. There were about 30 participants in each group. After 8 weeks of treatment the withdrawal of patients is further noted and it reduced to 25 in each group. So, this study finally analyzed and completed with 25 participants in each group" Reasons for dropouts are not stated, and information is vague ("about 30 participants in each group". Dropouts of 12/37 is a almost 30%. Insufficient information to be able to judge if missingness could depend on its true value. Number of dropouts is balanced between the groups.	High risk of bias	States "The initial values were evaluated by the separate assessor..." but it is not clear if this "separate assessor" assessed the outcomes. Insufficient information to judge if lack of a blinded outcome assessor could have influenced the result.	Some concerns	No protocol identified.	High risk of bias	Judged as high risk of bias due to having a 30% drop out rate and it being unclear whether there was a blinded outcome. Some uncertainty due to lack of information about intervention delivery and no protocol.
Duan 2011	Some concerns	States randomised. Lack of information about allocation concealment. No differences between groups at baseline.	Some concerns	Insufficient information to judge if there were deviations from intended interventions because of trial context. Participants were analysed according to assigned intervention.	Low risk of bias	No drop outs or missing data reported.	High risk of bias	Not clear if outcome assessor was blinded. Insufficient information to judge if outcome could have been influenced by knowledge of intervention received.	Some concerns	No protocol obtained.	High risk of bias	This is a Chinese language trial, with sections translated by one member of our team. There is generally a lack of information about the study methods. It is not clear whether the outcome assessor was blinded.
Kim 2016	Low risk of bias	"The randomization was performed using a sealed envelope technique." "After subjects passed the screening criteria, they provided their informed consent to participation in this study." "No statistically significant differences between the two groups were found at baseline"	Low risk of bias	Interventions were delivered in different settings (circuit group or individual), and it is considered unlikely that there were deviations from the intended interventions as a result of the study.	Low risk of bias	"Both types of treatment were well‐tolerated with an attendance rate of 100%, and all participants completed the study."	Low risk of bias	"The examiner was blinded as to whether the participants were in the experimental group or control group"	Some concerns	No protocol available.	Some concerns	Low risk of bias for all domains, but some concerns as no protocol available.
Richards 1993	Low risk of bias	Patients stratified then block randomization scheme used. Sealed envelopes, opened remotely by telephone request	Some concerns	Unclear whether participant was blinded Therapist not blinded The same two therapists provided treatment to both treatment groups, creating the possibility of contamination between groups.	Low risk of bias	23/27 completed intervention. Participants with missing data were dropped from analysis.	Low risk of bias	At no point were independent assessors aware of the group assignment	Some concerns	No protocol available.	Some concerns	Some concerns about potential deviations from intended interventions, and lack of protocol available,
Wang 2005	Some concerns	"The random assignment was achieved by an independent person who chose one of the sealed envelopes 30 min before the start of the intervention." No further information about allocation concealment. "There were no differences between these two groups in age, side of hemiparesis, duration of hemiparesis and other general characteristics."	Some concerns	"Bobath group: Both therapists had been qualified for more than 10 years with at least five years of Bobath practice.Orthapaedic group: The two treating physical therapists had been qualified for more than 10 years with at least five years of orthopaedic practice on patients with stroke." This information implies that the groups were treated by different physiotherapists, but there is insufficient information to judge whether there could have been contamination between groups, with physiotherapists using 'techniques' meant for the other group.	Low risk of bias	In both groups, all randomised participants completed the treatment, and there were no missing data.	Low risk of bias	"Test results for each patient were assessed and evaluated by a separate physical therapist who was not involved in the treatment programme and did not know about the patient's group"	Some concerns	No protocol identified.	Some concerns	Some concerns due to lack of information about allocation concealment and potential deviations from intended deviations, and lack of protocol.
Wang Wenwei 2012	Some concerns	States randomised. Lack of information about allocation concealment. No differences between groups at baseline.	Some concerns	Insufficient information to judge if there were deviations from intended interventions because of trial context. Participants were analysed according to assigned intervention.	Low risk of bias	No drop outs or missing data reported.	High risk of bias	Not clear if outcome assessor was blinded. Insufficient information to judge if outcome could have been influenced by knowledge of intervention received.	Some concerns	No protocol obtained.	High risk of bias	This is a Chinese language trial, with sections translated by one member of our team. There is generally a lack of information about the study methods. It is not clear whether the outcome assessor was blinded.

**Table:** Risk of bias for analysis 7.4 Functional task training compared to other: Gait velocity

**Study**	**Bias**											
	**Randomisation process**		**Deviations from intended interventions**		**Missing outcome data**		**Measurement of the outcome**		**Selection of the reported results**		**Overall**	
	**Authors' judgement**	**Support for judgement**	**Authors' judgement**	**Support for judgement**	**Authors' judgement**	**Support for judgement**	**Authors' judgement**	**Support for judgement**	**Authors' judgement**	**Support for judgement**	**Authors' judgement**	**Support for judgement**
**Subgroup 7.4.1 Functional task training compared to 'less' functional task training**												
Alabdulwahab 2015	Low risk of bias	"Randomly assigned by drawing one of two sealed envelopes designating the group membership. Patients were consented to the study and then randomly assigned." "No statistically significant differences between both groups were found with respect to the selected outcome variables. No statistically significant differences were found ( < 0.05) with respect to measurement of [other outcomes]"	Low risk of bias	Both interventions involved wearing a weight cuff. FLO group undewent task‐oriented gait training. LORT group underwent resistance‐training. Participants would have been aware of which group they were assigned to. Therapist would have been aware of which group patient assigned to. However both interventions followed pre‐specified training protocols, making deviations from interventions unlikely. Participants were analysed according to assigned intervention.	High risk of bias	26 participants randomised. Three/26 patients dropped out. All where from the LORT group. Drop outs were reported as due to motivational reasons. No drop outs from other group. It is possible that missingness related to acceptablity of the intervention.	High risk of bias	No discussion of assessor blinding. Insufficient information to judge if assessment of the outcome could be influenced by knowledge of intervention.	Some concerns	Authors refer to a study protocol, but this has not been obtained.	High risk of bias	High risk of bias due to imbalance in drop outs between group and lack of blinded outcome assessor. No protocol available.
Aloraini 2022	Low risk of bias	"Participants were randomized into two groups using a computerized (block) randomization scheme. Pre‐stratification was done according to the participant’s pre‐morbid footedness and also the participant’s score on the main outcome measure (FMA‐LE)..." "Randomization was conducted following the consent and pre‐treatment assessments. Randomization was administered by an independent researcher who was not involved in the treatment or the assessment of participants." "Results of independent t‐tests for baseline measures showed that both groups were not significantly different at the outset of the study across all four outcome measure"	Some concerns	Insufficient information relating to delivery of interventions to judge if there could have been deviations from intended iinterventions.	Low risk of bias	"The number of participants who were randomized and allocated in their respective groups were 38 individuals. All the included individuals received the intervention program, attended the follow‐up session and were included in the final analysis of the study"	High risk of bias	Does not state that there was a blinded outcome assessor.	Some concerns	No reference to a protocol.	High risk of bias	Lack of blinded outcome assessor could introduce bias; some concerns due to lack of information about delivered interventions and lack of protocol.
Bhatia 2014	Some concerns	Abstract states "randomly assigned". Methods describe study as a "randomised experimental study design" and state "Subjects were divided into two groups". No further information is provided. Baseline data not reported.	Low risk of bias	Group 1 completed circuit‐based training. Group 2 completed resistance training using weights. Judged to be little opportunity for deviations or contamination between groups. Participants analysed in assigned group.	Low risk of bias	30 participants randomised. No dropouts or missing data reported.	High risk of bias	Does not state who performed outcome assessments and whether there was blinding. Insufficient infomation to judge if outcome could have been influenced by knowledge of intervention received.	Some concerns	No protocol identfied.	High risk of bias	High risk of bias as no blinding of outcome assessor reported; some concerns about allocation concealment and lack of protocol.
Candan 2017	Low risk of bias	"After the inclusion criteria were fulfilled and the participants signed the written informed consent form, the randomization was conducted using the block randomization procedure by a physiotherapist who was not involved in this study. The randomization was stratified based on age, gender (male or female), the hemiplegic side (left or right) and type of stroke (ischemic or hemorrhagic) because of balanced distribution of the participants according to important parameters which have known prognostic effects." There were no significant differences between the groups at baseline, including demographic data and motor recovery of the lower extremity of Brunnstrom stages.	Some concerns	Intervention was delivered by the first author who "had eight years working experience in the neurologic rehabilitation field". Insufficient information to determine if this could have resulted in deviations from the intended intervention. Participants analysed according to assigned intervention.	Some concerns	33 participants randomised. 30 completed intervention. 3 drop outs in intervention group 3 drop outs were from intervention group ‐ "Two participants were excluded because of severe knee pain and having unstable blood pressure and one subject in the study group withdrew voluntarily from the study." Reasons for drop out appear to be related to the intervention, and could reflect participants who were not going to respond well to treatment. However numbers of drop outs are small.	Low risk of bias	"All participants were evaluated by a physiotherapist who was blinded to their grouping"	Some concerns	A protocol is described ("The Hacettepe University Non‐interventional Clinical Research Ethics Boards approved the study protocol of this study (approval no: GO 14/22‐15") but we not obtained this.	Some concerns	Some concerns due to potential deviations from intended interventions, greater number of drop outs from the treatment group and lack of protocol.
Chae 2017	Some concerns	Abstract states "randomly assigned" but no details of assignment in methods section. No observed differences at baseline.	Some concerns	Interventions were provided by different physical therapists, but there is insufficient information to judge whether there could have been contamination between groups or deviations from intended interventions. Participants were analysed according to the assigned group.	Low risk of bias	30 participants randomised. No missing data or drop outs reported.	High risk of bias	Does not state who performed outcome assessments and whether they were blinded to group allocation. Insufficient information to judge if lack of blinding could have influenced the result.	Some concerns	No protocol identified.	High risk of bias	Concerns due to potential lack of blinding of outcome assessor, uncertainty about randomisation and lack of information to judge if there could be deviations from intended interventions. No protocol identified.
DOSE 2020	Low risk of bias	Randomised "using a fully concealed internet‐based dynamic allocation randomization that was generated in real time" "baseline characteristics are similar to other stroke rehabilitation trials conducted." No differences between groups.	Low risk of bias	Different therapists provided treatment for each group, limiting opportunities for contamination. Participants were analysed according to assigned group.	Low risk of bias	"Seventy‐five subjects were randomized to Usual Care (n=25), DOSE1 (n=25), or DOSE2 (n=25). One subject (randomized to DOSE2) completed the baseline, but not the post‐evaluation, because of ongoing investigational work for a suspected cardiac arrhythmia, which resulted in discontinuation of the protocol and further evaluations. After the 12‐month post‐evaluation, one subject was found to not meet the inclusion criteria of having a primary diagnosis of stroke and their data were removed from the study (usual care group)". As usual care group is not considered in this review, this means there are data for 49/50 randomised participants.	Low risk of bias	Blinded‐assessor.	Low risk of bias	Protocol published and study registered. Reported results are as expected according to study protocol.	Low risk of bias	No concerns. Judged as low risk of bias for all domains.
Hendrey 2018	Some concerns	The randomization schedule was prepared by a statistician not involved in the study using an online generator (www.randomization.com) and block size of 6. Group allocation was concealed in sealed opaque envelopes, and randomization occurred after baseline assessment "Participants in the ballistic training group tended to be earlier poststroke, faster walkers, and mostly stronger compared to the control group"	Low risk of bias	Physiotherapists providing the intervention and participants were not blinded to group allocation. It was possibly the same physiotherapists who delivered interventions to both groups, although this is unclear ("Three physiotherapists with 5‐10 years of experience working in stroke rehabilitation were trained to deliver the standardized intervention protocol". However there was a detailed intervention protocol for both groups, limiting chances of deviations from the intended intervention. Participants were analysed according to assigned intervention.	Low risk of bias	1 withdrew consent at baseline, otherwise 100% (of 15 participants) completed in each group.	Low risk of bias	Assessor‐blinded study	Low risk of bias	Clinical Trial Registration No.: NCT01958736. Study protocol appears to be in accordance with published results.	Some concerns	Some concerns due to differences between groups at baseline. Judged as low risk of bias for other domains.
Knox 2018	Low risk of bias	"Randomized into the three intervention arms using computer‐generated random numbers and concealed allocation. A physiotherapist, blinded to group interventions, was responsible for the randomization scheme, preparation of the stratification envelopes, and group allocation." "At baseline, there were no significant differences in all the outcome measures among the groups"	Some concerns	Task intervention delivered by 1st author. Unclear if this could have introduced deviations.	Low risk of bias	144 randomised. 128 with outcome data at immediate tiimepoint. Drop outs accounted for, and not related to intervention.	Low risk of bias	"A physiotherapist, experienced in stroke rehabilitation, trained in the application of the outcome measures, and blinded to group allocation, performed the assessment."	Some concerns	Registered in clinical trials registry, but states "Retrospective registration ‐ This trial was registered after enrolment of the first participant" Trial was completed 2009‐2011, but not registered until 2018.	Some concerns	Some concerns due to lack of information about delivery of interventions, and retrospective registration of trial protocol.
Kumaran 2015	Low risk of bias	"Random sequence generation using lottery method was provided by one of the investigator blinded to the intervention." "Block randomization using 12 blocks, each consisting of six sealed envelopes" "Randomization sequence was done using sequentially numbered, opaque, sealed envelopes (SNOSE) method". No baseline differences.	Low risk of bias	States "Participants were blinded to the intervention, they received in this study". Assumed that this means participants were unaware of the two different interventions being investigated. As participants were unaware of the different interventions, contamination is considered unlikely. Participants were analysed according to assigned intervention.	Low risk of bias	62 participants randomised. No drop outs or missing data.	High risk of bias	Does not state if outcome assessor was blinded. Not clear who performed the outcome assessments. As this was a PhD study, possible that the researcher conducted outcome assessments, with potential for introduction of bias due to knowledge of intervention delivered.	Low risk of bias	Study registered in trials register. Pre‐published protocol and study results are similar in reported methods.	High risk of bias	Concerns due to lack of blinded outcome assessor and potential for influence of results. Judged as low risk of bias for other domains.
Kwakkel 2008	Low risk of bias	Participants were stratified by rehabilitation centre, and randomisation took place using an “online” minimisation procedure. Comparison between the observers’ guesses about allocation (circuit training or control) and actual allocation showed that 76 of 126 predictions were correct in the control group and 79 of 127 in the experimental group, resulting in a Cohen’s κ of 0.24. This suggests that the blinding procedure was successful. "We found significant baseline differences in favour of the circuit training group for a few secondary outcomes. All analyses, however, were adjusted for these covariates at baseline".	Low risk of bias	Interventions were delivered to participants living in the community; either a circuit training programme or usual outpatient physiotherapy. The outpatient physiotherapy was delivered by a "physiotherapist who had not been on the circuit training course at one of the participating rehabilitation centres."	Low risk of bias	"Of the 250 included patients, one patient in the circuit training group and seven in the usual care group were excluded from the analysis. Reasons were withdrawal from participation (n=3), death from cancer (n=2), and recurrent stroke (n=2), while one patient missed the 12 week assessment."	Low risk of bias	"Three trained research assistants (LW,HK,LK), who were blinded to treatment allocation, measured all outcomes"	Low risk of bias	Study protocol published, including a pre‐specified analysis plan.	Low risk of bias	No concerns ‐ study judged to be low risk of bias for all domains.
Langhammer 2007	High risk of bias	"Randomization was performed with a die: patients with uneven numbers went to group 1, an intensive exercise group, and those with even numbers to group 2, a regular exercise group. Stratification was according to gender and hemisphere lesion: the first male patient with a right hemisphere lesion and with an uneven number was allocated to the intensive exercise group, and the next male patient with a right hemisphere lesion was allocated to the regular exercise group. The procedure with the die was then used when the third male patient with a right hemisphere lesion entered the stroke unit and so on. A corresponding procedure was followed for female patients". "At discharge from the acute hospital, patients were randomized to one of two different groups by a person not involved with the patients or the treatment in the ward" "No significant differences between the groups regarding age, hemisphere lesion, marital status at baseline, or admission to the stroke unit". However, the regular exercise group had slightly higher baseline values for activities of daily living, motor function, balance and gait velocity, suggesting that the groups were not balanced.	High risk of bias	One fact that might be considered a weakness was that some therapists administered a submaximal programme to patients whom they had volunteered to exercise maximally. The reason for this was explained by the therapists involved as being a practical adaptation to pathological conditions such as heart failure, pain and a poor cognitive status, which inhibited a maximal effort. In order to carry out the exercises, adjustments were made so that routines could be maintained through the study period. This is probably also one of the reasons why so many patients complied with the exercise programmes.	Low risk of bias	8/75 dropped out; outcome data available for remaining participants.	Low risk of bias	"The study was an intention‐to‐treat trial with the aim of being double‐blind, that is, neither the investigator nor the participants knew to which group participants were allocated"	Some concerns	A protocol is referred to by the study authors, but this was not available.	High risk of bias	There was potential for bias due to deviations from the intended interventions, and some concerns over the randomisation process (which used a die) and no protocol available.
Lawal 2016	Low risk of bias	"Randomisation will be conducted using a computer‐generated random allocation sequence schedule held by a third party, who will randomly allocate recruited participants into the study group"	Low risk of bias	"All the activities for the control group were conducted by regular therapists (who are similar in qualification/experience to therapists implementing the CCT programme) in the Physiotherapy Department of AKTH." ‐ implies different therapists for the different groups, thus minimising potential for deviations.	Low risk of bias	"All participants completed the eight weeks therapy, with percentage range of drop‐out starting from a minimum of 7% in group D (control) to a maximum of 9% in groups A and B". Reasons for drop outs provided.	Low risk of bias	"To eliminate bias, the assessment of outcome will be performed by (experienced/trained) blinded assessors, who will be blinded to the nature/type of intervention as well as the intervention groups of the participants. Participants will also be instructed not to disclose their individual intervention groups to the assessors."	Low risk of bias	Published study protocol and trial registration. Final result presented as pre‐specified in protocol.	Low risk of bias	No concerns.
Li 2013	Some concerns	States "divided.....according to the random number table". No further information provided. Groups were clinically comparable at baseline.	Some concerns	Insufficient information to judge if there were potentially deviations due to trial context.	Low risk of bias	No evidence of any drop outs or missing data.	Some concerns	No discussion on blinding.	Some concerns	No protocol obtained.	Some concerns	Some concerns due to lack of information and lack of protocol.
Liu Yanhua 2020	Some concerns	States "randomly divided" but no further information. There was no statistically significant difference in the clinical data of the two groups of patients, and they were comparable.	Some concerns	Patients in both groups treated by "medical staff". Interventions are described, but insufficient information to know whether the same staff were delivering both interventions, whether patients in different groups could observe the treatment of the other group, and whether there could have been contamination or deviations. Participant data analysed according to assigned group.	Low risk of bias	42 people randomised to each group. No drop outs or missing data.	High risk of bias	Does not state who conducted the outcome assessments and whether they were blinded. Insufficient information to judge if assessment could be influenced by knowledge of intervention.	Some concerns	No protocol obtained.	High risk of bias	Judged as high risk of bias as no infromation relating to blinding of outcome assessor. Some concerns due to lack of information relating to allocation concealment, potential deviations from intended interventions and lack of a protocol.
Marigold 2005	Low risk of bias	A person independent of the study (i.e., concealed allocation) randomly assigned participants (using their codes) from each subgroup. "There were no differences between exercise groups for baseline descriptive variables (P>.20)"	Low risk of bias	The two exercise programme were held at a local community centre at different times, so unlikely to be opportunities for contamination between participants). Exercise programme was pre‐planned and prescribed so few opportunities for deviations from intended intervention. Participants were analysed according to assigned intervention.	Low risk of bias	"Thus, 61 persons underwent random assign‐ ment: 31 into the stretching/weight‐shifting and 30 into the agility program. Two individuals discontinued the study before baseline assessment because of time commitments. Eleven individuals discontinued the intervention..." Reasons for the 11 who discontinued intervention were: "because of time commitments (n=2), hip fracture (n=1, during exer‐cise in the agility program on a nonchallenging task that was included in both programs), illness (e.g., severe flu, hospital‐ization) (n=5), and personal reasons (n=3). Six partici‐pants were lost at retention testing because of illness (n=2), vacation (n=3), or personal reasons (n=1)". Withdrawals were balanced between groups, suggesting that the result was not biased.	Low risk of bias	All assessors were blinded to the group assignment, study design, and purpose.	Some concerns	No protocol identified.	Some concerns	Some concerns due to lack of protocol. Considered low risk of bias for all other domains.
Mendoza 2015	Low risk of bias	An allocation sequence using a random numbers table was generated and the envelopes were arranged accordingly. For every new participant, the top envelope was chosen to determine the participant’s intervention. After screening, eligible participants were oriented to the study protocol and provided written consent. demographic characteristics of the two groups were comparable at baseline	Low risk of bias	"The intervention was provided by trained physical therapists with clinical experience ranging from one to ten years, assisted by physical therapy students who were trained in the conduct of the treatment protocol. During the treatment sessions for both groups, one to two therapists were present to oversee the administration of the program." Each of the groups received circuit‐based exercises with pre‐defined exercises; delivery was overseen, limiting chances of deviations for the intended interventions. Participants were analysed according to assigned group.	Low risk of bias	All participants completed the treatment as allocated. "Only two absences were allowed to remain eligible to participate in the trial and none of the participants exceeded the limit."	Low risk of bias	"A physical therapist with more than 20 years of experience and who was blinded to the group allocation conducted the examination"	Some concerns	No protocol obained.	Some concerns	Some concerns as we have not obtained the study protocol, but judged as low risk of bias for other domains.
Meng 2022	Low risk of bias	"Patients who met the criteria were randomly allocated to the RAGT, ELLT, and CRT groups using the NCSS‐PASS program‐generated randomization table, at an allocation ratio of 1:1:1. A principal investigator generated random assignment sequences for participants in the NCSS‐PASS, and the random assignments were concealed in consecutively numbered sealed opaque enve‐ lopes, which were sequentially opened after each patient provided written informed consent" "There were no significant differences in age, sex, side and type of stroke, duration, and clinical measures (6MWT, FAC, TUG, DTW, Tinetti, BI, SS‐QOL, and gait parameters) at baseline among the three groups"	Some concerns	"Owing to the random and single‐blinded study design, only the evaluator and statistician were blinded to the grouping procedures" Insufficient information to judge if there may have been deviations from intended intervention.	Low risk of bias	Assume that data in unlabelled row in Table 2 is Barthel Index.	Low risk of bias	Evaluator was blinded.	High risk of bias	Protocol was registered ("registered in the Chinese Clinical Trial Registry (no. ChiCTR1900026225)"). However this states that this was a "Non‐randomized controlled study, no random methods involved", and only states two treatment groups (3 are reported). Outcomes in protocol are stated as NIHSS, Fugl Meyer and Barthel Index; outcomes in results paper are different, and do not include Fugl Meyer.	High risk of bias	Study described in results paper judged as low risk of bias for most domain, but published protocol differs from reported study in terms of study design and outcomes.
Outermans 2010	Some concerns	"Allocation was performed by drawing randomly generated lots enclosed in opaque envelopes." No further information provided. No statistically significant differences between both groups.	Low risk of bias	Therapists were instructed not to depart from their usual care during the trial. This was monitored using the available documentation. Participants were analysed in the group to which they were assigned.	Low risk of bias	At post‐trial assessment 17/23 intervention group and 15/21 control group. Reasons for drop out are provided and appear balanced between groups.	High risk of bias	"All clinical assessments were conducted by one assessor (JO), who was not blinded for allocation. To minimize bias the assessor was not present at the group training at any time." Insufficient information to judge if lack of blinding could have influenced assessment.	Some concerns	No protocol identified.	High risk of bias	High risk of bias as outcome assessor not blinded. Some concerns due to lack of information relating to allocation concealment and no protocol.
Signal 2014	Low risk of bias	"Following baseline assessment each participant was assigned using pseudo‐randomisation (minimisation)...". Minimisation is considered low risk of bias. "All participants were assigned within the same session by a blinded researcher not involved in other aspects of the trial to ensure that no selection bias was introduced." Intervention groups balanced.	Low risk of bias	"All participants were blinded to the study hypothesis and were only informed whether theyhad been allocated to a rehabilitation or control group." Unblinded physio observed each group to ensure fidelity to the intervention was maintained. Participants were analysed according to the group to which they were randomised.	Low risk of bias	20 randomised. Data from 19 analysed.	Low risk of bias	All assessments conducted by blinded personnel.	Low risk of bias	Trial registered: ACTRN12610000460000 Data reported and analysed according to pre‐stated plan.	Low risk of bias	Considered low risk of bias for all domains.
Wang Leilei 2020	Some concerns	States randomised. Lack of information about allocation concealment. No differences between groups at baseline.	Some concerns	Insufficient information to judge if there were deviations from intended interventions because of trial context. Participants were analysed according to assigned intervention.	Low risk of bias	No drop outs or missing data reported.	High risk of bias	Not clear if outcome assessor was blinded. Insufficient information to judge if outcome could have been influenced by knowledge of intervention received.	Some concerns	No protocol obtained.	High risk of bias	This is a Chinese language trial, with sections translated by one member of our team. There is generally a lack of information about the study methods. It is not clear whether the outcome assessor was blinded.
Yadav 2016	Some concerns	No details about randomisation process. Staes "Subjects were then randomly divided into 2 groups after getting the consent from the participants." "no statistically significant difference on pre intervention Berg balance scale (BBS) and Timed Up and Go Test (TUGT) scores was observed"	Some concerns	Groups conducted exercises at "stations". Insufficient information to judge whether there could have been deviations from the intended interventions. Participants analysed according to assigned group.	Low risk of bias	24 randomised participants. No drop outs or missing data reported.	High risk of bias	Does not state who conducted the outcome assessment and whether they were blinded. Insufficient information to judge whether lack of blinding could have influenced the results.	Some concerns	No protocol identified.	High risk of bias	High risk of bias as not clear if there was a blinded outcome assessor. Some concerns due to lack of information relating to randomisation and intervention delivered. No protocol identified.
**Subgroup 7.4.2 Functional Task Training compared to Neurophysiological**												
Duan 2011	Some concerns	States randomised. Lack of information about allocation concealment. No differences between groups at baseline.	Some concerns	Insufficient information to judge if there were deviations from intended interventions because of trial context. Participants were analysed according to assigned intervention.	Low risk of bias	No drop outs or missing data reported.	High risk of bias	Not clear if outcome assessor was blinded. Insufficient information to judge if outcome could have been influenced by knowledge of intervention received.	Some concerns	No protocol obtained.	High risk of bias	This is a Chinese language trial, with sections translated by one member of our team. There is generally a lack of information about the study methods. It is not clear whether the outcome assessor was blinded.
Kim 2016	Low risk of bias	"The randomization was performed using a sealed envelope technique." "After subjects passed the screening criteria, they provided their informed consent to participation in this study." "No statistically significant differences between the two groups were found at baseline"	Low risk of bias	Interventions were delivered in different settings (circuit group or individual), and it is considered unlikely that there were deviations from the intended interventions as a result of the study.	Low risk of bias	"Both types of treatment were well‐tolerated with an attendance rate of 100%, and all participants completed the study."	Low risk of bias	"The examiner was blinded as to whether the participants were in the experimental group or control group"	Some concerns	No protocol available.	Some concerns	Low risk of bias for all domains, but some concerns as no protocol available.
Lincoln 2003	Low risk of bias	Computer‐generated random sequence of numbers in opaque sealed envelopes opened sequentially by researcher. ("Allocation to treatment groups was by a computer generated random sequence provided by a therapist not involved with the trial, with notification delivered in opaque, sealed envelopes. Blocked randomisation was used to ensure approximately equal numbers of patients in each group at any time. Patients were screened consecutively on admission to the ward and those that met the inclusion criteria were referred for initial assessment. After the initial assessment was completed, a research therapist opened the next envelope and informed the therapists providing the treatments of the group allocation") "The groups were not significantly different in age, gender, side of stroke, type of lesion, or cognitive impairments"	High risk of bias	Different groups of physiotherapists delivering each intervention. Some possibility of contamination between groups, as physiotherapists providing the motor learning intervention were previously using Bobath therapy and therefore may have reverted to using some Bobath techniques. Also some possibility of contamination due to participants being inpatients on the same unit: the study authors state: "some aspects of the treatments could not be implemented because both treatments were occurring on the same rehabilitation wards and there was a risk of treatment contamination" Both groups had received treatment based on the Bobath approach before randomisationThe Bobath treatment was provided by physiotherapists who had previously used it, while the motor learning treatment was provided by physiotherapists previously inexperienced in motor learning who were given training before the interventions.	Low risk of bias	Dropouts were accounted for. 52/60 in Bobath group and 47/60 in motor learning group remained at one month.	Low risk of bias	"Patients were asked not to mention their treatment or therapist to the assessor"	Some concerns	No protocol available.	High risk of bias	Concerns about potential bias due to deviations from intended interventions; no protocol available.
Richards 1993	Low risk of bias	Patients stratified then block randomization scheme used. Sealed envelopes, opened remotely by telephone request	Some concerns	Unclear whether participant was blinded Therapist not blinded The same two therapists provided treatment to both treatment groups, creating the possibility of contamination between groups.	Low risk of bias	23/27 completed intervention. Participants with missing data were dropped from analysis.	Low risk of bias	At no point were independent assessors aware of the group assignment	Some concerns	No protocol available.	Some concerns	Some concerns about potential deviations from intended interventions, and lack of protocol available.
Verma 2011	Low risk of bias	"After the blocks were numbered, a random‐number generator program was used to select numbers that established the sequence in which blocks were allocated to either one or the other group." "The subjects were blinded for intervention of interest." "The groups did not significantly differ in any of the demographic and baseline clinical characteristics"	Low risk of bias	"The subjects were blinded for intervention of interest." "The experimental and control interventions were given by 2 independent therapists (K.N.A. and T.V.)." This is judged to make deviations from the intended intervention unlikely. Participants analysed according to assigned intervention.	Low risk of bias	There was one patient lost to follow‐up, from the experimental group because of a second stroke).	Low risk of bias	"The present study was an assessor‐blinded (R.V.), randomized controlled design."	Some concerns	Reported that "the present study was registered in the Clinical Trial Registry of India as CTRI/2010/091/000606.". However we have been unable to access this protocol.	Some concerns	Judged as low risk of bias for all domains, except for selection of reported result where we were unable to access the reported trials registration.
Wang Wenwei 2012	Some concerns	States randomised. Lack of information about allocation concealment. No differences between groups at baseline.	Some concerns	Insufficient information to judge if there were deviations from intended interventions because of trial context. Participants were analysed according to assigned intervention.	Low risk of bias	No drop outs or missing data reported.	High risk of bias	Not clear if outcome assessor was blinded. Insufficient information to judge if outcome could have been influenced by knowledge of intervention received.	Some concerns	No protocol obtained.	High risk of bias	This is a Chinese language trial, with sections translated by one member of our team. There is generally a lack of information about the study methods. It is not clear whether the outcome assessor was blinded.

**Table:** Risk of bias for analysis 7.5 Functional task training compared to other: Length of hospital stay

**Study**	**Bias**											
	**Randomisation process**		**Deviations from intended interventions**		**Missing outcome data**		**Measurement of the outcome**		**Selection of the reported results**		**Overall**	
	**Authors' judgement**	**Support for judgement**	**Authors' judgement**	**Support for judgement**	**Authors' judgement**	**Support for judgement**	**Authors' judgement**	**Support for judgement**	**Authors' judgement**	**Support for judgement**	**Authors' judgement**	**Support for judgement**
**Subgroup 7.5.1 Functional task training vs less functional task training**												
Langhammer 2007	High risk of bias	"Randomization was performed with a die: patients with uneven numbers went to group 1, an intensive exercise group, and those with even numbers to group 2, a regular exercise group. Stratification was according to gender and hemisphere lesion: the first male patient with a right hemisphere lesion and with an uneven number was allocated to the intensive exercise group, and the next male patient with a right hemisphere lesion was allocated to the regular exercise group. The procedure with the die was then used when the third male patient with a right hemisphere lesion entered the stroke unit and so on. A corresponding procedure was followed for female patients". "At discharge from the acute hospital, patients were randomized to one of two different groups by a person not involved with the patients or the treatment in the ward" "No significant differences between the groups regarding age, hemisphere lesion, marital status at baseline, or admission to the stroke unit". However, the regular exercise group had slightly higher baseline values for activities of daily living, motor function, balance and gait velocity, suggesting that the groups were not balanced.	High risk of bias	One fact that might be considered a weakness was that some therapists administered a submaximal programme to patients whom they had volunteered to exercise maximally. The reason for this was explained by the therapists involved as being a practical adaptation to pathological conditions such as heart failure, pain and a poor cognitive status, which inhibited a maximal effort. In order to carry out the exercises, adjustments were made so that routines could be maintained through the study period. This is probably also one of the reasons why so many patients complied with the exercise programmes.	Low risk of bias	8/75 dropped out; outcome data available for remaining participants.	Low risk of bias	"The study was an intention‐to‐treat trial with the aim of being double‐blind, that is, neither the investigator nor the participants knew to which group participants were allocated"	Some concerns	A protocol is referred to by the study authors, but this was not available.	High risk of bias	There was potential for bias due to deviations from the intended interventions, and some concerns over the randomisation process (which used a die) and no protocol available.

**Table:** Risk of bias for analysis 7.6 Functional task training compared to other approaches: Adverse events

**Study**	**Bias**											
	**Randomisation process**		**Deviations from intended interventions**		**Missing outcome data**		**Measurement of the outcome**		**Selection of the reported results**		**Overall**	
	**Authors' judgement**	**Support for judgement**	**Authors' judgement**	**Support for judgement**	**Authors' judgement**	**Support for judgement**	**Authors' judgement**	**Support for judgement**	**Authors' judgement**	**Support for judgement**	**Authors' judgement**	**Support for judgement**
DOSE 2020	Low risk of bias	Randomised "using a fully concealed internet‐based dynamic allocation randomization that was generated in real time" "baseline characteristics are similar to other stroke rehabilitation trials conducted." No differences between groups.	Low risk of bias	Different therapists provided treatment for each group, limiting opportunities for contamination. Participants were analysed according to assigned group.	Low risk of bias	"Seventy‐five subjects were randomized to Usual Care (n=25), DOSE1 (n=25), or DOSE2 (n=25). One subject (randomized to DOSE2) completed the baseline, but not the post‐evaluation, because of ongoing investigational work for a suspected cardiac arrhythmia, which resulted in discontinuation of the protocol and further evaluations. After the 12‐month post‐evaluation, one subject was found to not meet the inclusion criteria of having a primary diagnosis of stroke and their data were removed from the study (usual care group)".	High risk of bias	Data are provided for both DOSE groups combined, so it is unclear whether reported adverse events occured in DOSE 1 (analysed) or DOSE 2 (not analysed) group. Further, adverse events in DOSE groups occured after intervention period, while usual care groups adverse events occured during intervention period.	Low risk of bias	Protocol published and study registered. Reported results are as expected according to study protocol.	High risk of bias	Judged as high risk of bias due to concerns about reporting of adverse events (with data combined across groups).
Hendrey 2018	Some concerns	The randomization schedule was prepared by a statistician not involved in the study using an online generator (www.randomization.com) and block size of 6. Group allocation was concealed in sealed opaque envelopes, and randomization occurred after baseline assessment "Participants in the ballistic training group tended to be earlier poststroke, faster walkers, and mostly stronger compared to the control group"	Low risk of bias	Physiotherapists providing the intervention and participants were not blinded to group allocation. It was possibly the same physiotherapists who delivered interventions to both groups, although this is unclear ("Three physiotherapists with 5‐10 years of experience working in stroke rehabilitation were trained to deliver the standardized intervention protocol". However there was a detailed intervention protocol for both groups, limiting chances of deviations from the intended intervention. Participants were analysed according to assigned intervention.	Low risk of bias	1 withdrew consent at baseline, otherwise 100% (of 15 participants) completed in each group.	Low risk of bias	Recording of adverse events was pre‐planned and clearly reported.	Low risk of bias	Clinical Trial Registration No.: NCT01958736. Study protocol appears to be in accordance with published results.	Some concerns	Some concerns due to differences between groups at baseline. Judged as low risk of bias for other domains.
Kwakkel 2008	Low risk of bias	Participants were stratified by rehabilitation centre, and randomisation took place using an “online” minimisation procedure. Comparison between the observers’ guesses about allocation (circuit training or control) and actual allocation showed that 76 of 126 predictions were correct in the control group and 79 of 127 in the experimental group, resulting in a Cohen’s κ of 0.24. This suggests that the blinding procedure was successful. "We found significant baseline differences in favour of the circuit training group for a few secondary outcomes. All analyses, however, were adjusted for these covariates at baseline".	Low risk of bias	Interventions were delivered to participants living in the community; either a circuit training programme or usual outpatient physiotherapy. The outpatient physiotherapy was delivered by a "physiotherapist who had not been on the circuit training course at one of the participating rehabilitation centres."	Low risk of bias	"Of the 250 included patients, one patient in the circuit training group and seven in the usual care group were excluded from the analysis. Reasons were withdrawal from participation (n=3), death from cancer (n=2), and recurrent stroke (n=2), while one patient missed the 12 week assessment."	Low risk of bias	"The staff recorded patients’ attendance at the sessions and adverse events (such as falls, heart problems) during the intervention. Serious adverse events were defined as any fall or other adverse event related to treatment that required a hospital or GP visit. Serious adverse events were reported to the medical ethics committee"	Some concerns	Assessment of adverse events are not reported in the protocol.	Some concerns	Some concerns due to the assessment of adverse events not being pre‐stated in the protocol.
Mansfield 2018	Low risk of bias	"Participants will be assigned using blocked stratified randomization with allocation concealment to one of two training groups: 1) perturbation training, or 2)‘traditional’balance training (control). To maintainallocation concealment, a variable block size ranging from 4–8 will be used. There will be four strata based on two stratification factors: site (two levels), and frequencyof ‘failures’ during baseline reactive balance controlassessment (two levels). Stratification by site will ensure that the treatment groups are balanced within each insti‐tution accounting for potential differences in interventionadministration between sites" "no significant differences betweengroups on any baseline characteristics."	Some concerns	"Interventions will be administered on a 1:1 basis (i.e., onephysiotherapist per participant) by a trained and licensed physiotherapist. Interventions will follow a general guide but will be tailored to the individual participants’ability,and individualized instructions and task modifications willbe used to target participant‐specific impairments in bal‐ance control." Both interventions appear to be delivered by same physiotherapist meaning there could be contamination between groups.	Some concerns	88 participants randomised (44 to each group) and 39 and 38 respectively post‐training. "Eight participants who consented to participate in the study were excluded on the initial assessment because they could not tolerate the lean‐and‐release postural perturbations. Participants were withdrawn after randomisation because it became apparent that they did not meet the study criteria (one PBT participant had osteoporosis with history of fracture and one control participant had uncontrolled hypertension), or because they had a significant decline in health during the training portion of the study (one PBT and one control participant). One PBT participant withdrew from the study because she did not like the group allocation. Therefore, there were 42 control participants and 41 PBT participants available for analysis of the primary outcome (falls in daily life). Some concerns around reasons for study withdrawals.	Low risk of bias	Assessment of adverse events was pre‐planned and clearly reported.	Low risk of bias	Study protocol appears to be in accordance with published results.	Some concerns	Some concerns due to potential biases relating to missing outcome data.
SPIRES 2022	Low risk of bias	"After baseline assessment, participants were allocated(1:1) by computer‐generated assignment to interven‐tion or control group by the Peninsula Clinical TrialsUnit (PenCTU). A minimisation procedure was used to minimise imbalance between groups with regard to both baseline fatigue and OH, using a bespoke, web‐basedsystem designed by the PenCTU. The minimisation algorithm included a random element, with probability of 0.9 for least imbalance allocation and 0.1 for other allocation. " Baseline characteristics were "well‐balanced".	Low risk of bias	Detailed protocol which physiotherapists followed. Considered few oppotunities for deviations from intended intervention.	Low risk of bias	45 participants randomised. 18/22 received intervention. 22/23 received control. 17/18 in intervention group completed post treatment assessment. 22/22 in control group completed post treatment assessment. Reasons for drop‐outs fully described.	Low risk of bias	Number and nature of serious adverse events(SAEs) and adverse events (AEs) recorded for both groups. Adverse events detailed in supplementary information.	Low risk of bias	No apparent differences between reported study and protocol.	Low risk of bias	Judged as low risk of bias for all domains.
Signal 2014	Low risk of bias	"Following baseline assessment each participant was assigned using pseudo‐randomisation (minimisation)...". Minimisation is considered low risk of bias. "All participants were assigned within the same session by a blinded researcher not involved in other aspects of the trial to ensure that no selection bias was introduced." Intervention groups balanced.	Low risk of bias	"All participants were blinded to the study hypothesis and were only informed whether theyhad been allocated to a rehabilitation or control group." Unblinded physio observed each group to ensure fidelity to the intervention was maintained. Participants were analysed according to the group to which they were randomised.	Low risk of bias	20 randomised. Data from 19 analysed. Adverse events reported for all groups.	Low risk of bias	Recording of adverse events pre‐planned and clearly reported.	Low risk of bias	Trial registered: ACTRN12610000460000 Data reported and analysed according to pre‐stated plan.	Low risk of bias	Considered low risk of bias for all domains.

**Table:** Risk of bias for analysis 7.7 Neurophysiological approaches compared to other: Independence in ADL scales

**Study**	**Bias**											
	**Randomisation process**		**Deviations from intended interventions**		**Missing outcome data**		**Measurement of the outcome**		**Selection of the reported results**		**Overall**	
	**Authors' judgement**	**Support for judgement**	**Authors' judgement**	**Support for judgement**	**Authors' judgement**	**Support for judgement**	**Authors' judgement**	**Support for judgement**	**Authors' judgement**	**Support for judgement**	**Authors' judgement**	**Support for judgement**
**Subgroup 7.7.1 Neurophysiological compared to no (or minimal) neurophysiological**												
Epple 2020	Some concerns	"Patients were randomly assigned (1:1) with sealed, opaque and sequentially numbered envelopes to receive usual stroke unit care with Vojta therapy (intervention group) or standard physiotherapy (control group). Investigators were instructed to assign the patient according to the envelope with the lowest randomisation number. Randomisation was done by the investigator, who enrolled the subject." Unclear if allocation remained concealed until participants were enrolled. "Baseline characteristics did not differ statistically between the two groups, with the exception of the side of lesion, prior known orthopaedic conditions, and scores in TCT, CBS and NIHSS at baseline (Table 2), so that patients randomised to Vojta group were slightly more affected by the stroke."	Low risk of bias	Investigators and therapists were not blinded to the treatment allocation. Different physiotherapists provided the treatments to the two groups. The Vojta therapists "had received special training and certification". No Vojta‐therapist treated patients in the control group.	Low risk of bias	"One patient in the control group died at day 6, before reaching the primary outcome due to a malignant middle cerebral artery (MCA) infarction. Thirty‐nine patients (97.5%) were included in the ITT analysis. Two patients (one in each group) died after discharge from hospital before day 90, one due to pneumonia in the rehabilitation centre on day 65 (Vojta group) and one due to endocarditis on day 32 (control group). Thirty‐seven patients (92.5%) were included in the three‐months follow‐up assessment."	Low risk of bias	Clinical assessment at day 90 was blinded	Low risk of bias	Protocol and trials registration available; study results are as pre‐specified in the protocol.	Some concerns	Some concerns relating to allocation concealment, with randomisation conducted by the chief investigator.
Fan WS 2006	Some concerns	Randomisation process not described.	Some concerns	Insufficient information to judge if there were deviations from intended interventions.	Low risk of bias	No dropouts.	Some concerns	No discussion around blinding.	Some concerns	No protocol identified.	Some concerns	Some concerns due to lack of information and lack of protocol.
Ge Y 2020	Some concerns	States "random number table method" but no further information. "There was no significant difference in age, gender and course of disease between the two groups ( 0.05). "	Some concerns	Insufficient information to judge if there were deviations from intended interventions.	Low risk of bias	No evidence of dropouts or missing data.	Some concerns	No information relating to blinding of outcome assessor.	Some concerns	No protocol obtained.	Some concerns	Some concerns due to lack of information and lack of protocol.
Gelber 1995	Some concerns	States patients were randomised, but no information as to how. "NDT and TFR treated patients did not differ with respect to age, gender, side of stroke or days from stroke to entry in the study"	High risk of bias	Unclear whether the participant was blinded. The same therapists provided treatment to participants in both treatment groups, creating a possibility of contamination between the groups.	Low risk of bias	Dropouts accounted for. 27/27 completed intervention, with data for this outcome.	High risk of bias	Assessor was not blinded.	Some concerns	No protocol available.	High risk of bias	Lack of blinding of treating therapists and outcome assessors.
**Subgroup 7.7.2 Neurophysiological (Bobath) compared to Neurophysiological (other)**												
Huang 2014	Some concerns	States randomly divided, but no description of randomisation process. The MBI scores were significantly higher than those before treatment (P<0.05), and the MBI scores of the four technical groups were significantly higher than those of the control group (P<0.05), but there was no significant difference in the FMA scores between the five groups after treatment (P<0.05). P> 0.05), there was no significant difference in MBI score among the four technical groups (P>0.05).	Some concerns	Insufficient information to judge if there were deviations from intended interventions.	Low risk of bias	No evidence of dropouts or missing data.	Some concerns	Blinding not discussed.	Some concerns	No reference to a protocol .	Some concerns	Some concerns due to lack of information and lack of protocol.
Huang 2014	Some concerns	States randomly divided, but no description of randomisation process. The MBI scores were significantly higher than those before treatment (P<0.05), and the MBI scores of the four technical groups were significantly higher than those of the control group (P<0.05), but there was no significant difference in the FMA scores between the five groups after treatment (P<0.05). P> 0.05), there was no significant difference in MBI score among the four technical groups (P>0.05).	Some concerns	Insufficient information to judge if there were deviations from intended interventions.	Low risk of bias	No evidence of dropouts or missing data.	Some concerns	Blinding not discussed.	Some concerns	No reference to a protocol .	Some concerns	Some concerns due to lack of information and lack of protocol.
**Subgroup 7.7.3 Neurophysiological compared to Functional Task Training**												
Arya 2019	Low risk of bias	"They were allocated in a 1:1 by a simple, nonstratified randomization to undergo experimental or control intervention. The randomization process was conducted using computer‐generated random numbers by a staff not concerned with the trial." "The groups did not significantly differ in any of the characteristics"	Low risk of bias	"The outcome evaluation was conducted by a trained occupational therapist, blinded to the allocated group of the subjects. The participants were also blinded for the intervention of interest"	Low risk of bias	Data available for 47/50 participants (3 lost to follow‐up)	Low risk of bias	Assessors were blinded.	Some concerns	No protocol obtained (although a protocol is mentioned in relation to ethics approvals).	Some concerns	Low risk of bias for all domains, with exception of bias in selection of the reported result, where we have not obtained the protocol.
Bui 2019	Some concerns	States "randomized" but no further details about randomisation or allocation concealment. "There were no statistically significant difference between the two groups in population traits, stroke histories and accompanying diseases"	Some concerns	Insufficient information to judge if there were deviations from intended interventions.	Low risk of bias	No evidence of any dropouts or missing data.	Some concerns	Not stated who performed outcome assessment and whether they were blinded.	Some concerns	No protocol available.	Some concerns	Some concerns due to lack of information and lack of protocol.
Guo L 2013	Some concerns	States "randomly divided" but randomisation process not discussed. "There was no significant difference in general clinical data such as gender, age, lesion nature, hemiplegia side, and course of disease between the two groups (P>0.05)"	Some concerns	Insufficient information to judge if there were deviations from intended intervention.	Low risk of bias	No evidence of any dropouts or missing data.	Some concerns	No information relating to blinding of outcome assessor.	Some concerns	No protocol available.	Some concerns	Some concerns due to lack of information and lack of protocol.
Kim 2016	Low risk of bias	"The randomization was performed using a sealed envelope technique." "After subjects passed the screening criteria, they provided their informed consent to participation in this study." "No statistically significant differences between the two groups were found at baseline"	Low risk of bias	Interventions were delivered in different settings (circuit group or individual), and it is considered unlikely that there were deviations from the intended interventions as a result of the study.	Low risk of bias	"Both types of treatment were well‐tolerated with an attendance rate of 100%, and all participants completed the study."	Low risk of bias	"The examiner was blinded as to whether the participants were in the experimental group or control group"	Some concerns	No protocol available.	Some concerns	Low risk of bias for all domains, but some concerns as no protocol available.
Langhammer 2000	Some concerns	Double‐blind randomisation (stratified according to sex and side of lesion) and sealed coding. "The study was double blind, and the code was sealed until the last test was performed at three months follow‐up" The Bobath group was slightly more dependent at entry, a finding that could explain a poorer outcome in this group.	Some concerns	Unclear whether the participant was blinded. Therapist was not blinded ("Information concerning the physiotherapy used was known only by the therapists who treated the patients and the secretary of the ward, who was in charge of the randomization") The same therapists provided treatment to participants in both treatment groups, creating the possibility of contamination between groups. Treatment following hospital discharge may not have been administered according to the randomisation process, potentially introducing performance bias to the postdischarge results.	Low risk of bias	29/33 in motor learning group and 24/28 in Bobath group completed intervention and had data available.	Low risk of bias	Assessor was blinded ("The tests were conducted by the project leader who had no information about which group the patient belonged to")	Some concerns	No protocol available.	Some concerns	Some concerns about baseline differences and potential for deviations from intended interventions; no protocol available.
Li 2005	Some concerns	Divided by "draw method" (not clear what this is).	Some concerns	Design and implementation of study conducted by first study author. This means there is a risk of contamination relating to beliefs etc of the study author	Low risk of bias	No drop outs or missing data described.	Low risk of bias	Outcome assessor blinded	Some concerns	No protocol available.	Some concerns	Lack of information provided, and concerns around lack of blinding study author/treating therapist.
Lincoln 2003	Low risk of bias	Computer‐generated random sequence of numbers in opaque sealed envelopes opened sequentially by researcher. ("Allocation to treatment groups was by a computer generated random sequence provided by a therapist not involved with the trial, with notification delivered in opaque, sealed envelopes. Blocked randomisation was used to ensure approximately equal numbers of patients in each group at any time. Patients were screened consecutively on admission to the ward and those that met the inclusion criteria were referred for initial assessment. After the initial assessment was completed, a research therapist opened the next envelope and informed the therapists providing the treatments of the group allocation") "The groups were not significantly different in age, gender, side of stroke, type of lesion, or cognitive impairments"	High risk of bias	Different groups of physiotherapists delivering each intervention. Some possibility of contamination between groups, as physiotherapists providing the motor learning intervention were previously using Bobath therapy and therefore may have reverted to using some Bobath techniques. Also some possibility of contamination due to participants being inpatients on the same unit: the study authors state: "some aspects of the treatments could not be implemented because both treatments were occurring on the same rehabilitation wards and there was a risk of treatment contamination" Both groups had received treatment based on the Bobath approach before randomisationThe Bobath treatment was provided by physiotherapists who had previously used it, while the motor learning treatment was provided by physiotherapists previously inexperienced in motor learning who were given training before the interventions.	Low risk of bias	Dropouts were accounted for. 52/60 in Bobath group and 47/60 in motor learning group remained at one month.	Low risk of bias	"Patients were asked not to mention their treatment or therapist to the assessor"	Some concerns	No protocol available.	High risk of bias	Concerns about potential bias due to deviations from intended interventions; no protocol available.
Richards 1993	Low risk of bias	Patients stratified then block randomization scheme used. Sealed envelopes, opened remotely by telephone request	Some concerns	Unclear whether participant was blinded Therapist not blinded The same two therapists provided treatment to both treatment groups, creating the possibility of contamination between groups.	Low risk of bias	23/27 completed intervention. Participants with missing data were dropped from analysis.	Low risk of bias	At no point were independent assessors aware of the group assignment	Some concerns	No protocol available.	Some concerns	Some concerns about potential deviations from intended interventions, and lack of protocol available.
Shuai 2013	Some concerns	States "randomly divided" but no description of how randomised. There was no difference in general data between the two groups of patients.	Some concerns	Insufficient information to support a judgement.	Low risk of bias	No evidence of any dropouts or missing data.	Some concerns	No information on blinding provided.	Some concerns	No reference to a protocol .	Some concerns	Some concerns due to lack of information and lack of protocol.

**Table:** Risk of bias for analysis 7.8 Neurophysiological approaches compared to other: Motor function scales

**Study**	**Bias**											
	**Randomisation process**		**Deviations from intended interventions**		**Missing outcome data**		**Measurement of the outcome**		**Selection of the reported results**		**Overall**	
	**Authors' judgement**	**Support for judgement**	**Authors' judgement**	**Support for judgement**	**Authors' judgement**	**Support for judgement**	**Authors' judgement**	**Support for judgement**	**Authors' judgement**	**Support for judgement**	**Authors' judgement**	**Support for judgement**
**Subgroup 7.8.1 Neurophysiological compared to no (or minimal) neurophysiological**												
Bale 2008	Some concerns	"Patients who volunteered and gave written informed consent were randomly allocated to two different training groups, either a functional strength training group or a training‐as‐usual group by drawing lots. From a total number of 20, 10 were allotted to each training group." No further information about allocation concealment. "At inclusion there were no statistical significant differences between the groups, neither in demo‐ graphic variables (Table 1) nor in physical perfor‐ mance measured"	Low risk of bias	"Different physiotherapists trained patients in the two intervention groups". Treatment group was strength training, whiile control group was Bobath (treatment as usual). No evidence of any deviations from intended interventions.	Low risk of bias	18 participants randomised; data available for all 18.	Low risk of bias	"Two physiotherapists performed the physical measurements, and were blinded to the patients’ group assignment. Before the study started, the testers were trained to perform the measurements based on a test protocol. "	Some concerns	No protocol available.	Some concerns	Some concerns due to lack of information relating to allocation concealment, and lack of protocol.
Fan WS 2006	Some concerns	Randomisation process not described.	Some concerns	Insufficient information to judge if there were deviations from intended interventions.	Low risk of bias	No dropouts.	Some concerns	No discussion around blinding.	Some concerns	No protocol identified.	Some concerns	Some concerns due to lack of information and lack of protocol.
Ge Y 2020	Some concerns	States "random number table method" but no further information. "There was no significant difference in age, gender and course of disease between the two groups ( 0.05). "	Some concerns	Insufficient information to judge if there were deviations from intended interventions.	Low risk of bias	No evidence of dropouts or missing data.	Some concerns	No information relating to blinding of outcome assessor.	Some concerns	No protocol obtained.	Some concerns	Some concerns due to lack of information and lack of protocol.
**Subgroup 7.8.2 Neurophysiological (Bobath) compared to neurophysiological (other)**												
Huang 2014	Some concerns	States randomly divided, but no description of randomisation process. The MBI scores were significantly higher than those before treatment (P<0.05), and the MBI scores of the four technical groups were significantly higher than those of the control group (P<0.05), but there was no significant difference in the FMA scores between the five groups after treatment (P<0.05). P> 0.05), there was no significant difference in MBI score among the four technical groups (P>0.05).	Some concerns	Insufficient information to judge if there were deviations from intended interventions.	Low risk of bias	No evidence of dropouts or missing data.	Some concerns	Blinding not discussed.	Some concerns	No reference to a protocol .	Some concerns	Some concerns due to lack of information and lack of protocol.
Huang 2014	Some concerns	States randomly divided, but no description of randomisation process. The MBI scores were significantly higher than those before treatment (P<0.05), and the MBI scores of the four technical groups were significantly higher than those of the control group (P<0.05), but there was no significant difference in the FMA scores between the five groups after treatment (P<0.05). P> 0.05), there was no significant difference in MBI score among the four technical groups (P>0.05).	Some concerns	Insufficient information to judge if there were deviations from intended interventions.	Low risk of bias	No evidence of dropouts or missing data.	Some concerns	Blinding not discussed.	Some concerns	No reference to a protocol .	Some concerns	Some concerns due to lack of information and lack of protocol.
**Subgroup 7.8.3 Neurophysiological compared to functional task training**												
Arya 2019	Low risk of bias	"They were allocated in a 1:1 by a simple, nonstratified randomization to undergo experimental or control intervention. The randomization process was conducted using computer‐generated random numbers by a staff not concerned with the trial." "The groups did not significantly differ in any of the characteristics"	Low risk of bias	"The outcome evaluation was conducted by a trained occupational therapist, blinded to the allocated group of the subjects. The participants were also blinded for the intervention of interest"	Low risk of bias	Data available for 47/50 participants (3 lost to follow‐up)	Low risk of bias	Assessors were blinded.	Some concerns	No protocol obtained (although a protocol is mentioned in relation to ethics approvals).	Some concerns	Low risk of bias for all domains, with exception of bias in selection of the reported result, where we have not obtained the protocol.
Guo L 2013	Some concerns	States "randomly divided" but randomisation process not discussed. "There was no significant difference in general clinical data such as gender, age, lesion nature, hemiplegia side, and course of disease between the two groups (P>0.05)"	Some concerns	Insufficient information to judge if there were deviations from intended intervention.	Low risk of bias	No evidence of any dropouts or missing data.	Some concerns	No information relating to blinding of outcome assessor.	Some concerns	No protocol available.	Some concerns	Some concerns due to lack of information and lack of protocol.
Kim 2016	Low risk of bias	"The randomization was performed using a sealed envelope technique." "After subjects passed the screening criteria, they provided their informed consent to participation in this study." "No statistically significant differences between the two groups were found at baseline"	Low risk of bias	Interventions were delivered in different settings (circuit group or individual), and it is considered unlikely that there were deviations from the intended interventions as a result of the study.	Low risk of bias	"Both types of treatment were well‐tolerated with an attendance rate of 100%, and all participants completed the study."	Low risk of bias	"The examiner was blinded as to whether the participants were in the experimental group or control group"	Some concerns	No protocol available.	Some concerns	Low risk of bias for all domains, but some concerns as no protocol available.
Langhammer 2000	Some concerns	Double‐blind randomisation (stratified according to sex and side of lesion) and sealed coding. "The study was double blind, and the code was sealed until the last test was performed at three months follow‐up" The Bobath group was slightly more dependent at entry, a finding that could explain a poorer outcome in this group.	High risk of bias	Unclear whether the participant was blinded. Therapist was not blinded ("Information concerning the physiotherapy used was known only by the therapists who treated the patients and the secretary of the ward, who was in charge of the randomization") The same therapists provided treatment to participants in both treatment groups, creating the possibility of contamination between groups. Treatment following hospital discharge may not have been administered according to the randomisation process, potentially introducing performance bias to the postdischarge results.	Low risk of bias	29/33 in motor learning group and 24/28 in Bobath group completed intervention and had data available.	Low risk of bias	Assessor was blinded ("The tests were conducted by the project leader who had no information about which group the patient belonged to")	Some concerns	No protocol available.	High risk of bias	There was potential for bias due to deviations from the intended interventions, and some concerns over the randomisation process and no protocol available.
Lincoln 2003	Low risk of bias	Computer‐generated random sequence of numbers in opaque sealed envelopes opened sequentially by researcher. ("Allocation to treatment groups was by a computer generated random sequence provided by a therapist not involved with the trial, with notification delivered in opaque, sealed envelopes. Blocked randomisation was used to ensure approximately equal numbers of patients in each group at any time. Patients were screened consecutively on admission to the ward and those that met the inclusion criteria were referred for initial assessment. After the initial assessment was completed, a research therapist opened the next envelope and informed the therapists providing the treatments of the group allocation") "The groups were not significantly different in age, gender, side of stroke, type of lesion, or cognitive impairments"	High risk of bias	Different groups of physiotherapists delivering each intervention. Some possibility of contamination between groups, as physiotherapists providing the motor learning intervention were previously using Bobath therapy and therefore may have reverted to using some Bobath techniques. Also some possibility of contamination due to participants being inpatients on the same unit: the study authors state: "some aspects of the treatments could not be implemented because both treatments were occurring on the same rehabilitation wards and there was a risk of treatment contamination" Both groups had received treatment based on the Bobath approach before randomisationThe Bobath treatment was provided by physiotherapists who had previously used it, while the motor learning treatment was provided by physiotherapists previously inexperienced in motor learning who were given training before the interventions.	Low risk of bias	Dropouts were accounted for. 52/60 in Bobath group and 47/60 in motor learning group remained at one month.	Low risk of bias	"Patients were asked not to mention their treatment or therapist to the assessor"	Some concerns	No protocol available.	High risk of bias	Concerns about potential bias due to deviations from intended interventions; no protocol available.
Richards 1993	Low risk of bias	Patients stratified then block randomization scheme used. Sealed envelopes, opened remotely by telephone request	Some concerns	Unclear whether participant was blinded Therapist not blinded The same two therapists provided treatment to both treatment groups, creating the possibility of contamination between groups.	Low risk of bias	23/27 completed intervention. Participants with missing data were dropped from analysis.	Low risk of bias	At no point were independent assessors aware of the group assignment	Some concerns	No protocol available.	Some concerns	Some concerns about potential deviations from intended interventions, and lack of protocol available.
Shuai 2013	Some concerns	States "randomly divided" but no description of how randomised. There was no difference in general data between the two groups of patients.	Some concerns	Insufficient information to support a judgement.	Low risk of bias	No evidence of any dropouts or missing data.	Some concerns	No information on blinding provided.	Some concerns	No reference to a protocol .	Some concerns	Some concerns due to lack of information and lack of protocol.
Wang 2005	Some concerns	"The random assignment was achieved by an independent person who chose one of the sealed envelopes 30 min before the start of the intervention." No further information about allocation concealment. "There were no differences between these two groups in age, side of hemiparesis, duration of hemiparesis and other general characteristics."	Some concerns	"Bobath group: Both therapists had been qualified for more than 10 years with at least five years of Bobath practice.Orthapaedic group: The two treating physical therapists had been qualified for more than 10 years with at least five years of orthopaedic practice on patients with stroke." This information implies that the groups were treated by different physiotherapists, but there is insufficient information to judge whether there could have been contamination between groups, with physiotherapists using 'techniques' meant for the other group.	Low risk of bias	In both groups, all randomised participants completed the treatment, and there were no missing data.	Low risk of bias	"Test results for each patient were assessed and evaluated by a separate physical therapist who was not involved in the treatment programme and did not know about the patient's group"	Some concerns	No protocol identified.	Some concerns	Some concerns due to lack of information about allocation concealment and potential deviations from intended deviations, and lack of protocol.
Wang Wenwei 2012	Some concerns	States randomised. Lack of information about allocation concealment. No differences between groups at baseline.	Some concerns	Insufficient information to judge if there were deviations from intended interventions because of trial context. Participants were analysed according to assigned intervention.	Low risk of bias	No drop outs or missing data reported.	High risk of bias	Not clear if outcome assessor was blinded. Insufficient information to judge if outcome could have been influenced by knowledge of intervention received.	Some concerns	No protocol obtained.	High risk of bias	This is a Chinese language trial, with sections translated by one member of our team. There is generally a lack of information about the study methods. It is not clear whether the outcome assessor was blinded.

**Table:** Risk of bias for analysis 7.9 Neurophysiological approaches compared to other: Balance (Berg Balance Scale)

**Study**	**Bias**											
	**Randomisation process**		**Deviations from intended interventions**		**Missing outcome data**		**Measurement of the outcome**		**Selection of the reported results**		**Overall**	
	**Authors' judgement**	**Support for judgement**	**Authors' judgement**	**Support for judgement**	**Authors' judgement**	**Support for judgement**	**Authors' judgement**	**Support for judgement**	**Authors' judgement**	**Support for judgement**	**Authors' judgement**	**Support for judgement**
**Subgroup 7.9.1 Neurophysiological compared to no (or minimal) neurophysiological**												
Brock 2005	Low risk of bias	"Randomization was done through a compu‐ ter‐generated, stratified, blocked randomization procedure. Patients were stratified according to time period from date of stroke to date of com‐ mencement in the trial. The two strata were four weeks to eight weeks post‐stroke at commence‐ ment of the trial and more than eight weeks post‐stroke. This stratification aimed to improve the likelihood of the two groups being similar in terms of initial severity and speed of recovery post‐stroke, as those with milder stroke and a quicker rate of recovery are likely to improve more rapidly during the time period of the study. Separate computer‐generated randomiza‐ tions were used for each site. Opaque envelopes were used to conceal group allocation. Participants were randomized and assigned to the intervention groups after the baseline assess‐ ments were carried out." "There were no significant dif‐ferences between groups at baseline for the six‐ minute walk test (P=0.79), gait velocity (P=0.27) and Berg Balance Scale (P=0.77)."	Some concerns	Insufficient information to judge whether there could have been deviations from the intended intervenion. Participants were analysed according to assigned intervention.	Some concerns	29 participants recruited. 26 participants completed study. 2 participants (both from treatment group) did not complete as they were discharged from hospital earlier than expected; 1 dropout due to illhealth. Both discharged patients were from the treatment group, potentially meaning that this missing data could depend on its true value. For outcomes for balance and gait velocity this is unlikely to be linked directly to hospital discharge.	Low risk of bias	"Measures were taken at baseline, and following treatment, by a physiotherapist who was blind to group assignment. "	Low risk of bias	Trial registered: actrnumber 12606000235505. Reported study and outcome are in accordance with the registered trial details.	Some concerns	Some concerns due to lack of informaton about delivered interventions and inbalance in drop outs between groups. Judged low risk of bias for other domains.
Haral 2014	Some concerns	States "randomly divided" but no details about randomisation. Baseline demographic details are not presented.	Some concerns	Possibly same physiotherapists provided intervention across both groups. Insufficient information to judge whether there could have been deviations from the intended interventions. Participants were analysed according to assigned intervention.	Low risk of bias	30 participants randomised. No drop outs or missing data reported.	High risk of bias	Not stated who conducted the outcome assessment or whether they were blinded. Insufficient information to judge whether an unblinded outcome assessor could have influenced the result.	Some concerns	No protocol identified.	High risk of bias	High risk of bias due to possibly lack of blinded outcome assessor. Some concerns due to lack of information relating to randomisation process and intervention delivery, and lack of protocol.
**Subgroup 7.9.2 Neurophysiological (Bobath) compared to neurophysiological (other)**												
Choi YK 2013	Some concerns	States "randomly assigned" but no further information provided. No baseline demographic data provided.	Some concerns	Participants either received "PNF combination patterns" with elastic kinesio tape, or "neurodevelopmental treatment". Insufficient information to judge whether there could have been deviations from the intended intervention. Participants were analysed according to assigned intervention.	Low risk of bias	30 participants randomised. No drop outs or missing data reported.	High risk of bias	Does not report who conducted the outcome assessments or whether they were blinded. Insufficient information to judge whether lack of blinding could have influenced the result.	Some concerns	No protocol identified.	High risk of bias	High risk of bias due to lack of blinded outcome assessor; some uncertainty due to lack of information on randomisation and intervention delivery, and lack of protocol.
**Subgroup 7.9.3 Neurophysiological compared to functional task training**												
Anandan 2020	Low risk of bias	Computer assisted lottery method is used to divide the participants into the groups equally Written consent sought prior to the start of the study. "Data collected were homogenous in distribution, while comparing the data for the pre interventions there was no statistical significance."	Some concerns	Insufficient information on who delivered treatments to enable judgement on whether there could have been deviations from the intended intervention. Participants analysed according to assigned group.	High risk of bias	37 participants randomised to each group. "After 4 weeks there was decline in the participants noted in both the groups. There were about 30 participants in each group. After 8 weeks of treatment the withdrawal of patients is further noted and it reduced to 25 in each group. So, this study finally analyzed and completed with 25 participants in each group" Reasons for dropouts are not stated, and information is vague ("about 30 participants in each group". Dropouts of 12/37 is a almost 30%. Insufficient information to be able to judge if missingness could depend on its true value. Number of dropouts is balanced between the groups.	High risk of bias	States "The initial values were evaluated by the separate assessor..." but it is not clear if this "separate assessor" assessed the outcomes. Insufficient information to judge if lack of a blinded outcome assessor could have influenced the result.	Some concerns	No protocol identified.	High risk of bias	Judged as high risk of bias due to having a 30% drop out rate and it being unclear whether there was a blinded outcome. Some uncertainty due to lack of information about intervention delivery and no protocol.
Duan 2011	Some concerns	States randomised. Lack of information about allocation concealment. No differences between groups at baseline.	Some concerns	Insufficient information to judge if there were deviations from intended interventions because of trial context. Participants were analysed according to assigned intervention.	Low risk of bias	No drop outs or missing data reported.	High risk of bias	Not clear if outcome assessor was blinded. Insufficient information to judge if outcome could have been influenced by knowledge of intervention received.	Some concerns	No protocol obtained.	High risk of bias	This is a Chinese language trial, with sections translated by one member of our team. There is generally a lack of information about the study methods. It is not clear whether the outcome assessor was blinded.
Kim 2016	Low risk of bias	"The randomization was performed using a sealed envelope technique." "After subjects passed the screening criteria, they provided their informed consent to participation in this study." "No statistically significant differences between the two groups were found at baseline"	Low risk of bias	Interventions were delivered in different settings (circuit group or individual), and it is considered unlikely that there were deviations from the intended interventions as a result of the study.	Low risk of bias	"Both types of treatment were well‐tolerated with an attendance rate of 100%, and all participants completed the study."	Low risk of bias	"The examiner was blinded as to whether the participants were in the experimental group or control group"	Some concerns	No protocol available.	Some concerns	Low risk of bias for all domains, but some concerns as no protocol available.
Richards 1993	Low risk of bias	Patients stratified then block randomization scheme used. Sealed envelopes, opened remotely by telephone request	Some concerns	Unclear whether participant was blinded Therapist not blinded The same two therapists provided treatment to both treatment groups, creating the possibility of contamination between groups.	Low risk of bias	23/27 completed intervention. Participants with missing data were dropped from analysis.	Low risk of bias	At no point were independent assessors aware of the group assignment	Low risk of bias	No protocol available.	Some concerns	Some concerns about potential deviations from intended interventions, and lack of protocol available.
Wang 2005	Some concerns	"The random assignment was achieved by an independent person who chose one of the sealed envelopes 30 min before the start of the intervention." No further information about allocation concealment. "There were no differences between these two groups in age, side of hemiparesis, duration of hemiparesis and other general characteristics."	Some concerns	"Bobath group: Both therapists had been qualified for more than 10 years with at least five years of Bobath practice.Orthapaedic group: The two treating physical therapists had been qualified for more than 10 years with at least five years of orthopaedic practice on patients with stroke." This information implies that the groups were treated by different physiotherapists, but there is insufficient information to judge whether there could have been contamination between groups, with physiotherapists using 'techniques' meant for the other group.	Low risk of bias	In both groups, all randomised participants completed the treatment, and there were no missing data.	Low risk of bias	"Test results for each patient were assessed and evaluated by a separate physical therapist who was not involved in the treatment programme and did not know about the patient's group"	Some concerns	No protocol identified.	Some concerns	Some concerns due to lack of information about allocation concealment and potential deviations from intended deviations, and lack of protocol.
Wang Wenwei 2012	Some concerns	States randomised. Lack of information about allocation concealment. No differences between groups at baseline.	Some concerns	Insufficient information to judge if there were deviations from intended interventions because of trial context. Participants were analysed according to assigned intervention.	Low risk of bias	No drop outs or missing data reported.	High risk of bias	Not clear if outcome assessor was blinded. Insufficient information to judge if outcome could have been influenced by knowledge of intervention received.	Some concerns	No protocol obtained.	High risk of bias	This is a Chinese language trial, with sections translated by one member of our team. There is generally a lack of information about the study methods. It is not clear whether the outcome assessor was blinded.

**Table:** Risk of bias for analysis 7.10 Neurophysiological approaches compared to other approaches: Gait velocity

**Study**	**Bias**											
	**Randomisation process**		**Deviations from intended interventions**		**Missing outcome data**		**Measurement of the outcome**		**Selection of the reported results**		**Overall**	
	**Authors' judgement**	**Support for judgement**	**Authors' judgement**	**Support for judgement**	**Authors' judgement**	**Support for judgement**	**Authors' judgement**	**Support for judgement**	**Authors' judgement**	**Support for judgement**	**Authors' judgement**	**Support for judgement**
**Subgroup 7.10.1 Neurophysiological compared to no (or minimal) neurophysiological**												
Bale 2008	Some concerns	"Patients who volunteered and gave written informed consent were randomly allocated to two different training groups, either a functional strength training group or a training‐as‐usual group by drawing lots. From a total number of 20, 10 were allotted to each training group." No further information about allocation concealment. "At inclusion there were no statistical significant differences between the groups, neither in demo‐ graphic variables (Table 1) nor in physical perfor‐ mance measured"	Low risk of bias	"Different physiotherapists trained patients in the two intervention groups". Treatment group was strength training, whiile control group was Bobath (treatment as usual). No evidence of any deviations from intended interventions.	Low risk of bias	18 participants randomised; data available for all 18.	Low risk of bias	"Two physiotherapists performed the physical measurements, and were blinded to the patients’ group assignment. Before the study started, the testers were trained to perform the measurements based on a test protocol. "	Some concerns	No protocol available.	Some concerns	Some concerns due to lack of information relating to allocation concealment, and lack of protocol.
Brock 2005	Low risk of bias	"Randomization was done through a compu‐ ter‐generated, stratified, blocked randomization procedure. Patients were stratified according to time period from date of stroke to date of com‐ mencement in the trial. The two strata were four weeks to eight weeks post‐stroke at commence‐ ment of the trial and more than eight weeks post‐stroke. This stratification aimed to improve the likelihood of the two groups being similar in terms of initial severity and speed of recovery post‐stroke, as those with milder stroke and a quicker rate of recovery are likely to improve more rapidly during the time period of the study. Separate computer‐generated randomiza‐ tions were used for each site. Opaque envelopes were used to conceal group allocation. Participants were randomized and assigned to the intervention groups after the baseline assess‐ ments were carried out." "There were no significant dif‐ferences between groups at baseline for the six‐ minute walk test (P=0.79), gait velocity (P=0.27) and Berg Balance Scale (P=0.77)."	Some concerns	Insufficient information to judge whether there could have been deviations from the intended intervenion. Participants were analysed according to assigned intervention.	Some concerns	29 participants recruited. 26 participants completed study. 2 participants (both from treatment group) did not complete as they were discharged from hospital earlier than expected; 1 dropout due to illhealth. Both discharged patients were from the treatment group, potentially meaning that this missing data could depend on its true value. For outcomes for balance and gait velocity this is unlikely to be linked directly to hospital discharge.	Low risk of bias	"Measures were taken at baseline, and following treatment, by a physiotherapist who was blind to group assignment. "	Low risk of bias	Trial registered: actrnumber 12606000235505. Reported study and outcome are in accordance with the registered trial details.	Some concerns	Some concerns due to lack of informaton about delivered interventions and inbalance in drop outs between groups. Judged low risk of bias for other domains.
Ge Y 2020	Some concerns	States "random number table method" but no further information. "There was no significant difference in age, gender and course of disease between the two groups ( 0.05). "	Some concerns	Insufficient information to judge if there were deviations from intended interventions.	Low risk of bias	No evidence of dropouts or missing data.	Some concerns	No information relating to blinding of outcome assessor.	Some concerns	No protocol obtained.	Some concerns	Some concerns due to lack of information and lack of protocol.
Gelber 1995	Some concerns	States patients were randomised, but no information as to how. "NDT and TFR treated patients did not differ with respect to age, gender, side of stroke or days from stroke to entry in the study"	High risk of bias	Unclear whether the participant was blinded. The same therapists provided treatment to participants in both treatment groups, creating a possibility of contamination between the groups.	Low risk of bias	Dropouts accounted for. 27/27 completed intervention, with data for this outcome.	High risk of bias	Assessor was not blinded.	Some concerns	No protocol available.	High risk of bias	Lack of blinding of treating therapists and outcome assessors.
Haral 2014	Some concerns	States "randomly divided" but no details about randomisation. Baseline demographic details are not presented.	Some concerns	Possibly same physiotherapists provided intervention across both groups. Insufficient information to judge whether there could have been deviations from the intended interventions. Participants were analysed according to assigned intervention.	Low risk of bias	30 participants randomised. No drop outs or missing data reported.	High risk of bias	Not stated who conducted the outcome assessment or whether they were blinded. Insufficient information to judge whether an unblinded outcome assessor could have influenced the result.	Some concerns	No protocol identified.	High risk of bias	High risk of bias due to possibly lack of blinded outcome assessor. Some concerns due to lack of information relating to randomisation process and intervention delivery, and lack of protocol.
Kuberan 2017	Some concerns	States 'simple randomization' but no further details provided. No significant differences at baseline.	Low risk of bias	The exercise schedule for the two groups is detailed. The prescriptive nature of each exercise schedule limits opportunities for deviations from the intended interventions. The particpants were analysed according to assigned intervention.	Low risk of bias	26 patients randomised. No drop outs or missing data.	Low risk of bias	Blinded assessor.	Some concerns	No protocol identified.	Some concerns	Some concerns due to lack of information about randomisation process, and lack of protocol.
Thaut 2007	Low risk of bias	"Treatment allocation was accomplished by computerized random number generators in both centers." "Random numbers for the allocation‐to‐treatment sequence were concealed from the recruiter and the therapists carrying out the training. Patients were informed of the 2 possible treatment allocations but blinded to the aims of an experimental versus control condition" "At pretest, there were no significant differences between the 2 groups in each parameter"	Low risk of bias	"Patients were informed of the 2 possible treatment allocations but blinded to the aims of an experimental versus control condition" "Therapists were not blinded to the treatment conditions of the study. However, because both conditions are considered full treatment conditions, no performance bias was expected". "RAS training followed established protocols..." limiting opportunities for deviations from intended interventions. Participants were analysed according to assigned intervention.	High risk of bias	"The dropout rate in one center was 23% of initially included patients. There was a 10% dropout rate in the other center. Dropout reasons were due to hospital transfer, early discharge, medical complication, or unspecified personal reasons." (Nearly 1/4 drop out rate which feels high) This is a relatively high drop out rate. There is a lack of information about which groups the drop outs occured from, and whether this was balanced between groups.	Low risk of bias	Blinded outcome assessor.	Some concerns	No protocol identified.	High risk of bias	Judged as high risk of bias due to a high drop out rate and lack of information relating to drop outs; some concerns due to lack of a protocol, but judged as low risk of bias for other domains.
**Subgroup 7.10.2 Neurophysiological (Bobath) compared to neurophysiological (other)**												
Choi YK 2013	Some concerns	States "randomly assigned" but no further information provided. No baseline demographic data provided.	Some concerns	Participants either received "PNF combination patterns" with elastic kinesio tape, or "neurodevelopmental treatment". Insufficient information to judge whether there could have been deviations from the intended intervention. Participants were analysed according to assigned intervention.	Low risk of bias	30 participants randomised. No drop outs or missing data reported.	High risk of bias	Does not report who conducted the outcome assessments or whether they were blinded. Insufficient information to judge whether lack of blinding could have influenced the result.	Some concerns	No protocol identified.	High risk of bias	High risk of bias due to lack of blinded outcome assessor; some uncertainty due to lack of information on randomisation and intervention delivery, and lack of protocol.
Kim 2018	Low risk of bias	"For the randomization process, sealed envelopes—each containing a piece of paper marked O or X—were prepared, and the process was carried out by a physical therapist who did not participate in the subject’s intervention or evaluation prior to the pretest." "There were no significant differences in gender, stroke type, paralysis side, age, date of onset, MMSE‐K, 10MWT, GAIT, and TUG between the two groups."	Some concerns	"Two physical therapists performed treatment to reduce treatment bias. One physical therapist with more than five years of clinical experience performed the CSE ex‐ercises. The TST training was conducted by the researcher who has more than 5 years of clinical experience." Although the interventions are described for each group, it is not clear whether there could have been deviations, such as the completion of functional task training by participants in the control group. Participants were analysed according to assigned interventions.	Low risk of bias	13 participants randomised. No drop outs or missing data reported.	High risk of bias	Does not stated who conducted assessments and whether they were blinded.	Some concerns	No protocol identified.	High risk of bias	High risk of bias due to possibility of unblinded outcome assessor. Some concerns due to lack of information relating to interventiond delivery and lack of protocol.
Kim 2021	Low risk of bias	Block randomization was determined using a randomization procedure in which each participant drew a ball from a box. Participants provided informed consent prior to participating were included in this study. No baseline differences.	Some concerns	Insufficient information to judge if there may have been deviations from the intended interventions. Participants were analysed according to their assigned intervention.	Low risk of bias	"During the 8‐week experimental period, two, three, and three participants dropped out from Experimental I (n = 13), Experimental II (n = 12), and the control group (n = 12), respectively, for a final total of 37 study participants." Drop outs were balanced between groups and reasons were not related to the study intervention.	Low risk of bias	"Evaluators blinded to group allocation performed the evaluations"	Low risk of bias	The trial was registered under trial registration no. KCT0006579. Reported study was as expected from protocol. Outcomes and analyses were pre‐stated in protocol.	Some concerns	Some concerns due to insufficient information to judge if there could be deviations from intended interventions; judged as low risk of bias for other domains.
**Subgroup 7.10.3 Neurophysiological compared to functional task training**												
Duan 2011	Some concerns	States randomised. Lack of information about allocation concealment. No differences between groups at baseline.	Some concerns	Insufficient information to judge if there were deviations from intended interventions because of trial context. Participants were analysed according to assigned intervention.	Low risk of bias	No drop outs or missing data reported.	High risk of bias	Not clear if outcome assessor was blinded. Insufficient information to judge if outcome could have been influenced by knowledge of intervention received.	Low risk of bias	No protocol obtained.	High risk of bias	This is a Chinese language trial, with sections translated by one member of our team. There is generally a lack of information about the study methods. It is not clear whether the outcome assessor was blinded.
Kim 2016	Low risk of bias	"The randomization was performed using a sealed envelope technique." "After subjects passed the screening criteria, they provided their informed consent to participation in this study." "No statistically significant differences between the two groups were found at baseline"	Low risk of bias	Interventions were delivered in different settings (circuit group or individual), and it is considered unlikely that there were deviations from the intended interventions as a result of the study.	Low risk of bias	"Both types of treatment were well‐tolerated with an attendance rate of 100%, and all participants completed the study."	Low risk of bias	"The examiner was blinded as to whether the participants were in the experimental group or control group"	Some concerns	No protocol available.	Some concerns	Low risk of bias for all domains, but some concerns as no protocol available.
Lincoln 2003	Low risk of bias	Computer‐generated random sequence of numbers in opaque sealed envelopes opened sequentially by researcher. ("Allocation to treatment groups was by a computer generated random sequence provided by a therapist not involved with the trial, with notification delivered in opaque, sealed envelopes. Blocked randomisation was used to ensure approximately equal numbers of patients in each group at any time. Patients were screened consecutively on admission to the ward and those that met the inclusion criteria were referred for initial assessment. After the initial assessment was completed, a research therapist opened the next envelope and informed the therapists providing the treatments of the group allocation") "The groups were not significantly different in age, gender, side of stroke, type of lesion, or cognitive impairments"	High risk of bias	Different groups of physiotherapists delivering each intervention. Some possibility of contamination between groups, as physiotherapists providing the motor learning intervention were previously using Bobath therapy and therefore may have reverted to using some Bobath techniques. Also some possibility of contamination due to participants being inpatients on the same unit: the study authors state: "some aspects of the treatments could not be implemented because both treatments were occurring on the same rehabilitation wards and there was a risk of treatment contamination" Both groups had received treatment based on the Bobath approach before randomisationThe Bobath treatment was provided by physiotherapists who had previously used it, while the motor learning treatment was provided by physiotherapists previously inexperienced in motor learning who were given training before the interventions.	Low risk of bias	Dropouts were accounted for. 52/60 in Bobath group and 47/60 in motor learning group remained at one month.	Low risk of bias	"Patients were asked not to mention their treatment or therapist to the assessor"	Some concerns	No protocol available.	High risk of bias	Concerns about potential bias due to deviations from intended interventions; no protocol available.
Richards 1993	Low risk of bias	Patients stratified then block randomization scheme used. Sealed envelopes, opened remotely by telephone request	Some concerns	Unclear whether participant was blinded Therapist not blinded The same two therapists provided treatment to both treatment groups, creating the possibility of contamination between groups.	Low risk of bias	23/27 completed intervention. Participants with missing data were dropped from analysis.	Low risk of bias	At no point were independent assessors aware of the group assignment	Some concerns	No protocol available.	Some concerns	Some concerns about potential deviations from intended interventions, and lack of protocol available.
Verma 2011	Low risk of bias	"After the blocks were numbered, a random‐number generator program was used to select numbers that established the sequence in which blocks were allocated to either one or the other group." "The subjects were blinded for intervention of interest." "The groups did not significantly differ in any of the demographic and baseline clinical characteristics"	Low risk of bias	"The subjects were blinded for intervention of interest." "The experimental and control interventions were given by 2 independent therapists (K.N.A. and T.V.)." This is judged to make deviations from the intended intervention unlikely. Participants analysed according to assigned intervention.	Low risk of bias	There was one patient lost to follow‐up, from the experimental group because of a second stroke).	Low risk of bias	"The present study was an assessor‐blinded (R.V.), randomized controlled design."	Some concerns	Reported that "the present study was registered in the Clinical Trial Registry of India as CTRI/2010/091/000606.". However we have been unable to access this protocol.	Some concerns	Judged as low risk of bias for all domains, except for selection of reported result where we were unable to access the reported trials registration.
Wang Wenwei 2012	Some concerns	States randomised. Lack of information about allocation concealment. No differences between groups at baseline.	Some concerns	Insufficient information to judge if there were deviations from intended interventions because of trial context. Participants were analysed according to assigned intervention.	Low risk of bias	No drop outs or missing data reported.	High risk of bias	Not clear if outcome assessor was blinded. Insufficient information to judge if outcome could have been influenced by knowledge of intervention received.	Some concerns	No protocol obtained.	High risk of bias	This is a Chinese language trial, with sections translated by one member of our team. There is generally a lack of information about the study methods. It is not clear whether the outcome assessor was blinded.

**Table:** Risk of bias for analysis 7.11 Neurophysiological approach compared to other: Adverse events

**Study**	**Bias**											
	**Randomisation process**		**Deviations from intended interventions**		**Missing outcome data**		**Measurement of the outcome**		**Selection of the reported results**		**Overall**	
	**Authors' judgement**	**Support for judgement**	**Authors' judgement**	**Support for judgement**	**Authors' judgement**	**Support for judgement**	**Authors' judgement**	**Support for judgement**	**Authors' judgement**	**Support for judgement**	**Authors' judgement**	**Support for judgement**
Epple 2020	Some concerns	"Patients were randomly assigned (1:1) with sealed, opaque and sequentially numbered envelopes to receive usual stroke unit care with Vojta therapy (intervention group) or standard physiotherapy (control group). Investigators were instructed to assign the patient according to the envelope with the lowest randomisation number. Randomisation was done by the investigator, who enrolled the subject." Unclear if allocation remained concealed until participants were enrolled. "Baseline characteristics did not differ statistically between the two groups, with the exception of the side of lesion, prior known orthopaedic conditions, and scores in TCT, CBS and NIHSS at baseline (Table 2), so that patients randomised to Vojta group were slightly more affected by the stroke."	Low risk of bias	Investigators and therapists were not blinded to the treatment allocation. Different physiotherapists provided the treatments to the two groups. The Vojta therapists "had received special training and certification". No Vojta‐therapist treated patients in the control group.	Low risk of bias	"One patient in the control group died at day 6, before reaching the primary outcome due to a malignant middle cerebral artery (MCA) infarction. Thirty‐nine patients (97.5%) were included in the ITT analysis. Two patients (one in each group) died after discharge from hospital before day 90, one due to pneumonia in the rehabilitation centre on day 65 (Vojta group) and one due to endocarditis on day 32 (control group). Thirty‐seven patients (92.5%) were included in the three‐months follow‐up assessment."	Low risk of bias	"Adverse events (AE) and serious adverse events (SAE)during the hospital stay and all deaths and SAE until day 90 were recorded and assessed by the investigators ac‐cording to standard definitions. All AE and SAE wereevaluated and forwarded to a medical expert for assess‐ment of relatedness to the study treatment."	Low risk of bias	Protocol described plans for assessment and reporting of adverse events.	Some concerns	Some concerns relating to allocation concealment, with randomisation conducted by the chief investigator.

**Table:** Risk of bias for analysis 8.1 Functional task training compared to other approaches: Independence in ADL scales

**Study**	**Bias**											
	**Randomisation process**		**Deviations from intended interventions**		**Missing outcome data**		**Measurement of the outcome**		**Selection of the reported results**		**Overall**	
	**Authors' judgement**	**Support for judgement**	**Authors' judgement**	**Support for judgement**	**Authors' judgement**	**Support for judgement**	**Authors' judgement**	**Support for judgement**	**Authors' judgement**	**Support for judgement**	**Authors' judgement**	**Support for judgement**
**Subgroup 8.1.1 Functional task training compared to 'less' functional task training**												
Chen G 2014	Some concerns	States "randomly divided" but no further information. There were no significant differences in age, gender, paralyzed side and NIHSS score on admission between the two groups (P>0.05), which were comparable.	Some concerns	Insufficient information to judge if deviations arose because of trial context.	Low risk of bias	No evidence of drop outs or missing data.	Some concerns	No discussion of blinding.	Some concerns	No protocol available.	Some concerns	Some concerns due to lack of information and lack of protocol.
Ding 2015	Some concerns	States "randomly divided" but no details around randomisation process. There was no significant difference in general data such as gender and age between the two groups (P>0.05), and they were comparable.	Some concerns	Insufficient information to judge if deviations may have occured because of the trial context.	Low risk of bias	No evidence of any drop outs or missing data.	Some concerns	No information on blinding provided.	Some concerns	No protocol available.	Some concerns	Some concerns due to lack of information and lack of protocol.
Langhammer 2007	High risk of bias	"Randomization was performed with a die: patients with uneven numbers went to group 1, an intensive exercise group, and those with even numbers to group 2, a regular exercise group. Stratification was according to gender and hemisphere lesion: the first male patient with a right hemisphere lesion and with an uneven number was allocated to the intensive exercise group, and the next male patient with a right hemisphere lesion was allocated to the regular exercise group. The procedure with the die was then used when the third male patient with a right hemisphere lesion entered the stroke unit and so on. A corresponding procedure was followed for female patients". "At discharge from the acute hospital, patients were randomized to one of two different groups by a person not involved with the patients or the treatment in the ward" "No significant differences between the groups regarding age, hemisphere lesion, marital status at baseline, or admission to the stroke unit". However, the regular exercise group had slightly higher baseline values for activities of daily living, motor function, balance and gait velocity, suggesting that the groups were not balanced.	High risk of bias	One fact that might be considered a weakness was that some therapists administered a submaximal programme to patients whom they had volunteered to exercise maximally. The reason for this was explained by the therapists involved as being a practical adaptation to pathological conditions such as heart failure, pain and a poor cognitive status, which inhibited a maximal effort. In order to carry out the exercises, adjustments were made so that routines could be maintained through the study period. This is probably also one of the reasons why so many patients complied with the exercise programmes.	Low risk of bias	8/75 dropped out during/end of intervention. Further 12 dropped out before follow up (12 months). Split evenly across groups.	Low risk of bias	"The study was an intention‐to‐treat trial with the aim of being double‐blind, that is, neither the investigator nor the participants knew to which group participants were allocated"	Some concerns	A protocol is referred to by the study authors, but this was not available.	High risk of bias	There was potential for bias due to deviations from the intended interventions, and some concerns over the randomisation process (which used a die) and no protocol available.
Lawal 2016	Low risk of bias	"Randomisation will be conducted using a computer‐generated random allocation sequence schedule held by a third party, who will randomly allocate recruited participants into the study group"	Low risk of bias	"All the activities for the control group were conducted by regular therapists (who are similar in qualification/experience to therapists implementing the CCT programme) in the Physiotherapy Department of AKTH." ‐ implies different therapists for the different groups, thus minimising potential for deviations.	Low risk of bias	"All participants completed the eight weeks therapy, with percentage range of drop‐out starting from a minimum of 7% in group D (control) to a maximum of 9% in groups A and B". Reasons for drop outs provided.	Low risk of bias	"To eliminate bias, the assessment of outcome will be performed by (experienced/trained) blinded assessors, who will be blinded to the nature/type of intervention as well as the intervention groups of the participants. Participants will also be instructed not to disclose their individual intervention groups to the assessors."	Low risk of bias	Published study protocol and trial registration. Final result presented as pre‐specified in protocol.	Low risk of bias	No concerns.
Li 2013	Some concerns	States "divided.....according to the random number table". No further information provided. Groups were clinically comparable at baseline.	Some concerns	Insufficient information to judge if there were potentially deviations due to trial context.	Low risk of bias	No evidence of any drop outs or missing data.	Some concerns	No discussion on blinding.	Some concerns	No protocol obtained.	Some concerns	Some concerns due to lack of information and lack of protocol.
Meng Qingling 2015	Some concerns	States "According to the random number table method, they were divided" but no further information. No significant difference in general data between the two groups (P>0.05).	Some concerns	Insufficient information to judge if there were deviations from intended interventions.	Low risk of bias	No evidence of dropouts or missing data.	Some concerns	Blinding not discussed.	Some concerns	No protocol available.	Some concerns	Some concerns due to lack of information and lack of protocol.
**Subgroup 8.1.2 Functional Task Training compared to Neurophysiological**												
Langhammer 2000	Some concerns	Double‐blind randomisation (stratified according to sex and side of lesion) and sealed coding. "The study was double blind, and the code was sealed until the last test was performed at three months follow‐up" The Bobath group was slightly more dependent at entry, a finding that could explain a poorer outcome in this group.	High risk of bias	Unclear whether the participant was blinded. Therapist was not blinded ("Information concerning the physiotherapy used was known only by the therapists who treated the patients and the secretary of the ward, who was in charge of the randomization") The same therapists provided treatment to participants in both treatment groups, creating the possibility of contamination between groups. Treatment following hospital discharge may not have been administered according to the randomisation process, potentially introducing performance bias to the postdischarge results.	Low risk of bias	29/33 in motor learning group and 24/28 in Bobath group completed intervention and had data available at 3 months	Some concerns	Assessor was blinded ("The tests were conducted by the project leader who had no information about which group the patient belonged to")	Some concerns	No protocol available.	High risk of bias	There was potential for bias due to deviations from the intended interventions, and some concerns over the randomisation process (which used a die) and no protocol available.
Verma 2011	Low risk of bias	"After the blocks were numbered, a random‐number generator program was used to select numbers that established the sequence in which blocks were allocated to either one or the other group." "The subjects were blinded for intervention of interest." "The groups did not significantly differ in any of the demographic and baseline clinical characteristics"	Low risk of bias	"The subjects were blinded for intervention of interest." "The experimental and control interventions were given by 2 independent therapists (K.N.A. and T.V.)." This is judged to make deviations from the intended intervention unlikely. Participants analysed according to assigned intervention.	Low risk of bias	There was one patient lost to follow‐up, from the experimental group because of a second stroke).	Low risk of bias	"The present study was an assessor‐blinded (R.V.), randomized controlled design."	Some concerns	Reported that "the present study was registered in the Clinical Trial Registry of India as CTRI/2010/091/000606.". However we have been unable to access this protocol.	Some concerns	Judged as low risk of bias for all domains, except for selection of reported result where we were unable to access the reported trials registration.

**Table:** Risk of bias for analysis 8.2 Functional task training compared to other approaches: Motor function scales

**Study**	**Bias**											
	**Randomisation process**		**Deviations from intended interventions**		**Missing outcome data**		**Measurement of the outcome**		**Selection of the reported results**		**Overall**	
	**Authors' judgement**	**Support for judgement**	**Authors' judgement**	**Support for judgement**	**Authors' judgement**	**Support for judgement**	**Authors' judgement**	**Support for judgement**	**Authors' judgement**	**Support for judgement**	**Authors' judgement**	**Support for judgement**
**Subgroup 8.2.1 Functional task training compared to 'less' functional task training**												
Aloraini 2022	Low risk of bias	"Participants were randomized into two groups using a computerized (block) randomization scheme. Pre‐stratification was done according to the participant’s pre‐morbid footedness and also the participant’s score on the main outcome measure (FMA‐LE)..." "Randomization was conducted following the consent and pre‐treatment assessments. Randomization was administered by an independent researcher who was not involved in the treatment or the assessment of participants." "Results of independent t‐tests for baseline measures showed that both groups were not significantly different at the outset of the study across all four outcome measure"	Some concerns	Insufficient information relating to delivery of interventions to judge if there could have been deviations from intended iinterventions.	Low risk of bias	"The number of participants who were randomized and allocated in their respective groups were 38 individuals. All the included individuals received the intervention program, attended the follow‐up session and were included in the final analysis of the study"	High risk of bias	Does not state that there was a blinded outcome assessor.	Some concerns	No reference to a protocol.	High risk of bias	Lack of blinded outcome assessor could introduce bias; some concerns due to lack of information about delivered interventions and lack of protocol.
Chen G 2014	Some concerns	States "randomly divided" but no further information. There were no significant differences in age, gender, paralyzed side and NIHSS score on admission between the two groups (P>0.05), which were comparable.	Some concerns	Insufficient information to judge if deviations arose because of trial context.	Low risk of bias	No evidence of drop outs or missing data.	Some concerns	No discussion of blinding	Some concerns	No protocol available.	Some concerns	Some concerns due to lack of information and lack of protocol.
Ding 2015	Some concerns	States "randomly divided" but no details around randomisation process. There was no significant difference in general data such as gender and age between the two groups (P>0.05), and they were comparable.	Some concerns	Insufficient information to judge if deviations may have occured because of the trial context.	Low risk of bias	No evidence of any drop outs or missing data.	Some concerns	No information on blinding provided.	Some concerns	No protocol available.	Some concerns	Some concerns due to lack of information and lack of protocol.
Kwakkel 2008	Low risk of bias	Participants were stratified by rehabilitation centre, and randomisation took place using an “online” minimisation procedure. Comparison between the observers’ guesses about allocation (circuit training or control) and actual allocation showed that 76 of 126 predictions were correct in the control group and 79 of 127 in the experimental group, resulting in a Cohen’s κ of 0.24. This suggests that the blinding procedure was successful. "We found significant baseline differences in favour of the circuit training group for a few secondary outcomes. All analyses, however, were adjusted for these covariates at baseline".	Low risk of bias	Interventions were delivered to participants living in the community; either a circuit training programme or usual outpatient physiotherapy. The outpatient physiotherapy was delivered by a "physiotherapist who had not been on the circuit training course at one of the participating rehabilitation centres."	Low risk of bias	"Of the 250 included patients, one patient in the circuit training group and seven in the usual care group were excluded from the analysis. Reasons were withdrawal from participation (n=3), death from cancer (n=2), and recurrent stroke (n=2), while one patient missed the 12 week assessment."	Low risk of bias	"Three trained research assistants (LW,HK,LK), who were blinded to treatment allocation, measured all outcomes"	Low risk of bias	Study protocol published, including a pre‐specified analysis plan.	Low risk of bias	No concerns ‐ study judged to be low risk of bias for all domains.
Langhammer 2007	High risk of bias	"Randomization was performed with a die: patients with uneven numbers went to group 1, an intensive exercise group, and those with even numbers to group 2, a regular exercise group. Stratification was according to gender and hemisphere lesion: the first male patient with a right hemisphere lesion and with an uneven number was allocated to the intensive exercise group, and the next male patient with a right hemisphere lesion was allocated to the regular exercise group. The procedure with the die was then used when the third male patient with a right hemisphere lesion entered the stroke unit and so on. A corresponding procedure was followed for female patients". "At discharge from the acute hospital, patients were randomized to one of two different groups by a person not involved with the patients or the treatment in the ward" "No significant differences between the groups regarding age, hemisphere lesion, marital status at baseline, or admission to the stroke unit". However, the regular exercise group had slightly higher baseline values for activities of daily living, motor function, balance and gait velocity, suggesting that the groups were not balanced.	High risk of bias	One fact that might be considered a weakness was that some therapists administered a submaximal programme to patients whom they had volunteered to exercise maximally. The reason for this was explained by the therapists involved as being a practical adaptation to pathological conditions such as heart failure, pain and a poor cognitive status, which inhibited a maximal effort. In order to carry out the exercises, adjustments were made so that routines could be maintained through the study period. This is probably also one of the reasons why so many patients complied with the exercise programmes.	Low risk of bias	8/75 dropped out; outcome data available for remaining participants.	Low risk of bias	"The study was an intention‐to‐treat trial with the aim of being double‐blind, that is, neither the investigator nor the participants knew to which group participants were allocated"	Some concerns	A protocol is referred to by the study authors, but this was not available.	High risk of bias	There was potential for bias due to deviations from the intended interventions, and some concerns over the randomisation process (which used a die) and no protocol available.
Li 2013	Some concerns	States "divided.....according to the random number table". No further information provided. Groups were clinically comparable at baseline.	Some concerns	Insufficient information to judge if there were potentially deviations due to trial context.	Low risk of bias	No evidence of any drop outs or missing data.	Some concerns	No discussion on blinding.	Some concerns	No protocol obtained.	Some concerns	Some concerns due to lack of information and lack of protocol.
Meng Qingling 2015	Some concerns	States "According to the random number table method, they were divided" but no further information. No significant difference in general data between the two groups (P>0.05).	Some concerns	Insufficient information to judge if there were deviations from intended interventions.	Low risk of bias	No evidence of dropouts or missing data.	Some concerns	Blinding not discussed.	Some concerns	No protocol available.	Some concerns	Some concerns due to lack of information and lack of protocol.
**Subgroup 8.2.2 Functional task training compared to neurophysiological**												
Langhammer 2000	Some concerns	Double‐blind randomisation (stratified according to sex and side of lesion) and sealed coding. "The study was double blind, and the code was sealed until the last test was performed at three months follow‐up" The Bobath group was slightly more dependent at entry, a finding that could explain a poorer outcome in this group.	High risk of bias	Unclear whether the participant was blinded. Therapist was not blinded ("Information concerning the physiotherapy used was known only by the therapists who treated the patients and the secretary of the ward, who was in charge of the randomization") The same therapists provided treatment to participants in both treatment groups, creating the possibility of contamination between groups. Treatment following hospital discharge may not have been administered according to the randomisation process, potentially introducing performance bias to the postdischarge results.	Low risk of bias	29/33 in motor learning group and 24/28 in Bobath group completed intervention and had data available.	Low risk of bias	Assessor was blinded ("The tests were conducted by the project leader who had no information about which group the patient belonged to")	Some concerns	No protocol available.	High risk of bias	There was potential for bias due to deviations from the intended interventions, and some concerns over the randomisation process and no protocol available.
Wang Wenwei 2012	Some concerns	States randomised. Lack of information about allocation concealment. No differences between groups at baseline.	Some concerns	Insufficient information to judge if there were deviations from intended interventions because of trial context. Participants were analysed according to assigned intervention.	Low risk of bias	No drop outs or missing data reported.	High risk of bias	Not clear if outcome assessor was blinded. Insufficient information to judge if outcome could have been influenced by knowledge of intervention received.	Some concerns	No protocol obtained.	High risk of bias	This is a Chinese language trial, with sections translated by one member of our team. There is generally a lack of information about the study methods. It is not clear whether the outcome assessor was blinded.

**Table:** Risk of bias for analysis 8.6 Neurophysiological approaches compared to other approaches: Independence in ADL scales

**Study**	**Bias**											
	**Randomisation process**		**Deviations from intended interventions**		**Missing outcome data**		**Measurement of the outcome**		**Selection of the reported results**		**Overall**	
	**Authors' judgement**	**Support for judgement**	**Authors' judgement**	**Support for judgement**	**Authors' judgement**	**Support for judgement**	**Authors' judgement**	**Support for judgement**	**Authors' judgement**	**Support for judgement**	**Authors' judgement**	**Support for judgement**
**Subgroup 8.6.1 Neurophysiological compared to functional task training**												
Langhammer 2000	Some concerns	Double‐blind randomisation (stratified according to sex and side of lesion) and sealed coding. "The study was double blind, and the code was sealed until the last test was performed at three months follow‐up" The Bobath group was slightly more dependent at entry, a finding that could explain a poorer outcome in this group.	Some concerns	Unclear whether the participant was blinded. Therapist was not blinded ("Information concerning the physiotherapy used was known only by the therapists who treated the patients and the secretary of the ward, who was in charge of the randomization") The same therapists provided treatment to participants in both treatment groups, creating the possibility of contamination between groups. Treatment following hospital discharge may not have been administered according to the randomisation process, potentially introducing performance bias to the postdischarge results.	Low risk of bias	29/33 in motor learning group and 24/28 in Bobath group completed intervention and had data available at 3 months.	Low risk of bias	Assessor was blinded ("The tests were conducted by the project leader who had no information about which group the patient belonged to")	Some concerns	No protocol available.	Some concerns	Some concerns about baseline differences and potential for deviations from intended interventions; no protocol available.
Verma 2011	Low risk of bias	"After the blocks were numbered, a random‐number generator program was used to select numbers that established the sequence in which blocks were allocated to either one or the other group." "The subjects were blinded for intervention of interest." "The groups did not significantly differ in any of the demographic and baseline clinical characteristics"	Low risk of bias	"The subjects were blinded for intervention of interest." "The experimental and control interventions were given by 2 independent therapists (K.N.A. and T.V.)." This is judged to make deviations from the intended intervention unlikely. Participants analysed according to assigned intervention.	Low risk of bias	There was one patient lost to follow‐up, from the experimental group because of a second stroke).	Low risk of bias	"The present study was an assessor‐blinded (R.V.), randomized controlled design."	Some concerns	Reported that "the present study was registered in the Clinical Trial Registry of India as CTRI/2010/091/000606.". However we have been unable to access this protocol.	Some concerns	Judged as low risk of bias for all domains, except for selection of reported result where we were unable to access the reported trials registration.
**Subgroup 8.6.2 Neurophysiological compared to no (or minimal) neurophysiological**												
Epple 2020	Some concerns	"Patients were randomly assigned (1:1) with sealed, opaque and sequentially numbered envelopes to receive usual stroke unit care with Vojta therapy (intervention group) or standard physiotherapy (control group). Investigators were instructed to assign the patient according to the envelope with the lowest randomisation number. Randomisation was done by the investigator, who enrolled the subject." Unclear if allocation remained concealed until participants were enrolled. "Baseline characteristics did not differ statistically between the two groups, with the exception of the side of lesion, prior known orthopaedic conditions, and scores in TCT, CBS and NIHSS at baseline (Table 2), so that patients randomised to Vojta group were slightly more affected by the stroke."	Low risk of bias	Investigators and therapists were not blinded to the treatment allocation. Different physiotherapists provided the treatments to the two groups. The Vojta therapists "had received special training and certification". No Vojta‐therapist treated patients in the control group.	Low risk of bias	"One patient in the control group died at day 6, before reaching the primary outcome due to a malignant middle cerebral artery (MCA) infarction. Thirty‐nine patients (97.5%) were included in the ITT analysis. Two patients (one in each group) died after discharge from hospital before day 90, one due to pneumonia in the rehabilitation centre on day 65 (Vojta group) and one due to endocarditis on day 32 (control group). Thirty‐seven patients (92.5%) were included in the three‐months follow‐up assessment."	Low risk of bias	Clinical assessment at day 90 was blinded	Low risk of bias	Protocol and trials registration available; study results are as pre‐specified in the protocol.	Some concerns	Some concerns relating to allocation concealment, with randomisation conducted by the chief investigator.
Gelber 1995	Some concerns	States patients were randomised, but no information as to how. "NDT and TFR treated patients did not differ with respect to age, gender, side of stroke or days from stroke to entry in the study"	High risk of bias	Unclear whether the participant was blinded. The same therapists provided treatment to participants in both treatment groups, creating a possibility of contamination between the groups.	Low risk of bias	Dropouts accounted for. 27/27 completed intervention, with data for this outcome.	High risk of bias	Assessor was not blinded.	Some concerns	No protocol available.	High risk of bias	Lack of blinding of treating therapists and outcome assessors.

**Table:** Risk of bias for analysis 8.7 Neurophysiological approaches compared to other approaches: Motor function scales

**Study**	**Bias**											
	**Randomisation process**		**Deviations from intended interventions**		**Missing outcome data**		**Measurement of the outcome**		**Selection of the reported results**		**Overall**	
	**Authors' judgement**	**Support for judgement**	**Authors' judgement**	**Support for judgement**	**Authors' judgement**	**Support for judgement**	**Authors' judgement**	**Support for judgement**	**Authors' judgement**	**Support for judgement**	**Authors' judgement**	**Support for judgement**
**Subgroup 8.7.1 Neurophysiological compared to functional task training**												
Langhammer 2000	Some concerns	Double‐blind randomisation (stratified according to sex and side of lesion) and sealed coding. "The study was double blind, and the code was sealed until the last test was performed at three months follow‐up" The Bobath group was slightly more dependent at entry, a finding that could explain a poorer outcome in this group.	High risk of bias	Unclear whether the participant was blinded. Therapist was not blinded ("Information concerning the physiotherapy used was known only by the therapists who treated the patients and the secretary of the ward, who was in charge of the randomization") The same therapists provided treatment to participants in both treatment groups, creating the possibility of contamination between groups. Treatment following hospital discharge may not have been administered according to the randomisation process, potentially introducing performance bias to the postdischarge results.	Low risk of bias	29/33 in motor learning group and 24/28 in Bobath group completed intervention and had data available.	Low risk of bias	Assessor was blinded ("The tests were conducted by the project leader who had no information about which group the patient belonged to")	Some concerns	No protocol available.	High risk of bias	There was potential for bias due to deviations from the intended interventions, and some concerns over the randomisation process and no protocol available.
Wang Wenwei 2012	Some concerns	States randomised. Lack of information about allocation concealment. No differences between groups at baseline.	Some concerns	Insufficient information to judge if there were deviations from intended interventions because of trial context. Participants were analysed according to assigned intervention.	Low risk of bias	No drop outs or missing data reported.	High risk of bias	Not clear if outcome assessor was blinded. Insufficient information to judge if outcome could have been influenced by knowledge of intervention received.	Some concerns	No protocol obtained.	High risk of bias	This is a Chinese language trial, with sections translated by one member of our team. There is generally a lack of information about the study methods. It is not clear whether the outcome assessor was blinded.

## Appendices

### Appendix 1. Stakeholder involvement in this review update

#### Aims of stakeholder involvement in this review

The pre‐stated aims of the stakeholder group were to (i) clarify the focus of the review (including how physical rehabilitation was categorised within the review), (ii) inform decisions about subgroup analyses, and (iii) co‐produce statements relating to key implications arising from the review. Members of the stakeholder group later clarified that their role was to:

- update and inform the description and categorisation of physical rehabilitation following stroke, and make it useful and accessible to all interested parties;
- ask the right questions in physical rehabilitation research, informing the structure and conduct of analyses and subgroup analyses within the Cochrane review of physical rehabilitation following stroke;
- consider implications for clinical practice arising from the results of the Cochrane review of physical rehabilitation following stroke;
- help shape plans for dissemination of the synthesised research evidence, so that it reaches – and is useful to ‐ the right people/organisations.

#### Description of stakeholder involvement in this review

This description of stakeholder involvement in this review is structured using the ACTIVE framework (Pollock 2019), with an additional question about 'what changed'.

**Who was involved?**

A stakeholder group was formed. Members included four stroke survivors, four carers, and seven physiotherapists working in stroke care. Stakeholders were from England (n = 7), Scotland (n = 6), Wales (n = 1), and Ireland (n = 1). One stroke survivor and 1 physiotherapist dropped out of the group during the course of the review update.

**How were stakeholders recruited?**

An advert for stakeholder group members was circulated through local and national networks, via email, and through social media. Interested people contacted the research team, who provided a role description (including details of all planned meeting dates) and requested some personal/demographic details. Responses to personal/demographic details were collated anonymously and used to select a 'representative' sample. Representation considered: geographical location, time since stroke (stroke survivors and carers), years of experience, and area of work (physiotherapists).

**What was the mode of involvement?**

Stakeholder group members attended a series of online meetings (using Microsoft Teams). Five meetings of the stakeholder group were held between 25 November 2021 and 25 May 2023, supplemented with additional communication by email and individual telephone calls. In addition, two international webinars were held in order to gain wider perspectives on decisions around categorisation of physical rehabilitation within the review.

**At what stage in the review process did involvement occur? What was the level of involvement at each stage?**

This was planned as a 'top and tail' approach, with involvement at the initial and final stages, but no involvement during the conduct of the review search or data collection itself (Pollock 2019). We use standard terms to define the level of involvement: see below for definitions.

Meetings 1 to 3 were held at the start of the review update with the aim of informing the methods/ways of bringing studies of physical rehabilitation together. Stakeholders considered they had a range of different levels of involvement, ranging from controlling to receiving (see definitions below). However, all stakeholders agreed that their input had *influenced* decisions around categorisation of physical rehabilitation interventions and conduct of analyses within the review.

Meetings 4 and 5 were held when the included studies were identified, and data extraction and (preliminary) analyses was complete. Again, stakeholders agreed that their input had *influenced* the review. Wording agreed by the stakeholders relating to the implications of the review results is presented within the review text. Stakeholders worked in partnership with the review team to write the plain language summary, sharing drafts and comments by email, *influencing* the final wording and content.

**What changed as a result of the stakeholder involvement?**

Key changes in the review arising from the involvement of stakeholders were:

(i) The previous method of categorisation of treatment components, comprising 27 defined treatment components, was revised and expanded. This revised framework for categorising physical rehabilitation treatment components is presented in Table. Further, discussions with the stakeholder group informed decisions to amend the comparisons addressed by this review. Previous versions of this review compared physical rehabilitation with (1) no treatment, (2) usual care or control and (3) another active physical rehabilitation intervention. Discussions highlighted that the comparison with usual care would benefit from further consideration, ensuring that the content of the usual care was explored in depth. Stakeholders considered that trials of (i) physical rehabilitation with no treatment should not be combined with trials of (ii) physical rehabilitation + "usual care" versus "usual care" when "usual care" comprised active physical rehabilitation. Stakeholders argued that the delivery of "usual" physical rehabilitation to both treatment groups could not be assumed to 'cancel out', as there could potentially be complex clinical interactions between "additional" physical rehabilitation and the "usual" physical rehabilitation. This was considered particularly important when the physical rehabilitation was based on different approaches to physical rehabilitation (which is the focus of this review). For example, if all participants were receiving physical rehabilitation based on neurophysiological approaches as the "usual" physical rehabilitation, and the intervention studied was functional task training, it was argued that there could potentially be an interaction between the neurophysiological physical rehabilitation and functional task training, which could impact on the benefit of the functional task training. Thus, the effect of functional task training on participants in a study comparing functional task training + neurophysiological physical rehabilitation with neurophysiological physical rehabilitation cannot be assumed to be the same as a study comparing functional task training (only with no physical rehabilitation).

This led to an amendment in the comparisons conducted within this update to comparisons (1) physical rehabilitation versus no (or minimal) physical rehabilitation (where minimal was defined as a dose of less than 50% of that of the active physical rehabilitation group), (2) physical rehabilitation versus attention control (where attention control was a non‐physical rehabilitation intervention, with an equivalent dose to that of the physical rehabilitation), (3) additional physical rehabilitation (i.e. a comparison of physical rehabilitation + usual rehabilitation versus usual rehabilitation), and (4) different physical rehabilitation approaches.

(ii) The stakeholder group highlighted the importance of mode of delivery (face‐to‐face or virtual sessions; and one‐to‐one or group rehabilitation) and whether rehabilitation was individualised or followed a standard/prescribed set of exercises or treatments. Following this input, we planned additional subgroup analyses to explore these intervention characteristics, but found we had insufficient data for these (due to either lack of reporting, or because a large majority of the included trials all had the same mode of delivery). In addition, the stakeholder group informed amendments to the subgroups explored in relation to dose of treatment. Previous subgroup analyses had used a pragmatic approach to division of trials into groups according to dose, but for this update ‐ informed by the stakeholder group ‐ we pre‐planned to explore the impact of doses which were less or more than 2.5 hours/week. This dose was considered important as stakeholders reported 30 minutes a day, 5 days/week as being a dose often delivered in clinical settings (a dose which falls short of UK guidance of 45 minutes/day). Stakeholder input also informed the decision to explore whether the involvement of family/carers might impact on outcomes.

(iii) Clarification of wording around key implications stated within this review (see below), and co‐production of the plain language summary. Wording was discussed with all stakeholders during meeting 4 and 5, and six provided comments on written drafts of the plain language summary, abstract, and implications for practice sections of the review.

**Definitions of levels of involvement** (from Pollock 2019)

"LEADING: Initiating the review; lead responsibility for carrying out and completion of review. Tasks will include authorship of a review, and may include any activities associated with review completion, including key decisions relating to the methods and execution of the review.

CONTROLLING: Working in partnership with researchers, with varying degrees of control or influence over the review process. Making decisions and/or controlling one or more aspects of the review process, in collaboration with or under the guidance of the review authors. Tasks may include defining outcomes of interest, inclusion criteria, key messages arising from review findings and writing a plain language summary. In completing tasks people have control over final decisions, such as application of inclusion criteria, categorisation of interventions, or recommendations for clinical practice.

INFLUENCING: Stating, commenting, advising, ranking, voting, prioritising, reaching consensus. Providing data or information which should directly influence the review process, but without direct control over decisions or aspects of the review process. Tasks may include assisting with review tasks, such as hand‐searching, screening, data extraction and assessment of risk of bias, possibly in a co‐reviewer role. Tasks may include peer review, such as commenting on a protocol, systematic review or plain language summary.

CONTRIBUTING: Providing views, thoughts, feedback, opinions or experiences. Providing data or information which may indirectly influence the review process. People may be participants in a research study (e.g. focus groups or interviews). Tasks may include sharing views or opinions, for example within a focus group of interview. May include ranking, voting or prioritising as participants in a research study (e.g. Delphi study).

RECEIVING: Receiving information about the systematic review, or results of the review. Tasks may include attending events, or reading or listening to information about the review. While the results of a review may be discussed, these discussions do not influence the review process in any way."

#### Stakeholder interpretation of evidence

Members of the stakeholder group for this review agreed that key implications for practice arising from this evidence are for personnel involved in planning and delivering stroke rehabilitation, and educating therapists, to recognise that:

- Where a stroke survivor has limitations in independence in ADL or motor function, in order to maximise that patient’s potential, as much physical rehabilitation as possible (taking into account an individual’s tolerance and preference) should be delivered; 2.5 hours/week should be considered a minimum amount, with recognition that the greater the amount the greater the potential benefit.
- Selection of physical rehabilitation treatment components should be based on an assessment of the individual stroke survivor, with consideration of the full range of treatment techniques that they have the skills and expertise to administer.
- As concluded in previous versions of this Cochrane review, physical rehabilitation should not be limited/aligned to compartmentalised, named rehabilitation approaches, but should comprise clearly defined, well‐described, evidence‐based physical treatments, focused on practice of functional tasks, regardless of historical or philosophical origin.

### Appendix 2. Physical rehabilitation approaches: historical overview and named approaches

#### Physical rehabilitation approaches: historical overview

Before the 1940s, physical rehabilitation approaches primarily consisted of corrective exercises based on orthopaedic principles related to contraction and relaxation of muscles, with emphasis placed on regaining function by compensating with the unaffected limbs (Ashburn 1995; Partridge 1996). In the 1950s and 1960s, techniques based on available neurophysiological knowledge were developed to enhance recovery of the paretic side. These new approaches included the methods of Bobath (Bobath 1990; Davies 1985), Brunnström (Brunnström 1970) and Rood (Goff 1969), as well as the proprioceptive neuromuscular facilitation approach (Knott 1968; Voss 1985). In the 1980s, the potential importance of neuropsychology and motor learning was highlighted (Anderson 1986; Turnbull 1982) and the motor learning, or relearning, approach was proposed (Carr 1982). This suggested that active practice of context‐specific motor tasks with appropriate feedback would promote learning and motor recovery (Carr 1980; Carr 1982; Carr 1987a; Carr 1987b; Carr 1989; Carr 1990; Carr 1998). The practical application of these approaches appeared to result in substantial differences in patient treatment. Approaches based on neurophysiological principles seemingly involved the physiotherapist moving the patient through patterns of movement, with the therapist acting as problem solver and decision maker and the patient being a relatively passive recipient (Lennon 1996). In direct contrast, motor learning approaches stressed the importance of active involvement by the patient (Carr 1982), and orthopaedic approaches emphasised muscle strengthening techniques and compensation with the non‐paretic side.

Since the 1980s, the need to base neurological physiotherapy on scientific research in relevant areas such as medical science, neuroscience, exercise physiology and biomechanics, and to test the outcomes of physical interventions to develop evidence‐based physiotherapy has been increasingly emphasised. However, anecdotal evidence and the results of questionnaire‐based studies (Carr 1994a; Davidson 2000; Lennon 2001; Nilsson 1992; Sackley 1996) suggest that, traditionally, many physiotherapists continued to base their clinical practice around a 'named' treatment approach. From the 1990s, the Bobath approach, based on neurophysiological principles, came to be recognised as the most widely used method in Sweden (Nilsson 1992), Australia (Carr 1994a) and the UK (Davidson 2000; Lennon 2001; Sackley 1996). As a consequence, since this time, physiotherapists have often sought evidence related to these 'named' approaches to the physical rehabilitation of stroke patients.

In some parts of the world, clear preferences for one 'named' approach have prevailed; however in others, physical rehabilitation approaches for stroke have developed with greater eclecticism, resulting in geographical preferences for mixing particular approaches, or components from different approaches, as well as preferences for single 'named' approaches (see below). For example, in China, where stroke rehabilitation is not yet considered standard care (Zhang 2013), standard 'approaches' to rehabilitation have been proposed, including 'standardised tertiary rehabilitation' (Hu 2007a; Research Group 2007; Zhang 2004) and 'standardised three‐phase rehabilitation' (Bai 2008; Fan WK 2006; Zhu 2004b). These approaches arguably appear to draw on the full range of treatment interventions available from all orthopaedic, neurophysiological and motor learning approaches described in Western literature, while incorporating traditional Chinese therapies such as acupuncture (Zhang 2013; Zhuang 2012).

At the time of the last update of this review, a growing research evidence base supported the use of clearly defined and described techniques and task‐specific treatments, regardless of their historical or philosophical origin (Kollen 2009; Langhammer 2012; Mayston 2000; Pomeroy 2005; van Peppen 2004), and a move away from named approaches in preference of more evidence‐based approaches had been deliberately implemented in some countries, such as the Netherlands (Kollen 2009; van Peppen 2004). Despite this, debate continued about the evidence for doing this (Carlisle 2010), and some physiotherapists around the world continued to exhibit preferences for particular named approaches (Khan 2012; Tyson 2009a; Tyson 2009b).

Since the publication of the last version of this review there has been a growing body of evidence which shows that the Bobath (or neurodevelopment) approach is not more effective, and may be less effective, than other interventions ( Díaz‐Arribas 2020; Pathak 2021; Scrivener 2020). However, concerns about the application of Bobath therapy within trials have been raised (Vaughan‐Graham 2015a; Vaughan‐Graham 2015b) and the use of named approaches remain common in current clinical practice around the world.

#### Physical rehabilitation approaches: overview of 'named' approaches

**Table:**

**Name of approach**	**Philosophy/theory**	**Treatment principles**	**Descriptive terms**	**Supporting references**
Rood	Concerned with 'the interaction of somatic, autonomic, and psychic factors, and their role in regulations of motor behaviour'. Motor and sensory functions inseparable Focuses on the developmental sequence of recovery and the use of peripheral input to facilitate movement	Activate/facilitate movement and postural responses of patient in same automatic way as they occur in the normal Sequencing of movement from basic to complex (supine lying; rolling; prone lying; kneeling; standing; walking) Sensory stimulation (brushing, icing, tapping, pounding, stroking, slow stretch, joint compression) to stimulate movement at automatic level	Ontogenetic sequences Developmental sequences Postural stability Normal patterns of movement Joint and cutaneous receptors Golgi tendon organs Abnormal tone	Goff 1969; Rood 1954; Stockmeyer 1967
Proprioceptive neuromuscular facilitation (PNF) or Knott and Voss	Active muscle contractions intended to stimulate afferent proprioceptive discharges into the CNS increased excitation and recruitment of additional motor units Assumes that central and peripheral stimulation are enhanced and facilitated in order to maximise the motor responses required Cortex controls patterns of movement not singular muscular actions Necessary to return to normal developmental sequence for recovery	Diagonal and spiral patterns of active and passive movement Quick stretch at end of range to promote contraction following relaxation in antagonists Maximal resistance is given by therapist to facilitate maximal activity in the range of the required movement. Voluntary contraction of the targeted muscle(s) Manual contact and therapist's tone of voice to encourage purposeful movement Isometric and isotonic contractions, traction and approximation of joint surfaces to stimulate postural reflexes	Patterns of movement Stretch and postural reflexes Manual pressure Isometric and isotonic contraction Approximation of joint surfaces Afferent input	Kabat 1953; Voss 1967
Brunnström	Uses primitive reflexes to initiate movement and encourages use of mass patterns in early stages of recovery Aims to encourage return of voluntary movement through use of reflex activity and sensory stimulation Assumes recovery progresses from subcortical to cortical control of muscle function Stages of recovery: flaccidity; elicit major synergies at reflex level; establish voluntary control of synergies; break away from flexor and extensor synergies by mixing components from antagonist synergies; more difficult movement combinations mastered; individual joint movements become possible; voluntary movement is elicited	Use tasks that patient can master or almost master. Sensory stimulation: from tonic neck or labyrinthine reflexes, or from stroking, tapping muscles	Normal development Sensory cues Synergies Primitive reflexes Tonic neck reflexes Associated reactions Movement patterns Mass patterns Tactile, proprioceptive, visual, auditory stimuli	Brunnström 1956; Brunnström 1961; Brunnström 1970; Perry 1967; Sawner 1992
Bobath or neurodevelopmental approach (NDT)	Aim to control afferent input and facilitate normal postural reactions Aim to give patients the experience of normal movement and afferent input while inhibiting abnormal movement and afferent input To improve quality of movement on affected side, so that the 2 sides work together harmoniously Assumption that increased tone and increased reflex activity will emerge as a result of lack of inhibition from a damaged postural reflex mechanism. Movement will be abnormal if comes from a background of abnormal tone Tone can be influenced by altering position or movement of proximal joints of the body	Facilitation of normal movement by a therapist, using direct handling of the body at key points such as head and spine, shoulders and pelvic girdle and, distally, feet and hands Volitional movement by patient is requested only against a background of automatic postural activity NB. Techniques of treatment have changed over time; more recently they have become more active and functionally orientated However, there is a lack of published material describing the current treatment principles of the Bobath approach More recently (October 2000) it has been emphasised that the concepts of the Bobath approach 'integrate with the main ideas of motor learning theory', and that advocated key treatment principles include active participation, practice and meaningful goals (Mayston 2000)	Normal movement Abnormal postural reflex activity/tone Postural control Key points Reflex inhibitory patterns	Bobath 1959; Bobath 1966; Bobath 1970; Bobath 1978; Bobath 1990; Davies 1985; Davies 1990; Mayston 2000
Johnstone (neurophysiological)	To control spasticity by inhibiting abnormal patterns and using positioning to influence tone Assumes that damaged postural reflex mechanism can be controlled through positioning and splinting Based on hierarchical model that assumes recovery is from proximal to distal Aim to achieve central stability, with gross motor performance, before progressing to more skilled movements Inflatable air splints: apply even, deep pressure to address sensory dysfunction	Use of inflatable splints Emphasis on correct position and use of splints Early stages: patient in side lying, with splint on affected arm Treatment progresses through hierarchy of activities, progressing from rolling through to crawling Family involvement encouraged	Muscle tone Air/pressure splints Positioning Reflex inhibition Tonic neck reflex Anti‐gravity patterns	Johnstone 1980; Johnstone 1989
Carr and Shepherd or motor learning or motor relearning or movement science (motor learning)	Assumes that neurologically impaired people learn in the same way as healthy people. Assumes that motor control of posture and movement are interrelated and that appropriate sensory input will help modulate the motor response to a task Patient is an active learner Uses biomechanical analysis of movement Training should be context‐specific Essential for motor learning: elimination of unnecessary muscle activity; feedback; practice Focus is on cognitive learning	(1) Analysis of task (2) Practice of missing components (3) Practice of task (4) Transference of training Biomechanical analysis with movements compared to the normal Instruction, explanation and feedback are essential parts of training Training involves practice with guidance from therapist: guidance may be manual (but is used for support or demonstration, not for providing sensory input) Identifiable and specific goals Appropriate environment	Motor control Motor relearning Feedback Practice Problem solving Training	Carr 1980; Carr 1982; Carr 1987a; Carr 1987b; Carr 1990; Carr 1998
Conductive education or Peto (motor learning)	Aims to teach patient strategies for dealing with disabilities in order to encourage them to learn to live with or overcome disabilities Integrated approach emphasising continuity and consistency Assumes that feelings of failure can produce a dysfunctional attitude, which can prevent rehabilitation Teaches strategies for coping with disability Active movements start with an intention and end with the goal Conductor assists patient to achieve movement control through task analysis and rhythmical intention or verbal reinforcement Emphasis on learning rather than receiving treatments	Educational principles and repetition used as a method of rote learning Highly structured day Group work Task analysis Repetition and reinforcement of task through rhythmical intention or verbal chanting Activities broken down into components or steps Patient encouraged to guide movements bilaterally	Education Rhythmical intention Intention Integrated system Group work Conductor Independence	Bower 1993; Cotton 1983; Kinsman 1988
Affolter (motor learning)	Interaction between individual and environment fundamental part of learning Perception seen as having an essential role in the cycle of learning Incoming information is compared with past experience ('assimilation'), which leads to anticipatory behaviour Assimilation and anticipation seen as basic for planning and for performance of complex movements Feedback is important to learning process	NB. This approach started from theory, rather than from clinical practice Starting at an elementary level, there will be no anticipation The patient starts to initiate more steps There is increased anticipation of the steps to be taken As experience increases, the patient will start to search for missing objects The patient is able plan more than 1 stage ahead and can perform new sequences if functional signals are familiar Not only can the patient think ahead but is able to check all the steps of the task in advance	Perception Assimilation Anticipation Complex human performance	Affolter 1980
Sensory integration or Ayres (motor learning)	Functional limitations compounded by sensory and perceptual impairment Sensory feedback and repetition seen as important principles of motor learning	Sensory feedback Repetition	Sensory and perceptual impairment Behavioural goals Feedback Repetition Adaptive response	Ayres 1972

### Appendix 3. CENTRAL search strategy

ID Search Hits

#1 MeSH descriptor: [Cerebrovascular Disorders] this term only

#2 MeSH descriptor: [Basal Ganglia Cerebrovascular Disease] explode all trees

#3 MeSH descriptor: [Brain Ischemia] explode all trees

#4 MeSH descriptor: [Brain Infarction] this term only

#5 MeSH descriptor: [Brain Stem Infarctions] this term only

#6 MeSH descriptor: [Cerebral Infarction] this term only

#7 MeSH descriptor: [Infarction, Anterior Cerebral Artery] this term only

#8 MeSH descriptor: [Infarction, Middle Cerebral Artery] this term only

#9 MeSH descriptor: [Infarction, Posterior Cerebral Artery] this term only

#10 MeSH descriptor: [Ischemic Attack, Transient] this term only

#11 MeSH descriptor: [Carotid Artery Diseases] this term only

#12 MeSH descriptor: [Carotid Artery Thrombosis] this term only

#13 MeSH descriptor: [Carotid Stenosis] this term only

#14 MeSH descriptor: [Cerebral Arterial Diseases] this term only

#15 MeSH descriptor: [Intracranial Arteriosclerosis] this term only

#16 MeSH descriptor: [Intracranial Arteriovenous Malformations] explode all trees

#17 MeSH descriptor: [Intracranial Embolism and Thrombosis] explode all trees

#18 MeSH descriptor: [Intracranial Hemorrhages] this term only

#19 MeSH descriptor: [Cerebral Hemorrhage] this term only

#20 MeSH descriptor: [Cerebral Intraventricular Hemorrhage] this term only

#21 MeSH descriptor: [Intracranial Hemorrhage, Hypertensive] this term only

#22 MeSH descriptor: [Subarachnoid Hemorrhage] this term only

#23 MeSH descriptor: [Stroke] this term only

#24 MeSH descriptor: [Hemorrhagic Stroke] this term only

#25 MeSH descriptor: [Ischemic Stroke] explode all trees

#26 MeSH descriptor: [Vasospasm, Intracranial] this term only

#27 (stroke or poststroke or post‐stroke or cerebrovasc* or (cerebr* near/3 vasc*) or CVA* or apoplectic or apoplex* or (transient near/3 isch?emic near/3 attack) or tia* or SAH or AVM or ESUS or ICH or (cerebral small vessel near/3 disease*)):ti,ab,kw

#28 ((cerebr* or cerebell* or arteriovenous or vertebrobasil* or interhemispheric or hemispher* or intracran* or intracerebral or infratentorial or supratentorial or MCA* or ((anterior or posterior) near/3 circulat*) or lenticulostriate or ((basilar or brachial or vertebr*) near/3 arter*)) near/3 (disease or damage* or disorder* or disturbance or dissection or syndrome or arrest or accident or lesion or vasculopathy or insult or attack or injury or insufficiency or malformation or obstruct* or anomal*)):ti,ab,kw

#29 ((cerebr* or cerebell* or arteriovenous or vertebrobasil* or interhemispheric or hemispher* or intracran* or corpus callosum or intracerebral or intracortical or intraventricular or periventricular or posterior fossa or infratentorial or supratentorial or MCA* or ((anterior or posterior) near/3 circulation) or basal ganglia or ((basilar or brachial or vertebr*) near/3 arter*) or space‐occupying or brain ventricle* or lacunar or cortical or ocular) near/3 (isch?emi* or infarct* or thrombo* or emboli* or occlus* or hypoxi* or vasospasm or obstruct* or vasoconstrict*)):ti,ab,kw

#30 ((cerebr* or cerebell* or vertebrobasil* or interhemispheric or hemispher* or intracran* or corpus callosum or intracerebral or intracortical or intraventricular or periventricular or posterior fossa or infratentorial or supratentorial or MCA* or ((anterior or posterior) near/3 circulation) or basal ganglia or ((basilar or brachial or vertebr*) near/3 arter*) or space‐occupying or brain ventricle* or subarachnoid* or arachnoid*) near/3 (h?emorrhag* or h?ematom* or bleed*)):ti,ab,kw

#31 ((carotid or cerebr* or cerebell* or intracranial or ((basilar or brachial or vertebr*) near/3 arter*)) near/3 (aneurysm or malformation* or block* or dysplasia or disease* or bruit or injur* or narrow* or obstruct* or occlusion or constriction or presclerosis or scleros* or stenos* or atherosclero* or arteriosclero* or plaque* or thrombo* or embol* or arteriopathy)):ti,ab,kw

#32 MeSH descriptor: [Hemiplegia] this term only

#33 MeSH descriptor: [Paresis] this term only

#34 MeSH descriptor: [Gait Disorders, Neurologic] explode all trees

#35 (hemipleg* or hemipar* or paresis or paraparesis or paretic):ti,ab,kw

#36 {or #1‐#35}

#37 MeSH descriptor: [Physical Therapy Modalities] this term only

#38 MeSH descriptor: [Exercise Movement Techniques] explode all trees

#39 MeSH descriptor: [Exercise Therapy] explode all trees

#40 MeSH descriptor: [Rehabilitation] this term only

#41 MeSH descriptor: [Feedback] this term only

#42 MeSH descriptor: [Feedback, Psychological] this term only

#43 MeSH descriptor: [Biofeedback, Psychology] explode all trees

#44 MeSH descriptor: [Exercise] this term only

#45 MeSH descriptor: [Orthopedic Procedures] this term only

#46 MeSH descriptor: [Physical Therapists] this term only

#47 MeSH descriptor: [Occupational Therapists] this term only

#48 (physiotherapy or physical therapy or exercise therapy or rehabilitation):ti,ab,kw

#49 (neurorehabilitation or feedback or biofeedback):ti,ab,kw

#50 (motor NEAR/5 (train* or re?train* or learn* or re?learn*)):ti,ab,kw

#51 (((neuromuscular or neurodevelopmental or neuro‐developmental) NEAR/2 (facilitation or trainin* or retrain$)) or PNF or NDT):ti,ab,kw

#52 (movement NEAR/5 (therap* or science)):ti,ab,kw

#53 (((neurodevelopmental or neurophysiological or orthop?edic) NEAR/5 (therap* or treatment* or rehabilitation or principle* or approach* or component* or concept*))):ti,ab,kw

#54 ((Bobath or Carr or Brunnstrom or Rood or Johnstone or NDT or (standardi?ed NEAR/2 three NEAR/2 stage*))):ti,ab,kw

#55 {or #37‐#54}

#56 #36 AND #55

#57 MeSH descriptor: [Cerebrovascular Disorders] this term only and with qualifier(s): [rehabilitation ‐ RH]

#58 MeSH descriptor: [Basal Ganglia Cerebrovascular Disease] explode all trees and with qualifier(s): [rehabilitation ‐ RH]

#59 MeSH descriptor: [Brain Ischemia] explode all trees and with qualifier(s): [rehabilitation ‐ RH]

#60 MeSH descriptor: [Carotid Artery Diseases] explode all trees and with qualifier(s): [rehabilitation ‐ RH]

#61 MeSH descriptor: [Intracranial Arterial Diseases] explode all trees and with qualifier(s): [rehabilitation ‐ RH]

#62 MeSH descriptor: [Intracranial Embolism and Thrombosis] explode all trees and with qualifier(s): [rehabilitation ‐ RH]

#63 MeSH descriptor: [Intracranial Hemorrhages] explode all trees and with qualifier(s): [rehabilitation ‐ RH]

#64 MeSH descriptor: [Brain Infarction] 4 tree(s) exploded and with qualifier(s): [rehabilitation ‐ RH] 47

#65 MeSH descriptor: [Vasospasm, Intracranial] this term only and with qualifier(s): [rehabilitation ‐ RH] 0

#66 MeSH descriptor: [Vertebral Artery Dissection] this term only and with qualifier(s): [rehabilitation ‐ RH] 0

#67 MeSH descriptor: [Hemiplegia] this term only and with qualifier(s): [rehabilitation ‐ RH]

#68 MeSH descriptor: [Paresis] this term only and with qualifier(s): [rehabilitation ‐ RH]

#69 {or #57‐#68}

#70 #56 or #69

#71 MeSH descriptor: [Psychomotor Performance] explode all trees

#72 MeSH descriptor: [Learning] this term only

#73 MeSH descriptor: [Conditioning, Psychological] this term only

#74 MeSH descriptor: [Problem‐Based Learning] this term only

#75 MeSH descriptor: [Problem Solving] this term only

#76 MeSH descriptor: [Movement] this term only

#77 MeSH descriptor: [Locomotion] this term only

#78 MeSH descriptor: [Walking] this term only

#79 MeSH descriptor: [Gait] this term only

#80 MeSH descriptor: [Motor Activity] this term only

#81 MeSH descriptor: [Range of Motion, Articular] this term only

#82 MeSH descriptor: [Postural Balance] this term only

#83 MeSH descriptor: [Posture] explode all trees

#84 MeSH descriptor: [Supination] this term only

#85 MeSH descriptor: [Pronation] this term only

#86 MeSH descriptor: [Weight‐Bearing] this term only

#87 MeSH descriptor: [Lower Extremity] explode all trees

#88 MeSH descriptor: [Back] explode all trees

#89 (learning or conditioning):ti,ab,kw

#90 (movement or gait or locomotion or walking or walk or mobility):ti,ab,kw

#91 (equilibrium or balance or postur* or supination or pronation):ti,ab,kw

#92 (body sway or stance or strength or weight?bearing or body weight support):ti,ab,kw

#93 (locomotor NEAR/5 (recovery or training)):ti,ab,kw

#94 (weight NEAR/5 (distribut* or transfer*)):ti,ab,kw

#95 (sit or sitting or stand or standing or step or stepping or climb or climbing):ti,ab,kw

#96 (lower limb* or lower extremit* or ankle or leg or heel or calf or knee or hip or thigh or foot or trunk):ti,ab,kw

#97 {or #71‐#97}

#98 #70 AND #97 in Trials

### Appendix 4. MEDLINE Ovid search strategy

The stroke and intervention subject searches have been linked to the Cochrane Highly Sensitive Search Strategy (CHSSS) for identifying randomised trials (RCTs) in MEDLINE: sensitivity maximising version (2008 revision) (lines 43‐52), as referenced in the Box 3.d in Lefebvre 2024.

1. cerebrovascular disorders/ or exp basal ganglia cerebrovascular disease/ or brain ischemia/ or brain infarction/ or brain stem infarctions/ or cerebral infarction/ or infarction, anterior cerebral artery/ or infarction, middle cerebral artery/ or infarction, posterior cerebral artery/ or ischemic attack, transient/ or carotid artery diseases/ or carotid artery thrombosis/ or carotid stenosis/ or cerebral arterial diseases/ or intracranial arteriosclerosis/ or exp intracranial arteriovenous malformations/ or exp "intracranial embolism and thrombosis"/ or intracranial hemorrhages/ or cerebral hemorrhage/ or cerebral intraventricular hemorrhage/ or intracranial hemorrhage, hypertensive/ or subarachnoid hemorrhage/ or stroke/ or hemorrhagic stroke/ or exp ischemic stroke/ or vasospasm, intracranial/  2. (stroke or poststroke or post‐stroke or cerebrovasc$ or (cerebr$ adj3 vasc$) or CVA$ or apoplectic or apoplex$ or (transient adj3 isch?emic adj3 attack) or tia$ or SAH or AVM or ESUS or ICH).tw.  3. ((cerebr$ or cerebell$ or arteriovenous or vertebrobasil$ or interhemispheric or hemispher$ or intracran$ or intracerebral or infratentorial or supratentorial or MCA$ or ((anterior or posterior) adj3 circulat$) or lenticulostriate or ((basilar or brachial or vertebr$) adj3 arter$)) adj3 ((blood adj5 clot$) or disease$ or damage$ or disorder$ or disturbance or dissection or lesion or syndrome or arrest or accident or lesion or vasculopathy or insult or attack or injury or insufficiency or malformation or obstruct$ or anomal$)).tw.  4. ((cerebr$ or cerebell$ or arteriovenous or vertebrobasil$ or interhemispheric or hemispher$ or intracran$ or corpus callosum or intracerebral or intracortical or intraventricular or periventricular or posterior fossa or infratentorial or supratentorial or MCA$ or ((anterior or posterior) adj3 circulation) or basal ganglia or ((basilar or brachial or vertebr$) adj3 arter$) or space‐occupying or brain ventricle$ or lacunar or cortical or ocular) adj3 (isch?emi$ or infarct$ or thrombo$ or emboli$ or occlus$ or hypoxi$ or vasospasm or obstruct$ or vasoconstrict$)).tw.  5. ((cerebr$ or cerebell$ or vertebrobasil$ or interhemispheric or hemispher$ or intracran$ or corpus callosum or intracerebral or intracortical or intraventricular or periventricular or posterior fossa or infratentorial or supratentorial or MCA$ or ((anterior or posterior) adj3 circulation) or basal ganglia or ((basilar or brachial or vertebr$) adj3 arter$) or space‐occupying or brain ventricle$ or subarachnoid$ or arachnoid$) adj3 (h?emorrhag$ or h?ematom$ or bleed$)).tw.  6. ((carotid or cerebr$ or cerebell$ or intracranial or ((basilar or brachial or vertebr$) adj3 arter$)) adj3 (aneurysm or malformation$ or block$ or dysplasia or disease$ or bruit or injur$ or narrow$ or obstruct$ or occlusion or constriction or presclerosis or scleros$ or stenos$ or atherosclero$ or arteriosclero$ or plaque$ or thrombo$ or embol$ or arteriopathy)).tw.  7. hemiplegia/ or paresis/ or exp gait disorders, neurologic/  8. (hemipleg$ or hemipar$ or paresis or paretic or hemineglect).tw.  9. or/1‐8  10. physical therapy modalities/ or exp exercise movement techniques/ or exp exercise therapy/ or rehabilitation/ or neurological rehabilitation/ or stroke rehabilitation/ or occupational therapy/ or occupational therapists/ or physical therapists/  11. feedback/ or feedback, physiological/ or exp biofeedback, psychology/  12. exercise/ or orthopedic procedures/  13. (physiotherap$ or ((physical or occupational or exercise) adj3 therap$) or rehabilitation).tw.  14. (neurorehabilitation or feedback or biofeedback).tw.  15. (motor adj5 (train$ or re?train$ or learn$ or re?learn$)).tw.  16. (((neuromuscular or neurodevelopmental or neuro‐developmental) adj2 (facilitation or trainin$ or retrain$)) or PNF or NDT).tw.  17. (movement adj5 (therap$ or science)).tw.  18. ((neurodevelopmental or neurophysiological or orthop?edic) adj5 (therap$ or treatment$ or rehabilitation or principle$ or approach$ or component$ or concept$)).tw.  19. (Bobath or Carr or Brunnstrom or Rood or Johnstone or NDT or (standardi?ed adj2 three adj2 stage$)).tw.  20. or/10‐19  21. 9 and 20  22. cerebrovascular disorders/rh or exp basal ganglia cerebrovascular disease/rh or brain ischemia/rh or brain infarction/rh or brain stem infarctions/rh or cerebral infarction/rh or infarction, anterior cerebral artery/rh or infarction, middle cerebral artery/rh or infarction, posterior cerebral artery/rh or ischemic attack, transient/rh or carotid artery diseases/rh or carotid artery thrombosis/rh or carotid stenosis/rh or cerebral arterial diseases/rh or intracranial arteriosclerosis/rh or exp intracranial arteriovenous malformations/rh or exp "intracranial embolism and thrombosis"/rh or intracranial hemorrhages/rh or cerebral hemorrhage/rh or cerebral intraventricular hemorrhage/rh or intracranial hemorrhage, hypertensive/rh or subarachnoid hemorrhage/rh or stroke/rh or hemorrhagic stroke/rh or exp ischemic stroke/rh or vasospasm, intracranial/rh  23. hemiplegia/rh or exp paresis/rh  24. neurological rehabilitation/ or stroke rehabilitation/  25. 21 or 22 or 23 or 24  26. psychomotor performance/ or motor skills/ or "task performance and analysis"/  27. learning/ or "conditioning (psychology)"/ or problem‐based learning/ or problem solving/  28. movement/ or locomotion/ or walking/ or dependent ambulation/ or gait/  29. motor activity/ or range of motion, articular/  30. postural balance/ or exp posture/ or supination/ or pronation/ or weight‐bearing/  31. exp lower extremity/ or exp back/  32. (motor adj5 (skill$ or activit$ or function$)).tw.  33. (learning or conditioning).tw.  34. (movement or gait or locomotion or walking or walk or mobility).tw.  35. (equilibrium or balance or postur$ or supination or pronation).tw.  36. (body sway or stance or strength or weight?bearing or body weight support).tw.  37. (locomotor adj5 (recovery or training)).tw.  38. (weight adj5 (distribut$ or transfer$)).tw.  39. (sit or sitting or stand or standing or step or stepping or climb or climbing).tw.  40. (lower limb$ or lower extremit$ or ankle or leg or heel or calf or knee or hip or thigh or foot or trunk).tw.  41. or/26‐40  42. 25 and 41  43. randomized controlled trial.pt.  44. controlled clinical trial.pt.  45. randomized.ab.  46. placebo.ab.  47. randomly.ab.  48. trial.ab.  49. groups.ab.  50. or/43‐49  51. exp animals/ not humans.sh.  52. 50 not 51  53. 42 and 52

### Appendix 5. Embase Ovid search strategy

The subject search strategies have been linked to an adapted version of the Cochrane Highly Sensitive Search Strategy for identifying controlled trials in Embase: (2018 revision); Ovid format (lines 47‐64).

1. cerebrovascular disease/ or exp cerebrovascular accident/ or exp cerebrovascular malformation/ or exp basal ganglion haemorrhage/ or exp brain hemorrhage/ or exp brain infarction/ or exp brain ischemia/ or cerebral artery disease/ or exp carotid artery disease/ or brain atherosclerosis/ or exp intracranial aneurysm/ or occlusive cerebrovascular disease/ or basilar artery obstruction/ or exp cerebral sinus thrombosis/ or middle cerebral artery occlusion/ or vertebral artery stenosis/ or ocular ischemic syndrome/ or vertebrobasilar insufficiency/ or exp carotid artery/ or carotid artery surgery/ or carotid endarterectomy/ or stroke patient/ or stroke unit/  2. (stroke or poststroke or post‐stroke or cerebrovasc$ or (cerebr$ adj3 vasc$) or CVA$ or apoplectic or apoplex$ or (transient adj3 isch?emic adj3 attack) or tia$ or SAH or AVM or (cerebral small vessel adj3 disease)).tw.  3. ((cerebr$ or cerebell$ or arteriovenous or vertebrobasil$ or interhemispheric or hemispher$ or intracran$ or intracerebral or infratentorial or supratentorial or MCA$ or ((anterior or posterior) adj3 circulat$) or lenticulostriate or ((basilar or brachial or vertebr$) adj3 arter$)) adj3 (disease or damage$ or disorder$ or disturbance or dissection or lesion or syndrome or arrest or accident or lesion or vasculopathy or insult or attack or injury or insufficiency or malformation or obstruct$ or anomal$)).tw.  4. ((cerebr$ or cerebell$ or vertebrobasil$ or interhemispheric or hemispher$ or intracran$ or corpus callosum or intracerebral or intracortical or intraventricular or periventricular or posterior fossa or infratentorial or supratentorial or MCA$ or ((anterior or posterior) adj3 circulation) or basal ganglia or ((basilar or brachial or vertebr$) adj3 arter$) or space‐occupying or brain ventricle$ or subarachnoid$ or arachnoid$) adj3 (h?emorrhage or h?ematoma or bleed$ or microh?emorrhage or microbleed or (encephalorrhagia or hematencephal$))).tw.  5. ((cerebr$ or cerebell$ or arteriovenous or vertebrobasil$ or interhemispheric or hemispher$ or intracran$ or corpus callosum or intracerebral or intracortical or intraventricular or periventricular or posterior fossa or infratentorial or supratentorial or MCA$ or ((anterior or posterior) adj3 circulation) or basal ganglia or ((basilar or brachial or vertebr$) adj3 arter$) or space‐occupying or brain ventricle$ or lacunar or cortical or ocular) adj3 (isch?emi$ or infarct$ or thrombo$ or emboli$ or occlus$ or hypoxi$ or vasospasm or obstruct$ or vasculopathy or vasoconstrict$)).tw.  6. ((carotid or cerebr$ or cerebell$ or intracranial or basilar or brachial or vertebr$) adj3 (aneurysm or malformation$ or dysplasia or disease or bruit or injur$ or obstruct$ or occlusion or constriction or presclerosis or scleros$ or stenos$ or atherosclero$ or arteriosclero$ or plaque$ or thrombo$ or embol$ or arteriopathy)).tw.  7. exp hemiplegia/ or exp paresis/ or neurologic gait disorder/  8. (hemipleg$ or hemipar$ or paresis or paraparesis or paretic).tw.  9. or/1‐8  10. rehabilitation/ or physical therapy modalities/ or physiotherapists/ or occupational therapy modalities/ or occupational therapy techniques/ or occupational therapists/ or rehabilitation modalities/ or rehabilitation techniques/ or physiotherapy/ or physiotherapist/ or functional training/ or muscle training/ or exp neurorehabilitation/ or occupational therapy/ or occupational therapist/ or rehabilitation care/ or vocational rehabilitation/  11. exp kinesiotherapy/ or exercise/ or stretching exercise/ or muscle exercise/ or exp orthopedic equipment/  12. feedback system/ or exp biofeedback/ or physiological feedback/ or psychological feedback/ or exp sensory feedback/  13. (physiotherap$ or ((physical or occupational or exercise) adj3 therap$) or rehabilitation).tw.  14. (neurorehabilitation or feedback or biofeedback).tw.  15. (motor adj5 (train$ or re?train$ or learn$ or re?learn$)).tw.  16. (((neuromuscular or neurodevelopmental or neuro‐developmental) adj2 (facilitation or trainin$ or retrain$)) or PNF or NDT).tw.  17. (movement adj5 (therap$ or science)).tw.  18. ((neurodevelopmental or neurophysiological or orthop?edic) adj5 (therap$ or treatment$ or rehabilitation or principle$ or approach$ or component$ or concept$)).tw.  19. (Bobath or Carr or Brunnstrom or Rood or Johnstone or NDT or (standardi?ed adj2 three adj2 stage$)).tw.  20. or/10‐19  21. 9 and 20  22. cerebrovascular disease/rh or basal ganglion hemorrhage/rh or cerebral artery disease/rh or cerebrovascular accident/rh or stroke/rh or exp carotid artery disease/rh or exp brain hematoma/rh or exp brain hemorrhage/rh or exp brain ischemia/rh or exp intracranial aneurysm/rh or exp occlusive cerebrovascular disease/rh  23. hemiparesis/rh or hemiplegia/rh or paresis/rh  24. neurological rehabilitation/ or stroke rehabilitation/  25. 21 or 22 or 23 or 24  26. physical performance/ or motor performance/ or object manipulation/  27. psychomotor performance/ or task performance/  28. learning/ or conditioning/ or problem based learning/  29. "movement (physiology)"/ or limb movement/ or locomotion/ or exp walking/ or leg movement/ or leg exercise/ or physical mobility/ or "range of motion"/  30. motor activity/ or psychomotor activity/  31. body equilibrium/ or body posture/ or sitting/ or standing/ or weight bearing/  32. exp leg/ or exp back/  33. (motor adj5 (skill$ or activit$ or function$)).tw.  34. (learning or conditioning).tw.  35. (movement or gait or locomotion or walking or walk or mobility).tw.  36. (equilibrium or balance or postur$ or supination or pronation).tw.  37. (body sway or stance or strength or weight?bearing or body weight support).tw.  38. (locomotor adj5 (recovery or training)).tw.  39. (weight adj5 (distribut$ or transfer$)).tw.  40. (sit or sitting or stand or standing or step or stepping or climb or climbing).tw.  41. (lower limb$ or lower extremit$ or ankle or leg or heel or calf or knee or hip or thigh or foot or trunk).tw.  42. or/26‐41  43. 25 and 42  44. Randomized Controlled Trial/ or "randomized controlled trial (topic)"/  45. Randomization/  46. Controlled clinical trial/ or "controlled clinical trial (topic)"/  47. control group/ or controlled study/  48. clinical trial/ or "clinical trial (topic)"/ or phase 1 clinical trial/ or phase 2 clinical trial/ or phase 3 clinical trial/ or phase 4 clinical trial/  49. crossover procedure/  50. single blind procedure/ or double blind procedure/ or triple blind procedure/  51. placebo/ or placebo effect/  52. (random$ or RCT or RCTs).tw.  53. (controlled adj5 (trial$ or stud$)).tw.  54. (clinical$ adj5 trial$).tw.  55. clinical trial registration.ab.  56. ((control or treatment or experiment$ or intervention) adj5 (group$ or subject$ or patient$)).tw.  57. (quasi‐random$ or quasi random$ or pseudo‐random$ or pseudo random$).tw.  58. ((control or experiment$ or conservative) adj5 (treatment or therapy or procedure or manage$)).tw.  59. ((singl$ or doubl$ or tripl$ or trebl$) adj5 (blind$ or mask$)).tw.  60. (cross‐over or cross over or crossover).tw.  61. (placebo$ or sham).tw.  62. trial.ti.  63. (assign$ or allocat$).tw.  64. controls.tw.  65. or/44‐64  66. (exp animal/ or animal.hw. or nonhuman/) not (exp human/ or human cell/ or (human or humans).ti.)  67. 65 not 66  68. 43 and 67

### Appendix 6. CINAHL EBSCO search strategy

The subject search lines (S1‐S9, S10‐S21, S22‐S24, and S27‐S42) has been link to the Search filters for identifying randomized trials in CINAHL Plus (S44‐S60) from the Cochrane Centralized Search Service (CSS) and was published in February 2019 (Glanville 2019 ).

S1 (MH "Cerebrovascular Disorders") OR (MH "Basal Ganglia Cerebrovascular Disease+") OR (MH "Carotid Artery Diseases") OR (MH "Carotid Artery Dissections") OR (MH "Carotid Artery Thrombosis") OR (MH "Carotid Stenosis") OR (MH "Cerebral Ischemia") OR (MH "Cerebral Ischemia, Transient") OR (MH "Hypoxia‐Ischemia, Brain") OR (MH "Cerebral Small Vessel Diseases") OR (MH "Cerebral Vasospasm") OR (MH "Cerebral Arterial Diseases") OR (MH "Cerebral Aneurysm") OR (MH "Intracranial Arteriosclerosis") OR (MH "Moyamoya Disease") OR (MH "Intracranial Embolism and Thrombosis+") OR (MH "Intracranial Hemorrhage") OR (MH "Subarachnoid Hemorrhage") OR (MH "Stroke+") OR (MH "Vertebral Artery Dissections")

S2 TI ( (stroke or poststroke or post‐stroke or cerebrovasc* or (cerebr* N3 vasc*) or CVA* or apoplectic or apoplex* or (transient N3 isch?emic N3 attack) or tia* or SAH or AVM or (cerebral small vessel N3 disease)) ) OR AB ( (stroke or poststroke or post‐stroke or cerebrovasc* or (cerebr* N3 vasc*) or CVA* or apoplectic or apoplex* or (transient N3 isch?emic N3 attack) or tia* or SAH or AVM or (cerebral small vessel N3 disease)) )

S3 TI ( ((cerebr* or cerebell* or arteriovenous or vertebrobasil* or interhemispheric or hemispher* or intracran* or intracerebral or infratentorial or supratentorial or MCA* or ((anterior or posterior) N3 circulat*) or lenticulostriate or ((basilar or brachial or vertebr*) N3 arter*)) N3 (disease or damage* or disorder* or disturbance or dissection or lesion or syndrome or arrest or accident or lesion or vasculopathy or insult or attack or injury or insufficiency or malformation or obstruct* or anomal*)) ) OR AB ( ((cerebr* or cerebell* or arteriovenous or vertebrobasil* or interhemispheric or hemispher* or intracran* or intracerebral or infratentorial or supratentorial or MCA* or ((anterior or posterior) N3 circulat*) or lenticulostriate or ((basilar or brachial or vertebr*) N3 arter*)) N3 (disease or damage* or disorder* or disturbance or dissection or lesion or syndrome or arrest or accident or lesion or vasculopathy or insult or attack or injury or insufficiency or malformation or obstruct* or anomal*)) )

S4 TI ( ((cerebr* or cerebell* or vertebrobasil* or interhemispheric or hemispher* or intracran* or corpus callosum or intracerebral or intracortical or intraventricular or periventricular or posterior fossa or infratentorial or supratentorial or MCA* or ((anterior or posterior) N3 circulation) or basal ganglia or ((basilar or brachial or vertebr*) N3 arter*) or space‐occupying or brain ventricle* or subarachnoid* or arachnoid*) N3 (h?emorrhage or h?ematoma or bleed* or microh?emorrhage or microbleed or (encephalorrhagia or hematencephal*))) ) OR AB ( ((cerebr* or cerebell* or vertebrobasil* or interhemispheric or hemispher* or intracran* or corpus callosum or intracerebral or intracortical or intraventricular or periventricular or posterior fossa or infratentorial or supratentorial or MCA* or ((anterior or posterior) N3 circulation) or basal ganglia or ((basilar or brachial or vertebr*) N3 arter*) or space‐occupying or brain ventricle* or subarachnoid* or arachnoid*) N3 (h?emorrhage or h?ematoma or bleed* or microh?emorrhage or microbleed or (encephalorrhagia or hematencephal*))) )

S5 TI ( ((cerebr* or cerebell* or arteriovenous or vertebrobasil* or interhemispheric or hemispher* or intracran* or corpus callosum or intracerebral or intracortical or intraventricular or periventricular or posterior fossa or infratentorial or supratentorial or MCA* or ((anterior or posterior) N3 circulation) or basal ganglia or ((basilar or brachial or vertebr*) N3 arter*) or space‐occupying or brain ventricle* or lacunar or cortical or ocular) N3 (isch?emi* or infarct* or thrombo* or emboli* or occlus* or hypoxi* or vasospasm or obstruct* or vasculopathy or vasoconstrict*)) ) OR AB ( ((cerebr* or cerebell* or arteriovenous or vertebrobasil* or interhemispheric or hemispher* or intracran* or corpus callosum or intracerebral or intracortical or intraventricular or periventricular or posterior fossa or infratentorial or supratentorial or MCA* or ((anterior or posterior) N3 circulation) or basal ganglia or ((basilar or brachial or vertebr*) N3 arter*) or space‐occupying or brain ventricle* or lacunar or cortical or ocular) N3 (isch?emi* or infarct* or thrombo* or emboli* or occlus* or hypoxi* or vasospasm or obstruct* or vasculopathy or vasoconstrict*)) )

S6 TI ( ((carotid or cerebr* or cerebell* or intracranial or basilar or brachial or vertebr*) N3 (aneurysm or malformation* or dysplasia or disease or bruit or injur* or obstruct* or occlusion or constriction or presclerosis or scleros* or stenos* or atherosclero* or arteriosclero* or plaque* or thrombo* or embol* or arteriopathy)) ) OR AB ( ((carotid or cerebr* or cerebell* or intracranial or basilar or brachial or vertebr*) N3 (aneurysm or malformation* or dysplasia or disease or bruit or injur* or obstruct* or occlusion or constriction or presclerosis or scleros* or stenos* or atherosclero* or arteriosclero* or plaque* or thrombo* or embol* or arteriopathy)) )

S7 (MH "Hemiplegia")

S8 TI (hemipleg* or hemipar* or paresis or paraparesis or paretic) OR AB (hemipleg* or hemipar* or paresis or paraparesis or paretic)

S9 S1 OR S2 OR S3 OR S4 OR S5 OR S6 OR S7 OR S8

S10 (MH "Physical Therapy") OR (MH "Rehabilitation") OR (MH "Home Rehabilitation+") OR (MH "Occupational Therapy") OR (MH "Therapeutic Exercise+") OR (MH "Neuromuscular Facilitation") OR (MH "Occupational Therapists") OR (MH "Occupational Therapy Assistants") OR (MH "Physical Therapist Assistants") OR (MH "Physical Therapists")

S11 (MH "Exercise+")

S12 (MH "Feedback") OR (MH "Biofeedback") OR (MH "Biofeedback (Iowa NIC)")

S13 (MH "Orthopedic Equipment and Supplies+")

S14 TI ( physiotherap* or ((physical or occupational or exercise) N3 therap*) or rehabilitation ) OR AB ( physiotherap* or ((physical or occupational or exercise) N3 therap*) or rehabilitation )

S15 TI ( neurorehabilitation or feedback or biofeedback ) OR AB ( neurorehabilitation or feedback or biofeedback )

S16 TI ( motor N5 (train* or re?train* or learn* or re?learn*) ) OR AB ( motor N5 (train* or re?train* or learn* or re?learn*) )

S17 TI (((neuromuscular or neurodevelopmental or neuro‐developmental) N2 (facilitation or trainin* or retrain*)) or PNF or NDT) OR AB (((neuromuscular or neurodevelopmental or neuro‐developmental) N2 (facilitation or trainin* or retrain*)) or PNF or NDT)

S18 TI ( movement N5 (therap* or science) ) OR AB ( movement N5 (therap* or science) )

S19 TI ((neurodevelopmental or neurophysiological or orthop?edic) N5 (therap* or treatment* or rehabilitation or principle* or approach* or component* or concept*)) OR AB ((neurodevelopmental or neurophysiological or orthop?edic) N5 (therap* or treatment* or rehabilitation or principle* or approach* or component* or concept*))

S20 TI ( Bobath or Carr or Brunnstrom or Rood or Johnstone or NDT or (standardi?ed N2 three N2 stage*) ) OR AB ( Bobath or Carr or Brunnstrom or Rood or Johnstone or NDT or (standardi?ed N2 three N2 stage*) )

S21 S10 OR S11 OR S12 OR S13 OR S14 OR S15 OR S16 OR S17 OR S18 OR S19 OR S20

S22 S9 AND S21

S23 MH "Cerebrovascular Disorders+/RH"

S24 MH "Hemiplegia/RH"

S25 MH "Gait Disorders, Neurologic/RH"

S26 S22 OR S23 OR S24 OR S25

S27 (MH "Psychomotor Performance") OR (MH "Motor Skills+") OR (MH "Task Performance and Analysis")

S28 (MH "Learning") OR (MH "Conditioning (Psychology)") OR (MH "Problem Solving") OR (MH "Skill Retention")

S29 (MH "Movement") OR (MH "Locomotion") OR (MH "Walking") OR (MH "Gait+") OR (MH "Pronation") OR (MH "Range of Motion") OR (MH "Rising") OR (MH "Sitting") OR (MH "Squatting") OR (MH "Stair Climbing") OR (MH "Standing+") OR (MH "Supination") OR (MH "Weight‐Bearing") OR (MH "Weight Shifting")

S30 (MH "Motor Activity")

S31 (MH "Balance, Postural")

S32 (MH "Back") OR (MH "Torso") OR (MH "Lower Extremity+")

S33 TI ( motor N5 (skill* or activit* or function*) ) OR AB ( motor N5 (skill* or activit* or function*) )

S34 TI ( learning or conditioning ) OR AB ( learning or conditioning )

S35 TI ( movement or gait or locomotion or walking or walk or mobility ) OR AB ( movement or gait or locomotion or walking or walk or mobility )

S36 TI ( equilibrium or balance or postur* or supination or pronation ) OR AB ( equilibrium or balance or postur* or supination or pronation )

S37 TI ( body sway or stance or strength or weight#bearing or body weight support ) OR AB ( body sway or stance or strength or weight#bearing or body weight support )

S38 TI ( locomotor N5 (recovery or training) ) OR AB ( locomotor N5 (recovery or training) )

S39 TI ( weight N5 (distribut* or transfer*) ) OR AB ( weight N5 (distribut* or transfer*) )

S40 TI ( sit or sitting or stand or standing or step or stepping or climb or climbing ) OR AB ( sit or sitting or stand or standing or step or stepping or climb or climbing )

S41 TI ( lower limb* or lower extremit* or ankle or leg or heel or calf or knee or hip or thigh or foot or trunk ) OR AB ( lower limb* or lower extremit* or ankle or leg or heel or calf or knee or hip or thigh or foot or trunk )

S42 S27 OR S28 OR S29 OR S30 OR S31 OR S32 OR S33 OR S34 OR S35 OR S36 OR S37 OR S38 OR S39 OR S40 OR S41

S43 S26 AND S42

S44 MH randomized controlled trials

S45 MH double‐blind studies

S46 MH single‐blind studies

S47 MH random assignment

S48 MH pretest‐posttest design

S49 MH cluster sample

S50 TI (randomised OR randomized)

S51 AB (random*)

S52 TI (trial)

S53 MH (sample size) AND AB (assigned OR allocated OR control)

S54 MH (placebos)

S55 PT (randomized controlled trial)

S56 AB (control W5 group)

S57 MH (crossover design) OR MH (comparative studies)

S58 AB (cluster W3 RCT)

S59 ( (MH animals+ OR MH (animal studies) OR TI (animal model*)) ) NOT MH (human)

S60 S44 OR S45 OR S46 OR S47 OR S48 OR S49 OR S50 OR S51 OR S52 OR S53 OR S54 OR S55 OR S56 OR S57 OR S58

S61 S60 NOT S59

### Appendix 7. AMED Ovid search strategy

1. cerebrovascular disorders/ or cerebral hemorrhage/ or cerebral infarction/ or cerebral ischemia/ or cerebrovascular accident/ or stroke/  2. (stroke or poststroke or post‐stroke or cerebrovasc$ or (cerebr$ adj3 vasc$) or CVA$ or apoplectic or apoplex$ or (transient adj3 isch?emic adj3 attack) or tia$ or SAH or AVM or ESUS or ICH).tw.  3. ((cerebr$ or cerebell$ or arteriovenous or vertebrobasil$ or interhemispheric or hemispher$ or intracran$ or intracerebral or infratentorial or supratentorial or MCA$ or ((anterior or posterior) adj3 circulat$) or lenticulostriate or ((basilar or brachial or vertebr$) adj3 arter$)) adj3 ((blood adj5 clot$) or disease$ or damage$ or disorder$ or disturbance or dissection or lesion or syndrome or arrest or accident or lesion or vasculopathy or insult or attack or injury or insufficiency or malformation or obstruct$ or anomal$)).tw.  4. ((cerebr$ or cerebell$ or arteriovenous or vertebrobasil$ or interhemispheric or hemispher$ or intracran$ or corpus callosum or intracerebral or intracortical or intraventricular or periventricular or posterior fossa or infratentorial or supratentorial or MCA$ or ((anterior or posterior) adj3 circulation) or basal ganglia or ((basilar or brachial or vertebr$) adj3 arter$) or space‐occupying or brain ventricle$ or lacunar or cortical or ocular) adj3 (isch?emi$ or infarct$ or thrombo$ or emboli$ or occlus$ or hypoxi$ or vasospasm or obstruct$ or vasoconstrict$)).tw.  5. ((cerebr$ or cerebell$ or vertebrobasil$ or interhemispheric or hemispher$ or intracran$ or corpus callosum or intracerebral or intracortical or intraventricular or periventricular or posterior fossa or infratentorial or supratentorial or MCA$ or ((anterior or posterior) adj3 circulation) or basal ganglia or ((basilar or brachial or vertebr$) adj3 arter$) or space‐occupying or brain ventricle$ or subarachnoid$ or arachnoid$) adj3 (h?emorrhag$ or h?ematom$ or bleed$)).tw.  6. ((carotid or cerebr$ or cerebell$ or intracranial or ((basilar or brachial or vertebr$) adj3 arter$)) adj3 (aneurysm or malformation$ or block$ or dysplasia or disease$ or bruit or injur$ or narrow$ or obstruct$ or occlusion or constriction or presclerosis or scleros$ or stenos$ or atherosclero$ or arteriosclero$ or plaque$ or thrombo$ or embol$ or arteriopathy)).tw.  7. hemiplegia/  8. (hemipleg$ or hemipar$ or paresis or paretic or hemineglect).tw.  9. or/1‐8  10. physical therapy modalities/ or physiotherapists/ or occupational therapy modalities/ or occupational therapy techniques/ or occupational therapists/ or rehabilitation/ or rehabilitation modalities/ or rehabilitation techniques/  11. exp exercise therapy/ or exercise/ or muscle stretching exercises/ or exp orthopedic equipment/  12. feedback/ or biofeedback/  13. exp neurodevelopmental therapy/  14. (physiotherap$ or ((physical or occupational or exercise) adj3 therap$) or rehabilitation).tw.  15. (neurorehabilitation or feedback or biofeedback).tw.  16. (motor adj5 (train$ or re?train$ or learn$ or re?learn$)).tw.  17. (((neuromuscular or neurodevelopmental or neuro‐developmental) adj2 (facilitation or trainin$ or retrain$)) or PNF or NDT).tw.  18. (movement adj5 (therap$ or science)).tw.  19. ((neurodevelopmental or neurophysiological or orthop?edic) adj5 (therap$ or treatment$ or rehabilitation or principle$ or approach$ or component$ or concept$)).tw.  20. (Bobath or Carr or Brunnstrom or Rood or Johnstone or NDT or (standardi?ed adj2 three adj2 stage$)).tw.  21. or/10‐20  22. 9 and 21  23. psychomotor performance/ or exp motor skills/ or "task performance and analysis"/  24. learning/ or conditioning/ or problem solving/  25. balance/ or movement/ or exp gait/ or locomotion/ or walking/ or dependent ambulation/ or motor activity/ or pronation/ or "range of motion"/ or exp posture/ or sitting/ or weight bearing/  26. exp back/ or exp leg/  27. (motor adj5 (skill$ or activit$ or function$)).tw.  28. (learning or conditioning).tw.  29. (movement or gait or locomotion or walking or walk or mobility).tw.  30. (equilibrium or balance or postur$ or supination or pronation).tw.  31. (body sway or stance or strength or weight?bearing or body weight support).tw.  32. (locomotor adj5 (recovery or training)).tw.  33. (weight adj5 (distribut$ or transfer$)).tw.  34. (sit or sitting or stand or standing or step or stepping or climb or climbing).tw.  35. (lower limb$ or lower extremit$ or ankle or leg or heel or calf or knee or hip or thigh or foot or trunk).tw.  36. or/23‐35  37. 22 and 36  38. research design/  39. clinical trials/  40. randomized controlled trials/  41. comparative study/  42. double blind method/  43. meta analysis/  44. random allocation/  45. program evaluation/  46. placebos/  47. (clinical trial or clinical trial phase iii or meta analysis or clinical trialb or clinical trials or multicenter study or multicentre study or comparative studies or comparative study or randomised controlled trial or randomized controlled trial or controlled clinical trial or controlled trial).pt.  48. random$.tw.  49. (controlled adj5 (trial$ or stud$)).tw.  50. (clinical$ adj5 trial$).tw.  51. ((control or treatment or experiment$ or intervention) adj5 (group$ or subject$ or patient$)).tw.  52. (quasi‐random$ or quasi random$ or pseudo‐random$ or pseudo random$).tw.  53. ((control or experiment$ or conservative) adj5 (treatment or therapy or procedure or manage$)).tw.  54. ((singl$ or doubl$ or tripl$ or trebl$) adj5 (blind$ or mask$)).tw.  55. (cross‐over or cross over or crossover).tw.  56. placebo$.tw.  57. sham.tw.  58. (assign$ or allocat$).tw.  59. controls.tw.  60. trial.ti. or (RCT or RCTs).tw.  61. or/38‐60  62. 37 and 61

### Appendix 8. Chinese Biomedical Literature Database (CBM) SinoMed search strategy

**Table:**

**No**	**Search Query**
#1	"脑血管障碍"[不加权:不扩展] OR "脑血管基底神经节疾病"[不加权:扩展] OR "脑缺血"[不加权:不扩展] OR "脑梗死"[不加权:不扩展] OR "脑干梗死"[不加权:不扩展] OR "大脑梗死"[不加权:不扩展] OR "梗死, 大脑前动脉"[不加权:不扩展] OR "梗死, 大脑中动脉"[不加权:不扩展] OR "梗死, 大脑后动脉"[不加权:不扩展] OR "脑缺血发作, 短暂性"[不加权:不扩展] OR "颈动脉疾病"[不加权:不扩展] OR "颈动脉血栓形成"[不加权:不扩展] OR "颈动脉狭窄"[不加权:不扩展] OR "脑动脉疾病"[不加权:不扩展] OR "颅内动脉硬化"[不加权:不扩展]
#2	"颅内动静脉畸形"[不加权:扩展] OR "颅内栓塞和血栓形成"[不加权:扩展] OR "颅内出血"[不加权:不扩展] OR "脑出血"[不加权:不扩展] OR "颅内出血, 高血压性"[不加权:不扩展] OR "蛛网膜下腔出血"[不加权:不扩展] OR "卒中"[不加权:不扩展] OR "血管痉挛, 颅内"[不加权:不扩展]
#3	"卒中"[常用字段] OR "中风"[常用字段] OR "脑血管病"[常用字段] OR "脑内血管病"[常用字段] OR "脑血管意外"[常用字段] OR "颅内血管病"[常用字段] OR "脑动脉病"[常用字段] OR "颅内动脉病"[常用字段] OR "脑静脉病"[常用字段] OR "颅内静脉病"[常用字段]
#4	"脑缺血"[常用字段] OR "缺血性脑病"[常用字段] OR "脑梗死"[常用字段] OR "脑梗塞"[常用字段] OR "脑梗阻"[常用字段] OR "ESUS"[常用字段]
#5	"脑出血"[常用字段] OR "出血性脑病"[常用字段] OR "脑内出血"[常用字段] OR "颅内出血"[常用字段] OR "脑实质出血"[常用字段] OR "高血压性出血"[常用字段] OR "SAH"[常用字段] OR "ICH"[常用字段] OR "AVM"[常用字段]
#6	("短暂性"[常用字段] OR "暂时性"[常用字段]) AND "缺血"[常用字段] AND "发作"[常用字段]
#7	"脑%动脉"[常用字段] OR "脑%静脉"[常用字段] OR "颅内%脉"[常用字段] OR "脑%血管"[常用字段] OR "颅内%血管"[常用字段] OR "椎基底"[常用字段] OR "半球"[常用字段] OR "脑纵裂"[常用字段] OR "胼胝体"[常用字段] OR "皮层"[常用字段] OR "皮质"[常用字段] OR "脑室"[常用字段] OR "室周%白质"[常用字段] OR "后颅窝"[常用字段] OR "后颅凹"[常用字段] OR "颅后窝"[常用字段]
#8	"幕下"[常用字段] OR "幕上"[常用字段] OR "豆状核"[常用字段] OR "纹状体"[常用字段] OR "豆纹"[常用字段] OR "前循环"[常用字段] OR "后循环"[常用字段] OR "MCA"[常用字段] OR "ACA"[常用字段] OR "PCA"[常用字段] OR "基底核"[常用字段] OR "基底节"[常用字段] OR "基底神经节"[常用字段] OR "基底动脉"[常用字段] OR "椎动脉"[常用字段] OR "占位"[常用字段] OR"腔隙"[常用字段] OR "眼动脉"[常用字段] OR "脑干"[常用字段] OR "蛛网膜"[常用字段]
#9	(#8) OR (#7)
#10	"缺血"[常用字段] OR "供血不足"[常用字段] OR "供血不全"[常用字段] OR "缺氧"[常用字段] OR "低氧"[常用字段] OR "氧%不足"[常用字段] OR "血肿"[常用字段] OR "血凝"[常用字段] OR "血块"[常用字段] OR "血栓"[常用字段] OR "栓子"[常用字段] OR "栓塞"[常用字段]
#11	"梗死"[常用字段] OR "梗塞"[常用字段] OR "堵塞"[常用字段] OR "阻塞"[常用字段] OR "梗阻"[常用字段] OR "脑梗"[常用字段] OR "闭塞"[常用字段] OR "狭窄"[常用字段] OR "缩窄"[常用字段] OR "硬化"[常用字段] OR "血管收缩"[常用字段] OR "动脉收缩"[常用字段] OR "血管痉挛"[常用字段] OR "动脉痉挛"[常用字段] OR "出血"[常用字段] OR "溢血"[常用字段]
#12	(#11) OR (#10)
#13	(#12) AND (#9)
#14	"颈动脉"[常用字段] OR "颈内动脉"[常用字段] OR "颅内"[常用字段] OR "脑"[常用字段] OR "基底动脉"[常用字段] OR "椎动脉"[常用字段] OR "椎基底"[常用字段]
#15	"动脉瘤"[常用字段] OR "血管畸形"[常用字段] OR "血管畸型"[常用字段] OR "动静脉畸形"[常用字段] OR "动静脉畸型"[常用字段] OR "动‐静脉畸形"[常用字段] OR "动‐静脉畸型"[常用字段] OR "动脉畸形"[常用字段] OR "动脉畸型"[常用字段] OR "静脉畸形"[常用字段] OR "静脉畸型"[常用字段] OR "静脉异常"[常用字段] OR "动脉异常"[常用字段] OR "血管异常"[常用字段]
#16	"动脉杂音"[常用字段] OR "血管杂音"[常用字段] OR "血栓"[常用字段] OR "栓子"[常用字段] OR "栓塞"[常用字段] OR "梗死"[常用字段] OR "梗塞"[常用字段] OR "堵塞"[常用字段] OR "阻塞"[常用字段] OR "梗阻"[常用字段] OR "闭塞"[常用字段] OR "狭窄"[常用字段] OR "缩窄"[常用字段] OR "硬化"[常用字段] OR "粥样"[常用字段] OR "斑块"[常用字段]
#17	(#16) OR (#15)
#18	(#17) AND (#14)
#19	(#18) OR (#13) OR (#6) OR (#5) OR (#4) OR (#3) OR (#2) OR (#1)
#20	"物理治疗方法"[不加权:不扩展] OR "身体锻炼"[不加权:扩展] OR "运动疗法"[不加权:扩展] OR "康复"[不加权:不扩展] OR "神经康复"[不加权:不扩展] OR "中风康复"[不加权:不扩展] OR "职业疗法"[不加权:不扩展] OR "职业治疗师"[不加权:不扩展] OR "物理治疗师"[不加权:不扩展]
#21	"反馈"[不加权:不扩展] OR "反馈, 生理"[不加权:不扩展] OR "生物反馈, 心理学"[不加权:扩展]
#22	"运动(Exercise)"[不加权:不扩展] OR "矫形外科手术"[不加权:不扩展]
#23	"物理治疗"[常用字段] OR "物理疗法"[常用字段] OR "理疗"[常用字段] OR "康复"[常用字段] OR "复健"[常用字段] OR "运动治疗"[常用字段] OR "运动疗法"[常用字段] OR "活动治疗"[常用字段] OR "职业疗法"[常用字段] OR "职业治疗"[常用字段] OR "作业疗法"[常用字段] OR "作业治疗"[常用字段] OR "作业完成"[常用字段] OR "作业分析"[常用字段] OR "活动治疗"[常用字段] OR "活动疗法"[常用字段] OR "移动治疗"[常用字段]
#24	"反馈"[常用字段]
#25	("机体"[常用字段] OR "运动"[常用字段] OR "体能"[常用字段] OR "机能"[常用字段] OR "活动"[常用字段] OR "移动"[常用字段] OR "功能"[常用字段]) AND ("练习"[常用字段] OR "学习"[常用字段] OR "再学习"[常用字段] OR "培训"[常用字段] OR "训练"[常用字段])
#26	("神经肌肉"[常用字段] OR "神经发育"[常用字段]) AND ("促进"[常用字段] OR "增强"[常用字段] OR "强化"[常用字段] OR "练习"[常用字段] OR "学习"[常用字段] OR "再学习"[常用字段] OR "培训"[常用字段] OR "训练"[常用字段] OR "锻炼"[常用字段]) OR "PNF"[常用字段] OR "NDT"[常用字段]
#27	("神经肌肉"[常用字段] OR "神经发育"[常用字段] OR "神经生理"[常用字段] OR "矫形"[常用字段] OR "矫正"[常用字段]) AND ("治疗"[常用字段] OR "疗法"[常用字段] OR "疗程"[常用字段] OR "康复"[常用字段] OR "恢复"[常用字段] OR "复健"[常用字段] OR "功能训练"[常用字段] OR "功能锻炼"[常用字段] OR "机能训练"[常用字段])
#28	"Bobath"[常用字段] OR "巴氏球"[常用字段] OR "Carr"[常用字段] OR "Brunnstrom"[常用字段] OR "布氏"[常用字段] OR "Rood"[常用字段]
#29	(#28) OR (#27) OR (#26) OR (#25) OR (#24) OR (#23) OR (#22) OR (#21) OR (#20)
#30	(#29) AND (#19)
#31	"脑血管障碍/康复"[不加权:不扩展] OR "脑血管基底神经节疾病/康复"[不加权:扩展] OR "脑缺血/康复"[不加权:不扩展] OR "脑梗死/康复"[不加权:不扩展] OR "脑干梗死/康复"[不加权:不扩展] OR "大脑梗死/康复"[不加权:不扩展] OR "梗死, 大脑前动脉/康复"[不加权:不扩展] OR "梗死, 大脑中动脉/康复"[不加权:不扩展] OR "梗死, 大脑后动脉/康复"[不加权:不扩展] OR "脑缺血发作, 短暂性/康复"[不加权:不扩展] OR "颈动脉疾病/康复"[不加权:不扩展] OR "颈动脉血栓形成/康复"[不加权:不扩展] OR "颈动脉狭窄/康复"[不加权:不扩展]
#32	"脑动脉疾病/康复"[不加权:不扩展] OR "颅内动脉硬化/康复"[不加权:不扩展] OR "颅内动静脉畸形/康复"[不加权:扩展] OR "颅内栓塞和血栓形成/康复"[不加权:扩展] OR "颅内出血/康复"[不加权:不扩展] OR "脑出血/康复"[不加权:不扩展] OR "颅内出血, 高血压性/康复"[不加权:不扩展] OR "蛛网膜下腔出血/康复"[不加权:不扩展] OR "卒中/康复"[不加权:不扩展] OR "血管痉挛, 颅内/康复"[不加权:不扩展]
#33	"神经康复"[不加权:不扩展] OR "中风康复"[不加权:不扩展]
#34	(#33) OR (#32) OR (#31) OR (#30)
#35	"精神运动性行为"[不加权:不扩展] OR "运动技能"[不加权:不扩展] OR "作业完成和分析"[不加权:不扩展]
#36	"学习"[不加权:不扩展] OR "条件反射(心理学)"[不加权:不扩展] OR "基于问题的学习"[不加权:不扩展] OR "问题解决"[不加权:不扩展]
#37	"运动(Movement)"[不加权:不扩展] OR "移动"[不加权:不扩展] OR "步行"[不加权:不扩展] OR "辅助行走"[不加权:不扩展] OR "步态"[不加权:不扩展]
#38	"运动活动"[不加权:不扩展] OR "活动范围, 关节"[不加权:不扩展]
#39	"姿势平衡"[不加权:不扩展] OR "体位"[不加权:扩展] OR "旋后"[不加权:不扩展] OR "旋前"[不加权:不扩展] OR "负重"[不加权:不扩展]
#40	"下肢"[不加权:扩展] OR "背"[不加权:扩展]
#41	("运动"[常用字段] OR "活动"[常用字段] OR "移动"[常用字段] OR "体育"[常用字段]) AND ( "技能"[常用字段] OR "技术"[常用字段] OR "技巧"[常用字段] OR "功能"[常用字段] OR "机能"[常用字段] OR "机体"[常用字段] OR "体能"[常用字段] OR "训练"[常用字段] OR "锻炼"[常用字段] OR "练习"[常用字段] OR "学习"[常用字段] OR "再学习"[常用字段] OR "培训"[常用字段] OR "恢复"[常用字段])
#42	"体力"[常用字段] OR "体能"[常用字段] OR "力量"[常用字段] OR "负重"[常用字段] OR "减重"[常用字段] OR "体重支持"[常用字段] OR "重力"[常用字段] OR "重心"[常用字段] OR "平板"[常用字段]
#43	"体位"[常用字段] OR "姿势"[常用字段] OR "姿态"[常用字段] OR "步态"[常用字段] OR "步行"[常用字段] OR "步伐"[常用字段] OR "徒步"[常用字段] OR "散步"[常用字段] OR "走步"[常用字段] OR "行走"[常用字段] OR "健走"[常用字段] OR "健步"[常用字段] OR "快走"[常用字段] OR "快步"[常用字段] OR "慢走"[常用字段] OR "慢步"[常用字段]
#44	"坐姿"[常用字段] OR "坐位"[常用字段] OR "坐立"[常用字段] OR "坐‐站"[常用字段] OR "站位"[常用字段] OR "站姿"[常用字段] OR "站立"[常用字段] OR "旋前"[常用字段] OR "内旋"[常用字段] OR "俯卧"[常用字段] OR "旋后"[常用字段] OR "旋外"[常用字段]
#45	"仰卧"[常用字段] OR "平衡"[常用字段] OR "协调"[常用字段] OR "倾斜"[常用字段] OR "爬行"[常用字段] OR "爬楼"[常用字段] OR "爬坡"[常用字段] OR "爬梯"[常用字段] OR "踩车"[常用字段] OR "踩步"[常用字段] OR "踩踏"[常用字段] OR "踏步"[常用字段] OR "踏车"[常用字段] OR "腳踏"[常用字段]
#46	"下肢"[常用字段] OR "腿"[常用字段] OR "踝"[常用字段] OR "足"[常用字段] OR "脚"[常用字段] OR "膝"[常用字段] OR "臀"[常用字段] OR "髋"[常用字段] OR "股"[常用字段] OR "腓肠肌"[常用字段] OR "躯体"[常用字段] OR "躯体"[常用字段] OR "背"[常用字段]
#47	(#46) OR (#45) OR (#44) OR (#43) OR (#42) OR (#41) OR (#40) OR (#39) OR (#38) OR (#37) OR (#36) OR (#35)
#48	(#47) AND (#34)
#49	随机对照试验[文献类型] OR 临床试验[文献类型]
#50	"随机"[摘要] OR "安慰剂"[摘要] OR "试验"[摘要] OR "分组"[摘要] OR "对照"[摘要]
#51	(#50) OR (#49)
#52	("动物"[不加权:扩展] NOT "人类"[不加权:不扩展]) OR (动物[特征词] NOT 人类[特征词])
#53	(#51) NOT (#52)
#54	(#53) AND (#48)

* SinoMed database does not allow for full text search or text search as the pdf cannot be analysed directly.

*Commonly used fields [常用字段] Consists of four search terms: Chinese title, abstract, keywords and subject terms.

* Wildcard [%] Use % to search for zero or more characters.

### Appendix 9. Clinical trial registry search strategies

**US National Institutes of Health Ongoing Trials Register ClinicalTrials.gov (www.clinicaltrials.gov)**

(physical rehabilitation OR physical therapy OR physiotherapy OR exercise OR rehabilitation OR occupational therapy OR activity OR feedback OR biofeedback OR neurorehabilitation OR neuromuscular OR neurodevelopmental OR neurophysiological ) AND AREA[StudyType] EXPAND[Term] COVER[FullMatch] "Interventional" AND AREA[ConditionSearch] ( Carotid Artery OR Cerebral Artery OR Hemorrhagic Stroke OR Intracranial Hemorrhages OR Brain Infarction OR Cerebral Hemorrhage OR Brain Ischemia OR Cerebrovascular Disorders OR Stroke OR Paresis OR Hemiparesis OR Hemiplegia ) AND AREA[InterventionSearch] Randomized AND AREA[StdAge] EXPAND[Term] COVER[FullMatch] ( "Adult" OR "Older Adult" )

**World Health Organization International Clinical Trials Registry Platform (trialsearch.who.int/)**

Condition: cerebrovascular OR stroke

Intervention: physical rehabilitation OR physical therapy OR physiotherapy OR exercise OR rehabilitation OR occupational therapy OR activity OR feedback OR biofeedback OR neurorehabilitation OR neuromuscular OR neurodevelopmental OR neurophysiological

Recruitment status is: ALL

Phases are: ALL

### Appendix 10. REHABDATA search terms

REHABDATA (National Rehabilitation Information Center (https://www.naric.com/?q=en/REHABDATA)

Title/Abstract: cerebrovascular OR stroke Title/Abstract:: physical rehabilitation OR physical therapy OR physiotherapy OR exercise OR rehabilitation OR occupational therapy OR activity OR feedback OR biofeedback OR neurorehabilitation OR neuromuscular OR neurodevelopmental OR neurophysiological

### Appendix 11. Cochrane Screen4Me and Cochrane Crowd screening

17510 records assessed in Screen4Me:

- 1080 assessed as possible RCTs (taken into Covidence for review author screening)
- 314 rejected as not RCTs
- 16,116 assessed using RCT classifier:
  - 3840 rejected as not RCTs
  - 12,276 assessed by volunteers within Cochrane Crowd
    - 4084 rejected as irrelevant
    - 8192 considered possibly relevant (taken into Covidence for review author screening)

Thus, from the 17,510 records assessed; a total of 8238 were rejected and 9272 taken into Covidence for review author screening.

There were 111 volunteer screeners within Cochrane Crowd (see Acknowledgements).

## Data and analyses

**Comparison 1:** Physical rehabilitation versus no physical rehabilitation: immediate outcomes

Outcome or subgroup title	No. of studies	No. of participants	Statistical method	Effect size
1.1 Independence in ADL scales	52	5403	Std. Mean Difference (IV, Random, 95% CI)	1.32 [1.08, 1.56]
1.1.1 Barthel Index	50	5263	Std. Mean Difference (IV, Random, 95% CI)	1.32 [1.08, 1.57]
1.1.2 Other ADL measure	2	140	Std. Mean Difference (IV, Random, 95% CI)	1.31 [0.94, 1.68]
1.2 Motor function scales	50	5669	Std. Mean Difference (IV, Random, 95% CI)	1.01 [0.80, 1.22]
1.2.1 Fugl Meyer	46	5291	Std. Mean Difference (IV, Random, 95% CI)	1.09 [0.87, 1.30]
1.2.2 Other motor function measure	4	378	Std. Mean Difference (IV, Random, 95% CI)	0.16 [‐0.10, 0.41]
1.3 Balance (Berg Balance Scale)	9	452	Mean Difference (IV, Random, 95% CI)	4.54 [1.36, 7.72]
1.4 Gait velocity	18	1131	Std. Mean Difference (IV, Random, 95% CI)	0.23 [0.05, 0.42]
1.4.1 Gait speed (distance/time)	11	759	Std. Mean Difference (IV, Random, 95% CI)	0.26 [0.08, 0.43]
1.4.2 Timed walk (time to walk set distance)	2	137	Std. Mean Difference (IV, Random, 95% CI)	0.06 [‐0.27, 0.40]
1.4.3 Timed up and go test	4	211	Std. Mean Difference (IV, Random, 95% CI)	0.29 [‐0.47, 1.04]
1.4.4 Other measure relating to gait speed	1	24	Std. Mean Difference (IV, Random, 95% CI)	‐0.13 [‐0.94, 0.69]
1.5 Length of stay	1	110	Mean Difference (IV, Random, 95% CI)	0.80 [‐0.93, 2.53]
1.6 Adverse events	5	283	Risk Ratio (M‐H, Fixed, 95% CI)	8.70 [2.90, 26.06]

**Comparison 2:** Physical rehabilitation versus no physical rehabilitation: persisting outcomes

Outcome or subgroup title	No. of studies	No. of participants	Statistical method	Effect size
2.1 Independence in ADL scales	12	757	Std. Mean Difference (IV, Random, 95% CI)	0.52 [0.17, 0.88]
2.1.1 Barthel Index	12	757	Std. Mean Difference (IV, Random, 95% CI)	0.52 [0.17, 0.88]
2.2 Motor function scales	11	1967	Std. Mean Difference (IV, Random, 95% CI)	0.50 [0.22, 0.78]
2.2.1 Fugl Meyer	8	1692	Std. Mean Difference (IV, Random, 95% CI)	0.72 [0.47, 0.97]
2.2.2 Other motor function measure	3	275	Std. Mean Difference (IV, Random, 95% CI)	‐0.09 [‐0.32, 0.15]
2.3 Balance (Berg Balance Scale)	4	239	Mean Difference (IV, Random, 95% CI)	0.58 [‐1.71, 2.87]
2.4 Gait velocity	7	509	Std. Mean Difference (IV, Random, 95% CI)	0.01 [‐0.18, 0.20]
2.4.1 Gait speed (distance/time)	4	323	Std. Mean Difference (IV, Random, 95% CI)	0.06 [‐0.17, 0.30]
2.4.2 Timed walk (time to walk set distance)	1	78	Std. Mean Difference (IV, Random, 95% CI)	‐0.24 [‐0.69, 0.20]
2.4.3 Timed up and go test	2	108	Std. Mean Difference (IV, Random, 95% CI)	0.01 [‐0.51, 0.54]
2.5 Adverse events^a^	1		Risk Ratio (M‐H, Fixed, 95% CI)	Subtotals only

**Comparison 3:** Physical rehabilitation versus attention control: immediate outcomes

Outcome or subgroup title	No. of studies	No. of participants	Statistical method	Effect size
3.1 Independence in ADL scales	2	106	Std. Mean Difference (IV, Random, 95% CI)	0.91 [0.06, 1.75]
3.1.1 Other ADL measure	2	106	Std. Mean Difference (IV, Random, 95% CI)	0.91 [0.06, 1.75]
3.2 Motor function scales	5	237	Std. Mean Difference (IV, Random, 95% CI)	0.13 [‐0.13, 0.38]
3.2.1 Fugl Meyer	2	120	Std. Mean Difference (IV, Random, 95% CI)	0.32 [‐0.04, 0.68]
3.2.2 Other motor function measure	3	117	Std. Mean Difference (IV, Random, 95% CI)	‐0.07 [‐0.43, 0.30]
3.3 Balance (Berg Balance Scale)	4	240	Mean Difference (IV, Random, 95% CI)	6.61 [‐0.45, 13.66]
3.4 Gait velocity	9	474	Std. Mean Difference (IV, Random, 95% CI)	0.27 [‐0.01, 0.55]
3.4.1 Gait speed (distance/time)	7	405	Std. Mean Difference (IV, Random, 95% CI)	0.34 [0.14, 0.54]
3.4.2 Timed up and go test	2	69	Std. Mean Difference (IV, Random, 95% CI)	‐0.04 [‐1.46, 1.39]
3.5 Length of stay	1	30	Mean Difference (IV, Random, 95% CI)	‐33.00 [‐64.11, ‐1.89]
3.6 Adverse events	3	290	Risk Ratio (M‐H, Fixed, 95% CI)	1.92 [0.75, 4.93]

**Comparison 4:** Physical rehabilitation versus attention control: persisting outcomes

Outcome or subgroup title	No. of studies	No. of participants	Statistical method	Effect size
4.1 Independence in ADL scales	0	0	Std. Mean Difference (IV, Random, 95% CI)	Not estimable
4.2 Motor function scales	3	119	Std. Mean Difference (IV, Random, 95% CI)	‐0.10 [‐0.46, 0.26]
4.2.1 Other motor function measure	3	119	Std. Mean Difference (IV, Random, 95% CI)	‐0.10 [‐0.46, 0.26]
4.3 Balance (Berg Balance Scale)	0	0	Std. Mean Difference (IV, Random, 95% CI)	Not estimable
4.4 Gait velocity	5	156	Std. Mean Difference (IV, Random, 95% CI)	0.26 [‐0.05, 0.58]
4.4.1 Gait speed (distance/time)	3	89	Std. Mean Difference (IV, Random, 95% CI)	0.35 [‐0.08, 0.77]
4.4.2 Timed up and go test	2	67	Std. Mean Difference (IV, Random, 95% CI)	0.16 [‐0.38, 0.71]

**Comparison 5:** Additional physical rehabilitation + usual therapy versus usual therapy: immediate outcomes

Outcome or subgroup title	No. of studies	No. of participants	Statistical method	Effect size
5.1 Independence in ADL scales	21	1972	Std. Mean Difference (IV, Random, 95% CI)	1.26 [0.82, 1.71]
5.1.1 Barthel Index	21	1972	Std. Mean Difference (IV, Random, 95% CI)	1.26 [0.82, 1.71]
5.2 Motor function scales	22	1965	Std. Mean Difference (IV, Random, 95% CI)	0.69 [0.46, 0.92]
5.2.1 Fugl Meyer	15	1261	Std. Mean Difference (IV, Random, 95% CI)	0.84 [0.59, 1.09]
5.2.2 Other motor function measure	7	704	Std. Mean Difference (IV, Random, 95% CI)	0.31 [‐0.06, 0.69]
5.3 Balance (Berg Balance Scale)	15	795	Mean Difference (IV, Random, 95% CI)	5.74 [3.78, 7.71]
5.4 Gait velocity	19	1004	Std. Mean Difference (IV, Random, 95% CI)	0.59 [0.26, 0.91]
5.4.1 Gait speed (distance/time)	10	766	Std. Mean Difference (IV, Random, 95% CI)	0.40 [0.03, 0.76]
5.4.2 Timed walk (time to walk set distance)	6	139	Std. Mean Difference (IV, Random, 95% CI)	1.02 [0.08, 1.96]
5.4.3 Timed up and go test	2	78	Std. Mean Difference (IV, Random, 95% CI)	1.26 [‐0.69, 3.22]
5.4.4 Other measure relating to gait speed	1	21	Std. Mean Difference (IV, Random, 95% CI)	0.50 [‐0.38, 1.38]
5.5 Adverse events	4	702	Risk Ratio (M‐H, Fixed, 95% CI)	0.80 [0.64, 0.98]

**Comparison 6:** Additional physical rehabilitation + usual therapy versus usual therapy: persisting outcomes

Outcome or subgroup title	No. of studies	No. of participants	Statistical method	Effect size
6.1 Independence in ADL scales	0	0	Std. Mean Difference (IV, Random, 95% CI)	Not estimable
6.2 Motor function scales	3	103	Std. Mean Difference (IV, Random, 95% CI)	0.12 [‐0.60, 0.83]
6.2.1 Other motor function measure	3	103	Std. Mean Difference (IV, Random, 95% CI)	0.12 [‐0.60, 0.83]
6.3 Balance (Berg Balance Scale)	2	56	Mean Difference (IV, Random, 95% CI)	‐2.30 [‐15.41, 10.81]
6.4 Gait velocity	4	159	Std. Mean Difference (IV, Random, 95% CI)	0.20 [‐0.12, 0.52]
6.4.1 Gait speed (distance/time)	1	74	Std. Mean Difference (IV, Random, 95% CI)	0.05 [‐0.40, 0.51]
6.4.2 Timed walk (time to walk set distance)	2	37	Std. Mean Difference (IV, Random, 95% CI)	0.38 [‐0.33, 1.09]
6.4.3 Timed up and go test	1	48	Std. Mean Difference (IV, Random, 95% CI)	0.33 [‐0.24, 0.90]

**Comparison 7:** Comparison of different physical rehabilitation approaches: immediate outcomes

Outcome or subgroup title	No. of studies	No. of participants	Statistical method	Effect size
7.1 Functional task training compared to other: Independence in ADL scales	22	1535	Std. Mean Difference (IV, Random, 95% CI)	0.58 [0.29, 0.87]
7.1.1 Functional task training compared to 'less' functional task training	13	1067	Std. Mean Difference (IV, Random, 95% CI)	0.71 [0.29, 1.13]
7.1.2 Functional task training compared to Neurophysiological	9	468	Std. Mean Difference (IV, Random, 95% CI)	0.38 [0.13, 0.63]
7.2 Functional task training compared to other: Motor function scales	20	1671	Std. Mean Difference (IV, Random, 95% CI)	0.72 [0.21, 1.22]
7.2.1 Functional task training compared to 'less' functional task training	11	1229	Std. Mean Difference (IV, Random, 95% CI)	0.95 [0.19, 1.71]
7.2.2 Functional task training compared to neurophysiological	9	442	Std. Mean Difference (IV, Random, 95% CI)	0.41 [‐0.14, 0.96]
7.3 Functional task training compared to other: Balance (Berg Balance Scale)	25	1194	Mean Difference (IV, Random, 95% CI)	2.16 [‐0.24, 4.55]
7.3.1 Functional task training compared to 'less' functional task training	19	988	Mean Difference (IV, Random, 95% CI)	2.56 [0.47, 4.64]
7.3.2 Functional Task Training compared to Neurophysiological	6	206	Mean Difference (IV, Random, 95% CI)	‐0.07 [‐8.64, 8.50]
7.4 Functional task training compared to other: Gait velocity	27	1719	Std. Mean Difference (IV, Random, 95% CI)	0.28 [‐0.01, 0.56]
7.4.1 Functional task training compared to 'less' functional task training	21	1455	Std. Mean Difference (IV, Random, 95% CI)	0.30 [‐0.04, 0.63]
7.4.2 Functional Task Training compared to Neurophysiological	6	264	Std. Mean Difference (IV, Random, 95% CI)	0.13 [‐0.25, 0.51]
7.5 Functional task training compared to other: Length of hospital stay	1	75	Mean Difference (IV, Fixed, 95% CI)	6.00 [0.69, 11.31]
7.5.1 Functional task training vs less functional task training	1	75	Mean Difference (IV, Fixed, 95% CI)	6.00 [0.69, 11.31]
7.6 Functional task training compared to other approaches: Adverse events	6	473	Risk Ratio (M‐H, Fixed, 95% CI)	1.33 [0.91, 1.94]
7.7 Neurophysiological approaches compared to other: Independence in ADL scales	14	737	Std. Mean Difference (IV, Random, 95% CI)	‐0.34 [‐0.63, ‐0.06]
7.7.1 Neurophysiological compared to no (or minimal) neurophysiological	4	219	Std. Mean Difference (IV, Random, 95% CI)	‐0.20 [‐1.17, 0.77]
7.7.2 Neurophysiological (Bobath) compared to Neurophysiological (other)	1	50	Std. Mean Difference (IV, Random, 95% CI)	‐0.36 [‐0.96, 0.24]
7.7.3 Neurophysiological compared to Functional Task Training	9	468	Std. Mean Difference (IV, Random, 95% CI)	‐0.38 [‐0.63, ‐0.13]
7.8 Neurophysiological approaches compared to other: Motor function scales	13	663	Std. Mean Difference (IV, Random, 95% CI)	‐0.60 [‐1.32, 0.12]
7.8.1 Neurophysiological compared to no (or minimal) neurophysiological	3	171	Std. Mean Difference (IV, Random, 95% CI)	‐1.66 [‐5.54, 2.23]
7.8.2 Neurophysiological (Bobath) compared to neurophysiological (other)	1	50	Std. Mean Difference (IV, Random, 95% CI)	0.14 [‐0.45, 0.74]
7.8.3 Neurophysiological compared to functional task training	9	442	Std. Mean Difference (IV, Random, 95% CI)	‐0.41 [‐0.96, 0.14]
7.9 Neurophysiological approaches compared to other: Balance (Berg Balance Scale)	9	292	Mean Difference (IV, Random, 95% CI)	0.06 [‐5.90, 6.03]
7.9.1 Neurophysiological compared to no (or minimal) neurophysiological	2	56	Mean Difference (IV, Random, 95% CI)	0.04 [‐3.12, 3.19]
7.9.2 Neurophysiological (Bobath) compared to neurophysiological (other)	1	30	Mean Difference (IV, Random, 95% CI)	0.60 [‐3.96, 5.16]
7.9.3 Neurophysiological compared to functional task training	6	206	Mean Difference (IV, Random, 95% CI)	0.07 [‐8.50, 8.64]
7.10 Neurophysiological approaches compared to other approaches: Gait velocity	16	630	Std. Mean Difference (IV, Random, 95% CI)	‐0.17 [‐0.62, 0.27]
7.10.1 Neurophysiological compared to no (or minimal) neurophysiological	7	298	Std. Mean Difference (IV, Random, 95% CI)	‐0.06 [‐1.01, 0.88]
7.10.2 Neurophysiological (Bobath) compared to neurophysiological (other)	3	68	Std. Mean Difference (IV, Random, 95% CI)	‐0.17 [‐0.65, 0.31]
7.10.3 Neurophysiological compared to functional task training	6	264	Std. Mean Difference (IV, Random, 95% CI)	‐0.13 [‐0.51, 0.25]
7.11 Neurophysiological approach compared to other: Adverse events	1	40	Risk Ratio (M‐H, Fixed, 95% CI)	Not estimable

**Comparison 8:** Comparison of different physical rehabilitation approaches: persisting outcomes

Outcome or subgroup title	No. of studies	No. of participants	Statistical method	Effect size
8.1 Functional task training compared to other approaches: Independence in ADL scales	8	631	Std. Mean Difference (IV, Random, 95% CI)	1.20 [0.22, 2.18]
8.1.1 Functional task training compared to 'less' functional task training	6	553	Std. Mean Difference (IV, Random, 95% CI)	1.34 [0.11, 2.56]
8.1.2 Functional Task Training compared to Neurophysiological	2	78	Std. Mean Difference (IV, Random, 95% CI)	0.75 [‐0.29, 1.80]
8.2 Functional task training compared to other approaches: Motor function scales	9	917	Std. Mean Difference (IV, Random, 95% CI)	0.18 [‐0.16, 0.52]
8.2.1 Functional task training compared to 'less' functional task training	7	789	Std. Mean Difference (IV, Random, 95% CI)	0.34 [0.05, 0.63]
8.2.2 Functional task training compared to neurophysiological	2	128	Std. Mean Difference (IV, Random, 95% CI)	‐0.37 [‐1.38, 0.64]
8.3 Functional task training compared to other approaches: Balance (Berg Balance Scale)	7	541	Mean Difference (IV, Random, 95% CI)	0.44 [‐6.27, 7.14]
8.3.1 Functional task training compared to 'less' functional task training	6	461	Mean Difference (IV, Random, 95% CI)	2.64 [‐2.44, 7.72]
8.3.2 Functional task training compared to neurophysiological	1	80	Mean Difference (IV, Random, 95% CI)	‐10.63 [‐12.73, ‐8.53]
8.4 Functional task training compared to other approaches: Gait velocity	11	893	Std. Mean Difference (IV, Random, 95% CI)	0.33 [‐0.01, 0.66]
8.4.1 Functional task training compared to 'less' functional task training	9	783	Std. Mean Difference (IV, Random, 95% CI)	0.36 [0.05, 0.67]
8.4.2 Functional task training compared to neurophysiological	2	110	Std. Mean Difference (IV, Random, 95% CI)	0.26 [‐1.40, 1.92]
8.5 Functional task training compared to other approaches: Adverse events	1		Risk Ratio (M‐H, Fixed, 95% CI)	Subtotals only
8.6 Neurophysiological approaches compared to other approaches: Independence in ADL scales	4	142	Std. Mean Difference (IV, Random, 95% CI)	‐0.53 [‐1.01, ‐0.06]
8.6.1 Neurophysiological compared to functional task training	2	78	Std. Mean Difference (IV, Random, 95% CI)	‐0.75 [‐1.80, 0.29]
8.6.2 Neurophysiological compared to no (or minimal) neurophysiological	2	64	Std. Mean Difference (IV, Random, 95% CI)	‐0.34 [‐0.84, 0.15]
8.7 Neurophysiological approaches compared to other approaches: Motor function scales	2	128	Std. Mean Difference (IV, Random, 95% CI)	0.37 [‐0.64, 1.38]
8.7.1 Neurophysiological compared to functional task training	2	128	Std. Mean Difference (IV, Random, 95% CI)	0.37 [‐0.64, 1.38]
8.8 Neurophysiological approaches compared to other approaches: Balance (Berg Balance Scale)	1	80	Mean Difference (IV, Random, 95% CI)	10.63 [8.53, 12.73]
8.8.1 Neurophysiological compared to functional task training	1	80	Mean Difference (IV, Random, 95% CI)	10.63 [8.53, 12.73]
8.9 Neurophysiological approaches compared to other approaches: Gait velocity	3	124	Std. Mean Difference (IV, Random, 95% CI)	‐0.27 [‐1.41, 0.87]
8.9.1 Neurophysiological compared to functional task training	2	110	Std. Mean Difference (IV, Random, 95% CI)	‐0.26 [‐1.92, 1.40]
8.9.2 Neurophysiological compared to no (or minimal) neurophysiological	1	14	Std. Mean Difference (IV, Random, 95% CI)	‐0.33 [‐1.40, 0.74]

**Comparison 9:** Subgroup analysis: Physical rehabilitation versus no physical rehabilitation: immediate outcome ‐ independence in ADL scales

Outcome or subgroup title	No. of studies	No. of participants	Statistical method	Effect size
9.1 Length of time post stroke	29	2520	Std. Mean Difference (IV, Random, 95% CI)	1.53 [1.16, 1.91]
9.1.1 Early (2 weeks or less post‐stroke)	16	1463	Std. Mean Difference (IV, Random, 95% CI)	1.72 [1.18, 2.26]
9.1.2 Later (more than 2 weeks post‐stroke)	13	1057	Std. Mean Difference (IV, Random, 95% CI)	1.34 [0.82, 1.87]
9.2 Geographical location ‐ continent of study conduct	52	5403	Std. Mean Difference (IV, Random, 95% CI)	1.32 [1.08, 1.56]
9.2.1 Asia	46	4965	Std. Mean Difference (IV, Random, 95% CI)	1.41 [1.16, 1.67]
9.2.2 All other continents	6	438	Std. Mean Difference (IV, Random, 95% CI)	0.54 [‐0.19, 1.27]
9.3 Amount of treatment (less or more than 2.5 hours/week)	48	4192	Std. Mean Difference (IV, Random, 95% CI)	1.39 [1.11, 1.66]
9.3.1 Less than 2.5 hours/week	7	603	Std. Mean Difference (IV, Random, 95% CI)	0.67 [0.05, 1.28]
9.3.2 More than 2.5 hours/week	41	3589	Std. Mean Difference (IV, Random, 95% CI)	1.51 [1.21, 1.82]
9.4 Duration of intervention	50	5207	Std. Mean Difference (IV, Random, 95% CI)	1.36 [1.11, 1.61]
9.4.1 Short (≤ 2 months)	29	2851	Std. Mean Difference (IV, Random, 95% CI)	1.49 [1.12, 1.86]
9.4.2 Longer (> 2 months)	21	2356	Std. Mean Difference (IV, Random, 95% CI)	1.23 [0.89, 1.57]
9.5 Focus of treatment components	47	4934	Std. Mean Difference (IV, Random, 95% CI)	1.41 [1.16, 1.66]
9.5.1 Functional task training	19	1592	Std. Mean Difference (IV, Random, 95% CI)	1.47 [0.93, 2.00]
9.5.2 Mixed treatment components	28	3342	Std. Mean Difference (IV, Random, 95% CI)	1.34 [1.07, 1.61]
9.6 Named approaches	52	5403	Std. Mean Difference (IV, Random, 95% CI)	1.32 [1.08, 1.56]
9.6.1 Named as "three‐stage approach"	11	1592	Std. Mean Difference (IV, Random, 95% CI)	1.51 [0.96, 2.06]
9.6.2 Named as "Bobath" approach	12	1120	Std. Mean Difference (IV, Random, 95% CI)	1.59 [1.02, 2.15]
9.6.3 Other	30	2691	Std. Mean Difference (IV, Random, 95% CI)	1.16 [0.84, 1.48]
9.7 Provider of intervention	52	5403	Std. Mean Difference (IV, Random, 95% CI)	1.32 [1.08, 1.56]
9.7.1 Medical/therapeutic staff	17	1381	Std. Mean Difference (IV, Random, 95% CI)	1.45 [0.88, 2.01]
9.7.2 Medical/therapeutic staff + family/carers	16	2077	Std. Mean Difference (IV, Random, 95% CI)	1.52 [1.10, 1.94]
9.7.3 Others/not stated	19	1945	Std. Mean Difference (IV, Random, 95% CI)	1.17 [0.84, 1.49]

**Comparison 10:** Subgroup analysis: Physical rehabilitation versus no physical rehabilitation: immediate outcome ‐ motor function scales

Outcome or subgroup title	No. of studies	No. of participants	Statistical method	Effect size
10.1 Length of time post stroke	31	3114	Std. Mean Difference (IV, Random, 95% CI)	1.06 [0.79, 1.32]
10.1.1 Early (2 weeks or less post‐stroke)	21	2366	Std. Mean Difference (IV, Random, 95% CI)	0.89 [0.61, 1.17]
10.1.2 Later (more than 2 weeks post‐stroke)	10	748	Std. Mean Difference (IV, Random, 95% CI)	1.49 [0.82, 2.17]
10.2 Geographical location ‐ continent of study conduct	50	5669	Std. Mean Difference (IV, Random, 95% CI)	1.01 [0.80, 1.22]
10.2.1 Asia	47	5379	Std. Mean Difference (IV, Random, 95% CI)	1.07 [0.86, 1.28]
10.2.2 All other continents	3	290	Std. Mean Difference (IV, Random, 95% CI)	0.08 [‐0.26, 0.43]
10.3 Amount of treatment (less or more than 2.5 hours/week)	44	3980	Std. Mean Difference (IV, Random, 95% CI)	1.08 [0.83, 1.34]
10.3.1 Less than 2.5 hours/week	7	707	Std. Mean Difference (IV, Random, 95% CI)	0.83 [0.30, 1.35]
10.3.2 More than 2.5 hours/week	37	3273	Std. Mean Difference (IV, Random, 95% CI)	1.13 [0.84, 1.42]
10.4 Duration of intervention	47	5519	Std. Mean Difference (IV, Random, 95% CI)	0.98 [0.77, 1.19]
10.4.1 Short (≤ 2 months)	22	2332	Std. Mean Difference (IV, Random, 95% CI)	0.98 [0.59, 1.37]
10.4.2 Longer (> 2 months)	25	3187	Std. Mean Difference (IV, Random, 95% CI)	0.97 [0.75, 1.20]
10.5 Focus of treatment components	43	4694	Std. Mean Difference (IV, Random, 95% CI)	1.04 [0.81, 1.28]
10.5.1 Functional task training	19	1891	Std. Mean Difference (IV, Random, 95% CI)	1.34 [0.83, 1.86]
10.5.2 Mixed treatment components	24	2803	Std. Mean Difference (IV, Random, 95% CI)	0.82 [0.64, 1.01]
10.6 Named approaches	50	5669	Std. Mean Difference (IV, Random, 95% CI)	1.00 [0.79, 1.21]
10.6.1 Named as "three‐stage approach"	14	2274	Std. Mean Difference (IV, Random, 95% CI)	1.01 [0.71, 1.31]
10.6.2 Named as "Bobath" approach	9	879	Std. Mean Difference (IV, Random, 95% CI)	0.72 [0.35, 1.09]
10.6.3 Other	28	2516	Std. Mean Difference (IV, Random, 95% CI)	1.09 [0.72, 1.45]
10.7 Provider of intervention	50	5669	Std. Mean Difference (IV, Random, 95% CI)	1.01 [0.80, 1.22]
10.7.1 Medical/therapeutic staff	15	1240	Std. Mean Difference (IV, Random, 95% CI)	0.94 [0.48, 1.40]
10.7.2 Medical/therapeutic staff + family/carers	16	2391	Std. Mean Difference (IV, Random, 95% CI)	1.36 [0.94, 1.77]
10.7.3 Others/not stated	19	2038	Std. Mean Difference (IV, Random, 95% CI)	0.78 [0.53, 1.03]

**Comparison 11:** Subgroup analysis: Additional physical rehabilitation + usual therapy versus usual therapy: immediate outcomes ‐ independence in ADL scales

Outcome or subgroup title	No. of studies	No. of participants	Statistical method	Effect size
11.1 Length of time post stroke	15	1346	Std. Mean Difference (IV, Random, 95% CI)	0.97 [0.56, 1.38]
11.1.1 Early (2 weeks or less post‐stroke)	2	136	Std. Mean Difference (IV, Random, 95% CI)	0.74 [0.39, 1.09]
11.1.2 Later (more than 2 weeks post‐stroke)	13	1210	Std. Mean Difference (IV, Random, 95% CI)	1.02 [0.55, 1.49]
11.2 Geographical location ‐ continent of study conduct	21	1972	Std. Mean Difference (IV, Random, 95% CI)	1.22 [0.79, 1.66]
11.2.1 Asia	18	1554	Std. Mean Difference (IV, Random, 95% CI)	1.38 [0.90, 1.85]
11.2.2 All other continents	3	418	Std. Mean Difference (IV, Random, 95% CI)	0.04 [‐0.17, 0.25]
11.3 Amount of additional treatment (less or more than 2.5 hours/week)	21	1974	Std. Mean Difference (IV, Random, 95% CI)	1.26 [0.82, 1.71]
11.3.1 Less than 2.5 hours/week	2	401	Std. Mean Difference (IV, Random, 95% CI)	0.14 [‐0.36, 0.64]
11.3.2 Between 2.5 and 5 hours/week	7	508	Std. Mean Difference (IV, Random, 95% CI)	0.62 [0.32, 0.93]
11.3.3 More than 5 hours/week	12	1065	Std. Mean Difference (IV, Random, 95% CI)	1.82 [1.11, 2.53]
11.4 Duration of intervention	19	1834	Std. Mean Difference (IV, Random, 95% CI)	1.32 [0.83, 1.81]
11.4.1 Short (≤ 2 months)	15	1146	Std. Mean Difference (IV, Random, 95% CI)	1.60 [0.99, 2.22]
11.4.2 Longer (> 2 months)	4	688	Std. Mean Difference (IV, Random, 95% CI)	0.29 [‐0.06, 0.64]
11.5 Focus of treatment components	19	1502	Std. Mean Difference (IV, Random, 95% CI)	1.35 [0.86, 1.84]
11.5.1 Functional task training added to less functional approach	14	917	Std. Mean Difference (IV, Random, 95% CI)	1.59 [0.96, 2.22]
11.5.2 Neurophysiological approach added to a mixed or other approach	5	585	Std. Mean Difference (IV, Random, 95% CI)	0.67 [0.20, 1.15]
11.6 Provider of intervention	21	1972	Std. Mean Difference (IV, Random, 95% CI)	1.26 [0.82, 1.71]
11.6.1 Medical/therapeutic staff	8	831	Std. Mean Difference (IV, Random, 95% CI)	0.81 [0.24, 1.38]
11.6.2 Medical/therapeutic staff + family/carers	6	614	Std. Mean Difference (IV, Random, 95% CI)	1.52 [0.61, 2.44]
11.6.3 Others/not stated	7	527	Std. Mean Difference (IV, Random, 95% CI)	1.49 [0.73, 2.24]

**Comparison 12:** Subgroup analysis: Additional physical rehabilitation + usual therapy versus usual therapy: immediate outcomes ‐ motor function scales

Outcome or subgroup title	No. of studies	No. of participants	Statistical method	Effect size
12.1 Length of time post stroke	19	1695	Std. Mean Difference (IV, Random, 95% CI)	0.62 [0.38, 0.85]
12.1.1 Early (2 weeks or less post‐stroke)	2	200	Std. Mean Difference (IV, Random, 95% CI)	0.84 [0.55, 1.13]
12.1.2 Later (more than 2 weeks post‐stroke)	17	1495	Std. Mean Difference (IV, Random, 95% CI)	0.59 [0.33, 0.84]
12.2 Geographical location ‐ continent of study conduct	22	1965	Std. Mean Difference (IV, Random, 95% CI)	0.69 [0.46, 0.92]
12.2.1 Asia	15	1333	Std. Mean Difference (IV, Random, 95% CI)	0.87 [0.64, 1.10]
12.2.2 All other continents	7	632	Std. Mean Difference (IV, Random, 95% CI)	0.18 [‐0.08, 0.44]
12.3 Amount of additional treatment (less or more than 2.5 hours/week)	21	1873	Std. Mean Difference (IV, Random, 95% CI)	0.70 [0.46, 0.94]
12.3.1 Less than 2.5 hours/week	2	412	Std. Mean Difference (IV, Random, 95% CI)	0.02 [‐0.28, 0.32]
12.3.2 Between 2.5 and 5 hours/week	8	422	Std. Mean Difference (IV, Random, 95% CI)	0.52 [0.27, 0.76]
12.3.3 More than 5 hours / week	11	1039	Std. Mean Difference (IV, Random, 95% CI)	0.95 [0.64, 1.26]
12.4 Duration of intervention	20	1827	Std. Mean Difference (IV, Random, 95% CI)	0.68 [0.43, 0.93]
12.4.1 Short (≤ 2 months)	16	1245	Std. Mean Difference (IV, Random, 95% CI)	0.72 [0.46, 0.97]
12.4.2 Longer (> 2 months)	4	582	Std. Mean Difference (IV, Random, 95% CI)	0.57 [‐0.07, 1.21]
12.5 Focus of treatment components	19	1483	Std. Mean Difference (IV, Random, 95% CI)	0.75 [0.53, 0.97]
12.5.1 Functional task training added to less functional approach	13	910	Std. Mean Difference (IV, Random, 95% CI)	0.80 [0.51, 1.09]
12.5.2 Neurophysiological approach added to a mixed or other approach	6	573	Std. Mean Difference (IV, Random, 95% CI)	0.64 [0.31, 0.97]
12.6 Provider of intervention	22	1965	Std. Mean Difference (IV, Random, 95% CI)	0.69 [0.46, 0.92]
12.6.1 Medical/therapeutic staff	12	1094	Std. Mean Difference (IV, Random, 95% CI)	0.34 [0.12, 0.55]
12.6.2 Medical/therapeutic staff + family/carers	3	280	Std. Mean Difference (IV, Random, 95% CI)	1.03 [0.64, 1.42]
12.6.3 Others/not stated	7	591	Std. Mean Difference (IV, Random, 95% CI)	1.05 [0.76, 1.35]

## Characteristics of studies

### Characteristics of included studies [ordered by study ID]

**Table:** ACTIV 2021

***Study characteristics***	
Methods	**Study aim:** to investigate whether ACTIV improved physical function compared with usual care **Design:** parallel‐group RCT **Country:** New Zealand **Study recruitment and setting:** see Table **Involvement of patients/public/professionals/others:** unclear
Participants	**Study population (number randomised):** 95 **Inclusion criteria:** Adults aged > 20 years First‐ever hemispheric stroke of haemorrhagic or ischaemic origin Discharged from inpatient, outpatient, or community physiotherapy services to live in their own home **Exclusion criteria:** Confirmed brainstem or cerebellar stroke Unable to understand and speak 'basic‐level' English **Participants:** detailed in Table **PROGRESS‐plus:** detailed in Table
Interventions	(1) ACTIV (n = 47) "ACTIV focused on 2 functional categories: “staying upright” and “using your arm.” There were standard exercises for each category. Each exercise had parameters that could be selected and modified, allowing the program to be tailored to individuals. Further details of the program can be found in Supplemental material 2. The program was delivered by physical therapists who had completed ACTIV training, (Supplemental material 1, Table II). The physical therapists established patient‐centered goals at the first home visit. Next, they selected exercises and activities to address these goals, accessing advice from an expert neurological physical therapist if required. Each participant received 4 face‐to face visits, 5 structured phone calls, and personalized text messages." (2) Usual care control group (n = 48) "Standard care following discharge from rehabilitation services in New Zealand usually means no further formal rehabilitation. To ensure usual care, no attempt was made to discourage any additional care, and this was not measured".
Outcomes	**Independence in ADL scales:** NR **Motor function scales:** NR **Measures of balance:** balance assessed using the Step Test (not suitable for inclusion in meta‐analysis) **Measures of gait velocity:** NR **Length of stay:** NR **Adverse events:** monthly phone call to record adverse events; coded by 2 independent assessors **Other measures:** Stroke Impact Scale (SIS), hand grip strength, Stroke Self‐efficacy questionnaire, visual analogue scale of the EQ‐5D **Time points when outcomes were assessed:** baseline, at end of intervention (6 months after randomisation), follow‐up (12 months after randomisation)
Comparison	**Amount of physical rehabilitation in groups:** differs **Categorisation of comparison:** 1. Physical rehabilitation versus no or limited physical rehabilitation (dose differs) (limited = < 50% of dose)
Funding & conflicts of interest	**Funding statement:** Health Research Council of New Zealand 11/545 **Conflict of interest statement:** none
Synthesis	**Quantitative synthesis:** adverse events analysed: Analysis 1.6 No other outcomes suitable for inclusion in this review reported.
Notes	

**28:** Details of study participants ‐ stroke details and study numbers

**Study**	**Study group**	**No. of participants**	**Side ‐ LCVA/RCVA**	**Time since onset**	**Type of stroke**	**No. finished intervention**
ACTIV 2021	Augmented Community Telerehabilitation Intervention (ACTIV)	47	25/22	Mean 7.2 months SD 3.3 months	Ischaemic 40 Haemorrhagic 7	44
	Usual care	48	22/26	Mean 6.1 months SD 2.8 month	Ischaemic 44 Haemorrhagic 3	47
Ain 2022	Functional training	7	Whole group: Not stated	Whole group: Not stated	Whole group: Not stated	6
	Conventional therapy	7				6
Aksu 2001	Group 1	9	Not stated	Not stated	Not stated	9
	Group 2	7	Not stated	Not stated	Not stated	7
	Group 3	4	Not stated	Not stated	Not stated	4
Alabdulwahab 2015	Functional limb overloading (FLO)	13	Whole group 13/10 3 unknown	Whole group 6‐12 months 7 1‐2 years 13 2‐3 years 3 3 unknown	Not stated	13
	Limb Overloading Resistance Training (LORT)	13				10
Allison 2007	Intervention	7	Not stated	Mean 20.6 days SD 20.5 days Range 9‐57 days	Not stated	5
	Control	10	Not stated	Mean 15.1 days SD 16.0 days Range 6‐58 days	Not stated	10
Aloraini 2022	Constraint‐induced movement therapy	19	13/6	Mean 30.2 months SD 13.9 months	16 I 3 H	19
	Conventional rehabilitation	19	11/8	Mean 36.8 months SD 19.5 months	15 I 4 H	19
Anandan 2020	Task specific training (TST)	37	Whole group "Dominant side"	Whole group ≥ 6 months	Whole group MCA	25
	Proprioceptive neuromuscular facilitation group (PNG)	37				25
Arabzadeh 2018	Task‐oriented exercise programme	10	4/6	Mean 32.80 days SD 16.71 days	Ischaemic 10 Haemorrhagic 0	10
	Traditional physiotherapy	10	1/6	Mean 32.5 days SD 15.78 days	Ischaemic 10 Haemorrhagic 0	10
Aravind 2022	Community‐based exercise programmes supported through healthcare‐community partnership (CBEP‐HCP)	16	10/5 1 bilateral	6‐12 months 8 12‐18 months 4 > 18 months 4	Not stated	15?
	Wait list control	17	7/8 1 unknown	6‐12 months 10 12‐18 months 4 > 18 months 3		15
Aries 2021	Mobilisation and tactile stimulation + task‐specific gait training (MTS + TSGT)	19	11/8	Mean 59.5 days SD 18.1 days	Ischaemic 17 Haemorrhagic 2	19
	Unlimited textured insole wearing + task‐specific gait training (TI + TSGT)	15	9/6	Mean 53.9 days SD 12.4 days	Ischaemic 12 Haemorrhagic 3	13
Arya 2019	Interlimb coupling	26	15/11	Mean 15.44 months SD 5.36 months	Ischaemic 18 Haemorrhagic 8	26
	Conventional rehabilitation based on neurophysiological approaches (e.g. Brunnstrom, Bobath)	24	15/9	Mean 15.36 months SD 8.13 months	Ischaemic 19 Haemorrhagic 5	24
Baer 2007	Part practice	Whole group: 64	Whole group 26/38	Whole group Mean 30.3 months SD 28.8 months	Not stated	Not stated
	Whole practice	As above	As above	As above	Not stated	Not stated
	Control (no treatment)	As above	As above	As above	Not stated	Not stated
Bai 2008	Early rehabilitation	183	85/98	Mean 11.4 days SD 5.7 days	Not stated	175
	Control (no treatment)	181	87/94	Mean 10.9 days SD 5.5 days	Not stated	170
Bai 2013	Physiotherapy	41	18/23	Mean 40.54 days SD 21.44 days	Area of injury: Basal ganglia 16 Other 25	Not stated
	Acupuncture	39	21/18	Mean 37.13 days SD 21.34 days	Area of injury: Basal ganglia 12 Other 27	Not stated
	Combined	40	21/19	Mean 42.12 days SD 20.06 days	Area of injury: Basal ganglia 18 Other 22	
Bai 2014	Three‐stage rehabilitation intervention	83	47/36	Mean 10.20 days SD 5.72 days	Whole group Ischaemic	77
	Usual care (no physical rehabilitation included)	82	39/43	Mean 10.00 days SD 5.31 days		77
Bale 2008	Functional strength training	8	6/2	Mean 49.4 days SD 22.1 days	Cerebral infarct = 4 Haemorrhagic = 4	8
	Training as usual	10	3/7	Mean 32 days SD 18.5 days	Cerebral infarct = 8 Haemorrhagic = 2	10
Batchelor 2012	Multifactorial individually tailored falls prevention programme + usual care	71	24/43 2 bilateral 2 other/unknown	Mean 3.0 months SD 1.6 months	Infarct 55 Haemorrhage 13 Unknown 3	57
	Usual care	85	38/44 2 bilateral 1 other/unknown	Mean 3.1 months SD 1.9 months	Infarct 69 Haemorrhage 15 Unknown 1	75
Behrman 2011	Locomotor training programme	139	Not stated	Not stated	Not stated	139
	Home exercise programme	126	Not stated	Not stated	Not stated	126
	Usual care	143	Not stated	Not stated	Not stated	143
Bek 2016	Conductive Education	41	Not stated	Mean 34.5 months SD 39.8 months	Not stated	Completed intervention 32; completed assessments 30
	Control (2 meetings) wait list	36	Not stated	Mean 31.7 months SD 34.1 months	Not stated	Completed assessments 32
Bhatia 2014	Task specific strength training (TSST)	15	Not stated	Not stated	Whole group Ischaemic	Not stated
	Resistance training (RT)	15	Not stated	Not stated		Not stated
Blennerhassett 2004	Mobility	15	8/7	Mean 36.0 days SD 25.1 days	Haemorrhagic = 4 Infarct = 11	15
	Upper limb	15	6/9	Mean 50.1 days SD 49.2 days	Haemorrhagic = 4 Infarct = 11	15
Bordoloi 2020	Neuro‐facilitation (Rood) + Home Exercise Programme (HEP) + conventional physiotherapy (Group B)	118	Not stated	Not stated	Whole group Haemorrhagic stroke with supratentorial haematoma	118
	HEP with conventional physiotherapy (Group A)	118	Not stated	Not stated		118
Brock 2005	Bobath	12	2/9 Bilateral = 1	Mean 60.3 days SD 24.0 days Range 29‐101 days	Haemorrhagic = 2 Infarct = 8 Both = 2	12
	Task practice	14	10/3 Bilateral = 1	Mean 63.6 days SD 25.9 days Range 40‐126 days	Haemorrhagic =4 Infarct = 9 Both = 1	14
Brouwer 2018	Tune‐up group	51	22/29	Not stated	Ischaemic 37 Haemorrhagic 13 Not stated 1	35
	Control	52	15/37	Not stated	Ischaemic 43 Haemorrhagic 7 Not stated 2	35
Bui 2019	Modified acupuncture + Motor relearning method	33	Not stated	≤ 1 month 22 > 1 month 11	Not stated	Not stated
	Modified acupuncture + Bobath method	33	Not stated	≤ 1 month 23 > 1 month 10	Not stated	Not stated
Candan 2017	Modified constraint‐induced movement therapy (mCIMT)	18	5/10 3 Not stated	Mean 6.80 months SD 2.70 months 3 not stated	Ischaemia 11 Haemorrhage 4 3 Not stated	15
	Rehabilitation programme	15	5/10	Mean 6.63 months SD 3.18 months	Ischaemia 12 Haemorrhage 3	15
Cao 2014	Intensive walking training (SAT)	43	Not stated	Whole group: "early stroke hemiplegia"	Whole group: Infarction 62 Haemorrhage 24	43
	Routine rehabilitation therapy	43	Not stated			43
Capisizu 2016	Neuroprotective multimodal pharmacological therapy (NT)	Whole group: 115	Not stated	Whole group: 10 days	Not stated	Not stated
	Physical therapy (PT)		Not stated		Not stated	Not stated
	Combined treatment (NT+KT)		Not stated		Not stated	Not stated
Carlson 2006	Treatment	6	Not stated	Not stated	Not stated	6
	Control (no treatment)	5	Not stated	Not stated	Not stated	5
Chae 2017	Proprioceptive training group + general physical therapy	15	Not stated	Mean 13.53 months SD 5.13 months	Not stated	15
	General physical therapy	15	Not stated	Mean 15.27 months SD 4.18 months	Not stated	15
Chan DY 2006	Motor relearning	33	12/14	Mean 117.7 days SD not stated	Not stated	26
	Conventional therapy	33	12/14	Mean 88.8 days SD not stated	Not stated	26
Chan WN 2017	Tai Chi training	9	4/5	Mean 3.3 years SD 3.0 years	Ischaemic 7 Haemorrhage 1 Mixed 1	7
	Conventional exercise	8	2/3 Not stated 3	Mean 8.8 years SD 7.9 years	Ischaemic 2 Haemorrhage 3 Mixed 0 Not stated 3	5
	Control (no training)	9	7/8	Mean 4.6 years SD 3.0 years	Ischaemic 8 Haemorrhage 1 Mixed 0	6
Chang 2015	Three‐stage rehabilitation	25	Not stated	Mean 8.78 days SD 4.47 days Range 2‐14 days	Not stated	25
	No physical rehabilitation	25	Not stated	Mean 8.23 days SD 4.87 days Range 3‐13 days	Not stated	25
Chen L 2019	Motor relearning programme(Group A)	245	Not stated	Whole group: 0‐12 months	Whole group: Ischaemic	See comments
	Bobath approach (Group B)	243	Not stated			
Chen S 2021	Advanced practice nurse‐guided home‐based rehabilitation exercise programme (HREPro)	70	Not stated	Mean 3.41 months SD 0.79 months Not stated 11 participants	Infarction 25 Haemorrhage 34 Not stated 11	59
	Conventional rehabilitation	70	Not stated	Mean 3.23 months SD 0.82 months Not stated 8 participants	Infarction 27 Haemorrhage 35 Not stated 8	62
Chen G 2014	Task function training group	40	20/20	Whole group: 24h to 1 week	Not stated	40
	Strength training group	40	18/22		Not stated	40
Chen J 2014	Community‐level 3‐level rehabilitation therapy + conventional medical therapy	40	Not stated	Whole group ≤ 1.5 years	Cerebral infarction 29 Cerebral haemorrhage 11	40
	Community‐level rehabilitation therapy + conventional medical therapy	40	Not stated		Cerebral infarction 31 Cerebral haemorrhage 9	40
Chen P 2014	Functional exercise training	50	Not stated	Mean 66.26 days SD 23.74 days	Cerebral infarction 27 Cerebral haemorrhage 23	50
	Antispasmodic training	50	Not stated	Mean 65.80 days SD 24.20 days	Cerebral infarction 26 Cerebral haemorrhage 24	50
Chen Y 2011	Rehabilitation	33	Not stated	Not stated but participants were recruited 15‐30 days post‐stroke	Not stated	33
	No physical rehabilitation	33	Not stated	As above	Not stated	33
Cheng 2021	Three‐level rehabilitation treatment + conventional treatment	78	33/45	Mean 9.96 days SD 4.89 days	Basal ganglia 15 Other areas 63	78
	Conventional treatment	78	41/37	Mean 10.09 days SD 5.18 days	Basal ganglia 17 Other areas 61	78
Choi YK 2013	Proprioceptive neuromuscular facilitation (PNF) combination patterns and kinesio taping	15	6/9	Not stated	Not stated	15
	Neurodevelopmental treatment	15	7/8	Not stated	Not stated	15
Choi JU 2015	Task‐oriented training	10	4/6	Whole group > 6 months	Not stated	Not stated
	General physical therapy	10	4/6		Not stated	Not stated
Chu 2003	Rehabilitation	30	Whole group 32/26	Not stated	Whole group Haemorrhagic = 26 Ischaemic = 32	30
	Control (no treatment)	28	As above	Not stated		28
Cooke 2006	Additional conventional therapy (CPT+CPT)	35	13/22	Mean 32.43 days SD 21.29 days	Not stated	At 6 weeks, n = 32; At 3 month follow‐up, n = 28
	Functional strength training (FST +CPT)	36	12/24	Mean 33.86 days SD 16.50 days	Not stated	At 6 weeks, n = 36; At 3 month follow‐up, n = 29
	Conventional physiotherapy (CPT)	38	17/21	Mean 36.76 days SD 22.41 days	Not stated	At 6 weeks, n = 31; At 3 month follow‐up, n = 24
Dai 2015	Rehabilitation training + routine acupuncture	31	Not stated	Not stated	Not stated	31
	Routine acupuncture	31	Not stated	Not stated	Not stated	31
Dalal 2018	Prowling and proprioceptive training + routine physiotherapy	16	7/9	Not stated	Not stated	14
	Routine physiotherapy	16	8/8	Not stated	Not stated	15
Danlami 2017	Constraint‐induced movement therapy ‐ 4 tasks repeated 40 times (sCIMT)	7	2/3 Not stated 2	Mean 41.20 days SD 33.54 days	Ischaemic 4 Haemorrhagic 1 Not stated 2	5
	Constraint‐induced movement therapy ‐ 4 tasks repeated for 2 hours (tCIMT)	8	2/4 Not stated 2	Mean 74.67 days SD 55.93 days	Ischaemic 4 Haemorrhagic 2 Not stated 2	6
	Conventional therapy	7	2/5	Mean 75.50 days SD 47.88 days	Ischaemic 7 Haemorrhagic 0	7
Dean 2000	Motor learning	6	3/3	Mean 2.3 years SD 0.7 years	Not stated	5
	Placebo	6	4/2	Mean 1.3 years SD 0.9 years	Not stated	4
Dean 2006	Experimental	76	34/42	Mean 6.7 years SD 6.7 years Range 0.1‐24.8 years	Not stated	65
	Control	75	28/47	Mean 5.2 years SD 5.4 years Range 0.2‐25.1 years	Not stated	68
Deng 2011	Intervention	50	Not stated	Stroke onset to admission to hospital: ≤ 6 hours: 31 > 6 hours: 19	Not stated	50
	Control (no treatment)	50	Not stated	Stroke onset to admission to hospital: ≤ 6 hours: 30 > 6 hours: 20	Not stated	50
Ding 2015	Strength training group + routine treatment	50	27/23	Not stated	Whole group: Cerebral infarction	50
	Task function training group + routine treatment	50	26/24	Not stated		50
DOSE 2020	Determining Optimal Post‐Stroke Exercise (DOSE1) 1 hour/day	25	9/16	Mean 29 days SD 10 days	Cortical 6 Subcortical 19 Unknown 0	24
	Determining Optimal Post‐Stroke Exercise (DOSE2) 2 hours/day	25	15/10	Mean 27 days SD 10 days	Cortical 3 Subcortical 21 Unknown 1	25
	Usual care physical therapy	25	8/16	Mean 26 days SD 11 days	Cortical 5 Subcortical 16 Unknown 3	24
Du 2014	Intensive walking training	40	Not stated	Mean 14.6 days SD 5.6 days	Whole group: "acute cerebral infarction"	40
	Conventional rehabilitation	40	Not stated	Mean 15.1 days SD 5.3 days		40
Duan 2011	Task‐oriented training	10	Not stated	Mean (SD): 3.3 (1.4) months; range: 1‐5 months	Not stated	10
	Neurodevelopmental training (NDT)	10	Not stated	Mean (SD): 3.7 (2.7) months; range: 1‐8 months	Not stated	10
Duncan 1998	Mixed	10	4/6	Mean 66 days	Ischaemic = 10	10
	Control	10	4/5 + 1 brainstem	Mean 56 days	Haemorrhagic = 2 Ischaemic = 8	10
Duncan 2003	Mixed	50	18/22; 4 bilateral	Mean 77.5 days SD 28.7 days	Ischaemic = 39	44
	Control	50	22/22; 4 bilateral	Mean 73.5 days SD 27.1 days	Ischaemic = 44	48
Epple 2020	Vojta therapy	20	2/17 Neither 1	Whole group > 72 hours	Ischaemic stroke 20 Haemorrhagic stroke 0 Left MCA infarct 2 Right MCA infarct 17 Brainstem infarct 1 Other/multiple locations 0	20
	Standard physiotherapy	20	8/10 Neither 1 Not stated 1		Ischaemic stroke 19 Haemorrhagic stroke 1 Left MCA infarct 8 Right MCA infarct 10 Brainstem infarct 0 Other/multiple locations 1	19
Fan WK 2006	Treated	42	21/21	Mean 8.14 days SD 4.95 days	Haemorrhagic = 15 Ischaemic = 27	42
	Control (no treatment)	40	(unable to tell if data pertains to side of lesion or side of hemiplegia)	Mean 8.33 days SD 3.87 days	Haemorrhagic = 14 Ischaemic = 26	38
Fan Y 2015	Three‐stage rehabilitation	62	Not stated	Not stated	Brain haemorrhage 27 Cerebral infarction 35	Not stated
	Conventional treatment	62	Not stated	Not stated	Brain haemorrhage 29 Cerebral infarction 33	Not stated
Fan L 2014	Intense task training + early rehabilitation therapy	30	13/17	Whole group: ≤ 10 days	Whole group: acute cerebral infarction	30
	Early rehabilitation therapy	30	11/19			30
Fan WS 2006	Proprioceptive neuromuscular facilitation (PNF)	23	Not stated	Not stated	Ischaemic: 12 Haemorrhagic: 11	23
	Conventional rehabilitation	22	Not stated	Not stated	Ischaemic: 12 Haemorrhagic: 10	22
Fan X 2009	Exercise training	Whole group 80	Not stated	Not stated	Not stated	Insufficient information (abstract only)
	Conventional rehabilitation		Not stated	Not stated	Not stated	As above
Fang 2003	Additional early physiotherapy intervention	78	Not stated	Not stated	Haemorrhagic = 13 Cerebral infarct = 37	At day 30, n = 50 At 6 months, n = 12
	Routine therapy	78	Not stated	Not stated	Haemorrhagic = 11 Cerebral infarct = 67	At day 30, n = 78 At 6 months, n = 12
Fang YN 2004	Rehabilitation	25	Not stated	Not stated	As above	As above
	Control (no treatment)	33	Not stated	Not stated	As above	As above
Fang H 2010	Balance training	25	Not stated	Not stated but recruited within 3‐6 weeks post‐stroke	Cerebral infarction: 15 Haemorrhagic: 10	25
	Conventional rehabilitation	25	Not stated	As above	Cerebral infarction: 13 Haemorrhagic: 12	25
FeSTivaLS 2014	Functional Strength Training Lower Limb (FST‐LL)	27	13/12	Mean 24.4 months SD 13.7 months	LACS 9 PACS 9 TACS 6 POC 1 Haemorrhage 0	23
	Functional Strength Training Upper Limb (FST‐UL)	25	12/15	Mean 24.4 months SD 16.6 months	LACS 6 PACS 12 TACS 3 POCS 3 Haemorrhage 3	27
Frimpong 2014	Task‐oriented circuit training	10	4/6	Mean 2.2 months SD 0.8 months	Ischaemic 4 Haemorrhagic 2 Not stated 4	10
	Conventional therapy	10	3/7	Mean 2.4 months SD 0.9 months	Ischaemic 2 Haemorrhagic 1 Not stated 7	10
Ge W 2003	Rehabilitation	20	Not stated	Mean 50 days SD 22 days	Not stated	Unclear ‐ see notes in characteristics of included studies
	Control (no treatment)	28	Not stated	Mean 51 days SD 26 days	Not stated	Unclear ‐ see notes in characteristics of included studies
Ge Y 2020	Compulsory (Constraint) Exercise Therapy	48	Not stated	3‐11 years	Not stated	48
	Conventional exercise therapy	48	Not stated	3‐10 years	Not stated	48
Gelber 1995	Neurophysiological (NDT)	15	8/7	Mean 11.3 days SEM 1.1 days	Pure motor ischaemic = 15	15
	Orthopaedic (TFR)	12	5/7	Mean 13.8 days SEM 2.7 days	Pure motor ischaemic = 12	12
Ghasemi 2018	Functional stretching exercises	15	9/6	Mean 29.13 months SD 19.32 months	Ischaemic 5 Haemorrhagic 10	15
	Routine physical therapy	15	7/8	Mean 18.60 months SD 16.50 months	Ischaemic 4 Haemorrhagic 11	13
Gong Y 2009	Bilateral training	40	Not stated	Not stated	Not stated	40
	Unilateral training	40	Not stated	Not stated	Not stated	40
Green 2002	Mixed	85	56/26 + 3 other	Not stated	Not stated	81
	Control (no treatment)	85	44/40 + 1 other	Not stated	Not stated	80
Guan 2017	Motor relearning training	32	Not stated	Mean 6.00 days SD 2.59 days	All ischaemic stroke Basal ganglia 21 Cerebral cortex 7 Other 4	32
	Conventional rehabilitation training	32	Not stated	Mean 6.31 days SD 2.32 days	All Ischaemic stroke Basal ganglia 18 Cerebral cortex 12 Other 2	32
Guo L 2012	Rehabilitation	46	29/17	Mean 2.2 months SD 1.9 months	Ischaemic 28 Haemorrhagic 8	37
	Standard care	45	27/18	Mean 2.3 months SD 1.6 months	Ischaemic 29 Haemorrhagic 16	37
Guo L 2013	Task‐oriented training combined with muscle strength training	21	7/14	Mean 10.1 months SD 2.4 months	Ischaemic 13 Haemorrhagic 8	21
	Facilitation techniques	21	8/13	Mean 11.2 months SD 3.1 months	Ischaemic 14 Haemorrhagic 7	21
Guo Z 2015	Muscle strength exercises and lower limb balance training	40	Not stated	Not stated	Not stated	40
	Routine post‐stroke rehabilitation guidance	40	Not stated	Not stated	Not stated	40
Haral 2014	Sensorimotor Integration	Whole group: 30	Not stated	Whole group: 6 months ‐ 3 years	Not stated	Not stated
	Conventional training		Not stated		Not stated	Not stated
Harjpal 2021	Lower limb bilateral training	20	Whole group: Not stated	Whole group: Not stated	Whole group: Not stated	20
	Lower limb training to the affected side only	20				20
Hendrey 2018	Ballistic strength training	15	10/5 Bilateral 0	Median 36 days Q1‐Q3 23‐79 days	Study included ischaemic or haemorrhagic stroke infarct 10 Not stated 5	Completed intervention 12 Completed assessment 15
	Standard therapy	15	7/7 Bilateral 1	Median 56 days Q1‐Q3 45‐94 days	Study included ischaemic or haemorrhagic stroke infarct 8 Not stated 7	Completed intervention 14 Completed assessment 15
Holmgren 2006	Intervention	15	Not stated	Mean 139.7 days SD 37.3 days	Cardioembolic stroke = 4 Lacunar infarct = 2 Other specified stroke = 2 Unknown stroke = 6 Not applicable (because of intracerebral haemorrhage) =1	15
	Control	19	Not stated	Mean 126.8 days SD 28.2 days	Large artery thrombosis = 4 Cardioembolic stroke = 5 Lacunar infarct = 8 Unknown stroke = 2	19
Hong Cuicui 2016	Additional therapy	68	Not stated	Mean 10.21 days SD 2.61 days	Cerebral infarction: 50 Haemorrhagic: 18	68
	Conventional rehabilitation	68	Not stated	Mean 10.31 days SD 2.51 days	Cerebral infarction: 48 Haemorrhagic: 20	68
Hong Hye Jin 2012	Three‐stage rehabilitation	40	Not stated	Not stated	Cerebral infarction: 28 Haemorrhagic: 12	40
	Conventional rehabilitation	40	Not stated	Not stated	Cerebral infarction: 29 Haemorrhagic: 11	40
Hoseinabadi 2013	Physiotherapy	12	Not stated	Mean 31.5 months SD 11.85 months	Not stated	Not stated
	No treatment	12	Not stated	Mean 30.91 months SD 12.66 months	Not stated	Not stated
Hou 2006	Rehabilitation	40	Not stated	Mean 9.05 days SD 5.74 days	Haemorrhagic = 12 Ischaemic = 28	40
	Control (no treatment)	40	Not stated	Mean 8.65 days SD 5.38 days	Haemorrhagic = 12 Ischaemic = 28	40
Hou Zhi 2014	Three‐level rehabilitation training	58	33/25	Mean 44.5 days SD 18.4 days	Brain haemorrhage 19 Cerebral infarction 39	58
	Conventional rehabilitation training	58	32/26	Mean 45.3 days SD 16.4 days	Brain haemorrhage 18 Cerebral infarction 40	58
Howe 2005	Mixed	17	8/9	Mean 26.5 days SD 15.7 days	2 TACS / 7 PACS / 4 LACS / 1 POCS / 3 other	15
	Control (neurophysiological)	18	7/11	Mean 23.1 days SD 17.5 days	3 TACS / 6 PACS / 4 LACS / 3 POCS / 2 other	18
Hu 2007	Test (haemorrhagic group)	178	Not stated	Whole group Mean 11 days SD 6 days	Not stated	At 1 month after stroke n = 178; at 3 months after stroke, n = 178; at 6 months after stroke, n = 177
	Control (no treatment)	174	Not stated	As above	Not stated	At 1 month after stroke n = 174; at 3 months after stroke, n = 168; at 6 months after stroke, n = 168
Huang 2003	Rehabilitation	25	14/11	Mean 6.45 days SD 3.70 days	Haemorrhagic = 5 Ischaemic = 20	25
	Control (no treatment)	25	12/13	Mean 6.89 days SD 3.20 days	Haemorrhagic = 5 Ischaemic = 20	25
Huang 2014	Bobath	17	Not stated	Mean 4.14 days SD 2.61 days	Not stated	17
	Rood	17	Not stated	Mean 4.40 days SD 2.84 days	Not stated	17
	Brunnstrom	17	Not stated	Mean 4.64 days SD 2.41 days	Not stated	17
	PNF	17	Not stated	Mean 4.34 days SD 2.31 days	Not stated	17
	Basic treatment (control)	17	Not stated	Mean 4.74 days SD 2.91 days	Not stated	17
Huang Yangfang 2016	Three‐stage rehabilitation	28	Not stated	Not stated	Not stated	28
	No physical rehabilitation	27	Not stated	Not stated	Not stated	27
Hui‐Chan 2009	PLBO + TRT	25	Not stated	Whole group mean 4.7 years SD 3.4 years	Not stated	25
	Control (no treatment)	29	Not stated	As above	Not stated	29
Imhof 2015	Mobility Enhancing Nursing Intervention	70	Not stated	Not stated	MS 27 Stroke 41 Traumatic brain injury 2	Finished intervention 70 Analysed 61
	Standard care	70	Not stated	Not stated	MS 32 Stroke 35 Traumatic brain injury 2	Finished intervention 70 Analysed 65
Indurkar 2013	Task oriented activities + physiotherapy	15	Not stated	Not stated	Not stated	Not stated
	Physiotherapy	15	Not stated	Not stated	Not stated	Not stated
Jandaghi 2021	Visual deprivation‐stable based training	15	9/6	Mean 15.4 months SD 4.7 months	Ischaemic 8 Haemorrhagic 7	15
	Unstable base training	15	8/7	Mean 14.4 months SD 4.5 months	Ischaemic 7 Haemorrhagic 8	15
	General physiotherapy exercise (control)	15	10/5	Mean 14.0 months SD 6.3 months	Ischaemic 7 Haemorrhagic 8	15
Jeon 2018	Bilateral lower limb strengthening exercise	10	6/4	Mean 15.4 months SD 1.6 months	Ischaemic 5 Haemorrhagic 5	10
	Unilateral therapy (paretic lower limb)	10	2/8	Mean 15.3 months SD 1.5 months	Ischaemic 2 Haemorrhagic 8	10
Ji Pei 2014	Bobath technique‐based exercise therapy	48	Not stated	Whole group: 15‐60 days	Whole group ‐ site: Basal ganglia 54 Frontal lobe 22 Parietal lobe 9 Temporal lobe 5	48
	Routine treatment (no physical rehabiltation)	42	Not stated			42
Jing 2006	Exercise and occupational therapy	120	Whole group 73/87	Mean 5.2 days SD 4.2 days	Whole group Haemorrhagic = 66 Ischaemic = 94	120
	Exercise therapy	40	As above	Mean 4.6 days SD 3.7 days	As above	40
Jongbloed 1989	Sensorimotor Integration	43	Whole group: 44/46	Whole group: Mean 40 days SD 42 days	Not stated	Not stated
	Functional training	47			Not stated	Not stated
Khallaf 2014	Task specific exercises, gait training, and visual biofeedback	8	5/3	Mean 9.88 months SD 2.80 months	Whole group: Ischaemic stroke	Not stated
	Traditional physical therapy program	8	4/4	Mean 10.00 months SD 2.39 months		Not stated
Kim 2007	Task‐related circuit training	12	3/9	Mean 6.8 months SD 3.1 months	Not stated	12
	Conventional physical therapy	12	5/4 3 unknown	Mean 8.5 months SD 7.3 months	Not stated	9
Kim 2012	Experimental	10	Not stated	Mean 7.7 years SD 6.11 years	Not stated	10
	Control	10	Not stated	Mean 13.1 years SD 10.62 years	Not stated	10
Kim 2012a	Rhythmic auditory stimulation gait training group	10	5/5	Mean 5.68 months SD 1.04 months	Infarction 6 Haemorrhage 4	9
	Conventional physical therapy	10	3/7	Mean 4.76 months SD 2.65 months	Infarction 2 Haemorrhage 8	9
Kim 2014	Gross motor group exercise group plus conventional	14	10/4	Mean 11.4 years SD 2.7 years	Not stated	14
	Conventional therapy (morning bed exercise)	14	8/6	Mean 9.4 years SD 3.1 years	Not stated	14
Kim 2016	Inpatient circuit training program	10	4/6	Mean 30.1 days SD 21.8 days	Ischaemic 8 Haemorrhagic 2	10
	individual neurodevelopmental physiotherapy	10	5/5	Mean 29.9 days SD 20.3 days	Ischaemic 8 Haemorrhagic 2	10
Kim 2017	Task‐oriented circuit training	15	8/7	Mean 3.3 months SD 1.3 months	Infarct 6 Haemorrhage 9	15
	Conventional physical therapy	15	10/5	Mean 4.4 months SD 1.6 months	Infarct 7 Haemorrhage 8	15
Kim 2018	Coordinative locomotor training	7	3/4	Mean 33.85 days SD 29.61 days	Ischaemic 4 Haemorrhage 3	7
	Conventional neurodevelopment treatment	6	3/3	Mean 29.16 days SD 34.86 days	Ischaemic 4 Haemorrhage 2	6
Kim 2021	Task‐specific training after cognitive sensorimotor exercise	13	7/6	Mean 12.07 months SD 3.57 months	Infarct 6 Haemorrhage 7	13
	Task‐specific training	12	6/6	Mean 13.17 months SD 3.90 months	Infarct 6 Haemorrhage 6	12
	Conventional physical therapy	12	5/7	Mean 11.83 months SD 3.71 months	Infarct 8 Haemorrhage 4	12
Knox 2018	Task‐oriented training	51	23/25	Mean 8 weeks SD 7 weeks	Whole group: first ischaemic or Haemorrhagic stroke. Numbers not stated	45
	Strength training of lower extremities	45	26/19	Mean 9 weeks SD 7 weeks		40
	No physical therapy	48	25/26	Mean 10 weeks SD 8 weeks		43
Koç 2015	Home‐based exercise	Whole group: 134	Not stated	Whole group: Not stated	Whole group: ischaemic stroke	35
	Usual care (no treatment)		Not stated			37
Krawczyk 2014	Closed chain exercises	Whole group: 51	Whole group: 15/36	Whole group: Mean 39.6 days SD 17.0 days	Whole group: first ischaemic stroke	Not stated
	Open chain exercises					Not stated
Krukowska 2016	Bobath – Neurodevelopmental Treatment (NDT‐Bobath)	38	17/21	Whole group: ≤ 6 months	Whole group: ischaemic stroke	38
	Proprioceptive Neuromuscular Facilitation (PNF)	34	17/17			34
Kuberan 2017	Task oriented training	13	Not stated	Mean 11.2 months SD 4.2 months	Ischaemic 9 Haemorrhagic 4	Not stated
	Conventional physical therapy	13	Not stated	Mean 10.9 months SD 3.7 months	Ischaemic 5 Haemorrhagic 8	Not stated
Kumaran 2015	Task and context based exercise program	34	13/18	Median 6 months Q1‐Q3 3‐18 months	Ischaemic 22 Haemorrhagic 9	31
	Conventional training	32	15/16	Median 6 months Q1‐Q3 3‐14 months	Ischaemic 21 Haemorrhagic 10	31
Kunkel 2013	Balance training	7	2/2 Bilateral 2 Not stated 1	Whole group (including excluded group): 4.6 weeks (min 1 max 14 weeks)	TACI 1 PACI 2 POCI 2 LACI 1 PICH 1	7
	Usual care	7	4/2 Bilateral 1		TACI 0 PACI 1 POCI 2 LACI 4 PICH 0	7
Kwakkel 2002	Lower extremities	17	7/10	Mean 4.8 weeks SD 3.1 weeks Range 2‐9 weeks	TACI = 6 PACI = 10 LACI = 1	17
	Upper extremities	18	8/10	Mean 5.9 weeks SD 3 weeks Range 2‐10 weeks	TACI = 8 PACI = 7 LACI = 3	18
	Control	18	7/11	Mean 7.3 weeks SD 3.6 weeks Range 2‐10 weeks	TACI = 9 PACI = 6 LACI = 3	18
Kwakkel 2008	Circuit training	126	49/57 Brainstem = 6 Cerebellum = 14	Mean 91 days SD 42 days	Haemorrhagic = 23 Ischaemic = 103	125
	Usual physiotherapy	124	43/61 Brainstem = 14 Cerebellum = 6	Mean 103 days SD 51 days	Haemorrhagic = 24 Ischaemic = 100	117
Langhammer 2000	Neurophysiological (Bobath)	28	17/11	Not stated	Not stated	24
	Motor learning	33	17/16	Not stated	Not stated	29
Langhammer 2007	Intensive exercise	35	16/19	Not stated	"Cause of the stroke was thrombosis or embolism with 29 such cases in the intensive exercise group & 6 being haemorrhages"	32
	Regular exercise	40	21/19	Not stated	"Cause of the stroke was thrombosis or embolism with 36 such cases in the regular exercise group and 4 being haemorrhages"	32
LAST 2018	Individualised coaching	186	Not stated	Mean 111.3 days SD 24.5 days	Infarction 172 Haemorrhage 14	144
	Usual care	194	Not stated	Mean 112.0 days SD 17.2 days	Infarction 174 Haemorrhage 20	185
Lawal 2016	Circuit training	21	15/6	Mean 12.9 months SD 10.8 months	Ischaemic 18 Haemorrhagic 3	20
	Standard physiotherapy	23	12/11	Mean 12.5 months SD 10.5 months	Ischaemic 19 Haemorrhagic 4	21
Lee 2015	Combined aerobic and resistance exercise	15	6/8 Not stated 1	Mean 5.98 years SD 3.27 years	Infarct 7 Haemorrhage 7 Not stated 1	14
	Unsystematic physical activities or played Korean chess	15	5/7 Not stated 3	Mean 5.83 years SD 2.51 years	Infarct 6 Haemorrhage 6 Not stated 3	12
Lee 2018	Conventional rehabilitation followed by caregiver‐mediated Exercise	40	18/17	Mean 10.0 days SD 1.8 days	Ischaemic 22 Haemorrhagic 13	35
	Conventional rehabilitation followed by no treatment	40	16/21	Mean 10.0 days SD 1.9 days	Ischaemic 25 Haemorrhagic 12	37
Lennon 2006	Bobath	30	Not stated	Not stated	Not stated	30
	Gait specific group	31	Not stated	Not stated	Not stated	31
Letombe 2010	Early post‐stroke physical exercises	9	4/5	Mean 21 days SD 3 days	Ischaemic stroke 5 Haemorrhagic 4	Not stated
	Conventional rehabilitation	9	4/5	Mean 20 days SD 2 days	Ischaemic stroke 5 Haemorrhagic 4	Not stated
Li 1999	Early rehabilitation	30	Not stated	Not stated	Haemorrhagic = 12 Ischaemic = 18	30
	Control (no treatment)	31	Not stated	Not stated	Haemorrhagic = 12 Ischaemic = 19	31
Li 2005	Motor relearning	31	Not stated	Mean 8.8 days SD 6.0 days	Not stated	31
	Neurodevelopmental therapy	30	Not stated	Mean 8.3 days SD 5.3 days	Not stated	30
Li 2013	Early intensive walking basic skills training	88	41/47	Whole group: < 4 weeks	Cerebral infarction 36 Cerebral haemorrhage 43 Mixed stroke 9	88
	Conventional rehabilitation training	88	44/44		Cerebral infarction 37 Cerebral haemorrhage 39 Mixed stroke11 Not stated 1	88
Li Jingqian 2013	Motor re‐learning programme (MRP)	60	Not stated	Not stated	Ischaemic 46 Haemorrhagic 14	60
	Conventional rehabilitation	60	Not stated	Not stated	Ischaemic 45 Haemorrhagic 15	60
Li Weiwei 2015	Task‐oriented training	30	Not stated	Mean 40.47 days SD 18.23 days	Cerebral infarction 14 Cerebral haemorrhage 7 Cerebral trauma 9	30
	Routine rehabilitation	30	Not stated	Mean 37.87 days SD 13.12 days	Cerebral infarction 17 Cerebral haemorrhage 9 Cerebral trauma 4	30
Li Xiaojun 2016	Task‐oriented training	37	25/12	Mean (SD): 11.4 (3.1) months; range: 5‐23 months	Ischaemic: 31 Haemorrhagic: 6	37
	Conventional rehabilitation	37	23/14	Mean (SD): 10.9 (3.8) months; range; 6‐25 months	Ischaemic: 29 Haemorrhagic: 8	37
Li Yuanzheng 2014	Bobath therapy	60	Not stated	Not stated	Not stated	60
	Conventional treatment	62	Not stated	Not stated	Not stated	62
Li Yuanzheng 2014a	Motor relearning	28	Not stated	Whole group: < 3 months	Not stated	28
	Routine rehabilitation training	30	Not stated		Not stated	30
Lincoln 2003	Neurophysiological (Bobath)	60	30/29; 1 bilateral	Inclusion criteria: Stroke less than 2 weeks previously	9 TACS / 29 PACS / 14 LACS / 4 POCS / 4 unsure	At 1 month ‐ 52
	Motor learning	60	31/27; 2 bilateral	Inclusion criteria: Stroke less than 2 weeks previously	8 TACS / 32 PACS / 11 LACS / 6 POCS / 3 unsure	At 1 month ‐ 47
Lindvall 2014	Body awareness therapy	24	11/13	Mean 4.1 years SD 3.8 years Range 0‐15 years	Intracerebral infarction 16 Intracerebral haemorrhage 8	21
	No intervention	22	15/7	Mean 4.2 years SD 4.5 years Range 0‐15 years	Intracerebral infarction 18 Intracerebral haemorrhage 4	22
Liu 2014	Task oriented training	40		Mean 9.21 months SD 1.01 months	16 I 24 H	Not stated
	Conventional rehabilitation	40	24/10 6 bilateral	Mean 9.31 months SD 0.89 months	17 I 23 H	Not stated
Liu Xuan 2016	Three‐stage rehabilitation	30	Not stated	Mean 9. 12 days SD 0.78 days	Ischaemic: 30	30
	Conventional rehabilitation	30	Not stated	Mean 9. 34 days SD 0. 67 days	Ischaemic: 30	30
Liu Yanhua 2020	Compulsory exercise therapy	42	18/24	Not stated	Not stated	42
	Routine exercise therapy	42	25/17	Not stated	Not stated	42
Lu 2004	Three‐stage rehabilitation	21	Not stated	Whole group: 1‐15 days	Whole group: Cerebral infarction 22 Cerebral haemorrhage 20	Not stated
	Standard care	21	Not stated			Not stated
Lu 2014	Three‐stage rehabilitation	90	Not stated	Mean 9.9 days SD 4.7 days	Cerebral infarction 65 Cerebral haemorrhage 25	Not stated
	Standard care	90	Not stated	Mean 9.8 days SD 4.8 days	Cerebral infarction 67 Cerebral haemorrhage 23	Not stated
Lu Liangyan 2014	Three‐level rehabilitation treatment	44	Not stated	Not stated	Whole group: cerebral haemorrhage (1st occurrence)	44
	Routine rehabilitation guidance	44	Not stated	Not stated		44
Ma Xue 2010	Rehabilitation	31	Not stated	Mean 37.29 days SD 17.07 days	Ischaemic: 18 Haemorrhagic: 14	31
	Attention control	29	Not stated	Mean 36.72 days SD 12.12 days	Ischaemic: 13 Haemorrhagic: 15	29
Mai Guanghuai 2016	Lower limb training	45	21/24	Mean: 48.9 days SD: 9.2 days Range: 21‐68 days	Ischaemic: 28 Haemorrhagic: 17	45
	Conventional rehabilitation	45	26/19	Mean: 47.7 days SD: 9.5 days Range: 25‐74 days	Ischaemic: 30 Haemorrhagic: 15	45
Mansfield 2018	Perturbation‐based balance training	44	19/22 Not stated 3	Median 2.0 years IQR 3.3 years Not stated 3	Not stated	41
	Keep Moving with Stroke programme (balance and mobility exercise programme for community‐dwelling individuals)	44	20/22 Not stated 2	Median 3.2 years IQR 4.5 years Not stated 2	Not stated	42
Marigold 2005	Agility exercise	30	10/11 Not applicable 1	Mean 3.6 years SD 1.8 years	Cortical 4 Subcortical 7 Brainstem/cerebellum 6 Cortical‐subcortical 0 Unknown 5	22
	Stretching/weight‐shifting exercise	31	8/18	Mean 3.8 years SD 2.4 years	Cortical 10 Subcortical 8 Brainstem/cerebellum 4 Cortical‐subcortical 0 Unknown 4	26
Martins 2020	Task‐specific circuit training	18	10/8	Median 52 months IQR 64 months	Ischaemic 7 Haemorrhagic 5 Unknown 6	15
	Global stretching, memory exercises, and education sessions	18	9/9	Median 41 months IQR 39 months	Ischaemic 11 Haemorrhagic 5 Unknown 2	13
Matthew Hall 2013	Task‐oriented training	20	12/8	Mean 87 days SD 9.72 days	Cerebral infarction 10 Cerebral haemorrhage 10	Not stated
	Standard rehabitation	20	14/6	Mean 90 days SD 6.81 days	Cerebral infarction 8 Cerebral haemorrhage 12	Not stated
McClellan 2004	Motor learning	15	8/5 (at end of intervention)	Median 6.5 months IQR 5.5 months	Not stated	13
	Placebo (upper limb control)	11	3/6; 1 bilateral (at end of intervention)	Median 4.5 months IQR 3.0 months	Not stated	10
Medina‐Rincón 2019	Balance impairment exercise programme	7	5/2	Whole group: ≤ 3 months	Ischaemic stroke 6 Haemorrhagic stroke 1	Not stated
	Usual rehabilitation	7	3/4		Ischaemic stroke 6 Haemorrhagic stroke 1	Not stated
Meier 2021	Coordinative training treatment group	3	Not stated	Whole group: Not stated	Not stated	Not stated
	Conventional physical therapy	4	Not stated		Not stated	Not stated
Mendoza 2015	Task‐oriented circuit class	9	6/3	Mean 38.7 months SD 17.5 months	Not stated	9
	Impairment‐focused circuit class	9	7/2	Mean 59 months SD 55.4 months	Not stated	9
Meng 2022	Robot‐assisted gait training (not relevant to this review)	64	28/34	Whole group: Not stated	Whole group: Ischaemic stroke	62
	Enhanced lower limb therapy	64	26/38			64
	Conventional rehabilitation	64	30/10			61
Meng Fanda 2021	Routine rehabilitation training	20	Not stated	Not stated	Cerebral infarction 14 Cerebral haemorrhage 6	20
	Routine rehabilitation nursing	20	Not stated	Not stated	Cerebral infarction 13 Cerebral haemorrhage 7	20
Meng Qingling 2015	Task functional training	45	23/22	Not stated	Whole group: Cerebral infarction	45
	Strength training	45	25/20	Not stated		45
Mikolajewska 2017	Bobath neuro‑developmental treatment (NDT‐Bobath)	15	7/8	Mean 52.25 weeks SD 13.47 weeks	Whole group: Ischaemic stroke	15
	Traditional treatment	15	8/7	Mean 57.21 weeks SD 16.02 weeks		15
Mohaideen 2014	Symmetrical weight bearing + conventional stroke training	15	Not stated	Whole group: < 6 months	Not stated	Not stated
	Conventional stroke training	15	Not stated		Not stated	Not stated
Moore 2016	Community group exercise programme	20	Not stated	Whole group: Mean 19 months SD 26 months Range 6‐144 months	Not stated	20
	Matched duration home stretching programme	20	Not stated		Not stated	20
Morreale 2016	Proprioceptive Neuromuscular Facilitation (early)	110	74/36	Mean 17 hours SD 2 hours	Whole group: sub‐cortical ischaemic stroke in MCA	Whole group: 38 dropouts at 3 months follow‐up time point
	Cognitive therapeutic exercise (early)	60	75/35	Mean 17 hours SD 3 hours		
	Proprioceptive Neuromuscular Facilitation (standard)	110	41/19	Mean 18 hours SD 1 hour		
	Cognitive therapeutic exercise (standard)	60	40/20	Mean 17 hours SD 1 hour		
Mudge 2009	Exercise	31	11/20	Median 3.33 years Range 0.6‐13.3 years	Not stated	At 3 months follow‐up ‐ 27
	Control	27	12/14 1 brainstem	Median 5.8 years Range 0.5‐18.7 years	Not stated	At 3 months follow‐up ‐ 23
Mustafaoğlu 2018	Body weight support treadmill training (BWSTT)	15	8/7	Median 12.0 months Range 7‐18 months	Ischaemic 8 Haemorrhagic 7	15
	BWSTT + conventional therapy	15	6/9	Median 12.5 months Range 3‐36 months	Ischaemic 7 Haemorrhagic 8	15
Nagy 2017	Conductive education	11	5/4 Not stated 2	3‐6 months: 3 >6 months: 6 Not stated 2	Not stated	9
	Conventional physiotherapy	9	4/4 Not stated 1	3‐6 months: 2 >6 months: 6 Not stated 1	Not stated	8
Ni 1997	Comprehensive rehabilitation training	34	Not stated	Mean 19.21 days SD 7.59 days	Ischaemic = 19 Haemorrhagic = 15	34
	Control (no treatment)	34	Not stated	Mean 18.31 days SD 9.64 days	Ischaemic = 20 Haemorrhagic = 14	34
Nindorera 2022	Physical rehabilitation (IG)	23	15/8	Median 24 months Q1‐Q3 11‐37 months	11 I 11 H 1 Undetermined	23
	Attention control (DG)	23	10/13	Median 21 months Q1‐Q3 9‐32 months	8 I 9 H 6 undetermined	23
Outermans 2010	High‐intensity task‐oriented training	23	11/11 Not stated 1	Mean 22.5 days SD 8.2 days	Not stated	17
	Low‐intensity physiotherapy	21	10/11	Mean 23.5 days SD 7.8 days	Not stated	15
Pan 2004	Rehabilitation	48	26/22	Not stated	Ischaemic = 30 Haemorrhagic = 18	48
	Control	48	22/26	Not stated	Ischaemic = 32 Haemorrhagic = 16	48
Pandian 2014	Motor training of both sides	20	9/11	Mean 12.72 months SD 8.26 months	Ischaemic 13 Haemorrhagic 6 Frontoparietal 4 Basal ganglia 2 Thalamic 2 Multiple areas 10 Others 2	17
	Conventional (standard motor rehabilitation based on Brunnstrom’s movement therapy)	19	11/8	Mean 13.32 months SD 6.68 months	Ischaemic 9 Haemorrhagic 11 Frontoparietal 6 Basal ganglia 4 Thalamic 2 Multiple areas 6 Others 1	18
Pang 2003	Rehabilitation	50	Not stated	Not stated	Haemorrhagic = 21 Infarction = 29	50
	Control (no treatment)	36	Not stated	Not stated	Haemorrhagic = 15 Infarction = 21	36
Pang 2005	Community‐based Fitness and Mobility Exercise (FAME) Program	32	13/19	Mean 5.2 years SD 5.0 years	Ischaemic stroke 18 Not stated 14	30
	Seated upper extremity program	31	9/22	Mean 5.1 years SD 3.6 years	Ischaemic stroke 19 Not stated 12	30
Pang 2006	Treatment	41	Not stated	Not stated	Not stated	41
	Control (no treatment)	39	Not stated	Not stated	Not stated	37
Pang 2018	Single‐Task Exercise	28	12/16	Mean 66.6 months SD 41.6 months	Ischaemic 18 Haemorrhagic 10 TAC 0 PAC 18 LAC 1 POC 9	26
	Upper‐limb exercise group	28	13/15	Mean 87.5 months SD 83.3 months	Ischaemic 14 Haemorrhagic 14 TAC 0 PAC 23 LAC 0 POC 5	26
Park 2021	Affected side cross‐training	20	10/5 Not stated 5	Mean 8.73 months SD 4.99 months Not stated 5	Ischaemic 7 Haemorrhage 8 Not stated 5	15
	Unaffected side cross‐training	20	8/10 Not stated 2	Mean 13.50 months SD 6.81 months Not stated 2	Ischaemic 8 Haemorrhage 10 Not stated 2	18
	General neurological physiotherapy	20	10/9 Not stated 1	Mean 13.16 months SD 6.43 months Not stated 1	Ischaemic 10 Haemorrhage 9 Not stated 1	19
Pirayesh 2021	Otago exercises group	23	Not stated	Whole group: > 6 months	Whole group: Ischaemic stroke	Not stated
	No treatment	23	Not stated			Not stated
Puckree 2014	Balance and stability exercise program	25	12/13	6‐12 months 12 0‐6 months 9 Not stated 4	Whole group: Not stated ‐ participants unaware of type of stroke	25
	Regular physiotherapy program	25	14/11	6‐12 months 10 0‐6 months 12 Not stated 3		25
Qian 2004	Treatment	23	Not stated	Mean 13.9 days SD 8.5 days	Ischaemic = 15 Haemorrhagic = 8	23
	Control (no treatment)	19	Not stated	Mean 12.1 days SD 9.7 days	Ischaemic = 14 Haemorrhagic = 5	19
Qin 2013	Motor re‐learning programme (MRP)	20	Not stated	Mean 4.08 months SD 1.39 months	Not stated	Not stated
	Motor re‐learning programme (MRP) + acupuncture	20	Not stated	Mean 4.20 months SD 1.33 months	Not stated	Not stated
	Acupuncture	20	Not stated	Mean 4.38 months SD 1.31 months	Not stated	Not stated
Qin JianJian 2014	Bobath treatment	30	Not stated	Mean 4.48 months SD 1.35 months	Cerebral infarction 15 Intracerebral haemorrhage 15	30
	Conventional rehabilitation training	31	Not stated	Mean 4.76 months SD 1.27 months	Cerebral infarction 14 Intracerebral haemorrhage 17	31
Rahayu 2020	Neurorestoration	34	15/17 Not stated 2	Whole group: 24 hours post attack	Whole group: Ischaemic stroke	34
	Conventional	33	20/12 Not stated 1			33
Renner 2016	Group task training	34	19/15	Mean 39 days SD 25 days	Ischaemic 22 Haemorrhagic 12	29
	Individual task training	39	17/22	Mean 32 days SD 11 days	Ischaemic 31 Haemorrhagic 8	35
ReTrain 2018	Community‐based rehabilitation training (ReTrain)	23	Not stated	≤3 months 1 >3 months 22	Ischaemic 15 Haemorrhagic 3 Both 0 Not stated 5	Received intervention 20 Assessed at 6 months 21
	No training (exercise after stroke advice booklet)	22	Not stated	≤3 months 0 >3 months 22	Ischaemic 15 Haemorrhagic 1 Both 1 Not stated 5	Received intervention 20 Assessed at 6 months 19
Richards 1993	Mixed (early)	10	2/8	Mean 8.3 days SD 1.4 days	Canadian Stroke Score (maximum score = 15) Mean 5.3 SD 1.4	9
	Neurophysiological (early)	8	6/2	Mean 8.8 days SD 1.5 days	Canadian Stroke Score (maximum score = 15) Mean 5.2 SD 1.7	6
	Neurophysiological (conventional)	9	3/6	Mean 13.0 days SD 2.8 days	Canadian Stroke Score (maximum score = 15) Mean 6.0 SD 1.8	8
Salbach 2004	Motor learning	44	27/17	Mean 239 days SD 83 days	Mild gait deficit = 19 Moderate = 17 Severe = 8	41
	Placebo (upper limb control)	47	24/22; 1 bilateral	Mean 217 days SD 73 days	Mild gait deficit = 17 Moderate = 20 Severe = 10	43
Sekhar 2013	Isokinetic Strength Training and Balance Exercises	20	Not stated	Not stated	Not stated	20
	Conventional physiotherapy	20	Not stated	Not stated	Not stated	20
Seo 2015	Ramp gait exercise + proprioceptive neuromuscular facilitation (PNF) gait pattern traning	15	6/4 Not stated 5	Mean 14.1 months SD 7.0 months	Whole group: cerebral haemorrhage	10
	PNF gait pattern training	15	5/5 Not stated 5	Mean 12.2 months SD 5.2 months		10
Severinsen 2014	Resistance training	14	6/8	Median 19 months Range 8‐36 months	Whole group: Ischaemic stroke	14
	Low‐intensity sham training	17	11/5 Not stated 1	Median 16 months Range 9‐38 months		16
Shin 2011	Combined exercise	11	8/3	Not stated	Not stated	11
	Conventional exercise	10	5/5	Not stated	Not stated	10
Shuai 2013	Motor relearning programme	33	Not stated	Mean 8.7 days SD 4.7days	Cerebral infarction 29 Haemorrhage 4	33
	Routine rehabilitation therapy	29	Not stated	Mean 9.2 days SD 5.2 days	Numbers reported are erroneous (total 250)	29
Signal 2014	Strength and task‐specific training (STT)	5	2/3	Whole group: Average 42.85 months Range 5‐152 months	Not stated	5
	Strength training (PRST)	5	2/3		Not stated	4
	Task‐specific training (TST)	5	2/3		Not stated	5
	Usual care (UCC)	5	1/4		Not stated	5
Song 2015	Conventional physiotherapy	10	Not stated	Mean 36.67 months SD 15.12 months	Not stated	Not stated
	Individual‐based task‐oriented circuit training + conventional physiotherapy.	10	Not stated	Mean 30.70 months SD 14.68 months	Not stated	Not stated
	Class‐based task‐oriented circuit training + conventional physiotherapy.	10	Not stated	Mean 27.66 months SD 19.35 months	Not stated	Not stated
SPIRES 2022	Functional standing frame programme plus usual physiotherapy	22	Not stated	Median 10.0 days Range 0‐31 days IQR 11 days	Whole group: Not stated ‐ cerebral haemorrhage or infarct	18
	Usual physiotherapy	23	Not stated	Median 6.0 days Range 0‐36 days IQR 10 days		22
Stephenson 2004	Body Weight Support Treadmill Training	6	Not stated	Not stated	Not stated	6
	Proprioceptive Neuromuscular Facilitation‐PNF training	6	Not stated	Not stated	Not stated	6
	Control (no treatment)	6	Not stated	Not stated	Not stated	6
Stuart 2019	Adaptive Physical Activity exercise programmefor stroke survivors (APA‐Stroke)	43	13/27 Not stated 3	Mean 5.5 years SE 0.81 years	Not stated	23
	Sittercise (seated, nonprogressive aerobic upper body general exercise program)	33	13/18 Not stated 2	Mean 4.7 years SE 1.5 years	Not stated	25
Sun Juanjuan 2014	Motor function rehabilitation training and acupoint pressing	50	Not stated	Whole group: "first onset"	Whole group: Cerebral infarction	50
	Routine nursing	50	Not stated			50
SunRISe 2021	Home‐based physiotherapy intervention	20	13/7	Mean 3.1 years SD 3.5 years	Ischaemic 18 Haemorrhage 2	Received intervention 20 Provided follow‐up data 14
	Usual care	10	4/6	Mean 4.5 years SD 3.1 years	Ischaemic 9 Haemorrhage 1	Received intervention 10 Provided follow‐up data 10
Tang 2009	Observation	35	Not stated	Not stated	Not stated	35
	Control	35	Not stated	Not stated	Not stated	35
Tang Yao 2015	Three‐stage rehabilitation	50	Not stated	Not stated	Cerebral embolism: 23 Haemorrhage: 27	50
	No physical rehabilitation	50	Not stated	Not stated	Cerebral embolism: 20 Haemorrhage: 30	50
Teixeira‐Salmela 1999	Aerobic exercise and muscle strength training	6	3/3	Mean 9.15 years SD 12.72 years.	Not stated	6
	No treatment	7	3/4	Mean 6.40 years SD 6.23 years	Not stated	7
Thaut 2007	Rhythmic auditory stimulation	43	20/23	Mean 21.3 days SD 11 days	Location of stroke: MCA = 35 Internal capsule = 4 Basal ganglia/thalamus = 3 Subdural haematoma = 1	43
	Neurodevelopmental therapy (NDT)/Bobath− based training	35	16/19	Mean 22.2 days SD 12 days	Location of stroke: MCA = 30 Internal capsule = 4 Basal ganglia/thalamus = 1	35
Torres‐Arreola 2009	Strategy 1	59	Not stated	Mean 7.1 days SD 5.9 days	Not stated	At 6 months follow‐up = 32
	Strategy 2	51	Not stated	Mean 6.3 days SD 3.1 days	Not stated	At 6 months follow‐up = 35
Tyson 2015	Patient‐led upper‐limb mirror therapy	63	27/35 Not stated 1	Mean 26 days SD 18 days Not stated 1	Ischaemic 50 Not stated 13	Received intervention 62 Assessed at 8w 59
	Patient‐led lower leg exercises	31	11/20	Mean 35 days SD 27 days	Ischaemic 26 Not stated 5	Received intervention 31 Assessed at 8w 26
Vahlberg 2017	Progressive resistance and balance exercise programme	20	Not stated	Median 14 months IQR 4 months	Cerebral infarction 17 Intracerebral haemorrhage 4	20
	Usual care	23	Not stated	Median 14 months IQR 4 months	Cerebral infarction 20 Intracerebral haemorrhage 4	23
Verma 2011	Experimental	15	8/7	Mean 6.07 weeks SD 3.30 weeks	Haemorrhagic = 4 Ischaemic = 11	15
	Control	15	7/8	Mean 6.60 weeks SD 3.20 weeks	Haemorrhagic = 3 Ischaemic = 12	15
Wade 1992	Mixed	49	25/19 5 brainstem	Mean 53.1 months SD 29.5 months	Not stated	48
	Control (no treatment)	45	21/21 3 brainstem	Mean 59.6 months SD 35.3 months	Not stated	41
Wan Xueli 2014	Bobath therapy	24	Not stated	Not stated	Cerebral infarction 10 Cerebral haemorrhage 14	24
	Usual care (no physical therapy)	24	Not stated	Not stated	Cerebral infarction 11 Cerebral haemorrhage 13	24
Wang 2004a	Rehabilitation	70	Not stated	Not stated	Not stated	66
	Control (no treatment)	35	Not stated	Not stated	Not stated	32
Wang 2004b	Treatment	25	Not stated	Mean 54.2 days SD 37.5 days	Haemorrhagic = 11 Ischaemic = 14	25
	Control (no treatment)	25	Not stated	Mean 55.7 days SD 35.3 days	Haemorrhagic = 9 Ischaemic = 16	25
Wang 2005	Neurophysiological	21	11/10	Patients with spasticity Mean 21.9 days SD 7.4 days Patients with relative recovery Mean 21.6 days SD 9.3 days	Haemorrhagic = 7 Ischaemic = 14	21
	Orthopaedic	23	9/14	Patients with spasticity Mean 20.7 days SD 5.9 days Patients with relative recovery Mean 19.6 days SD 7.9 days	Haemorrhagic = 7 Ischaemic = 14	23
Wang 2013	Rehabilitation exercises (Bobath)	65	Not stated	Whole group: Not stated ‐ "acute"	Not stated	65
	Usual care (no physical therapy)	65	Not stated		Not stated	65
Wang 2015	Caregiver‐Mediated Intervention	25	13/13	Median 18.5 months Q1‐Q3 8.75‐31.75 months	Not stated	25
	No treatment	26	11/14	Median 18 months Q1‐Q3 11.5‐32 months	Not stated	26
Wang 2021	Rehabilitation nursing	121	Not stated	Mean 3.81 days SD 1.61 days	Whole group: Ischaemic stroke	108
	Usual Therapist‐led treatment	103	Not stated	Mean 4.07 days SD 1.81 days		101
Wang 2022	Nurse‐led rehabilitation	52	Whole group: Not stated	Mean 3.81 days SD 1.61 days	Whole group: Ischaemic stroke	43
	Usual care	52		Mean 4.07 days SD 1.81 days		45
Wang Dongya 2015	Three‐stage rehabilitation	63	31/32	Not stated	Cerebral infarction 47 Cerebral haemorrhage 16	63
	Usual care	63	24/39	Not stated	Cerebral infarction 41 Cerebral haemorrhage 22	63
Wang Leilei 2020	Task‐oriented training + acupuncture	100	Not stated	Mean (SD): 16 (0.6) months; range: 1‐3 months	Cerebral infarction 67 Cerebral haemorrhage 33	100
	Conventional rehabilitation + acupuncture	100	Not stated	Mean (SD): 1.7 (0.4) months; range: 3 weeks‐3 months	Cerebral infarction 69 Cerebral haemorrhage 31	100
Wang Wenwei 2012	Bobath training	40	21/19	Mean (SD): 4.03 (1.14) months	Cerebral infarction 20 Cerebral haemorrhage 15 Mixed 5	40
	Conventional rehabilitation	40	23/17	Mean (SD): 3.96 (1.07) months	Cerebral infarction 17 Cerebral haemorrhage 15 Mixed 8	40
Wei 2014	Three‐stage rehabilitation (all stages)	40	Not stated	Mean 9.78 days SD 5.36 days	Cerebral ischaemia 23 Cerebral haemorrhage 17	40
	Three‐stage rehabilitation (1st and 2nd stages only)	40	Not stated	Mean 10.54 days SD 4.76 days	Cerebral ischaemia 23 Cerebral haemorrhage 17	40
	Three‐stage rehabilitation (1st and 3rd stages only)	40	Not stated	Mean 10.09 days SD 5.02 days	Cerebral ischaemia 23 Cerebral haemorrhage 17	40
Werner 1996	Intensive outpatient rehabilitation programme	33	Not stated	Mean 2.9 years SD 1.8 years Not stated 5	Not stated	28
	No treatment	16	Not stated	Mean 3.3 years SD 1.9 years Not stated 4	Not stated	7
Wu 2006	Rehabilitation	50	21/27 location of disease	Mean 7.38 days SD 5.83 days	Haemorrhagic = 14 Ischaemic = 34	48
	Control (no treatment)	50	27/25 location of disease	Mean 6.33 days SD 5.00 days	Haemorrhagic = 13 Ischaemic = 35	48
Wu 2020	Collaborative Care Model Based Telerehabilitation Exercise Training Programme	32	18/12 Not stated 2	Not stated	Ischaemia 20 Haemorrhage 10	30
	Usual care	32	20/11 Not stated 1	Not stated	Ischaemia 24 Haemorrhage 7	31
Wu Jiaming 2006	Rehabilitation	40	Not stated	Whole group: 43 (8.1) days	Whole group: Ischaemic: 71 Haemorrhagic: 9	40
	Conventional rehabilitation	40	Not stated			40
Wu Jing 2015	Three‐stage rehabilitation	46	22/24	Mean 23.6 hours SD 5.2 hours Range 3‐45 hours	Cerebral infarction 32 Cerebral haemorrhage 14 Basal ganglia 29 Other areas 17	46
	Conventional treatment patients self exericse)	46	23/23	Mean 24.0 hours SD 5.5 hours Range 1‐47 hours	Cerebral infarction 34 Cerebral haemorrhage 12 Basal ganglia 31 Other areas 15	46
Wu Lotus 2016	Walking training	40	Not stated	Not stated	Not stated	40
	Conventional rehabilitation	40	Not stated	Not stated	Not stated	40
Xiao 2003	Intensive rehabilitation	67	Not stated	Mean 14.7 days SD 1.3 days	Haemorrhagic = 20 Ischaemic = 47	67
	Conventional (no treatment)	67	Not stated	Mean 12.9 days SD 0.9 days	Haemorrhagic = 23 Ischaemic = 44	67
Xiao Yuhua 2015	Three‐stage rehabilitation	42	Not stated	Not stated	Not stated	Not stated
	Routine treatment	41	Not stated	Not stated	Not stated	Not stated
Xiao Zhen‐dong 2014	Balance training	30	Not stated	Not stated	Cerebral infarction 14 Cerebral haemorrhage 16	30
	Routine rehabilitation therapy	30	Not stated	Not stated	Cerebral infarction 15 Cerebral haemorrhage 15	30
Xie 2003	Rehabilitation	32	Not stated	Whole group Mean 17 hours SD 7 hours Range : 6‐52 hours	Whole group Cerebral infarct = 52 Cerebral haemorrhage = 12	32
	Control (no treatment)	32	Not stated	As above	As above	32
Xie 2005	Rehabilitation	35	Not stated	Not stated	Haemorrhagic = 10 Ischaemic = 25	35
	Control (no treatment)	35	Not stated	Not stated	Haemorrhagic = 10 Ischaemic = 25	35
Xu 1999	Rehabilitation	32	Not stated	Not stated	Haemorrhagic = 14 Ischaemic = 18	32
	Control (no treatment)	30	Not stated	Not stated	Haemorrhagic = 16 Ischaemic = 14	30
Xu 2003a	Rehabilitation	94		'Mean time from onset of disease to hospitalisation was 3.5 days'	Not stated	94
	Control (no treatment)	92	Not stated	'Mean time from onset of disease to hospitalisation was 4 days'	Not stated	92
Xu 2003b	Rehabilitation	92	42/50	Mean 2.3 days SD not stated	Infarct in 66 cases in basal ganglion, 16 cases in lobar and 10 cases in corona radiate and oval centre	92
	Control (no treatment)	88	40/48	Mean 2.5 days SD not stated	Infarct in 64 cases in basal ganglion, 15 cases in lobar and 9 cases in corona radiate and oval centre	88
Xu 2004	Rehabilitation	30	9/21	Mean 14.8 days SD 3.7 days	Haemorrhagic = 2 Ischaemic = 28	30
	Control (no treatment)	27	9/18	Mean 15.1 days SD 4.3 days	Haemorrhagic = 1 Ischaemic = 26	27
Xu 2013	Medical exercise	58	Not stated	Not stated	Whole group: Cerebral infarction 78 Cerebral haemorrhage 39 (non‐acute phase)	58
	Conventional medicine therapy	59	Not stated	Not stated		56
Xu 2015	EMG‐triggered stimulation + comprehensive rehabilitation	20	9/11	Mean 46.3 days SD 14.9 days	Ischaemic 11 Haemorrhagic 9	Not stated
	Conventional therapy	20	8/12	Mean 47.8 days SD 16.1 days	Ischaemic 13 Haemorrhagic 7	Not stated
Xu 2022	Physical rehabilitation	80	33/47	Whole group: Not stated	54 I 26 H	82
	No physical rehabilitation	80	34/48		55 I 25 H	82
Xu Wenyu 2012	"inertial guided gait rehabilitation training"	50	Not stated	Not stated	Cerebral infarction 41 Cerebral haemorrhage 9	50
	"conventional rehab gait training"	50	Not stated	Not stated	Cerebral infarction 36 Cerebral haemorrhage 14	50
Xu Yumei 2013	Rehabilitation training	20	Not stated	Mean 42.57 days SD 23.51 days	Cerebral infarction 8 Cerebral haemorrhage 12	20
	Conventional facilitation techniques (Bobath) and TCM	20	Not stated	Mean 44.32 days SD 26.57 days	Cerebral infarction 9 Cerebral haemorrhage 11	20
Xue 2006	Training	78	Not stated	Not stated	Haemorrhagic = 37 Infarct = 41	78
	Control (no treatment)	72	Not stated	Not stated	Haemorrhagic = 34 Infarct = 38	72
Yadav 2016	Specific Balance Strategy Training Programme	12	7/5	Mean 15.17 months SD 6.96 months	Not stated	12
	General balance training	12	4/8	Mean 23.42 months SD 21.31 months	Not stated	12
Yan 2002	Rehabilitation	40	16/24	Mean 14.8 days SD 3.7 days	Haemorrhagic = 14 Ischaemic = 26	40
	Control (no treatment)	38	16/22	Mean 15.1 days SD 4.3 days	Haemorrhagic = 11 Ischaemic = 27	38
Yan 2015	Three‐stage rehabilitation	30	8/22	Mean 9.8 months SD 1.2 months	Not stated	30
	Convnetional rehabilitation training	30	10/20	Mean 9.1 months SD 2.1 months	Not stated	30
Yang 2006	Task‐oriented progressive resistance strength training	24	8/16	Mean 62.7 months SD 27.4 months Range 42 ‐ 120 months	Not stated	24
	No rehabilitation training	24	12/12	Mean 64.0 months SD 40.4 months Range 36 ‐ 120 months	Not stated	24
Yang 2017	Bobath + electroacupuncture	29	Not stated	Mean 5.27 months SD 3.16 months Range 2‐10 months	Ischaemic: 16 Haemorrhagic: 13	29
	Electroacupuncture	29	Not stated	Mean 5.63 months SD 3.23 months Range 2‐13 months	Ischaemic: 17 Haemorrhagic: 12	29
Yang Aiguo 2015	Bobath therapy combined with acupuncture	48	Not stated	Mean 9.06 days SD 5.35 days	Not stated	48
	Acupuncture	48	Not stated	Mean 7.37 days SD 5.10 days	Not stated	48
	No physical therapy	48	Not stated	Mean 8.26 days SD 3.77 days	Not stated	48
Yang Jian 2007	Rehabilitation	31	Not stated	Mean 37.29 days SD 17.07 days	Ischaemic 17 Haemorrhagic 14	31
	Attention control	29	Not stated	Mean 36.72 days SD 12.12 days	Ischaemic 40 Haemorrhagic 7	29
Yang Zhihong 2015	Three‐stage rehabilitation	37	Not stated	Not stated	Whole group: Cerebral infarction 32 Cerebral haemorrhage 28 Heart disease caused by hypertension 14	37
	Traditional nursing	37	Not stated	Not stated		37
Yazici 2021	Neurodevelopmental‐Bobath approach	23	Not stated	Not stated	Whole group: Ischaemic stroke	21
	Standard rehabilitation	18	Not stated	Not stated		18
Ye Dayong 2010	Rehabilitation	30	Not stated	Mean: 7.2 (2.65) days	Ischaemic: 11 Haemorrhagic: 19	30
	No physical rehabilitation	30	Not stated	Mean: 7.2 (3.04) days	Ischaemic: 10 Haemorrhagic: 20	30
Yelnik 2008	NDT‐based treatment	35	17/16	Mean 218.4 days SD 93.4 days	Ischaemic = 24 Not stated = 11	35
	Multisensorial	33	20/15	Mean 217.2 days SD 92.9 days	Ischaemic = 25 Not stated = 8	33
Yin 2003a	Rehabilitation	30	Not stated	Not stated	Not stated	30
	Rehabilitation with therapy with intermediate frequency	30	Not stated	Not stated	Not stated	30
	Control (no treatment)	30	Not stated	Not stated	Not stated	30
Yue Chunjiang 2014	Exercise re‐learning rehabilitation + acupuncture	45	20/25	Mean 41.28 days SD 14.26 days	Whole group: Ischaemic stroke	45
	Routine rehabilitation training	45	22/23	Mean 42.25 days SD 13.64 days		45
Yue Lin 2012	Balance training	46	Whole group: Not stated	Whole group: Not stated	18 I 28 H	46
	Conventional rehabilitation	46			17 I 29 H	46
Zang 2013	Three‐stage rehabilitation TCM programme	50	Not stated	Whole group: Not stated but rehabilitation given from onset	Whole group: Ischaemic stroke	49
	2‐week western medicine rehabilitation (1st stage)	50	Not stated			49
Zhang 1998	Early rehabilitation	29	Not stated	Not stated	Not stated	29
	Control (no treatment)	27	Not stated	Not stated	Not stated	27
Zhang 2004	Rehabilitation	439	Not stated	Not stated	Haemorrhage = 61 Ischaemic = 278	439
	Control (no treatment)	463	Not stated	Not stated	Haemorrhage = 172 Ischaemic = 291	463
Zhang Huiyu 2021	Goal‐oriented functional exercise	52	Not stated	Whole group: Not stated	Whole group: Cerebral haemorrhage Basal ganglia 18 Cerebral lobe 14 Thalamus 13 Cerebellum 7	52
	Routine therapy	52	Not stated		Basal ganglia 17 Cerebral lobe 12 Thalamus 15 Cerebellum 8	52
Zhang Jianhong 2013	Neurorehabilitation	84	Not stated	Whole group: Not stated	Putamen‐external capsule infarction 46 Thalamus‐internal capsule infarction 28 Lobar infarction 10	84
	Self‐training without regulated rehabilitation	82	Not stated		Putamen‐external capsule infarction 50 Thalamus‐internal capsule infarction 22 Lobar infarction 10	82
Zhang Lifang 2015	Balance function training	26	Not stated	Whole group: Average 4.35 months Variation 1.17 months Range 1‐6 months	Whole group: Cerebral infarction 32 Cerebral haemorrhage 20	26
	Routine training	26	Not stated			26
Zhao 2002	Rehabilitation nursing	100	39/61	Not stated	Not stated	100
	Control (no treatment)	80	34/46	Not stated	Not stated	80
Zhao 2003	Rehabilitation	150	82/68	Not stated	"cerebral infarction"	150
	Control (no treatment)	150	79/71	Not stated	"cerebral infarction"	150
Zhao Ailiang 2016	Rehabilitation + acupuncture	65	30 cases right limb hemiplegia; 28 cases left limb hemiplegia and 7 cases bilateral hemiplegia	Mean (SD): 2.63 (0.56) months; range: 5 days‐ 12 months	Not stated	65
	Acupuncture	61	29 cases right limb hemiplegia; 26 cases left limb hemiplegia and 6 cases bilateral hemiplegia	Mean (SD): 2.59 (0.41) months; range: 7 days‐ 12 months	Not stated	61
Zhao Haihong 2013	Bobath technique plus conventional treatment	30	Not stated	Whole group: Not reported	Cerebral infarction 19 Cerebral haemorrhage 11	Not stated
	Conventional neurological treatment	30	Not stated		Cerebral infarction 18 Cerebral haemorrhage 12	Not stated
Zheng 2014	Rehabilitation therapy (Bobath and motor relearning) + routine medical treatment	30	Not stated	Whole group: 7‐30 days	Not stated	30
	Routine medical treatment (self‐rehabilitation training)	30	Not stated		Not stated	30
Zhong Qiue 2014	Bilateral limb function training	32	15/17	Mean 70.0 days SD 15.0 days Range 40 days ‐ 12 months	Ischaemic disease 14 Haemorrhagic disease 18	32
	Unilateral (affected) limb function training	32	17/15	Mean 70.0 days SD 15.0 days Range 40 days ‐ 12 months	Ischaemic disease 18 Haemorrhagic disease 14	32
Zhu 2001	Rehabilitation	72	Not stated	Mean 9.51 days SD 5.36 days	Bleeding after decompression surgery = 1 Haemorrhagic = 20 Ischaemic = 51	72
	Control (no treatment)	53	Not stated	Mean 9.91 days SD 7.90 days	Bleeding after decompression surgery = 1 Haemorrhagic = 12 Ischaemic = 40	53
Zhu 2004b	Treated	26	Not stated	Mean 8 days SD 5 days	Haemorrhagic = 10 Ischaemic = 16	26
	Controlled (no treatment)	26	Not stated	Mean 8 days SD 4 days	Haemorrhagic = 10 Ischaemic = 16	26
Zhu 2006	Test	35	Not stated	Mean 30.4 days SD 6.8 days	Haemorrhagic = 8 Ischaemic = 27	35
	Controlled (no treatment)	35	Not stated	Mean 31.6 days SD 6.2 days	Haemorrhagic = 7 Ischaemic = 28	35
Zhu 2007	Cerebral haemorrhage rehabilitation	12	4/8	Mean 16 days SD 5 days	Haemorrhagic = 12	12
	Cerebral haemorrhage control	10	3/7	Mean 17 days SD 7 days	Haemorrhagic = 10	10
Zhu 2016	Modified constraint‐induced movement therapy	11	7/4	Mean 3.90 months SD 0.83 months	Whole group: Not stated. Both cerebral infarction and haemorrhagic cerebrovascular accident were included	Not stated
	Conventional therapy	11	6.5	Mean 3.72 months SD 0.78 months		Not stated
Zhuang 2012	Physiotherapy	86	47/39	Mean 34.24 days SD 21.53 days Range 15‐86 days	Ischaemic encephalic region: Basal ganglia = 62 Other = 24	86
	Acupuncture	91	50/41	Mean 30.89 days SD 21.67 days Range 15‐80 days	Ischaemic encephalic region: Basal ganglia = 70 Other = 21	91
	Combination therapy	97	51/48	Mean 29.73 days SD 18.57 days Range 16‐88 days	Ischaemic encephalic region: Basal ganglia = 72 Other = 25	97

H: haemorrhagic; I: ischaemic; IQR: interquartile range; LCVA: left cerebrovascular accident; LAC: lacunar; LACI: lacunar infarct; LACS: lacunar stroke; MCA: middle cerebral artery; mo: months; NR: not reported; PAC: partial anterior circulation; PACI: partial anterior circulation infarct; PACS: partial anterior circulation stroke; POCS: posterior circulation; POCS: posterior circulation stroke; PICH: primary intracerebral haemorrhage; RCVA: right cerebrovascular accident; SD: standard deviation; SEM: standard error of the mean; TAC: total anterior circulation; TACI: total anterior circulation infarct; TACS: total anterior circulation stroke; y: years

**Table:** Ain 2022

***Study characteristics***	
Methods	**Study aim:** "To compare the effects of functional training program and conventional therapy on posturalcontrol and functional mobility in chronic stroke" **Design:** parallel‐group RCT **Country:** Pakistan **Study recruitment and setting:** see Table **Involvement of patients/public/professionals/others:** no
Participants	**Study population (number randomised):** 12 **Inclusion criteria:** Clinically diagnosed stroke Aged 40 to 65 years, both sexes Not able to walk independently **Exclusion criteria:** Unstable cardiovascular status Significant lower limb or musculoskeletal condition or other neurological condition which could impact on posture Communication barriers and/or cognitive impairment Unable to maintain a sitting or standing position for 30s **Participants:** detailed in Table **PROGRESS‐plus:** detailed in Table
Interventions	(1) Functional training "received functional training program including baseline treatment of TENS, hot pack with assisted equipment theraband, Swiss ball, parallel bars, balance boards, chairs and stairs of different heights, free weight training including modifiable weights, and task oriented activities with different resistance activities." (2) Conventional therapy "received TENS, hot pack, and active assisted exercise, then resisted exercises with increased repetitions with time."
Outcomes	**Independence in ADL scales:** NR **Motor function scales:** NR **Measures of balance:** Berg Balance Scale **Measures of gait velocity:** timed up and go test; 6‐minute walk test **Length of stay:** NR **Adverse events:** NR **Other measures:** trunk control measurement scale **Time points when outcomes were assessed:** "post‐treatment" (time of this unclear)
Comparison	**Amount of physical rehabilitation in groups:** equivalent (unclear) **Categorisation of comparison:** 3.(i) Comparison of different approaches (dose equivalent; with no other physical rehab)
Funding & conflicts of interest	**Funding statement:** not reported **Conflict of interest statement:** "no conflict of interest to declare by any author"
Synthesis	**Qualitative synthesis:** data presented as difference from baseline only
Notes	

**Table:** Aksu 2001

***Study characteristics***	
Methods	**Study aim:** to determine the effects of numbers of exercises on basic mobility activities and extremity function in early rehabilitation of acute stroke patients **Design:** multi‐arm RCT (3‐arms) **Country:** Turkey **Study recruitment and setting:** see Table **Involvement of patients/public/professionals/others:** no
Participants	**Study population (number randomised):** 20 **Inclusion criteria:** Acute stroke with similar functional levels **Exclusion criteria:** Not reported **Participants:** detailed in Table **PROGRESS‐plus:** detailed in Table
Interventions	(1) Bobath Group 1 (n = 9) "Four exercises ... were chosen from Bobath’s neurodevelopmental approach" Length of intervention period: not stated Number of sessions and length of individual sessions: not stated Intervention provider: not stated (2) Bobath Group 2 (n = 7) "Six exercises ... were chosen from Bobath’s neurodevelopmental approach" Length of intervention period: not stated Number of sessions and length of individual sessions: not stated Intervention provider: not stated (3) Bobath Group 3 (n = 4) "Eight exercises ... were chosen from Bobath’s neurodevelopmental approach" Length of intervention period: not stated Number of sessions and length of individual sessions: not stated Intervention provider: not stated
Outcomes	**Independence in ADL scales:** NR **Motor function scales:** NR **Measures of balance:** NR **Measures of gait velocity:** NR **Length of stay:** NR **Adverse events:** NR **Other measures:** Stroke Rehabilitation Assessment of Movement (STREAM) **Time points when outcomes were assessed:** "after the stroke in the first week, motor assessment was performed"
Comparison	**Amount of physical rehabilitation in groups:** equivalent **Categorisation of comparison:** 4. Comparison of similar approaches (dose equivalent)
Funding & conflicts of interest	**Funding statement:** not reported **Conflict of interest statement:** not reported
Synthesis	**Qualitative****synthesis**: no data available for analysis
Notes	Abstract only

**Table:** Alabdulwahab 2015

***Study characteristics***	
Methods	**Study aim:** "evaluate whether intervention programs that combine lower limb overload with functional task training are more effective at improving walking outcomes and community participation than lower limb overload training alone" **Design:** parallel‐group RCT **Country:** India **Study recruitment and setting:** see Table **Involvement of patients/public/professionals/others:** no
Participants	**Study population (number randomised):** 26 **Inclusion criteria:** First stroke at least 6 months before recruitment with unilateral motor deficits affecting gait pattern and/or speed Independent walking ability with or without walking aid for at least 10 m Ability to perform closed chain exercise **Exclusion criteria:** Significant psychiatric or cognitive deficits Major cardiorespiratory diseases, chronic pain, or lower limb joint complications **Participants:** detailed in Table **PROGRESS‐plus:** detailed in Table
Interventions	(1) FLO (Functional Limb Overloading) group (n = 13) "Task‐oriented gait training of one‐hour duration three times per week for four weeks. They were requested to wear the weight cuff, equivalent to 5% of the total body weight, for most of their awake time (approximately 90% of the time)" "During the training session, after 5–10 minutes of simple warm‐up exercises, the patients performed various functional activities including forward, sideways, and backward walking, turning to 90 and 180 degrees during walking, stairclimbing, and so forth. During each session the patients were also made to walk on treadmill for 15 minutes at speed of 10% above comfortable walking. The progression of the training was done by increasing the loading or steps height,or by reducing the speed, or decreasing the patient’s support and/or doing more individually tailored sets, but as a rule the difficulty level was adjusted after six sessions" (2) LORT (Limb Overloading Resistance Training) group (n = 10) "Resistance‐training regime consisting of isotonic exercise using weight cuff tied to ankle or foot.The exercise algorithm was designed by the therapist accounting for strength as well as movement synergy level, to determine repetition maximum (RM) with the given load. The training protocol required each participant to isotonically exercise the affected lower extremity (LE) using external resistance of weight cuff, equivalent to 5% of the total body weight. The training session consisted of 5–10 minutes of simple warm‐up exercises and then three sets of 10–15 repetitions maximum, to moderate fatigue with limb overloading. The therapist follows an exercise algorithm that accounted for the participant’s strength as well as movement synergy level to determine RM for specific muscle groups, including hip flexors, hip extensors, knee flexors, knee extensors, ankle dorsi flexors, and ankle plantar flexors, with the given weight."
Outcomes	**Independence in ADL scales:** NR **Motor function scales:** NR **Measures of balance:** NR **Measures of gait velocity:** fast gait speed, slow gait speed **Length of stay:** NR **Adverse events:** NR **Other measures:** Stroke Impact Scale (mobility and participation domains only), cadence, weight‐bearing on affected limb **Time points when outcomes were assessed:** immediately post‐intervention
Comparison	**Amount of physical rehabilitation in groups:** equivalent **Categorisation of comparison:** comparison of different approaches (dose equivalent; with no other physical rehab)
Funding & conflicts of interest	**Funding statement:** Funded by Sheikh Abdullah Bin Abdul Mohsen Al Tuwaijri Chair for Applied Research in Stroke, Majmaah University, Saudi Arabia **Conflict of interest statement:** none
Synthesis	**Quantitative synthesis:**Analysis 7.4 Fast gait speed used for gait velocity analysis.
Notes	

**Table:** Allison 2007

***Study characteristics***	
Methods	**Study aim:** to investigate whether provision of additional standing practice increases motor recovery and mobility post stroke **Design:** parallel‐group RCT **Country:** UK **Study recruitment and setting:** see Table **Involvement of patients/public/professionals/others:** no
Participants	**Study population (number randomised):** 17 **Inclusion criteria:** Confirmed diagnosis of recent stroke **Exclusion criteria:** Terminally ill Suffering from an unstable co‐morbidity Unable to participate safely (physically or mentally) in additional sessions of standing **Participants:** detailed in Table **PROGRESS‐plus:** summarised in Table
Interventions	(1) Standing practice (n = 7) "The second treatment group (intervention group) received the conventional treatment session, and in addition had a further session of 45 minutes standing practice on each working day. This was provided by a physiotherapy assistant and typically involved the use of either standing frames, tilt tables or standing at tables to provide support while enabling standing to occur" "Participants progressed to standing by a table for support or free standing during rehabilitation as able. Participants were encouraged to be active whilst standing—practising reaching tasks, sit‐to‐stand movements and so on, and were given rest periods as necessary throughout the 45‐minute session. It was not possible for the physiotherapists providing the conventional treatment to be blind to the extra intervention occurring, due to the organization of therapy on the ward. This treatment regime was continued throughout the participant’s stay in the rehabilitation unit. After discharge from the unit the participant was referred for outpatient or community‐based physiotherapy. Intensity of follow‐up offered was based on community assessment and was typically one or two sessions of treatment per week" Length of intervention period: ranged from 14 to 28 days dependent upon length of stay on the unit Number of sessions and length of individual sessions: "received the conventional treatment session, and in addition had a further session of 45 minutes standing practice on each working day" Intervention provider: "conventional physiotherapy sessions were provided [by] one of three physiotherapists on the ward; the additional session of standing practice was provided by a physiotherapy assistant" (2) Conventional rehabilitation (n = 10) "Control group received conventional physiotherapy treatment from one of the three physiotherapists working on the ward. This was typically a session of 45 minutes treatment on each working day, including work on strengthening, improving movement, mobility, and upper limb function" Length of intervention period: ranged from 14 to 28 days dependent upon length of stay on the unit Number of sessions and length of individual sessions: "session of 45 minutes treatment on each working day" Intervention provider: "conventional physiotherapy sessions were provided [by] one of 3 physiotherapists on the ward"
Outcomes	**Independence in ADL scales:** NR **Motor function scales:** gross functional tool section of Rivermead Motor Assessment **Measures of balance:** Berg Balance Scale **Measures of gait velocity:** NR **Length of stay:** NR **Adverse events:** NR **Other measures:** trunk control test **Time points when outcomes were assessed:** week 1, week 2 and week 12
Comparison	**Amount of physical rehabilitation in groups:** differs **Categorisation of comparison:** additional therapy + usual therapy versus usual therapy (dose differs)
Funding & conflicts of interest	**Funding statement:** not reported **Conflict of interest statement:** not reported
Synthesis	**Qualitative synthesis:** no outcomes suitable for analysis (data reported as median and IQR)
Notes	

**Table:** Aloraini 2022

***Study characteristics***	
Methods	**Study aim:** "to investigate the effectiveness of CIMT for lower extremity for improving balance and ambulation" **Design:** parallel‐group RCT **Country:** Saudi Arabia **Study recruitment and setting:** see Table **Involvement of patients/public/professionals/others:** no
Participants	**Study population (number randomised):** 38 **Inclusion criteria:** First stroke (ischaemic or haemorrhagic) > 1 year study enrolment resulting in LE hemiparesis Age 18 years+ MMSE ≥ 24 Able to walk 10 m independently (with/without walking aid) BBS ≥ 35 Able to participate in study and adhere to therapy sessions **Exclusion criteria:** Excessive pain in the more affected LE (i.e. > 4/10 on VAS) Increased hypertonia in the LE (i.e. MAS ≥ 3) Currently enrolled in a physical rehabilitation programme Terminal illness Life‐threatening comorbidity Concomitant neurological or psychiatric illness **Participants:** detailed in Table **PROGRESS‐plus:** summarised in Table
Interventions	(1) Constraint‐induced movement therapy (lower extremity) "For the CIMT‐LE group, three hours of the session were allocated for physical exercises and the reminder of the session time (30 minutes) was dedicated for the transfer package (TP). The physical exercises provided to participants were functionally‐oriented and supervised. These exercises were targeted towards the more effected lower extremity and administered using a shaping technique. These shaping tasks were selected by the therapist based on the targeted movements (e.g., knee extension). Subsequently, the difficulty of exercises are determined based on the participant’s level and progressed periodically. Frequent feedback was provided to participants during task practice. Each task was performed for 10 trials, with each trial lasting 30–45 seconds. For example, one of the tasks given to the CIMT‐LE group was step‐up exercises. In this exercise the participant is asked to step up on a stool with the more affected lower extremity and then return to the starting position with both feet on level floor. Progressing the task would include increasing the height of the stool, increasing number of repetitions and/or increasing the distance between the stool and the participant. The TP includes a number of techniques, including a behavioural contract. This contract was signed by all participants in the CIMT‐LE group on the first day of the program. It included an agreement to commit to the therapeutic program and provided a list of activities that are typically performed daily and emphasized the use of the more affected lower extremity. Further, the behavioural contract included a number of home‐exercises that should be performed daily while at home (outside treatment session and during weekends) and following the conclusion of the therapeutic program." (2) Conventional rehabilitation "For the control group, participants received a conventional post‐stroke rehabilitation program. The program included range of motion and stretching exercises, balance, walking and endurance training. Further, participants in the control group were provided with transfer training, rehabilitation education, and encouraged to practice some of the exercises at home and following the conclusion of the program. " "Regardless of group assignment, therapeutic sessions for all participants were 3.5 hours, five days per week for a duration of 2 weeks."
Outcomes	**Independence in ADL scales:** NR **Motor function scales:** Fugl‐Meyer lower extremity **Measures of balance:** Berg Balance Scale **Measures of gait velocity:** 10‐m walk test; 6‐minute walk test **Length of stay:** NR **Adverse events:** NR **Other measures:** NR **Time points when outcomes were assessed:** immediately after the end of the programme and at 3 months follow up
Comparison	**Amount of physical rehabilitation in groups:** equivalent **Categorisation of comparison:** 3.(i) Comparison of different approaches (dose equivalent; with no other physical rehab)
Funding & conflicts of interest	**Funding statement:** "Qassim University represented by the Deanship of Scientific Research, for the financial support for this research (number 20007‐fcohsb‐2020‐1‐1‐W) during the academic years 1442AH/2021AD" **Conflict of interest statement:** "author reports no conflict of interest"
Synthesis	**Quantitative synthesis:**Analysis 7.2, Analysis 7.3, Analysis 7.4, Analysis 8.2, Analysis 8.3, Analysis 8.4
Notes	

**Table:** Anandan 2020

***Study characteristics***	
Methods	**Study aim:** to evaluate the "effect of task specific training and PNF on various functions in stroke survivors" **Design:** parallel‐group RCT **Country:** India **Study recruitment and setting:** see Table **Involvement of patients/public/professionals/others:** no
Participants	**Study population (number randomised):** 74 **Inclusion criteria:** Aged 45 to 69 years MCA stroke with hemiplegia on dominant side at least 6 months Brunnstrom recovery stage of 4 Grade 2 on MAS No perceptual or cognitive deficits, No other associated problems (e.g. limb malformations) Medically stable **Exclusion criteria:** NR **Participants:** detailed in Table **PROGRESS‐plus:** summarised in Table
Interventions	(1) Task specific training (TST) (n = 37) "The basic premise is goal directed practice of functional tasks instead of focusing on impairment reduction exercises. Task specific training involves assessment and training given with multiple tasks related to activities of daily living. Task specific training is effective which depends on the amount of practice necessarily given to improve motor performances, it enhances the daily activities whereby improves the performances in stroke survivors" (2) PNF therapy (n = 37) "PNF therapy for a duration of 10 weeks" "Treatment duration for each group consists of 60 minutes of exercises."
Outcomes	**Independence in ADL scales:** NR **Motor function scales:** NR **Measures of balance:** Berg Balance Scale **Measures of gait velocity:** NR **Length of stay:** NR **Adverse events:** NR **Other measures:** Modified Ashworth Scale, Action Reach Arm Scale, dynamic gait index **Time points when outcomes were assessed:** before and after the intervention
Comparison	**Amount of physical rehabilitation in groups:** equivalent **Categorisation of comparison:** 3.(i) Comparison of different approaches (dose equivalent; with no other physical rehab)
Funding & conflicts of interest	**Funding statement:** not reported **Conflict of interest statement:** none
Synthesis	**Quantitative synthesis:**Analysis 7.3
Notes	

**Table:** Arabzadeh 2018

***Study characteristics***	
Methods	**Study aim:** To evaluate "the effects of a task‐oriented exercise program on balance in patients with hemiplegia following stroke" **Design:** parallel‐group RCT **Country:** Iran, Islamic Rep. **Study recruitment and setting:** see Table **Involvement of patients/public/professionals/others:** no
Participants	**Study population (number randomised):** 20 **Inclusion criteria:** Hemiplegia following stroke (in the past 3 months) Clear asymmetry in weight‐bearing Able to walk 10 m independently without an assistive device BBS range of 30 to 40 **Exclusion criteria:** Impaired vision Other neurological or orthopaedic diseases affecting postural function (e. g. spoliomyelitis) Other conditions that would prevent the patient from participation **Participants:** detailed in Table **PROGRESS‐plus:** summarised in Table (this will be the PROGRESS‐plus that we said we would extract)
Interventions	(1) Task oriented exercise programme (n = 10) "The patients performed the following task‐oriented exercise program: 1) sitting on a chair and reaching for objects in all directions at a distance of more than arm’s length, 2) stepping forward, backward, and sideways on the exercise step, 3) performing flexion and extension of the affected knee, with the affected foot located on the exercise step and the unaffected foot off the step, 4) stepping over obstacles with different heights, 5) standing up from a chair, walking four steps forward, touching a stool, and then returning to the chair, 6) sitting on a Swiss ball while doing a range of motion and balance exercises in the trunk and upper extremities, 7) double leg standing for 10 seconds, 8) tandem standing, or placing the heel of one foot in front of the other foot, for 10 seconds, 9)standing up from a chair without using the arms, and 10) tandem walking forward and backward" (2) Traditional physiotherapy (n = 10) "For the control group, the patients received 50 minutes of traditional physiotherapy, including mat exercises, a range of motion exercises, and walking education" "Both groups were matched in the number of exercises and time allocated to each session"
Outcomes	**Independence in ADL scales:** NR **Motor function scales:** NR **Measures of balance:** Berg Balance Scale (Persian version) **Measures of gait velocity:** NR **Length of stay:** NR **Adverse events:** NR **Other measures:** postural sway parameters **Time points when outcomes were assessed:** immediately post‐intervention
Comparison	**Amount of physical rehabilitation in groups:** equivalent **Categorisation of comparison:** comparison of different approaches (dose equivalent; with no other physical rehab)
Funding & conflicts of interest	**Funding statement:** supported and funded by the Tabriz University of Medical Sciences. **Conflict of interest statement:** not reported
Synthesis	**Quantitative synthesis:**Analysis 7.3
Notes	

**Table:** Aravind 2022

***Study characteristics***	
Methods	**Study aim:** to evaluate the feasibility and acceptability of the TIME programme compared to usual care in improving everyday function in ambulatory people discharged home post‐stroke **Design:** parallel‐group RCT (mixed methods) **Country:** Canada **Study recruitment and setting:** see Table **Involvement of patients/public/professionals/others:** yes
Participants	**Study population (number randomised):** 33 **Inclusion criteria:** Ambulatory adults living in the community post‐stroke, with a clinical diagnosis of stroke documented in the health record Age ≥ 18 years Living at home for at least 3 months post‐hospitalisation Self‐reported ability to walk ≥ 10 m with or without walking aids without assistance from another person Ability to follow verbal instructions and speak and read English Willingness to sign a liability waiver verifying medical clearance from a healthcare provider **Exclusion criteria:** Self‐reported involvement in another exercise/rehabilitation programme Self‐reported condition/symptoms which may prevent participation in the intervention (e.g. unstable cardiovascular disease or significant joint pain) Cognitive or behavioural conditions which may prevent co‐operation in a group Unable to walk ≥ 20 min without a seated rest Self‐reported ability to manage environmental barriers (kerbs, ramps, and stairs) with relative ease **Participants:** detailed in Table **PROGRESS‐plus:** summarised in Table
Interventions	(1) Together in Movement Exercise (TIME) programme "Together in Movement and Exercise (TIMETM) is a 12‐week, community‐based exercise program" "The program involves two 1‐h exercise classes per week for 12 weeks. Each 1‐h class involves a seated warm‐up, repetitive and progressive practice of functionally relevant balance and mobility tasks, and a seated cool down. Warm‐up consists of active range‐of‐motion exercises, aerobic exercise, lower extremity weight‐bearing, stretching, and sit‐to‐stand training. The cool down involves exercises similar to the warm‐up but with an emphasis on stretching and relaxation. Participants, grouped by ability level complete exercises organized in a 3‐station circuit as follows: station 1: walking, aerobic training, and wall work (standing and reaching, wall push‐ups); station 2: standing weight shifts, stepping, and lunging; and station 3: tap‐ups, step‐ups, and heel/toe raises, hamstring curls, marching‐on‐the‐spot, and mini‐squats. Each exercise has several levels of challenge to enable tailoring by instructors. Instructors are advised to have participants exercise at an intensity of 3–4 (moderate to somewhat hard) on the modifed Borg scale." (2) Usual care
Outcomes	**Independence in ADL scales:** Barthel Index, Subjective Index of Physical and Social Outcome (SIPSO) (10‐item, self‐reported, higher score indicates better outcome) **Motor function scales:** NR **Measures of balance:** Berg Balance Scale **Measures of gait velocity:** 10‐m walk test, 6‐minute walk test **Length of stay:** NR **Adverse events:** falls/injurious falls **Other measures:** Nottingham Extended Activities of Daily Living, Stroke Impact Scale, EQ‐5D‐5L, caregiver assistance scale, activity specific balance confidence, 30 second timed sit‐to‐stand, trail making test, geriatric depression scale **Time points when outcomes were assessed:** 0, 3, 6, and 12 months after recruitment
Comparison	**Amount of physical rehabilitation in groups:** differs **Categorisation of comparison:** 1. Physical rehabilitation versus no or limited physical rehabilitation (dose differs) (limited = < 50% of dose)
Funding & conflicts of interest	**Funding statement:** "This project was funded by the Heart and Stroke Foundation (G‐16‐00013979). They had no role in the design, analysis, or writing the manuscript. NMS held a Heart and Stroke Foundation Mid‐Career Investigator Award and the Toronto Rehabilitation Institute Chair at the University of Toronto to complete this work." **Conflict of interest statement:** "JH is one of the lead developers of the TIMETM program but was not involved in data collection or data analysis".
Synthesis	**Quantitative synthesis:**Analysis 1.1, Analysis 1.3, Analysis 1.4, Analysis 1.6, Analysis 2.1, Analysis 2.3, Analysis 2.4
Notes	Authors state: "TIME^TM^ was intended as a wellness program, not as rehabilitation or physical therapy"

**Table:** Aries 2021

***Study characteristics***	
Methods	**Study aim:** "To determine whether task‐specific gait training is more effective following either (a) intensive hands‐on somatosensory stimulation or (b) wearing textured insoles" **Design:** parallel‐group RCT (mixed methods) **Country:** UK **Study recruitment and setting:** see Table **Involvement of patients/public/professionals/others:** yes
Participants	**Study population (number randomised):** 34 **Inclusion criteria:** Adults (aged 18 years or older) with stroke occurring in any brain area 42 to 112 days prior to providing informed consent Able to follow simple commands and imitate actions using the less paretic (ipsilesional) upper limb Able to walk independently prior to index stroke with or without a walking aid and unable to step on and off a 7.5 cm high block more than 12 times in 15 s with either the more paretic (contralesional) or less paretic (ipsilesional) lower limb **Exclusion criteria:** Any pathology or previous stroke affecting lower‐limb sensation (e.g. diabetic neuropathy, pressure sores etc.) Botox injection to the contralesional lower limb in the previous 6 months Pain sufficient to prevent participation in the intervention or completing the outcomes Known HIV, hepatitis non‐A or related condition (to meet sponsor requirements) **Participants:** detailed in Table **PROGRESS‐plus:** summarised in Table
Interventions	(1) Task‐specific gait training (TSGT) + mobilisation and tactile stimulation (MTS) (n = 19) "MTS+TSGT group participants received 30–60 min of standardised lower‐limb MTS ... MTS consisted of physical therapy techniques from six intervention categories: (1) passive and accessory movementsof joints, (2) massage and soft tissue stretch, (3) creating an active foot in preparation for stance/balance, (4) specific sensory input, (5) isolated/selective joint movement, and (6) patterns of coordinated movement underlying functional activity. The MTS schedule is a module rather than a single intervention, with flexibility to provide individualised interventions to each participant within the confines of the module. Research therapists selected and delivered appropriate combinations of techniques to address specific clinical needs of participants" (2) Task‐specific gait training (TSGT) + unlimited textured insole (TI) wearing (n = 15) "For TI+TSGT group participants, one TI was placed in the shoe worn on the contralesional foot, and one smooth insole in the shoe worn on the ipsilesional foot ... participants were encouraged to wear the insoles daily for as long as they chose, with advice to gradually increasewearing duration during the 6‐week intervention period." "Thirty minutes of TSGT were delivered to all participants in both groups, either immediately following MTS treatment (MTS+TSGT group), or while participants wore the insoles(TI+TSGT group)".
Outcomes	**Independence in ADL scales:** NR **Motor function scales:** modified Rivermead Mobility Index, lower extremity motricity index **Measures of balance:** NR **Measures of gait velocity:** 5‐m walk test **Length of stay:** NR **Adverse events:** adverse events recorded **Other measures:** ankle range of movement during gait, touch/pressure sensory thresholds in the feet, pressure under the feet during stance phase, Functional Ambulation Category **Time points when outcomes were assessed:** post‐intervention; 1‐month follow‐up
Comparison	**Amount of physical rehabilitation in groups:** differs **Categorisation of comparison:** additional therapy + usual therapy versus usual therapy (dose differs)
Funding & conflicts of interest	**Funding statement:** "AMA was funded by a National Institute for Health Research (NIHR), Clinical Academic Doctoral Fellowship for this research project. This report is independent research supported by the National Institute for Health Research (NIHR/HEE CAT Clinical Doctoral Research Fellowship, Mrs Alison Aries, CDRF‐2014‐05‐ 065). VMP was supported by the National Institute for Health Research (NIHR) Brain Injury MedTech Co‐operative based at Cambridge University Hospitals NHS Foundation Trust and University of Cambridge" **Conflict of interest statement:** "declare that the research was conducted in the absence of any commercial or financial relationships that could be construed as a potential conflict of interest."
Synthesis	**Quantitative synthesis:**Analysis 5.2, Analysis 5.4, Analysis 5.5, Analysis 6.2, Analysis 6.4 Gait results not suitable for quantitative analysis (not normally distributed).
Notes	

**Table:** Arya 2019

***Study characteristics***	
Methods	**Study aim:** "To develop an interlimb coupling protocol and assess its feasibility and effect on motor recovery, gait and disability among post‐stroke subjects" **Design:** parallel‐group RCT **Country:** India **Study recruitment and setting:** see Table **Involvement of patients/public/professionals/others:** no
Participants	**Study population (number randomised):** 50 **Inclusion criteria:** Unilateral stroke of > 6 months Paresis of right or left side Age between 40 and 70 years Functional ambulation category 2 and above Brunnstrom recovery stage of upper extremity and lower extremity ≥ 3 Muscle tone (using modified Ashworth scale): grade 1 to 2 **Exclusion criteria:** Receptive communication disorder Uncontrolled systemic or cardiovascular disease Ataxia Severe cognitive/perceptual deficit Musculoskeletal or peripheral nerve injury of any of the limbs **Participants:** detailed in Table **PROGRESS‐plus:** summarised in Table
Interventions	(1) Experimental group: Interlimb coupling "The principles utilized for formulating the experimental intervention are provided in Table 1. Various types of activities to impart experimental protocol are classified as Figure 1. Three criteria were used to classify the activities: bilateral/all limbs, symmetrical/asymmetrical movement, and iso‐/antidirectional movement. The bilateral movement is performed by either both the upper limbs or both the lower limbs. Bimanual performance, a type of bilateral movement, in which both the upper or lower limbs move independently without supporting each other, was also utilized. In the symmetrical movements, a joint of the one upper or lower limb moves in the similar pattern corresponding to the other upper or lower limb, whereas in an asymmetrical pattern, the movements of the same joints between the limbs are not similar. The iso‐/antidirectional movements are the movements that happen in either the same or different direction between the one upper limb and one lower limb. A matrix for selection of the activities as per the recovery stages of upper and lower limbs was also used to have a uniform distribution of various combinations of activities (Table 2). A total of 10 activities for each combination were provided to every experimental subject corresponding to the recovery stages of the limbs. Activities such as rowing machine, bicycle ergometer, wall cycle, and elliptical machine were utilized to perform the movement of either upper limbs or lower limbs or all four limbs (Figure 2(a–d)). The lists of the therapeutic activities developed are provided ...." (2) Control group: conventional rehabilitation "The conventional rehabilitation based on neurophysiological approaches such as Brunnstrom and Bobath approaches were imparted for the duration, frequency, and sessions similar to that of the experimental group. The techniques based on the Brunnstrom approach utilized reflexes to facilitate motor control and specific motor patterns to enhance and to break the synergistic movement patterns. The treatment method follows the hierarchical model of recovery as per the Brunnstrom recovery stage ... The Bobath, a neurodevelopmental technique, is imparted through the facilitation and handling methods in order to inhibit abnormal reflex activity and to relearn the normal movement patterns."
Outcomes	**Independence in ADL scales:** NR **Motor function scales:** Fugl‐Meyer Assessment **Measures of balance:** NR **Measures of gait velocity:** NR **Length of stay:** NR **Adverse events:** adverse events reported by participants **Other measures:** feasibility, Functional Ambulation Category, modified Rankin Scale **Time points when outcomes were assessed:** NR
Comparison	**Amount of physical rehabilitation in groups:** equivalent **Categorisation of comparison:** comparison of different approaches (dose equivalent; with no other physical rehab)
Funding & conflicts of interest	**Funding statement:** "funded by Indian Council of Medical Research." **Conflict of interest statement:** not reported
Synthesis	**Quantitative synthesis:**Analysis 7.1, Analysis 7.2
Notes	

**Table:** Baer 2007

***Study characteristics***	
Methods	**Study aim:** to evaluate the effectiveness of different task‐specific exercise practice regimes ‐ whole or part practice ‐ of functional tasks in a population of chronic stroke subjects **Design:** multi‐arm RCT (3 arms) **Country:** UK **Study recruitment and setting:** see Table **Involvement of patients/public/professionals/others:** no
Participants	**Study population (number randomised):** 64 **Inclusion criteria:** Chronic stroke **Exclusion criteria:** Not reported **Participants:** detailed in Table **PROGRESS‐plus:** summarised in Table
Interventions	(1) Walking practice (part) (n = not stated) Participants in the part practice group were taught "part practice exercises targeted at mobility and lower limb function". Length of intervention period: 4 weeks Number of sessions and length of individual sessions: total number of sessions: 3, but the length of individual sessions was not stated Intervention provider: research physiotherapist (2) Walking practice (whole) (n = not stated) Participants in the whole practice group were taught "whole practice exercises targeted at mobility and lower limb function". Length of intervention period: 4 weeks Number of sessions and length of individual sessions: total number of sessions: 3, but the length of individual sessions was not stated Intervention provider: research physiotherapist (3) Information (n = not stated) Participants in the control group were "given information about stroke" Length of intervention period: 4 weeks Number of sessions and length of individual sessions: total number of sessions: 3, but the length of individual sessions was not stated Intervention provider: research physiotherapist Comparisons relevant to this review: Comparison (a) ‐ walking practice (part) vs walking practice (whole). Comparison (b) ‐ walking practice vs information.
Outcomes	**Independence in ADL scales:** NR **Motor function scales:** Motor Assessment Scale **Measures of balance:** NR **Measures of gait velocity:** gait speed **Length of stay:** NR **Adverse events:** NR **Other measures:** Timed Up and Go over 2 metres (TUG2m), step test **Time points when outcomes were assessed:** "Baseline outcome measures were taken on two occasions prior to the intervention phase and meaned (base). The baseline was compared to outcomes taken at the end of intervention (OM3) and a 48 hour retention test (OM4)"
Comparison	**Comparison (a) ‐ walking practice (part) vs walking practice (whole)** **Amount of physical rehabilitation in groups:** equivalent **Categorisation of comparison:** 4. Comparison of similar approaches (dose equivalent) **Comparison (b): walking practice vs information** **Amount of physical rehabilitation in groups:** differs **Categorisation of comparison:** 1. Physical rehabilitation versus no or limited physical rehabilitation (dose differs) (limited = < 50% of dose)
Funding & conflicts of interest	**Funding statement:** Primary Care Research Fund, Chief Scientist Office, Scotland (grant 00B/3/19/F26) **Conflict of interest statement:** not reported
Synthesis	**Qualitative synthesis:** no suitable data available for analysis
Notes	Abstract only—research report platform presentation

**Table:** Bai 2008

***Study characteristics***	
Methods	**Study aim:** to evaluate the effects of standardised 3‐stage rehabilitation treatment on ADL and neurological deficit scores in people with ischaemic stroke **Design:** parallel‐group RCT **Country:** China **Study recruitment and setting:** see Table **Involvement of patients/public/professionals/others:** no
Participants	**Study population (number randomised):** 364 **Inclusion criteria:** Aged 40 to 80 years Admission[s] were stabilised within 1 week of symptoms Glasgow Coma Scale (GCS) score > 8 Limb disability **Exclusion criteria:** History of cerebrovascular disease History with residual symptoms Onset of ICH > 3 weeks prior Tetraplegia History of dementia Not resident locally **Participants:** detailed in Table **PROGRESS‐plus:** summarised in Table
Interventions	(1) Three‐stage rehabilitation (n = 183) "All patients underwent the same routine internal medical intervention, but those in the rehabilitation group underwent a standardized three‐stage rehabilitation program. Patients from both groups could receive help from their relatives, if necessary, patients from both groups had access to daily training for unilateral spatial neglect, speech deficit, and for swallowing, bowel, and bladder dysfunction" "The standardized three‐stage rehabilitation program included physical and occupational therapies and emphasized ADL training immediately after enrolment. These training programs were carried out by therapists in emergency hospitals, rehabilitation centers, and communities and were divided into primary, secondary, and third rehabilitation. The primary rehabilitation, which was aimed at practicing the basic ADL, was conducted at the Emergency Department or Neurology Department during the first month after stroke. The secondary rehabilitation was conducted at the Physical Department during the second and third month after stroke to help develop balance and walking. The third rehabilitation was conducted to enhance ADL and motor functions during the fourth to sixth month" Length of intervention period: 6 months Number of sessions and length of individual sessions: "During primary rehabilitation, individual physical therapy began within 24 hours of admission and was performed for 45 minutes per day, five days per week" Intervention provider: "Their relatives or nurses were trained to rehabilitate the patients at home. If patients were transferred to community centers instead, they were visited by therapists who directed their training every two weeks..treatment was conducted by one therapist per patient" (2) No physical rehabilitation (n = 181) "Routine internal medical intervention" Length of intervention period: no intervention Number of sessions and length of individual sessions: no intervention Intervention provider: no intervention
Outcomes	**Independence in ADL scales:** modified Barthel Index **Motor function scales:** Fugl‐Meyer Assessment **Measures of balance:** NR **Measures of gait velocity:** NR **Length of stay:** NR **Adverse events:** NR **Other measures:** Functional Comprehensive Assessment **Time points when outcomes were assessed:** "were administered at various times, including at admission and at one, three, and six months after the stroke"
Comparison	**Amount of physical rehabilitation in groups:** differs **Categorisation of comparison:** physical rehabilitation versus no or limited physical rehabilitation (dose differs)
Funding & conflicts of interest	**Funding statement:** not reported **Conflict of interest statement:** not reported
Synthesis	**Qualitative****synthesis** ‐ no data suitable for analysis (data shown in graphical representation only and SD not reported). Clarification of data sought but not obtained.
Notes	

**Table:** Bai 2013

***Study characteristics***	
Methods	**Study aim:** "To assess the value of acupuncture for promoting the recovery of patients with ischemic stroke and to determine whether the outcomes of combined physiotherapy and acupuncture are superior to those with physiotherapy alone" **Design:** multi‐arm RCT (3‐arm) **Country:** China **Study recruitment and setting:** see Table **Involvement of patients/public/professionals/others:** no
Participants	**Study population (number randomised):** 120 **Inclusion criteria:** Chinese patients aged 40 to 75 years with first time ischaemic stroke confirmed by CT or MRI Conscious after stroke Medically stable Admission 15 to 90 days after stroke onset Neurologic deficiency score for limb impairment **Exclusion criteria:** Medically unstable Conditions such as quadriplegia or pre‐existing cerebrovascular disease and left‐limb motor dysfunctions Patients who could not be followed up **Participants:** detailed in Table **PROGRESS‐plus:** summarised in Table
Interventions	(1) Physiotherapy + acupuncture (combined group) Physiotherapy: "According to the phase of hemiplegia, we adopted specific physiotherapies to improve patients’ activities of daily living (ADLs). Program for atonic stage (Brunnstrom stages 1–2). The goals of this stage are to prevent contracture of joints and secondary damages, promote normal sensorimotor sensation, and encourage optional independent movement. Patients were required to complete the following exercises: (1)correct limb placement; (2)passive joint movement; (3)turning the body over, following a correct method; (4)transferring from a decubitus to a sitting position; (5) bend‐the‐crab; and (6)neuromuscular facilitation. Each patient in this group underwent the preceding exercises 45 minutes perday, 6 consecutive days per week, for 4 weeks. Program for spasm stage (Brunnstrom stages 3–5). The goals of this stage were to decrease muscle tension, prevent spasms in the limbs, and promote separate movement. Patients were required to complete the following exercises: (1)antispasm limb placement, (2)trunk muscle distraction, (3)sitting balance, (4)transferring from a sitting to a standing position, (5)standing balance, (6)walking, and (7)upper‐limb control. Each patient in this group underwent the preceding exercises 45 minutes per day, 6 consecutive days per week, for 4 weeks." Acupuncture ‐ same as below (2) Acupuncture "The main acupoints were as follows: Baihui(GV20), Jianyu(LI15), Jianzhen(SI9), Quchi(LI11), Waiguan(TE5), Hegu(LI4), Yanglingquan(GB34), Kunlun(BL60), Juegu(GB39), Huantiao(GB30), Fengshi(GB31), Neiguan(PC6), Shangqiu(SP5), Tai‐chong(LR3), Yinlingquan(SP9), Sanyinjiao(SP6), Yingu(KI10), Daling(PC7), Hegu(LI4), Houxi(SI3), Jiquan(HT1), Chize(LU5), and Quze(PC3). Selection of the acupoints was based on Traditional Chinese Medicine theory. The depth of acupuncture depended on the somatotype of the patient. After a needle sensation was obtained, the needle was kept in situ for 30 minutes. Each patient in this group received acupuncture 30 minutes per day, 6 consecutive days per week, for 4 weeks." (3) Physiotherapy Physiotherapy only [this treatment group was not considered relevant to this review].
Outcomes	**Independence in ADL scales:** Modified Barthel Index **Motor function scales:** Simplified Fugl‐Meyer Assessment scale **Measures of balance:** NR **Measures of gait velocity:** NR **Length of stay:** NR **Adverse events:** NR **Other measures:** NR **Time points when outcomes were assessed:** day 0, day 28, and day 56
Comparison	**Amount of physical rehabilitation in groups:** differs **Categorisation of comparison:** 1. Physical rehabilitation versus no or limited physical rehabilitation (dose differs) (limited = < 50% of dose)
Funding & conflicts of interest	**Funding statement:** "supported by the major research project of Zhabei District Health Bureau (no. 2008‐05) and the scientific research project of Shanghai Municipal Health Bureau (no. 2009‐250)" **Conflict of interest statement:** "No competing financial interests"
Synthesis	**Quantitative synthesis:**Analysis 1.1, Analysis 1.2, Analysis 2.1, Analysis 2.2
Notes	

**Table:** Bai 2014

***Study characteristics***	
Methods	**Study aim:** "To investigate the effects of rehabilitation interventions on spasticity and ADL in ischemic stroke patients" **Design:** parallel‐group RCT **Country:** China **Study recruitment and setting:**Table **Involvement of patients/public/professionals/others:** no
Participants	**Study population (number randomised):** 165 **Inclusion criteria:** Aged 40 to 80 years First ever ischaemic stroke Stabilised for 1 week after stroke onset Medically stable (i.e. heart rate and blood pressure) Glasgow Coma Scale > 8 Motor function impairment consistent with brain lesion Agreed to sign a consent form **Exclusion criteria:** Major internal organ failures (i.e. the heart, lung, kidney, liver) Haematopoietic system dysfunction Malignant tumour Hypertensive emergency active infectious diseases (i.e. hepatitis, tuberculosis, HIV) Dementia and psychiatric history with an existing cognitive disorder History of traumatic brain injury resulting in current side effects Disability for another reason/or diseases (e.g. joint contracture caused by previous injury) Unable to undergo follow‐up evaluation **Participants:** detailed in Table **PROGRESS‐plus:** summarised in Table
Interventions	(1) Three stage rehabilitation programme First stage ‐ First month: "The rehabilitation treatments mainly involved physical therapy, which included passive movement and positioning of the affected limbs in a proper posture to avoid spasticity. We also strongly advocated patients in the TG doing early active exercisein bed, such as trying to move the paralyzed arm(s) or leg(s). Other fundamental treatments, such as sitting, standing, and balancing training, were performed according to the functional recovery of the patients. The treatments were performed 45 min/day and 5 days/week." Second stage ‐ Second and third month: "Passive stretching of spastic muscles was performed to ease the increased muscle tone in the elbow flexors, wrist, and finger flexors and plantar flexors. Antagonistic muscles were trained to enhance muscle strength and to counter balance the force among the muscles. Walking and balancing training were dominant components of rehabilitation at this stage. Walking up and down the stairs is a higher level of walking training and was performed if the abilities to walk and balance were good enough for the patients. Occupational therapy was emphasized in the functional recovery of upper extremities. Greater focus was on active exercise related to ADL. The treatments were performed 45 min twice a day and 5 days/week." Third stage ‐ Fourth to sixth month: "....visited patients every 2 weeks to provide rehabilitation advice at the patients’ homes. The relatives or caregivers of the patients were trained to supervise and assist the patients in performing active exercises on a daily basis for most of the waking hours. The training focused on ADL, such as using the affected arm to hold a bowl, putting clothes on and off, and putting trousers on and off, and soon. The treatments lasted until the end of the sixth month after enrollment". (2) No physical rehabilitation "only received standard medications in the hospital ward and at home."
Outcomes	**Independence in ADL scales:** Modified Barthel Index **Motor function scales:** NR **Measures of balance:** NR **Measures of gait velocity:** NR **Length of stay:** NR **Adverse events:** NR **Other measures:** Modified Ashworth Scale **Time points when outcomes were assessed:** after 3 and 6 months (6 month outcome = end of intervention)
Comparison	**Amount of physical rehabilitation in groups:** differs **Categorisation of comparison:** physical rehabilitation versus no or limited physical rehabilitation (dose differs)
Funding & conflicts of interest	**Funding statement:** "supported by the National Key Technologies Program ‘The Research of Three‐Stage Rehabilitation Program after Acute Cerebral Vascular Disease’ in the 10th 5‐year‐plan (Grant No. 2001BA703B21)." **Conflict of interest statement:** none
Synthesis	**Qualitative synthesis:** Results not presented in a format suitable for quantitative analysis.
Notes	

**Table:** Bale 2008

***Study characteristics***	
Methods	**Study aim:** to evaluate the effectiveness of functional strength training in subacute stroke patients **Design:** parallel‐group RCT **Country:** Norway **Study recruitment and setting:** see Table **Involvement of patients/public/professionals/others:** no
Participants	**Study population (number randomised):** 18 **Inclusion criteria:** First onset of stroke Reduced muscle strength in the affected leg Able to understand verbal information Able to sit without support **Exclusion criteria:** Significant sensory or cognitive sequelae Arrhythmia Uncontrolled angina pectoris or hypertension or co‐morbidities that could mask the sequelae from the stroke No motor control of the affected leg **Participants:** detailed in Table **PROGRESS‐plus:** summarised in Table
Interventions	(1) Strength training (n = 8) "Had training to improve the muscle strength of the lower extremities three days a week, and trained arm functions and activities of daily living the remaining two days. The functional strength training programme was designed to facilitate appropriate power in the weak muscles of the affected leg in graded activities or sequences of activities. Most of the exercises were weight‐bearing and also challenged standing balance" The exercises are well described in the appendix—including strength training exercises such as step‐ups, standing, sit‐to‐stand‐to‐sit, heel and toe rises, and bridging "Each strengthening exercise was performed according to the principle of 10 to 15 repetitions maximum to achieve moderate fatigue in one set" "The patients trained in ADLs such as walking, sitting‐to‐standing, stair climbing, etc. if time permitted after the strengthening exercises" Length of intervention period: 4 weeks Number of sessions and length of individual sessions: 50 minutes/d, 5 days a week Intervention provider: different physiotherapists trained participants in the 2 intervention groups (2) Bobath (n = 10) "The patients in the training‐as‐usual group had traditional training influenced by the Bobath Concept, with a central focus on normalizing muscle tone and movements on the affected side, symmetrical use of the body and relearning activities of daily living, often using manual guiding and facilitation techniques. Use of excessive muscle power was avoided to prevent associated reactions during training" "As part of their basic rehabilitation, all patients participated in multidisciplinary training programmes. Questionnaires were filled in by nurses and occupational therapists at week 3 of each patient’s training period to obtain information about attendance and quantity of training in the wards, and in sessions with occupational therapists" Length of intervention period: 4 weeks Number of sessions and length of individual sessions: 50 minutes/d, 5 days a week Intervention provider: different physiotherapists trained participants in the 2 intervention groups
Outcomes	**Independence in ADL scales:** NR **Motor function scales:** Motor Assessment Scale **Measures of balance:** NR **Measures of gait velocity:** gait speed over 12 metres **Length of stay:** NR **Adverse events:** NR **Other measures:** dynamometer measures of knee extension and flexion, maximum weight‐bearing, Patient Global Impression of Change **Time points when outcomes were assessed:** at inclusion and after 4 weeks
Comparison	**Amount of physical rehabilitation in groups:** equivalent **Categorisation of comparison:** comparison of different approaches (dose equivalent; with no other physical rehab)
Funding & conflicts of interest	**Funding statement:** "financially supported by theNorwegian Fund for Postgraduate Training inPhysiotherapy and by the Department forResearch in Førde Central Hospital (FSS)" **Conflict of interest statement:** not reported
Synthesis	**Quantitative synthesis:**Analysis 7.8, Analysis 7.10
Notes	

**Table:** Batchelor 2012

***Study characteristics***	
Methods	**Study aim:** "To determine whether a multifactorial falls prevention program reduces falls in people with stroke at risk of recurrent falls and whether this program leads to improvements in gait, balance, strength, and fall‐related efficacy" **Design:** parallel‐group RCT **Country:** Australia **Study recruitment and setting:** see Table **Involvement of patients/public/professionals/others:** no
Participants	**Study population (number randomised):** 156 **Inclusion criteria:** Aged 45 years+ Discharged home after rehabilitation At high risk of falls, based on whether they had fallen during hospital admission or had a Step Test (worse leg score of less than 7), or a BBS < 49 **Exclusion criteria:** Discharged to residential care facilities or with homes > 100 km from study sites **Participants:** detailed in Table **PROGRESS‐plus:** summarised in Table
Interventions	(1) Multifactorial falls prevention program (n = 71) "Implemented by a physiotherapist including: 1) an individualised home exercise program with strength, balance and walking components based on the Otago Exercise Programme ... Exercises will be selected to address balance and mobility problems identified in the baseline assessment and will be tailored to the level of each participant. The program will be monitored and modified by the physiotherapist at two subsequent home visits during the 12 month study period. Participants will be advised to undertake the exercise program at least five times per week. Participants will be provided with an exercise recording sheet to indicate completed exercises, and adherence to the exercise program will be assessed by the physiotherapist at the review visits. 2) falls risk minimisation strategies based on the falls risk factors identified in the baseline assessment in the context of falls prevention activities already in place, together with participant preference ... 3) education of the patient and their carer about identified falls risk factors and risk minimisation, including provision of information in verbal and written forms. This will be undertaken by the physiotherapist implementing the exercise program. 4) injury risk minimisation strategies for those identified as having a high risk for fracture based on falls risk, delayed walking post‐stroke or previous diagnosis of osteoporosis. These strategies will include prescription of hip protectors and liaison with the participant's general practitioner for consideration of vitamin D and calcium supplementation." "Participants in the intervention group will continue to receive usual care which consists of any activities undertaken by the participants recommended or administered by their treating team." (2) Control group (n = 85) "The control group will receive usual care which consists of any activities undertaken by the participants that are recommended or administered by their treating team. In addition, control participants will receive a general information booklet on falls prevention".
Outcomes	**Independence in ADL scales:** Functional Independence Measure (FIM), Human Activity Profile **Motor function scales:** NR **Measures of balance:** Step Test **Measures of gait velocity:** gait speed over 5 metres **Length of stay:** NR **Adverse events:** falls and proportion of fallers, injurious falls **Other measures:** sit‐to‐stand test, Falls Efficacy Scale, Falls Risk for Older People **Time points when outcomes were assessed:** after 12 months
Comparison	**Amount of physical rehabilitation in groups:** differs **Categorisation of comparison:** physical rehabilitation versus no or limited physical rehabilitation (dose differs)
Funding & conflicts of interest	**Funding statement:** "Supported by Australian National Health and Medical Research Council project (grant ID 385002) and training fellowship (ID310612)" **Conflict of interest statement:** "No commercial party having a direct financial interest in the results of the research supporting this article has or will confer a benefit on the authors or on any organization with which the authors are associated."
Synthesis	**Quantitative synthesis:** gait speed data included in Analysis 1.4 ADL data (FIM) presented as median and interquartile range, so not included in analysis.
Notes	

**Table:** Behrman 2011

***Study characteristics***	
Methods	**Study aim:** to establish the effectiveness and timing of locomotor training (including use of body‐weight support in treadmill stepping) to improve the ability to walk after a stroke **Design:** multi‐arm RCT (3‐arm) **Country:** USA **Study recruitment and setting:** see Table **Involvement of patients/public/professionals/others:** no
Participants	**Study population (number randomised):** 408 **Inclusion criteria:** Adults aged 18 years+ Stroke within 45 days before study entry Able to undergo randomisation within 2 months following stroke Residual paresis in leg affected by stroke Able to walk 3 m with assistance (from no more than one person) and able to walk 10 m of less than 0.8 m/s Able to follow a 3‐step command Treating physician approval to participate in the study Living in the community **Exclusion criteria:** Prior to the stroke, required assistance to complete ADLs Any contraindications to exercise Pre‐existing neurologic disorders Unable to travel to the treatment site **Participants:** detailed in Table **PROGRESS‐plus:** summarised in Table
Interventions	(1) Locomotor training programme group (n = 139) (also described as early locomotor training) Task‐specific walking training using body‐weight‐support‐treadmill and over‐ground practice provided in clinics plus usual care Length of intervention period: 12 to 16 weeks Number of sessions and length of individual sessions: 36 sessions; length of individual sessions not stated Intervention provider: physical therapists (2) Home exercise programme group (n = 126) "Impairment‐based, progressive strength and balance exercise at home plus usual care" Length of intervention period: 12 to 16 weeks Number of sessions and length of individual sessions: 36 sessions; length of individual sessions not stated Intervention provider: physical therapists (3) Usual care group (n = 143) (also described as late locomotor group; locomotor training started at 6 months) Length of intervention period: 12 to 16 weeks Number of sessions and length of individual sessions: 36 sessions; length of individual sessions not stated Intervention provider: physical therapists Comparison relevant to this review: home‐based training versus usual care, 6‐month data only
Outcomes	**Independence in ADL scales:** NR **Motor function scales:** Fugl Meyer score **Measures of balance:** Berg Balance Scale, activities‐specific balance confidence scale score **Measures of gait velocity:** walking speed **Length of stay:** NR **Adverse events:** NR (adverse events data reported after delivery of late locomotor training to 'usual care' group) **Other measures:** 6‐minute walk test, daily step number, Stroke Impact Scale **Time points when outcomes were assessed:** 6 months and 12 months post stroke
Comparison	**Amount of physical rehabilitation in groups:** differs **Categorisation of comparison:** additional therapy + usual therapy versus usual therapy (dose differs)
Funding & conflicts of interest	**Funding statement:** "Supported by the National Institute of Neurological Disordersand Stroke (RO1 NS050506) and the National Center for MedicalRehabilitation Research. The body‐weight support treadmill sys‐tems and the cost of all study screenings and interventions weresupported by funding from the National Institutes of Health" **Conflict of interest statement:** "Disclosure forms provided by the authors are available with the full text of this article at NEJM.org": https://www.nejm.org/doi/suppl/10.1056/NEJMoa1010790/suppl_file/nejmoa1010790_disclosures.pdf
Synthesis	**Qualitative synthesis only:** all data presented as changes from baseline.
Notes	

**Table:** Bek 2016

***Study characteristics***	
Methods	**Study aim:** evaluated the feasibility and efficacy of conductive education in stroke survivors in a RCT **Design:** parallel‐group RCT **Country:** UK **Study recruitment and setting:** see Table **Involvement of patients/public/professionals/others:** no
Participants	**Study population (number randomised):** 77 **Inclusion criteria:** Adults at any stage of postacute recovery from stroke were eligible Able to give informed consent No current medical concerns that precluded safe participation in a rehabilitation programme Sufficient level of language comprehension to complete questionnaires administered at baseline assessment (with assistance from a carer if required) **Exclusion criteria:** None ("no further inclusion or exclusion criteria were specified, with the expectation that the characteristics of the sample would reflect the broad range of individuals who might potentially benefit from a stroke rehabilitation programme") **Participants:** detailed in Table **PROGRESS‐plus:** summarised in Table
Interventions	(1) Conductive education "consisted of weekly 1.5‐hour sessions for 10 weeks, with up to five participants and two conductors per group. Sessions took place at the National Institute for Conductive Education in Birmingham, UK. All conductors had undergone three years of practical and theoretical training for a BA degree in Conductive Education. Partners or family members were permitted to attend and observe the session. Within each session, fine and gross motor skills were practised within task series, which were carried out in lying, sitting, and standing positions. Conductors use “rhythmical intention” to facilitate learning and action. Rhythmical intention is a link between motor and speech rhythm and is applied to each task in the programme. It is a loudly stated intention, expressed in the first person singular by the participants when performing the tasks; for example, “I lift my right arm up; 1‐2‐3‐4‐5”. Through verbalisation the conductor is able to present the required movement and its rhythm, which later becomes internalised as the skill becomes more automatic". (2) Control group "Participants in the waiting list group were invited to two introductory meetings during the 10‐week waiting period. In the first meeting, participants watched a short film about Conductive Education and were given copies of the CE participant handbook, followed by aninformal discussion session. In the second meeting, more detailed information on the CE programme was provided, and participants were given the opportunity to discuss their rehabilitation goals. All participants had access to routine NHS care and were asked to record any rehabilitation received within the study period"
Outcomes	**Independence in ADL scales:** Barthel Index, Stroke Impact Scale **Motor function scales:** NR **Measures of balance:** NR **Measures of gait velocity:** Timed Up and Go Test, 10‐metre walk test **Length of stay:** NR **Adverse events:** NR **Other measures:** Hospital Anxiety & Depression Scale **Time points when outcomes were assessed:** after 10 week intervention
Comparison	**Amount of physical rehabilitation in groups:** differs **Categorisation of comparison:** physical rehabilitation versus no or limited physical rehabilitation (dose differs)
Funding & conflicts of interest	**Funding statement:** "supported by Birmingham City Council (Adults and Communities) " **Conflict of interest statement:** "No competing interests"
Synthesis	**Quantitative synthesis:**Analysis 1.1, Analysis 1.4
Notes	

**Table:** Bhatia 2014

***Study characteristics***	
Methods	**Study aim:** "to compare the effect and effectiveness of task specific strength training and resistance training to improve strength and function in hemiparetic lower limb following stroke" **Design:** parallel‐group RCT **Country:** India **Study recruitment and setting:** see Table **Involvement of patients/public/professionals/others:** no
Participants	**Study population (number randomised):** 30 **Inclusion criteria:** Adults aged 45 to 65 years Ischaemic unilateral hemiparesis and MMSE >20, Brunstrom recovery stage > 3 and hemispheric stroke scale – gait component 3 **Exclusion criteria:** Medically unstable Medical conditions such as perceptual dysfunction, sensory deficits, or any other medical/orthopaedic problem involving the lower limb **Participants:** detailed in Table **PROGRESS‐plus:** summarised in Table
Interventions	(1) Task‐specific strength training "45 min/day, 3 days/week for a period of 4 weeks. The workstations are designed to strengthen the muscles of lower limbs in a functionally relevant way. The six workstations incorporated into the circuit were: standing and reaching in different directions for objects located beyond arm’s length to promote loading of the lower limbs and activation of lower limb muscles; sit‐to‐stand from various chair heights ; stepping forward and backward onto blocks of various heights; stepping sideways onto blocks of various heights; forward step‐up onto blocks of various heights; heels raises and lower while maintaining in a standing posture ... The amount and intensity of the exercise at each station were graded according to each subject’s functional level, which were given for 5 min in each workstation with 2 minutes rest between the workstations ... Progressions were made by increasing the number of repetitions completed within 5 min at a workstation and increasing complexity of the exercise performed at each workstation" (2) Resistance training "45mins/day, 3days/week, for 4 weeks. Hip flexors, extensors, abductors, adductors, knee flexors, extensors, ankle planexors, extensors, abductors, adductors, knee flexors, extensors, ankle plantar and Dorsiflexors were strengthened. Each muscle was given four warm‐up repetitions at 25% of the 1‐repetition maximum (1RM) with weight cuffs followed by 3 sets (8 to 10 repetitions per set) at 70% of the 1RM. 2 minutes rest given in between each set. Training intensity is adjusted biweekly by reassessing the 1RM".
Outcomes	**Independence in ADL scales:** NR **Motor function scales:** NR **Measures of balance:** NR **Measures of gait velocity:** Timed Up and Go Test **Length of stay:** NR **Adverse events:** NR **Other measures:** lower limb muscle strength **Time points when outcomes were assessed:** NR
Comparison	**Amount of physical rehabilitation in groups:** equivalent **Categorisation of comparison:** comparison of different approaches (dose equivalent; with no other physical rehab)
Funding & conflicts of interest	**Funding statement:** "None" **Conflict of interest statement:** "None"
Synthesis	**Quantitative synthesis:**Analysis 7.4
Notes	

**Table:** Blennerhassett 2004

***Study characteristics***	
Methods	**Study aim:** "to investigate whether additional practice of either upper limb or mobility tasks improved functional outcome during inpatient stroke rehabilitation" **Design:** parallel‐group RCT **Country:** Australia **Study recruitment and setting:** see Table **Involvement of patients/public/professionals/others:** no
Participants	**Study population (number randomised):** 30 **Inclusion criteria:** Able to walk 10 metres with close supervision (with or without walking aids) Able to provide informed consent **Exclusion criteria:** Deteriorating medical condition Independent community ambulators **Participants:** detailed in Table **PROGRESS‐plus:** summarised in Table
Interventions	(1) Movement science (n = 15) "In addition to the study intervention, all subjects received their usual interdisciplinary rehabilitation, which included one hour of physiotherapy, five days a week. This physiotherapy was based predominantly upon the Movement Science approach (Carr 2002). The duration of interdisciplinary therapy was recorded" "Both the Mobility and Upper Limb Groups received additional task‐related practice for one hour a day, five days per week for four weeks. After the four weeks training, participants ceased the additional practice and continued with their interdisciplinary rehabilitation program. Each session consisted of a circuit of 10 five‐minute workstations, with up to four subjects in each session. A physiotherapy department staff member supervised all sessions closely, and all activities were customised and progressed to suit individual subjects" Mobility circuit classes were conducted separately from the upper limb sessions. "Mobility Group activities included warm‐up and endurance tasks using stationary bikes and treadmills, followed by functional tasks such as sit to stand, step‐ups, obstacle course walking, standing balance, stretching as required, and strengthening using traditional gymnasium equipment" Length of intervention period: 4 weeks Number of sessions and length of individual sessions: "Usual interdisciplinary rehabilitation, which included one hour of physiotherapy, five days a week. Additional task‐related practice for one hour a day, five days per week" Intervention provider: physiotherapists (2) Upper limb training(n = 15) "Upper Limb Group activities commenced with a warm‐up (arm ergometer) followed by functional tasks to improve reach and grasp, hand‐eye coordination activities, stretching as required, and strengthening using traditional gymnasium equipment. Therapist‐assisted exercises were incorporated for subjects with limited control of arm or hand movement" Length of intervention period: 4 weeks Number of sessions and length of individual sessions: "Usual interdisciplinary rehabilitation, which included one hour of physiotherapy, five days a week. Additional task‐related practice for one hour a day, five days per week" Intervention provider: physiotherapists
Outcomes	**Independence in ADL scales:** NR **Motor function scales:** NR **Measures of balance:** NR **Measures of gait velocity:** Timed Up and Go test **Length of stay:** NR **Adverse events:** NR **Other measures:** Motor Assessment Scale (upper arm and hand), step test, Six‐Minute Walk test, Jebsen Taylor Hand Function Test **Time points when outcomes were assessed:** "Measures of both mobility and upper limb function were performed on three occasions: (1) prior to commencement in the trial, (2) immediately after the 4‐week additional training, and (3) at follow‐up six months after completing the additional training"
Comparison	**Amount of physical rehabilitation in groups:** equivalent **Categorisation of comparison:** physical rehabilitation versus attention control (dose of physical rehab differs; dose of intervention equivalent)
Funding & conflicts of interest	**Funding statement:** "financial support from the Royal Talbot Rehabilitation Centre Research Grant to employ independent assessor" **Conflict of interest statement:** not reported
Synthesis	**Quantitative synthesis:**Analysis 3.4; Analysis 3.5; Analysis 4.4
Notes	

**Table:** Bordoloi 2020

***Study characteristics***	
Methods	**Study aim:** "to determine, the effect of incorporating Rood’s approach in the HEP on the ability of independent self‐care in case of post stroke individuals of Assam" **Design:** parallel‐group RCT **Country:** India **Study recruitment and setting:** see Table **Involvement of patients/public/professionals/others:** no
Participants	**Study population (number randomised):** 236 **Inclusion criteria:** Adults aged 20 to 65 years Haemorrhagic stroke with supratentorial haematoma with hemiplegia Muscle power: 0 to 3 muscle grade by manual muscle testing **Exclusion criteria:** Health conditions including uncontrolled hypertension, severe dysphasia or cognitive impairment Previous disability in self‐care and/or previously living in a nursing home prior to the stroke **Participants:** detailed in Table **PROGRESS‐plus:** summarised in Table
Interventions	(1) Rood approach "taught exercises based on the Rood’s approach which included facilitation and inhibition with the help of exteroceptive stimulation, proprioceptive stimulation, vestibular stimulation, and repetitive purposeful activity. For facilitation quick stretch, resistance, tapping, quick icing, fast brushing, light touch, traction, approximation, heavy joint compression has been given. On the other hand for inhibition prolonged stretch, inhibitory tendon pressure, prolonged ice, slow rolling, rocking has been given. Along with the stimulation, patients were advised to do some repetitive purposeful activity; such as: For the upper limb‐wipe the table for 5 minutes, grasp a glass [jar?] and try to open it, touch a wall at the shoulder level and touch his/her cheek, touch hair, and slide a ball with the help of extensor aspect of forearm. For lower limb‐sitting to standing with support, kick a ball, standing to half sitting, walk with support." (2) Regular physiotherapy "All the patients and caretakers (both Group A and B) were instructed to follow a common HEP (home exercise programme) which included range of motion exercises, strengthening, stretching, weight bearing, balance and coordination exercises"
Outcomes	**Independence in ADL scales:** Barthel Index **Motor function scales:** NR **Measures of balance:** NR **Measures of gait velocity:** NR **Length of stay:** NR **Adverse events:** NR **Other measures:** NR **Time points when outcomes were assessed:** after 3 months of treatment
Comparison	**Amount of physical rehabilitation in groups:** differs **Categorisation of comparison:** additional therapy + usual therapy versus usual therapy (dose differs)
Funding & conflicts of interest	**Funding statement:** "None" **Conflict of interest statement:** "None"
Synthesis	**Quantitative synthesis:**Analysis 5.1
Notes	

**Table:** Brock 2005

***Study characteristics***	
Methods	**Study aim:** "To compare the short‐term effects of two physiotherapy approaches for improving ability towalk in different environments following stroke: (i) interventions based on the Bobath concept, in con‐junction with task practice, compared to (ii) structured task practice alone" **Design:** parallel‐group RCT **Country:** Australia and Germany **Study recruitment and setting:** see Table **Involvement of patients/public/professionals/others:** no
Participants	**Study population (number randomised):** 26 **Inclusion criteria:** First or recurrent stroke (haemorrhage or infarct) 4 to 20 weeks post‐stroke at commencement of trial Currently participating in an inpatient or outpatient rehabilitation programme Able to walk 15 metres indoors on a level surface (with or without an aid), with supervision **Exclusion criteria:** Independent mobility indoors achieved within four weeks post‐stroke Premorbid mobility limited to walking indoors only Unable to follow single stage verbal commands with visual prompts Mobility disability due primarily to pathologies other than stroke **Participants:** detailed in Table **PROGRESS‐plus:** summarised in Table
Interventions	(1) Bobath (n = 12) "Intervention A was based on the Bobath concept. In this intervention, participants received individual treatment prescription based on the Bobath concept towards the goal of improving walking ability in different environmental contexts. This intervention included detailed assessment of the individual’s movement strategies and the neurological and neuromuscular deficits underlying motor dysfunction. Treatment strategies were individualized and aimed at both reducing the severity of impairments where they impacted on function, and optimizing postural and movement strategies to improve efficiency and maximize function" "The specific goal of therapy in this study was to improve the ability of the participant to walk safely in different environments, including components of endurance, walking on slopes, going up and down a single step and walking over rough ground. The session incorporated structured task practice (as described in intervention B) for 1/6 of the treatment time allocated" Length of intervention period: 2 weeks Number of sessions and length of individual sessions: "Participants in both groups received six one hour physiotherapy sessions over a two‐week period. During the intervention period, participants did not receive any other physiotherapy aimed at improving mobility, posture, balance or lower limb function. Instead, the intervention sessions replaced the usual physiotherapy treatment for mobility. Additional physiotherapy was provided in sitting or lying for other rehabilitation goals, such as independence in bed mobility and recovery of upper limb function" Intervention provider: "Both interventions were performed by physiotherapists with at least five years’ postgraduate experience and at least two years’ experience in the fields of rehabilitation or neurology. In addition, therapists providing Intervention A had to have also completed a Basic Bobath Course and at least two Advanced Bobath Courses (a minimum of 180 hours of formal training acquired over a minimum of three years)" (2) Task practice (n = 14) "Participants receiving Intervention B undertook physiotherapy based on structured task practice. The supervised exercise programme aimed to provide repeated task specific practice of environmental contexts frequently encountered in walking outdoors. The tasks practised focused on increasing endurance, walking on slopes, going up and down a single step and walking over rough ground. Based on principles of motor learning, the therapist provided instruction in how to perform the task, including demonstration, verbal cueing to correct ineffective adaptive motor patterns and feedback on the performance of the task as specified by the study protocol, as well as supervision for safety. The therapist did not provide hands‐on assistance or guidance during tasks. Half of the task practice component was conducted as repetitive practice using standardized equipment in the gymnasium, reflecting the closed skill stage of learning. The other half of the time was spent in environments outside the gymnasium, walking on varying surfaces indoors and outdoors, bringing in other environmental contexts, such as differing attentional demands and changes in direction" Intervention and functional task training Length of intervention period: 2 weeks Number of sessions and length of individual sessions: "Participants in both groups received six one hour physiotherapy sessions over a two‐week period" Intervention provider: "Physiotherapists with at least five years’ postgraduate experience and at least two years’ experience in the fields of rehabilitation or neurology"
Outcomes	**Independence in ADL scales:** NR **Motor function scales:** NR **Measures of balance:** Berg Balance Scale **Measures of gait velocity:** gait velocity **Length of stay:** NR **Adverse events:** NR **Other measures:** adapted Six‐Minute Walk test **Time points when outcomes were assessed:** "Measures were taken at baseline, and following treatment…Post‐intervention assessment measures were recorded between one and five days following the intervention"
Comparison	**Amount of physical rehabilitation in groups:** equivalent **Categorisation of comparison:** comparison of different approaches (dose equivalent; with no other physical rehab)
Funding & conflicts of interest	**Funding statement:** "supported by the St Vincent’sHospital, Melbourne Research Endowment Fundgrant number 2005/53" **Conflict of interest statement:** "Authors Kim Brock and Gerlinde Haase are members of the International Bobath Instructor Training Association and teach short courses for postgraduate physiotherapists for a teaching fee"
Synthesis	**Quantitative synthesis:**Analysis 7.9; Analysis 7.10
Notes	

**Table:** Brouwer 2018

***Study characteristics***	
Methods	**Study aim:** "To explore the effectiveness of a 2‐week client‐centered rehabilitation intervention (tune‐up) delivered 6 months after inpatient discharge on community" **Design:** parallel‐group RCT **Country:** Canada and UK **Study recruitment and setting:** see Table **Involvement of patients/public/professionals/others:** no
Participants	**Study population (number randomised):** 103 **Inclusion criteria:** First major unilateral hemispheric stroke requiring inpatient rehabilitation English speaking Adequate verbal communication Scheduled for discharge home **Exclusion criteria:** Serious comorbidities (e.g. cancer, amputation, mobility‐limiting arthritis) **Participants:** detailed in Table **PROGRESS‐plus:** summarised in Table
Interventions	(1) Tune‐up intervention "Participants collaborated with the therapist to identify 3 specific goals, at least 2 of which were related to mobility. This client‐centered approach was adopted to optimize the treatment relevance, which is known to influence its effectiveness in improving outcomes, and to mimic typical clinical practice ... Tune‐up interventions comprised 6 individualized treatment sessions based on the goals identified, each lasting about 1 hour and delivered 3 times/wk for 2 weeks in participants’ homes. Sessions included 5 key elements tailored to each participant’s needs and specific goals: strength/power, balance, cardiovascular endurance, motor coordination, and education about relevant community resources, as well as how to monitor their mobility and activities" (2) Control "....were invited to participate in a longitudinal study to examine the natural progression of mobility and function through regular assessment over a 15‐month period, thus eliminating any possibility of biases caused by conscious or uncon‐scious participant opinions about the benefit of additional therapy..."
Outcomes	**Independence in ADL scales:** NR **Motor function scales:** NR **Measures of balance:** Berg Balance Scale **Measures of gait velocity:** Timed Up and Go Test, 6‐minute Walk Test **Length of stay:** NR **Adverse events:** NR **Other measures:** Subjective Index of Physical and Social Outcome, Stair Ascent Test, SF‐36 **Time points when outcomes were assessed:** baseline, 3, 6, 9, 12, and 15 months
Comparison	**Amount of physical rehabilitation in groups:** differs **Categorisation of comparison:** physical rehabilitation versus no or limited physical rehabilitation (dose differs)
Funding & conflicts of interest	**Funding statement:** "Supported by the Heart and Stroke Foundation of Ontario (grant no. SRA 5974)" **Conflict of interest statement:** "None"
Synthesis	**Quantitative synthesis:**Analysis 1.3; Analysis 1.4; Analysis 2.3; Analysis 2.4
Notes	

**Table:** Bui 2019

***Study characteristics***	
Methods	**Study aim:** "To determine whether ther combination between modified acupuncture and motor relearning method can improve treatment outcomes" **Design:** parallel‐group RCT **Country:** Vietnam **Study recruitment and setting:** see Table **Involvement of patients/public/professionals/others:** no
Participants	**Study population (number randomised):** 66 **Inclusion criteria:** Conscious and co‐operative with Barthel scores ≤ 60 Able to give consent **Exclusion criteria:** Other conditions including ulceration, inflammation or cachexia due to lengthy lying in bed, or complicated changes that needed other treatments during the research process **Participants:** detailed in Table **PROGRESS‐plus:** summarised in Table
Interventions	(1) Motor relearning + acupuncture "Patients in the trial group did 4 specific tasks, including balance, walking, standing up ‐ sitting down, and reaching ‐ manipulation. In each task, the exercises were conducted based on patients ability and performance. Every day, patients did the four exercises at least once, and the tasks were repeated based on the patients health status" (2) Bobath + acupuncture "Patients in the control group did exercises with 4 different positions including lying position, sitting poistion, standing position and walking exercise" Modified acupuncture comprised 3 steps: "patients were given electroacupuncture in 20 minutes. This process was conducted 1 time/day from Monday to Friday until finishing 30 days of treatment"
Outcomes	**Independence in ADL scales:** Barthel Index **Motor function scales:** NR **Measures of balance:** NR **Measures of gait velocity:** "ability to walk" (dichotomised into can or cannot walk); abstract states 10‐m walk test, but no data provided **Length of stay:** NR **Adverse events:** NR **Other measures:** 10‐hole Peg Test **Time points when outcomes were assessed:** Every 10 days of treatment (10, 20, and 30 days)
Comparison	**Amount of physical rehabilitation in groups:** equivalent **Categorisation of comparison:** comparison of different approaches (dose equivalent; with no other physical rehab)
Funding & conflicts of interest	**Funding statement:** "Funding provided in part by research grants from the Department of Science and Technology, Soc Trang province" **Conflict of interest statement:** not reported
Synthesis	**Quantitative synthesis:**Analysis 7.1
Notes	

**Table:** Candan 2017

***Study characteristics***	
Methods	**Study aim:** "To examine the efficacy of modified constraint‐induced movement therapy (mCIMT), which was applied to lower extremity, on strength and quality of life (QoL) in patients with stroke" **Design:** parallel‐group RCT **Country:** Turkey **Study recruitment and setting:** see Table **Involvement of patients/public/professionals/others:** no
Participants	**Study population (number randomised):** 30 **Inclusion criteria:** Clinical diagnosis of a single, unilateral stroke and medically stable Adult aged 18 years + Time since the stroke 3 and 12 months Mild to moderate disability according to lower extremity recovery stages (Brunnstrom recovery stages III‐V) Not involved in any rehabilitation programme Able to participate in an intensive rehabilitation programme **Exclusion criteria:** Recurring stroke Medical comorbidities or additional neurological and/or orthopaedic problems Cognitive impairment (MMSE < 24) Excluded from the study if they missed ≥ 3 consecutive treatment sessions **Participants:** detailed in Table **PROGRESS‐plus:** summarised in Table
Interventions	(1) mCIMT (modified constraint induced movement therapy) (n = 18) "mCIMT was conducted for the paretic lower limb in the study group for two weeks, per week five day and each session 120 minutes. CIMT included three main components: the intensive practice of the functional activities, limited use of the nonparetic lower limb and transferring the gains from the training session to the patient’s real environment with “transfer package”. The intensive practice was applied to the selection of the appropriate functional activities by the “shaping” principles by the feedback, coaching, modeling, and encouragement of the physiotherapist. The functional activities are described in Table 1. Each of the functional activities was performed intensively by the paretic lower limb, unilaterally, over 20 minutes, repetitively. After each 20‐minute activity period, a rest period of 5 minutes was given to the patients. The constraint of the nonparetic lower limb was known to be difficult because of the bilateral characteristics of lower limb function. Therefore, the constraint was fulfilled by a combination of 2 different methods; 1) immobilization of the knee of the nonparetic limb with a whole leg orthosis and 2) use of a shoe insert on the nonparetic lower limb (Figure 1)." "The restriction was used during treatment sessions, including on the treadmill, and for 90% of the participants’ waking hours. When the patient took off the orthosis, the range of motion exercises of the knee was asked to perform to prevent the contracture. The used functional activities during therapy session were set to home as a home program with transfer package, and the participants were strongly encouraged to incorporate the use of their paretic lower limb into their daily activities." (2) Rehabilitation programme (n = 15) Note: study authors describe group (2) as "neurodevelopmental therapy (NDT)". However, this name is not used here as the intervention description differs from what is traditionally considered NDT. "....program was applied for two weeks, per week five per day and each session lasted 120 minutes in the control group. The program consisted several exercises that ba‐sed on motor‐learning principles (specificity, progressive overload, and variation). Functional exercises were chosen according to the participants’ abilities and needs. NDT required continuous observation, evaluation, progression and modification of the program dealing with participants’ progression. Exercises in NDT program were performed in different positions (supine, prone, sitting and standing)including weight‐bearing exercises, balance activities and walking ".
Outcomes	**Independence in ADL scales:** **Motor function scales:** **Measures of balance:** Berg Balance Scale **Measures of gait velocity:** 10‐metre walk test, cadence **Length of stay** **Adverse events** **Other measures:** Stroke Impact Scale (SIS) v 3.0 [Turkish] lower extremity strength and mobility, Functional Ambulation Classification, step length asymmetry **Time points when outcomes were assessed:** post‐treatment
Comparison	**Amount of physical rehabilitation in groups:** equivalent **Categorisation of comparison:** comparison of different approaches (dose equivalent; with no other physical rehab)
Funding & conflicts of interest	**Funding statement:** "None" **Conflict of interest statement:** "None"
Synthesis	**Quantitative synthesis:**Analysis 7.3; Analysis 7.4
Notes	

**Table:** Cao 2014

***Study characteristics***	
Methods	**Study aim:** "To study the effect of intensive walking training (SAT) in the limb rehabilitation of early stroke patients with hemiplegia" **Design:** parallel‐group RCT **Country:** China **Study recruitment and setting:** see Table **Involvement of patients/public/professionals/others:** no
Participants	**Study population (number randomised):** 86 **Inclusion criteria:** Diagnosed using head CT or MRI, and met the HAS diagnostic criteria formulated in the Fourth National Cerebrovascular Disease Conference Conscious and able to co‐operate with treatment **Exclusion criteria:** Severe cognitive impairment other co‐morbid conditions involving the lung, kidney, and other organs or malignant tumours or conditions affecting one limb Other illnesses which may preclude participation Poor co‐operation **Participants:** detailed in Table **PROGRESS‐plus:** summarised in Table
Interventions	(1) Walking training Provided in addition to routine rehabilitation. This comprised: ‐ Health education ‐ assessment by designated rehabilitation therapist who assigns a training plan. Education of patient and family, including on assisting with rehabilitation training. ‐ Seated balance training: "Sitting training can be implemented 48‐72 hours after the patient's main vital signs are stable and the symptoms of the nervous system no longer progress. Ask the patient to sit up from the lateral position to the bedside, bend the neck and trunk sideways, use the arm of the unaffected upper limb as a lever to hook the affected leg, and then move the legs to the bedside. Push the legs to facilitate the patient to sit up. For training, choose a bench with a height of 40 cm, which is conducive to a more stable landing of the lower limbs. Gradually make the patient transition from an unsupportive sitting position to a stationary sitting position that does not require support. Ask the patient to train. At the same time, adjust the posture by moving the center of gravity, and finally obtain the balance in the sitting position." ‐ Standing balance training: "Getting the patient to stand as early as possible is usually the most ideal rehabilitation goal. During the training, the patient's feet can be moved back, the hips are bent, and the trunk is required to keep straight and forward, and then the back shoulders are moved forward over their toes and knees, the center of gravity reaches their feet, and then the body is forward and upward. stand up. During training, the upper and lower limbs on the affected side were immobilized with splints and supported externally. Avoid synergistic and antagonistic imbalances that lead to overstretching of muscle groups. Actively correct the resting alignment of the patient's joints to improve their functional position. Avoid knee flexion, fix a 1kg sandbag on the ankle of the affected limb to make it bear the weight, and the patient stands with the help of the walker with both hands. When in a standing position, the feet should be shoulder‐width apart with the hips and knees extended, keeping the torso upright. Ask them to stand with weight and move their center of gravity back and forth to perform static and dynamic balance training." ‐ Walking training: "Standing with weight and actively implementing walking training in the early stage is more likely to promote the recovery of trunk strength. In the early stage, post‐weight‐bearing walking training was carried out, 2 times a day, 10‐15 minutes/time, and gradually extended to 45 minutes/time. During walking training, the splint should be fixed on the upper and lower limbs of the affected side, and a 1kg sandbag should be fixed on the ankle of the lower limb to make the affected leg bear weight, and it is easier to train the patient to stand. The trainer stands on the lateral side of the patient's affected side, and holds the patient's belt to control the patient to avoid falling injury. The patient was asked to first step the affected leg, extend the hip, and control the knee extension to prepare for the swing phase of the unaffected leg. If you have difficulty walking, you can be assisted by a trainer." ‐ Stair training: "When the Brunstrom level of the patient's lower extremities exceeds level 3, the training of going up and down the stairs can be implemented, 2 times/day, 45 minutes/time. The trainer is located on the hemiplegic side behind the patient to assist him in completing the training. When going up the stairs, the affected leg should go up first, and when going down the stairs, the healthy leg should go down first. Actively encourage and praise patients for their progress and build confidence." (2) Routine rehabilitation therapy "The control group received routine rehabilitation therapy, including good limb placement and instructing family members to perform passive massage of the upper and lower limbs on the affected side."
Outcomes	**Independence in ADL scales:** modified Barthel Index **Motor function scales:** NR **Measures of balance:** NR **Measures of gait velocity:** NR **Length of stay:** NR **Adverse events:** NR **Other measures:** upper limb motor function score; number able to walk independently **Time points when outcomes were assessed:** before and after 4 weeks of treatment
Comparison	**Amount of physical rehabilitation in groups:** differs **Categorisation of comparison:** 5. Additional therapy + usual therapy versus usual therapy (dose differs)
Funding & conflicts of interest	**Funding statement:** not reported **Conflict of interest statement:** not reported
Synthesis	**Quantitative synthesis:**Analysis 5.1
Notes	This paper was published in Chinese and translated using Google Translate.

**Table:** Capisizu 2016

***Study characteristics***	
Methods	**Study aim:** not reported **Design:** multi‐arm RCT (3‐arm) **Country:** Romania **Study recruitment and setting:** see Table **Involvement of patients/public/professionals/others:** no
Participants	**Study population (number randomised):** 115 **Inclusion criteria:** First unilateral stroke, 10 days post stroke No severe cognitive impairment or depression **Exclusion criteria:** MMSE ≤ 12 Clock drawing test ≤ 4 Geriatric Depression Scale ≥ 8 **Participants:** detailed in Table **PROGRESS‐plus:** summarised in Table
Interventions	(1) Cerebrolysin "neuroprotection/neurotrophicity/neuroplasticity therapy (NT) pharmacological multimodal therapy consisted of Cerebrolysin (60 mg Cerebroprotein hydrolysate diluted in 250 ml of normal saline solution) administered in the morning over ten consecutive days per treatment period" (2) Physical therapy (PT) Physical therapy (PT) consisting of isolated passive and active movements followed by switching from bed to a chair and walking with or without assistance, as well as other complex movements involving co‐ordination and balance (e.g. walking up or down the stairs, moving safely between obstacles, etc.) (3) Combined ‐ Cerebrolysin + PT Comparison of relevance to this review = (1) vs (3)
Outcomes	**Independence in ADL scales:** Barthel Index **Motor function scales:** NR **Measures of balance:** Tinetti Balance Assessment Tool **Measures of gait velocity:** NR **Length of stay:** NR **Adverse events:** NR **Other measures:** Reisberg Global Deterioration Scale (cognitive function) **Time points when outcomes were assessed:** post‐treatment
Comparison	**Amount of physical rehabilitation in groups:** differs **Categorisation of comparison:** physical rehabilitation versus no or limited physical rehabilitation (dose differs)
Funding & conflicts of interest	**Funding statement:** not reported **Conflict of interest statement:** not reported
Synthesis	**Qualitative synthesis:** data for Barthel Index are not presented in the paper
Notes	

**Table:** Carlson 2006

***Study characteristics***	
Methods	**Study aim:** "investigated the effects of intense massed practice on balance, gait speed, and weight bearing ratios" **Design:** prallel‐group RCT **Country:** USA **Study recruitment and setting:** see Table **Involvement of patients/public/professionals/others:** no
Participants	**Study population (number randomised):** 11 **Inclusion criteria:** Not reported ("Eleven subjects with hemiparesis... participated in this study") **Exclusion criteria:** Not reported **Participants:** detailed in Table **PROGRESS‐plus:** summarised in Table
Interventions	(1) Rehabilitation (n = 6) "received intense massed practice ... with interventions focused on forcing use of the affected lower extremity" Length of intervention period: 2 weeks Number of sessions and length of individual sessions: 3 hours/d Intervention provider: not stated (2) No intervention (n = 5) "Control subjects did not receive any intervention" Length of intervention period: no intervention Intervention provider: no intervention Number of sessions and length of individual sessions: no intervention
Outcomes	**Independence in ADL scales:** NR **Motor function scales:** NR **Measures of balance:** Berg Balance Scale **Measures of gait velocity:** self‐selected gait speed **Length of stay:** NR **Adverse events:** NR **Other measures:** weight‐bearing ratios in quiet standing and weight‐bearing ratios during ambulation **Time points when outcomes were assessed:** "testing was performed at pre‐test, post‐test, and again at 3‐month follow‐up"
Comparison	**Amount of physical rehabilitation in groups:** differs **Categorisation of comparison:** physical rehabilitation versus no or limited physical rehabilitation (dose differs)
Funding & conflicts of interest	**Funding statement:** not reported **Conflict of interest statement:** not reported
Synthesis	**Qualitative synthesis:** no data suitable for analysis
Notes	Abstract only

**Table:** Chae 2017

***Study characteristics***	
Methods	**Study aim:** "To investigate the effect of phase proprioceptive training on balance in patients with chronic stroke" **Design:** parallel‐group RCT **Country:** Korea, Rep. **Study recruitment and setting:** see Table **Involvement of patients/public/professionals/others:** no
Participants	**Study population (number randomised):** 30 **Inclusion criteria:** History of stroke (within the last 6 months or more) Able to understand the directions i.e. MMSE‐K score of ≥ 24 **Exclusion criteria:** Presence of acute musculoskeletal symptoms Inability to communicate, or unable to participate in the study as judged by the researchers **Participants:** detailed in Table **PROGRESS‐plus:** summarised in Table
Interventions	(1) Proprioceptive training (n = 15) "Stage 1: Stable stage (Stable surface) Standing training on stable floor (1st set: EO, 2nd set: EC) 4 mins/set; Maintain standing position 10 sec/5 times/set; Both heels up and down 10 sec/5 times/set; Maintain standing on one leg by turns 10 sec/5 times/set. Stage 2: Dynamic stage (Unstable surface) Standing training on unstable surface (Airex balance surface)(1st set: EO, 2nd set: EC) 6 mins/set; Maintain standing position10 sec/5 times/set; Both heels up and down10 sec/5 times/set; Maintain standing on one leg by turns 5 sec/10 times/set; Forward one step by turns 10 sec/5 times/set. Stage 3: Functional stage; Standing training on unstable surface (Airex balance surface) 10 min; Walking on the same place 6 min; Both feet together, standing with back and forth foot position 10 sec/5 times; Sit down→ Squat position→ Stand up 1 min" (2) General physical therapy group (n = 15) "The control group underwent Bobath neuro development treatment and proprioceptive neuromuscular facilitation19), range of motion exercises, stretch exercises, upper and lower limb muscle strength exercises, gait training, bike exercise 20), and other general physical therapies applied to the group for 60 minutes, 5 times a week for 4 weeks." "Participants in the proprioceptive training group underwent proprioceptive training and received general physical therapy each for a total of 20 thirty‐minute sessions, five times per week, during a period of four weeks; the control group received general physical therapy for a total of 20 sixty‐minute sessions, five times per week, during a period of four weeks"
Outcomes	**Independence in ADL scales:** NR **Motor function scales:** NR **Measures of balance:** Berg Balance Scale **Measures of gait velocity:** Timed Up and Go Test **Length of stay:** NR **Adverse events:** NR **Other measures:** Activities‐specific Balance Confidence Scale **Time points when outcomes were assessed:** post‐intervention
Comparison	**Amount of physical rehabilitation in groups:** equivalent **Categorisation of comparison:** comparison of different approaches (dose equivalent; with both/all groups also receiving other physical rehab)
Funding & conflicts of interest	**Funding statement:** not reported **Conflict of interest statement:** not reported
Synthesis	**Quantitative synthesis:**Analysis 7.3; Analysis 7.4
Notes	

**Table:** Chan DY 2006

***Study characteristics***	
Methods	**Study aim:** "To study the efficacy of the motor relearning approach in promoting physical function and task performance for patients after a stroke" **Design:** matched‐pair RCT **Country:** Hong Kong **Study recruitment and setting:** see Table **Involvement of patients/public/professionals/others:** no
Participants	**Study population (number randomised):** 66 **Inclusion criteria:** 21 to 65 years of age (eligible admission to outpatient setting) Diagnosed as having suffered a first stroke The stroke must have occurred within the previous 12 months Able to follow simple instructions (as screened by the Chinese version of the Cognistat) **Exclusion criteria:** Conditions were complicated by other musculoskeletal injuries and/or visual deficit **Participants:** detailed in Table **PROGRESS‐plus:** summarised in Table
Interventions	(1) Motor relearning group (n = 33) "Both programmes were conducted in the same treatment area located in the occupational therapy department of the hospital. The patients in both programmes also received physical therapy in the form of lower limb strengthening and trunk balance exercises. The patients attended physical therapy from one to three times a week. Their attendance was monitored throughout the study, and the number of sessions was incorporated into the analysis of the results" "In the motor relearning programme, the intervention technique followed four sequential steps: identification of the missing performance components (step 1), training using remedial exercises (step 2), training using functional task components (step 3), and transfer of skills to functional task performance (step 4). A total of 24 remedial tasks (used in step 2) and 10 functional tasks (used in step 4) were designed to cover deficits in static and dynamic sitting balance, and static and dynamic standing balance. Throughout the training session, the therapist stressed the importance of relating the training processes taking place in steps 2 and 3 to practices in step 4. The training progressed by advancing from a sitting to a standing position, and from static to dynamic balancing. The criteria established for progression were clearly defined, and those patients who demonstrated the desirable skills were upgraded to another stage of training" Length of intervention period: 6 weeks Number of sessions and length of individual sessions: 3 x 2‐hour sessions each week (18 sessions total) Intervention provider: occupational therapist: "The researcher conducted the motor relearning programme" (2) Skills training group (n = 33) "The skill training method was adopted for use with patients receiving the conventional therapy programme. The same number of remedial and functional tasks was covered as in the motor relearning programme. The selection of remedial tasks followed the principle of progressing from a sitting to a standing position, and from static to dynamic balance. The training of functional tasks began with simple self‐care and bedside tasks, such as bed mobility, and progressed to more complicated tasks, such as use of community facilities. Unlike the motor relearning programme, the patients were not involved in identifying their own missing performance components (Table 22). The relationships between the practices of the remedial tasks and the entire functional task performance were not reinforced" Length of intervention period: 6 weeks Number of sessions and length of individual sessions: 3 x 2‐hour sessions each week (18 sessions total) Intervention provider: "another occupational therapist conducted the conventional therapy programme. The therapist responsible for the conventional therapy programme was trained in the standardized procedure for conducting the control programme"
Outcomes	**Independence in ADL scales:** Functional Independence Measure **Motor function scales:** NR **Measures of balance:** Berg Balance Scale **Measures of gait velocity:** Timed Up and Go test **Length of stay:** NR **Adverse events:** NR **Other measures:** instrumental ADL, Community Integration Questionnaire **Time points when outcomes were assessed:** "The assessment schedule was laid out at baseline (after randomization), two weeks, four weeks, and six weeks"
Comparison	**Amount of physical rehabilitation in groups:** equivalent **Categorisation of comparison:** 4. Comparison of similar approaches (dose equivalent)
Funding & conflicts of interest	**Funding statement:** not reported **Conflict of interest statement:** not reported
Synthesis	**Qualitative synthesis:** Not included in analysis as the two active treatment groups were classified as being similar treatment approaches.
Notes	

**Table:** Chang 2015

***Study characteristics***	
Methods	**Study aim:** not reported **Design:** parallel RCT **Country:** China **Study recruitment and setting:** see Table **Involvement of patients/public/professionals/others:** no
Participants	**Study population (number randomised):** 50 **Inclusion criteria:** Diagnosed in accordance with Chinese guidelines medicine for stroke CT or MRI confirmation of the stroke Within 2 weeks of stroke onset Medically stable Persistent limb disability GCS > 8 **Exclusion criteria:** Not reported **Participants:** detailed in Table **PROGRESS‐plus:** summarised in Table
Interventions	Both groups received conventional bedside treatment and nursing care. (1) Three‐stage rehabilitation Stage 1: breathing training, positioning to reduce spasticity and contractures while the stroke survivor is in bed, using neural promotion technology, passive movement of the joints of the paretic limb and active movement of the joints of the non‐paretic limb, neural network and functional electrical stimulation. Sitting and balance training, bed ADL training. Tailored exercises chosen based on what the stroke survivor can do. Two sessions per day, each session 40 mins, delivered 5 days/week Stage 2: when the patient's physical fitness has improved, they are transferred to the rehabilitation centre for further treatment, including transfer training, sit‐to‐stand training, standing balance training, neural promotion technology, weight training, gait training, and stair climbing training. Three sessions per day, each session 40 mins delivered 6 days/week Stage 3: carried out at the patient's home, and the rehabilitation instructor regularly visits the stroke survivor to guide the patient to perform some necessary training, including guiding the patient to adapt to the home environment, independently performing ADL and other activities. One session per week with the follow‐up ending after 6 months of treatment (2) No physical rehabilitation Participants received conventional clinical treatment and nursing intervention without any relevant rehabilitation training
Outcomes	**Independence in ADL scales:** Barthel index **Motor function scales:** NR **Measures of balance:** NR **Measures of gait velocity:** NR **Length of stay:** NR **Adverse events:** NR **Other measures:** Brunnstrom assessment **Time points when outcomes were assessed:** before and after treatment
Comparison	**Amount of physical rehabilitation in groups:** differs **Categorisation of comparison:** 1. Physical rehabilitation versus no or limited physical rehabilitation (dose differs) (limited = < 50% of dose)
Funding & conflicts of interest	**Funding statement:** not reported **Conflict of interest statement:** not reported
Synthesis	**Quantitative synthesis:**Analysis 1.1
Notes	This study was published in Chinese and relevant sections translated into English by our author team (PLC).

**Table:** Chan WN 2017

***Study characteristics***	
Methods	**Study aim:** "This study investigated the effect of Tai Chi training on dual‐tasking performance that involved stepping down and compared it with that of conventional exercise among stroke survivors" **Design:** multi‐arm RCT (3‐arm) **Country:** Hong Kong SAR, China **Study recruitment and setting:** see Table **Involvement of patients/public/professionals/others:** no
Participants	**Study population (number randomised):** 26 **Inclusion criteria:** Aged 50 or above Diagnosed with stroke 6 or more months previously Able to perform a stepping down manoeuvre without any physical assistance Able to follow instructions in Cantonese **Exclusion criteria:** Any neurological disease other than stroke Severe visual or hearing impairment MMSE (Cantonese version) < 18 Any major surgery or severe musculoskeletal injury during the previous 6 months **Participants:** detailed in Table **PROGRESS‐plus:** summarised in Table
Interventions	(1) Tai Chi (n = 9) "Subjects in the Tai Chi group were taught a modified 12‐form Yang‐style Tai Chi" (2) Conventional exercise (n = 8) "Conventional exercises, which included upper and lower limbs mobilization, stretching, and muscle strengthening with elastic bands, as well as walking, were conducted in this group. Most of the exercises were performed in sitting except for some of the lower limb and walking exercises." "Subjects in this group attended two one‐hour training sessions each week, with a total of 24 sessions." (3) Control (n = 9) "No training was provided to the control group during the 12‐week intervention period. They continued their regular activities." Comparison of relevance to this review = (2) vs (3)
Outcomes	**Independence in ADL scales:** NR **Motor function scales:** NR **Measures of balance:** Berg Balance Scale, functional reach (measured at baseline only) **Measures of gait velocity:** Timed Up and Go test (measured at baseline only) **Length of stay:** NR **Adverse events:** falls or injury **Other measures:** cognitive ability, centre of pressure measures **Time points when outcomes were assessed:** after intervention, follow‐up
Comparison	**Amount of physical rehabilitation in groups:** differs **Categorisation of comparison:** 1. Physical rehabilitation versus no or limited physical rehabilitation (dose differs) (limited = < 50% of dose)
Funding & conflicts of interest	**Funding statement:** not reported **Conflict of interest statement:** "None"
Synthesis	**Qualitative synthesis:** no relevant outcomes measured after intervention
Notes	

**Table:** Chen G 2014

***Study characteristics***	
Methods	**Study aim:** To explore the effect of targeted limb task function or strength training on the life function of hemiplegic patients with cerebral infarction in the early stage **Design:** parallel‐group RCT **Country:** China **Study recruitment and setting:** see Table **Involvement of patients/public/professionals/others:** no
Participants	**Study population (number randomised):** 80 **Inclusion criteria:** No previous history of cerebral infarction (i.e. first stroke) confirmed by head CT or MRI Onset within 24 h to 1 week Hemiplegia as a result of the stroke Conscious during hospitalisation Able to co‐operate with nursing intervention **Exclusion criteria:** Not reported **Participants:** detailed in Table **PROGRESS‐plus:** summarised in Table
Interventions	(1) Task‐specific training "mainly focused on functional training in targeted limb training" "The specific task function training emphasizes the systematic and repetitive practice of the task within the feasible range. The tasks are customizable and are designed to train the extension or grasping activities of the peripheral appendage rehabilitation mode. All tasks are designed to be standardized, repeatable, and contain functional goals (eg, pointing, grasping, stirring). In the practice process, the daily affairs that can be done can be arranged arbitrarily to promote learning and simulate the activities in real life [3]. In the training, grasp the soft ball to imitate the action of eating bananas and count the number of independent actions. Active and passive exercise durations are determined by the patient's recovery, starting with passive (assisted) functional exercises and progressing from semi‐assisted to active as ability increases. All exercises are done in front of the mirror, the purpose is to make the patient continuously adjust his movements through visual feedback to ensure the quality of the movements [5]. Except for routine training, there was no overlap in the type of training (strength or function) between the two groups during targeted limb training. During the training, the nurses closely observe to avoid excessive fatigue, and ensure that there is no fatigue symptoms and other discomfort during the training the next day." (2) Strength training group "focused on strength training during targeted limb training" "Mainly use resistance exercise to improve muscle strength. When the muscle strength of the hemiplegic limb of the patient is zero, the patient is instructed to consciously instruct the hemiplegic limb to exert force in a certain direction, and the nurse supports the hemiplegic limb with both hands to give strength support along the direction of the patient's consciousness. Generally 2d or 3d, the patient's voluntary movement can be seen, and the muscle strength reaches grade 1 or 2. Then, on this basis, a certain resistance is given, that is, resistance motion [4]. The training intensity was 1 time a day, 40 min each time, 5 times a week. During the training process, the corresponding treatment method should be selected according to the patient's condition, and attention should be paid to avoid inducing increased muscle tension." "The rest of the early physical activity training was the same". This included positioning, passive movement, sitting up and sitting balance training, standing balance training, walking training, daily life ability training.
Outcomes	**Independence in ADL scales:** Barthel Index **Motor function scales:** Fugl‐Meyer motor function scale **Measures of balance:** NR **Measures of gait velocity:** NR **Length of stay:** NR **Adverse events:** NR **Other measures:** NR **Time points when outcomes were assessed:** admission and months 3 and 6
Comparison	**Amount of physical rehabilitation in groups:** equivalent **Categorisation of comparison:** 3 (ii) Comparison of different approaches, (dose equivalent; with both/all groups also receiving other physical rehab)
Funding & conflicts of interest	**Funding statement:** not reported **Conflict of interest statement:** not reported
Synthesis	**Quantitative synthesis:**Analysis 7.1; Analysis 7.2; Analysis 8.1; Analysis 8.2
Notes	This paper was published in Chinese and translated using Google Translate.

**Table:** Cheng 2021

***Study characteristics***	
Methods	**Study aim:** to investigate the effect of standardised three‐level rehabilitation treatment on motor dysfunction recovery in ischaemic stroke patients **Design:** parallel‐group RCT **Country:** China **Study recruitment and setting:** see Table **Involvement of patients/public/professionals/others:** no
Participants	**Study population (number randomised):** 156 **Inclusion criteria:** Stable vital signs in first week following ischaemic stroke GCS > 8 Limb motor dysfunction Aged 40 to 80 years old Informed consent **Exclusion criteria:** Intellectual impairment or language communication impairment and not able to co‐operate with rehabilitation treatment Malignant tumour Ischaemic stroke duration > 15 days Previous history of stroke Unable to participate in the research study **Participants:** detailed in Table **PROGRESS‐plus:** summarised in Table
Interventions	(1) Three‐stage rehabilitation "(1) Level‐one rehabilitation therapy. Patients with ischemic stroke are mainly hospitalized in the neurology ward within 1 month of onset, and the patient's condition has improved after routine symptomatic and supportive treatment. After the patient is stabilised, physical therapy (PT) is given at the bedside in the ward in the early stage. (2) Secondary rehabilitation treatment. Patients with ischemic stroke from the beginning of the second month to the end of the third month, according to the patient's condition and motor dysfunction, continue rehabilitation treatment in the rehabilitation department ward or rehabilitation department outpatient clinic. (3) Tertiary rehabilitation treatment. From the beginning of the fourth month to the end of the sixth month, patients with ischemic stroke should continue to perform necessary rehabilitation training at home or in community health service centers under the guidance of therapists or door‐to‐door training until the end of follow‐up. The above rehabilitation treatment is a combination of PT and occupational therapy (OT), that is, according to the Brunnstrom classification, PT is mainly used in the early stage of the patient's disease, and OT is gradually replaced after the patient's disease is stable. Rehabilitation therapists teach patients’ families some methods to assist patients in rehabilitation training when performing primary and secondary treatments." (2) No rehabilitation "The control group was given conventional symptomatic and supportive treatment"
Outcomes	**Independence in ADL scales:** NR **Motor function scales:** Fugl Meyer assessment **Measures of balance:** NR **Measures of gait velocity:** NR **Length of stay:** NR **Adverse events:** NR **Other measures:** NR **Time points when outcomes were assessed:** admission, month 3 and 6
Comparison	**Amount of physical rehabilitation in groups:** differs **Categorisation of comparison:** 1. Physical rehabilitation versus no or limited physical rehabilitation (dose differs) (limited = < 50% of dose)
Funding & conflicts of interest	**Funding statement:** Guangdong Provincial Science and Technology Program (2017A020213027); Guangzhou Famous Traditional Chinese Medicine Studio Construction Project (ky01050002) **Conflict of interest statement:** not reported
Synthesis	**Quantitative synthesis:**Analysis 1.2
Notes	

**Table:** Chen J 2014

***Study characteristics***	
Methods	**Study aim:** to explore the clinical effect of community‐based tertiary rehabilitation therapy on stroke sequelae **Design:** parallel‐group RCT **Country:** China **Study recruitment and setting:** see Table **Involvement of patients/public/professionals/others:** no
Participants	**Study population (number randomised):** 80 **Inclusion criteria:** Diagnosis meets the stroke‐related diagnostic criteria formulated by the Fourth National Cerebrovascular Academic Conference and was diagnosed with stroke by cranial CT or MRI examination Duration of the patient's disease is ≤ 1.5 years Medically stable GCS > 8 Limb motor dysfunction or cognitive impairment No malignant tumour No serious cardiopulmonary, liver, and kidney insufficiency No history of dementia and mental illness, and can be followed up at any time Informed consent **Exclusion criteria:** Not reported **Participants:** detailed in Table **PROGRESS‐plus:** summarised in Table
Interventions	(1) Three‐stage rehabilitation "the patients in the observation group were treated with community‐based three‐level rehabilitation therapy. The treatment methods were as follows: 1. First‐level rehabilitation therapy: During the hospitalization period, the rehabilitation physicians began to conduct one‐to‐one rehabilitation training for the patients. The training lasts for 1 to 1.5 months, mainly to teach the patient's family members how to carry out auxiliary training for them. Patients were assisted by their family members to carry out training 1 to 2 times/d. 2. Secondary rehabilitation treatment: The patients received rehabilitation training for 1.5 to 2 months in the community rehabilitation station, the number of training sessions per week was ≥ 2 times, and each training session was 40 minutes. 3. Third‐level rehabilitation treatment: home rehabilitation training. Patients should train once a day, and the training time is 40min/time. 4. Rehabilitation teachers evaluate the patient's training effect every month and provide relevant training guidance. 5. The contents of rehabilitation training include: posture placement, rollover training, self‐assistance training in bed, active limb movement, passive limb movement, sitting training, breathing training, balance control training, etc" (2) Conventional rehabilitation "The patients in the control group were treated with conventional medical therapy and community‐level rehabilitation therapy. " Note: it is unclear whether the conventional rehabilitation group received "first level" rehabilitation therapy during the hospital phase. We have assumed that they have, since the intervention of interest is described as being "community‐based".
Outcomes	**Independence in ADL scales:** modified Barthel Index **Motor function scales:** Fugl‐Meyer Assessment **Measures of balance:** NR **Measures of gait velocity:** NR **Length of stay:** NR **Adverse events:** NR **Other measures:** National Institute of Health Stroke Scale (NIHSS) **Time points when outcomes were assessed:** before treatment, 1 month, and 6 months after treatment
Comparison	**Amount of physical rehabilitation in groups:** differs **Categorisation of comparison:** 1. Physical rehabilitation versus no or limited physical rehabilitation (dose differs) (limited = < 50% of dose)
Funding & conflicts of interest	**Funding statement:** not reported **Conflict of interest statement:** not reported
Synthesis	**Quantitative synthesis:**Analysis 1.1; Analysis 1.2
Notes	This paper was published in Chinese and translated using Google Translate.

**Table:** Chen L 2019

***Study characteristics***	
Methods	**Study aim:** not reported **Design:** parallel‐group RCT **Country:** China **Study recruitment and setting:** see Table **Involvement of patients/public/professionals/others:** no
Participants	**Study population (number randomised):** 488 **Inclusion criteria:** First‐ever acute cerebral infarction stroke diagnosed using CT or MRI Aged 18 years + Medically stable (i.e. temperature, pulse, respiration, BP) Able to give signed consent **Exclusion criteria:** Cognitive and/or communication disorders which may not allow completion of outcome measures Concurrent diagnosis of PD or terminal illness Administered thrombolysis therapy Diagnosed with low mood prior to the stroke History of schizophrenia, depression, anxiety, or other mental health disorder Unable to give informed consent **Participants:** detailed in Table **PROGRESS‐plus:** summarised in Table
Interventions	(1) Motor relearning programme (2) Bobath approach "The 2 physiotherapy programs were standardized as follows; a manual describing the main philosophy behind the 2 physiotherapy methods was produced according to back‐ground literature. These treatments were discussed among the physiotherapists and the project leader in order to coordinate and as far as possible identify treatment variables in a “Bobath respectively Motor Relearning Program manner” and as described in the manuals true to the background literature. The patients included in the study were given physiotherapy 5 days weekly with a minimum of 40 minutes duration for a period of 4 weeks."
Outcomes	**Independence in ADL scales:** NR **Motor function scales:** NR **Measures of balance:** NR **Measures of gait velocity:** NR **Length of stay:** NR **Adverse events:** NR **Other measures:** Apathy Evaluation Scale **Time points when outcomes were assessed:** NR
Comparison	**Amount of physical rehabilitation in groups:** equivalent **Categorisation of comparison:** 3. (i) Comparison of different approaches (dose equivalent; with no other physical rehab)
Funding & conflicts of interest	**Funding statement:** "supported by grants from the Natural Science Foundation of China [Nos. 81871104, 81100243, and 81502181], Liaoning Science and Technology Plan Projects [No. 2011225020], the Natural Science Foundation of Liaoning Province in China [No. L2013296], Shenyang Science and Technology Plan Projects [17‐230‐9‐17], and the Key Project for Planned Projects of Liaoning Province in 2017 [No. 2017225027]" **Conflict of interest statement:** "Not reported"
Synthesis	**Qualitative synthesis:** no relevant outcomes assessed
Notes	The focus of this study is on prevention of post stroke apathy.

**Table:** Chen P 2014

***Study characteristics***	
Methods	**Study aim:** to observe the efficacy of functional exercise training in the rehabilitation of lower limb muscle strength in stroke patients with hemiplegia **Design:** parallel‐group RCT **Country:** China **Study recruitment and setting:** see Table **Involvement of patients/public/professionals/others:** no
Participants	**Study population (number randomised):** 100 **Inclusion criteria:** Met the diagnostic criteria for stroke formulated by the Fourth National Cerebrovascular Disease Conference of the Chinese Medical Association and confirmed by head CT or MRI Medically stable Disease duration 15 days to 3 months No severe cognitive function Hemiplegia on one side of the limb Muscle strength of each key muscle group of the hemiplegic lower extremity < grade V with muscle tension of each key muscle of the hemiplegic lower extremity < grade 2 based on MAS **Exclusion criteria:** Severe comorbidities (acute heart failure, upper gastrointestinal bleeding, respiratory failure, liver and kidney failure, severe pulmonary infection, etc.) Parkinson's syndrome Previous lower extremity peripheral nerve injury **Participants:** detailed in Table **PROGRESS‐plus:** summarised in Table
Interventions	(1) Intervention group ‐ functional exercise training "on the basis of basic treatment, the treatment group adopted functional exercise training to enhance the muscle strength of the lower limbs, and comprehensively applied PNF technology. Theraband technology, squatting training and other technologies, using the balance and torso strength training system, hand training system, underwater training" "PNF is the use of stretch, proprioceptive stimulation such as joint compression and traction, application of resistance ... apply a spiral‐diagonal movement to promote activity" "The practice is similar to the daily sit‐up movement, by squatting and standing training strengthens the quadricepts and improves knee control ability" (2) Control group "antispasmodic training for the lower limbs" "on the basis of basic treatment, exercise control on key muscle groups of lower limbs training, anti‐spasmodic patterns such as Rood Technique, Bobath technique" "Routine rehabilitation is given according to the patient's functional status, that is bed turn over, transfer, balance, sit, stand, walk, activities of daily living and other training to improve limb motor function and daily life function"
Outcomes	**Independence in ADL scales:** Barthel Index **Motor function scales:** Fugl‐Meyer **Measures of balance:** NR **Measures of gait velocity:** NR **Length of stay:** NR **Adverse events:** NR **Other measures:** muscle strength **Time points when outcomes were assessed:** after treatment
Comparison	**Amount of physical rehabilitation in groups:** equivalent **Categorisation of comparison:** 3 (ii) Comparison of different approaches, (dose equivalent; with both/all groups also receiving other physical rehab)
Funding & conflicts of interest	**Funding statement:** Fund of Science and Information Bureau of Panyu District, Guangzhou (No. 2012‐Z‐03‐25) **Conflict of interest statement:** not reported
Synthesis	**Quantitative synthesis:**Analysis 7.1; Analysis 7.2
Notes	Article published in Chinese and translated using Google Translate.

**Table:** Chen S 2021

***Study characteristics***	
Methods	**Study aim:** to evaluate the effectiveness of advanced practice nurse guided home‐based rehabilitation exercise programme (HREPro) in people with post‐stroke lower limb spasticity **Design:** parallel‐group RCT **Country:** China **Study recruitment and setting:**Table **Involvement of patients/public/professionals/others:** no
Participants	**Study population (number randomised):** 140 **Inclusion criteria:** Clinically diagnosed with stroke and lower limb spasticity Able to comprehend and follow instructions **Exclusion criteria:** Cognitive impairment with MMSE < 18 Pre‐existing history of anxiety or depression as measured by HADS **Participants:** detailed in Table **PROGRESS‐plus:** summarised in Table
Interventions	(1) Home‐based rehabilitation exercise programme (HREPro) "Patients in the intervention group were provided with an HREPro in addition to the conventional rehabilitation regimen. The HREPro was an individually tailored, year‐long rehabilitation intervention programme conducted at the participants' homes by an ARPN who had received professional physiotherapy training. The HREPro consisted of familiarizing the patient with navigating their home environment, as well as psychological preparation, and an exercise component. A nurse, during their first visit, evaluated the home environment, modified any environmental hazards, and guided the patient regarding how to walk safely in their home. The program involved fostering meaningful communication between the nurse and the patient. This helped the nurse understand each patient's specific concerns, including the fear of falling and satisfaction with particular walking aids. This, in turn, enabled them to motivate the patient on the basis of their own self‐sufficiency. It was hoped that this would improve the patient's confidence in their own recovery and also help optimize program adherence. A comprehensive exercise program was used, which mainly included strengthening the lower limb muscle groups with exercises, such as joint training, sit‐ups, balance training while standing, standing, bending to pick things up, straight leg‐lifting, and climbing stairs. The exercise regimen was formulated with a view to reducing lower limb spasticity and improving mobility after stroke. Patients underwent three exercise sessions per week during the first 3 months, supervised by an APRN, with each session lasting 30 minutes. This was followed by one supervised session per week during the next 3 months. Thereafter, the frequency of the supervised exercise dropped to once a month, and once every other month, up to 12 months. HREPro was guided by the APRN with the assistance of family members who participated in all training sessions. If the patient encountered any difficulty during the whole training period, they could communicate with the nurse via email or phone at any time." (2) Control "Patients in the CG were provided conventional rehabilitation after discharge from the hospital. This included issuing a rehabilitation manual for stroke (standard health education, consisting of information on physical activity, drugs, diet, follow‐up time, etc.), performing telephonic follow‐up (within 1 month), and completing follow‐up medical appointments for assessment of recovery at 3, 6, and 12 months"
Outcomes	**Independence in ADL scales:** Barthel Index **Motor function scales:** Fugl‐Meyer Assessment **Measures of balance:** NR **Measures of gait velocity:** 10‐m walk test **Length of stay:** NR **Adverse events:** NR **Other measures:** Modified Ashworth scale **Time points when outcomes were assessed:** 3, 6, and 12 months after initiation of 12‐month intervention
Comparison	**Amount of physical rehabilitation in groups:** differs **Categorisation of comparison:** 1. Physical rehabilitation versus no or limited physical rehabilitation (dose differs) (limited = < 50% of dose)
Funding & conflicts of interest	**Funding statement:** supported by Wenzhou Science and Technology Bureau (Y20180385) **Conflict of interest statement:** none
Synthesis	**Quantitative synthesis:**Analysis 1.1; Analysis 1.2; Analysis 1.4
Notes	

**Table:** Chen Y 2011

***Study characteristics***	
Methods	**Study aim:** to investigate the effect of regular visits to the hospital for individualised rehabilitation training guidance on the rehabilitation effect of stroke‐induced limb dysfunction **Design:** parallel‐group RCT **Country:** China **Study recruitment and setting:** see Table **Involvement of patients/public/professionals/others:** no
Participants	**Study population (number randomised):** 66 **Inclusion criteria:** Hemiplegia Confirmed stroke on MRI that meets the Fourth National Cerebrovascular Diseases diagnostic criteria Within 14 to 30 days of stroke onset **Exclusion criteria:** Other causes of dystonia and previous motor dysfunction Severe hypertension Cardiovascular heart disease Severe diabetes patients **Participants:** detailed in Table **PROGRESS‐plus:** summarised in Table
Interventions	(1) Rehabilitation The intervention group followed the Brunnstrom 6‐stage assessment period. Participants were evaluated according to these stages and given the corresponding rehabilitation guidance: *Stage I and II:* keep the limbs in a good position when lying down and massage the limbs first when moving. Passive activities of the lower limbs included: flexion and extension of the hip joints, knee joints, ankle joints and toe joints. Passive movement of upper limbs: included shoulder joint, internal and external rotation, internal and external abduction, straight arm flexion and extension, elbow flexion and extension, wrist and finger joint activities and dorsiflexion. Four sessions/day with each session lasting 30 mins. *Stage III and IV:* the upper and lower extremity joints and muscles are moved using passive activities, followed by active activities with assistance. Lower limbs perform active movements in the normal movement mode, and the upper limbs assist the patient in adduction and abduction of the shoulder joint, flexion and extension of the elbow joint, pronation and supination of the forearm, wrist joint movement. Pronation, supination, flexion and extension of fingers and fine function training of fingers. After each activity, the stroke survivor should sit up on the bed for 15 minutes and then gradually transition to sitting on the side of the bed, stepping on the ground with both feet, and then standing, and gradually increase the muscle strength of the affected limb by alternating legs until they can bear their whole‐body weight. Four sessions/day with each session lasting 15 mins. *Stage V and VI*: stroke survivors are asked to practice walking and do what they can. This includes activities such as brushing teeth, eating with a spoon, combing hair and wiping face, walking by themselves in the ward. Four to six sessions/day with each session lasting 20 mins. (2) No physical rehabilitation Conventional medical follow‐up only. They were also routinely followed post‐discharge.
Outcomes	**Independence in ADL scales:** Barthel Index **Motor function scales:** NR **Measures of balance:** NR **Measures of gait velocity:** NR **Length of stay:** NR **Adverse events:** NR **Other measures:** NR **Time points when outcomes were assessed:** before and after intervention
Comparison	**Amount of physical rehabilitation in groups:** differs **Categorisation of comparison:** 1. Physical rehabilitation versus no or limited physical rehabilitation (dose differs) (limited = < 50% of dose)
Funding & conflicts of interest	**Funding statement:** not reported **Conflict of interest statement:** not reported
Synthesis	**Qualitative synthesis:** Results presented in a format not suitable for quantitative analysis.
Notes	This study was published in Chinese and relevant sections translated into English by our author team (PLC).

**Table:** Choi JU 2015

***Study characteristics***	
Methods	**Study aim:** to determine whether a task‐oriented training programme improved balance, ADL performance, and self‐efficacy in stroke patients **Design:** parallel‐group RCT **Country:** Korea, Rep. **Study recruitment and setting:** see Table **Involvement of patients/public/professionals/others:** no
Participants	**Study population (number randomised):** 20 **Inclusion criteria:** Diagnosis of hemiplegia due to stroke Morbidity period of 6+ months MMSE (Korean version): > 25 MAS Stage 2 or lower Able to understand study aims and follow instructions, and give consent **Exclusion criteria:** Not reported **Participants:** detailed in Table **PROGRESS‐plus:** summarised in Table
Interventions	(1) Task‐oriented training programme "The Canadian Occupational Performance Measure (COPM) was used in the task selection. The COPM uses a 10‐point scale to rate the importance of individual tasks. Priority is placed on five tasks, with the greatest importance placed on the patient’s evaluation of the outcomes of the training program. Following an interview with each subject by the researcher, five tasks were selected: indoor walking, outdoor walking, staircase climbing, wearing clothes, and picking up objects. The selected tasks comprised four stages. If the patient was unable to complete the tasks in one week, they were repeated the following week. The participants practiced the tasks five times a week for 4 weeks. In each 30‐min session, the tasks were performed three times for 10 mins, with a 2‐min break between each task." (2) Control group "received general physical therapy, including exercises aimed at improving gait ability and balance, for 4 weeks, five times a week, with each session lasting 30min"
Outcomes	**Independence in ADL scales:** Modified Barthel Index **Motor function scales:** NR **Measures of balance:** Berg Balance Scale **Measures of gait velocity:** NR **Length of stay:** NR **Adverse events:** NR **Other measures:** self‐efficacy scale **Time points when outcomes were assessed:** after training
Comparison	**Amount of physical rehabilitation in groups:** equivalent **Categorisation of comparison:** 3. (i) Comparison of different approaches (dose equivalent; with no other physical rehab)
Funding & conflicts of interest	**Funding statement:** not reported **Conflict of interest statement:** not reported
Synthesis	**Quantitative synthesis:**Analysis 7.1; Analysis 7.3
Notes	

**Table:** Choi YK 2013

***Study characteristics***	
Methods	**Study aim:** compares the effects of taping on the articular angle of the knee joint and on the functioning in people with hemiplegia as a result of stroke **Design:** parallel‐group RCT **Country:** Korea, Rep. **Study recruitment and setting:** see Table **Involvement of patients/public/professionals/others:** no
Participants	**Study population (number randomised):** 30 **Inclusion criteria:** No visual field defects No vestibular deficits post‐stroke No hearing aids and able to understand instructions and able to give consent **Exclusion criteria:** Artificial joint in a lower extremity Previous fracture Not adopting the proper position when PNF combination patterns were applied Unique skin disease **Participants:** detailed in Table **PROGRESS‐plus:** summarised in Table
Interventions	(1) PNF plus kinesiotape Three 30‐minute PNF treatments per week for 4 weeks, a total of 12 times Elastic Kinesio tape was attached: 1) Patella inferior gliding taping, 2) Patella medial gliding taping, 3) Quadriceps femoris muscle taping. "The combination patterns of PNF treatment were described by Dietz. In these patterns, movement of flexion‐adduction‐external rotation of the paretic upper extremities and simultaneous flexion‐adduction‐external rotation of the non‐paretic lower extremities are performed. In the present study, extension‐abduction‐internal rotation of the non‐paretic upper extremities and simultaneous extension‐abduction‐internal rotation of the paretic lower extremities were also performed. All these integrally performed movements are called PNF combination patterns. The subjects performed these patterns independently, but when they could not do so, the therapist aided the movement of the paretic upper extremities and encouraged the movement of the non‐paretic parts with verbal instruction. In this study, PNF combination patterns were conducted with subjects in a side‐lying position, half‐standing position, and modified plantigrade position. During the experiment, subjects’ trunk alignment condition and pattern movement were checked continuously, and verbal encouragement was given to the subjects to keep them in the proper position. This intervention was conducted 10 times for 10 seconds after pattern alignment was completed in each position, and 10 seconds was allowed for an interval break time. About 10 minutes was spent performing the patterns in each position." (2) Neurodevelopmental treatment (no description)
Outcomes	**Independence in ADL scales:** NR **Motor function scales:** NR **Measures of balance:** Berg Balance Scale **Measures of gait velocity:** 10‐m walk test **Length of stay:** NR **Adverse events:** NR **Other measures:** joint ROM **Time points when outcomes were assessed:** NR
Comparison	**Amount of physical rehabilitation in groups:** equivalent (assumed to be equivalent; no information provided on neurodevelopmental treatment) **Categorisation of comparison:** 3.(i) Comparison of different approaches (dose equivalent; with no other physical rehab)
Funding & conflicts of interest	**Funding statement:** not reported **Conflict of interest statement:** not reported
Synthesis	**Quantitative synthesis:**Analysis 7.9; Analysis 7.10
Notes	

**Table:** Chu 2003

***Study characteristics***	
Methods	**Study aim:** "to determine the effectiveness of early facilitation techniques on motor function of stroke" **Design:** parallel‐group RCT **Country:** China **Study recruitment and setting:** see Table **Involvement of patients/public/professionals/others:** no
Participants	**Study population (number randomised):** 58 **Inclusion criteria:** Met the diagnosis criteria instituted on the 4th National Cerebrovascular Disease Conference in 1995 Stroke confirmed by CT and MRI No cognitive disorder **Exclusion criteria:** Not reported **Participants:** detailed in Table **PROGRESS‐plus:** summarised in Table
Interventions	(1) Rehabilitation group (n = 30) "Rehabilitation group received unobstruction exercises after receiving first evaluation. Unobstruction techniques: (a) position of limbs, (b) active and passive exercises of joint of upper and lower limbs including flexion, extension, internal rotation, abduction; actions in each direction repeated 10 to 20 times, (c) bridge movement on bed: patient lay on their back, flexed knee and hip, clamped a small pillow between knees, played breech elevation and hip extension and repeated these actions, (d) wiping or striking suffered limbs up to down with brush or little hammer in order to promote recovery of sensory, (e) transferring and balance exercises Exercise of sitting balance ability: patient's healthy hand was bound on the tail of bed by a special belt. Head of bed was elevated to 30 degrees. Nurse put patient’s upper limb on trunk, pushed healthy upper limb. This method could enhance strength of extensor muscle of upper limbs and trunk muscle. We increased sitting angle everyday, elongated sitting time and control sitting balance. Exercises from sitting to standing position: nurse stood in front of patient, patient embrace nurse’s waist with healthy hand. Nurse fixed patient’s affected hand on waist. The other hand was put in the subaxillary position of the affected side, which delivered sufficient weight loading to affected side. When patients stood up, nurse called patient's attention to look forward to the direction of nurse’s face. Exercise of standing balance: patients received bedside bridge movement and exercise of lower limb swing in order to practice standing balance ability. Exercise of plain walking: patients walked with supporter or step at first, then feet moved by turn. Nurse also could bind suffered foot and help elevation if needed. Before walking, the lower part of affected limb was hanged in front of chest in order to keep functional position of upper limb and shoulder joint. We should try to avoid tiredness and try to ensure exercise quality. Exercise of going upstairs and downstairs: according to the rule of 'healthy leg up first, affected leg down first,' training flexor and extensor muscles and joints of lower limbs. Exercise of functions of upper limb and hand: Bobath shaking hands, suffered side upper limb takes assisted active movement with help of healthy side upper limb; inducing upper limb muscle movement and hand performance exercise; patients took flexion and extension of shoulder, elbow, twist and finger joint, grasp, hold and pinch movement. Exercises were performed from easy to difficult, from tough to delicate" Length of intervention period: varied from "20 days to 14 months with a mean rehabilitation time of 41.3 days" Number of sessions and length of individual sessions: "above exercises were taken 1 per day, 40 to 60 min/time" Intervention provider: nurses (2) No physical rehabilitation (n = 28) "Two group received routine neurologic treatment and nursing. Control group didn't receive rehabilitation exercise" The individual components delivered are listed in Table 28. Based on the individual components, this intervention is categorised as no intervention. Length of intervention period: no intervention Number of sessions and length of individual sessions: no intervention Intervention provider: no intervention
Outcomes	**Independence in ADL scales:** Barthel Index **Motor function scales:** Fugl‐Meyer Assessment **Measures of balance:** NR **Measures of gait velocity:** NR **Length of stay:** NR **Adverse events:** NR **Other measures:** NR **Time points when outcomes were assessed:** "was taken when patients’ signs and symptoms of nerve system were stable. The same doctor evaluated patients again when treatment was over"
Comparison	**Amount of physical rehabilitation in groups:** differs **Categorisation of comparison:** 1. Physical rehabilitation versus no or limited physical rehabilitation (dose differs) (limited = < 50% of dose)
Funding & conflicts of interest	**Funding statement:** not reported **Conflict of interest statement:** not reported
Synthesis	**Quantitative synthesis:**Analysis 1.1; Analysis 1.2
Notes	Short paper only with limited detail regarding randomisation. This paper was written in English, and the extracts above are direct quotes.

**Table:** Cooke 2006

***Study characteristics***	
Methods	**Study aim:** to determine whether adding functional strength training to conventional physical therapy improves muscle function, gait and functional ability compared to conventional physical therapy or conventional therapy plus neurofacilitation **Design:** multi‐arm RCT (3 arms) **Country:** UK **Study recruitment and setting:** see Table **Involvement of patients/public/professionals/others:** no
Participants	**Study population (number randomised):** 109 **Inclusion criteria:** Inpatients 18 years+ 1 to 13 weeks after anterior circulatory stroke (haemorrhage or infarction) Some voluntary muscle contraction in the paretic lower limb (a score of at least 28/100 on the lower limb section of the motricity index) Potential for clinically important improvement Able to follow a 1‐stage command Independently mobile (with or without aids) Prior to the index stroke No orthopaedic surgery No trauma affecting the lower limb in the last 8 weeks No previous history of other neurological disease **Exclusion criteria:** Not reported **Participants:** detailed in Table **PROGRESS‐plus:** summarised in Table
Interventions	Note: "All participants received routine CPT from their clinical physiotherapists using a treatment schedule." This is the conventional CPT described for group (3). (1) Additional conventional therapy (CPT + CPT) group (n = 35) "Focused on those interventions in the treatment schedule that emphasized control/quality of movement and gave prominence to sensory stimulation and preparation of joint and muscle alignment prior to activating muscle or a functional task. Additional CPT was therefore strongly therapist hands‐on, with provision of passive movements, active assisted exercise, and/or hands‐on intervention to facilitate muscle activity or functional ability. Some active exercise and repetitive practice of functional tasks [were] included but without systematic progression in resistance or repetition" Length of intervention period: 6 weeks Number of sessions and length of individual sessions: standardised treatment schedules for up to 1 hour, 4 days a week for 6 weeks (total = 24 hours) (in addition to the convention CPT) Intervention provider: research physiotherapists (independent of the clinical team) (2) Functional strength training (FST + CPT) group (n = 36) "Delivery of FST directed participants’ attention to the exercise/activity being performed, appropriate verbal feedback on performance, and repetition (therapist hands‐off). Content of FST focused on repetitive, progressive resistive exercise during goal‐directed functional activity. The emphasis was on producing appropriate muscle force for the functional activity being practiced. Treatment progressed systematically using repetition and increase in resistance by, for example, changing the limb’s relationship to gravity, increasing the range of movement or distance over which bodyweight was transported, and changing the weight of external objects used to provide resistance. Treatment activities progressed systematically from light to heavy loads and from few to many repetitions. Participants performed repetitive exercise of functional tasks such as sit‐to‐stand‐to‐sit, stair climbing/step ups, inside and outside walking, transfer training, bed mobility, and treadmill training with and without the use of a bodyweight support system" Length of intervention period: 6 weeks Number of sessions and length of individual sessions: standardised treatment schedules for up to 1 hour, 4 days a week for 6 weeks (total = 24 hours) Intervention provider: research physiotherapists (independent of the clinical team) (3) Conventional physiotherapy (CPT) group (n = 38) "Routine CPT included soft tissue mobilization, facilitation of muscle activity, facilitation of coordinated multijoint movement, tactile and proprioceptive input, resistive exercise, and functional retraining" Length of intervention period: 6 weeks Number of sessions and length of individual sessions: "duration of each session was recorded" Intervention provider: clinical physiotherapists Comparisons relevant to this review: a. Group (1) versus (2) b. Group (2) versus (3)
Outcomes	**Independence in ADL scales:** NR **Motor function scales:** modified Rivermead Motor Assessment **Measures of balance:** NR **Measures of gait velocity:** 10‐Metre Walk test **Length of stay:** NR **Adverse events:** NR **Other measures:** ability to walk at 0.8 m/s or more, temporal‐spatial gait parameters (symmetry of step length and step time), torque around the paretic knee during concentric isokinetic extension, followed immediately by flexion (using the CYBEX NORM isokinetic dynamometer), EuroQol **Time points when outcomes were assessed:** "Participants provided written informed consent and then completed baseline measures. Intervention began on the following day for 6 weeks. Participants repeated the measurement battery on completion of the intervention phase (outcome) and 12 weeks later (follow‐up)"
Comparison	**Comparison a: Group (1) versus Group (2)** **Amount of physical rehabilitation in groups:** equivalent **Categorisation of comparison:** 4. Comparison of similar approaches (dose equivalent) **Comparison b: Group (2) versus Group (3)** **Amount of physical rehabilitation in groups:** differs **Categorisation of comparison:** 5. Additional therapy + usual therapy versus usual therapy (dose differs)
Funding & conflicts of interest	**Funding statement:** "Funding was provided by the Healthcare Foundation and the Dowager Countess Eleanor Peel Trust" **Conflict of interest statement:** "no conflicts of interest to declare with regard to this article"
Synthesis	**Qualitative synthesis:** Group (1) vs Group (2): these two treatment groups were classified as including similar treatment components, and data from this study have not been included within the comparisons of one active intervention versus another active intervention **Quantitative synthesis:** Group (2) vs Group (3): Analysis 2.2; Analysis 2.4; Analysis 5.2; Analysis 5.4
Notes	

**Table:** Dai 2015

***Study characteristics***	
Methods	**Study aim:** to evaluate the impact of acupuncture and rehabilitation training on the motor function and daily life of stroke patients **Design:** parallel‐group RCT **Country:** China **Study recruitment and setting:** see Table **Involvement of patients/public/professionals/others:** no
Participants	**Study population (number randomised):** 62 **Inclusion criteria:** Diagnosed in line with the diagnostic criteria stipulated by the Fourth National Cerebrovascular Disease Academic Conference, with a clinical diagnosis plus CT and/or MRI examinations **Exclusion criteria:** Not reported **Participants:** detailed in Table **PROGRESS‐plus:** summarised in Table
Interventions	(1) Rehabilitation + acupuncture "Rehabilitation training methods include: Rehabilitation training in good limb position, which is a temporary training position designed from the perspective of treatment, elbow, shoulder, knee, ankle and other joint functions. Rehabilitation training in bed: After the vital signs are stable, passive exercise is the main method, and active exercise is supplemented. Training upper and lower limb muscle strength and weight‐bearing function; rehabilitation training while standing, assisting patients to stand; under the protection of the operator, do balance training to the left and right of the body. Walking period training: This is the most important item in motor function training. The training content is mainly based on the Bobath method, mainly on the training of sitting balance and turning, standing center transfer, striding, walking and daily life ability [2]. With the continuous recovery of the patient's disease and the continuous enhancement of the weight‐bearing capacity, the training is to move from the bed to the chair, enter and exit the bathroom, walk on flat ground, and go up and downstairs. The entire rehabilitation training should not be rushed, but should be carried out gradually, gradually allowing the patient to complete it independently." (2) Acupuncture
Outcomes	**Independence in ADL scales:** Barthel Index **Motor function scales:** Fugl Meyer scale **Measures of balance:** NR **Measures of gait velocity:** NR **Length of stay:** NR **Adverse events:** NR **Other measures:** NR **Time points when outcomes were assessed:** after 6 months of treatment
Comparison	**Amount of physical rehabilitation in groups:** differs **Categorisation of comparison:** 1. Physical rehabilitation versus no or limited physical rehabilitation (dose differs) (limited = < 50% of dose)
Funding & conflicts of interest	**Funding statement:** not reported **Conflict of interest statement:** not reported
Synthesis	**Quantitative synthesis:**Analysis 1.1; Analysis 1.2
Notes	

**Table:** Dalal 2018

***Study characteristics***	
Methods	**Study aim:** to test the efficacy of prowling along with proprioceptive training on reducing the knee hyperextension **Design:** parallel‐group RCT **Country:** India **Study recruitment and setting:** see Table **Involvement of patients/public/professionals/others:** no
Participants	**Study population (number randomised):** 32 **Inclusion criteria:** Medically stable First episode stroke subjects with presence of knee hyperextension MMSE ≥ 23 Able to walk 5 m (with or without support including assistive devices) BRS of affected lower extremity ≥ 3 **Exclusion criteria:** Severe plantar flexor tightness Musculoskeletal, cardiopulmonary, or any neurological deficits (other than stroke) that could influence performance **Participants:** detailed in Table **PROGRESS‐plus:** summarised in Table
Interventions	(1) Experimental ('prowling') In addition to routine physiotherapy: "received prowling along with proprioceptive training as an adjunct which lasted for another 15–20 min. For the affected limb, proprioceptive training consisted of partial squats, single limb stance, and single limb dynamic partial squats in standing on ground as well as foam mat (Fig. 2A–D). Subjects in the experimental group were trained to prowl with bilateral knee flexion with trunk in mild forward flexion (Fig. 2E). Throughout the stance phase of the gait, the participants were instructed to maintain a minimum of 15 ° to amaximum of 45 ° of bilateral knee flexion and avoid hyperextension. When required, the study participants were given manual assistance during exercises. In addition to the above, the participants of the experimental group were also instructed and encouraged to walk with knees bent for routine activities, just the way they were trained during the therapy sessions." (2) Control Routine physiotherapy: both the groups received 6 sessions of treatment of routine physiotherapy which lasted for 45 to 60 min including rest periods
Outcomes	**Independence in ADL scales:** NR **Motor function scales:** NR **Measures of balance:** NR **Measures of gait velocity:** time taken to cover 5 m **Length of stay:** NR **Adverse events:** NR **Other measures:** gait parameters, Wisconsin gait scale **Time points when outcomes were assessed:** pre‐ and post‐intervention
Comparison	**Amount of physical rehabilitation in groups:** differs **Categorisation of comparison:** 5. Additional therapy + usual therapy versus usual therapy (dose differs)
Funding & conflicts of interest	**Funding statement:** not reported **Conflict of interest statement:** none
Synthesis	**Quantitative synthesis:**Analysis 5.4
Notes	Data presented as medians and IQR

**Table:** Danlami 2017

***Study characteristics***	
Methods	**Study aim:** to compare the use of number of repetitions and hours of task practice as measures of dose during lower limb CIMT in patients ≤ 6 months post‐stroke **Design:** multi‐arm RCT (3‐arm) **Country:** Nigeria **Study recruitment and setting:**Table **Involvement of patients/public/professionals/others:** no
Participants	**Study population (number randomised):** 22 **Inclusion criteria:** ≤ 6 months post stroke Lower limb impairment Ability to follow verbal and visual instructions No significant cognitive impairment i.e. MMSE ≥ 17 Low risk of fall (Tinetti gait and balance score ≥ 24) **Exclusion criteria:** Not reported **Participants:** detailed in Table **PROGRESS‐plus:** summarised in Table
Interventions	(1) sCIMT ‐ repetitions during constraint induced movement therapy "The sCIMT group performed sit‐to‐stand, as well as forward and backward stepping, stairclimbing and descending (only the first stair was used), and side‐to‐side stepping with the affected limb, 40 times each per session for 2 sessions a day,5 times a week for 4 weeks. Thus, each participant in the group performed 240 repetitions per session (480 repetitions in 2 sessions)" (2) tCIMT ‐ time spent in constraint induced movement therapy "the tCIMT group practiced the same tasks as the sCIMT group, but for 2 hours a day, 5 times a week for 4 weeks." "In both groups, the unaffected limbs were constrained using a tight knee brace for about 90% of waking hours throughout the study duration." (3) Control ‐ conventional therapy "The control group received usual physical therapy including passive movement, therapeutic positioning, strengthening exercise and over‐ground gait training for 2 hours a day, 5 times a week for 4 weeks. All tasks were initially administered by a physiotherapist, but patients and relatives were taught how to carry out the tasks at home."
Outcomes	**Independence in ADL scales:** NR **Motor function scales:** Fugl‐Meyer (LL motor function subscale) **Measures of balance:** NR **Measures of gait velocity:** NR **Length of stay:** NR **Adverse events:** NR **Other measures:** NR **Time points when outcomes were assessed:** after treatment
Comparison	**Amount of physical rehabilitation in groups:** equivalent **Categorisation of comparison:** Group (1) or (2) vs (3) ‐ 3.(i) Comparison of different approaches (dose equivalent; with no other physical rehab) Group (1) vs (2) ‐ 4. Comparison of similar approaches (dose equivalent)
Funding & conflicts of interest	**Funding statement:** not reported **Conflict of interest statement:** not reported
Synthesis	**Qualitative synthesis:** data presented in paper not suitable for analysis (no standard deviations or confidence intervals/ranges presented).
Notes	

**Table:** Dean 2000

***Study characteristics***	
Methods	**Study aim:** "to evaluate the immediate and retention effects of a 4‐week training program on the performance of locomotor‐related tasks in chronic stroke" **Design:** parallel‐group RCT **Country:** Canada **Study recruitment and setting:**Table **Involvement of patients/public/professionals/others:** no
Participants	**Study population (number randomised):** 12 **Inclusion criteria:** First stroke > 3 months post stroke Discharged from rehabilitation Able to attend rehabilitation centre 3 times a week for 4 weeks Able to walk 10 m independently **Exclusion criteria:** Any medical condition that would prevent participation in a training programme **Participants:** detailed in Table **PROGRESS‐plus:** summarised in Table
Interventions	(1) Motor learning (n = 6) Standardised circuit programme designed to strengthen the muscles in the affected leg in a functionally relevant way and provide for practice of locomotor‐related tasks "For the experimental group, the exercise class was designed as a circuit program, with subjects completing practice at a series of work stations as well as participating in walking races and relays with other members of the group. The workstations were designed to strengthen the muscles in the affected leg in a functionally relevant way and provide for practice of locomotor related tasks. The 10 workstations incorporated into the circuit were: (1) sitting at a table and reaching in different directions for objects located beyond arm’s length to promote loading of the affected leg and activation of affected leg muscles; (2) sit‐to‐stand from various chair heights to strengthen the affected leg extensor muscles and practice this task; (3) stepping forward, backward, and sideways onto blocks of various heights to strengthen the affected leg muscles; (4) heel lifts in standing to strengthen the affected plantarflexor muscles; (5) standing with the base of support constrained, with feet in parallel and tandem conditions reaching for objects, including down to the floor, to improve standing balance; (6) reciprocal leg flexion and extension using the Kinetron in standing to strengthen leg muscles; (7) standing up from a chair, walking a short distance, and returning to the chair to promote a smooth transition between the two tasks; the remaining stations (8) walking on a treadmill; (9) walking over various surfaces and obstacles and (10) walking over slopes and stairs provided the opportunity for practice of walking under variant conditions" Length of intervention period: 4 weeks Number of sessions and length of individual sessions: 1‐hour programme, 3 days/wk Intervention provider: "All training sessions were organized into a group exercise class, conducted by the one of the investigators who was assisted by another physiotherapist" (2) Attention control (n = 6) Standardised circuit programme designed to improve function of the upper limb Length of intervention period: 4 weeks Number of sessions and length of individual sessions: one‐hour programme, three days/wk Intervention provider: "All training sessions were organized into a group exercise class, conducted by the one of the investigators who was assisted by another physiotherapist"
Outcomes	**Independence in ADL scales:** NR **Motor function scales:** NR **Measures of balance:** NR **Measures of gait velocity:** timed 10‐Metre Walk **Length of stay:** NR **Other measures:** Six‐Minute Walk test; Step test; Timed Up and Go test; laboratory gait assessment, strength and dexterity of the upper limb **Time points when outcomes were assessed:** "Subjects were evaluated three times: before the training (pretraining), at the end of the training (posttraining), and 2 months later (follow‐up)"
Comparison	**Amount of physical rehabilitation in groups:** differs (but amount of intervention is equivalent) **Categorisation of comparison:** physical rehabilitation versus attention control (dose of physical rehab differs; dose of intervention equivalent)
Funding & conflicts of interest	**Funding statement:** "Supported by a grant from the ‘‘Fonds de recherche en santé du Québec.’’Dr. Dean was a recipient of a postdoctoral fellowship from the Fonds en réadaptationde l’Université Laval" **Conflict of interest statement:** "No commercial party having a direct financial interest in the results of the researchsupporting this article has or will confer a benefit upon the authors or upon anyorganization with which the authors are associated."
Synthesis	**Quantitative synthesis:**Analysis 3.4; Analysis 4.4
Notes	

**Table:** Dean 2006

***Study characteristics***	
Methods	**Study aim:** "investigate whether an exercise intervention can enhance mobility, prevent falls, and increase physical activity among community‐dwelling people after stroke" **Design:** parallel‐group RCT **Country:** Australia **Study recruitment and setting:** see Table **Involvement of patients/public/professionals/others:** no
Participants	**Study population (number randomised):** 151 **Inclusion criteria:** One or more strokes Able to walk 10 m independently (with or without a mobility aid) Gained medical clearance Willing to join the NSW Stroke Recovery Association Commit to a weekly exercise class and home programme for 12 months Able to give informed consent **Exclusion criteria:** MMSE < 20 Insufficient language skills to participate in assessment and intervention Medical condition precluding exercise (e.g. unstable cardiovascular disease or other uncontrolled chronic conditions) **Participants:** detailed in Table **PROGRESS‐plus:** summarised in Table
Interventions	(1) Task‐specific training (n = 76) The EG received an exercise intervention designed to enhance mobility, prevent falls and increase physical activity—the WEBB programme. The programme involved "task‐related" training with progressive balance and strengthening exercises, as well as walking and stair climbing. Typical exercises included calf raisers while standing, sit‐stand, step‐ups, standing with reduced base of support, graded reaching activities in standing and forward, backward and sideways stepping and walking. The intervention was delivered in a weekly circuit‐style group exercise class and a home exercise programme, and advice to increase walking was given. "Interventions were tailored to the participant’s functional ability. The nature and difficulty of the exercises were progressed regularly to ensure that the intervention remained challenging" Length of intervention period: "Classes planned to be delivered weekly for 40 weeks over a 1‐year period and a home exercise program to be completed at least 3 times per week" Number of sessions and length of individual sessions: "Each class and home program session was designed to take 45 to 60 minutes" Intervention provider: "exercise classes were delivered by a physiotherapist who also designed individual home programs, which were reviewed and modified monthly" (2) Attention control (CG) (n = 75) "The CG exercise class was designed to improve upper‐limb function, manage upper‐limb contracture with task‐related strength and coordination training, and improve cognition with matching, sorting and sequencing tasks. The CG was also prescribed a home program aimed to make them use their affected arm and keep their mind occupied with cognitive leisure tasks such as word and number puzzles" Length of intervention period: "Classes planned to be delivered weekly for 40 weeks over a 1‐year period and a home exercise program to be completed at least 3 times per week" Number of sessions and length of individual sessions: "Each class and home program session was designed to take 45 to 60 minutes" Intervention provider: "Exercise classes were delivered by a physiotherapist who also designed individual home programs, which were reviewed and modified monthly"
Outcomes	**Independence in ADL scales:** Adelaide Activities Profile **Motor function scales:** NR **Measures of balance:** NR **Measures of gait velocity:** 10‐Metre Walk test **Length of stay:** NR **Adverse events**: falls and adverse events during exercise class **Other measures:** Timed Up and Go test, Step test, Timed 5 STS, maximum balance range, Six‐Minute Walk test, knee strength (affected and intact), Health‐Related Quality of Life SF (Short Form)‐12, version two, falls risk (Short‐Form Physiological Profile Assessment), 7‐day pedometer count, choice stepping reaction time, co‐ordinated stability **Time points when outcomes were assessed:** at baseline and at month 12
Comparison	**Amount of physical rehabilitation in groups:** differs **Categorisation of comparison:** physical rehabilitation versus attention control (dose of physical rehab differs; dose of intervention equivalent)
Funding & conflicts of interest	**Funding statement:** "disclosed receipt of the following financial supportfor the research, authorship, and/or publication of this article: Theproject was funded by NSW Health through the NSW Health Promotion Demonstration Research Grants Scheme and conducted by the University of Sydney, Stroke Recovery Associationof NSW, and Health Promotion Service of Sydney South West Area Health Servicel" **Conflict of interest statement:** "no potential conflicts of interest with respect to the research, authorship, and/or publication of this article"
Synthesis	**Quantitative synthesis:**Analysis 3.4; Analysis 3.6
Notes	

**Table:** Deng 2011

***Study characteristics***	
Methods	**Study aim:** "to study the influence of ADL system intervention on the movement function and QoL of stroke patients, and clarify its improvement on the QoL of the patients" **Design:** parallel‐group RCT **Country:** China **Study recruitment and setting:** see Table **Involvement of patients/public/professionals/others:** no
Participants	**Study population (number randomised):** 100 **Inclusion criteria:** First ever stroke Confirmed by CT scan or MRI Satisfied 1995 evaluative standard revised by the 4th [National] Academic Conference of Cerebral Vascular Disease GCS > 8 Persistent motor deficits Participant or family consent Willing to sign on informed consent sheet **Exclusion criteria:** Severe active liver disease Insufficient function of the liver and/or kidney, cardiovascular issues, malignant tumour(s), cardiorespiratory issues Inability to comply with investigators Haemorrhage in the eyes Home inaccessible to visiting personnel **Participants:** detailed in Table **PROGRESS‐plus:** summarised in Table
Interventions	(1) Rehabilitation (n = 50) "Both groups received conventional therapy and nursing care in the Department of Neurology" "The intervention group were also given ADL system intervention. The team looking after the intervention group comprised therapists and rehabilitation nurses. The therapists used Brunnstrom stages of motor recovery to deliver the intervention training, the rehabilitation nurses provided guidance on patient self‐care, based on the assessment of patient self‐care needs and self‐care ability, through 3 care systems, using the methods of full substitution, guidance, encouragement and provision of environment and education to improve the patients’ ADL ability" "ADL system intervention content: (a) Acute phase (flaccid paralysis phase): The aim of training is to prevent disuse, prevent possible complications so as to create the conditions for initial training. Initial bedside activities for ADL training include: maintaining positions which prevent spasms; changing body positions; passive ranging exercises through normal joint ranges of motion; passive massages; active bed training: mainly focused on training of truncal muscles, including bridging exercises, leg exercises, hip exercises, self‐assisted upper limb extensor exercises, shoulder exercises, rolling to both sides, sitting up from non‐affected and affected sides etc. (b) Early recovery phase (spastic phase): The aim of training is to reduce muscle tension, resolve spasticity and exercise in isolated movements, detailed content includes: sitting balance training at levels 1, 2, 3; sit‐stand training; sitting knee and ankle dorsiflexion exercises; standing balance training at levels 1, 2, 3; supported and assisted stepping exercises (c) Middle and late recovery phase (equivalent to recovery phase): When spasms are more or less under control, the aim of training is to generate fine, co‐ordinate, quick random movements. Detailed content includes: continuation of gait re‐education and postural correction, so as to allow further improvement of body function; upper limb and hand isolated movement training" Length of intervention period: 6 weeks Number of sessions and length of individual sessions: intervention frequency was 60 minutes/session, 2/wk. Therapists provided training for the participants 45 minutes/session, 1/d, 5/wk. Rehabilitation nurses provided ADL supervision and guidance at least 60 minutes/d, 5/wk. After discharge from the hospital, therapists continued to provide training during participants' follow‐up visits, frequency unchanged; rehabilitation nurses provided home ADL system Intervention provider: therapists and rehabilitation nurses (2) No physical rehabilitation (n = 50) "... received conventional therapy and nursing care in the Department of Neurology" The individual components delivered are listed in Table 28. Based on the individual components, this intervention is categorised as comprising no intervention Length of intervention period: not stated Number of sessions and length of individual sessions: not stated Intervention provider: not stated (Table 24)
Outcomes	**Independence in ADL scales:** NR **Motor function scales:** Fugl‐Meyer Assessment (FMA) **Measures of balance:** NR **Measures of gait velocity:** NR **Length of stay:** NR **Adverse events:** NR **Other measures:** Stroke Impact Scale **Time points when outcomes were assessed:** "outcome assessments for both groups were completed before and after 6 weeks of intervention"
Comparison	**Amount of physical rehabilitation in groups:** differs **Categorisation of comparison:** 1. Physical rehabilitation versus no or limited physical rehabilitation (dose differs) (limited = < 50% of dose)
Funding & conflicts of interest	**Funding statement:** not reported **Conflict of interest statement:** not reported
Synthesis	**Quantitative synthesis:**Analysis 1.2
Notes	Original study translated from Chinese to English by member of our team (PLC).

**Table:** Ding 2015

***Study characteristics***	
Methods	**Study aim:** to analyse and explore the effect of early targeted limb task function or strength training in hemiplegic patients with cerebral infarction **Design:** parallel‐group RCT **Country:** China **Study recruitment and setting:** see Table **Involvement of patients/public/professionals/others:** no
Participants	**Study population (number randomised):** 100 **Inclusion criteria:** Not reported **Exclusion criteria:** Not reported **Participants:** detailed in Table **PROGRESS‐plus:** summarised in Table
Interventions	(1) Task‐specific training "Task function training can be implemented by imitating actions such as eating bananas and grasping soft balls, and the number of independent actions completed by the patient is calculated. Some patients' disease recovery degree set passive and active practice time, adjust the patient's own movement by means of visual feedback, so as to continuously improve the quality of the patient's movement completion" (2) Strength training "Through resistance exercise to continuously improve the patient's muscle strength [2]. If the muscle strength of the hemiplegic limb of the patient is zero, the patient should be encouraged to instruct the hemiplegic limb to exert force in the same direction. The clinical nurses support the hemiplegic limb of the patient with both hands and provide strength support according to the direction of the patient's consciousness. Usually after about 3 days, the patient can move voluntarily." Both groups: "physical rehabilitation exercise was started. The strength training group mainly carried out strength training during the limb training process, and the task function training group mainly carried out functional training. The specific training steps are: 1. Change the patient's position regularly. When turning over to the affected side independently, the patient is in a supine position, holding both hands crossed, the upper limb of the unaffected side is used to form a driving effect on the affected side, and the lower limb of the unaffected side is flexed; Cross your hands, stretch your upper body upwards, and turn to the unaffected side. 2. Ensure a good limb position, prevent upper limb flexion, stretch lower limb, and fix the patient's foot position through the foot plate to avoid foot drop [1]. 3. Joint activity training. The patient's joints move in all directions about 4 times a day, 15 to 20 times/time, and the affected limb is driven by the healthy limb to implement passive movement. 4. Sitting balance training. Use the patient's healthy leg to hook the lower part of the affected leg, lift the affected leg through the healthy leg, move it to the edge of the bed, and use the healthy leg to support the body to sit up. 5. Targeted physical training. [this comprised the trial intervention, stated above] 6. Standing balance training. Instruct the patient to bend the trunk forward, move the center of gravity forward, flex the knee joint and hip joint forward, then the upper body hip joint, and the magical trunk and lower limbs stand slowly; 7. Walking training. Focus on the implementation of ankle dorsiflexors, knee flexors and weight‐bearing capacity training on the affected side. Move the healthy leg back and forth to perform further training on the balance of the patient's affected leg. 8. Daily life ability training. The patients were trained to brush their teeth, undress and eat, 4‐5 times/d, about 30 min/time."
Outcomes	**Independence in ADL scales:** Barthel Index **Motor function scales:** Fugl‐Meyer assessment **Measures of balance:** NR **Measures of gait velocity:** NR **Length of stay:** NR **Adverse events:** NR **Other measures:** NR **Time points when outcomes were assessed:** admission, month 3 and 6
Comparison	**Amount of physical rehabilitation in groups:** equivalent **Categorisation of comparison:** 3 (ii) Comparison of different approaches, (dose equivalent; with both/all groups also receiving other physical rehab)
Funding & conflicts of interest	**Funding statement:** not reported **Conflict of interest statement:** not reported
Synthesis	**Quantitative synthesis:**Analysis 7.1; Analysis 7.2; Analysis 8.1; Analysis 8.2
Notes	This study was published in Chinese and translated into English using Google Translate.

**Table:** DOSE 2020

***Study characteristics***	
Methods	**Study aim:** investigated the effect of higher therapeutic exercise doses on walking during inpatient rehabilitation **Design:** multi‐arm RCT (3‐arm) **Country:** Canada **Study recruitment and setting:** see Table **Involvement of patients/public/professionals/others:** no
Participants	**Study population (number randomised):** 75 **Inclusion criteria:** Aged 18 years+ Within 10 weeks of stroke with lower extremity hemiparesis (< 4/5 manual muscle grade in at least one of the major lower extremity muscles) Pre‐stroke disability < 2 on the modified Rankin Scale Able to ambulate ≥5 metres with up to one person maximum assist and assistive/orthotic device Overground walking speed < 1.0 m/s Able to understand and follow directions Successful completion of a graded exercise stress test using criteria established by the American College of Sports Medicine **Exclusion criteria:** Pre‐existing health condition that included a gait disorder Another neurological condition (e.g. Parkinson’s) Serious medical or painful condition (e.g. active cancer) Enrolled in a drug or exercise rehabilitation study **Participants:** detailed in Table **PROGRESS‐plus:** summarised in Table
Interventions	(1) DOSE1 "Participants randomized to the DOSE1 intervention will have their standard inpatient physical therapy session replaced by the experimental intervention program for a total of 20 sessions (1 h/day, 5 days/week, for four weeks). The intervention protocol will focus on the completion of a minimum of 30 min of weight‐bearing, walking‐related activities that will progressively increase in intensity (heart rate and step count) over the four‐week intervention period using the available equipment in a standard stroke rehabilitation setting (e.g. parallel bars, overhead harness, treadmill support harness). The remaining time in the 60 min therapy session will be allocated to other physical therapy activities specific to the participant’s recovery (e.g. upper extremity exercises, home exercise program). Participants will wear the same monitoring equipment as the control group, but they will be worn in all 20 intervention sessions." (2) DOSE2 "Participants randomized to the DOSE2 intervention group will have their standard inpatient physical therapy session replaced by the exact same procedures that the DOSE1 group receives. In addition, theDOSE2 group will also receive an extra, 1 h exercise session, 5 days/week, for four weeks, that will occur outside of the standard rehabilitation day (i.e. from 4 to 5 pm daily). The content of the second daily exercise session will be similar to the DOSE1 protocol, in that it will contain a minimum of 30 min of weight‐bearing, walking‐related activities and the remaining time will be dedicated to weight‐bearing lower extremity exercises (e.g. strengthening, balanceexercises)." (3) Control ‐ usual care "The control intervention consisted of usual care, inpatient physical therapy which progressed upper and lower limb functional exercises as tolerated and typically provided 5, 1‐hour sessions per week, until the subject was discharged (normally after 4–6 weeks of inpatient rehabilitation)" "usual care, inpatient physical therapy for four weeks, which typically progresses upper and lower limb functional exercises as tolerated. The physicaltherapist(s) treating control participants will have no information on the study protocol. During 10 of the physical therapy sessions, the study coordinator will place monitoring equipment on the participant, but will not disclose any of the monitoring information to the participant or treating therapist. The study coordinator will advise the treating physical therapist to provide the participant’s usual physical therapy care, using the available equipment in a standard stroke rehabilitation setting (e.g. parallel bars, overhead harness, treadmill support harness), when the participant is wearing the monitoring devices. "The therapy sessions for both DOSE1 and DOSE2 were ≈10 minutes longer than Usual Care because the protocol dictated the duration of the DOSE sessions while the Usual Care duration varied depending on the site’s usual practice" Comparison relevant to this review = DOSE1 vs usual care
Outcomes	**Independence in ADL scales:** NR **Motor function scales:** NR **Measures of balance:** Berg Balance Scale **Measures of gait velocity:** 6MWT, 5‐m walk **Length of stay:** NR **Adverse events:** reported by study staff and subjects **Other measures:** Patient Health Questionnaire, EQ‐5DL index, knee strength **Time points when outcomes were assessed:** baseline, post‐evaluation (end of the 4‐week intervention and approximately 5 weeks after the baseline evaluation), 6‐ and 12‐month post‐stroke. Balance outcome reported at end of intervention only.
Comparison	**Amount of physical rehabilitation in groups:** equivalent (Note: approx 10 minutes less in control group) **Categorisation of comparison:** 3.(i) Comparison of different approaches (dose equivalent; with no other physical rehab)
Funding & conflicts of interest	**Funding statement:** Canadian Institutes of Health Research (Doctoral award to Dr Klassen; Operating Grant FDN 143340 to Dr Eng); Heart and Stroke Foundation Canadian Partnership for Stroke Recovery Operating Grant (Dr Eng); Canadian Stroke Network infrastructure (Dr Hill); Brenda Strafford Foundation Chair in Alzheimer Research (Dr Poulin) **Conflict of interest statement:** none
Synthesis	**Quantitative synthesis:**Analysis 7.3; Analysis 7.4; Analysis 7.6; Analysis 8.4
Notes	

**Table:** Du 2014

***Study characteristics***	
Methods	**Study aim:** to investigate the early rehabilitation effect of intensive walking training on hemiplegia patients with acute cerebral infarction **Design:** parallel‐group RCT **Country:** China **Study recruitment and setting:** see Table **Involvement of patients/public/professionals/others:** no
Participants	**Study population (number randomised):** 80 **Inclusion criteria:** Meet the criteria for the diagnosis of acute cerebral infarction in the Chinese Guidelines for the Prevention and Treatment of Cerebrovascular Diseases and the Chinese Guidelines for the Diagnosis and Treatment of Acute Ischemic Stroke and confirmed cerebral infarction by brain MRI or CT Limited function of at least one limb Gait imbalance requires assistive devices or assistants to stand Medically stable, conscious and able to co‐operate with relevant exercises No serious complications (e.g. severe cardiac and pulmonary insufficiency, angina pectoris, fractures and cognitive impairment Disease duration 2 to 4 weeks **Exclusion criteria:** Brain MRI or CT confirmed cerebral haemorrhage or old cerebral infarction Combining severe complications (such as severe cardiopulmonary insufficiency) Severe cognitive impairment Unable to co‐operate with trainers **Participants:** detailed in Table **PROGRESS‐plus:** summarised in Table
Interventions	(1) Walking training "The treatment group carried out early intensive walking training on the basis of basic treatment. The specific methods are as follows: (1)To carry out rehabilitation knowledge education to family members, and guide them to master the relevant knowledge, so as to be able and assist patients to carry out rehabilitation exercises. Rehabilitation specialists develop complete rehabilitation exercise goals and plans for patients. (2)Training while sitting. When the patient's neurological symptoms do not worsen and the vital signs are relatively stable, the sitting training starts. The patient can sit on the side of the bed, bend the trunk and neck sideways, and use the unaffected leg to hook the affected leg to move to the bedside. At the bedside, the nurse pushes on the patient's calf and knees so that the patient can sit up. Sitting training requires the patient's feet to land on the ground stably. When the patient changes from being unable to sit to being able to maintain a still sitting position without assistance, the posture can be adjusted during the training to move the patient's center of gravity to achieve sitting balance. (3)Standing training. The goal of treatment is to get the patient to stand as early as possible. During the training, ask the patient to move the legs back while the body stands up and forward. The affected limb can be fixed with a splint. With the protection of the splint, it can prevent excessive stretching of the muscles of the affected limb due to imbalance and avoid injury. At the same time, pay attention to the correct alignment of the joints to maintain the correct functional posture, and to maintain the correct functional posture. Keep the knee straight and prevent it from flexing, with the patient's hands supported. Hold the equipment to maintain a standing position. When the patient is standing, the legs should be separated from the shoulders, parallel, while training the patient to stand with weight, the patient's centre of gravity can be moved back and forth to perform dynamic and static balance training. (4) Sit‐stand training after the patient completes the standing. During the training phases, before the walking training, alternate sitting and standing training exercise so that the patient can adapt to the state from bed rest to upright at the beginning of walking training. (5) Walking training. Weight bearing walking training," 3 times a day, 5 to 10 minutes initially, can be gradually extended to 30 min each time. "The patient can perform leg training first, bending the affected knee slightly to stand with support, and then slightly step forward with the unaffected leg to exercise the lower part of the affected side. Limb weight bearing and knee flexion control; leg exercise, first step out the affected leg and control the knee extension to swing the unaffected side next leg. However when it is difficult to step out of the affected leg, the nurse can guide and assist the patient's affected leg from the side. Move forward, but in as small a stride as possible and observe the alignment to keep the patient normal walking posture" (2) Conventional rehabilitation "The control group received conventional rehabilitation therapy, that is, during the acute period, the patients were allowed to move in bed or sitting as much as possible, and the patients were allowed to perform non‐ weight‐ bearing limb exercises and receive physical therapy such as massage."
Outcomes	**Independence in ADL scales:** Barthel Index **Motor function scales:** Fugl Meyer assessment **Measures of balance:** NR **Measures of gait velocity:** NR **Length of stay:** NR **Adverse events:** NR **Other measures:** NR **Time points when outcomes were assessed:** before treatment and after 1 month
Comparison	**Amount of physical rehabilitation in groups:** differs **Categorisation of comparison:** 5. Additional therapy + usual therapy versus usual therapy (dose differs)
Funding & conflicts of interest	**Funding statement:** not reported **Conflict of interest statement:** not reported
Synthesis	**Quantitative synthesis:**Analysis 5.1; Analysis 5.2
Notes	

**Table:** Duan 2011

***Study characteristics***	
Methods	**Study aim:** "to study the effect of task‐oriented training on balance function and walking ability in stroke patients" **Design:** parallel‐group RCT **Country:** China **Study recruitment and setting:** see Table **Involvement of patients/public/professionals/others:** no
Participants	**Study population (number randomised):** 20 **Inclusion criteria:** Stroke diagnosis met the Chinese diagnostic criteria Stroke confirmed by CT or MRI Medically stable **Exclusion criteria:** Joint disease Organ disease Unable to complete or undergo assessment **Participants:** detailed in Table **PROGRESS‐plus:** summarised in Table
Interventions	Both groups received upper limb neuromuscular training, electrotherapy, acupuncture, conventional medical and rehabilitation therapy. (1) Task‐oriented training This group also used the exercise methodology reported in (2) to undergo repeated training focusing the stroke survivor’s attention on target movement. (2) Neurodevelopmental training (NDT) Use NDT principles for balance and gait training. The therapist guided the training. Exercises included sitting and standing practice, transverse practice, stepping forward/backward, side‐to‐side, up and down stairs and sit‐to‐stand practice.
Outcomes	**Independence in ADL scales:** NR **Motor function scales:** NR **Measures of balance:** Berg Balance Scale **Measures of gait velocity:** 5‐m walk test **Length of stay:** NR **Adverse events:** NR **Other measures:** NR **Time points when outcomes were assessed:** after 4 weeks of treatment
Comparison	**Amount of physical rehabilitation in groups:** equivalent **Categorisation of comparison:** 3.(ii) Comparison of different approaches (dose equivalent; with both/all groups also receiving other physical rehab)
Funding & conflicts of interest	**Funding statement:** not reported **Conflict of interest statement:** not reported
Synthesis	**Quantitative synthesis:**Analysis 5.1; Analysis 5.2
Notes	This study was published in Chinese and relevant sections translated into English by our author team (PLC).

**Table:** Duncan 1998

***Study characteristics***	
Methods	**Study aim:** "to develop a home‐based balance, strength, and endurance program; to evaluate the ability to recruit and retrain stroke subjects and to assess the effects of the interventions used" **Design:** parallel‐group RCT **Country:** USA **Study recruitment and setting:** see Table **Involvement of patients/public/professionals/others:** no
Participants	**Study population (number randomised):** 20 **Inclusion criteria:** 30 to 90 days post stroke Fugl‐Meyer score of 40 to 90 Orpington prognostic score two to 52 Ambulatory with supervision or assistive device, or both Living at home (less than 50 miles from Kansas) No medical condition that would limit participation MMSE > 18 Able to follow three‐step command **Exclusion criteria:** Medical condition that interfered with outcome assessments or limited participation in submaximal exercise program MMSE < 18 Receptive aphasia that interfered with the ability to follow a 3‐step command **Participants:** detailed in Table **PROGRESS‐plus:** summarised in Table
Interventions	(1) Home‐based rehabilitation (n = 10) Home‐based programme aiming to improve "strength, balance and endurance and to encourage more use of the affected extremity" Assistive and resistive exercises; proprioceptive neuromuscular facilitation (PNF); Theraband exercises; balance exercises; functional activities for the affected upper extremity; progressive walking programme; progressive bicycle ergometer exercise "The study investigator and co‐investigator observed at least 1 therapy session for each subject to ensure standard application of interventions" Treatments followed a detailed written protocol for intervention. Length of intervention period: 8 weeks and instructed to continue programme on own for further 4 weeks Number of sessions and length of individual sessions: approximately 1.5 hours, 3/wk Intervention provider: physical therapist and occupational therapist (2) Usual care (n = 10) Usual care: "the therapy programmes received by the control group varied in intensity, frequency and duration" Three participants received physiotherapy; 7 had physiotherapy and occupational therapy. Types of exercise interventions given were balance training (60%), progressive resistive exercises (40%), bimanual activities (50%) and facilitative exercises (30%). Length of intervention period: "Participants in this group were visited by a research assistant every 2 weeks to assess the patients’ exercise and activity level. Duration of surveillance was 12 weeks" Number of sessions and length of individual sessions: average number of visits for physiotherapy and occupational therapy patients was 39, average duration was 44 minutes Intervention provider: physical therapist and occupational therapist (Table 24)
Outcomes	**Independence in ADL scales:** Barthel Index; Lawton Instrumental ADL **Motor function scales:** Fugl‐Meyer Motor score **Measures of balance:** Berg Balance Scale **Measures of gait velocity:** timed 10‐Metre Walk; Six‐Minute Walk test **Length of stay:** NR **Adverse events:** NR **Other measures:** Orpington Prognostic Scale; Medical Outcomes Study‐36 Health Status Measure; Jebsen test of hand function **Time points when outcomes were assessed:** "Baseline and postintervention assessments; follow‐up testing for postintervention results was performed 12 weeks after the baseline function assessment"
Comparison	**Amount of physical rehabilitation in groups:** differs **Categorisation of comparison:** additional therapy + usual therapy versus usual therapy (dose differs) Note: This comparison has been categorised as "additional therapy + usual therapy versus usual therapy" as consensus decision judged this the best "fit" for this study. However, the "Mixed group" did not actually receive the same "usual therapy" as the control group; rather the Mixed Group received a programme which was similar (but not identical) in content to the usual therapy, but which was greater in dose (but on average the dose of the control group was > 50% of the dose of the Mixed group).
Funding & conflicts of interest	**Funding statement:** "supported by the US Department of Veterans Affairs, VA Rehabilitation Research and Development Service, Washington DC (E94 to 852AP), and by intramural funds from the Center on Aging at the University of Kansas Medical Center." **Conflict of interest statement:** not reported
Synthesis	**Quantitative****synthesis:**Analysis 5.1; Analysis 5.2; Analysis 5.3; Analysis 5.4 **Sensitivity analysis** ‐ removed study to explore the effect of including this (as it does not clearly meet the specific criteria for being a comparison of "Additional therapy + usual therapy versus usual therapy").
Notes	Data provided by study authors used for analyses.

**Table:** Duncan 2003

***Study characteristics***	
Methods	**Study aim:** "to determine the effect of a structured reproducible physiologically based, progressive exercise program on strength, balance, endurance and upper‐extremity function after stroke" **Design:** parallel‐group RCT **Country:** USA **Study recruitment and setting:** see Table **Involvement of patients/public/professionals/others:** no
Participants	**Study population (number randomised):** 100 **Inclusion criteria:** Stroke within 30 to 150 days Able to walk 25 m independently Mild to moderate stroke deficits MMSE > 16 **Exclusion criteria:** Subarachnoid haemorrhage Lethargic, obtunded or comatose Uncontrolled blood pressure Hepatic or renal failure NYHA III/IV heart failure Known limited life expectancy Pre‐stroke disability in self‐care Lived in a nursing home before the stroke **Participants:** detailed in Table **PROGRESS‐plus:** summarised in Table
Interventions	(1) Home‐based rehabilitation (n = 50) Exercise programme at home aimed at improving strength, balance, endurance, upper limb use Included a variety of techniques from different theoretical 'approaches' Techniques included ROM (range of movement exercises), PNF (proprioceptive neuromuscular facilitation), task‐specific training Structured protocols for the exercise tasks, criteria for progression and guidelines for reintroducing therapy after intercurrent illness Length of intervention period: 12 to 14 weeks Number of sessions and length of individual sessions: 36 sessions of 90 minutes Intervention provider: supervised by physiotherapist or occupational therapist (2) Usual care (n = 50) Usual care: Participants in the usual care group had services as prescribed by their physicians. Treating therapists for usual care participants completed a treatment log. "In the usual care group, 46% of the subjects did not receive any postacute rehabilitation services from physical or occupational therapy. Two thirds were provided recommendations for an unsupervised exercise program. Among the usual care group members who did receive therapy, participants received an average of 8.7 (SD 5.3) physical therapy visits and 10.4 (SD 7.1) occupational therapy visits. Physical and occupational therapy services were received separately as prescribed by their physicians" The therapy that participants received was primarily directed at strength, balance, endurance, upper extremity, range of motion, mobility, and ADL/IADL Length of intervention period: varied Number of sessions and length of individual sessions: visited by researcher every 2 weeks Intervention provider: physiotherapists and occupational therapists
Outcomes	**Independence in ADL scales:** NR **Motor function scales:**Fugl‐Meyer (LL) **Measures of balance:** Berg Balance Scale score **Measures of gait velocity:** gait velocity **Length of stay:** NR **Adverse events:** serious adverse events reported **Other measures:** ankle and knee strength **Time points when outcomes were assessed:** "primary outcomes of the study were assessed at 3 months, immediately after the intervention"
Comparison	**Amount of physical rehabilitation in groups:** differs **Categorisation of comparison:** additional therapy + usual therapy versus usual therapy (dose differs). Note: This comparison has been categorised as "additional therapy + usual therapy versus usual therapy" as consensus decision judged this the best "fit" for this study. However, the "Mixed group" did not actually receive the same "usual therapy" as the control group; rather the Mixed Group received a programme which was similar (but not identical) in content to the usual therapy, but which was ‐ on average ‐ greater in dose.
Funding & conflicts of interest	**Funding statement:** "funded by the National Institute on Aging, Claude D. Pepper Center Older Americans Center grant 5P60AG14635" **Conflict of interest statement:** not reported
Synthesis	**Quantitative****synthesis:**Analysis 5.2; Analysis 5.3; Analysis 5.4; Analysis 5.5 **Sensitivity analysis** ‐ remove study to explore effect of including this (as it does not clearly meet the specific criteria for being a comparison of "Additional therapy + usual therapy versus usual therapy").
Notes	

**Table:** Epple 2020

***Study characteristics***	
Methods	**Study aim:** to test the hypothesis that Vojta therapy ‐ as a new physiotherapeutic approach in early stroke recovery ‐ improves postural control and motor function **Design:** parallel‐group RCT **Country:** Germany **Study recruitment and setting:** see Table **Involvement of patients/public/professionals/others:** no
Participants	**Study population (number randomised):** 40 **Inclusion criteria:** Age ≥ 18 years Confirmed (using CT or MRI proven acute ischaemic or haemorrhagic stroke Randomisation within 72 h after onset of symptoms or after a patient was last seen normal Presence of a severe hemiparesis (MRC scale for muscle strength of the arm ≤ 2) Pre‐morbid mRS ≤ 3 Maximum NIHSS score of 25 Informed consent Treatment with recombinant tissue activator (rtPA) and thrombectomy before randomisation was allowed **Exclusion criteria:** Severe cognitive impairment or limitation in communication skills due to aphasia Deteriorated consciousness or dementia (prohibiting comprehension and implementation of physiotherapy) Participation in another clinical trial Pregnant **Participants:** detailed in Table **PROGRESS‐plus:** summarised in Table
Interventions	(1) Vojta therapy "Vojta therapy was implemented by stimulating the chest zone, which is located between the 7th and 8th rib, use of additional zones was allowed to support the activation. The starting positions for Vojta therapy were supine or side lying position with the head turned 30° toward the side being stimulated or side lying. Patients were treated for 30 min with Vojta therapy and afterwards were mobilised with gait training, if feasible." "Vojta therapy, also referred to as the Vojta method or reflex‐locomotion according to Vojta, is a specialised type of physiotherapy that was discovered and developed by the Czech neurologist and child neurologist Václav Vojta. In the 1960s Vojta observed that maintained peripheral pressure stimulation evoked a stereotypic wide‐spread motor response, as a pattern of tonic muscle contractions in both sides of the neck, trunk and limbs as a result of spatial summation that lead to improvement of postural control [5]. Based on the principles of ontogenetic development Vojta defined postural regulation as the control of body posture and uprighting of the body against gravity as elementary components of loco‐motion, as well as target oriented movements of the limbs. To accomplish postural control the individual requires plans and programs (“innate patterns”) that assemble task‐related automatic adjustment of movements and posture [6]. The basic principle of Vojta therapy is the regulation of posture [7], which is attained within the innate movement sequences of reflex‐locomotion, is retrievable at all times and can be found in all forms of human locomotion representing the basis for human movement [5–7]. To activate these innate patterns of movement, the therapist applies pressure to defined zones. There are ten zones distributed over the trunk, the arms and legs (e.g. aponeurosis of the musculus gluteus medius, epicondylus medialis femoris, spina iliac anterior superior, margo medialis scapulae, chest zone between the 7th and 8th rib) [5,8,9]. Vojta therapy is a standardised therapy with defined starting positions (prone, supine or side lying position) and specific initial angular positioning of the trunk and the extremities." (2) Conventional physiotherapy "consisted of repetitive sensoriomotor exercises using existing function of the affected extremity in task‐oriented training and movements used during daily activity, passive movements of the limbs, trunk strengthening exercises, goal directed movements and mobilisation including gait training." Both groups received one treatment session daily consisting of 40 min. Seven sessions in total were given to all patients in both groups.
Outcomes	**Independence in ADL scales:** Barthel Index **Motor function scales:** NR **Measures of balance:** trunk control test **Measures of gait velocity:** NR **Length of stay:** NR **Adverse events:** adverse and serious adverse events **Other measures:** neglect (Catherine Bergego Scale), arm motor function, stroke severity (NIHSS), modified Rankin Scale **Time points when outcomes were assessed:** before therapy; after therapy (day 9); follow‐up (day 90) ‐ Barthel Index and modified Rankin Scale only
Comparison	**Amount of physical rehabilitation in groups:** equivalent **Categorisation of comparison:** comparison of different approaches (dose equivalent; with no other physical rehab)
Funding & conflicts of interest	**Funding statement:** supported by the Hermann and Lilly Schilling foundation for medical research. The funder of the study had no role in study design, data collection, data analysis, data interpretation, or writing of the report **Conflict of interest statement:** BM received a speaker honoraria from the International Vojta Society (IVS). The other authors declare that they have no competing interests.
Synthesis	**Quantitative****synthesis:**Analysis 7.7;Analysis 7.11; Analysis 8.6. (Data presented as median and interquartile range ‐ mean and standard deviations computed from these).
Notes	

**Table:** Fang 2003

***Study characteristics***	
Methods	**Study aim:** "investigate whether additional early physiotherapy after strokeimproved functional recovery in stroke patients" **Design:** parallel‐group RCT **Country:** China **Study recruitment and setting:** see Table **Involvement of patients/public/professionals/others:** no
Participants	**Study population (number randomised):** 156 **Inclusion criteria:** Stroke was defined as acute onset of neurological deficit lasting > 24 hours or leading to death, with no apparent cause other than cerebrovascular disease. Diagnosis of stroke was based on history and clinical examination. Stroke confirmed by CT scan or MRI scan within the first week of stroke onset to confirm the diagnosis **Exclusion criteria:** Signs and symptoms of subarachnoid haemorrhage, TIA, and those with severe cerebral oedema GCS score of 8 or less or with affected limb muscle power grading 3 Premorbid dementia or premorbid severe impairment of the limb Reached the hospital more than 1 week after stroke onset Abnormal high fever, severe pneumonia and cardiac infarction, severe high blood pressure over 200/120 mmHg, Unable to tolerate a 45‐minute physiotherapy session daily Scheduled to be discharged from the hospital within the first week **Participants:** detailed in Table **PROGRESS‐plus:** summarised in Table
Interventions	(1) Rehabilitation (n = 78) "The early therapy included Bobath techniques and passive movements training of the affected limb, and was initiated within the first week after stroke onset. Passive movement training included a series [of] movements of the joints of completely paralytic limbs to prevent contracture and malformation" Length of intervention period: 4 weeks Number of sessions and length of individual sessions: 45 minutes/d, 5 days/wk Intervention provider: "... two experienced rehabilitation therapists from the department of rehabilitation in the hospital" (2) No physical rehabilitation(n = 78) "... routine therapy group received no professional or regular physiotherapy during the whole hospitalization period" "Stroke related symptoms and complications in each group were treated with multidisciplinary approaches in the stroke centre by a special team. No special cognitive or acupuncture therapy was administered" Length of intervention period: no intervention Number of sessions and length of individual sessions: no intervention Intervention provider: no intervention
Outcomes	**Independence in ADL scales:** modified Barthel Index **Motor function scales:** Fugl‐Meyer Assessment (upper limb and lower limb) **Measures of balance:** NR **Measures of gait velocity:** NR **Length of stay:** NR **Adverse events:** NR **Other measures:** Glasgow Coma Scale (GCS), Mini‐Mental State Examination (MMSE), Clinical Neurological Deficit Scale (CNDS) **Time points when outcomes were assessed:** "follow‐up assessments of the above outcome measures were performed 30 days and six months respectively since stroke onset"
Comparison	**Amount of physical rehabilitation in groups:** differs **Categorisation of comparison:** 1. Physical rehabilitation versus no physical rehabilitation (dose differs)
Funding & conflicts of interest	**Funding statement:** "funded by the Chinese Foundation for the Ninth Five‐year Key Task Project (grant number: 96‐906‐02‐23)" **Conflict of interest statement:** not reported
Synthesis	**Quantitative****synthesis:**Analysis 1.1; Analysis 1.2; Analysis 2.1; Analysis 2.2
Notes	This study had high numbers of dropouts from the treatment group (28/78 from treatment group during treatment period) as compared with no dropouts from the control group. Large numbers were lost to follow‐up from both groups for the 6‐month follow‐up (with only 12/50 and 14/78 included at 6 months).

**Table:** Fang H 2010

***Study characteristics***	
Methods	**Study aim:** to discuss the influence of balance training on stroke hemiplegia patients gait and step **Design:** parallel RCT **Country:** China **Study recruitment and setting:** see Table **Involvement of patients/public/professionals/others:** no
Participants	**Study population (number randomised):** 50 **Inclusion criteria:** First ever stroke (haemorrhagic or ischaemic) Met the diagnostic criteria of the Fourth National Conference on Cerebrovascular Diseases Stroke diagnosed using CT or MRI Medically stable Impairment with balance Within 3 to 6 weeks from stroke onset **Exclusion criteria:** Not reported **Participants:** detailed in Table **PROGRESS‐plus:** summarised in Table
Interventions	In addition to standard care (see 2 below). Participants were also given balance training. Four components involved in the balance training method: *Sitting position balance training:* including static balance training in long sitting position and upright sitting position, gradually reducing the base support. Dynamic balance training involved stimulating the overall response of the head and body to the midline *Stand balance training:* knee control training of the pelvic forward and backward tilting movement gradually transitioning to standing on one leg (static) ‐ standing on one leg (moving) ‐ standing on the balance board ‐ standing position balance response training *Sit ‐ stand ‐ sit training:* involving lowering the height of the stool *Walking training and complexity training:* balance training on the support point of the activity, with the help of balance bars, parallel bars, and balance boards. Functional balance training involved 2 sessions/day with each session lasting 30 minutes and delivered 6 days/week over 8 weeks. (2) Standard care Both groups received conventional medical treatment as well as rehabilitation. Includes good limb placement, acupuncture, functional electrical stimulation, neuromuscular promotion technology, standing bed training and ADL training.
Outcomes	**Independence in ADL scales:** Barthel Index **Motor function scales:** NR **Measures of balance:** Berg Balance Scale **Measures of gait velocity:** NR **Length of stay:** NR **Adverse events:** NR **Other measures:** Functional Ambulation Categories (Holden) **Time points when outcomes were assessed:** before and after treatment
Comparison	**Amount of physical rehabilitation in groups:** differs **Categorisation of comparison:** 5. Additional therapy + usual therapy versus usual therapy (dose differs)
Funding & conflicts of interest	**Funding statement:** not reported **Conflict of interest statement:** not reported
Synthesis	**Quantitative synthesis:**Analysis 5.1; Analysis 5.3
Notes	This study was published in Chinese and relevant sections translated into English by our author team (PLC).

**Table:** Fang YN 2004

***Study characteristics***	
Methods	**Study aim:** "to discuss the factors which affect the functional rehabilitation of old patients with stroke" **Design:** parallel‐group RCT **Country:** China **Study recruitment and setting:** see Table **Involvement of patients/public/professionals/others:** no
Participants	**Study population (number randomised):** n = 128 (presented as "young" group (≤ 64 years old), n = 58, and "old" (≥ 65 years old) group, n = 70) **Inclusion criteria:** Met the 1995 evaluative standard revised by the Fourth [National] Academic Conference of Cerebral Vascular Disease **Exclusion criteria:** Not reported **Participants:** detailed in Table **PROGRESS‐plus:** summarised in Table
Interventions	(1) Rehabilitation (n = 50). ("young" n = 25; "old" n = 25) "Both groups used conventional medication, without using acupuncture. In addition, the 'rehabilitation group' used the Bobath technique to deliver massage to paretic limbs, passive ranging exercises. In supine, professional therapists helped exercise the paretic limbs, 1/day, 45min/session, starting 0‐7 days after stroke, for a treatment duration of 3 days" Length of intervention period: 3 days Number of sessions and length of individual sessions: 1/d, 45 minutes/session Intervention provider: therapists (2) No physical rehabilitation (n = 78) ("young" n = 33; "old" n = 45) "Both groups used conventional medication, without using acupuncture. Control group did not undertake this intervention" Length of intervention period: not stated Number of sessions and length of individual sessions: not stated Intervention provider: not stated
Outcomes	**Independence in ADL scales:** modified Barthel Index **Motor function scales:** Fugl‐Meyer Assessment (FMA) (upper and lower limbs) **Measures of balance:** NR **Measures of gait velocity:** NR **Length of stay:** NR **Adverse events:** NR **Other measures:** Glasgow Coma Score, Neurological Functional Deficit Score, Mini Mental State Examination **Time points when outcomes were assessed:** "before intervention, 30 days after intervention, 6 months after intervention"
Comparison	**Amount of physical rehabilitation in groups:** differs **Categorisation of comparison:** physical rehabilitation versus no or limited physical rehabilitation
Funding & conflicts of interest	**Funding statement:** "supported by the grants from Guangdong Administration of Traditional Chinese Medicine, No 103052" **Conflict of interest statement:** not reported
Synthesis	**Quantitative****synthesis:**Analysis 1.1; Analysis 1.2; Analysis 2.1; Analysis 2.2
Notes	Original study translated from Chinese to English by member of our team (PLC). Data presented as a "young" and "old" group for each intervention group.

**Table:** Fan L 2014

***Study characteristics***	
Methods	**Study aim:** to investigate whether task‐intensive rehabilitation training will improve walking function in acute cerebral infarction **Design:** parallel‐group RCT **Country:** China **Study recruitment and setting:** see Table **Involvement of patients/public/professionals/others:** no
Participants	**Study population (number randomised):** 60 **Inclusion criteria:** Clinical diagnosis of first cerebral infarction confirmed by head CT or MRI Aged 40 to 70 years old Stroke onset ≤ 10 days Stable vital signs and condition has stabilised Conscious and willing and able to co‐operate with the treatment Muscle strength of the lower extremity on the affected side of the hemiplegic side of one limb is grade 0‐2 No vestibular function or cerebellar dysfunction Informed consent (patient and family) **Exclusion criteria:** Hemiplegia caused by haemorrhagic stroke or TBI Severe heart and lung disease Uncontrolled hypertension Severe cognitive impairment Severe hearing and/or visual impairment Bone and joint disease Poor compliance **Participants:** detailed in Table **PROGRESS‐plus:** summarised in Table
Interventions	(1) Task specific training In addition to conventional rehabilitation: "task‐intensive training was gradually added, including: sitting training, sitting one‐stop training, standing training and walking training. Provide guidance and training to patients, family members and escorts (nanny or care worker) in stages, teach family members and escorts the correct auxiliary training, and judge whether the training movements of patients are correct, and are supervised by the responsible nurse every day. Week 1 Seated training: Raised bedside seat training, bedside seat training, bedside seat training, and out‐of‐bed seat training. Raise the head of the bed in steps from 30 deg to 80 deg patient tolerated on the bed. After sitting for 30 minutes, start bedside sitting training, and then gradually transition to a wheelchair or stool. 10‐20 min/time, 1‐2 times/d. Week 2 Sit‐One‐Station Training After the patient is acclimated to the wheelchair and stool, the Sit‐One‐Station training begins. The seat is slightly higher for the initial training to allow the patient to stand up and can then be performed in a standard wheelchair, stool or couch. During the standing process, the patient's upper limbs are placed on the bed rail or treatment pole, and the family members or accompanying persons assist the patient to stand up or maintain standing with the help of a healthy hand. 20 min/time, 2 times/d. In the 3rd week, the sitting‐to‐stand training and the standing balance training are to strengthen the sitting‐to‐stand training and at the same time perform the standing training, and at the same time guide the patients to perform the center of gravity transfer training, especially the weight‐bearing training of the lower limbs on the affected side. 30min/time, 2 times/d. In the 4th week and later, standing balance training and walking training continued to strengthen standing balance training. When the patient's standing balance reached the level, the walking training was gradually transitioned. The assistant should stand on the affected side of the patient for proper protection to prevent the patient from falling. During the training, the patients were instructed to adjust the center of gravity, correct the gait, and try to avoid the formation of abnormal gait such as knee hyperextension and circle strokes. Standing balance training 30 min/time, 2 times/d; walking training 10‐30 min/time, 2‐3 times/d, and the training time was gradually extended according to the actual situation of the patients. In all the above training process, it is necessary to observe the patient's condition and pay attention to the gradual and individualization within the patient's tolerance range." (2) Conventional rehabilitation "Routine neurology treatment and nursing were given to the existing comorbidities, and corresponding symptomatic treatment was given; routine early rehabilitation therapy was received, including neuromuscular facilitation, air pressure therapy, electroacupuncture, neuromuscular electrical stimulation, electric stand‐up bed and other treatments."
Outcomes	**Independence in ADL scales:** NR **Motor function scales:** NR **Measures of balance:** NR **Measures of gait velocity:** NR **Length of stay:** NR **Adverse events:** "records of adverse reactions or adverse events include whether there is aggravation, blood pressure, heart rate changes, falls, etc. during training." **Other measures:** Functional Ambulation Categories **Time points when outcomes were assessed:** before and after 8 weeks of training
Comparison	**Amount of physical rehabilitation in groups:** differs **Categorisation of comparison:** 5. Additional therapy + usual therapy versus usual therapy (dose differs)
Funding & conflicts of interest	**Funding statement:** not reported **Conflict of interest statement:** not reported
Synthesis	**Qualitative synthesis:** no outcomes suitable for inclusion in meta‐analyses.
Notes	This study was published in Chinese and relevant sections translated into English using Google Translate.

**Table:** Fan WK 2006

***Study characteristics***	
Methods	**Study aim:** "to explore the effects of standardized three stages' rehabilitation on the motor function in stroke patients with hemiplegia" **Design:** parallel‐group RCT **Country:** China **Study recruitment and setting:** see Table **Involvement of patients/public/professionals/others:** no
Participants	**Study population (number randomised):** 82 (of whom 2 in the control group died) **Inclusion criteria:** Aged 40 to 80 years, and had motor deficits Met the 1995 evaluative standard revised by the Fourth [National] Academic Conference of Cerebral Vascular Disease Assessed whether stroke type was ischaemic or haemorrhagic and confirmed by CT scan or MRI Medically stable within one week GCS > 8 Willing to provide informed consent **Exclusion criteria:** Active liver disease, compromised liver or kidney function Cardiovascular disorder Malignant tumour(s) History of intellectual disorders, cardiorespiratory issue(s) Paralysis of four limbs Ischaemic or haemorrhagic stroke outwith 3 weeks Inaccessible homes located in the outskirts Psychological illness(es) Deaf, mute individuals **Participants:** detailed in Table **PROGRESS‐plus:** summarised in Table
Interventions	(1) Three‐stage rehabilitation (n = 42) "Therapists delivering the rehabilitative treatment all underwent the same training. For acute stroke patients, up to one month after stroke, the first phase of treatment was undertaken, mainly carried out in the Department of Neurology ward, and while given conventional medical treatment, these patients were given early bedside rehabilitative treatment after becoming medically stable, focusing on physiotherapy, with rehabilitative treatment commencing within 1 week after patients were medically stable; for subacute stroke patients, the second (beyond 1 to 3 months after stroke) and third phase (beyond 3 to 6 months after stroke) of treatment [were] undertaken, and based on the patient’s condition and level of functional recovery, patients were transferred to rehabilitation ward/centre for further rehabilitation, or discharged home, where a therapist would conduct home visits to guide the patient, and help the patient conduct necessary functional training, until the end of follow‐up. Here, ‘stage one rehabilitation' referred to the patient’s early conventional medicine treatment at the hospital’s emergency or Department of Neurology ward, as well as early stage rehabilitative treatment, 'stage two rehabilitation' referred to the patient’s rehabilitative treatment at the rehabilitation ward/centre, 'stage three rehabilitation' referred to the continuation of rehabilitative treatment at the community or home setting. The rehabilitative treatment method combined physiotherapy and occupational therapy into a holistic method: early stage of treatment was focused on physiotherapy, progressing to occupational therapy. Physiotherapy treatment included: (1) supine and sitting positions to combat spasticity; (2) passive ranging exercise of all affected limb joints, including mobilisation of the shoulder girdle, starting from small to large ranges of motion, to avoid causing pain to the patient; (3) rolling practice (from affected and non‐affected sides); (4) bridging exercises; (5) exercises in ankle dorsiflexion and wrist extension; (6) outwith therapy time, sitting practice: headrest of bed lifted to 30 degrees, and, if participant could tolerate the angle for longer than 30 minutes, the angle was increased by another 10 degrees the following day, until participant could tolerate 90 degrees for longer than 30 minutes, which would then be followed by lying‐to‐sitting up training (from non‐affected and affected sides); (7) sitting balance training (sitting on edge of bed); (8) sit‐to‐stand training; (9) standing balance training; (10) gait training, etc. During the flaccid phase, focus was on postural correction, passive activities and active/passive practice, rolling, lying‐to‐sitting on edge of bed. During the spasticity phase, focus was on relaxation practice for spastic muscles, antispasticity manual techniques and muscle training for non‐spastic muscles, as well as practising exercises in isolated movements. Occupational therapy treatment was based largely on the participant's functional ability at each stage; the appropriate intervention would be given, namely, feeding, grooming, donning, bed‐to‐wheelchair and wheelchair‐to‐bed transfer and other ADLs, as well as practice of woodwork, sewing and other handicrafts and ring‐insertion games, jigsaws and other leisure activities. Stage one rehabilitation included antispasticity positioning, passive training of limbs, active training of non‐affected limbs under guidance and deep breathing, as well as training of abdominal muscles, sitting up from lying, sitting balance and standing up training, etc, to train the participant's ability to get up from the bed (treatment was done), one/d, 45 minutes/session, five/wk, during the training period, the participant's family or nursing workers were taught the correct supplementary exercises and methods of care concurrently, to achieve partial training out with therapy times, while reducing the damage to affected limbs due to inappropriate nursing care; stage two rehabilitation mainly consisted of standing training, standing balance, single‐leg standing, gait training and up‐and‐down stair training etc, to train the participant's ability to ambulate (treatment was done) 2x/day, 30‐45 minutes/session, 5x/week; stage three rehabilitation mainly consisted of feeding, donning, grooming, personal hygiene management and other ADL ability training (treatment was done) 2x/day, 30‐45 minutes/session, five to seven days/wk. Stage one early rehabilitation and stage two rehabilitation within rehabilitation ward/centre were delivered by therapists, while concurrently teaching participant's family or nursing workers how to assist the participant in training; during stage three community rehabilitation, therapists conducted fortnightly home visits, and while delivering occupational therapy and necessary physiotherapy, also taught the participant's family or nursing workers how to help the participant train, leaving them to assist the participant in completing the remaining daily necessary training." Length of intervention period: 6 months Number of sessions and length of individual sessions: "stage one rehabilitation‐1/day, 45 min/session, 5 x/week; stage two rehabilitation‐2/day, 30 – 45 min/session, 5x/week and stage three rehabilitation‐ 2/day, 30 – 45 min/session, 5 – 7 days/week" Intervention provider: "Stage one early rehabilitation and stage two rehabilitation within rehabilitation ward/centre, was delivered by therapists, while concurrently teaching patient’s family or nursing workers how to assist the patient in training; some patients during the second stage community rehabilitation had therapists conducting home visits to provide guidance on rehabilitation treatment 1x/week, teaching patient’s family or nursing workers how to assist the patient in training while treating the patient, leaving them to assist the patient to complete the remaining bulk of the training in the week; during stage three community rehabilitation, therapists conducted fortnightly home visits, and while delivering occupational therapy and necessary physiotherapy, also taught the patient’s family or nursing workers how to help the patient train, leaving them to assist the patient in completing the remaining daily necessary training)" (2) No physical rehabilitation (n = 40) (of whom 2 died) "Patients in the control group were not given any standardised rehabilitation therapy, but were given the same usual medical treatment as the treated group. It was noted that some patients self‐trained after verbal advice from their doctor, while some patients’ families assisted the patient in movement based on their own knowledge, and the possibility that some patients underwent certain rehabilitation treatment after seeking help from other rehabilitation organisations upon discharge could not be excluded" Length of intervention period: not stated Number of sessions and length of individual sessions: not stated Intervention provider: not stated
Outcomes	**Independence in ADL scales:** NR **Motor function scales:** NR **Measures of balance:** NR **Measures of gait velocity:** NR **Length of stay:** NR **Adverse events:** NR **Other measures:** Functional Comprehensive Assessment **Time points when outcomes were assessed:** "at recruitment, 1 month after stroke, 3 months after stroke, 6 months after stroke"
Comparison	**Amount of physical rehabilitation in groups:** differs **Categorisation of comparison:** 1. Physical rehabilitation versus no physical rehabilitation (dose differs)
Funding & conflicts of interest	**Funding statement:** not reported **Conflict of interest statement:** not reported
Synthesis	**Quantitative synthesis:**Analysis 1.2 **Qualitative synthesis:** no data suitable for analysis
Notes	Original study (paper by Fan et al, 2006) translated from Chinese to English by member of our team (PLC). Paper by Sun Li‐min et al 2007 not translated from Chinese, and therefore not used during data extraction and assessment.

**Table:** Fan WS 2006

***Study characteristics***	
Methods	**Study aim:** to explore the effects of proprioceptive neuromuscular facilitation (PNF) therapy in stroke patients with diabetes **Design:** parallel‐group RCT **Country:** China **Study recruitment and setting:** see Table **Involvement of patients/public/professionals/others:** no
Participants	**Study population (number randomised):** 45 **Inclusion criteria:** Stroke confirmed by examination and met the diagnostic criteria of the 1995 National Conference on Cerebrovascular Diseases Hemiplegia Clear consciousness 5 to 10 years of diabetes history Application of insulin therapy **Exclusion criteria:** Previous history of stroke or transient ischaemic attack or reversible ischaemic neurological disorder or subarachnoid haemorrhage Severe complications Bilateral lesions **Participants:** detailed in Table **PROGRESS‐plus:** summarised in Table
Interventions	Both groups received routine neurological treatment. (1) PNF "Used PNF training methodology". Rehabilitation used PNF concepts such as hold‐relax, proprioceptive and 'exteroceptor' factors. Examples of exercises included vibration, compression and brushing. One session per day with each session lasting 30‐45 minutes/session. (2) Conventional rehabilitation The control group received "typical rehabilitation training" which involved good limb positioning, bed limb function training, bed up training, sitting balance training, standing balance training, gait training, walking down stairs training and ADL training. One session per day with each session lasting 30‐45 minutes/session. Dose‐matched. One time/day, 30 to 40 minutes a session
Outcomes	**Independence in ADL scales:** Barthel Index **Motor function scales:** Fugl‐Meyer Assessment **Measures of balance:** NR **Measures of gait velocity:** NR **Length of stay:** NR **Adverse events:** NR **Other measures:** NR **Time points when outcomes were assessed:** before and after 40 days of treatment
Comparison	**Amount of physical rehabilitation in groups:** equivalent **Categorisation of comparison:** 3.(i) Comparison of different approaches (dose equivalent; with no other physical rehab)
Funding & conflicts of interest	**Funding statement:** not reported **Conflict of interest statement:** not reported
Synthesis	**Quantitative synthesis:**Analysis 7.7; Analysis 7.8
Notes	This study was published in Chinese and relevant sections translated into English by our author team (PLC).

**Table:** Fan X 2009

***Study characteristics***	
Methods	**Study aim:** "To study the effect of active and passive exercise training on the functional recovery oflower limbs in stroke patients with hemiplegia" **Design:** parallel‐group RCT **Country:** China **Study recruitment and setting:** see Table **Involvement of patients/public/professionals/others:** no
Participants	**Study population (number randomised):** 80 **Inclusion criteria:** Full text not available **Exclusion criteria:** Full text not available **Participants:** detailed in Table **PROGRESS‐plus:** summarised in Table
Interventions	(1) Exercise training Intervention group ‐ in addition to the conventional rehabilitation: active and passive exercise training. Some electrical modalities. Dose = once daily, 30 mins/session, 6 times a week, for 4 weeks (2) Conventional rehabilitation Everybody received medical treatment + bed exercises, sitting balance training, sit to stand, standing, standing balance, gait training, upper limb and hand function limb.
Outcomes	**Independence in ADL scales:** NR **Motor function scales:** Fugl‐Meyer assessment **Measures of balance:** NR **Measures of gait velocity:** NR **Length of stay:** NR **Adverse events:** NR **Other measures:** manual muscle testing **Time points when outcomes were assessed:** before and after 4 weeks of treatment
Comparison	**Amount of physical rehabilitation in groups:** differs **Categorisation of comparison:** 5. Additional therapy + usual therapy versus usual therapy (dose differs)
Funding & conflicts of interest	**Funding statement:** not reported **Conflict of interest statement:** not reported
Synthesis	**Qualitative synthesis:** No results presented (abstract data only available)
Notes	This abstract was published in Chinese and the abstract was translated into English using Google Translate. We requested the full text from University of Edinburgh Library (twice) but the wrong paper was provided.

**Table:** Fan Y 2015

***Study characteristics***	
Methods	**Study aim:** to study the clinical effect of rehabilitation treatment for patients with cerebral vascular disease **Design:** parallel‐group RCT **Country:** China **Study recruitment and setting:** see Table **Involvement of patients/public/professionals/others:** unclear
Participants	**Study population (number randomised):** 124 **Inclusion criteria:** Met diagnostic criteria of the Fourth National Cerebrovascular Disease Conference using CT or MRI Aged < 85 years old GCS > 8 points Neurological deficits **Exclusion criteria:** Transient cerebral ischaemia or subarachnoid haemorrhage Other deteriorating disease New infarction or haemorrhage Severe heart, liver, lung, kidney, and other organ dysfunction **Participants:** detailed in Table **PROGRESS‐plus:** summarised in Table
Interventions	(1) Tertiary rehabilitation therapy "patients in the observation group also received tertiary rehabilitation therapy" (2) Conventional "patients in the control group received conventional neurological treatment, mainly for patients with Intracranial decompression and dehydration should be performed to maintain stable blood pressure levels, and brain cell metabolism should be improved." (no other intervention details provided)
Outcomes	**Independence in ADL scales:** Barthel Index **Motor function scales:** Fugl Meyer Assessment **Measures of balance:** NR **Measures of gait velocity:** NR **Length of stay:** NR **Adverse events:** NR **Other measures:** National Institute of Health Stroke Score (NIHSS) **Time points when outcomes were assessed:** before and after treatment
Comparison	**Amount of physical rehabilitation in groups:** differs **Categorisation of comparison:** 1. Physical rehabilitation versus no or limited physical rehabilitation (dose differs) (limited = < 50% of dose) [Lack of details provided on intervention ‐ assumed that conventional rehabilitation is < 50% dose of the "tertiary" rehabilitation]
Funding & conflicts of interest	**Funding statement:** not reported **Conflict of interest statement:** not reported
Synthesis	**Qualitative synthesis:** insufficient details/data provided
Notes	Study published in Chinese and translated to English using Google Translate.

**Table:** FeSTivaLS 2014

***Study characteristics***	
Methods	**Study aim:** "to estimate the recruitment and attrition rates for a subsequent clinical trial; to assess and refine the practicality of a participant recruitment strategy for use in a subsequent clinical trial; to assess the occurrence of potential adverse reactions to functional strength training provided for people in their homes between six months and five years after stroke" **Design:** parallel‐group RCT **Country:** UK **Study recruitment and setting:** see Table **Involvement of patients/public/professionals/others:** no
Participants	**Study population (number randomised):** 52 **Inclusion criteria:** Aged 18 years+ Between 6 months ‐ 5 years after a stroke (infarct or haemorrhage) in the anterior circulation (anterior or middle cerebral artery) "able to walk four steps with support from one person and/or an assistive device, but in 15 seconds unable to step on and off a 7.5 cm high block, with either leg, more than 14 times (Step Test)" "able to move the paretic hand from lap to table surface, but unable to pick up £1 coins individually and stack four in an even pile" "able to follow a one‐stage command with the non‐paretic upper limb" No known pathology contraindicating participation in FST Not participating in formal physical therapy **Exclusion criteria:** Not reported **Participants:** detailed in Table **PROGRESS‐plus:** summarised in Table
Interventions	(1) Functional strength training ‐ lower limb (FST‐LL) "FST ... involves repetitive progressive resistive exercise during functional task‐specific training ... Examples of FST‐LL exercises used in this trial include variations of: standing up and sitting down; ascending and descending stairs and/or using a block for step up/step down exercise; practice of balance activity including one‐leg standing; and walking whilst avoiding and/orstepping over obstacles." (2) Functional strength training ‐ upper limb (FST‐UL) "Examples of FST‐UL exercises used in this trial include variations of: reaching, picking up a jug containing water and pouring contents into a container; picking up a container and removing the screw lid; reaching down to a foot and then using both hands to lace up a shoe; and picking up and then moving everyday objects of various weights and sizes to position them in different locations of diverse heights." For both groups: "Activities were progressed systematically, increasing the amount of resistance and number of repetitions. Resistance was varied using external resistance bands and/or weights and also increasing task difficulty through strategies such as decreasing seat height for sit or stand activities and increasing or decreasing the requirement for hand grip span. Progression was informed by the Oxford program. This provided a framework for advancing the strengthening program." "A research therapist provided participants with their allocated intervention for up to one hour a day, four days a week, for six weeks."
Outcomes	**Independence in ADL scales:** NR **Motor function scales:** Modified Rivermead Mobility Index **Measures of balance:** NR **Measures of gait velocity:** Timed Up and Go Test **Length of stay:** NR **Adverse events:** "For adverse reactions it was specifically postulated that paretic limb pain could occur if FST was provided in a dose (amount in hours) that was too much for a participant. Pain was considered an adverse reaction if the therapist providing FST received a verbal or behavioral report of pain on four consecutive treatment days. Paretic limb pain was included in the monitoring as it was highlighted to the research team that this was a specific clinical concern." **Other measures:** cost‐effectiveness, EuroQol‐5D, functional ambulation categories, Action Research Arm Test (ARAT), nine hole peg test **Time points when outcomes were assessed:** baseline, after 6 weeks of intervention, 6 weeks later (follow‐up)
Comparison	**Amount of physical rehabilitation in groups:** differs **Categorisation of comparison:** 2. Physical rehabilitation versus attention control (dose of physical rehab differs; dose of intervention equivalent)
Funding & conflicts of interest	**Funding statement:** "Stroke Association" **Conflict of interest statement:** "no competing interests"
Synthesis	**Quantitative synthesis:**Analysis 3.2; Analysis 3.4; Analysis 4.2; Analysis 4.4
Notes	The trial is registered on the Current Controlled Trials database (ISRCTN71632550).The full protocol has also been published (see Mares et al (2013) (FeSTivaLS 2014))

**Table:** Frimpong 2014

***Study characteristics***	
Methods	**Study aim:** to investigate the effect of task‐oriented circuit training on ambulatory functions of stroke survivors in early stages of rehabilitation **Design:** parallel‐group RCT **Country:** Ghana **Study recruitment and setting:** see Table **Involvement of patients/public/professionals/others:** no
Participants	**Study population (number randomised):** 20 **Inclusion criteria:** First‐episode single stroke Stroke duration < 3 months Able to walk 10 metres independently with or without walking aid FAC ≥ 3 (FAC) score of 3 or more **Exclusion criteria:** aphasia, cardiac arrhythmias or any condition which may affect exercise participation **Participants:** detailed in Table **PROGRESS‐plus:** summarised in Table
Interventions	(1) Circuit training "The circuit comprised six (6) exercise stations: (1)Station 1 ‐ Treadmill walking ... 3 minutes with speed of 0.9 km/h (2)Station 2 ‐ Push‐Ups ... 1 setof 10 repetitions in a minute (3)Station 3 ‐ Squatting exercise ... 1 set of 15 repetitions in a minute (4)Station 4 ‐ Straight Leg Raise exercise ... 1 set of 10 repetitions in a minute (5)Station 5 ‐ Stairs walking ... 3minutes (6)Station 6 ‐ Cycling ... 3minutes The frequency of the circuit training was three times per week, for eight weeks. The circuit was performed twice per session. The intensity of the circuit training was targeted between 10 (light) and 15 (hard or heavy) of the Borg’s scale of rating of perceived exertion. Them duration per training session was 35 minutes including: warm‐up (5 minutes), first circuit training (12 minutes), rest interval (5 minutes) and the second or repeat circuit training (12 minutes). Thus, subjects performed 105 minutes of circuit training per week for 8 weeks. The type and nature of exercise stations was to achieve a total lower limb as well as total body work‐out. The same circuit was used throughout the study period. The resting heart rate and blood pressure were monitored before and after each session. In addition to the circuit training, the CTG received the usual conventional therapy as the CG." (2) Control ‐ conventional "The control group received only the usual conventional therapy of passive and active exercises. Subjects also performed upper limb strengthening exercises, walking re‐education, as well as standing and balance retraining carried out between parallel bars. Subjects performed walking re‐education by starting between parallel bars and progressing to free overground walking with aids (cane)."
Outcomes	**Independence in ADL scales:** NR **Motor function scales:** NR **Measures of balance:** NR **Measures of gait velocity:** 10‐m walk test, 6‐minute walk test **Length of stay:** NR **Adverse events:** NR **Other measures:** Functional Ambulation Category **Time points when outcomes were assessed:** baseline and 4th and 8th week (NB: treatment period was 8 weeks)
Comparison	**Amount of physical rehabilitation in groups:** differs **Categorisation of comparison:** 5. Additional therapy + usual therapy versus usual therapy (dose differs)
Funding & conflicts of interest	**Funding statement:** not reported **Conflict of interest statement:** not reported
Synthesis	**Quantitative synthesis:**Analysis 5.4
Notes	Results presented in Table 2 and 3 contradict results presented in Tables 5 and 6. Results from Table 5 and 6 used as they match the textual summary “Task‐oriented circuit training improved ambulatory functions of stroke survivors in the acute stage”.

**Table:** Gelber 1995

***Study characteristics***	
Methods	**Study aim:** to compare 2 therapy approaches in the rehabilitation of pure motor hemiparetic stroke **Design:** parallel‐group RCT **Country:** USA **Study recruitment and setting:** see Table **Involvement of patients/public/professionals/others:** no
Participants	**Study population (number randomised):** 27 **Inclusion criteria:** Pure motor hemiparetic ischaemic stroke Less than 1 month post stroke No cognitive, language, visual, sensory or bilateral deficits No history of stroke No premorbid use of walking stick **Exclusion criteria:** Not reported **Participants:** detailed in Table **PROGRESS‐plus:** summarised in Table
Interventions	(1) Neurodevelopmental Technique (NDT) group (n = 15) "Inhibition of abnormal muscle tone and initiation of normal (good quality) motor movements with progression through developmental sequences prior to advancing to functional activities. Therapy techniques included tone inhibition and weight bearing activities, and encouraged patients to use their affected side. Resistance exercises and use of abnormal reflexes and mass movements were avoided." All therapists had received training and evaluation in use of the approaches and were given "strict guidelines" for treatment. Length of intervention period: "continued for the duration of the inpatient and outpatient rehabilitation programmes" Number of sessions and length of individual sessions: not stated Intervention provider: "these treatment approaches were used by both the physiotherapists and occupational therapists who treated the patients, and were used throughout the patients' time as both inpatients and outpatients. All interventions were administered according to allocated treatment group. The nursing staff reinforced any practice of techniques that patients were to carry out outside their treatment sessions" (2) Functional training (n = 12) "Practicing functional tasks as early as possible even in the presence of spasticity or abnormal postures" "Passive range of movement; resistive exercises; assistive devices and bracing allow use of unaffected side to perform functional tasks. Therapists had all received training and evaluation in the use of the approaches, and were given ‘strict guidelines' for treatment" Length of intervention period: "continued for the duration of the inpatient and outpatient rehabilitation programmes" Number of sessions and length of individual sessions: not stated Intervention provider: as above
Outcomes	**Independence in ADL scales:** Functional Independence Measure **Motor function scales:** NR **Measures of balance:** NR **Measures of gait velocity:** parameters of gait **Length of stay:** length of stay and inpatient hospital costs **Adverse events:** NR **Other measures:** Box and Block test; 9‐Hole Peg test **Time points when outcomes were assessed:** "All of the outcome measures were evaluated at admission, discharge, six months and at twelve months follow‐up"
Comparison	**Amount of physical rehabilitation in groups:** equivalent **Categorisation of comparison:** 3.(i) Comparison of different approaches (dose equivalent; with no other physical rehab)
Funding & conflicts of interest	**Funding statement:** not reported **Conflict of interest statement:** not reported
Synthesis	**Quantitative synthesis:**Analysis 7.7; Analysis 7.10; Analysis 8.6; Analysis 8.9
Notes	

**Table:** Ge W 2003

***Study characteristics***	
Methods	**Study aim:** "to observe the impact of early rehabilitation therapy on ADL in stroke patients with paralysis" **Design:** parallel‐group RCT **Country:** China **Study recruitment and setting:** see Table **Involvement of patients/public/professionals/others:** no
Participants	**Study population (number randomised):** n = 40 (or 48—see notes below) **Inclusion criteria:** Head CT confirmed first onset of acute stroke followed by paralysis **Exclusion criteria:** History of stroke Onset of transient cerebral ischaemia, reversible and ischaemic nerve disorder, subarachnoid haemorrhage Serious complications Bilateral lesions **Participants:** detailed in Table **PROGRESS‐plus:** summarised in Table
Interventions	(1) Rehabilitation (n = 20) "Rehabilitation group received rehabilitation therapy after primary assay, including Bobath, middle‐frequency electrotherapy, and auxiliary acupuncture and massage. Bobath method was the focus of movement training, such as position treatment such as lateral lying down for minutes during relaxing paralysis; to support sitting position with affected limbs before spasmodic paralysis, active and passive movements of joints, flexion and extension of limbs, anteversion and rotation of torso, to place the affected limb on health limb; lying down‐sitting‐standing gait training" Length of intervention period: not stated Number of sessions and length of individual sessions: "once a day, 30‐45 minutes for each time" Intervention provider: not stated (2) No physical rehabilitation (n = 28—see notes below) "All patients received the same routine therapy but the control group received no rehabilitation therapy" Length of intervention period: no intervention Number of sessions and length of individual sessions: no intervention Intervention provider: no intervention
Outcomes	**Independence in ADL scales:** ADL; "ADL was assayed for all participants" **Motor function scales:** NR **Measures of balance:** NR **Measures of gait velocity:** NR **Length of stay:** NR **Adverse events:** NR **Other measures:** NR **Time points when outcomes were assessed:** "primary assay should be done within 7 days after onset, while the final assay [should] be carried out 3 months after onset"
Comparison	**Amount of physical rehabilitation in groups:** differs **Categorisation of comparison:** physical rehabilitation versus no or limited physical rehabilitation (dose differs)
Funding & conflicts of interest	**Funding statement:** not reported **Conflict of interest statement:** not reported
Synthesis	**Qualitative synthesis:** no data suitable for analysis
Notes	Abstract only—limited information available Mismatch in number of participants reported (total of 40, but when broken down into the 2 groups, 20 in the rehabilitation group and 28 in the control group). Need to contact study authors to clarify this point Middle‐frequency therapy is well described in the methods.

**Table:** Ge Y 2020

***Study characteristics***	
Methods	**Study aim:** to observe the effect of mandatory exercise therapy in the rehabilitation of stroke hemiplegia **Design:** parallel‐group RCT **Country:** China **Study recruitment and setting:** see Table **Involvement of patients/public/professionals/others:** no
Participants	**Study population (number randomised):** 96 **Inclusion criteria:** Meets the diagnostic criteria for stroke hemiplegia formulated by the 4th National Cerebrovascular Disease Conference **Exclusion criteria:** Patients with serious systemic diseases such as heart, liver and kidney Patients with depressive psychosis, Alzheimer's, epilepsy, and other psychiatric disorders **Participants:** detailed in Table **PROGRESS‐plus:** summarised in Table
Interventions	(1) Compulsory exercise therapy "Give mandatory exercise therapy: 1. Carry out treadmill training, the training intensity is controlled at 8.30~16.70 m/min, and the nursing staff accurately records the training time, 20 min/time; 2. For rehabilitation treadmill training, medical staff should give patients necessary physical support for treadmill training to prevent falls; 3. Quadriceps resistance training should be completed with the help of medical staff. During the training process, the patient should be instructed to slow down as much as possible to avoid muscle strain, 30 min/time, which can be adjusted according to the patient's tolerance; 4. For sit‐up training, the patient reciprocates on the chair to complete the standing and sitting movements, the initial height is 50 cm, and each training is 150‐200 times; 5. Single‐leg weight training, balance training, up and down stairs training, accompanied by medical staff throughout the whole process, 2 times/d, according to the actual situation, go to the outdoor for 1~2 times of walking training every day, the distance is 1000 m. The training time of the whole process was 5 days per week, 2 months of continuous treatment, 2 times/d, 2 h/time." (2) Conventional exercise therapy "conventional exercise therapy: Implementing lower extremity physiotherapy training based on neuro developmental techniques. The training content mainly includes stasis balance ability training, trunk training, joint training, etc. According to the actual situation of the patient, the corresponding training is carried out step by step, from the initial passive movement to the patient's voluntary excessive movement; the glenoid is based on the patient's tolerance, adjust the training intensity reasonably; the training time of elm is 5 days per week, continuous treatment for 2 months, 2 times/d, 2 h/time. "
Outcomes	**Independence in ADL scales:** Barthel Index **Motor function scales:** Fugl‐Meyer assessment **Measures of balance:** NR **Measures of gait velocity:** 10‐metre walk test **Length of stay:** NR **Adverse events:** NR **Other measures:** NR **Time points when outcomes were assessed:** before and after treatment
Comparison	**Amount of physical rehabilitation in groups:** equivalent **Categorisation of comparison:** 3.(i) Comparison of different approaches (dose equivalent; with no other physical rehab)
Funding & conflicts of interest	**Funding statement:** not reported **Conflict of interest statement:** not reported
Synthesis	**Quantitative synthesis:**Analysis 7.7; Analysis 7.8; Analysis 7.10
Notes	This study was published in Chinese and translated to English using Google Translate.

**Table:** Ghasemi 2018

***Study characteristics***	
Methods	**Study aim:** to determine the impact of functional stretching exercises on functional outcomes in stroke patients **Design:** parallel‐group RCT **Country:** Iran, Islamic Rep. **Study recruitment and setting:** see Table **Involvement of patients/public/professionals/others:** no
Participants	**Study population (number randomised):** 30 **Inclusion criteria:** Stroke diagnosed by experienced neurologist Presence of spasticity in the medial gastrocnemius muscle based on modified MAS Able to follow instructions based on MMSE (Persian version) Able to go to rehabilitation centre three times/week for four weeks Able to walk unaccompanied for 10m with or without assistive device Able to do exercises **Exclusion criteria:** Deformity or contracture in ankle joint Pain in lower limb Participating in special training program Using anti‐spastic drugs or Botox injections Other CNS lesions e.g. Parkinson's or multiple sclerosis **Participants:** detailed in Table **PROGRESS‐plus:** summarised in Table
Interventions	(1) Functional stretching training "The experimental group had a warm‐up protocol, including static stretching exercises before the functional exercises. These static stretching exercises were done in the standing position with the use of a wedge. Overall, 2 stretching positions were assumed alternatively by the participants and a total of 5 stretches (30 s for each position) were done (resting duration, 30 s between the stretches) (Guissard and Duchateau, 2004). The participants were asked to perform the stretches at maximum dorsiflexion angle. The following exercises were performed: (1) The patient was positioned in front of the parallel bar, holding the bars with his/her hand. The affected leg was placed on the wedge and the patient moved forward slowly while the knee was held bent (Fig. 2). (2) With the same position as earlier, the normal leg was placed on the wedge and the patient moved forward slowly while the knee was held bent. In this position, the therapist forced the affected knee to a more extended position (Fig. 3). For functional stretching exercises, we selected three functional challenges (Carr and Shepherd, 2011; Dean et al., 2000): (1) sit‐to‐stand from chair with an appropriate height (Fig. 4); (2) forward and backward slope walking (Fig. 5); and (3) climbing up from fivestairs (Fig. 6). The ability of the patients determined the duration of each training program (Lederman, 2014)." (2) Control ‐ routine physical therapy "Subjects in control group received their routine physical therapy program including electrical stimulation, gait training exercises and passive stretching exercises for lower extremity."
Outcomes	**Independence in ADL scales:** NR **Motor function scales:** NR **Measures of balance:** NR **Measures of gait velocity:** 10‐m walk test, Timed Up and Go test **Length of stay:** NR **Adverse events:** NR **Other measures:** muscle tone (MMAS), passive range of movement, subjective experience of impact of ankle spasticity, electrophysiology measures **Time points when outcomes were assessed:** baseline, post‐training, follow‐up (2 months after end of programme)
Comparison	**Amount of physical rehabilitation in groups:** unclear/not stated, but assumed that the functional stretching is provided in addition to routine physical therapy, and therefore dose differs. **Categorisation of comparison:** 5. Additional therapy + usual therapy versus usual therapy (dose differs)
Funding & conflicts of interest	**Funding statement:** funded and ethically approved by Shahid Beheshti. University of Medical Sciences as part of a Physical therapy PhD thesis. The sponsor has no role in data collection, data analysis, and drafting the manuscript. **Conflict of interest statement:** none
Synthesis	**Qualitative synthesis:** Results not presented in a format suitable for quantitative analysis.
Notes	

**Table:** Gong Y 2009

***Study characteristics***	
Methods	**Study aim:** to compare the effect of unilateral and bilateral limb function training on the rehabilitation effect of stroke hemiplegia **Design:** parallel‐group RCT **Country:** China **Study recruitment and setting:** see Table **Involvement of patients/public/professionals/others:** no
Participants	**Study population (number randomised):** 80 **Inclusion criteria:** Met the diagnostic criteria established by the Fourth National Conference on Cerebrovascular Diseases Stroke confirmed by CT or MRI Stroke onset within first week Hemiplegia on one side Fully conscious **Exclusion criteria:** Not reported **Participants:** detailed in Table **PROGRESS‐plus:** summarised in Table
Interventions	Both groups received conventional rehab training/conventional neurology treatment. Rehabilitation training was conducted by the same therapist. Training started on day 7 following hospitalisation. Early‐stage rehabilitation focussed mainly on passive joint activities. Later in the recovery period, training focussed on sitting, standing, walking and ADL tasks. In the maintenance period, training focus was on enhancing physical strength and maximising the function of the residual limb. (1) Bilateral training Bilateral training ‐ treat both limbs. Non‐paretic limb ‐ focus on active exercise. Upper and lower limb training. Bilateral limb training is mainly based on PNF bilateral mode training including symmetrical or asymmetrical mode, symmetrical cross‐over or asymmetrical cross‐over mode. The non‐paretic limb is mainly engaged in voluntary activities, including lower limb straight leg raising, straight leg abduction and adduction, single leg flexion, knee flexion, ankle dorsiflexion, and knee flexion. Upper limb activities included finger flexion and extension, arm internal and external rotation, arm flexion and extension. (2) Unilateral training Control group ‐ functional training for paretic limb only
Outcomes	**Independence in ADL scales:** Barthel Index **Motor function scales:** NR **Measures of balance:** NR **Measures of gait velocity:** NR **Length of stay:** NR **Adverse events:** NR **Other measures:** Brunnstrom **Time points when outcomes were assessed:** before and after treatment
Comparison	**Amount of physical rehabilitation in groups:** equivalent **Categorisation of comparison:** 3.(i) Comparison of different approaches (dose equivalent; with no other physical rehab)
Funding & conflicts of interest	**Funding statement:** not reported **Conflict of interest statement:** not reported
Synthesis	**Qualitative synthesis:** Results presented in a format not suitable for quantitative analysis.
Notes	This study was published in Chinese and relevant sections translated into English by our author team (PLC).

**Table:** Green 2002

***Study characteristics***	
Methods	**Study aim:** "to assess the effectiveness of this treatment in patients whohad mobility problems 1 year after stroke" **Design:** parallel‐group RCT **Country:** UK **Study recruitment and setting:** see Table **Involvement of patients/public/professionals/others:** no
Participants	**Study population (number randomised):** 170 **Inclusion criteria:** Aged 50 years+ Stroke longer than one year previously Persisting mobility problems **Exclusion criteria:** Non‐stroke mobility problem Dementia Severe co‐morbidity Bed bound Physiotherapy in previous 6 months **Participants:** detailed in Table **PROGRESS‐plus:** summarised in Table
Interventions	(1) Home‐based rehabilitation (n = 85) "Physiotherapy treatment was done by an established community physiotherapy service (13 staff) as part of their usual work" Community physiotherapists assessed using a ‘problem‐solving approach' and administered interventions according to the problem identified. Physiotherapy interventions included: "gait re‐education, exercise therapy, balance re‐education, counselling and advice, neurological mobilisations, functional exercises, posture re‐education, other interventions" Length of intervention period: maximum of 13 weeks Number of sessions and length of individual sessions: minimum of 3 contacts per participant ("median number of treatments per patient was three (IQR 2 to 7, range 0 to 22) and the mean duration of every treatment was 44 min") Intervention provider: physiotherapists (2) No physical rehabilitation (n = 85) No treatment Length of intervention period: no intervention Number of sessions and length of individual sessions: no intervention Intervention provider: no intervention
Outcomes	**Independence in ADL scales:** Barthel Index **Motor function scales:** Rivermead Mobility Index, Frenchay Activities Index **Measures of balance:** NR **Measures of gait velocity:** gait speed **Length of stay:** NR **Adverse events:** NR **Other measures:** Hospital Anxiety and Depression Scale; Depression, General Health Questionnaire 28; number of participants who had falls **Time points when outcomes were assessed:** "After baseline assessment, follow‐up assessments were 3‐monthly until 9 months"
Comparison	**Amount of physical rehabilitation in groups:** differs **Categorisation of comparison:** physical rehabilitation versus no or limited physical rehabilitation (dose differs)
Funding & conflicts of interest	**Funding statement:** "Stroke Association for funding the study, and The Hospitals SavingsAssociation for the award of a grant enabling John Green to study for a higher degree" **Conflict of interest statement:** "none declared"
Synthesis	**Quantitative synthesis:**Analysis 1.1; Analysis 1.2; Analysis 1.4; Analysis 2.1; Analysis 2.2; Analysis 2.4
Notes	

**Table:** Guan 2017

***Study characteristics***	
Methods	**Study aim:** to investigate the effect of motor relearning training on the motor function of stroke patients with acute hemiplegia **Design:** parallel‐group RCT **Country:** China **Study recruitment and setting:** see Table **Involvement of patients/public/professionals/others:** no
Participants	**Study population (number randomised):** 64 **Inclusion criteria:** Diagnosis of ischaemic stroke confirmed to the standard set by the Fourth National Cerebrovascular Disease Academic Conference in 1995, and confirmed by head MRI examination Acute hemiplegia Aged 40 to 80 years old Disease duration ≤ 14 days Informed consent **Exclusion criteria:** Disease duration > 14 days Previous history of hemiplegia History of transient ischaemic attack (TIA) or reversible ischaemic neurological deficit Severe cognitive function, speech function, emotional and psychological disorders and/or unable to correctly execute motor commands Lack of awareness of active rehabilitation training, poor compliance and enthusiasm Poor physical fitness cannot tolerate rehabilitation training **Participants:** detailed in Table **PROGRESS‐plus:** summarised in Table
Interventions	(1) Motor relearning In addition to conventional treatment: "According to the main clinical manifestations of hemiplegia patients in the acute stage of stroke (decreased muscle tone, loss of voluntary movements, mobility impairment and balance dysfunction), the training steps are designed and divided into 4 steps, namely the analysis of missing motor components; the missing motor components practice; functional task practice; refinement of skills, transfer of practice to real‐life situations. The training content includes: (1) Trunk movement training. 1. Induction training of trunk, waist and abdomen, and waist and back muscles. 2. Turn over and transfer training from supine position to unaffected lateral position. 3. Transfer training from lying position on the healthy side to sitting up at the bedside. (2) Reaching enough objects and operation training. 1. The shoulder girdle is assisted by the rehabilitation therapist to perform up and down, left and right, and circular rotation activities. 2. Induce the upper limb to reach the object (support the upper limb on the affected side to bend forward 90°, move within a certain range with the hand of the rehabilitation therapist, or touch the forehead, pillow, bed surface, etc.). (3) Walking preparation training. 1. Soft tissue stretching training such as gastrocnemius and rectus femoris. 2. Simple active training of lower limb muscles (such as active hip extension and knee flexion in prone position). (4) Stand up and sit down training. 1. Hip lift off training in high sitting position. 2. Bed and chair transfer training. (5) Seated training. 1. Adjust the posture when the center of gravity of the sitting position is shifted. 2. When sitting, touch objects in front, front and bottom, left and right. 3. Soleus soft tissue stretching training. 4. Rear‐foot training. The above training should be carried out under the guidance of a rehabilitation therapist, 45 minutes a day, 5 days a week, and continuous training for 2 weeks. Rehabilitation therapists need to make full use of skills such as demonstration, instruction, and audio‐visual feedback during training, so that patients can successfully form the three stages of cognition, association and spontaneous motor skills; With the help of family members, training can be performed twice a day for 30 minutes each time." (2) Conventional treatment "After admission, all patients were treated with conventional drugs for stroke, including drugs to improve cerebral circulation, nutritional nerve drugs, antiplatelet drugs and lipid‐lowering drugs. Routine rehabilitation training, including nerve stimulation techniques and physical therapy, was started 24 to 48 hours after clinical symptoms no longer progressed and vital signs stabilized. (1) Nerve communication technology: Based on neurodevelopmental theory, active training is carried out under the guidance of rehabilitation therapists to promote normal movement patterns, inhibit and avoid abnormal movement patterns, including good limb placement, passive joint assisted movement, Bridge sports, etc. (2) Physical therapy: mainly includes neuromuscular electrical stimulation, circulatory air pressure therapy, ultrashort wave therapy, acupuncture and moxibustion therapy, etc."
Outcomes	**Independence in ADL scales:** Barthel Index **Motor function scales:** Rivermead Mobility Index, Fugl‐Meyer ‐ balance subscale **Measures of balance:** NR **Measures of gait velocity:** NR **Length of stay:** NR **Adverse events:** NR **Other measures:** NR **Time points when outcomes were assessed:** before and after 2 weeks of training
Comparison	**Amount of physical rehabilitation in groups:** differs **Categorisation of comparison:** 5. Additional therapy + usual therapy versus usual therapy (dose differs)
Funding & conflicts of interest	**Funding statement:** not reported **Conflict of interest statement:** not reported
Synthesis	**Quantitative synthesis:**Analysis 5.1; Analysis 5.2
Notes	Paper written in Chinese and translated using Google Translate.

**Table:** Guo L 2012

***Study characteristics***	
Methods	**Study aim:** to observe the curative effect of task‐oriented training home rehabilitation training in the treatment of stroke patients with hemiplegia. **Design:** parallel‐group RCT **Country:** China **Study recruitment and setting:** see Table **Involvement of patients/public/professionals/others:** no
Participants	**Study population (number randomised):** 74 **Inclusion criteria:** Patients in the convalescent period 1.5 to 3 months post‐stroke at time of discharge Main lesion was cerebral apoplexy at the blood supply part of the internal carotid artery system Aged 35 to 70 years old Unilateral hemiplegia Conscious Able to follow instructions and take part in exercise training Provided informed consent (patient and family) **Exclusion criteria:** Severe depression Cognitive impairment; Severe heart, lung, liver organ complications No family support available or family unable to take part in auxiliary training Living too far away or unable to attend regular follow‐up appointments **Participants:** detailed in Table **PROGRESS‐plus:** summarised in Table
Interventions	Both groups were given routine pharmacological therapy (antihypertensive, lipid‐lowering, anti‐arteriosclerosis, anti‐depression, and muscle tension‐reducing drugs). (1) Rehabilitation Frequent, interval guided family rehabilitation training. Before discharge from the hospital, the rehabilitation therapist in charge provides guidance on recent family rehabilitation training to the patients and their families in the family rehabilitation group. Follow‐up visits are scheduled at the hospital every 2 to 4 weeks. During this appointment, the therapist will update the family practice plan based on the patient's functional level and guide the patient and their family members. The plan includes details about the number of times completed, the weight of the load, precautions. Family rehabilitation training mainly involves task‐oriented training and muscle strength training, upper and lower limb, balance and ADL practice. Daily practice 30 to 60 minutes, for 6 months. (2) Standard care The control group received general family exercise following discharge. For example, practice of usual activities such as sitting, standing, walking, assisted by the family members. Request them to do self‐guided practice, but not guided by therapist. 3 and 6 month follow‐up.
Outcomes	**Independence in ADL scales:** Barthel Index **Motor function scales:** Fugl‐Meyer assessment **Measures of balance:** NR **Measures of gait velocity:** NR **Length of stay:** NR **Adverse events:** NR **Other measures:** NR **Time points when outcomes were assessed:** before and after 6 months of rehabilitation
Comparison	**Amount of physical rehabilitation in groups:** differs **Categorisation of comparison:** 1. Physical rehabilitation versus no or limited physical rehabilitation (dose differs) (limited = < 50% of dose)
Funding & conflicts of interest	**Funding statement:** not reported **Conflict of interest statement:** not reported
Synthesis	**Quantitative synthesis:**Analysis 1.1; Analysis 1.2
Notes	This study was published in Chinese and relevant sections translated into English by our author team (PLC).

**Table:** Guo L 2013

***Study characteristics***	
Methods	**Study aim:** to investigate the effect of task‐oriented training combined with muscle strength training on the motor function of hemiplegia patients with stroke sequelae **Design:** parallel‐group RCT **Country:** China **Study recruitment and setting:** see Table **Involvement of patients/public/professionals/others:** no
Participants	**Study population (number randomised):** 42 **Inclusion criteria:** Aged 35 to 65 years old Movement disorder of one limb Stroke onset 6 to 24 months Main lesion was stroke at the blood supply of the internal carotid artery system **Exclusion criteria:** Severe depression Severe hearing loss or cognitive impairment Severe spasticity of hemiplegic limb(s) (Ashworth spasticity grade 3‐4) or pain Heart, lung, liver, kidney complications or complications with other important organs Resistant hypertension Unable to adhere to treatment **Participants:** detailed in Table **PROGRESS‐plus:** summarised in Table
Interventions	(1) Task‐oriented training "The treatment group was given exercise therapy combining task‐oriented training and muscle strength training. Upper limb function training: 1. The patient is in a supine position, the therapist assists the upper limb of the patient, and the patient is required to touch the contralateral shoulder, mouth, forehead, etc. with the affected hand, and the upper limb is straightened and raised in all directions to reach the therapist's hand; 2. The patient sits, the upper limb is placed on the desktop, the patient is required to push the building blocks, plastic cups and other items with the back of the hand, the forearm, and the thumb in all directions to reach the set target point; 4. The task of reaching the object in the sitting position: assist the patient to lift the upper limb forward, upward, and sideways. (4) Moving objects in a sitting position: the affected hand grasps the objects and places them from one place to another. During the training process, gradually increase the weight of pushing and grasping objects and the distance to be moved to increase the muscle strength of shoulder flexors, abductors and external rotators, elbow extensors, wrist and finger extensors. During the training, attention should be paid to correcting the patient's excessive shoulder shrug and trunk tilt to compensate for insufficient shoulder abduction or forward flexion. Lower extremity functional training: patients in supine position, perform double‐bridge, single‐bridge, dynamic bridge exercises, lower limb abduction, adduction, straight leg raising, lateral knee flexion, prone knee flexion and other exercises. Target points are set for each movement, and the patient's attention during the movement is required to focus on the target, not the lower extremity in motion. For example, during the bridge exercise, the therapist places the hands 5‐15cm above the patient's abdomen, and asks the patient to raise the pelvis to the point where the abdomen touches the therapist's palm and controls it for more than 3‐5 seconds. When the patient can control the hip extension, ask the patient to move the pelvis to both sides to contact the hands of the therapist located 5‐10cm on both sides of the hip to promote the pelvic motor control ability. During the training, gradually increase the distance between the target and the starting position of the lower limbs and the time required for the patient to control, and the calf can be tied with sandbags or applied with elastic bands to provide resistance for exercise, focusing on increasing the hip flexors, hip extensors, knee flexors, and knee extensors. Muscle and tibialis anterior muscle strength of the calf. Body position change and balance training: 1. Lie on your back, hold your head with both hands, lift your head and feet off the bed surface separately or at the same time, to strengthen the strength of trunk muscles; 2. From supine position to bedside sit‐up training: training from supine to healthy side lying position 3. Sitting posture balance training: sitting forward trunk leaning, trunk lateral bending, sitting forward, side, back, and top movement, picking up objects, etc. Gradually increase the distance that the hand can reach, increase the weight and volume of the object, participate in activities with both upper limbs at the same time, and train time‐limited activities such as catching or throwing a ball; 4. Stand up and sit training: strengthen the training of the trunk in front of the hips, tilt, relative movement of trunk, hip, knee and ankle during standing and sitting; 5. Station balance training: standing and sitting training, low bench sit‐up, squatting training; the affected foot is in front and the healthy foot is in the back. Standing, stepping on the pedals alternately with both feet, standing to reach objects in all directions, throwing and catching the ball, etc. The training instructs the patient to transfer each motor skill learned to activities of daily living." (2) Conventional rehabilitation "The control group adopted the facilitation technique. Including: upper limb Bobath handshake self‐help passive exercise and power‐assisted exercise; bridge exercise; Rood excitatory and inhibitory stimulation application; reflex inhibition mode and key point control; induced shoulder, elbow, wrist, hip, knee separation exercise; bed rollover training; Recumbent sit‐up training, sitting balance training; standing and standing balance, walking training, etc" "Both groups of patients were given conventional drugs for the treatment of cerebrovascular disease and the prevention of recurrence of cerebrovascular disease, and rehabilitation therapy was given for hemiplegia." "The two groups were treated twice a day, 40‐60 minutes each time, 5 days a week, for 6 consecutive weeks. "
Outcomes	**Independence in ADL scales:** Barthel Index **Motor function scales:** Fugl Meyer assessment **Measures of balance:** NR **Measures of gait velocity:** NR **Length of stay:** NR **Adverse events:** NR **Other measures:** Elderly Mobility Scale **Time points when outcomes were assessed:** before and after 6 weeks of treatment
Comparison	**Amount of physical rehabilitation in groups:** equivalent **Categorisation of comparison:** 3.(i) Comparison of different approaches (dose equivalent; with no other physical rehab)
Funding & conflicts of interest	**Funding statement:** not reported **Conflict of interest statement:** not reported
Synthesis	**Quantitative synthesis:**Analysis 7.1; Analysis 7.2
Notes	This paper was published in Chinese and translated to English using Google Translate.

**Table:** Guo Z 2015

***Study characteristics***	
Methods	**Study aim:** to evaluate the effect of special muscle strength exercises and lower limb balance training on the recovery of lower limb function in patients with post‐stroke hemiplegia **Design:** parallel‐group RCT **Country:** China **Study recruitment and setting:** see Table **Involvement of patients/public/professionals/others:** no
Participants	**Study population (number randomised):** 80 **Inclusion criteria:** Met the diagnostic criteria for stroke according to the 4th National Academic Conference on Cerebrovascular Diseases Confirmed by CT or MRI **Exclusion criteria:** Large cerebral infarction Unclear consciousness Severe liver and kidney complications Malignant tumours Poor compliance or unable to complete the whole course of treatment **Participants:** detailed in Table **PROGRESS‐plus:** summarised in Table
Interventions	(1) Physical rehabilitation "Physical rehabilitation training guided by the community health service center affiliated to the hospital. The training program is guided by specialized personnel. Lower extremity rehabilitation training mainly includes muscle strength and coordination function training of affected limbs. The details are as follows. (1) Limb flexion and extension training: In the first step, assist the patient to lie supine on the bed to repeatedly practice the flexion and extension of the legs, gradually transition to the sitting position according to the progress of muscle strength, and then gradually perform standing flexion and extension exercises with the assistance of medical staff. (2) Lateral step training: After assisting the patient to stand, the healthy limb first, step in a straight line, move laterally to the unaffected side, and the affected limb retracts the leg and follows the line. (3) Cross‐walking training: With the assistance of medical staff, patients practice cross‐footing training. (4) Squat training: With the help of fixed armrests, patients perform lower limb squatting and lifting exercises. (5) Weight‐bearing training: The patient is in an upright position, and with the help of the armrest, the unaffected limb performs a back‐and‐forth swing exercise to gradually exercise the weight‐bearing function of the affected limb. The above training follows the order of passive‐assistance‐active, with gradual transition, 2 times each time, 30min each time. The duration of treatment was 6 months. " (2) Standard care "the control group was given routine post‐stroke rehabilitation guidance on the basis of drug control after discharge"
Outcomes	**Independence in ADL scales:** Functional Independence Measure (FIM) **Motor function scales:** Fugl Meyer assessment **Measures of balance:** NR **Measures of gait velocity:** NRNR **Length of stay:** NR **Adverse events:** NR **Other measures:** isokinetic muscle strength **Time points when outcomes were assessed:** before and after treatment
Comparison	**Amount of physical rehabilitation in groups:** differs **Categorisation of comparison:** 1. Physical rehabilitation versus no or limited physical rehabilitation (dose differs) (limited = < 50% of dose)
Funding & conflicts of interest	**Funding statement:** not reported **Conflict of interest statement:** not reported
Synthesis	**Quantitative synthesis:**Analysis 1.1; Analysis 1.2
Notes	This study was published in Chinese and translated into English using Google Translate.

**Table:** Haral 2014

***Study characteristics***	
Methods	**Study aim:** to study the efficacy of sensorimotor integration approach along with conventional physiotherapy programme for improving balance and gait in chronic stroke patients **Design:** parallel‐group RCT **Country:** Iceland **Study recruitment and setting:** see Table **Involvement of patients/public/professionals/others:** no
Participants	**Study population (number randomised):** 30 **Inclusion criteria:** First episode of stroke, > 6 months onset ‐ 3 years Stability of neurological severity Absence of any cognitive impairment based on MMSE Able to stand and walk with/without an aid **Exclusion criteria:** Perceptual and visual impairment Deficits of somatic sensation involving the paretic limb Presence of neurological conditions e.g. neglect, hemianopsia and contraversive pushing syndrome Presence of orthopaedic disease involving lower limb Vestibular disorders **Participants:** detailed in Table **PROGRESS‐plus:** summarised in Table
Interventions	(1) Sensorimotor integration "Exercises were: ‐ standing (progression from eyes open to closed and floor to mat), including weight shifts and reach outs, tandem standing, and reachouts, one leg standing, standing with head turns, and eyes closed ‐ standing on inclined wedge (progression with eyes closed and head turns) ‐ step up from floor to mat, floor to wedge, and back ‐ walking on mat ‐ sitting on ball and weight shifts / reach outs; progresion with feet on mat, eyes closed, head turns, reach outs ‐ balance board ‐ scooter board" (2) Conventional training "Exercises were: ‐ strengthening of trunk and LL muscles ‐ weight shifts in different positions, e.g. standing, kneeling ‐ activities in standing, including standing with feet close together, tandem standing, standing reachouts, one leg standing, stepping, passing a ball, marching, perturbations ‐ activities in walking, including tandem walking, walking over/around obstacles, picking up objects, carrying weights, changing direction"
Outcomes	**Independence in ADL scales:** NR **Motor function scales:** NR **Measures of balance:** Berg balance **Measures of gait velocity:** 10‐m walk test **Length of stay:** NR **Adverse events:** NR **Other measures:** modified clinical test of sensory integration and balance, dynamic gait index **Time points when outcomes were assessed:** NR
Comparison	**Amount of physical rehabilitation in groups:** not clear/not stated, but assumed to be equivalent **Categorisation of comparison:** 3.(i) Comparison of different approaches (dose equivalent; with no other physical rehab)
Funding & conflicts of interest	**Funding statement:** none **Conflict of interest statement:** none
Synthesis	**Quantitative synthesis:**Analysis 7.9; Analysis 7.10
Notes	No group sizes reported. Analysis carried out using sizes of 15 for each group.

**Table:** Harjpal 2021

***Study characteristics***	
Methods	**Study aim:** "designed to study the effect of bilateral lower‐limb training over unilateral lower‐limb training on balance and walking in post‐stroke survivors" **Design:** parallel‐group RCT **Country:** India **Study recruitment and setting:** see Table **Involvement of patients/public/professionals/others:** no
Participants	**Study population:** 40 **Inclusion criteria:** Aged 40 to 65 years First stroke and in the sub‐acute phase of stroke with hemiparesis or hemiplegia Able to understand and follow instructions and able to provide consent **Exclusion criteria:** < 40 years and > 65 years Diagnosed with brainstem stroke or MCA stroke or history of TIA Any joint or muscular problem arising from condition other than stroke Unstable cardiovascular condition Diagnosed with the failure of vital organs, such as lung, heart, kidney and liver Registered in another clinical trial **Participants:** detailed in Table **PROGRESS‐plus:** summarised in Table
Interventions	(1). Lower limb bilateral training "This group got bilateral lower‐limb training that comprised a task‐oriented strategy for the damaged side, such as the MRP and PNF, as well as strengthening of the less affected side[20,21]. A physiotherapist prescribed strength training for 20 minutes on the side that was less affected, as well as 20 minutes of lower‐and upper‐limb exercises for the involved side five days a week for six weeks. The hip flexors, abductors and extensors, knee extensors, and ankle dorsiflexors were stengthened[12]. Each muscle was provided strength training for three minutes with a rest period of one minute in between each muscle group. Before training, each muscle group was tested for one repetition maximum(RM) using DeLorme’s boot. Post measurement, the strengthening was provided via a weight cuff tied over the ankle according to the strengthening regimen prescribed by DeLorme." (2). Lower limb training to the affected side only "For five days a week for a period of six weeks, the participants in this group underwent 20 minutes of lower limb training on the affected side only by a physiotherapist. The MRP and PNF (Figure2) were both part of it, task‐specific training and multi planar motions of the affected lower and upper extremities are included." NB: the bilateral training was delivered in addition to the training to the affected side.
Outcomes	**Independence in ADL scales:** NR **Motor function scales:** FMA‐lower extremity (stated as secondary outcome in protocol, but not in results paper) **Measures of balance:** Berg Balance Scale **Measures of gait velocity:** 10‐m walk test **Length of stay:** NR **Adverse events:** NR **Other measures:** functional reach test, one legged stance test or single leg stance test, Dynamic Gait Index, Brunnstrom recovery stages **Time points when outcomes were assessed:** baseline and immediately post‐intervention (6 weeks)
Comparison	**Amount of physical rehabilitation in groups:** differs **Categorisation of comparison:** 5. Additional therapy + usual therapy versus usual therapy (dose differs)
Funding & conflicts of interest	**Funding statement:** not reported **Conflict of interest statement:** "In compliance with the ICMJE uniform disclosure form, all authors declare the following : Payment/services info: All authors have declared that no financial support was received from any organization for the submitted work. Financial relationships: All authors have declared that they have no financial relationships at present or with in the previous three years with any organizations that might have an interest in the submitted work. Other relationships : All authors have declared that there are no other relationships or activities that could appear to have influenced the submitted work"
Synthesis	**Quantitative synthesis:**Analysis 5.3; Analysis 5.4
Notes	

**Table:** Hendrey 2018

***Study characteristics***	
Methods	**Study aim:** to establish the feasibility and effectiveness of a 6‐week ballistic strength training protocol in people with stroke **Design:** parallel‐group RCT (mixed methods) **Country:** Australia **Study recruitment and setting:** see Table **Involvement of patients/public/professionals/others:** unclear
Participants	**Study population (number randomised):** 30 **Inclusion criteria:** Primary diagnosis first ever stroke Presence of lower limb weakness FAC score ≥ 3 i.e. able to walk with continuous/intermittent light touch to support balance/co‐ordination Able to walk 14m+ with/without aids or orthoses **Exclusion criteria:** Unable to provide consent Diagnosed with another CNS disorder that affects mobility Medically unstable Recent orthopaedic trauma and/or osteoarthritis (limiting participation in physical exercise) Unwilling to continue to take part if they were discharged home prior to the end of the programme **Participants:** detailed in Table **PROGRESS‐plus:** summarised in Table
Interventions	(1) Ballistic strength training Ballistic strength training protocol reported in Appendix 2 of paper. Exercises included: 1. Jump squats on the leg sled (resistance equipment) 2. Single leg hopping on the leg sled 3. Jogging on the leg sled 4. Mini trampoline 5. High speed hip flexion (using weights and pulley if required) 6. Bounding (using step if required) 3x per week for 6 weeks. Progressed. (2) Control ‐ usual care Standard therapy protocol reported in Appendix 1 of paper. Exercises included: 1. Gait practice 2. Dynamic balance (wobble board, standing, walking exercises) 3. Squats 4. Calf raises 5. Step ups 6. Sit‐to‐stand 7. Hip extension strength 8. Stretching 9. Static balance 10. Cardiovascular fitness 3x per week for 6 weeks. Personalised and progressed "Both groups received three 45‐minute sessions of training (including rest breaks) each week for 6 weeks while continuing to participate in their existing rehabilitation program."
Outcomes	**Independence in ADL scales:** NR **Motor function scales:** NR **Measures of balance:** NR **Measures of gait velocity:** 10‐m walk test, timed up and go test **Length of stay:** NR **Adverse events:** safety **Other measures:** Functional Ambulation Categories, muscle power, muscle strength, health related quality of life **Time points when outcomes were assessed:** pre‐ and post‐training
Comparison	**Amount of physical rehabilitation in groups:** equivalent **Categorisation of comparison:** 3.(i) Comparison of different approaches (dose equivalent; with no other physical rehab)
Funding & conflicts of interest	**Funding statement:** Supported by an Australian Government Research Training Program Scholarship and research grants received by Alfred Health and the Australian Catholic University. Ross A. Clark is supported by a National Health and Medical Research Council RD Wright Biomedical Fellowship. **Conflict of interest statement:** none
Synthesis	**Quantitative synthesis:**Analysis 7.4; Analysis 7.6
Notes	

**Table:** Holmgren 2006

***Study characteristics***	
Methods	**Study aim:** "to evaluate a high intensive exercise program in stroke subjects with risk of falls regarding balance, ADL, falls efficacy, number of falls and lifestyle activities" **Design:** parallel‐group RCT **Country:** Sweden **Study recruitment and setting:** see Table **Involvement of patients/public/professionals/others:** no
Participants	**Study population (number randomised):** 34 **Inclusion criteria:** First ever or recurrent ischaemic or haemorrhagic stroke 3 to 6 months before enrolment Age ≥ 55 years Ability to walk 10 metres with or without a walking device Ability to understand and comply with instructions in Swedish At risk of fall (at the time of enrolment) **Exclusion criteria:** Ability to walk outdoors independently Without personal assistance or walking device Severe aphasia or severe vision or hearing impairment Medical condition that a physician determined was inconsistent with study participation (e.g. cancer or severe congestive heart failure with expected short remaining life expectancy, recurrent stroke within three months before study start) Lived farther than 100 km away from the training facilities ("this was considered as too far away, since it would take too much time and energy away from the individual each day of the intervention") **Participants:** detailed in Table **PROGRESS‐plus:** summarised in Table
Interventions	(1) Exercise group (n = 15) "The focus of the exercise was on physical activity and functional performance (based on the HIFE program), to improve the subjects' lower‐limb strength, balance and gait ability. The program includes lower‐limb strength (e.g. chair stand) and balance exercises (e.g. weight shifting outside support surface), standing (e.g. knee bend) and walking (e.g. obstacle crossing course)" "The two daily training sessions were divided between exercise according to the HIFE program and implementing of the same in to everyday life activities, e.g. walking outdoors or sweep the yard. All exercises were performed at a high intensity, if possible, for each subject. 'High intensity' was defined as (i) strength exercises comprising at least two sets of exercises with 8 – 12 repetitions (maximum), (ii) the balance exercises were close to the subjects’ balance maximum, and (iii) the subject did not rest more than necessary, all according to the HIFE program" "In addition, there was a 1‐hour educational discussion session, per week. These discussions were about the increased risks of complications after stroke, such as falls. During the last week of intervention, an individualized home‐based exercise program was designed by the physiotherapist. This home exercise program was part of the intervention program and consisted of a maximum of three different exercises that were based on the exercises performed during the 5‐week intervention. It was easy to adjust the intensity of all the exercises so that they could be modified as the subject progressed. The instructions were to perform this home‐based exercise program three times a week at least until the 3‐month follow‐up" Length of intervention period: 5 weeks. In addition, participants were instructed to continue to perform the home‐based exercise programme 3 times a week at least until the 3‐month follow‐up. Number of sessions and length of individual sessions: "the exercise sessions, which lasted approximately 45 min each, were performed six times each week, a total of 30 exercise sessions over 5 weeks. Subjects also received a 45‐min session per day of activities related to real‐life situations. In addition, one day each week there was a 1‐hour educational discussion session, a total of five educational sessions over 5 weeks" Intervention provider: physiotherapist and occupational therapist (2) Control group (n = 19) Education only "The group discussions were about communication difficulties, fatigue, depressive symptoms, mood swings, personality changes and dysphagia, all more or less hidden dysfunctions after stroke and how to cope with these difficulties. There was no special focus on the risks of falling in these discussions" Length of intervention period: 5 weeks Number of sessions and length of individual sessions: "met once a week for 1 hour of educational session" Intervention provider: occupational therapist
Outcomes	**Independence in ADL scales:** Barthel Index **Motor function scales:** NR **Measures of balance:** Berg Balance Scale **Measures of gait velocity:** NR **Length of stay:** NR **Adverse events:** NR **Other measures:** Frenchay Activities Index **Time points when outcomes were assessed:** "Assessments were done at baseline, post‐intervention, 3‐ and 6‐month follow‐up"
Comparison	**Amount of physical rehabilitation in groups:** differs **Categorisation of comparison:** 1. Physical rehabilitation versus no or limited physical rehabilitation (dose differs) (limited = < 50% of dose)
Funding & conflicts of interest	**Funding statement:** "supported by grants from Vårdal‐institutet, the Swedish Institute for Health Sciences, the Swedish Stroke Foundation, the Swedish Heartand Lung Foundation, the Northern Swedish Stroke fund, the“Spjutspetsprojekt”at the County of Väster‐botten, the Medical Faculty of Umeå University, Umeå University Hospital and the Erik and Anne‐Marie Detlof Foundation at UmeåUniversity." **Conflict of interest statement:** "authors report no conflicts of interest"
Synthesis	**Quantitative synthesis:**Analysis 1.1; Analysis 1.3; Analysis 2.1; Analysis 2.3
Notes	SD computed from confidence intervals and P value.

**Table:** Hong Cuicui 2016

***Study characteristics***	
Methods	**Study aim:** to investigate the effect of optimising motor skills on the recovery of walking function after stroke **Design:** parallel‐group RCT **Country:** China **Study recruitment and setting:** see Table **Involvement of patients/public/professionals/others:** no
Participants	**Study population (number randomised):** 136 **Inclusion criteria:** Aged 53 to 71 years Stroke onset 7 to 20 days First cerebral infarction or cerebral haemorrhage Stroke confirmed by CT or MRI Met the diagnostic criteria of the Fourth National Cerebrovascular Disease Conference Conscious Co‐operative No serious speech or cognitive impairment MMSE > 15 points No other neurological disorder No previous history of mental health disorders Motivated Good family support **Exclusion criteria:** Not reported **Participants:** detailed in Table **PROGRESS‐plus:** summarised in Table
Interventions	Both groups received the usual medical treatment and nursing care. Both received conventional rehabilitation training. Rehabilitation started once the participant was medically stable (i.e. between 3 and 7 days for those with cerebral infarction and 7 and 14 days for those with a cerebral haemorrhage. Therapy was delivered 1:1 by professionally trained therapists. (1) Additional therapy Addition of optimal training was based on Bobath principles and PNF principles. Training was progressive and involved repeating training and strengthening. Training started with trunk muscle training, body position transfer, balance training, standing balance, composition analysis (analysing how the person moves during standing balance), gait analysis and incremental gait training. Training was delivered in 2 sessions/day, session duration of 45 mins, delivered 5 days/week over 6 weeks. Family members supported additional training outside of this treatment. (2) Conventional rehabilitation This involved: *Standing alignment:* assessing the ability of the active activities of the limbs, trunk and head when they perform different degrees of exercise. *Training of cardiac standing balance:* when performing standing balance in the first few days of training. Weight‐bearing training on both legs including joint alignment training, knee flexion prevention training, quadriceps contraction training, training posture adjustment training when the centre of gravity is shifted and increasing complexity. *Training of walking components:* joint alignment training, knee joint flexion prevention training, quadriceps contraction training, training posture adjustment when the centre of gravity is shifted. *Increasing walking complexity training:* exercises take place in different directions e.g., stepping to catch a ball, picking up different objects with both hands and one hand, increasing the patient's balance by walking, changing direction, stepping over objects, transferring training to everyday life activities. Training was delivered in 2 sessions/day, session duration of 45 mins, delivered 5 days/week over 6 weeks.
Outcomes	**Independence in ADL scales:** NR **Motor function scales:** Fugl‐Meyer assessment **Measures of balance:** NR **Measures of gait velocity:** NR **Length of stay:** NR **Adverse events:** NR **Other measures:** functional ambulation categories (Holden) **Time points when outcomes were assessed:** before and after training
Comparison	**Amount of physical rehabilitation in groups:** equivalent **Categorisation of comparison:** 5. Additional therapy + usual therapy versus usual therapy (dose differs)
Funding & conflicts of interest	**Funding statement:** not reported **Conflict of interest statement:** not reported
Synthesis	**Quantitative synthesis:**Analysis 5.2
Notes	This study was published in Chinese and relevant sections translated into English by our author team (PLC).

**Table:** Hong Hye Jin 2012

***Study characteristics***	
Methods	**Study aim:** "to explore limb function recovery efforts of three‐stage rehabilitation training on stroke patients with hemiplegia" **Design:** parallel‐group RCT **Country:** China **Study recruitment and setting:** see Table **Involvement of patients/public/professionals/others:** no
Participants	**Study population (number randomised):** 80 **Inclusion criteria:** Stroke confirmed by CT or MRI Stroke diagnosis met the criteria reported in the 4th National Guidelines **Exclusion criteria:** None reported **Participants:** detailed in Table **PROGRESS‐plus:** summarised in Table
Interventions	Both groups received conventional neurology treatment and rehabilitation did not start until stroke survivors were medically stable. "(1) Three‐stage rehabilitation Standardised three‐stage rehabilitation provided at different time periods. Stage I: Early stage started 1‐7 days after stroke onset, when stroke survivors were stable. They were encouraged to work towards a new goal for paretic limbs or limbs that did not have complete paralysis. Nurses encouraged mental imagery and motivation to get the stroke survivors to contract their paralysed muscles and then they would proceed with the assistive exercises before starting any active exercises. Nurses would help with passive mobilisation of all joints; 2‐4 sessions per day, with each activity involving 10‐15 repetition/minute, gradually increasing the intensity, amplitude and time spent on each activity. They aimed to passive to assisted active, to active control. Stroke survivors were encouraged to do mobilisation bridging, supporting the non‐paretic limb to do all the training and positioning. The goal was to improve the self‐care of the stroke survivor. Two components (a) timely changes in position, changing the stroke survivor every two hours depending on whether they are lying on paretic or non‐paretic side. Positioning more frequently (1/hour) if paretic side is facing downward, supporting the limbs with pillow/cushions to prevent compression. During these position changes, 25% alcohol was used to massaged compressed areas; (b) massage was conducted 2 sessions per day, 20 minutes/session. Paretic limb was massaged, focusing on acupressure points. Massage strokes were light and heavy; shallow and deep, and slow and quick. Massage was delivered gently with tempo/rhythm. Stage II: Middle stage Aim of the rehabilitation delivered in this stage is to facilitate normal posture and exercise movement. Good positioning is essential to reduce the onset of spasticity. In this stage they also focus on balance training, hand on the back of a chair to support balance, practice sit‐to‐stand as well as marching on the spot, heel lifts and exercises to improve core muscles and help prepare for gait training. Gait training begins once lower limbs have sufficient energy and balance ability. The goal is to try and improve functional recovery of the lower limbs as much as possible. Training involves 1‐2 sessions/day; session duration: 45 minutes delivered 4‐5 days/week. Nursing components comprise the following: getting into different positions balance training ‐ sitting‐balance training progressing to stand balance training. gait training ‐ walking in the wards, walking to the lift and then more independent walking. undergo some occupational therapy e.g., kicking a ball, playing tumbling tower game (Jenga) Stage III: Late stage During this stage they continue to work on gait training and refining movements to improve co‐ordination and strengthening. They use more challenging equipment e.g., cycling and work on activity of daily living. (2) Conventional rehabilitation Traditional rehabilitation nursing care. Nurses would deliver positioning and massage the paretic limb. Passive movement therapy started with larger joints, moving onto smaller joints, and moving the joints in different directions. Rehabilitation was individualised for each patient depending on tolerance. Two sessions per day, each session would last 30 minutes, and this was delivered for 30 days."
Outcomes	**Independence in ADL scales:** NR **Motor function scales:** NR **Measures of balance:** NR **Measures of gait velocity:** NR **Length of stay:** NR **Adverse events:** NR **Other measures:** muscle strengthening **Time points when outcomes were assessed:**
Comparison	**Amount of physical rehabilitation in groups:** differs **Categorisation of comparison:** 5. Additional therapy + usual therapy versus usual therapy (dose differs)
Funding & conflicts of interest	**Funding statement:** not reported **Conflict of interest statement:** not reported
Synthesis	**Qualitative synthesis:** no relevant outcomes assessed
Notes	This study was published in Chinese and relevant sections translated into English by our author team (PLC).

**Table:** Hoseinabadi 2013

***Study characteristics***	
Methods	**Study aim:** to determine the effects of physical therapy on balance, exaggerated muscle tonicity and quality of life in patients with hemiparesis **Design:** parallel‐group RCT (also described as "quasi‐experimental" study) **Country:** Iran, Islamic Rep. **Study recruitment and setting:** see Table **Involvement of patients/public/professionals/others:** no
Participants	**Study population (number randomised):** 24 **Inclusion criteria:** Stroke at least a year before the study Aged between 40 and 60 years Diagnosed with hemiparesis secondary to stroke Able to stand feet apart with open eyes for at least 30 seconds Able to understand instructions Able to change direction Not part of any out‐of‐study rehabilitation programme during the intervention **Exclusion criteria:** Not reported **Participants:** detailed in Table **PROGRESS‐plus:** summarised in Table
Interventions	(1) Physiotherapy "The experimental protocol training was conducted for 4 weeks; three one‐hour sessions every week. After 5 minutes of warming up through walking on a flat surface, the patients did the mobility and movement exercises of the hip joints, knees, ankles, and a series of general mobility exercises, balance exercises, strength training and flexor, extensor and abductor of hip, flexor and extensor of knee, repeating 10 times." (2) No treatment
Outcomes	**Independence in ADL scales:** Barthel Index **Motor function scales:** NR **Measures of balance:** Berg Balance Scale **Measures of gait velocity:** NR **Length of stay:** NR **Adverse events:** NR **Other measures:** Modified Ashworth Scale **Time points when outcomes were assessed:** after 4 weeks of treatment
Comparison	**Amount of physical rehabilitation in groups:** differs **Categorisation of comparison:** 1. Physical rehabilitation versus no or limited physical rehabilitation (dose differs) (limited = < 50% of dose)
Funding & conflicts of interest	**Funding statement:** not reported **Conflict of interest statement:** not reported
Synthesis	**Quantitative synthesis:**Analysis 1.1; Analysis 1.3
Notes	Quality of life results reported are assumed to be Barthel Activities of Daily Living (ADL) Index results.

**Table:** Hou 2006

***Study characteristics***	
Methods	**Study aim:** "to explore the effects of 3‐phase rehabilitation on functional outcome in patients with hemiplegia after stroke" **Design:** parallel‐group RCT **Country:** China **Study recruitment and setting:** see Table **Involvement of patients/public/professionals/others:** no
Participants	**Study population (number randomised):** 80 Using the 1995 evaluative standard revised by the Fourth [National] Academic Conference of Cerebral Vascular Disease Confirmation of a first ever stroke from CT or MRI scan Medically stable within 2 weeks of stroke onset GCS score of > 8 Deficits in motor function Aged between 40 and 80 years old **Exclusion criteria:** Active liver/kidney disease Paralysis of four limbs Deaf and/or mute Issues with co‐ordinating assessment or inaccessible homes **Participants:** detailed in Table **PROGRESS‐plus:** summarised in Table
Interventions	(1)Three‐stage rehabilitation (n = 40) "Both groups of patients received conventional clinical treatment and care. Rehabilitation group, besides conventional clinical treatment, also strictly followed 'fifteen' research topics 'cerebrovascular disease three level rehabilitation programme' in carrying out rehabilitation; control group did not perform any standard rehabilitation Rehabilitation group patients, on entering the group, immediately commenced level one rehabilitation programme (within neurology ward), comprising anti‐spasticity positioning on bed; breathing exercises; passive ranging exercises on limbs of affected side; use of neural stimulation technique (mainly Rood technique and Brunnstrom technique); active ranging exercises of limbs of non‐affected side; lying to sitting training; sitting‐balance training; ADL training on bed; neural network and functional electrical stimulation etc. Based on individual situation, selectively performed, 1 – 2/day, 30 – 40 min/session, 5 days/week" "Over time, patient’s condition gradually improved, and they were transferred from neurology ward to rehabilitation zone or rehabilitation centre to continue rehabilitation, i.e. level two rehabilitation, with content based on patient’s condition to further intensify the measures taken in level one rehabilitation; sit to stand training; transference training; use of neural stimulation technique (mainly Bobath technique and PNF technique); standing‐balance training; weight‐bearing exercises on affected limbs; gait training and stair (up and down) training, while concurrently adding in relevant occupational therapy. Therapy was done at least 2/day, 40 min/session, 5 – 6 days/week" "After a period of level two rehabilitation, most patients were discharged to their homes or community, and thus level three rehabilitation referred to patient’s continued rehabilitation at home or in the community setting. Mainly involved therapist making regular home visits, to aid the patient in performing some necessary functional training, for example further enhancing exercise ability; guiding patient on how to adapt to the home living environment; how to independently complete ADLs, etc. Therapy was done usually once every 1 – 2 weeks until six months post stroke. 'Three level rehabilitation' training always required participant's family members or nurse to be present to learn the key points of the movements, to allow participant to receive some training outwith therapy time. In particular, the participant, after discharge from hospital, still had to perform rehabilitative training with assistance from family members or nurse." Length of intervention period: 6 months Number of sessions and length of individual sessions: dependent on phase of treatment (as above) Intervention provider: "therapists; outwith therapy time, patient’s family members and nurses assisted patients with rehabilitative training" (2) No physical rehabilitation (n = 40) Received conventional clinical treatment and care Length of intervention period: no intervention Number of sessions and length of individual sessions: no intervention Intervention provider: no intervention
Outcomes	**Independence in ADL scales:** Barthel Index **Motor function scales:** NR **Measures of balance:** NR **Measures of gait velocity:** NR **Length of stay:** NR **Adverse events:** NR **Other measures:** Brunnstrom. Modified Ashworth Scale **Time points when outcomes were assessed:** at group allocation, 1 month, 3 months, and 6 months after stroke
Comparison	**Amount of physical rehabilitation in groups:** differs **Categorisation of comparison:** 1. Physical rehabilitation versus no or limited physical rehabilitation (dose differs)
Funding & conflicts of interest	**Funding statement:** not reported **Conflict of interest statement:** not reported
Synthesis	**Quantitative synthesis:**Analysis 1.1
Notes	Original study translated from Chinese to English by member of our team (PLC).

**Table:** Hou Zhi 2014

***Study characteristics***	
Methods	**Study aim:** to investigate the effect of three‐level rehabilitation training on the recovery of limb function and quality of life in stroke patients with hemiplegia **Design:** parallel‐group RCT **Country:** China **Study recruitment and setting:** see Table **Involvement of patients/public/professionals/others:** no
Participants	**Study population (number randomised):** 116 **Inclusion criteria:** Met the clinical diagnostic criteria for stroke (either cerebral infarction or cerebral haemorrhage) Diagnosed with CT **Exclusion criteria:** Poor compliance Severe mental health condition Hepatic and renal insufficiency **Participants:** detailed in Table **PROGRESS‐plus:** summarised in Table
Interventions	(1) Three‐stage rehabilitation "three‐level rehabilitation training on the basis of the control group, which mainly included: (1) Level 1 rehabilitation training: Instruct the patient on the placement of the healthy limb, the training of the affected limb and the healthy limb, the exercise of the patient's waist and abdominal muscles, and the training of standing balance, each lasting about 1 hour, once a day. The medical staff will visit the ward once every half an hour. During this period, family members must take care of the patient. The main inspection contents of the nursing staff include whether the family members are properly assisted in training, whether there is infusion or not, and whether the patient is in the correct position. (2) Level 2 rehabilitation training: The focus of rehabilitation training at this level is to supervise the implementation of rehabilitation training for patients, prevent improper training, emphasize the coordination of limb functions, and increase training intensity, 2 times/d, 45min/time. The main contents include stand‐up training, walking and normal living ability exercise. (3) Three‐level rehabilitation training: training for patients in dressing, handling personal hygiene, etc. The focus of rehabilitation training is to guide the exercise of the compensatory function of the healthy limb to the affected limb." (2) Routine care "The patients in the control group received routine rehabilitation training and nursing measures in neurology"
Outcomes	**Independence in ADL scales:** Barthel Index **Motor function scales:** Fugl Meyer assessment **Measures of balance:** NR **Measures of gait velocity:** NR **Length of stay:** NR **Adverse events:** NR **Other measures:** NR **Time points when outcomes were assessed:** "Before the rehabilitation training and at the end of 1, 3 and 6 months after the rehabilitation training"
Comparison	**Amount of physical rehabilitation in groups:** differs **Categorisation of comparison:** 1. Physical rehabilitation versus no or limited physical rehabilitation (dose differs) (limited = < 50% of dose)
Funding & conflicts of interest	**Funding statement:** not reported **Conflict of interest statement:** not reported
Synthesis	**Quantitative synthesis:**Analysis 1.1; Analysis 1.2
Notes	This study was published in Chinese and translated into English using Google Translate.

**Table:** Howe 2005

***Study characteristics***	
Methods	**Study aim:** "To evaluate a training programme aimed at improving lateral weight transference in patients following acute stroke to determine main treatment effects, if any, to inform the design of future studies." **Design:** parallel‐group RCT **Country:** UK **Study recruitment and setting:** see Table **Involvement of patients/public/professionals/others:** no
Participants	**Study population (number randomised):** 35 **Inclusion criteria:** Aged over 18 years Acute vascular stroke Previously independently mobile indoors and in personal ADL **Exclusion criteria:** Other neurological pathology Drugs or conditions affecting balance Impaired consciousness Dementia Unable to tolerate therapy 'Pusher' syndrome Severe perceptual problems **Participants:** detailed in Table **PROGRESS‐plus:** summarised in Table
Interventions	(1) Additional therapy (n = 17) Exercises aimed at improving lateral weight transference in sitting and standing. Incorporated elements of motor learning, including repetition (practice) of self‐initiated goal‐oriented activities with, where appropriate, manual guidance and verbal encouragement (feedback). Specific techniques are detailed with an appendix to the published paper. Participants in this group received the same usual care as participants in the usual care group Length of intervention period: 4 weeks Number of sessions and length of individual sessions: "as their usual care, 217 sessions, total duration 7135 min." Participants received 12 additional therapy sessions — total of six additional hours over the intervention period ("between them received 181 additional treatment sessions, mean 10.6 sessions, each of 30 min duration, total 5430 min") Intervention provider: delivered by trained physiotherapy assistants (2) Usual care (n = 18) "Physiotherapists reported that usual care was loosely based on 'neurophysiological' principles, however, their choice of specific physical interventions during each session was determined on an individual basis based on the symptomatic presentation of the patient at the time" Length of intervention period: 4 weeks Number of sessions and length of individual sessions: "Patients in the usual care group (n = 18) received 255 sessions of therapy, total duration 8643 min" Intervention provider: physiotherapists
Outcomes	**Independence in ADL scales:** NR **Motor function scales:** NR **Measures of balance:** NR **Measures of gait velocity:** NR **Length of stay:** NR **Adverse events:** NR **Other measures:** lateral reach test, weight distribution in standing, sit‐to‐stand **Time points when outcomes were assessed:** "at baseline, four weeks (retest) and eight weeks (follow‐up)"
Comparison	**Amount of physical rehabilitation in groups:** differs **Categorisation of comparison:** 5. Additional therapy + usual therapy versus usual therapy (dose differs)
Funding & conflicts of interest	**Funding statement:** "Physiotherapy Research Foundation" **Conflict of interest statement:** not reported
Synthesis	**Qualitative synthesis:** no outcomes included in analysis; all outcomes were specific to goal of lateral weight transference
Notes	

**Table:** Hu 2007

***Study characteristics***	
Methods	**Study aim:** "to investigate the effect of standardized tertiary rehabilitation (STR) on the upper and lower limbs' motor function in the patients with cerebral stroke accompanied by hemiplegia" **Design:** parallel‐group RCT **Country:** China **Study recruitment and setting:** see Table **Involvement of patients/public/professionals/others:** no
Participants	**Study population (number randomised):** 1365 (n = 965 ischaemic; n = 352 haemorrhagic) **Inclusion criteria:** Not reported **Exclusion criteria:** Not reported **Participants:** detailed in Table **PROGRESS‐plus:** summarised in Table
Interventions	(1) Three‐stage rehabilitation (n = 688) (n = 485 ischaemic; n = 178 haemorrhagic) "Patients from both groups received the same routine neurological intervention, but the treated group received additional standardised tertiary rehabilitation (STR), with details of training content outlined in references (Research Group 2007)" Length of intervention period: 6 months Number of sessions and length of individual sessions: not stated Intervention provider: not stated (2) No physical rehabilitation (n = 677) (n = 480 ischaemic; n = 174 haemorrhagic) Received routine neurological intervention Length of intervention period: not stated Number of sessions and length of individual sessions: not stated Intervention provider: not stated
Outcomes	**Independence in ADL scales:** NR **Motor function scales:** Fugl‐Meyer Assessment **Measures of balance:** NR **Measures of gait velocity:** NR **Length of stay:** NR **Adverse events:** NR **Other measures:** NR **Time points when outcomes were assessed:** at enrolment, after 1 month after stroke, after 3 months after stroke, and 6 months after stroke
Comparison	**Amount of physical rehabilitation in groups:** differs **Categorisation of comparison:** physical rehabilitation versus no or limited physical rehabilitation (dose differs)
Funding & conflicts of interest	**Funding statement:** not reported **Conflict of interest statement:** not reported
Synthesis	**Quantitative synthesis:**Analysis 1.2; Analysis 2.2
Notes	Dropouts not accounted for. Number of participants with data for extraction varied. Data analysis conducted by dividing participants by type of stroke ‐ results are presented according to "haemorragic" or "ischaemic" stroke. Original study translated from Chinese to English by member of our team (PLC).

**Table:** Huang 2003

***Study characteristics***	
Methods	**Study aim:** "to explore the importance and effect of early movement therapy on functional recovery in patients with stroke" **Design:** parallel‐group RCT **Country:** China **Study recruitment and setting:** see Table **Involvement of patients/public/professionals/others:** no
Participants	**Study population (number randomised):** 50 **Inclusion criteria:** Aged 40 to 80 years Diagnosis of stroke Confirmed by CT or MRI scan Medically stable within a week Did not exceed 2 weeks on enrolment to study GCS > 8 Deficits in limb function **Exclusion criteria:** No malignant tumour(s) High blood pressure No psychological conditions Not deaf/ mute Quadriplegic **Participants:** detailed in Table **PROGRESS‐plus:** summarised in Table
Interventions	(1) Rehabilitation (n = 25) "Both groups received routine treatment (such as medicine). For the participants in the treatment group, exercise therapy combined Bobath, Rood, Motor Relearning Program and proprioceptive neuromuscular facilitation (PNF) techniques; in terms of electrotherapy, early use of electrical stimulation, acupuncture is carried out, with additional appropriate electrical stimulation for participants with shoulder‐hand syndrome. Recovery of each motor function was targeted by using sequential and progressive therapy, using the following methods: (i) correct positioning: teaching patient’s family and caregivers the correct limb positioning of limbs in supine position, lateral position with affected limbs at lower side and healthy limbs at upper side, and lateral position with affected limbs at upper side and healthy limbs at lower side, requesting changing of body position every two hours. (ii) Rolling practice: with both hands intertwined and both elbows extended in front of the body, participants practised rolling to the left and right, with knees in flexion. (iii) Self‐assisted bed exercises: with both hands intertwined, participants extended both elbows in front of the body, overhead, to the left and right, touched the nose and did bridging exercises etc. (iv) Passive ranging bed exercises: upper limb: scapula, shoulder, elbow, wrist joints; truncal extension, shoulder retraction: lower limb: hip, knee, talo‐crural, metatarsal joints. (v) Techniques to facilitate muscle contractions. (vi) Lung clearance: percussions to the posterior segments of the lungs. (vii) Upright sitting training: gradually increasing the inclining angle of the bed, participants sat upright for 30 minutes. Once this was accomplished, the inclining angle is increased by 10 degrees until the participant could sit on the edge of bed. (viii) Stimulation to the muscles of the face, tongue and lips: opening of mouth, bulging of cheeks, gritting of teeth, extending the tongue, placing the tongue on the upper palate, iced cotton swabs (or placing ice cubes in the buccal cavity) and stimulation of taste. (ix) Breathing control practice: participants were requested to take a deep breath, slowly exhale and then relax. (x) Sitting on the edge of bed training: participants practised pushing up from side lying to sitting on the edge of bed without the inclining angle of the bed increased to 90 degrees. (xi) Sitting balance: postural correction, balance training while sitting on the edge of bed, including perturbations to the front, back, left and right. (xii) Exercises in sitting: to further train sitting balance, participants reached forwards, sideways, touched the nose, pointed to objects with intertwined hands and extended arms; lower limb strengthening exercises, taught to participant’s family and caregivers so they could supervise practice of the exercises several times a day. (xiii) Positioning from bed to wheelchair (or chair) training. (xiv) Sit to stand practice: Training of standing tolerance (beside the bed) commenced early to allow re‐gaining of gravitational sense, re‐gaining of control of muscles working against gravity, normalisation of blood pressure, correct standing balance as well as to overcome postural hypotension. Typically, participants with ischemic stroke were expected to be able to sit on the edge of bed within 3‐4 days of rehabilitation, commence standing training within 2 weeks, with the level of assistance given depending on the medical status of the participants; participants with haemorrhagic stroke should aim to sit on the edge of bed within 2 weeks of rehabilitation and commencement of standing training within 4 weeks. (xv) Participants with contractures were treated accordingly. (xvi) Neuromuscular facilitation techniques. (xvii) Gait training. (xviii) Stair practice (up and down)" Length of intervention period: 30 days Number of sessions and length of individual sessions: each session lasting at least 45 minutes, one/d Intervention provider: "one to one sessions with a therapist" (2) No physical rehabilitation (n = 25) Participants in the control group received routine treatment (such as medicine) only The individual components delivered are listed in Table 28. Based on the individual components, this intervention is categorised as comprising no treatment Length of intervention period: no intervention Number of sessions and length of individual sessions: no intervention Intervention provider: no intervention
Outcomes	**Independence in ADL scales:** Modified Barthel index **Motor function scales:** simplified Fugl‐Meyer **Measures of balance:** NR **Measures of gait velocity:** NR **Length of stay:** NR **Adverse events:** NR **Other measures:** cognitive ability rating (translated) **Time points when outcomes were assessed:** time points when outcomes were assessed: at enrolment and 30 days after enrolment
Comparison	**Amount of physical rehabilitation in groups:** differs **Categorisation of comparison:** physical rehabilitation versus no or limited physical rehabilitation (dose differs)
Funding & conflicts of interest	**Funding statement:** "supported by the National Tenth Five‐years Tackle Key Program, No. 2001BA703B21" **Conflict of interest statement:** not reported
Synthesis	**Quantitative synthesis:**Analysis 1.1; Analysis 1.2
Notes	Original study translated from Chinese to English by member of our team (PLC).

**Table:** Huang 2014

***Study characteristics***	
Methods	**Study aim:** to observe the rehabilitation effect of Bobath technique, Brunnstrom technique, Rood technique and PNF technique on delayed cerebrovascular disease **Design:** multi‐arm (5‐arms) **Country:** China **Study recruitment and setting:** see Table **Involvement of patients/public/professionals/others:** no
Participants	**Study population (number randomised):** 85 **Inclusion criteria:** Met the relevant diagnostic criteria of the "Key Points for Diagnosis of Various Cerebrovascular Diseases" revised by the Fourth National Cerebrovascular Disease Academic Conference of the Chinese Medical Association in 1995 and diagnosed by CT or MRI 2 to 7 days after the onset of cerebral infarction 7 to 14 days after the onset of cerebral haemorrhage Medically stable for 48 hours Aged 40 to 85 years Brunnstrom staging was delayed stage Able to co‐operate **Exclusion criteria:** TIA, severe heart, liver and kidney disease and cognitive impairment Other diseases affecting limb movement Severe infectious diseases or severe diabetes, malignant tumour diseases Mental health disorders **Participants:** detailed in Table **PROGRESS‐plus:** summarised in Table
Interventions	(1) Bobath group "Bobath technique rehabilitation training methods include Bobath handshake, reflex inhibition, keypoint control, facilitation techniques, proprioceptive and skin stimulation[3] , good limb placement, trunk flexion and rotation, rollover training, bridge movement, transfer training, sitting balance and other training‐based. For example, during the unaffected side roll over training, the patient Bobath shakes hands and drives the affected arm forward with the help of the healthy arm. The therapist instructs the patient to rotate and flex the head toward the unaffected side, while helping the patient lift the affected leg and rotate the trunk toward the unaffected side. 40min/time, once a day". (2) Rood group "Rood technology ‐ Tactile stimulation: brush quickly and lightly with a brush. Touch the skin of the affected side and stimulate the dermatome of the corresponding muscle group in the spinal cord segment for 3 to 5 seach time. If there is no response, repeat the process until the muscles respond. Temperature stimulation:use icecubes to stimulate the palms and soles of the affected side. Or the dorsal skin between fingers and toes for 3~5s,and then wipe dry; Rapidly and lightly pull the muscles[4] :Pulling the intrinsic muscles of the hand or foot can cause synergistic contraction of the adjacent fixed muscles; Tap the dorsal fingers of the hand, the skin between the dorsum and toes of the foot, the palm, the sole of the foot, etc. can cause the retraction of the limb, and repeated stimulation can also cause the crossed extensor response, causing the muscles around the joints to contract; visual and auditory stimulation: such as playing music with strong rhythm during training in a brightly lit, brightly colored treatment room. 40min/time, once a day." (3) Brunnstrom group "Brunnstrom technique ‐ Resistance exercise is applied to the patient's head and the unaffected limb to induce the joint response or joint movement of the affected limb. The patient is in a sitting or supine position with the unaffected upper limb stretched obliquely forward. The therapist instructs the patient to add in the unaffected upper limb and apply resistance above the elbow joint. This action can cause contraction of the pectoralis major, which can appear on the affected side after repeated practice. The affected limb uses passive and active induction of combined flexion response to promote muscle response, such as shoulder lifting and pressing. The patient is seated with the upper limb on the affected side on the abdomen, and the therapist holds the wrist joint with the hand to keep the elbow joint flexed; when the patient cannot lift the shoulder girdle at will, the therapist pushes the elbow and stimulates the upper skin of the trapezius muscle. 40min/time, once a day" (4) PNF group "PNF technique Manipulative contact: The therapist stimulates the skin, muscles, tendons, and proprioceptors of the affected side by manipulation, and induces the patient to move in the desired direction. Stretch: The therapist stretches the main muscle groups involved in the exercise to the maximum extent. The passive elongation of muscle fibers will automatically generate stretch stimulation, which in turn stimulates the stretched muscles and related synergistic muscle groups to produce contractions. Closely linked to the password, it increases proprioceptive excitability. Traction and squeezing: Pulling the affected side joint can increase the joint space, squeezing the affected side joint to reduce the joint interval, both activate the affected side joint receptors and stimulate the muscles around the affected side joint. Diffusion and reinforcement: Diffusion can stimulate the spread of responses, and reinforcement can stimulate various parts of the body to elicit purposeful coordinated movements. Timing: The sequence of the muscles of the affected limb contracting from the distal end to the proximal end, emphasizing the sequence of movements. Visual stimulation: When performing the upper part of the head, neck and trunk movement mode, vision can guide the correct movement direction, so that the patient's eye balls follow the movement direction, making it easier to complete the movement, and also helping to activate and coordinate the movement. Password and communication: The therapist should issue the appropriate password to stimulate the patient to exercise actively and improve the quality of the movement completion; the password includes the preparation password, the password in the movement and the correction password. Movement mode: including unilateral, bilateral movement mode and the movement mode of spiral diagonal crossing[5].40min/time, once a day" (5) Usual care "The five groups were all given basic treatment, and the four technical groups were given corresponding rehabilitation techniques.1.3.1 The basic treatment is symptomatic treatment with antihypertensive, hypoglycemic and lipid‐lowering drugs, and aspirin enteric‐coated tablets 100mg to resist platelet aggregation, once a day. Regularly turn over and pat on the back to prevent lung and urinary tract infections and bedsores"
Outcomes	**Independence in ADL scales:** Barthel Index **Motor function scales:** Fugl Meyer **Measures of balance:** NR **Measures of gait velocity:** NR **Length of stay:** NR **Adverse events:** NR **Other measures:** NR **Time points when outcomes were assessed:** before and after treatment
Comparison	**Amount of physical rehabilitation in groups:** between groups (1)‐(4) ‐ equivalent; between groups (1)‐(4) and (5) ‐ differs. Group (3) only appears to include upper limb treatment. **Categorisation of comparison:** 1. Physical rehabilitation versus no or limited physical rehabilitation (dose differs) (limited = < 50% of dose) (for comparison of groups (1), (2) and (4) with (5)) 3.(i) Comparison of different approaches (dose equivalent; with no other physical rehab) (for comparison of groups (1). vs (2). and (1) vs (4)).
Funding & conflicts of interest	**Funding statement:** Funded by: Guangxi Medical and Health Key Scientific Research Project, No.: 2010116 **Conflict of interest statement:** not reported
Synthesis	**Quantitative synthesis:**Analysis 1.1; Analysis 1.2; Analysis 7.2; Analysis 7.3; Analysis 7.5; Analysis 7.7
Notes	Multiple comparisons relevant to this review. Publication in Chinese, translated using Google Translate.

**Table:** Huang Yangfang 2016

***Study characteristics***	
Methods	**Study aim:** to investigate the effect of standardised three‐level rehabilitation therapy on the motor function of stroke patients with hemiplegia **Design:** parallel‐group RCT **Country:** China **Study recruitment and setting:** see Table **Involvement of patients/public/professionals/others:** no
Participants	**Study population (number randomised):** 55 **Inclusion criteria:** Stroke diagnosed using CT or MRI Met diagnostic criteria established by the Fourth National Cerebrovascular Disease Academic Conference (1995) GCS > 8 points **Exclusion criteria:** Severe heart, liver, kidney, and other vital organ diseases Cerebral haemorrhage Severe cognitive impairment **Participants:** detailed in Table **PROGRESS‐plus:** summarised in Table
Interventions	(1) Three‐stage rehabilitation Standardised three‐stage rehabilitation programme Combination of physical therapy and occupational therapy "Stage 1: Once the stroke survivors’ condition had stabilised, they started stage 1 (early) rehabilitation. This was mainly physical therapy comprising positioning, sitting, and lying, turning exercises on the paretic and non‐paretic side and dorsiflexion of the ankle joint and wrist joint, stretching exercises, bedside sitting‐balance training, sit‐stand training, gait training, and standing balance training. Occupational therapy exercises included exercises to help eating, dressing, washing, getting out of bed, activities such as puzzles etc. The first stage training mainly focused on correct positioning, passive movements and active exercises aimed at improving the stroke survivor’s ability to get up. During this period, family members were also taught to do the training and nursing so that they could get rehabilitation when they were sent home. One session/day; session duration: 45 minutes, 5 sessions/week. Stage 2: took place from the start of the 2nd month to the end of the 3rd month and involved standing balance, standing training, stair climbing training and gait training. Two sessions/week; session duration: 35‐45 minutes; 5 days/week Stage 3: took place between beginning of the 4th month and end of the 6th month. Target exercises to improve walking function and ADLs such as eating, dressing, washing, personal hygiene. Two sessions/day; session duration 30‐45 minutes; 5‐7 days/week" (2) No physical rehabilitation "did not receive any rehab treatment" ‐ doctor instructions and family involvement in getting out of bed and doing activities. Both groups received conventional neurology treatment.
Outcomes	**Independence in ADL scales:** NR **Motor function scales:** NR **Measures of balance:** NR **Measures of gait velocity:** NR **Length of stay:** NR **Adverse events:** NR **Other measures:** functional comprehensive assessment **Time points when outcomes were assessed:** before and after treatment
Comparison	**Amount of physical rehabilitation in groups:** differs **Categorisation of comparison:** 1. Physical rehabilitation versus no or limited physical rehabilitation (dose differs) (limited = < 50% of dose)
Funding & conflicts of interest	**Funding statement:** not reported **Conflict of interest statement:** not reported
Synthesis	**Qualitative synthesis:** no relevant outcomes assessed
Notes	This study was published in Chinese and relevant sections translated into English by our author team (PLC).

**Table:** Hui‐Chan 2009

***Study characteristics***	
Methods	**Study aim:** "to compare the effectiveness of three active home‐based treatment programmes (TENS, TENS+TRT, and placebo‐TENS with TRT[PLBO+TRT]) versus no active treatment on spasticity, muscle strength and walking performance in chronic stroke patients" **Design:** multi‐arm RCT (3‐arms) **Country:** Hong Kong, SAR **Study recruitment and setting:** see Table **Involvement of patients/public/professionals/others:** no
Participants	**Study population (number randomised):** 54 (109 in whole study—but only 54 in groups relevant to this review: see notes) **Inclusion criteria:** Sustained a single stroke more than 1 year previously Able to walk 10 m unassisted (with or without walking aids) Composite Spasticity Score of ≥ 10 in their ankle plantar flexors **Exclusion criteria:** Medical comorbidity, e.g. unstable cardiopulmonary disease (acute myocardial infarction wearing a cardiac pacemaker, shortness of breath, tachycardia) Uncontrolled diabetes mellitus Pre‐existing neurological disorders such as multiple sclerosis, receptive dysphasia, or cognitive impairment (denoted by scoring < 7 out of 10 on the Abbreviated Mental Test)" **Participants:** detailed in Table **PROGRESS‐plus:** summarised in Table
Interventions	(1) Task‐related training (TRT) (n = 25) "received 60 minutes of placebo‐TENS from TENS devices with the electrical circuit disconnected inside, followed by 60 minutes of TRT which included six exercises: (i) loading exercise on the affected leg, (ii) stepping up exercise with the affected leg, (iii) stepping down exercise with the unaffected leg, (iv) heel lifts from a dorsiflexed position when standing, (v) standing up from a chair, walking a short distance, and returning to the chair, and (vi) walking with rhythmic auditory cues generated by a metronome" Length of intervention period: 4 weeks Number of sessions and length of individual sessions: "home rehabilitation programme daily, 5 days a week" Intervention provider: "The treatment compliance and safety of the programme [were] closely monitored by the physiotherapist in charge" (2) No physical rehabilitation (n = 29) "received no treatment" Length of intervention period: no intervention Number of sessions and length of individual sessions: no intervention Intervention provider: no intervention Note: This study also included a TENS group (n = 28) and a TENS + TRT group (n = 27) that we judged not to be relevant to this review, and no data relating to these groups have been extracted.
Outcomes	**Independence in ADL scales:** NR **Motor function scales:** NR **Measures of balance:** NR **Measures of gait velocity:** gait velocity **Length of stay:** NR **Adverse events:** NR **Other measures:** Timed Up and Go (TUG) test, Six‐Minute Walk test, Composite Spasticity Scale, surface electromyography and torque measurements **Time points when outcomes were assessed:** "assessor blinded to the treatment allocation assessed the subjects at four time intervals: before and after 2 and 4 weeks of treatment, and 4 weeks after treatment"
Comparison	**Amount of physical rehabilitation in groups:** differs **Categorisation of comparison:** physical rehabilitation versus no or limited physical rehabilitation (dose differs)
Funding & conflicts of interest	**Funding statement: "**HSRF project number: 01030981; Food and Health Bureau, Hong Kong SAR Government. SSM Ng was additionally supported by a scholarship from The Hong Kong Polytechnic University" **Conflict of interest statement:** not reported
Synthesis	**Quantitative synthesis:**Analysis 1.4; Analysis 2.4
Notes	

**Table:** Imhof 2015

***Study characteristics***	
Methods	**Study aim:** investigated the effect of a new nursing intervention (Mobility Enhancing Nursing Intervention—MFP) designed to improve rehabilitation outcomes **Design:** parallel‐group RCT **Country:** Switzerland **Study recruitment and setting:** see Table **Involvement of patients/public/professionals/others:** no
Participants	**Study population (number randomised):** 140 mixed population **Inclusion criteria:** Diagnosed with MS, stroke, or brain injuries German‐speaking Aged 18 years + Cognitively able to give written consent **Exclusion criteria:** Not reported **Participants:** detailed in Table **PROGRESS‐plus:** summarised in Table
Interventions	(1) mobility‐enhancing nursing intervention (MFP) "MFP is a nursing intervention based on the assumption that learning takes place through movement [8]. Human development is seen to be based on the interaction between a person and the environment. Tactile‐kinaesthetic perception is important with regard to the way that the environment is perceived and fundamental to the development, organisation, and reorganisation of the brain. Thus, tactile‐kinaesthetic stimulation is used by nurses during the mobilisation process. It was hypothesised that the way the patients perceived themselves, their environment, their body position changes, and hence cognitive‐linguistic, social, emotional, and motor behaviour would be enhanced. For these purposes, the patients’ mattresses were placed on the floor, which enabled the patients to explore their environment safely without the risk of falling. Additionally, the patients’ environment was arranged in accordance with a nursing assessment pertaining to the patients’ impairment and abilities, their goals in terms of improved mobility, and the mobility they would require in order to live at home as independently as possible. Initially, most patients favoured a specific side to get up. The goal of the intervention was to teach the patients to get up step by step and to move indepen‐dently over both sides. Compared to standard mobilisation procedure from a bed which uses gravity, MFP care enables patients to overcome gravity which requires tailored support. Hence this technique improves the spatial orientation of the patients. Constant tactile‐kinaesthetic stimulation was applied by guiding the person from her current position, for example, standing, laying, or sitting in a wheelchair, to the floor. With successive gestures and position changes, the person was guided back into the original position using kinaesthetic [9]. In order to implement the intervention, all nurses on the wards received training in two units of 3 and 5 days and ongoing clinical training (2–4 hours a month) on kinaesthetic principles. In the intervention group, MFP was applied during 30 days in addition to the standard rehabilitation program in the control group, which was provided by physicians, physiotherapists, occupational therapists, and standard nurses." (2) Control group ‐ standard rehabilitation
Outcomes	**Independence in ADL scales:** Extended Barthel Index **Motor function scales:** NR **Measures of balance:** NR **Measures of gait velocity:** NR **Length of stay:** yes **Adverse events:** NR **Other measures:** self care index; WHOQoL‐Bref, Fall Efficacy Scale **Time points when outcomes were assessed:** NR
Comparison	**Amount of physical rehabilitation in groups:** differs **Categorisation of comparison:** 5. Additional therapy + usual therapy versus usual therapy (dose differs)
Funding & conflicts of interest	**Funding statement:** Swiss National Science Foundation (no. 13DPD6 132090) and the Swiss Multiple Sclerosis Society **Conflict of interest statement:** none
Synthesis	**Qualitative synthesis:** study has a mixed population of stroke survivors and people with MS, and outcome data is not presented for stroke survivors only.
Notes	

**Table:** Indurkar 2013

***Study characteristics***	
Methods	**Study aim:** to evaluate the efficacy of a task oriented intervention on walking distance, speed and balance efficiency in post‐stroke patients **Design:** parallel‐group RCT **Country:** India **Study recruitment and setting:** see Table **Involvement of patients/public/professionals/others:** no
Participants	**Study population (number randomised):** 30 **Inclusion criteria:** Clinical diagnosis of first or recurrent stroke Residual walking deficit resulting from most recent stroke Able to comprehend the instructions Able to walk 10 m independently using aids/orthoses Able to provide informed consent **Exclusion criteria:** Deteriorating medical condition Recovery of walking ability, age and gender‐specific norms on 6‐minute walk test Perceptual dysfunction Musculoskeletal and cardiorespiratory dysfunction **Participants:** detailed in Table **PROGRESS‐plus:** summarised in Table
Interventions	(1) Task oriented activities In addition to routine physiotherapy ‐ 30 minutes of task‐oriented activities. Activities included step‐ups, balance beam, walking, kicking ball, obstacle course, treadmill walking in a circuit. Each activity was timed. Carried out 6 days/week for 3 weeks. (2) Physiotherapy One hour of physiotherapy, which included bed mobility exercises, mat exercises, stretching for the required muscles, exercises on vestibular ball, gait training
Outcomes	**Independence in ADL scales:** NR **Motor function scales:** NR **Measures of balance:** Berg balance scale **Measures of gait velocity:** 6‐minute walk test, 5, walk test, Timed Up and Go test **Length of stay:** NR **Adverse events:** NR **Other measures:** NR **Time points when outcomes were assessed:** before and after 3 week intervention
Comparison	**Amount of physical rehabilitation in groups:** differs **Categorisation of comparison:** 5. Additional therapy + usual therapy versus usual therapy (dose differs)
Funding & conflicts of interest	**Funding statement:** not reported **Conflict of interest statement:** none
Synthesis	**Qualitative synthesis:** data not suitable for analysis. (Data presented as medians and IQR.)
Notes	

**Table:** Jandaghi 2021

***Study characteristics***	
Methods	**Study aim:** to compare effects of balance training on an unstable surface with balance training under visual deprivation conditions in persons with stroke **Design:** multi‐arm RCT (3‐arm) **Country:** Iran, Islamic Rep. **Study recruitment and setting:** see Table **Involvement of patients/public/professionals/others:** no
Participants	**Study population (number randomised):** 45 **Inclusion criteria:** First ever stroke at least 6 months after stroke Able to walk at least 10 m without assistance **Exclusion criteria:** Severe cognitive problems, neurological disease, and musculoskeletal conditions preventing participation in rehabilitation programme **Participants:** detailed in Table **PROGRESS‐plus:** summarised in Table
Interventions	(1) Visual deprivation ‐ stable base training "instructed to perform balance training exercise on a firm floor while they are keeping their eyes closed. The balance training program consisted of Weight shifting; toe/heel rising; heel/toe standing and one leg standing exercise" (2) Unstable base training "performed balance training on a firm foam (balance pad:Mambo balance pad 37, 22, 6 cm Europe bvba) with open eyes." (3) Control group "received general physiotherapy exercise" "All groups received general physical therapy exercise including muscle stretching and strengthening exercise that was specific for each patient by a trained researcher." "Each exercise session consisted of 4 sets in 30 minutes with one minute of rest interval between each set. The interventions were carried out for one month, 3 sessions per week" Comparison of relevance to this review: (1) vs (3)
Outcomes	**Independence in ADL scales:** NR **Motor function scales:** NR **Measures of balance:** NR **Measures of gait velocity:** Timed Up and Go Test **Length of stay:** NR **Adverse events:** NR **Other measures:** Four Square Test, Five Times Sit to Stand test **Time points when outcomes were assessed:** baseline and after intervention
Comparison	**Amount of physical rehabilitation in groups:** differs **Categorisation of comparison:** 5. Additional therapy + usual therapy versus usual therapy (dose differs)
Funding & conflicts of interest	**Funding statement:** no specific grant from any funding agency in the public, commercial, or not‐for‐profit sectors **Conflict of interest statement:** none
Synthesis	**Quantitative synthesis:**Analysis 5.4
Notes	

**Table:** Jeon 2018

***Study characteristics***	
Methods	**Study aim:** to evaluate the effect of bilateral lower limb strengthening designed to improve balance and walking in stroke patients **Design:** parallel‐group RCT **Country:** Korea, Rep. **Study recruitment and setting:** see Table **Involvement of patients/public/professionals/others:** no
Participants	**Study population (number randomised):** 20 **Inclusion criteria:** Age < 65 years Diagnosis of hemiplegia < 24 months after stroke Able to understand and follow instructions Able to walk independently for 20 m without an assistive device **Exclusion criteria:** Joint or muscular problems arising from conditions other than stroke Unstable hypertension or cardiovascular condition Stroke involving the brainstem **Participants:** detailed in Table **PROGRESS‐plus:** summarised in Table
Interventions	(1) Unilateral therapy group (UTG) "The UTG completed the strengthening exercise only in the paretic lower limb (paretic side, 50 minutes; passive range of motion exercises, 10 minutes)" (2) Bilateral therapy group (BTG) "The BTG completed the strengthening exercise in the paretic and non‐paretic lower limbs (paretic side, 30 minutes; non‐paretic side, 20 minutes; passive range of motion exercises, 10 minutes)." "The exercise program targeted the hip and the ankle regions. The hip exercise was performed in a prone position with the leg to be exercised positioned off the side of the examination table. With a straight leg, and with a dorsiflexed ankle, the patient slowly raised their leg as high as possible without causing pain, then slowly returned their leg to the starting position and repeated the motion. When performing this exercise, the therapist stabilized the pelvis to prevent the patient from twisting or arching their back. The ankle exercise was performed in a supine position on the examination table. From a relaxed ankle and foot position, the patient dorsiflexed their ankle, keeping the knee straight and holding the position, then slowly returned their foot to the starting position and repeated the motion. The exercises were initiated with moderate assistance and progressed to minimal assistance."
Outcomes	**Independence in ADL scales:** NR **Motor function scales:** NR **Measures of balance:** Berg Balance Scale **Measures of gait velocity:** 10‐m walk test, Timed Up and Go test **Length of stay:** NR **Adverse events:** NR **Other measures:** functional reach test **Time points when outcomes were assessed:** before and after treatment
Comparison	**Amount of physical rehabilitation in groups:** equivalent **Categorisation of comparison:** 4. Comparison of similar approaches (dose equivalent)
Funding & conflicts of interest	**Funding statement:** not reported **Conflict of interest statement:** none
Synthesis	**Qualitative synthesis only:** studies comparing similar approaches were not combined within statistical analyses.
Notes	

**Table:** Jing 2006

***Study characteristics***	
Methods	**Study aim:** "to observe the effects of the early intervention of occupational therapy plus exercise therapy on the ADL in stroke patients and compare the effects with those of exercise therapy only" **Design:** parallel‐group RCT **Country:** China **Study recruitment and setting:** see Table **Involvement of patients/public/professionals/others:** no
Participants	**Study population (number randomised):** 160 **Inclusion criteria:** Stroke diagnosis according to the evaluative standard revised by the 1995 Fourth National Academic Conference of Cerebral Vascular Disease Stroke confirmed by CT or MRI scan First ever stroke, within 3 months of stroke Cognitively sound Able to co‐operate Understood and agreeable to intervention **Exclusion criteria:** Incomplete patient information Memory loss **Participants:** detailed in Table **PROGRESS‐plus:** summarised in Table
Interventions	(1) Exercise and occupational therapy group (n = 120) "For the exercise + occupational therapy participants, while following the principles of the intervention of the exercise therapy group, the following intervention was done: early rolling, donning, feeding, transfers etc and re‐learning and practising ADL activities, focusing on activities of choice for therapy, focusing on the dexterity of affected limbs, through active and active‐assisted means of training, and compensating with the non‐affected limb training etc. Emphasised on activities with the largest ADL limitation. In the ward, patient’s family and nurse supervised ADL activities, rendering as little assistance as possible. 45‐60 min/day, one to one therapy session with an occupational therapist" Length of intervention period: not stated Number of sessions and length of individual sessions: 45 to 60 minutes/d Intervention provider: occupational therapist (2) Exercise therapy group (n = 40) "For the exercise therapy participants, after becoming medically stable, while concurrently receiving conventional treatment, had the following intervention: positioning of the unaffected limbs, passive ranging exercises of the joints, bridging exercises, neuromuscular facilitation technique, sitting balance, standing balance and gait training etc" Intervention Length of intervention period: on average 7 weeks Number of sessions and length of individual sessions: 40 to 50 minutes/d Intervention provider: one‐to‐one therapy session with an exercise therapist
Outcomes	**Independence in ADL scales:** Barthel Index **Motor function scales:** Fugl‐Meyer Assessment **Measures of balance:** NR **Measures of gait velocity:** NR **Length of stay:** NR **Adverse events:** NR **Other measures:** NR **Time points when outcomes were assessed:** within 24 hours of commencement of therapy, every 2 weeks thereafter
Comparison	**Amount of physical rehabilitation in groups:** equivalent **Categorisation of comparison:** comparison of similar approaches (dose equivalent)
Funding & conflicts of interest	**Funding statement:** "supported by grants from National Key Basic Research Development Program, No 2003CB517103; Key Technological Program of Henan Department of Technology in 2002, No. 0324410001" **Conflict of interest statement:** not reported
Synthesis	**Qualitative synthesis:** As the two active treatment groups were classified as including similar treatment components, data from this study have not been included within analyses comparing approaches.
Notes	Original study translated from Chinese to English

**Table:** Ji Pei 2014

***Study characteristics***	
Methods	**Study aim:** "to investigate the the clinical effect of Bobath technique‐based exercise therapy on limb motor dysfunction after stroke" **Design:** parallel‐group RCT **Country:** China **Study recruitment and setting:** see Table **Involvement of patients/public/professionals/others:** no
Participants	**Study population (number randomised):** 90 **Inclusion criteria:** Met the diagnostic criteria for stroke and confirmed by CT or MRI Lesion located in one cerebral hemisphere and one limb hemiplegic First‐onset stroke within 15 to 60 days Provided informed consent **Exclusion criteria:** Other secondary disease (e.g. cerebral haemorrhage or another stroke) TIA or reversible neurological function Cerebral embolism, brain trauma, parasitic disease, brain tumour or cases requiring craniocerebral surgery Other heart disease combined with cerebrovascular disorder Serious liver, kidney, endocrine system and other diseases as the primary disease Psychiatric disease, disturbance of consciousness, severe aphasia or cognitive impairment **Participants:** detailed in Table **PROGRESS‐plus:** summarised in Table
Interventions	(1) Rehabilitation "On the basis of the above treatment, the treatment group adopted exercise therapy based on Bobath technique, as follows. 1) The placement of good posture in the acute stage, and passive movement to maintain muscle tension and joint range of motion. In the early stage, the passive movement of the limbs is 10‐15 min·time‐1, 2‐3 times·d‐1. The conscious person can hold the affected hand with the unaffected hand to drive the affected limb to complete active‐assisted movement and complete active movement. Induced limb pathological patterns should be avoided and coordinated movements should be used to accomplish this. 2) Bed training during the recovery period. If the condition allows, you can perform bed turning training (the healthy side to the affected side, the affected side to the healthy side), move the body up and down, left and right, low back muscles, abdominal muscles and respiratory muscles training, hip extension training (bridge exercise, by double bridge to a single bridge). 3) Sit up and sit balance training in bed. Such as level 3 sitting balance training, it is required to achieve level 3 balance. 4) Standing balance training. Center of gravity transfer, weight‐bearing of affected limbs, standing bed training, etc.; bed‐chair transfer, sit‐stand transfer and standing training (from standing electric upright bed ‐ standing frame ‐ supporting standing ‐ standing alone). 5) Walking training. Such as walking with support, walking within the balance bar, changing gait training, training up and down steps, and complex walking exercises. 6) Upper body and hand function training. 7) Occupational therapy. Mainly carry out activities of daily living and above training once a day, about 1 hour each time, training for 5 days as a course of treatment, with a 2‐day rest in between. " (2) No physical rehabilitation "Patients in both groups were routinely treated with neurological drugs, underlying diseases and acupuncture."
Outcomes	**Independence in ADL scales:** modified Barthel Index **Motor function scales:** NR **Measures of balance:** NR **Measures of gait velocity:** NR **Length of stay:** NR **Adverse events:** NR **Other measures:** Criteria for Assessment of the Degree of Clinical Neurological Impairment in Stroke Patients (CSS) **Time points when outcomes were assessed:** before and after 1 month of treatment
Comparison	**Amount of physical rehabilitation in groups:** differs **Categorisation of comparison:** 1. Physical rehabilitation versus no or limited physical rehabilitation (dose differs) (limited = < 50% of dose)
Funding & conflicts of interest	**Funding statement:** not reported **Conflict of interest statement:** not reported
Synthesis	**Quantitative synthesis:**Analysis 1.1
Notes	This study was published in Chinese and translated into English using Google Translate.

**Table:** Jongbloed 1989

***Study characteristics***	
Methods	**Study aim:** to compare the effectiveness of the sensorimotor integrative approach with the functional approach in people with stroke **Design:** parallel‐group RCT **Country:** Canada **Study recruitment and setting:** see Table **Involvement of patients/public/professionals/others:** no
Participants	**Study population (number randomised):** 90 **Inclusion criteria:** Admitted to the hospital within 12 weeks after a first stroke Weakness in the upper and lower extremities on one side of the body on admission (Brunnstrom synergy score of 1‐5) Not have resided in an extended care setting before the stroke Not be severely aphasic Signed an informed consent form **Exclusion criteria:** Not reported **Participants:** detailed in Table **PROGRESS‐plus:** summarised in Table
Interventions	(1) Sensorimotor integration treatment "....combines sensiorimotor theories, primarilty those described by Bobath, Rood and Ayres ... emphasizes treating the cause of the dysfunction rather than compensating for, or adapting to, the problem. The principles of treatment are (a) provide planned and controlled sensory input, (b) elicit an adaptive response, (c) enhance organisation of brain mechnanisms, (d) facilitate the developmental sequence, (e) consider the interdependence of sensory systems, (f) maintain a balance of sensory integration, (g) focus on sensory integration and its integration with movement" (See Appendix of paper) (2) Functional treament "This approach emphasizes the practice of particular tasks, usually activities of daily living, which make the patient more independent in meeting his or her basic needs.These tasks may include dressing, grooming, bathing, toileting, mobility, and homemaking. The emphasisis on treating the symptom rather than the cause of the dysfunction. The functional approach can be subdivided into two aspects: compensation and adaptation." Compensation ‐ therapist facilitates awareness of a problem and helps patient make allowances for it. Adaptation ‐ modifications of environment to allow person to function with greater independence Physical function ‐ opportunities to participate in functional activities to improve limb function Splinting ‐ functional and resting splints may be provided (See Appendix of paper) "All subjects received similar assistance in morning and evening self‐care. For example, subjects dressed their affected side first and transfers were performed to the unaffected side. After the self‐care routine had been established by the occupational therapist, it was carried out by a nurse's aide. All subjects received similar medical and nursing care and physical therapy"
Outcomes	**Independence in ADL scales:** Barthel Index **Motor function scales:** NR **Measures of balance:** NR **Measures of gait velocity:** NR **Length of stay:** NR **Adverse events:** NR **Other measures:** meal preparation, Sensorimotor Integration Test Battery **Time points when outcomes were assessed:** 4 and 8 weeks after randomisation
Comparison	**Amount of physical rehabilitation in groups:** equivalent **Categorisation of comparison: 3**. (ii) Comparison of different approaches, (dose equivalent; with both/all groups also receiving other physical rehab)
Funding & conflicts of interest	**Funding statement:** Funding for this study was provided by the British Columbia Health Care Research Foundation **Conflict of interest statement:** not reported
Synthesis	**Qualitative synthesis:** Results not presented in a suitable format for quantitative analysis ‐ no variance reported.
Notes	

**Table:** Khallaf 2014

***Study characteristics***	
Methods	**Study aim:** to investigate the effect of task‐specific exercises, gait training, and visual biofeedback on correcting equinovarus gait among individuals with stroke **Design:** parallel‐group RCT **Country:** Egypt, Arab Rep. **Study recruitment and setting:** see Table **Involvement of patients/public/professionals/others:** no
Participants	**Study population (number randomised):** 16 **Inclusion criteria:** History of unilateral first ever stroke causing hemiparesis with duration of illness not less than 3 months Medically stable Able to understand and give study consent form In at least stage 4 of motor recovery of foot according to Chedoke‐McMaster Stroke Assessment with spasticity < grade 2 by MAS Able to walk independently with/without assistive devices for 6 minutes **Exclusion criteria:** Lower limb sensory impairment or cognitive, mental, and visual deficits Ankle, knee contractures or receiving a muscle relaxant **Participants:** detailed in Table **PROGRESS‐plus:** summarised in Table
Interventions	(1) Intensive mobility training ‐ task specific exercise "included manual stretching, muscle‐specific progressive‐resistive exercise, balance training, and walking program (50–70% age adjusted heart rate maximum) with BFB from the E‐med pedography. Manual prolonged stretch technique was applied for the calf and hip adductor muscles with holding time of 30 seconds. Manual resistance exercises were followed by theraband strengthening for foot evertors, ankle dorsiflexors, knee flexors, hip extensors, hipabductors, and knee extensors. As the participant is able to master the proposed movement, therabands were used for increasing the number of repetitions and resistance. Functional strengthening exercises were also used for evertors and hip abductors. Patients were asked to do abduction while standing against a wall by sliding the heel against the wall with weight cuffs just above the ankle. Sideway walking onto blocks was used for the same groups of muscles. An exercise like horse pawing was used for increasing knee flexors and planter flexors strength as preparation for gait training [24]. With fully extended knees, patients were also asked to raise, hold, and lower the forefoot from the floor midway between eversion and inversion while standing against wall. Knee taps to the wall and wall calf stretch exercises were practiced for stretching the calf muscles and forward pivot training. For balance training, step up and sideways onto a step, chair rise, marching, kicking a ball laterally, stops and turns while walking were used. These exercises are applied for 90 minutes 5 times a week for 8 weeks" "received also walking program (50–70% age adjusted heart rate maximum) with biofeedbackfrom the pedography. During gait training, the patient was instructed to step over the E‐med platform five times." (2) Control group ‐ traditional physiotherapy "program of strengthening exercises for the foot evertors and ankle dorsiflexors in addition to prolonged stretching of the calf muscles. The patients also received gait training with cones between the parallel bars. Participants were given five sessions per week for 8 weeks, 50 minutes for each session. A solid ankle foot orthosis (AFO) was also used as a traditional treatment of the foot equinovarus deformity. The patients were instructed to use the AFO for at least eight hours a day."
Outcomes	**Independence in ADL scales:** NR **Motor function scales:** NR **Measures of balance:** NR **Measures of gait velocity:** NR **Length of stay:** NR **Adverse events:** NR **Other measures:** pressure measurements during gait **Time points when outcomes were assessed:** 1 week and 4 weeks after last session
Comparison	**Amount of physical rehabilitation in groups:** equivalent **Categorisation of comparison:** 3.(i) Comparison of different approaches (dose equivalent; with no other physical rehab)
Funding & conflicts of interest	**Funding statement:** not reported **Conflict of interest statement:** none
Synthesis	**Qualitative synthesis:** no relevant outcome measures assessed
Notes	

**Table:** Kim 2007

***Study characteristics***	
Methods	**Study aim:** to investigate the effect of task‐related circuit training in old‐aged stroke patients **Design:** parallel‐group RCT **Country:** Korea, Rep. **Study recruitment and setting:** see Table **Involvement of patients/public/professionals/others:** no
Participants	**Study population (number randomised):** 24 **Inclusion criteria:** Older patients who had had a stroke for > 3 months Able to walk more than 10‐m independently regardless of the use of assistive devices **Exclusion criteria:** Unstable medical conditions Musculoskeletal disorders that could affect gait **Participants:** detailed in Table **PROGRESS‐plus:** summarised in Table
Interventions	(1) Circuit training "The experimental group received cyclical exercise therapy 3 times a week for 50 minutes, palliative exercise therapy was performed twice a week for 30 minutes...... Treatment was conducted for a total of 6 weeks. Circulatory exercise therapy consisted of a total of 9 functional tasks, and each task was performed in pairs for 5 minutes, repeated for 5 minutes. In the process, 4‐5 physical therapists supervised and adjusted the exercise intensity and difficulty according to the patient's level. The circular exercise therapy program was developed and applied as 9 functional tasks that can improve the patient's flexibility, sense of balance, and motor function of the paralyzed upper and lower extremities (Table 2). Range of motion exercise, functional mat exercise, and gait training were performed". Plus conventional physical therapy, the same as control group. (2) Control group "conventional physical therapy five times a week for 6 weeks." This was delivered "3 times a week for 50 minutes, twice a week for 30 minutes".
Outcomes	**Independence in ADL scales:** modified Barthel Index **Motor function scales:** NR **Measures of balance:** Berg Balance Scale **Measures of gait velocity:** 6‐minute walking test **Length of stay:** NR **Adverse events:** NR **Other measures:** step test **Time points when outcomes were assessed:** baseline and after treatment
Comparison	**Amount of physical rehabilitation in groups:** differs **Categorisation of comparison:** 5. Additional therapy + usual therapy versus usual therapy Note: the additional therapy was provided in "replacement" for some of the usual therapy, so the total dose of treatment was the same for both groups in this study. We have noted this within analyses.
Funding & conflicts of interest	**Funding statement:** not reported **Conflict of interest statement:** not reported
Synthesis	**Quantitative synthesis:**Analysis 5.1; Analysis 5.3; Analysis 5.4
Notes	Paper in Korean and translated using Google Translate.

**Table:** Kim 2012

***Study characteristics***	
Methods	**Study aim:** "examined the effects of task‐oriented training on the trunk control ability, balance and gait of stroke patients to suggest effective training methods for the functional improvement of stroke patients." **Design:** parallel‐group RCT **Country:** Rep. Korea **Study recruitment and setting:** see Table **Involvement of patients/public/professionals/others:** no
Participants	**Study population (number randomised):** 20 **Inclusion criteria:** Able to walk 10 m independently (using an aid or orthotic with/without supervision or aid) Minimum score of 20 on the MMSE (Korean version) **Exclusion criteria:** Joint contraction Pain or fracture of the musculoskeletal system Hemianopia **Participants:** detailed in Table **PROGRESS‐plus:** summarised in Table
Interventions	(1) Task‐oriented training (n = 10) "Subjects in both groups underwent conservative physical therapy" "The experimental group also participated in task‐oriented training for 1 hour per day, 3 days a week, for 4 weeks.The training consists of 10 walking‐related tasks designed to strengthen the lower extremities, and enhance the walking balance, speed and distance in a progressive manner. The 10 tasks were (i) step‐ups, (ii) balance beam, (iii) kicking a ball, (iv) stand up and walk, (v) obstacle course, (vi) treadmill, (vii) walk and carry, (viii) speed walk, (ix) walk backwards, and (x) stairs. Before commencing training, the subjects warmed up for 5 minutes to improve their range of motion and flexibility. Each item was practiced for 5 minutes, and 1 minute of rest time was allowed between each item" Length of intervention period: 4 weeks Number of sessions and length of individual sessions: conservative physical training for 1 hour per day, 5 days a week in addition to task‐oriented training for 1 hour per day, 3 days per week Intervention provider: "supervised by a physical or occupational therapist" (2) Conventional physiotherapy (n = 10) "Conservative physical therapy consisted of joint mobilization, muscle strengthening, and balance training" Length of intervention period: 4 weeks Number of sessions and length of individual sessions: conservative physical training for 1 hour per day, 5 days a week Intervention provider: not stated
Outcomes	**Independence in ADL scales:** NR **Motor function scales:** NR **Measures of balance:** Berg Balance Scale **Measures of gait velocity:** 10‐Metre Walk test, Timed‐Up and Go test (TUG) **Length of stay:** NR **Adverse events:** NR **Other measures:** Trunk Impairment Scale **Time points when outcomes were assessed:** "measured before and after the 4 weeks of therapy"
Comparison	**Amount of physical rehabilitation in groups:** differs **Categorisation of comparison:** 5. Additional therapy + usual therapy versus usual therapy (dose differs)
Funding & conflicts of interest	**Funding statement:** not reported **Conflict of interest statement:** not reported
Synthesis	**Quantitative synthesis:**Analysis 5.3; Analysis 5.4
Notes	

**Table:** Kim 2012a

***Study characteristics***	
Methods	**Study aim:** to evaluate the effects of rhythmic auditory stimulation gait training on dynamic balance and gait ability of patients with subacute stroke using a smartphone metronome application **Design:** parallel‐group RCT **Country:** Korea, Rep. **Study recruitment and setting:** see Table **Involvement of patients/public/professionals/others:** no
Participants	**Study population (number randomised):** 20 **Inclusion criteria:** Subacute hemiplegic with time after stroke < 6 months following diagnosis No cognitive impairment (i.e. MMSE ≥ 24) No visual, auditory, or orthopaedic injuries that might affect balance Able to understand instructions and give informed consent **Exclusion criteria:** Not reported **Participants:** detailed in Table **PROGRESS‐plus:** summarised in Table
Interventions	(1) Gait training "Subjects in the RAS (rhytmic auditory stimulation) group participated in 15 sessions of gait training 30min per session three times per week. In this study, a metronome application for smartphone was used for easier and more convenient provision of RAS. The strength of a smartphone application is that by virtue of rapid increase in the number of smartphone users, it can easily and instantly be used. The metronome application used in the performance of this experiment was the ZyMi MetronomeFREE, which is provided free of charge for the Android Operating system, and its beat was provided as auditory stimulation through individual earphones. RAS training sessions were conducted in a rectangular gait training space, 20m × 5m. The subject’s cadence was calculated by multiplying by six the number of steps, measured during 10m walk at a comfortable speed, prior to training. The training program was composed of five stages, and each stage was conducted for five minutes. A one‐minute break was given after each stage. Participants were instructed to walk a 20‐m course repeatedly for a period of one minute of training so that they could adapt to the beat of the metronome. In the first stage, participants practiced forward walking, backward walking, and side walking to the metronome beat. In the second stage, subjects were required to stand up from a chair with arm rests, walk 3 m, turn around, return to the chair, and sit down. In the third stage, they were encouraged to cross obstacles placed in font of them to the metronome beat. The obstacles were 5cm, 10cm, and 15cm high, respectively,and randomly arranged. In the fourth stage, the subjects climbed upstairs and downstairs to the metronome beat. For the stair gait, a staircase located outside the treatment room, with a step height x width of 15 × 150cm, was used. During stair gait, cadence was initially provided at a comfortable speed. When the given metronome beat was too fast, a decrease in cadence of approximately 20% was offered. In the fifth stage, forward walking was performed by increasing the cadence of a comfortable speed by 5%. When participants were capable of maintaining a certain gait velocityfor longer than one minute with the increased cadence, they were instructed to maintain the velocity on their own by gradually turning down the volume of the metronome and eventually muting it completely." (2) Control "Subjects in both the RAS and control groups were provided with 30‐minute conventional therapy sessions twice a day, five times a week. Conventional therapy, which is defined as one‐on‐one neurodevelopmental therapy between a patient and a therapist, was composed of sitting up from lying down, sit to stand, and trunk and limb training aimed at learning normal gait patterns."
Outcomes	**Independence in ADL scales:** NR **Motor function scales:** NR **Measures of balance:** NR **Measures of gait velocity:** Timed Up and Go Test, gait velocity **Length of stay:** NR **Adverse events:** NR **Other measures:** Activities‐Specific Balance Confidence Scale, Dynamic Gait Index, Four Square Step Test, Functional Ambulation Category, Spatiotemporal gait parameters **Time points when outcomes were assessed:** before and after intervention
Comparison	**Amount of physical rehabilitation in groups:** differs **Categorisation of comparison:** 5. Additional therapy + usual therapy versus usual therapy (dose differs)
Funding & conflicts of interest	**Funding statement:** not reported **Conflict of interest statement:** not reported
Synthesis	**Quantitative synthesis:**Analysis 5.4
Notes	

**Table:** Kim 2014

***Study characteristics***	
Methods	**Study aim:** to understand the effects of task‐oriented gross motor group exercise based on motor development on chronic stroke patients’ joint, bone, muscle, and motor functions and ADLs **Design:** parallel‐group RCT **Country:** Korea, Rep. **Study recruitment and setting:** see Table **Involvement of patients/public/professionals/others:** no
Participants	**Study population (number randomised):** 28 **Inclusion criteria:** Chronic stroke patients at least 5 years stroke diagnosis Brain lesions degree was 1 or 2 MMSE‐Korean version ≥ 20 Able to understand and communicate **Exclusion criteria:** Not reported **Participants:** detailed in Table **PROGRESS‐plus:** summarised in Table
Interventions	(1) Gross motor group exercise group "The experimental group additionally performed the gross muscle group exercise for 50 minutes a day, 3 times a week for 6 weeks in total. The gross muscle exercise program comprised the 1st phase of the sensorimotor stage (ages from 0 to 2), 2nd phase of pre‐operational stage (ages from 2 to 7), 3rd phase of the concrete operational stage (ages from 7 to 11), and 4th phase of the formal operational stage (ages from 11 and over) based on Piaget’s cognitive development theory. Each phase consisted of elements such as power, balance maintenance, transferability, and coordination. Thresholds for the study were built for 6 areas: personal daily activity self‐reliance, social sentiment, language development, cognitive ability, gross motor ability, and fine motor ability development" (2) Control group "Both of the groups performed morning exercise led by a trainer. For the morning exercise, the subjects performed a video‐based exercise program with the assistance of a trainer. They performed the exercise program on their own bed in the morning for 30 minutes in total; the program comprised an introduction, warm‐up, flexibility and muscle exercise, and cool‐down segments, respectively, and included repetition. The actions performed included hand clapping, clapping the back of the hands, trunk twists, back/leg stretches, shoulder movements, and breathing."
Outcomes	**Independence in ADL scales:** modified Barthel Index **Motor function scales:** NR **Measures of balance:** NR **Measures of gait velocity:** NR **Length of stay:** NR **Adverse events:** NR **Other measures:** NR **Time points when outcomes were assessed:** pre‐ and post‐training
Comparison	**Amount of physical rehabilitation in groups:** differs **Categorisation of comparison:** 5. Additional therapy + usual therapy versus usual therapy (dose differs)
Funding & conflicts of interest	**Funding statement:** not reported **Conflict of interest statement:** not reported
Synthesis	**Quantitative synthesis:**Analysis 5.1
Notes	

**Table:** Kim 2016

***Study characteristics***	
Methods	**Study aim:** to investigate how task‐oriented circuit training for the recovery motor control of the lower extremity, balance and walking endurance in subacute stroke inpatient group therapy **Design:** parallel‐group RCT **Country:** Korea, Rep. **Study recruitment and setting:** see Table **Involvement of patients/public/professionals/others:** no
Participants	**Study population (number randomised):** 20 **Inclusion criteria:** First‐ever cerebral stroke involving cortical or subcortical areas confirmed by CT or MRI Hemiparesis with stroke onset ≤ 3 months Able to follow instructions Mild to moderate walking deficit (FAC range between 3 and 46) **Exclusion criteria:** Severe cognitive impairment (K‐MMSE ≤ 10) or aphasia Previous stroke history Not independent ‘sit to stand’ activity (BBS <18) Acute systemic illness or infection Significant orthopaedic condition or pain that could limit participation in exercise Visual impairment or vestibular system deficit that could impair balance **Participants:** detailed in Table **PROGRESS‐plus:** summarised in Table
Interventions	(1) Circuit training group "In the circuit training group, participants participated in 90‐min circuit‐training classes, 5 times per week for 4 weeks. At least two patients under the supervision of one physiotherapist attended all the classes of circuit training. Circuit training consisted of a 5‐min warm‐up period, five classes of 15‐min duration of complex exercises interspersed by 1‐min rest, and a 5‐min cool‐down period. The total duration of the structured protocol was, therefore, 90min. All instructions were standardized and provided to the patients using multimedia and instruction (video and color instruction guideline brochure )by a physical therapist. There were five categories of complex exercises including trunk exercise and active sitting practice, sit‐to‐stand practice, standing and walking practice, aerobic exercise training and strengthening training. Categories 1–3 were core activities including mobility‐related tasks that address key impairments and functional limitations, similar to the study of Dean et al. 3), and categories 4 and 5 were physical fitness training activities. The core activities were active sitting (easiest; category 1), sit‐to‐stand from chairs of various heights (more difficult; category 2), and standing and walking (most difficult; category 3). The core activities were individually adapted for each patient according to their functional levels and progress, such that the level of complexity was appropriate for the ability of the patient. Participants’ progress was continuously monitored. The physical fitness training activities were aerobic exercise training (category 4; performed in the fourth 15min period of the circuit training class) and strengthening training (category 5; performed in the fifth 15min period). Aerobic exercise was performed on a treadmill and workload was increased by increasing treadmill speed or adding an inclination. When a patient could not perform a treadmill activity safely, a stationary bike was used and the workload was increased by increasing bicycle resistance. For strengthening training, participants performed the following targeted movements: hip abduction/flexion, knee flexion/extension and ankle dorsiflexion. A Thera‐Band®(Hygenic Corporation, Akron, Ohio, USA) was used to provide resistance, and participants performed two sets of 8–15 repetitions of each exercise. Progression was achieved by increasing the number of repetitions or load according to subjective tolerance." (2) Bobath group "Participants in the control group received conventional individual physiotherapy for 30min twice a day (total 60min), 5 days a week for the 4 weeks. The content of the individual physiotherapy sessions was based on neurodevelopmental treatment for motor recovery" (referenced to Bobath) "Multidisciplinary rehabilitation programs except for physiotherapy were provided equally to both groups per usual practice"
Outcomes	**Independence in ADL scales:** Modified Barthel Index (Korean version) **Motor function scales:** Fugl Meyer Assessment lower limb score **Measures of balance:** Berg Balance Scale **Measures of gait velocity:** 6‐minute walk test **Length of stay:** NR **Adverse events:** NR **Other measures:** NR **Time points when outcomes were assessed:** baseline and after intervention
Comparison	**Amount of physical rehabilitation in groups:** equivalent (note ‐ circuit training group is 90 minutes/day; control group is 60 minutes/day; however, as this is a comparison of 2 different active approaches, the dose has been categorised as equivalent) **Categorisation of comparison:** 3.(i) Comparison of different approaches (dose equivalent; with no other physical rehab)
Funding & conflicts of interest	**Funding statement:** supported by the research grant of the Jeju National University in 2012 **Conflict of interest statement:** not reported
Synthesis	**Quantitative synthesis:**Analysis 7.1; Analysis 7.2; Analysis 7.3; Analysis 7.4
Notes	

**Table:** Kim 2017

***Study characteristics***	
Methods	**Study aim:** to investigate the effects of the task‐oriented circuit training on balance and gait ability in subacute patients with stroke **Design:** parallel‐group RCT **Country:** Korea, Rep. **Study recruitment and setting:** see Table **Involvement of patients/public/professionals/others:** no
Participants	**Study population (number randomised):** 30 **Inclusion criteria:** Stroke diagnosed by CT or MRI Within 6 months after stroke Able to walk at least 10 m alone or with an aid, but without standby assistance **Exclusion criteria:** Atrial fibrillation Uncontrolled hypertension Symptoms of unstable cardiac disease Recent pulmonary embolism Subacute systemic illness or infection **Participants:** detailed in Table **PROGRESS‐plus:** summarised in Table
Interventions	(1) Task‐oriented circuit training "Subjects in the intervention group participated in a task‐oriented circuit training at the rehabilitation centre, for a total of 50 minutes, five times a week, for 4 weeks. The length of intervention was 4 weeks, 5 sessions of weekly training, for a total of 20 sessions. All training sessions were organized into groups, with at least 2–3 people/group, and were conducted by two physical therapists (with 3 years of experience in stroke rehabilitation). Task‐oriented circuit training program was modified according to the studies by Dean et al.6, and Outermans et al.9, and incorporated 10 workstations. It consisted of task‐oriented activities for improving balance, walking competency, and respiration ability. At all stations patients practiced for 3 minutes, and this session was followed by a 1 minute transfer to the next station. And stretching time (5 minutes) was offered before and after the exercise. The total exercise time was 50 minutes.These core practice activities were: sit to stand; stepping; tandem standing; one leg standing; and reaching; walking practice included obstacles, reaching, slope and stairs. These core activities were individually adjusted for each subject by the treating therapist. Progress was assessed as required, such that the level of difficulty, complexity, and dosage (number of repetitions) matched the ability of each individual 10). The Borg Scale of Perceived Exertion was used to adjust the effort intensity in order to maintain an “hard” level 11). Details about exercise intensity and/or repetitions performed at each station were recorded for each participant." (2) Task‐oriented exercise "The control group was performed through exercise focused on task‐oriented exercise, such as strengthening exercise (resistance exercise), standing balance (using varying methods), and functional activities for gait improvement." "Both the groups received neuro‐developmental treatment (postural control exercise, resistance exercise, and functionalactivity exercise) for approximately 1 hour per day. In addition, they received some other therapies, including occupationaland speech therapy, as needed."
Outcomes	**Independence in ADL scales:** NR **Motor function scales:** NR **Measures of balance:** Berg Balance Scale **Measures of gait velocity:** timed up and go test; 6‐minute walk test **Length of stay:** NR **Adverse events:** NR **Other measures:** Functional Ambulation Categories **Time points when outcomes were assessed:** before and after treatment
Comparison	**Amount of physical rehabilitation in groups:** unclear/not stated, but assumed to be equivalent **Categorisation of comparison:** 4. Comparison of similar approaches (dose equivalent)
Funding & conflicts of interest	**Funding statement:** not reported **Conflict of interest statement:** not reported
Synthesis	**Qualitative synthesis:** Studies comparing similar approaches were not combined within statistical analyses. Note: main difference being the mode of delivery (i.e. circuit/group vs individualised).
Notes	

**Table:** Kim 2018

***Study characteristics***	
Methods	**Study aim:** to investigate the effects of co‐ordinative locomotor training on coordination and gait in people with chronic stroke **Design:** parallel‐group RCT **Country:** Korea, Rep. **Study recruitment and setting:** see Table **Involvement of patients/public/professionals/others:** no
Participants	**Study population (number randomised):** 13 **Inclusion criteria:** Diagnosed with stroke > 6 months No cognitive impairment (MMSE‐K ≥ 24) No abnormality in visual and auditory senses and proprioception Fair+ or higher on manual muscle tests of the upper and lower limbs on the affected side Able to walk independently with/without assistive devices **Exclusion criteria:** Orthopaedic disorders in the lower limbs Other diseases that may affect gait Participating in another rehabilitation programme **Participants:** detailed in Table **PROGRESS‐plus:** summarised in Table
Interventions	(1) Co‐ordinative locomotor training (CLT) "During the intervention period, the experimental group was provided with a CLT program consisting of four basic postures (supine, bridge, sitting, and standing), along with gait training. The total training time was 30 min per day: 20 min for the four postures (5 min each) and 10 min for gait training. The sprinter and skater patterns were performed for 2 min and 30 sec for each pattern in supine, bridge, sitting, and standing postures. The sprinter pattern was performed by imitating a runner’s movement, including the flexion‐adduction‐external rotation pattern in the paralyzed upper limb and the extension‐abduction‐internal rotation pattern in the lower limb of the same side. For the opposite upper limb, the extension‐abduction‐internal rotation pattern was applied, and for the lower limb of the same side, the flex‐ion‐adduction‐external rotation pattern was applied. The skater pattern was performed by imitating the skater’s actions, including the flexion‐abduction‐external rotation pattern in the paralyzed upper limb and the extension‐adduction‐external rotation pattern in the lower limb of the same side. For the upper limb on the opposite side, the extension‐adduction‐internal rotation pattern was applied, while the flexion‐abduction‐internal rotation pattern was applied on the lower limb on the same side. If the patient was unable to raise the paralyzed upper or lower limb to execute the pattern properly in each posture, therapist’s assistance was provided." (2) Neurodevelopmental treatment "During the intervention period, a physical therapist with clinical experience of 2 or more years performed the 30‐min treatment for central‐nervous‐system development in the control group. The intervention was conducted with focus on the mat and gait training. Patients practiced the transitional posture to be able to change from the lying to standing posture, with therapist’s assistance if necessary. After exercising the shifting of the weight to the front, back, left, and right in standing posture, gait training was carried out by removing one foot at a time with assistance" "interventions were provided to all patients in twelve 30‐min sessions during a 4‐week period (3 sessions aweek). "
Outcomes	**Independence in ADL scales:** NR **Motor function scales:** NR **Measures of balance:** NR **Measures of gait velocity:** 10‐m walk test, timed up and go test **Length of stay:** NR **Adverse events:** NR **Other measures:** gait assessment and intervention tool (GAIT), gait parameters **Time points when outcomes were assessed:** pre‐ and post‐treatment
Comparison	**Amount of physical rehabilitation in groups:** equivalent **Categorisation of comparison:** 3.(i) Comparison of different approaches (dose equivalent; with no other physical rehab)
Funding & conflicts of interest	**Funding statement:** not reported **Conflict of interest statement:** none
Synthesis	**Quantitative synthesis:**Analysis 7.10
Notes	

**Table:** Kim 2021

***Study characteristics***	
Methods	**Study aim:** examine the effects of task‐specific training combined with cognitive sensorimotor exercise on proprioception, spasticity and gait speed in people with stroke **Design:** multi‐arm RCT (3 arms) **Country:** Korea, Rep. **Study recruitment and setting:** see Table **Involvement of patients/public/professionals/others:** no
Participants	**Study population (number randomised):** 45 **Inclusion criteria:** Diagnosis of stroke with onset of ≥ 6 months Able to walk at least 10 m MMSE‐Korean version ≥ 24 Able to communicate and follow the instructions Brunnstrom exercise recovery stages of 3–4 or higher No problem walking due to ankle joint contractures No sensory deficiencies of the lower extremities Able to provide informed consent **Exclusion criteria:** Vestibular problems Cerebellar‐related diseases Visual or hearing impairments; Unable to participate due to severe cognitive decline or aphasia Unilateral neglect **Participants:** detailed in Table **PROGRESS‐plus:** summarised in Table
Interventions	(1) Task specific training (TST) combined with cognitive sensorimotor exercise (CSE) "performed sense, trunk stability, lower‐extremity movements, and gait training in a sitting or standing position by applying CSE and the TST. In this study, 30min of CSE and 30min of the TST were performed five times per week for eight weeks. Two physical therapists performed treatment to reduce treatment bias. One physical therapist with more than five years of clinical experience performed the CSE exercises. The TST training was conducted by the researcher who has more than 5 years of clinical experience. The participants were trained to recognize their bodies using visual and somatosensory techniques. The CSE consisted of proprioceptive training, tactile training, heel pressure, and space tasks. For CSE using a spatial task, exercise programs were given to distinguish distance and direction. The CSE was applied for a total of 30 min for 5min each with the subjects." Cognitive sensorimotor exercise program is detailed in table 1 ‐ comprises of 5 minutes each of proprioceptive training, tactile stimulation training, pressure stimulation training, and two spatial tasks. "The 30 min TST consisted of the following: 10 min of trunk stability and sit to stand training while controlling the movement trunk and lower extremities [25], 10 min of lower extremity movements and gait training while controlling scapular movement [26,27], 10 min of progressive body weight support treadmill training while controlling the movement of the lower extremities" (see note below about TST). (2) Task specific training (TST) combined with conventional physical therapy "The Experimental II group performed trunk stability and sit‐to‐stand training while controlling the movement of the trunk and lower extremities, lower extremity movements and gait training while controlling scapular movement, and progressive body weight support treadmill training while controlling the movement of the lower extremities. Experimental II group received 30 min of the TST and 30 min of conventional physical therapy five times per week for eight weeks." NOTE: in the trials registry, this (TST) intervention is described as "Bobath therapy motor program". Stating that this: "targets trunk stabilisation and the sit‐to‐stand motion, lower extremity movement and gain training, and progressive body weight support treadmill training". (3) Conventional physical therapy "The conservative physical therapy program consisted of ROM exercises, stretching exercises, upper‐ and lower‐extremity muscle strengthening exercise, ground gait training, bike exercises, balance training, and superdynamics exercises. The control group received conservative physical 30 min twice daily, five times per week for 8 weeks."
Outcomes	**Independence in ADL scales:** NR **Motor function scales:** NR **Measures of balance:** NR **Measures of gait velocity:** 10‐m walk test **Length of stay:** NR **Adverse events:** NR **Other measures:** proprioception text, Composite Spasticity Score, Gastronemius muscle tone **Time points when outcomes were assessed:** before and after intervention
Comparison	**Comparisons relevant to this review** Comparison (a) ‐ Group (1) vs Group (2) Comparison (b) ‐ Group (2) vs Group (3) **Comparison (a)** **Amount of physical rehabilitation in groups:** equivalent **Categorisation of comparison:** 3. (ii) Comparison of different approaches (dose equivalent; with both/all groups also receiving other physical rehab) **Comparison (b)** **Amount of physical rehabilitation in groups:** differs **Categorisation of comparison:** 5. Additional therapy + usual therapy versus usual therapy (dose differs)
Funding & conflicts of interest	**Funding statement:** supported by the National Research Foundation Korea (NRF) grant funded by the Korean government (MSIT) (No. 2019R1G1A1011657) **Conflict of interest statement:** none
Synthesis	**Quantitative synthesis:**Analysis 5.4; Analysis 7.10
Notes	

**Table:** Knox 2018

***Study characteristics***	
Methods	**Study aim:** to evaluate if a low‐dose task‐oriented gait training approach delivered in a circuit training mode and tailored to South African needs could promote locomotor recovery and walking competency in persons post stroke health sector **Design:** multi‐arm RCT (3 arms) **Country:** South Africa **Study recruitment and setting:** see Table **Involvement of patients/public/professionals/others:** no
Participants	**Study population (number randomised):** 144 **Inclusion criteria:** Aged 20 to 79 years > 6 months after the onset of first ischaemic or haemorrhagic stroke Medically stable Able to walk at least 10 m at less than 1.1 m/s without assistance with/without assistive devices or supervision Able to understand and follow instructions Live close enough to attend the rehabilitation sessions **Exclusion criteria:** Conditions that could interfere with post‐stroke recovery (e.g. active HIV neurological disorders, or musculoskeletal injuries interfering with gait rehabilitation) **Participants:** detailed in Table **PROGRESS‐plus:** summarised in Table
Interventions	(1) Task group "The Task intervention was conducted for 4–6pairs of participant and caregiver per session by thef irst author (M.K.). It consisted of a series of six exercises (5,6) based on the movement science approach (18), focused on improving strength, balance, and task performance while standing and walking, and included an endurance walking station (5–7). Chairs, tables, and walls were used to ensure safety and provide support as necessary. Exercises were progressed by reducing the use of these supports and increasing the complexity of the tasks. The caregiver was encouraged to give the correct assistance to the participant in the execution of the tasks and was directed to assist the participant in working on a progressively more difficult structured walking program at home between the formal therapy sessions. All of the participants and their caregivers attended all of the six sessions required of them over the 12‐week period that the intervention was undertaken." (2) Strength group "The Strength intervention was delivered by a physiotherapist, experienced in neurological rehabilitation, after training with the first author. It included 10 exercises while sitting and lying targeting the major muscles in the lower extremities. Each exercise consisted of three sets of 10 repetitions and progressed as per the participants’ performance and feedback (5). Resistance was provided with gravity, free weights, elastic bands, and balls." "Sessions for the Task and Strength groups were available every week over a 12‐week period. The participants attended six 1‐hour sessions over this 12‐week period. (3) Control group "The participants in the Control intervention attended only one 90‐minute educational session on stroke management that included 20 minutes of exercises, provided by an occupational or physiotherapist employed in stroke rehabilitation by the hospital."
Outcomes	**Independence in ADL scales:** NR **Motor function scales:** NR **Measures of balance:** Berg Balance Scale **Measures of gait velocity:** 6‐minute walk test, 10‐m gait speed test, timed up and go **Length of stay:** NR **Adverse events:** NR **Other measures:** NR **Time points when outcomes were assessed:** baseline, 12 weeks post intervention, follow‐up 12 weeks later
Comparison	**Comparisons relevant to this review** Comparison (a) ‐ Group (1) vs Group (3), AND Group (2) vs Group (3) Comparison (b) ‐ Group (1) vs Group (2) **Comparison (a)** **Amount of physical rehabilitation in groups:** differs **Categorisation of comparison:** 1. Physical rehabilitation versus no or limited physical rehabilitation (dose differs) (limited = < 50% of dose) **Comparison (b)** **Amount of physical rehabilitation in groups:** equivalent **Categorisation of comparison:** 3.(i) Comparison of different approaches (dose equivalent; with no other physical rehab)
Funding & conflicts of interest	**Funding statement:** Medical Research Council of South Africa and the Fonds de Recherche du Québec‐Santé (FRQS) **Conflict of interest statement:** none
Synthesis	**Quantitative synthesis:**Analysis 1.3; Analysis 1.4; Analysis 2.4; Analysis 7.8; Analysis 7.10; Analysis 8.6; Analysis 8.8
Notes	

**Table:** Koç 2015

***Study characteristics***	
Methods	**Study aim:** to determine whether a structured, physiologically based exercise program for subacute stroke produces larger gains than those attributable to spontaneous recovery and usual care **Design:** parallel‐group RCT **Country:** Turkey **Study recruitment and setting:** see Table **Involvement of patients/public/professionals/others:** no
Participants	**Study population (number randomised):** 134 **Inclusion criteria:** 30 to 90 days after ischaemic stroke Baseline BI scores of 60 to 80 Ambulatory with supervision and/or an assistive device Living at home Living within Yozgat **Exclusion criteria:** Medical condition that may interfere with outcome assessments or limit ability to participate in the exercise programme Memory and communication impairment Receptive aphasia that may interfere with the ability to follow a 3‐step command Serious cardiac symptoms or other health problems that could limit exercising **Participants:** detailed in Table **PROGRESS‐plus:** summarised in Table
Interventions	(1) Home‐exercises "The home‐based exercises were designed to improve strength, balance and endurance and to encourage use of the affected extremity. It was a home‐based exercise program provided by a nurse researcher. The program included two visits a week for 12 weeks, and the patients were instructed to continue the exercise program. Each exercise session lasted 1h. Exercise sessions were divided into 3 blocks preceded by a 15‐min warm up session of stretching and flexibility exercises. The first block included assisted and resisted exercises to the major muscle groups of the upper and lower extremities. The second block included active‐assisted ROM exercises perfored in a way to enable a slight range of motion for the purpose of performing a voluntary movement; the movement patterns included (1) flexion, abduction, and external rotation of the shoulder with the elbow extended and with wrist and finger extension; (2) extention, adduction and internal rotation of the shoulder and the elbow extended and with finger and wrist extention; (3) flexion, adduction, external rotation of the hips with knee flexion and ankle dorsiflexion; and (4) extension, abduction, internal rotation of the hips with knee extension and ankle plantar flexion. The patients were then encouraged to increase their exercise time until they could exercise continuously for 15 minutes. The final block included a progressive walking program and relaxation. A detailed protocol of this intervention is available from the authors" (2) Usual care No intervention ‐ "received home visits by the research staff every 4 weeks for health education and to check their vital signs"
Outcomes	**Independence in ADL scales:** Barthel Index **Motor function scales:** NR **Measures of balance:** NR **Measures of gait velocity:** NR **Length of stay:** NR **Adverse events:** NR **Other measures:** NR **Time points when outcomes were assessed:** baseline and 4, 8, and 12 weeks post intervention (length of intervention is 12 weeks)
Comparison	**Amount of physical rehabilitation in groups:** differs **Categorisation of comparison:** 1. Physical rehabilitation versus no or limited physical rehabilitation (dose differs) (limited = < 50% of dose)
Funding & conflicts of interest	**Funding statement:** not reported **Conflict of interest statement:** not reported
Synthesis	**Quantitative synthesis:**Analysis 1.1
Notes	

**Table:** Krawczyk 2014

***Study characteristics***	
Methods	**Study aim:** to compare two therapeutic programs (open‐chain vs closed chain exercises) in stroke survivors **Design:** parallel‐group RCT **Country:** Poland **Study recruitment and setting:** see Table **Involvement of patients/public/professionals/others:** no
Participants	**Study population (number randomised):** 51 **Inclusion criteria:** Patients within 3 months of first ischaemic stroke Willing to cooperate Able to walk 10 m independently without aids **Exclusion criteria:** Neglect syndrome (diagnosed clinically by neuropsychologist) Deep dysphasia (3 points or more on the Goodglass‐Kaplan Aphasia Examination) Medical contraindications to perform intensive training **Participants:** detailed in Table **PROGRESS‐plus:** summarised in Table
Interventions	(1) "Close Chain" rehabilitation programme "1. Kinesiotherapy conducted mainly in standing or sitting positions. 2. In the first period of therapy (when motordeficit was more severe) most of the exercises were done in isokinetic condition (co‐contraction of antagonistic muscles) and exercises with mobility of the joints were introduced gradually, according to degree of motor recovery. Increasing the range of motion depended on the improved patient’s motor control. 3. Most exercises were of "closed chain” type; i.e., when the extremity (either arm or leg) was stabilized distally to stable surface (ironing board, wall, coach), object (ladder, spade) or other part of the body (clasped hands). 4. Only exercises simultaneously engaging whole paretic side of the body were performed." (2) Standard rehabilitation program ("Open chain" exercises) "1. Kinesiotherapy conducted in lying position of the patient (at least for 50% of total time of treatment). Standing position was used only when standing or walking skills (activity level) were practised. 2. Most of the time the trunk was stabilized, and the limbs were moving freely during exercise. The type of exercises was independent of the amount of motor deficit. 3. Lack of exercises simultaneously engaging whole paretic side of the body." "All patients participated in one of the two 12 week rehabilitation programs: standard one, or “closed chain” one. At the beginning all patients were treated for 6 weeks at the inpatient neurorehabilitation department, and then received physiotherapy for 6 weeks at the outpatient neurorehabilitation department. Individual physiotherapy in both groups was based on movement therapy, and lasted for 2 hours daily."
Outcomes	**Independence in ADL scales:** NR **Motor function scales:** Fugl‐Meyer lower limb subscale, Rivermead Motor Assessment ‐ lower extremity **Measures of balance:** Berg Balance Scale **Measures of gait velocity:** speed, as percentage of reference value **Length of stay:** NR **Adverse events:** NR **Other measures:** size of brain lesion using CT scans, gait analysis **Time points when outcomes were assessed:** start of study; after 6 weeks of programme; after 12 weeks at end of programme
Comparison	**Amount of physical rehabilitation in groups:** equivalent **Categorisation of comparison:** 4. Comparison of similar approaches (dose equivalent)
Funding & conflicts of interest	**Funding statement:** supported by the research grant 0521/P01/2007/32 of the Polish Ministry of Science and Higher Education **Conflict of interest statement:** not reported
Synthesis	**Qualitative synthesis:** Studies comparing similar approaches were not combined within statistical analyses. (Note: Fugl‐Meyer had normal distribution and data are presented as means and SD; Rivermead, Berg balance, and speed are presented as medians, minimum and maximum.)
Notes	No group sizes reported. Analysis carried out using sizes of 25 for each group.

**Table:** Krukowska 2016

***Study characteristics***	
Methods	**Study aim:** "to evaluate the effect of the NDT‐Bobath and PNF methods on the field support and total path length measure foot pressure after stroke" **Design:** multi‐arm RCT (4‐arm) **Country:** Poland **Study recruitment and setting:** see Table **Involvement of patients/public/professionals/others:** no
Participants	**Study population (number randomised):** 72 **Inclusion criteria:** Stroke survivors treated in the Rehabilitation Clinic within 6 months onset **Exclusion criteria:** More than one stroke or other neurological diseases with CNS damage Not able to remain standing Incomplete or no logical‐verbal contact Pusher syndrome and/or hemineglect syndrome Limited range of motion in the lower extremities due to osteoarthritis or amputation **Participants:** detailed in Table **PROGRESS‐plus:** summarised in Table
Interventions	(1a) NDT‐Bobath method ‐ stroke survivors with right‐sided paresis (1b) NDT‐Bobath method ‐ stroke survivors with left‐sided paresis "The NDT‐Bobath method it is a key factor in the correct sensation of movement, and the freeing of posture and movement patterns under the influence of incorrect posture reflex, through priming based on correct sitting and balance responses. An appropriate support, obtained by moving the key points (head, neck and torso, shoulder girdle and upper limbs, hip girdle and lower limbs), initiates the activity of restoring reflex posture, balance and self‐use by the patient and allows the accumulation of normal sensorimotor sensations..." (2a) PNF method ‐ stroke survivors with right‐sided paresis (2b) PNF method ‐ stroke survivors with left‐sided paresis "In the PNF method the main goal involves re‐education of movement activities, which are assisted with previous experience and derived from the patterns of motor development as well as multi‐sensory impulses. The procedure is based on the summation of various types of afferent stimuli (strong multi sensory stimulation)" "35 therapy sessions, everyday for a period of six weeks (except for Sundays). The classes were conducted individually by professional physiotherapists."
Outcomes	**Independence in ADL scales:** NR **Motor function scales:** NR **Measures of balance:** NR **Measures of gait velocity:** NR **Length of stay:** NR **Adverse events:** NR **Other measures:** centre of pressure measures **Time points when outcomes were assessed:** after treatment
Comparison	**Amount of physical rehabilitation in groups:** equivalent **Categorisation of comparison:** 3.(i) Comparison of different approaches (dose equivalent; with no other physical rehab)
Funding & conflicts of interest	**Funding statement:** not reported **Conflict of interest statement:** none
Synthesis	**Qualitative synthesis:** no relevant outcome measures assessed
Notes	

**Table:** Kuberan 2017

***Study characteristics***	
Methods	**Study aim:** evaluate Task Oriented Training with sensory manipulation to enhance balance and functional mobility in chronic stroke **Design:** parallel‐group RCT **Country:** India **Study recruitment and setting:** see Table **Involvement of patients/public/professionals/others:** no
Participants	**Study population (number randomised):** 26 **Inclusion criteria:** First episode of unilateral supratentorial stroke Brunnstrom’s recovery stage 5 or above for the lower limb Able to stand for 5 mins without support MMSE ≥ 23 BBS of 40 or above **Exclusion criteria:** Any severe limitation that would limit participation in intervention or functional assessments Presence of vestibular disorders, vertigo or somatosensory deficits Presence of visual problems, e.g. homonymous hemianopsia Presence of severe orthopaedic conditions involving the lower limbs **Participants:** detailed in Table **PROGRESS‐plus:** summarised in Table
Interventions	(1) Task‐oriented training "task‐oriented training with manipulations of sensory inputs and provision of sensory conflict for the trunk and lower limb...." "1. Sitting on a swiss ball and reaching out for objects(10repetitions) 2. Sit‐to‐stand from a stool (10 repetitions) 3. Performing double legged stance for 10 counts (normal base of support) and then with decreased base of support for 10 counts 4. Performing tandem stance for 10 counts (If the patients finds tandem stance difficult, then he can maintain the same by increasing the step length) 5. Performing unilateral stance for 10 counts with or without minimal support 6. Reciprocal leg flexion and extension in standing i.e. marching with or without minimal support 7. Stepping forward, backward and sideways on a stepper 8. Stepping over boards of various heights (6 inches, 8 inches, 10 inches) 9. Standing up from a chair, walking four steps, performing a bilateral stool touch and walking back to the chair. Forward and backward walking in tandem walking pattern" (2) Conventional physiotherapy "1. Facilitatory techniques like quick icing, quick stretching, muscle belly tapping, joint approximation for paretic muscles and Inhibitory techniques like prolonged icing, sustained stretching for spastic muscles. 2. Strengthening for weak muscles. 3. Bed mobility exercises with emphasis on transitions. 4. Balance training in Single limb Stance, Reaching. 5. Gait training. 6. Functional training involving activities of daily living" "intervention period was 15 sessions, 5 days a week for 3 weeks."
Outcomes	**Independence in ADL scales:** NR **Motor function scales:** NR **Measures of balance:** NR **Measures of gait velocity:** Timed Up and Go Test **Length of stay:** NR **Adverse events:** NR **Other measures:** Dynamic Gait Index, Fall Efficacy Scale **Time points when outcomes were assessed:** baseline and after treatment
Comparison	**Amount of physical rehabilitation in groups:** equivalent **Categorisation of comparison:** 3.(i) Comparison of different approaches (dose equivalent; with no other physical rehab)
Funding & conflicts of interest	**Funding statement:** not reported **Conflict of interest statement:** not reported
Synthesis	**Quantitative synthesis:**Analysis 7.10
Notes	

**Table:** Kumaran 2015

***Study characteristics***	
Methods	**Study aim:** "to determine the effect of task and context based exercise program using ICF model on quality of life in patients with stroke" **Design:** parallel‐group RCT **Country:** India **Study recruitment and setting:** see Table **Involvement of patients/public/professionals/others:** no
Participants	**Study population (number randomised):** 202 **Inclusion criteria:** First stroke discharged from hospital Ischaemic stroke Aged 30 to 65 years; both sexes; Minimum of 3 months post stroke duration Able to ambulate at least 5 m with supervision or guarding Able to understand instructions and follow simple commands **Exclusion criteria:** Present history of severe, uncontrolled, or unstable cardiac disease Other systemic disorders for which exercise is contraindicated Terminally ill Hearing and visually challenged Other coexisting conditions that would interfere with outcome assessments or participation in treatment regimens **Participants:** detailed in Table **PROGRESS‐plus:** summarised in Table
Interventions	(1) Exercise training First 8 weeks: "Participants in the experimental group received supervised exercise program. The exercise program focusing on treating body structure/function, activity and participation domains of ICF included the following guidelines: Body structure/function training: Muscle power through functional training; muscle tone through weight bearing exercises; control of voluntary movement through eccentric control training using motor learning principles; and muscle tightness through active elongation exercises. Activity & participation training (based on motor learning principles): Changing body position through training of turning, getting up from lying and sit to stand; Maintaining a body position through sitting balance training and standing balance training; Walking and moving around through walking in different contexts; negotiation of obstacles, narrow spaces, slopes, steps, stairs, uneven terrain; implementation of dual task and multiple tasks for e.g.,walking while talking; walking, memorizing and carrying an object such as glass; Using transportation through training. Motor learning principles such as task analysis, training of components of task, whole task training, repetition, feedback etc., were followed during intervention regimen. Progression of the exercises were done individually depends on the status and response of the participants." "...one hour, 3 times a week for 8 weeks" Weeks 8 to 12: "After a period of eight weeks, the participants in the experimental group received an additional training program for a period of four weeks that focused on assisting or modifying the contextual factors (environmental factors). This includes an education program to participant and participant’s family, caregiver training,provision of assistive devices and modification of architectural barriers" (2) Conventional physiotherapy "Participants in the control group received conventional exercise program which includes self‐assisted exercises, pelvic bridging exercises, reaching activities in sitting and standing, weight shifting in sitting and standing and walking. In addition, they were given instructions and education about exercise performance and safety. The participants were asked to perform the exercises under the supervision of caregiver and were advised to continue with the same exercise program for throughout the period of 12 weeks."
Outcomes	**Independence in ADL scales:** NR **Motor function scales:** NR **Measures of balance:** Berg Balance Scale **Measures of gait velocity:** 5‐Metre Timed Walk test, Six‐Minute Walk test **Length of stay:** NR **Adverse events:** NR **Other measures:** Stroke Impact Scale score, Motricity Index score, impact on participation and autonomy questionnaire score, falls efficacy scale‐international score **Time points when outcomes were assessed:** pre 8 weeks, post 8 weeks, post 12 weeks, post 16 weeks
Comparison	**Amount of physical rehabilitation in groups:** equivalent **Categorisation of comparison:** 3. (ii) Comparison of different approaches, (dose equivalent; with both/all groups also receiving other physical rehab) (Note it is unclear whether the dose in the treatment groups was equivalent, or whether the exercise programme received a greater amount than the conventional physiotherapy).
Funding & conflicts of interest	**Funding statement:** states there was no monetary support (conducted as a PhD study) **Conflict of interest statement:** not reported
Synthesis	**Quantitative synthesis:**Analysis 7.3; Analysis 7.4; Analysis 8.3; Analysis 8.4
Notes	CTRI/2010/091/000278

**Table:** Kunkel 2013

***Study characteristics***	
Methods	**Study aim:** pilot feasibility study assessing the delivery of functional electrical stimulation to the gluteus maximus and quadriceps femoris for acute stroke survivors in a hospital rehabilitation setting **Design:** multi‐arm RCT (3‐arm) **Country:** United Kingdom **Study recruitment and setting:** see Table **Involvement of patients/public/professionals/others:** no
Participants	**Study population (number randomised):** 21 **Inclusion criteria:** Recruit participants as soon as possible after admission to hospital when they were medically stable Able to follow simple instructions and give informed consent First stroke fulfilling the WHO definition Able to stand for 10 sec unsupported Independently mobile prior to stroke Demonstrate a need for a physiotherapy programme targeted at improving standing balance and weight transfer **Exclusion criteria:** Able to maintain single limb stance for 10 sec and were able to walk independently without the use of an assistive device Medical problems potentially adversely affected by FES Skin conditions, or had allergies **Participants:** detailed in Table **PROGRESS‐plus:** summarised in Table
Interventions	(1) Exercise "exercise program comprising evidence‐based exercises aimed at: • improving standing balance ability and stability (33,34); • increasing weight transferred onto the affected lower limb (33–35). Exercises were documented, adjusted, and progressed according to ability, with participants starting the exercise training in a supported or unsupported parallel stance and progressing to supported and unsupported stride standing positions; the number of repetitions was increased as appropriate." (2) Exercise + FES "FES was delivered in conjunction with the above‐outlined exercise program. Stimulation was initiated and terminated via a pressure switch activated either by the participant, in which case it was placed in the shoe, or manually by the treating therapist throughout the exercise program in an on/off regimen while practicing specific balance tasks. Following full assessment, FES was applied using a two‐channel Odstock stimulator (O2CHSII and CE02104) from Odstock Medical (36). Stimulation was applied via surface electrodes using Pals Neurostimulation Electrodes (model 879300, 7‐cm round gel pads) (37). Two electrodes each were positioned over the gluteus maximus and over quadriceps femoris muscles (Fig. 1), according to the manufacturer’s guidelines. Stimulation was delivered at 40 Hz at a level between 17.8 and 33 mA for the quadriceps femoris muscle and between 17.8 and 35 mA for gluteus maximus muscles, using an asymmetrical biphasic waveform and an average pulse width of 162mS. In addition, arising and falling edge ramp time was set to help avoid stretch reflexes, delay the start of, but prevent the sudden end of a muscle contraction in order to make the stimulation more comfortable. The level of stimulation was set with the aim of producing a muscle contraction resulting in a joint movement (JM) within the limit of discomfort. The treating therapist documented treatment frequency, duration, and content, as well as FES parameters. " (3) Usual care "Usual Care: was delivered by National Health Service therapists who documented the type and time spent on specific treatments. Therapists choose techniques that enable patients to move independently, such as standing and walking practice, predominantly based on the Bobath approach, but included techniques such as motor relearning. Usual care was described in a national survey of approaches and treatment packages used by UK physiotherapists" "Participants in all three groups of our trial received usual care in their respective rehabilitation settings." "length of the intervention was set as two weeks (starting as soon as possible post stroke onset) with a two‐week follow‐up period, with the aim of delivering four‐hour‐long sessions per week (a maximum of eight sessions). The intervention was delivered in hospital but was continued in the community if a participant was discharged before trial completion" Comparison of relevance to this review = (1) exercise vs (3) usual care
Outcomes	**Independence in ADL scales:** NR **Motor function scales:** Rivermead Mobility Index **Measures of balance:** Berg Balance Scale **Measures of gait velocity:** 10 m walk (normal and fast) **Length of stay:** NR **Adverse events:** NR **Other measures:** symmetry of weight distribution **Time points when outcomes were assessed:** baseline, 1 to 3 days post intervention, 2 weeks post intervention
Comparison	**Amount of physical rehabilitation in groups:** differs **Categorisation of comparison:** 5. Additional therapy + usual therapy versus usual therapy (dose differs)
Funding & conflicts of interest	**Funding statement:** supported by a grant from the Stroke Association **Conflict of interest statement:** none
Synthesis	**Quantitative synthesis:**Analysis 5.2; Analysis 5.3; Analysis 5.4; Analysis 6.2; Analysis 6.3; Analysis 6.4
Notes	

**Table:** Kwakkel 2002

***Study characteristics***	
Methods	**Study aim:** investigated the effects of different durations of rehabilitation sessions for upper and lower extremities on the recovery of interlimb co‐ordination in hemiplegic gait in stroke patients **Design:** multi‐arm RCT (3‐arms) **Country:** The Netherlands **Study recruitment and setting:** see Table **Involvement of patients/public/professionals/others:** no
Participants	**Study population (number randomised):** 53 **Inclusion criteria:** Primary, first‐ever stroke in the territory of the middle cerebral artery as revealed by CT or MRI Aged 30 to 80 years Impaired lower extremities (LE) and upper extremities (UE) motor function as assessed with the Motricity Index (i.e. scores < 100 points for each paretic limb) Unable to walk without assistance on admission No complicating medical history on the basis of review of medical records, e.g. cardiac, pulmonary or neurological disorders No severe deficits in communication, memory, or understanding Provided written or verbal informed consent Sufficiently motivated to participate **Exclusion criteria:** Not reported **Participants:** detailed in Table **PROGRESS‐plus:** summarised in Table
Interventions	(1) Rehabilitation group (n = 17) "focused on the recovery of tasks such as turning over and maintaining sitting and standing balance. In addition, the LE intervention was designed to improve the symmetry in interlimb coordination during walking" "The guidelines were based on evidence‐based practice patterns derived from findings reported in 165 intervention studies in the field of stroke rehabilitation. We used what we believe is an eclectic approach based on research indicating that subjects’ practice of motor skills needs to be both task and context specific" Length of intervention period: for a period of 20 weeks post stroke ("from week 20 onward, type of treatment and its duration [were] determined by the physical therapists and occupational therapists involved, on average 3 times half an hour a week") Number of sessions and length of individual sessions: 30 minutes of LE training, 5 days a week. In addition, all 3 groups participated daily in a basic treatment programme of 15 minutes of LE exercises and 15 minutes of UE exercises, as well as a weekly 90‐minute session of ADL training administered by an occupational therapist. Intervention provider: physical therapists and occupational therapists (2) Attention control group (n = 18) "focused on the improvement of grasping, reaching, leaning, and dressing and hair combing" Length of intervention period: for a period of 20 weeks post stroke ("from week 20 onward, type of treatment and its duration [were] determined by the physical therapists and occupational therapists involved, on average 3 times half an hour a week") Number of sessions and length of individual sessions: 30 minutes of LE training, 5 days a week. In addition, all 3 groups participated daily in a basic treatment programme of 15 minutes of LE exercises and 15 minutes of UE exercises, as well as a weekly 90‐minute session of ADL training administered by an occupational therapist. Intervention provider: physical therapists and occupational therapists (3) No physical rehabilitation group (n = 18) "Immobilisation of the paretic LE and UE by means of an inflatable pressure splint, which was applied for 30 minutes in a lying position, 5 days a week" Length of intervention period: for a period of 20 weeks post stroke ("from week 20 onward, type of treatment and its duration [were] determined by the physical therapists and occupational therapists involved, on average 3 times half an hour a week") Number of sessions and length of individual sessions: 30 minutes of LE training, 5 days a week. In addition, all 3 groups participated daily in a basic treatment programme of 15 minutes of LE exercises and 15 minutes of UE exercises, as well as a weekly 90‐minute session of ADL training administered by an occupational therapist Intervention provider: physical therapists and occupational therapists
Outcomes	**Independence in ADL scales:** NR **Motor function scales:** NR **Measures of balance:** NR **Measures of gait velocity:** comfortable and maximal walking speeds **Length of stay:** NR **Adverse events:** NR **Other measures:** mean continuous relative phase **Time points when outcomes were assessed:** "patients were assessed during the first 10 weeks on a weekly basis and biweekly from week 10 to week 20. With exception of kinematic measurements, final assessment took place at 26 weeks poststroke"
Comparison	**Comparison a: Group (1) versus Group (3)** **Amount of physical rehabilitation in groups:** differs **Categorisation of comparison:** 1. Physical rehabilitation versus no or limited physical rehabilitation (dose differs) (limited = < 50% of dose) **Comparison b: Group (1) versus Group (2)** **Amount of physical rehabilitation in groups:** differs **Categorisation of comparison:** 2. Physical rehabilitation versus attention control (dose of physical rehab differs; dose of intervention equivalent)
Funding & conflicts of interest	**Funding statement:** not reported **Conflict of interest statement:** not reported
Synthesis	**Qualitative synthesis:** no outcomes included in analysis because it was unclear at which time points the data were collected
Notes	Intervention 2 comprised upper limb training. Although upper limb training might be classed as a subcomponent of functional task training, for the purposes of comparisons in this review we have categorised this intervention as an attention control. This is because the upper limb training was delivered alone (i.e. no other subcomponents of functional task training were delivered), and therefore no active treatment was aimed at lower limb or balance outcomes.

**Table:** Kwakkel 2008

***Study characteristics***	
Methods	**Study aim:** "to analyse the effect of task oriented circuit training compared with usual physiotherapy interms of self reported walking competency for patients with stroke discharged from a rehabilitation centre to their own home" **Design:** parallel‐group RCT **Country:** The Netherlands **Study recruitment and setting:** see Table **Involvement of patients/public/professionals/others:** no
Participants	**Study population (number randomised):** 250 **Inclusion criteria:** Verified stroke according to the WHO definition Able to walk a minimum of 10 m without physical assistance (functional ambulation categories ≥ 3) Discharged home from a rehabilitation centre Needed to continue physiotherapy during outpatient care to improve walking competency or physical condition, or both Able to give informed consent Motivated to participate in a 12‐week intensive programme of physiotherapy **Exclusion criteria:** Cognitive deficits as evaluated by the MMSE (< 24 points) Unable to communicate (< 4 points on the Utrechts Communicatie Onderzoek, UCO) Lived farther than 30 km from the rehabilitation centre **Participants:** detailed in Table **PROGRESS‐plus:** summarised in Table
Interventions	(1) Circuit training (n = 126) "Graded task oriented circuit training programme (‘warming up (5 minutes), circuit training (60 minutes), evaluation and a short break (10 minutes), and group game (15 minutes) twice a week over a 12 week period (24 sessions)" "Training included eight different workstations, intended to improve meaningful tasks relating to walking competency" Length of intervention period: 12 weeks Number of sessions and length of individual sessions: "90 minute session twice a week (24 sessions). The circuit training group received 4461 treatment sessions, average treatment time per session was 72 (SD 39) minutes" Intervention provider: physiotherapist and sports therapist (2) Usual physiotherapy (n = 124) "Patients allocated to the control group received usual outpatient physiotherapy, mainly one to one treatments tailored to the patient with a physiotherapist who had not been on the circuit training course at one of the participating rehabilitation centres. Sessions designed to improve control of standing balance, physical condition, and walking competency were provided according to Dutch physiotherapy guidelines" Length of intervention period: not stated Number of sessions and length of individual sessions: "no additional restrictions with respect to content, time, or duration of the physiotherapy. The group received 4378 with an average of 34 (SD 10) minutes per session" Intervention provider: physiotherapist
Outcomes	**Independence in ADL scales:** NR **Motor function scales:** Rivermead Mobility Index (RMI) **Measures of balance:** NR **Measures of gait velocity:** 5‐metre comfortable walking speed test, Timed Up and Go **Length of stay:** NR **Adverse events:** serious adverse events **Other measures:** timed balance test, modified stairs test, Six‐Minute Walk test, functional ambulation categories, Stroke Impact Scale, Falls Efficacy Scale (FES), Hospital Anxiety and Depression Scale (HADS), Fatigue Severity Scale (FSS), Letter cancellation task, the Motricity index (MI‐arm and MI‐leg) **Time points when outcomes were assessed:** "measured all outcomes before randomisation at baseline and after 12 and 24 weeks"
Comparison	**Amount of physical rehabilitation in groups:** differs* **Categorisation of comparison:** 3.(i) Comparison of different approaches (dose equivalent; with no other physical rehab) *Note: the amount of time spent in physical rehabilitation differs between groups, BUT the comparison is still categorised as a comparison of different approaches. Due to the difference in amount, inclusion of this study should be explored using sensitivity analysis.
Funding & conflicts of interest	**Funding statement:** "This study was funded by the Netherlands Organisation for Health Research and Development (ZonMw), No80‐82310‐98‐08303" **Conflict of interest statement:** "All authors have completed the ICMJE uniform disclosure form at www.icmje.org/coi_disclosure.pdf (available on request from the corresponding author) and declare: no support from any organisation for the submitted work; no financial relationships with any organisations that might have an interest in the submitted work in the previous three years; no other relationships or activities that could appear to have influenced the submitted work"
Synthesis	**Quantitative synthesis:**Analysis 7.2; Analysis 7.4; Analysis 7.6; Analysis 8.2; Analysis 8.4 Sensitivity analysis to explore inclusion of this study
Notes	Group sizes for follow‐up results unclear. Sizes stated in article results, Table 2 assumed correct.

**Table:** Langhammer 2000

***Study characteristics***	
Methods	**Study aim:** "examine whether two different physiotherapy regimes causedany differences in outcome in rehabilitation after acute stroke" **Design:** parallel‐group RCT **Country:** Norway **Study recruitment and setting:** see Table **Involvement of patients/public/professionals/others:** no
Participants	**Study population (number randomised):** 61 **Inclusion criteria:** First stroke, verified clinically and by CT scan No subarachnoid bleeding No tumours No severe medical conditions Not more than 4 points on each MAS section **Exclusion criteria:** Not reported **Participants:** detailed in Table **PROGRESS‐plus:** summarised in Table
Interventions	(1) Bobath (n = 28) A "theoretical framework in a reflex‐hierarchical theory" Physiotherapists attended workshops and discussed the treatment approaches and were provided with a manual, based on the supporting texts, which described the key philosophy of the approaches. Techniques were not described Length of intervention period: "as long as they were hospitalized" Number of sessions and length of individual sessions: 5 days/wk for a minimum of 40 minutes, while an inpatient. Following discharge, attempts were made to ensure that physiotherapy continued based on the assigned approach, and physiotherapists involved in the treatment were able to discuss treatments with hospital physiotherapists and project leaders. Intervention provider: physiotherapists (2) Motor learning (n = 33) "Based in system theory, and is basically task‐oriented" Physiotherapists attended workshops and discussed the treatment approaches and were provided with a manual, based on the supporting texts, that described the key philosophy of the approaches. Techniques were not described Length of intervention period: "as long as they were hospitalized" Number of sessions and length of individual sessions: as above Intervention provider: physiotherapists
Outcomes	**Independence in ADL scales:** Barthel Index **Motor function scales:** MAS; Sodring Motor Evaluation **Measures of balance:** NR **Measures of gait velocity:** NR **Length of stay:** length of stay **Adverse events:** NR **Other measures:** Nottingham Health Profile, use of assistive devices, discharge destination **Time points when outcomes were assessed:** "patients were tested three times: (1) three days after admission to the hospital, (2) two weeks thereafter, and (3) three months post stroke". Data for 3 months were used in the immediate time point analyses. Follow‐up outcomes were measured at 1 and 4 years post stroke. Data for 1‐year follow‐up were used in follow‐up time point analyses.
Comparison	**Amount of physical rehabilitation in groups:** equivalent **Categorisation of comparison:** 3.(i) Comparison of different approaches (dose equivalent; with no other physical rehab)
Funding & conflicts of interest	**Funding statement:** "supported by a grant from the Norwegian Society of Physiotherapists (NFF)" **Conflict of interest statement:** not reported
Synthesis	**Quantitative synthesis:**Analysis 7.1; Analysis 7.2; Analysis 8.1; Analysis 8.2
Notes	

**Table:** Langhammer 2007

***Study characteristics***	
Methods	**Study aim:** "to find out if there were any differences in improvement and maintenance of motor function, activity of daily living and grip strength between patients with first‐ever stroke receiving two different strategies of physical exercise during the first year after stroke" **Design:** parallel‐group RCT **Country:** Norway **Study recruitment and setting:** see Table **Involvement of patients/public/professionals/others:** no
Participants	**Study population (number randomised):** 75 **Inclusion criteria:** First‐time‐ever stroke with neurological signs CT confirmed stroke Voluntary participation **Exclusion criteria:** > 1 stroke incident Subarachnoid bleeding Tumour Other serious illness Brainstem or cerebellar stroke **Participants:** detailed in Table **PROGRESS‐plus:** summarised in Table
Interventions	(1) Exercise training (n = 35) "The subsequent training for the intensive exercise group included a functional exercise programme with emphasis on high intensity of endurance, strength and balance. The individualized training programmes were aimed at functional improvements but with variations, for example: getting up from a chair, walking indoors, Nordic walking outdoors, stationary bicycling, and stair walking, where the physiotherapist monitored the levels of intensity through Borg’s Scale or through the pulse rate. A protocol with suggestions of types of exercises and levels of intensity was developed in discussion with all physiotherapists involved. This protocol was intended as a guideline. The goal of these exercises was to improve and maintain motor function, activities of daily living and grip strength. Patients in the intensive exercise group were also encouraged to maintain a high activity level apart from that in the training sessions" Length of intervention period: 12 months Number of sessions and length of individual sessions: "Arrangements were made for patients allocated to the intensive exercise group to have physiotherapy during four periods, with a minimum of 20 hours every third month, in the first year after the stroke. The intervention sessions started immediately after discharge, two or three times a week if the patient was at home or attending a private physiotherapy practice, and daily if he or she was in a rehabilitation ward. This intervention was repeated after three months, six months and one year" Intervention provider: physiotherapists (2) Usual physiotherapy (n = 40) "If the patients in the regular exercise group were considered to be in need of follow‐up treatment or rehabilitation they were assigned to that, but not on a regular basis. No specific treatment was recommended to this group. On the other hand, the same encouragement to maintain a high activity level besides the training, if any, was given to the regular exercise group". A greater proportion of participants in the usual physiotherapy group undertook self‐training. Length of intervention period: 12 months Number of sessions and length of individual sessions: "The regular exercise group patients were given follow‐up treatment according to their needs, as considered by the rehabilitation staff at the stroke unit/rehabilitation department and by the rehabilitation team in the community after discharge" Intervention provider: physiotherapists
Outcomes	**Independence in ADL scales:** Barthel index **Motor function scales:** motor assessment scale **Measures of balance:** Berg Balance Scale **Measures of gait velocity:** 6‐minute walk test (gait velocity calculated) **Length of stay:** NR **Adverse events:** NR **Other measures:** grip strength, Nottingham Health Profile, The Duke Older Americans Resources and Service Procedures (OARS) Multidimensional FunctionalAssessment of Older Adults by Fillenbaum **Time points when outcomes were assessed:** "patients were tested on admission, at discharge, and three months, six months and one year after stroke by an experienced investigator, blinded to group allocation". Subsequent follow‐up assessments at 2 years and 3 years post stroke. Data from 3‐month assessment were used for the immediate time point, and data from 1 year for the follow‐up time point for analyses.
Comparison	**Amount of physical rehabilitation in groups:** differs* **Categorisation of comparison:** 3.(i) Comparison of different approaches (dose equivalent; with no other physical rehab) *Although this is a comparison of "intensive" with "usual care", data collected on the delivered interventions demonstrated that the usual care group only received slightly fewer supervised sessions than the intensive treatment group: "The intensity of the programme was high in both groups, with a tendency to therapeutically steered training in the intensive exercise group and more self‐initiated training in the regular exercise group (Table 1). Between the three‐month and the 12‐month follow‐up the mean number of occasions of supervised exercise in the intensive exercise group was 2.1 times per week and in the regular exercise group 2.2 times per week. This amounts to approximately 40 weeks of exercise per year in both groups, which represents the 80 hours we initially aimed at in the intensive exercise group."
Funding & conflicts of interest	**Funding statement:** not reported **Conflict of interest statement:** not reported
Synthesis	**Quantitative synthesis:**Analysis 7.1; Analysis 7.2; Analysis 7.3; Analysis 7.4; Analysis 7.5; Analysis 8.1; Analysis 8.2; Analysis 8.3
Notes	During the acute phase of rehabilitation at the hospital, both groups received functional task‐oriented training tailored to their specific needs. The amount of training was equal in the two groups, with two periods per day, the two periods comprising a total of one hour of physiotherapy in combination with other specialised therapies according to the participant's needs. At discharge, participants were randomly assigned to two separate groups—an intensive exercise group and a regular exercise group, as described above.

**Table:** LAST 2018

***Study characteristics***	
Methods	**Study aim:** to evaluate the efficacy and safety of an 18‐month follow‐up programme of individualised regular coaching on physical activity and exercise **Design:** parallel‐group RCT ('pragmatic') **Country:** Norway **Study recruitment and setting:** see Table **Involvement of patients/public/professionals/others:** no
Participants	**Study population (number randomised):** 380 **Inclusion criteria:** Aged ≥ 18 years Confirmed first‐ever or recurrent stroke (infarction or intracerebral haemorrhage) Discharged from hospital or inpatient rehabilitation and were community dwelling Modified Rankin Scale (mRS) score < 5 No serious comorbidities that made it difficult to perform the intervention, and were capable of providing consent **Exclusion criteria:** Serious medical comorbidity with short life expectancy Cognitive deficits as evaluated by the MMSE < 21 (or < 17 for people with aphasia) Contraindication to participation in motor training, or inclusion in another study **Participants:** detailed in Table **PROGRESS‐plus:** summarised in Table
Interventions	(1) Long‐term follow‐up programme/individualised coaching (n = 186) In addition to standard care ‐ "Patients will in addition to usual care be given a long term follow‐up program comprising a physiotherapist who will meet and coach the patients, preferably in the patients' home, on a monthly basis for 18 consecutive months. The intevention consists of coaching on physical activity which is a significant part of health coaching, an exercise schedule in accordance with [the patient's] goal and phyysical activity preferences will be made. The program will comprise 45‐60 minutes of physical excercise once a week and 30 minutes of physical activity every day." (2) Usual care (n = 194) "Patients randomised to usual care will be given standard community based treatment, given as outpatient rehabilitation or as rehabilitation in the patients' home according to the patients' needs. The main approach for physiotherapists is a nonspecialised approach, which means that they are expected to treat a range of different patient groups at older age" "Usually consists of 45 minutes of physiotherapy per week, often limited to the first 3 months, but can last for up to 6 months"
Outcomes	**Independence in ADL scales:** Barthel Index, Stroke Impact Scale **Motor function scales:** Motor Assessment Scale **Measures of balance:** Berg Balance Scale Item 14 **Measures of gait velocity:** gait speed, timed up and go test, 6‐minute walk test **Length of stay:** NR **Adverse events:** adverse events **Other measures:** modified Rankin Scale, compliance **Time points when outcomes were assessed:** end of 18‐month intervention
Comparison	**Amount of physical rehabilitation in groups:** differs **Categorisation of comparison:** additional therapy + usual therapy versus usual therapy (dose differs)
Funding & conflicts of interest	**Funding statement:** funded by Norwegian Research Council, Liaison Committee between Central Norway Regional Health Authority and Norwegian University of Science and Technology (NTNU), Joint Research Committee between St. Olavs Hospital and NTNU, Norwegian Fund for Postgraduate Training in Physiotherapy, and Stroke Unit Research Fund at St. Olavs Hospital **Conflict of interest statement:** none
Synthesis	**Quantitative synthesis:**Analysis 5.1; Analysis 5.2; Analysis 5.4; Analysis 5.5 (Note: Berg Balance Scale Item 14 not suitable for inclusion in meta‐analysis of full Berg Balance Scale scores).
Notes	

**Table:** Lawal 2016

***Study characteristics***	
Methods	**Study aim:** study investigated the effectiveness of differing intensities of CCT in the rehabilitation of stroke survivors using the ICF framework to guide patients‘ response assessments after training **Design:** multi‐arm RCT (4‐arm) **Country:** South Africa **Study recruitment and setting:** see Table **Involvement of patients/public/professionals/others:** no
Participants	**Study population (number randomised):** 91 **Inclusion criteria:** Clinical diagnosis of stroke resulting in unilateral sensorimotor deficits Able to give written informed consent Aged ≥ 18 years Sufficient cognition to participate (scoring ≥ 24 points on MMSE) Willing to participate in an 8‐week intensive CCT programme **Exclusion criteria:** Precluding medical comorbidities to exercise Unable to stand from sitting without assistance and walk a minimum of 50 m independently prior to morbidity History of any major surgical procedure significant enough to interfere with performance (general or orthopaedic) in an exercise therapy intervention **Participants:** detailed in Table **PROGRESS‐plus:** summarised in Table
Interventions	(1a) Circuit class therapy ‐ 120 minutes (1b) Circuit class therapy ‐ 90 minutes (1c) Circuit class therapy ‐ 60 minutes 10 workstations ‐ "made up of task specific activities for both upper and lower extremities, placed alternatively across the circuit". Included: 1. tasks for warm up specific for upper extremity; 2. tasks for warm‐up specific for lower extremity; 3. tasks to achieve reaching, gripping and transferring light objects; 4. tasks to achieve lower extremity flexibility and function, 5. tasks to achieve upper extremity strength/control; 6. tasks to achieve balance/coordination while walking; 7. tasks to achieve improved grip, precision and dexterity with upper extremity; 8. tasks to achieve lower extremity strength/control of gait; 9. tasks to achieve advanced motor task with upper extremity; 10. tasks to achieve improved outdoor activities with the lower extremity. "The intervention was an 8‐week, 3‐times weekly training programme, with a total of 24 sessions. Activities were individualised allowing each participant to perform at a select level/pace based on ability, and progressed steadily". Ratio of patients to therapists ‐ 3:1 (2) Control (standard physiotherapy) "comprised of one‐to‐one therapist/patient sessions engaging in impairment‐centred mobilisation techniques, standing balance (using varying methods) and functional activities for both upper and lower extremities"; 60 minute session. Comparison of relevance to this review = Group (1c) vs Group (2)
Outcomes	**Independence in ADL scales:** modified Barthel Index **Motor function scales:** NR **Measures of balance:** NR **Measures of gait velocity:** 10 m walk test, 6‐minute walk test **Length of stay:** NR **Adverse events:** NR **Other measures:** Modified Tardieu Scale (spasticity), manual muscle testing, modified Rankin Scale, ARAT, Motor Activity Log, Stroke Specific Quality of Life, acceptability **Time points when outcomes were assessed:** baseline, outcome, and 6‐month follow‐up
Comparison	**Amount of physical rehabilitation in groups:** equivalent **Categorisation of comparison:** 3.(i) Comparison of different approaches (dose equivalent; with no other physical rehab) (Note: comparison of interest ‐ (1c) vs (2))
Funding & conflicts of interest	**Funding statement:** Tertiary Education Trust Fund (Tetfund) **Conflict of interest statement:** unclear
Synthesis	**Quantitative synthesis:**Analysis 7.1; Analysis 7.4; Analysis 8.1; Analysis 8.4
Notes	

**Table:** Lee 2015

***Study characteristics***	
Methods	**Study aim:** to determine the independent effect of moderate‐intensity aerobic and resistance exercise intervention on central arterial stiffness and gait velocity in Korean patients with chronic post‐stroke hemiparesis **Design:** parallel‐group RCT **Country:** Korea, Rep. **Study recruitment and setting:** see Table **Involvement of patients/public/professionals/others:** no
Participants	**Study population (number randomised):** 35 **Inclusion criteria:** Time since stroke > 12 months No arrhythmia, orthopaedic disorders, and other neurologic diseases MMSE > 24 Able to walk 10 m independently without assistance **Exclusion criteria:** History of TIA without symptoms of hemiparesis Low fitness function based on 6MWT **Participants:** detailed in Table **PROGRESS‐plus:** summarised in Table
Interventions	(1) Aerobic and resistance exercise "The combined aerobic and resistance exercise group performed a 60‐min combined exercise session three times a week for 16 wks." "Each exercise intervention comprised a 5‐min warm‐up, a 10‐min stretching exercise, a 20‐min resistance exercise, a 20‐min aerobic exercise, and a 5‐min cool down (Table 1). Subjects performed standardized whole‐body stretching and light walking during the warm‐up and cool‐down periods. The main stretching exercise included 10 stretching movements (shoulder flexion/extension, shoulder abduction/adduction, trunk flexion/extension, and hip flexion/extension, and hip abduction/adduction) with a 6‐sec passive stretching, 3‐sec muscular contractions, and a 2‐sec relaxation. Aerobic exercise included a 10‐min fast‐walking on a sloping way and another 10 min walking in upstairs. Exercise intensity was established at 50% to 60% of heartrate reserve using the heart rate monitor system (RX800CX, Polar Finland) for 8 wks and then increased to 60% to 70% of heart rate reserve for the remaining 8 wks. Resistance exercise was carried out using elastic resistance bands. Subjects performed resistance exercise, which included lunges, squats, hip flexion/extension, hip abduction/adduction, knee flexion/extension, shoulder abduction/adduction, shoulder flexion/extension, and abdominal crunch/back extension. Exercise intensity was progressed from 10 to 15 repetitions with two to three sets for each motion for 8 wks, and the resistance levels of the elastic band was gradually increased for the remaining 8 wks. Resistance exercise was performed using a rating of perceived exertion of 11 to 16 (light to hard) using the Borg Scale. The exercise program was supervised by a trained exercise rehabilitation specialist and physical therapist." (2) Control "The control group received unsystematic physical activities or played Korean chess at the identical time. The control group did not receive any exercise intervention. They were asked to continue their normal daily activities. Most of them performed very light physical activities such as slow cycling with a cycle ergometer, walking on the hallway, or playing Korean chess."
Outcomes	**Independence in ADL scales:** NR **Motor function scales:** NR **Measures of balance:** NR **Measures of gait velocity:** 6MWT, 10‐m walk test (10MWT), Timed Up‐and‐Go(TUG) test **Length of stay:** NR **Adverse events:** NR **Other measures:** anthropometry, arterial stiffness, blood pressure, muscular strength, 30‐second chair‐stand test, sit and reach test **Time points when outcomes were assessed:** baseline and at 16 weeks
Comparison	**Amount of physical rehabilitation in groups:** differs **Categorisation of comparison:** 1. Physical rehabilitation versus no or limited physical rehabilitation (dose differs) (limited = < 50% of dose)
Funding & conflicts of interest	**Funding statement:** not reported **Conflict of interest statement:** none
Synthesis	**Quantitative synthesis:**Analysis 1.4
Notes	

**Table:** Lee 2018

***Study characteristics***	
Methods	**Study aim:** assess the efficacy and safety of our 4‐week caregiver‐mediated exercise in improving trunk control capacity, gait, and balance and in decreasing concerns about post‐stroke falls when there is an increase in its efficacy **Design:** parallel‐group RCT **Country:** Korea, Rep. **Study recruitment and setting:** see Table **Involvement of patients/public/professionals/others:** no
Participants	**Study population (number randomised):** 72 **Inclusion criteria:** *For stroke survivors*: Aged 18 years+ First‐ever ischaemic or haemorrhagic stroke in the cerebral hemisphere confirmed by CT or MRI Post‐stroke duration < 2 months Post‐stroke hemiplegia with decreased stability of the trunk or lower limb MMSE‐K scores ≥ 24 Able to keep static sitting balance for more than 2 mins Able to keep standing posture when receiving mild‐to‐moderate assistance *For caregivers:* Able to understand instructions on CME Motivated for CME Medically stable Physically able to perform exercises together with stroke survivors **Exclusion criteria:** *For stroke survivors*: Poor visual acuity and/or severe unilateral neglect Severe aphasia Neurological deficits due to causes other than cerebral infarction (e.g. multiple sclerosis, Parkinson's disease, fractures, or congestive heart failure) Serious underlying medical conditions that might affect mobility training (e.g. unstable blood pressure) or musculoskeletal disorders that might affect motor performance Vestibular abnormalities Amputation or joint replacement surgery within 6 months prior to study Cardiac pacemaker or a defibrillator Fed using a NG tube Using a urine or tracheal tube Uncooperative or unable to comply with instructions on CME Others who may not be unable to participate (as judged by trialists) *For caregivers:* Serious comorbidities Unable to walk 100 m, stand, and/or keep their balance **Participants:** detailed in Table **PROGRESS‐plus:** summarised in Table
Interventions	(1) Care‐giver Mediated Exercise (CME) "patients of the trial group received a 1‐hour conventional post‐stroke rehabilitation in the morning and did a 1‐hour CME for 2 hr/day, 5 times a week for 4 weeks" "CME was done by a physical therapist under the supervision of a physician" "A CME protocol as a way to improve trunk control capacity, gait, and balance." "CME was gradually performed. The frequency of its repetition was determined according to patients’ performance. Finally, compliance was monitored using a self‐reported diary during CME." CME protocol consisted of 3 phases: Phase 1 ‐ exercise in a lying posture; Phase 2 ‐ exercise in a sitting posture; Phase 3 ‐ exercise in a standing posture. (2) Control "Patients of the control group solely received a 1‐hour conventional post‐stroke rehabilitation in the morning at 1 hr/day, 5 times a week for 4 weeks." "The conventional post‐stroke rehabilitation was a multi‐disciplinary, patient‐specific intervention that was composed of physiotherapy, occupational therapy, and nursing care. It also included neuropsychological and speech therapy if needed. Various neurological treatment elements were combined, for which motor re‐learning strategies as well as neuro‐developmental recovery were considered as priority."
Outcomes	**Independence in ADL scales:** modified Barthel Index **Motor function scales:** NR **Measures of balance:** Berg Balance Scale **Measures of gait velocity:** NR **Length of stay:** NR **Adverse events:** NR **Other measures:** functional ambulation categories, Trunk Impairment Scale, Fall Efficacy Scale‐International **Time points when outcomes were assessed:** baseline and at 4 weeks
Comparison	**Amount of physical rehabilitation in groups:** differs **Categorisation of comparison:** 5. Additional therapy + usual therapy versus usual therapy (dose differs)
Funding & conflicts of interest	**Funding statement:** not reported **Conflict of interest statement:** none
Synthesis	**Quantitative synthesis:**Analysis 5.1; Analysis 5.3
Notes	

**Table:** Lennon 2006

***Study characteristics***	
Methods	**Study aim:** "to find out whether introducing more gait specific practice for 50% of a Bobath physiotherapy session would improve the patients ability to walk more effectively than usual Bobath therapy" **Design:** parallel‐group RCT **Country:** UK **Study recruitment and setting:** see Table **Involvement of patients/public/professionals/others:** no
Participants	**Study population (number randomised):** 61 **Inclusion criteria:** All consecutive patients within 8 to 14 days post‐stroke Medically stable with a first‐ever stroke Able to stand or walk with assistance **Exclusion criteria:** Not reported **Participants:** detailed in Table **PROGRESS‐plus:** summarised inTable
Interventions	(1) Bobath (n = 30) "Conventional Bobath therapy" Length of intervention period: 4 weeks Number of sessions and length of individual sessions: 5 sessions/wk (total of 20 sessions) lasting 40 minutes Intervention provider: not stated (2) Bobath with walking training (n = 31) "introducing more walking practice into Bobath therapy (spending 50% of the daily physiotherapy session working on walking practice)" Length of intervention period: 4 weeks Number of sessions and length of individual sessions: 5 sessions/wk (total of 20 sessions) lasting 40 minutes Intervention provider: not stated
Outcomes	**Independence in ADL scales:** Barthel Index **Motor function scales:** Motor Assessment Scale, modified Rivermead Mobility Index **Measures of balance:** NR **Measures of gait velocity:** 10‐Metre Walk test **Length of stay:** NR **Adverse events:** NR **Other measures:** step test **Time points when outcomes were assessed:** "All measures were performed by a blinded assessor at baseline, post intervention, at 3 and 6 months post stroke"
Comparison	**Amount of physical rehabilitation in groups:** equivalent **Categorisation of comparison:** 4. Comparison of similar approaches (dose equivalent)
Funding & conflicts of interest	**Funding statement:** Multiple publications, however the following were mentioned: "funded by the Northern Ireland Chest, Heart and Stroke Association"; "The Public Health Agency, Research and Development Office, Northern Ireland" **Conflict of interest statement:** not reported
Synthesis	**Qualitative synthesis:** interventions are similar, therefore not considered suitable for pooling in analyses. Data presented not suitable for analysis.
Notes	Abstracts only

**Table:** Letombe 2010

***Study characteristics***	
Methods	**Study aim:** to evaluate the value of an early post‐stroke evaluation of cardiorespiratory fitness in hemiparetic patients using a one‐leg cycling exercise test and to analyse the impact of an adapted physical activity programme 12 weeks after the stroke **Design:** parallel‐group RCT **Country:** France **Study recruitment and setting:** see Table **Involvement of patients/public/professionals/others:** no
Participants	**Study population (number randomised):** 18 **Inclusion criteria:** Right or left hemiplegia following ischaemic or haemorrhagic hemispheric stroke Full set of aetiological data (CT and/or MRI scans, Holter ECG, Doppler, cardiac ultrasound) Medically stable Well‐balanced treatment (especially antihypertensives and anticoagulants) **Exclusion criteria:** Existence of disorders associated with hemiplegic motor damage (e.g. cognitive and memory disorders, hemisensory neglect Existence of an intercurrent affection or unstable brain lesions **Participants:** detailed in Table **PROGRESS‐plus:** summarised in Table
Interventions	(1) Exercise training (Adapted physical activities, APA) "APA programmes were personalized to suit each patient’s motor capacity and initial exercise results. General exercise training was implemented, with cardiorespiratory exercise (monitored by a heart rate monitor), muscle strengthening, gait exercises and work focused on executive functions. The APA lasted for between 40 and 60 minutes per day, four times a week. In order to develop the patients’ aerobic capacities, steady exercise on a semi‐recumbent cycle ergometer (with both feet pedalling) was performed at between 70 and 80% ofthe maximum power (in W) achieved in the test. We ensured that the patient had already performed a warm‐up session. Stretching‐based physiotherapy sessions were often scheduled after the exercise training session to aid the patient’s recovery. The aerobic programme was modified as a function to the tests performed throughout the rehabilitation care, according to the recommendations of Tailliar et al.[28]. To promote independent gait, we used a treadmill (with or without use of a technical aid, such as an ankle‐foot orthosis) and a stepper. Symmetric balancing stances and leg motor control were developed by using an isokinetic exercise machine (the CybexKinetron). For arm exercises, we used visual biofeedback. The shoulder girdle muscles, trunk muscles and main gait muscles were primarily exercised by using multiple, high‐rep series (i.e. six series of 10 repetitions) of moderate intensity (i.e. between 50 and 60% of the force measured in three maximal, concentric‐mode repetitions)...." (2) Control "All the patients received full hospital care from Monday to Friday, with 5 hours per day of rehabilitation. The standardized, multidisciplinary rehabilitation protocol combined physiotherapy, occupational therapy, speech therapy and neuropsychological therapy for 3 hours a day. The rehabilitation programme was mainly based on improving personal autonomy in ADL, with work focused on use of the legs: gait exercises, stance exercises, the treatment of orthopaedic disorders, balance work (with a view to subsequently withdrawing gait aids), use of support stockings and braces and maintenance of the freedom of movement of the proximal‐distal limb joints. Learning to use a wheelchair and performing transfers were also priorities. For the arms, the application of compression strapping, prehension work and coordination exercises were combined with balance work in the sitting and standing positions. All the therapists were involved in rehabilitation of the higher functions(cognition, memory, etc.). The choice of the techniques used (e.g., Perfetti, Bobath, Kabat and other methods) was left to thetherapists."
Outcomes	**Independence in ADL scales:** Barthel Index, Katz ADL scale **Motor function scales:** NR **Measures of balance:** NR **Measures of gait velocity:** NR **Length of stay:** NR **Adverse events:** NR **Other measures:** maximum aerobic power test, respiratory parameters **Time points when outcomes were assessed:** baseline and after 4 weeks
Comparison	**Amount of physical rehabilitation in groups:** differs **Categorisation of comparison:** 5. Additional therapy + usual therapy versus usual therapy (dose differs)
Funding & conflicts of interest	**Funding statement:** not reported **Conflict of interest statement:** none
Synthesis	**Quantitative synthesis:**Analysis 5.1
Notes	

**Table:** Li 1999

***Study characteristics***	
Methods	**Study aim:** "to explore the effects of early rehabilitation in hemiparalysis of acute cerebral apoplexy" **Design:** parallel‐group RCT **Country:** China **Study recruitment and setting:** see Table **Involvement of patients/public/professionals/others:** no
Participants	**Study population (number randomised):** 61 **Inclusion criteria:** Met 1986 2nd National Conference of Cerebral Vascular Disease assessment guidelines Stroke diagnosis confirmed by CT or MRI scan **Exclusion criteria:** Not reported **Participants:** detailed in Table **PROGRESS‐plus:** summarised inTable
Interventions	(1) Rehabilitation (n = 30) "Basic treatment for the two groups: use of medication to prevent cerebral oedema during the acute phase, dosage and time dependent on the medical condition. In addition, the early rehabilitation group used Bobath technique for training. Commencement of training: ischaemia, between 24 hours and 8 days after stroke onset; haemorrhage, 48 hours and 10 days after stroke onset; medically stable. Main method of rehabilitation was as follows: (I) supine: (i) maintain anti‐spasticity positions, with regular positional change. ii) Passive ranging of all joints. iii) Active rolling, moving. iv) Bridging exercise training. v) Truncal movement training. vi) Independently complete transfer from supine to sitting up. (II) Sitting: (i) Crawling or kneeling position training. (ii) Sitting balance training – 3 stages. (iii) Independently complete transfer from sitting to standing up. (III) Standing: (i) Standing balance training. (ii) Affected lower limb load training. (iii) Practice of knee flexion. (iv) Gait training: In standing, while loading onto the affected lower limb, step forward and backward in increasing amount of angle with the non‐affected lower limb. Without extending the hip, move the knee forward, dorsiflex ankle, and heel strike" Length of intervention period: 1 month Number of sessions and length of individual sessions: "noon and afternoon/daily, 30 min/session" Intervention provider: not stated (2) No physical rehabilitation group (n = 31) "Basic treatment for the two groups: use of medication to prevent cerebral oedema during the acute phase, dosage and time dependent on the medical condition. Control group did not have rehabilitation practice" Length of intervention period: no intervention Number of sessions and length of individual sessions: no intervention Intervention provider: no intervention
Outcomes	**Independence in ADL scales:** Barthel Index **Motor function scales:** Fugl‐Meyer Assessment **Measures of balance:** NR **Measures of gait velocity:** NR **Length of stay:** NR **Adverse events:** NR **Other measures:** neurological deficit score **Time points when outcomes were assessed:** before rehabilitation practice and after 1 month of intervention
Comparison	**Amount of physical rehabilitation in groups:** differs **Categorisation of comparison:** physical rehabilitation versus no or limited physical rehabilitation (dose differs)
Funding & conflicts of interest	**Funding statement:** not reported **Conflict of interest statement:** not reported
Synthesis	**Quantitative synthesis:**Analysis 1.1; Analysis 1.2
Notes	Original study translated from Chinese to English by member of our team (PLC).

**Table:** Li 2005

***Study characteristics***	
Methods	**Study aim:** "to explore the effects of motor re‐learning therapy on movement function and ADL in patients with stroke, and compared with neurodevelopmental therapy" **Design:** parallel‐group RCT **Country:** China **Study recruitment and setting:** see Table **Involvement of patients/public/professionals/others:** no
Participants	**Study population (number randomised):** 61 **Inclusion criteria:** Met the 1995 Fourth National Conference on Cerebral Vascular Disease classification guidelines and 1982 International Meeting on Neurology assessment guidelines First‐ever stroke Aged < 70 years of age exclusive 1 month within stroke onset inclusive Participant’s informed consent **Exclusion criteria:** Previous stroke Does not include TIA or TIA with neurological deficits, conjunctival haemorrhage etc Severe complications Dementia **Participants:** detailed in Table **PROGRESS‐plus:** summarised in Table
Interventions	(1) Motor learning group (n = 31) "Motor relearning group strictly followed the Motor Relearning Program for rehabilitation. Consisting [of] seven components, each component was subdivided into the following 4 subsections for practice: (i) observation, analysis, comparison, and description of normal activity patterns and using the observation and comparison of occupational activity limitation to analyse the missing basic components and abnormal performance. (ii) Practising the missing exercise components, including practice explanation, instruction, language, visual feedback and manual guidance. (iii) ADL practice, including explanation, instruction, practice, language, visual feedback and manual guidance, re‐assessing, encouraging agility. (iv) Change in practice schedule, including scheduled and block practice, practice requiring self‐supervision, creating a positive learning environment, involving the participation of family members and relevant personnel" "Training method: (i) Upper limb function training: stimulate muscle activity and training control of reaching to objects ‐> maintaining muscle length, prevent contractures ‐> stimulate muscle control in the hand and training exercise control ‐> transferring from practice to ADL. (ii) Actinal surface function training: practise swallowing, facial exercise, improve breathing control ‐> transferring from practice to ADL. (iii) Lying to sitting on the edge of bed training ‐> transferring from practice to ADL. (iv) Sitting balance training: training postural adjustment during gravitational shifts ‐> increasing the complexity of training ‐> transferring from practice to ADL. (v) Sit to stand and stand to sit training. (vi) Standing balance training: bilateral lower limb loading training ‐> hip alignment practice ‐> initiation of quadriceps contraction practice ‐> training postural adjustment during gravitational shifts ‐> increasing difficulty ‐> transferring from practice to ADL. (vii) Gait training: standing training‐> stepping practice ‐> ambulatory training ‐> increasing difficulty ‐> transferring from practice to ADL" "Rehabilitation sequentially and progressively used the above seven components for intervention, adapting to the individual participant’s treatment progress" Length of intervention period: not stated Number of sessions and length of individual sessions: "45 min/session, 1/day, till the end of the study" Intervention provider: not stated (2) Neurodevelopmental therapy group (n = 30) "Followed a combination of Bobath, proprioceptive neuromuscular facilitation (PNF) technique, Brunnstrom and Rood for rehabilitation" "Neurodevelopmental therapy intervention: Using Bobath as the main component, intervention included 3 stages to treat: retardation stage, spasm stage and relative recovery stage: maintaining the correct supine position ‐> rolling practice ‐> sitting up from bed practice ‐> sitting balance training ‐> transfers training ‐> sit to stand training ‐> standing balance training ‐> gait training ‐> ADL training. The above followed the sequence and principles of neurodevelopment" Length of intervention period: not stated Number of sessions and length of individual sessions: "45 min/session, 1/day, till the end of the study" Intervention provider: not stated
Outcomes	**Independence in ADL scales:** Barthel Index **Motor function scales:** NR **Measures of balance:** NR **Measures of gait velocity:** NR **Length of stay:** NR **Adverse events:** NR **Other measures:** stroke lesion score evaluation form **Time points when outcomes were assessed:** day 1 of hospital admission and every month thereafter. For participants who were discharged before 1 month, assessment was done on discharge.
Comparison	**Amount of physical rehabilitation in groups:** equivalent **Categorisation of comparison:** 3.(i) Comparison of different approaches (dose equivalent; with no other physical rehab)
Funding & conflicts of interest	**Funding statement:** not reported **Conflict of interest statement:** not reported
Synthesis	**Quantitative synthesis:**Analysis 7.1
Notes	Original study translated from Chinese to English by member of our team (PLC).

**Table:** Li 2013

***Study characteristics***	
Methods	**Study aim:** to analyse the effect of early intensive walking basic skills training on stroke prognosis **Design:** parallel‐group RCT **Country:** China **Study recruitment and setting:** see Table **Involvement of patients/public/professionals/others:** no
Participants	**Study population (number randomised):** 176 **Inclusion criteria:** Met the diagnostic criteria established by the 4th National Cerebrovascular Disease Conference Confirmed by CT or MRI **Exclusion criteria:** Course of disease > 4 weeks Vital signs not stable Cognitive impairment Unable to follow motor commands Liver, kidney and other vital organ complications Poor 'centre of gravity' **Participants:** detailed in Table **PROGRESS‐plus:** summarised in Table
Interventions	(1) Strength training "the group took the early strengthening step Do basic skills training: 1. Hip joint control ability training: Use bridge training such as improved bridge exercise hip swing training, bridge stepping training, etc., and do left and right hip swinging and alternating small‐scale stepping exercises with both feet; 2. Healthy hand patting on the affected knee: Use the spiral diagonal mode to perform hip flexion training in adduction and internal rotation, and guide the affected knee with the unaffected hand patting to suppress the common movement pattern of the flexors; 3. Hip flexion and extension ability training: The patient stands with his back against the wall and performs hip squat 0‐60° training. If the knee cannot be flexed, a nurse or therapist can assist him. Stand up with side single leg support; 4. Front and back leg training of the unaffected leg; 5. Impact leg training; 6. Inward and outer stride training of the unaffected leg [5,6]. (2) Conventional rehabilitation "Both groups of patients received conventional drug treatment and rehabilitation training[4], including single‐leg weight‐bearing, center of gravity conversion, hip extension step training, affected leg up and down steps, affected leg supported hip extension standing, side step and in‐situ step, etc. " "To ensure that the training volume of the two groups was basically the same, each training lasted 30 min, 2 times a day, 5 days a week, and 4 weeks of continuous training. "
Outcomes	**Independence in ADL scales:** modified Barthel Index **Motor function scales:** Fugl Meyer assessment (lower extremity) **Measures of balance:** Berg Balance Scale **Measures of gait velocity:** "average pace" **Length of stay:** NR **Adverse events:** NR **Other measures:** step length and width **Time points when outcomes were assessed:** before treatment, after treatment, and 4 weeks after treatment
Comparison	**Amount of physical rehabilitation in groups:** equivalent **Categorisation of comparison:** 3. (ii) Comparison of different approaches (dose equivalent; with both/all groups also receiving other physical rehab)
Funding & conflicts of interest	**Funding statement:** Funded project: 2012 Hainan Provincial Medical Research Project (Qiong Wei 2012PT‐95) **Conflict of interest statement:** not reported
Synthesis	**Quantitative synthesis:**Analysis 7.1; Analysis 7.2; Analysis 7.3; Analysis 7.4; Analysis 8.1; Analysis 8.2; Analysis 8.3; Analysis 8.4
Notes	This study was published in Chinese and translated into English using Google Translate.

**Table:** Li Jingqian 2013

***Study characteristics***	
Methods	**Study aim:** to examine the effectiveness of exercise re‐learning rehabilitation training for stroke patients **Design:** parallel‐group RCT **Country:** China **Study recruitment and setting:** see Table **Involvement of patients/public/professionals/others:** no
Participants	**Study population (number randomised):** 120 **Inclusion criteria:** Met the diagnostic criteria adopted by the Fourth National Cerebrovascular Disease Academic Conference Stroke confirmed by head CT or MRI **Exclusion criteria:** Not reported **Participants:** detailed in Table **PROGRESS‐plus:** summarised in Table
Interventions	(1) Motor re‐learning "The treatment group underwent rehabilitation function training according to the motor relearning program. Each patient developed an individualized training plan after evaluation, and the training content mainly consisted of 7 parts: upper limb function, orofacial function, bedside sitting, sitting balance, standing and sitting, standing balance, and walking [2]. Training once a day for 45 minutes each time." Includes: "1. Motor re‐learning has a complete evaluation system, which should be followed every day during training: evaluation‐treatment‐re‐evaluation‐re‐treatment. 2. Active movement should be induced in the position that is most likely to induce active movement, so as to enhance the patient's confidence in recovery. 3. Emphasize that in the early stage, as long as the vital signs are stable, start rehabilitation training, including getting out of bed and standing. 4. Emphasize active movement. Only active movement is the patient's own movement. 5. Emphasize feedback to promote neuromuscular recovery. 6. Guided by functional rehabilitation, it is easier for patients to return to society and integrate into daily life. 7. Let patients and their families learn the basics of exercise re‐learning so that they can enter community rehabilitation and family rehabilitation. " (2) Conventional rehabilitation "Both groups received conventional treatments such as acupuncture, physiotherapy, and exercise therapy. " (no further details provided)
Outcomes	**Independence in ADL scales:** modified Barthel Index **Motor function scales:** "Motor function assessment scale (MAS)" **Measures of balance:** NR **Measures of gait velocity:** NR **Length of stay:** NR **Adverse events:** NR **Other measures:** NR **Time points when outcomes were assessed:** "Before and after rehabilitation treatment"/"an average of 30 days of treatment"
Comparison	**Amount of physical rehabilitation in groups:** differs **Categorisation of comparison:** 5. Additional therapy + usual therapy versus usual therapy (dose differs)
Funding & conflicts of interest	**Funding statement:** not reported **Conflict of interest statement:** not reported
Synthesis	**Quantitative synthesis:**Analysis 5.1; Analysis 5.2
Notes	This study was published in Chinese and translated into English using Google Translate.

**Table:** Lincoln 2003

***Study characteristics***	
Methods	**Study aim:** evaluated the effect of Bobath based and movement science‐based physiotherapy interventions on movement abilities and functional independence **Design:** parallel‐group RCT **Country:** UK **Study recruitment and setting:** see Table **Involvement of patients/public/professionals/others:** no
Participants	**Study population (number randomised):** 120 **Inclusion criteria:** Stroke < 2 weeks previously **Exclusion criteria:** Unconscious on admission Unable to toilet self before stroke Unable to tolerate more than 30 minutes of physical tasks Living farther than 25 km from hospital No informed consent given **Participants:** detailed in Table **PROGRESS‐plus:** summarised in Table
Interventions	(1) Bobath (n = 60) "Treatment delivered by different groups of physiotherapists using prepared written guidelines, consisting of theoretical concepts for practice and main clinical objectives, based on their own knowledge and experience and their interpretation of the literature" Techniques were not described. Bobath‐based treatment was delivered by the unit's existing physiotherapists, who had used this approach routinely before the start of the study. Prepared written guidelines were available. Length of intervention period: "treatment continued for as long as was needed"....approach continued as outpatient if necessary... Amount matched to "typical amount" given by existing ward physiotherapists Number of sessions and length of individual sessions: median of 23 minutes per weekday Intervention provider: more time was spent with physiotherapist and physiotherapy assistant together for this treatment group. Stated that occupational therapists also used this approach (2) Motor learning (n = 60) "Treatment delivered by different groups of physiotherapists using prepared written guidelines, consisting of theoretical concepts for practice and main clinical objectives, based on their own knowledge and experience and their interpretation of the literature" Techniques were not described. Motor‐learning treatment was delivered by two physiotherapists, who previously had 'insufficient experience of treatment' but who were given training. Prepared written guidelines were available. Length of intervention period: "treatment continued for as long as was needed"....approach continued as outpatient if necessary. Amount matched to "typical amount" given by existing ward physiotherapists Number of sessions and length of individual sessions: median of 23 minutes per weekday Intervention provider: more time was spent with physiotherapy assistant alone in this treatment group. Stated that occupational therapists also used this approach.
Outcomes	**Independence in ADL scales:** Barthel Index; EADL **Motor function scales:** Motor Assessment Scale; Rivermead Motor Assessment **Measures of balance:** NR **Measures of gait velocity:** 10‐Metre Walk test **Length of stay:** length of stay **Adverse events:** NR **Other measures:** Modified Ashworth Scale, Nine‐Hole Peg test; Nottingham Sensory Assessment **Time points when outcomes were assessed:** "Measures were performed by a blinded assessor at baseline, and then at 1, 3, and 6 months after baseline"
Comparison	**Amount of physical rehabilitation in groups:** equivalent **Categorisation of comparison:** 3.(i) Comparison of different approaches (dose equivalent; with no other physical rehab)
Funding & conflicts of interest	**Funding statement:** "funded by the Stroke Association, UK" **Conflict of interest statement:** "none declared"
Synthesis	**Quantitative synthesis:**Analysis 7.1; Analysis 7.2; Analysis 7.4
Notes	

**Table:** Lindvall 2014

***Study characteristics***	
Methods	**Study aim:** to investigate the effects of body awareness therapy on balance, mobility, balance confidence, and subjective health status in persons with stroke **Design:** parallel‐group RCT **Country:** Sweden **Study recruitment and setting:** see Table **Involvement of patients/public/professionals/others:** unclear
Participants	**Study population (number randomised):** 46 **Inclusion criteria:** > 6 months since onset of the most recent stroke Able to walk a distance of 100 m with or without assistance Subjectively experienced balance impairment (i.e. a yes on the question ‘do you feel that your balance is impaired?’) **Exclusion criteria:** Medical, physical or cognitive impairment that affected the ability to actively participate or to understand written and verbal instructions **Participants:** detailed in Table **PROGRESS‐plus:** summarised in Table
Interventions	(1) Body awareness therapy (n = 24) "The experimental intervention was an add‐on therapy to the participants’ usual daily activities" "The experimental intervention consisted of body awareness therapy in groups for eight weeks, 60 minutes once a week. Four sessions was set as the minimum for participation in the intervention. The intervention was led by physiotherapists with further training and 15–20 years of clinical experience in body awareness therapy. The choice of movements in the intervention was based on earlier body awareness therapy studies, and experiences from the physiotherapists responsible for the intervention. Before the intervention started these physiotherapists and the authors decided upon a framework for the intervention. The framework consisted of a list of which movements to use. Movements were performed in sitting, standing, and supine." (2) Control (n = 22) "For participants randomized to the control group, no further intervention was introduced." "All participants were instructed to continue with their usual daily activities, ongoing physiotherapy or other training, regarding both amount and content."
Outcomes	**Independence in ADL scales:** NR **Motor function scales:** NR **Measures of balance:** Berg Balance Scale **Measures of gait velocity:** Timed Up and Go Test, 6‐minute walk test **Length of stay:** NR **Adverse events:** NR **Other measures:** Timed Up and Go Cognitive Test, Swedish version of the Activities‐specific Balance Confidence Scale, Short Form 36 (SF‐36) questionnaire **Time points when outcomes were assessed:** 1 week and 6 weeks after intervention
Comparison	**Amount of physical rehabilitation in groups:** differs **Categorisation of comparison:** additional therapy + usual therapy versus usual therapy (dose differs)
Funding & conflicts of interest	**Funding statement:** supported by the Swedish Stroke Association (2011), Norrbacka Eugenia Foundation (803‐11) and Research Committee Örebro County Council (OLL‐227701) **Conflict of interest statement:** none
Synthesis	**Quantitative synthesis:**Analysis 5.3; Analysis 5.4; Analysis 6.3; Analysis 6.4 Gait results only. Balance results not suitable for quantitative analysis (not normally distributed).
Notes	

**Table:** Liu 2014

***Study characteristics***	
Methods	**Study aim:** "to investigate the effect of task orientation combined with muscle strength training on lower extremity motor function and ADL in patients with stroke sequelae" **Design:** parallel‐group RCT **Country:** China **Study recruitment and setting:** see Table **Involvement of patients/public/professionals/others:** no
Participants	**Study population (number randomised):** 80 **Inclusion criteria:** Not reported **Exclusion criteria:** Not reported **Participants:** detailed in Table **PROGRESS‐plus:** summarised in Table
Interventions	**(1) Task oriented training** "The experimental group implemented rehabilitation training with task orientation combined with muscle strength training: (1) The patient is in a supine position, and double‐bridge or single‐bridge training is performed to strengthen the strength of the lumbar back muscles and the control ability of the affected side. During the training process of the bridge exercise, the therapist raises the hand to a distance of 5‐15 cm from the patient's abdomen. Height, instruct the patient to raise the buttocks, the abdomen touches the palm, and maintain 3 ~ 5s, 20 times / time. (2) The patient was placed in the supine position, and the hip joint flexion training was performed, and the elbow joint of the healthy side was used to touch the knee joint of the affected side. Patients who are unable to complete this item should be placed in a lateral recumbent position and complete hip flexion with the help of a therapist, 40 times/time. (3) Sit‐up training: The patient underwent postural transformation training, from supine position to healthy side lying position, and then sat up, 10 times/time. (4) Sitting balance training: after the patient sits up from the unaffected side, sit on the side of the bed, with both feet on the ground, the distance is the same as the shoulder width, the knee joint is flexed at about 90 degrees, and a wooden board is placed beside the bed, at the same height as the bed, in front of the patient's torso. Bend and reverse the wooden board with the healthy hand, 10 times/time. After completion, the trunk will return to the original position, and do 40 times in total. Adjust the height of the wooden board according to the specific situation of the patient. After the sitting balance reaches level 2, start the lateral transfer train. (5) Stand‐up training: the patient stands on the bedside with both feet on the ground, the distance is the same as the shoulder width, and the knee joint is flexed at about 90 degrees. The therapist fixes the knee joint on the affected side. Bend, push the lower limbs on the healthy side, leave the buttocks off the bed, look up, stand up straight, and maintain for 15‐45s, 20 times/time. (6) Standing balance training: The patient stands with the feet shoulder‐width apart. The therapist fixes the knee joint on the affected side and places a wooden board in front of the patient. The height is adjusted according to the patient's height. (7) Center of gravity transfer and weight‐bearing training on the affected side: the patient stands on the ground with both feet on the ground, the distance is shoulder‐width, the therapist helps adjust the center of gravity on the affected side, and the unaffected lower limbs do step training in all directions, and the trunk is continuously adjusted and balanced, 50 steps/time (Some patients can do step‐step training for the lower limbs on the affected side in the later stage). The implementation should be considered from the perspective of safety, effectiveness and rationality, and it should be implemented according to the specific situation of the patient during the training period." (2) Conventional rehabilitation "The control group was treated with conventional rehabilitation methods, namely: trunk control training, standing‐sit training, sitting balance training, standing balance training, center of gravity transfer training, and walking training under parallel bars. The therapist assisted the patient to perform a complete training session every day, and 30 training sessions were used as a rehabilitation training cycle."
Outcomes	**Independence in ADL scales:** Barthel Index **Motor function scales:** Fugl‐Meyer scale **Measures of balance:** NR **Measures of gait velocity:** gait speed **Length of stay:** NR **Adverse events:** NR **Other measures:** NR **Time points when outcomes were assessed:** before and after treatment (unclear how long the treatment period was)
Comparison	**Amount of physical rehabilitation in groups:** differs (amount not clear) **Categorisation of comparison:** none of these comparisons (comparison of different approaches, but dose differs)
Funding & conflicts of interest	**Funding statement:** not reported **Conflict of interest statement:** not reported
Synthesis	**Qualitative synthesis:** study does not fit into any of the pre‐stated comparisons, as both groups receive intervention, but with different doses.
Notes	This study was published in Chinese and translated using Google Translate.

**Table:** Liu Xuan 2016

***Study characteristics***	
Methods	**Study aim:** To observe the actual clinical effect of comprehensive rehabilitation in treating ischaemic stroke patients with hemiplegia. **Design:** parallel‐group RCT **Country:** China **Study recruitment and setting:** see Table **Involvement of patients/public/professionals/others:** no
Participants	**Study population (number randomised):** 60 **Inclusion criteria:** Early onset < 14 days since stroke Stable vital signs Conscious GCS > 8 points Hemiplegia Aged < 80 years **Exclusion criteria:** Severe hepatic and renal disease Complete quadriplegia Stroke onset > 14 days Malignant tumours Disturbed consciousness History of mental illness History of respiratory failure Did not provide informed consent **Participants:** detailed in Table **PROGRESS‐plus:** summarised in Table
Interventions	(1) Three‐stage rehabilitation Usual treatment as well as comprehensive "three stages of rehabilitation". Three level rehabilitation divided into 3 time‐periods: Stage I: rehabilitation on the ward (up to 1 month) Comprises the following: different positioning and turning on paretic and non‐paretic side, sitting training by progressively raising the angle of the bed and when the stroke survivor is at 90 degrees, allowing them to get out of bed to perform sit up exercises on non‐paretic limb. Training is tailored for each stroke survivor and these exercises usually take place over 5 days. Stage II: rehabilitation in a rehabilitation centre (up to 2 to 3 months) Comprises the following: standing up training, standing balance training, gait training; standing on one leg training; walking training; up and down stairs training. Stage III: rehabilitation at home (months 3 to 6) Continuing the exercises from stage II at home, carrying out ADLs and other activities (e.g. sports and entertainment) (2) Conventional rehabilitation Usual treatment as well as rehab treatment. Usual rehabilitation treatment "as would be expected in neurology". Appears to be dose matched.
Outcomes	**Independence in ADL scales:** Barthel Index **Motor function scales:** NR **Measures of balance:** NR **Measures of gait velocity:** NR **Length of stay:** NR **Adverse events:** NR **Other measures:** Brunnstrom **Time points when outcomes were assessed:** after 1, 3, and 6 months of treatment
Comparison	**Amount of physical rehabilitation in groups:** equivalent **Categorisation of comparison:** 3.(i) Comparison of different approaches (dose equivalent; with no other physical rehab)
Funding & conflicts of interest	**Funding statement:** not reported **Conflict of interest statement:** not reported
Synthesis	**Qualitative synthesis:** study does not fit into any of the comparisons explored within meta‐analyses.
Notes	This study was published in Chinese and relevant sections translated into English by our author team (PLC).

**Table:** Liu Yanhua 2020

***Study characteristics***	
Methods	**Study aim:** to explore the application value of compulsory exercise therapy in the rehabilitation treatment of stroke hemiplegia **Design:** parallel‐group RCT **Country:** China **Study recruitment and setting:** see Table **Involvement of patients/public/professionals/others:** no
Participants	**Study population (number randomised):** 84 **Inclusion criteria:** Diagnosed with stroke‐related hemiplegia Able to actively complete the daily functional exercises required by medical staff Written informed consent **Exclusion criteria:** Presence of mental illness Associated with severe organ insufficiency **Participants:** detailed in Table **PROGRESS‐plus:** summarised in Table
Interventions	(1) Compulsory exercise therapy "The patients in the observation group were given compulsory exercise therapy, and the main training was as follows: 1. Quadriceps resistance training and rehabilitation treadmill training. In the quadriceps resistance training, the patient is assisted by the medical staff. In order to prevent the patient's muscle strain, the medical staff instructs the patient to slow down the exercise as much as possible. In the treadmill training, in order to prevent the patient from falling, etc. The medical staff gave physical support to the patient, and the training time was about 30 minutes, once a day. 2. Sit up training. The medical staff instructed the patient to stand up and sit down continuously on the chair, 50 cm was the initial training height, and then gradually decreased to 20 cm according to the patient's training situation. The number of times is determined by the degree of exposure, once a day. 3. Exercise plank training. During the training, the intensity was controlled at 8.0‐16.5 m/min, and the medical staff recorded the training time for the patients during the training process, 20 min each time, twice a day. 4. Balance training and training up and down stairs. Balance training can be performed in the balance bar initially, mainly for forward and backward movement, left and right movement, and side walking. After the above training meets the requirements, dynamic training can be performed on the balance board; for up and down stairs training, patients need the assistance of medical staff. Go up and down the stairs, and determine the number of floors up and down according to the actual situation of the patient, once a day." (2) Conventional "The patients in the control group were treated with conventional exercise therapy, and the patients were mainly trained to carry out lower limb physical therapy (PT) training and neurodevelopmental techniques, including joint training, trunk training and balance ability training, 2 hours each time, once a day. According to the actual situation of the patient, the medical staff will guide and carry out the training step by step, so that the patient can gradually move from the initial passive movement to the active movement. The training intensity needs to be adjusted according to the patient's tolerance." "The training period for both groups was 6 weeks."
Outcomes	**Independence in ADL scales:** NR **Motor function scales:** NR **Measures of balance:** Berg Balance Scale **Measures of gait velocity:** 10‐metre walk, timed up and go **Length of stay:** NR **Adverse events:** NR **Other measures:** patient satisfaction **Time points when outcomes were assessed:** baseline and 6 weeks (after intervention)
Comparison	**Amount of physical rehabilitation in groups:** equivalent **Categorisation of comparison:** 3.(i) Comparison of different approaches (dose equivalent; with no other physical rehab)
Funding & conflicts of interest	**Funding statement:** NR **Conflict of interest statement:** NR
Synthesis	**Quantitative synthesis:**Analysis 7.3; Analysis 7.4
Notes	Paper published in Chinese and translated using Google Translate

**Table:** Li Weiwei 2015

***Study characteristics***	
Methods	**Study aim:** to investigate the effect of task‐oriented training on ADL of stroke patients in rehabilitation department **Design:** parallel‐group RCT **Country:** China **Study recruitment and setting:** see Table **Involvement of patients/public/professionals/others:** no
Participants	**Study population (number randomised):** 60 **Inclusion criteria:** Hemiplegia following a cerebral infarction, cerebral haemorrhage, and postoperative cerebral trauma Met the diagnostic criteria established by the 4th National Cerebrovascular Disease Conference **Exclusion criteria:** Transient cerebral ischaemia Reversible stroke Subarachnoid haemorrhage Cognitive impairment Unable to understand the instructor **Participants:** detailed in Table **PROGRESS‐plus:** summarised in Table
Interventions	(1) Task‐oriented training "In addition to routine rehabilitation therapy and general rehabilitation care, the experimental group guided patients to take the initiative to participate in ADL training for the missing components of ADL in a task‐oriented manner, using explanations, instructions, exercises combined with language, visual feedback and manual guidance. It is appropriate to succeed with a little effort; set homework exercises that conform to daily life habits; the determination of goals, the setting of tasks, and the combination of training and real life" (2) Conventional rehabilitation "The control group received routine rehabilitation treatment and nursing according to doctor's orders, and was given general rehabilitation nursing guidance" "Both groups were trained for 30 min each time, 2 times a day, 5 days a week. "
Outcomes	**Independence in ADL scales:** Barthel Index **Motor function scales:** NR **Measures of balance:** NR **Measures of gait velocity:** NR **Length of stay:** NR **Adverse events:** NR **Other measures:** NR **Time points when outcomes were assessed:** before and after training
Comparison	**Amount of physical rehabilitation in groups:** equivalent **Categorisation of comparison:** 3. (ii) Comparison of different approaches (dose equivalent; with both/all groups also receiving other physical rehab)
Funding & conflicts of interest	**Funding statement:** Wujiang City Social Development Science and Technology Project (No.: WS201213) **Conflict of interest statement:** not reported
Synthesis	**Quantitative synthesis:**Analysis 7.1
Notes	This study was published in Chinese and translated into English using Google Translate.

**Table:** Li Xiaojun 2016

***Study characteristics***	
Methods	**Study aim:** to explore the application effect of task‐oriented training in stroke patients with hemiplegia **Design:** parallel‐group RCT **Country:** China **Study recruitment and setting:** see Table **Involvement of patients/public/professionals/others:** no
Participants	**Study population (number randomised):** 74 **Inclusion criteria:** Met the diagnostic criteria of the 4th National Cerebrovascular Academic Conference Stroke confirmed by MRI or CT Informed consent In the recovery period Unilateral hemiplegia with onset 6‐24 months No mental, intellectual or consciousness issues Aged 18 years+ Ashworth spasticity graded as grade 1 or 2 **Exclusion criteria:** Cerebral haemorrhage, haemorrhagic tendency, and gradual aggravation of the disease Severe cognitive impairment Ashworth spasticity graded as grade 3 or 4 Complicated with intractable hypertension, depression, learning disabilities, and visual impairment **Participants:** detailed in Table **PROGRESS‐plus:** summarised in Table
Interventions	(1) Task‐oriented training "Intervention group ‐ in addition to conventional ‐ task oriented training Upper limb task‐oriented training: lying down on their back they are instructed to touch their forehead, mouth, and shoulder of the non‐paretic side with the hand on the paretic side. The therapist raises the hand, instructs the patient to straighten the upper limb as much as possible, and then touch the therapist's hand. In a seated position the stroke survivor is asked to put the upper limb of the paretic side on the table, they are then instructed to use the thumb, forearm, back of the hand and other parts to push a building block to the pre‐set position, then grasp the building block with the hand and place it to another pre‐set position, During the exercise, the distance and weight of the block is gradually increased. Exercises are tailored to the individual and their ability. Lower limb task‐oriented training comprised of bridging exercises (the dynamic bridge exercise, single bridge exercise and double bridge exercise). The paretic lower limb performed prone knee flexion, lateral knee flexion, pelvic exercise, straight leg raising, lower limb adduction and lower limb abduction and other exercises. A target was set based on the stroke survivors’ condition and they aimed to meet the target as much as possible. During the training process the distance was increased and stroke survivors asked to prolong the touch time; when performing training such as straight leg raising, prone knee bending. Sandbags could be used to provide resistance (e.g., by tying it to the calf). Body balance task‐oriented training: comprised (a) sitting balance exercise e.g. trunk lateral flexion, trunk forward leaning, sitting position touching objects, catching a ball; (b) standing position balance exercise: the patient takes a standing position and performs squats training, low stool sit‐ups, sit‐down training, stand‐up training, alternating pedals with both feet, standing with the healthy foot behind the affected foot, throwing and catching the ball, reaching for objects in different directions. The complexity of the exercise was gradually increased based on the stroke survivor’s condition." 1 to 2 sessions/day, session length 30 to 45 minutes, 5 days/week delivered over 8 weeks (2) Conventional rehabilitation Both received conventional rehabilitation intervention. Little description reported for this intervention.
Outcomes	**Independence in ADL scales:** Barthel Index **Motor function scales:** Fugl Meyer assessment **Measures of balance:** NR **Measures of gait velocity:** NR **Length of stay:** NR **Adverse events:** NR **Other measures:** Elderly Mobility Scale **Time points when outcomes were assessed:** NR
Comparison	**Quantitative synthesis:**Analysis 5.1; Analysis 5.2
Funding & conflicts of interest	**Funding statement:** NR **Conflict of interest statement:** NR
Synthesis	**Amount of physical rehabilitation in groups:** differs **Categorisation of comparison:** 5. Additional therapy + usual therapy versus usual therapy (dose differs)
Notes	This study was published in Chinese and relevant sections translated into English by our author team (PLC). We identified two papers, both with different author groups, and different clinical sites, which appear to report the same study. These studies have the same methods, recruitment dates, and participant numbers. The results data for the treatment group are the same in both papers, but there were some differences in Fugl‐Meyer scores reported between papers for the control group. Barthel Index was the same in both papers. We have reported this as one study. We have used results data from the paper marked as the primary paper.

**Table:** Li Yuanzheng 2014

***Study characteristics***	
Methods	**Study aim:** "to describe the effectiveness of Bobath therapy in rehabilitation after stroke" **Design:** parallel‐group RCT **Country:** China **Study recruitment and setting:** see Table **Involvement of patients/public/professionals/others:** no
Participants	**Study population (number randomised):**122 **Inclusion criteria:** Not reported **Exclusion criteria:** Not reported **Participants:** detailed in Table **PROGRESS‐plus:** summarised in Table
Interventions	(1) Bobath "the observation group was additionally treated with Bobath technique. The main methods were: good limb placement during the flaccid phase, passive joint activity, recovery of muscle strength and muscle tension, rollover training, sit‐up training, combination of active‐passive activities, etc. Mainly training; in the spastic period, anti‐spasm, inducing dissociative exercise, and improving motor function are the main elements; in the recovery period, inducing dissociative exercise, overcoming abnormal patterns, improving walking ability, and daily life movement training, etc., qd, 45 min/time, 2 months of treatment" (2) Control "Both groups were treated according to the conventional treatment plan for stroke in the Department of Neurology"
Outcomes	**Independence in ADL scales:** Barthel Index **Motor function scales:** Fugl‐Meyer motor function assessment **Measures of balance:** NR **Measures of gait velocity:** NR **Length of stay:** NR **Adverse events:** NR **Other measures:** NR **Time points when outcomes were assessed:** before and after treatment
Comparison	**Amount of physical rehabilitation in groups:** differs **Categorisation of comparison:** 1. Physical rehabilitation versus no or limited physical rehabilitation (dose differs) (limited = < 50% of dose)
Funding & conflicts of interest	**Funding statement:** not reported **Conflict of interest statement:** not reported
Synthesis	**Quantitative synthesis:**Analysis 1.1; Analysis 1.2
Notes	Paper published in Chinese, and translated using Google Translate.

**Table:** Li Yuanzheng 2014a

***Study characteristics***	
Methods	**Study aim:** to observe the effect of exercise relearning on balance disorder training in stroke patients **Design:** parallel‐group RCT **Country:** China **Study recruitment and setting:** see Table **Involvement of patients/public/professionals/others:** no
Participants	**Study population (number randomised):** 58 **Inclusion criteria:** Stable disease with stable vital signs Clear consciousness Disease duration < 3 months No serious complications **Exclusion criteria:** Not reported **Participants:** detailed in Table **PROGRESS‐plus:** summarised in Table
Interventions	(1) Motor re‐learning "exercise re‐learning rehabilitation training. The training was carried out under the specific demonstration of the therapist. The patients repeated the simulation until they could complete the movements correctly. Head muscle training, walking hip extension exercises, pelvic level training, etc. 30 min/time, 5 d/week, continuous treatment for 4 weeks. " (2) Conventional rehabilitation "the control group was given routine rehabilitation training: good limb placement; Bobath technique; Brunstorm technique; PNF technique; Rood technique; bridge exercise; separation exercise training; balance training; standing training, Gait training. Each rehabilitation training lasted about 30 min, 2 times/d, 5 d/week, and the treatment was continued for 4 weeks. " "Both groups were given routine rehabilitation training, and the observation group received additional exercise relearning therapy."
Outcomes	**Independence in ADL scales:** NR **Motor function scales:** NR **Measures of balance:** Berg Balance score, Fugl‐Meyer Balance assessment **Measures of gait velocity:** NR **Length of stay:** NR **Adverse events:** NR **Other measures:** NR **Time points when outcomes were assessed:** before and after treatment
Comparison	**Amount of physical rehabilitation in groups:** differs **Categorisation of comparison:** 5. Additional therapy + usual therapy versus usual therapy (dose differs)
Funding & conflicts of interest	**Funding statement:** not reported **Conflict of interest statement:** not reported
Synthesis	**Qualitative synthesis:** Data not included in meta‐analysis due to serious concerns about the data: reported data are ‘opposite’ from what is reported narratively (i.e. the text states that observation group is significantly better than control, but data in table show control to be significantly better). Further, text states that the maximum Berg score was 56, but mean reported values are > 56.
Notes	This study was published in Chinese and translated into English using Google Translate.

**Table:** Lu 2004

***Study characteristics***	
Methods	**Study aim:** to investigate the effect of a three‐stage rehabilitation scheme on acute stroke patients **Design:** parallel‐group RCT **Country:** China **Study recruitment and setting:** see Table **Involvement of patients/public/professionals/others:** no
Participants	**Study population (number randomised):** 42 **Inclusion criteria:** Met the diagnostic criteria of the 4th National Cerebrovascular Disease Conference Confirmed by CT or MRI **Exclusion criteria:** Initial onset or previous seizures but no residual neurological dysfunction Receiving thrombolytic therapy or cerebral haemorrhagic surgery Subarachnoid haemorrhage TIA, RIND or disease progression **Participants:** detailed in Table **PROGRESS‐plus:** summarised in Table
Interventions	(1) Three‐stage rehabilitation ‐ Level 2 Level 2 (> 28 days post stroke): "transfer, standing, centre of gravity transfer, striding, walking, cane use, going up and down stairs, ADL, etc" (2) Standard care "trained at home on their own under the regular guidance of a rehabilitation therapist"
Outcomes	**Independence in ADL scales:** modified Barthel Index **Motor function scales:** Fugl Meyer Assessment **Measures of balance:** NR **Measures of gait velocity:** NR **Length of stay:** NR **Adverse events:** NR **Other measures:** National Institute of Health Stroke Scale (NIHSS) **Time points when outcomes were assessed:** "Before treatment and at 2, 3, 4, 5 and 6 months after treatment"
Comparison	**Amount of physical rehabilitation in groups:** differs **Categorisation of comparison:** 1. Physical rehabilitation versus no or limited physical rehabilitation (dose differs) (limited = < 50% of dose)
Funding & conflicts of interest	**Funding statement:** Funded by the National "Tenth Five‐Year Plan" Project (2001BA703B22). **Conflict of interest statement:** not reported
Synthesis	**Quantitative synthesis:**Analysis 1.1; Analysis 1.2; Analysis 2.1; Analysis 2.2
Notes	This study was published in Chinese and translated into English using Google Translate.

**Table:** Lu 2014

***Study characteristics***	
Methods	**Study aim:** to explore the effect of three stage rehabilitation treatment on limb motor function after stroke **Design:** parallel‐group RCT **Country:** China **Study recruitment and setting:** see Table **Involvement of patients/public/professionals/others:** no
Participants	**Study population (number randomised):** 180 **Inclusion criteria:** Diagnosed with CT or MRI including patients with cerebral infarction and cerebral haemorrhage **Exclusion criteria:** Long‐term chronic disease and cerebrovascular disease **Participants:** detailed in Table **PROGRESS‐plus:** summarised in Table
Interventions	(1) Three‐stage rehabilitation Within one month of stroke: "they receive early physical therapy in the ward; the second and third months after the onset of the disease are the second stage of treatment; the fourth to sixth months are the third stage. The second and third stages can be transferred to the outpatient rehabilitation treatment department or home for rehabilitation training according to the patient's personal situation, and the whole process is followed up by our specialists. The whole rehabilitation training process is divided into three levels. The main contents of the first‐level rehabilitation training are correct body position, active and passive training of limbs, waist and abdominal muscle training and breathing methods, sitting, lying and standing balance training. The training time is 1 hour per day. Teach the family members the correct nursing and auxiliary training methods, as a supplement to the treatment, so as to reduce the secondary injury of the patient's limbs. After the first‐level rehabilitation training, the patient should be able to get up and bed on his own. The main content of the second‐level rehabilitation training is the systematic training of standing, walking, and going up and downstairs, once a day in the morning and evening for 0.5 to 1 hour. The training purpose of the tertiary rehabilitation is to improve the patient's living ability, including being able to eat, groom, put on and take off clothes, and organize personal hygiene. The training time is 0.5 to 1h per day. In the second and third stages of rehabilitation treatment, if the patient is in the rehabilitation center, the therapist will help him to train. If he is at home, the hospital will send a special person to the patient's home to guide the training once a week." (2) Standard care "they did not receive any standardized and systematic rehabilitation treatment after the condition was stabilized. The patient only moves on his own after the doctor has described the precautions, and some family members will help the patient carryout rehabilitation training according to their personal understanding, but none of them belong to the standardized system of graded rehabilitation treatment"
Outcomes	**Independence in ADL scales:** NR **Motor function scales:** NR **Measures of balance:** NR **Measures of gait velocity:** NR **Length of stay:** NR **Adverse events:** NR **Other measures:** functional comprehensive assessment scale **Time points when outcomes were assessed:** baseline and after 1, 3, and 6 months of treatment
Comparison	**Amount of physical rehabilitation in groups:** differs **Categorisation of comparison:** 1. Physical rehabilitation versus no or limited physical rehabilitation (dose differs) (limited = < 50% of dose)
Funding & conflicts of interest	**Funding statement:** not reported **Conflict of interest statement:** not reported
Synthesis	**Qualitative synthesis:** no relevant outcomes measured
Notes	This study was published in Chinese and translated into English using Google Translate.

**Table:** Lu Liangyan 2014

***Study characteristics***	
Methods	**Study aim:** to investigate the effect of tertiary rehabilitation therapy on motor function recovery in patients with cerebral haemorrhage **Design:** parallel‐group RCT **Country:** China **Study recruitment and setting:** see Table **Involvement of patients/public/professionals/others:** no
Participants	**Study population (number randomised):** 88 **Inclusion criteria:** Diagnosed with cerebral haemorrhage according to the guidelines for the diagnosis and treatment of cerebrovascular disease Confirmed by CT Aged < 80 years Neurological deficit score > 8 points First occurrence of cerebral haemorrhage Informed consent **Exclusion criteria:** Subarachnoid haemorrhage Congestive heart failure Quadriplegia New bleeding during hospitalisation Malignant tumour History of dementia Liver, kidney or lung complications History of mental health condition Failure to follow‐up **Participants:** detailed in Table **PROGRESS‐plus:** summarised in Table
Interventions	(1) Three‐stage rehabilitation "The first stage: within 1 month of onset, that is, the acute stage of cerebral hemorrhage, all patients received routine treatment in the neurology department of the hospital. The patients in the group received rehabilitation therapy to prevent limb spasm, complications, etc., and maintain the correct body position. The specific rehabilitation contents include: placement of the affected limb in the sitting and lying position, breathing training, passive training of the joints of the affected limb, guidance of active movement of the healthy limb, acupuncture physiotherapy, and training in the elevated sitting position at the head of the bed, etc. During the training period, auxiliary training and nursing method training are carried out for the patient's family members and nurses to reduce the harm caused to the patient due to improper nursing, and also enable the patient to get part of the training during the non‐treatment period. 1.3.2 The second stage: at the end of the second to third months after the onset of the disease, the patient has been discharged from the hospital and came to the rehabilitation department of our hospital for recovery of limb motor function rehabilitation treatment. The specific rehabilitation contents include (1) good limb placement, health side rollover exercises on the affected side, maintenance of the range of motion of the joints, balance muscles in the sitting position, endurance training, standing balance and stance training, bridge exercises, postural turns, movement training, up and down stairs training etc. 2 times a day, 40 minutes each time, train 5 to 7 days a week. (2) According to the specific situation of the patient, continue acupuncture physiotherapy, hyperbaric oxygen therapy if necessary and dress, eat, go to the toilet etc, according to the degree of dysfunction of the patient. Regular activities, application training of fune motor items, roller exercise, slanted scrub board, desktop training board and other training, electric stimulation of the affected limb to promote function recovery, 20 minutes each time, once a day, treatment for 20‐30 days. During this period, family members and carers help the patient to complete the daily tasks. For functional training, the therapist is responsible for the main therapeutic work. 1.3.3. The third stage: 4 to 6 months after the onset. Activities of dialy living, requiring patients to carry out self‐rehabilitation at home. Rehabilitation exercise, you can also go to the community for rehabilitation exercise, during this periorid rehabilitation treatment. The therapist provides on‐site guidance to the patient's rehabilitation once every 2 weeks, mainly referring to guide and supervise pateints to carry out the first and second stage of basic training every day. 2 times a day, 40 minute each time, 6 days / week." (2) Standard care "only given general rehabilitation guidance"
Outcomes	**Independence in ADL scales:** Barthel Index, Functional Independence Measure (FIM) **Motor function scales:** NR **Measures of balance:** NR **Measures of gait velocity:** NR **Length of stay:** NR **Adverse events:** NR **Other measures:** Neurological Defect Degree Rating Scale, Brunnstrom scale **Time points when outcomes were assessed:** "Before rehabilitation, after level 1 rehabilitation, after level 2 rehabilitation, and level 3"
Comparison	**Amount of physical rehabilitation in groups:** differs **Categorisation of comparison:** 1. Physical rehabilitation versus no or limited physical rehabilitation (dose differs) (limited = < 50% of dose)
Funding & conflicts of interest	**Funding statement:** not reported **Conflict of interest statement:** not reported
Synthesis	**Quantitative synthesis:**Analysis 1.1
Notes	This study was published in Chinese and translated into English using Google Translate.

**Table:** Mai Guanghuai 2016

***Study characteristics***	
Methods	**Study aim:** "to observe the effects of intensive training on the lower limb in patients following cerebral stroke" **Design:** parallel‐group RCT **Country:** China **Study recruitment and setting:** see Table **Involvement of patients/public/professionals/others:** no
Participants	**Study population (number randomised):** 90 **Inclusion criteria:** Aged 30 to 75 years First ever stroke Less than 3 months since stroke onset Medically stable Cognitively able to follow rehabilitation Willing to participate and able to sign consent **Exclusion criteria:** Cognitive impairment Hearing and/or visual impairment Any condition of the lower limb that may preclude participation in rehabilitation Other neurological conditions Fall(s) Diseases of internal organs Lower limb strength < 3 on grading scheme (grading scheme not reported) Not able to participate in entire training **Participants:** detailed in Table **PROGRESS‐plus:** summarised in Table
Interventions	(1) Lower limb training In additional to conventional rehabilitation (described below) participants in the lower limb training group received lower limb motor intensive practice. Specific training included: Positional training involves change of position with joint movement control and heart position training. Stroke survivors were asked to lie supine, and the joints are kept in external rotation and abduction in a neutral position. Active control training, keep the healthy side in a neutral position, and the skeletal joints of the affected side actively perform adduction, internal rotation, abduction, and external rotation movements. Dynamic stability training, the affected footsteps on the bed (ensuring that the healthy side remains neutral position), adduction, internal rotation, abduction, and external rotation. Positioning and contraction training, the healthy side maintains a neutral position, and the affected side joints perform internal rotation and adduction position positioning and contraction exercises. Quick response training, the healthy side maintains in a neutral position, and the lower limb on the affected side quickly rotates from the external rotation position to stay in a neutral position and slightly deviates from the internal rotation position. Functional movements, step‐up training, try to make the panic joints without internal rotation and external rotation. Knee joint motion control training method: cardio‐ stretch knee short bow exercise, the stroke survivor lies on the back, the rehabilitation doctor resists and squeezes the affected foot, and at the same time does the knee extension exercise, the range of motion is <30 degrees; On the sliding bed, bend the knee joints at different angles, and keep the set angles without sliding down. Knee joint standing control training, the stroke survivor stands and the knee joint flexes and extends within a range of 30 degrees, which is reduced to 15 degrees range. Knee flexion start control training, the knee joint gradually moves from the straight position to the 30 degrees range of flexion and narrows to the 15 degrees range as the function improves. Single‐leg up right knee extension motion control, both knees flex 30 degrees, the non‐paretic side lower limb lifted off the ground and support the body with the lower limb on the affected side. Single‐leg upright and knee‐bending movement control, the stroke survivor stands, and lift the lower limb of the healthy side forward as much as possible as soon as possible. After completing the same routine rehabilitation training as the control group (see 2 below), participants in this group performed 20 intensive training for lower extremity motor control and between 3 to 6 items of the above training content tailored to the specific situation of the stroke survivor, from easy to difficult. Training was carried out 6 days/week over a total of 6 weeks. (2) Conventional rehabilitation This involved conventional medical treatment and routine rehabilitation training, which consisted of relaxation massage (calf and triceps), triceps stretching training and muscle strength training, one session /day (20 mins). At the same time the participant was also walking up and down stairs and undertaking walking training; 2 sessions/day (20 mins/session). Conventional rehabilitation training was carried out 6 days/week over a total of 6 weeks.
Outcomes	**Independence in ADL scales:** Barthel Index **Motor function scales:** Fugl Meyer assessment **Measures of balance:** Berg Balance Scale **Measures of gait velocity:** NR **Length of stay:** NR **Adverse events:** NR **Other measures:** functional ambulation categories (Holden) **Time points when outcomes were assessed:** before and after treatment
Comparison	**Amount of physical rehabilitation in groups:** differs **Categorisation of comparison:** 5. Additional therapy + usual therapy versus usual therapy (dose differs)
Funding & conflicts of interest	**Funding statement:** not reported **Conflict of interest statement:** not reported
Synthesis	**Quantitative synthesis:**Analysis 5.1; Analysis 5.2; Analysis 5.3
Notes	This study was published in Chinese and relevant sections translated into English by our author team (PLC).

**Table:** Mansfield 2018

***Study characteristics***	
Methods	**Study aim:** to determine if perturbation based balance training can reduce falls in daily life among individuals with chronic stroke **Design:** parallel‐group RCT **Country:** Canada **Study recruitment and setting:** see Table **Involvement of patients/public/professionals/others:** no
Participants	**Study population (number randomised):** 88 **Inclusion criteria:** Community‐dwelling adults with chronic stroke (> 6 months post‐stroke) Able to stand independently without upper limb support for > 30 s and tolerate at least 10 postural perturbations **Exclusion criteria:** > 2.1 m tall and/or weighing > 150 kg Other neurological conditions Lower extremity amputation Unable to understand instructions in English Recent (last 6 months) significant illness Injury or surgery Severe osteoporosis (diagnosis of osteoporosis with fracture) Poorly controlled diabetes or hypertension Contraindications to physical exercise Receiving physiotherapy or supervised exercise targeting balance and mobility between the time of recruiting and the post‐training assessment and/or received PBT in the year before enrolment **Participants:** detailed in Table **PROGRESS‐plus:** summarised in Table
Interventions	(1) Perturbation‐based balance training (PBT) "PBT sessions included a 5–10 min warm‐up, voluntary tasks intended to induce internal perturbations, voluntary tasks combined with external perturbations and a 5–10 min cool‐down. Participants were supervised by the physiotherapist and wore a custom safety harness (ABG Concept Médical, Valcourt, Quebec, Canada) attached to the overhead support. Internal perturbations occurred when participants failed to control balance during voluntary movement; ‘agility’ tasks, such as kicking a soccer ball, were used to induce internal perturbations. External perturbations were caused by forces outside participants’ control (eg, push or pull from the physiotherapist). We aimed for at least 60 postural perturbations per session and set the task difficulty such that participants required an upper extremity response, external assistance (ie, from the overhead harness or physiotherapist) or a multistep response~50% of the time. The progression in voluntary tasks occurred on a continuum from stable to mobile and from predictable to unpredictable. Additionally, progression occurred by increasing the magnitude ofexternal perturbation, or imposing sensory or environ‐mental challenges." (2) Control group ‐ exercise "The control group completed the Keep Moving with Stroke programme. This is an exercise programme for community‐dwelling individuals with stroke, based on balance and mobility interventions evaluated in clinical trials. This programme was designed to be delivered in a group but was delivered 1:1 in this study to match attention received from the physiotherapist by the PBT group. Each session included a 5–10min warm‐up, 40 min of mobility and balance exercises and a 5–10min cool‐down with stretching. Exercises included walking, sit‐to‐stand, heel raises, walking while carrying an object, tap‐ups or step‐ups (forward and sideways), reaching and weight shifting, and standing with reduced base of support." "Participants completed two 1‐hour training sessions perweek for 6 weeks and two 1‐hour ‘booster’ training sessions 3 months and 9 months after the initial training period. Interventions were administered by a physiotherapist (CJD or SK) on a 1:1 basis."
Outcomes	**Independence in ADL scales:** NR **Motor function scales:** NR **Measures of balance:** Berg Balance Scale **Measures of gait velocity:** timed up and go test **Length of stay:** NR **Adverse events:** adverse events, serious adverse events **Other measures:** falls, mini‐Balance Evaluations Systems test, activities‐specific balance confidence questionnaire, Physical Activity Scale for Individuals with Physical Disabilities, Subjective Index of Physical & Social Outcome **Time points when outcomes were assessed:** immediately before, immediately after, and 6 months and 12 months after the end of the initial training period
Comparison	**Amount of physical rehabilitation in groups:** equivalent **Categorisation of comparison:** 3.(i) Comparison of different approaches (dose equivalent; with no other physical rehab)
Funding & conflicts of interest	**Funding statement:** Canadian Institutes of Health Research (MOP 133577). The authors also acknowledge the support of the Toronto Rehabilitation Institute; equipment and space have been funded with grants from the Canada Foundation for Innovation, Ontario Innovation Trust and the Ministry of Research and Innovation. AM holds a New Investigator Award from the Canadian Institutes of Health Research (MSH 141983). DB holds a Canada Research Chair **Conflict of interest statement:** none
Synthesis	**Quantitative analysis**: adverse events ‐ Analysis 7.6 **Qualitative synthesis:** Other results data not presented in a format suitable for quantitative analysis.
Notes	Due to difficulty recruiting, the protocol was amended to allow individuals < 50 years old to participate.

**Table:** Marigold 2005

***Study characteristics***	
Methods	**Study aim:** to determine the effect of 2 different community‐based group exercise programmes on functional balance, mobility, postural reflexes, and falls in older adults with chronic stroke **Design:** parallel‐group RCT **Country:** Canada **Study recruitment and setting:** see Table **Involvement of patients/public/professionals/others:** no
Participants	**Study population (number randomised):** 61 **Inclusion criteria:** Adults aged 50 years+ living in the community Single stroke > 12 months from onset Able to walk, with or without an assistive device for a minimum of 10 m Activity tolerance of 60 mins with rest intervals **Exclusion criteria:** Medically unstable Neurological conditions unrelated to stroke (e.g. Parkinson’s disease) or severe musculoskeletal conditions (e.g. recent joint replacement surgery, amputation) MMSE < 22 BBS > 52/56 (i.e. minimal balance deficit) **Participants:** detailed in Table **PROGRESS‐plus:** summarised in Table
Interventions	(1) Agility exercise programme "The agility exercise program challenged dynamic balance, and the tasks progressively increased in difficulty based on set criteria and dependent on an individual’s ability. This program emphasized agility and a multisensory approach. Tasks included standing in various postures (e.g., tandem or feet apart, one‐foot stance, and weight‐shifting) and walking with various challenges (e.g., different step lengths and speeds, tandem walking, figure‐eight walking, stepping up and over low risers, side stepping, crossover stepping, and stepping over obstacles). Additional exercises included sit‐to‐stand movements, rapid knee raise while standing, and standing perturbations (i.e., instructor pushing participant in a con‐trolled manner or participant pushing instructor to destabilize balance and elicit postural reflexes). Eyes‐closed conditions and foam surfaces were incorporated for many of the tasks." (2) Stretching/weight‐shifting exercise programme "The stretching/weight‐shifting exercise program focussed on slow, low‐impact movements consisting of stretching and weight shifting. Weight‐shifting exercises incorporated taichi–like movements and reaching tasks, which encouraged increased force to be taken through the paretic lower limb. Stretching of major muscle groups was performed while standing and on mats on the floor. The act of getting down, on, and up from the floor was considered an exercise in itself and was practiced with the aid of the instructors." "The two exercise programs consisted of 1‐hour sessions, three times a week for 10 weeks held at a local community center supervised by three instructors (physical therapist, kinesiologist, and recreation therapist). There were six classes (three for each exercise program) with a 1:3 instructor:participant ratio. Each of the exercise programs began with a 5‐minute warm‐up consisting of walking and light stretching and ended with a 5‐minute cool‐down of light stretching."
Outcomes	**Independence in ADL scales:** NR **Motor function scales:** NR **Measures of balance:** Berg Balance Scale **Measures of gait velocity:** timed up and go **Length of stay:** NR **Adverse events:** NR **Other measures:** step reaction time, activities specific balance confidence, Nottingham Health Profile, standing postural reflexes and induced falls **Time points when outcomes were assessed:** before, immediately after, and 1 month after the intervention
Comparison	**Amount of physical rehabilitation in groups:** equivalent **Categorisation of comparison:** 3.(i) Comparison of different approaches (dose equivalent; with no other physical rehab)
Funding & conflicts of interest	**Funding statement:** supported by an operating grant from the Canadian Institutes of Health Research (CIHR) (MOP‐57862), salary support to JJE from CIHR and the Michael Smith Foundation for Health Research (MSFHR), and trainee support to DSM from MSFHR and the Natural Sciences and Engineering Research Council of Canada. **Conflict of interest statement:** not reported
Synthesis	**Quantitative synthesis:**Analysis 7.3; Analysis 7.4; Analysis 8.3; Analysis 8.4
Notes	

**Table:** Martins 2020

***Study characteristics***	
Methods	**Study aim:** to investigate the efficacy of TSCT, focused on both upper and lower limbs, in improving PA levels and mobility as well as muscle strength, exercise capacity, and quality of life in stroke survivors **Design:** parallel‐group RCT **Country:** Brazil **Study recruitment and setting:** see Table **Involvement of patients/public/professionals/others:** no
Participants	**Study population (number randomised):** 36 **Inclusion criteria:** Clinical diagnosis of stroke of at least 6 months ≥ 19 years of age Able to walk 10 m independently Muscle tone of the elbow flexor muscles < 4 on the MAS Inactive or insufficiently active based on standardised criteria Medical permission for regular practice of monitored PA **Exclusion criteria:** Cognitive impairments (based on MMSE) and/or comprehensive aphasia History of severe heart disease and/or uncontrolled blood pressure Pain or other adverse health conditions which could compromise the performance of the tests or their participation in the proposed interventions **Participants:** detailed in Table **PROGRESS‐plus:** summarised in Table
Interventions	(1) Task‐specific circuit training 30 minutes of UL and 30 minutes of LL tasks. Circuit comprising activities of "reaching, grasping, manipulation of different objects, writing, sit‐to‐stand, step and heel raise activities, and walking". 5 minutes of exercise in each work station, except for gait training with auditory cue, which lasted 10 minutes. (2) Control group "40 minutes of static global stretching and 20 minutes of memory exercises, and/or health education sessions. Stretches were performed in three‐series of 30 seconds and involved different muscle groups. Most of the stretches were performed in a sitting or lying position. When participants could not self stretch, they received help" "all participants received 60 minute interventions, in groups of two to six, three times a week over 12 weeks, totalling 36 sessions"
Outcomes	**Independence in ADL scales:** NR **Motor function scales:** NR **Measures of balance:** NR **Measures of gait velocity:** 10 m walk test **Length of stay:** NR **Adverse events:** NR **Other measures:** Physical Activity levels, Human Activity Profile, UL Mobility (TEMPA), muscle strength, exercise capacity (6‐minute walk test), quality of life (stroke specific quality of life scale) **Time points when outcomes were assessed:** baseline, post‐intervention (week 12), follow‐up (week 16)
Comparison	**Amount of physical rehabilitation in groups:** differs **Categorisation of comparison:** 2. Physical rehabilitation versus attention control (dose of physical rehab differs; dose of intervention equivalent)
Funding & conflicts of interest	**Funding statement:** funded by the following research funding agencies: Coordenação de Aperfeiçoamento de Pessoal Ensino Superior (CAPES – Financial Code 001), Conselho Nacional de Desenvolvimento Científico e Tecnológico (CNPq), Fundação de Amparo à Pesquisa de Minas Gerais (FAPEMIG), and Pro‐reitoria de Pesquisa da Universidade Federal de Minas Gerais (PRPq/UFMG) **Conflict of interest statement:** none
Synthesis	**Quantitative synthesis:**Analysis 3.4; Analysis 4.4
Notes	

**Table:** Matthew Hall 2013

***Study characteristics***	
Methods	**Study aim:** to investigate the effect of task‐oriented method on exercise retraining of lower limb dysfunction in stroke patients **Design:** parallel‐group RCT **Country:** China **Study recruitment and setting:** see Table **Involvement of patients/public/professionals/others:** no
Participants	**Study population (number randomised):** 40 **Inclusion criteria:** Met the diagnostic criteria established by the 4th National Cerebrovascular Conference Aged < 70 years Physical dysfunction No language or communication barriers Will to recover and can actively co‐operate with trainer Able to provide informed consent Hospitalised for 90 days **Exclusion criteria:** Not reported **Participants:** detailed in Table **PROGRESS‐plus:** summarised in Table
Interventions	(1) Task‐oriented training "the observation group gradually increased transfer training, sitting‐stand training, balance training, walking training, and up‐and‐down stairs training, etc. The above rehabilitation training lasted 45‐60 min each time, once a day, and received 6 d of weekly treatment. And by using the specific intervention of task‐oriented method to retrain lower extremity function. In the training process, it is emphasized that the patient's attention is focused on the goal of the movement rather than the movement itself, and the therapist gradually reduces the feedback of the patient's language and the guidance of the body. In this process, the therapist adopts the principle of step‐by‐step and uses the key points of control to intervene and suppress the abnormal movement patterns of patients through repeated training from three aspects: injury level, strategy level, and adaptability. At the same time, pay attention to environmental training, and let patients train in as lightly darker light, on a different floor, andi n a different room. The therapist always pays attention to maintaining the correctness and safety of the patient's posture during the training process." (2) Standard care "The patients in the control group and the observation group were given treatments such as nourishing nerves, regulating intracranial pressure, and preventing infection in the early stage of the disease. The patients were guided to place their limbs in a good position, and the therapists carried out passive movements of the limbs and joints. After the 2‐week acute phase, patients with stable conditions were treated with traditional acupuncture, massage techniques and neurodevelopmental therapy according to the recovery of the patient"
Outcomes	**Independence in ADL scales:** modified Barthel Index **Motor function scales:** Fugl‐Meyer assessment **Measures of balance:** Berg Balance Scale **Measures of gait velocity:** NR **Length of stay:** NR **Adverse events:** NR **Other measures:** NR **Time points when outcomes were assessed:** before and after training
Comparison	**Amount of physical rehabilitation in groups:** unsure **Categorisation of comparison:** not possible to determine whether the dose of physical rehabilitation was similar in both groups (i.e. comparison of different approaches), or differed.
Funding & conflicts of interest	**Funding statement:** Fund Project: Jinan Military Region Logistics Scientific Research Project (CJN12L062) **Conflict of interest statement:** not reported
Synthesis	**Qualitative synthesis:** insufficient information to enable categorisation of comparison
Notes	This study was published in Chinese and translated into English using Google Translate.

**Table:** Ma Xue 2010

***Study characteristics***	
Methods	**Study aim:** "to observe the impact of individualized active rehabilitation exercises on the motion function of lower limbs of stroke patients with hemiplegia" **Design:** parallel‐group RCT **Country:** China **Study recruitment and setting:** see Table **Involvement of patients/public/professionals/others:** no
Participants	**Study population (number randomised):** 60 **Inclusion criteria:** Met the diagnostic criteria for the Fourth National Cerebrovascular Disease Conference Aged 40 to 80 years Stroke onset within 2 to 12 weeks First stroke accompanied by hemiplegia Medically stable No obvious cognitive impairment Can follow verbal instructions Able to sign a consent form **Exclusion criteria:** Not reported **Participants:** detailed in Table **PROGRESS‐plus:** summarised in Table
Interventions	Both groups received routine medical treatment and started rehabilitation once they were medically stable. (1) Rehabilitation "Active", including Bobath approach, motor relearning, Rood, speech therapy. Active training was individualised for each participant and involved a combination of exercise therapy, physical factor therapy and dynamic and static balance instrument therapy. In the early stage this involved turning over regularly in the early stage of standing, adopting lateral and supine positions, and lying on the healthy side more often, to prevent the upper limb and shoulder joint of the affected side from being compressed. The paretic limb was placed in a functional position, and lower limbs mainly flexed to prevent joint contracture and spasm. Other exercises involved: Active and passive activities of limb joints on the bed Facilitated training of lower limb voluntary movement: including joint control training and flexion lower limb facilitation training, Lower limb weight training Lower limb extension movement induction, facilitation, strengthening training and upper and lower limb control training. Strengthening and balance training ‐ sitting and standing Walking preparation training, walking training, gait correction and training of going up and down stairs ADL training and configuration and use of orthotics and other auxiliary devices Application of myoelectric biofeedback. (2) Attention control Participants in this group received mainly passive exercise training: including maintenance training of joint range of motion, bed and bed and chair transfer training and gait training. Note: similar interventions to Yang Jian 2007 (but this does not appear to be the same, or an overlapping, study).
Outcomes	**Independence in ADL scales:** NR **Motor function scales:** Fugl‐Meyer assessment **Measures of balance:** NR **Measures of gait velocity:** NR **Length of stay:** NR **Adverse events:** NR **Other measures:** NR **Time points when outcomes were assessed:** before and after treatment
Comparison	**Amount of physical rehabilitation in groups:** equivalent **Categorisation of comparison:** 2. Physical rehabilitation versus attention control (dose of physical rehab differs; dose of intervention equivalent)
Funding & conflicts of interest	**Funding statement:** Special support from the Health Bureau of Xuhui District, Shanghai (Project No. SHXH2 0050101) **Conflict of interest statement:** not reported
Synthesis	**Quantitative synthesis:**Analysis 3.2
Notes	This study was published in Chinese and relevant sections translated into English by our author team (PLC).

**Table:** McClellan 2004

***Study characteristics***	
Methods	**Study aim:** "to investigate the efficacy of resource‐efficient physiotherapy services in improving mobility and quality of life after stroke" **Design:** parallel‐group RCT **Country:** Australia **Study recruitment and setting:** see Table **Involvement of patients/public/professionals/others:** no
Participants	**Study population (number randomised):** 26 **Inclusion criteria:** Stroke < 18 months previously Aged over 45 years Living in community Score greater than 0 and less than 6 on item 5 of MAS, and less than 6 on items 7 and 8 of MAS **Exclusion criteria:** Not reported **Participants:** detailed in Table **PROGRESS‐plus:** summarised inTable
Interventions	(1) Motor learning (n = 15) "Intervention was standardised by prescribing the first five exercises that the subject could perform successfully from a list of 23 predetermined exercises. The exercises were arranged loosely hierarchically, based on their challenge to balance...exercises were progressed systematically..." Length of intervention period: 6 weeks Number of sessions and length of individual sessions: instructed to practise twice per day with videotape. Telephoned after one week. Returned for exercise review at end of weeks two and four. Record of practice kept for six weeks Intervention provider: exercises prescribed by physiotherapist (2) Attention control (motor learning, upper limb) (n = 11) Similar to above, but aimed at improving function of the affected upper limb Length of intervention period: 6 weeks Number of sessions and length of individual sessions: instructed to practise twice per day with videotape. Telephoned after one week. Returned for exercise review at end of weeks 2 and 4. Record of practice kept for 6 weeks. Intervention provider: exercises prescribed by physiotherapist
Outcomes	**Independence in ADL scales:** NR **Motor function scales:** MAS (item 5) **Measures of balance:** NR **Measures of gait velocity:** NR **Length of stay:** NR **Adverse events:** NR **Other measures:** Functional Reach Test **Time points when outcomes were assessed:** "were measured prior to, immediately after, and two months after intervention"
Comparison	**Amount of physical rehabilitation in groups:** differs **Categorisation of comparison:** 2. Physical rehabilitation versus attention control (dose of physical rehab differs; dose of intervention equivalent)
Funding & conflicts of interest	**Funding statement:** "Physiotherapy Research Foundation, Health Research Foundation Sydney South West, and the Illawarra Area Health Service for funding" **Conflict of interest statement:** not reported
Synthesis	**Quantitative synthesis:**Analysis 3.2; Analysis 4.2
Notes	

**Table:** Medina‐Rincón 2019

***Study characteristics***	
Methods	**Study aim:** to design, validate, and assess the feasibility of a comprehensive exercise programme intervention to improve balance impairments in poststroke older adults **Design:** parallel‐group RCT **Country:** Spain **Study recruitment and setting:** see Table **Involvement of patients/public/professionals/others:** partial
Participants	**Study population (number randomised):** 14 **Inclusion criteria:** Post‐stroke (ischaemic or haemorrhagic) adults admitted to a rehabilitation unit of an intermediate care hospital Sitting trunk control **Exclusion criteria:** Stroke occurred > 3 months before admission Presence of previous severe functional dependence i.e. BI ≤ 60 Previous orthopaedic, neurological, or other impairments that could influence balance Wernicke aphasia or mixed aphasia previous or after stroke Delirium at admission Possible cognitive impairment, i.e. MMSE ≤ 24 Previous visual deficits **Participants:** detailed in Table **PROGRESS‐plus:** summarised in Table
Interventions	(1) Balance impairment rehabilitation exercise Level 1 ‐ stimulation by foot pressure on the foot support points (pressure by physiotherapist, weight‐bearing, sit‐to‐stand). Level 2 ‐ unbalance exercise while standing (standing perturbations, one‐legged knee bends). "Participants in the intervention group received usual‐care treatment (45 minutes/day) plus 15 minutes/day of the post‐stroke rehabilitation programme proposed, 5 times/week, 4 weeks in total (20 sessions)." (2) Usual care "Participants included in the usual‐care arm, received the usual post‐stroke rehabilitation programme, which included a 4‐weeks period, 60 minutes/session, 5 times a week (20sessions) of physiotherapy techniques and exercises (eg, tone facilitation, stretching, passive mobilization, and range‐of‐motion exercises for the paretic side and walking between parallel bars). As part of the multidisciplinary interventionin the intermediate care hospital, participants received also occupational therapy and nursing and medical care. Additionally, activities to promote postural control and task directed movements were performed."
Outcomes	**Independence in ADL scales:** NR **Motor function scales:** NR **Measures of balance:** NR **Measures of gait velocity:** NR **Length of stay:** NR **Adverse events:** NR **Other measures:** Mini‐BESTest **Time points when outcomes were assessed:** NR
Comparison	**Amount of physical rehabilitation in groups:** differs **Categorisation of comparison:** 5. Additional therapy + usual therapy versus usual therapy (dose differs)
Funding & conflicts of interest	**Funding statement:** no funding support **Conflict of interest statement:** none
Synthesis	**Qualitative synthesis:** no relevant outcome measures assessed
Notes	

**Table:** Meier 2021

***Study characteristics***	
Methods	**Study aim:** pilot study to design a larger RCT, which should further test the hypothesis that coordinative training is more effective at improving balance in people with ataxia after acute stroke than conventional physiotherapy **Design:** parallel‐group RCT **Country:** Austria **Study recruitment and setting:** see Table **Involvement of patients/public/professionals/others:** no
Participants	**Study population (number randomised):** 7 **Inclusion criteria:** Not reported **Exclusion criteria:** Not reported **Participants:** detailed in Table **PROGRESS‐plus:** summarised in Table
Interventions	(1) Co‐ordinative training (Doris‐Broetz Concept) "Participants receive ten 45 minute sessions of coordinative training (based on the Doris‐Broetz‐Concept). This treatment involves a range of exercises to improve static and dynamic balance control and coordination skills and to reduce fear and stiffness. The main aims of this therapy method is to define goals based on problems in daily living, “Sway – don't make yourself stiff”, exercise every day, coordinative challenging movements, doing lots of repetitions by having variation. The exercises differ in difficulty depending on the patient." (2) Conventional physical therapy "Participants receive ten 45 minute sessions of physical therapy according to current practice. Treatment will include active exercises including balance training, gait training, core stability and training of ADL's (activities of daily living). The exercises will differ in difficulty depending on the patient." "For participants in both groups, a home exercise program is also given to be completed by the patient for 15 minutes every day, starting on day 2. Home exercise should be continued for another four weeks after post‐intervention testing."
Outcomes	**Independence in ADL scales:** Scores of Independence for Neurologic and Geriatric Rehabilitation (SINGER) **Motor function scales:** NR **Measures of balance:** Berg Balance Scale* **Measures of gait velocity:** timed up and go test **Length of stay:** NR **Adverse events:** NR **Other measures:** functional ambulation categories*, Trunk Control Test, EQ 5D 3L, adverse events **Time points when outcomes were assessed:** pre and post (day 16) intervention, and 12‐month follow‐up *Data only published for these outcomes
Comparison	**Amount of physical rehabilitation in groups:** equivalent **Categorisation of comparison:** 3.(i) Comparison of different approaches (dose equivalent; with no other physical rehab)
Funding & conflicts of interest	**Funding statement:** not reported **Conflict of interest statement:** not reported
Synthesis	**Qualitative synthesis only:** insufficient information to categorise
Notes	

**Table:** Mendoza 2015

***Study characteristics***	
Methods	**Study aim:** to gather preliminary information to compare the effects of a task‐oriented circuit class program and an impairment‐focused circuit class program on the walking ability among patients with chronic stroke **Design:** parallel‐group RCT **Country:** Philippines **Study recruitment and setting:** see Table **Involvement of patients/public/professionals/others:** No
Participants	**Study population (number randomised):** 18 **Inclusion criteria:** Stroke that occurred ≥ 6 months ago Able to follow 3‐step instructions in English or Filipino Unable to walk 0.8 km of level surface and/or climb at least one flight of stairs without support or assistance Able to ambulate at least 10 m with or without a gait aid **Exclusion criteria:** Already receiving exercise training or physical therapy Multiple strokes, or medically unstable or severe cardiovascular, musculoskeletal, and neurological conditions Unable to did not ambulate independently in the community or walked with an assistive device prior to the stroke **Participants:** detailed in Table **PROGRESS‐plus:** summarised in Table
Interventions	(1) Circuit training ‐ task group "the participants performed ten different functional tasks as shown in Table 2. These functional tasks mimicked the different mobility demands inside and outside the home. The group was instructed to perform the activities with as many repetitions as possible and as safely as they could for five minutes before moving to the next station." Functional tasks ‐ ascending/descending ramp and stairs, walking, heel lifting, sit to stand, stepping, walking on different surfaces, walking while carrying, walking while turning head, sit‐to‐stand‐to‐sit from different heights, walking on floor markers (2) Circuit training ‐ impairment group "The impairment group performed impairment‐focused exercises as shown in Table 3. Exercises were based on the exercise protocol for older adults published by Tufts University and the United States Centers for Disease Control and Prevention(30). The exercises aimed to improve muscle strength, weight‐bearing symmetry, and gait symmetry. Exercises for each station lasted for 5 to 7 minutes. The therapists tailored the progression of the exercises to each participant’s level of ability. The number of sets were increased when the exercise was deemed no longer challenging enough for the participant." Impairment exercises ‐ pelvic bridging, hamstring curls, knee extension, hip abduction, step ups, weight shifting, walking. "The participants in both groups received the allocated treatment three times a week for four weeks with each session lasting for 60 minutes." "Rest periods for both circuit class therapy groups were provided, if needed. Both groups started and ended their sessions with supervised standardized warm‐up and cool‐down periods comprised of walking, and range of motion and stretching exercises of the extremities and trunk for five minutes."
Outcomes	**Independence in ADL scales:** NR **Motor function scales:** NR **Measures of balance:** NR **Measures of gait velocity:** 6‐minute walk test, 10 m walk test **Length of stay:** NR **Adverse events:** NR **Other measures:** timed up and down stairs **Time points when outcomes were assessed:** pre‐ and post‐treatment
Comparison	**Amount of physical rehabilitation in groups:** equivalent **Categorisation of comparison:** 3.(i) Comparison of different approaches (dose equivalent; with no other physical rehab)
Funding & conflicts of interest	**Funding statement:** self‐funded by the authors and had no external funding **Conflict of interest statement:** none
Synthesis	**Quantitative synthesis:**Analysis 7.4
Notes	Abstract only

**Table:** Meng 2022

***Study characteristics***	
Methods	**Study aim:** "compared the clinical effectiveness of early integrated RAGT using the Walkbot robotic gym with an intensity‐matched enhanced lower limb therapy (ELLT) program and with conventional rehabilitation therapy (CRT) in patients with acute ischemic stroke" **Design:** multi‐arm RCT (3‐arms) **Country:** China **Study recruitment and setting:** see Table **Involvement of patients/public/professionals/others:** no
Participants	**Study population (number randomised):** 192 **Inclusion criteria:** Aged 18 to 80 years old First‐ever, unilateral, ischaemic stroke confirmed by CT or MRI Stroke onset < 48 hours prior to inclusion Able to comprehend research‐related information FAC score < 4 Limitations in walking independently **Exclusion criteria:** Medically unstable MMSE < 23 Conditions that limited the use of the lower limb prior to presentation of stroke Severe cardiomyopathy and other heart diseases or comorbidities that could restrict daily activities **Participants:** detailed in Table **PROGRESS‐plus:** summarised in Table
Interventions	(1) Robot‐assisted gait training (RAGT) [Note ‐ this intervention group is not relevant to this review]. (2) Enhanced lower limb therapy (ELLT) "The ELLT program aims to match the intensity and duration of RAGT sessions, consisting of muscle strength training, passive stretching training, sit‐to‐stand training, bed‐wheelchair training, stepping training, balance training, and walking training. ELLT aims to drive neuroplasticity and motor recovery after stroke using the principles of person‐centered goal‐setting and repetitive functional task practice" (3) Conventional rehabilitation "included training through joint ROM, muscle strength training, balance training, and exercise therapy in clinical routine." "All groups were trained for 45 min each day, 3 days per week, for four consecutive weeks, and were controlled for other necessary medications and adjunctive treatments". "All participants were treated with comprehensive rehabilitation, including physical, occupational, and speech therapies. Differences among the three groups were observed only in terms of physical therapy components."
Outcomes	**Independence in ADL scales:** Barthel Index **Motor function scales:** NR **Measures of balance:** NR **Measures of gait velocity:** gait velocity, 6‐minute walk test, timed up and go test **Length of stay:** NR **Adverse events:** NR **Other measures:** Functional Ambulation Category, gait parameters, stroke specific quality of life scale **Time points when outcomes were assessed:** before and after 4 weeks of treatment
Comparison	**Amount of physical rehabilitation in groups:** equivalent **Categorisation of comparison:** 3.(i) Comparison of different approaches (dose equivalent; with no other physical rehab)
Funding & conflicts of interest	**Funding statement:** "disclosed receipt of the following financial support for the research, authorship, and/or publication of this article: This study was supported by the Shanghai Science and Technology Commission Project (grant nos22Y31900204, 19441908500, and 20142202900) and the National Natural Science Foundation of China (grant nos 82101537, 82071206, and81771131)" **Conflict of interest statement:** "no potential conflicts of interest with respect to the research, authorship, and/or publication of this article"
Synthesis	**Quantitative synthesis:**Analysis 7.1; Analysis 7.4
Notes	

**Table:** Meng Fanda 2021

***Study characteristics***	
Methods	**Study aim:** "to explore the effect of routine rehabilitation training on stroke sequelae" **Design:** parallel‐group RCT **Country:** China **Study recruitment and setting:** see Table **Involvement of patients/public/professionals/others:** no
Participants	**Study population (number randomised):** 40 **Inclusion criteria:** All stroke cases clinically diagnosed using routine examinations; hemiplegia of varying degrees Provided informed consent **Exclusion criteria:** Impaired consciousness Severe organ dysfunction Malignant tumours **Participants:** detailed in Table **PROGRESS‐plus:** summarised in Table
Interventions	(1) Rehabilitation "The training contents mainly included: (1) Physical rehabilitation training: 1) After the patient regains consciousness, according to the actual situation of the patient, according to the principle of training strength from weak to strong, and training time from short to long, the patient is trained, 30min/time, 1~2 times/cL 2) If the patient's condition is more serious, help the patient to turn over (3h/time) and slap the patient's back at the same time; at the same time, properly change the body position (mainly the lateral position and the supine position) to prevent foot drop and foot valgus. In addition, the patient should be given full body massage, and appropriate limb training should be carried out according to the specific conditions of the patients, such as body training, turning over the affected and healthy limbs, etc. (3min/time). Upper limb rehabilitation. 3) Lower extremity training: After the patient's condition is stable, the patient can be guided to perform standing and balance rehabilitation training. By getting up and standing on the bed, gradually move towards independent standing and walking. If necessary, a lower extremity orthosis can be used for auxiliary training. The training process follows a step‐by‐step principle. 4) After the patient's physical activity has improved, the patient can be guided to perform exercises such as swimming and Tai Chi (3min/time), and the weight‐bearing time can be gradually extended to train the strength of the lower limbs. (2) Language rehabilitation training. Starting from simple pronunciation, according to the order from easy to difficult, the patients are given language rehabilitation training, 30min/time, 1~2 times/d." (2) Routine care "The control group received routine nursing care, which mainly included routine medication guidance, dietary guidance, and psychological guidance. "
Outcomes	**Independence in ADL scales:** NR **Motor function scales:** Fugl Meyer assessment **Measures of balance:** Berg Balance Scale **Measures of gait velocity:** NR **Length of stay:** NR **Adverse events:** NR **Other measures:** National Institutes of Health Stroke Scale (NIHSS score) **Time points when outcomes were assessed:** before and after treatment
Comparison	**Amount of physical rehabilitation in groups:** differs **Categorisation of comparison:** 1. Physical rehabilitation versus no or limited physical rehabilitation (dose differs) (limited = < 50% of dose)
Funding & conflicts of interest	**Funding statement:** not reported **Conflict of interest statement:** not reported
Synthesis	**Quantitative synthesis:**Analysis 1.2; Analysis 1.3
Notes	This study was published in Chinese and translated into English using Google Translate.

**Table:** Meng Qingling 2015

***Study characteristics***	
Methods	**Study aim:** to investigate the effect of early targeted limb task function or strength training on limb function and living ability in hemiplegic patients with cerebral infarction **Design:** parallel‐group RCT **Country:** China **Study recruitment and setting:** see Table **Involvement of patients/public/professionals/others:** no
Participants	**Study population (number randomised):** 90 **Inclusion criteria:** First cerebral infarction confirmed by CT Limb dysfunction, i.e. hemiplegia **Exclusion criteria:** Not reported **Participants:** detailed in Table **PROGRESS‐plus:** summarised in Table
Interventions	(1) Functional training "Group A was given task functional training, and focused on functional training in targeted limb training". "In the practice process, the daily affairs that can be done can be arranged arbitrarily to promote learning and simulate the activities in real life. The implementation method is carried out according to the literature " (2) Strength training "group B was given strength training, and focused on targeted limb training with strength training". "resistance exercise was mainly used to improve muscle strength, and the specific method was carried out according to the literature" "Both groups were given routine neurological nursing treatment and early limb function training." "Both groups were given the following training: (1) The patient's position was changed regularly every day: independent turning to the affected side and independent turning to the unaffected side. (2) Maintain the appropriate limb position of the patient: keep the affected limb in the functional position, including the affected side lying position and the healthy side lying position. ( 3) Do joint activity training: Do joint training 4 times in all directions every day, 15 times/time. (4) Sit up and sit balance training. (5) Targeted limb training: [see above]. The training types of the two groups did not overlap, and at the same time, it was ensured that the patients had no symptoms of fatigue and other discomfort during the next day's training. (6) Abilities of daily living training: including the patient eating, brushing teeth and dressing alone, 2 times/d, 15min/time"
Outcomes	**Independence in ADL scales:** Barthel Index **Motor function scales:** Fugl Meyer Assessment **Measures of balance:** NR **Measures of gait velocity:** NR **Length of stay:** NR **Adverse events:** NR **Other measures:** NR **Time points when outcomes were assessed:** admission and 3rd and 6th month after training
Comparison	**Amount of physical rehabilitation in groups:** equivalent **Categorisation of comparison:** 3.(ii) Comparison of different approaches (dose equivalent; with both/all groups also receiving other physical rehab)
Funding & conflicts of interest	**Funding statement:** not reported **Conflict of interest statement:** not reported
Synthesis	**Quantitative synthesis:**Analysis 7.1; Analysis 7.2; Analysis 8.1; Analysis 8.2
Notes	This study was published in Chinese and translated into English using Google Translate.

**Table:** Mikolajewska 2017

***Study characteristics***	
Methods	**Study aim:** to compare the outcomes of a study of post‐stroke gait re‐education using the Bobath neuro‑developmental treatment (NDT‐Bobath) method and the traditional approach **Design:** parallel‐group RCT **Country:** Poland **Study recruitment and setting:** see Table **Involvement of patients/public/professionals/others:** no
Participants	**Study population (number randomised):** 30 **Inclusion criteria:** Aged > 18 years old Ischaemic stroke Time after ischaemic stroke from 4 weeks to 3 years Lack of contraindications to rehabilitation Able to walk **Exclusion criteria:** < 18 years old Haemorrhagic stroke or TIA Time after ischaemic stroke < 4 weeks or > 3 years Contraindications to rehabilitation Unable to walk **Participants:** detailed in Table **PROGRESS‐plus:** summarised in Table
Interventions	(1) Bobath + traditional "therapy was conducted by the same physical therapist with more than fifteen years of experience in neurorehabilitation, and NDT‐Bobath skills confirmed by basic and advanced courses in NDT‐Bobath for adults, and additionally, to broaden knowledge and experience in the NDT‐Bobath approach, by NDT‐Bobath for children and NDT‐Bobath Baby courses" (2) Traditional (no description provided) "each patient underwent the same number of therapeutic sessions (ten) through two weeks (ten days of the therapy)"
Outcomes	**Independence in ADL scales:** NR **Motor function scales:** NR **Measures of balance:** NR **Measures of gait velocity:** 10 m walk test **Length of stay:** NR **Adverse events:** NR **Other measures:** gait parameters **Time points when outcomes were assessed:** before and after treatment
Comparison	**Amount of physical rehabilitation in groups:** equivalent **Categorisation of comparison:** 3.(ii) Comparison of different approaches, (dose equivalent; with both/all groups also receiving other physical rehab)
Funding & conflicts of interest	**Funding statement:** not reported **Conflict of interest statement:** none
Synthesis	**Qualitative synthesis:** Results data not presented in a format suitable for quantitative analysis.
Notes	

**Table:** Mohaideen 2014

***Study characteristics***	
Methods	**Study aim:** aimed at justifying the effectiveness of conventional stroke training combined with symmetrical weight training among stroke patients to reduce the fall by improving the symmetrical weight bearing **Design:** parallel‐group RCT **Country:** India **Study recruitment and setting:** see Table **Involvement of patients/public/professionals/others:** no
Participants	**Study population (number randomised):** 30 **Inclusion criteria:** < 6 months of hemiparesis cases (MAS‐3 to 4) **Exclusion criteria:** Not reported **Participants:** detailed in Table **PROGRESS‐plus:** summarised in Table
Interventions	(1) Symmetrical weight training + conventional "The symmetrical weight training skills used in this study was, a) Sit‐to‐stand transitions.This was practiced in experimental group with an emphasis on symmetrical weight bearing and controlled responses of trunk. Initially the patients must actively flex the trunk and use momentum to shift the weight forward (Flexion) which can be assisted by the therapist and clasped hands of the patient himself. Then the patient’s movement is directed into extension or upward phase by recruiting knee extensors. This method is successfully used for many patients. b) Visual bio feed back by standing bio feedback trainer, which includes a height adjustable worktable, weight bearing sensors, and a real time visual and auditory feedback system. c) Static bicycling and balancing exercise with bilateral weighing machines. This training was repeated with the help of physiotherapists, Government hospital, Warangal, India 5 days a week for 3 months along with conventional stroke training." (2) Conventional stroke training "Conventional Stroke Training includes the Passive movements, passive stretching, Free exercises and Gait training"
Outcomes	**Independence in ADL scales:** NR **Motor function scales:** NR **Measures of balance:** NR **Measures of gait velocity:** NR **Length of stay:** NR **Adverse events:** NR **Other measures:** Falls **Time points when outcomes were assessed:** NR
Comparison	**Amount of physical rehabilitation in groups:** differs **Categorisation of comparison:** 5. Additional therapy + usual therapy versus usual therapy (dose differs)
Funding & conflicts of interest	**Funding statement:** not reported **Conflict of interest statement:** none
Synthesis	**Qualitative synthesis:** no outcomes of relevance to this review.
Notes	

**Table:** Moore 2016

***Study characteristics***	
Methods	**Study aim:** to explore the physiological factors affecting exercise‐induced changes in peak oxygen consumption and function post‐stroke **Design:** parallel‐group RCT **Country:** United Kingdom **Study recruitment and setting:** see Table **Involvement of patients/public/professionals/others:** no
Participants	**Study population (number randomised):** 40 **Inclusion criteria:** Aged 50 years + Stroke diagnosed (> 6 months previously) by a specialist Able to complete a 6MWT with/without stick Living at home Discharged from all conventional physiotherapy interventions Not already performing regular exercise (≥ 3x/week, moderate intensity) **Exclusion criteria:** Absolute and relative contraindications to exercise testing as stated by the American Heart Association Diabetes Neurological disorders other than stroke Pain on walking (visual analogue scale score, > 5) Unable to follow 2 stage commands Cognitive impairment (MMSE <24) Untreated major depression **Participants:** detailed in Table **PROGRESS‐plus:** summarised in Table
Interventions	(1) Exercise "The intervention was adapted from the Fitness and Mobility Exercise Program designed by Eng in 2006. The intervention was a “mixed” exercise program consisting of functional exercises designed to improve flexibility, strength, aerobic capacity, and balance. The intervention was delivered using a previously described protocol. In brief, classes were delivered in the com‐munity for 19 weeks by a fitness instructor and a physiotherapist (3 times/wk, 45‐60min). Participants wore a heart rate monitor, and the intensity of the exercise was gradually increased, working within a heart rate zone determined using the Karvonen formula (40%‐50% of participant’s maximum heart rate, with increasing increments of 10% every 4 wks up to 70%‐80%). Repetition and resistance were used to progress strength and balance exercises" "The exercise intervention lasts 19 weeks and participants will be asked to attend three times a week for one hour " (2) Control "The control group completed a matched duration home stretching program. Ten seated stretches were repeated 3 times for the upper and lower body. Participants were given an instruction booklet and diary to record activity and changes in medication/diet/physical activity and telephoned fortnightly for progress."
Outcomes	**Independence in ADL scales:** NR **Motor function scales:** NR **Measures of balance:** Berg Balance Scale **Measures of gait velocity:** 6‐minute walk test, 10 m walk test, timed up and go **Length of stay:** NR **Adverse events:** NR **Other measures:** glucose control, cerebral blood flow, cardiorespiratory fitness, body composition, stroke impairment, cognition, physical activity, quality of life **Time points when outcomes were assessed:** pre‐ and post‐treatment
Comparison	**Amount of physical rehabilitation in groups:** equivalent **Categorisation of comparison:** 2. Physical rehabilitation versus attention control (dose of physical rehab differs; dose of intervention equivalent)
Funding & conflicts of interest	**Funding statement:** Supported by the Newcastle Centre for Brain Ageing and Vitality, the UK National Institute for Health Research Biomedical Research Centre for Ageing and Age‐Related Disease award to the Newcastle upon Tyne Hospitals NHS Foundation Trust, and the Medical Research Council (reference G0802536). Also supported by a senior research fellowship, a Senior Investigator Award from the National Institute for Health Research, and the Centre for Brain Ageing and Vitality, Newcastle University **Conflict of interest statement:** none
Synthesis	**Quantitative synthesis:**Analysis 3.3; Analysis 3.4
Notes	

**Table:** Morreale 2016

***Study characteristics***	
Methods	**Study aim:** to compare PNF and CTE methods in two different time settings (early vs standard) in order to evaluate the different role of time and techniques in functional recovery after acute ischaemic stroke with hemiplegia **Design:** parallel‐group RCT **Country:** Italy **Study recruitment and setting:** see Table **Involvement of patients/public/professionals/others:** no
Participants	**Study population (number randomised):** 340 **Inclusion criteria:** All consecutive patients with first ever subcortical ischaemic stroke Contralateral hemiplegia admitted 6 to 24 hours from symptom onset **Exclusion criteria:** NIHSS < 2 Aphasia Visual disturbances, neglect and/or other spatial representation defects Disorientation and/or confusional state at onset Ongoing seizures MMSE < 26 Cardiovascular instability defined as uncontrolled hypertension, high‐frequency arrhythmias, congestive heart failure and concomitant or incident myocardial infarction Neurological instability (worsening and improving stroke) Incident haemorrhagic transformation Previous diagnosis of other neurological disease with sensorimotor and cognitive symptoms, chronic inflammatory disease with arthritis and/or neuropathies, psychiatric diseases, amputation, fractures, neoplasms and psychological/social causes Not able to give informed consent **Participants:** detailed in Table **PROGRESS‐plus:** summarised in Table
Interventions	(1) Early rehabilitation ‐ PNF "First developed by professor Hermann Kabat and physical therapist Margaret Knott, PNF is a dynamic approach to the evaluation and treatment of neuromuscular dysfunction that take into account the basic neurophysiologic and kynesiologic principles of the sensory‐motor system. By selecting motor elements and developmental components of complex motor patterns, the emphasis is placed upon selective re‐education of motor behaviour through training the fundamental skills of trunk and limbs control, stability and coordination. Technically, specific diagonal and spiral motor patterns against an appropriate resistance was sequentially used in order to facilitate and modulate stretch reflex, irradiation and other proprioceptive response toward a motor response in a proximal‐distal perspective and in different postural conditions...." (2) Early rehabilitation ‐ Cognitive therapeutic exercise (CTE) "CTE was based on Professor Carlo Perfetti’s intuition that movement is a more complex interaction modality with environment that depends on integration of several cognitive functions. Giving a straight relationship between muscular recruitment and perception, motor recovery has been as a consequence considered a learning process in pathological conditions. This method consists in a 4‐phase treatment: conversation upon motivation, relaxation through passive mobilization, guided passive mobilization and active mobilization. The third and fourth phases take place with the help of some devices such as soft and hard blocks or panels in order to ensure a correct motor performance" "Early rehabilitation programs consist of a daily bed‐side and out‐of‐bed 45‐minutes intervention with proximal joints passive/active mobilization according to Kabat’s schemes in PNF group with subsequent postural alignment and in bed and out of bed positioning (for further 15 minutes) or with guided and passive/active movements according to Perfetti's technique in the CTE group with subsequent postural alignment and position (for further 15 minutes). Thereafter the specific rehabilitation program with PNF or CTE continued daily (2.15 hours/day) in an intensive rehabilitation unit from the 5th to 60th day after discharge (14 weeks) and after in a day‐service setting (1.30 hours/day for 5 days/week) for a mean duration of 38 weeks...." (3) Standard protocol ‐ PNF (4) Standard protocol ‐ CTE "Patients in standard protocol received only postural alignment and in bed and out of bed positioning (for a total duration of 60 minutes in order toresemble the duration of early intervention treatment)during first 4 days after symptom onset and was subsequently transferred in an intensive rehabilitation unitfrom the 5th to 60th day after discharge where they were further randomized to PNF or CTE treatment regimen (2.15 hours/day for 14 weeks). Thus, they continued the same rehabilitation program in a day‐service regimen until the last follow‐up (1.30 hours/day for 5 days/week,mean duration of 38 weeks)" Comparisons of relevance to this review = (1) vs (2) and (3) vs (4)
Outcomes	**Independence in ADL scales:** Barthel Index **Motor function scales:** Motricity Index **Measures of balance:** NR **Measures of gait velocity:** 6‐minute walking test **Length of stay:** NR **Adverse events:** immobility‐related adverse events, serious adverse events **Other measures:** modified Rankin Scale, Beck Depression Inventory **Time points when outcomes were assessed:** baseline and 3 months
Comparison	**Amount of physical rehabilitation in groups:** equivalent **Categorisation of comparison:** 3.(i) Comparison of different approaches (dose equivalent; with no other physical rehab)
Funding & conflicts of interest	**Funding statement:** not reported **Conflict of interest statement:** none
Synthesis	**Qualitative synthesis only:** the interventions compared were both focussed on neurophysiological techniques, and it was not appropriate to combine the results of these groups with those of other studies comparing different physical rehabilitation approaches.
Notes	

**Table:** Mudge 2009

***Study characteristics***	
Methods	**Study aim:** "to determine whether circuit‐based rehabilitation would increase the amount and rate that individuals with stroke walk in their usual environments" **Design:** parallel‐group RCT **Country:** New Zealand **Study recruitment and setting:** see Table **Involvement of patients/public/professionals/others:** no
Participants	**Study population (number randomised):** 58 **Inclusion criteria:** 1 or more strokes more than 6 months earlier Discharged from rehabilitation Able to walk independently (with an aid if necessary) Some residual gait difficulty was required, as defined by a score of less than 2 on at least 1 of the walking items of the physical functioning scale of the SF‐36 **Exclusion criteria:** Progressive neurologic disease Other significant health problems that adversely affected walking ability > 2 falls in the previous 6 months Unstable cardiac conditions Uncontrolled hypertension Congestive heart failure **Participants:** detailed in Table **PROGRESS‐plus:** summarised in Table
Interventions	(1) Exercise training group (n = 31) "There were 15 stations in the circuit, which were graded to each participant’s ability and progressed as tolerated. Each station contained either a task‐oriented gait or standing balance activity, or strengthening of a lower extremity muscle in a way designed to improve gait. Details of the content of each station and examples of progressions are provided in an Appendix. The total exercise time was 30 minutes, although sessions lasted between 50 to 60 minutes, including stretching. Participants spent 2 minutes at each station of the circuit, with time allowed to move between stations and receive instructions for the next station. Details about exercise intensity and/or repetitions performed at each station were recorded for each participant" Length of intervention period: 4 weeks Number of sessions and length of individual sessions: in 12 group circuit exercise sessions 3 times a week Intervention provider: "groups contained up to 9 participants and were led by 1 of the investigators (S.M.) assisted by 2 physiotherapy students" (2) Attention control (n = 27) "The control group was run by an occupational therapist and consisted of 4 social and 4 educational sessions. Detailed content of the sessions is available in Appendix. The duration of the control group sessions was designed to match the duration of the intervention sessions in order to control for possible effects of dosage" "Matching for duration and not number of sessions was a pragmatic choice based on resources, allowing 1 intervention session a weekday to be scheduled over the 4‐week intervention period" Length of intervention period: 4 weeks Number of sessions and length of individual sessions: 8 x 90‐minute session weeks in groups of up to 8 Intervention provider: occupational therapist
Outcomes	**Independence in ADL scales:** NR **Motor function scales:** Rivermead Mobility Index **Measures of balance:** NR **Measures of gait velocity:** Timed 10‐Metre Walk Test **Length of stay:** NR **Adverse events:** NR **Other measures:** Six‐Minute Walk test, Physical Activity and Disability Scale (PADS), Activities‐Specific Balance and Confidence Scale, mean number of steps/d (measured by the StepWatch Activity Monitor) **Time points when outcomes were assessed:** "Two baseline testing sessions 3 weeks apart were performed to ensure that participant measures were stable. The testing sessions were repeated immediately after the group sessions (postintervention) and at 3 months (follow‐up). All tests were performed once, and all testing sessions were identical"
Comparison	**Amount of physical rehabilitation in groups:** differs **Categorisation of comparison:** 2. Physical rehabilitation versus attention control (dose of physical rehab differs; dose of intervention equivalent)
Funding & conflicts of interest	**Funding statement:** not reported **Conflict of interest statement:** not reported
Synthesis	**Quantitative synthesis:**Analysis 3.2; Analysis 3.4; Analysis 4.2; Analysis 4.4
Notes	Data provided are means and ranges. For analysis, standard deviations have been estimated by calculating (upper range ‐ lower range)/4

**Table:** Mustafaoğlu 2018

***Study characteristics***	
Methods	**Study aim:** to compare effectiveness of isolated body weight‐supported treadmill training with conventional and combined training on balance, mobility, and fear of falling in stroke patients **Design:** multi‐arm RCT (3‐arm) **Country:** Turkey **Study recruitment and setting:** see Table **Involvement of patients/public/professionals/others:** no
Participants	**Study population (number randomised):** 45 **Inclusion criteria:** Stroke onset ≥ 3 months Age range of 18 to 75 years Able to walk 10 m independently or under supervision, Able to understand all instructions during treatment sessions (MMSE ≥ 24) **Exclusion criteria:** Previous stroke Musculoskeletal disorders causing contracture or limited range of motion in their lower extremities affecting walking Severe heart disease or medically uncontrolled hypertension **Participants:** detailed in Table **PROGRESS‐plus:** summarised in Table
Interventions	(1) Conventional training (CT) "a rehabilitation program was planned according to each participant’s functional level and requirements. The CT program focused on trunk stabilization, weight transfer to the paretic leg, and walking between parallel bars or on theground. Treatment activities were designed to improve balance, while encouraging the participant to use his or her more paretic lower limb. Verbal and tactile cues were used to encourage symmetrical weight distribution. The training was also complicated by adding arm activities and reaching activities while walking forward, backward and side to side. The CTG received a session lasted 45 min according to the government’s health insurance rules for five week daysover six weeks." (2) Body weight‐supported treadmill training (BWSTT) "The Lokomat system (Hocoma AG, Volketswil,Switzerland) with an integrated treadmill and a motor‐driven body weight support system with real‐time feedback control for precise body weight unloading was used for the BWSTT. Each session included set‐up, commands, and resting time. Verbal instructions were used for encouragement, but no manual assistance was provided to improve gait pattern. All parameters were individually adjusted for each session. The actual training time was 45 min per session, excluding time required for putting on equipment and operation of the computer. The BWSTTG received 45 min, two non‐consecutive days per week for six‐week gait training on the Lokomat." (3) BWSTT + CT "Combined training patients received inpatient rehabilitation. The CombTG received BWSTT(Lokomat; 45 min, two non‐consecutive days per week for six weeks) combined with CT (45 min, five days perweek for six weeks)." Comparisons of relevance to this review = (2) vs (3)
Outcomes	**Independence in ADL scales: NR** **Motor function scales:** Rivermead Mobility Index **Measures of balance:** Berg Balance Scale **Measures of gait velocity:** timed up and go test, 10‐m walk test **Length of stay:** NR **Adverse events:** states "no adverse events" **Other measures:** single leg stance test, Falls Efficacy Scale‐International, stair climbing test **Time points when outcomes were assessed:** pre‐ and post‐intervention
Comparison	**Amount of physical rehabilitation in groups:** differs **Categorisation of comparison:** 5. Additional therapy + usual therapy versus usual therapy (dose differs) (Note ‐ in this case the "usual therapy" is the BWSTT, and the additional therapy is the "conventional therapy").
Funding & conflicts of interest	**Funding statement:** no financial support for the research and/or authorship of this article **Conflict of interest statement:** none
Synthesis	**Quantitative synthesis:**Analysis 5.2; Analysis 5.3; Analysis 5.4
Notes	

**Table:** Nagy 2017

***Study characteristics***	
Methods	**Study aim:** to assess the benefits of the conductive education approach added to conventional physiotherapy in gait functions of post‐stroke, hemiparetic patients **Design:** parallel‐group RCT **Country:** Hungary **Study recruitment and setting:** see Table **Involvement of patients/public/professionals/others:** no
Participants	**Study population (number randomised):** 20 **Inclusion criteria:** 18 to 75 years Late (3 to 6 months after stroke) or chronic (> 6 months) hemiparetic post‐stroke state with gait disturbances Adequate endurance Able to walk with/without walking aid and able to walk on a treadmill **Exclusion criteria:** Presence of a neurologic disorder, which can influence gait Ataxia because of a non‐neurologic cause Severe pain (> 5 on a 10‐point VAS) Cardio‐respiratorially unstable Uncontrolled epilepsy Severe dementia (MMSE < 19 points) Obesity (BMI > 40) **Participants:** detailed in Table **PROGRESS‐plus:** summarised in Table
Interventions	(1) Conductive Education (CE) + conventional physical therapy (CPT) "The CE+CPT group, in addition to the three CPT sessions (60 min), received 45 min of individual and 30 min of group training by CE methods." (2) Conventional physical therapy (CPT) "The CPT group performed three sessions per day (each 20 min long) of gait training therapy (muscle strengthening, balance training, spasticity reduction, and teaching the proper gait function in individual and group sessions)." "The trainings were performed on 18 consecutive week‐days"
Outcomes	**Independence in ADL scales:** Functional Independence Measure (motor subscale) **Motor function scales:** NR **Measures of balance:** NR **Measures of gait velocity:** gait velocity, 3‐min walk test, timed up and go **Length of stay:** NR **Adverse events:** NR **Other measures:** Fugl‐Meyer balance standing, gait parameters, muscle strength, muscle tone, range of movement **Time points when outcomes were assessed:** before and after treatment
Comparison	**Amount of physical rehabilitation in groups:** differs **Categorisation of comparison:** 5. Additional therapy + usual therapy versus usual therapy (dose differs)
Funding & conflicts of interest	**Funding statement:** not reported **Conflict of interest statement:** none
Synthesis	**Qualitative synthesis:** results data not presented in a format suitable for quantitative analysis
Notes	Data presented as change from baseline, medians, and interquartile ranges

**Table:** Ni 1997

***Study characteristics***	
Methods	**Study aim:** "to explore the influence of early rehabilitation on walking function of the patients with stroke, 6 months after their stroke" **Design:** parallel‐group RCT **Country:** China **Study recruitment and setting:** see Table **Involvement of patients/public/professionals/others:** no
Participants	**Study population (number randomised):** 68 **Inclusion criteria:** Not reported **Exclusion criteria:** Not reported **Participants:** detailed in Table **PROGRESS‐plus:** summarised in Table
Interventions	(1) Rehabilitation (n = 34) "Participants from both groups received conventional neurological treatment. The comprehensive rehabilitation training group additionally received Bobath and Brunnstrom focused exercise therapy, emphasising on standing balance and lower limb exercise control ability training. For participants weak in dorsiflexion, the thumb or other finger could be used to apply pressure to the dorsal area between the first and second metatarsal, so as to stimulate dorsiflexion, this method could be repeatedly used; during ambulation, the affected limb was supported by an elastic bandage into 90 degrees of ankle dorsiflexion. Training of the muscles at the shin area used the FZ‐1 model to deliver low frequency electrical stimulation pulse, with intensity selected as appropriate, 1/day, 20min/session, 20‐40x of treatment on average, with those requiring more than 20x of treatment having a 10 day rest before commencement of the next session" Length of intervention period: "2 months of treatment on average" Number of sessions and length of individual sessions: 2/d, 30 to 45 minutes/session Intervention provider: not stated (2) No physical rehabilitation group (n = 34) Conventional neurological treatment Length of intervention period: no intervention Number of sessions and length of individual sessions: no intervention Intervention provider: no intervention
Outcomes	**Independence in ADL scales:** Functional Independence Measure (FIM) (measured, but no data provided) **Motor function scales:** Fugl‐Meyer Assessment **Measures of balance:** NR **Measures of gait velocity:** NR **Length of stay:** NR **Adverse events:** NR **Other measures:** Brunnstrom **Time points when outcomes were assessed:** "1 week after stroke onset or before commencement of intervention and 3 months after stroke"
Comparison	**Amount of physical rehabilitation in groups:** differs **Categorisation of comparison:** 1. Physical rehabilitation versus no or limited physical rehabilitation (dose differs) (limited = < 50% of dose)
Funding & conflicts of interest	**Funding statement:** not reported **Conflict of interest statement:** not reported
Synthesis	**Quantitative synthesis:**Analysis 1.2
Notes	Original study translated from Chinese to English by member of our team (PLC).

**Table:** Nindorera 2022

***Study characteristics***	
Methods	**Study aim:** "to investigate the feasibility and efficacy of a new intervention: Circuit walking, balance, cycling and strength training (CBCS) on activity of daily living (ADL) limitations, motor performance, and social participation restrictions in people after stroke" **Design:** cross‐over RCT **Country:** Burundi **Study recruitment and setting:** see Table **Involvement of patients/public/professionals/others:** no
Participants	**46 Study population (number randomised):** 46 **Inclusion criteria:** Clinical diagnosis of primary or recurring unilateral stroke based on neuroimaging or a clinical neurological examination Chronic stroke stage (≥ 6 months since stroke) ≥ 18 years of age Able to walk 10 m (with/without an assistive device) Moderate disability (modified Rankin scale ≤ 3) **Exclusion criteria:** Major cognitive impairment (Community Screening Interview for Dementia score ≥ 7) Aphasia Permanent degenerative damage **Participants:** detailed in Table **PROGRESS‐plus:** summarised in Table
Interventions	(1) Physical rehabilitation "The experimental group participants were split into 3 subgroups of 6‐8 participants with similar within‐subgroup disability levels for CBCS training. All participants performed a 15‐min warm‐up before training and a 15‐min cool down with stretching after training. The training consisted of 30 min of circuit walking followed by 30 min of recreational activities intended to improve balance, coordination, and strength, as well as 30 min of cycling at 50–75% of their maximal heart rate. Disability level subgroups were trained concurrently, with each group randomly assigned to a CBCS component, such that while one group was walking, another was cycling, and the third was performing balance, strength, and coordination exercises. Shaping was adapted to each individual. To help increase the duration of targeted motor involvement, encouragement was given through verbal feedback, distance covered was tracked, and level of exertion was followed with heart rate monitors during walking and cycling. Activity intensity was increased as sessions progressed to promote energy expenditure." (2) Attention control "... participants participated in a sociocultural intervention aimed to improve social interaction through 3 activities that did not involve energy expenditure: board games (Awalé and Checkers), cultural discourse and drama. The board game component was performed in randomly allocated subgroups of 6 to 7 participants. Awalé (a.k.a. awele) is an informal combinatorial board game created in Africa. It is the most widespread of the mancala family of games, a set of African count and capture games in which pebbles, seeds, or shells are distributed in bowls or holes, sometimes dug in the ground. Checkers is an informal combinatorial board game wherein each player captures the opponent's pieces. For each game, participants solely used their non‐affected hand and progressed through learning, forming playoff groups, and competing phases. After the board game period of each session, participants reformed groups of 4∼6 people for the discourse and drama components. Topics were offered for cultural discourse and drama (topic pools were updated every 2 weeks)". "The interventions were performed 2 hours/day, 3 times a week for 12 weeks (36 sessions: 72 hours)."
Outcomes	**Independence in ADL scales:** ACTIVLIM‐Stroke scale **Motor function scales:** NR **Measures of balance:** Berg Balance Scale **Measures of gait velocity:** 10‐m walk test; 6‐minute walk test **Length of stay:** NR **Adverse events:** NR **Other measures:** Stroke Impairment Assessment Set, Hospital Anxiety & Depression scale, Participation Measurement scale **Time points when outcomes were assessed:** "Participants were assessed at four timepoints, T1–T4: T1, baseline (at enrolment); T2, at 3 mos. (immediately post‐intervention); T3, at 6 mos. (after crossover); and T4, at 9 mos. (3‐mo. follow‐up)." As this was a cross‐over design, only the immediate time point (T2) was relevant to our analyses.
Comparison	**Amount of physical rehabilitation in groups:** differs **Categorisation of comparison:** 2. Physical rehabilitation versus attention control (dose of physical rehab differs; dose of intervention equivalent)
Funding & conflicts of interest	**Funding statement:** "Authors thank the Association pour la Promotion del Éducation et de la Formationa l’Étranger (APEFE), Wallonie‐Bruxelles International (WBI) and Universite Catholique de Louvainfor their scholarships to Nduwimana I, and Nindorera F. We also acknowledge manuscript editing by Write Science Right." **Conflict of interest statement:** "declare that they have no known competing financial interests or personal relationships that could have appeared to influence the work reported in this paper"
Synthesis	**Quantitative synthesis:**Analysis 3.1; Analysis 3.3; Analysis 3.4
Notes	

**Table:** Outermans 2010

***Study characteristics***	
Methods	**Study aim:** to investigate the feasibility and the effects on gait of a high intensity task‐oriented training, incorporating a high cardiovascular workload and large number of repetitions, in patients with subacute stroke, when compared to a low‐intensity physiotherapy‐programme **Design:** parallel‐group RCT **Country:** Netherlands **Study recruitment and setting:** see Table **Involvement of patients/public/professionals/others:** no
Participants	**Study population (number randomised):** 44 **Inclusion criteria:** Diagnosis of hemiplegia following first or recurrent stroke 2 to 8 weeks since most recent stroke and recruitment Able to walk 10 m without assistance FAC ≥ 3 **Exclusion criteria:** Cardiovascular instability Acute impairment of lower extremities influencing walking Sensory communicative disorders **Participants:** detailed in Table **PROGRESS‐plus:** summarised in Table
Interventions	(1) High‐intensity task‐oriented training "The high‐intensity task‐oriented training‐programme incorporated 10 standardized work‐stations, focused on improving walking competency, similar to the study by Deanet al. (19). Participants in the high‐intensity training‐group performed 45 minutes of circuit class training, held at the rehabilitation clinic three times a week for four weeks. All stations were practised for 2.5 minutes, followed by a 1‐minute transfer to the next station. Afterwards the participants joined in walking relays and races for 10 minutes.The high‐intensity training‐programme focused on improving postural control and gait‐related activities such as stair walking, turning, making transfers, walking quickly and walking for specified distances. In line with the recommendations of the American Heart Association, (22) cardiorespiratory workload started at 40–50% of heart rate reserve. Progression was attained by increasing the worknload to a maximum of 70–80% of heart rate reserve, (25), and increasing the number of repetitions, both according to the observations and estimation of the therapists in charge and the patient's perceived exertion. A 6–20 Borg Scale was used to rate subjects’ perceived exertion" (2) Low‐intensity physiotherapy "The focus in the low‐intensity physiotherapy‐group was on improving motor control of the hemiparetic leg and balance. In contrast to the high‐intensity training group there were no components of physical fitness training such as strengthen‐ing exercises or cardiorespiratory training, indicating that the training was set at a low‐intensity profile aimed at learning gait‐related activities. The participants in the low‐intensity physiotherapy group went through a 45‐minute programme of group exercises, three times a week for four weeks, thus matching therapy time to the high‐intensity training‐group. The low‐intensity physiotherapy‐programme was also based on a 10 work stations circuit. All stations were practised for 2.5 minutes, followed by a 1‐minute gap to transfer to the next station. Afterwards the participants joined in games, like passing through a ball, for 10 minutes. Progression, according to the observations and estimation of the therapists in charge, was achieved by enhancing motor control challenge, not in enhancing the number of repetitions like the high‐intensity training‐group." "All participants engaged in usual individual physiotherapy for half an hour each day. Information about intensity and content of the therapy beyond the trial were documented in a patient’s record. Therapists were instructed not to depart from their usual care during the trial."
Outcomes	**Independence in ADL scales:** NR **Motor function scales:** NR **Measures of balance:** Berg Balance Scale, functional reach test **Measures of gait velocity:** 6‐minute walking test, 10‐m walk test **Length of stay:** NR **Adverse events:** NR **Other measures:** NR **Time points when outcomes were assessed:** baseline and after intervention (or earlier if there was early discharge)
Comparison	**Amount of physical rehabilitation in groups:** equivalent **Categorisation of comparison:** 3.(ii) Comparison of different approaches, (dose equivalent; with both/all groups also receiving other physical rehab)
Funding & conflicts of interest	**Funding statement:** not reported **Conflict of interest statement:** none
Synthesis	**Quantitative synthesis:**Analysis 7.3; Analysis 7.4
Notes	

**Table:** Pan 2004

***Study characteristics***	
Methods	**Study aim:** "to study the role of early rehabilitation in motor function of upper and lower extremities and ADL for patients with hemiplegia after stroke" **Design:** parallel‐group RCT **Country:** China **Study recruitment and setting:** see Table **Involvement of patients/public/professionals/others:** no
Participants	**Study population (number randomised):** 96 **Inclusion criteria:** Not reported **Exclusion criteria:** Not reported **Participants:** detailed in Table **PROGRESS‐plus:** summarised in Table
Interventions	(1) Rehabilitation (n = 48) "Both groups received usual medical care and acupuncture. Participants in the treatment group commenced therapy once medically stable, where participants with ischemic stroke commenced therapy within 5 days of hospitalisation and participants with haemorrhagic stroke commencing therapy between 7 to 14 days of hospitalisation" "Treatment intervention: (i) Regular change in bed position: for participants not able to turn in bed independently, turning was done every two hours in the supine position, lateral position with affected limbs at lower side and healthy limbs at upper side, and lateral position with affected limbs at upper side and healthy limbs at lower side. (ii) Optimal placement of limbs in functional positions. (iii) Joint movement: Daily movement of each joint 2‐3 times/day, 5‐10 times/session. Upon gaining consciousness, participants were encouraged to do active assisted exercises of shoulder flexion with intertwined hands and lower limb bridging exercises with attention to pelvic control, until participants were able to do the exercises actively. (iv) Lying to sitting and sitting balance training: participants first shifted to edge of bed, dangled both legs off the bed with the unaffected leg supported the top affected leg, and pushed into sitting position with the unaffected arm. For sitting balance training, participants practised reaching for/placing objects from one side to another, increasing the amount of reach as much as possible. Participants were also trained to self‐correct their sitting balance by subjecting them to perturbations in all directions. (v) Sit to stand and standing balance training: participants adopted the Bobath method of intertwining both arms, forward extension of arms, head and trunk, shifted body weight to the arms in order to lift the pelvis, hips, knee and stand up. If needed, therapist assisted by pushing the affected knee forward and putting one hand on the unaffected pelvis to help lift it. Participants stood in parallel bars, supporting with the unaffected arm and with assistance from the doctor or family, and gradually increased the time in standing until 30 mins could be attained. Participants progressed to sit to stand practice from using a high chair to a low chair for added challenge. (vi) Gait re‐education and stair training (up and down). (vii) ADL practice: inculcated ADL tasks practice into therapy sessions, 3‐4x/day, 30 min/session. Family of participants were instructed to assist in practice" Length of intervention period: not stated Number of sessions and length of individual sessions: dependent on participant's condition (see above) Intervention provider: therapist and "family of participants were instructed to assist in practice" (2) No physical rehabilitation group (n = 48) "Received usual medical care and acupuncture" Length of intervention period: no intervention Number of sessions and length of individual sessions: no intervention Intervention provider: no intervention
Outcomes	**Independence in ADL scales:** Barthel Index **Motor function scales:** Fugl‐Meyer Assessment (upper and lower limbs) **Measures of balance:** NR **Measures of gait velocity:** NR **Length of stay:** NR **Adverse events:** NR **Other measures:** neurological deficit (CNS) **Time points when outcomes were assessed:** "24 hrs before commencement of intervention and 3‐4 weeks after intervention"
Comparison	**Amount of physical rehabilitation in groups:** differs **Categorisation of comparison:** 1. Physical rehabilitation versus no or limited physical rehabilitation (dose differs) (limited = < 50% of dose)
Funding & conflicts of interest	**Funding statement:** not reported **Conflict of interest statement:** not reported
Synthesis	**Quantitative synthesis:**Analysis 1.1; Analysis 1.2
Notes	Original study translated from Chinese to English by member of our team (PLC).

**Table:** Pandian 2014

***Study characteristics***	
Methods	**Study aim:** to examine the effect of a motor therapy programme primarily involving the non‐paretic side on balance and function in chronic stroke **Design:** parallel‐group RCT **Country:** India **Study recruitment and setting:** see Table **Involvement of patients/public/professionals/others:** no
Participants	**Study population (number randomised):** 39 **Inclusion criteria:** Ischaemic or haemorrhagic stroke < 60 years of age > 24 weeks post‐stroke Either right or left side hemiparesis Brunnstrom recovery stage 2 and above and FAC level III (i.e. independent walking with verbal supervision) **Exclusion criteria:** Patients with acute medical illness Neuromusculoskeletal complications on non‐paretic side Uncontrolled hypertension Severe cognitive and perceptual deficits **Participants:** detailed in Table **PROGRESS‐pluss:** summarised in Table
Interventions	(1) Motor training of both sides "the experimental protocol comprised motor training of both the sides. The program had two components, resistive exercise for the nonparetic side and bimanual activities, 30 minutes each. The resistive exercises were based on the progressive resistive exercise principle of Delorme and Watkins [25]. The strengthening exercises were performed for the weak muscles (grade 4 or less) of the nonparetic upper and lower limbs. The strength for all the muscle groups (shoulder, elbow, forearm, wrist, hip, knee, and ankle) of nonparetic upper and lower limbs was assessed by manual testing method [26,27]. In addition to the resistive exercises, various bimanual activities such as arm cycling, rowing, and postural transition were also provided." "The protocol was carried out for 8 weeks with a frequency of 3/week. The control group subjects underwent similar duration of therapy" (2) Standard motor rehabilitation "The control group subjects underwent standard motor rehabilitation (based on Brunnstrom’s movement therapy) incorporating the affected side only [23]. The protocol comprised reflexive, synergistic, and out‐of‐synergy movements of the paretic upper limb and lower limb. The main focus of the intervention was to induce voluntary motor control. Various functional activities such as sit‐to‐stand, stepping, reaching, and side‐ward walking and straight‐line walking were also provided." "The control group subjects were provided dose‐matched (in terms of number and duration of sessions) conventional program based on neurophysiological approach"
Outcomes	**Independence in ADL scales:** Barthel Index **Motor function scales:** Fugl‐Meyer Assessment **Measures of balance:** Berg Balance Scale, functional reach test **Measures of gait velocity:** NR **Length of stay:** NR **Adverse events:** NR **Other measures:** Brunnstrom Recovery Stage, Minnesota manual dexterity test, Purdue peg board test, Manual Muscle Testing **Time points when outcomes were assessed:** before and after intervention
Comparison	**Amount of physical rehabilitation in groups:** equivalent **Categorisation of comparison:** 3.(i) Comparison of different approaches (dose equivalent; with no other physical rehab)
Funding & conflicts of interest	**Funding statement:** financially supported by Pandit Deendayal Upadhyaya Institute for the Physically Handicapped **Conflict of interest statement:** none
Synthesis	**Quantitative synthesis:**Analysis 7.1; Analysis 7.2; Analysis 7.3
Notes	There are 2 publications, both of which report slightly different participant numbers. However, as other aspects of these publications are identical, we have assumed that these are two reports of the same study.

**Table:** Pang 2003

***Study characteristics***	
Methods	**Study aim:** to evaluate the effectiveness of early rehabilitation in acute stroke **Design:** parallel‐group RCT **Country:** China **Study recruitment and setting:** see Table **Involvement of patients/public/professionals/others:** no
Participants	**Study population (number randomised):** 86 **Inclusion criteria:** "diagnosed according to diagnostic criteria established in the Fourth National Cerebral Vascular Disease Conference in 1995" **Exclusion criteria:** Not reported **Participants:** detailed in Table **PROGRESS‐plus:** summarised in Table
Interventions	(1) Rehabilitation (n = 50) "Both groups received routine treatment in department of internal neurology. Cerebral edema [was] controlled by 20% mannitol and support treatment. In rehabilitation group, superearly stage, flaccid paralysis and recovery stage rehabilitation care were performed based on routine internal medicine treatment" "(i) Super early stage care: Carefully observe and keep stable life signs and improve basical care within the first 3 days. Keep functional position, including flexion and abduct of shoulder joint, extension of the elbow, dorsal extension of wrist, extension or light flexion of fingers, flexion of knee and hip joint and keep ankle joint in medium position. Turn the body over in regular time and change position, healthy side lateral recumbent position and injured side lateral recumbent position. Still, the patients were required to take injured side lateral recumbent position as much as possible to simulate feeling of the injury side and is advantageble to motion of the healthy side of the body" "(ii) Flaccid paralysis stage care: After 3 weeks’ basic treatment, proximal to distal massaging can be taken based on stable life signs and maintaining functional position. After that, proper short time passive flexion and extension were practiced within the limit of the various articular motion. Motion sequence is started from the bigger joint to the smaller one and stick to from little to large range, proper and step by step principle. Violence was avoided to prevent soft tissue injured. Motion is according to the fatigue status of the patients, with 2 or 3 times a day and 30 min each time" "(iii) Early rehabilitation care: It is important to perform ADL exercises 3 to 4 weeks after injury. The patients were required to practice initiative and passive motion, including position transversion, balance and functional exercise of upper extremities and hand, overload exercise of the lower extremities and flexion and extension of the hip, knee and ankle joint and language exercise, with 1 time per day and 30 to 60 min each time" Length of intervention period: 4 weeks Number of sessions and length of individual sessions: dependent on phase of recovery Intervention provider: not stated (2) No physical rehabilitation group (n = 36) "Patients in the control group were taken routine care during the experiment" The individual components delivered are listed in Table 28. Based on the individual components, this intervention is categorised as comprising no intervention. Length of intervention period: no intervention Number of sessions and length of individual sessions: no intervention Intervention provider: no intervention
Outcomes	**Independence in ADL scales**: Barthel Index **Motor function scales:** NR **Measures of balance:** NR **Measures of gait velocity:** NR **Length of stay:** NR **Adverse events:** NR Time points when outcomes were assessed: "BI comparison between two groups when were received" and "BI comparison between two group[s] 4 weeks after reception"
Comparison	**Amount of physical rehabilitation in groups:** differs **Categorisation of comparison:** 1. Physical rehabilitation versus no or limited physical rehabilitation (dose differs) (limited = < 50% of dose)
Funding & conflicts of interest	**Funding statement:** not reported **Conflict of interest statement:** not reported
Synthesis	**Qualitative synthesis:** no data suitable for analysis presented in the abstract.
Notes	Abstract only This paper was written in English, and the extracts above are direct quotes.

**Table:** Pang 2005

***Study characteristics***	
Methods	**Study aim:** to examine the effects of a community‐based group exercise programme for older individuals with chronic stroke **Design:** parallel‐group RCT **Country:** Canada **Study recruitment and setting:** see Table **Involvement of patients/public/professionals/others:** no
Participants	**Study population (number randomised):** 63 **Inclusion criteria:** Single stroke > 1 year onset Age ≥ 50 years Able to walk > 10 m independently with/without walking aids Living at home **Exclusion criteria:** History of serious cardiac disease (i.e. myocardial infarction) Uncontrolled blood pressure (systolic blood pressure > 140, diastolic blood pressure > 90) Pain while walking Neurological conditions in addition to stroke Other serious diseases that preclude the individual from participating in the study **Participants:** detailed in Table **PROGRESS‐plus:** summarised in Table
Interventions	(1) Fitness and Mobility Exercise (FAME) programme Station 1 ‐ cardiorespiratory fitness and mobility; station 2 ‐ mobility and balance; station 3 ‐ leg muscle strength (2) Control ‐ upper limb programme Station 1 ‐ shoulder muscle strength; station 2 ‐ elbow/wrist muscle strength and range of movement; station 3 ‐ hand activities. "Both the intervention and control groups underwent an exercise program for 19 weeks (1‐hour sessions, 3 sessions per week) in the same multi‐purpose room of a community hall."
Outcomes	**Independence in ADL scales:** NR **Motor function scales:** NR **Measures of balance:** Berg Balance Scale **Measures of gait velocity:** NR **Length of stay:** NR **Adverse events:** "adverse events (e.g. falls) were monitored and recorded" **Other measures:** cardiorespiratory fitness, 6‐minute walk test, leg muscle strength, Physical Activity Scale for Individuals with Physical Disabilities, femoral neck bone mineral density **Time points when outcomes were assessed:** NR
Comparison	**Amount of physical rehabilitation in groups:** equivalent **Categorisation of comparison:** 2. Physical rehabilitation versus attention control (dose of physical rehab differs; dose of intervention equivalent)
Funding & conflicts of interest	**Funding statement:** "M.Y.C.P. was supported by a post‐doctoral fellowship from Natural Sciences and Engineering Research Council of Canada. This study was supported by a grant‐in‐aid from the Heart Stroke Foundation of British Columbia and Yukon (J.J.E.) and from career scientist awards from Canadian Institute of Health Research (J.J.E) (MSH‐63617) and the Michael Smith Foundation for Health Research (J.J.E. and H.A.Mc.)" **Conflict of interest statement:** none
Synthesis	**Quantitative synthesis:**Analysis 3.3; Analysis 3.6
Notes	

**Table:** Pang 2006

***Study characteristics***	
Methods	**Study aim:** "to study the effects of cocktail treatment on motor function of patients with ischaemic stroke" **Design:** parallel‐group RCT **Country:** China **Study recruitment and setting:** see Table **Involvement of patients/public/professionals/others:** no
Participants	**Study population (number randomised):** 80 **Inclusion criteria:** Met the 1995 Fourth National Conference on Cerebral Vascular Disease classification guidelines Ischaemic stroke as confirmed by CT or MRI scan Informed consent from participants **Exclusion criteria:** Impaired consciousness Unable to comply with assessment and/or treatment Sensory aphasia European Stroke Scale score > 80 History of stroke, severe psychological and/or cognitive issues Severe impairment in cardiac liver and kidney function No consent from participant or family members **Participants:** detailed in Table **PROGRESS‐plus:** summarised in Table
Interventions	(1) Rehabilitation(n = 41) "The treatment group received cocktail treatment in addition to conventional therapy. Cocktail treatment comprised notoginseng saponin, rehabilitation training, electroacupuncture and hyperbaric oxygen treatment. Notoginseng saponin treatment: Injections given since allocation to treatment group, saline (or 50g/L glucose) 200mL+0.4g, for 14 consecutive days. Hyperbaric oxygen treatment: using 202 kPa (gradual pressure increment time 20 min, suction 202 kPa oxygen 20 min, thereafter gradual pressure decrement time 20 min), treatment time: 1/day, 60min/session, 5/week, for a total of 10x" "Bobath treatment method: using Bobath method of therapy for active, passive rehabilitation training, 1/day, 30 min/session, 5/week, for a total of 10x'" "Electroacupuncture treatment: stimulated selected acupuncture points hegu, quchi, zusanli, sanyinjiao, stimulation frequency 0.5Hz, intermittent, 2‐wave amplitude, amplitude 10V, pulse width 400µs, intensity 20mA. Treatment time arranged as: 1/day, 30min/session, 5/week, for a total of 10x" Length of intervention period: see above Number of sessions and length of individual sessions: see above Intervention provider: not stated (2) No physical rehabilitation group (n = 37) "Received conventional therapy (expectant therapy, anti‐platelet aggregation, decompression by dehydration, neurotrophy, prevention and cure of complications etc)" Length of intervention period: no intervention Number of sessions and length of individual sessions: no intervention Intervention provider: no intervention
Outcomes	**Independence in ADL scales:** Barthel Index **Motor function scales:** NR **Measures of balance:** NR **Measures of gait velocity:** NR **Length of stay:** NR **Adverse events:** NR **Other measures:** European Stroke Scale (ESS) **Time points when outcomes were assessed:** "before and after intervention"
Comparison	**Amount of physical rehabilitation in groups:** differs **Categorisation of comparison:** 1. Physical rehabilitation versus no or limited physical rehabilitation (dose differs) (limited = < 50% of dose)
Funding & conflicts of interest	**Funding statement:** not reported **Conflict of interest statement:** not reported
Synthesis	**Quantitative synthesis:**Analysis 1.1
Notes	Original study translated from Chinese to English Note: treatment group received both notoginseng saponin treatment and hyperbaric oxygen treatment, as well as the physiotherapy components

**Table:** Pang 2018

***Study characteristics***	
Methods	**Study aim:** to examine the effects of dual‐task exercise in chronic stroke patients **Design:** multi‐arm RCT (3‐arm) **Country:** Hong Kong SAR, China **Study recruitment and setting:** see Table **Involvement of patients/public/professionals/others:** no
Participants	**Study population (number randomised):** 84 **Inclusion criteria:** Diagnosis of stroke ≥ 6 months after stroke onset ≥ 50 years of age Community dwelling (defined as living in one’s own home or the home of a relative, friend, or caregiver) Medically stable Having balance deficits (a Mini‐Balance Evaluation System Test score ≤ 25) Able to follow 3‐step commands Able to walk at least 10 m without manual assistance **Exclusion criteria:** Having neurological conditions other than stroke Not community‐dwelling before the stroke event Significant receptive or expressive aphasia Substantial cognitive impairment (MoCA < 21) Other serious illnesses that precluded participation in the study **Participants:** detailed in Table **PROGRESS‐plus:** summarised in Table
Interventions	(1) Dual task training "the dual‐task group underwent 30 minutes of cognitive‐motor dual‐task exercise training and 30 minutes of flexibility exercises (i.e., stretching of the major upper and lower extremity muscle groups). For the dual‐task training component, the balance and mobility exercises were designed based on the task‐oriented approach using the principles from Gentile’s taxonomy of tasks as well as Carr and Shepherd’s Motor Relearning Program. The difficulty level of the balance/mobility tasks progressed in a variety of ways, such as: decreasing the base of support, increasing the demand for movement speed, and standing/walking on compliant surfaces. The cognitive tasks used were adapted from previous research and involved a wide range of activities, such as: naming objects, carrying out a conversation, and remembering numbers..." (2) Single task training "The single‐task balance and mobility training group engaged in the same balance and mobility activities as the dual‐task group, but without concurrent cognitive task for 30 minutes. These participants also practiced the same cognitive exercises as in the dual‐task group for 30minutes in a sitting position. Therefore, the amount of time spent in mobility/balance and cognitive activities was similar between the dual‐and single‐task groups." (3) Attention control ‐ upper limb exercise "Participants in this group only engaged in flexibility exercises of all limbs and strengthening exercises of the upper limbs without additional cognitive tasks. The exercises were performed mainly in a sitting or lying position to control for possible training effects on balance and gait. Over the course of training, the level of difficulty progressed by increasing the resistance of the theraband, depending on individual performance" All groups: "three 60‐minute sessions per week) for 8 weeks" Comparison of relevance to this review = (2) vs (3) (dual task training is covered by another Cochrane review)
Outcomes	**Independence in ADL scales:** Frenchay Activities Index **Motor function scales:** NR **Measures of balance:** NR **Measures of gait velocity:** forward walking test, timed up and go **Length of stay:** NR **Adverse events:** Falls **Other measures:** obstacle crossing test, cognitive tasks, Activities‐specific Balance Confidence (ABC) scale, Stroke‐Specific Quality of Life Scale **Time points when outcomes were assessed:** before intervention, after intervention, 8 weeks after intervention
Comparison	**Amount of physical rehabilitation in groups:** equivalent **Categorisation of comparison:** 2. Physical rehabilitation versus attention control (dose of physical rehab differs; dose of intervention equivalent)
Funding & conflicts of interest	**Funding statement:** Dr Yang was supported by a PhD studentship provided by the Institute for Disaster Management and Reconstruction, Sichuan University–Hong Kong Polytechnic University. Dr Pang and H. Ouyang were supported by a research grant provided by the Research Grants Council (General Research Fund; 151594/16M). H. Ouyang had an employment position in the Guangdong Provincial Work Injury Rehabilitation Hospital. M. Huang and Dr Lam were supported by a PhD studentship provided by the Hong Kong Polytechnic University. Dr Jehu was supported by the Research Student Attachment Program from the Hong Kong Polytechnic University, the Queen Elizabeth II Scholarship in Science and Technology, and an Excellence Scholarship **Conflict of interest statement:** none
Synthesis	**Qualitative synthesis:** data not presented in a format suitable for inclusion in analyses
Notes	

**Table:** Park 2021

***Study characteristics***	
Methods	**Study aim:** to investigate the effects of cross‐training on gait and balance in hemiplegic patients when applied to the affected and unaffected lower extremities **Design:** multi‐arm RCT (3‐arm) **Country:** Korea, Rep. **Study recruitment and setting:** see Table **Involvement of patients/public/professionals/others:** no
Participants	**Study population (number randomised):** 60 **Inclusion criteria:** Diagnosed with stroke by a physician, had hemiplegic symptoms Informed consent **Exclusion criteria:** Experienced their lesion within the last 3 months Visual or vestibular injury Orthopaedic surgery or had an orthopaedic condition FAC < 4 points Unable to walk ≥ 10 m even when using an assistive device MAS > grade 2 ≥ 30 s in a Timed Up and Go Test **Participants:** detailed in Table **PROGRESS‐plus:** summarised in Table
Interventions	(1) Direct cross training group (DCG) "received cross‐training to the lower extremity on the affected side," (2) Indirect cross training group (ICG) "received cross‐training to the lower extremity on the unaffected side," (3) Conventional "did not receive cross‐training" "cross training, which may also be referred to as over‐flow, contralateral effect, irradiation, and cross‐education, is commonly used to intervene with compound movements such as gait. This method started gaining attention after several studies reported that training the non‐lesion side could affect function on the lesion side. Cross‐training refers to the possibility of promoting muscle activity on the paralyzed side by applying resistance exercise to stronger parts of the body..." "all groups underwent general neurological physiotherapy for 30 mins, twice daily, 5 days/week for 4 weeks. the two intervention groups underwent 30 mins of cross‐training instead of general neurological physiotherapy once daily, 3 days/week for 4 weeks. Cross‐training was targeted to the lower extremity on the affected and unaffected side in the DCG and ICG, respectively. The basic neurological physical therapy consisted of joint movement exercise, strength training, and balance exercise. For cross‐training, a strong resistance was applied by the therapist until a response was achieved on the contralateral lower extremity at the end range of motion, resistance was consistently applied for 10 seconds once a response was achieved, depending on the patient’s performance level, basic neurological physiotherapy and cross‐training were performed in various positions, including sitting position, standing position, supine position, and prone position." Comparisons of relevance to this review: (1) vs (2); and (1) or (2) vs (3)
Outcomes	**Independence in ADL scales:** NR **Motor function scales:** NR **Measures of balance:** NR **Measures of gait velocity:** timed up and go, 10‐m walk test **Length of stay:** NR **Adverse events:** NR **Other measures:** centre of pressure measures **Time points when outcomes were assessed:** pre‐ and post‐intervention
Comparison	**Comparison a ‐ Group (1) vs (2)** **Amount of physical rehabilitation in groups:** equivalent **Categorisation of comparison**: 4. Comparison of similar approaches (dose equivalent) **Comparison b ‐ Group (1) or (2) vs (3)** **Amount of physical rehabilitation in groups:** differs **Categorisation of comparison:** 5. Additional therapy + usual therapy versus usual therapy (dose differs)
Funding & conflicts of interest	**Funding statement:** supported by the National Research Foundation of Korea (NRF) funded by the Korea government (MSIT) No. 2017R1C1B5074040 **Conflict of interest statement:** none
Synthesis	**Quantitative synthesis:**Analysis 5.4
Notes	

**Table:** Pirayesh 2021

***Study characteristics***	
Methods	**Study aim:** "aimed to compare the effect of core stability training using Otago exercises on quality of life in patients with stroke" **Design:** multi‐arm RCT (3‐arm) **Country:** Iran **Study recruitment and setting:** see Table **Involvement of patients/public/professionals/others:** no
Participants	**Study population (number randomised):** 46 **Inclusion criteria:** Aged 40 to 70 years Diagnosed with ischaemic stroke by a neurologist > 6 months after the stroke Able to walk independently Able to participate in exercise Provided informed consent **Exclusion criteria:** Heart, lung, and neurological disorders Previously participated in similar studies **Participants:** detailed in Table **PROGRESS‐plus:** summarised in Table
Interventions	(1) Core stability exercises "Core stability exercises are performed to reduce the simultaneous activity of the paraspinal, abdominal, and gluteal muscles and reduce the risk of injury and pain to these muscles. These exercises stimulate the sensory and motor receptors of the muscles and stabilize the spine" Exercises included ‐ cat camel stretch, straight leg crunch, bridge exercises (2) Otago exercises "program includes strength and balance exercises that can improve mobility, functional balance, the balance of one leg, and lower limb strength and walk" Exercises including walking forward and backwards, sit to stand "Both groups performed the exercises for 8 weeks and 3 individual sessions per week for 30‐45minutes (starting from 30 minutes and reaching 45 minutes in the last weeks); the number of movements and the time of performing increased during eight weeks. " (3) Control "The control group received no intervention." Comparison of relevance to this review = (2) vs (3)
Outcomes	**Independence in ADL scales:** NR **Motor function scales:** NR **Measures of balance:** NR **Measures of gait velocity:** NR **Length of stay:** NR **Adverse events:** NR **Other measures:** quality of life **Time points when outcomes were assessed:** before and after intervention (1 month)
Comparison	**Amount of physical rehabilitation in groups:** differs **Categorisation of comparison:** 1. Physical rehabilitation versus no or limited physical rehabilitation (dose differs) (limited = < 50% of dose)
Funding & conflicts of interest	**Funding statement:** "research was supported by theYasuj University of Medical Sciences." **Conflict of interest statement:** not reported
Synthesis	**Qualitative synthesis:** no outcomes of relevance to this review
Notes	

**Table:** Puckree 2014

***Study characteristics***	
Methods	**Study aim:** to compare the effect of a balance and stability focused outpatient community‐based rehabilitation and a regular physiotherapy programme on balance, stability, and perceptions of improvement after acute stroke **Design:** parallel‐group RCT **Country:** South Africa **Study recruitment and setting:** see Table **Involvement of patients/public/professionals/others:** no
Participants	**Study population (number randomised):** 50 **Inclusion criteria:** First stroke Cognitively functional with or without communication impairment Informed consent **Exclusion criteria:** More than one stroke Previous lower limb fractures Unable to participate in low‐intensity exercise programmes due to severe complications from comorbidities or were HIV positive due to its neurologic sequelae **Participants:** detailed in Table **PROGRESS‐plus:** summarised in Table
Interventions	(1) Balance and stability programme Sitting and reaching, kneeling to half kneeling, standing and walking exercises (2) Traditional physiotherapy Auto‐assisted upper and lower limb exercises, rolling, bridging, trunk rotation, joint approximation, ball therapy, hand activities, gait re‐education in parallel bars. "The physiotherapy program for patients with stroke is an individualized 1‐hour session offered once every 2 weeks, which comprises individual therapy by therapists or standardized group therapy administered by therapists or assistants. Patients who are mobile, either with or without an assistive device, and who require minimal individual attention are treated in the stroke group. At the end of each session, each patient is given his or her next appointment date. To reduce variability in this study, the participants in both groups received individual treatments by a therapist. All the patients were requested to perform the exercises at home." "Physiotherapy for both the experimental and control groups continued for 12 sessions, once every 2 weeks, for 6 months."
Outcomes	**Independence in ADL scales:** NR **Motor function scales:** NR **Measures of balance:** postural assessment scale, Berg Balance Scale **Measures of gait velocity:** NR **Length of stay:** NR **Adverse events:** NR **Other measures:** NR **Time points when outcomes were assessed:** pre‐ and post‐treatment
Comparison	**Amount of physical rehabilitation in groups:** equivalent **Categorisation of comparison:** 3.(i) Comparison of different approaches (dose equivalent; with no other physical rehab)
Funding & conflicts of interest	**Funding statement:** not reported **Conflict of interest statement:** none
Synthesis	**Quantitative synthesis:**Analysis 7.3
Notes	

**Table:** Qian 2004

***Study characteristics***	
Methods	**Study aim:** "to observe the changes of functional comprehensive assessment (FAC) scores 1, 3 and 6 months after early rehabilitation in hemiplegic patients induced by stroke" **Design:** parallel‐group RCT **Country:** China **Study recruitment and setting:** see Table **Involvement of patients/public/professionals/others:** no
Participants	**Study population (number randomised):** 42 **Inclusion criteria:** Not reported **Exclusion criteria:** Not reported **Participants:** detailed in Table **PROGRESS‐plus:** summarised in Table
Interventions	(1) Rehabilitationgroup (n = 23) "Participants in the treatment group received therapy from a mix of approaches: (i) Exercises focused on the affected limbs with appropriate progression, 20 min/session, 1/day. (ii) Neuromuscular facilitation techniques, including Rood, PNF, Brunnstrom, Bobath and functional stimulative techniques, 20 min/session, 1x/day. (iii) Electrical stimulation: using Auto Move AM800, current set at between 0‐60mA into 2.5k Ω; set at 100‐400 µs; frequency 10‐100Hz; continuous stimulation time at between 2‐ 20s; resting time between stimulation 2‐50s. Intensity is set to as tolerated by participants, with each treatment lasting 20 min/session, 1/day. (iv) ADL practice: 1/day. (v) Rehabilitation included limb positioning, regular change in body position in bed etc. (vi) Acupuncture, 1/day" Length of intervention period: not stated Number of sessions and length of individual sessions: session length dependent on technique applied (see above) Intervention provider: "Exercises were assisted by a nurse or family under supervision by a therapist" (2) No physical rehabilitation group (n = 19) "The control group received routine rehabilitative instruction only" Length of intervention period: no intervention Number of sessions and length of individual sessions: no intervention Intervention provider: no intervention
Outcomes	**Independence in ADL scales:** NR **Motor function scales:** NR **Measures of balance:** NR **Measures of gait velocity:** NR **Length of stay:** NR **Adverse events:** NR **Other measures:** Functional Comprehensive Assessment (FCA) **Time points when outcomes were assessed**: before and 1, 3, and 6 months after intervention
Comparison	**Amount of physical rehabilitation in groups:** differs **Categorisation of comparison:** 1. Physical rehabilitation versus no or limited physical rehabilitation (dose differs) (limited = < 50% of dose)
Funding & conflicts of interest	**Funding statement:** not reported **Conflict of interest statement:** not reported
Synthesis	**Qualitative synthesis:** no data suitable for analysis Add link to table which shows which analyses
Notes	Original study translated from Chinese to English

**Table:** Qin 2013

***Study characteristics***	
Methods	**Study aim:** to observe the clinical effect of penetrating acupuncture at scalp points and retaining the needles in combination with exercise re‐learning training on hemiplegic gait in stroke patients **Design:** multi‐arm RCT (3‐arm) **Country:** China **Study recruitment and setting:** see Table **Involvement of patients/public/professionals/others:** no
Participants	**Study population (number randomised):** 60 **Inclusion criteria:** Permanent residents of Yinhang Community, aged 50 to 75 years Met the diagnostic criteria of the 4th National Cerebrovascular Disease Diagnosed as stroke patients with initial onset by CT or MRI Comply with the encephalopathy emergency cooperation group of the State Administration of Traditional Chinese Medicine. Diagnosis and Efficacy Evaluation Criteria for Stroke (Trial) Stroke onset 2 to 6 months Typical hemiplegic gait pattern (e.g. the visual swing phase includes foot drop, foot varus, straight knee, hip joint external rotation, circle gait, standing phase hip joint extension, insufficient ankle dorsiflexion, excessive horizontal lateral displacement of the pelvis, etc.) Step width is > 8 cm, the step length < 50 cm No previous formal gait training before **Exclusion criteria:** Obvious disturbance of consciousness and severe cognitive impairment, severe cardiopulmonary insufficiency Bone and joint diseases Malignant tumours Other neurological diseases, especially vestibular and cerebellar dysfunction Unilateral neglect or other complications such as severe proprioceptive loss Previous history of dementia and/or mental health condition **Participants:** detailed in Table **PROGRESS‐plus:** summarised in Table
Interventions	(1) Motor re‐learning programme (MRP) "Treatment was given once a week on Monday, Wednesday and Friday for 3 months. The specific operations of MRP include: 1. Standing period: a. Training the hip extension in the standing position: Lift the affected hip in the supine position to cause extensor activity; in the standing position, the hip is correctly aligned, and the healthy leg is used to train forward and backward steps. b. Knee control training in standing phase: extend the knee in a sitting position, the therapist applies pressure from the heel to the knee, and practice the eccentric concentric contraction of the quadriceps femoris and isometric contraction of the knee joint by flexing and extending the knee from 0° to 15°, improving the Quadriceps control of the knee; the healthy leg steps up and down 8 cm high steps. c. Training the pelvis to move horizontally: Stand in a standing position, with the hip in front of the ankle, and shift the center of gravity from one foot to the other. The range of movement is controlled at 2． About 5 cm, train sideways. 2. Swing period: a. Knee flexion at the initial stage of swing training: flex the knee joint less than 90° in the prone position, train the eccentric and concentric contraction of the flexor muscles through a small range of flexion and extension activities, maintain the knee joint at different angles for a certain period of time, and cannot flex the hip; In a standing position, help slightly bend the knees, train eccentric concentric contraction control, do not bend the knees too much to cause tension in the rectus femoris to induce hip flexion; when the affected leg moves forward, the therapist controls the initial knee flexion, and ensures that the hip is extended when stepping forward, Instruct knee flexion and foot dorsiflexion as you step back. b. Extend your knees and dorsiflex your feet when training your heels. 3. Walking training: The healthy and affected legs step alternately. If the affected leg is difficult to move, the therapist guides the patient with his own leg and gives a password to let him walk rhythmically. And observe and analyze the alignment of the patient, find out the problem, and improve the walking posture. 4. Increase the difficulty: Let the patient train in a public environment with people and objects moving. 5. Develop a home training plan for patients: It is recommended not to overuse assistive devices to help walking and prevent interference with correct feedback of balance control". (2) Motor relearning programme (MRP) + acupuncture "In the community hospital, the patients received scalp acupuncture and exercise re‐learning training was given under the needle retention. The acupuncture method is as follows: Take the side of the lesion, and perform 4 consecutive relay acupuncture needles on the connecting line from Baihui to the temple. After routine disinfection of local skin with 75% ethanol, use 0． The 25 mm × 40 mm Tianxie brand disposable acupuncture needles were first inserted vertically into the subcutaneous tissue, and after reaching the subgaleal aponeurosis, the needles were inserted into the skin gently, rapidly, and without twisting 30 mm at a 15° angle. Replenishing and reducing method: Quickly twist for 2 minutes, the frequency is more than 200 rpm, the needle is retained for 30 minutes, and the needle is performed once at an interval of 8 minutes. After repeating 3 times, the needle is quickly released without twisting. Motor relearning programme...[as above]" (3) Acupuncture "Simple acupuncture treatment was performed by acupuncturists in our hospital outpatient or door‐to‐door. The acupuncture operation was the same as that of the comprehensive treatment group. The needle retention time was also 30 min. " Comparison relevant to this review: (2) versus (3)
Outcomes	**Independence in ADL scales:** NR **Motor function scales:** Fugl Meyer assessment **Measures of balance:** NR **Measures of gait velocity:** timed up and go test **Length of stay:** NR **Adverse events:** NR **Other measures:** Satisfaction With Life Scale (SWLS) **Time points when outcomes were assessed:** before and after 3 months of treatment
Comparison	**Amount of physical rehabilitation in groups:** differs **Categorisation of comparison:** 1. Physical rehabilitation versus no or limited physical rehabilitation (dose differs) (limited = < 50% of dose) [note: both treatment groups receive acupuncture]
Funding & conflicts of interest	**Funding statement:** not reported **Conflict of interest statement:** not reported
Synthesis	**Quantitative synthesis:**Analysis 1.2; Analysis 1.4
Notes	This study was published in Chinese and translated into English using Google Translate.

**Table:** Qin JianJian 2014

***Study characteristics***	
Methods	**Study aim:** to investigate the clinical efficacy of Bobath technique in stroke patients with hemiplegia **Design:** parallel‐group RCT **Country:** China **Study recruitment and setting:** see Table **Involvement of patients/public/professionals/others:** no
Participants	**Study population (number randomised):** 61 **Inclusion criteria:** Met the diagnostic criteria for stroke established by the 4th National Cerebrovascular disease **Exclusion criteria:** Not reported **Participants:** detailed in Table **PROGRESS‐plus:** summarised in Table
Interventions	(1) Bobath therapy "the observation group was given Bobath technique treatment. The main methods were as follows: during the flaccid phase, the training mainly included good limb placement, passive joint activities, promotion of muscle strength and muscle tension recovery, and combination of active and passive activities; during the spastic phase, anti‐spasm, induce dissociative exercise, improve motor function; recovery period is mainly to induce dissociative exercise, overcome abnormal patterns, daily life action training, etc., 1 time/d, 45 min/time, treatment for 4 weeks." "Bobath technique is co‐founded by British physiotherapist Berta Bobath and her husband Karel Bobath. It is mainly used for the treatment of hemiplegia patients and children with cerebral palsy. Reflex inhibiting pattern (RIP) and good limb placement are used to suppress spasticity" (2) Conventional rehabilitation "Both groups received conventional rehabilitation training."
Outcomes	**Independence in ADL scales:** Barthel Index **Motor function scales:** Fugl Meyer assessment **Measures of balance:** NR **Measures of gait velocity:** NR **Length of stay:** NR **Adverse events:** NR **Other measures:** NR **Time points when outcomes were assessed:** before and after treatment
Comparison	**Amount of physical rehabilitation in groups:** differs **Categorisation of comparison:** 5. Additional therapy + usual therapy versus usual therapy (dose differs) Note: lack of detail about conventional rehabilitation. It has been assumed that Bobath therapy is provided 'in addition' to other physical rehabilitation.
Funding & conflicts of interest	**Funding statement:** not reported **Conflict of interest statement:** not reported
Synthesis	**Quantitative synthesis:**Analysis 5.1; Analysis 5.2
Notes	This study was published in Chinese and translated into English using Google Translate.

**Table:** Rahayu 2020

***Study characteristics***	
Methods	**Study aim:** to investigate the effect of physiotherapy interventions on brain neuroplasticity by evaluating the brain plasticity regeneration, balance and functional ability **Design:** parallel‐group RCT **Country:** Indonesia **Study recruitment and setting:** see Table **Involvement of patients/public/professionals/others:** no
Participants	**Study population (number randomised):** 64 **Inclusion criteria:** Diagnosed with ischaemic stroke 24 hours post‐attack and without deterioration in neurological conditions Decreased motor control Sensory deficit and proprioceptive in upper and lower limbs Muscle strength of at least 2+ by manual muscle testing No aphasia Informed consent **Exclusion criteria:** Ischaemic stroke patients with serious complications (e.g. decreased consciousness, sepsis, shock due to embolism) Severe cognitive damage (MMSE < 19) **Participants:** detailed in Table **PROGRESS‐plus:** summarised in Table
Interventions	(1) Neurorestoration intervention "The neurorestoration intervention comprised three stages: cognitive, associative and automation. The standard intervention protocol used were 1) Bobath, 2) PNF, 3) Rood, Carr & Shepherd and 4) CIMT. Each standard protocol was implemented every day according to the needs and patient's improvement" (2) Conventional physiotherapy "Activities given were not fixed, however they were in concordance with the standard operation procedure of the institutions and comprised position change, breathing exercise and exercise therapy in passive and active mobilisation". Both interventions ‐ 60 minutes a day for 7 days
Outcomes	**Independence in ADL scales:** Barthel Index **Motor function scales:** NR **Measures of balance:** Berg Balance Scale **Measures of gait velocity:** NR **Length of stay:** NR **Adverse events:** NR **Other measures:** brain‐derived neurotropic factor (BDNF) **Time points when outcomes were assessed:** pre‐ and post‐intervention
Comparison	**Amount of physical rehabilitation in groups:** equivalent **Categorisation of comparison:** 3.(i) Comparison of different approaches (dose equivalent; with no other physical rehab)
Funding & conflicts of interest	**Funding statement:** not reported, but authors note "conducted as part of the first author’s PhD study at the Universitas Gadjah Mada Yogyakarta, Indonesia." **Conflict of interest statement:** none
Synthesis	**Quantitative synthesis:**Analysis 7.1; Analysis 7.3
Notes	

**Table:** Renner 2016

***Study characteristics***	
Methods	**Study aim:** to compare the efficacy of intensive daily applied progressive group therapy task training with equally dosed individual progressive task training on self‐reported mobility for patients with moderate to severe stroke during inpatient rehabilitation **Design:** parallel‐group RCT **Country:** Germany **Study recruitment and setting:** see Table **Involvement of patients/public/professionals/others:** no
Participants	**Study population (number randomised):** 73 **Inclusion criteria:** Verified stroke according to WHO confirmed by CT scan Able to sit and stand independently Able to walk with assistance (i.e. FAC ⩾ 2 and ⩽ 4) with or without an aid or orthosis Informed consent Motivated to participate in a 6‐week intensive programme of physiotherapy Able to understand instructions (MMSE > 23) **Exclusion criteria:** Lived > 70 km from rehabilitation centre **Participants:** detailed in Table **PROGRESS‐plus:** summarised in Table
Interventions	(1) Group therapy task training "Patients assigned to the experimental group received a 90‐minute, structured progressive task training program five times a week over a six week period (30 sessions). Each training included eight out of ten available work stations, intended to improve tasks relating to walking competency, such as balance control, stair walking, turning, transfers and speed walking. Graded progression was achieved by (1) increasing the difficulty of the task, (2) adding weights, or (3) increasing the number of repetitions. At each work station, participants worked together in pairs: while one participant performed the task for three minutes, the other one observed him and counted the number of repetitions. After three minutes of practice or observation, they reversed roles. After six minutes at one work station, each pair had one minute to go to the next work station." "The total group training program included four stages: warming up (10 minutes), task training (60 minutes), sports and games (15 minutes) and cooling down (5 minutes)." (2) Individual task training "Patients allocated to the individual training received a 90‐minute, progressive individually tailored task training 5 times a week over a 6 week period (30 sessions) offered by one of the staff physical therapists. The training was tailored to the deficits of the patient and aimed to improve balance, physical condition and walking competency, preferably using a graded progression" "Both interventions lasted six weeks and were given during in‐patient rehabilitation, the broad aim of both types of intervention was to improve the patients’ mobility to allow safe discharge to their homes. Both groups received all other therapies including neuropsychology, speech, and occupational therapy for the upper paretic limb, as needed."
Outcomes	**Independence in ADL scales:** NR **Motor function scales:** Rivermead Mobility Index, Motricity Index **Measures of balance:** NR **Measures of gait velocity:** 10‐m walk test, timed up and go **Length of stay:** NR **Adverse events:** "The physiotherapist recorded adverse events,including falls and heart problems" **Other measures:** Stroke Impact Scale, mobility domain; Falls Efficacy Scale; Hospital Anxiety & Depression scale; Fatigue Severity scale; functional ambulation categories, 6‐minute walk test, timed balance test, chair rise test, stair climb test **Time points when outcomes were assessed:** baseline, after 6 weeks, and after 24 weeks
Comparison	**Amount of physical rehabilitation in groups:** equivalent **Categorisation of comparison:** 4. Comparison of similar approaches (dose equivalent)
Funding & conflicts of interest	**Funding statement:** "This research received no specific grant from any funding agency in the public, commercial, or not‐for‐profit sectors." **Conflict of interest statement:** "The authors declare that there is no conflict of interest"
Synthesis	**Qualitative synthesis:** studies comparing similar approaches were not combined within statistical analyses. Note: main difference is the mode of delivery (i.e. group vs individualised), making this study relevant for inclusion in a subgroup focussed on mode of delivery.
Notes	

**Table:** ReTrain 2018

***Study characteristics***	
Methods	**Study aim:** "to assess acceptability and feasibility of trial processes and the Rehabilitation Training (ReTrain)intervention including an assessment of intervention fidelity" **Design:** parallel‐group RCT (with parallel mixed methods process and economic evaluations) **Country:** United Kingdom **Study recruitment and setting:** see Table **Involvement of patients/public/professionals/others:** yes (authors "are very grateful for PenCLAHRC for their support of the study and to the Peninsula Patient Involvement Group for helping guide the trial design")
Participants	**Study population (number randomised):** 45 **Inclusion criteria:** Diagnosis of stroke Any time since stroke but > 1 month since discharge from National Health Service (NHS) physical rehabilitation services Able to walk independently indoors (with/without mobility aids) but with self‐reported difficulty with stairs, slopes or uneven surfaces Willing to be randomised and attend the training venue Sufficient cognitive capacity and communication ability to allow participation **Exclusion criteria:** < 18 years old Currently (or within 1 month of) receiving ARNI training Contraindications to moderate to vigorous physical activity **Participants:** detailed in Table **PROGRESS‐plus:** summarised in Table
Interventions	(1) "ReTrain" "Training is based on a manual and led by personal trainers on the UK Register of Exercise Professionals (level 3 or above) who are ARNI‐trained and accredited and have had additional training in the delivery of ReTrain. There was a maximum ratio of one trainer to four stroke survivors. ReTrain was delivered in a community setting (one gym, two church halls and one community centre) with twice‐weekly 2‐hour sessions over 3 months, comprising:an introductory one‐to‐one session (home visit); 10, twice‐weekly group classes with up to 2 trainers and 8 clients (training venue); a closing one‐to‐one session (home visit); followed by 3(one per month) drop‐in sessions. Participants completed bespoke home‐based training (homework) throughout." (2) Control "All participants received treatment as usual. This ranged from zero treatment to engagement with any health service(s). We requested that all trial participants did not participate in additional physical rehabilitation (either NHS or private) but we could not prevent them from doing so. We did not monitor control group participation in any treatments during the trial but did record health service use at the end of the trial for all participants. The control group also received an advice booklet about exercise after stroke."
Outcomes	**Independence in ADL scales:** NR **Motor function scales:** Rivermead Mobility Index, modified Patient‐Specific Functional Scale **Measures of balance:** NR **Measures of gait velocity:** timed up and go test **Length of stay:** NR **Adverse events:** adverse and serious adverse events during intervention and assessment period. **Other measures:** feasibility, acceptability and process outcomes; acceptability of randomisation, outcome measurement burden and the intervention; safety (adverse events); intervention fidelity; accelerometry; Stroke Self‐Efficacy Questionnaire; Fatigue Assessment Scale; Exercise Beliefs and Exercise Self‐Efficacy questionnaires,33SF12,34EQ‐5D‐5L; Stroke Quality of Life (QoL) questionnaires; Carer Burden Index; Health and Social Service use through a Service Receipt Inventory **Time points when outcomes were assessed:** "baseline assessments (completed by research team) and follow‐up assessments (at 6 and 9months, completed by blinded assessor)"
Comparison	**Amount of physical rehabilitation in groups:** differs **Categorisation of comparison:** physical rehabilitation versus no physical rehabilitation (dose differs)
Funding & conflicts of interest	**Funding statement:** "The Stroke Association TSA 2014‐13" **Conflict of interest statement:** "Dean reports grants from The Stroke Association, during the conduct of the study; other from Wiley Blackwell, outside the submitted work; Forster, Poltawski, Spencer, Taylor, James, Allison, Stevens, Pulsford and Norris report grants from the Stroke Association; Calitri, Landa, Hollands, Shepherd were employed by the Stroke Association grant"
Synthesis	**Quantitative synthesis:**Analysis 1.2; Analysis 1.4; Analysis 1.6; Analysis 2.2; Analysis 2.4
Notes	

**Table:** Richards 1993

***Study characteristics***	
Methods	**Study aim:** "to estimate the effects of early, intensive, gait‐focused physical therapy on ambulatory ability in acute stroke patients" **Design:** parallel‐group RCT **Country:** Canada **Study recruitment and setting:** see Table **Involvement of patients/public/professionals/others:** no
Participants	**Study population (number randomised):** 27 **Inclusion criteria:** Middle cerebral artery infarct Confirmed by CT scan Living less than 50 km from Quebec 40 to 80 years old Zero to 7 days since onset of stroke No other neurological problems No major medical problems that would interfere with rehabilitation Not independent in ambulation Not unconscious at onset **Exclusion criteria:** Not reported **Participants:** detailed in Table **PROGRESS‐plus:** summarised in Table
Interventions	(1) Motor learning (n = 10) Intensive and focused "Goal was to promote gait relearning through locomotor activities that were adapted to the individual level of motor recovery" Techniques included tilt table; limb load monitor, resistive exercises, with isokinetic exercises; treadmill training Length of intervention period: whilst inpatient Number of sessions and length of individual sessions: "intensive" Intervention provider: physiotherapists (2) Bobath (n = 8) Techniques not described but communication with the study author confirms this intervention as ‘neurodevelopmental or Bobath' Length of intervention period: whilst inpatient Number of sessions and length of individual sessions: "intensive" Intervention provider: physiotherapists (3) Conventional—neurophysiological (Bobath) (n = 9) Length of intervention period: whilst inpatient Number of sessions and length of individual sessions: as usual care (less than treatment groups) Intervention provider: physiotherapists Comparison of relevance to this review: Group (1) versus Group (2). No data have been extracted for group 3.
Outcomes	**Independence in ADL scales:** Barthel Index **Motor function scales:** Fugl‐Meyer Motor Assessment **Measures of balance:** Berg Balance Scale **Measures of gait velocity:** temporal gait parameters **Length of stay:** NR **Adverse events:** NR **Other measures:** Canadian Stroke Scale **Time points when outcomes were assessed:** "Gait movements and muscle activations were evaluated in the Motor Evaluation Laboratory six weeks, three months, and six months after stroke"
Comparison	**Amount of physical rehabilitation in groups:** equivalent **Categorisation of comparison:** 3.(i) Comparison of different approaches (dose equivalent; with no other physical rehab)
Funding & conflicts of interest	**Funding statement:** "supported by grants from the National Health Research and Development Program (Canada) and the L'Hopital de l'Enfant‐Jesus Foundation" **Conflict of interest statement:** "no commercial party having a direct or indirect interest in the subject matter of this article has conferred or will confer a benefit upon the authors or upon any organization with which the authors are associated"
Synthesis	**Quantitative synthesis:**Analysis 7.1; Analysis 7.2; Analysis 7.3; Analysis 7.4
Notes	Analysis based on comparison of neurophysiological (early) with mixed (early), as these two groups are comparable in terms of timing and intensity

**Table:** Salbach 2004

***Study characteristics***	
Methods	**Study aim:** "to evaluate the efficacy of a task‐orientated intervention in enhancing competence in walking in people with stroke" **Design:** parallel‐group RCT **Country:** Canada **Study recruitment and setting:**Table **Involvement of patients/public/professionals/others:** no
Participants	**Study population (number randomised):** 91 **Inclusion criteria:** Clinical diagnosis of stroke < 12 months post‐stroke Residual walking deficit but able to walk 10 m (with or without aid or supervision) Discharged from physical rehabilitation Living in community **Exclusion criteria:** Not reported **Participants:** detailed in Table **PROGRESS‐plus:** summarised in Table
Interventions	(1) Task‐specific training (n = 44) Task‐orientated training of walking "Standardised programme, supervised by a physical or occupational therapist, of 10 walking‐related tasks designed to strengthen the lower extremities and enhance walking balance, speed and distance in a progressive manner" Length of intervention period: 6 weeks Number of sessions and length of individual sessions: 18 sessions given three times per week. Recommended that participants carry over walking component of the programme to home. Intervention provider: physical or occupational therapist (2) Attention control (motor learning, upper limb) (n = 47) Functional upper extremity tasks, done sitting Length of intervention period: 6 weeks Number of sessions and length of individual sessions: 18 sessions given 3 times per week. Recommended that participants carry over walking component of the programme to home Intervention provider: physical or occupational therapist
Outcomes	**Independence in ADL scales:** NR **Motor function scales:** NR **Measures of balance:** Berg Balance Scale **Measures of gait velocity:** gait speed **Length of stay:** NR **Adverse events:** NR **Other measures:** NR **Time points when outcomes were assessed:** "evaluations were conducted by trained evaluators at baseline, and on completion of the intervention (mean four days)"
Comparison	**Amount of physical rehabilitation in groups:** differs **Categorisation of comparison:** 2. Physical rehabilitation versus attention control (dose of physical rehab differs; dose of intervention equivalent)
Funding & conflicts of interest	**Funding statement:** "NMS received a fellowship from the Canadian Institutes of Health Research to conduct this study. Operating funds were provided by the *Quebec Reseau provincial de recherche en adaptation‐readaptation*, the Heart and Stroke Foundation of Canada, and the Canadian Stroke Network" **Conflict of interest statement:** not reported
Synthesis	**Quantitative synthesis:**Analysis 3.3; Analysis 3.4
Notes	Intervention 2 comprised upper limb training. Although upper limb training might be classed as a subcomponent of functional task training, for the purposes of comparison in this review, we have categorised this intervention as an attention control. This is because the upper limb training was delivered alone (i.e. no other subcomponents of functional task training were delivered), and therefore no active treatment was aimed at lower limb or balance outcomes.

**Table:** Sekhar 2013

***Study characteristics***	
Methods	**Study aim:** "to evaluate the efficacy of isokinetic strength training through isokinetic device in people with stroke" **Design:** parallel‐group RCT **Country:** India **Study recruitment and setting:** see Table **Involvement of patients/public/professionals/others:** no
Participants	**Study population (number randomised):** 40 **Inclusion criteria:** Aged 40 to 60 years; both sexes First time stroke with hemiplegia MAS of 1‐2 **Exclusion criteria:** Haemianopia Lower limb contractures Cognitive or sensory deficits Other neurological and musculoskeletal conditions Cardiovascular disease Recurrent stroke Unable to comprehend or obey verbal commands **Participants:** detailed in Table **PROGRESS‐plus:** summarised in Table
Interventions	(1) Isokinetic strengthening and balance exercises "A 6 week program which includes 5 min warm‐up on stationary bicycle at low resistance followed by 65 seconds stretches for hamstrings and quadriceps group of muscles on effected side. Strength training with knee extensors on isokinetic machine. 3 sets of 6 to 8 repetition of maximum efforts followed by cool down section of 5 min of cycling, followed by balance exercises". Balance exercises include sitting, standing and dynamic balance." (2) Conventional physiotherapy "A 6 weeks program which includes Active exercises for all groups of muscles in lower limb on effected side, Pelvic bridging followed by Modified pelvic bridging, resistance exercises for all group of muscle in lower limb with one kg weight cuff and manual resistance, weight bearing exercise in quadripod position, kneeling, half kneeling, standing, standing with single limb support, manual perturbations..."
Outcomes	**Independence in ADL scales:** NR **Motor function scales:** NR **Measures of balance:** Berg Balance Scale **Measures of gait velocity:** NR **Length of stay:** NR **Adverse events:** NR **Other measures:** peak torque in knee extensors **Time points when outcomes were assessed:** NR
Comparison	**Amount of physical rehabilitation in groups:** equivalent **Categorisation of comparison:** 3.(i) Comparison of different approaches (dose equivalent; with no other physical rehab)
Funding & conflicts of interest	**Funding statement:** not reported **Conflict of interest statement:** not reported
Synthesis	**Quantitative synthesis:**Analysis 7.3
Notes	

**Table:** Seo 2015

***Study characteristics***	
Methods	**Study aim:** "examined the effects of ramp gait training using lower extremity patterns of proprioceptive neuromuscular facilitation (PNF) on chronic stroke patients’ dynamic balance ability" **Design:** parallel‐group RCT **Country:** Rep. Korea **Study recruitment and setting:** see Table **Involvement of patients/public/professionals/others:** no
Participants	**Study population (number randomised):** 20 **Inclusion criteria:** Chronic stroke patients diagnosed as having stroke resulting from a cerebral haemorrhage using CT or MRI Stroke onset > 6 months Able to maintain an independent standing posture for 30 s or longer Able to walk 30 m or longer alone indoors Able to communicate enough and to understand oral instructions Not using assistive devices or receiving medication to alleviate spasticity **Exclusion criteria:** Not reported **Participants:** detailed in Table **PROGRESS‐plus:** summarised in Table
Interventions	(1) Ramp gait exercise with PNF "The experimental group conducted flexion of the knee joint of the paretic side and flexion, adduction, and external rotation of the hip joint of the paretic side. The therapist held the ankle and anterior medial part of the knee of the experimental group and directed, “Raise your ankle and flex the lower limb up the diagonal line”. The experimental group moved their paretic lower limb joint to the end of the range of motion at the same time, and to provide the appropriate resistance, the therapist with the right hand placed on the knee joint applied force to the lateral part of the knee and applied resistance to the adduction and external rotation of the hip joint with the other hand. The experimental group received training on a specially devised ramp whose angle of inclination, length, and width were 10°, 10 m, and 0.8 m, respectively, for 30 min per day. Gait training involving traveling back and forth over 10m was conducted three times per week for four weeks. The subjects received PNF lower extremity gait pattern training with the help of a physical therapist in front of the ramp gait‐training device. The training method was the same as that for the control group." (2) PNF Gait pattern training "The control group received PNF gait pattern training by the therapist, wherein they walked back and forth over 10m. This exercise was conducted for 30min three times per week for four weeks. "
Outcomes	**Independence in ADL scales:** NR **Motor function scales:** NR **Measures of balance:** Berg Balance Scale **Measures of gait velocity:** timed up and go test **Length of stay:** NR **Adverse events:** NR **Other measures:** NR **Time points when outcomes were assessed:** before and after intervention
Comparison	**Amount of physical rehabilitation in groups:** equivalent **Categorisation of comparison:** 4. Comparison of similar approaches (dose equivalent)
Funding & conflicts of interest	**Funding statement:** "Research was supported by the Korean NazareneUniversity Research Grants 2015" **Conflict of interest statement:** not reported
Synthesis	**Qualitative synthesis:** Studies comparing similar approaches were not combined within statistical analyses.
Notes	

**Table:** Severinsen 2014

***Study characteristics***	
Methods	**Study aim:** "to directly compare the effects of aerobic training (AT) with progressive resistance training (RT) after stroke to determine whether AT‐induced fitness gains or RT‐induced strength gains translate into improved ambulation across a 12wk intervention and whether gains are retained 1 yr after cessation of formal training" **Design:** multi‐arm RCT (3‐arm) **Country:** Denmark **Study recruitment and setting:** see Table **Involvement of patients/public/professionals/others:** no
Participants	**Study population (number randomised):** 43 **Inclusion criteria:** Non‐haemorrhagic stroke verified by CT 6 to 36 months post‐stroke Aged 50 to 80 years MRC muscle strength > 3 on for the paretic lower limb Walking velocity < 1.4 m/sec at a fast 10 mWT with/without assistive walking devices **Exclusion criteria:** Global aphasia and spatial neglect syndrome Other neurologic disorders, including previous stroke Psychiatric disorders Severe orthopaedic or medical morbidity Moderate to severe depression (Multiple Depression Inventory cutoff value of 25 Dementia (MMSE cutoff value of 20) ≥ 6 training sessions missed (resulted in termination of study participation) **Participants:** detailed in Table **PROGRESS‐plus:** summarised in Table
Interventions	(1) Resistance training (RT) "High‐intensity progressive RT of both lower limbs (n= 14) consisted of three sets of eight repetitions targeted at an intensity of 80% of one‐repetition maximum [27](1RM; i.e., the maximal load that can be lifted once). 1RM was adjusted every second week" (2) Aerobic training (AT) "High‐intensity AT (n= 13) consisted of 15 mins of strenuous cycle ergometer (Monark) exercise, three times at each session. Training intensity was regularly modified by the physiotherapist, with the aim of reaching a pulse rate of 75% of the heart rate reserve" (3) Sham training "Low‐intensity RT of the arms (n= 16) consisted of three sets of 15 repetitions less than 60% of 1RM bilaterally. The participants performed elbow flexion and extension and shoulder abduction and combined shoulder movements using a pulley" Comparison of relevance to this review: (1) vs (3)
Outcomes	**Independence in ADL scales:** NR **Motor function scales:** NR **Measures of balance:** NR **Measures of gait velocity:** 6‐minute walk test, 10‐m walk test **Length of stay:** NR **Adverse events:** NR **Other measures:** Physical Activity Scale, aerobic capacity, knee muscle strength, Short Form 36 **Time points when outcomes were assessed:** before and after training, 1 year follow‐up
Comparison	**Amount of physical rehabilitation in groups:** differs **Categorisation of comparison:** 2. Physical rehabilitation versus attention control (dose of physical rehab differs; dose of intervention equivalent)
Funding & conflicts of interest	**Funding statement:** "Supported by grants from the VELUX Foundation and the BEVICA Foundation, both in Denmark" **Conflict of interest statement:** "Financial disclosure statements have been obtained, and no conflicts of interest have been reported by the authors or by any individuals in control of the content of this article"
Synthesis	**Qualitative synthesis:** data not presented in format suitable for inclusion in meta‐analysis.
Notes	

**Table:** Shin 2011

***Study characteristics***	
Methods	**Study aim:** "to investigate the effects of combined exercise training with aerobic and functional strengthening exercises on balance ability of hemiplegic stroke patients" **Design:** parallel‐group RCT **Country:** Republic of Korea **Study recruitment and setting:** see Table **Involvement of patients/public/professionals/others:** no
Participants	**Study population (number randomised):** 21 **Inclusion criteria:** Between 6 months and 5 years since diagnosis of stroke Subjects with hemiplegia of the lower extremities **Exclusion criteria:** Unable to ride a bicycle or perform functional exercise due to arthritis Low‐back pain, or degenerative joint disease Receiving medical treatment due to other symptoms Unable to follow the instructions due to low perceptive abilities, cognitive disorder, or communication disorder **Participants:** detailed in Table **PROGRESS‐plus:** summarised in Table
Interventions	(1) Exercise training (n = 11) "Exercise combined with aerobic and functional strengthening exercises for balance" "The first exercise was 30 min of functional strength training, consisting of six sub‐categories: bridge exercise, lifting toes, and ankles, sitting and standing, stretching out the arms while standing, step exercise, and stairs exercise. Bridge exercise is lifting pelvis using the legs, from bending hips and knees with supine. Lifting toes and ankles is dorsiflexion and plantar flexion of the hemiplegic leg in the sitting position. Sitting and standing is standing from sitting and sitting again until the hip touches chair. Stretching out the arms while standing is stretching out the arms upward, downward, right‐side, left‐side and diagonally. Step exercise is shifting of weight bearing to a leg on a step. The hemiplegic leg and non‐hemiplegic legs are placed in turn on the step and the location of step alternates from the front to one side of the subject. Stairs exercise is walking up stairs with the hemiplegic leg supporting the body weight and walking down stairs with the non‐hemiplegic leg support body weight. Before exercise, 5 minutes warming‐up exercise of breathing exercise and stretching were conducted. Each exercise was repeated at medium intensity without fatigue ten to fifteen times. The second exercise for the combined exercise training group was aerobic exercise. Treadmill walking and riding a bicycle were conducted for fifteen minutes each. Treadmill walking started at 0.5 m/s and the initial 5 minutes was on adaption period. In the next 10 minutes walking velocity increased or walking was done with less support from the hand‐rail. A stationary bicycle was used for the bicycle riding exercise. At the beginning a patient started with a velocity which he/she could feel comfortable with. As time went by the velocity was increased. The intensity of the two aerobic exercises was determined by checking the heart rate" Length of intervention period: 4 weeks Number of sessions and length of individual sessions: 60 minutes per day, 5 times a week Intervention provider: physical therapist ("aerobic exercise was conducted with the assistance of a caregiver or family member under the supervision of a physical therapist") (2) Conventional group (n = 10) "Special instructions were not given to the physical therapist in charge, and the conventional training was done as usual. The therapist focused on re‐educating normal movement during functional activities that were meaningful to the patients. Training was composed of balance exercise, posture control exercise, and gait exercise. Keeping normal movement of the pelvis for balance and posture control was emphasized. The therapist judged that working on increasing anterior and posterior pelvic tilt would improve weight transfer and hip extension during gait, leading to improvements in selective distal control of the knee and the foot. Trunk control and alignment can affect muscle tone, range of motion, and control of the limb. For the upper limbs, treatment was conducted focusing on movement of the scapular. For balance exercise, weight transfer exercise and reaching exercise were alternately performed on the affected side and the unaffected side in the sitting or standing position. Bridging exercise was performed to strengthen the trunk muscles. Selective movement of each joint of the shoulder, elbow, knee and ankle joint was performed to facilitate upper and lower limb movement. For gait exercise, training in weight transfer during gait was conducted by planting the unaffected side foot at the front and back of the body. Gait training was divided between the stance phase and the swing phase, and exercises for each phase were performed. In addition, stair climbing practice and gait training for crossing obstacles were conducted. Patients did not do the same exercise every training day but suitable exercises were selected according to the goals of each patient and the therapist. The intensity of each exercise was decided by the therapist considering each patient’s capacity for exercise" Length of intervention period: 4 weeks Number of sessions and length of individual sessions: 60 minutes per day, 5 times a week Intervention provider: physical therapist Note: The conventional exercise group described facilitation of 'normal movement', referenced to Bobath/Davies.
Outcomes	**Independence in ADL scales:** NR **Motor function scales:** NR **Measures of balance:** Berg Balance Scale **Measures of gait velocity:** NR **Length of stay:** NR **Adverse events:** NR **Other measures:** force platform (static balance) **Time points when outcomes were assessed:** "start of the intervention ... and after completion of the 4‐week intervention"
Comparison	**Amount of physical rehabilitation in groups:** equivalent **Categorisation of comparison:** 3.(i) Comparison of different approaches (dose equivalent; with no other physical rehab)
Funding & conflicts of interest	**Funding statement:** not reported **Conflict of interest statement:** not reported
Synthesis	**Quantitative synthesis:**Analysis 7.3
Notes	

**Table:** Shuai 2013

***Study characteristics***	
Methods	**Study aim:** "to observe the effect of motor relearning therapy on the recovery of motor function in patients with stroke hemiplegia" **Design:** parallel‐group RCT **Country:** China **Study recruitment and setting:** see Table **Involvement of patients/public/professionals/others:** no
Participants	**Study population (number randomised):** 62 **Inclusion criteria:** Met the diagnostic criteria revised by the Fourth National Cerebrovascular Disease Academic Conference Stroke confirmed by CT or MRI **Exclusion criteria:** Not reported **Participants:** detailed in Table **PROGRESS‐plus:** summarised in Table
Interventions	(1) Motor re‐learning "The observation group used motor relearning therapy to train the patients[2‐4]. During the training process, it is necessary to use repeated explanations, instructions, exercises, language and manual guidance to strengthen the patient's training, and guide the patient to integrate various training into their daily life, and continuously use the learned movements to complete various daily routines. life activities." (2) Conventional rehabilitation "The control group was mainly treated with Bobath technique, combined with Brunstrum technique and neuromuscular proprioceptive technique" "Both groups were treated with one‐to‐one treatment, 45‐60 min, once a day, 5 days a week."
Outcomes	**Independence in ADL scales:** modified Barthel Index **Motor function scales:** Fugl‐Meyer motor function **Measures of balance:** NR **Measures of gait velocity:** NR **Length of stay:** NR **Adverse events:** NR **Other measures:** clinical neurological deficit score (CNS) **Time points when outcomes were assessed:** before and after 8 weeks of treatment
Comparison	**Amount of physical rehabilitation in groups:** equivalent **Categorisation of comparison:** 3.(i) Comparison of different approaches (dose equivalent; with no other physical rehab)
Funding & conflicts of interest	**Funding statement:** not reported **Conflict of interest statement:** not reported
Synthesis	**Quantitative synthesis:**Analysis 7.1; Analysis 7.2
Notes	This study was published in Chinese and translated into English using Google Translate.

**Table:** Signal 2014

***Study characteristics***	
Methods	**Study aim:** "to determine if Strength for Task Training (STT) has potential as an effective rehabilitation strategy to improve walking ability in people following stroke and to establish the feasibility of the research methods planned to conduct a larger Randomised Clinical Trial" **Design:** multi‐arm RCT (4‐arms) (mixed methods) **Country:** New Zealand **Study recruitment and setting:** see Table **Involvement of patients/public/professionals/others:** yes (acknowledgements state: "A special mention goes to those people who participated in the Stroke Advisory Group and those who contributed to the pilot study....")
Participants	**Study population (number randomised):** 20 **Inclusion criteria:** Aged 18 years+ Experienced single disabling stroke in the previous 3 to 9 months Able to walk 10 m (with/without aid or 'standby assistance') Gait speed of 0.05 to 1.2 m/s **Exclusion criteria:** Behaviour that may interfere with participation in a group setting (e.g. agitation, aggression) Significant cognitive deficit (MMSE ≤ 23) Unable to follow a 1‐step English verbal command Unable to provide informed consent Medically unsuitable Participating in another study Excessive pain or another condition that could impact results (e.g. substance abuse, significant mental illnesses such as major depression) Contra‐indications to TMS (e.g. pacemaker, artificial heart valves, other metal implants, pregnancy, skull abnormalities, history of seizures or epilepsy or taking medications that may lower seizure threshold) Cautions or contra‐indications to blood sampling (e.g. fear of needles, reception of a blood product or blood transfusion, on medications which adversely affect blood coagulation) **Participants:** detailed in Table **PROGRESS‐plus:** summarised in Table
Interventions	(1) Strength and task‐specific training Strength for task training (STT) "The STT intervention was designed to harness the priming effects of strength training and maximise the effects of strength and task‐specific training for improving locomotor abilities" Progressive resisted strength training (PRST) is undertaken immediately prior to task‐specific training (TST) of locomotor skills. "Participants complete up to three circuits of seven exercise stations during the hour, with each station comprising one set of the PRST exercise, a maximum transition time of 30 seconds followed by two minutes of TST of a related locomotor ability." Exercise stations: 1. Quadriceps (seated leg press); sit‐to‐stand‐to‐sit 2. Hamstrings (Westminster Pulley); walking backwards 3. Hip extensors (Westminster Pulley); stairs/getting off floor 4. Hip abductors (Theraband); walking sideways 5. Hip flexors (rotary hip); walking (comfortable speed) 6. Plantarflexors (supine leg press); walking (fast speed)/ramps 7. Dorsiflexors/evertors (Theraband); obstacles (2) Strength training Progressive resisted strength training (PRST) "received only the strength training component of the STT programme" (3) Task‐specific training (TST) "received only the task‐specific training component of the intervention" (4) Usual care (UCC) "The UCC group continued to receive standard rehabilitation through public and private health care services; no effort was made to influence the type or amount of rehabilitation these participants received. It should be noted that in the context of the NZ healthcare system people with stroke are likely to be receiving minimal or no active rehabilitation at 3‐9months post‐stroke" "Participants who were allocated to the STT, PRST or TST groups were scheduled to attend a group based exercise programme for twelve weeks, whilst the UCC group kept a record of any physical rehabilitation or organised exercise they engaged induring the intervention phase". Three times per week for 1 hour. Comparisons of relevance to this review: (1) vs (4); (2) vs (3)
Outcomes	**Independence in ADL scales:** NR **Motor function scales:** NR **Measures of balance:** NR **Measures of gait velocity:** walking speed, fast walking speed **Length of stay:** NR **Adverse events:** minor, moderate, serious adverse events **Other measures:** neural plasticity, leg muscle strength, 30‐second chair stand test, step test, stair ascent/descent, activities specific balance confidence questionnaire, Stroke Impact Scale, Subjective Index of Physical and Social Outcome **Time points when outcomes were assessed:** before and after intervention
Comparison	**Comparison (a) ‐ Group (1) vs (4)** **Amount of physical rehabilitation in groups:** differs **Categorisation of comparison:** 1. Physical rehabilitation versus no or limited physical rehabilitation (dose differs) (limited = < 50% of dose) **Comparison (b) ‐ Group (2) vs (3)** **Amount of physical rehabilitation in groups:** equivalent **Categorisation of comparison:**3.(i) Comparison of different approaches (dose equivalent; with no other physical rehab)
Funding & conflicts of interest	**Funding statement:** "Strategy to Advance Research Grant (STAR Grant) from Tertiary Education Commission's Building Research Capability in Strategically Relevant Areas (BRCSRA) fund" **Conflict of interest statement:** not reported
Synthesis	**Quantitative synthesis:**Analysis 1.4; Analysis 7.6; Analysis 7.10
Notes	

**Table:** Song 2015

***Study characteristics***	
Methods	**Study aim:** "to determine differences in gait abilities by comparing class‐based task‐oriented circuit training (CTCT) and individual‐based task‐oriented circuit training (ITCT)" **Design:** multi‐arm RCT (3‐arm) **Country:** Rep. Korea **Study recruitment and setting:** see Table **Involvement of patients/public/professionals/others:** no
Participants	**Study population (number randomised):** 30 **Inclusion criteria:** Not reported **Exclusion criteria:** Not reported **Participants:** detailed in Table **PROGRESS‐plus:** summarised in Table
Interventions	(1) Conventional physiotherapy + individual‐based task oriented circuit training (ITCT) Completed same tasks as CTCT under the supervision of 1 physiotherapist (2) Conventional physiotherapy + class‐based task‐oriented training (CTCT) 4 to 6 people completed circuits, under the supervision of 2 physiotherapists "The task‐oriented circuit training proposed by Salbach et al. was modified into a circular mode. The training tasks were sitting in chair and walking, walking over obstacles, carrying goods, turning the goods upside down, and walking fast in a circle" (3) Conventional physiotherapy (not described) "All subjects received conventional therapy for 30 minutes a day, five times a week; ... [treatment groups] received task oriented circuit training for 30 minutes a day, three times a week for four weeks." Comparison of most relevance to this review: (1) vs (3) (Comparison of (1) vs (2) is comparing two similar approaches).
Outcomes	**Independence in ADL scales:** NR **Motor function scales:** NR **Measures of balance:** NR **Measures of gait velocity:** gait velocity **Length of stay:** NR **Adverse events:** NR **Other measures:** gait parameters **Time points when outcomes were assessed:** before and after treatment
Comparison	**Comparison of group (1) vs (3)** **Amount of physical rehabilitation in groups:** differs **Categorisation of comparison:** 5. Additional therapy + usual therapy versus usual therapy (dose differs) **Comparison of group (1) vs (2)** **Amount of physical rehabilitation in groups:** equivalent **Categorisation of comparison:** comparison of similar approaches
Funding & conflicts of interest	**Funding statement:** "paper was supported by research funds provided by the Howon University" **Conflict of interest statement:** not reported
Synthesis	**Quantitative synthesis:**Analysis 5.4 (Comparison of similar approaches included in qualitative synthesis only)
Notes	

**Table:** SPIRES 2022

***Study characteristics***	
Methods	**Study aim:** "to explore these functionally linked tasks (prolonged standing and task‐specific training) in combination for people with severe sub‐acute stroke" **Design:** parallel‐group RCT (mixed methods ‐ nested qualitative evaluation) **Country:** United Kingdom **Study recruitment and setting:** see Table **Involvement of patients/public/professionals/others:** yes (patient and public contributors are acknowledged)
Participants	**Study population (number randomised):** 45 **Inclusion criteria:** Confirmed clinical diagnosis of new (first/recurrent) severe stroke Aged ≥ 18 years Graded as modified Rankin: 4 or 5 **Exclusion criteria:** Systolic blood pressure ≤ 100 mmHg or ≥ 220 mmHg at rest lying or sitting Oxygen saturation ≤ 87% with or without supplementary oxygen Resting heart rate of ≤ 40 or ≥ 110 beats per minute Temperature ≥ 38.5 degrees centigrade or ≤ 35 degrees centigrade Orthopaedic impairments which prevent full weight‐bearing in standing Malnutrition Universal Screening Tool score of ≥ 2, or not meeting nutritional demands for therapeutic interventions Documented clinical decision for receiving end of life care Unstable coronary or other medical condition that is judged to impose a medical risk to the patient Severe communication and/or cognitive deficit **Participants:** detailed in Table **PROGRESS‐plus:** summarised in Table
Interventions	(1) Functional standing frame programme "Functional standing frame programme (30min standing plus sit‐to‐stand repetitions) plus 15 min of usual physiotherapy. The intervention was protocolised to be delivered once a day for a minimum of fve and maximum of seven sessions per week for 3 weeks" "Each session was protocolised to last for 45 min to align with national UK recommendations [4]. This comprised 30 min (or as long as tolerated) using the standing frame (if required) which included standing and repeated sit‐to‐stand (up to 8–12 repetitions). An additional 15 min (or as long as tolerated) enabled practise of other activities deemed pertinent for discharge, such as transfers. The initial frequency and duration of standing was anticipated to vary according to physical capability; aiming to progress standing time and sit‐to‐stand repetitions by 30% in each session. If participants improved such that support from the standing frame was not required, they could progress to unsupported standing or walking for the remainder of the 3‐week intervention period as well as undertake sit‐to‐stand repetitions within each 30‐min session." (2) Usual physiotherapy "Participants allocated to the control group received usual physiotherapy (routine stroke rehabilitation physiotherapy delivered in each SRU) for 45 min once a day (or as long as tolerated)."
Outcomes	**Independence in ADL scales:** Barthel Index, Edmans ADL Index **Motor function scales:** NR **Measures of balance:** NR **Measures of gait velocity:** NR **Length of stay:** NR **Adverse events**: "Safety indicators AEs and SAEs were documented by treating physiotherapists in the Case Report Forms" **Other measures:** knee extensor strength, hip flexor length, muscle tone, trunk control test, fatigue, health‐related quality of life **Time points when outcomes were assessed:** before and after intervention, 15, 29, and 55 weeks follow‐up
Comparison	**Amount of physical rehabilitation in groups:** equivalent **Categorisation of comparison:** 3.(i) Comparison of different approaches (dose equivalent; with no other physical rehab)
Funding & conflicts of interest	**Funding statement:** "This protocol presents independent research funded by the National Institute for Health Research (NIHR) (ICA‐CDRF‐ 2015‐01‐044)." **Conflict of interest statement:** "authors declare that they have no competing interests"
Synthesis	**Quantitative analysis:** adverse events ‐ Analysis 7.6 **Qualitative synthesis:** Other results data not presented in a format suitable for quantitative analysis.
Notes	

**Table:** Stephenson 2004

***Study characteristics***	
Methods	**Study aim:** "to determine whether a whole task approach (body weight support treadmill training) or part task approach (proprioceptive neuromuscular facilitation training) is more effective in improving gait characteristics of individuals with chronic stroke" **Design:** multi‐arm RCT (3‐arm) **Country:** USA **Study recruitment and setting:** see Table **Involvement of patients/public/professionals/others:** no
Participants	**Study population (number randomised):** 18 **Inclusion criteria:** Not reported (18 "subjects with chronic (> 6 mo) stroke participated in this study") **Exclusion criteria:** Not reported **Participants:** detailed in Table **PROGRESS‐plus:** summarised in Table
Interventions	No (1) Body weight support treadmill training (n = 6) "whole task approach" "gait training on a treadmill while an overhead harness supported a percentage of the subject’s body weight (< 30%)" Length of intervention period: 4 weeks Number of sessions and length of individual sessions: 20 minutes, 3 times a week Intervention provider: not stated This intervention is not relevant to this review. (2) Proprioceptive neuromuscular facilitation (PNF) training (n = 6) "part task approach" "received PNF resisted mat activities and PNF gait training" Length of intervention period: 4 weeks Number of sessions and length of individual sessions: 20 minutes, 3 times a week Intervention provider: not stated (3) No physical rehabilitation group (n = 6) "no physical therapy interventions between pre‐ and post‐tests" Length of intervention period: no intervention Number of sessions and length of individual sessions: no intervention Intervention provider: no intervention Comparison of relevance to this review: Group (2) vs Group (3). No data relating to Group (1) have been extracted.
Outcomes	**Independence in ADL scales:** NR **Motor function scales:** NR **Measures of balance:** NR **Measures of gait velocity:** 10‐Metre Walk test **Length of stay:** NR **Adverse events:** NR **Other measures:** Stride Lengths test, Wisconsin Gait Scale **Time points when outcomes were assessed:** "all subjects received pre‐ and post testing in three clinical gait tests"
Comparison	**Amount of physical rehabilitation in groups:** differs **Categorisation of comparison:** 1. Physical rehabilitation versus no or limited physical rehabilitation (dose differs) (limited = < 50% of dose)
Funding & conflicts of interest	**Funding statement:** not reported **Conflict of interest statement:** not reported
Synthesis	**Qualitative synthesis:** no data suitable for inclusion is presented in the abstract.
Notes	Abstract only

**Table:** Stuart 2019

***Study characteristics***	
Methods	**Study aim:** "To conduct a phase II trial examining whether the community‐based Italian Adaptive Physical Activity exercise program for stroke survivors (APA‐Stroke) is safe, effective, and feasible in the United States" **Design:** parallel‐group RCT (the RCT was followed by a non‐randomised translation study in which the Howard County Office on Aging and Independence assumed day‐to‐day operations for APA‐Stroke) **Country:** USA **Study recruitment and setting:** see Table **Involvement of patients/public/professionals/others:** no
Participants	**Study population (number randomised):** 76 **Inclusion criteria:** Age ≥ 40 years ≥ 6 months post‐stroke with a residual hemiparetic gait deficit Completed all conventional inpatient and outpatient therapy Able to complete the 6MWT Able to rise unaided from a chair BBS > 36 Cleared by their personal physician to exercise. ("Patients with depression were referred to their physician for follow‐up and could be enrolled once the depression was adequately treated") **Exclusion criteria:** History of active, unstable angina Recent (<3 months) myocardial infarction Congestive heart failure or poorly controlled hypertension (> 180/100 mmHg on 2 readings separated by 5‐minute rest) Active cancer or other conditions (orthopaedic, circulatory, or chronic pain) restricting exercise Severe receptive or global aphasia with inability to follow 2‐step commands Comorbid non‐stroke neurological disorders that impair mobility Dementia or untreated clinical depression **Participants:** detailed in Table **PROGRESS‐plus:** summarised in Table
Interventions	(1) Adaptive physical activity programme (APA) "The APA‐Stroke program was designed specifically for individuals with hemiparetic gait deficits due to stroke [15]. The manualized protocol provides progressive exercise with components of gait, standing and seated coordination exercises. APA‐Stroke exercises are done both standing and seated. The standing exercises are designed to improve lower extremity function, strength, and awareness, and are performed holding onto a bar for support and balance. At the beginning of the class, walking is done around the perimeter of the room and starts with a 6‐minute stint done at each participant’s comfortable pace. Next, bar exercises are completed, weight shifts (side to side, forward and back), forward, side, and backward stepping, mezzo squats, leg extensions to back forward and side, and marching in place. After completion of the bar exercises, a series of low‐intensity exercises of the upper extremity are performed while seated in a chair. The seated exercises in the APA protocol are designed to improve upper extremity function, strength and awareness in addition to facilitating crossing of the midline. The exercises include arm rotation, trunk rotation, reaching for ankles, and sit to stand. Initially, allbar and seated exercises are done with 5 repetitions per side with rest periods as needed. They progress to 8 and then 10 repetitions per side by the end of week 9. At the end of the class, 6 minutes of walking is done with ropes placed on the floor to serve as obstacles for participants to step around. After the first month, both components of the walking program (non‐obstacle and obstacle) are lengthened by a minute each per week, until week 13 when walking time reaches 15 minutes. Time spent on other exercises is correspondingly reduced to maintain the 1‐hour time frame.The program includes a homework component. A manual and log were provided for participants. Exercises included walking, raising arms, lower trunk rotations, bridging, leg stretch, and sit to stand." (2) Sittercise "Sittercise was developed by the Howard County Office on Aging and Independence, an exercise class for frail seniors in their day programs. It is a non‐progressive exercise manualized protocol designed for general range‐of‐motion and minor strengthening for the trunk, arms and legs. The exercises are all performed in a seated position and are not designed with the intent of providing any neurotherapeutic benefit. There is no gait component and no attention is paid to improving function or awareness of any of the limbs. There are no exercises that encourage the crossing of midline and no homework component. Exercises included range of motion for upper and lower extremities, upper extremity exercises with light weights, seated marching, seated toe‐taps, hand clapping and beach ball toss" "Both interventions provided a supervised, community‐based group class, offered for 1 hour, 3 times a week, for 6 months"
Outcomes	**Independence in ADL scales:** NR **Motor function scales:** NR **Measures of balance:** Berg Balance Scale **Measures of gait velocity:** gait velocity (6‐minute walk test) **Length of stay:** NR **Adverse events:** "safety" **Other measures:** Stroke Impact Scale **Time points when outcomes were assessed:** before and after intervention
Comparison	**Amount of physical rehabilitation in groups:** equivalent **Categorisation of comparison:** 2. Physical rehabilitation versus attention control (dose of physical rehab differs; dose of intervention equivalent) (note: we have categorised the Sittercise intervention as an attention control intervention, as there were no components directly relating to mobility during standing or walking).
Funding & conflicts of interest	**Funding statement:** "authors disclosed receipt of the following financial support for the research, authorship, and/or publication of this article: This study was funded by VA Rehabilitation Research and Development (Project ID # B6329‐R)." **Conflict of interest statement:** "no potential conflicts of interest with respect to the research, authorship, and/or publication of this article"
Synthesis	**Quantitative analysis:** adverse events ‐ Analysis 3.6 **Qualitative synthesis:** other data not presented in a format suitable for inclusion in meta analysis.
Notes	

**Table:** Sun Juanjuan 2014

***Study characteristics***	
Methods	**Study aim:** to observe and evaluate the nursing effect of functional exercise combined with acupoint pressing on hemiplegia patients with cerebral infarction **Design:** parallel‐group RCT **Country:** China **Study recruitment and setting:** see Table **Involvement of patients/public/professionals/others:** no
Participants	**Study population (number randomised):** 100 **Inclusion criteria:** First stroke combined with hemiplegia **Exclusion criteria:** Severe liver and kidney disease Neuropsychiatric disease Intellectual disability **Participants:** detailed in Table **PROGRESS‐plus:** summarised in Table
Interventions	(1) Rehabilitation "... should start functional exercise as soon as possible on the premise that their vital signs are stable, their consciousness is awake, and their neurological symptoms do not worsen. 1. Assist the patient with a limb massage every 4 hours, and help the hemiplegic patient with joint extension, abduction and internal rotation every half an hour. The massage activities are from passive to active, from large joints to small joints. Holding a bouncy ball in hand to exercise hand muscle strength, and massaging and moving the limbs of hemiplegic patients is to prevent joint contractures and muscle atrophy. 2. Help the patient to change the body position on the bed, including bed translation, turning over, changing the body position, etc., to promote the recovery of the patient's muscle strength. 3. Assist the patients to perform short sitting, long sitting, standing, assisted walking, and independent walking in sequence. The specific method of acupoint pressing is as follows: Press the corresponding acupoints on the affected side of the patient twice a day in the morning and evening, and press each acupuncture point for 3 to 4 minutes. Such as Biguan, Liangqiu, Futu, Zusanli, Yanglingquan, Xuanzhong, Sanyinjiao, Chengshan, Xiexi, Kunlun and Taichong. If the patient has upper limb dysfunction, it is necessary to press the corresponding acupoints of the upper limb on the affected side, such as Jianjing, Fengchi, Quchi, Chize, Hegu and Neiguan. Each compression ends with the patient feeling sore." (2) No physical rehabilitation "both groups were given drug treatment and routine nursing, including medication nursing, diet nursing, and psychological counseling‐level rehabilitation guidance."
Outcomes	**Independence in ADL scales:** NR **Motor function scales:** Fugl Meyer assessment **Measures of balance:** NR **Measures of gait velocity:** NR **Length of stay:** NR **Adverse events:** NR **Other measures:** muscle strength **Time points when outcomes were assessed:** before and after treatment
Comparison	**Amount of physical rehabilitation in groups:** differs **Categorisation of comparison:** 1. Physical rehabilitation versus no or limited physical rehabilitation (dose differs) (limited = < 50% of dose)
Funding & conflicts of interest	**Funding statement:** not reported **Conflict of interest statement:** not reported
Synthesis	**Qualitative synthesis:** no motor results presented
Notes	This study was published in Chinese and translated into English using Google Translate.

**Table:** SunRISe 2021

***Study characteristics***	
Methods	**Study aim:** "to assess feasibility and preliminary effectiveness of a home‐based semi‐supervised physiotherapy intervention to promote post‐stroke mobility in a low resource setting" **Design:** parallel‐group RCT **Country:** Republic of Suriname **Study recruitment and setting:** see Table **Involvement of patients/public/professionals/others:** no
Participants	**Study population (number randomised):** 30 **Inclusion criteria:** Chronic stroke patients with stroke onset > 6 months Medical clearance to participate in a moderately intense exercise programme Living at home Not undertaking regular exercise or physiotherapy Mild‐moderate stroke deficit (Fugl Meyer score 27 to 90 for upper and lower limb) FAC ≥ 3 Able to understand measurement procedures (MMSE > 24) **Exclusion criteria:** Any contraindications to exercise testing Neurological deficits leading to disability **Participants:** detailed in Table **PROGRESS‐plus:** summarised in Table
Interventions	(1) Home‐based physiotherapy "Overall, the intervention employed a holistic approach bringing together physical fitness improving exercise, functional exercise, upper limb exercise as well as patient education according to the needs of the patients (Table1). The home‐based physiotherapy program included stair climbing, sit‐to‐stand exercise and walking. Upper extremity exercises were incorporated in the start‐up and ending phases of each training session and consisted of proprioceptive neuromuscular facilitation techniques and mobility exercises. All treatment sessions between T0 and T4 (supervised phase, 3 times/week, 70minutes/session) were supervised by a physiotherapist. Between T4 and T8 (tele‐coaching phase), the instructions were to continue the same individually tailored program without supervision, with help from the participants’ usual caregiver if necessary..." "home‐based, semi‐supervised physiotherapy program" (2) Usual care "received usual care. At this moment usual care for chronic stroke patients in Suriname consists of ten physiotherapy sessions (with only 54% of the costs per session are refundable) a year for people insured by the largest governmental health insurance company. So, by the time people after stroke are in the chronic phase after stroke, they do not have any therapy sessions left for that year. Moreover, if no physiotherapy is requested by the patient, none is given which is most often the case."
Outcomes	**Independence in ADL scales:** NR **Motor function scales:** NR **Measures of balance:** Berg Balance Scale **Measures of gait velocity:** 6‐minute walk test **Length of stay:** NR **Adverse events:** adverse events **Other measures:** patient satisfaction, Disabilities of the Arm, Shoulder and Hand (DASH) score, hand grip strength, SCI exercise self‐efficacy scale **Time points when outcomes were assessed:** baseline and at 8 weeks
Comparison	**Amount of physical rehabilitation in groups:** differs **Categorisation of comparison:** 1. Physical rehabilitation versus no or limited physical rehabilitation (dose differs) (limited = < 50% of dose)
Funding & conflicts of interest	**Funding statement:** "authors received no specific funding for this work" **Conflict of interest statement:** "authors have declared that no competing interests exist"
Synthesis	**Quantitative synthesis:**Analysis 1.3; Analysis 1.4
Notes	

**Table:** Tang 2009

***Study characteristics***	
Methods	**Study aim:** "to probe into influence of sensory function training on motor function of cerebral apoplexy complicated with hemiplegia" **Design:** parallel‐group RCT **Country:** China **Study recruitment and setting:** see Table **Involvement of patients/public/professionals/others:** no
Participants	**Study population (number randomised):** 70 **Inclusion criteria:** Met the diagnostic criteria of the 4th National Conference on Cerebral Vascular Disease classification guidelines First ever stroke as confirmed by CT or MRI scan **Exclusion criteria:** Obvious barriers to understanding and severe pathological changes to organs **Participants:** detailed in Table **PROGRESS‐plus:** summarised in Table
Interventions	(1) Sensory function training (n = 35) "Both groups received standard pharmacotherapy and motor function training using mainly Bobath method: including positioning of non‐affected limbs and training on bed; transfer training; stepping training and gait re‐education; occupational training and ADL training. In addition, the observation group also received sensory function training. Superficial sensation training: (i) Using tip of large‐headed pin with constant force to lightly poke the skin on affected side, and compare with non‐affected side; (ii) Using cotton swab to lightly touch the affected side’s skin and mucosa; (iii) Using towel soaked in hot water (40°C – 50°C) and cold water (5°C – 10°C) to wipe and train sensation of temperature; (iv) During the early period especially during flaccid paralysis period, light tapping, hitting, light touching, rapid brushing, etc, performed on affected limbs. Training could be done initially with eyes closed, but if there was obvious difficulty, could be trained with eyes opened instead, with training done with eyes closed after there is improvement, such that there is repeated training, following eyes closed ‐> eyes opened ‐> eyes closed sequence" "Deep sensation training: (i) Maintenance of non‐affected limb position during early period, with appropriate increase in time spent lying on affected side, giving extra protection of affected limbs when changing position; (ii) Using fingers, therapist lightly held patient’s affected side’s finger or toe to perform passive ranging exercises, or placed affected limbs in a particular position, to allow patient to feel the position of his limbs, while staying motionless, training repeatedly until the patient himself could complete certain movements with affected limb, being especially useful for low muscle tone and poor control of lower limbs; (iii) While sitting or standing, paying attention to weight borne by affected limbs, and while performing active or passive ranging exercises, could also use an elastic strap to wrap and place pressure on joint being moved" "Touch sensation training: once patient had recovered sensation on fingers, this training could immediately commence. (i) Patient closed eyes and used touch to identify common objects such as key, pen, toothbrush, button, etc, and if unable to identify, could also allow touching with eyes opened or using non‐affected hand to touch; (ii) Plastic pieces, paper, cloth and fur, etc, were mixed together, for patient to identify using touch with eyes closed, and if there is error in identification, could similarly use non‐affected hand or open eyes; (iii) Patient showed a picture, and then asked to find a similar object in a black box" "Balance training: using balance assessment training system, based on patient’s condition, either sitting or standing position was used, initially using corresponding pressure sensor, patient focused on the display unit during training, adjusted their own position according to change in display lights on display unit, to undergo training on maintenance of centre of gravity, shifting of centre of gravity, single‐leg weight‐bearing, etc. After becoming stable with eyes opened, this training could also be performed with eyes closed" Length of intervention period: 8 weeks Number of sessions and length of individual sessions: 1 time a day, each time 45 minutes Intervention provider: therapist (2) Conventional physiotherapy (n = 35) "Control group used standard pharmacotherapy and motor function training using mainly Bobath method: including positioning of non‐affected limbs and training on bed; changing of position training; ambulatory training and gait re‐education; occupational training and ADL training" Length of intervention period: not stated Number of sessions and length of individual sessions: not stated Intervention provider: not stated
Outcomes	**Independence in ADL scales:** NR **Motor function scales:** Fugl‐Meyer Assessment (sensory, motor) **Measures of balance:** NR **Measures of gait velocity:** NR **Length of stay:** NR **Adverse events:** NR **Other measures:** NR **Time points when outcomes were assessed:** before and after 8 weeks of intervention
Comparison	**Amount of physical rehabilitation in groups:** differs **Categorisation of comparison:** 5. Additional therapy + usual therapy versus usual therapy (dose differs)
Funding & conflicts of interest	**Funding statement:** not reported **Conflict of interest statement:** not reported
Synthesis	**Quantitative synthesis:**Analysis 5.2
Notes	Original study translated from Chinese to English by a member of our team (PLC).

**Table:** Tang Yao 2015

***Study characteristics***	
Methods	**Study aim:** to explore the effect of three‐level rehabilitation training in improving the motor and cognitive dysfunction of stroke patients, and to provide scientific guidance for clinically promoting the functional recovery of stroke patients **Design:** parallel‐group RCT **Country:** China **Study recruitment and setting:** see Table **Involvement of patients/public/professionals/others:** no
Participants	**Study population (number randomised):** 100 **Inclusion criteria:** First ever stroke Met diagnostic criteria for stroke Diagnosed using CT Normal cognition and conscious at the time of onset Limb dysfunction Medically stable for a week **Exclusion criteria:** Participants with the following health conditions were excluded: liver, lung, kidney, heart and other important organ failure Any other health condition/disease that caused dysfunction Serious deterioration of the disease **Participants:** detailed in Table **PROGRESS‐plus:** summarised in Table
Interventions	(1) Three‐stage rehabilitation "Standardised three‐stage approach. Specifically, First‐level rehabilitation: onset ‐ not one month after the onset, early bedside rehabilitation treatment is given, including passive limb training waist and abdominal muscle training, deep breathing training, standing training and recumbent position training, sitting balance training and non‐paretic limb active activity training, etc. Training one session/day, 45 minutes/session Secondary rehabilitation: delivered not three months after onset. Aim is to improve the stroke survivor's walking function training. Generally, standing training, standing on one leg, standing balance training and walking training can be carried out. Training is one session/day, 30 ‐ 45 minutes/session. Tertiary rehabilitation: main training content is to train stroke survivors to eat, dress, wash, clean‐up, and other basic activities. The training is delivered in one session/day, 30‐45 minutes/session. In addition, cognitive dysfunction rehabilitation training is also carried out for each stroke survivor according to the needs/characteristics of each. The training content mainly includes memory logic function training, digital exercise, object classification, colour building block arrangement, problem solving etc. When performing cognitive impairment rehabilitation training for stroke survivors, it is necessary to pay special attention to: if the stroke survivor has obstacles such as viewing and hearing, it is necessary to equip the stroke survivor with relevant tools, and to help the stroke survivor solve psychological obstacles Finally, if any of the following situations occur during rehabilitation training, training should stop: stroke survivors with severe arrhythmia and palpitations during training, stroke survivors with myocardial infarction within 1 month." (2) No physical rehabilitation Received usual treatment and conventional nursing care. Usual rehabilitation guidance includes ensuring respiratory tract is clear, body position management including maintaining position of the healthy limbs, and maintaining range of motion of the joints, bowel management and nutrition management.
Outcomes	**Independence in ADL scales:** NR **Motor function scales:** NR **Measures of balance:** NR **Measures of gait velocity:** NR **Length of stay:** NR **Adverse events:** NR **Other measures:** Functional Comprehensive Assessment **Time points when outcomes were assessed:** before and after treatment
Comparison	**Amount of physical rehabilitation in groups:** differs **Categorisation of comparison:** 1. Physical rehabilitation versus no or limited physical rehabilitation (dose differs) (limited = < 50% of dose)
Funding & conflicts of interest	**Funding statement:** not reported **Conflict of interest statement:** not reported
Synthesis	**Qualitative synthesis:** no relevant outcomes assessed
Notes	This study was published in Chinese and relevant sections translated into English by our author team (PLC).

**Table:** Teixeira‐Salmela 1999

***Study characteristics***	
Methods	**Study aim:** "to evaluate the impact of a program of muscle strengthening and physical conditioning on impairment and disability in chronic stroke subjects" **Design:** parallel‐group RCT **Country:** Canada **Study recruitment and setting:** see Table **Involvement of patients/public/professionals/others:** no
Participants	**Study population (number randomised):** 13 **Inclusion criteria:** Unilateral stroke with residual lower limb weakness and/or spasticity Independently ambulatory for 15 mins (with/without assistive devices) Activity tolerance of 45 mins (with rest intervals) No 'comprehensive' aphasia **Exclusion criteria:** Non‐stroke related disability **Participants:** detailed in Table **PROGRESS‐plus:** summarised in Table
Interventions	(1) Training programme "The training program consisted of supervised exercise sessions by an exercise physiologist and physiotherapist conducted 3 mornings a weeks for 10 weeks. Each session lasted from 60 to 90 minutes and all sessions were accompanied by appropriate music relevant to the participants' age". Training included: warm up, aerobic exercises, strength training, cool down. Strength training "was an adaptation of standardised rehabilitation principles of progressive‐resistance training, utilising isometric, concentric and eccentric muscle contractions..." (2) No treatment "received no intervention for 10 weeks"
Outcomes	**Independence in ADL scales:** NR **Motor function scales:** NR **Measures of balance:** NR **Measures of gait velocity:** gait speed **Length of stay:** NR **Adverse events:** NR **Other measures:** stair climbing, Human Activity Profile, Nottingham Health Profile, lower extremity muscle strength, muscle tone **Time points when outcomes were assessed:** NR
Comparison	**Amount of physical rehabilitation in groups:** differs **Categorisation of comparison:** 1. Physical rehabilitation versus no or limited physical rehabilitation (dose differs) (limited = < 50% of dose)
Funding & conflicts of interest	**Funding statement:** "supported by grant ST2714 from the Heart and Stroke Foundation of Ontario. Dr Teixeira‐Salmela was supported by a doctoral studentship from CNPQ (Brazilian Government Agency) and Dr Nadeau by fellowships from MRC and REPAR of Quebec" **Conflict of interest statement:** not reported
Synthesis	**Quantitative synthesis:**Analysis 1.4
Notes	

**Table:** Thaut 2007

***Study characteristics***	
Methods	**Study aim:** "to examine the clinical efficacy of rhythmic auditory stimulation (RAS) comparing 3 weeks of RAS against the NDT / Bobath method, which is one of the most widely used gait therapies" **Design:** parallel‐group RCT **Country:** Germany and USA **Study recruitment and setting:** see Table **Involvement of patients/public/professionals/others:** no
Participants	**Study population (number randomised):** 78 **Inclusion criteria:** Not reported ("Patients entered the study within 4 weeks of onset, as soon as they could complete 5 stride cycles with handheld assistance by the therapist, that is, with no more than support of the forearm, wrist, and elbow at approximately 90 degrees of elbow flexion on the nonparetic side. Handheld assistance was available to all patients throughout training when needed") **Exclusion criteria:** Not reported **Participants:** detailed in Table **PROGRESS‐plus:** summarised in Table
Interventions	(1) Task‐specific training (rhythmic auditory stimulation) (n = 43) "RAS training followed established protocols using a metronome and specifically prepared music tapes in digital MIDI format to ensure temporal precision and tempo stability as well as full capacity for frequency modulation of the stimulus based on patient needs. After an initial cadence assessment, cuing frequencies were matched to the gait cadence for the first quarter of the session. During the second quarter, cue frequencies were increased in 5% increments as kinematically indicated without compromising postural and dynamic stability. During the third quarter, adaptive gait patterns, for example, ramp or step walking, were practiced. The last quarter was spent fading the cues intermittently to train for independent carryover" Length of intervention period: 3 weeks Number of sessions and length of individual sessions: "gait training daily for 30 minutes, 5 times per week" Intervention provider: "Four gait therapists for each group conducted the training to ensure consistency in training protocols and procedures. Each center had its own independently trained pool of therapists" (2) Neurodevelopmental therapy (NDT)/Bobath‐based training (n = 35) "control group trained the same amount of time and distance, following NDT and Bobath principles as well as using similar instructions about gait parameters to practice, but without rhythmic auditory cuing" Length of intervention period: 3 weeks Number of sessions and length of individual sessions: "gait training daily for 30 minutes, 5 times per week" Intervention provider: "Four gait therapists for each group conducted the training to ensure consistency in training protocols and procedures. Each center had its own independently trained pool of therapists".
Outcomes	**Independence in ADL scales:** Barthel Index **Motor function scales:** Fugl‐Meyer Assessment **Measures of balance:** NR **Measures of gait velocity:** gait velocity **Length of stay:** NR **Adverse events:** NR **Other measures:** stride length, cadence, and symmetry **Time points when outcomes were assessed:** "All patients were tested 1 day before the training sessions started and 1 day after the last training session"
Comparison	**Amount of physical rehabilitation in groups:** equivalent **Categorisation of comparison:** 3.(i) Comparison of different approaches (dose equivalent; with no other physical rehab)
Funding & conflicts of interest	**Funding statement:** not reported **Conflict of interest statement:** not reported
Synthesis	**Quantitative synthesis:**Analysis 7.10
Notes	"All available participant data after removing dropout participants were analyzed in an intention‐to‐treat analysis"

**Table:** Torres‐Arreola 2009

***Study characteristics***	
Methods	**Study aim:** "to evaluate the effectiveness of the early rehabilitation at hospital and its continuity at home provided by nurses, on the functional recovery of basic and social activities in stroke patients compared with conventional care" **Design:** parallel‐group RCT **Country:** Mexico **Study recruitment and setting:** see Table **Involvement of patients/public/professionals/others:** no
Participants	**Study population (number randomised):** 110 **Inclusion criteria:** Age 40 years+ Diagnosis of first stroke Ischaemic stroke Mild and moderate neurological damage **Exclusion criteria:** Not reported **Participants:** detailed in Table **PROGRESS‐plus:** summarised in Table
Interventions	(1) Rehabilitation (Strategy 1, S1) (n = 59) "The team of two trained nurses (see notes) applied one of the two rehabilitation strategies to each patient, beginning 72 hours after initial hospitalisation, when the patient’s clinical condition had been stabilised" "The first strategy (S1) included two parts: education and physiotherapy. The education portion consisted of providing information on general care such as feeding, hygiene and mobilisation through a Manual of Physical Rehabilitation for the patient with Stroke and also verbally and through a practical rehabilitation training session provided by the nurse to the caregiver. This was an individual plan of daily rehabilitation according to the stage of Brunnstrom’s classification that the caregiver had to perform. The Manual was drafted by the research group, especially for this study. The physiotherapy part of the strategy was designed and applied based on the basal condition of the patients and their stage of physical recovery. The latter was defined using the Brunnstrom scale (I–III), which reflects voluntary activity, movement, coordination, postural stability, muscular tonicity and sphincter control (further details supplied in Table 1). The physiotherapy provided by nurse was divided into three phases. The intensive phase consisted of daily physical therapy initiated at the hospital and continued for two weeks postdischarge through in‐home visits. The intermediate phase consisted of bi‐weekly in‐home visits during the following two weeks (weeks three and four postdischarge). Finally, the support phase consisted of weekly in‐home visits during the following three months, up to four months postdischarge." Length of intervention period: 4 months Number of sessions and length of individual sessions: "average number of visits for the S1 group was 15 and the average duration of each visit had to be about 90 minutes" Intervention provider: nurse (see notes) and caregiver (2) No physical rehabilitation (Strategy two, S2) (n = 51) "The second strategy (S2) consisted of education alone. The information and individual plan of rehabilitation provided by the nurse to the patient and caregiver were the same as in the S1 group. This intervention also began at the hospital and was continued in‐home with weekly visits" Length of intervention period: 4 months Number of sessions and length of individual sessions: "The average number of visits in this group was eight and the average duration of each visit was approximately 45 minutes" Intervention provider: nurse and caregiver
Outcomes	**Independence in ADL scales:** Barthel Index **Motor function scales:** NR **Measures of balance:** NR **Measures of gait velocity:** NR **Length of stay:** NR **Adverse events:** NR **Other measures:** Frenchay Activities Index, MMSE, Canadian Neurological Scale **Time points when outcomes were assessed:** "Before randomisation, the patient and/or relatives were interviewed in the hospital to evaluate the patient’s basic ADL (Barthel index) and social activities (Frenchay index), which were used as the main outcome variables" "Follow‐up measurements of the basic and social ADL and the cognitive state of the stroke patient were obtained at one, three and six months postdischarge"
Comparison	**Amount of physical rehabilitation in groups:** differs **Categorisation of comparison:** 1. Physical rehabilitation versus no or limited physical rehabilitation (dose differs) (limited = < 50% of dose)
Funding & conflicts of interest	**Funding statement:** "supported by grants from the ResearchPromotion Fund of the Mexican Institute of Social Security(FOFOI) and the National Council of Science and Technol‐ogy (CONACYT‐2002‐c01‐40686/A‐1)." **Conflict of interest statement:** not reported
Synthesis	**Quantitative analysis:**Analysis 1.5 **Qualitative synthesis:** data reported not suitable for inclusion in analyses
Notes	Further details on intervention/intervention provider: "Two general nurses received two months of theoretical (80 hours) and practical training (80 hours) in using the intervention strategy, as taught by a physical medicine and rehabilitation doctor, a physiotherapist and a specialised nurse. The theoretical part included information about stroke, general aspects of physical rehabilitation, stages of recovery and physiotherapy according to Brunnstrom’s stage. The practical part was performed with healthy volunteers and later with stroke patients in the hospital. In addition, as a product of this phase of the study, the Stroke Rehabilitation Manual for Nurses was elaborated; this had to be used in the rehabilitation strategy. The physical medicine and rehabilitation doctor and physiotherapist established the minimal criteria to consider that the nurse was sufficiently trained to perform physiotherapy"

**Table:** Tyson 2015

***Study characteristics***	
Methods	**Study aim:** "to assess the feasibility and acceptability of two patient‐led therapies during the acute stages of stroke care: mirror therapy for the upper limb and lower‐limb exercises for the lower limb" **Design:** parallel‐group RCT **Country:** United Kingdom **Study recruitment and setting:** see Table **Involvement of patients/public/professionals/others:** no
Participants	**Study population (number randomised):** 94 **Inclusion criteria:** Experienced a stroke at least 1 week previously Upper‐ and lower‐limb weakness that limited activity No premorbid conditions limiting upper‐ or lower‐limb function Sufficient cognitive and communication skills to provide informed consent **Exclusion criteria:** Not reported **Participants:** detailed in Table **PROGRESS‐plus:** summarised in Table
Interventions	(1) Patient‐led mirror therapy Upper limb mirror therapy ‐ "fingers, wrist, and elbow movements; reaching; and at the highest level, functional activities were exercised." (2) Patient‐led lower‐limb exercise "The lower‐limb exercises involved the ankles, knees, and hips. Level 1 involved flexion, extension, abduction, and adduction movements with the limb fully supported. Level 2 involved multiplanar multiple joint movements, some against gravity. Level 3 involved movements against gravity. Level 4 involved functional activities such as grasping and moving objects (for the upper limb) and standing up and sitting down (for the lower limb). "All participants received usual care."
Outcomes	**Independence in ADL scales:** NR **Motor function scales:** Rivermead Mobility Index **Measures of balance:** Brunel Balance Assessment **Measures of gait velocity:** NR **Length of stay:** NR **Adverse events:** NR **Other measures:** Motricity Index, grip strength, Rivermead Assessment of Sensory Perception, Ashworth scale, Star cancellation test, Box and block test, Action Research Arm Test **Time points when outcomes were assessed:** baseline and after treatment (8 weeks)
Comparison	**Amount of physical rehabilitation in groups:** differs **Categorisation of comparison:** 2. Physical rehabilitation versus attention control (dose of physical rehab differs; dose of intervention equivalent)
Funding & conflicts of interest	**Funding statement:** "disclosed receipt of the following financial support for the research, authorship, and/or publication of this article: This article presents independent research funded by the NationalInstitute for Health Research under its Research for Patient Benefit (RfPB) Programme (Grant Reference Number PB‐PG‐1208‐18077)" **Conflict of interest statement:** "authors declared no potential conflicts of interest with respect to the research, authorship, and/or publication of this article"
Synthesis	**Qualitative synthesis:** results data not presented in a format suitable for quantitative analysis.
Notes	Results presented as changes from baseline.

**Table:** Vahlberg 2017

***Study characteristics***	
Methods	**Study aim:** to evaluate the effects of progressive resistance and balance (PRB) exercises in individuals with chronic stroke **Design:** parallel‐group RCT **Country:** Sweden **Study recruitment and setting:** see Table **Involvement of patients/public/professionals/others:** no
Participants	**Study population (number randomised):** 67* (see notes section) **Inclusion criteria:** Verified stroke of any type within the previous 1 to 3 years Able to walk ≥ 10 m One of the following: Not walking outdoors on at least 5 days/week Low fall‐related self‐efficacy (Falls Efficacy Scale‐Swedish version < 115 points) Balance difficulties (BBS ≤ 52 points) Repeated falls in previous 12 months No contraindications to the proposed exercise Medically stable conditions (as assessed by their general practitioner) **Exclusion criteria:** Cognitive deficits (Short Portable Mental Status Questionnaire < 7 points) Dementia Severe communication problems (assessed through medical records or revealed during examination) Systolic blood pressure > 180 mmHg. **Participants:** detailed in Table **PROGRESS‐plus:** summarised in Table
Interventions	(1) Progressive resistance and balance exercise "Training was performed in circuit classes that were conducted twice weekly over a 3‐month period and consisted of different work stations with functional exercises that involved the major muscle groups (particularly lower extremity function; the intervention included the following sessions: a warm‐up that consisted of stationary cycling or walking (10 minutes), a circuit class (approximately 45 minutes) and a motivational session that included discussions about issues and personal goals that are related to physical activity (20 minutes). The first seven exercises in the circuit were static and dynamic‐balance exercises in combination with lower‐limb strength exercises. The next seven were dynamic‐balance exercises that were performed while walking. The ratio of the trainers (one physiotherapist and one assistant) to participants was two to seven. One individually tailored at home exercise to be performed daily was also provided to each participant to implement in their daily life and support the progressive resistance and balance exercise program". (2) Control (no treatment) "The individuals who were allocated to the control group were encouraged to continue their regular activities and were not restricted from participating in ordinary physical activities and rehabilitation programs."
Outcomes	**Independence in ADL scales:** NR **Motor function scales:** NR **Measures of balance:** Berg Balance Scale **Measures of gait velocity:** 6‐minute walk test, 10‐m walk test **Length of stay:** NR **Adverse events:** NR **Other measures:** Short Physical Performance Battery (SPPB), PASE (physical activities of older adults), EQ‐5D, Geriatric Depression Scale, fall‐related self‐efficacy **Time points when outcomes were assessed:** pre‐ and post‐intervention, and at 6 and 15 months
Comparison	**Amount of physical rehabilitation in groups:** differs **Categorisation of comparison:** 1. Physical rehabilitation versus no or limited physical rehabilitation (dose differs) (limited = < 50% of dose)
Funding & conflicts of interest	**Funding statement:** "supported by grants from the Medical Faculty at Uppsala University, STROKE‐Riksforbundet and the Uppsala County Council and municipality in Sweden" **Conflict of interest statement:** "no conflicts of interest"
Synthesis	**Qualitative synthesis:** data not suitable for analyses (presented as medians and IQRs, and/or mean changes).
Notes	There are two journal publications which both report different participant numbers and recruitment dates (n = 67; October 2009 to April 2011; and n = 43 February 2008 to April 2010). Publications are by the same team, describe the same intervention and inclusion criteria. There are some differences in reported outcomes. One paper (n = 43) reports only 3‐month outcome data, while the other (n = 67) reports 3, 6, and 15 month data.

**Table:** Verma 2011

***Study characteristics***	
Methods	**Study aim:** "to evaluate the effectiveness of the task‐oriented circuit class training (TOCCT) with motor imagery (MI) on the gait during the subacute phase after a stroke" **Design:** parallel‐group RCT **Country:** India **Study recruitment and setting:** see Table **Involvement of patients/public/professionals/others:** no
Participants	**Study population (number randomised):** 30 **Inclusion criteria:** First episode of unilateral stroke with hemiparesis during the last month Functional ambulation classification level II and above Able to understand instructions (Hindi Mental State Examination (HMSE0 > 24) Ambulatory before stroke Able to cope with the intensive training programme Mental imaging (Movement Imagery Questionnaire—revised second version (MIQ‐RS) ≥ 25) and National Institutes of Health Stroke Scale (NIHSS) score < 14 Exclusion criteria: History of any other neurological pathology such as Parkinson's disease and epilepsy Conditions affecting balance Neglect Dementia Impaired vision Impaired conscious level Concomitant medical illness Musculoskeletal conditions affecting lower limbs Cardiovascular instability (resting systolic blood pressure > 200 mm Hg and resting diastolic blood pressure > 100 mm Hg) and/or serious cardiac conditions (hospitalisation for heart disease within 3 months active angina, serious cardiac arrhythmias, hypertrophic cardiomyopathy, severe aortic stenosis) **Participants:** detailed in Table **PROGRESS‐plus:** summarised in Table
Interventions	(1) Task‐specific training (n = 15) "The program included different workstations and was intended to improve the meaningful tasks related to walking competency, such as balance control, stair walking, turning, transfers, and speed walking. Further, each session consisted of a continuous practice of standing and walking‐related tasks on specified workstations with a minimal break". Circuit training "Motor imagery comprised imagining walking abilities and tasks related to a real‐life situation" Length of intervention period: 2 weeks Number of sessions and length of individual sessions: "15 minutes of motor imagery followed by 25 minutes of task‐oriented circuit class training for a total of 40 minutes, 7 days per week for 2 weeks (14 sessions)" Intervention provider: "Task‐oriented circuit class training was provided to groups comprising up to 4 patients at any one time with a physiotherapist or occupational therapist for supervision" (2) Bobath (n = 15) "Conventional post stroke lower extremity rehabilitation program based on the Bobath’s neurodevelopmental technique" Length of intervention period: 2 weeks Number of sessions and length of individual sessions: "The control group program was matched for duration, number, and frequency of the sessions with the experimental group program" Intervention provider: not stated
Outcomes	**Independence in ADL scales:** Barthel Index **Motor function scales:** NR **Measures of balance:** NR **Measures of gait velocity:** 10‐Metre Walk Test **Length of stay:** NR **Adverse events:** NR **Other measures:** Functional Ambulation Classification, Rivermead Visual Gait Assessment, 10‐Metre Walk test, Six‐Minute Walk test, step length, stride length, step width and cadence **Time points when outcomes were assessed:** time points when outcomes were assessed: "All the participants were assessed using the outcome measures at baseline (pre‐intervention), week 2 (post‐intervention) except for Barthel Index (BI), and at week 6 (follow‐up)"
Comparison	**Amount of physical rehabilitation in groups:** equivalent **Categorisation of comparison:** 3.(i) Comparison of different approaches (dose equivalent; with no other physical rehab)
Funding & conflicts of interest	**Funding statement:** not reported **Conflict of interest statement:** not reported
Synthesis	**Quantitative synthesis:**Analysis 7.4; Analysis 8.1; Analysis 8.4; Analysis 8.9
Notes	

**Table:** Wade 1992

***Study characteristics***	
Methods	**Study aim:** "to determine whether the intervention of a physiotherapist improved mobility inpatients seen more than one year after stroke" **Design:** cross‐over RCT **Country:** UK **Study recruitment and setting:** see Table **Involvement of patients/public/professionals/others:** no
Participants	**Study population (number randomised):** 94 **Inclusion criteria:** First stroke > 1 year previously Mobility problems (fall within last 3 months, used a walking aid, limited mobility or slow gait speed) **Exclusion criteria:** Not reported **Participants:** detailed in Table **PROGRESS‐plus:** summarised in Table
Interventions	(1) Home‐based rehabilitation (n = 49) Community physiotherapy "'Problem solving' approach: patients were assessed with particular reference to their mobility, and problem areas were identified. Realistic, achievable goals were discussed with the patient and carers and then the physiotherapist intervened if required" Re‐education of abnormal components of gait Practice walking inside and outside For standing balance: exercises to stimulate reactions, obstacle courses, practice on uneven surfaces Re‐education of sitting to standing Equipment: removal, provision, maintenance, adjustment Activities of daily living: advice, referral to community occupational therapist Demonstrate participant's ability to participant/carer Graduated exercise programme Length of intervention period: not stated Number of sessions and length of individual sessions: range 1 to 11 visits, mean number of visits = 4 (standard deviation 2.5). Time (including travel and administration) ranged from one hour 10 minutes to 3 hours 10 minutes Intervention provider: physiotherapist and occupational therapist (2) No physical rehabilitation (n = 45) Length of intervention period: no intervention Number of sessions and length of individual sessions: no intervention Intervention provider: no intervention
Outcomes	**Independence in ADL scales:** Barthel Index, Frenchay Activities Index, Nottingham EADL Scale **Motor function scales:** Rivermead Mobility Assessment **Measures of balance:** NR **Measures of gait velocity:** gait speed **Length of stay:** NR **Adverse events:** NR **Other measures:** NR **Time points when outcomes were assessed:** "Once accepted into the trial, each patient was assessed by an independent (non‐treating) physiotherapist immediately, one to six weeks later, and then about three, six, and nine months after the second assessment"
Comparison	**Amount of physical rehabilitation in groups:** differs **Categorisation of comparison:** 1. Physical rehabilitation versus no or limited physical rehabilitation (dose differs) (limited = < 50% of dose)
Funding & conflicts of interest	**Funding statement:** "funded by the Medical Research Council" **Conflict of interest statement:** not reported
Synthesis	**Quantitative synthesis:**Analysis 1.1; Analysis 1.2; Analysis 1.4; Analysis 2.1; Analysis 2.2; Analysis 2.4
Notes	Cross‐over design: participants in the control group received treatment after 3‐month assessment. This study was initially excluded from this review, as the review authors assessed, based on the abstract, that this study explored timing of intervention. Comments from peer reviewers for the 2007 version led to the inclusion of this trial.

**Table:** Wang 2004a

***Study characteristics***	
Methods	**Study aim:** "to observe the influence of exercise therapy on the recovery of motor function of limbs and its safety in stroke patients, and explore whether somatosensory evoked potential (SEP) can be used as an index for evaluating the recovery of motor function of limbs in stroke patients" **Design:** parallel‐group RCT **Country:** China **Study recruitment and setting:** see Table **Involvement of patients/public/professionals/others:** no
Participants	**Study population (number randomised):** 105 **Inclusion criteria:** Aged between 42 and 78 years old Commenced therapy (approximately) within 3 days of stroke onset Met the 1995 Fourth National Conference on Cerebral Vascular Disease classification guidelines Stroke as confirmed by CT or MRI scan **Exclusion criteria:** Stroke onset > 1 week Severe impaired cognitive and/or speech issues **Participants:** detailed in Table **PROGRESS‐plus:** summarised in Table
Interventions	(1) Rehabilitation (n = 70 before dropout; n = 66) (data available: see Table 5) "Both groups received conventional treatment during the critical period, with treatment group commencing early‐stage physical rehabilitation once medically stable. Based on the condition of each patient, a treatment plan was developed prior to treatment. Treatment method utilised exercise therapy, including techniques and equipment, locations included bedside and treatment room, treatment was conducted by therapist. Mainly utilised motor relearning methods, including: (i) Positioning of healthy limbs. (ii) Passive ranging exercises on joints of limbs on affected side. (iii) Functional training of upper limbs, including stimulation of muscle motion as well as training of control of extension, separation of fingers, prevention of contracture, etc. (iv) Training of rolling on bed, as well as training of lying to bedside sitting, training of bedside sitting balance, sit‐to‐stand training. (v) Upright training (bed). (vi) Bedside standing and sitting training as well as bedside standing balance training. (vii) Gait training. (viii) Weight‐bearing and weight‐assisted training, etc. Throughout entire treatment period, emphasis on correct training posture and relaxation training of spastic muscles, anti‐spasticity techniques and muscle training of non‐spastic muscles. Family members were taught the correct complementary training and care methods, while concurrently focusing on psychological treatment, during the course of the training, frequently use language that will boost the patient’s confidence in recovery" Length of intervention period: 30 days Number of sessions and length of individual sessions: 1 to 2/d, 45 minutes/session, with exercise time and intensity reduced as appropriate if participant lacked the capability Intervention provider: therapist (2) No physical rehabilitation group (n = 35 before dropout, n = 32 data available: see Table 5) "Received conventional treatment during the critical period but did not receive any rehabilitation treatment" Length of intervention period: no intervention Number of sessions and length of individual sessions: no intervention Intervention provider: no intervention
Outcomes	**Independence in ADL scales:** NR **Motor function scales:** Fugl‐Meyer Assessment (upper limb, lower limb) **Measures of balance:** NR **Measures of gait velocity:** NR **Length of stay:** NR **Adverse events:** NR **Other measures:** somatosensory evoked potential (SEP) **Time points when outcomes were assessed:** "Each patient, within 2 days of being medically stable, would undergo 1st assessment, and 30 days later would undergo the 2nd assessment"
Comparison	**Amount of physical rehabilitation in groups:** differs **Categorisation of comparison:** 1. Physical rehabilitation versus no or limited physical rehabilitation (dose differs) (limited = < 50% of dose)
Funding & conflicts of interest	**Funding statement:** "supported by the Bureau of Health of Guangxi Zhuang Autonomous Region, No 29951" **Conflict of interest statement:** not reported
Synthesis	**Quantitative synthesis:**Analysis 1.2
Notes	Original study translated from Chinese to English by a member of our team (PLC).

**Table:** Wang 2004b

***Study characteristics***	
Methods	**Study aim:** "to observe and analyse the effect of neural facilitation combined with low frequency pulse current in treating the spasticity in hemiplegic patients within 4 months after the attack of stroke, and to explore its mechanism" **Design:** parallel‐group RCT **Country:** China **Study recruitment and setting:** see Table **Involvement of patients/public/professionals/others:** no
Participants	**Study population (number randomised):** 50 **Inclusion criteria:** Met the 1995 Fourth National Conference on Cerebral Vascular Disease classification guidelines Stroke confirmed by CT or MRI scan First ever stroke Within 4 months since stroke onset Spastic hemiplegic limbs Ashworth assessment range: 1 to 3 **Exclusion criteria:** Impairment in consciousness, psychology, cognition, agnosia or apraxia Parkinson’s disease Epilepsy Electrolyte imbalance Cardiac pacemaker Severe malnutrition Severe cardiorespiratory disease Poor adherence **Participants:** detailed in Table **PROGRESS‐plus:** summarised in Table
Interventions	(1) Rehabilitation(n = 25) "Both groups were given routine drug treatment and basic rehabilitative training, looking over brain circulation, anti‐coagulation, nutrition and rest, electrolyte balance, prevention of various secondary complications; maintaining optimal limb positioning, passive and active joint range of motion, tissue massage etc. Patients in the treatment group had additional neural facilitation combined with the use of the muscular spasm machine, following the characteristics of the stages of spasticity. Prior to neural facilitation, patients had to undergo relaxation. Neural facilitation training included prone positioning, slow traction to relax tensed muscles; striking spastic muscles including both agonists and antagonists to restore the appropriate muscular balance; traction to muscles and gentle striking to the muscle belly, guiding the affected limbs to exercise, so as to stimulate a balancing reaction, overcoming over activated muscles and compensatory movements; using co‐contraction principles, allowing resistive forces exerted during flexion and extension of the non‐affected upper limb to illicit flexion and extension of the affected upper limb, and assisting or encouraging the patient to actively flex and extend the limb; making use of asymmetric tonic neck reflex mechanisms, reducing the tone in both upper and lower limbs and stimulating limb movement by rotating the patient’s neck, holding on to the lower limbs while moving them with momentum, or through reverse action by moving the non‐affected shoulder and elbow joints rhythmically so as to reduce muscle tension; making use of body weight to optimise ankle joint integrity; stretching the thumb and externally rotating the forearm to reduce tension in the wrist joint and finger flexors; using cold and hot sensations etc to stimulate and activate contraction of relevant muscles, suppress spasticity etc, compressing the joint to reduce tension, reducing spasticity etc. Low‐frequency pulse current treatment: using a Beijing manufactured KX‐3A model for spasticity treatment" Length of intervention period: 4 weeks Number of sessions and length of individual sessions: 30 to 45 minutes/session, 1 session/d, 5/wk Intervention provider: doctor, nurse (2) Usual care group (n = 25) "Both groups were given routine drug treatment and basic rehabilitative training, looking over brain circulation, anti‐coagulation, nutrition and rest, electrolyte balance, prevention of various secondary complications; maintaining optimal limb positioning, passive and active joint range of motion, tissue massage etc" Length of intervention period: not stated Number of sessions and length of individual sessions: not stated Intervention provider: not stated
Outcomes	**Independence in ADL scales:** NR **Motor function scales:** Fugl‐Meyer Assessment **Measures of balance:** NR **Measures of gait velocity:** NR **Length of stay:** NR **Adverse events:** NR **Other measures:** Ashworth Scale **Time points when outcomes were assessed:** at enrolment and after 4 weeks of intervention
Comparison	**Amount of physical rehabilitation in groups:** differs **Categorisation of comparison:** 5. Additional therapy + usual therapy versus usual therapy (dose differs)
Funding & conflicts of interest	**Funding statement:** not reported **Conflict of interest statement:** not reported
Synthesis	**Quantitative synthesis:**Analysis 5.2
Notes	Original study translated from Chinese to English by a member of our team (PLC)

**Table:** Wang 2005

***Study characteristics***	
Methods	**Study aim:** "to investigate the effectiveness of Bobath on stroke patients at different motor stages by comparing their treatment with orthopaedic treatment" **Design:** parallel‐group RCT **Country:** China **Study recruitment and setting:** see Table **Involvement of patients/public/professionals/others:** no
Participants	**Study population (number randomised):** 44 **Inclusion criteria:** Hemiparesis secondary to CVA Lower extremity Brunnström motor recovery 2 to 5 Able to communicate and co‐operate **Exclusion criteria:** Not reported **Participants:** detailed in Table **PROGRESS‐plus:** summarised in Table
Interventions	(1) Bobath "Based on Bobath philosophy" "Approach used strictly adhered to the principles described in detail in the Bobath and Davis texts" Treatment was "individualised, constantly modified according to subject response" Techniques included facilitating normal movement patterns and retraining normal alignment through appropriate sensory and proprioceptive input, direct manual facilitation, key point control, verbal and visual feedback Length of intervention period: 4 weeks Number of sessions and length of individual sessions: 40 minutes, 5 sessions per week for 20 sessions Intervention provider: Bobath‐trained therapists (2) Orthopaedic Passive, assistive, active, and progressive resistive exercise Multiple repetitions of practice of functional activities: rolling, sitting up, transfer, and gait Gait training using parallel bars Length of intervention period: 4 weeks Number of sessions and length of individual sessions: 40 minutes, 5 sessions per week for 20 sessions Intervention provider: physical therapists
Outcomes	**Independence in ADL scales:** NR **Motor function scales:** Motor Assessment Scale **Measures of balance:** Berg Balance Scale **Measures of gait velocity:** NR **Length of stay:** NR **Adverse events:** NR **Other measures:** Stroke Assessment Impairment Set **Time points when outcomes were assessed:** "The patients were assessed twice: once before and once after treatment"
Comparison	**Amount of physical rehabilitation in groups:** equivalent **Categorisation of comparison:** 3.(i) Comparison of different approaches (dose equivalent; with no other physical rehab)
Funding & conflicts of interest	**Funding statement:** "supported partly by the National Science Council of the Republic of China, grant no.91‐2314‐B‐010‐069, and partly by the National Health Research Institutes of the Republic of China, grant no.NHRI‐EX91‐9129EI" **Conflict of interest statement:** not reported
Synthesis	**Quantitative synthesis:**Analysis 7.2; Analysis 7.3
Notes	

**Table:** Wang 2013

***Study characteristics***	
Methods	**Study aim:** to explore the effect of early rehabilitation of ADL on patients with stroke **Design:** parallel‐group RCT **Country:** China **Study recruitment and setting:** see Table **Involvement of patients/public/professionals/others:** no
Participants	**Study population (number randomised):** 130 **Inclusion criteria:** Acute cerebral apoplexy, hospitalised for the first time Met the standards set by the 4th National Cerebrovascular Disease Conference Confirmed with CT or MRI **Exclusion criteria:** Any condition that precludes participation in intervention Normal limb function **Participants:** detailed in Table **PROGRESS‐plus:** summarised in Table
Interventions	(1) Rehabilitation "the patients start rehabilitation exercises with Bobath technique, 2 times/d, once for 30 minutes, by the same doctor in the rehabilitation. The specific rehabilitation exercise method is as follows. 1.2.1 Recumbent position (1) Keep the patient in a correct supine position, prop up the affected shoulder, perform elbow extension and wrist joint movements of the upper limb, flexion and adduction of the lower limb, knee joint, dorsiflexion of the foot. The body position should be changed regularly. (2) Passive activities of the joints of the affected limbs of the patient. (3) Let the patient take the initiative to turn over and move the body. (4) Bridge exercise training. (5) Carry out the activity training of the trunk part. (6) The patient can independently complete the position from the supine position to the sitting position. 1.2.2 Seating (1) Sitting balance training allows the patient to maintain a correct sitting posture and independently complete the movement from a supine position to a sitting position. (2) For sitting and standing transfer, the center of gravity should be evenly distributed on both sides during the process of sitting and standing, and the training of standing up, sitting down, leaving the bed with weight, and transferring the center of gravity should be paid attention to during the training process. Exercise of the affected limb. 1.2.3 (1) Standing balance training. (2) Carry out weight‐bearing training for the lower limbs on the affected side. (3) Extend the medullary joint, flex the knee joint and touch the ground on the affected lower limb, and train up and down the stairs. 1.2.4 Walking training and training up and down stairs Under the condition of weight bearing on the lower limb of the affected side, the normal side of the limb makes a small step forward and backward, trains the balance and the step movement of the knee joint without raising the medulla joint, and the back bends the heel to the ground, up and down Stair training." (2) No physical rehabilitation "All 130 cases of stroke (ischemic, hemorrhagic) were treated by conventional neurology"
Outcomes	**Independence in ADL scales:** Barthel Index **Motor function scales:** NR **Measures of balance:** NR **Measures of gait velocity:** NR **Length of stay:** NR **Adverse events:** NR **Other measures:** Clinical Neurological Impairment Scoring Standard **Time points when outcomes were assessed:** 1 day before the exercise and 1 month after the treatment
Comparison	**Amount of physical rehabilitation in groups:** differs **Categorisation of comparison:** 1. Physical rehabilitation versus no or limited physical rehabilitation (dose differs) (limited = < 50% of dose)
Funding & conflicts of interest	**Funding statement:** not reported **Conflict of interest statement:** not reported
Synthesis	**Quantitative synthesis:**Analysis 1.1
Notes	This study was published in Chinese and translated into English using Google Translate.

**Table:** Wang 2015

***Study characteristics***	
Methods	**Study aim:** "examined whether caregiver‐mediated, home‐based intervention (CHI) could improve physical functioning and social participation in these patients" **Design:** parallel‐group RCT **Country:** Taiwan **Study recruitment and setting:** see Table **Involvement of patients/public/professionals/others:** no
Participants	**Study population (number randomised):** 51 **Inclusion criteria:** Experienced a single ischaemic or haemorrhagic stroke confirmed using CT or MRI > 6 months post‐onset Mild to moderate disability (Brunnstrom recovery stages III‐V) Undergoing < 2 rehabilitation activities per week Home‐dwelling Family members, friends, or paid workers as caregivers (defined as "someone who was most responsible for patient’s daily care and who lived with the patient") Still required assistance to accomplish daily activities **Exclusion criteria:** *Person with stroke:* Use of a nasogastric feeding, urine, or tracheal tube Recurring stroke Dementia (based on medical records) Global or receptive aphasia Severe orthopaedic disability Unstable medical condition (e.g. severe acute myocardial infarction) *Caregivers:* Poor physical health Mental health illness or behavioural disorders (e.g. alcohol abuse, severe orthopaedic disability, uncontrolled diabetes, and hypertension) Unable to provide at least 2 x 60‐ to 90‐min sessions of rehabilitation training per week **Participants:** detailed in Table **PROGRESS‐plus:** summarised in Table
Interventions	(1) Care‐giver mediated home based intervention (CHI) "physical therapist outlined a personalized weekly training schedule for each patient according to the CHI program. The program was roughly divided into 3 phases: phase 1 (weeks 1‐4), to improve patients’ body functions and structural components; phase 2 (weeks 5‐8), to improve patients’ ability to undertake everyday activities within their living environments using task‐specific restorative and compensatory training methods; and phase 3 (weeks 9‐12), to help the patients reintegrate into the society by participating in restorative outdoor leisure activities (Figure 2). A physical therapist visited each patient in the intervention group once weekly for approximately 90 minutes to teach patients personalized rehabilitation skills and to teach the caregivers the skills necessary to assist the patients in performing the planned tasks during trial period. In each visit, the physical therapist explained and demonstrated the tasks to the patient and the caregiver and requested that they practice the tasks to ensure correctness." (2) Control ‐ no treatment "Patients in the control group maintained their everyday routines but also received weekly visits or telephone calls by the therapist to talk about their rehabilitation progress, daily activities, and general health conditions. However, patients in the control group were not given specific instructions or guidance related to rehabilitation skills."
Outcomes	**Independence in ADL scales:** Barthel Index **Motor function scales:** NR **Measures of balance:** Berg Balance Scale **Measures of gait velocity:** 10‐m walk test, 6‐minute walk test **Length of stay:** NR **Adverse events:** NR **Other measures:** Stroke Impact Scale, Caregiver Burden Scale **Time points when outcomes were assessed:** before and after treatment
Comparison	**Amount of physical rehabilitation in groups:** differs **Categorisation of comparison:** 1. Physical rehabilitation versus no or limited physical rehabilitation (dose differs) (limited = < 50% of dose)
Funding & conflicts of interest	**Funding statement:** "disclosed receipt of the following financial support for the research, authorship, and/or publication of this article: Funding for this study was provided by a grant from the National Science Council of Taiwan (NSC99‐2314‐B‐468‐001)" **Conflict of interest statement:** "no potential conflicts of interest with respect to the research, authorship, and/or publication of this article"
Synthesis	**Quantitative synthesis:**Analysis 1.1; Analysis 1.3; Analysis 1.4
Notes	

**Table:** Wang 2021

***Study characteristics***	
Methods	**Study aim:** "to determine the effectiveness of rehabilitation nursing program interventions inpatients with acute ischemic stroke" **Design:** parallel‐group RCT **Country:** China **Study recruitment and setting:** see Table **Involvement of patients/public/professionals/others:** no
Participants	**Study population (number randomised):** 224 **Inclusion criteria:** Age 18 to 90 years old NIHSS consciousness score: level 0 or 1 Acute ischaemic stroke with risk of motor impairment Admitted to the hospital < 7 days of the initial stroke onset **Exclusion criteria:** Successful response to intravenous thrombolysis Haemorrhagic transformation Restricted mobilisation, disability, or other conditions affecting limb movement Other circumstances that precluded patients from co‐operating **Participants:** detailed in Table **PROGRESS‐plus:** summarised in Table
Interventions	(1) Rehabilitation nursing "The interventions included PT like Bobath therapy, bridge exercise, passive and active limb movement training, articular mobility training, sit‐up training, transfer training, balance training, sit‐to‐stand training, walking training, stepping training, limb weight training, OT including repetitive task‐oriented training (wiping tables, transferring screwcaps, and picking up beans), ADL training for dressing and undressing, drinking, and others." "A session included 30 minutes of training, two sessions per day for seven consecutive days." (2) Usual care "the doctor prescribes usual therapist‐led rehabilitation treatment and usual rehabilitation nursing (good limb placement, turning over, and out‐of‐bed mobilization education), with no requirement for frequency or intensity of treatment. The control group received the usual rehabilitation by a therapist, with the start time, duration, and frequency of treatment consistent with the intervention group. The content of usual rehabilitation training was the same as those delivered in the experimental group, but was less specific. The treatments involved more PT, including muscle strength training, limb resistance training, anti‐spasm training, knee extension, and ankle plantar flexion training to induce separation movement, gait training, trunk control, and postural correction training. There was also OT such as grasping training, coordination and active movement training of the affected limbs and hands, and ADL training."
Outcomes	**Independence in ADL scales:** NR **Motor function scales:** Motor Assessment Scale, Fugl‐Meyer Assessment **Measures of balance:** NR **Measures of gait velocity:** NR **Length of stay:** NR **Adverse events:** NR **Other measures:** Action Research Arm Test **Time points when outcomes were assessed:** baseline and after 7 days of treatment
Comparison	**Amount of physical rehabilitation in groups:** equivalent **Categorisation of comparison:** 4. Comparison of similar approaches (dose equivalent)
Funding & conflicts of interest	**Funding statement:** "supported by the National Natural Science Foundation of China (no: 81871839)" **Conflict of interest statement:** "no conflicts of interest in this work"
Synthesis	**Qualitative synthesis:** the interventions compared are similar, with the key difference being whether they are delivered by a rehabilitation nurse, or by a therapist, with prescription by a doctor. The comparisons are therefore not directly relevant to this review.
Notes	This study is funded by the same grant as Wang 2022, but has a different clinical trial registration number and a different recruitment time period.

**Table:** Wang 2022

***Study characteristics***	
Methods	**Study aim:** "to evaluate the feasibility and effectiveness of implementing a nurse‐led motor function rehabilitation program on patients with acute ischemic stroke." **Design:** parallel‐group RCT **Country:** China **Study recruitment and setting:** see Table **Involvement of patients/public/professionals/others:** no
Participants	**Study population (number randomised):** 104 **Inclusion criteria:** Not reported **Exclusion criteria:** Severe medical diseases Severe consciousness Severe cognitive impairment Skeletal, muscular, and neurological dysfunction **Participants:** detailed in Table **PROGRESS‐plus:** summarised in Table
Interventions	(1) Nurse‐led rehabilitation "The experimental group received training that included ADL training and EM such as bed and chair transfer, sitting balance, walking, and sit‐to‐stand. The intervention was performed within 7 days of stroke onset, with each session lasting 30 minutes for 7 consecutive days. Six intervention nurses were supervised by their department head nurse and randomly checked once weekly. Three head nurses attended the monthly meeting to review data quality and performance reports and discussed processes that need improvement." "The interventions were variations of already established PT or OT. PT included limb and trunk movement, whereas OT included repetitive task‐oriented training and ADL training to help patients improve their self‐care ability." "The program training items adopted in this study mainly involve the movement of the trunk, upper, and lower limbs as well as EM (early mobilisation), task‐oriented training, and ADL training." (2) Usual care "The control group received usual rehabilitation care, including good limb placement, regular turning‐over, and EM education following the hierarchical care system included in the Chinese National Health Council standards." "the length of our intervention was set at 7 days. The intervention was conducted twice daily for 30 minutes each."
Outcomes	**Independence in ADL scales:** modified Barthel Index **Motor function scales:** Motor Assessment Scale **Measures of balance:** NR **Measures of gait velocity:** NR **Length of stay:** NR **Adverse events:** adverse events monitored **Other measures:** adverse events, acceptability **Time points when outcomes were assessed:** baseline and 7 days after the intervention
Comparison	**Amount of physical rehabilitation in groups:** differs **Categorisation of comparison:** 1. Physical rehabilitation versus no or limited physical rehabilitation (dose differs) (limited = < 50% of dose)
Funding & conflicts of interest	**Funding statement:** "funded by the National Natural Science Foundation of China (81871839)." **Conflict of interest statement:** "no conflicts of interest"
Synthesis	**Quantitative analysis:**Analysis 1.1; Analysis 1.2; Analysis 1.6
Notes	This study is funded by the same grant as Wang 2021, but has a different clinical trial registration number and a different recruitment time period.

**Table:** Wang Dongya 2015

***Study characteristics***	
Methods	**Study aim:** "to investigate the clinical effect of three‐level rehabilitation training on improving motor dysfunction in stroke patients" **Design:** parallel‐group RCT **Country:** China **Study recruitment and setting:** see Table **Involvement of patients/public/professionals/others:** no
Participants	**Study population (number randomised):** 126 **Inclusion criteria:** Not reported **Exclusion criteria:** Severe medical diseases Severe consciousness Severe cognitive impairment Skeletal, muscular, and neurological dysfunction **Participants:** detailed in Table **PROGRESS‐plus:** summarised in Table
Interventions	(1) three‐level rehabilitation training "A detailed three‐level rehabilitation training plan was formulated for the patients, which included instructing the patients to carry out bedside rehabilitation training during the stable disease period (first‐level rehabilitation) to prevent muscle contractures and joint pains. Complications such as activity limitation; transfer to the rehabilitation department for treatment (second‐level rehabilitation) to use rehabilitation equipment to train muscles and joints to exercise independently and improve joint coordination; transfer to home (third‐level rehabilitation). The content of training is free to use. For the affected limb, improve the speed of limb movement on the basis of ensuring the quality of movement, and maximize the quality of life of the patient. The specific methods are: first‐level rehabilitation training for stasis: the nurses instruct the patient to take the correct posture, and teach the patient to turn over, once every 2 hours; instruct the patient to carry out passive limb training and active movement training of the unaffected limb; teach the patient to take deep breaths and the training method of psoas and abdominal muscles; instruct patients to perform training such as lying position, sitting up and sitting balance, standing balance, etc. The main purpose is to improve the patient's function of getting up. (2) Secondary rehabilitation training: Through rehabilitation exercise equipment, patients are mainly trained to stand on one leg, stand balance, walk, go up and downstairs, etc. The main purpose is to improve the patient's walking function. (3) Level 3 rehabilitation training: Level 3 rehabilitation training is carried out at home, and the nurses visit the patients once every 1 week, and after 3 months, the nurses visit the patients once every 2 weeks, 2 hours each time. First of all, the nursing staff should guide the patient's home environment modification, remove obstacles on the ground, install bed rails and special toilets, and install handrails in the bathroom. Instruct patients to carry out trainings such as body movement, body balance, joint activities, walking, and wheel chair use, and formulate training content registration cards for them. Judging the completion of the patient's training, the patients who have completed the training well should be praised and encouraged in a timely manner. Instruct patients to carry out fine coordination training, let them repeatedly practice holding a pen, dressing, and teach patients to brush their teeth, wash their face, eat, etc. (4) Instruct patients to take medicines on time, and explain to them the knowledge of disease recovery and other aspects, so as to improve the patient's self‐protection awareness, strengthen the patient's psychological rehabilitation guidance, so that the patient can maintain a more optimistic attitude, and cooperate with clinical care and treatment with a positive attitude." Control "The patients in the control group were given routine nursing care, and the patients were given routine treatment and nursing according to the doctor's orders. After the patients' condition was stabilized, the nurses gave rehabilitation training guidance."
Outcomes	**Independence in ADL scales:** NR **Motor function scales:** Fugl Meyer motor function scale **Measures of balance:** NR **Measures of gait velocity:** NR **Length of stay:** NR **Adverse events:** NR **Other measures:** neurological deficit scale **Time points when outcomes were assessed:** before and after intervention
Comparison	**Amount of physical rehabilitation in groups:** differs **Categorisation of comparison:** 1. Physical rehabilitation versus no or limited physical rehabilitation (dose differs) (limited = < 50% of dose)
Funding & conflicts of interest	**Funding statement:** not reported **Conflict of interest statement:** not reported
Synthesis	**Quantitative synthesis:**Analysis 1.2
Notes	Paper published in Chinese and translated using Google Translate.

**Table:** Wang Leilei 2020

***Study characteristics***	
Methods	**Study aim:** "to explore the effects of acupuncture plus rehabilitation based on task‐oriented training on lower limb motor function in Brunnstrom stage III stroke" **Design:** multi‐arm RCT (3‐arms) **Country:** China **Study recruitment and setting:** see Table **Involvement of patients/public/professionals/others:** no
Participants	**Study population (number randomised):** 300 **Inclusion criteria:** Met the diagnostic criteria for stroke formulated by the Fourth National Conference on Cerebrovascular Diseases Stroke confirmed by brain CT or MRI examination Aged between 35 to 75 years First stroke NIHSS > 10 MMSE > 23 Conscious Medically stable Receiving treatment within 1 month after the condition Informed consent **Exclusion criteria:** Patients with severe heart, liver, and kidney dysfunction Diseases of the blood system, endocrine system, immune system, and respiratory system Intracranial space‐occupying lesions Malignant tumours Severe infections Active peptic ulcers Malnutrition Speech comprehension Hearing impairment Cognitive dysfunction Learning disabilities Unilateral neglect and/or severe proprioception loss Previous musculoskeletal and spinal cord lesions Nervous system lesions Vestibular and cerebellar dysfunction Parkinson's disease Lower extremity venous thrombosis and other causes of lower extremity motor dysfunction Implicit lower extremity modified Ashworth spasticity assessment > grade 3 Severe pain Skin infection or ulceration at the acupuncture points of the scalp Pregnant and lactating women History of mental illness and disturbance of consciousness Serious adverse events Incomplete clinical data and lost follow‐up data Participated in other clinical trials during the time of this study **Participants:** detailed in Table **PROGRESS‐plus:** summarised in Table
Interventions	(1) Task‐oriented training + acupuncture ("Group C"). In addition to conventional rehabilitation + acupuncture. See details for acupuncture in (2) below. Delivered in the morning. In the afternoon, lower limb task‐oriented training was conducted. Task‐oriented training includes sit to standing practice, crossing obstacles of different heights and lengths, extension exercises, training bowel muscles, stepping practice, walking sideways, backwards, up a slope, kicking a ball against a wall and walking up and downstairs. Six days per week, delivered for 8 weeks. (2) Conventional rehabilitation + Acupuncture ("Group B") Receives additional conventional rehabilitation to dose‐match the task‐oriented training. Acupuncture was performed at the same time as the rehabilitation training. Cluster needling site: the parietal and anterior parietal areas. The method of puncturing long needles and keeping needles intermittently twisting. Scalp cluster needling one session per day, 6 days per week delivered over 8 weeks. (3) Conventional rehabilitation ("Group A") Used techniques such as Bobath and Brunnstrom for routine rehabilitation training. Training was delivered twice a day ‐ once in the morning and once in the afternoon; session duration: 40 minutes, 6 days a week for a total of 8 weeks. Treatment time for Group (1)/C and (2)/B is the same. Comparison relevant to this review: (1) vs (2)
Outcomes	**Independence in ADL scales:** NR **Motor function scales:** Fugl Meyer assessment **Measures of balance:** NR **Measures of gait velocity:** 10‐m walk test, timed up and go **Length of stay:** NR **Adverse events:** NR **Other measures:** NR **Time points when outcomes were assessed:** NR
Comparison	**Amount of physical rehabilitation in groups:** equivalent **Categorisation of comparison:** 3. (ii) Comparison of different approaches, (dose equivalent; with both/all groups also receiving other physical rehab)
Funding & conflicts of interest	**Funding statement:** not reported **Conflict of interest statement:** not reported
Synthesis	**Quantitative synthesis:**Analysis 7.2; Analysis 7.4
Notes	This study was published in Chinese and relevant sections translated into English by our author team (PLC).

**Table:** Wang Wenwei 2012

***Study characteristics***	
Methods	**Study aim:** "to observe the effects of gait training based on Bobath concept framework intervening on walking capability of stroke patients in convalescent phase" **Design:** parallel‐group RCT **Country:** China **Study recruitment and setting:** see Table **Involvement of patients/public/professionals/others:** no
Participants	**Study population (number randomised):** 80 **Inclusion criteria:** Met the diagnostic criteria of the Fourth National Cerebrovascular Disease Academic Conference Stroke confirmed by CT or MRI Aged 40 to 75 years First stroke, within 1 to 3 months of the disease Stable vital signs Clear consciousness Good cooperation Able to walk more than 10‐m independently or with the help of a cane **Exclusion criteria:** Previous history of stroke, brain tumour, traumatic brain injury, and other neuropsychiatric diseases **Participants:** detailed in Table **PROGRESS‐plus:** summarised in Table
Interventions	All participants received traditional neurology drug treatment. Training was delivered by experienced physical therapists. Both groups received 2 training sessions/day, each session of 30 mins; 5 sessions/week delivered over 4 weeks. (1) Bobath training Participants received Bobath based gait training aimed at improving core stability and improve postural control barriers. The stroke survivor was asked to place their non‐paretic leg on the bed and sit sideways on the bed (facing out of bed). They are asked to keep their body upright, tilt the centre of gravity to the paretic side with the therapist supporting the participant. If core stability is weak, active backward translation training is performed to improve the tension and strength of the abdominal muscles on the affected side. When abdominal strength on the affected side improves, active back rotation training is performed to balance the stroke survivor. The stroke survivor's bilateral abdominal strength can be improved, and the overall dynamic stability can be improved. Two sessions/day with each session lasting 10 mins. Dynamic plantar sensory integration was conducted to establish a physiological support surface. Stroke survivor sits and the therapist grasps the toe of the affected side with his left hand, and the heel of the affected foot with his right hand and uses his own thigh as the axis to guide the affected foot to perform continuous movements of toe flexion during ankle dorsiflexion and toe extension during ankle joint flexion. Manual therapy is applied repeatedly around the soles of the feet to affect proprioception. Finally, the ankle swing phase and stance phase induction of an upright gait was performed on a towel. Stroke survivors need to concentrate and experience the changes in sensation. Two sessions/day with each session lasting 10 mins. Induce normal walking rhythm. Treatment bed was raised to same height as the stroke survivor's anterior superior iliac spine, and the stroke survivor lies on the treatment bed with bare feet and upper body prone. Move the affected foot back to be parallel to the heel of the healthy side. The therapist grasps the quadriceps muscle of the affected side with both hands in a semi‐kneeling position and uses the body to drive the lower limb of the affected side to repeatedly complete flexion and extension until the quadriceps muscle is tense. Then let the lower limb of the healthy side of the stroke survivor repeatedly bend the knee and raise the heel, but the toe cannot leave the ground, and then the stroke survivor’s healthy side foot moves back and forth repeatedly to activate the gluteus medius muscle of the affected side. Therapist grasps the heel of the affected heel with his left hand and the quadriceps femoris with his right hand and assists the affected foot to complete the action of closing the heel and induced the normal gait swing phase and support phase. Two sessions/day with each session lasting 10 mins. (2) Conventional training Participants received conventional gait training including single‐leg weight‐bearing, shifting centre of gravity, stretching, and stepping training, up and down steps using the paretic leg, standing with the affected leg supported, extended side stepping and stepping in place.
Outcomes	**Independence in ADL scales:** NR **Motor function scales:** Fugl‐Meyer assessment **Measures of balance:** Berg Balance Scale **Measures of gait velocity:** NR **Length of stay:** NR **Adverse events:** NR **Other measures:** NR **Time points when outcomes were assessed:** before and after treatment, and 3 weeks after discharge
Comparison	**Amount of physical rehabilitation in groups:** equivalent **Categorisation of comparison:** 3.(i) Comparison of different approaches (dose equivalent; with no other physical rehab)
Funding & conflicts of interest	**Funding statement:** not reported **Conflict of interest statement:** not reported
Synthesis	**Quantitative synthesis:**Analysis 7.2; Analysis 7.3; Analysis 7.4; Analysis 8.2; Analysis 8.3; Analysis 8.4; Analysis 8.9
Notes	This study was published in Chinese and relevant sections translated into English by our author team (PLC).

**Table:** Wan Xueli 2014

***Study characteristics***	
Methods	**Study aim:** to investigate the effect of neurodevelopmental therapy (Bobath technique) on the rehabilitation of stroke patients with hemiplegia, and to provide clinical experience **Design:** parallel‐group RCT **Country:** China **Study recruitment and setting:** see Table **Involvement of patients/public/professionals/others:** no
Participants	**Study population (number randomised):** 48 **Inclusion criteria:** Met clinical diagnostic criteria for stroke in the "Guidelines for the Prevention and Treatment of Cerebrovascular Diseases in China" Obvious symptoms of stroke Diagnosed with CT or MRI Provided informed consent **Exclusion criteria:** Acute haemorrhagic stroke, deep coma or brain herniation Severe drug allergies Mental health condition Poor co‐operation or unable to co‐operate **Participants:** detailed in Table **PROGRESS‐plus:** detailed in Table
Interventions	(1) Bobath therapy "The patients in the treatment group were additionally used Bobath technology for rehabilitation treatment on this basis. The main content: patients received treatment in the early stage of stroke. According to the actual situation of the patient, repeatedly conduct training with the patient, such as shaking hands, sitting, standing, turning over, etc., and at the same time actively give muscle strength training, such as standing, walking, bridge exercise, etc. The content of rehabilitation training needs to be gradual, and you can do sit‐ups successively Rehabilitation training should be limited to 2 times a day, 30‐60 minutes each time. At the same time, family members should be asked to participate together as much as possible, so as to facilitate the patients after returning to the ward. There are still people instructing him to carry out rehabilitation training." (2) No physical rehabilitation "According to the specific conditions of the two groups of patients, symptomatic treatment with appropriate drugs was used." "Both groups received continuous treatment for 4 courses, and 15 d was a course of treatment."
Outcomes	**Independence in ADL scales:** Barthel Index **Motor function scales:** NR **Measures of balance:** NR **Measures of gait velocity:** NR **Length of stay:** NR **Adverse events:** NR **Other measures:** NR **Time points when outcomes were assessed:** Before and after treatment
Comparison	**Amount of physical rehabilitation in groups:** differs **Categorisation of comparison:** 1. Physical rehabilitation versus no or limited physical rehabilitation (dose differs) (limited = < 50% of dose)
Funding & conflicts of interest	**Funding statement:** not reported **Conflict of interest statement:** not reported
Synthesis	**Quantitative synthesis:**Analysis 1.1
Notes	This study was published in Chinese and translated into English using Google Translate.

**Table:** Wei 2014

***Study characteristics***	
Methods	**Study aim:** to investigate the effect of the three‐level rehabilitation system on the clinical neurological function and ADL of stroke patients, and to formulate the best rehabilitation method for stroke patients **Design:** multi‐arm RCT (3‐arm) **Country:** China **Study recruitment and setting:** see Table **Involvement of patients/public/professionals/others:** no
Participants	**Study population (number randomised):** 120 **Inclusion criteria:** Met diagnostic criteria established by the 4th National Cerebrovascular Disease Conference Diagnosis of cerebral infarction or cerebral haemorrhage confirmed by CT or MRI Initial onset Age < 79 years Neurological defect scale (CSS) > 20 points Able to provide informed consent **Exclusion criteria:** TIA, subarachnoid haemorrhage or other disease that affects movement, sensation, and cognition Underlying disease poorly controlled by specialist treatment Changes in condition during treatment New infarction or haemorrhage Severe cognitive impairment Cognitive dysfunction Complete aphasia Severe organ failure Unable to sign consent form **Participants:** detailed in Table **PROGRESS‐plus:** detailed in Table
Interventions	(1) Three‐stage rehabilitation (all stages) "The research group was given the first, second and third level rehabilitation treatment" (2) Three‐stage rehabilitation (1st and 2nd stages only) "the first and second level rehabilitation group was given the first level + second level rehabilitation treatment" (3) Three‐stage rehabilitation (1st and 3rd stages only) "the first and third level rehabilitation group was given the first level + third level rehabilitation treatment" "①First‐level rehabilitation treatment: All patients in the acute stage received routine medical treatment in the neurology department of our hospital, and 48 hours after the vital signs were stabilized, interventional rehabilitation treatment was started. It mainly includes the placement of good limbs, prevention of complications, passive movement of the affected limb, guidance of active movement, physical factor therapy, acupuncture, etc., with an average of 2 to 4 weeks. ②Second‐level rehabilitation treatment: 3‐5 days after the patient's vital signs became stable, they were transferred to the rehabilitation center of our hospital for second‐level rehabilitation treatment. Continue to prevent and control complications, induce gross movements, inhibit abnormal movement patterns, and formulate exercise therapy, occupational therapy, and daily living ability training programs according to different stages of the disease. At all stages of disease development, patients should be encouraged to take the initiative to complete various daily activities that can be completed by themselves, such as eating, grooming, putting on and undressing, etc. If the patient's condition is stable, the CSS score is less than 15, and the simplified Fugl‐Meyer motor function score is more than 70, referral to a community health service center for maintenance rehabilitation can be considered. The average time is 4 to 8 weeks. ③Third‐level rehabilitation treatment: During the maintenance period, the patients will be transferred to the Jinqiao Community Health Service Center affiliated to our hospital for maintenance rehabilitation treatment, and the rehabilitation physicians and therapists of our hospital will provide regular rehabilitation guidance. Treatment focuses on improving gait, improving practical walking ability, improving practical functional training of upper limbs, promoting the recovery of fine motor function of fingers, improving coordination ability, strengthening daily life skills and pre‐vocational rehabilitation training. In the course of treatment, correct wrong actions and patterns in time to avoid misuse syndrome." Comparisons potentially of relevance to this review: (1) vs (2) (i.e. stage 3 versus no treatment), and (1) vs (3) (i.e. stage 2 versus no treatment). Time point of relevance: end of treatment stage being investigated (i.e. month 2 for stage 2; month 6 for stage 3).
Outcomes	**Independence in ADL scales:** Barthel Index **Motor function scales:** Fugl‐Meyer motor function **Measures of balance:** NR **Measures of gait velocity:** NR **Length of stay:** NR **Adverse events:** NR **Other measures:** CSS **Time points when outcomes were assessed:** "time of enrollment, at the end of the 2nd, 6th, 12th and 6th month."
Comparison	(For both comparisons): **Amount of physical rehabilitation in groups:** differs **Categorisation of comparison:** not able to categorise (unclear whether patients continued to receive the other 'stage' treatments, or received no treatment)
Funding & conflicts of interest	**Funding statement:** not reported **Conflict of interest statement:** not reported
Synthesis	**Qualitative synthesis:** insufficient information, and complex study design, means that it is not possible to categorise the comparisons within this multi‐arm trial.
Notes	This study was published in Chinese and translated into English using Google Translate.

**Table:** Werner 1996

***Study characteristics***	
Methods	**Study aim:** "to demonstrate the effectiveness of intensive outpatient therapy" **Design:** parallel‐group RCT **Country:** USA **Study recruitment and setting:** see Table **Involvement of patients/public/professionals/others:** no
Participants	**Study population (number randomised):** 49 **Inclusion criteria:** Between 6 months and 5 years after hospital discharge Acute middle cerebral artery vascular event (documented by computed tomographic scan or clinical presentation) Functional Communication Profile score > 60% No significant medical comorbidities or generalised sensory deficit No history of recurrent stroke(s) "no ongoing therapeutic rehabilitation intervention in the last 4 mo (patients could be seen for routine follow‐up of maintenance therapy (i.e., home exercise program, brace check, or medication adjustments))" Evidence of a functional limitation in the area of dressing, walking, eating, or bathing (< 7 FIM in any of the motor or ADL subscales) **Exclusion criteria:** Not reported **Participants:** detailed in Table **PROGRESS‐plus:** detailed in Table
Interventions	(1) Outpatient rehabilitation "The treatment intervention consisted of an intensive 12‐wk outpatient rehabilitation program." "The treatment patients received 1 hr of physical and 1 hr of occupational therapy, 4 days/wk, for a duration of 12 wk. All physical and occupational therapy interventions were performed by one registered physical and occupational therapist. An initial assessment by a physiatrist, physical therapist, and occupational therapist followed by a team meeting defined the necessary interventions, which were oriented toward functional tasks such as transfers, walking, self‐care, and feeding. Treatment modalities included strengthening, stretching,mobilization, and muscle retraining/facilitation." (2) No treatment "The control group did not receive any outpatient therapy. "
Outcomes	**Independence in ADL scales:** Functional Independence Measure ‐ motor measure (FIM‐MM) **Motor function scales:** NR **Measures of balance:** NR **Measures of gait velocity:** NR **Length of stay:** NR **Adverse events:** NR **Other measures:** Jebsen Hand evaluation, Brunnstrom Motor Score, Becks Depression Inventory, Sickness Index Profile **Time points when outcomes were assessed:** baseline, and after 3 and 9 months
Comparison	**Amount of physical rehabilitation in groups:** differs **Categorisation of comparison:** 1. Physical rehabilitation versus no or limited physical rehabilitation (dose differs) (limited = < 50% of dose)
Funding & conflicts of interest	**Funding statement:** "Supported, in part, by Grant 157‐PR/91‐01 from the Blue Cross Blue Shield of Michigan Foundation, an independent licensee of the Blue Cross Shield Association. Its contents are solely the responsibility of the authors and do not necessarily represent the official views of the Blue Cross Blue Shield of Michigan Foundation." **Conflict of interest statement:** not reported
Synthesis	**Quantitative synthesis:** data presented in paper does not include standard deviations/confidence intervals and is therefore not suitable for inclusion in meta‐analysis
Notes	

**Table:** Wu 2006

***Study characteristics***	
Methods	**Study aim:** "to study the effect of early rehabilitation on spasticity, ADL and motor function in patients with hemiplegia after stroke" **Design:** parallel‐group RCT **Country:** China **Study recruitment and setting:** see Table **Involvement of patients/public/professionals/others:** no
Participants	**Study population (number randomised):** 100 **Inclusion criteria:** Ischaemic or haemorrhagic stroke participants identified from the 1995 Fourth National Conference on Cerebral Vascular Disease classification guidelines Confirmed by CT or MRI scan as first ever stroke Aged 40 to 80 years old with deficits in limb function Medically stable within 24 hours after stroke onset GCS score > 8 Provided informed consent **Exclusion criteria:** Not reported **Participants:** detailed in Table **PROGRESS‐plus:** detailed in Table
Interventions	(1) Rehabilitation (n = 50) "Received clinical treatment and regular rehabilitation training. Standardised rehabilitation training commenced after the patients were cognitively and medically stable for 48 hours with no aggravation of existing stroke symptoms (within 21 days of stroke, as stage 1 rehabilitation); rehabilitation treatment from early recovery to transfer to Rehabilitation ward (within 3 months of stroke, as stage 2 rehabilitation); community rehabilitation (e.g. home ward) from middle to late recovery (within 4‐6 months after stroke, as stage 3 rehabilitation), with the following method: Prevention and treatment of spasticity: (i) appropriate positioning. (ii) Bobath method of holding hands: both hands intertwined, use the non‐affected fingers to lock the affected fingers, with the affected thumb at the top, stretch forward, flex shoulders, lift arms to touch the forehead, hold for a few seconds before returning to original position, practising multiple times daily. (iii) active stretch: extend affected fingers, place 20cm from body on a support, use body weight to stretch spastic muscles, for 3‐5minutes per stretch, practising multiple times daily. (iv) Traction: apply traction techniques to affected spastic joints, on feeling resistance, continue with technique for at least 2‐3 minutes on the same spot, relax, and repeat. (v) standing bed training: stand on a sloped plank in front of a ladder wall, correct foot inversion and foot, target lower limb spasticity, 5‐8 minutes daily. (vi) critical control of Bobath technique, PNF’s technique of upper limb stretch and lower limb flexion; both tonic labyrinthine reflex and the asymmetrical tonic neck reflex were also used in the rehabilitation process" "On top of preventing and treating spasticity, appropriate therapy was given depending on the stroke phase, including: rolling practice on bed, bridging practice, lying to sitting and sitting balance training, sit‐to‐stand and standing balance training, gait and up‐and‐down stairs training, ADL practice, manual therapy, speech and language therapy, psychological counselling" Length of intervention period: 6 months Number of sessions and length of individual sessions: dependent on stage of recovery (see above) Intervention provider: not stated (2) No physical rehabilitation group (n = 50) "Received clinical treatment and unguided self‐training" Length of intervention period: 6 months Number of sessions and length of individual sessions: not stated Intervention provider: not stated
Outcomes	**Independence in ADL scales:** Modified Barthel Index **Motor function scales:** Fugl‐Meyer Assessment **Measures of balance:** NR **Measures of gait velocity:** NR **Length of stay:** NR **Adverse events:** NR **Other measures:** Modified Ashworth Scale **Time points when outcomes were assessed:** "pre‐treatment' and after 6 months of intervention"
Comparison	**Amount of physical rehabilitation in groups:** differs **Categorisation of comparison:** 1. Physical rehabilitation versus no or limited physical rehabilitation (dose differs) (limited = < 50% of dose)
Funding & conflicts of interest	**Funding statement:** not reported **Conflict of interest statement:** not reported
Synthesis	**Quantitative synthesis:**Analysis 1.1; Analysis 1.2
Notes	Original study translated from Chinese to English by a member of our team (PLC).

**Table:** Wu 2020

***Study characteristics***	
Methods	**Study aim:** "To explore the feasibility and effectiveness of collaborative care model based telerehabilitation exercise training for acute stroke patients" **Design:** parallel‐group RCT **Country:** China **Study recruitment and setting:** see Table **Involvement of patients/public/professionals/others:** no
Participants	**Study population (number randomised):** 64 **Inclusion criteria:** 18 to 80 years Diagnosis of ischaemic or haemorrhagic stroke based on the Chinese Medical Association Neurology Branch (2014) Confirmed by CT and/or MRI scans NIHSS: 5 to 15 points Limb dysfunction (Brunnstrom function stage II‐III) Home recouperation after discharge Ability of caregivers ≤ 40 points according to the caregiver capacity scale Provided informed consent **Exclusion criteria:** Serious heart, liver, lung, kidney and other organ diseases Severe cognitive disabilities or mental disorder Other diseases that affect motor function (e.g. osteoarthrosis) Complete aphasia Legal blindness or serious visual impairment **Participants:** detailed in Table **PROGRESS‐plus:** detailed in Table
Interventions	(1) Remote home‐based rehabilitation "After discharge, patients in the intervention group received home remote rehabilitation based on a collaborative care model. A collaborative care team consisting of neurologists, nurses, rehabilitation therapists, counselors, and caregivers was established. Rehabilitation therapists assess the extent of patient dysfunction and work with family caregivers to develop rehabilitation plans and goals. The home remote rehabilitation guidance uses the Internet‐based TCMeeting v6.0 video conferencing system" Intervention includes sitting up training, balance training, antispasmodic training, strength training, walking training, task‐oriented training. (2) Usual care "After discharge, Patients in the control group received only routine rehabilitation and nursing measures, including dietary guidance, medication guidance,and rehabilitation guidance, which were conducted by telephone follow‐up once a week. Patients can go to the rehabilitation clinic to get rehabilitation instructions as needed"
Outcomes	**Independence in ADL scales:** Modified Barthel Index **Motor function scales:** Fugl Meyer motor assessment **Measures of balance:** Berg Balance Scale **Measures of gait velocity:** timed up and go test, 6‐minute walking test **Length of stay:** NR **Adverse events:** NR **Other measures:** stroke‐specific quality of life **Time points when outcomes were assessed:** baseline, 4, 8, and 12 weeks after enrolment
Comparison	**Amount of physical rehabilitation in groups:**differs **Categorisation of comparison:** 1. Physical rehabilitation versus no or limited physical rehabilitation (dose differs) (limited = < 50% of dose)
Funding & conflicts of interest	**Funding statement:** "This study was funded by Changzhou Health Committee(Guiding project WZ201906)" **Conflict of interest statement:** "no conflicts of interest"
Synthesis	**Quantitative synthesis:**Analysis 1.1; Analysis 1.2; Analysis 1.3; Analysis 1.4
Notes	

**Table:** Wu Jiaming 2006

***Study characteristics***	
Methods	**Study aim:** "to explore the effect of postural control training on daily living function of stroke patients" **Design:** parallel‐group RCT **Country:** China **Study recruitment and setting:** see Table **Involvement of patients/public/professionals/others:** no
Participants	**Study population (number randomised):** 80 **Inclusion criteria:** Aged between 40 and 75 years Medically stable Unilateral limb paralysis No other serious complications **Exclusion criteria:** Previous history of dementia Lower limb impairment or other bone/joint disease affecting balance **Participants:** detailed in Table **PROGRESS‐plus:** detailed in Table
Interventions	(1) Rehabilitation Mainly utilised neurofacilitative techniques such as Bobath, PNF, electrotherapy (electrical physical agent). Active and resistance exercise were carried out at the same time and involved ADL training, exercises to improve walking, progressive gait training including up and down stairs. Also received induction electrotherapy and posture control training. Induction electrotherapy: stimulates upper limb flexors and lower limbs extensors during the flaccid paralysis phase, stimulates upper limbs extensors and lower limbs flexors during the spasm phase, and selectively stimulates weak muscle groups during the improvement phase. Delivered for 10 minutes for upper and lower extremities each time. Postural control training includes lying position to increase trunk flexion. Abdominal muscles are stimulated by manipulation, promote abdominal breathing, changing from lying position to long sitting position on the bed and the opposite movement, to train the contraction control of the flexor muscles of the trunk. Recumbent body rotation control training: rotation of the lower body and turning over to the affected side and the healthy side. Supine position for stretching and lateral flexion control ability training: a double bridge, single bridge movement. Application of facilitation manipulation to stimulate the flexor muscles on the affected side to induce contraction control. Extension, lateral flexion, and rotation control training for long sitting position. Sitting position: (a) the stroke survivor turns their head and torso over their shoulder with their hands on their legs and returns to the neutral position to improve the rotation control of their body in a sitting position; (b) the therapist helps the stroke survivor to support the forearm of the affected side and the healthy side on one or two pillows to the side and return to the neutral position. (2) Conventional rehabilitation Both groups received conventional neurology treatment. Rehabilitation training started once the condition had stabilised.
Outcomes	**Independence in ADL scales:** Barthel Index **Motor function scales:** Fugl Meyer Assessment **Measures of balance:** NR **Measures of gait velocity:** NR **Length of stay:** NR **Adverse events:** NR **Other measures:** Postural Assessment Score (PASS) **Time points when outcomes were assessed:** before and after training
Comparison	**Amount of physical rehabilitation in groups:** equivalent/differs **Categorisation of comparison:** 5. Additional therapy + usual therapy versus usual therapy (dose differs)
Funding & conflicts of interest	**Funding statement:** not reported **Conflict of interest statement:** not reported
Synthesis	**Quantitative synthesis:**Analysis 5.1; Analysis 5.2
Notes	This study was published in Chinese and relevant sections translated into English by our author team (PLC).

**Table:** Wu Jing 2015

***Study characteristics***	
Methods	**Study aim:** to investigate the effect of tertiary rehabilitation therapy on motor dysfunction and cognitive dysfunction in young people with stroke **Design:** parallel‐group RCT **Country:** China **Study recruitment and setting:** see Table **Involvement of patients/public/professionals/others:** no
Participants	**Study population (number randomised):** 92 **Inclusion criteria:** Met the diagnostic criteria established by the 4th National Cerebrovascular Disease Academic Conference Stroke confirmed by CT or MRI Admitted within 48 hours after onset Age < 45 years GCS > 8 Limb dysfunction **Exclusion criteria:** Insufficiency of heart, lung, liver, and kidney function Malignant tumour History of dementia, mental health condition, deafness or quadriplegia **Participants:** detailed in Table **PROGRESS‐plus:** detailed in Table
Interventions	(1) Three‐stage rehabilitation "①First‐level rehabilitation treatment: Routine neurology treatment is given from the onset to 1 month after the onset of the disease, and early bedside rehabilitation treatment is given after the condition is stabilized, mainly physical therapy, such as the placement of anti‐spasmodic postures, passive and physical therapy of limbs. Active training, waist and abdominal muscle training, lying‐to‐sitting training, sitting balance training, sitting‐to‐standing training, deep breathing training, etc. Training 40‐45 min/time, 1 time/d, instructing nurses, patients and their families to train during training, so that a certain degree of training can be obtained during non‐treatment time. The first‐level rehabilitation treatment is carried out in the ward and mainly solves the problem of the patient's ability to get up. ②Secondary rehabilitation treatment: It is carried out in the ward in the 2‐3 months after the onset of the disease. The rehabilitation measures are standing, walking and going up and down the stairs. The training is 40‐45 min/time, 2 times/d. This stage mainly solves the patient's walking function. question. ③Third‐level rehabilitation treatment: From the 4th to 6th month after the onset of the disease, the patient will be transferred from the ward to the home or community for rehabilitation treatment according to the recovery of the disease. This stage mainly includes training in activities of daily living, such as eating, dressing, and grooming. Etc., training 40‐45 min/time, 2 times/d, rehabilitation doctors come to guide patients once every 15 days, and patients and their family members mainly complete the training program at this stage. ④Cognitive function training: During the first, second and third level rehabilitation treatment, according to the understanding, memory and language barriers of the patient, targeted training, such as collar, puzzle training, as well as screw installation, woodworking, Weaving and other manual simulation training; instruct the patient's family to assist the patient's cognitive ability training, so that the patient can complete the cognitive function training at home or in the community." (2) No physical rehabilitation "Only routine neurological treatment was given, no standardized rehabilitation treatment was given, and the clinicians were given oral medical instructions to exercise on their own. "
Outcomes	**Independence in ADL scales:** NR **Motor function scales:** Fugl‐Meyer motor function score **Measures of balance:** NR **Measures of gait velocity:** NR **Length of stay:** NR **Adverse events:** NR **Other measures:** cognitive dysfunction **Time points when outcomes were assessed:** before and 6 months after treatment
Comparison	**Amount of physical rehabilitation in groups:** differs **Categorisation of comparison:** 1. Physical rehabilitation versus no or limited physical rehabilitation (dose differs) (limited = < 50% of dose)
Funding & conflicts of interest	**Funding statement:** not reported **Conflict of interest statement:** not reported
Synthesis	**Quantitative synthesis:**Analysis 1.2
Notes	This study was published in Chinese and translated into English using Google Translate.

**Table:** Wu Lotus 2016

***Study characteristics***	
Methods	**Study aim:** to analyse the effect of intensive walking training on the rehabilitation of hemiplegic patients with early stroke **Design:** parallel‐group RCT **Country:** China **Study recruitment and setting:** see Table **Involvement of patients/public/professionals/others:** no
Participants	**Study population (number randomised):** 80 **Inclusion criteria:** Not reported **Exclusion criteria:** Not reported **Participants:** detailed in Table **PROGRESS‐plus:** detailed in Table
Interventions	(1) Walking training Same conventional rehabilitation as below plus intensive gait training. This comprised: *Standing balance training:* feet position should be slightly backward, and the upper body is kept straight and slightly forward. Shoulders should be moved forward over the toes and forward of the knees, with the centre of gravity shifted to the feet, participants should lean forward and stand up. Splints can be used to fix the limbs on the affected side to prevent excessive stretching of the muscle groups by co‐ordination and anti‐imbalance. A sandbag (1 kg) can be used to immobilise the ankle of the affected limb to prevent the knee joint from flexing. *Gait training:* after early weight bearing should be 2 sessions/day with each session lasting 10 to 15 mins progressively increasing to 45 mins per session. Splints should be used to fix the upper and lower limbs of the affected side, and a 1 kg sandbag should be loaded on the ankle of the lower limbs. The instructor should be positioned on the posterolateral side of the affected limb, holding the stroke survivor's belt to prevent a fall. Participants should step on the paretic leg first, stretch and stretch the knee, and prepare for the swing of the healthy leg. *Stairs training:* 2 sessions/day with each session lasting 45 mins. The instructor should stand on the hemiplegic side behind the stroke survivor. When going up the stairs, go up the affected limb first, and when going down the stairs, go down the unaffected limb first. Treatment should be stopped if BP > 180/100 mmHg during training, or participant complains of dizziness, chest tightness. (2) Conventional rehabilitation Involved good limb positioning, body positioning, encouragement of activity ‐ passive or active, sitting up, trying to sit for 30 mins, before trying to stand up, standing practice.
Outcomes	**Independence in ADL scales:** Barthel index **Motor function scales:** NR **Measures of balance:** NR **Measures of gait velocity:** NR **Length of stay:** NR **Adverse events:** NR **Other measures:** NR **Time points when outcomes were assessed:** before and after treatment
Comparison	**Amount of physical rehabilitation in groups:** differs **Categorisation of comparison:** 5. Additional therapy + usual therapy versus usual therapy (dose differs)
Funding & conflicts of interest	**Funding statement:** not reported **Conflict of interest statement:** not reported
Synthesis	**Quantitative synthesis:**Analysis 5.1; Analysis 5.2
Notes	This study was published in Chinese and relevant sections translated into English by our author team (PLC).

**Table:** Xiao 2003

***Study characteristics***	
Methods	**Study aim:** "to see whether early intensive rehabilitation was able to reduce the length of stay and reduce medical costs in stroke" **Design:** parallel‐group RCT **Country:** China **Study recruitment and setting:** see Table **Involvement of patients/public/professionals/others:** no
Participants	**Study population (number randomised):** 134 **Inclusion criteria:** Met the diagnostic criteria of the 1995 Fourth National Conference on Cerebral Vascular Disease classification guidelines Carotid artery system, ischaemic stroke participants with no previous history of stroke or no obvious deficits if had stroke history Premorbid independence in ADL Hemiplegic limb 4th grade on muscle strength Informed consent obtained **Exclusion criteria:** Subarachnoid haemorrhage or TIA Vertebrobasilar arterial thrombosis Obvious awareness issues during stroke onset Hemiplegic limb > 4th grade on muscle strength Severe organ diseases during stroke onset **Participants:** detailed in Table **PROGRESS‐plus:** detailed in Table
Interventions	(1) Intensive rehabilitation (n = 67) "After classifying patients as ICA or HCA, patients were randomly allocated to either the intensive rehabilitation or conventional group. Both groups received conventional treatment and rehabilitation, with rehabilitative training up to 2 weeks in duration. The intensive rehabilitation group made use of: combined Bobath and PNF therapy. This was done 3/week, 30min/session; using UTU‐500 ultrasound at the same time, 1/day, 30 min/session" Length of intervention period: up to 2 weeks in duration Number of sessions and length of individual sessions: 3/week, 30 minutes/session; using UTU‐500 ultrasound at the same time, 1/d, 30 minutes/session Intervention provider: not stated (2) Conventional physiotherapy (n = 67) "The conventional group made use of usual therapeutic and Bobath techniques" / "given clinical treatment and unguided self‐training." [Not clear whether this group received active rehabilitation or not] Length of intervention period: "up to 2 weeks in duration" Number of sessions and length of individual sessions: 3/week, 30 minutes/session Intervention provider: not stated
Outcomes	**Independence in ADL scales:** NR **Motor function scales:** NR **Measures of balance:** NR **Measures of gait velocity:** NR **Length of stay:** NR **Adverse events:** NR **Other measures:** neurological deficit score, total activity ability score **Time points when outcomes were assessed:** before and after intervention
Comparison	**Amount of physical rehabilitation in groups:** differs **Categorisation of comparison:** 5. Additional therapy + usual therapy versus usual therapy (dose differs) (Note: assumption has been made that the conventional group did receive some active physical rehabilitation).
Funding & conflicts of interest	**Funding statement:** not reported **Conflict of interest statement:** not reported
Synthesis	**Qualitative synthesis:** no data suitable for analysis
Notes	Original study translated from Chinese to English by a member of our team (PLC).

**Table:** Xiao Yuhua 2015

***Study characteristics***	
Methods	**Study aim:** "To investigate the effect of tertiary rehabilitation nursing on motor function and ADL in stroke patients" **Design:** parallel‐group RCT **Country:** China **Study recruitment and setting:** see Table **Involvement of patients/public/professionals/others:** not reported
Participants	**Study population (number randomised):** 83 **Inclusion criteria:** Not reported (abstract only available) **Exclusion criteria:** Not reported (abstract only available) **Participants:** detailed in Table **PROGRESS‐plus:** detailed in Table
Interventions	(1) Three‐stage rehabilitation "The rehabilitation group was given three‐level rehabilitation care..." (2) No physical rehabilitation "Both groups received routine diagnosis, treatment and nursing in neurology."
Outcomes	**Independence in ADL scales:** modified Barthel Index **Motor function scales:** Fugl‐Meyer assessment **Measures of balance:** NR **Measures of gait velocity:** NR **Length of stay:**NR **Adverse events:** shoulder complications **Other measures:** patient satisfaction **Time points when outcomes were assessed:** after 1 and 6 months of treatment
Comparison	**Amount of physical rehabilitation in groups:** differs **Categorisation of comparison:** 1. Physical rehabilitation versus no or limited physical rehabilitation (dose differs) (limited = < 50% of dose)
Funding & conflicts of interest	**Funding statement:** not reported **Conflict of interest statement:** not reported
Synthesis	**Qualitative synthesis only:** abstract only ‐ unable to obtain full paper (see notes)
Notes	This study was published in Chinese and the abstract translated into English using Google Translate. We requested the article as an inter‐library loan from the British Library, but (on 2 occasions) were sent the wrong paper (Requested: Xiao Yuhua; Lu Hongjian; Geng Guilin; Wang Cuifang; Zhu Hongmei; Hu Yonglin, 2015. Effects of tertiary rehabilitation nursing on motor function and activities of daily living in stroke patients. Recovery in China, 30(4) Received: Li Yuan Di Shuyun, 2018. Effects of tertiary rehabilitation nursing on motor function and activities of daily living in stroke patients. Nursing Experience 7)

**Table:** Xiao Zhen‐dong 2014

***Study characteristics***	
Methods	**Study aim:** to investigate the effect of early balance training on the balance function of stroke patients **Design:** parallel‐group RCT **Country:** China **Study recruitment and setting:** see Table **Involvement of patients/public/professionals/others:** no
Participants	**Study population (number randomised):** 60 **Inclusion criteria:** Met diagnostic criteria for stroke and confirmed used CT or MRI No obvious cognitive dysfunction Different degrees of balance dysfunction **Exclusion criteria:** Not reported **Participants:** detailed in Table **PROGRESS‐plus:** detailed in Table
Interventions	(1) Balance training "The balance training group added balance function training on the basis of conventional rehabilitation therapy, 3 times a day, 30 minutes each time. The balance training is as follows: 1. 2. 1 Seated balance training The patient was instructed to actively adjust the center of gravity to perform static balance training on a chair with upper limb support function, and at the same time, the patient was also instructed to actively move the head, upper and lower limbs and trunk for dynamic balance training. During training, support the patient's upper limb and trunk on the inclined side with one hand, and support the patient's shoulder with the other hand, and apply force to the inclined side to strengthen the adjustment response of the patient's head and the lateral flexion function of the contralateral limb, 3 times a day, 10 training sessions each time. min. 1. 2. 2 standing balance training Instruct the patient to support the body weight with both lower limbs, slightly flex his knees at about 20°, control the patient's lower limbs to be in abduction and external rotation, and use a stepper at the same time, and gradually increase the amount of exercise under relevant conditions, 3 times a day, each training session 10min 1. 2. 3 walking training The patient held the balance bar in both hands for walking training along a straight line, and at the same time, the multi‐functional combination box was used for training, from low to high, from easy to difficult, 3 times a day, each training for 10 minutes. (2) Routine rehabiltiation "Both groups were given routine rehabilitation therapy for one month, which included peripheral sensory stimulation, motor control training, acupuncture and massage therapy."
Outcomes	**Independence in ADL scales:** NR **Motor function scales:** NR **Measures of balance:** Berg Balance Scale, Tinetti Scale **Measures of gait velocity:** NR **Length of stay:** NR **Adverse events:** NR **Other measures:** NR **Time points when outcomes were assessed:** before and after 1 month of treatment
Comparison	**Amount of physical rehabilitation in groups:** differs **Categorisation of comparison:** 5. Additional therapy + usual therapy versus usual therapy (dose differs)
Funding & conflicts of interest	**Funding statement:** not reported **Conflict of interest statement:** not reported
Synthesis	**Quantitative synthesis:**Analysis 5.3
Notes	This study was published in Chinese and translated into English using Google Translate.

**Table:** Xie 2003

***Study characteristics***	
Methods	**Study aim:** to explore the effectiveness of early rehabilitation nursing on ADL in people with stroke **Design:** parallel‐group RCT **Country:** China **Study recruitment and setting:** see Table **Involvement of patients/public/professionals/others:** no
Participants	**Study population (number randomised):** 64 **Inclusion criteria:** Met the diagnostic criteria established by 4th National Cerebrovascular Disease Conference Stroke confirmed by head CT **Exclusion criteria:** Not reported **Participants:** detailed in Table **PROGRESS‐plus:** detailed in Table
Interventions	(1) Rehabilitation (n = 32) "Following methods were adopted in acute phase: (i) Keeping limbs in function position; (ii) Passive joint motion within its maximal bound. Beginning from healthy side to hemiplegia one, from bigger joint to smaller one. Paying more attention to elbow, finger and ankle, because they are vulnerable to stiff. Each joint was moved five to six times in articular direction, practising two to three times each day. (iii) Relax of hemiplegia side could be achieved through gentle and regular massage. Pectoral muscles were massaged to lessen contracture and avoid shoulder dysfunction." "The massage was practised five to six times each day, and each massage lasted 15 – 20 min" "The recover phase began one to three weeks after stroke attack. Integrated treatment could be adopted to deal with this phase. It included the rehabilitation of stiff joints and spastic muscles, the training of upper and lower limbs. Several treatments could be used in joint rehabilitation, including passive motion, intermittent or constant traction, muscle massage, drug and biological feedback treatment. Both positive and passive motions were adopted in upper limb training, stepping up and down stairs. The training was practised twice a day, with 30 min each time. ADL training could be divided into lying and sitting ADL training, so as to avoid deformity and correct abnormal motion mode" Length of intervention period: not stated Number of sessions and length of individual sessions: dependent on stage of recovery (see above) Intervention provider: not stated (2) No physical rehabilitation group (n = 32) No intervention Length of intervention period: no intervention Number of sessions and length of individual sessions: no intervention Intervention provider: no intervention
Outcomes	**Independence in ADL scales:** Barthel Index **Motor function scales:** NR **Measures of balance:** NR **Measures of gait velocity:** NR **Length of stay:** NR **Adverse events:** NR **Other measures:** NR **Time points when outcomes were assessed:** "first assessment began within two days after patients vital sign steady, the other two assessments were adopted in 30 days and 60 days"
Comparison	**Amount of physical rehabilitation in groups:** differs **Categorisation of comparison:** 1. Physical rehabilitation versus no or limited physical rehabilitation (dose differs) (limited = < 50% of dose)
Funding & conflicts of interest	**Funding statement:** not reported **Conflict of interest statement:** not reported
Synthesis	**Quantitative synthesis:**Analysis 1.1; Analysis 2.1
Notes	Short Chinese study published in English (brief report only)

**Table:** Xie 2005

***Study characteristics***	
Methods	**Study aim:** "to explore effects of rehabilitation training on the QoL in stroke patients by observing the QoL in stroke patients during different duration" **Design:** parallel‐group RCT **Country:** China **Study recruitment and setting:** see Table **Involvement of patients/public/professionals/others:** no
Participants	**Study population (number randomised):** 70 **Inclusion criteria:** Stroke participants who met the 1995 Fourth National Conference on Cerebral Vascular Disease classification guidelines First ever stroke confirmed by CT or MRI scan GCS > 8 Aged 40 to 80 years old Deficits in limb function Not more than 3 weeks after stroke Provided informed consent **Exclusion criteria:** Active liver disease or impaired function of the liver, kidney or congestive heart failure Dementia History of deafness/muteness Impaired respiratory system Quadriplegia **Participants:** detailed in Table **PROGRESS‐plus:** detailed in Table
Interventions	(1) Rehabilitation (n = 35) "In addition to conventional drug treatment, therapists administered 6 months (average of 12.2 ± 9.2 days) of one‐to‐one rehabilitation training to patients in the rehabilitation treatment group. Approximately one month after stroke, these patients underwent Level 1 rehabilitation in the ward, 1/day, 45min/session, with the therapists teaching the family members and nursing aides the correct method of assisting with exercises and nursing care, requesting them to facilitate practice of exercises outwith therapy time, 1‐2/day" "Training content included: ‘putting good posture, turning the body over training, self‐assistance exercises on the bed (plugging the two hands, bridge‐like movement, shifting on the bed, controlling the coax), the passive motion of upper limb, trunk and lower limb, sit‐decubitus training, standing training, concordant training of every joint, gait training and activities of daily living training etc. Additional intervention information in reported in the text includes: sitting and lying training; facial, tongue and lip muscle training; breathing control training; balance control training" "During 2nd and 3rd month, participants transferred to Level 2 rehabilitation hospital to continue with rehabilitation training (Level 2 rehabilitation) depending on participant’s medical condition and functional recovery status, with rehabilitation delivered by Level 2 rehabilitation hospital’s therapists, 2‐3/week, 45min/session, with nursing aides and family members assisting participants with exercises everyday outwith therapy time; or participants were transferred home with therapists conducting home visits 2/week, assisting participants with necessary functional training, till home visits ended (Level 3 rehabilitation)" Length of intervention period: 6 months Number of sessions and length of individual sessions: dependent on stage of recovery (see above) Intervention provider: therapists (2) No physical rehabilitation group (n = 35) "The control group underwent similar conventional drug treatment as the rehabilitation treatment group, without any rehabilitation by therapists" Length of intervention period: no intervention Number of sessions and length of individual sessions: no intervention Intervention provider: no intervention
Outcomes	**Independence in ADL scales:** NR **Motor function scales:** NR **Measures of balance:** NR **Measures of gait velocity:** NR **Length of stay:** NR **Adverse events:** NR **Other measures:** Chinese edition of the World Health Organization quality of life scale **Time points when outcomes were assessed:** "at the moment of selecting and 1, 3 and 6 months of progress"
Comparison	**Amount of physical rehabilitation in groups:** differs **Categorisation of comparison:** 1. Physical rehabilitation versus no or limited physical rehabilitation (dose differs) (limited = < 50% of dose)
Funding & conflicts of interest	**Funding statement:** "supported by the Key Program during the 10th Five‐Year Plan Period, No 2001BA703B18*" **Conflict of interest statement:** not reported
Synthesis	**Qualitative synthesis:** no relevant outcomes for inclusion in analysis
Notes	Published in Chinese. Translated by a member of our team (PLC).

**Table:** Xu 1999

***Study characteristics***	
Methods	**Study aim:** to study the effects of rehabilitation treatment on hemiplegia following a stroke **Design:** parallel‐group RCT **Country:** China **Study recruitment and setting:** see Table **Involvement of patients/public/professionals/others:** no
Participants	**Study population (number randomised):** 62 **Inclusion criteria:** Not reported **Exclusion criteria:** Not reported **Participants:** detailed in Table **PROGRESS‐plus:** detailed in Table
Interventions	(1) Rehabilitation (n = 32) "Patients in the ‘rehabilitation treatment' group were given clinical treatment and regular recovery training. They used various exercise treatment techniques which had Bobath and Brunnstrom as their focus" Length of intervention period: 1 month Number of sessions and length of individual sessions: one‐to‐one treatment approach, 2 times/d, 1 hour/session Intervention provider: not stated but "with participation from family" (2) No physical rehabilitation group (n = 30) "Received ‘traditional clinical treatment'" Length of intervention period: no intervention Number of sessions and length of individual sessions: no intervention Intervention provider: no intervention
Outcomes	**Independence in ADL scales:** Barthel Index **Motor function scales:** NR **Measures of balance:** NR **Measures of gait velocity:** NR **Length of stay:** NR **Adverse events:** NR **Other measures:** "Degree of neural defect" **Time points when outcomes were assessed:** before and after intervention
Comparison	**Amount of physical rehabilitation in groups:** differs **Categorisation of comparison:** 1. Physical rehabilitation versus no or limited physical rehabilitation (dose differs) (limited = < 50% of dose)
Funding & conflicts of interest	**Funding statement:** not reported **Conflict of interest statement:** not reported
Synthesis	**Quantitative synthesis:**Analysis 1.1
Notes	Original study translated from Chinese to English by a member of our team (PLC).

**Table:** Xu 2003a

***Study characteristics***	
Methods	**Study aim:** "to explore the effects of early rehabilitation on paralysis after cerebral infarction" **Design:** parallel‐group RCT **Country:** China **Study recruitment and setting:** see Table **Involvement of patients/public/professionals/others:** no
Participants	**Study population (number randomised):** 186 **Inclusion criteria:** Diagnosis met the 4th National Cerebrovascular Disease Diagnosis Standards Stroke confirmed by CT **Exclusion criteria:** "mild type or combined with mental disturbance, disorder of consciousness, mixed and sensory aphasia" **Participants:** detailed in Table **PROGRESS‐plus:** detailed in Table
Interventions	(1) Rehabilitation (n = 94) "rehabilitation treatment of acute phase advanced by Yu duisheng of rehabilitation research center (1997), Beijing" "Early rehabilitation procedure included: (i) Design of position of healthy limbs (massage position) including supine position, lateral position with affected limbs at lower side and healthy limbs at upper side and lateral position with affected limbs at upper side and healthy limbs at lower side. (ii) Training of motion range of joints; (iii) Bridging training; (iv) Balance training including sitting position and erect position; (v) Walk training" Length of intervention period: 21 days Number of sessions and length of individual sessions: "once a day" Intervention provider: not stated (2) No physical rehabilitation group (n = 92) "Two groups received routine treatment in department of neurology" No treatment Length of intervention period: no intervention Number of sessions and length of individual sessions: no intervention Intervention provider: no intervention
Outcomes	**Independence in ADL scales:** Barthel Index **Motor function scales:** Fugl‐Meyer 'Analysis' **Measures of balance:** NR **Measures of gait velocity:** NR **Length of stay:** NR **Adverse events:** NR **Other measures:** walking recovery rate **Time points when outcomes were assessed:**"After first evaluation, rehabilitation group received rehabilitation treatment of acute phase advanced by Yu duisheng of rehabilitation research center (1997), Beijing, once a day for 21 days and evaluated for the second time after treatment"
Comparison	**Amount of physical rehabilitation in groups:** differs **Categorisation of comparison:** 1. Physical rehabilitation versus no or limited physical rehabilitation (dose differs) (limited = < 50% of dose)
Funding & conflicts of interest	**Funding statement:** not reported **Conflict of interest statement:** not reported
Synthesis	**Quantitative synthesis:**Analysis 1.1; Analysis 1.2
Notes	Short Chinese study published in English—brief report only

**Table:** Xu 2003b

***Study characteristics***	
Methods	**Study aim:** "to investigate the recovery effect of early rehabilitation on hemiplegic limb following stroke" **Design:** parallel‐group RCT **Country:** China **Study recruitment and setting:** see Table **Involvement of patients/public/professionals/others:** no
Participants	**Study population (number randomised):** 180 **Inclusion criteria:** In‐patients diagnosed with stroke First stroke Met the diagnostic criteria of the 4th National Cerebrovascular Meeting **Exclusion criteria:** Diagnosed as light type (score of TMA > 85) Disturbance of consciousness Sensory aphasia **Participants:** detailed in Table **PROGRESS‐plus:** detailed in Table
Interventions	(1) Rehabilitation (n = 92) "The rehabilitation included facilitation of nerve and muscle, controlling of posture and functional training et al, one hour every day and continuously for four weeks" Length of intervention period: 4 weeks "The earliest rehabilitation training began after two days of disease and the latest began after ten days of disease (with the average of 6 days)" Number of sessions and length of individual sessions: "one hour every day" Intervention provider: not stated (2) No physical rehabilitation(n = 88) No intervention Length of intervention period: no intervention Number of sessions and length of individual sessions: no intervention Intervention provider: no intervention
Outcomes	**Independence in ADL scales:** Barthel Index **Motor function scales:** Fugl‐Meyer Assessment **Measures of balance:** NR **Measures of gait velocity:** NR **Length of stay:** NR **Adverse events:** NR **Other measures:** NR **Time points when outcomes were assessed:** "The assessment was given on the 28th day respectively before and after treatment"
Comparison	**Amount of physical rehabilitation in groups:** differs **Categorisation of comparison:** 1. Physical rehabilitation versus no or limited physical rehabilitation (dose differs) (limited = < 50% of dose)
Funding & conflicts of interest	**Funding statement:** not reported **Conflict of interest statement:** not reported
Synthesis	**Quantitative synthesis:**Analysis 1.1; Analysis 1.2
Notes	Short Chinese study published in English: brief report only This paper was written in English, and the extracts above are direct quotes. No definition of TMA is provided.

**Table:** Xu 2004

***Study characteristics***	
Methods	**Study aim:** to investigate the effectiveness of early rehabilitation on ADL and complications within a month after a first stroke **Design:** parallel‐group RCT **Country:** China **Study recruitment and setting:** see Table **Involvement of patients/public/professionals/others:** no
Participants	**Study population (number randomised):** 57 **Inclusion criteria:** Ischaemic or haemorrhagic stroke First ever stroke confirmed by CT or MRI scan < 80 years old GCS > 8 No physical deficits from other illnesses before stroke onset **Exclusion criteria:** Thrombolytic therapy or surgery for haemorrhage, subarachnoid haemorrhage, TIA, reversible ischaemic neurological deficit Worsening condition New tissue ischaemia or haemorrhage Mini Mental State Examination (MMSE) (illiteracy ≤ 17, primary learning ≤ 20,secondary learning and other sections ≤ 24) Severe loss of language ability affecting communication **Participants:** detailed in Table **PROGRESS‐plus:** detailed in Table
Interventions	(1) Rehabilitation (n = 30) "Both groups had similar medical treatment. The ‘rehabilitation' group underwent comprehensive treatment techniques focused on Bobath, including the following content: (i) passive ranging of all affected limbs with shoulder (extension, circumduction, various range of motion involving the scapular and torso regions), hand (wrist extension and extension exercises of all finger joints), hip and foot joints, from proximal to distal joints, from small to larger range of motion, seeking to achieve the largest range of motion within pain‐free threshold; (ii) rolling, sitting up training; (iii) bridging exercises; (iv) sitting and standing balance training; (v) getting up from bed training; (vi) gait training; (vii) ADL training" Length of intervention period: 1 month after stroke Number of sessions and length of individual sessions: 1/d, 40 to 50 minutes/session, 5/week Intervention provider: not stated but "on commencement of intervention, participants were taught appropriate positioning and family members were taught basic exercises so they could supervise participants" (2) No physical rehabilitation(n = 27) "Did not receive any rehabilitation training or guidance" Length of intervention period: no intervention Number of sessions and length of individual sessions: no intervention Intervention provider: no intervention
Outcomes	**Independence in ADL scales:** Barthel Index **Motor function scales:** Fugl‐Meyer Assessment **Measures of balance:** NR **Measures of gait velocity:** NR **Length of stay:** NR **Adverse events:** NR **Other measures:** "degree of deficit of neural function (DDNF)" **Time points when outcomes were assessed:** within 7 to 23 days after stroke and 1 month after stroke
Comparison	**Amount of physical rehabilitation in groups:** differs **Categorisation of comparison:** 1. Physical rehabilitation versus no or limited physical rehabilitation (dose differs) (limited = < 50% of dose)
Funding & conflicts of interest	**Funding statement:** not reported **Conflict of interest statement:** not reported
Synthesis	**Quantitative synthesis:**Analysis 1.1
Notes	Original study translated from Chinese to English by a member of our team (PLC)

**Table:** Xu 2013

***Study characteristics***	
Methods	**Study aim:** to explore the effect of medical exercise on paralysis rehabilitation of stroke patients **Design:** parallel‐group RCT **Country:** China **Study recruitment and setting:** see Table **Involvement of patients/public/professionals/others:** no
Participants	**Study population (number randomised):** 114 **Inclusion criteria:** Met the diagnostic criteria adopted by the 4th National Academic Conference on Cerebrovascular Disease Confirmed by CT or MRI **Exclusion criteria:** Severe cardiopulmonary disease History of dementia Unstable vital signs Unable to co‐operate **Participants:** detailed in Table **PROGRESS‐plus:** detailed in Table
Interventions	(1) Medical gymnastics "a set of simple and easy‐to‐learn medical gymnastics according to the characteristics of the patients, instructing and teaching the stroke hemiplegia patients and their families to carry out self‐function exercise" "Medical gymnastics training was implemented on the basis of the control group. A training group composed of bed doctors, responsible nurses, patients and their caregivers made an objective and systematic assessment of the patients' daily life ability and balance ability, and formulated a medical gymnastics training plan according to the condition of hemiplegia (see Table 1). The training process follows the principle of gradual progress and persistence. In the initial stage, it is not required to complete a full set of medical gymnastics every day, but choose 5 to 6 movements that the patient can complete for training, and pay attention to maintaining the balance of the body during various activities. During the training process, medical staff or their family members are required to accompany them, closely observe the changes of the patient's condition, and arrange the training time and training volume reasonably." Medical gymnastics training included seated leg exercises, sit‐to‐stand, standing balance exercises, walking, stairs. (2) Conventional rehabilitation "After receiving conventional drug treatment in the neurology department, the patient's vital signs are stable and the condition no longer progresses, and hemiplegia limb function training is performed, and general rehabilitation instructions are given, such as massage of the affected limb, maintaining a good posture, and turning over exercises. "
Outcomes	**Independence in ADL scales:** Barthel Index **Motor function scales:** NR **Measures of balance:** Berg Balance Scale **Measures of gait velocity:** NR **Length of stay:** NR **Adverse events:** NR **Other measures:** NR **Time points when outcomes were assessed:** before and after 1 month of treatment
Comparison	**Amount of physical rehabilitation in groups:** differs **Categorisation of comparison:** 5. Additional therapy + usual therapy versus usual therapy (dose differs)
Funding & conflicts of interest	**Funding statement:** not reported **Conflict of interest statement:** not reported
Synthesis	**Quantitative synthesis:**Analysis 5.1
Notes	This study was published in Chinese and translated into English using Google Translate.

**Table:** Xu 2015

***Study characteristics***	
Methods	**Study aim:** "to investigate the effect of electromyography stimulation (EMGS) combined with comprehensive rehabilitation training on muscle tension of paretic limb in poststroke hemiparetic patients" **Design:** parallel‐group RCT **Country:** China **Study recruitment and setting:** see Table **Involvement of patients/public/professionals/others:** no
Participants	**Study population (number randomised):** 40 **Inclusion criteria:** Met the diagnosis criteria developed at the 4th National Cerebrovascular Diseases Conference in 1995 Cerebral infarction or haemorrhage confirmed by CT or MRI Initial onset of stroke or having prior history of stroke with no residual neurological dysfunction Age between 40 and 66 years Stable vital signs with no cognitive deficits Able to communicate and full comprehension Physical dysfunction stable ≥ stage 3 hemiparesis Brunnstrom’s Scale for lower limbs Controlled blood pressure with no myocardial infarction, angina and with good heart function No other comorbidity that would restrict activities **Exclusion criteria:** Subarachnoid haemorrhage, TIA and reversible ischaemic neurological deficit Progression of disease (i.e. with occurrence of new cerebral infarction or haemorrhage) Seizures without effective control Functional deterioration or failure of vital organs including heart, lung, liver, kidney etc. Severe cognitive and communication impairments Lower limb bone or joint diseases **Participants:** detailed in Table **PROGRESS‐plus:** detailed in Table
Interventions	(1) Comprehensive rehabilitation "Electromyography‐triggered stimulation (EMGS) combined with comprehensive training in hemiplegia." EMGS ‐ 20 min treatment once a day for 5 days a week, plus: "Experimental group patients received 40 min comprehensive rehabilitation training once a day for 5 days a week as suggested in The Comprehensive Treatment of Patients with Hemiplegia" [Ref: "Davies PM. Steps to Follow: The Comprehensive Treatment of Patients with Hemiplegia. Berlin: Springer Science & Business Media; 2000."] (2) Control "Control group patients were treated with conventional therapy including neurotrophic medication and acupuncture treatment"
Outcomes	**Independence in ADL scales:** NR **Motor function scales:** Fugl‐Meyer assessment **Measures of balance:** NR **Measures of gait velocity:** NR **Length of stay:** NR **Adverse events:** NR **Other measures:** Functional Ambulation Categories, EMG of gastrocnemius **Time points when outcomes were assessed:** pre‐ and post‐treatment
Comparison	**Amount of physical rehabilitation in groups:** differs **Categorisation of comparison:** 1. Physical rehabilitation versus no or limited physical rehabilitation (dose differs) (limited = < 50% of dose)
Funding & conflicts of interest	**Funding statement:** not reported **Conflict of interest statement:** not reported
Synthesis	**Quantitative synthesis:**Analysis 1.2
Notes	

**Table:** Xu 2022

***Study characteristics***	
Methods	**Study aim:** "To study the effect of evidence‐based physical exercise on the physical function and daily life ability of stroke patients with hemiplegia" **Design:** parallel‐group RCT **Country:** China **Study recruitment and setting:** see Table **Involvement of patients/public/professionals/others:** no
Participants	**Study population (number randomised):** 160 **Inclusion criteria:** First onset with stroke confirmed by CT or MRI Had limb dysfunction Prior to stroke no other diseases caused disability No severe aphasia and cognitive impairment No serious organ diseases **Exclusion criteria:** Not reported **Participants:** detailed in Table **PROGRESS‐plus:** detailed in Table
Interventions	(1) Physical rehabilitation "the exercise group started one‐to‐one rehabilitation training 48‐72h after the onset of the disease. One time a day, 30‐45min each time, 4‐6 times a week. At the same time, explain to patients and their families the importance of rehabilitation and actively cooperate with rehabilitation.In this way, the concept of rehabilitation runs through the 24h daily management and rehabilitation lifestyle.5 The training method is based on the basic theory of exercise therapy for stroke hemiplegia. We formulate different treatment plans based on the initial evaluation of each patient. Specifically, it mainly includes: (1) Maintaining each joint’s normal range of motion to avoid the limitation of joint motion caused by joint braking, adopting a passive motion of the whole joint, and assisting active motion. Joint movement is mostly in the contralateralor prone position. It usually starts from the proximal joint in sequence.(2) Good limb placement is used in anti‐spasm mode. (3) Promote the recovery of the motor function of the affected limb through techniques such as functional electrical stimulation and acupuncture.(4) Trunk muscle training: a. Upper trunk flexion and rotation training. The patient lies on the contralateral side, and the therapist stands on the affected side. The therapist puts one hand on the affected side of the thorax and the other on the affected side of the scapula andinduces flexion and rotation of the upper trunk. b. Lower trunk flexion and rotation training. The patient is lying on the contralateral side. The therapist stands on the affected side. The hand or forearm is placed othe affected pelvis to induce flexion and rotation of the lower trunk.c. Exercise recovery in bed. Turnover, sit up, transfer, pelvic 6‐position training, etc. (5) Seat balance training: a. The patient takes a seat and shifts the center of gravity to the affected and healthy sides. b. Cross the legs with the diseased leg up and shift the center of gravity to the healthy side. (6) Standing balance training: a. Standing pelvic tilting forward and backward training. b. Support the sick leg, and perform abduction training with the healthy leg adducted. c. Support the sickleg and step on the steps for a healthy leg. (7) Preparation training before walking: a. The support phase stimulates the hip extensors;b. The swing phase stimulates the hip flexors. (8) Walking training: a. Promote hip joint extension and center of gravity shift. b. Instruct the torso to rotate and promote walking. c. Knee joint control training. d. Walk sideways, walk backward, and train up and downstairs. (9) ADLtraining." (2) No physical rehabilitation "Both the exercise group and the control group were treated with conventional neurology drugs in the acute phase." "Patients in both groups were treated for four weeks after their condition was stable for 48 hours"
Outcomes	**Independence in ADL scales:** Barthel Index **Motor function scales:** Fugl Meyer assessment **Measures of balance:** NR **Measures of gait velocity:** NR **Length of stay:** NR **Adverse events:** NR **Other measures:** NR **Time points when outcomes were assessed:** before and after 4 weeks of treatment
Comparison	**Amount of physical rehabilitation in groups:** differs **Categorisation of comparison:** 1. Physical rehabilitation versus no or limited physical rehabilitation (dose differs) (limited = < 50% of dose)
Funding & conflicts of interest	**Funding statement:** not reported **Conflict of interest statement:** "author declare no potential conflict of interest related to this article"
Synthesis	**Quantitative synthesis:**Analysis 1.1; Analysis 1.2
Notes	

**Table:** Xue 2006

***Study characteristics***	
Methods	**Study aim:** to determine the effectiveness of rehabilitation training of motor relearning programme plus Bobath on motor function, nerve function, and ADLs in people with stroke and hemiplegia **Design:** parallel‐group RCT **Country:** China **Study recruitment and setting:** see Table **Involvement of patients/public/professionals/others:** no
Participants	**Study population (number randomised):** 150 **Inclusion criteria:** Met the diagnostic standards about stroke set by the 5th National Academic Meeting for Cerebrovascular Disease Stroke diagnosis confirmed by CT and MRI Informed with the interventions and the items of evaluation **Exclusion criteria:** TIA, infarction of vertebral basilar artery and subarachnoid haemorrhage **Participants:** detailed in Table **PROGRESS‐plus:** detailed in Table
Interventions	(1) Rehabilitation(n = 78) "those in the training group received rehabilitation training by motor relearning program and Bobath technique" "The rehabilitation training began after the vital signs became stable within 24 hours to 3 days after attack for the patients with cerebral infarction and 48 hours to five days after attack for those with cerebral hemorrhage respectively" "The patients in the training group passively or actively learned, imitated and reinforced following the normal motor pattern of limbs. (i) Lying position: The patients should keep the anti‐spasm posture in the supine position, contralateral and ipsilateral lying positions, and the postures should be changed regularly; The patients should exercise each joint passively; turn the body over and move actively; They should also perform the bringing training, and the weight loading training for the affected upper limb. (ii) Sitting position: The patients should finish the conversion from a lying position to a sitting one independently; They also took the training in crawling position or kneeling position, as well as training for sitting balance of grade 3. (iii) Standing position: The patients should finish the conversion from a sitting position to a standing one independently; They also took the training for standing balance, weight loading training for the affected lower limb. The dorsiflexion of ankle was extended repeatedly. The walking training should be performed when the affected lower limb could support 2/3 of body mass. (iv) Walking training: In a standing position supported by the affected lower limb, the unaffected one stepped in small range forward and backward; Each process of balancing and controlling standing position was trained by supporting the body mass by the affected lower limb; In swinging position, the independent movement of the ipsilateral knee joint was trained in alternation of flexion and extension, in order to reach the results of without raising coxa, but relaxing knee, stepping with flexing knee and the dorsiflexion of ankle when the heel touch the ground" Length of intervention period: 1 month Number of sessions and length of individual sessions: "three times a day in the morning, at noon and in the evening respectively, 30 minutes for each time" Intervention provider: not stated (2) No physical rehabilitation group (n = 72) "All the patients were given routine treatments, including managing blood pressure, maintaining the balance of hydrolyte and electrolure, reducing intracranial pressure by dehydration, and venous injection of citicoline" Length of intervention period: no intervention Number of sessions and length of individual sessions: no intervention Intervention provider: no intervention
Outcomes	**Independence in ADL scales:** Modified Barthel Index **Motor function scales:** Fugl‐Meyer Assessment **Measures of balance:** NR **Measures of gait velocity:** NR **Length of stay:** NR **Adverse events:** NR **Other measures:** neurological deficit score **Time points when outcomes were assessed:** "patients were evaluated by the professional group (the fourth and fifth authors) before treatment and 1 month after treatment respectively"
Comparison	**Amount of physical rehabilitation in groups:** differs **Categorisation of comparison:** 1. Physical rehabilitation versus no or limited physical rehabilitation (dose differs) (limited = < 50% of dose)
Funding & conflicts of interest	**Funding statement:** not reported **Conflict of interest statement:** not reported
Synthesis	**Quantitative synthesis:**Analysis 1.1; Analysis 1.2
Notes	This paper was written in English, and the extracts above are direct quotes.

**Table:** Xu Wenyu 2012

***Study characteristics***	
Methods	**Study aim:** to explore the application of dynamic stereotyping principle in the walking training of stroke patients with hemiplegia **Design:** parallel‐group RCT **Country:** China **Study recruitment and setting:** see Table **Involvement of patients/public/professionals/others:** no
Participants	**Study population (number randomised):** 100 **Inclusion criteria:** Met the diagnostic criteria for stroke from the Fourth National Conference on Cerebrovascular Diseases Early stroke onset (i.e. within 2 weeks) Aged 35 to 70 years Stable vital signs No cognitive impairment Good auditory comprehension No serious heart disease or related complications affecting rehabilitation training **Exclusion criteria:** Disturbance of consciousness, poor hearing comprehension, and cognitive dysfunction Unable to co‐operate with training Heart, brain, lung, and other multiple organ failure Other diseases that restrict activities **Participants:** detailed in Table **PROGRESS‐plus:** detailed in Table
Interventions	Participants in both groups were treated with conventional rehabilitation methods, e.g. neuromuscular facilitation techniques. Two sessions/day (once in the morning and once in the afternoon); session duration: 45 mins delivered over 2 months. (1) Walking training "inertial guided gait rehabilitation training" ‐ treatment group received gravity/inertial principles to give gait training. (2) Conventional rehabilitation "conventional rehab gait training" ‐ conventional rehab with gait training, without any motivation.
Outcomes	**Independence in ADL scales:** Barthel Index **Motor function scales:** NR **Measures of balance:** NR **Measures of gait velocity:** NR **Length of stay:** NR **Adverse events:** NR **Other measures:** Functional Ambulation Categories **Time points when outcomes were assessed:** before and after treatment
Comparison	**Amount of physical rehabilitation in groups:** equivalent **Categorisation of comparison:** 3.(ii) Comparison of different approaches, (dose equivalent; with both/all groups also receiving other physical rehab)
Funding & conflicts of interest	**Funding statement:** not reported **Conflict of interest statement:** not reported
Synthesis	**Qualitative synthesis:** study does not fit into any of the comparisons explored within meta‐analyses.
Notes	This study was published in Chinese and relevant sections translated into English by our author team (PLC).

**Table:** Xu Yumei 2013

***Study characteristics***	
Methods	**Study aim:** "To investigate the effect of rehabilitation training on the balance and coordination function of stroke patients with hemiplegia" **Design:** parallel‐group RCT **Country:** China **Study recruitment and setting:** see Table **Involvement of patients/public/professionals/others:** no
Participants	**Study population (number randomised):** 40 **Inclusion criteria:** Not reported ("all cases were examined by CT or MRI. Except for motor dysfunction, there was no obvious cognitive impairment") **Exclusion criteria:** Not reported **Participants:** detailed in Table **PROGRESS‐plus:** detailed in Table
Interventions	(1)Balance training group "The programs adopted in the rehabilitation group mainly included: (1) Recumbent training: ① Heel‐knee‐shin test, in which the lower limbs were flexed and extended alternately when the heels were in contact with the bed surface. Lift the heel off the bed and alternate flexion and extension of both lower limbs; ② Place the heel accurately on the bed or the position specified by the therapist on the opposite lower limb; ③ Turn over on the bed; ④ Bridge exercise and bridge stepping; ⑤ Prone position Lower the torso to stretch; ⑥ four‐point kneeling position ‐ three‐point kneeling position ‐ two‐point kneeling position ‐ kneeling position exercises. (2) Seating training: ①Practice the correct sitting posture; ②Take turns to make the heel or toe slide forward, backward, left and right along the drawn "X"; ③Get up from the chair and sit down; ④Get down from the sitting position Shift the center of gravity in all directions; ⑤ Power bike training. (3) Standing exercises: ①Practice the maintenance and control of the correct standing posture through the posture mirror; ②Stand with your feet together ‐ stand with eyes closed ‐ stretch and move your upper limbs forward ‐ pass and catch the ball in a standing position ‐ pick up from the ground Object ‐ Turning from a standing position ‐ Loading one lower limb and placing the other foot on a 20cm‐high step ‐ Standing with two feet in front of one another ‐ Interference ‐ Standing on one leg. (4) Walking training: ① Weight loss plank walking training; ② Side walking training; ③ Walking training between parallel lines with a width of 35cm; ④ Forward walking training; ⑤ Weight bearing walking training; ⑥ Up and down stairs training. ⑦ Outdoor walking training. (5) Upper limb function: ① Rotation test; ② Finger test; ③ Finger test; ④ Hand clap test" (2) Conventional rehabilitation "The control group adopted conventional facilitation techniques and traditional Chinese medicine therapy proposed by Bobath, including exercise therapy for the upper and lower limbs on the affected side, peripheral sensory stimulation, motor control training, transfer, gait training, etc., as well as acupuncture and massage therapy." "The training was performed by uniformly trained therapists, 2 times a day, 45 minutes each time, 6 days a week; the rehabilitation course was 12 weeks. "
Outcomes	**Independence in ADL scales:** NR **Motor function scales:** NR **Measures of balance:** Berg Balance Scale **Measures of gait velocity:** NR **Length of stay:** NR **Adverse events:** NR **Other measures:** NR **Time points when outcomes were assessed:** before and after training
Comparison	**Amount of physical rehabilitation in groups:** differs **Categorisation of comparison:** 5. Additional therapy + usual therapy versus usual therapy (dose differs)
Funding & conflicts of interest	**Funding statement:** not reported **Conflict of interest statement:** not reported
Synthesis	**Quantitative synthesis:**Analysis 5.3
Notes	This paper was published in Chinese and translated using Google Translate. The dose of "conventional" rehabilitation is unclear; it has been assumed that the balance training programme was delivered in addition to the conventional rehabilitation.

**Table:** Yadav 2016

***Study characteristics***	
Methods	**Study aim:** "to compare the effects of specific balance strategy training programme with general balance training in individuals with chronic stroke" **Design:** parallel‐group RCT **Country:** India **Study recruitment and setting:** see Table **Involvement of patients/public/professionals/others:** no
Participants	**Study population (number randomised):** 24 **Inclusion criteria:** First stroke with duration > 6 months Able to follow verbal commands MMSE > 24 Able to walk independently (with/without assistive devices) **Exclusion criteria:** Not reported **Participants:** detailed in Table **PROGRESS‐plus:** detailed in Table
Interventions	(1) Specific strategy balance training "The exercise for group 1 was designed to focus on a specific task that addresses various aspects for balance including functional strength, flexibility, balance strategy practice, sensory integration and added attention demands during function and multi task practice. Various simple tasks are selected such as sit to stand and this task will be practiced using different heights of chairs, with or without upper limb assistance, balancing a cup with or without water on a saucer or while adding a cognitive task to the manual task. Each station task is graded to cater to various levels of ability so that participants can have the level of difficulty progressed to increase the challenges" (2) General balance exercises "The components of exercise in group 2 consisted of active stretching and strengthening of the upper and lower limb muscles, marching on spot, forwards, backwards and to the sides. This programme initially started with a low level of intensity (low frequency and repetitions) of individual exercises and was progressive over two weeks"
Outcomes	**Independence in ADL scales:** NR **Motor function scales:** NR **Measures of balance:** Berg Balance Test **Measures of gait velocity:** timed up and go test **Length of stay:** NR **Adverse events:** NR **Other measures:** NR **Time points when outcomes were assessed:** pre‐ and post‐training
Comparison	**Amount of physical rehabilitation in groups:** equivalent **Categorisation of comparison:**3.(i) Comparison of different approaches (dose equivalent; with no other physical rehab)
Funding & conflicts of interest	**Funding statement:** not reported **Conflict of interest statement:** not reported
Synthesis	**Quantitative synthesis:**Analysis 7.3; Analysis 7.4
Notes	

**Table:** Yan 2002

***Study characteristics***	
Methods	**Study aim:** to examine the effects of early rehabilitation on lower limb motor function in people with stroke and hemiplegia **Design:** parallel‐group RCT **Country:** China **Study recruitment and setting:** see Table **Involvement of patients/public/professionals/others:** no
Participants	**Study population (number randomised):** 78 **Inclusion criteria:** Not reported **Exclusion criteria:** Not reported **Participants:** detailed in Table **PROGRESS‐plus:** detailed in Table
Interventions	(1) Rehabilitation (n = 40) "Both groups of patients received conventional neurological drug treatment, treatment group after initial assessment commenced rehabilitative treatment procedure. The rehabilitative training procedure was as follows: based on the extent of the patient’s hemiplegia and affected limbs’ functional status, procedure was divided into 3 phases: early phase, rehabilitative treatment on bed, and rehabilitative treatment after leaving the bed" "(i) Early phase: commenced 48 h after patient was medically stable and neurological symptoms stopped progressing. Healthy limbs: therapists should be familiar with positioning of healthy limbs, and let family members understand the reason for doing so and to master these techniques. Upper limb: a pillow slightly higher than torso was placed below extended upper limb and maintained upper limb in supine position; Pelvis and lower limb: those with tendency for lower limb to buckle, while in lying position should have a pillow supporting the affected side of the limb to prevent excessive flexion. Pillows were used to prevent excessive abduction, external rotation. Participants with excessive ankle curvature or inversion used a splint board for positioning. Passive joint range of motion: from distal to proximal, 10x/joint, 2/day. Movement should be of ease, of short duration, used Brunnstrom technique’s rapid traction, stimulation through overpressuring of joints, 2/day, 15min/session" "(ii) rehabilitative treatment on bed: This phase commenced when participants possessed good sense of awareness and communication ability: using Bobath method of holding hands, with elbow extension, shoulder elevation to 90 degrees, using both upper limbs to lead during rolling, before turning the hips. After gaining independence in rolling, participants can commence bridging exercises, ankle dorsiflexion exercises. Sitting up from bed training: increase angle of bed to 30 degrees, once participants could tolerate longer than 30mins in the angle, the angle of bed is increased by 10 degrees, until participants can maintain at 90 degrees for 30 mins. Lying to sitting and sitting balance training. The above were practised 2/day, 30 min/session, increasing to 3‐4/day if participants had no discomfort" "(iii) Rehabilitative treatment after leaving the bed: In the rehabilitation centre, standing upright training ‐> forward, backward, left and right weight shifting training ‐> training by using the affected lower limb to support (single leg standing) ‐> limb loading of the affected lower limb (rising training, sitting down training, sit to stand training) ‐> ankle dorsiflexion ‐> anterior, posterior pelvic tilt training ‐> gait training with pelvic stabilisation (parallel bar ‐> flat ground ‐> slope ‐> stairs), 2/day, 1hr/session. Average length of treatment: 38 days" Length of intervention period: 38 days Number of sessions and length of individual sessions: session length dependent on technique applied (see above) Intervention provider: not stated (2) No physical rehabilitation (n = 38) "Received conventional neurological drug treatment but did not receive any standard rehabilitative treatment" Length of intervention period: no intervention Number of sessions and length of individual sessions: no intervention Intervention provider: no intervention
Outcomes	**Independence in ADL scales:** Barthel Index **Motor function scales:** NR **Measures of balance:** NR **Measures of gait velocity:** NR **Length of stay:** NR **Adverse events:** NR **Other measures:** Brunnstrom, 3‐level balance rating **Time points when outcomes were assessed:** within 24 hours before start of intervention and on the last day of intervention
Comparison	**Amount of physical rehabilitation in groups:** differs **Categorisation of comparison:** 1. Physical rehabilitation versus no or limited physical rehabilitation (dose differs) (limited = < 50% of dose)
Funding & conflicts of interest	**Funding statement:** not reported **Conflict of interest statement:** not reported
Synthesis	**Quantitative synthesis:**Analysis 1.1
Notes	Original study translated from Chinese to English by a member of our team (PLC)

**Table:** Yan 2015

***Study characteristics***	
Methods	**Study aim:** to investigate the effectiveness of standardised 3‐level rehabilitation therapy in hemiplegia, and its effect on improving joint activity and limb function recovery **Design:** parallel‐group RCT **Country:** China **Study recruitment and setting:** see Table **Involvement of patients/public/professionals/others:** no
Participants	**Study population (number randomised):** 60 **Inclusion criteria:** Met the diagnostic criteria for cerebrovascular disease Stroke confirmed by CT or MRI First onset Stroke at the blood supply site of the internal carotid artery, and no new lesion NIHSS ≤ 15 points Stable vital signs and conscious Provision of informed consent **Exclusion criteria:** Previous joint disease or neuromuscular disease affecting walking Severe structural heart disease Anxiety or depression Malignant tumours or other serious chronic diseases **Participants:** detailed in Table **PROGRESS‐plus:** detailed in Table
Interventions	(1) Three‐stage rehabilitation "received standardized three‐level rehabilitation therapy, as follows. First‐level rehabilitation therapy (acute phase): It is carried out during hospitalization; it mainly includes moderate exercise of the affected limb, bridge exercise, combined with neuromuscular facilitation techniques; instructs patients to perform basic daily activities, and trains family members to learn correct training methods to avoid Misuse or disuse syndrome. The patients rehabilitated once a day, 40 minutes each time, 5 times a week, and continued training for 2 months. Secondary rehabilitation therapy (pre‐recovery): The patient enters the recovery period after continuous training for 2 months, and further strengthens the primary rehabilitation training according to the patient's condition, strengthens standing and sitting training, bed exercise training, neuromuscular facilitation techniques, walking training, Stair walking training, and join the corresponding homework training. The therapist also gave stretching and antispasmodic manipulations to relieve the patient's muscle level; instructed the patient to use the affected limb correctly, and the patient recovered twice a day, 40 minutes each time, for 2 months. Tertiary rehabilitation therapy (late stage of recovery): The patient mainly continues to recover at home, including daily training on eating, washing hands, dressing, etc. The therapist visits the door once a week to guide the patient and their family members on the correct rehabilitation methods, and the family members are required to cooperate with the patient to complete each training session. Daily training exercises to incorporate rehabilitation methods into daily life." (2) Conventional rehabilitation "Bobath technique was used for upper limbs for active and passive movement; both legs were flexed to keep hip extension, hip lift and other bridge exercises; Rood inhibition and excitation Sexual stress training; shoulder, wrist, hip, knee, and elbow induced separation exercises; bed rolling exercises; standing balance exercises, motor relearning, and walking training. Rehabilitation 3 times a week, 20 to 40 minutes each time." "The total course of treatment for both groups was 6 months."
Outcomes	**Independence in ADL scales:** NR **Motor function scales:** Fugl Meyer assessment (upper and lower) **Measures of balance:** NR **Measures of gait velocity:** NR **Length of stay:** NR **Adverse events:** NR **Other measures:** joint range of motion, quality of life index **Time points when outcomes were assessed:** before and after rehabilitation
Comparison	**Amount of physical rehabilitation in groups:** differs **Categorisation of comparison:** 1. Physical rehabilitation versus no or limited physical rehabilitation (dose differs) (limited = < 50% of dose) (Note: the conventional rehabilitation group did receive physical rehabilitation, however this is judged to be less than 50% of the dose of the 3‐stage rehabilitation group)
Funding & conflicts of interest	**Funding statement:** not reported **Conflict of interest statement:** not reported
Synthesis	**Quantitative synthesis:**Analysis 1.1; Analysis 1.2
Notes	This study was published in Chinese and translated into English using Google Translate.

**Table:** Yang 2006

***Study characteristics***	
Methods	**Study aim:** "to examine the effectiveness of task‐oriented progressive resistance strength training on lower extremity strength and functional performance in chronic stroke subjects" **Design:** parallel‐group RCT **Country:** Taiwan **Study recruitment and setting:** see Table **Involvement of patients/public/professionals/others:** no
Participants	**Study population (number randomised):** 48 **Inclusion criteria:** Hemiparetic from a single stroke occurring at least a year earlier Currently not receiving any rehabilitation Able to walk 10 m independently without an assistive device Medically stable Able to understand instructions and follow commands **Exclusion criteria:** Medical condition that would prevent participation in the training programme Uncontrolled health condition for which exercise was contraindicated **Participants:** detailed in Table **PROGRESS‐plus:** detailed in Table
Interventions	(1) Task‐oriented progressive resistance strength training "Subjects in the experimental group participated in 30 min of task‐oriented progressive resistance strength training three times a week for four weeks. The progressive resistance strength training programme was designed as a circuit class,with subjects completing practice at a series of workstations. The workstations were designed to strengthen the muscles in the bilateral lower limbs in a functionally relevant way. The six workstations incorporated into the circuit were: (1) standing and reaching in different directions for objects located beyond arm’s length to promote loading of the lower limbs and activation of lower limb muscles; (2) sit‐to‐stand from various chair heights to strengthen the lower limb extensor muscles;(3) stepping forward and backward onto blocks of various heights to strengthen the lower limb muscles; (4) stepping sideways onto blocks of various heights to strengthen the lower limb muscles; (5) forward step‐up onto blocks of various heights to strengthen the lower limb muscles; (6) heel(s) raise and lower while maintaining in a standing posture to strengthen the plantar‐flexor muscles.14 Each workstation was 5 min in duration for each exercise class." (2) No treatment "did not receive any rehabilitation training"
Outcomes	**Independence in ADL scales:** NR **Motor function scales:** NR **Measures of balance:** NR **Measures of gait velocity:** gait velocity, 6‐minute walk test, timed up and go test **Length of stay:** NR **Adverse events:** NR **Other measures:** muscle strength, gait analysis, step test **Time points when outcomes were assessed:** before and after intervention
Comparison	**Amount of physical rehabilitation in groups:** differs **Categorisation of comparison:** 1. Physical rehabilitation versus no or limited physical rehabilitation (dose differs) (limited = < 50% of dose)
Funding & conflicts of interest	**Funding statement:** "This study was supported by the National Health Research Institutes of the Republic of China (grant no. NHRI‐EX93‐9129EI)" **Conflict of interest statement:** not reported
Synthesis	**Quantitative synthesis:**Analysis 1.4
Notes	

**Table:** Yang 2017

***Study characteristics***	
Methods	**Study aim:** "to determine the therapeutic effect of electroacupuncture combined with the Bobath technique on the lower limb motor function in stroke patients" **Design:** multi‐arm RCT (3‐arms) **Country:** China **Study recruitment and setting:** see Table **Involvement of patients/public/professionals/others:** no
Participants	**Study population (number randomised):** 87 **Inclusion criteria:** Met diagnostic criteria for stroke and confirmed by MRI or CT First stroke and lower limb impairment Conscious and able to co‐operate and complete treatment Provides consent **Exclusion criteria:** Suffered cranial trauma or encephalitis Other conditions e.g. heart, liver and kidney disease No lower limb impairment after stroke and still able to take care of themselves Arthritis Other neurological condition **Participants:** detailed in Table **PROGRESS‐plus:** summarised in Table
Interventions	All participants received conventional medical treatment (e.g. low‐dose aspirin, lipid‐lowering drugs and/or anti‐hypertensives and allocated into one of 3 interventions: (1) Bobath alone Involved correct placement of affected limbs, exercises to improve spasticity of limbs and promote the extension of movements to form voluntary movements. Lower body training involved lower body flexion and extension, single leg and double leg bridge, knee joint flexion and extension, ankle dorsiflexion, skeletal joint flexion, and extension facilitation exercises. ADL training was also provided including training stroke survivors to walk, go to the toilet, go up and down stairs, eat, undress. Upper body training was also delivered as part of this intervention and involved cross‐hands lift and swing, shoulder and elbow joint extension, forward flexion and external rotation training, wrist joint flexion to the back of the wrist, thumb to finger and knuckle flexion and extension exercises. Sessions were 45 mins length and delivered once/day. (2) Electroacupuncture stimulation Selected acupoints on the lower limbs of the stroke survivor involved the insertion into multiple acupoints depending on underlying condition, (gently twirled, lifted and inserted, and taken out after the stroke survivor gets Qi). Three acupoints were pierced with an electroacupuncture stimulator (Xinsheng G6805). Type I excitation, frequency 10 Hz. The current intensity was gradually increased during stimulation and stopped when the stroke survivor's muscles contract. The intervention was delivered once a day (20 mins / session) for a total of 12 sessions. (3) Bobath + electroacupuncture A combination of interventions as described in 1 and 2 above. Comparison of relevance to this review: (3) vs (2)
Outcomes	**Independence in ADL scales:** modified Barthel Index **Motor function scales:** Fugl‐Meyer assessment, Motor Assessment Scale **Measures of balance:** NR **Measures of gait velocity:** NR **Length of stay:** NR **Adverse events:** NR **Other measures:** Functional Ambulation Categories **Time points when outcomes were assessed:** before and after treatment
Comparison	**Amount of physical rehabilitation in groups:** differs **Categorisation of comparison:** 5. Additional therapy + usual therapy versus usual therapy (dose differs) (This study has been categorised as additional therapy (Bobath) plus usual therapy (electroacupuncture) versus usual therapy; arguably electroacupuncture is not "usual therapy", but has been categorised this way as it has been delivered to both groups).
Funding & conflicts of interest	**Funding statement:** Scientific Research Project of Liaoning Provincial Department of Education (LK201655) **Conflict of interest statement:** not reported
Synthesis	**Quantitative synthesis:**Analysis 5.1; Analysis 5.2
Notes	Paper is in Chinese. Extracted data based on English abstract only.

**Table:** Yang Aiguo 2015

***Study characteristics***	
Methods	**Study aim:** to explore the effect of Bobath therapy combined with acupuncture on the recovery of lower extremity function in the flaccid stage of stroke **Design:** multi‐arm RCT (3‐arm) **Country:** China **Study recruitment and setting:** see Table **Involvement of patients/public/professionals/others:** no
Participants	**Study population (number randomised):** 144 **Inclusion criteria:** Met the diagnostic criteria established by the 4th National Cerebrovascular Disease Conference **Exclusion criteria:** Not reported **Participants:** detailed in Table **PROGRESS‐plus:** detailed in Table
Interventions	(1) Bobath + acupuncture "Bobath manipulation therapy: Instruct the patient to place the good limbs, adopt various excitability‐promoting manipulations, use various sensory inputs to promote the contraction and movement of the flaccid muscles, and squeeze and stretch the joints, muscles, and ligaments of the lower limbs to improve the Increase proprioceptive stimulation and enhance the contraction of muscles around the joints; passive movement of the affected limb joints, while performing resistance exercise on the unaffected lower limb, instruct the patient to perform corresponding adduction or abduction movement of the affected lower limb, if the patient has voluntary movements Minimize the use of primitive reflexes and joint responses afterwards; double bridge training assisted by a therapist, adjust the flexion angle of the patient’s knees to improve the muscle strength and muscle tone of the quadriceps femoris and triceps calf on the affected side; flexion and extension of the lower limbs on the affected side Control training, weight‐bearing training for the lower limbs on the affected side; for those with relaxation of the abdominal core muscles, assisted lower pelvic tilt training, assisted sit‐ups and rotation training to the lateral body axis; under the premise of core control, the limbs should be flexed or stretched at the same time Exercise training, under the premise of core control, do alternate flexion and extension exercises of the lower limbs; perform auxiliary‐active stretching exercises for the affected trunk in a sitting position, and perform flexion training of the affected lower limbs under the premise of core control; in a standing position, feet together, unsupported standing training, standing turning and looking back; unsupported standing with one foot on a step, and repeated lower extremity cross‐steps on the non‐paralyzed side. Bobath manipulation therapy was performed for 45 minutes each time, once a day, 15 times as a course of treatment, with an interval of 3 days, for a total of 2 courses of treatment. " Acupuncture as below. (2) Acupuncture "Acupuncture: body acupuncture, select Jianliao, Quchi, Shousanli, Waiguan, Yangchi, Hegu, Houxi points on the affected side for the upper limb, and select Fengshi, Xuehai, Xueyangguan and Yangling on the affected side for the lower limb. Spring, Zusanli, Shangjuxu, Fenglong, Chengshan, Zhaohai, Juegu, Jiexi, Xiaxi points, abdominal acupoints are selected according to Bo Zhiyun abdominal acupuncture....... both body acupuncture and scalp acupuncture are kept for 30 min, once a day, 15 times as a course of treatment, the course of treatment is intermittent for 3 days, a total of 2 treatments are given course of treatment" (3) No treatment "Routine drug therapy" Comparison of relevance to this review: (1) versus (2)
Outcomes	**Independence in ADL scales:** Barthel Index **Motor function scales:** Fugl‐Meyer assessment **Measures of balance:** NR **Measures of gait velocity:** NR **Length of stay:** NR **Adverse events:** NR **Other measures:** NR **Time points when outcomes were assessed:** 14 and 28 days after treatment
Comparison	**Amount of physical rehabilitation in groups:** differs **Categorisation of comparison:** 1. Physical rehabilitation versus no or limited physical rehabilitation (dose differs) (limited = < 50% of dose) [note: both treatment groups receive acupuncture]
Funding & conflicts of interest	**Funding statement:** not reported **Conflict of interest statement:** not reported
Synthesis	**Quantitative synthesis:**Analysis 1.1; Analysis 1.2; Analysis 2.1; Analysis 2.2
Notes	This study was published in Chinese and translated into English using Google Translate.

**Table:** Yang Jian 2007

***Study characteristics***	
Methods	**Study aim:** to observe the effect of individual initiative rehabilitation on motion function and ADL in hemiplegia after stroke **Design:** parallel‐group RCT **Country:** China **Study recruitment and setting:** see Table **Involvement of patients/public/professionals/others:** no
Participants	**Study population (number randomised):** 60 **Inclusion criteria:** Met the diagnostic criteria established by the 4th National Cerebrovascular Disease Conference and stroke confirmed by MRI or CT Aged between 40 and 80 years Stroke onset between 2 and 4 weeks First ever stroke accompanied by hemiplegia Not receiving rehabilitation before entering the study No cognitive deficits which could impact on participation in the study Able to provide signed consent **Exclusion criteria:** Uncontrolled medical conditions Malignant tumours Deficits in communication and cognition Mental health issues Visual deficits Frailty Issues which may negatively impact on participation in intervention or trial **Participants:** detailed in Table **PROGRESS‐plus:** detailed in Table
Interventions	(1) Rehabilitation "Active" motor rehabilitation Includes: Bobath approach, motor relearning, Rood, speech therapy. Acupressure, "Chinese herb fumigation & steaming therapy". Exercise therapy is mainly based on the Bobath technique, combined with motor relearning programme as well as utilising Rood and speech and language therapy. Training was individualised based on stage of recovery. Exercises included optimal limb positioning rehabilitation training to develop and maintain correct postures in daily life (e.g. eating, drinking, grooming, toileting, etc.). Body position change, transfer training (use adjustable bed, stand up bed, etc. for body position change training). Various types of bridge training (double bridge, single bridge, variant single bridge, etc.). Maintenance of range of motion of joints (full range of motion of all joints). Multiple types of stretching exercises including anti‐spasticity training and limb strengthening training (e.g. crossing hands and holding knees, leaning the head as far as possible to the chest, shaking the body back and forth from side to side to relieve the spasm of the lower limbs). Facilitation training of voluntary movement of upper and lower limbs. Including controlling the paretic elbow, instructing the affected elbow to complete active flexion and extension and circular movement of the paretic shoulder, facilitation and stretching of fingers, etc. Facilitation training of lower limb follow‐up exercise includes joint control training, facilitation training of flexion lower limbs, and assisted stretching of lower limbs. Loading training using weights for upper and lower body. Strengthening training of the upper and lower limbs extension movement and the control training of the upper and lower limbs. Extension exercises of the upper limbs. Stroke survivors while in the sitting position completed several extension exercises by flexing of the paretic shoulder and extension of the affected elbow. This included flexion of the shoulder and elbow, and then asking the stroke survivor to touch their opposite shoulder to complete the flexion, abduction, and adduction of the paretic shoulder. Rotation movement involved placing the paretic hand on the non‐paretic shoulder, lifting the elbow, touch the forehead with the paretic elbow, and then lowering it so that the paretic elbow touches the chest, adduct the heart and shoulders, and inwardly rotate the stroke survivor. Ask them to pat the healthy side with the affected hand shoulder joint, then return to the starting position. Repeat: 7 to 10 times. Upper limb control training: (a) stretch the affected elbow and flex the affected shoulder to 90 degrees, holding for 10 seconds if possible. Reduce the angle of flexion of the paretic shoulder if 90 degrees is not possible; (b) Upper limb extension exercise facilitation training. Ask the stroke survivor to stand or sit facing the wall with their shoulders bent 90 degrees, extend their elbows, support the wall with the palms, pushing against the wall with the strength of their shoulders; (c) ask the stroke survivor while sitting or standing to use the upper limb of the non‐paretic side to drive the upper limb of the paretic side to lift up, stretching their fingers with the palm facing upwards and maintain the extension of the elbow joint, extend the trunk of the paretic side, and shift the centre of gravity of the body to the paretic side. Intensive training of lower limb extension exercise. Comprised of (a) stretching of the paretic knee joint and facilitation training of joint abduction; (b) ankle dorsiflexion training in a sitting position, the therapist controls the affected toes with one hand to prevent hyperextension, stretches the ankle back, controls the affected knee with the other hand, instructs the affected leg to be lifted upwards, and placed on the knee of the other leg, and then lifts the affected leg upwards and places it on the ground, the lower limbs take turns to do the above actions alternately (b) Lower extremity control training: lying down with the therapist controlling the toes (paretic side) with one hand to dorsiflex the ankle joint, and control the paretic knee with the other hand to prevent hyperextension of the affected knee joint, externally rotate the joint; (c) knee joint control training: lying down with the therapist controlling the toes (paretic side) with one hand to extend the ankle joint, and controls the paretic knee with the other hand, fixes the thigh on the bed, and instructs the patient to extend or flex the knee joint at any angle. When the knee on the paretic side cannot be extended, the quadriceps arm can be tapped; when the knee cannot be flexed, the extensor leg can be tapped. Continue to flex and extend the knee on the paretic side; (d) lying down with knees up and flexed, and foot placed downward, put a piece of paper between knees, instruct stroke survivor to do double bridge exercise for a few seconds after the hips are raised, and do not let the paper fall at the same time; (e) lower limb extension exercise facilitation training, tapping and knee joint flexion facilitation training; (f) Put the hand on the paretic big toe and lift the front foot upwards, so that the affected ankle is dorsiflexed and the heel touches the ground, and the ankle does not appear to be flexed. The therapist instructs the patient to move the centre of gravity to the healthy side and fully extend the joint. The knee joint must not be hyperextended; (g) stroke survivor leaning against the wall and doing wall slides; (h) standing practice in various positions and directions; (i) therapist controls the paretic knee with one hand, and the other hand to extend the ankle and slowly lift it up for foot clearance and putting it back into the original position. Other activities: anti‐spasticity treatment using Chinese herbal medicine; sitting standing, balance strengthening and intensifying practise including: knee‐hand balance training, kneeling balance training, intensifying balance training, control of centre of mass, pre‐gait training, gait training, gait correction and training for going up and down stairs; gymnastics in which the non‐paretic limb helps voluntary movement on the paretic side; use of assistive devices such as home‐based practice, ADL training and orthotics; language and cognitive training; counselling, care, support; application of traditional physical therapy and electromyographic biofeedback; family rehabilitation physical fitness guidance. (2) Attention control "Passive healing group" ‐ passive transfers from bed to chair, sitting up, "passive gait training", and joint range of motion. The primary aim is passive movement, and not active training. Use of assisted devices or technologies. Postural correction/positioning. If the patient self‐initiates, then this cannot be ruled out, but the focus is passive movement.
Outcomes	**Independence in ADL scales:** Functional Independence Measure (FIM) **Motor function scales:** Fugl‐Meyer Assessment **Measures of balance:** NR **Measures of gait velocity:** NR **Length of stay:** NR **Adverse events:** NR **Other measures:** Clinical Neurological Score **Time points when outcomes were assessed:** NR
Comparison	**Amount of physical rehabilitation in groups:** equivalent **Categorisation of comparison:** 2. Physical rehabilitation versus attention control (dose of physical rehab differs; dose of intervention equivalent)
Funding & conflicts of interest	**Funding statement:** not reported **Conflict of interest statement:** not reported
Synthesis	**Quantitative synthesis:**Analysis 3.1; Analysis 3.2
Notes	This study was published in Chinese and relevant sections translated into English by our author team (PLC).

**Table:** Yang Zhihong 2015

***Study characteristics***	
Methods	**Study aim:** to explore the clinical effect and promotion value of the three‐level rehabilitation nursing model applied to stroke patients **Design:** parallel‐group RCT **Country:** China **Study recruitment and setting:** see Table **Involvement of patients/public/professionals/others:** no
Participants	**Study population (number randomised):** 74 **Inclusion criteria:** Confirmed stroke using CT and MRI diagnosis **Exclusion criteria:** Poor compliance Severe hepatic and renal insufficiency Severe mental health conditions Disturbance of consciousness **Participants:** detailed in Table **PROGRESS‐plus:** detailed in Table
Interventions	(1) Three‐stage rehabilitation "the three‐level rehabilitation nursing model to care for the patients on the basis of traditional nursing. ①First‐level rehabilitation nursing: a. Maintain the correct body position. Whether the patient is lying on the back or the side, the head should be raised by 15°~30°, the upper forearm should be semi‐flexed, and the hand should hold a round object or cloth roll. b. Conduct psychological counseling. Due to the acute onset of stroke patients and the existence of different degrees of limb dysfunction at the same time, it is easy to cause patients to be anxious, fearful, and emotional. Establish the determination and confidence to overcome the disease subjectively, and actively cooperate with medical staff to implement treatment. c. Prevention of complications. Nursing staff pat the patient's back every 3 hours for 20 minutes each time to keep the patient breathing smoothly and prevent phlegm and suffocation; change the patient's position every 2 hours, and perform a limb and muscle massage to keep blood circulation smooth and prevent suffocation. Ischemic necrosis of limbs; guide patients to develop good defecation habits, and use drugs to relieve constipation when necessary. d. Keep the room clean and hygienic, ventilate frequently, and adjust the indoor temperature appropriately, so that patients can always maintain a clean, quiet, comfortable and relaxed treatment environment. ②Second‐level rehabilitation nursing: The second‐level rehabilitation nursing is the continuation of the first‐level rehabilitation nursing. After the patient's vital signs are stabilized, the patient's physical function strengthening training is strengthened. a. 3‐4 times a day, exercise the patient's limbs for 30 minutes each time. b. After a certain recovery, guide the patient to walk outdoors, gradually get rid of the help of nursing staff and family members, and exercise independently. ③Third‐level rehabilitation nursing: Medical staff and family members formulate a scientific and reasonable three‐phase rehabilitation nursing plan according to the specific situation of the patient, which is supervised and guided by the patient's family after the patient is discharged. During the tertiary rehabilitation nursing period, the medical staff visited the home for follow‐up once a week, and adjusted the rehabilitation plan according to the actual rehabilitation situation of the patients. Instruct the patient's family members to pay attention to the organic combination of psychological care and rehabilitation care in the tertiary rehabilitation care of the patient, so that the patient can use the remaining limb function as much as possible, exercise scientifically, and reduce the occurrence of disease sequelae." (2) Routine care "The control group used traditional nursing methods to care for the patients."
Outcomes	**Independence in ADL scales:** NR **Motor function scales:** Fugl Meyer motor function scale **Measures of balance:** NR **Measures of gait velocity:** NR **Length of stay:** NR **Adverse events:** NR **Other measures:** NR **Time points when outcomes were assessed:** 2 weeks, 1 month, 3 months, 6 months, and 12 months
Comparison	**Amount of physical rehabilitation in groups:** differs **Categorisation of comparison:** 1. Physical rehabilitation versus no or limited physical rehabilitation (dose differs) (limited = < 50% of dose)
Funding & conflicts of interest	**Funding statement:** not reported **Conflict of interest statement:** not reported
Synthesis	**Qualitative synthesis:** results presented in a format not suitable for quantitative analysis.
Notes	This study was published in Chinese and translated into English using Google Translate.

**Table:** Yazici 2021

***Study characteristics***	
Methods	**Study aim:** "to investigate the effects of NDT‐B approach on functional recovery in patients with acute ischemic stroke" **Design:** parallel‐group RCT **Country:** Turkey **Study recruitment and setting:** see Table **Involvement of patients/public/professionals/others:** no
Participants	**Study population (number randomised):** 39 **Inclusion criteria:** > 18 years of age Disability level of ≤ 4 according to modified Rankin Scale Able to respond to verbal commands **Exclusion criteria:** History of previous stroke Other progressive neurological disturbances Acute coronary disease Severe heart failure Rheumatologic diseases Fracture or an acute orthopaedic disorder **Participants:** detailed in Table **PROGRESS‐plus:** detailed in Table
Interventions	(1) Neurodevelopmental ‐ Bobath "NDT‐B principles and exercises were performed daily for five days a week, 60 minutes per session. The participants were included in the rehabilitation program as early as possible and the program continued until the participants were discharged. The program was initiated according to the principles of the Bobath method by an experienced NDT‐B physiotherapist. NDT‐B exercises were implemented according to the status of the participants and were used to maintain and improve muscle strength and endurance. Both the unaffected and affected side were included in rehabilitation. The exercises given were designed to be simple, understandable, task‐oriented and repetitive in accordance to the Bobath approach and were performed in supine, sitting and standing positions. Mobilization was initiated in the first session and in accordance to their conditions, the participants were mobilized in sitting or standing. Mobilization level was gradually increased by changing the participants’ position (sitting on the edge of the bed, sitting in a chair, walking) and increasing duration and walking distance. Alongside basic extremity exercises, weight transfer exercises and balance training were applied. During the exercises the importance of the quality of the movement was emphasized and external support was provided if necessary. The patient was asked to actively participate in the exercises. In order to prevent motor amnesia and neglect of the affected side correct positioning and sensory input were provided. Additionally, the patient and their caregivers were trained about resting positions, transfers and walking. Participants and their caregivers were asked to repeat the given exercises at least twice during the day and whether the exercises were performed or not was checked the next session" (2) Standard rehabilitation "The standard rehabilitation sessions were performed by clinical physiotherapists according to the hospital routine, five days a week. The standard rehabilitation consisted of joint range of motion exercises in‐bed and bedside sitting and walking according to the participants’ functional capacity."
Outcomes	**Independence in ADL scales:** Barthel Index **Motor function scales:** NR **Measures of balance:** Berg Balance Scale **Measures of gait velocity:** NR **Length of stay:** hospitalisation period **Adverse events:** NR **Other measures:** Stroke Rehabilitation Assessment of Movement Scale (STREAM), Trunk Impairment Scale **Time points when outcomes were assessed:** baseline and at discharge
Comparison	**Amount of physical rehabilitation in groups:** equivalent **Categorisation of comparison:** 3.(i) Comparison of different approaches (dose equivalent; with no other physical rehab) (Note: dose of standard rehabilitation is not reported; this is assumed to be similar to that of the Bobath rehabilitation)
Funding & conflicts of interest	**Funding statement:** "this study received no financial support" **Conflict of interest statement:** "no conflict of interest was declared by the authors"
Synthesis	**Qualitative synthesis:** outcomes measured at hospital discharge, rather than at a specific time point, therefore not suitable for inclusion within meta‐analysis. Hospital discharge data presented as medians only, so not suitable for analysis.
Notes	

**Table:** Ye Dayong 2010

***Study characteristics***	
Methods	**Study aim:** to observe the effect of tertiary rehabilitation therapy on lower limb motor function and walking ability of stroke patients **Design:** parallel‐group RCT **Country:** China **Study recruitment and setting:** see Table **Involvement of patients/public/professionals/others:** no
Participants	**Study population (number randomised):** 60 **Inclusion criteria:** First stroke with onset within 1 week Stroke confirmed by CT or MRI Lower limb motor dysfunction Stable vital signs Clear‐headed Able to sign informed consent **Exclusion criteria:** Not reported **Participants:** detailed in Table **PROGRESS‐plus:** detailed in Table
Interventions	(1) Three‐stage rehabilitation (level 3) First‐level rehabilitation treatment: delivered in first month usually in the neurological/surgical wards. In addition to medical and surgical treatment, stroke survivors were given bedside early rehabilitation intervention once they were medically stable. Rehabilitation was provided by rehabilitation therapists, and the methods also taught to the stroke survivors and nurses to assist with rehabilitation training. Treatment comprised: posture placement, passive limb training, nerve facilitation, active movement training of non‐paretic limbs, supine sit‐up, sitting balance and standing training, combined with myoelectric biofeedback therapy. The standing bed was used for postural adaptation training. Training is 1 session/day with each session lasting 45 minutes, and 5 days/week. Secondary rehabilitation treatment: 2 to 3 months after stroke onset and usually delivered mainly in the rehabilitation medicine ward. Treatment mainly includes standing and balance training, single leg standing training, walking training, and stair climbing training with the main purpose of improving the patient's walking function. Training is 1 session/day with each session lasting 45 minutes, and 5 days/week. Third‐level rehabilitation: usually 4 to 6 months after the onset of the stroke and is carried out in the community. Training is based on stroke survivors ADLs, strengthening gait training by walking outside (e.g., on pavements), walking over obstacles, and improving the pace of walking, to improve ability to walk safely and independently. Two sessions/day, with each session lasting 30 to 45 min, and training 5 days/week. Treatment is mainly carried out by trained full‐time rehabilitation instructors from the Disabled Persons’ Federation. Rehabilitation. Medicine therapists come to guide patients once every 2 weeks, and stroke survivors can also go to the limited rehabilitation medicine outpatient clinic to receive systematic rehabilitation guidance. (2) No physical rehabilitation Patients in the control group received first‐level and second‐level rehabilitation in addition to routine neurological and surgical treatment.
Outcomes	**Independence in ADL scales:** NR **Motor function scales:** Fugl‐Meyer assessment **Measures of balance:** NR **Measures of gait velocity:** NR **Length of stay:** NR **Adverse events:** NR **Other measures:** Functional Ambulation Categories (Holden) **Time points when outcomes were assessed:** NR
Comparison	**Amount of physical rehabilitation in groups:** differs **Categorisation of comparison:** 1. Physical rehabilitation versus no or limited physical rehabilitation (dose differs) (limited = < 50% of dose)
Funding & conflicts of interest	**Funding statement:** not reported **Conflict of interest statement:** not reported
Synthesis	**Quantitative synthesis:**Analysis 1.2
Notes	This study was published in Chinese and relevant sections translated into English by our author team (PLC).

**Table:** Yelnik 2008

***Study characteristics***	
Methods	**Study aim:** to compare 2 different rehabilitation strategies to improve balance following a stroke **Design:** parallel‐group RCT **Country:** France **Study recruitment and setting:** see Table **Involvement of patients/public/professionals/others:** no
Participants	**Study population (number randomised):** 68 **Inclusion criteria:** Hemiplegia following a single hemispheric stroke due to an infarct or haemorrhage Confirmed by CT or MRI Stroke within 3 to 15 months prior to entry At onset of stroke, subjects had to be unable to walk for at least 2 weeks, but not exceeding 3 months (i.e. defined as the ability to walk at least 50 metres with an orthosis or cane if needed (but without human assistance) Aged < 80 years old Ambulatory Living at home **Exclusion criteria:** Previous history of walking disorder Cognitive disorders that may affect comprehension of the rehabilitation programme History of a vestibular disorder **Participants:** detailed in Table **PROGRESS‐plus:** detailed in Table
Interventions	(1) Neurodevelopmental technique (NDT) (n = 35) "Based on global sensory motor rehabilitation derived from the neurodevelopmental concepts described by Bobath, more attention paid to the quality of the gesture and gait control, the spasticity, and abnormal movement inhibition than to the quantity of exercise and an increase of the difficulty from one session to another depending on the ability of the patients" "Sessions 1 to 4: Exercises conducted on the Bobath platform, weight shifting, waist dissociation, pelvis control, crawling, turning over, four footing, and standing on the knees. Sessions 5 to 8: Exercises on the edge of the platform in sitting position, transfers from lying to sitting, sitting to standing, sitting on the platform to a chair, upper limb used for bearing. Analytic exercises for upper limb were associated for a maximum of one third of the session. Sessions 9 to 20: Walking in the corridor and on the steps, control of the weight bearing and shifting, quality of the heel strike, knee control, and waist dissociation" "During the 20 sessions, visual deprivation, head movements, or training with unstable bases of support were forbidden for the progression of exercise difficulties" Length of intervention period: "Physical therapy had to begin within 7 days, and conducted 5 days a week for the following 4 weeks" Number of sessions and length of individual sessions: total of 20 successive sessions conducted 5 days a week "Each session lasted 60 to 70 minutes, depending on the rest required, and included 5 minutes for spasticity inhibition, 40 to 45 minutes of exercises specific to the session, 10 to 20 minutes of rest distributed throughout the session, and related to need" Intervention provider: not stated (2) Multisensory (n = 33) "Physical rehabilitation based on the manipulation of the sensory information required to maintain balance, attention being paid to the amount of exercise, that is, duration and intensity, rather than the quality of the movement. Most of the exercises were conducted in visual deprivation, thus challenging the selection and synthesis by the brain of vestibular and somatosensory information" "Each type of exercise was related to the patient’s progress,with progression under visual control for repetition of the exercises, then as much under visual deprivation as possible, and using unstable planes and foam ground‐sheet, tilting the head back, rightward and leftward. The duration of the exercises under visual deprivation was not exactly fixed and took approximately half of the session" "The exercises had to be repeated for patients to learn them and moreover improve their performance in terms of duration or intensity by slowly increasing the difficulty. There were 4 types of modalities, conducted as follows: sessions 1 to 4, modality 1; sessions 5 to 8, modality 2; sessions 9 to 20, by alternating modality 3 once and modality 4 twice. Modality 1: On the foam Bobath platform, four footing, standing on the knees, anteroposterior and lateral weight shifting, moving objects with the upper limb, external destabilization. Modality 2: Sitting on the edge of the platform and sitting on a ball, weight shifting, upper limb movements, moving objects with the upper limb, external destabilizations. Modality 3: Static standing with feet together, tandem position, one foot standing, control of weight shifting, moving objects with upper limbs, external destabilizations. Modality 4: Walking with movements of the upper limbs, while speaking, with external destabilization, walking laterally and backward, 10 minutes of treadmill training without upper limb support, opening eyes at various speeds, closing eyes at constant speed. In each modality, the variations that can be used were head movements, foam support, unstable platform, rolling skate, irregular floor, and constant visual deprivation" Length of intervention period: "Physical therapy had to begin within 7 days, and conducted 5 days a week for the following 4 weeks" Number of sessions and length of individual sessions: total of 20 successive sessions conducted 5 days a week "Each session lasted 60 to 70 minutes, depending on the rest required and included 5 minutes for spasticity inhibition, 30 to 35 minutes to specific modalities, 10 minutes of walking and stepping, and 10 to 20 minutes of rest distributed throughout the session" Intervention provider: not stated
Outcomes	**Independence in ADL scales:** Functional Independence Measure **Motor function scales:** NR **Measures of balance:** Berg Balance Scale **Measures of gait velocity:** speed of walking **Length of stay:** NR **Adverse events:** NR **Other measures:** posturographic limits of stability, percentage of double‐stance phase, time to climb 10 steps and return, daily time of walking (minutes), Nottingham Health Profile, security sensation during walking, number of falls since stroke **Time points when outcomes were assessed:** "The first visit for evaluation (day 0 [D0]) was conducted by one of the blinded evaluators. Posttreatment evaluation (day 30 [D30]) was carried out within 7 days of the end of the physical rehabilitation program. The second posttreatment evaluation (day 90 [D90]) was carried out 3 months after the first evaluation"
Comparison	**Amount of physical rehabilitation in groups:** equivalent **Categorisation of comparison:** 3.(i) Comparison of different approaches (dose equivalent; with no other physical rehab)
Funding & conflicts of interest	**Funding statement:** "supported by grants provided by the Health Ministry Program for Clinical Research PHRC no. AOM 01 102" **Conflict of interest statement:** "no conflicts of interest to report"
Synthesis	**Qualitative synthesis:** data not suitable for analysis. "Sixty‐seven of the 68 enrolled patients completed the study but the analysis, conducted for intention to treat, included all of the patients: 35 in the NDT group and 33 in the multisensorial group"
Notes	

**Table:** Yin 2003a

***Study characteristics***	
Methods	**Study aim:** to determine the effectiveness of early rehabilitation on people with stroke and hemiplegia **Design:** multi‐arm RCT (3‐arms) **Country:** China **Study recruitment and setting:** see Table **Involvement of patients/public/professionals/others:** no
Participants	**Study population (number randomised):** 90 **Inclusion criteria:** Met the diagnostic criteria of the 2nd National Cerebral Vascular Disease Conference Stroke diagnosed by CT or MRI Stable vital signs 2 weeks after stroke **Exclusion criteria:** Abnormalities in liver, kidney, heart, and lung function **Participants:** detailed in Table **PROGRESS‐plus:** detailed in Table
Interventions	(1) Rehabilitation (n = 30) "the routine drugs in three groups were same" "Patients in the rehabilitation group were administered with rehabilitation exercise and general education of rehabilitation" "We employed Bobath method on hemiplegia extremities. Pay attention to the position of the suffered extremities. When the patients lying on the healthy side, suffered upper limb were padded with a pillow and the upper limb were straight, with center of the palm down. The wrists were lifted with a pad. When the patients lied with the suffered side, suffered should extended forward, elbow extended and center of the palm down. For cases with the increased strength in flexor muscle, finger differentiation board or pad [was] put in the center of the hand and keep the finger straight forward as much as possible. The lower [limbs] were lightly flexioned, with back of the foot and lower leg [kept] perpendicular as much as possible. When the patients lying with back, suffered upper limb were in lightly extension conditions, with elbow lightly flexed, support shoulder and hand with pad to keep center of the palm up and keep upper limb posterior rotation position to prevent scapula down and dislocation of shoulder joint. Lower limb, pelvis and hip were supported with pad to keep perpendicular conditions between back of the foot and lower leg...Turn exercise, sitting exercise and standing exercise were according to regulations of Bobath adult hemiplegia exercise" Length of intervention period: not stated Number of sessions and length of individual sessions: "rehabilitation therapy lasted for 40 min with once a day" Intervention provider: not stated (2) Rehabilitation "with therapy with intermediate frequency" (n = 30) Length of intervention period: not stated Number of sessions and length of individual sessions: not stated Intervention provider: not stated (3) Control group (n = 30) "another 30 persons in internal neurology department were selected as the control group with pharmacy treatment alone" "No rehabilitation exercise or intermediate frequency therapy were administered in control group" Length of intervention period: no intervention Number of sessions and length of individual sessions: no intervention Intervention provider: no intervention
Outcomes	**Independence in ADL scales:** NR **Motor function scales:** Fugl‐Meyer Assessment **Measures of balance:** NR **Measures of gait velocity:** NR **Length of stay:** NR **Adverse events:** NR **Other measures:** NR **Time points when outcomes were assessed:** "the patients in three groups were evaluated at the beginning, 2 weeks, 4 weeks and 8 weeks after therapy and the patients in control group were evaluated in their family"
Comparison	**Amount of physical rehabilitation in groups:** differs **Categorisation of comparison:** 1. Physical rehabilitation versus no or limited physical rehabilitation (dose differs) (limited = < 50% of dose)
Funding & conflicts of interest	**Funding statement:** not reported **Conflict of interest statement:** not reported
Synthesis	**Quantitative synthesis:**Analysis 1.2 Not clear how the dose varied between groups (1) and (2): both groups used for analysis, with control group 'shared' between Mean and SD computed from categorical data
Notes	Short Chinese study published in English

**Table:** Yue Chunjiang 2014

***Study characteristics***	
Methods	**Study aim:** to observe the effect of acupuncture and moxibustion combined with exercise re‐learning rehabilitation technique on acute ischaemic stroke patients with hemiplegia **Design:** parallel‐group RCT **Country:** China **Study recruitment and setting:** see Table **Involvement of patients/public/professionals/others:** no
Participants	**Study population (number randomised):** 90 **Inclusion criteria:** Met the diagnostic criteria of the 4th National Academic Conference on Cerebrovascular Disease using CT or MRI Aged < 70 years Onset of stroke < 1 month First stroke Conscious score using GCS ≥ 8 Hemiplegia Stable vital signs **Exclusion criteria:** Other types of stroke confirmed by CT or MRI Intracranial infection or tumour History of craniocerebral trauma or surgery Severe heart, liver, and kidney conditions Skin lesions on acupuncture points and surrounding skin area Severe diabetes and poor blood sugar control Coagulation dysfunction, bleeding diseases or related conditions Myocardial infarction, angina pectoris, severe emphysema or other conditions which may preclude participation in exercise activities Moderate or severe demential Unable to follow motor commands Unable to co‐operate with the intervention or complete the intervention Lost to follow‐up or died before the end of the study **Participants:** detailed in Table **PROGRESS‐plus:** detailed in Table
Interventions	(1) Exercise training + acupuncture "The routine drug treatment was the same as that of the control group, and acupuncture and moxibustion therapy and exercise re‐learning rehabilitation techniques were administered. ①Acupuncture treatment: According to the specific conditions of the patient, choose Shangguan, Dicang, Shoulder, Armru, Shoulder, Quchi, Waiguan, Hegu, Shousanli, Chize, Weizhong, Yangguan, Juegu, Kunlun, Taichong, Liangqiu, Xuehai, Dubi, Yanglingquan, Sanyinjiao and other points. After deqi, use Huatuo brand SDZ‐1 electronic acupuncture apparatus, the continuous wave rate is 120 times·min‐1, and the output current intensity is gradually increased until the patient can accept it and can cause muscle contraction. Each treatment 30 minutes sea day 1 time. ②Sports re‐learning rehabilitation techniques: after the completion of acupuncture treatment, upper limb function training, orofacial function training, sitting up training, sitting balance training, standing up and squatting training, half squat training, standing Balance training, pelvic control training, walking training. Follow 4 steps to describe normal activity components, practice lost motor components, practice assignments and transfer training. The training session lasted 54 minutes once a day. Continuous treatment for 6 months." (2) Conventional rehabilitation "Carry out health education and psychological counseling, and give routine drug treatment and rehabilitation training. Drug treatment is based on the principle of symptomatic and supportive treatment such as improving circulation, anti‐platelet aggregation, and nutritional nerve. Rehabilitation training includes passive movement, power‐assisted movement, active movement, and pelvic control training. Once a day, each training lasts 45 min. Continuous treatment for 6 months."
Outcomes	**Independence in ADL scales:** Barthel Index **Motor function scales:** Fugl Meyer assessment **Measures of balance:** Berg Balance Scale **Measures of gait velocity:** NR **Length of stay:** NR **Adverse events:** NR **Other measures:** Functional Ambulation Categories, Neurological Deficit Score **Time points when outcomes were assessed:** 1st, 3rd, and 6th months after treatment
Comparison	**Amount of physical rehabilitation in groups:** differs **Categorisation of comparison:** 5. Additional therapy + usual therapy versus usual therapy (dose differs)
Funding & conflicts of interest	**Funding statement:** not reported **Conflict of interest statement:** not reported
Synthesis	**Quantitative synthesis:**Analysis 5.1; Analysis 5.2; Analysis 5.3
Notes	This study was published in Chinese and translated into English using Google Translate.

**Table:** Yue Lin 2012

***Study characteristics***	
Methods	**Study aim:** to observe the effect of balance training on the walking ability of people with stroke and hemiplegia **Design:** parallel‐group RCT **Country:** China **Study recruitment and setting:** see Table **Involvement of patients/public/professionals/others:** no
Participants	**Study population (number randomised):** 92 **Inclusion criteria:** Met the diagnostic criteria of the 4th National Cerebrovascular Disease Conference First stroke diagnosed using CT or MRI Stroke onset < 6 months Condition was stable, conscious, and could co‐operate with rehabilitation treatment. Balance dysfunction No diseases or complications which might preclude rehabilitation training **Exclusion criteria:** Not reported **Participants:** detailed in Table **PROGRESS‐plus:** detailed in Table
Interventions	(1) Balance training "The treatment group was given balance function training at the same time, 30 min/time, 2 times/d, 11 times/week for 8 weeks. 1.2.1 Sitting balance training: including sitting static balance training and dynamic balance training when the position of the head and trunk changes. 1.2.2 Sit‐stand‐sit training: instruct the patient to shift the center of gravity and control the trunk when changing positions. 1.2.3 Standing balance training: there are static balance training and dynamic balance training, including double knee joint control, static standing balance training and standing balance response training. 1.2.4 Walking training and complexity training: With the improvement of balance function and lower body weight‐bearing capacity, walking training can be carried out, and training of going up and down stairs and crossing obstacles can be added." (2) Conventional rehabilitation "Conventional rehabilitation treatment includes exercise therapy, intermediate frequency, low frequency, electro‐acupuncture, electric stand‐up bed and other training." (Unclear if balance training is given in addition to the conventional rehabilitation; assumption is that this is the case).
Outcomes	**Independence in ADL scales:** NR **Motor function scales:** Fugl Meyer assessment **Measures of balance:** NR **Measures of gait velocity:** NR **Length of stay:** NR **Adverse events:** NR **Other measures:** Holden functional walking classification **Time points when outcomes were assessed:** after 8 weeks of treatment
Comparison	**Amount of physical rehabilitation in groups:** differs **Categorisation of comparison:** 5. Additional therapy + usual therapy versus usual therapy (dose differs)
Funding & conflicts of interest	**Funding statement:** not reported **Conflict of interest statement:** not reported
Synthesis	**Quantitative analysis:**Analysis 5.2 (insufficient details to enable inclusion in subgroup analyses)
Notes	This study was published in Chinese and translated using Google Translate.

**Table:** Zang 2013

***Study characteristics***	
Methods	**Study aim:** to explore the effect of 3‐stage Traditional Chinese Medicine rehabilitation on neurological function and motor function in patients with cerebral ischaemic stroke **Design:** parallel‐group RCT **Country:** China **Study recruitment and setting:** see Table **Involvement of patients/public/professionals/others:** no
Participants	**Study population (number randomised):** 100 **Inclusion criteria:** Met the diagnostic criteria of the 4th National Academic Conference on Cerebrovascular Disease for acute cerebral infarction Type of cerebral infarction is consistent with the Oxfordshire community stroke classification for a partial anterior circulation infarction or posterior circulation infarct **Exclusion criteria:** TIA or reversible ischaemic neurological deficit Severe cerebral oedema or coma and other unstable conditions Confirmed by examination that it was caused by brain tumour, brain trauma, blood disease, etc. History of cerebral infarction or haemorrhage, with obvious limb dysfunction Severe infection or serious diseases of liver, kidney, haematopoietic system, endocrine system, and osteoarthritis complications Severe mental health symptoms Severe cognitive impairment Dementia Currently participating in other drug research and treatment Unwilling to take part in treatment and rehabilitation or unable to actively cooperate **Participants:** detailed in Table **PROGRESS‐plus:** detailed in Table
Interventions	(1) Three‐stage rehabilitation (stage 2 and 3) "underwent the first‐level rehabilitation to the 14th day after onset....after the patient's condition was stabilised, early bedside rehabilitation therapy, acupuncture and massage therapy were given. At the same time, psychological care and rehabiliation are provided to patients. From the 15th day to the 28th day. the second‐level rehabilitation is carried out.....from the 29th day to the 90th day, the thrid level rehabilitation will be carried out....in the township hospital or at home". "Exercise therapy includes: maintenance of good limb positions in lying and sitting positions; training on bed position changes; bedside position changes training; bed position changes training. Occupational therapy includes: daily life activities such as eating, grooming, dressing, bed and wheel chair transfer, as well as manual simulation operations such as woodworking and knitting, and training in sports and entertainment such as rings and puzzles. The first‐level rehabilitation is mainly based on exercise therapy, including the placement of good limbs, passive training of affected limbs, training of active limbs, deep breathing training, waist and abdominal muscle training, turning over and sitting up, sitting balance training and standing up training, etc. , in order to solve the function of patients getting up, 45 minutes each time, once a day, 5 days a week, for a total of 2 weeks. While training patients, try to teach the patient's family members or nurses the correct auxiliary training and nursing methods. The second‐level rehabilitation mainly includes balance training, single‐leg standing training, walking training, up and downstairs training, coordination training, etc. to solve the patient's walking function, 30‐45 minutes each time, 2 times a day, 5 days a week, a total of 2 week. The tertiary rehabilitation mainly trains patients to take care of themselves in daily life, such as eating, grooming, dressing, transferring beds and wheelchairs, handling personal hygiene, etc.,30‐45 minutes each time, twice a day, 5 days a week, for a total of 2 months. 1.2.2 Tuina manipulation trains various joints and muscles of the affected limbs to refresh the brain, activate blood and remove blood stasis, relax muscles and collaterals, nourish yin and reduce fire, nourish the heart and soothe the mind, so as to regulate the viscera, balance yin and yang, and promote the movement of hemiplegic limbs recovery. 1.2.3 Acupuncture treatment wind‐phlegm blocking collaterals: acupuncture at Baihui, Fengchi, Fengfu, Fenglong, Zusanli, Xuehai, Geshu;.....1.2.4 Psychological rehabiliation and nursing medical staff should focus on the specific situation of the patient....provide psychological counselling in a timely manner, let patients and their family the importance of rehabilitation training....improve language condiction, and promote functional recovery...." (2) No physical rehabilitation "The control group was only given the first‐level rehabilitation... The patients in the control group were in the rehabilitation treatment period from the onset of the disease to the 14th day after the onset of the disease. They only received the first‐level rehabilitation treatment of western medicine, and were discharged home after the end of the treatment" "Both the experimental group and the control group were treated with conventional western medicine symptomatic treatment"
Outcomes	**Independence in ADL scales:** NR **Motor function scales:** Fugl‐Meyer assessment **Measures of balance:** NR **Measures of gait velocity:** NR **Length of stay:** NR **Adverse events:** NR **Other measures:** National Institutes of Health Stroke Scale (NIHSS) **Time points when outcomes were assessed:** before and after 4 weeks of treatment
Comparison	**Amount of physical rehabilitation in groups:** differs **Categorisation of comparison:** 1. Physical rehabilitation versus no or limited physical rehabilitation (dose differs) (limited = < 50% of dose)
Funding & conflicts of interest	**Funding statement:** Qingdao Public Sector Science and Technology Support Program Project (No. 10‐3‐3‐5‐4‐nsh) **Conflict of interest statement:** not reported
Synthesis	**Quantitative synthesis:**Analysis 1.2
Notes	This study was published in Chinese and translated into English using Google Translate.

**Table:** Zhang 1998

***Study characteristics***	
Methods	**Study aim:** to examine the effects of early rehabilitation therapy on 56 people with stroke and hemiplegia **Design:** parallel‐group RCT **Country:** China **Study recruitment and setting:** see Table **Involvement of patients/public/professionals/others:** no
Participants	**Study population (number randomised):** 56 **Inclusion criteria:** Diagnosed using clinical appearance and cerebral CT as having cerebrovascular disease **Exclusion criteria:** TIA or reversible ischaemic neurological deficit (RIND) **Participants:** detailed in Table **PROGRESS‐plus:** detailed in Table
Interventions	(1) Three‐stage rehabilitation (n = 29) "The intervention group… underwent early rehabilitation therapy in conjunction with normal medical therapy" "Stage 1 (lying exercises): The patient is without any independent movement, and is in a state of flaccid hemiplegia. The patient is placed in a functional position and lightly massaged on the hands and limbs. Limbs are passively extended and retracted in small and large movements. This is in addition to rolling the patient on the bed and single and double leg bridge movements. The goal is to gradually lead the patient to balancing the trunk in a sitting position" "Stage 2 (sitting exercises): When the patient gains some muscle strength and muscle groups can react and work in groups, continue the exercises described above until the goals in stage one are achieved. In the meantime, add balancing exercises as well as resistance training in the sitting position. At the same time, stimulate sensory input by using cold and hot compresses and light taps to the antagonistic muscles. Gradually lead the patient into training for balancing while standing up" "Stage 3 (standing exercises): When the patient is at the stage where his limbs spasm and can freely initiate movement in groups, continue the exercises in stage 2 until goals are met as well as correcting and controlling irregular positions and increasing normal movement and co‐ordination. In order to prepare for walking, exercise the extensor carpi muscles, supination of forearm, standing with support, standing with weights on the lower limbs and walking with support" "Stage 4 (walking exercises): when the patient’s spasming eases, continue to complete the goals set out in stage 3 as well as walking indoors and on stairs, correcting walking posture, co‐ordination and completing ADL activities" "Patients about to be discharged from hospital were also trained to perform ADL. For individuals with slow recovery of function, we encouraged them to overcome dependence on others and actively participate in social activities. For some patients, it was necessary to advise occupational training" "Psychological therapy: Patients with depression, pessimism, loss of confidence and unwillingness to cooperate with therapy were promptly given psychological care, allowing the patient to achieve optimal psychological status, as a proactive approach from the patient to rehabilitation is essential" Length of intervention period: not stated Number of sessions and length of individual sessions: "Therapy was conducted in a one‐to‐one approach in one hour sessions once daily" Intervention provider: not stated but does mention that "therapy was conducted… with the help of the patient's family" (2) No physical rehabilitation (n = 27) "underwent conventional neurological medical therapy" The individual components delivered are listed in Table 28. Based on the individual components, this intervention is categorised as comprising no intervention. Length of intervention period: no intervention Number of sessions and length of individual sessions: no intervention Intervention provider: no intervention
Outcomes	**Independence in ADL scales:** Barthel Index **Motor function scales:** Fugl‐Meyer Assessment **Measures of balance:** NR **Measures of gait velocity:** NR **Length of stay:** NR **Adverse events:** NR **Other measures:** "recovery of mobility was assessed according to the Brunnstrom 6 classification" **Time points when outcomes were assessed:** "assessments of all patients’ condition[s] were conducted 3 months after onset of symptoms"
Comparison	**Amount of physical rehabilitation in groups:** differs **Categorisation of comparison:** 1. Physical rehabilitation versus no or limited physical rehabilitation (dose differs) (limited = < 50% of dose)
Funding & conflicts of interest	**Funding statement:** not reported **Conflict of interest statement:** not reported
Synthesis	**Quantitative synthesis:**Analysis 1.1; Analysis 1.2
Notes	Study translated by Cochrane Stroke Group

**Table:** Zhang 2004

***Study characteristics***	
Methods	**Study aim:** to evaluate the effectiveness of tertiary rehabilitation therapy for acute stroke **Design:** parallel‐group RCT **Country:** China **Study recruitment and setting:** see Table **Involvement of patients/public/professionals/others:** no
Participants	**Study population (number randomised):** 1078 **Inclusion criteria:** Aged < 85 years old Ischaemic or haemorrhagic stroke confirmed by CT or MRI scan Met the 4th National Conference on Cerebral Vascular Disease classification guidelines First ever stroke GCS > 8 Deficits in limb function **Exclusion criteria:** TIA or subarachnoid haemorrhage Aggravation of medical condition or progressing ischaemia or haemorrhage Impaired function of the heart, lungs, liver, kidney, and other vital organs **Participants:** detailed in Table **PROGRESS‐plus:** detailed in Table
Interventions	(1) Rehabilitation (n = 439) "Building a tertiary rehabilitation network: Establish a tertiary rehabilitation network (3‐tier medical, rehabilitation network) from several hospitals’ Department of Neurology—rehabilitation centres (or merge hospitals’ Department of Rehabilitation)—community rehabilitation organisations (or home therapy) from several cities in the whole country Patients from the rehabilitation group first stayed at the hospital's Department of Neurology, receiving 28 days of critical phase routine medical care and early rehabilitation. Based on ADL ability, patients who were ADL independent, who discharged to home, transferred to community rehabilitation organisations to undertake their rehabilitative training, to further improve their exercise ability, integration ability and ADL ability. Patients who were unable to reach ADL independence or still had moderate to severe functional disability after the early rehabilitation period, were transferred to rehabilitation centres or the tertiary rehabilitation network hospitals’ Department of Rehabilitation to undertake specific strengthening training, to minimise the patient’s functional disability. This phase usually took approximately 2 months. After which, patients transferred to community rehabilitation organisations to undertake further rehabilitative training, to consolidate effects from previous rehabilitation, and to continue rehabilitation for other persisting functional disabilities. Patients in the control group discharged home after 28 days of routine treatment and early rehabilitation, were given pre‐arranged guidance from professionals, and undertook rehabilitative training on their own or with assistance from their family. Community rehabilitation in certain regions was not developed, were unable to provide community rehabilitation, thus patients had to return to the tertiary rehabilitation network Department of Rehabilitation to continue training. Each tertiary rehabilitation network utilised uniform inclusion criteria to select patients, with randomisation to the rehabilitation or control group. Personnel involved in the tertiary rehabilitation network included doctors, therapists, assessors who undertook training sessions to ensure uniformity at all centres. All tertiary rehabilitation network centres used a uniform rehabilitation training method and outcome assessment" "Tertiary rehabilitation network treatment: Treatment during the critical phase was guided by Zhonghua Medical Association’s recommendations. Rehabilitative training focused on physiotherapy (PT) and occupational therapy (OT), with speech and language therapy and psychological therapy given at centres with the capabilities. Physiotherapists delivered therapy uniformly, using Bobath techniques and methods from the motor relearning program mainly, with added use of PNF techniques, Brunnstrom etc. Therapy and training followed the patient’s stroke recovery characteristics, adjusting to the patient’s actual functional status, and delivered systematically and progressively" "Training content: During the period when patient was confined in bed, passive ranging, optimal limb positioning, rolling, sitting up, bridging exercises in bed, sitting on bed, sitting balance training etc was given. When patient could achieve sitting for 30 min without postural hypotension or other symptoms, the patient could use a wheelchair to undertake training in the gym. Training content mainly included: active‐passive exercises, standing and standing balance training, lower limb weight training and shifting of centre of mass training, stepping practice and gait correction, ankle dorsiflexion, forearm pronation and supination, finger grasping etc. Family members were taught rehabilitative training methods, and at the same time, patient and family underwent health counselling, given a health education booklet, shown a video on neurological recovery etc, with the aim of furthering the patient’s recovery" Length of intervention period: 6 months Number of sessions and length of individual sessions: dependent on stage of recovery (see above) Intervention provider: doctors and therapists (2) No physical rehabilitation (n = 463) "The control group received conventional neurological medical therapy only. Patients in the control group discharged home after 28 days of routine treatment and early rehabilitation, were given pre‐arranged guidance from professionals, and undertook rehabilitative training on their own or with assistance from their family" Length of intervention period: 6 months Number of sessions and length of individual sessions: no intervention Intervention provider: no intervention
Outcomes	**Independence in ADL scales:** Barthel Index **Motor function scales:** Fugl‐Meyer Assessment **Measures of balance:** NR **Measures of gait velocity:** NR **Length of stay:** NR **Adverse events:** NR **Other measures:** Ashworth Scale, SF‐36, Loewenstein Occupational Therapy Cognitive Assessment (LOTCA), NIHSS, WAB (speech assessment), Hamilton (depression assessment) **Time points when outcomes were assessed:** within 1 week after stroke and at the end of each month for 6 months
Comparison	**Amount of physical rehabilitation in groups:** differs **Categorisation of comparison:** 1. Physical rehabilitation versus no or limited physical rehabilitation (dose differs) (limited = < 50% of dose)
Funding & conflicts of interest	**Funding statement:** not reported **Conflict of interest statement:** not reported
Synthesis	**Quantitative synthesis:**Analysis 1.1; Analysis 1.2
Notes	Original study translated from Chinese to English by a member of our team (PLC).

**Table:** Zhang Huiyu 2021

***Study characteristics***	
Methods	**Study aim:** to investigate the effect of goal‐oriented functional exercise on rehabilitation of patients with cerebral haemorrhage **Design:** parallel‐group RCT **Country:** China **Study recruitment and setting:** see Table **Involvement of patients/public/professionals/others:** no
Participants	**Study population (number randomised):** 104 **Inclusion criteria:** Met the diagnostic criteria of the 4th National Academic Conference on Cerebrovascular Disease using imaging methods Provided informed consent **Exclusion criteria:** Severe heart, liver, lung, kidney, and other organ dysfunction Malignant tumour Intracranial haemorrhage or infection Alzheimer's disease **Participants:** detailed in Table **PROGRESS‐plus:** detailed in Table
Interventions	(1) Functional exercise "Implement goal‐directed functional exercises" "(1) Specific interventions for short‐term goals: ① Posture training. The intervention staff assisted the patient in the supine position to maintain the wrist dorsiflexion, and the angle was controlled at 20°. ‐30°, shoulder flexion 30 degrees. , valgus 45. , Internal rotation 15. , elbow flexion, maintain the angle of 90. ; When lying on the unaffected side, keep the wrist dorsi‐extended by 20. Left and right, shoulder flexion 90. Skull joint abduction 20°, lower extremity ankle dorsiflexion 90°. , the knee joint is naturally bent. ② passive limb training. The intervention staff assists the patient to move the body to the bedside and place the unaffected thigh on the affected leg with natural flexion. Then the intervention personnel rotated the patient's body and swung their legs back and forth, 15min/time, 2 times/d. (2) Specific interventions for medium‐term goals: ① Standing balance exercise. The intervention staff instructed the patients to move their feet forward and straighten their upper limbs upward to ensure that the knee joints were straight, and the specific standing time was determined according to their adaptation and comfort. ② cognitive function training. The intervention staff encouraged patients to use their brains and asked them to repeat information such as date, time, phone number, home address, etc., and gradually increased the amount of information to repeat to exercise and improve their memory. (3) Specific interventions for long‐term goals: ①Exercise by walking. The intervention personnel first maintained the balance in the sitting posture, and then trained them to implement balance bar walking exercise, and then perform up and down stairs exercise. The time of each walking exercise was controlled within 5‐10 minutes, and the exercise time was reduced for those who had discomfort during exercise. ②Daily rehabilitation training. The intervention staff instructed the patients in daily activities such as eating, defecation, and washing. ③ intelligence training. Interventions guide patients through activities such as puzzles, sensory stimulation, and numerical calculations, and improve their memory skills by asking them to retell stories. ④Language ability training. The intervention staff instructed the patients to speak clearly and fluently, and repeated exercises for those with poor language skills." (2) Conventional rehabilitation "Routine postoperative functional exercise, guided by the intervention staff, mainly includes passive rotation of the limbs and joints, 30min/time, 2 times/d; active exercises such as leg raising and chest expansion, 30min/time, 1 time/d, based on the patient Tolerance is the degree, step by step, it can be instructed to stand up voluntarily, 2 times/d, and the sitting time is 15min/time."
Outcomes	**Independence in ADL scales:** Barthel Index **Motor function scales:** "Fugl‐Meyer balance scale" **Measures of balance:** NR **Measures of gait velocity:** NR **Length of stay:** NR **Adverse events:** NR **Other measures:** quality of life scale, National Institutes of Health Neurological Impairment Scale (NIHSS) **Time points when outcomes were assessed:** before and after 6 months of rehabilitation
Comparison	**Amount of physical rehabilitation in groups:** equivalent **Categorisation of comparison:** 3.(i) Comparison of different approaches (dose equivalent; with no other physical rehab) (Note: the total dose of rehabilitation in both groups is unclear; we have assumed that this is equivalent)
Funding & conflicts of interest	**Funding statement:** not reported **Conflict of interest statement:** not reported
Synthesis	**Quantitative synthesis:**Analysis 7.1; Analysis 7.2
Notes	This study was published in Chinese and translated into English using Google Translate.

**Table:** Zhang Jianhong 2013

***Study characteristics***	
Methods	**Study aim:** to explore the effect of neurorehabilitation in the treatment of cerebral infarction **Design:** parallel‐group RCT **Country:** China **Study recruitment and setting:** see Table **Involvement of patients/public/professionals/others:** no
Participants	**Study population (number randomised):** 166 **Inclusion criteria:** Met the diagnostic criteria of the 4th National Academic Conference on Cerebrovascular Disease All cases were confirmed by head CT **Exclusion criteria:** Not reported **Participants:** detailed in Table **PROGRESS‐plus:** detailed in Table
Interventions	(1) Rehabilitation "Treatments include: ①Place in a good posture; ② passive movement of joints; ③ Bobath technology [4]; ④Brunstrom technology; ⑤ROOD technique; 2 to 3 times/d, 0.5 to 1 h/d, the treatment time is 5 weeks. " (2) No physical rehabilitation "Self‐training without regulated rehabilitation. The observation period was 5 weeks."
Outcomes	**Independence in ADL scales:** Barthel Index **Motor function scales:** Fugl Meyer motor function **Measures of balance:** NR **Measures of gait velocity:** NR **Length of stay:** NR **Adverse events:** NR **Other measures:** Eciberg plus Scandinavian modified scale **Time points when outcomes were assessed:** admission and after 5 weeks of treatment
Comparison	**Amount of physical rehabilitation in groups:** differs **Categorisation of comparison:** 1. Physical rehabilitation versus no or limited physical rehabilitation (dose differs) (limited = < 50% of dose)
Funding & conflicts of interest	**Funding statement:** not reported **Conflict of interest statement:** not reported
Synthesis	**Quantitative synthesis:**Analysis 1.1; Analysis 1.2
Notes	This study was published in Chinese and translated into English using Google Translate.

**Table:** Zhang Lifang 2015

***Study characteristics***	
Methods	**Study aim:** to investigate the effect of balance function training on the function of the lower limbs on the hemiplegic side of stroke patients **Design:** parallel‐group RCT **Country:** China **Study recruitment and setting:** see Table **Involvement of patients/public/professionals/others:** no
Participants	**Study population (number randomised):** 52 **Inclusion criteria:** First stroke confirmed by cranial MRI or CT using diagnostic criteria of the 4th National Academic Conference on Cerebrovascular Disease Within 3 weeks of the onset Stable vital signs Conscious and can co‐operate to complete the treatment Informed consent **Exclusion criteria:** Severe cognitive problems Severe cardiopulmonary problems, such as arrhythmia, heart failure, Severe mental health issues or stress which may exacerbate their condition Severe infection Severe lower extremity orthopaedic disease Unable to participate fully in intervention exercises **Participants:** detailed in Table **PROGRESS‐plus:** detailed in Table
Interventions	(1) Balance training "Training in the treatment group: ① The patient lies in the supine position on the treatment bed, and the lower limbs are flexed, stretched, adducted, and abducted in turn, and the lower limbs are kept in a suspended position. ②Sit, the patient is sitting on a chair, when the patient's posture is tilted to one side, the therapist should not immediately support the patient, but should gently push the patient to the tilted direction to induce the posture reflex and make the patient stand upright. ③ Standing, after 5 weeks of training, perform standing balance training. The patient stands a few inches apart from the lower limbs, and the body weight is swayed back and forth, left and right to maintain the standing balance; Do not grasp the support and carry, swing back and forth left and right to keep the standing position balanced and walk, and the patient is trained to cross obstacles in the standing position. In the process of balance function training, you should pay attention to maintaining the regulation of your mood. Balance function training was performed 5 times a week, 30‐40 min/time, for 12 weeks." (2) Routine rehabilitation "The routine group training: ① joint range of motion training; ② functional electrical stimulation; ③ acupuncture treatment; ④ crawling training; ⑤ stretch technique; ⑥ parabola training. The above treatments were performed 5 times a week, 40 min/time, for 5 consecutive weeks."
Outcomes	**Independence in ADL scales:** Modified Barthel Index **Motor function scales:** Fugl Meyer motor function score **Measures of balance:** Berg Balance Scale **Measures of gait velocity:** NR **Length of stay:** NR **Adverse events:** NR **Other measures:** modified Ashworth Scale **Time points when outcomes were assessed:** before and after treatment
Comparison	**Amount of physical rehabilitation in groups:** differs **Categorisation of comparison:** 1. Physical rehabilitation versus no or limited physical rehabilitation (dose differs) (limited = < 50% of dose) (Note: routine rehabilitation group did get an equivalent dose of physical rehabilitation for the first 5 weeks, but then no physical rehabilitation for the subsequent 7 weeks).
Funding & conflicts of interest	**Funding statement:** not reported **Conflict of interest statement:** not reported
Synthesis	**Qualitative synthesis:** Data not included in analyses due to serious concerns about the data: ADL and motor function data indicate that the regular group had better outcome than the intervention group, but the narrative text presents data the other way round. Berg Balance Scale score data were not suitable for inclusion in the meta‐analysis, as these did not appear to relate to the standard range of Berg balance scores.
Notes	This study was published in Chinese and translated into English using Google Translate.

**Table:** Zhao 2002

***Study characteristics***	
Methods	**Study aim:** to examine the effectiveness of early stage rehabilitation and nursing in people with stroke and hemiplegia **Design:** parallel‐group RCT **Country:** China **Study recruitment and setting:** see Table **Involvement of patients/public/professionals/others:** no
Participants	**Study population (number randomised):** 180 **Inclusion criteria:** Not reported **Exclusion criteria:** Not reported **Participants:** detailed in Table **PROGRESS‐plus:** detailed in Table
Interventions	(1) Rehabilitation (n = 100) "The rehabilitation nursing group received conventional treatment and rehabilitative treatment. During acute cerebral oedema, rehabilitation commenced with bedside therapy, postural positioning and passive ranging exercises. 7‐14 days, after reduction in cerebral oedema, active training became the focus, including six stages: (i) active or passive ranging bed exercises; (ii) sit up from bed and sitting on the edge of bed balance training; (iii) sit‐to‐stand training; (iv) standing balance training; (v) flat ground gait training; (vi) up‐and–down stair and ADL training (e.g. donning, grooming, feeding, showering etc). After discharging to home, rehabilitation focused on maintaining joint range of motion and ADL, seeking the assistance of family members. After discharge, both groups had follow up visits to the hospital fortnightly, and monthly after a period of 3 months" Length of intervention period: total therapy duration 31.6 ± 11.2 days Number of sessions and length of individual sessions: five/wk, with the first phase having 30 minutes/session, and 45 minutes/session thereafter Intervention provider: 1:1 nurse‐to‐participant ratio (2) No physical rehabilitation (n = 80) "Received conventional treatment, including control of hypertension, lowering intracranial pressure, anti‐coagulant treatment, neuro‐regenerative treatment and self‐treatment, with treatment duration being 29.1 days ± 8.4 days" The individual components delivered are listed in Table 28. Based on the individual components, this intervention is categorised as comprising no intervention. Length of intervention period: treatment duration 29.1 ± 8.4 days Number of sessions and length of individual sessions: no intervention Intervention provider: no intervention
Outcomes	**Independence in ADL scales:** Barthel Index **Motor function scales:** Fugl‐Meyer Assessment **Measures of balance:** NR **Measures of gait velocity:** NR **Length of stay:** NR **Adverse events:** NR **Other measures:** degree of neurological deficit (translated) **Time points when outcomes were assessed:** before intervention and at 1 week, 3 weeks, 5 weeks, 3 months, 6 months, and 12 months after stroke
Comparison	**Amount of physical rehabilitation in groups:** differs **Categorisation of comparison:** 1. Physical rehabilitation versus no or limited physical rehabilitation (dose differs) (limited = < 50% of dose)
Funding & conflicts of interest	**Funding statement:** not reported **Conflict of interest statement:** not reported
Synthesis	**Quantitative synthesis:**Analysis 1.2
Notes	Original study translated from Chinese to English by a member of our team (PLC).

**Table:** Zhao 2003

***Study characteristics***	
Methods	**Study aim:** to investigate the influence of early rehabilitation on ADLs in people with cerebral infarction **Design:** parallel‐group RCT **Country:** China **Study recruitment and setting:** see Table **Involvement of patients/public/professionals/others:** no
Participants	**Study population (number randomised):** 300 **Inclusion criteria:** Met the diagnostic criteria of the Fourth Cerebrovascular Disease Academic Conference Stroke confirmed by clinic, CT or cerebral MR Suffered with different levels of paralysis No loss of consciousness **Exclusion criteria:** Not reported **Participants:** detailed in Table **PROGRESS‐plus:** detailed in Table
Interventions	(1) Rehabilitation (n = 150) "Both groups received drug therapy after hospitalization. Rehabilitation group began to receive rehabilitation as soon as state of illness was stable" "Patients in Atonia stage received posture transfer exercise with active and passive exercises. In spasm stage, patients received exercise of inhibiting spasms, limb weight loading exercise, trunk control exercise, exercise for bilateral limbs coordination, limb normal motor pattern exercise and exercises for movement coordination. Patients in recovery stage received exercise for speed, mental movement training, walking training, up and down stair exercise and ADL exercise" Length of intervention period: "(PT) :.. 10 days as a treatment course, persisting 2 courses. (OT):.. 10 days as a treatment course, persisting 2 courses" Number of sessions and length of individual sessions: "(PT): 1 time per day, 40 mins per time, (OT): 1 time per day, 30‐40 minutes per day" Intervention provider: not stated (2) No physical rehabilitation group (n = 150) No intervention Length of intervention period: no intervention Number of sessions and length of individual sessions: no intervention Intervention provider: no intervention
Outcomes	**Independence in ADL scales:** Barthel Index **Motor function scales:** NR **Measures of balance:** NR **Measures of gait velocity:** NR **Length of stay:** NR **Adverse events:** NR **Other measures:** NR **Time points when outcomes were assessed:** "patients were evaluated by the same doctor before and after treatment"
Comparison	**Amount of physical rehabilitation in groups:** differs **Categorisation of comparison:** 1. Physical rehabilitation versus no or limited physical rehabilitation (dose differs) (limited = < 50% of dose)
Funding & conflicts of interest	**Funding statement:** not reported **Conflict of interest statement:** not reported
Synthesis	**Quantitative synthesis:**Analysis 1.1 Mean and standard deviations computed from categorical data
Notes	Short Chinese study published in English—brief report only

**Table:** Zhao Ailiang 2016

***Study characteristics***	
Methods	**Study aim:** to observe the clinical effect of acupuncture combined with rehabilitation training in the treatment of post‐stroke dyskinesia **Design:** parallel‐group RCT **Country:** China **Study recruitment and setting:** see Table **Involvement of patients/public/professionals/others:** no
Participants	**Study population (number randomised):** 126 **Inclusion criteria:** Met the diagnostic criteria for stroke Provided consent **Exclusion criteria:** Other serious heart, liver, and kidney diseases Did not co‐operate with the treatment **Participants:** detailed in Table **PROGRESS‐plus:** detailed in Table
Interventions	All participants were given routine western medicine treatment, such as drugs that promote the recovery of brain tissue function. Once vital signs were stabilised, then intervention(s) were delivered. (1) Rehabilitation + acupuncture Conventional acupuncture as below in (2). Rehabilitation included passive movement of the joints is mainly to stimulate the paralysed side shoulder, wrist, shoulder, and ankle joints, and insist on performing a full range of joint movements every day. Power‐assisted exercises were also given to participants who could manage. Training was delivered for 2 sessions/day with each session lasting for 20 to 30 minutes. Participants were also given daily self‐care ability training with the assistance of the patient's family members, such as turning over and sitting and standing training. In addition, balance function training (sitting, standing), were completed by the patient's family members under the guidance of the doctor. Two to four sessions/day with each session lasting 20 to 30 minutes. (2) Acupuncture Conventional acupuncture. Participants received scalp and body acupuncture combined with acupoint selection for 30 minutes. Acupoints were selected based on the specific condition of the patient. The G6805 electroacupuncture device was used for continuous wave stimulation (1~4 Hz), based on the tolerance of the participant.
Outcomes	**Independence in ADL scales:** Barthel Index **Motor function scales:** NR **Measures of balance:** NR **Measures of gait velocity:** NR **Length of stay:** NR **Adverse events:** NR **Other measures:** GCQoLi74, Brunnstrom, ability to sit and stand (categorised) **Time points when outcomes were assessed:** before and after treatment
Comparison	**Amount of physical rehabilitation in groups:** differs **Categorisation of comparison:** 1. Physical rehabilitation versus no or limited physical rehabilitation (dose differs) (limited = < 50% of dose)
Funding & conflicts of interest	**Funding statement:** not reported **Conflict of interest statement:** not reported
Synthesis	**Quantitative synthesis:**Analysis 1.1
Notes	This study was published in Chinese and relevant sections translated into English by our author team (PLC).

**Table:** Zhao Haihong 2013

***Study characteristics***	
Methods	**Study aim:** to evaluate the effect of Bobath technology for patients with hemiplegia after acute ischaemic stroke **Design:** parallel‐group RCT **Country:** China **Study recruitment and setting:** see Table **Involvement of patients/public/professionals/others:** no
Participants	**Study population (number randomised):** 60 **Inclusion criteria:** Met diagnostic criteria for stroke **Exclusion criteria:** Not reported **Participants:** detailed in Table **PROGRESS‐plus:** detailed in Table
Interventions	(1) Rehabilitation "On the premise that the observation group was given the same conventional treatment as the control group, they were given Bobath technique treatment, as follows: (1) During the bed rest period, the patients were given bed activity training, including the stretching of their trunk muscles, knee flexion exercises in supine and prone positions, sitting up, turning over, and hip joint control training; (2) When the patient is able to sit up, give him sitting balance training, including level 1, 2, and 3 training, and the training from sitting to standing is guided by the nursing staff; (3) When the patient can transition to standing, give his lower limbs weight‐bearing training, and achieve the purpose of inducing muscle tension through upright stimulation [3]; (4) When the patient transitions from standing to walking, give him corresponding walking training, and the whole process of training is carried out between the parallel bars to correct the patient's poor gait; (5) After the patient's gait is corrected, give him up and down stairs training to exercise his coordination [4]; (6) Give patients auxiliary treatment through walkers, orthoses and wheelchairs, and instruct the patients by nursing staff during their training to avoid accidents. All trainings were performed once/d, and 7 d was a course of treatment." (2) Standard care "conventional neurological treatment, such as oral vitamin B1, methylcobalamin and other comprehensive symptomatic treatment, and at the same time, the patients were given Naofukang injection every day, 4‐8 g/time, 1. times/d, mixed with 0.9% sodium chloride injection or 5% glucose injection to 250 mL for intravenous drip. For patients over 60 years old, the dosage should be halved, and acupuncture physiotherapy and traditional rehabilitation therapy should be given at the same time."
Outcomes	**Independence in ADL scales:** NR **Motor function scales:** NR **Measures of balance:** NR **Measures of gait velocity:** NR **Length of stay:** NR **Adverse events:** NR **Other measures:** Chinese Stroke Patient Neurological Function Scale **Time points when outcomes were assessed:** before and after treatment
Comparison	**Amount of physical rehabilitation in groups:** differs **Categorisation of comparison:** 1. Physical rehabilitation versus no or limited physical rehabilitation (dose differs) (limited = < 50% of dose)
Funding & conflicts of interest	**Funding statement:** not reported **Conflict of interest statement:** not reported
Synthesis	**Qualitative synthesis:** no relevant outcomes assessed
Notes	This study was published in Chinese and translated into English using Google Translate.

**Table:** Zheng 2014

***Study characteristics***	
Methods	**Study aim:** "to analyze the effect of the rehabilitation therapeutic on ischemic stroke patients in anaphase and investigate the effective therapeutic schemes to patients with ischemic stroke" **Design:** parallel‐group RCT **Country:** China **Study recruitment and setting:** see Table **Involvement of patients/public/professionals/others:** no
Participants	**Study population (number randomised):** 60 **Inclusion criteria:** Diagnosed using the 4th National Cerebrovascular Disease Academic Conference (1995) criteria Limb dysfunction No serious consciousness and cognitive impairment Stable vital signs No cardiopulmonary failure No evidence of mental health issues No history of dementia **Exclusion criteria:** Not reported **Participants:** detailed in Table **PROGRESS‐plus:** detailed in Table
Interventions	(1) Rehabilitation "a targeted rehabilitation training program under the guidance of professional rehabilitation physicians...." "rehabilitation training according to Bobath and motor re‐learning method. 1 time/day, 45 minu/time, 5‐6 times/week. The main methods are as follows: (1)The patient should follow the principle of step‐by‐step passive training and active activities of each joint of the limbs;(2)The waist and abdominal muscles training, sitting and standing balance and transfer training, and bed‐chair transfer training are performed on the bed and bedside;(3) Single‐leg standing, walking training, and up and down steps training;(4) Application training of braces and walking frames;(5)Training of daily life activities such as dressing, eating, urinating, combing hair,and washing face" (2) No physical rehabilitation "The patients in the control group were only given routine medical treatment, followed up regularly, carried out health education, and measured vital signs. The patients were given self‐rehabilitation training by their families to the best of their ability"
Outcomes	**Independence in ADL scales:** Barthel Index **Motor function scales:** Fugl‐Meyer assessment **Measures of balance:** NR **Measures of gait velocity:** NR **Length of stay:** NR **Adverse events:** NR **Other measures:** NR **Time points when outcomes were assessed:** before and after 60 days of treatment
Comparison	**Amount of physical rehabilitation in groups:** differs **Categorisation of comparison:** 1. Physical rehabilitation versus no or limited physical rehabilitation (dose differs) (limited = < 50% of dose)
Funding & conflicts of interest	**Funding statement:** not reported **Conflict of interest statement:** not reported
Synthesis	**Quantitative synthesis:**Analysis 1.1; Analysis 1.2; Analysis 2.1; Analysis 2.2
Notes	This study was published in Chinese and translated into English using Google Translate.

**Table:** Zhong Qiue 2014

***Study characteristics***	
Methods	**Study aim:** to investigate the effect of bilateral limb function training on the rehabilitation of stroke patients with hemiplegia **Design:** parallel‐group RCT **Country:** China **Study recruitment and setting:** see Table **Involvement of patients/public/professionals/others:** no
Participants	**Study population (number randomised):** 64 **Inclusion criteria:** Patients who met the diagnostic criteria for stroke Diagnosed by head CT or MRI Limb paralysis and unconsciousness **Exclusion criteria:** Secondary onset or onset > two years (as diagnosed by head CT or MRI) **Participants:** detailed in Table **PROGRESS‐plus:** detailed in Table
Interventions	(1) Bilateral limb training "functional and compensatory training of the unaffected limbs was performed. The limbs are mainly based on voluntary movements, and the activities of various joints are carried out at the same time. Upper limbs include wrist and finger flexion and extension, forearm internal rotation and external rotation, elbow flexion and extension, shoulder flexion and extension, abduction and internal rotation and external rotation; lower limbs include ankle dorsiflexion, plantar flexion, varus and valgus, knee flexion and extension, hip flexion and extension, adduction and abduction and internal rotation. Training for activities of daily living, including bed activities, wheelchair activities, self‐care activities, writing, using the phone, walking (including the use of walkers), going up and down stairs, and hand coordination training, etc. 1 time/d, 45 min to 1 h each time." (2) Conventional rehabilitation "Perform functional training of the affected limb: ①The responsible nurse instructs the patient to focus on exercise therapy, including early bed positioning, mat training, muscle strength training, endurance training, sitting and standing balance training, transfer training, walking training, up and down stairs training, etc., to improve joint mobility , control key points, antispasmodic [1]; ② homework training. Mainly focus on activities of daily living, and guide patients to complete various actions in a certain order, such as putting on a shirt. Put on the other side of the sleeve → tie the front button correctly → unbutton the button → take off the shirt below the shoulder → take off the sleeve from the healthy arm → take off the sleeve from the affected arm, repeat the training, Until the patient masters coherent movements [2]. 1 time/d, 45 min to 1 h each time."
Outcomes	**Independence in ADL scales:** Barthel Index **Motor function scales:** Fugl Meyer motor function **Measures of balance:** NR **Measures of gait velocity:** NR **Length of stay:** NR **Adverse events:** NR **Other measures:** NR **Time points when outcomes were assessed:** before and after 1 month of treatment
Comparison	**Amount of physical rehabilitation in groups:** equivalent **Categorisation of comparison:** 4. Comparison of similar approaches (dose equivalent)
Funding & conflicts of interest	**Funding statement:** not reported **Conflict of interest statement:** not reported
Synthesis	**Qualitative synthesis:** studies comparing similar approaches were not combined within statistical analyses.
Notes	This study was published in Chinese and translated into English using Google Translate.

**Table:** Zhu 2001

***Study characteristics***	
Methods	**Study aim:** to investigate the effect of 3‐month rehabilitation on motor function in people with hemiplegia following a stroke **Design:** parallel‐group RCT **Country:** China **Study recruitment and setting:** see Table **Involvement of patients/public/professionals/others:** no
Participants	**Study population (number randomised):** 125 **Inclusion criteria:** Met the 1995 Fourth National Conference on Cerebral Vascular Disease classification guidelines First ever stroke confirmed by CT or MRI scan Functional deficit of limbs Aged between 45 and 70 years old No major heart, liver, kidney, and other organ diseases GCS ≥ 8 within 4 to 7 days for ischaemic stroke, within 10 to 14 days for haemorrhagic stroke Vital signs stable **Exclusion criteria:** Not reported **Participants:** detailed in Table **PROGRESS‐plus:** detailed in Table
Interventions	(1) Rehabilitation (n = 72) "Both groups received conventional medical treatment during the critical acute phase. Participants in the treatment group commenced rehabilitation once medically stable. Rehabilitation comprised exercise therapy, occupational therapy and electro therapy etc. Exercise therapy consisted of techniques mainly from the Motor Relearning Program and Bobath, customised to the impairment of the individual participants and progressed appropriately. Exercise therapy included: (i) Passive ranging exercises of all joints of the affected limbs (including scapula), starting from small to large movements, without any pain caused to the participants; (ii) Rolling practice to both sides; (iii) bridging exercises; (iv) Exercises involving the wrist and ankle joints; (v) outwith therapy, participants trained in upright sitting, beginning at an inclination angle of 30 degrees. Once participants could tolerate the angle for 30 min, the inclination was increased by 10 degrees until participants could sit upright at 90 degrees for 30mins. Thereafter, participants commenced sitting on the edge of bed exercises and lying to sitting training; (vi) sitting on the edge of bed balance training; (vii) sit to stand practice; (viii) standing balance training; (ix) gait training etc. Throughout therapy, participants’ family were taught the exercises, so that they could assist with practice outwith therapy. Electrotherapy was conducted for the affected limbs. Psychological therapy was given throughout to strengthen participant’s motivation to recover" "Although the control group was not given any therapy, some participants exercised based on the doctor’s advice, and some participants’ family assisted with exercises from their own knowledge. Upon discharge, participants from the treatment group continued with outpatient therapy or home therapy or assistance with daily exercises by their family under telephone supervision at least 3/week" Length of intervention period: not stated (outcome assessment done three months after stroke) Number of sessions and length of individual sessions: rehabilitation programme: exercise therapy and occupational therapy 1/d, 45 minutes/session, 5/week, electrotherapy 1/d, 20 minutes/session, 5/week Intervention provider: not stated (2) No physical rehabilitation(n = 53) "Received conventional medical treatment during the critical acute phase" Length of intervention period: no intervention Number of sessions and length of individual sessions: no intervention Intervention provider: no intervention
Outcomes	**Independence in ADL scales:** **Motor function scales:** Fugl‐Meyer Assessment **Measures of balance:** NR **Measures of gait velocity:** NR **Length of stay:** NR **Adverse events:** NR **Other measures:** NR **Time points when outcomes were assessed:** "Within 2 days of being medically stable or at the commencement of training as first assessment, the second assessment being 3 months after stroke"
Comparison	**Amount of physical rehabilitation in groups:** differs **Categorisation of comparison:** 1. Physical rehabilitation versus no or limited physical rehabilitation (dose differs) (limited = < 50% of dose)
Funding & conflicts of interest	**Funding statement:** not reported **Conflict of interest statement:** not reported
Synthesis	**Quantitative synthesis:**Analysis 1.2
Notes	Original study translated from Chinese to English by a member of our team (PLC).

**Table:** Zhu 2004b

***Study characteristics***	
Methods	**Study aim:** to examine the effects of standardised three‐stage rehabilitation on the neurological function in stroke patients with hemiplegia **Design:** parallel‐group RCT **Country:** China **Study recruitment and setting:** see Table **Involvement of patients/public/professionals/others:** no
Participants	**Study population (number randomised):** 52 **Inclusion criteria:** Met the 4th National Conference on Cerebral Vascular Disease classification guidelines Ischaemic or haemorrhagic stroke, confirmed by CT or MRI scan as first ever stroke Aged between 40 and 80 years old Within 1 week of being medically stable Glasgow Coma Score > 8 Functional deficit of limbs Provided informed consent **Exclusion criteria:** Liver disease or impaired kidney function Congestive heart failure Malignant tumours History of dementia Impaired respiratory system Paralysis of 4 limbs > 3 weeks post stroke Living rurally and unable to travel to be re‐assessed History of psychological disorders Deafness and/or muteness **Participants:** detailed in Table **PROGRESS‐plus:** detailed in Table
Interventions	(1) Rehabilitation(n = 26) "The intervention combined physiotherapy (PT) and occupational therapy (OT): The earlier stage of recovery (from stroke onset to one month after stroke) focused on physiotherapy, and the later stage on occupational therapy PT included: (i) anti‐spasticity positioning in supine and sitting; (ii) passive ranging of all joints of affected limbs from small to larger range, within pain‐free thresholds, including scapular activity; (iii) rolling from both sides; (iv) bridging practice; (v) wrist extension and ankle dorsiflexion training; (vi) Outwith therapy, participants trained sitting tolerance, starting from a bed height of 30 degrees, and increasing the height by 10 degrees each day if participants were able to cope for 30 mins until 30 mins at 90 degrees is achieved; (vii) Sitting balance training on the bedside; (viii) sit to stand training; (ix) Standing balance training. (x) Gait training etc" "In the flaccid phase, participants focused on correct positioning, passive exercises and active‐passive training, rolling, lying to sitting; in the spastic phase, participants focused on relaxation practice (biofeedback technique), anti‐spasticity techniques and training of non‐spastic muscles, and isolation training. OT: Depending on participant’s ability, feeding, washing, donning, transfer from bed to wheelchair and vice versa and other ADL training, deep breathing and abdominal training, supine to sitting up, sitting balance and sit to stand training etc, 1/day, 45min/session, 5/week" "During the intervention, family members were taught how to facilitate training and care so that participants could get practice even out with therapy, and to prevent injuries due to inappropriate handling of the affected limbs" "Second stage of rehabilitation (from the second to end of third month after stroke) mainly involved standing training, standing balance training, single leg standing, gait training and stairs training etc, in order to resolve the participant’s ambulatory ability, 2/day, 30‐45min/session, 5 days/week; third stage of rehabilitation (from the fourth to the end of the sixth month after stroke) focused on feeding, donning, washing, hygiene issues and other ADL training, 2/day, 30‐40min/session, 5‐7days/week" Length of intervention period: 6 months Number of sessions and length of individual sessions: dependent on stage of recovery (see above) Intervention provider: therapists (2) No physical rehabilitation (n = 26) "The control group was not given any therapy. However, some participants exercised based on the doctor’s advice, and some participants’ family assisted with exercises from their own knowledge. Participants might also have sought help from other rehabilitative services upon discharge" Length of intervention period: no intervention Number of sessions and length of individual sessions: no intervention Intervention provider: no intervention
Outcomes	**Independence in ADL scales:** NR **Motor function scales:** NR **Measures of balance:** NR **Measures of gait velocity:** NR **Length of stay:** NR **Adverse events:** NR **Other measures:** clinical neurological deficit score (translated) **Time points when outcomes were assessed:** at enrolment and end of 1, 3, and 6 months after stroke
Comparison	**Amount of physical rehabilitation in groups:** differs **Categorisation of comparison:** 1. Physical rehabilitation versus no or limited physical rehabilitation (dose differs) (limited = < 50% of dose)
Funding & conflicts of interest	**Funding statement:** research funding reported, source unclear ("基金项目：国家”卡五．．攻关基金~助项IJ(200lllA7031l2 门") **Conflict of interest statement:** none reported
Synthesis	**Qualitative synthesis:** no relevant outcomes for inclusion in meta‐analysis
Notes	Original study translated from Chinese to English

**Table:** Zhu 2006

***Study characteristics***	
Methods	**Study aim:** to evaluate the effectiveness of early nursing rehabilitative care and treatment on the recovery of both motor function and ADL in stroke **Design:** parallel‐group RCT **Country:** China **Study recruitment and setting:** see Table **Involvement of patients/public/professionals/others:** no
Participants	**Study population (number randomised):** 70 **Inclusion criteria:** Ischaemic or haemorrhagic stroke, confirmed by CT or MRI scan First ever stroke Aged between 55 and 80 years old Functional deficit of limbs No severe cognitive issues No severe diseases of the heart, liver, kidney, and other organs Within 1 week of being medically stable GCS > 8 **Exclusion criteria:** Not reported **Participants:** detailed in Table **PROGRESS‐plus:** detailed in Table
Interventions	(1) Rehabilitation (n = 35) "Both groups of patients received the same drug therapy, including treatment for cerebral edema, brain care and improving blood circulation. 3 – 7 days after becoming medically stable, the test group underwent rehabilitative therapy using Bobath technique, Rood technique, as well as ADL training, etc, with training conducted by rehabilitation nurses, rehabilitative therapy once a day, 1 hr each time, 5 times a week" "The contents were as follows: Physiotherapy: (i) On‐bed positioning of healthy limbs, with regular turning over; (ii) Passive ranging exercises of joints of limbs on affected side, including passive ranging exercise of scapular, motion progressing from proximal joints to distal joints, range of motion progressed from small to large, within pain‐free thresholds, while concurrently, patients were encouraged to use healthy limbs to aid motion of affected limbs, for example exercise involving crossing both sides and lifting, lower limbs bridge‐style exercise; (iii) Utilising Rood technique to brush, tap, pat etc arbitrary exercises to stimulate affected limbs; (iv) Sitting exercise involving lifting headrest, headrest gradually lifted, maintaining each position 30min, repeating training with 10 degree increments until able to sit upright at bedside; (v) Bedside sitting balance training: correct sitting posture, starting from static balance to dynamic balance training, torso back‐and‐forth, side‐to‐side and rotation training, and finally training of maintaining balance while being pushed externally; (vi) Sit‐to‐stand balance training, patients holding hands Bobath‐style, extending upper limbs, head and torso leaning forward, moving center‐of‐gravity forward, torso, hip and knee extending until standing, during standing process, body weight distributed equally on both sides, and then undergoing training of moving body weight back‐and‐forth, side‐to‐side; (vii) Gait training, after patient’s standing balance and affected limbs weight bearing ability improved, starting from gait training between parallel bars to gait training using walking stick and eventually progressing to training of stair climbing and descending" "Occupational therapy: (i) For patients with difficulty swallowing, training was done to stimulate face, tongue and lips, opening and closing of lips, opening and closing of lower jaw, tongue pushing upper palate, extension of tongue, etc, or using ice‐cold cotton bud to stimulate swallowing reflex; (ii) Activity involving the palm and all joints of the fingers as well as agility, coordination and dexterity of the fingers training; (iii) ADL training, including brushing, feeding, washing, donning, passing bowels etc, encouraging the completion of tasks using the affected limbs, or breaking the tasks into components and getting participants to train specific components" Length of intervention period: not stated Number of sessions and length of individual sessions: once a day, 1 hour each time, 5 times a week Intervention provider: rehabilitation nurses (2) No physical rehabilitation (n = 35) Both groups of patients received the same drug therapy, including treatment for cerebral oedema, brain care, and improving blood circulation. Length of intervention period: no intervention Number of sessions and length of individual sessions: no intervention Intervention provider: no intervention
Outcomes	**Independence in ADL scales:** Modified Barthel Index **Motor function scales:** Fugl‐Meyer Assessment (simplified) **Measures of balance:** NR **Measures of gait velocity:** NR **Length of stay:** NR **Adverse events:** NR **Other measures:** Brunnstrom Grading Scale **Time points when outcomes were assessed:** before and after intervention
Comparison	**Amount of physical rehabilitation in groups:** differs **Categorisation of comparison:** 1. Physical rehabilitation versus no or limited physical rehabilitation (dose differs) (limited = < 50% of dose)
Funding & conflicts of interest	**Funding statement:** not reported **Conflict of interest statement:** not reported
Synthesis	**Quantitative synthesis:**Analysis 1.1; Analysis 1.2
Notes	Original study translated from Chinese to English by a member of our team (PLC).

**Table:** Zhu 2007

***Study characteristics***	
Methods	**Study aim:** "investigate the effects of standardized rehabilitation treatment on ADLs in patients with hemiplegia after stroke" **Design:** parallel‐group RCT **Country:** China **Study recruitment and setting:** see Table **Involvement of patients/public/professionals/others:** no
Participants	**Study population (number randomised):** 78 **Inclusion criteria:** Provided informed consent Medically stable for > 48 hours Glasgow Coma Scale > 8 Functional deficit of limbs **Exclusion criteria:** Active liver disease Impaired liver or kidney function Congestive heart failure Impaired respiratory system Malignant tumours History of dementia History of psychological disorders Paralysis of 4 limbs > 4 weeks post stroke History of previous stroke which resulted in functional deficit of limbs Unable to allow re‐assessments to take place Deafness and/or muteness **Participants:** detailed in Table **PROGRESS‐plus:** detailed in Table
Interventions	(1) Rehabilitation (n = 40) (infarct group ‐ n = 28, haemorrhage group ‐ n = 12) "All patients received routine clinical treatment and care. Treatment group was transferred to rehabilitation centre after becoming medically stable, and under guidance from the therapists, utilised physiotherapy and occupational therapy to undergo integrated rehabilitative treatment; patients on being discharged to their homes, were visited by therapists who would teach the required training, until the follow‐up ended" "Key training contents included: (i) From 1 week after medical stabilisation till one month after onset of stroke, focus was on bed and bed‐side exercises, including anti‐spasticity positioning, passive exercises on affected limbs and neuro‐muscular stimulative technique, active exercises on non‐affected limbs, truncal muscle control training, shifting on bed, rolling (affected side, non‐affected side), sitting up, sitting‐balance training, sitting position–standing position transfer as well as eating, grooming, dressing etc ADL training, once a day, 45 min each time, 5 days a week. (ii) From start of 2nd month till end of 3rd month after onset of stroke, focus was on standing training, including standing‐balance training, single‐leg weight bearing, gait and stair climbing/descending, and other trainings, while providing guidance on toileting, bed‐chair transferring, indoors or outdoors walking, use of stairs, washing and other practical ADLs; twice a day, 45 min each time, 5 days a week. Because muscle tone could increase gradually during this period, it was necessary to increase the intensity of trainings to reduce muscle tone and inhibit abnormal exercise patterns. For some patients who returned to community setting, therapists would conduct weekly home‐visits to guide the patient on rehabilitative treatment. (iii) Once training had commenced, therapists concurrently taught the patients’ family members or caregiver on the correct assistive training methods and care methods, such that they could provide some training outwith therapy time, while also reducing the secondary damage due to inappropriate care. (iv) Patients learned to monitor their own body for discomfort, and report on time to therapist and caregiver" Length of intervention period: not stated Number of sessions and length of individual sessions: dependent on stage of recovery (see above) Intervention provider: therapists (2) No physical rehabilitation (n = 38) (infarct group ‐ n = 28; haemorrhage group ‐ n = 10) "Control group was not given standard rehabilitative treatment, but were allowed to perform activities independently under doctor’s advice or with assistance from nurses" Length of intervention period: no intervention Number of sessions and length of individual sessions: no intervention Intervention provider: no intervention
Outcomes	**Independence in ADL scales:** Barthel Index **Motor function scales:** Fugl‐Meyer Assessment **Measures of balance:** NR **Measures of gait velocity:** NR **Length of stay:** NR **Adverse events:** NR **Other measures:** NR **Time points when outcomes were assessed:** at allocation to groups and end of 1 and 3 months after stroke
Comparison	**Amount of physical rehabilitation in groups:** differs **Categorisation of comparison:** 1. Physical rehabilitation versus no or limited physical rehabilitation (dose differs) (limited = < 50% of dose)
Funding & conflicts of interest	**Funding statement:** not reported **Conflict of interest statement:** not reported
Synthesis	**Quantitative synthesis:**Analysis 5.4
Notes	Original study translated from Chinese to English by a member of our team. Data provided are means and ranges. For analysis, standard deviations have been estimated by calculating (upper range ‐ lower range)/4

**Table:** Zhu 2016

***Study characteristics***	
Methods	**Study aim:** "to qualify the improvements of modified constraint‐induced movement therapy (m‐CIMT) on the lower limb of stroke patients via assessing the centre of mass (COM) displacement and the basic gait parameters." **Design:** parallel‐group RCT **Country:** China **Study recruitment and setting:** see Table **Involvement of patients/public/professionals/others:** no
Participants	**Study population (number randomised):** 22 **Inclusion criteria:** Persistent hemiplegia Persistent hemiplegia for 3 to 6 months following first‐time stroke (cerebral infarction or haemorrhagic) Confirmed using CT or MRI Stable vital signs Clear consciousness Able to follow the study related instructions Brunnstrom stages of lower extremity were II–III No serious uncontrolled medical comorbidities (e.g. cognitive dysfunction, Alzheimer's disease, cardiopulmonary insufficiency and other lower limb orthopaedic impairments) **Exclusion criteria:** MMSE ≤ 23 Severe medical history (e.g. myocardial ischaemia, hypertension, diabetic nephropathy, proliferative retinopathy) Unable to exercise **Participants:** detailed in Table **PROGRESS‐plus:** detailed in Table
Interventions	(1) Constraint‐induced movement therapy (CIMT) "Apart from the standardised comprehensive rehabilitation treatment, patients in the m‐CIMT group also received m‐CIMT gait training for about 2 h per day which included:(a) sit to stand transfers by using a suitable chair (controlling the position of the paretic leg), 200–300 times per day; (b) indoor walking training under physical therapy guidance, 20 min and about 1000 m per day, not faster than 1.3 km/h; (c) climbing up and down stairs training for 18 steps/time and totally four times per day with a specialised staircase for indoor rehabilitation exercise; (d) balance training by sitting on Bobath‐based approach under therapist’s guidance, or standing on an inclined plate with angle of 0, 7,18, 30, 40, and 45 from 2 min to 4 min, respectively; (e) one leg weight training on paretic and non‐paretic leg; (f) muscle strength training by bridging exercises for 8–12 times and totally 4 times per day. The participant was strongly encouraged to incorporate the use of the hemiparetic lower limb into his daily activities." (2) Conventional therapy "The patients in the control group received standardised comprehensive rehabilitation treatment for 45 min per day which consisted of three main elements. These included: (a)passive exercise: range of motion exercises and stretching exercises; (b) active exercise: balance training and walking training; (c) rehabilitation education and guidance, and some adjuvant therapy such as position transfer practise under families’ supervision." "Patients in both the groups had been treated for five days per week for four weeks (totally 20 consecutive weekdays)."
Outcomes	**Independence in ADL scales:** NR **Motor function scales:** NR **Measures of balance:** NR **Measures of gait velocity:** gait velocity **Length of stay:** NR **Adverse events:** NR **Other measures:** gait analysis **Time points when outcomes were assessed:** pre‐ and post‐treatment
Comparison	**Amount of physical rehabilitation in groups:** differs **Categorisation of comparison:** 5. Additional therapy + usual therapy versus usual therapy (dose differs)
Funding & conflicts of interest	**Funding statement:** "supported by biological medicine major projects of Shanghai Science and Technology Commission (Grant No. 10DZ1950800) and Provincial Key Laboratory Open Fund of Shanghai University of Sport (Grant No. YZ201205)." **Conflict of interest statement:** not reported
Synthesis	**Quantitative synthesis:**Analysis 5.4
Notes	

**Table:** Zhuang 2012

***Study characteristics***	
Methods	**Study aim:** "to compare acupuncture and physiotherapy for effectiveness and reliability in treating hemiplegic patients after stroke" **Design:** multi‐arm RCT (3‐arm) **Country:** China **Study recruitment and setting:** see Table **Involvement of patients/public/professionals/others:** no
Participants	**Study population (number randomised):** 274 **Inclusion criteria:** Recent (> 2 weeks to < 3 months) history of ischaemic stroke Clinical signs and imaging confirmed stroke Admitted to a specialist stroke unit as an inpatient Age from 45 to 70 years, either sex Able to give informed consent "score better than 10 on the Neurologic Defect Scale (NDS), which ranges from 0 to 45, with 45 as most severe" **Exclusion criteria:** Haemorrhagic cerebrovascular disease, vascular disease, and dysfunction History of dementia or other mental illness Cancer Diseases transmissible by blood Severe disease of the heart, liver, kidney, haematopoietic system, or endocrine system Severe visual or hearing impairment History of previous acupuncture Fear of needling **Participants:** detailed in Table **PROGRESS‐plus:** detailed in Table
Interventions	(1) Rehabilitation (n = 86) "The research team based the physiotherapy, a conventional rehabilitative method for stroke, on the Bobath approach, which intends to restore normal movement and improve muscle strength" The individual components delivered are listed in Table 28. Based on the individual components, this intervention is categorised as comprising neurophysiological intervention. Length of intervention period: 4 weeks Number of sessions and length of individual sessions: "six 60‐minute sessions per week of standard physiotherapy and six 45‐minute sessions per week of occupational therapy" Intervention provider: "qualified therapists. A physiotherapist tailored the treatment protocol to each participant’s needs, based on recovery stage" (2) Acupuncture (n = 91) "All participants received conventional care as needed, including psychological counselling, standard nursing care, and daily medical evaluation. Attending physicians, blinded to the participant’s treatment assignment, prescribed medications when appropriate, including drugs for controlling blood glucose concentration, blood lipid levels, and blood pressure. The study allowed antiplatelet agents and anticoagulants at the discretion of the attending physician" "Well‐trained, qualified, experienced acupuncturists performed the acupuncture treatments, with participants in the supine position. The acupuncturists used sterile, disposable needles: 30‐gauge (0.3 mm in diameter), 40‐mm–long needles for limb points and 32‐gauge (0.25 mm in diameter), 25‐mm–long needles on the head. When the participant felt *de qi*—the sensation characterized by heaviness, distension, soreness, or numbness—the acupuncturist kept the needles in situ for 30 minutes without manual or electrical stimulation. The acupuncturists followed the recommendations of a standard acupuncture textbook for the depth and angle of insertion into each acupoint. The acupuncturist needled three primary scalp points on the stroke side: the first, 2 in above the ear apex and the others, 1 in anterior and 1 in posterior to the first. The acupuncturist selected secondary acupoints based on traditional Chinese medicine (TCM) theory. Patients with flaccid paralysis received *Quchi* (LI11), Waiguan (TE5), and *Hegu* (LI4) for the upper limb and *Futu* (ST32), *Zusanli* (ST36), and *Taichong* (LR3) for the lower limb. Patients with spastic paralysis received *Jiquan* (HT1), *Chize* (LU5), and *Neiguan* (PC6) for the upper limb and *Yinlingquan* (SP9) and *Sanyinjiao* (SP6) for the lower limb" Length of intervention period: 4 weeks Number of sessions and length of individual sessions: each session lasted at least 30 minutes. "Participants received treatments once a day except on Sundays" Intervention provider: "Well‐trained, qualified, experienced acupuncturists" (3) Rehabilitation + acupuncture (n = 97) "Received both acupuncture and physiotherapy" (as previously described). "The acupuncture session took place randomly before or after the physiotherapy session and during the same half‐day" The individual components delivered are listed in Table 28. Based on the individual components, this intervention is categorised as comprising modality and neurophysiological intervention. Length of intervention period: 4 weeks Number of sessions and length of individual sessions: "participants received treatments once a day except on Sundays" Intervention provider: "qualified therapists" Comparison of relevance to this review: Group (3) vs Group (2)
Outcomes	**Independence in ADL scales:** Modified Barthel Index **Motor function scales:** Fugl‐Meyer Assessment **Measures of balance:** NR **Measures of gait velocity:** NR **Length of stay:** NR **Adverse events:** adverse events **Other measures:** Neurologic Defect Scale (NDS) **Time points when outcomes were assessed:** "research team evaluated all patients at baseline, after 2 weeks, and after 4 weeks"
Comparison	**Amount of physical rehabilitation in groups:** differs **Categorisation of comparison:** 5. Additional therapy + usual therapy versus usual therapy (dose differs) (Note: we have categorised acupuncture as 'usual therapy').
Funding & conflicts of interest	**Funding statement:** "The National Key Technology R&D Program (2006BAI12B02‐03) supported the present study" **Conflict of interest statement:** not reported
Synthesis	**Quantitative synthesis:**Analysis 5.1; Analysis 5.2; Analysis 5.5
Notes	For analysis in the previous version of this review, we just used Groups (1) and (2). However, for this update we have re‐considered the relevant comparison, and have now used Group (3) and (2).

ADL: activities of daily living AEP: additional early physiotherapy BBS: Berg Balance Scale BI: Barthel Index BP: blood pressure BPM: balance performance monitor BRS: Brunnstrom Recovery Stage CCT: circuit class therapy CG: control group CIMT: constraint‐induced movement therapy CME: care‐giver mediated exercise CNDS: Clinical Neurological Deficit Scale CNS: central nervous system CPT: computed physiotherapy CSS: Chinese scoring criteria system for clinical neurological deficits CT: computed tomography CTE: cognitive therapeutic exercise CVA: cerebrovascular accident DDNF: degree of deficit of neural function EADL: extended activities of daily living EG: experimental group EMG: electromyograph ESS: European Stroke Scale FAI‐3: Frenchay Activities Index FAC: Functional Ambulatory Category FCA: Functional Comprehensive Assessment FES‐I: Falls Efficacy Scale–International FIM: Functional Independence Measure FMA: Fugl‐Meyer Assessment FRT: Functional Reach test FSS: Fatigue Severity Scale FST: functional strength training GCS: Glasgow Coma Scale HADS: Hospital Anxiety and Depression Scale HCA: haemorrhagic cerebral accident HIFE: High Intensity Functional Exercises HMSI: Hindi Mental State Examination IADL: instrumental activities of daily living ICA: infarct cerebral accident ICH: intracerebral haemorrhage IG: intervention group IQR: interquartile range JTHFT: Jebsen Taylor Hand Function Test LE: lower extremity LL: lower limb LOTCA: Loewenstein Occupational Therapy Cognitive Assessment m: metre MAS: Modified Ashworth Scale MCA: middle cerebral artery  MRS: Modified Rankin Score MAS: Motor Assessment Scale MI: Motricity Index MIDI: Musical Instrument Digital Interface MIQ‐RS: Movement Imagery Questionnaire—revised second version MMSE: Mini Mental State Examination MRI: magnetic resonance imaging MS: multiple sclerosis n: number of participants NDS: Neurologic Defect Scale NDT: neurodevelopmental treatment NEADL: Nottingham Extended Activities of Daily Living NIHSS: National Institutes of Health Stroke Scale NR: not reported NYHA: New York Heart Association OT: occupational therapy PADS: Physical Activity and Disability Scale PBT: perturbation‐based balance training PA: physical activity PD: Parkinson's Disease PLBO: placebo PNF: proprioceptive neuromuscular facilitation PT: physiotherapy RCT: randomised controlled trial RIND: reversible ischaemic neurological deficit RMI: Rivermead Mobility Index ROM: range of movement RS: rhythmic stabilisation RT: routine therapy SAIS: Stroke Assessment Impairment Set SARS: severe acute respiratory syndrome SD: standard deviation SEP: somatosensory evoked potential SMES: Sodring Motor Evaluation of Stroke patients SPSS: Statistical Package for the Social Sciences SR: stabilising reversal TBI: traumatic brain injury TENS: transelectrical nerve stimulation TFR: traditional functional retraining TIA: transient ischaemic attack TIS: Trunk Impairment Scale TRT: treatment TSCT: task‐specific circuit training TUG: Timed Up and Go test UE: upper extremity VAS: visual analogue scale WAB: Wester Aphasia Battery WHO: World Health Organization

### Characteristics of excluded studies [ordered by study ID]

**Table:**

Study	Reason for exclusion
Ambrosini 2019	Intervention is focussed on use of technology rather than physical rehabilitation
Büyükavcı 2016	Intervention falls within scope of another Cochrane review: 'Trunk training for improving activities in people with stroke'
Cabanas‐Valdés 2016	Intervention falls within scope of another Cochrane review: 'Trunk training for improving activities in people with stroke'
Cabanas‐Valdés 2021	Intervention falls within scope of another Cochrane review: 'Trunk training for improving activities in people with stroke'
Chan 2015	Intervention falls within scope of another Cochrane review: 'Trunk training for improving activities in people with stroke'
ChiCTR2000038142 2020	Intervention falls within scope of another Cochrane review: 'Trunk training for improving activities in people with stroke'
Clay 2018	Aim of the intervention was to improve the health and wellbeing of people following stroke rather than improving physical function
Dean 1997	Focus of intervention is sitting balance/trunk training. Intervention is all done sitting. Study was included in previous versions of this review, but excluded in 2022 update.
Dean 2007	Focus of intervention is sitting balance/trunk training. Intervention is all done sitting. Study was included in previous versions of this review, but was excluded from 2022 update.
Dubey 2018	Intervention falls within scope of another Cochrane review: 'Trunk training for improving activities in people with stroke'
Du Yuzhen 2014	Not a randomised study
Fan 2020	Intervention falls within scope of another Cochrane review: 'Trunk training for improving activities in people with stroke'
Giraux 2008	This study was listed as awaiting assessment in previous version of this review, requiring further details of intervention. No further details have been obtained, therefore it has been excluded.
Haruyama 2017	Intervention falls within scope of another Cochrane review: 'Trunk training for improving activities in people with stroke'
Inoue 2022	Intervention falls within scope of another Cochrane review: 'Trunk training for improving activities in people with stroke'
Karthikbabu 2018	Intervention falls within scope of another Cochrane review: 'Trunk training for improving activities in people with stroke'
Karthikbabu 2022	Intervention falls within scope of another Cochrane review: 'Trunk training for improving activities in people with stroke'
Khallaf 2020	Intervention falls within scope of another Cochrane review: 'Trunk training for improving activities in people with stroke'
Kilinç 2016	Intervention falls within scope of another Cochrane review: 'Trunk training for improving activities in people with stroke'
Kim 2011	Focus of intervention is trunk training (given in addition to conventional rehabilitation). This study was previously included, but has been excluded from the 2022 update following clarifications to inclusion criteria, which now exclude studies focussed on trunk training (which is the focus of a separate Cochrane review).
Lee 2019	Intervention falls within scope of another Cochrane review: 'Interventions for improving community ambulation in individuals with stroke'
Lee 2020	Intervention falls within scope of another Cochrane review: 'Trunk training for improving activities in people with stroke'
Li 2000	This study was listed as awaiting assessment in previous version of the review. Original study translated from Chinese to English. This study is a follow‐up from an earlier study undertaken in 1998. British Library unable to supply this earlier publication, and, despite extensive searching, the review authors have not been able to find any other known UK locations for it. Clarification of intervention from the earlier RCT was sought from the study authors but was not obtained.
Liao 2006	Focus of intervention is trunk training (given in addition to conventional rehabilitation). This study was previously included, but has been excluded from the 2022 update following clarifications to inclusion criteria, which now exclude studies focussed on trunk training (which is the focus of a separate Cochrane review).
Liu 2017	Intervention falls within scope of another Cochrane review: 'Dual task training for improving balance and gait in people with stroke'
Marzouk 2019	Intervention falls within scope of another Cochrane review: 'Trunk training for improving activities in people with stroke'
Matsumoto 2010	This study was listed as awaiting assessment in previous version of the review, requiring further clarification of the intervention. Changes to our inclusion criteria now mean that this is excluded on the basis that the focus is on trunk training.
Mudie 2002	Focus of intervention is sitting balance/trunk training. Intervention is all done sitting (or lying). This study was previously included, but has been excluded from the 2022 update following clarifications to inclusion criteria, which now exclude studies focussed on trunk training (which is the focus of a separate Cochrane review).
Poletto 2015	Intervention falls within scope of another Cochrane review: 'Very early versus delayed mobilisation after stroke'
Pollock 1998	Focus of intervention is sitting balance/trunk training. Intervention is all done sitting. This study was previously included, but has been excluded from the 2022 update following clarifications to inclusion criteria, which now exclude studies focussed on trunk training (which is the focus of a separate Cochrane review).
Qian 2005	This study was included in previous versions of this review. However, the decision was made to exclude from this updated version as the intervention was judged to be a single treatment component (flexor reflex therapy).
Richardson 2011	This study was listed as awaiting assessment in previous version of the review. The completed study was assessed during screening and considered not to meet inclusion criteria.
Sanchez‐Sanchez 2011	This study was listed as awaiting assessment in previous version of the review. The completed study was assessed during screening and considered not to meet inclusion criteria.
Shahimoridi 2020	Aim of study is to evaluate functional electrical stimulation rather than physical rehabilitation
Sharma 2017	Intervention falls within scope of another Cochrane review: 'Trunk training for improving activities in people with stroke'
Sivertsen 2022	Intervention falls within scope of another Cochrane review: 'Trunk training for improving activities in people with stroke'
Sun 2016	Intervention falls within scope of another Cochrane review: 'Trunk training for improving activities in people with stroke'
SWEAT2 2020	Intervention falls within scope of another Cochrane review: 'Trunk training for improving activities in people with stroke'
Verheyden 2006	Focus of intervention is trunk training. This study was previously included, but has been excluded from the 2022 update following clarifications to inclusion criteria, which now exclude studies focussed on trunk training (which is the focus of a separate Cochrane review).
Wang 2005a	This study was listed as awaiting assessment in previous version of the review. British Library unable to supply this publication, and, despite extensive searching, the review authors have not been able to find any other known UK locations for it. Clarification of intervention was sought from the study authors but was not obtained. We have therefore excluded this study.
Wang 2012	This study was listed as awaiting assessment in previous version of this review, requiring further details of intervention. No further details have been obtained, therefore it has been excluded.
Wei 1998	Focus of intervention is trunk training. This study was previously included, but has been excluded from the 2022 update following clarifications to inclusion criteria, which now exclude studies focussed on trunk training (which is the focus of a separate Cochrane review).
Wellmon 1997	This study was included in previous versions of this review. The decision was made to exclude it from this updated version, as the intervention studied (a stepping task) was re‐considered and judged to comprise a single treatment component.
Wright 2021	This study was initially included as it was thought that it was a 3‐arm trial comparing: (1) "bionic leg" training, (2) "normal physiotherapy", and (3) "no physiotherapy". The comparison of group (2) with group (3) was considered relevant to this review. However, on publication of the results paper, and further consideration of the protocol, we noted that the "no physiotherapy" group was NOT a randomised allocation, but rather was a non‐randomised cohort "recruited from community‐based support groups". As the comparison of group (1) with group (2) is not relevant to this review, we have excluded this study.
Yau 2010	This study was listed as awaiting assessment in previous version of the review, but was published as an abstract only. Clarification of physical therapy intervention was sought but was not obtained. This study has therefore been excluded.
Yen 2020	Intervention falls within scope of another Cochrane review: 'Very early versus delayed mobilisation after stroke'
Yoo 2010	Intervention falls within scope of another Cochrane review: 'Trunk training for improving activities in people with stroke'
Zhang 2008	This study was listed as awaiting assessment in previous version of the review, but was published as an abstract only and no further details were obtained despite contacting authors. It is unclear if this study is randomised, or whether the intervention meets the inclusion criteria, so it has therefore been excluded.
Zhao Dianwei 2014	The intervention is focussed on pelvic/trunk training. Ankle dorsiflexion passive stretch is also given to the treatment group, however this was considered a 'single component', and was passive/not focussed on improving functional ability.

n: number of participants RCT: randomised controlled trial

### Characteristics of studies awaiting classification [ordered by study ID]

**Table:** Chang 2017

Methods	RCT
Participants	"78 cases with cerebral infarction treated in our hospital fromJuly 2015 to July 2016 are taken as research objects. All patients meet the diagnosis standard about “cerebral infarction”in cerebrovascular system diseases. According to random number table, the groups are divided into the observation group and control group, having 35 cases respectively."
Interventions	(1) Early nursing intervention ‐ including: body positioning, movement, standing and walking training, language training (2) Control group
Outcomes	Fugl‐Meyer Assessment for language and limb function
Notes	Study initially included, but then re‐assessed to be an "early nursing" intervention, similar to the other studies of Chinese early nursing which are awaiting assessment. This study has therefore been moved to "awaiting assessment", and will be considered at the same time as the other "early nursing" studies.

**Table:** Chen 2004

Methods	Study design: RCT Method of randomisation: not stated
Participants	Number of participants: n = 78 Inclusion criteria: "Met the 1995 National stroke diagnosis guidelines, first stroke as confirmed by CT or MRI scan, persistent deficits in motor function, within three weeks after stroke, no severe conditions of the heart, liver, kidney and medically stable" Age stated in paper as "between 40‐48." However, review authors have assumed that this is a typo, as mean age reported in the study is 60.95 years for the rehabilitation group and 62.36 years for the control group.
Interventions	(1) Rehabilitation group (n = 39) "Patients in the rehabilitation group were given physical training in addition to routine treatment. The treatment group commenced daily therapy after they were medically stable. Each training session began with the therapist guiding and delivering the therapy lasting 45 min/day. Participant's family learned the exercises alongside each training session, using approximately 0.5 hour/day to consolidate and reinforce the exercises taught" The intervention mainly consisted of the following. (a) Correct positioning of limbs in bed (supine position, lateral position with affected limbs at lower side and healthy limbs at upper side and lateral position with affected limbs at upper side and healthy limbs at lower side) (b) Passive ranging exercises of all joints of the affected limbs (therapist placed one hand on the proximal joint and another hand on the distal joint to deliver gentle, slow and rhythmic movements to the joints) (c) Neuromuscular facilitation techniques (combined joint repositioning and compression while performing ranging exercises on the limbs; tapping, brushing and striking the skin) (d) Active ranging exercises of the affected limbs (used a band to assist the affected limbs in carrying out active‐assisted range of motion exercises at the shoulder, elbow and wrist, as well as active ranging exercises of all joints) (e) Training in sitting balance, standing and gait re‐education, commencing up stair and down stair practice when possible (f) ADL training (participants actively worked on completing eating, washing, combing, dressing activities); learned all possible techniques to achieve the above actions, participants with severe impairment to learn single‐hand and single‐leg techniques of manoeuvring the wheelchair Length of intervention period: not stated, but final outcome assessment was done after 3 months of therapy Number of sessions and length of individual sessions: 45 minutes per day. No further details stated Intervention provider: therapist during training session. Participants’ family provided reinforcement of exercises taught during training session (2) No rehabilitation group (n = 39) "Patients in the control group received routine treatment" Length of intervention period: not stated Number of sessions and length of individual sessions: not stated Intervention provider: not stated
Outcomes	Measures of independence in ADL: Barthel Index Time points when outcomes were assessed: "before therapy (not stated when specifically), after 1 month of therapy, after 3 months of therapy"
Notes	Original study translated from Chinese to English This study was previously included, but has been excluded from the 2022 update following identification of numerous studies, conducted in China, investigating the efficacy of "early rehabilitation" or "early nursing". This study has therefore been moved to "awaiting assessment" and will be re‐considered at the same time as the other "early nursing" studies.

**Table:** Chen 2006

Methods	Study design: RCT Method of randomisation: not stated
Participants	Number of participants: n = 45 Inclusion criteria: "stroke patients diagnosed according to the evaluative standard revised by the Fourth [National] Academic Conference of Cerebral Vascular Disease and lived in communities around the Second Hospital of Zhengzhou University from 2003 to 2005"
Interventions	(1) Rehabilitation group (n = 25) "All patients underwent the same routine medical treatment, patients from the rehabilitation group [were] additionally treated with community‐based rehabilitation for 3 months. Appropriate therapies were given after detailed examination and rehabilitation assessment, giving community‐based rehabilitation training at home, giving the patient and family members comprehensive guidance, with regular follow up assessment, with all questions encountered during the rehabilitation process answered timely, and continuously adjusting the rehabilitation treatment according to the patient’s response" Rehabilitation measures: "Treatment during the flaccid stage was on preventing joint contractures and deformity, preventing secondary complications; treatment during the spasticity stage was on controlling muscle spasticity and abnormal movements to encourage normal movement patterns to emerge. Main content included: passive ranging exercises of all joints, rolling from affected and non‐affected sides practice, balance ability training (including sitting and standing balance practice), transfers (bed to chair transfer, sit to stand transfer) training, gait training (ambulation, up and down stairs) and stretching in wrist extension and ankle dorsiflexion. ADL training included feeding, donning, personal hygiene management etc. At the same time, psychological recovery and social adaptation training were given, mainly on fostering good patient‐doctor relationships to help them build confidence, release negative emotions, guide and encourage them to express their feelings. Training was given in a way that adapted to the training environment, overcame limitation in resources through simplification, adapted to the situation and presenting condition, focused on involving the family members’ participation, alterations to the home environment, maximising the resources at home" Length of intervention period: 3 months Number of sessions and length of individual sessions: 2/wk; no other details given Intervention provider: not stated (2) No rehabilitation group (n = 20) "Routine medical treatment" Length of intervention period: not stated Number of sessions and length of individual sessions: not stated Intervention provider: not stated
Outcomes	Measures of independence in ADL: Barthel Index Other secondary outcomes: Mini Mental State Examination (MMSE) Time points when outcomes were assessed: before intervention and after 3 months of community‐based rehabilitation
Notes	Original study translated from Chinese to English This study was previously included, but has been excluded from the 2022 update following identification of numerous studies, conducted in China, investigating the efficacy of "early rehabilitation" or "early nursing". This study has therefore been moved to "awaiting assessment" and will be re‐considered at the same time as the other "early nursing" studies.

**Table:** Chen 2010

Methods	Study design: RCT Method of randomisation: not stated
Participants	Number of participants: n = 106 Inclusion criteria: first‐ever stroke between February 2006 and December 2008, no obvious sign of psychological or memory deficits, fulfilled neurological examination standards and confirmed by CT scan or MRI
Interventions	(1) Rehabilitation group (n = 53) "Test group used rehabilitation exercise therapy (a) Passive ranging exercises (rehabilitation method during flaccid period): while patient was in supine, therapist provided exercise therapy to each of the patient’s joints according to movable range of each joint (see Table 29), with the following exercise principles: (i) Exercise progressed from proximal joints to distant joints; (ii) Exercise single joints first ‐> gradually progressing to combined movement of several joints; (iii) Exercise upper and lower limbs on non‐affected side first, until patient became used to it, before exercising affected limbs; (iv) Each exercise done slowly 3s – 5s, repeated 5 times – 10 times, at beginning using slow and gentle motion, avoiding overly fast flexing and extending, paying attention to patient’s pain level, avoiding straining. Only if exercise direction was correct, would a safe and effective rehabilitation goal be realised. (b) Active ranging exercises (rehabilitation exercise during recovery period): patient independently chose the exercise position and exercise method, with emphasis on hand exercises, assisted by some equipment, we gave appropriate guidance and monitoring, with exercise speed, repetition number and interval being determined by patient’s specific condition. Active ranging exercises had to obey: (i) Active ranging exercises performed on the foundation of passive ranging exercises, in order not to induce tiredness and pain; (ii) Among the exercises, more practice done for relaxation of tensed muscles; (iii) First simple movements, then complicated movements; (iv) During practice, same actions done for non‐affected limbs, to aid in recovery of function of paralysed limbs. During the process of active ranging exercise, coordination practice must be emphasised, to gradually improve level of coordination through a long period of training. From individual joints, and uni‐directional simple exercises to complex coordinated movements, movement complexity and precision [were] gradually increased; starting from exercises symmetrical to both sides of body; during gait training, initial requirement was for gait to be stable, accurate and natural, and afterwards practised walking forwards in a straight line and crossing obstacles etc. Repeated practice of a single movement, in order to develop the biological foundation of a habit, and form a new neural pathway" The individual components delivered are listed in Table 28. Based on the individual components, this intervention is categorised as comprising functional task training, musculoskeletal intervention (active), and musculoskeletal intervention (passive). Length of intervention period: 4 weeks Number of sessions and length of individual sessions: not stated Intervention provider: not stated (2) Control group (n = 53) Used Traditional Chinese Tui Na The individual components delivered are listed in Table 28. Based on the individual components, this intervention is categorised as comprising massage. Length of intervention period: 4 weeks Number of sessions and length of individual sessions: not stated Intervention provider: Traditional Chinese Tui Na practitioner This study is classified as intervention (functional task training, musculoskeletal (active), musculoskeletal (passive)) versus attention control (massage) (Table 24)
Outcomes	Measures of independence in ADL: Barthel Index Measure of motor function: Fugl‐Meyer Assessment (FMA) Measures of tone/spasticity: "Modified Ashworth Spasticity Rating Scale" Time points when outcomes were assessed: before intervention and after 4 weeks of intervention
Notes	Both groups were given the same conventional medicine to reduce intracranial pressure, nourish nerves, prevent and cure symptoms, maintain electrolyte balance, and provide symptomatic and supportive treatment; on admission, while lying on non‐affected side and supine, all used orthopaedic devices on affected side. Four weeks equals one treatment cycle. Original study translated from Chinese to English This study was previously included, but has been excluded from the 2022 update following identification of numerous studies, conducted in China, investigating the efficacy of "early rehabilitation" or "early nursing". This study has therefore been moved to "awaiting assessment" and will be re‐considered at the same time as the other "early nursing" studies.

**Table:** Li 2003

Methods	Study design: RCT Method of randomisation: not stated ("Total 174 patients were selected in this study, and these patients were randomly divided")
Participants	Number of participants: n = 174 Inclusion criteria: not stated ("359 patients with paralysis after cerebral hemorrhage were admitted from March 2001 to May 2002")
Interventions	(1) Rehabilitation group (n = 87) "received regular nursing and rehabilitation nursing" "In the early phase, passive activity for affected side and active activity for health side [were] suggested, such as combing the hair and hitting with health hands, raising the legs, clipping legs, contacting hands and feet" "During acute phase, turning over every 1‐2 hours was practiced under supine position. Lateral recumbent position was avoided to prevent compression of limbs. During lateral position, pillow was used to support the affected side and the health upper limbs and raise elbow. During the lateral position with the health side, elbow joint was stretched with palm toward health side, and the lower health limbs were extended backwardly. Exercise of limbs included the gentle pressing, massage, malaxtion from the distal end to proximal end twice a day, 20 min each time. Massage with safflower of regions surrounding should joint and from forearms to fingers was performed. Doctors should make patients believe their limbs were capable of moving for patients who were clear. The active movement dominated by the big nerves was suggested when patients showed signs of limbs activity, including raising hands, shoulder and leg and antielbow extension, hitting palms along the diagonal direction" "During rehabilitation phase, patients were asked to sit by the bed with the health hands holding the bed, and legs dropping, and nurses standing by the affected side to prevent inclination toward the affected side. Once patients were capable of sitting stably by self, sitting exercise was initiated with body against bed, health hands holding bed, and nurses sitting by the side. Sitting exercises lasted from a few seconds to minutes, during which, patients were asked to swing affected limbs, 5 min each time and times and duration can be increased gradually. Walking exercise was initiated if patients were capable of sitting for 10‐15 min without assistance. First patients were asked to do stepping on under the help of crutches. During exercise, center of gravity was gradually shifted to the affected side. Patients were asked to support the center of gravity under the nurses assistance, then began the walking with health limbs till patients could take care of themselves" "Psychological rehabilitation, support, encourage, assiliation were given during the different psychological stage to make them exercise actively under good environment" Length of intervention period: unclear: "The average hospitalisation was (20 ± 9) days and (31 ± 11) days for rehabilitation group and control group respectively. Therapeutic effect of rehabilitation group was significantly superior to that of control group 15 days after treatment" Number of sessions and length of individual sessions: dependent on patient condition (see above) Intervention provider: nursing staff (2) No rehabilitation group (n = 87) "received the general nursing" Length of intervention period: no intervention Number of sessions and length of individual sessions: no intervention Intervention provider: no intervention
Outcomes	Other outcome measures: 'Score of Neural Defection,' average length of hospitalisation Time points when outcomes were assessed: before and after treatment ... "15 days after treatment"
Notes	Abstract only. No data suitable for analysis. This paper was written in English, and the extracts above are direct quotes. This study was previously included, but has been excluded from the 2022 update following identification of numerous studies, conducted in China, investigating the efficacy of "early rehabilitation" or "early nursing". This study has therefore been moved to "awaiting assessment" and will be re‐considered at the same time as the other "early nursing" studies.

**Table:** Liu 2003

Methods	Study design: RCT Method of randomisation: not stated
Participants	Number of participants: n = 120 Inclusion criteria: "There were 120 hemiplegic patients with cerebral apoplexy from July 2000 to February 2001 (not counting those with serious heart, lung, kidney, and stomach complication and disturbance of consciousness). We made a diagnosis according to WHO standards after using CT or MRI head diagnosing without exception"
Interventions	(1) Rehabilitation group (n = 60) "All the 120 patients were treated according to endoneurological routine. On this basis we made recovery training for the rehabilitation group using modern technology. In accordance with patients condition, we took appropriate recovery measures (PT, OT), such as favourable limb position in bed, particular passive movement, healthy limb active movement and sick limb movement with the help of the healthy, sitting position balancing training, dressing and eating, speaking and ADL training" "The rehabilitation group started to accept the treatment in 3‐5 days after attack when the patients had been conscious, vital signs had been smooth, nervous signs had not advanced within 48 hours" Length of intervention period: 15 days Number of sessions and length of individual sessions: "four times a day, 30 minutes at every turn" Intervention provider: not stated (2) No rehabilitation group (n = 60) No intervention Length of intervention period: no intervention Number of sessions and length of individual sessions: no intervention Intervention provider: no intervention
Outcomes	Measures of Independence in ADL: Barthel Index Measures of motor function: Fugl‐Meyer Assessment State time points when outcomes were assessed: "we evaluated from the very beginning of treatment and on the fifteenth day of treatment"
Notes	Abstract only This study was previously included, but has been excluded from the 2022 update following identification of numerous studies, conducted in China, investigating the efficacy of "early rehabilitation" or "early nursing". This study has therefore been moved to "awaiting assessment" and will be re‐considered at the same time as the other "early nursing" studies.

**Table:** Pan 2020

Methods	"Altogether 196 patients with acute stroke admitted to Zhejiang University of Traditional Chinese Medicine Affiliated Wenzhou Hospital were selected and divided into two groups according to different nursing methods."
Participants	"The intervention group (103) included 54 males and 49 females with an average age of 64.15 ± 3.15 years. The control group (93) included 46 males and 47 females with an average age of 65.01 ± 4.25 years. "
Interventions	(1) Early nursing group Early nursing included psychological care, limb rehabilitation training, cognitive function guidance, prevention of adverse events, life ability guidance (2) Control group "Control group with routine nursing: Simple safety and health education were carried out for patients, and conventional treatment such as intracranial pressure reduction, blood sugar control and prevention of complications were given"
Outcomes	Berg balance scale Fugl‐Meyer Assessment NIHSS Barthel Index Pain Cognitive function Adverse events
Notes	Study initially included, but then re‐assessed to be a "Chinese nursing" intervention, similar to the other studies of Chinese nursing which are awaiting assessment. This study has therefore been moved to "awaiting assessment", and will be considered at the same time as the other Chinese nursing studies.

**Table:** Zhang 2013

Methods	RCT
Participants	Participants with ischaemic stroke (n = 69)
Interventions	(1) Integrated Rehabilitation Techniques of Traditional Chinese Medicine (IRT‐TCM) "An expert committee composed of clinical experts and researchers working on stroke rehabilitation, Chinese internal medicine, massage, and acupuncture created the IRT‐TCM based on published literature and textbooks. The IRT‐TCM started as soon as the vital signs of the patients became stable. Patients received IRT‐TCM once a day until their discharge. The IRT‐TCM consisted of 30 mins of acupuncture and another30 mins of massage. Eight acupoints on the affected side were selected for needling and massaging: Jianyu(LI15), Quchi (LI11), Hegu (LI4), Xuehai (SP10), Yanglingquan (GB34), Huantiao(GB30), Qiuxu (GB40), and Taichong (LR3). All acupoints were located according to theWHO standard acupuncture point locations in the Western Pacific Region. Disposable stainless steel acupuncture needles (0:2540,0:2575, Ande Co., Guizhou, China) were inserted to a depth of approximately 1.5–2.5 cm (5.0–6.0 cm for Huantiao). Each acupuncture needle was twisted until the patient felt a“de‐qi” sensation and retained for30 mins. Acupuncture treatment was performed by an independent practitioner with seven years of clinical experience. Massage treatment included point‐pressing manipulations on the eight acupoints mentioned above and functional training of affected limbs. The point‐pressing manipulations including pushing, pressing, and rolling, were operated with patients lying in bed and the functional training differed according to the patients’ condition. Patients with limb atony received passive joint movements. Those with limb spasticity received passive joint movements, flexor inhibition, and extensor facilitation movements. Those with limb syn‐kinesis were inhibited of synkinesis movements and induced of disjunctive movements. The massage treatment lasted for 30 mins and was performed by an independent practitioner with five years of clinical experience. (2) Conventional rehabilitation "patients in the control group were given the conventional rehabilitation programs, which consisted of basic treatments and conventional rehabilitation techniques. The basic treatments were established according to the Chinese Cerebrovascular Disease Prevention and Treatment Guidelines which included: keeping the respiratory tract unobstructed; preventing and treating aspiration pneumonia; monitoring and treating arrhythmia and ischemic heart disease; normalizing the blood pressure; and appropriate using of anticoagulants and antiplatelet aggregation agents. The conventional rehabilitation techniques following the Bobath Concept, which is also known as neuro‐developmental treatment (NDT) in America, were applied to the patients in the control group as soon as their vital signs became stable (Pereiraet al.,2012). The techniques consist of therapeutic handling, facilitation, inhibition, and key points of control. Before being discharged from the hospital, patients had been clearly informed of a reasonable long‐term rehabilitation plan and health education (Strauset al.,2002). All Bobath rehabilitation techniques were performed by an independent practitioner with seven years of clinical experience. Rehabilitation techniques lasted one hour every day until the discharge"
Outcomes	Fugl‐Meyer Assessment, Barthel Index, NIHSS, modified Rankin
Notes	Study initially included, but then re‐assessed to be a "Chinese traditional medicine" intervention, similar to the other studies which compare 'Western' and Chinese approaches and which are awaiting assessment. This study has therefore been moved to "awaiting assessment", and will be considered at the same time as the other related studies.

ADL: activities of daily living NIHSS: National Institutes of Health Stroke Scale RCT: randomised controlled trial

### Characteristics of ongoing studies [ordered by study ID]

**Table:** ACTRN12612001228875 2012

Study name	The effects of a multimodality balance training compared with conventional balance training on physical and psychological function in stroke survivors
Methods	**Design:** Parallel group RCT **Country:** Malaysia **Trial registration details:** Australian New Zealand Clinical Trials Registry (ANZCTR) ACTRN12612001228875
Participants	**Study population (planned):** 48 **Inclusion criteria:** Aged 40‐60 years with a diagnosed ischemic or haemorrhagic brain injury (using MRI or CT) > 6 month after the onset of stroke. BBS scoring < 41 at baseline Moderate‐high level of depression (i.e. BDI > 30 at baseline) able to walk 10m without the use of assistive device. **Exclusion criteria:** other neurological disorder such as PD or cerebellar stroke. severe heart disease unable to understand instructions receiving rehabilitation from another institution or personal healthcare provider
Interventions	1. Multisensorial training regime plus specific lower limb training regime in multimodality balance training 2. Conventional balance training
Outcomes	**Primary outcome(s):** BBS **Secondary outcome(s):** FMA, lower limb muscle strength as measured by 1RM, 30‐Seconds Chair‐Rise test, mobility and dynamic balance measured by DGI, TUG, BDI, BI, SS‐QOL **Data collection:** baseline, week 12 and week 24 post‐intervention
Starting date	01/01/2013
Contact information	Zoolfaiz Salleh, Physiotherapy Department, Faculty of Health Sciences, Universiti Teknologi MARA (UiTM), Puncak Alam Campus 42300 Kuala Selangor, Selangor, Malaysia Email: mohdzoolfaiz@gmail.com
Notes

**Table:** ACTRN12622000169741 2021

Study name	Effect of a targeted exercise intervention on independent mobility and motor functioning in post‐stroke older people
Methods	**Design:** Parallel group RCT **Country:** Spain **Trial registration details:** Australian New Zealand Clinical Trials Registry (ANZCTR) ACTRN12622000169741
Participants	**Study population (planned):** 80 **Inclusion criteria:** Aged 60‐80 years Community living independent living individuals before the stroke, who are acutely hospitalized due to being diagnosed after having suffered their first stroke at the Royo Villanova (HRV) or the Provincial Nuestra Señora de Gracia de Zaragoza (HNSG) and the Cruces Hospital in Bizkaia ( HC) Cognitive status preserved Able to follow the instructions, both during testing and intervention. Ability to maintain a sitting position on a chair without assisstance with a back. **Exclusion criteria:** Severe heart disease or severe bronchopathies Other types of medical conditions that prevent the treatment from being implemented Cognitive impairment‐ Inability to understand the instructions during the test or intervention. Unstable orthopedic injuries such as unconsolidated fractures or with unstable osteosynthesis systems. Inability to maintain a sitting position on a chair without assisstance with a back
Interventions	1. Target intervention programme combines a repertory of standardized exercises that have been previously described in scientific literature to improve balance. 2. Conventional Physiotherapy
Outcomes	**Primary outcome(s):** Time taken (days) to achieve independent gait (≥ 1 m/s) Gait speed Both tested using 15m walk test without assistive devices. **Secondary outcome(s):** Physiotherapist‐related feasibility questionnnaires: adequacy and acceptation of (i) testing methods, (ii) intervention method, SPPB, BESTest, MiniBESTest, Patient‐related feasibility questionnaires: adequacy and acceptation of (i) testing methods, (ii) intervention method, IPAQ, Falls Efficacy Scale, number of falls. **Data collection:** Baseline, immediately after achievement of independent gait, and/or changing patients status (acute, and/or post acute), and/or 90 days post stroke (intervention completion 3 months).
Starting date	Registered 2/02/2022
Contact information	Dr Mirian Aranzazu Garrués Irisarri. Department of Physiology, Faculty of Medicine and Nursery, University of the Basque Country, Barrio Sarriena, s/n, Leioa | 48940, Spain. Email: mirianaranzazu.garrues@ehu.eus
Notes

**Table:** Armed4Stroke 2022

Study name	Armed4Stroke
Methods	**Design:** Parallel group RCT **Country:** Netherlands **Trial registration details:** Netherlands Trial Register NTR7664
Participants	**Study population (planned):** 72 or 74 dyads **Inclusion criteria:** For both stroke survivor and caregiver: >18 years written informed consent able to understand the Dutch language and understand instructions motivated for CM For stroke survivor only: < 3 months poststroke living independently prior to stroke; discharged or planned to be discharged home able to follow instructions MoCA score > 21 points able and willing to appoint an informal caregiver. For the caregiver: medically stable and able to support the patient as judged by a trained physiotherapist. **Exclusion criteria:** For both stroke survivor and caregiver: serious comorbidity that interferes with participation (e.g. existing condition that may restrict mobility) not enrolled in another clinical trial
Interventions	1. Armed4Stroke program plus usual care: 8 weeks of complementary exercises executed with a caregiver 2. Usual inpatient or outpatient care according to the Royal Dutch Guidelines of Physical Therapy
Outcomes	**Primary outcome(s):** SIS, **Secondary outcome(s):** For both stroke survivor and caregiver: GSES, PCS, TPS, HADS, CAT, FSS, FAD‐GF For stroke survivor: LOS, SSEQ, RMI measures self‐reported mobility poststroke, functional walking ability, 6MWT, 5MWT, MI lower extremity, BBS, MOX‐2, NEADL, CAQ, EuroQoL 5D, mRS For caregiver: CSI, CarerQoL **Data collection:** baseline, week 9 (immediately post‐intervention), 6 months post‐intervention
Starting date	02/09/2019
Contact information	Gert Kwakkel, Department of Rehabilitation Medicine, Amsterdam University Medical Centre, location VU University Medical Centre, Amsterdam Movement Sciences, PO Box 7057, 1007, MB, Amsterdam, The Netherlands Email: G.Kwakkel@amsterdamumc.nl
Notes

**Table:** ChiCTR2000032705 2020

Study name	The effect of caregiver‐mediated exercises in‐hospital rehabilitation on limb function of patients with hemiplegia after stroke
Methods	**Design:** Parallel group RCT **Country:** China **Trial registration details:** Chinese Clinical Trial Registry ChiCTR2000032705
Participants	**Study population (planned): 5**0 **Inclusion criteria:** The patients diagnosed as ischemic stroke by CT or MRI are in line with the diagnostic criteria set by the 4th Chinese society of cerebrovascular diseases; Patients over 18 years of age, the first onset, within 2 months, Brunnstrom stage I ~ II; Hemiplegic patients with unilateral limb dysfunction; Patients who are conscious and whose MMSE score is more than 20. Caregiver inclusion criteria: Over the age of 18, the patient's immediate relatives can accompany the patient during the whole training process, with stable physical condition, and can assist the patient to complete the training; Patients who voluntarily participated in the study and filled in informed consent. **Exclusion criteria:** Objects with severe cognitive impairment and language disorder that can not cooperate;Subjects with serious complications and unstable vital signs. Exclusion criteria for caregivers: Objects with severe cognitive impairment and language disorder that can not cooperate;Objects with serious organic diseases and unstable vital signs;The object of economic compensation.
Interventions	1. Routine rehabilitation treatment + caregiver‐mediated rehabilitation training 2. Routine rehabilitation treatment
Outcomes	**Primary outcome(s):** FMA **Secondary outcome(s):** BI, BBS, SAS (Anxiety), Zung Self‐Rating Anxiety/Depression Scale (SAS/SDS), MMSE, SS‐QOL **Data collection:** Not stated
Starting date	10/05/2020
Contact information	Study leader: Guo Lirong 965 Xinjiang Street, Chaoyang District, Changchun, Jilin, China. Email: guolr@jlu.edu.cn
Notes

**Table:** CTRI/2018/10/016163 2018

Study name	Impact of task specific training for paretic lower extremity on lower limb motor recovery and functions in early subacute phase following stroke
Methods	**Design:** Parallel group RCT **Country:** India **Trial registration details:** Clinical Trials Registry ‐ India CTRI/2018/10/016163
Participants	**Study population (planned):** 40 **Inclusion criteria:** aged 35‐70 years with a clinical diagnosis of first stroke (ischaemic or haemorrhagic) confirmed through CT or MRI stroke onset between 7 days to 3 months MMSE > 24 no cognitive dysfunction mild‐moderate severity on NIHSS **Exclusion criteria:** previous history of stroke other neurological conditions (e.g. PD) or musculoskeletal conditions (e.g. joint replacement, amputation that limits participation history of cardiac disease or cardiac surgery visual impairment aphasia vestibular system deficit brainstem stroke or bilateral Infarction history of thrombolysis for present episode
Interventions	1. Task specific training 2. Conventional physiotherapy
Outcomes	**Primary outcome(s):** FMA (lower extremity) **Secondary outcome(s):** 5xSTS, Motor Assessment Scale (lower extremity), FAC **Data collection:** day 7, 15 and 30 post‐enrolment
Starting date	01/01/2019
Contact information	Meenakshi Jharbade, Department of Physiotherapy Sri Ramachandra Medical College and Research Institute. Chennai, TAMIL NADU 600116, India Email: physiomeenakshi20@gmail.com
Notes	Status Reported as Recruitment complete on Trial register. No publications found.

**Table:** CTRI/2022/06/043037 2022

Study name	Effectiveness of dynamic neuromuscular stabilization, neurodevelopmental techniques and proprioceptive neuromuscular facilitation on trunk and gait parameters in patients with subacute stroke, a three arm parallel randomized clinical trial
Methods	**Design:** Parallel group RCT **Country:** India **Trial registration details:** Clinical Trials Registry ‐ India CTRI/2022/06/043037
Participants	**Study population (planned):** 60 **Inclusion criteria:** aged 40 to 60 years, either gender persons who can to understand, communicate and follow the instructions those persons with Sub acute stroke. **Exclusion criteria:** patients who has severe cardiac illness patients those are having fracture patients with recurrent stroke
Interventions	1. Dynamic Neuromuscular stabilization 2. Neurodevelopmental technique 3. Proprioceptive Neuromuscular facilitation
Outcomes	**Primary outcome(s):** TIS, DGI, Gait Parameters **Secondary outcome(s):** MMSE, VCG, MAS **Data collection:** baseline, 4 weeks post‐intervention
Starting date	13/06/2022
Contact information	Principal Investigator: Swadha P Udhoji. MPT Student. Department of Neurophysiotherapy, Ravi Nair Physiotherapy College, Datta Meghe Institute of Medical Sciences, Sawangi, Wardha MAHARASHTRA, 442001, India. Email: swadhamushriff@gmail.com
Notes

**Table:** DRKS00020825 2020

Study name	GLAAS II ‐ The effect of coordinative training on balance and walking ability in ataxic people with acute cerebral stroke
Methods	**Design:** Parallel group RCT **Country:** Austria **Trial registration details:** German Clinical Trials Register DRKS00020825
Participants	**Study population (planned):** 94 **Inclusion criteria:** acute cerebral stroke aged ≥18 years ataxia of gait and/or stance and/or sitting and/or positive heel‐shin slide assessed using SARA BBS ≤ 47 points able to provide written informed consent **Exclusion criteria:** cerebral stroke in case history with persistent motor deficits (mRS ≥ 3) comorbidity, which would restrict participation mRS ≥ 5 any physical or mental condition which would not allow safe participation in the study or would complicate assessment of outcomes (e.g. dementia, cardiac insufficiency, severe Aphasia, people having a procurator appointed pregnancy people fulfilling military service
Interventions	1. Physiotherapy using coordinative exercises (similar to the Doris‐Broetz‐Concept). 2. Standard physiotherapy
Outcomes	**Primary outcome(s):** BBS **Secondary outcome(s):** TUG, FAC, SARA, changes relating to balance ability measured using FRT, FGA, number of falls, posturography (Tymo), activities of daily living measured using the Scores of Independence for Neurologic and Geriatric Rehabilitation, EQ 5D 3L **Data collection:** baseline and immediately post‐intervention (after 4 weeks)
Starting date	20/07/2020
Contact information	Patricia Meier, Medizinische Universität Innsbruck, Universitätsklinik für Neurologie, Anichstraße 35, 6020 Innsbruck, Austria Email: patricia.meier@vascage.at
Notes

**Table:** Ghrouz 2022

Study name	Effect of motor relearning on balance, mobility and performance of activities of daily living among post‐stroke patients
Methods	**Design:** Parallel group RCT **Country:** Spain **Trial registration details:** ClinicalTrials.gov NCT05076383
Participants	**Study population (planned):** 66 **Inclusion criteria:** first‐ever sub‐acute (1–6 months) stroke able to provide informed onsent; hemiparesis with MRC muscle scale between 2 ‐ 4; able to stand independently for at least 1 min able to ambulate 25 feet/10 m (with or without an assistive device) **Exclusion criteria:** stroke survivors with MoCA score ≤20 and/or communication impairments that may restrict participation (e.g. deafness and aphasia) currently receiving other rehabilitation which may impact on the study any contraindications to start rehabilitation (i.e. severe uncontrolled hypertension, uncontrolled diabetes or unstable angina) history of neurological deficits other than stroke
Interventions	1. Motor relearning program (MRP) 2. Conventional physical therapy program
Outcomes	**Outcome(s):** BBS, TUG, 10mWT, BI and instrumental analysis of balance and gait **Data collection:** baseline, immediately post‐intervention (week 9‐10), and 3 months follow‐up
Starting date	01/10/2021
Contact information	Corresponding author: Amer Ghrouz, Department of Medicine, Universitat Aut`onoma de Barcelona, Passeig de la Vall d’Hebron, 119‐129, Barcelona 08035, Spain. Email: aghrouz@najah.edu
Notes

**Table:** Gracies 2019

Study name	NEURORESTORE
Methods	**Design:** Parallel group RCT **Country:** France **Trial registration details:** ClinicalTrials.gov NCT02202954
Participants	**Number randomised (planned):** 124 **Inclusion criteria**: hemiparesis due to stroke, for over a year before enrolment age > 18 years; ability to ambulate over 10m independently, barefoot and without technical aid maximal 10‐m ambulation speed between 0.1 and1.3 m/sec modified Frenchay Scale score > 2/10 and < 8/10 written consent to participate in the protocol, signed by the patient **Exclusion criteria:** recurrent stroke significant orthopedic disorder in lower limb cognitive, phasic or behavioural dysfunction affecting patient participation non‐affiliation to medical insurance system
Interventions	1. Guided Self‐rehabilitation Contract 2. Conventional rehabilitation in the community
Outcomes	**Primary outcome(s):** fast 10‐m barefoot ambulation speed (no assistive device), upper limb function using the modified Frenchay scale **Secondary outcome(s):** Speed, step length and cadence over 10 meters at comfortable speed, barefoot and with shoes, with no assistive device, speed, step length, cadence and physiological cost index over 2 minutes at maximal speed, with shoes, Disability Assessment Scale, BI, EQ‐5D, GDS, questionnaire evaluating monthly frequency of physical therapy sessions and amount of home aid during the whole study period, estimation of the total cost of care, including medical costs, social expenses, amount of social benefits, from the point of view of the medical insurance and of the state, to include all payors. **Data collection:** baseline and one assessment visit every 6 months for 2 years
Starting date	March 2014
Contact information	Maud Pradines, 1EA 7377 BIOTN, Laboratoire Analyse et Restauration du Mouvement, Université Paris Est Créteil (UPEC), F‐94010 Créteil, France, and 2AP‐HP, Service de Rééducation Neurolocomotrice, Unité de Neurorééducation, Hôpitaux Universitaires Henri Mondor, F‐94010 Créteil, France. Email: maudprad@gmail.com
Notes

**Table:** IRCT20140304016830N9 2018

Study name	A randomized controlled trial to comparative study of the effect of single and dual cognitive task‐oriented balance exercises on postural control and functional balance in subjects with chronic stroke
Methods	**Design:** Parallel group RCT **Country:** Iran, Islamic Rep **Trial registration details:** Iranian Registry of Clinical Trials IRCT20140304016830N9
Participants	**Study population:** 54 **Inclusion criteria:** aged 35‐65 years first experience of stroke; 6‐24 months since stroke able to walk at least 10m without assistive devices able to perform the most difficult condition of laboratory test (quiet standing on foam surface with closed eyes while performing cognitive task) cognitive function level MMSE ≥ 23 **Exclusion criteria:** unilateral visuospatial neglect (i.e., obtaining score < 44 at star cancellation test) co‐morbid/other neurological diseases orthopedic disorders (such as low back pain, arthritis and flat foot) diabetes according to the report of patient, or the patient's family or physician addiction according to the report of patient, or the patient's family or physician
Interventions	1. Cognitive dual task‐oriented balance exercise 2. Single task‐oriented balance exercise (control)
Outcomes	**Primary outcome(s):** BBS, Tinetti Balance Test, Postural sway (force plate) **Secondary outcome(s):** TUG (single and dual), DGI **Data collection:** Baseline, post‐intervention, 8 weeks after intervention
Starting date	Estimated 04/02/2018
Contact information	Assistant Professor Ghorban Taghizadeh Ph.D., Iran University of Medical Sciences, Shahid Hemmat, Highway, Tehran, 1449614535. Email: taghizadeh.gh@iums.ac.ir
Notes	Funded by Iran University of Medical Sciences

**Table:** IRCT20201204049596N1 2020

Study name	
Methods	**Design:** Parallel group RCT **Country:** Iran, Islamic Rep. **Trial registration details:** Iranian Registry of Clinical Trials IRCT20201204049596N1
Participants	**Study population:** 30 **Inclusion criteria:** ischaemic or haemorrhagic stroke diagnosed using CT or MRI hemiplegia at least one month since the stroke able to execute a 3‐step command impaired balance and gait able to walk with/without support FAC II ‐ IV high‐speed Internet access and telephone knowledgeable family member available for all exercise sessions and conversation **Exclusion criteria:** bilateral paralysis Injury to the cerebellum or brain stem proprioception injury unilateral hemianopsis/forgetfulness or any other visual impairment vestibular dysfunction neurological diseases other than stroke e.g. neuropathy severe postural instability orthopedic problems significant cognitive problems perceptual aphasia other conditions that prevent exercise, e.g. cardiovascular disease
Interventions	1. Remote rehabilitation 2. Rehabilitation treatment without the therapist supervision
Outcomes	**Primary outcome(s):** BBS, TUG, FES **Secondary outcome(s):** not stated **Data collection:** baseline, post‐intervention, 1 month and 3 months post‐intervention
Starting date	Estimated 04/01/2021
Contact information	Assistant Professor Simin Sajjadi, Department of Physical Medicine and Rehabilitation, Firoozgar Hospital, Vali Asr Square, Tehran, 1449614555. Email: attari.a@iums.ac.ir
Notes	Funded by: Iran University of Medical Sciences Status Reported as Complete on Trial register. No publications found.

**Table:** Johnson 2019

Study name	
Methods	**Design:** cluster RCT with nested qualitative evaluation **Country:** United Kingdom **Trial registration details:** ClinicalTrials.gov NCT03792126
Participants	**Study population:** 6 clusters (stroke unit); Criteria for stroke unit eligibility are a dedicated unit that: routinely admits patients with acute stroke dedicated therapy (occupational therapy and physiotherapy) service for at least 5 days per week **Inclusion criteria:** clinical diagnosis of stroke, presenting with lower limb paresis has rehabilitation goals relating to lower limb mobility orfunction within 14 days of stroke onset medically stable able to tolerate daily therapy for a minimum of 30 min persession, sit for more than 5 seconds without support, and understand and follow single stage commands **Exclusion criteria:** previous stroke with residual impairments other neurological diagnosis (e.g., Parkinson disease, Multiple Sclerosis) clinically relevant premorbid disability levels (required physical assistance of 1‐2 people to transfer from bed to chair and/or unable to mobilize without physical assistanceof 1‐2 people).
Interventions	1. Implicit learning approach (ILA) 2. Standard care (control)
Outcomes	**Primary outcome(s):** mRMI **Secondary outcome(s):** Swedish Postural Adjustment in Stroke Scale (SwePASS), FMA ‐ Motor Leg Sub Section, mRS, EQ‐5D, Movement Specific Reinvestment Scale **Data collection:** 3 months
Starting date	01/03/2019
Contact information	Louise Johnson, PhD. Stroke Unit, Royal Bournemouth and Christchurch Hospitals NHS Foundation Trust, Castle Lane East, Bournemouth, BH7 7DW, United Kingdom. Email: Louise.Johnson@uhd.nhs.uk
Notes	LJ is funded by a National Institute of Health Research Clinical Lectureship for this research project. This project is funded by the NIHR (ICA‐CL‐2017‐03‐011)

**Table:** Kei 2020

Study name	
Methods	**Design:** Parallel group RCT **Country:** Malaysia **Trial registration details:** Australian New Zealand Clinical Trials Registry (ANZCTR) ACTRN12619001182189
Participants	**Study population (planned):** 72 **Inclusion criteria:** clinically diagnosed with stroke completed hospital‐based rehabilitation and scheduled for discharge age 18 to 80 years old availability of carer to monitor home‐based interventions FAC score 3 or more **Exclusion criteria:** MoCA < 20 modified Rankin Scale 4 or more attended by a home physiotherapist after discharged from hospital HADS score > 11 presence of other medical conditions such as severe neurological and musculoskeletal disorders or comorbidities
Interventions	1. Home‐based exercise program (HBT) 2. Usual practice (UP)
Outcomes	**Primary outcome(s):** Falls risk and mobility status using TUG, 10mWT **Secondary outcome(s):** SSEQ, HADS, modified CSI **Data collection:** baseline, 12 weeks (post‐intervention)
Starting date	01/10/2019
Contact information	Nor Azlin Mohd Nordin PhD, Physiotherapy Program, Center for Rehabilitation and Special Needs Studies, Faculty of Health Sciences, Universiti Kebangsaan Malaysia, Kuala Lumpur 50586, Malaysia. Email: norazlin8@ukm.edu.my.
Notes	Partly funded by University Kebangsaan Malaysia (code: NN‐2019–149) as this is a post‐graduate (master) project and all equipments to be used in this study are properties of the university

**Table:** Menezes‐Oliveira 2021

Study name	
Methods	**Design:** Parallel group RCT **Country:** Brazil **Trial registration details:** The Brazilian Registry of Clinical Trials (ReBEC) RBR‐467cv6
Participants	**Study population:** 42 **Inclusion criteria:** medical diagnoses of stroke (ischaemic or haemorrhagic) stroke at least 6 months before study participation clinically stable hemiparesis caused by stroke; gait deficit caused by stroke able to begin hip and knee flexion on the affected side able tomove from sitting to standing independently even with aid or support of upper limbs able to transfer while sitting independently able to sustain body weight on the affected side even with the support of another person and/or aid able to walk at least 10m with/without aid, or with/without support of another person, barefoot have only one or two main caregivers or relatives who live with the patient or spend substantial parts of the day with them available to go to the rehabilitation center for 17 consecutive day sand stay there for 3 h per day (15 days of treatment and the first day for initial tests and the last day for the final tests) not subjected to orthopedic surgery for at least 6 months not been subjected to chemical block for at least 3 months does not present an independent community gait (ability of walking alone in the community with or without gait advice at speed of ≤0.8m/s while walking) **Exclusion criteria:** do not accept the protocol for which they were randomly allocated speech deficits that render them unable to understandand/or answer properly to evaluation scales and exercises selected suffered any clinical event between the screening and the beginning of the protocol two consecutive absences without medical justification or clinical occurrence that makes continuing with the exercises impossible
Interventions	1. Lower extremity constraint‐induced movement therapy (LE‐CIT) 2. Intensive conventional therapy
Outcomes	**Primary outcome(s):** MiniBESTest, 6MWT **Secondary outcome(s):** TUG, 10mWT, Gait analysis: Gait Deviation Index (GDI); Gait Variable Scores (GVS),;Gait Profile Score (GPS), Lower Extremity Motor Activity Log (LE‐MAL) **Data collection:** baseline, end of intervention, 6 months after end of intervention
Starting date	30/04/2019
Contact information	Elaine Menezes‐Oliveira. Neurology/Neuroscience program, Federal University of São Paulo – UNIFESP, Botucatu street, 862 ‐ 5° floor Edifico Ciências Biomédicas, São Paulo, Brazil, and Adults Physiotherapy Department, Associação de Assistência à Criança Deficiente, Professor Ascendino Reis avenue, 724 – Ibirapuera, São Paulo, Brazil. Email: elaine_m_oliveira@yahoo.com.br; emdoliveira@aacd.org.br
Notes	No specific funding for the conduction of the study; we do not have financial support

**Table:** Miller 2014

Study name	FAST
Methods	**Design:** Parallel group RCT **Country:** Canada **Trial registration details:** ClinicalTrials.gov NCT01573585
Participants	**Study population:** 60 **Inclusion criteria:** First stroke (confirmed by admission CT scan) within the previous 6‐months that resulted in unilateral hemiparesis and required inpatient rehabilitation. Sufficient motor control in the paretic lower limb to perform the stepping activities in the FAST program. program: Chedoke McMaster Stroke Assessment leg and foot scored as stages 3–6 (stage 7 consider ‘normal’). Standing balance ability necessary to participate safely in the FAST program: Berg Balance Score (BBS) ≥ 30/56 [26]. Cognitive capacity to provide informed consent: Mini‐Mental Status Examination score ≥ 24/30. **Exclusion criteria:** Bilateral stroke or a history of previous stroke(s) for which inpatient rehabilitation was received. Severe co‐morbidities likely to dominate the pattern of care (e.g. metastatic disease, severe congestive heart failure, etc.), co‐existing peripheral neuropathies or vestibular disorders likely to independently have a negative impact on balance, and severe musculoskeletal problems or pain; as these conditions would be likely to impede participation in the study interventions. Global aphasia, receptive aphasia or language barriers who do not have someone to assist them in translating information, as these individuals would have difficulty providing informed consent or understanding exercise instructions.
Interventions	1. Fast muscle Activation and Stepping Training (FAST) program 2. Conventional physiotherapy directed at improving balance and mobility that includes no targeted fast movement training (control).
Outcomes	**Primary outcome(s):** Community Balance and Mobility Scale **Secondary outcome(s):** 10 Metre Walk Test (fast walking speed) Activities‐specific Balance Confidence questionnaire Helpfulness of treatment received in improving balance Gait – kinematic, kinetic and EMG parameters Biodex – maximum voluntary contraction EMG Chedoke‐McMaster Stroke Assessment leg & foot score Berg Balance Scale Self‐selected walking speed Physiological Balance Test – Postural stress test (external perturbations) Physiological Balance Test – Arm raise task (internal perturbations) Physiological Balance Test – Stepping Reactions (internal perturbations) **Data collection:** baseline, within one week of the final intervention session, one month after final intervention session
Starting date	November 2012
Contact information	Jayne Garland. The University of British Columbia, 212 Friedman Building, 2177 Wesbrook Mall, Vancouver, BC V6T 1Z3, Canada. Email: jayne.garland@ubc.ca
Notes

**Table:** NCT00908479 2009

Study name	Exercise programs for stroke: effect of an inpatient supplementary practice program on lower extremity function
Methods	**Design:** Parallel group RCT **Country:** Canada **Trial registration details:** ClinicalTrials.gov NCT00908479
Participants	**Study population:** 142 **Inclusion criteria:** admitted to a hospital unit for stroke treatment 19 years or older experiencing difficulty using legs able to understand and follow instructions **Exclusion criteria:** uncontrolled hypertension or unstable cardio‐vascular condition Injuries to muscles, bones, or joints of the leg unable to verbally speak or understand the investigators when asked questions
Interventions	1. Leg exercise group 2. Leg management (Control group)
Outcomes	**Primary outcome(s):** gait speed **Secondary outcome(s):** balance and physical activity outcomes (outcome measures not specified) **Data collection:** 4 weeks post‐baseline, then 6 and 12 months post‐stroke
Starting date	09/2009
Contact information	Principal Investigator: Dr. Janice Eng, Department of Physical Therapy, University of British Columbia Email: janice.eng@ubc.ca
Notes

**Table:** NCT03757026 2018

Study name	Balance training post stroke: intense harnessed multidirectional training as compared to reactive and conventional protocols
Methods	**Design:** Parallel group RCT **Country:** USA **Trial registration details:** ClinicalTrials.gov NCT03757026
Participants	**Study population (planned):** 66 **Inclusion criteria:** Aged 18 years or older Stroke more than 6 months ago Self‐identified balance issues Independent ambulation ‐ specifically: Walk at least one‐half block (150') with or without standard cane or similar device Stand independently for at least 30 seconds without physical assistance and without any device At least ten steps without physical assistance and without any cane or similar device Ability to answer two‐step questions **Exclusion criteria:** Allergic reaction to adhesive tapes Height above 74" Weight above 250 lbs Medical Condition (self‐reported) ‐ any musculoskeletal, neuromuscular, cardiopulmonary, or other conditions that would limit them from participation
Interventions	1. Slip training 2. Harnessed gaming 2. Conventional physical therapy
Outcomes	**Primary outcome(s):** Activities Specific Balance Confidence Scale (ABC), MiniBEST, Mobility life space scale (MLSS), anterior, posterior, and lateral limits of stability (LOS) scores, treadmill perturbation stability scores, number of falls (falls diary) **Secondary outcome(s):** FMA (lower extremity), Monofilament sensory testing, 5xSTS, SIS, BBS **Data collection:** Through study (approximately 3 months) and 6 months post‐intervention
Starting date	12/12/2018
Contact information	Principal Investigator Ann Reinthal, PT, PhD. Cleveland State University Email: a.karas@csuohio.edu Sub‐Investigator: Deborah Espy, PT, PhD. Email: d.espy@csuohio.edu
Notes

**Table:** NCT04673838 2020

Study name	The effect of lower limb sensory training on functional capacity in hemiparetic individuals
Methods	**Design:** Parallel group RCT **Country:** Turkey **Trial registration details:** ClinicalTrials.gov NCT04673838
Participants	**Study population (planned):** 42 **Inclusion criteria:** aged 20‐65 years and discharged from hospital diagnosed with hemiparesis at least 4 weeks ago First time and one‐sided hemiparesis Modified Rankin Score ≤3 Hodkinson Mental Test ≥ 6 agree to having treatment **Exclusion criteria:** vision and hearing problems other neurological, psychiatric and/or orthopedic problems other than hemiparesis medically unstable other diagnosed diseases that will affect lower extremity sensation open wounds, circulatory problems, skin lesions in the area to be treated will be excluded from the study
Interventions	1. Lower extremity sensory training + Bobath therapy 2. Bobath therapy (control group)
Outcomes	**Primary outcome(s):** MI, foot sole sense using Semmes Weinstein Monofilaments, Lower Extremity Position Test, kinesthesia sense, balance assessment, gait parameters **Secondary outcome(s):** Not stated **Data collection:** 4 weeks
Starting date	20/12/2020
Contact information	Gulsum Tikac, MSc Pt, Pamukkale University Email: gtikac@pau.edu.tr
Notes

**Table:** NCT04757467 2021

Study name	Comparison of frequency & duration of task practice during constraint‐induced movement therapy on lower limb in stroke patients
Methods	**Design:** Parallel group RCT **Country:** Pakistan **Trial registration details:** ClinicalTrials.gov NCT04757467
Participants	**Study population:** 96 **Inclusion criteria:** Aged 36‐60 years Stroke population (ACA) Lower limb impairment Ability to follow verbal and visual instructions No significant cognitive impairment (MMSE score ≥ 24) Moderate risk of fall (Tinetti gait and balance score 19‐23). FMA Lower extremity score of 21 or below out of 34 **Exclusion criteria:** Other neurological conditions Lower limb impairment due to any other reason (fracture, diabetic neuropathy etc.)
Interventions	1. Repetition‐CIMT (constraint‐induced movement therapy) 2. Hour‐CIMT 3. Standard physiotherapy neuro‐rehabilitation (control group)
Outcomes	**Primary outcome(s):** FMA Lower extremity, Tinetti Balance and Gait Test **Secondary outcome(s):** Not stated **Data collection:** Week 4
Starting date	01/08/2020
Contact information	Principal Investigator: Ayesha Afridi, PhD, Riphah International University
Notes

**Table:** NCT04816929 2021

Study name	Investigation of effects of bobath method and task‐oriented approach on architectural features and activation of trunk muscles and functional performance in stroke patients
Methods	**Design:** Parallel group RCT **Country:** Turkey **Trial registration details:** ClinicalTrials.gov NCT04816929
Participants	**Study population (planned): 30** **Inclusion criteria:** Unilateral stroke for more than 3 months Being over the age of 18 trunk impairment (not having full points in the Trunk Impairment Scale) able to walk without support or with walking aid. **Exclusion criteria:** recurrent stroke orthopedic or neurological disorders other than stroke that may affect motor performance cognitive problem (not having Mini Mental State Examination score ≥ 24)
Interventions	1. Bobath method group 2. Task‐oriented approach group
Outcomes	**Primary outcome(s):** Muscle Thickness (using ultrasonography), muscle activation (superficial electromyography), gait parameters (GAITRite analysis system), balance (Bertec Balance Check ScreenerTM force platform system) **Secondary outcome(s):** TIS, Stroke Rehabilitation Assessment of Movement (STREAM), Goal Attainment Scaling (GAS) **Data collection:** baseline, 8 weeks
Starting date	18/01/2021
Contact information	Principal Investigator: Gülşah Sütçü, MSc, , Hacettepe University, Ankara, Altındağ, Turkey, 06100 Email: gulsahsutcu92@gmail.com
Notes

**Table:** NCT04823546 2021

Study name	Comparison of effects of activity base therapy vs strength training on gait performance in chronic stroke
Methods	**Design:** Parallel group RCT **Country:** Pakistan **Trial registration details:** ClinicalTrials.gov NCT04823546
Participants	**Study population:** 16 **Inclusion criteria:** Aged 55‐65 years at least 6 months post stroke. able to walk independently or with help of walking aids ischemic stroke patients. Mini mental state examination patients fully understand the commands **Exclusion criteria:** neurological disorders. psychological problems. cognitive issues. severe visual issues. other medical complications foot ulcers, orthopaedic or other neurological impedance
Interventions	1. activity based therapy 2. strength training
Outcomes	**Primary outcome(s):** TUG, FMA lower extremity **Secondary outcome(s):** Not stated **Data collection:** 2 months
Starting date	15/02/2020
Contact information	Principal Investigator: Binash Afzal, PHD, Riphah International university, Lahore, Punjab, Pakistan, 54000
Notes

**Table:** NCT04908241 2021

Study name	Telerehabilitation with aims to improve lower extremity recovery post‐stroke (TRAIL‐RCT): a randomized controlled trial
Methods	**Design:** Parallel group RCT **Country:** Canada **Trial registration details:** ClinicalTrials.gov NCT04908241
Participants	**Study population (planned):** 96 **Inclusion criteria:** ≥19 years of age, ≤12 months post‐stroke with lower extremity hemiparesis Able to walk ≥10 meters with or without a gait aid and without physical assistance of another person Can tolerate 50 minutes of activity (including rest breaks) Has cognitive‐communicative ability to participate, per clinical judgement Able to provide consent Has a caregiver, friend, or family member available to provide physical support during the assessment sessions **Exclusion criteria:** Currently participating in formal in‐ or out‐patient stroke rehabilitation focusing on lower extremity training Living in long‐term care Severe vision or hearing loss Significant musculoskeletal or other neurological conditions Not medically stable Comorbidities (e.g. limb amputation), pain or other symptoms that significantly impact lower extremity function Planned surgery that would preclude or affect participation in the protocol
Interventions	1. TRAIL ‐ 4‐week progressive exercise and self‐management intervention for lower extremity recovery 2. Education
Outcomes	**Primary outcome(s):** TUG **Secondary outcome(s):** SIS, Activities‐Specific Balance (ABC) Scale, FRT, Modified Virtual FMA, 30 second Sit to Stand, Health Resource Utilization Questionnaire, EQ‐5D, Tandem Stand **Other outcome(s):** Feasibility Indicators: Recruitment Rate, Retention Rate, Perceived Benefit of Telerehabilitation, Treatment Fidelity, Blinding of Outcome Assessors, Appropriateness of Randomization Process, Number of Participants Excluded based on Eligibility Criteria, Participant and Assessor Burden, Participant Burden, Ease of Using Equipment, Safety, Processing Time, GENESIS‐PRAXY Questionnaire **Data collection:** baseline, post‐intervention (4 weeks), 3 months, 6 months
Starting date	08/11/2021
Contact information	Principal Investigator: Brodie Sakakibara, PhD, McMaster University, Vancouver, British Columbia, Canada, V6T 1Z4. Email: brodie@mail.ubc.ca Principal Investigator: Ada Tang, PhD, McMaster University, Vancouver, British Columbia, Canada, V6T 1Z4
Notes

**Table:** NCT05033873 2021

Study name	Comparison of Universal Exercise Unit Therapy With Sling Exercise Therapy on Lower Limb Kinematics in Chronic Stroke Patients
Methods	**Design:** Parallel group RCT **Country:** Pakistan **Trial registration details:** ClinicalTrials.gov NCT05033873
Participants	**Study population (planned):** 90 **Inclusion criteria:** Patient with chronic (course of disease at least six month) Recently discharge from in‐patients setting with in 06 month of onset Hemiplegia (either right or left) Both gender Age between 30 to 70 Medically stable No balance disorders before this stroke. History of mental illness or severe cognitive impairment (MINI‐MENTAL SCALE SCORE > 25) **Exclusion criteria:** Stroke Patient with complication like shoulder hand syndrome, adhesive capsulitis or shoulder partial dislocation Stroke patients with behavioral issue, significant cognitive deficit Patients with arthritis and fracture Chronic stroke with deformities Serious viscera dysfunction, such as cardiovascular system, Lung, liver and kidney History of mental Illness or severe cognitive impairment (MINI‐MENTAL SCALE SCORE > 25) Audio‐visual understanding obstacle, unable to cooperate with instructions; Infection and ulcer skin
Interventions	1. Universal Exercise Unit Therapy (UEU) 2. Sling Exercise Therapy (SET) 3. Control Group ‐ routine physical therapy
Outcomes	**Primary outcome(s):** BBS, BI, SF‐12, Smart phone motion analysis for lower limbb kinematics during walking, FRT, TIS **Secondary outcome(s):** not stated **Data collection:** 2 months
Starting date	15/07/21
Contact information	Principal Investigator: Farjad Afzal, PhD. Study Chair: Rabiya Noor, PhD Riphah International University. Email: rabiya.noor@riphah.edu.pk
Notes

**Table:** NCT05158543 2021

Study name	Intensity‐dependent effects of 'functional activities specific training‐table' on physical performance in stroke
Methods	**Design:** Parallel group RCT **Country:** Pakistan **Trial registration details:** ClinicalTrials.gov NCT05158543
Participants	**Study population (planned):** 120 **Inclusion criteria:** both Genders age between 40‐60 years. ≥3 months post stroke. middle & anterior Cerebral Artery stroke 18‐25 points Mild cognitive impairment Montreal Cognitive Assessment (MoCA) FMA UE and LE collectively motor score 50‐70 Modified Rankin scale 3‐4 **Exclusion criteria:** Inability to follow 2‐step commands Ashworth scale 3‐4 current participation in other stroke treatments other neurological diagnoses, history of fall & fractures pregnancy
Interventions	1. task oriented training (moderate intensity) 2. task oriented training (high intensity) 2. conventional intervention for upper and lower limb motor function & balance (control group)
Outcomes	**Primary outcome(s):** FMA, Wolf Motor Function Test, TUG, BBS, Wisconsin Gait Scale **Secondary outcome(s):** MoCA, SS‐QOL **Data collection:** week 12
Starting date	01/01/2023
Contact information	Principal Investigator: Arshad Nawaz Malik, PhD Riphah International University, Rawalpindi, Pakistan, 64400 Email: arshad.nawaz@riphah.edu.pk
Notes

**Table:** NCT05191524 2022

Study name	Comparison of Constraint Induced Movement Therapy and Proprioceptive Neuromuscular Facilitation on Lower Limb Motor Function in Stroke Patients
Methods	**Design:** Parallel group RCT **Country:** Pakistan **Trial registration details:** ClinicalTrials.gov NCT05191524
Participants	**Study population (planned):** 40 **Inclusion criteria:** aged 30‐60 years Both male and female diagnosed with hemiplegia due to stroke Patients with asymmetrical stance, ability to walk and stand with minimal assistance Patients must have 15 degrees of knee flexion in the affected limb Mini mental state examination >23 **Exclusion criteria:** Patients who cannot perform the active movement of a limb due to pre stroke musculoskeletal problems Cardiopulmonary diseases which could hinder their ability to take part in rehabilitation Patient with impaired cognition
Interventions	1. Constrained induced movement therapy (CIMT) 2. Proprioceptive Neuromuscular facilitation therapy (PNF)
Outcomes	**Primary outcome(s):** FMA, TUG, BBS **Secondary outcome(s):** not stated **Data collection:** week 6
Starting date	15/01/22
Contact information	Principal Investigator: Ayesha Afridi, PhD Riphah International University
Notes

**Table:** NCT05323916 2022

Study name	Motor Recovery Following the Comprehensive Intensive Rehabilitation Program After Stroke Implementing the International Classification of Functioning, Disability and Health Model: A Randomized Controlled Trial Protocol
Methods	**Design:** Parallel group RCT **Country:** Czech Republic **Trial registration details:** ClinicalTrials.gov NCT05323916
Participants	**Study population (planned):** 280 **Inclusion criteria:** adults aged 18‐85 years after first ischemic stroke early sub‐acute phase slight to moderately severe disability [2 ‐ 4 on the Modified Rankin Scale] 0 ‐ 2 on the Pre‐Stroke Modified Rankin Score potential to accept 4 hours of comprehensive rehabilitation per day and to benefit from physiotherapy minimal or moderate motor deficit of upper or lower extremities (on NIHSS Item 5 or 6 scores 1‐3 points) able to perform activities of daily living prior to stroke event (0 ‐ 2 on the Pre‐Stroke Modified Rankin Score) Native or fluent Czech language speaker **Exclusion criteria:** low level of consciousness or severe cognitive decline that would interfere with administration of the tests premorbid illiteracy, severe visual and/or auditory deficit that would prevent proper completion of the tests behavioural disorders and/or lack of cooperation with therapist severe medical problems with a poor prognosis, (e.g., severe frailty, advanced and incurable cancer, fracture, cardiovascular disorders as chronic heart failure NYHA III, IV, symptomatic coronary artery disease Angina Severity Class III, IV, respiratory insufficiency as chronic obstructive pulmonary disease GOLD IV, and other severe disease)
Interventions	1. Effectively managed rehabilitation implementing the recommendations of the World Health Organization 2. Neuroproprioceptive "facilitation, inhibition" 3. Technology based physical therapy 4. Control group ‐ standard care.
Outcomes	**Primary outcome(s):** PROMIS ‐ Global Health, WHODAS 2.0, GAS **Secondary outcome(s):** Nine Hole Peg Test, Action Research Arm Test, Motor Activity Log, TUG, BBS, 10mWT, 6MWT, FIM , The Gugging Swallowing Screen, The 3F Test Dysarthric Profile, Mississippi Aphasia Screening Test, Image Naming Test, Amnesia Light and Brief Assessment, Naming Pictures and Their Equipment (POBAV), Neuro ‐ Quality of Life (Neuro‐QOL)‐ depression, MoCA **Data collection:** baseline, 3 weeks and follow‐ups: 3 months, 12 months after admission.
Starting date	01/05/2022
Contact information	Principal Investigator: Dr. Kamila Řasovár. Third Faculty of Medicine Charles University, Czech Republic. Email: kamila.rasova@gmail.com
Notes

**Table:** NCT05425082 2022

Study name	Comparison of Motor Relearning and Neurodevelopmental Therapy on Motor Performance and Quality of Life in Stroke Patients
Methods	**Design:** Parallel group RCT **Country:** Pakistan **Trial registration details:** ClinicalTrials.gov NCT05425082
Participants	**Study population (planned):** 32 **Inclusion criteria:** male and female, with age 45‐70 years First‐time unilateral stroke confirmed by magnetic resonance imaging or computed axial tomography scan. Clinically stable with fully oriented and conscious. Subacute stroke patients. Patients having lower limb dysfunction. Patients with Mini Mental State Examination MMSE score ≥ 24 **Exclusion criteria:** Recurrent stroke Foot drop Cardiac disease that limit function by exertional dyspnea, angina or severe fatigue Any visual and hearing problem Subarachnoid or extradural hemorrhage, progressive hydrocephalus, previous history of brain injury
Interventions	1. Motor relearning program 2. Neurodevelopmental therapy
Outcomes	**Primary outcome(s):** FMA, MBI, TUG **Secondary outcome(s):** not stated **Data collection:** week 6
Starting date	04/04/2022
Contact information	Principal Investigator: Binash Afzal, PHD Riphah International University, Lahore campus.
Notes

**Table:** NCT05616858 2022

Study name	Study on Efficacy and Safety of Complex Exercise Program for the Stroke Patients After Discharge
Methods	**Design:** Parallel group RCT **Country:** Republic of Korea **Trial registration details:** ClinicalTrials.gov NCT05616858
Participants	**Study population (planned):** 100 **Inclusion criteria:** aged 40 to 60 years, either gender persons who can to understand, communicate and follow the instructions those persons with Sub acute stroke. **Exclusion criteria:** patients who has Severe cardiac illness patients those are having fracture patients with recurrent stroke
Interventions	1. Complex exercise program (Stretching, aerobic exercise, strengthening and balancing exercise) 2. Control group (no exercise program)
Outcomes	**Primary outcome(s):** Peak VO2 **Secondary outcome(s):** FAC, BBS, TUG, Grip strength, 6MWT, Figure‐of‐eight walk test, Sit‐and‐reach test, Chair sit‐to‐stand test, FMA, Manual function test, MBI (Korean version), Body composition analysis, Korean MMSE, EQ‐5D, Geriatic Depression Scale, IPAQ **Data collection:** baseline, post‐intervention (6 weeks)
Starting date	18/03/2022
Contact information	Sung‐Hwa Ko, PhD. Pusan National University Yangsan Hospital. Email: ijsh6679@gmail.com
Notes

**Table:** PACTR201611001646207 2016

Study name	Effects of combined modified constraint‐induced movement therapy upper and lower limb on physical functions and health‐related quality of life of hemiparetic stroke survivors
Methods	**Design:** Parallel group RCT **Country:** Nigeria **Trial registration details:** Pan African Clinical Trials Registry PACTR201611001646207
Participants	**Study population:** 56 **Inclusion criteria:** aged 18‐65 years. stroke survivors who volunteered to participate in every aspect of the study. first ever stroke at least 6 months prior to the study minimal or no cognitive impairements based on ability to comprehend and execute 3‐word commands. at least 90 degrees of passive range of motion of shoulder flexion and abduction, 45 degrees of shoulder external rotation, not less than 30 degrees of elbow extension, 45 degrees of forearm supination/pronation, no metacarpophalangeal joint should have greater than a 30 degree contracture. minimum score of 2/3 on the Upright Motor Control Test (extension) which is a clinical indicator of ambulation potential post‐ stroke. **Exclusion criteria:** uncontrolled blood pressure score greater than 2 on Modified Ashworth scale participating in any experimental rehabilitation or drug studies excessive pain in the affected upper or lower limb, as measured by a score of 6 or higher on a 10‐point visual analog scale
Interventions	1. Modified Combined Constraint Induced Movement Therapy (CIMT) for upper and Lower Limb 2. Modified CIMT for Lower Limb 3. Modified CIMT for Upper Limb
Outcomes	**Primary outcome(s):** Height and body weight, Lower Limb Motor Function (LLMF), FMA, Lower‐Extremity Motor Activity Log, Weight Asymmetry Ratio (WAR), spatiotemporal gait parameters (gait speed (m/s) and stride length (m)) **Secondary outcome(s):** SIS, Activities‐specific Balance Confidence Scale **Data collection:** baseline, post‐intervention: week 2, week 4
Starting date	21/08/2014
Contact information	Principal Investigator: Lawan Umar, Ibadan, Nigeria. Email: lwnumar12@yahoo.com
Notes

**Table:** PACTR201712002689193  2017

Study name	Effect of 6 weeks task‐oriented circuit training on balance and quality of life of stroke survivors
Methods	**Design:** RCT **Country:** Nigeria **Trial registration details:** Pan African Clinical Trials Registry PACTR201712002689193
Participants	**Study population (planned):** 30 **Inclusion criteria:** outpatient stroke survivors aged 18‐60 years ability to walk 10 meters without with or without walking aid post stroke duration of 1‐6 months **Exclusion criteria:** uncontrolled hypertension form of balance impairment other than stroke history of fracture or surgical procedure in the lower limb any musculoskeletal disorder such as osteoarthritis
Interventions	1. Task‐oriented circuit training 2. Conventional physiotherapy
Outcomes	**Primary outcome(s):** BBS **Secondary outcome(s):** SS‐QOL **Data collection:** Unclear
Starting date	Estimated: 15/01/2018
Contact information	Wasinda Malgwi. Damboa Road, Maiduguri, Nigeria. Email: sinda4life@gmail.com
Notes

**Table:** PACTR201810717634701  2018

Study name	Task‐specific training with multi‐sensory biofeedback on ambulation, balance, cognition and societal participation in individuals post stroke
Methods	**Design:** Parallel group RCT **Country:** Nigeria **Trial registration details:** Pan African Clinical Trials Registry PACTR201810717634701
Participants	**Study population (planned):** 96 **Inclusion criteria:** Post‐stroke individuals who are ambulant and can comprehend information in English and/or Hausa Languages. aged 18 ‐ 90 years. first‐ever diagnosed stroke of at most 6 months duration. ability to walk independently unaided at least for 10 meters and at self faced speed able to maintain balance of > 30 seconds unsupported without falling >3 on functional ambulation classification/ categories (FAC). Modified ashworth scale score of less than 3 . **Exclusion criteria:** severe cognitive impairment (determined using MMSE) or perceptual impairment, psychiatric disorders and seizure disorders. severely cardiopulmonary compromised severe visual and auditory defects that can interfere with walking ability. history of pre‐existing neurological conditions that would influence gait (e.g. Parkinson’s disease, Cerebella Ataxia). bed‐ridden. unstable cardiovascular disease/status. musculoskeletal problems such as amputation, previous lower limb Orthopaedic surgeries, prosthetics devices, any form of severe incapacitating arthritis, myopathies and other athropathies that can interfere with walking. other severe medical conditions, such as severe diseases of the heart, liver, kidney and lungs
Interventions	1. Mobility related overground task specific training with multisensory biofeedback. 2. Balance related overground task specific training. 3. Balance related overground task specific training with multisensory biofeedback. 4. Mobility related overground task specific training (control group).
Outcomes	**Primary outcome(s):** spatiotemporal gait variables (10mMWT), 6MWT, Ambulatory self confidence questionnaire, lower extremity functional scale, FMA, Modified Emory Functional Ambulatory Profile, Rivermead Mobility Index, BBS, MMSE **Secondary outcome(s):** FAC, MAS **Data collection:** baseline, 6 weeks (during intervention), 12 weeks (intervention end) and 3 months post‐intervention.
Starting date	Estimated: 30/09/2018
Contact information	Jibrin Sammani Usman, Gwarzo Road, Kano, 3011, Nigeria. Email: jibrilphysio@yahoo.com
Notes

**Table:** PACTR202001617788536 2020

Study name	TASSRET
Methods	**Design:** Parallel group RCT **Country:** Nigeria **Trial registration details:** Pan African Clinical Trials Registry PACTR202001617788536
Participants	**Study population (planned):** 286 **Inclusion criteria:** aged 18‐64 years Diagnosed of a first ever episode of ischemic or hemorrhagic stroke (including intracerebral hemorrhage and subarachnoid hemorrhage). Attained mild functional motor capacity of the affected upper limb, as indicated by a score of 10 on the Action Research Arm Test. Have a Mini Mental State Examination (MMSE) score of above 18 Speaks Hausa. **Exclusion criteria:** cerebrovascular events due to malignancy or head trauma limited comprehension and receptive aphasia diagnosed with other neurological disorder not available for 5 months follow up currently attending physiotherapy out‐patient departments for rehabilitation services
Interventions	1. Task specific self rehabilitation training 2. Usual Care
Outcomes	**Primary outcome(s):** Motor function and mobility (for both upper and lower extremities), Upper extremity dexterity, Basic mobility and dynamic balance, walking speed **Secondary outcome(s):** Spasticity, functional exercise capacity, Upper extremity functional recovery, Social participation, Gross manual dexterity **Data collection:** baseline, baseline, 3 and 6 months
Starting date	Estimated 29/08/2020
Contact information	Principal Investigator: Rabiu Ibrahim, Assistant Director of Physiotherapy National Assembly Clinic Abuja. National Assembly Complex, Three Arm Zone, Garki, CBD, FCT Abuja, 900001, Nigeria. Email: tok2rabs@gmail.com
Notes

**Table:** PACTR202012569619981  2020

Study name	Augmented‐exercise therapy with gait and gait‐related functional training in improving functional independence, quality of life and productivity in stroke survivors: a clinical control trial
Methods	**Design:** RCT **Country:** Nigeria **Trial registration details:** Pan African Clinical Trials Registry PACTR202012569619981
Participants	**Study population (planned):** 60 **Inclusion criteria:** clinically‐diagnosed first‐ever stroke survivors within the first year of stroke. aged 18 ‐ 65 years. a score of greater than or equal to 23 on MMSE. are able to understand verbal, graphic, pictorial and written instructions. able to walk 10 meters independenly with or without a walking aid without human support. **Exclusion criteria:** pre‐existing or comorbid neurological or psychiatric conditions, such as dementia, schizophrenia, seizure or epilepsy. significant visual impairment which is not amendable by corrective visual aids. concomitant health conditions like anthropathy, osteopathy, myopathy, severe diseases of the lung, heart liver or kidney.
Interventions	1. Multimodal treadmill group 2. Progressive resistance training group 3. Conventional physiotherapy group
Outcomes	**Primary outcome(s):** 10mMWT (step length, stride length, speed, maximum speed and cadence) **Secondary outcome(s):** Borg rating of Borg rating of perceived exertion (during intervention) **Data collection:** Pre, mid and post intervention
Starting date	Estimated: 08/12/2020
Contact information	Nwaedozie Obianuju 18 Sam Wobo Street, Port Harcourt, 500211, Nigeria. Email: uju4christ2000@yahoo.com
Notes

**Table:** RBR‐43x6kh 2018

Study name	Effects of Contraint Induced Movement Therapy for lower extremities on gait and balance functionality in post‐stroke patients
Methods	**Design:** Parallel group RCT **Country:** Brazil **Trial registration details:** The Brazilian Registry of Clinical Trials (ReBEC) RBR‐43x6kh
Participants	**Study population:** 40 **Inclusion criteria:** aged 21‐85 years. medical diagnosis of stroke (ischemic or hemorrhagic), with only one event, and have suffered stroke for 6 months or more at the beginning of the protocol. clinically stable. hemiparesis caused by stroke. defict of gait caused by stroke. hip and knee flexion intiation on the affected side can move from sit to stand independently even with the aid of upper limbs and/or auxiliary device and/or furniture. able to transfer in an independent way when seated able to sustain the body weight on the affected side even requiring aid from a third person, auxiliary device or furniture. able to walk 10 meters with or without requiring aid from a third person, auxiliary device or furniture able to be accompanied by the same caregiver (we will accept at most 2). available for going to the rehabilitation center during 17 followed days (15 days for treatment, and 2 days for evaluation). have not undergone orthopedic surgery in the last 6 months, or neuromuscular blockade in the last 3 month. not be able to walk outside of home independently. **Exclusion criteria:** does not accept the protocol allocated by randomization. language change that will not allow the participant to understand, as well as respond appropriately to the proposed assessment scales and exercises selected for the protocol. has clinical complications between the screening and the beginning of the protocol.
Interventions	1. Constraint Induced Movement Therapy (CIMT) 2. Intensive conventional therapy
Outcomes	**Primary outcome(s):** gait speed, cadence, single support time on the affected side, step length, measured by tridimensional gait analysis, MiniBESTest **Secondary outcome(s):** None planned **Data collection:** Not stated
Starting date	11/30/2017
Contact information	Elaine Menezes Oliveira. Rua Rosa do Céu, 29, São Paulo, Brazil, 08061‐410. Email: elaine‐m_oliveira@yahoo.com.br Gabriela da Silva Matuti. Rua Lourdes Lopes Sanches, 300 ap16A22, Guarulhos, Brazil, 07190‐033. Email: gabrielamatuti@hotmail.com
Notes

**Table:** Richardson 2018

Study name	FIT for FUNCTION
Methods	**Design:** Parallel group (mixed methods) RCT **Country:** Canada **Trial registration details:** ClinicalTrials.gov NCT02703805
Participants	**Study population:** 216 **Inclusion criteria:** age ≥ 18years living in the community able to independently ambulate ≥ 10 m with or without an assistive device able to tolerate 60 min of activity with rest intervals not actively engaged in active rehabilitation able to follow instructions; medically cleared to participate in the program MoCA will be used to describe level of cognitive impairment that would prevent participation able to communicate in English and not have a comprehensive or global aphasia **Exclusion criteria:** not reported
Interventions	1. FIT for FUNCTION program exercise sessions, self management and education) 2. Standard YMCA membership
Outcomes	**Primary outcome(s):** Reintegration to Normal Living Index (RNLI) **Secondary outcome(s):** MoCA, Centre for Epidemiological Studies Depression Scale (CES‐D), Vital signs, Falls Assessment, BBS, EQ‐5D, Self‐Efficacy, Rapid Assessment of Physical Activity (RAPA), Patient Activation Measure (PAM), Bloodwork, Healthcare utilization questionnaire, physical activity level (accelerometer), adverse events, Short Physical Performance Battery (SPPB), 6MWT, grip strength, isometric knee extension strength, interviews **Data collection:** baseline, 12 weeks, and 24 weeks
Starting date	May 2014
Contact information	Julie Richardson, School of Rehabilitation Science, McMaster University, Hamilton, ON, Canada. Email: richard@mcmaster.ca
Notes	Study is supported by an operating grant from the Canadian Institutes of Health Research, FRN 133486

**Table:** Traxler 2021

Study name	SATMANT
Methods	**Design:** Multi‐arm mixed methods RCT (3‐arms) **Country:** Germany **Trial registration details:** German Clinical Trials Register DRKS00023068
Participants	**Study population:** 36 **Inclusion criteria:** 18 to 98 years of age ischaemic or haemorraghic stroke at least 6 months prior to study start limited dorsiflexion ROM in the upper ankle joint on the hemiparetic side: maximum 0° active ROM;maximum 5° of passive ROM4. able to independently transfer from a seating to a standing position without an assistive device‐ use of the hands is allowed able to independently walk without an assistive device for at least 10m no contraindications connected to exposure to active training **Exclusion criteria:** any contraindication to MT (ankle instability, ankle inflammation or traumas etc.) any not fully recovered injuries of the musculoskeletal system previous surgery in the lower extremity region leading to walking impairment due to orthopaedic reasons relevant ROM reduction of the hip or knee joints any cognitive or communication impairment hindering participation (e.g.dementia, severe aphasia, being under legal guardianship etc.) diabetes mellitus rheumatic diseases chronic pain treated by drug therapy history of previous stroke with persistent, impairing neurological symptoms (defined by a Modified Rankin Scale score of ≥ 3) comorbidity hindering participation (e.g. life expectancy < 12 months, cancer,s evere heart insufficiency etc.) mRS score ≥ 4 pregnancy military service
Interventions	1. Talocrural joint amnual therapy plus task‐oriented training. 2. Task‐oriented training. 3. Usual care (control)
Outcomes	**Primary outcome(s):** MiniBESTest **Secondary outcome(s):** 10mMWT (maximum speed) TUG (maximum speed), TUG (dual‐tasking), active and passive loaded and unloaded ankle joint ROM, SIS v2.0, Feasibility parameters: recruitment rate; retention rate; home exercise adherence rate, Falls diary, Participants’ acceptance of the intervention structured interview. **Data collection:**
Starting date	17/09/2020
Contact information	Barbara Seebacher. Clinical Department of Neurology, Medical University of Innsbruck, Innsbruck, Austria and VASCAGE GmbH, Research Centre on Vascular Ageing and Stroke, Innsbruck, Austria. Email: barbara.seebacher@i‐med.ac.at
Notes	This study is self‐funded by the initiating investigator (KT) in the course of her Master’s studies in neurological physiotherapy at the Department for Health Sciences, Medicine and Research, Faculty of Health and Medicine, Danube University Krems, Austria. VASCage GmbH, Research Centre on Vascular Ageing and Stroke, Innsbruck, Austria supports the indemnity insurance costs and has no role in the design of the study and collection, analysis, and interpretation of data

10mWT: 10 meter walk test 5mWT: Five‑meter Walk Test 5xSTS: Five Times Sit to Stand Test 6MWT: Six‑minute Walking Test BBS: Berg Balance Scale BDI: Beck Depression Inventory BESTest: Balance Evaluation Systems Test BI: Barthel Index CAQ: Community Ambulation Questionnaire CarerQoL: Care‐related Quality of Life Instrument CME: Caregiver‐mediated exercises CSI: Caregiver Strain Index CT: Computerized tomography DGI: Dynamic Gait Index EQ‐5D: EuroQol 5D FAC: Functional Ambulation Category FAD‐GF: General functioning subscale of the McMaster Family Assessment Device FES: Falls Efficacy Scale FIM: Functional Independence Measure FMA: Fugl Meyer Assessment FRT: Functional Reach Test FSS: Fatigue Severity Scale GCS: Glasgow Coma Score GSES: General Self‑efficacy Scale HADS: Hospital Anxiety and Depression Scale HAMA: Hamilton Anxiety Scale Score IPAQ: International Physical Activity Questionnaire LOS: Length of Stay MAS: Modified Ashworth Scale MASS: Massachusetts Acupuncture Sensation Scale MBI: Modified Barthel Index MI: Motricity Index MiniBESTest: Mini Balance Evaluation Systems Test MMSE: Mini‐Mental State Examination MoCA: Montreal Cognitive Assessment MOX‑2: MOX‑2 physical activity monitor MRI: Magnetic resonance imaging mRMI: Modified Rivermead Mobility Index mRS: Modified Rankin Score NEADL: Nottingham Extended Activities of Daily Living index NIHSS: National Institutes of Health Stroke Scale PCS: Preparedness for Caregiving Scale PD: Parkinson's disease ROM: Range of Movement SARA: Scale for the Assessment and Rating of Ataxia SIS: Stroke Impact Scale SPPB: Short Physical Performance Battery SSEQ: Stroke Self‑efficacy Questionnaire SS‐QOL: Stroke Specific Quality of Life Scale TAI: Transient ischemic attack TIS: Trunk Impairment Scale TPS: Transition Preparedness Scale TUG: Timed Up and Go Test

## Differences between protocol and review

For differences between the protocol and earlier versions of the review, see Pollock 2007; Todhunter‐Brown 2014.

### Differences between the previous 2014 review version and this update

We summarise the differences (and similarities) between the 2014 review version (Todhunter‐Brown 2014) and this updated version below:

**Objectives:** no change

**Criteria for considering studies for this review:**

- Types of studies ‐ no change.
- Types of participants ‐ no change.
- Types of interventions ‐ we made additional clarifications for interventions classed as single components; in this update we excluded studies focussed on trunk training, which were previously included. We decided to exclude studies in which the intervention was a programme of stroke rehabilitation that included a physical rehabilitation element as part of a broader post‐stroke rehabilitation programme. These changes led to the exclusion of a number of studies that were previously included in this review (see Characteristics of excluded studies).
- Types of outcomes ‐ primary outcomes: no change.
- Types of outcomes ‐ secondary outcomes: we added clarification around which measures of gait velocity we would include. We added adverse events as a secondary outcome, defined as adverse events possibly, probably or definitely relating to the study/intervention, as attributed by the study authors.
- Adverse events ‐ we added adverse events as an outcome measure (not previously systematically recorded).

**Search methods and selection of studies:** search strategies comprehensively updated. Use of Covidence for management of study selection.

**Data extraction and management:**

- Methods: previously, two independent review authors performed data extraction for study characteristics and also outcome data. For this update, due to the number of studies included, one author extracted study characteristics from study reports, and a second checked this and entered these into RevMan.
- Interventions: we used an updated framework to describe and categorise the interventions within the studies. This was co‐produced with our stakeholder group.
- Comparisons: previously we grouped and analysed studies into three comparison groups (physical rehabilitation versus no treatment, physical rehabilitation versus usual care or attention control, different approaches to physical rehabilitation). For this update, we grouped and analysed studies into four comparison groups. The comparison of 'additional physical rehabilitation + usual rehabilitation versus usual rehabilitation' is new. Justification for this change is provided in Appendix 1.

**Assessment of risk of bias:** in this review version, we used the new risk of bias tool, RoB 2. Previously, we used the original Cochrane tool for assessing risk of bias.

**Data analysis:**

- For outcomes of independence in activities of daily living, motor function, balance, gait velocity, or length of stay, there were no changes to methods of meta‐analysis. We did change how we entered data for the main analyses, grouping data according to the method of assessment (i.e. outcome measure) rather than the type of intervention. We made some minor changes to the groupings for our subgroup analyses, based on comments and feedback from our stakeholder group. We introduced and presented a structured, sequential approach to our sensitivity analyses.
- For adverse events, we have introduced analyses of adverse events, calculating risk ratios using Mantel‐Haenszel fixed‐effect meta‐analysis methods. When planning this update, we anticipated that adverse events would be assessed and reported in a wide range of different ways, and would not be suitable for meta‐analyses. We therefore did not originally plan to conduct any quantitative analyses relating to adverse events. Instead, we planned to tabulate and describe available data relating to adverse events. However, during the course of the editorial process, we re‐evaluated this plan and eventually pursued analysis of the identified adverse event data.

**Assessment of reporting biases:** in this update, we have produced funnel plots, which were not previously described in the protocol.

**Data synthesis:** for this update, we received advice and support from the Cochrane Methods Support Unit. This led to a number of changes to the structure and presentation of the review, limiting the amount of information presented as text and using additional tables to summarise data (e.g. results of subgroup and sensitivity analyses; results of analyses for follow‐up data).

**Summary of findings:** for this review version, we only included outcomes measured at the 'immediate' time point in our summary of findings tables. We added findings on adverse events to the summary of findings tables.
